# A Locus Encoding Variable Defense Systems against Invading DNA Identified in *Streptococcus suis*

**DOI:** 10.1093/gbe/evx062

**Published:** 2017-04-01

**Authors:** Masatoshi Okura, Takashi Nozawa, Takayasu Watanabe, Kazunori Murase, Ichiro Nakagawa, Daisuke Takamatsu, Makoto Osaki, Tsutomu Sekizaki, Marcelo Gottschalk, Shigeyuki Hamada, Fumito Maruyama

**Affiliations:** 1 Division of Bacterial and Parasitic Diseases, National Institute of Animal Health, National Agriculture and Food Research Organization, Tsukuba, Japan; 2 Department of Microbiology, Kyoto University Graduate School of Medicine, Japan; 3 Research Center for Food Safety, Graduate School of Agricultural and Life Sciences, The University of Tokyo, Japan; 4 The United Graduate School of Veterinary Sciences, Gifu University, Japan; 5 Groupe de Recherche sur les Maladies Infectieuses du Porc, Faculté de Médecine Vétérinaire, Université de Montréal, St-Hyacinthe, Quebec, Canada; 6 Research Institute for Microbial Diseases, Thailand-Japan Collaboration Center for Emerging and Re-emerging Infections, Osaka University, Suita-Osaka, Japan

**Keywords:** bacterial defense systems, mobile genetic elements, competence system, *Streptococcus suis*

## Abstract

*Streptococcus suis*, an important zoonotic pathogen, is known to have an open pan-genome and to develop a competent state. In *S. suis*, limited genetic lineages are suggested to be associated with zoonosis. However, little is known about the evolution of diversified lineages and their respective phenotypic or ecological characteristics. In this study, we performed comparative genome analyses of *S. suis*, with a focus on the competence genes, mobile genetic elements, and genetic elements related to various defense systems against exogenous DNAs (defense elements) that are associated with gene gain/loss/exchange mediated by horizontal DNA movements and their restrictions. Our genome analyses revealed a conserved competence-inducing peptide type (pherotype) of the competence system and large-scale genome rearrangements in certain clusters based on the genome phylogeny of 58 *S. suis* strains. Moreover, the profiles of the defense elements were similar or identical to each other among the strains belonging to the same genomic clusters. Our findings suggest that these genetic characteristics of each cluster might exert specific effects on the phenotypic or ecological differences between the clusters. We also found certain loci that shift several types of defense elements in *S. suis*. Of note, one of these loci is a previously unrecognized variable region in bacteria, at which strains of distinct clusters code for different and various defense elements. This locus might represent a novel defense mechanism that has evolved through an arms race between bacteria and invading DNAs, mediated by mobile genetic elements and genetic competence.

## Introduction

*Streptococcus suis* is a zoonotic pathogen that can cause meningitis and systemic diseases in pigs and humans, and asymptomatic pigs typically carry *S. suis* in their upper respiratory tract (Goyette-Desjardins et al. 2014). Serotyping and multilocus sequence typing (MLST) have been used globally for epidemiological and genetic diversity studies on *S. suis*. The accumulated data indicate that numerous isolates worldwide from humans and pigs with meningitis or severe systemic infections were serotype 2 isolates and belonged to clonal complex 1 (CC1) (King et al. 2002; Goyette-Desjardins et al. 2014; Okura et al. 2016). Therefore, serotype 2-CC1 strains have been regarded as the genetic lineages potentially most hazardous to humans and pigs. CC20, CC25, CC28, and CC104 also include several serotype 2 strains from both humans and diseased pigs; however, isolates belonging to these CCs appear to be endemically collected (Goyette-Desjardins et al. 2014). Most of the strains typed into other CCs or sequence types (STs) were isolated from healthy pigs, pigs with noninvasive diseases, or from other animals. These MLST analyses suggest that some of the multifarious genetic lineages are associated with zoonosis in *S. suis.* However, limited information is available on the evolutionary processes that generate the diversified lineages in *S. suis* and the phenotypic or ecological characteristics of the respective lineages.

Bacterial genomes are diversified by gaining, losing, and exchanging genes through various processes. One of the major driving forces related to these processes is horizontal DNA transfer (HDT) mediated by mobile genetic elements (MGEs) such as phages, integrative conjugative elements (ICEs), and plasmids (Frost et al. 2005; Wiedenbeck and Cohan 2011). Moreover, in certain bacterial species, genetic competence participates in genome diversification processes (Johnsborg et al. 2007; Johnston et al. 2014). Small mutations affecting genes (nucleotide substitutions or deletions and insertions of one or more extra nucleotides) and chromosomal rearrangements during DNA replication or repair can also contribute to bacterial genome diversity through gene conversions as well as inactivation or genesis of genes (Lawrence 2002; Vetsigian and Goldenfeld 2005; Darling et al. 2008; Raeside et al. 2014). If the genomic changes that occur as a result of genome diversification lead to fitness enhancement of the bacteria in their habitats (including their host environment), the changes could be maintained in the bacterial genomes as beneficial mutations (Rankin et al. 2011; Werren 2011). However, even when genes are gained from external sources, the genomic changes can also involve several risks, such as the replicative, transcriptional, and metabolic burden of the changes, as well as the possible disruption of regulatory and protein interaction networks (Croucher et al. 2016).

For protection against invading exogenous DNAs, bacteria possess defense mechanisms. Restriction-modification (R-M), DNA phosphorothioation (DND), bacteriophage exclusion (BREX), and clustered regularly interspaced short palindromic repeats (CRISPR)/CRISPR-associated protein (CRISPR/Cas) systems are bacterial defense systems for cleaving or inactivating incorporated foreign DNAs (Williams 2003; Horvath and Barrangou 2010; Makarova et al. 2013; Vasu and Nagaraja 2013; Goldfarb et al. 2015). Among these, the CRISPR/Cas system functions as an acquired immune system in prokaryotic organisms: It integrates a partial short sequence of foreign DNA between CRISPR repeats as a spacer and attacks reinvading foreign DNA based on recognizing sequence similarity with the spacers (Horvath and Barrangou 2010). Toxin–antitoxin (T-A) and abortive infection (Abi) systems also defend against invading DNAs, but through mechanisms differing from those of the aforementioned systems: T-A and Abi systems hinder the propagation of MGEs (especially prophages) in bacterial populations by inducing programmed cell death in response to infection or invasion (Makarova et al. 2013). Comparative genomics focused on these defense systems in bacteria previously indicated the involvement of transposable elements in the HDT of genetic elements including genes related to defense systems (hereafter “defense elements”) (Makarova et al. 2011). Furthermore, certain defense systems were shown or suggested to be capable of playing a role in stabilizing genomic islands or MGEs carrying the defense elements (Vasu and Nagaraja 2013; Mruk and Kobayashi 2014; Barrangou 2015). Conversely, prophage insertion into a CRISPR locus was also identified, alongside certain MGEs exhibiting anti-RM or anti-CRISPR ability (McMahon et al. 2009; Bondy-Denomy et al. 2013; Pawluk et al. 2014; Croucher et al. 2016). These findings imply that defense elements could be acquired, lost, or exchanged through an arms race between bacteria and invading DNAs. In *S. suis*, previously performed comparative genomics suggested that this bacterium has an open pan-genome and undergoes recombination at high rates (Zhang et al. 2011; Chen et al. 2013; Weinert et al. 2015). However, little is known about defense systems in *S. suis*, except for few R-M systems (Sekizaki et al. 2001, 2005; Willemse and Schultsz 2016).

In naturally competent bacterial species, genetic competence is a transient physiological state and is tightly controlled by species-specific processes, including quorum sensing and nutritional signaling (Chen and Dubnan 2004). Competence-related genes can be classified into two groups: Genes involved in the development of the competent state (early competence genes) and genes related to DNA uptake, import, and recombination (late competence genes) (Johnsborg et al. 2007). In several species, various mutations in competence genes, which are likely to affect competence, occurred in certain strains (Jorth and Whiteley 2012; Croucher et al. 2016). Moreover, considerable diversity of “pherotype,” which is determined by competence-inducing peptide pheromones, was shown to exist among naturally competent streptococci presenting a high frequency of mosaic structures in the related early competence genes, hinting that pherotype switch occurred frequently among the streptococci (Håvarstein et al. 1997). In *S. suis*, the competence state can be induced by competence-inducing peptides, and at least two pherotypes are recognized (Zaccaria et al. 2014).

Here, to gain insights into the evolutionary processes that generate the diversified lineages in *S. suis*, we analyzed the genome sequences of 64 *S. suis* strains. These covered 35 serotypes and genetically diverse strains including several strains classified into the same CCs through MLST, together with the (publicly available) genome sequences of 42 other *Streptococcus* spp. strains, with a particular focus on the defense systems and competence systems that provide the central mechanisms for acquiring and restricting incoming DNA. The results of our retrospective genomic analyses demonstrated the genetic characteristics of the defense systems and competence systems conserved in the strains that were classified into the same clusters based on the genomic phylogeny of *S. suis* strains. We also found the common large-scale inversion that occurs in the *S. suis* strains clustered into certain phylogenetic branches. Furthermore, we identified an unprecedented genomic locus where various defense elements across several classes, prophages, and/or genetic elements encoding hypothetical proteins are shifted.

## Materials and Methods

### Bacterial Strains

We determined the draft genome sequences of 47 *S. suis* strains (supplementary table S1, Supplementary Material online). Of these, 34 strains were *S. suis* serotype reference strains (serotypes 1, 3–34, and 1/2), which were found to show various STs through MLST (King et al. 2002). The other 13 strains, which were isolated from humans and pigs in Thailand or Japan, were typed into CC1 (three strains), CC25 (two strains), CC28 (four strains), CC104 (two strains), and ST94 (two strains) (Takamatsu et al. 2008, 2009). Although eight strains were isolated from human patients with meningitis, endocarditis, and/or pulmonary edema, 39 were from livestock animals: diseased pigs (29 strains), clinically healthy pigs (seven strains), diseased calves (two strains), and a diseased lamb (one strain). Here, we used these 47 genome sequences for comparative genome analysis with the publicly available genome sequences of 17 *S. suis* strains (supplementary table S1, Supplementary Material online) and each strain of the 42 *Streptococcus* spp. (supplementary table S2, Supplementary Material online) obtained from the NCBI database. The 17 *S. suis* strains comprised 10 CC1 strains (including serotype 2 reference strain S735^T^), 2 CC25 strains, 2 CC28 strains, and 3 other CC-type strains.

### DNA Extraction, Genome Sequencing, and Gene Annotation

Genomic DNA extracted from each strain was used for constructing genome sequence libraries, and then genome sequence data were obtained using an Illumina Genome Analyzer IIx as described previously (Okura et al. 2013). Generated read sequences were assembled after trimming low-quality sequences by using Trimmomatic (Bolger et al. 2014) with Velvet (Zerbino 2010) after optimizing k-mer from 17 to 75. Genes were predicted and annotated by the Rapid Annotation using Subsystems Technology (RAST; http://RAST.nmpdr.org; last accessed August 20, 2013) and Microbial Genome Annotation Pipeline (MiGAP; http://www.migap.org; last accessed August 20, 2013) servers, respectively (Aziz et al. 2008; Sugawara et al. 2009). All coding sequences (CDSs) were clustered into homology groups (HGs) by using the gene family method implemented in PGAP-1.01 with default parameters and employing the MCL algorithm (Enright et al. 2002; Zhao et al. 2012).

### Construction of Phylogenetic Trees

The pan-genome tree was constructed based on the presence/absence of all the HGs assigned in this study. We used kSNP program version 3.021 (Gardner et al. 2015), an alignment-free sequence analysis tool, to build whole-genome phylogenies based on single nucleotide polymorphisms (SNPs) in the whole-genome data of all 106 *Streptococcus* strains and 57 *S. suis* strains. For SNP determination, we applied default parameters and a k-mer selected as the optimal value predicted by the kSNP-associated Kchooser script. A consensus parsimony tree based on all of the SNPs was constructed. We also constructed genome-wide trees according to the amino acid sequence alignment of the core genome of all 106 strains and 58 *S. suis* strains. The single-copy CDSs conserved in all of the tested strains were aligned by using MAFFT with an iterative refinement method (L-INS-i) (Katoh and Standley 2013) and were selected to exclude the CDSs exhibiting a high probability of recombination as per the pairwise homoplasy index (PHI) test (cut-off value: *P* ≥ 0.05) in SplitsTree (Huson and Bryant 2006). The amino acid sequences of the selected CDSs were concatenated and used for constructing maximum likelihood phylogenetic trees by the Randomized Axelerated Maximum Likelihood program (Stamatakis 2011) and a neighbor-joining tree by MEGA 6.06 program (Tamura et al. 2013) under the Jones–Taylor–Thornton model and 100 bootstrap iterations. The trees were visualized using Dendroscope (Huson and Scornavacca 2012).

### Inference of Gene Gain and Loss

To infer gain and loss of gene families during evolution, we analyzed the matrix of presence/absence of each of the HGs assigned in this study by using COUNT software (Csuros 2010; Kamneva and Ward 2014). Wagner parsimony with a gene gain/loss penalty of 1 was used to infer the most parsimonious ancestral gene sets, and inferred patterns were mapped on the kSNP tree of 57 *S. suis* strains.

### Identification of the Genes Characteristically Present/Absent in Specific Genomic Clusters

The genes characteristically present (or absent) in specific genomic clusters of *S. suis* were extracted by sorting the pan-genome data. 05HAS68 was excluded from this analysis owing to the unfinished draft genome sequence (Chen et al. 2007). In this study, we defined the characteristic genes of a genomic cluster by identifying genes that were found (or not found) in all the strains belonging to that genomic cluster, but not found (or found) in more than 75% of strains in other clusters.

### Calculation of Average Nucleotide Identity Values

Average nucleotide identity (ANI) values between two genomes were calculated using JSpecies software (Richter and Rosselló-Móra 2009). ANI was calculated based on BLAST (ANIb) and MUMmer (ANIm) by using default parameters. The species demarcation boundary was set to a value of 95–96% of ANI (Goris et al. 2007).

### Detection of Large-Scale Rearrangements in Genomes

The draft genome of *S. suis* 22083 (serotype 9, ST82) and the complete genomes of P1/7 (serotype 2, ST1, CC1), D9 (serotype 7, ST29, CC25), ST3 (serotype 3, ST35, CC28), and ST1 (serotype 1, ST13, CC13) were selected, and large-scale genome rearrangements were detected using Mauve (Darling et al. 2004). The presence of each detected rearrangement was examined in all *S. suis* genomes. For certain strains, polymerase chain reaction (PCR) methods were used when the corresponding regions were located on gaps between contigs.

### Identification of MGEs and Defense Elements

Prophages, ICEs, and RM elements were searched using PHAST (Zhou et al. 2011), ICEberg (Bi et al. 2013), and Rebase (Roberts et al. 2007), respectively. Some of these elements and the TA and Abi elements were found manually from the annotation data and a search of the conserved domain database (Marchler-Bauer et al. 2011). Cas genes were identified using CRISPRFinder (Grissa et al. 2007).

### Identification of Competence Genes and Evaluation of Competence

We identified the competence genes that were reported previously (Zaccaria et al. 2014). For typing of competence pheromone peptides (i.e., to obtain pherotypes), we produced the single-core CDS phylogenetic trees of the related protein ComS and its regulator ComR. The pherotypes were assigned based on the amino acid sequence of ComS, a precursor of the competence-inducing peptide. Competence was confirmed based on whether the plasmid vector pMX1 carrying a spectinomycin-resistance gene (Okura et al. 2011) was introduced into bacterial cells when the N-terminal-truncated ComS peptide was added, as per procedures described previously (Zaccaria et al. 2014). Briefly, *S. suis* strains were grown in Todd-Hewitt broth (THB) (Becton Dickinson, Sparks Glencoe, MD) at 37 °C under 5% CO_2_, and then the cultures were diluted 50-fold with prewarmed THB and incubated at 37 °C without shaking. After incubation for 1 h, 100-µl aliquots were collected from the main culture, and one of two peptides (both at a final concentration of 250 µM) and pMX1 DNA (1 µg) in EB buffer (10 mM Tris-Cl, pH 8.5) were added. After incubation for 2 h at 37 °C under 5% CO_2_, the samples were diluted and plated on THB agar plates with or without spectinomycin (100 µg/ml) supplementation. The peptides used in this study (GNWGTWVEE for active ComS_I, ENWWVK for active ComS_II) were purchased from GenScript (Piscataway Township, NJ). Competency was evaluated based on whether transformants that grew in the presence of spectinomycin were obtained (+) or not (−).

### CRISPR Analysis

On the basis of amino acid sequence similarity and a protein motif of Cas, CRISPR types and structures in *S. suis* were determined in accordance with the CRISPR layouts reported previously (Makarova et al. 2011). In each CRISPR type, consensus sequences of all repeat sequences were determined using WebLogo v3.3 (Crooks et al. 2004). The repeat sequences were further analyzed using CRISPRmap v3.1.5 to characterize their directions (Lange et al. 2013). The phage-like structures in the CRISPRs were identified using both nucleotide sequence similarity with the reported phage sequences in the NCBI nucleotide database and gene annotations. For each CRISPR type, a nonredundant unique spacer list was obtained by performing an all-to-all BLASTN search (Camacho et al. 2009) with the following criterion: Two spacers were regarded to exhibit high nucleotide similarity to each other if the bit-score of BLASTN was >50 (Watanabe et al. 2013). We used the BLASTN search under the following conditions: Word size, 7; dust filter, off. To characterize a target of each spacer sequence, the spacer list was subjected to a BLASTN search against seven databases by using a word size of 7 and the dust filter off, as described previously (Lange et al. 2013). Hits were considered significant when the bit-score was >50. The subject sequences were annotated by performing a BLASTX search in the NCBI GenBank nr database and using the predicted protein of the query sequences under the thresholds of the highest bit-score and *E*-value (≤ 1e-03).

## Results

### Various Genomic Clusters in *S. suis* and Its Divergent Lineages

**Fig. 1.—:**
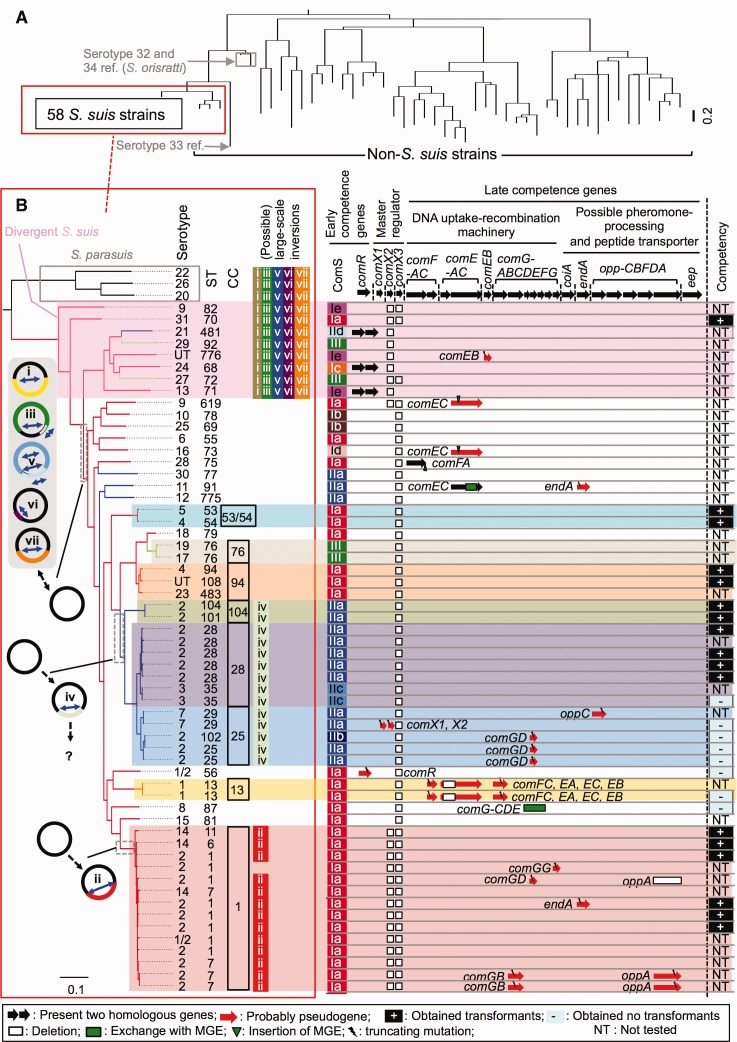
Conserved pherotype and large-scale changes in genome arrangement across deep-branching clades in *S. suis*. (*A*) A consensus parsimony tree based on all of the SNPs of 106 *Streptococcus* strains. Tip labels are described in supplementary figure S1, Supplementary Material online. Branch lengths are proportional to the amount of sequence change. (*B*) Left panel: A part of the kSNP tree of 106 strains that represents the node containing only 58 *S. suis* and 3 *S. parasuis* strains. Eight genomic clusters (clusters_CC1, _CC13, _CC25, _CC28, _CC104, _CC94, _CC76, and _CC53/54) and divergent strains of *S. suis* are highlighted. Characteristic large-scale changes in genome arrangement found in divergent *S. suis* strains, cluster_CC1 strains, and cluster_CC25, _CC28, and _CC104 strains are shown to the right of the tree. Schematic maps of the characteristic changes in the genomes are also appended at the positions of the tree where the genomic events were predicted to have occurred along the branches. The genome of ST1 is used as the reference of the maps, and the locations of these changes are detailed in supplementary figure S7, Supplementary Material online. Right panel: Schematic representations of competence genes, which showed the defective patterns listed in the bottom panel, and the presence/absence of competence efficiency. Gene clusters in competence genes are indicated by the brackets on the top of the schematic representations. Branches of the tree are color-coded according to the change of pherotypes (type of ComS); ComS_I–III are traced using red, blue, and light-green, respectively. Types of ComS are described in supplementary figure S8, Supplementary Material online. Competency was confirmed in 25 strains based on the introduction of the plasmid vector pMX1 upon addition of active ComS peptides.

We constructed kSNP trees, genome-wide trees, and pan-genome trees using whole-genome sequence data of 106 *S. suis* and other *Streptococcus* spp. strains (fig. 1 and supplementary figs. S1–S3, Supplementary Material online). All of the trees demonstrated that *S. suis* reference strains of serotypes 20, 22, 26, and 32–34 were distinct from the other 58 *S. suis* strains; thus, these data confirm previous studies indicating that these reference strains belong to species other than *S. suis* (Hill et al. 2005; Tien et al. 2013; Nomoto et al. 2015). Our analyses further confirmed the other 58 strains, excluding the aforementioned reference strains, as *S. suis*.

In close agreement with the results of previous MLST and whole-genome sequencing analyses (King et al. 2002; Takamatsu et al. 2008; Chen et al. 2013), the kSNP tree of 106 strains and genome-wide trees of the 58 *S. suis* strains showed genomic diversity in the serotype reference strains and the genomic clusters containing the strains typed into the same CC or ST through MLST (clusters_CC1, _CC13, _CC25, _CC28, _CC104, _CC94, _CC76, and _CC53/54; fig. 1*B* and supplementary fig. S4, Supplementary Material online). Four of the clusters (clusters_CC1, _CC25, _CC28, and _CC104) included the serotype 2 strains from humans belonging to the CCs recognized as potentially hazardous groups for zoonosis (fig. 1*B*). Our whole-genome-based phylogenetic analysis also revealed a close relationship between clusters_CC25 and _CC28 as shown in previous MLST-based phylogenetic analysis (King et al. 2002) (supplementary fig. S4, Supplementary Material online). These 58 strains were also clustered according to the CCs on the pan-genome tree, except for 05HAS68, whose determined draft genome sequence was clearly shorter (1.64 Mb) than the sequences of the other strains (supplementary fig. S3, Supplementary Material online). The kSNP tree was constructed without considering the effect of recombination, but the topology was very similar to that of the genome-wide tree that was generated with recombination removed (excluding the CDSs exhibiting a high probability of recombination as per the PHI test) (fig. 1*B* and supplementary fig. S4, Supplementary Material online), suggesting that the kSNP tree represented phylogenetic relationships between genomic clusters in 58 *S. suis* strains with sufficient accuracy.

Our data on the ANI of the total genomic sequence shared between two strains indicated eight “divergent *S. suis*” strains (R61 and reference strains of serotypes 9, 13, 21, 24, 27, 29, and 31), which showed the lowest values or slightly lower than standard values for species demarcation in ANI (>95–96%) (supplementary fig. S5 and tables S3 and S4, Supplementary Material online). These strains were also distinguishable from the 50 *S. suis* strains that could be considered as *S. suis* according to the ANI standard in the phylogenetic trees based on whole-genome sequence data (fig. 1 and supplementary figs. S1, S3, and S4, Supplementary Material online).

### Genes Characteristically Present/Absent in CC-Clusters

Pan-genome analysis identified 7,650 HGs in the 57 *S. suis* strains used in this analysis (except for 05HAS68), 998 and 2999 of which were the core of 57 strains and specific to a certain strain, respectively (supplementary table S5, Supplementary Material online). To estimate the gene gain/loss events that occurred in the 57 *S. suis* strains, COUNT software (Csuros 2010; Kamneva and Ward 2014) was used with the kSNP tree of the 57 *S. suis* strains serving as the guide-tree topology (supplementary fig. S6, Supplementary Material online). Although the inferred numbers of gained/lost genes in this analysis might contain a few errors owing to the use of draft genome sequences, >100 genes were gained in more than one-third of the branches on the tree (41 of 112 branches). Among them, 32 were terminal branches of the tree, including those in the same clusters. These findings support the view that *S. suis* has an open pan-genome (Zhang et al. 2011; Chen et al. 2013; Weinert et al. 2015) and suggest that many HDT events have occurred in this bacterium.

The genes characteristically present/absent in each CC-cluster and divergent *S. suis* strains were identified by sorting the pan-genome data (supplementary table S6, Supplementary Material online). These genes might play a key role in the ecological or phenotypic characteristics of each cluster and divergent *S. suis* strains. Cluster_CC1 (supplementary table S6, Supplementary Material online; lines 1–73) is recognized as the most hazardous phylogenetic group, and cluster_CC1 strains distinctively possess genes related to sialic acid metabolism (lines 53–57) and nicotinamide adenine dinucleotide (NAD) biosynthesis (lines 29–31), although a few strains belonging to other clusters also possess such genes. We also detected 11 cluster_CC1-specifc genes (lines 1–9, 11, 12), but almost all of the genes encode hypothetical proteins, and four are considered to be pseudogenes owing to mutations.

### Characteristic Genomic Inversions Found in Strains Belonging to Certain Genomic Clusters

Previous comparative analyses of 13 complete genomes (Zhang et al. 2011) revealed a large-scale inversion distinctively observed in the genomes of eight CC1 strains (all analyzed CC1 strains except BM407; site ii in supplementary fig. S7*A*, Supplementary Material online) and another inversion found only in the genomes of two strains, D9 (CC25) and ST3 (CC28) (site iv in supplementary fig. S7*A*, Supplementary Material online). Our genome sequencing and examination of gene orders through PCR suggested that all cluster_CC1 strains, except for BM407, exhibited a distinctive large-scale change in genome arrangement at site ii (fig. 1*B* and supplementary fig. S7*B*, Supplementary Material online). Furthermore, the change in genome arrangement at site iv was distinctively found in cluster_CC104 strains and in all cluster_CC25 and _CC28 strains (fig. 1*B* and supplementary fig. S7*B*, Supplementary Material online). Five cluster_CC25 and _CC28 strains (89-1591, MNCM04, MNCM25, 4961, and 05HAS68) are likely to harbor additional changes in their genome arrangements (supplementary fig. S7*B*, Supplementary Material online). The breakpoints of these changes in genome arrangement at sites ii and iv were near an inverted duplication of an insertion sequence (IS) element (site ii) and were approximately 60-bp sequences (site iv) (supplementary fig. S7*A*, Supplementary Material online). No clear genes spanning the breakpoints of the respective events were detected, although one gene (of unknown function), which was 114 bp long (including the aforementioned repeats), was generated by the changes at site iv (supplementary fig. S7*A*, Supplementary Material online). Therefore, the effect that these characteristic large-scale rearrangements in the genome produce on their phenotypes remains unclear, but each of these changes has been maintained in cluster_CC1 strains and cluster_CC25, _CC28, and _CC104 strains, respectively.

The eight *S. suis* strains (R61 and reference strains of serotypes 9, 13, 21, 24, 27, 29, and 31) shared at least five common large-scale changes in genome arrangement at sites i, iii, and v–vii that were not observed in the other 50 *S. suis* strains, but were probably present in *Streptococcus parasuis* strains (reference strains of serotypes 20, 22, and 26; fig. 1*B* and supplementary fig. S7, Supplementary Material online). This also supports the conclusion that these eight strains are divergent strains of *S. suis*.

### Conserved Pherotype and Mutations in Competence Genes across Deep-Branching Clades in *S. suis*

Next, we performed genetic and phenotypic analyses of competence, particularly in the strains belonging to clusters_CC1, _CC25, _CC28, and _CC104, to investigate the association of pherotype and competence loss with the diversity of genomic clusters in *S. suis*. Our data identified various mutations in competence genes of certain strains (fig. 1*B*). Among the mutations, genetic conversions between *comRS* regions, which determine the pherotype of *S. suis*, were found (fig. 1*B* and supplementary fig. S8, Supplementary Material online). Two tandemly located genes, *comR* (encoding a regulator of early competence) and *comS* (encoding a competence pheromone precursor), are recognized to be essential for an early competence state (Zaccaria et al. 2014). At least three pherotypes were detected in *S. suis* (ComS_I–III), including two known types (supplementary fig. S8*A*, Supplementary Material online). In *S. suis*, the pherotype was conserved across deep-branching clades (fig. 1*B*), as observed in *S. pneumoniae* (Croucher et al. 2014). Cluster_CC25, _CC28, and _CC104 strains showed a pherotype distinct from that of cluster_CC1 strains (fig. 1*B* and supplementary fig. S8*B*, Supplementary Material online). The results of a genetic transformation test (introduction of plasmid vector pMX1 DNA) indicated that the competence state was induced by N-terminally truncated ComS peptides compatible with their pherotype.

Our genetic transformation test results also showed that certain strains were not transformed with the plasmid vector through competence. Almost all of the nontransformable strains carried mutation(s) in one or some of the competence genes (fig. 1*B*). These mutations are probably associated with no (or low) competency of the strains, although further analyses are required to examine the effects of introducing the mutations into a transformable strain or introducing intact genes into mutated strains. All analyzed cluster_CC1 strains possessed only one copy of *comX* (which encodes the master regulator of competence), whereas all of the other analyzed strains, except for D12 (serotype 9) and three divergent *S. suis* strains, possessed two copies of the gene. However, genetic competence of the cluster_CC1 strains was not affected (fig. 1*B*). Therefore, as previously described in *Streptococcus pneumoniae* (Lee and Morrison 1999), one copy of *comX* in the genome might be adequate to induce genetic competence in *S. suis*. In certain populations within one genomic cluster, as in all serotype 2-cluster_CC25 strains, a specific mutation leading to competence loss (in this case, a truncation of *comGD* due to the frameshift mutation that exchanges a partial region of the gene along with a 13-bp insertion) was maintained among the strains. However, even in the strains belonging to the same clusters, the existence or patterns of mutations in competence genes differed in many cases. Therefore, in *S. suis*, competence loss might occur more frequently than pherotype conversion.

### Profiles of Defense Elements in Genomes Characteristic of Genomic Clusters

**Fig. 2.—:**
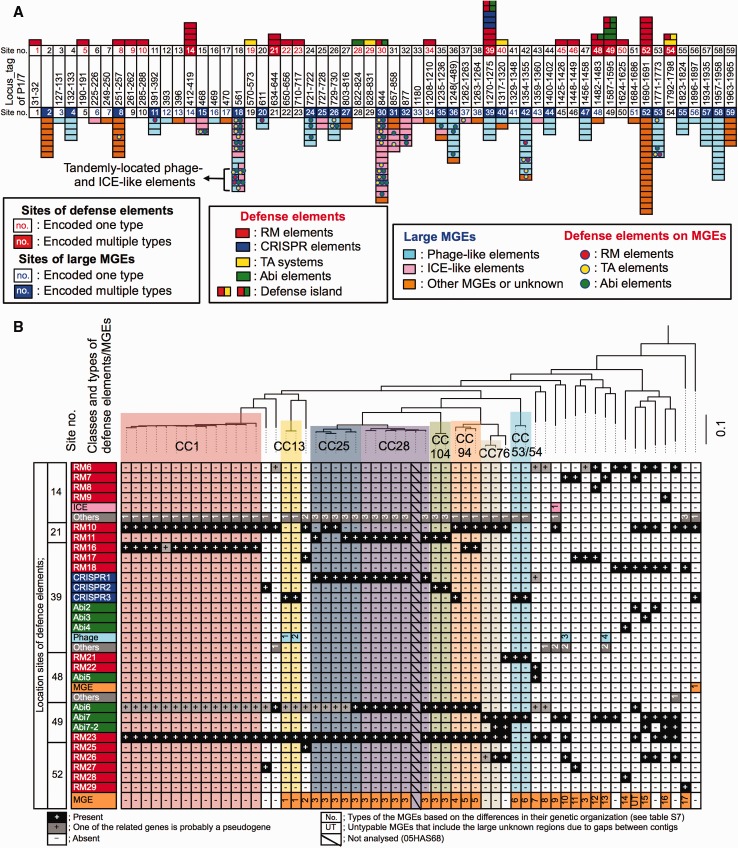
Several loci-shifting defense elements found in *S. suis*. (*A*) Chromosomal positions of all types of defense elements (upper side) and MGEs (lower side) identified in this study. Locus tags on the P1/7 genome (cluster_CC1) of the corresponding sites are displayed. Descriptions of color-coding are listed in the bottom panels. (*B*) Loci shifting in several types of defense elements. Certain loci also encode MGEs or other genetic elements. Presence (+ or no.) or absence (−) of each of the genetic elements is shown below a part of the kSNP tree of 106 strains that represents the node containing only 58 *S. suis* strains. Types of defense elements, MGEs, or other genetic elements at each locus are shown in supplementary table S7, Supplementary Material online. Genome data of 05HAS68 were not analyzed owing to the unfinished draft genome sequence.

Pan-genome analyses in this study indicated that certain cluster-distinctive genes were located on MGEs and related to defense systems (supplementary table S7, Supplementary Material online). Therefore, we investigated the association between variations in the defense elements and MGEs and intraspecific evolution in *S. suis*. Defense elements and MGEs including site-specific recombinases (ICEs, prophages, conjugative plasmids, etc.) were found at 59 chromosomal positions (sites 1–59; fig. 2*A*).

At least 334 MGEs were detected at 44 chromosomal locations, and their genetic organization varied among the strains at 27 sites (fig. 2*A* and supplementary fig. S9, Supplementary Material online). All of the prophage- or ICE-like elements at the same sites shared the same integrase/site-specific recombinase gene (supplementary table S7, Supplementary Material online). The detected MGEs were classified into 178 types according to their genetic organization (supplementary table S7, Supplementary Material online), and some of these MGE types (33/178 types) were shared by several strains (supplementary fig. S9 and table S7, Supplementary Material online). For example, cluster-specific phage-like elements were found in cluster_CC104 (sites 42, 44, and 58) and cluster_CC28 (site 11) strains, and a conjugative plasmid-like element (site 52) was distinctively shared among cluster_CC25, _CC28, and _CC104 strains (supplementary fig. S9, Supplementary Material online). However, most MGE types identified here (145/178 types) were strain-specific. Therefore, MGEs including site-specific recombinases (such as ICEs, prophages, and conjugative plasmids) in *S. suis* were likely to contribute to the diversification within the respective genomic clusters and be capable of persisting in specific genomic clusters in certain cases.

Defense elements related to R-M, CRISPR/Cas, T-A, and Abi systems (hereafter abbreviated as RM, CRISPR, TA, and Abi elements, respectively) were identified at 36 chromosomal positions (RM1–33, CRISPR1–3, TA1–10, Abi1–15), although no gene showed similarity with those related to BREX and DND systems. We also detected either a TA or an Abi element colocalized with an RM element in *S. suis* (sites 30, 39, 48, 49, and 54) (fig. 2*A* and supplementary table S7, Supplementary Material online). The MGEs identified in this study harbored 16 types of the defense elements (RM31–33, TA5–10, Abi8–15), some of which were located at distinct positions (fig. 2*A* and supplementary fig. S10 and table S7, Supplementary Material online). Excluding the defense elements on MGEs, the profiles of defense elements in genomes were common between the strains that were closely related to each other on the kSNP trees, except for cluster_CC25, _CC94, and _CC97 strains (supplementary fig. S10, Supplementary Material online).

CRISPR elements were found in nine clusters among eight CC-clusters and 21 single-node clusters and were classified into three types (supplementary fig. S11, Supplementary Material online). Two of the CRISPR spacers conserved in cluster_CC104 strains (spacer types 60 and 62) showed sequence similarity to an integrase gene related to phages specific to cluster_CC28 strains and serotype 2-cluster_CC25 strains (site 10) (supplementary tables S8 and S9, Supplementary Material online), which resulted in the absence of phage-like elements at site 10 of cluster_104 strains. Taken together, our findings suggest that defense element profiles in the genome have been retained in the respective genomic clusters in *S. suis* in many cases.

### Loci-Shifting Defense Systems against Invading DNA

At six chromosomal locations (sites 14, 21, 39, 48, 49, and 52), variations were detected in defense element types and classes (fig. 2*B* and supplementary fig. S10 and table S7, Supplementary Material online), among which >3 types of defense elements were present at sites 14, 39, and 52 (fig. 2*B* and supplementary table S7, Supplementary Material online). The strains that possessed no defense element at site 14 harbored genetic regions including a gene encoding one of three different types of DNA helicase domain-containing proteins (supplementary table S7, Supplementary Material online). At site 52, more than ten types of MGEs and five types of RM elements were found, although all of the analyzed cluster_CC1 strains did not harbor genetic elements at the site. Intriguingly, CRISPR elements were only located at one genomic locus (site 39). Moreover, this locus encoded the most diverse types of defense elements, including the RM element distinctive of cluster_CC1 strains (supplementary fig. S10, Supplementary Material online).

**Fig. 3.—:**
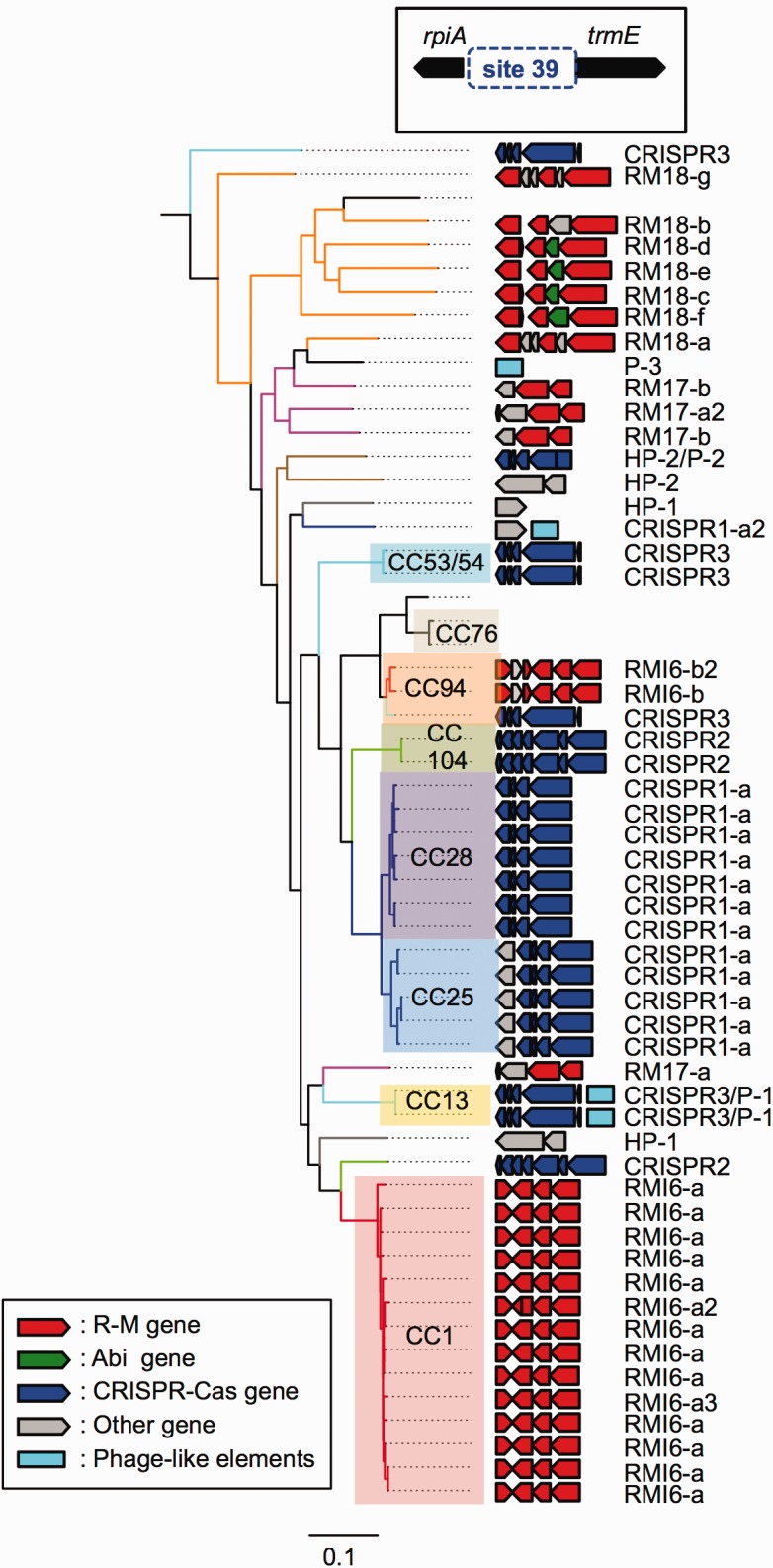
Variable region of defense elements (site 39). This locus is located between the genes *rpiA* (which encodes ribose 5-phosphate isomerase) and *trmE* (which encodes a GTPase and tRNA-U34 5-formylation enzyme). Genetic elements at this locus are shown to the right of a part of the kSNP tree of 106 strains that represents the node containing only 58 *S. suis* strains. Color code of arrows: red, R-M genes; green, Abi genes; blue, CRISPR-Cas genes; gray, other genes. Phage-like elements are represented by cyan rectangles. Their genetic organization and types are detailed in supplementary figure S12 and table S7, Supplementary Material online. Acquisition of defense elements at this locus is indicated by color changes of branches; RM16–18 and CRISPR1–3 are traced using red, magenta, orange, blue, light-green, and light-blue branches, respectively.

We further focused on this genomic locus (site 39). All but nine of the *S. suis* strains analyzed in this study harbored one of three types of RM elements (RM16–18) and three types of CRISPR elements (CRISPR1–3) at this locus (fig. 3 and supplementary fig. S12, Supplementary Material online). Four strains possessed one Abi element (Abi2–4) in addition to an RM element (RM18), and a prophage was located upstream of a CRISPR element (CRISPR3) in two strains. In five strains, prophage-like elements and/or hypothetical protein-coding genes were present instead of defense elements at the locus (supplementary fig. S12, Supplementary Material online). In each type of RM element at this locus, the restriction protein-coding gene was conserved, but variations were present in the entire genetic organization and/or the encoded amino acid sequences of the genes for specificity proteins and DNA methylation proteins (fig. 3 and supplementary fig. S12, Supplementary Material online). The variation in RM elements was not correlated with the presence or absence of prophage at this locus. Genetic organization of CRISPR elements and the amino acid sequences of the genes were shared among the strains carrying the same CRISPR types (supplementary fig. S11, Supplementary Material online). However, CRISPR spacer sequences differed completely between strains that were distant from each other on the genome tree, although three common spacers were observed in cluster_CC104 strains and a reference strain of serotype 15 (supplementary fig. S11 and table S8, Supplementary Material online). Moreover, all clade_2a strains harbored a common IS element at the same position, which was inside of one of the CRISPR repeats (supplementary fig. S12, Supplementary Material online). In many cases, the type and/or genetic organization of the defense element(s) at this locus differed depending on the genomic clusters of the *S. suis* strains (fig. 3). These findings imply that in this bacterium, this locus could serve as a variable region that participates in lineage diversification, although why various and distinct defense elements were mainly shifted at the locus remains unknown.

## Discussion

*Streptococcus suis*, an important swine and zoonotic agent, is considered to have an open pan-genome and shown to develop a competent state. Some of the lineages in this bacterium are suggested to be associated with zoonosis; however, little is known about the evolutionary processes that generate the diversified lineages in *S. suis* and the phenotypic or ecological characteristics of the respective lineages. In this study, to gain insights into the lineage diversification in *S. suis*, we used a retrospective comparative genomics approach, with a focus on large-scale changes in genome arrangements, competence genes, MGEs, and defense elements.

Our genome analyses of *S. suis* indicated the presence of various genomic clusters of this species, which is in close agreement with the results of previous MLST analyses. In addition, the pan-genome analyses identified the characteristic genes of each genomic cluster, which will be useful for analyzing the phenotypic or ecological characteristics of the respective clusters in the future. Among the identified genes, the genes that are distinctively present or absent in the genomic cluster that is recognized as the most hazardous (cluster_CC1) might be linked to increasing the risk of zoonosis. At least one of these NAD biosynthesis genes, *nadR*, has been suggested to be one of the poorly characterized transcription factors involved in the modulation of *S. suis* virulence based on experimental infection of colostrum-deprived pigs (Wilson et al. 2007; Fittipaldi et al. 2012).

Our results showed that large-scale genomic inversions were conserved in certain genomic clusters that are potentially related to zoonosis (clusters_CC1, _CC25, _CC28, and _CC104 in this study) in *S. suis*, although we did not clarify the impact on the phenotype of the respective inversion events. The appearance or change of gene(s) mediated by large-scale inversions is known to affect the virulence phenotypes in *Pseudomonas aeruginosa* (Kresse et al. 2003), *Staphylococcus aureus* (Cui et al. 2012), and *Helicobacter pylori* (Furuta et al. 2011). Therefore, the inversions detected in this study might have remained within the lineages by exerting effects on infectivity and pathogenicity due to the genesis of a small gene (clusters_CC25, _CC28, and _CC104; supplementary fig. S7, Supplementary Material online) or a change in the expression of genes positioned near the rearrangement breakpoints. Of note, among the 14 *S. suis* strains whose genomes were completely sequenced, all ten cluster_CC1 strains contained more copies of the IS element related to IS30 family (four or five copies), which were localized near both breakpoints of a cluster_CC1-distinctive inversion event (supplementary fig. S7*A*, Supplementary Material online), than did the other four noncluster_CC1 strains (0 or 1 copy). This IS element was absent in most of the other noncluster_CC1 strains (31/39 strains). This implied that the genomic profiles of small MGEs such as IS elements might be involved in the occurrence of certain inversion events in *S. suis.*

As shown in *S. pneumoniae* (Carrolo et al. 2009; Croucher et al. 2014), pherotype was conserved across deep-branching clades in *S. suis*. Interpherotype DNA exchange in pneumococcus is reported to be infrequent (Carrolo et al. 2009). Taken together with our data on competence genes in *S. suis*, these findings suggest that the mutations in competence genes, including pherotype-related genes, appear to have occurred at certain frequencies in the respective genomic clusters. Moreover, in the cases where the mutations converted the pherotype, they could separate between two different pherotype populations by affecting DNA exchange and ultimately lead to lineage-splitting. We identified genes related to metabolism and transport of sugar and cell-wall modification that were absent or present in all or almost all of the ComS_II and ComS_III strains in *S. suis* (supplementary table S10, Supplementary Material online). In *Bacillus subtilis*, quorum-sensing type was strongly associated with different ecologically distinct phylogenetic groups (Stefanic et al. 2012). Therefore, if these genes encode factors that result in ecological differences, such as adaptation to changes in the host or environment, these differences can be linked to the pherotype conversion. Further analysis of the linkage between pherotype and ecological isolation, with a focus on these genes, would enhance our understanding of the involvement of pherotype conversion in the evolution and population structure of *S. suis*.

Our data indicate similar or identical profiles of defense elements in the genomes of the *S. suis* strains that belong to the same genomic clusters. In bacteria, a switch between alternative DNA methylation patterns, including those mediated by R-M systems, has been proposed to be capable of splitting clonal populations into epigenetic lineages (Casadesús and Low 2006). In fact, distinct profiles of R-M systems in genomes depending on their phylogeny have been reported in *Neisseria meningitidis* (Budroni et al. 2011) and *Burkholderia pseudomallei* (Nandi et al. 2015). RM elements were the most abundant defense elements in *S. suis*, and thus the epigenetic status that depends on the R-M profile might be associated with phenotypic differences between various lineages in this species. In this study, the possession of a CRISPR element appeared to influence the profile of MGEs, but no clear correlation existed between the total number of MGEs and defense elements in genomes as well as the number of TA and Abi elements identified in this study (supplementary figs. S9 and S10, Supplementary Material online). Therefore, the respective defense systems must be functionally analyzed in future studies to elucidate the relationship between defense element profiles and diversification of genomic clusters in *S. suis*.

In *S. suis*, cassette-like variations of self–nonself-discriminating defense elements were found at several chromosomal locations. Similar variation of only RM elements in other bacterial species has been reported previously (Sibley and Raleigh 2004; Eutsey et al. 2015). In *S. pneumoniae*, such loci have been proposed to play a role in the fine-tuning of the extent of genomic plasticity (Eutsey et al. 2015). One of these loci in *S. suis* is a previously undetected variable region where not only RM elements, but also CRISPR elements, Abi elements, prophages, and/or other genes, are replaced with each other. This is an unrecognized mechanism of acquiring defense systems, which differs completely from that of a “defense island” where several distinct classes of defense elements are colocated (Makarova et al. 2011, 2013). In certain bacterial species, including *Escherichia coli* and *Campylobacter jejuni* (Didelot et al. 2012; Yahara et al. 2014), hot-regions, where DNA is transferred frequently between isolates, were identified. Most of these hot-regions contributed to the variability of surface antigenicity and host specificity in the species, and the variability was probably not due to specific hotspots, the sites where recombination breakpoints occur frequently. In the variable region of defense elements found in *S. suis*, the breakpoints differed according to the type of element, although AT-rich sequences including one consensus region (ATCCCxxAxCTGxxCTTTTxxxTTxxTCATxCxxTGT) were present around breakpoints shifting the genetic elements (supplementary fig. S13, Supplementary Material online). Because there are no data about the recombination rate at this locus, it remains unclear whether this locus is a hot-region. However, our data suggest that this locus could serve as a variable region for protection against fitness reduction by invading DNAs that has been evolved in *S. suis*, which is exposed to continual horizontal movements of DNA mediated by MGEs and genetic competence. Interestingly, our data indicate that the shift of defense systems at the locus was coincident with the branching of the genomic clusters in many cases. However, the number of strains used in this study is too small for analyzing the population structure. Thus, to further understand the role of this locus in intraspecific evolution, several additional analyses must be performed with a large number of strains, including the quantification of horizontal transfer and homologous recombination in each genomic cluster by using programs such as GUBBINS (Croucher et al. 2015) or CLONALORIGIN (Ansari and Didelot 2014). In this study, several loci that shift defense elements were revealed through comprehensive analyses of the defense elements and their locations. The variable region of defense elements across several classes found in *S. suis* has not yet been detected in other bacterial species because little information is available on the intraspecific variations in defense elements and their locations. Thus, future comparative genome analysis, with a focus on the defense elements, will help elucidate whether the use of such a variable region of defense elements is a mechanism specific to *S. suis*. If other bacterial species are also found to possess a similar variable region of defense elements, our data should serve as a clue that facilitates our comprehension of bacterial intraspecific diversification.

## Supplementary Material

Supplementary data are available at *Genome Biology and Evolution* online.

## Supplementary Material

Supplementary Data
