# Supplementary material for: A Locus Encoding Variable Defense Systems against Invading DNA Identified in Streptococcus suis
Source: Genome Biol Evol. 2017 Apr 1;9(4):1000–12. doi: 10.1093/gbe/evx062 (PMC5398294; doi:10.1093/gbe/evx062)
Supplement: Supplementary Data [file evx062_Supp.pdf]

Table S1. *S. suis* strains analysed in this study and the general properties of their genomes

Strain[a]	Source	Diagnosis	Origin	Year	Sero-type	ST (CC)	Status of genome sequencing	No. of contigs	N50 (bp)	Total Length (Mbp)	No. of coding DNA sequence	Accession no.
Determend the genome sequences in this study												
NCTC 10237	Diseased pig	No information	The Netherlands	1961	1	13 (13)	Draft	111	44,411	2.00	1,949	DRA001201
2651	Diseased pig	No information	The Netherlands	1980-82	1/2	56	Draft	63	82,928	1.99	1,932	DRA001201
4961	Diseased pig	Not meningitis	Denmark	1980-82	3	35 (28)	Draft	119	48,337	2.03	1,998	DRA001201
6407	Diseased pig	No information	Denmark	1980-82	4	54 (54/53)	Draft	64	110,903	2.26	2,219	DRA001201
11538	Diseased pig	Not meningitis	Denmark	1980-82	5	53 (54/53)	Draft	49	92,411	2.51	2,431	DRA001201
2524	Diseased pig	No information	Denmark	1980-82	6	55	Draft	106	46,897	1.89	1,790	DRA001201
8074	Diseased pig	No information	Denmark	1980-82	7	29 (25)	Draft	143	35,243	2.07	2,023	DRA001201
14636	Diseased pig	No information	Denmark	1980-82	8	87	Draft	74	72,902	2.21	2,144	DRA001201
22083	Diseased pig	Meningitis	Denmark	before 1989	9	82	Draft	63	108,742	1.99	1,932	DRA001201
4417	Diseased pig	Meningitis	Denmark	before 1989	10	78	Draft	251	58,801	2.25	2,199	DRA001201
12814	Diseased pig	Septicemia	Denmark	before 1989	11	91	Draft	122	61,978	2.08	2,008	DRA001201
8830	Diseased pig	Septicemia	Denmark	before 1989	12	775[c]	Draft	109	80,529	2.21	2,144	DRA001201
10581	Diseased pig	Arthritis	Denmark	before 1989	13	71	Draft	83	87,339	2.35	2,226	DRA001201
13730	Patient	Meningitis	The Netherlands	before 1989	14	6 (1)	Draft	161	28,299	1.96	1,890	DRA001201
NCTC 10446	Diseased pig	Probably meningitis	The Netherlands	before 1989	15	81	Draft	79	75,002	2.03	1,902	DRA001201
2726	Diseased pig	Meningitis	Denmark	before 1989	16	73	Draft	134	50,159	2.23	2,164	DRA001201
93A	Pig	Clinically healthy	Canada	before 1989	17	76 (76)	Draft	340	20,766	2.36	2,303	DRA001201
NT77	Pig	Clinically healthy	Canada	before 1989	18	79	Draft	327	22,030	2.30	2,228	DRA001201
42A	Pig	Clinically healthy	Canada	before 1989	19	76 (76)	Draft	268	17,503	2.18	2,096	DRA001201
86-5192[b]	Diseased calf	Pneumonia	USA	before 1989	20	N.D.[d]	Draft	294	15,648	2.03	2,088	DRA001201
14A	Pig	Clinically healthy	Canada	before 1989	21	481	Draft	299	28,133	2.42	2,333	DRA001201
88-1861[b]	Diseased pig	Septicemia	Canada	before 1989	22	N.D.[d]	Draft	487	14,389	2.26	2,375	DRA001201
89-2479	Diseased pig	Pneumonia	Canada	before 1991	23	483[c] (94)	Draft	98	161,510	2.18	2,131	DRA001201
88-5299A	Diseased pig	Meningitis	Canada	before 1991	24	68	Draft	165	57,415	2.37	2,308	DRA001201
89-3576-3	Diseased pig	Pneumonia	Canada	before 1991	25	69	Draft	100	64,146	2.14	2,058	DRA001201
89-4109-1[b]	Diseased pig	Arthritis	Canada	before 1991	26	N.D.[d]	Draft	118	63,826	2.19	2,122	DRA001201
89-5259	Diseased pig	Meningitis	Canada	before 1991	27	72	Draft	320	31,744	2.51	2,484	DRA001201
89-590	Diseased pig	Septicemia	Canada	before 1991	28	75	Draft	93	58,376	2.17	2,104	DRA001201
92-1191	Diseased pig	Meningitis	Canada	before 1995	29	92	Draft	305	25,897	2.40	2,318	DRA001201
92-1400	Diseased pig	Aborted fetus	Canada	before 1995	30	77	Draft	205	54,817	2.31	2,228	DRA001201
92-4172	Diseased calf	Cerebral edema	Canada	before 1995	31	70	Draft	368	16,767	2.26	2,295	DRA001201
EA1172.91[b]	Diseased pig	Septicemia	Canada	before 1995	32	N.D.[d]	Draft	313	17,846	2.26	2,220	DRA001201
EA1832.92[b]	Diseased lamb	Arthritis	Canada	before 1995	33	N.D.[d]	Draft	150	65,797	2.03	1,908	DRA001201
92-2742[b]	Diseased pig	Aborted fetus	Canada	before 1995	34	N.D.[d]	Draft	199	63,943	2.37	2,324	DRA001201

Table S1. *S. suis* strains analysed in this study and the general properties of their genomes

Strain[a]	Source	Diagnosis	Origin	Year	Sero-type	ST (CC)	Status of genome sequencing	No. of contigs	N50 (bp)	Total Length (Mbp)	No. of coding DNA sequence	Accession no.
DAT260	Diseased pig	Meningitis	Japan	1992	2	28 (28)	Draft	90	75,085	2.10	2,050	DRA001201
DAT290	Pig	Clinically healthy	Japan	2005-2006	4	94 (94)	Draft	69	94,530	2.20	2,158	DRA001201
DAT273	Patient	Meningitis	Japan	2002	2	1 (1)	Draft	55	170,301	2.03	1,932	DRA001201
DAT292	Pig	Clinically healthy	Japan	2005-2006	2	28 (28)	Draft	91	75,094	2.10	2,079	DRA001201
DAT234	Diseased pig	Endocarditis	Japan	2006	UT	108 (94)	Draft	66	127,772	2.19	2,137	DRA001201
MNCM04	Patient	Meningitis	Thailand	2000	2	25 (25)	Draft	176	33,326	2.15	2,095	DRA001201
MNCM07	Patient	Meningitis	Thailand	2000	14	11 (1)	Draft	64	82,613	2.05	1,938	DRA001201
MNCM21	Patient	Meningitis	Thailand	1998	2	101 (104)	Draft	57	91,578	2.10	2,053	DRA001201
MNCM25	Patient	Meningitis	Thailand	2001	2	102 (25)	Draft	194	33,215	2.14	2,096	DRA001201
MNCM43	Patient	Endocarditis	Thailand	2002	2	28 (28)	Draft	90	68,286	2.12	2,065	DRA001201
MNCM50	Patient	Pulmonary edema	Thailand	2002	2	104 (104)	Draft	66	89,527	2.10	2,060	DRA001201
NIAH11434	Diseased pig	Meningitis	Japan	1987	2	1 (1)	Draft	61	130,791	2.04	1,935	DRA001201
NIAH11435	Pig	Clinically healthy	Japan	1987	2	28 (28)	Draft	106	60,803	2.11	2,045	DRA001201
Publicly available genome sequences of *S. suis* strains used in this study												
S735[T] (NCTC 10234)	Diseased pig	Pneumonia	The Netherlands	1961	2	1 (1)	Complete			1.98	1,879	CP003736
P1/7	Diseased pig	Meningitis	UK	before 1994	2	1 (1)	Complete			2.01	1,918	AM946016
BM407	Patient	Meningitis	Vietnam	2004	2	1 (1)	Complete			2.17	2,062	FM252032
SC84	Patient	Streptococcal toxic-shock syndrome (STSS)	China	2005	2	7 (1)	Complete			2.10	2,014	FM252031
98HAH33	Patient	STSS	China	1998	2	7 (1)	Complete			2.10	2,017	CP000408
05ZYH33	Patient	STSS	China	2005	2	7 (1)	Complete			2.10	2,017	CP000407
GZ1	Patient	Septicemia	China	2005	2	1 (1)	Complete			2.04	1,950	CP000837
A7	Diseased pig	Probably meningitis	China	2007	2	7 (1)	Complete			2.04	1,938	CP002570
JS14	Diseased pig	No information	China	before 2011	14	7 (1)	Complete			2.14	2,063	CP002465
ST3	Diseased pig	Pneumonia	China	before 2011	3	35 (28)	Complete			2.03	1,958	CP002633
ST1	No information	No information	China	before 2011	1	13 (13)	Complete			2.03	1,986	CP002651
SS12	No information	No information	China	before 2011	1/2	1 (1)	Complete			2.10	2,035	CP002640
D9	No information	No information	China	before 2011	7	29 (25)	Complete			2.18	2,108	CP002641
D12	Diseased pig	Pneumonia	China	2006	9	619	Complete			2.18	2,100	CP002644
89/1591	Diseased pig	Meningitis and septicemia	Canada	before 1989	2	25 (25)	Draft			2.14	2,093	AAFA03000001-AAFA03000082
R61	Diseased pig	No information	China	2008	ND	776	Draft			2.39	2,329	AEYY01000001-AEYY01000053
05HAS68	Pig	Clinically healthy	China	2005	2	28 (28)	Draft (incomplete)			1.64	1,612	AARD01000001-AARD01000332

a: The strains isolated from pigs and humans are shown in red and blue characters, respectively. Serotype reference strains are highlighted in light blue. S735 is the *S. suis* type strain.

b: Regarded as non-*S. suis*.

c: Different from the types in a previous study (King et al. 2002).

d: Not determined.

Table S2. Streptococcal strains analysed together in this study and the general properties of their genome sequences

Species	Strain	Source or isolation site	Origin	Total Length (Mbp)	No. of coding DNA sequence[a]	Status of genome sequencing	Accession no.
Streptococcus agalactiae	NEM316	Patient with septicemia	No information	2.21	2,140	Complete	AL732656
Streptococcus pyogenes	SF370	Patient with wound infection	No information	1.85	1,820	Complete	AE004092
Streptococcus pneumoniae	TIGR4	Blood from a patient	Norway	2.16	2,201	Complete	AE005672
Streptococcus mutans	UA159	Child with dental caries	USA	2.03	1,958	Complete	AE014133
Streptococcus equi subsp. *zooepidemicus*	ATCC 35246	Dead pig	China	2.17	2,141	Complete	CP002904
Streptococcus equi subsp. *equi*	4047	Horse with strangles	UK	2.25	2,257	Complete	FM204883
Streptococcus mitis	B6	From a hospital	Germany	2.15	2,068	Complete	FN568063
Streptococcus uberis	0140J	Milk from a bovine with mastitis	England	1.85	1,821	Complete	AM946015
Streptococcus intermedius	JTH08	No information	No information	1.93	1,869	Complete	AP010969
Streptococcus salivarius	JIM8780	Human blood	No information	2.12	1,988	Complete	FR873481
Streptococcus sanguinis	SK36	Dental plaque	No information	2.39	2,303	Complete	CP000387
Streptococcus thermophilus	CNRZ1066	Yogurt	France	1.8	1,874	Complete	CP000024
Streptococcus dysgalactiae subsp. *equisimilis*	GGS_124	Patient with STSS	No information	2.11	2,115	Complete	AP010935
Streptococcus gordonii	Challis substr. CH1	Laboratory strain	No information	2.2	2,093	Complete	CP000725
Streptococcus oralis	Uo5	Human oral cavity	Hungary	1.96	1,854	Complete	FR720602
Streptococcus infantarius	CJ18	Fermented camel milk	Kenya	2.01	1,977	Complete	CP003295
Streptococcus parasanguinis	ATCC 15912	Human throat	No information	2.15	1,976	Complete	CP002843
Streptococcus pseudopneumoniae	IS7493	Patient	No information	2.2	2,263	Complete	CP002925
Streptococcus gallolyticus subsp. *gallolyticus*	ATCC BAA-2069	Blood from a patient with endocarditis	Germany	2.38	2,291	Complete	FR824043
Streptococcus macedonicus	ACA-DC 198	Naturally fermented Greek Kasseri cheese	Greece	2.14	2,162	Complete	HE613569
Streptococcus pasteurianus	ATCC 43144	Human blood	Belgium	2.1	2,042	Complete	AP012054
Streptococcus dysgalactiae subsp. *dysgalactiae*	ATCC 27957	Bovine udder infection	No information	2.14	2,186	Draft	CM001076
Streptococcus parauberis	NCFD 2020	Milk from a bovine with mastitis	No information	2.16	2,150	Draft	AEUT02000001
Streptococcus iniae	9117	Blood from a patient with cellulitis	No information	2.08	2,001	Draft	JH930418
Streptococcus porcinus	Jelinkova 176	Hemorrhagic swine lymph nodes	No information	2.03	1,956	Draft	AEUU02000001
Streptococcus macacae	NCTC 11558	Dental plaque of monkey	No information	1.91	1,882	Draft	AEUW02000001
Streptococcus urinalis	2285-97	Urine from a patient with cystitis	USA	2.13	2,164	Draft	AEUZ02000001
Streptococcus vestibularis	ATCC 49124	Human oral cavity	UK	1.84	1,781	Draft	GL831112-GL831132
Streptococcus cristatus	ATCC 51100	Human periodontal abscess	No information	1.97	1,922	Draft	GL732518-GL732522
Streptococcus equinus	ATCC 9812	Horse faeces	No information	1.72	1,688	Draft	GL698429-GL698448
Streptococcus peroris	ATCC 700780	Tooth surface and pharynx of a patient with Kawasaki disease	Japan	1.63	1,608	Draft	GL732463-GL732465
Streptococcus infantis	ATCC 700779	Pharynx of a healthy child	Japan	1.88	1,843	Draft	GL732439-GL732448
Streptococcus criceti	HS-6	Oral cavity of a hamster	No information	2.42	2,241	Draft	AEUV02000001-AEUV02000002
Streptococcus australis	ATCC 700641	Saliva from children	Australia	2.13	2,000	Draft	GL636091-GL636096
Streptococcus pseudoporcinus	LQ 940-04	Genitourinary tract of a human	Canada	2.13	2,039	Draft	AEUY02000001-AEUY02000005
Streptococcus ratti	DSM 20564	Caries lesion in laboratory rat	No information	2.08	2,040	Draft	AJTZ01000001-AJTZ01000006
Streptococcus canis	FSL Z3-227	Bovine mastitis	Belgium	2.27	2,222	Draft	AIDX01000001-AIDX01000003
Streptococcus ictaluri	707-05	Catfish with osteolytic bone lesions	USA	2.23	2,401	Draft	AEUX02000001-AEUX02000008
Streptococcus downei	F0415	Human oral cavity	No information	2.24	2,164	Draft	AEKN01000001-AEKN01000017
Streptococcus constellatus subsp. *constellatus*	SK53	Probably from a patient with purulent pleurisy	No information	1.84	1,820	Draft	AICQ01000001-AICQ01000054
Streptococcus anginosus	SK52	Tissue, human throat	No information	1.89	1,858	Draft	AFIM01000001-AFIM01000109
Streptococcus constellatus subsp. *pharyngis*	SK1060	Throat of a human with pharyngitis	UK	1.96	2,174	Draft	AFUP01000001-AFUP01000010

a: Based on the results analysed together with the genome sequence determined in this study to compare the results obtained by the same method.

Table S3. ANIb values between two genomes among 61 *S. suis* strains including serotype 20, 22, 26, and 33 reference strains

ANIm values between query and subject genomes

Subject (columns); Query (rows)

Strain	Serotype	ST (CC)	98HAH33	05ZYH33	SC84	A7	SS12	P1/7	DAT273	NIAH11434	JS14	GZ1	BM407	S735	13730	MNCM07	NCTC10446	14636	NCTC10237	ST1	2651	89-1591	MNCM04	MNCM25	8074	D9	4961	ST3	MNCM43	DAT292	DAT260	05HAS68	NIAH11435	MNCM21	MNCM50	89-2479	DAT234	DAT290	93A	42A	NT77	6407	11538	8830	12814	92-1400	89-590	2726	2524	89-3576-3	4417	D12	10581	89-5259	88-5299A	R61	92-1191	14A	92-4172	22083	86-5192	89-4109-1	88-1861	EA1832.92
EA1832.92	33		79.67	79.66	79.74	79.75	80.28	79.72	79.70	79.74	79.75	79.74	79.70	79.75	79.75	79.65	79.65	79.68	79.83	79.47	79.70	79.67	79.68	79.65	79.69	79.65	79.63	79.65	79.70	79.66	79.65	79.76	79.73	79.57	79.61	79.61	79.53	79.59	79.59	79.78	79.80	79.64	79.75	79.86	79.63	79.77	79.67	79.83	79.80	79.79	79.83	79.95	79.65	79.66	79.64	79.79	79.57	79.44	79.82	79.85	77.79	77.99	77.91	100
88-1861	22		83.81	83.76	83.86	84.04	84.03	84.06	84.25	83.98	84.09	84.04	83.96	83.99	84.03	83.93	83.62	83.60	83.44	83.73	83.89	83.92	83.69	83.97	83.88	83.86	83.83	83.68	83.89	83.84	84.02	83.94	83.93	83.74	84.01	83.76	84.03	82.82	84.00	83.92	84.02	83.75	83.92	83.81	84.06	83.65	83.57	83.78	83.84	83.91	83.81	83.60	83.13	83.73	83.56	83.67	83.70	83.68	83.76	83.44	94.40	94.69	100	78.10
89-4109-1	26		83.52	83.45	83.55	83.34	83.23	83.72	83.79	83.96	83.80	83.36	83.66	83.38	83.38	83.71	83.17	83.18	83.26	84.08	84.21	84.19	83.46	83.92	83.46	83.42	83.80	83.75	83.98	83.78	83.33	83.87	83.87	83.89	83.95	83.86	83.17	82.08	83.62	83.80	83.82	83.51	83.35	83.95	83.84	83.55	83.61	83.72	83.75	83.69	83.62	83.22	83.42	83.79	83.54	83.42	83.42	83.25	83.56	83.92	97.27	100	94.62	77.85
86-5192	20		83.75	83.71	83.78	83.58	83.48	83.83	83.85	83.90	83.85	83.58	83.74	83.58	83.59	84.06	83.16	83.12	83.15	83.80	83.89	83.91	83.39	84.06	83.46	83.46	83.78	83.75	83.75	83.76	83.26	83.77	83.76	83.77	83.91	83.71	83.23	81.83	83.67	83.84	83.81	83.65	83.53	83.72	83.70	83.43	83.51	83.64	83.63	83.55	83.55	83.19	83.35	83.65	83.46	83.37	83.31	83.60	83.60	83.74	100	97.30	94.65	77.98
22083	9	82	95.11	95.04	95.20	95.18	95.22	95.22	95.22	95.24	95.21	95.27	95.21	95.20	95.22	95.21	95.27	95.24	95.15	95.14	95.17	95.04	95.29	95.29	95.28	95.24	95.27	95.34	95.20	95.20	95.19	95.18	95.19	95.12	95.13	95.15	95.17	95.29	94.63	94.70	94.63	95.14	95.11	95.27	95.18	95.16	95.45	95.41	95.59	95.43	95.52	95.64	93.41	93.02	93.27	93.71	93.58	93.41	95.86	100	83.41	83.45	83.52	79.59
92-4172	31	70	95.40	95.37	95.51	95.67	95.56	95.72	95.54	95.55	95.42	95.77	95.56	95.74	95.71	95.52	95.78	95.62	95.75	95.76	95.69	95.47	95.66	95.65	95.19	95.58	95.78	95.77	95.61	95.51	95.67	95.18	95.53	95.55	95.56	95.66	95.66	95.62	94.43	94.45	94.86	95.66	95.68	95.72	95.62	95.41	95.83	95.92	96.24	95.82	95.38	95.67	93.88	93.26	93.42	93.62	93.83	93.76	100	95.86	83.40	83.39	83.42	79.69
14A	21	481	93.93	93.92	94.03	94.17	94.13	94.07	94.15	94.02	94.09	94.12	94.17	94.04	94.05	94.20	93.84	94.18	94.14	94.08	93.93	94.40	94.46	94.42	94.08	94.48	94.29	94.36	94.12	94.27	94.40	93.96	94.29	94.27	94.26	94.12	93.98	94.00	94.21	94.48	94.27	94.06	94.09	94.25	94.24	93.87	93.67	94.39	94.34	94.08	94.13	94.14	94.52	94.61	94.39	94.65	95.45	100	93.53	93.26	83.44	83.47	83.48	79.69
92-1191	29	92	93.97	93.98	94.08	94.30	94.30	94.15	94.11	94.06	94.15	94.24	94.13	94.13	94.19	94.14	94.11	94.50	94.09	94.21	94.08	94.29	94.27	94.24	93.87	94.29	94.40	94.43	94.12	94.18	94.35	93.99	94.33	94.39	94.37	94.21	94.17	94.22	94.48	94.58	94.57	94.16	94.18	94.15	94.33	93.88	94.09	94.36	94.77	94.21	94.33	94.18	94.56	94.58	94.32	94.61	100	95.40	93.92	93.66	83.30	83.27	83.60	79.65
R61	UT	776	93.65	93.64	93.77	94.00	93.87	93.77	93.86	93.82	94.00	93.99	93.74	93.78	93.75	93.76	93.65	94.27	94.07	94.09	93.85	93.94	94.09	94.11	93.76	94.13	94.24	94.23	94.09	94.15	94.12	93.73	94.16	94.17	94.18	93.93	94.02	94.04	93.69	93.81	94.04	93.95	93.92	94.10	94.20	93.85	93.76	94.25	94.45	94.16	94.23	94.17	94.90	95.22	94.66	100	94.66	94.82	93.66	93.47	83.26	83.26	83.75	80.24
88-5299A	24	68	93.44	93.41	93.53	93.63	93.58	93.56	93.74	93.64	93.81	93.67	93.69	93.55	93.60	93.67	93.53	93.83	93.56	93.59	93.57	93.64	93.72	93.69	93.47	93.84	93.84	93.85	93.69	93.87	93.72	93.39	93.88	93.70	93.67	93.39	93.49	93.45	93.47	93.49	93.75	93.62	93.63	93.68	93.91	93.42	93.48	93.79	94.09	93.86	93.93	93.75	94.66	94.72	100	94.72	94.31	94.36	93.32	93.02	83.50	83.57	83.59	79.56
89-5259	27	72	94.06	94.05	94.18	94.26	94.29	94.24	94.37	94.39	94.25	94.35	94.19	94.21	94.27	94.24	94.06	94.50	94.17	94.41	93.98	94.27	94.33	94.37	93.96	94.51	94.42	94.45	94.48	94.37	94.29	93.96	94.47	94.52	94.50	94.16	94.31	94.33	93.92	93.95	94.31	93.99	94.15	94.41	94.49	94.38	94.08	94.27	94.67	94.47	94.33	94.32	94.54	100	94.79	95.16	94.51	94.82	93.83	93.48	83.58	83.77	83.77	79.61
10581	13	71	93.71	93.68	93.82	93.99	93.79	93.98	93.92	93.83	93.82	93.91	93.92	93.98	94.01	93.73	93.85	93.67	93.96	93.97	94.11	93.71	93.94	93.95	93.23	94.00	94.13	94.14	93.96	93.96	93.58	92.85	93.73	93.90	93.91	93.85	93.85	93.89	93.15	93.02	94.02	93.75	93.75	93.95	94.11	93.63	93.65	93.64	94.43	94.01	94.13	93.92	100	94.64	94.54	95.00	94.56	94.61	93.71	93.39	83.68	83.90	83.66	79.70
D12	9	619	95.80	95.79	95.93	96.02	96.01	96.01	95.99	95.97	95.95	96.11	95.95	96.01	96.05	95.99	96.07	95.93	96.04	96.03	95.93	96.02	96.05	96.01	95.81	96.02	96.22	96.16	96.12	96.17	96.11	95.77	96.13	96.15	96.16	96.12	96.09	96.09	94.99	95.05	95.44	95.80	95.86	96.08	96.08	95.98	96.36	96.28	96.50	96.65	97.06	100	94.15	94.35	93.79	94.20	94.36	94.37	95.72	95.62	83.68	83.79	83.70	79.66
4417	10	78	95.92	95.90	96.04	96.19	96.10	96.20	96.12	96.21	96.18	96.28	96.05	96.22	96.18	96.07	96.26	96.12	96.25	96.25	96.17	96.25	96.23	96.27	95.51	96.27	96.42	96.41	96.29	96.39	96.28	95.54	96.32	96.19	96.19	96.25	96.28	96.22	94.78	94.79	95.61	96.22	96.17	96.23	96.22	95.97	96.30	96.48	96.75	96.85	100	97.01	94.09	94.49	94.00	94.29	94.46	94.25	95.67	95.52	83.38	83.26	83.87	79.70
89-3576-3	25	69	95.99	95.97	96.11	96.18	96.17	96.17	96.17	96.13	96.12	96.24	96.07	96.18	96.17	96.11	96.14	96.02	95.99	96.09	96.19	96.06	96.16	96.14	95.73	96.20	96.21	96.33	96.23	96.17	96.18	95.87	96.24	96.27	96.28	96.27	96.14	96.21	95.18	95.19	95.82	96.01	96.27	96.38	96.26	96.15	96.19	96.67	96.82	100	97.14	96.65	94.15	94.57	94.05	94.36	94.36	94.29	96.02	95.50	83.50	83.58	83.13	79.58
2524	6	55	96.11	96.08	96.23	96.22	96.26	96.24	96.23	96.23	96.26	96.30	96.22	96.23	96.22	96.18	96.37	96.35	96.29	96.31	96.24	96.14	96.20	96.21	96.43	96.38	96.49	96.50	96.29	96.28	96.28	96.30	96.26	96.31	96.31	96.49	96.50	96.50	95.84	95.80	95.99	96.53	96.52	96.43	96.47	96.25	96.51	97.01	100	96.79	96.83	96.54	94.64	94.61	94.10	94.61	94.79	94.63	96.26	95.57	83.21	83.32	83.56	79.65
2726	16	73	95.37	95.35	95.50	96.13	95.68	96.15	95.67	95.63	95.57	96.10	95.70	96.12	96.12	95.67	96.20	95.78	96.07	96.18	96.18	95.59	95.80	95.80	95.30	95.70	96.27	96.29	95.65	95.63	95.74	95.81	95.65	95.63	95.62	95.85	95.75	95.83	95.18	95.15	95.61	96.23	96.28	95.79	95.80	95.48	95.74	100	96.89	96.27	96.24	95.97	93.46	93.83	93.26	93.74	94.03	93.98	95.59	95.39	83.18	83.32	83.78	79.56
89-590	28	75	96.42	96.42	96.56	96.65	96.71	96.68	96.64	96.56	96.57	96.65	96.64	96.65	96.68	96.51	96.60	96.33	96.43	96.42	96.37	96.34	96.36	96.37	96.09	96.34	96.48	96.58	96.46	96.43	96.47	96.11	96.44	96.54	96.53	96.48	96.25	96.35	95.26	95.28	95.90	96.41	96.48	96.49	96.55	96.10	100	96.30	96.48	96.15	96.33	96.28	93.76	94.41	93.51	94.08	94.48	93.85	96.16	95.52	83.75	83.72	83.66	79.60
92-1400	30	77	96.08	96.07	96.19	96.18	96.26	96.39	96.36	96.36	96.26	96.21	96.20	96.36	96.41	96.18	96.45	96.11	96.23	96.24	96.29	96.13	96.16	96.16	95.93	95.91	96.44	96.43	95.95	96.32	96.17	95.90	96.27	96.25	96.24	95.79	95.82	95.72	94.85	95.20	95.29	96.28	96.25	96.16	96.35	100	96.22	95.95	96.17	96.01	96.19	95.91	93.56	94.48	93.45	93.84	93.88	93.92	95.53	95.21	82.99	83.35	83.45	79.54
12814	11	91	96.61	96.59	96.74	96.78	96.72	96.84	96.85	96.82	96.78	96.77	96.78	96.82	96.84	96.67	96.77	96.54	96.65	96.65	96.59	96.48	96.61	96.61	96.25	96.56	96.75	96.73	96.75	96.73	96.73	96.39	96.73	96.74	96.73	96.49	96.55	96.50	95.34	95.36	95.95	96.63	96.60	96.87	100	96.28	96.41	96.30	96.38	96.14	96.23	96.09	94.36	94.62	93.98	94.32	94.50	94.31	95.80	95.17	83.34	83.66	83.44	79.58
8830	12	775	96.53	96.50	96.66	96.69	96.66	96.73	96.69	96.67	96.63	96.73	96.68	96.73	96.73	96.61	96.70	96.61	96.53	96.52	96.46	96.48	96.56	96.55	96.21	96.58	96.74	96.72	96.67	96.66	96.67	96.36	96.65	96.56	96.56	96.41	96.47	96.46	95.45	95.40	95.99	96.63	96.62	100	96.88	96.10	96.32	96.23	96.37	96.27	96.16	96.06	94.13	94.55	93.83	94.11	94.31	94.42	95.93	95.25	82.94	83.18	83.48	79.73
11538	5	53	95.89	95.93	96.02	96.48	96.04	96.48	96.06	96.03	95.94	96.47	96.09	96.50	96.46	96.06	96.42	95.76	96.11	96.21	96.27	96.02	96.09	96.12	95.83	96.03	96.73	96.72	96.04	96.21	96.18	96.25	96.18	96.27	96.28	95.92	95.90	95.93	95.00	95.23	95.45	99.21	100	95.66	95.74	95.26	95.51	95.82	95.98	95.38	95.42	95.13	93.02	93.46	92.87	93.19	93.47	93.46	94.94	94.52	83.90	84.37	83.49	79.60
6407	4	54	96.43	96.40	96.56	97.01	96.53	97.00	96.56	96.54	96.38	96.97	96.64	97.00	96.97	96.61	96.65	96.17	96.46	96.52	96.67	96.52	96.62	96.62	96.15	96.50	97.07	97.04	96.38	96.54	96.59	96.60	96.50	96.58	96.59	96.37	96.32	96.34	95.33	95.60	95.80	100	99.65	96.04	96.14	95.68	95.92	96.12	96.43	95.54	95.89	95.58	93.54	93.74	93.15	93.53	93.71	93.91	95.33	95.05	83.45	84.15	83.68	79.40
NT77	18	79	95.22	95.21	95.35	95.96	95.38	96.12	95.44	95.42	95.44	96.09	95.32	96.08	96.12	95.28	95.99	95.00	95.97	96.01	95.95	95.55	95.77	95.78	95.61	95.74	96.55	96.57	95.76	95.67	95.30	95.27	95.32	95.56	95.57	97.40	97.42	97.41	98.12	98.00	100	95.70	95.68	95.16	95.15	94.51	95.12	95.28	95.94	95.03	95.08	94.91	93.35	93.60	93.35	93.30	93.98	93.91	94.34	94.49	83.55	84.15	83.72	79.38
42A	19	76	94.95	94.96	95.11	95.87	95.18	95.87	95.27	95.16	95.15	95.90	95.16	95.84	95.87	95.18	95.86	95.23	95.64	95.72	95.75	95.53	95.80	95.77	95.97	95.71	96.43	96.42	95.61	95.51	95.56	95.88	95.58	95.56	95.56	97.20	97.22	97.27	99.56	100	98.21	95.72	95.70	94.98	94.94	94.81	94.93	95.14	95.76	94.74	94.91	94.81	93.27	93.52	93.28	93.33	94.06	94.28	94.29	94.54	84.08	84.03	83.92	79.67
93A	17	76	94.94	94.97	95.09	95.65	95.10	95.82	95.18	95.12	95.12	95.88	95.18	95.83	95.84	95.01	95.78	94.87	95.63	95.75	95.71	95.47	95.69	95.68	95.92	95.67	96.41	96.43	95.72	95.58	95.18	95.52	95.20	95.42	95.42	97.13	97.27	97.29	100	99.49	98.21	95.48	95.50	95.09	94.92	94.55	94.86	95.13	95.72	94.73	95.03	94.70	93.56	93.43	93.32	93.27	93.99	94.13	94.14	94.49	83.03	83.32	83.47	79.40
DAT290	4	94 (94)	96.39	96.38	96.52	96.44	96.61	96.66	96.60	96.55	96.59	96.62	96.68	96.64	96.66	96.33	96.46	96.07	96.26	96.26	96.55	97.58	97.76	97.75	97.24	97.45	97.48	97.62	97.46	97.41	96.96	96.75	96.95	97.08	97.08	99.44	99.46	100	97.59	97.59	98.05	96.69	96.74	96.38	96.36	95.79	96.14	96.15	96.41	95.97	96.18	96.04	94.10	94.35	93.37	93.86	94.05	93.92	95.63	95.16	83.10	83.52	83.74	79.53
DAT234	UT	108 (94)	96.51	96.49	96.64	96.55	96.71	96.75	96.70	96.67	96.72	96.71	96.67	96.74	96.73	96.52	96.59	96.21	96.45	96.48	96.65	97.65	97.77	97.81	97.37	97.72	97.67	97.72	97.54	97.51	97.04	96.69	97.01	97.08	97.09	99.39	100	99.64	97.64	97.65	98.06	96.78	96.78	96.39	96.39	95.90	96.23	96.14	96.47	96.15	96.28	96.05	94.19	94.50	93.47	93.89	94.16	94.17	95.70	95.14	83.28	83.71	84.06	79.55
89-2479	23	483 (94)	96.30	96.29	96.43	96.47	96.59	96.63	96.50	96.44	96.50	96.59	96.67	96.60	96.59	96.30	96.54	96.15	96.40	96.49	96.60	97.68	97.79	97.77	97.01	97.36	97.68	97.68	97.47	97.39	97.09	96.57	96.96	96.94	96.94	100	99.29	99.44	97.33	97.22	98.00	96.79	96.77	96.35	96.20	95.70	96.29	96.22	96.40	96.11	96.15	96.08	93.80	94.10	93.32	93.81	94.18	94.24	95.70	95.03	83.22	83.56	83.63	79.33
MNCM50	2	104 (225)	96.65	96.64	96.79	96.88	96.85	96.89	96.89	96.89	96.79	96.86	96.83	96.89	96.89	96.88	96.69	96.66	96.68	96.71	96.72	97.55	97.57	97.58	97.18	97.47	97.73	97.72	97.53	97.70	97.64	97.37	97.74	99.97	100	97.08	97.13	97.19	95.85	96.00	96.40	97.09	97.20	96.64	96.72	96.32	96.46	96.12	96.37	96.27	96.28	96.19	94.03	94.56	93.90	94.21	94.51	94.39	95.77	95.09	83.38	83.75	83.56	79.42
MNCM21	2	101 (225)	96.66	96.64	96.79	96.90	96.86	96.92	96.92	96.93	96.82	96.86	96.84	96.93	96.92	96.90	96.74	96.60	96.64	96.67	96.67	97.52	97.58	97.53	97.17	97.41	97.70	97.69	97.53	97.68	97.61	97.35	97.72	100	99.98	97.16	97.12	97.23	95.86	96.01	96.43	97.07	97.15	96.63	96.72	96.31	96.46	96.16	96.29	96.25	96.29	96.19	94.01	94.61	93.82	94.20	94.55	94.47	95.80	95.17	83.18	83.68	83.61	79.32
NIAH11435	2	28 (28)	96.62	96.59	96.75	96.86	96.80	96.89	96.86	96.91	96.74	96.81	96.77	96.89	96.90	96.76	96.71	96.75	96.71	96.68	96.69	98.91	98.95	98.96	98.78	98.72	99.71	99.67	99.30	99.74	99.75	99.49	100	97.74	97.75	97.05	97.04	97.01	95.54	96.00	96.01	97.01	97.05	96.70	96.69	96.33	96.38	96.16	96.26	96.12	96.32	96.15	93.78	94.49	93.84	94.26	94.42	94.36	95.71	95.15	83.25	83.73	83.54	79.39
05HAS68	2	28 (28)	96.17	96.14	96.30	96.76	96.34	96.80	96.30	96.33	96.30	96.70	96.47	96.82	96.80	96.21	96.64	96.26	96.53	96.47	96.63	98.54	98.56	98.51	98.57	98.22	99.58	99.56	98.95	99.30	99.39	100	99.25	97.09	97.11	96.71	96.58	96.72	95.68	96.21	95.86	96.90	96.91	96.26	96.16	95.83	95.93	96.06	96.30	95.68	95.85	95.70	93.38	93.74	93.25	93.55	93.84	93.88	95.17	95.08	82.80	83.28	84.03	79.54
DAT260	2	28 (28)	96.62	96.56	96.72	96.84	96.77	96.88	96.74	96.78	96.68	96.78	96.82	96.88	96.88	96.73	96.73	96.76	96.67	96.66	96.69	98.97	98.99	98.97	98.81	98.67	99.72	99.68	99.33	99.74	100	99.56	99.75	97.59	97.59	97.07	97.04	97.03	95.53	95.96	95.92	97.03	97.02	96.70	96.57	96.25	96.34	96.21	96.25	96.14	96.26	96.02	93.56	94.23	93.55	94.02	94.34	94.44	95.81	95.19	83.33	83.62	83.58	79.46
DAT292	2	28 (28)	96.63	96.61	96.77	96.84	96.75	96.87	96.87	96.95	96.84	96.82	96.83	96.87	96.88	96.77	96.71	96.68	96.60	96.64	96.67	99.01	99.01	99.04	98.85	99.02	99.71	99.68	99.85	100	99.78	99.50	99.80	97.70	97.70	97.53	97.51	97.47	95.93	95.95	96.37	96.98	97.08	96.71	96.67	96.29	96.36	96.15	96.24	96.06	96.34	96.05	94.09	94.38	93.69	94.20	94.21	94.30	95.59	95.13	83.42	83.72	83.72	79.60
MNCM43	2	28 (28)	96.52	96.48	96.65	96.56	96.70	96.76	96.76	96.74	96.66	96.68	96.69	96.76	96.76	96.51	96.65	96.16	96.58	96.59	96.61	98.94	99.01	99.02	98.72	98.98	99.70	99.67	100	99.80	99.34	99.04	99.34	97.51	97.52	97.51	97.55	97.49	95.99	95.95	96.46	96.77	96.84	96.67	96.65	96.01	96.34	96.08	96.23	96.14	96.30	96.04	94.25	94.47	93.64	94.12	94.14	94.17	95.71	95.17	83.42	83.84	83.41	79.39
ST3	3	35 (28)	96.61	96.58	96.74	96.73	96.75	96.75	96.76	96.75	96.76	96.74	96.77	96.72	96.73	96.69	96.73	96.68	96.59	96.47	96.54	99.08	99.08	98.99	99.16	99.07	99.90	100	99.65	99.67	99.64	99.66	99.58	97.59	97.60	97.75	97.67	97.65	96.44	96.43	96.44	97.06	97.12	96.78	96.76	96.36	96.51	96.39	96.59	96.31	96.40	96.14	94.11	94.37	93.77	94.27	94.46	94.46	95.81	95.25	83.55	83.75	83.56	79.36
4961	3	35 (28)	96.70	96.67	96.83	96.83	96.81	96.83	96.84	96.84	96.84	96.84	96.84	96.80	96.83	96.80	96.62	96.74	96.52	96.54	96.59	99.12	99.12	99.11	99.21	99.17	100	99.93	99.71	99.69	99.70	99.73	99.72	97.66	97.66	97.73	97.69	97.62	96.50	96.51	96.58	97.18	97.20	96.81	96.75	96.40	96.44	96.43	96.50	96.24	96.42	96.15	94.05	94.52	93.83	94.31	94.45	94.38	95.88	95.29	83.23	83.45	83.46	79.50
D9	7	29 (25)	96.24	96.21	96.37	96.66	96.34	96.73	96.41	96.37	96.40	96.74	96.30	96.71	96.74	96.34	96.69	96.32	96.64	96.61	96.65	99.19	99.14	99.12	99.57	100	99.21	99.22	98.78	98.80	98.80	98.72	98.77	97.18	97.19	97.16	97.36	97.32	96.35	96.02	96.38	96.73	96.66	96.29	96.36	95.91	96.03	95.83	96.38	95.69	95.95	95.75	93.67	94.09	93.40	93.87	93.88	94.06	95.41	95.26	82.59	82.83	83.88	79.49
8074	7	29 (25)	96.35	96.32	96.49	96.45	96.55	96.59	96.56	96.51	96.53	96.59	96.53	96.58	96.66	96.56	96.55	96.13	96.38	96.24	96.34	99.44	99.35	99.35	100	99.54	99.13	99.14	98.92	98.95	98.69	98.32	98.64	97.19	97.19	97.46	97.61	97.54	95.96	96.44	96.33	96.76	96.69	96.67	96.63	95.92	96.25	96.05	96.33	96.16	96.13	95.94	93.92	94.50	93.70	94.16	94.35	94.52	95.69	95.17	81.01	81.10	82.21	78.57
MNCM25	2	102 (25)	96.39	96.35	96.52	96.55	96.60	96.66	96.55	96.50	96.47	96.56	96.71	96.66	96.63	96.50	96.50	96.43	96.38	96.39	96.35	99.74	99.97	100	98.76	99.13	98.97	98.93	98.73	98.73	98.85	98.42	98.69	97.30	97.32	97.65	97.54	97.67	95.62	95.73	96.23	96.81	96.76	96.43	96.36	95.97	96.11	96.05	96.02	95.93	96.07	95.99	94.02	94.09	93.42	93.96	94.10	94.49	95.76	95.07	82.85	83.02	83.53	79.67
MNCM04	2	25 (25)	96.33	96.32	96.47	96.55	96.59	96.67	96.56	96.50	96.48	96.59	96.74	96.67	96.63	96.45	96.50	96.42	96.39	96.41	96.39	99.76	100	99.97	98.76	99.07	98.96	98.94	98.74	98.72	98.83	98.41	98.69	97.33	97.34	97.69	97.52	97.69	95.59	95.75	96.15	96.85	96.81	96.41	96.34	95.98	96.15	96.08	96.02	95.91	95.99	95.93	94.03	94.18	93.51	94.04	94.18	94.51	95.74	95.10	83.12	83.51	83.43	79.64
89-1591	2	25 (25)	96.27	96.26	96.40	96.46	96.48	96.54	96.44	96.39	96.33	96.55	96.58	96.57	96.59	96.33	96.42	96.09	96.31	96.15	96.26	100	99.72	99.68	98.70	99.23	98.97	98.99	98.75	98.70	98.83	98.26	98.63	97.16	97.16	97.60	97.48	97.60	95.49	95.64	96.15	96.56	96.54	96.41	96.31	95.98	96.11	95.96	96.04	95.89	95.98	95.86	93.77	94.23	93.33	93.96	94.15	94.43	95.77	94.99	82.88	83.29	83.42	79.23
2651	1/2	56	96.88	96.84	97.00	96.97	96.98	97.00	97.03	97.01	96.97	97.00	97.01	96.99	96.97	96.97	96.74	96.82	96.94	96.94	100	96.77	96.78	96.78	96.77	96.78	96.77	96.74	96.92	96.89	96.91	96.91	96.90	96.77	96.78	96.69	96.74	96.69	95.92	95.91	96.02	96.83	96.86	96.56	96.62	96.34	96.35	96.34	96.33	96.25	96.29	95.99	94.13	94.28	93.69	94.17	94.36	94.22	95.83	95.28	83.24	83.52	83.53	79.45
ST1	1	13 (13)	97.16	97.13	97.28	97.32	97.26	97.28	97.28	97.24	97.40	97.32	97.26	97.30	97.30	97.26	96.57	96.47	99.96	100	97.08	96.73	96.76	96.75	96.77	96.76	96.71	96.75	96.81	96.79	96.80	96.81	96.78	96.80	96.80	96.66	96.61	96.56	95.92	95.97	96.10	96.77	96.81	96.68	96.76	96.32	96.50	96.39	96.49	96.23	96.41	96.29	94.21	94.60	93.89	94.31	94.49	94.44	95.86	95.17	83.38	83.27	83.61	79.53
NCTC 10237	1	13 (13)	97.15	97.12	97.28	97.29	97.24	97.28	97.31	97.28	97.26	97.29	97.27	97.32	97.28	97.30	96.54	96.53	100	99.98	97.11	96.61	96.69	96.68	96.71	96.69	96.67	96.75	96.80	96.80	96.80	96.81	96.78	96.81	96.82	96.57	96.52	96.51	95.81	95.83	96.11	96.56	96.70	96.68	96.66	96.21	96.43	96.17	96.40	96.09	96.30	96.15	94.17	94.36	93.68	94.15	94.25	94.25	95.71	95.11	83.54	83.78	83.61	79.74
14636	8	87	96.89	96.83	97.02	97.08	96.99	97.13	97.01	97.03	97.06	97.10	96.98	97.09	97.09	96.92	96.90	100	96.42	96.38	96.70	96.42	96.61	96.57	96.30	96.35	96.75	96.85	96.22	96.66	96.76	96.47	96.75	96.58	96.58	96.33	96.26	96.27	95.17	95.62	95.62	96.66	96.70	96.58	96.46	96.20	96.30	96.25	96.39	96.05	96.25	95.97	93.83	94.58	93.93	94.32	94.54	94.44	95.77	95.14	83.55	83.72	84.00	79.47
NCTC 10446	15	81	97.60	97.57	97.73	97.65	97.71	97.74	97.74	97.74	97.74	97.70	97.74	97.75	97.81	97.65	100	96.98	96.60	96.60	96.68	96.68	96.74	96.74	96.77	96.76	96.71	96.85	96.75	96.75	96.76	96.77	96.75	96.73	96.74	96.73	96.66	96.71	95.88	95.92	96.12	96.86	96.97	96.75	96.77	96.46	96.67	96.48	96.47	96.22	96.42	96.24	93.93	94.39	93.71	94.06	94.35	94.19	95.87	95.31	83.24	83.90	83.86	79.53
MNCM07	14	1 (1)	99.41	99.38	99.55	99.59	99.50	99.70	99.60	99.59	99.51	99.53	99.61	99.69	99.73	100	97.65	97.02	97.28	97.30	96.94	96.63	96.68	96.70	96.37	96.78	96.88	96.85	96.71	96.85	96.82	96.51	96.84	96.90	96.91	96.58	96.66	96.57	95.52	95.69	96.02	97.11	97.03	96.66	96.75	96.30	96.51	96.19	96.29	96.17	96.27	96.13	93.88	94.52	93.84	94.21	94.43	94.37	95.78	95.24	83.39	83.72	83.31	79.37
13730	14	6 (1)	99.75	99.72	99.89	99.76	99.80	99.89	99.87	99.89	99.81	99.76	99.91	99.89	100	99.73	97.80	97.20	97.25	97.24	96.95	96.73	96.77	96.76	96.76	96.78	96.86	96.85	96.92	96.90	96.89	96.93	96.90	96.79	96.81	96.82	96.78	96.79	95.93	95.94	96.21	97.11	97.04	96.82	96.82	96.36	96.65	96.29	96.25	96.23	96.28	96.10	93.91	94.36	93.67	94.11	94.33	94.18	95.85	95.21	83.95	84.18	83.31	79.57
S735	2	1 (1)	99.76	99.72	99.90	99.78	99.82	99.90	99.87	99.90	99.81	99.79	99.92	100	99.90	99.67	97.74	97.13	97.25	97.24	96.99	96.80	96.85	96.84	96.84	96.81	96.91	96.89	96.98	96.97	96.93	96.98	96.94	96.97	96.97	96.85	96.84	96.86	96.02	96.05	96.19	97.14	97.09	96.82	96.87	96.41	96.63	96.32	96.32	96.21	96.33	96.11	94.15	94.46	93.83	94.23	94.45	94.30	95.87	95.27	83.50	83.66	83.69	79.75
BM407	2	1 (1)	99.52	99.49	99.66	99.70	99.48	99.81	99.52	99.51	99.40	99.59	100	99.80	99.81	99.32	97.58	96.79	97.07	97.05	96.87	96.62	96.72	96.69	95.93	96.50	96.81	96.79	96.64	96.63	96.65	96.17	96.60	96.56	96.57	96.62	96.48	96.56	95.07	95.10	95.87	96.92	96.85	96.52	96.54	95.95	96.31	95.86	96.17	95.89	95.95	95.89	94.10	94.16	93.49	93.99	94.15	94.10	95.64	95.18	82.96	83.44	83.45	79.51
GZ1	2	1 (1)	99.66	99.61	99.78	99.73	99.73	99.84	99.79	99.81	99.66	100	99.72	99.75	99.71	99.47	97.70	97.05	97.24	97.21	96.99	96.72	96.77	96.78	96.75	96.68	96.89	96.87	96.86	96.90	96.80	96.85	96.85	96.88	96.89	96.71	96.67	96.70	95.86	95.92	96.07	97.03	96.95	96.75	96.78	96.27	96.62	96.22	96.34	96.23	96.25	96.09	93.86	94.42	93.65	94.10	94.34	94.19	95.89	95.28	83.43	83.66	83.69	79.77
JS14	14	7 (1)	99.58	99.55	99.72	99.76	99.63	99.86	99.77	99.78	100	99.72	99.69	99.78	99.82	99.49	97.74	97.12	97.20	97.24	96.93	96.60	96.71	96.72	96.39	96.69	96.88	96.87	96.84	96.89	96.75	96.50	96.83	96.79	96.80	96.76	96.74	96.72	95.61	95.60	96.17	96.90	96.89	96.75	96.85	96.33	96.57	96.13	96.31	96.13	96.34	96.05	94.00	94.46	93.85	94.12	94.43	94.40	95.70	95.31	82.95	83.26	83.59	79.57
NIAH11434	2	1 (1)	99.70	99.67	99.85	99.86	99.76	99.99	99.89	100	99.75	99.84	99.81	99.90	99.86	99.54	97.71	97.12	97.24	97.23	96.96	96.69	96.79	96.83	96.42	96.71	96.89	96.87	96.95	97.02	96.85	96.56	96.98	96.91	96.92	96.73	96.72	96.70	95.54	95.57	96.11	96.98	96.92	96.75	96.82	96.31	96.52	96.19	96.25	96.18	96.27	96.16	93.97	94.50	93.68	94.03	94.28	94.25	95.81	95.31	82.98	83.48	83.38	79.52
DAT273	2	1 (1)	99.71	99.68	99.85	99.84	99.77	99.98	100	99.89	99.78	99.84	99.82	99.88	99.87	99.57	97.70	97.11	97.29	97.28	96.95	96.74	96.82	96.80	96.52	96.74	96.91	96.90	96.96	96.93	96.86	96.54	96.94	96.89	96.90	96.77	96.73	96.73	95.59	95.63	96.11	97.05	96.95	96.79	96.90	96.38	96.64	96.21	96.29	96.22	96.23	96.17	93.92	94.51	93.91	94.23	94.38	94.27	95.87	95.30	83.13	83.44	83.40	79.56
P1/7	2	1 (1)	99.85	99.82	99.99	99.86	99.92	100	99.96	99.98	99.85	99.87	99.94	99.90	99.89	99.68	97.74	97.16	97.23	97.23	96.99	96.79	96.88	96.88	96.83	96.82	96.92	96.90	96.98	96.97	96.95	97.00	96.97	96.99	97.00	96.89	96.86	96.86	95.96	95.96	96.25	97.10	97.05	96.80	96.84	96.34	96.66	96.31	96.31	96.22	96.26	96.16	94.17	94.39	93.81	94.14	94.41	94.25	95.82	95.27	83.34	83.58	83.71	79.34
SS12	1/2	1 (1)	99.62	99.59	99.76	99.81	100	99.91	99.75	99.73	99.64	99.75	99.77	99.82	99.82	99.50	97.71	97.05	97.18	97.23	96.95	96.80	96.85	96.84	96.36	96.73	96.93	96.91	96.88	96.83	96.87	96.57	96.85	96.87	96.89	96.87	96.78	96.81	95.61	95.68	96.15	97.05	97.00	96.74	96.77	96.25	96.68	96.31	96.34	96.24	96.23	96.14	94.05	94.47	93.84	94.15	94.50	94.34	95.82	95.34	82.99	83.46	83.62	79.58
A7	2	7 (1)	99.72	99.69	99.86	100	99.82	99.87	99.84	99.86	99.72	99.73	99.80	99.77	99.75	99.57	97.63	97.11	97.24	97.21	96.97	96.73	96.81	96.80	96.73	96.72	96.92	96.87	96.77	96.96	96.92	96.97	96.95	96.94	96.94	96.66	96.62	96.62	95.79	96.00	95.98	97.13	97.05	96.75	96.77	96.15	96.59	96.25	96.30	96.22	96.27	96.16	93.91	94.46	93.80	94.26	94.58	94.45	95.78	95.31	83.00	83.45	83.49	79.50
SC84	2	7 (1)	99.85	99.82	100	99.87	99.78	99.99	99.84	99.84	99.73	99.85	99.88	99.88	99.89	99.57	97.70	97.05	97.22	97.22	96.99	96.71	96.81	96.78	96.43	96.71	96.88	96.88	96.86	96.86	96.84	96.54	96.82	96.78	96.80	96.81	96.76	96.78	95.58	95.60	96.12	97.02	96.94	96.79	96.78	96.28	96.56	96.08	96.31	96.21	96.27	96.08	94.00	94.40	93.71	94.14	94.39	94.23	95.83	95.29	83.75	83.73	83.49	79.69
05ZYH33	2	7 (1)	99.69	100	99.83	99.70	99.59	99.82	99.66	99.66	99.53	99.66	99.71	99.72	99.72	99.36	97.52	96.90	97.07	97.08	96.83	96.58	96.65	96.62	96.30	96.53	96.76	96.74	96.71	96.71	96.68	96.34	96.67	96.66	96.67	96.56	96.53	96.56	95.39	95.42	95.94	96.85	96.78	96.63	96.67	96.12	96.39	95.94	96.20	96.04	96.05	95.94	94.03	94.30	93.55	93.99	94.25	94.12	95.63	95.14	83.43	83.75	83.78	79.76
98HAH33	2	7 (1)	100	99.69	99.85	99.72	99.65	99.85	99.71	99.71	99.58	99.70	99.73	99.73	99.75	99.42	97.52	96.91	97.15	97.12	96.87	96.58	96.64	96.64	96.32	96.60	96.81	96.79	96.76	96.75	96.75	96.47	96.73	96.67	96.68	96.63	96.60	96.61	95.41	95.42	95.92	96.84	96.76	96.63	96.58	96.11	96.40	95.90	96.22	96.05	96.11	95.93	93.88	94.33	93.47	93.99	94.21	94.11	95.65	95.16	83.46	83.77	83.20	79.81

ANIm values;

- 100%
- 98.0-99.99%
- 96.0-97.99%
- 95.0-95.99%
- 93.0-94.99%
- 90.0-92.99%
- 90% <

Table S4. ANIm values between two genomes among 61 *S. suis* strains including serotype 20, 22, 26, and 33 reference strains

Query			ANIm values between query and subject genomes																																																											
			Subject																																																										
Strain	Serotype	ST (CC)	98HAH33	05ZYH33	SC84	A7	SS12	P1/7	DAT273	NIAH11434	JS14	GZ1	BM407	S735	13730	MNCM07	NCTC10446	14636	NCTC10237	ST1	2651	89-1591	MNCM04	MNCM25	8074	D9	4961	ST3	MNCM43	DAT292	DAT260	05HAS68	NIAH11435	MNCM21	MNCM50	89-2479	DAT234	DAT290	93A	42A	NT77	6407	11538	8830	12814	92-1400	89-590	2726	2524	89-3576-3	4417	D12	10581	89-5259	88-5299A	R61	92-1191	14A	92-4172	22083	86-5192	89-4109-1	88-1861	EA1832.92
EA1832.92	33		81.58	81.51	81.57	81.48	81.97	81.59	81.61	81.62	81.48	81.51	81.48	81.62	81.58	81.53	81.61	81.51	81.67	81.63	81.59	81.50	81.52	81.41	81.52	81.47	82.05	81.64	81.39	81.60	81.48	81.47	81.53	81.45	81.58	81.51	81.43	81.54	81.50	81.65	81.46	81.57	81.56	81.58	81.61	81.50	81.52	81.64	81.61	81.55	81.58	81.45	81.52	81.51	81.50	81.53	81.53	81.47	81.49	81.55	80.92	80.94	80.90	100
88-1861	22		86.47	86.09	86.39	86.46	86.58	86.31	85.98	86.29	86.38	86.38	86.46	86.56	86.35	86.39	86.37	86.46	86.33	86.43	86.29	86.59	86.35	86.36	86.59	86.49	86.42	86.26	86.25	86.34	86.54	86.14	86.02	86.60	86.33	86.34	86.35	86.41	86.48	87.02	86.12	86.55	86.39	86.44	86.32	86.32	86.36	86.34	86.34	86.33	86.41	86.39	86.55	86.47	86.57	86.46	86.39	86.09	86.40	86.47	95.14	95.22	100	80.93
89-4109-1	26		85.97	85.86	86.11	86.27	86.30	86.34	85.27	86.19	86.30	86.22	86.26	86.18	85.95	86.43	86.09	85.94	86.35	86.23	86.10	86.67	85.90	86.30	86.30	86.37	85.94	86.38	86.29	86.19	85.89	85.88	85.69	86.43	86.41	86.02	86.28	86.02	86.30	87.00	86.05	86.38	85.93	86.45	86.32	86.22	85.97	86.15	86.16	86.44	85.95	86.48	86.32	85.98	86.66	86.18	86.24	85.83	86.29	86.30	97.28	100	95.20	80.94
86-5192	20		85.74	85.80	85.85	86.16	86.54	86.01	85.23	86.01	86.23	86.11	86.16	85.94	86.00	86.17	85.92	86.01	86.08	86.13	86.16	86.39	86.02	86.03	86.24	86.24	85.96	86.30	86.25	86.11	85.67	85.90	85.57	86.34	86.21	85.95	86.11	85.91	86.12	86.61	85.84	86.26	85.96	86.29	86.35	86.11	85.81	86.20	86.22	86.34	85.98	86.24	86.26	85.84	86.39	86.04	86.11	85.75	86.10	86.37	100	97.24	95.08	81.00
22083	9	82	95.15	95.11	95.26	95.25	95.29	95.26	95.20	95.20	95.27	95.27	95.26	95.28	95.19	95.20	95.18	95.13	95.16	95.27	95.20	94.91	95.07	95.06	95.18	95.28	95.22	95.30	95.16	95.16	95.16	95.27	95.15	95.20	95.19	95.11	95.13	95.15	94.73	95.01	94.74	95.08	95.04	95.22	95.22	95.18	95.43	95.32	95.51	95.48	95.56	95.68	93.95	93.88	93.53	93.99	93.92	93.77	95.19	100	86.05	86.03	86.22	81.52
92-4172	31	70	95.78	95.76	95.89	95.94	95.90	95.97	95.86	95.81	95.92	95.98	95.88	95.96	95.85	95.78	95.81	95.80	95.80	95.95	95.83	95.51	95.69	95.69	95.71	95.96	95.86	95.98	95.81	95.76	95.88	95.71	95.77	95.77	95.77	95.82	95.80	95.78	94.93	95.17	94.98	95.61	95.56	95.84	95.79	95.69	96.12	95.79	96.27	96.14	95.95	96.18	94.52	94.29	93.97	94.23	94.48	94.37	100	94.98	86.01	85.98	86.19	81.49
14A	21	481	94.42	94.41	94.52	94.61	94.62	94.55	94.54	94.49	94.55	94.55	94.55	94.57	94.51	94.59	94.43	94.61	94.49	94.57	94.44	94.54	94.60	94.60	94.45	94.68	94.51	94.57	94.51	94.52	94.63	94.43	94.57	94.61	94.60	94.54	94.46	94.38	94.70	95.03	94.61	94.36	94.35	94.53	94.57	94.36	94.38	94.49	94.79	94.68	94.68	94.61	94.98	94.91	94.73	95.08	95.37	100	94.51	93.87	86.04	86.02	86.24	81.54
92-1191	29	92	94.52	94.50	94.62	94.76	94.69	94.67	94.61	94.58	94.66	94.69	94.65	94.68	94.60	94.66	94.59	94.64	94.54	94.66	94.48	94.51	94.54	94.55	94.47	94.69	94.54	94.62	94.50	94.51	94.57	94.43	94.57	94.72	94.71	94.55	94.59	94.52	94.74	94.96	94.61	94.36	94.35	94.56	94.68	94.33	94.50	94.50	94.90	94.77	94.70	94.67	95.00	94.85	94.60	94.83	100	95.26	94.56	94.03	85.82	85.82	86.30	81.48
R61	UT	776	94.38	94.39	94.50	94.58	94.51	94.49	94.45	94.42	94.54	94.52	94.46	94.50	94.42	94.48	94.29	94.48	94.49	94.57	94.38	94.27	94.42	94.41	94.37	94.58	94.46	94.52	94.46	94.46	94.49	94.35	94.49	94.51	94.50	94.31	94.37	94.35	94.14	94.51	94.16	94.11	94.13	94.47	94.54	94.26	94.30	94.35	94.76	94.64	94.63	94.58	95.21	95.18	94.93	100	94.80	94.98	94.40	94.05	85.80	85.82	86.30	81.96
88-5299A	24	68	94.09	94.09	94.19	94.24	94.27	94.21	94.21	94.19	94.28	94.23	94.19	94.22	94.17	94.20	94.12	94.23	94.19	94.25	94.16	94.09	94.09	94.09	94.03	94.29	94.17	94.24	94.17	94.24	94.20	94.14	94.26	94.23	94.23	94.05	94.07	94.00	93.97	94.16	94.02	93.92	93.87	94.19	94.32	94.00	94.04	94.08	94.38	94.28	94.35	94.32	94.83	94.93	100	94.95	94.50	94.68	94.12	93.63	86.10	86.27	86.45	81.48
89-5259	27	72	94.52	94.51	94.63	94.68	94.69	94.67	94.68	94.69	94.70	94.69	94.63	94.69	94.65	94.68	94.53	94.65	94.58	94.66	94.50	94.56	94.53	94.53	94.47	94.75	94.62	94.67	94.69	94.61	94.61	94.46	94.69	94.82	94.83	94.56	94.69	94.60	94.37	94.60	94.36	94.26	94.31	94.68	94.68	94.63	94.53	94.44	94.81	94.85	94.81	94.73	94.99	100	94.95	95.22	94.82	94.84	94.42	94.02	86.18	86.26	86.49	81.54
10581	13	71	94.59	94.57	94.68	94.65	94.62	94.66	94.55	94.57	94.61	94.66	94.73	94.66	94.60	94.55	94.51	94.43	94.58	94.65	94.61	94.42	94.49	94.49	94.54	94.73	94.59	94.64	94.62	94.55	94.34	94.22	94.38	94.56	94.57	94.53	94.57	94.58	94.38	94.47	94.39	94.34	94.29	94.57	94.70	94.27	94.41	94.34	94.84	94.62	94.70	94.57	100	94.93	94.81	95.18	94.90	94.91	94.57	93.96	86.15	86.33	86.34	81.48
D12	9	619	95.98	95.97	96.10	96.10	96.16	96.13	96.00	96.00	96.06	96.15	96.02	96.14	96.00	95.96	95.96	95.92	95.97	96.09	95.88	95.49	95.89	95.88	95.87	96.03	96.02	96.08	95.97	96.01	95.94	95.86	96.03	95.93	95.93	95.99	95.99	95.95	95.22	95.57	95.25	95.75	95.74	95.98	96.00	95.83	96.19	96.12	96.41	96.71	96.91	100	94.42	94.50	94.14	94.39	94.47	94.39	96.06	95.53	86.22	86.30	86.46	81.51
4417	10	78	96.10	96.07	96.21	96.29	96.24	96.30	96.15	96.22	96.26	96.31	96.14	96.29	96.21	96.11	96.18	96.12	96.14	96.25	96.10	95.74	96.02	96.03	96.07	96.27	96.29	96.38	96.18	96.26	96.20	96.07	96.21	96.13	96.13	96.14	96.14	96.09	95.21	95.50	95.28	95.88	95.79	96.20	96.12	95.95	96.28	96.26	96.73	97.05	100	97.04	94.62	94.67	94.31	94.54	94.59	94.57	95.98	95.57	85.79	85.81	86.26	81.46
89-3576-3	25	69	96.11	96.08	96.22	96.24	96.25	96.27	96.19	96.15	96.22	96.31	96.16	96.28	96.20	96.15	96.13	96.12	96.16	96.26	96.14	95.74	95.98	95.98	96.02	96.22	96.19	96.29	96.11	96.10	96.10	96.02	96.10	96.15	96.16	96.14	96.16	96.10	95.35	95.65	95.47	95.88	95.84	96.27	96.16	95.97	96.25	96.42	96.72	100	97.04	96.84	94.58	94.72	94.27	94.56	94.70	94.56	96.19	95.48	86.22	86.20	86.25	81.51
2524	6	55	96.22	96.19	96.34	96.33	96.36	96.33	96.22	96.20	96.35	96.36	96.33	96.32	96.21	96.17	96.22	96.25	96.28	96.40	96.18	95.77	96.07	96.07	96.29	96.40	96.36	96.46	96.23	96.23	96.22	96.38	96.21	96.23	96.24	96.29	96.32	96.25	95.75	96.03	95.83	96.30	96.24	96.31	96.35	96.00	96.32	96.86	100	96.69	96.73	96.50	94.80	94.67	94.32	94.68	94.78	94.68	96.27	95.48	85.84	85.84	86.25	81.47
2726	16	73	95.87	95.84	95.97	96.23	96.04	96.25	95.95	95.91	95.97	96.26	95.97	96.23	96.13	95.91	96.16	95.98	96.14	96.27	96.11	95.26	95.62	95.62	95.93	96.05	96.19	96.27	95.88	95.87	95.86	96.00	95.84	95.82	95.82	95.90	96.00	95.90	95.28	95.54	95.19	95.71	95.70	95.96	96.00	95.69	95.87	100	96.90	96.43	96.23	96.23	94.33	94.31	93.98	94.27	94.40	94.38	95.86	95.31	85.95	85.88	86.36	81.53
89-590	28	75	96.39	96.38	96.50	96.58	96.60	96.59	96.48	96.43	96.52	96.56	96.48	96.58	96.52	96.40	96.52	96.16	96.19	96.29	96.21	95.71	96.04	96.04	96.14	96.31	96.30	96.38	96.28	96.25	96.24	96.12	96.25	96.26	96.25	96.22	96.22	96.16	95.27	95.54	95.35	96.04	96.02	96.26	96.35	96.06	100	95.85	96.34	96.23	96.26	96.31	94.36	94.38	93.99	94.16	94.36	94.24	96.11	95.43	86.27	86.24	86.33	81.54
92-1400	30	77	96.02	96.00	96.13	96.12	96.20	96.22	96.17	96.13	96.17	96.20	96.10	96.22	96.18	96.08	96.23	96.10	96.09	96.15	96.17	95.77	95.97	95.97	96.05	96.01	96.28	96.30	96.02	96.22	96.20	96.09	96.23	96.11	96.11	95.74	95.89	95.74	94.96	95.43	95.02	95.83	95.77	96.13	96.22	100	96.15	95.74	96.09	96.04	95.99	96.02	94.25	94.58	93.98	94.23	94.24	94.33	95.73	95.18	85.68	85.78	86.01	81.48
12814	11	91	96.61	96.60	96.72	96.75	96.73	96.77	96.65	96.64	96.74	96.74	96.68	96.77	96.67	96.54	96.68	96.48	96.45	96.55	96.46	96.12	96.31	96.30	96.36	96.57	96.61	96.68	96.55	96.56	96.53	96.49	96.51	96.49	96.48	96.28	96.36	96.24	95.35	95.61	95.45	96.24	96.14	96.74	100	96.15	96.35	96.02	96.39	96.17	96.14	96.15	94.60	94.54	94.20	94.45	94.55	94.48	95.84	95.21	85.65	85.75	85.96	81.38
8830	12	775	96.62	96.60	96.73	96.71	96.70	96.78	96.64	96.64	96.70	96.73	96.66	96.78	96.71	96.60	96.65	96.65	96.42	96.49	96.42	96.17	96.37	96.37	96.49	96.67	96.66	96.70	96.64	96.63	96.59	96.51	96.60	96.45	96.46	96.38	96.44	96.35	95.50	95.73	95.58	96.23	96.21	100	96.77	96.12	96.30	95.98	96.40	96.34	96.25	96.14	94.54	94.60	94.18	94.44	94.49	94.45	95.90	95.26	85.65	85.78	86.02	81.50
11538	5	53	96.66	96.65	96.79	96.96	96.77	96.96	96.69	96.68	96.76	96.89	96.79	96.96	96.91	96.73	96.77	96.53	96.55	96.64	96.73	96.19	96.54	96.54	96.83	96.85	97.06	97.13	96.75	96.85	96.86	96.95	96.81	96.81	96.81	96.53	96.62	96.53	95.68	96.07	95.60	99.32	100	96.30	96.34	95.88	96.17	95.91	96.36	96.01	95.96	95.96	94.39	94.41	94.06	94.26	94.47	94.42	95.80	95.15	86.28	86.62	86.21	81.42
6407	4	54	96.64	96.63	96.76	96.98	96.74	96.99	96.67	96.67	96.76	96.94	96.76	96.99	96.90	96.71	96.69	96.49	96.44	96.57	96.64	96.05	96.47	96.48	96.78	96.86	97.01	97.07	96.66	96.75	96.75	96.88	96.70	96.61	96.61	96.44	96.51	96.43	95.48	95.81	95.39	100	99.09	96.21	96.29	95.79	96.10	95.82	96.33	95.94	95.89	95.92	94.29	94.17	93.86	94.09	94.32	94.25	95.68	95.12	86.34	86.59	86.43	81.53
NT77	18	79	95.81	95.79	95.91	96.04	95.97	96.17	95.85	95.84	95.93	96.13	95.90	96.17	96.10	95.83	95.96	95.59	95.85	95.95	95.96	95.67	96.05	96.06	96.11	96.31	96.39	96.49	96.19	96.10	95.88	95.92	95.85	96.02	96.03	97.60	97.74	97.69	97.68	97.71	100	95.52	95.50	95.69	95.69	95.11	95.52	95.40	95.90	95.66	95.46	95.53	94.54	94.42	94.13	94.26	94.72	94.54	95.23	94.79	86.29	86.57	86.38	81.47
42A	19	76	95.94	95.93	96.07	96.36	96.10	96.39	95.95	95.98	96.16	96.40	96.08	96.41	96.27	95.99	96.21	95.94	96.05	96.27	96.16	95.74	96.21	96.21	96.46	96.55	96.57	96.77	96.25	96.24	96.24	96.60	96.23	96.23	96.25	97.44	97.58	97.54	98.94	100	97.59	95.91	95.88	95.84	95.81	95.54	95.68	95.67	96.20	95.80	95.63	95.88	94.58	94.63	94.30	94.56	95.10	94.95	95.40	95.18	85.89	86.00	86.17	81.54
93A	17	76	95.56	95.54	95.67	95.88	95.71	96.00	95.63	95.62	95.74	96.00	95.62	96.01	95.95	95.59	95.82	95.40	95.67	95.80	95.85	95.50	95.86	95.85	96.21	96.18	96.31	96.40	96.03	95.93	95.70	95.97	95.72	95.82	95.83	97.36	97.50	97.45	100	98.98	97.65	95.52	95.54	95.55	95.45	94.94	95.30	95.32	95.82	95.40	95.22	95.38	94.36	94.29	93.98	94.16	94.71	94.55	94.99	94.72	86.19	86.32	86.19	81.45
DAT290	4	94 (94)	96.57	96.56	96.69	96.62	96.73	96.76	96.66	96.64	96.68	96.72	96.66	96.78	96.72	96.58	96.60	96.39	96.40	96.51	96.62	97.13	97.45	97.44	97.42	97.53	97.38	97.49	97.38	97.32	97.02	96.92	97.00	97.09	97.09	99.25	99.39	100	97.49	97.55	97.61	96.54	96.49	96.41	96.37	95.76	96.25	96.04	96.38	96.20	96.19	96.13	94.59	94.57	94.01	94.33	94.51	94.34	95.87	95.21	85.82	85.97	86.36	81.48
DAT234	UT	108 (94)	96.58	96.57	96.70	96.63	96.78	96.77	96.67	96.65	96.70	96.73	96.68	96.78	96.70	96.63	96.63	96.37	96.41	96.53	96.62	97.10	97.38	97.37	97.40	97.67	97.43	97.56	97.38	97.31	97.03	96.90	97.01	97.00	97.01	99.08	100	99.24	97.43	97.47	97.54	96.49	96.43	96.46	96.39	95.85	96.21	96.00	96.40	96.21	96.17	96.13	94.55	94.61	94.08	94.34	94.53	94.40	95.84	95.13	85.66	85.85	86.21	81.41
89-2479	23	483 (94)	96.57	96.55	96.69	96.63	96.76	96.77	96.65	96.62	96.67	96.72	96.66	96.76	96.68	96.55	96.59	96.36	96.37	96.48	96.54	97.07	97.44	97.43	97.29	97.49	97.49	97.58	97.42	97.38	97.12	96.97	97.08	96.94	96.95	100	99.12	99.09	97.33	97.36	97.44	96.47	96.44	96.40	96.35	95.76	96.24	95.98	96.37	96.18	96.16	96.16	94.51	94.48	94.00	94.27	94.50	94.48	95.89	95.16	86.05	86.22	86.27	81.55
MNCM50	2	104 (104)	96.65	96.64	96.77	96.79	96.86	96.85	96.77	96.77	96.76	96.81	96.75	96.88	96.79	96.81	96.57	96.47	96.56	96.66	96.54	96.90	97.21	97.21	97.16	97.39	97.43	97.51	97.40	97.49	97.43	97.34	97.48	99.70	100	96.91	96.99	96.97	95.79	96.13	95.89	96.57	96.62	96.45	96.51	96.08	96.26	95.84	96.28	96.16	96.14	96.08	94.48	94.72	94.17	94.37	94.67	94.50	95.80	95.22	86.06	86.20	86.19	81.50
MNCM21	2	101 (104)	96.66	96.64	96.77	96.82	96.87	96.86	96.78	96.79	96.77	96.81	96.76	96.89	96.79	96.81	96.58	96.50	96.57	96.67	96.56	96.97	97.23	97.24	97.16	97.37	97.43	97.50	97.39	97.51	97.44	97.36	97.49	100	99.74	96.95	97.00	97.02	95.76	96.11	95.90	96.57	96.62	96.45	96.51	96.10	96.29	95.89	96.28	96.17	96.18	96.08	94.49	94.73	94.18	94.39	94.65	94.53	95.82	95.21	86.07	86.28	86.30	81.50
NIAH11435	2	28 (28)	96.67	96.64	96.79	96.80	96.84	96.87	96.77	96.79	96.77	96.82	96.74	96.88	96.78	96.70	96.65	96.62	96.53	96.65	96.59	98.37	98.62	98.62	98.77	98.74	99.46	99.55	99.28	99.59	99.60	99.52	100	97.48	97.48	97.03	97.00	96.89	95.63	96.09	95.68	96.64	96.61	96.56	96.55	96.18	96.27	95.82	96.27	96.14	96.20	96.14	94.33	94.55	94.23	94.40	94.48	94.47	95.77	95.16	86.04	86.26	86.35	81.56
05HAS68	2	28 (28)	96.47	96.41	96.56	96.75	96.56	96.81	96.50	96.55	96.57	96.77	96.54	96.86	96.79	96.47	96.64	96.46	96.50	96.55	96.59	98.14	98.35	98.34	98.74	98.45	99.47	99.34	99.09	99.43	99.50	100	99.40	97.24	97.22	96.80	96.77	96.74	95.79	96.34	95.67	96.68	96.68	96.35	96.37	95.96	96.01	95.91	96.29	95.94	95.92	95.85	94.04	94.28	93.99	94.16	94.27	94.24	95.61	95.19	85.61	85.90	86.33	81.46
DAT260	2	28 (28)	96.68	96.66	96.80	96.82	96.83	96.88	96.74	96.74	96.78	96.83	96.76	96.89	96.79	96.73	96.66	96.67	96.56	96.68	96.63	98.52	98.67	98.67	98.83	98.76	99.50	99.59	99.30	99.60	100	99.60	99.59	97.44	97.44	97.09	97.04	96.92	95.63	96.12	95.69	96.70	96.66	96.57	96.57	96.17	96.29	95.87	96.28	96.12	96.22	96.08	94.27	94.52	94.14	94.38	94.51	94.53	95.91	95.19	86.10	86.22	86.36	81.48
DAT292	2	28 (28)	96.70	96.67	96.81	96.85	96.84	96.89	96.77	96.86	96.86	96.85	96.78	96.92	96.81	96.73	96.65	96.56	96.55	96.67	96.61	98.51	98.62	98.62	98.78	98.96	99.47	99.59	99.63	100	99.60	99.56	99.59	97.50	97.49	97.35	97.31	97.20	95.86	96.14	95.92	96.69	96.67	96.57	96.60	96.19	96.26	95.88	96.29	96.13	96.30	96.13	94.49	94.53	94.20	94.37	94.48	94.42	95.77	95.18	86.07	86.23	86.32	81.51
MNCM43	2	28 (28)	96.70	96.67	96.82	96.75	96.86	96.90	96.79	96.78	96.81	96.86	96.78	96.92	96.81	96.68	96.69	96.27	96.58	96.67	96.63	98.52	98.64	98.63	98.77	98.98	99.46	99.60	100	99.60	99.29	99.21	99.25	97.40	97.39	97.39	97.36	97.26	95.95	96.15	96.02	96.60	96.56	96.61	96.58	95.99	96.30	95.89	96.28	96.15	96.20	96.09	94.53	94.61	94.16	94.37	94.43	94.44	95.83	95.18	86.22	86.47	86.32	81.50
ST3	3	35 (28)	96.61	96.58	96.73	96.69	96.76	96.73	96.63	96.64	96.70	96.70	96.74	96.74	96.68	96.62	96.62	96.60	96.45	96.52	96.44	98.33	98.65	98.62	98.89	98.93	99.65	100	99.41	99.42	99.41	99.32	99.38	97.33	97.33	97.42	97.38	97.25	96.19	96.46	96.24	96.91	96.93	96.54	96.56	96.15	96.25	96.16	96.34	96.16	96.26	96.08	94.47	94.43	94.05	94.23	94.41	94.33	95.86	95.18	86.23	86.38	86.43	81.49
4961	3	35 (28)	96.72	96.68	96.83	96.78	96.83	96.83	96.72	96.72	96.82	96.79	96.83	96.83	96.74	96.71	96.65	96.64	96.50	96.59	96.52	98.58	98.73	98.73	98.94	99.07	100	99.81	99.50	99.50	99.53	99.61	99.49	97.43	97.44	97.49	97.45	97.30	96.23	96.49	96.30	97.01	96.96	96.64	96.63	96.25	96.33	96.25	96.42	96.22	96.32	96.15	94.57	94.51	94.14	94.37	94.46	94.41	95.89	95.25	86.10	86.28	86.30	81.48
D9	7	29 (25)	96.48	96.44	96.60	96.62	96.68	96.67	96.58	96.54	96.60	96.63	96.58	96.66	96.60	96.64	96.61	96.31	96.46	96.53	96.55	98.88	99.02	99.00	99.55	100	98.94	98.94	98.85	98.86	98.65	98.44	98.63	97.22	97.23	97.36	97.52	97.31	96.02	96.30	96.05	96.71	96.63	96.52	96.46	95.88	96.21	95.93	96.30	96.13	96.18	96.03	94.60	94.58	94.11	94.35	94.53	94.52	95.87	95.17	85.64	85.86	86.45	81.52
8074	7	29 (25)	96.57	96.53	96.68	96.72	96.66	96.76	96.54	96.54	96.64	96.73	96.70	96.77	96.68	96.59	96.65	96.47	96.50	96.62	96.55	98.96	98.96	98.95	100	99.68	98.98	99.08	98.83	98.81	98.87	98.91	98.82	97.18	97.19	97.32	97.41	97.30	96.13	96.39	96.04	96.77	96.71	96.48	96.41	96.03	96.17	95.96	96.34	96.08	96.10	96.01	94.50	94.37	94.02	94.27	94.39	94.35	95.75	95.21	85.42	85.50	86.25	81.78
MNCM25	2	102 (25)	96.54	96.53	96.67	96.72	96.73	96.75	96.63	96.57	96.63	96.70	96.66	96.76	96.64	96.60	96.56	96.49	96.45	96.56	96.46	99.08	99.55	100	98.98	99.15	98.78	98.85	98.71	98.69	98.73	98.55	98.67	97.26	97.26	97.49	97.43	97.36	95.84	96.21	95.92	96.49	96.43	96.38	96.41	95.98	96.11	95.69	96.16	96.05	96.07	96.06	94.47	94.44	94.07	94.25	94.45	94.54	95.79	95.10	85.94	86.08	86.36	81.58
MNCM04	2	25 (25)	96.53	96.50	96.64	96.70	96.70	96.73	96.61	96.56	96.60	96.68	96.65	96.74	96.63	96.59	96.56	96.47	96.45	96.54	96.43	99.04	100	99.51	99.01	99.13	98.77	98.83	98.71	98.68	98.70	98.59	98.66	97.27	97.28	97.46	97.41	97.33	95.90	96.19	95.93	96.47	96.41	96.34	96.36	95.95	96.09	95.68	96.16	96.02	96.06	96.05	94.45	94.45	94.04	94.26	94.46	94.51	95.78	95.10	86.04	86.20	86.31	81.66
89-1591	2	25 (25)	96.45	96.44	96.56	96.61	96.65	96.66	96.58	96.53	96.51	96.62	96.53	96.67	96.62	96.53	96.54	96.36	96.43	96.48	96.48	100	99.35	99.34	99.20	99.22	98.82	98.86	98.73	98.69	98.75	98.53	98.67	97.28	97.28	97.42	97.35	97.26	95.83	96.24	95.86	96.56	96.54	96.34	96.38	95.96	96.07	95.78	96.13	96.00	96.06	95.86	94.41	94.43	94.05	94.15	94.45	94.47	95.84	95.02	86.19	86.31	86.47	81.71
2651	1/2	56	96.83	96.80	96.94	96.89	96.91	96.94	96.81	96.82	96.92	96.93	96.93	96.95	96.83	96.82	96.58	96.65	96.83	96.95	100	96.25	96.44	96.43	96.54	96.68	96.50	96.58	96.62	96.60	96.64	96.70	96.61	96.58	96.58	96.53	96.62	96.55	95.81	96.04	95.92	96.66	96.66	96.42	96.52	96.16	96.20	96.14	96.26	96.16	96.18	96.02	94.60	94.39	94.12	94.21	94.40	94.41	95.90	95.25	86.19	86.26	86.27	81.49
ST1	1	13 (13)	97.04	97.03	97.17	97.12	97.15	97.16	97.05	97.02	97.15	97.08	97.18	97.19	97.04	97.06	96.42	96.35	99.71	100	96.80	96.02	96.40	96.40	96.47	96.54	96.46	96.53	96.53	96.54	96.55	96.55	96.52	96.50	96.52	96.37	96.39	96.31	95.66	96.02	95.81	96.43	96.45	96.35	96.43	96.01	96.17	96.15	96.30	96.16	96.15	96.09	94.55	94.49	94.10	94.41	94.47	94.37	95.87	95.14	86.00	85.87	86.28	81.53
NCTC 10237	1	13 (13)	97.08	97.05	97.18	97.13	97.15	97.18	97.11	97.08	97.18	97.11	97.19	97.21	97.09	97.12	96.46	96.38	100	99.86	96.83	96.17	96.45	96.44	96.50	96.58	96.50	96.60	96.57	96.56	96.57	96.64	96.55	96.58	96.58	96.40	96.43	96.35	95.66	96.00	95.86	96.44	96.45	96.40	96.47	96.06	96.23	96.16	96.32	96.15	96.20	96.11	94.54	94.44	94.13	94.40	94.46	94.41	95.90	95.17	86.15	86.46	86.33	81.62
14636	8	87	96.90	96.88	97.04	97.03	97.04	97.09	96.93	96.95	97.03	97.04	96.98	97.07	97.01	96.88	96.84	100	96.39	96.47	96.63	95.90	96.43	96.42	96.45	96.40	96.62	96.71	96.26	96.56	96.67	96.55	96.61	96.46	96.47	96.34	96.37	96.30	95.37	95.82	95.46	96.44	96.35	96.61	96.48	96.07	96.17	95.97	96.29	96.08	96.13	96.04	94.42	94.58	94.17	94.40	94.56	94.49	95.83	95.14	86.25	86.39	86.55	81.49
NCTC 10446	15	81	97.51	97.47	97.63	97.56	97.62	97.63	97.49	97.50	97.60	97.60	97.61	97.62	97.56	97.40	100	96.82	96.40	96.52	96.55	96.23	96.52	96.51	96.61	96.74	96.64	96.77	96.65	96.66	96.65	96.76	96.64	96.55	96.54	96.55	96.56	96.48	95.78	96.08	95.88	96.68	96.64	96.62	96.69	96.18	96.54	96.16	96.26	96.11	96.16	96.12	94.51	94.42	94.07	94.20	94.50	94.35	95.86	95.17	85.96	86.16	86.24	81.45
MNCM07	14	1 (1)	99.33	99.30	99.47	99.46	99.42	99.58	99.36	99.35	99.40	99.45	99.46	99.57	99.48	100	97.39	96.87	97.09	97.19	96.79	96.22	96.54	96.53	96.55	96.77	96.70	96.76	96.66	96.73	96.71	96.62	96.71	96.81	96.82	96.52	96.60	96.49	95.55	95.91	95.67	96.71	96.57	96.59	96.61	96.05	96.43	95.90	96.23	96.14	96.10	96.11	94.51	94.51	94.11	94.42	94.55	94.46	95.84	95.19	86.21	86.24	86.19	81.42
13730	14	6 (1)	99.65	99.62	99.79	99.65	99.71	99.79	99.65	99.66	99.70	99.66	99.81	99.80	100	99.49	97.55	96.99	97.11	97.18	96.81	96.29	96.64	96.64	96.66	96.78	96.74	96.84	96.81	96.81	96.78	96.93	96.79	96.77	96.78	96.68	96.70	96.65	95.93	96.20	96.06	96.91	96.82	96.70	96.68	96.15	96.56	96.12	96.25	96.16	96.22	96.15	94.66	94.56	94.13	94.40	94.53	94.40	95.89	95.21	86.17	86.40	86.31	81.56
S735	2	1 (1)	99.57	99.52	99.71	99.59	99.61	99.72	99.62	99.64	99.64	99.59	99.76	100	99.67	99.45	97.51	96.94	97.08	97.19	96.82	96.17	96.61	96.60	96.64	96.68	96.71	96.74	96.81	96.81	96.79	96.87	96.77	96.75	96.76	96.63	96.65	96.62	95.90	96.20	96.02	96.88	96.80	96.66	96.68	96.10	96.49	96.13	96.23	96.17	96.19	96.15	94.53	94.47	94.06	94.35	94.50	94.41	95.88	95.18	85.97	86.25	86.27	81.54
BM407	2	1 (1)	99.53	99.50	99.66	99.65	99.49	99.77	99.49	99.48	99.47	99.64	100	99.76	99.66	99.32	97.48	96.83	97.05	97.18	96.77	96.12	96.50	96.50	96.53	96.60	96.69	96.74	96.66	96.66	96.64	96.54	96.62	96.61	96.63	96.55	96.56	96.51	95.48	95.82	95.64	96.62	96.54	96.52	96.57	95.96	96.37	95.81	96.23	96.04	96.02	96.02	94.59	94.37	94.01	94.33	94.43	94.36	95.78	95.14	85.93	86.07	86.36	81.62
GZ1	2	1 (1)	99.53	99.51	99.67	99.55	99.58	99.68	99.58	99.59	99.56	100	99.64	99.59	99.53	99.33	97.47	96.92	96.98	97.08	96.79	96.08	96.56	96.55	96.60	96.63	96.67	96.69	96.74	96.74	96.73	96.77	96.70	96.66	96.67	96.59	96.60	96.57	95.87	96.16	95.99	96.82	96.74	96.61	96.65	96.08	96.47	96.15	96.26	96.21	96.21	96.16	94.53	94.50	94.07	94.36	94.50	94.37	95.90	95.17	85.93	86.15	86.17	81.60
JS14	14	7 (1)	99.44	99.41	99.59	99.60	99.49	99.71	99.53	99.56	100	99.57	99.47	99.64	99.57	99.27	97.48	96.90	97.05	97.15	96.79	96.03	96.47	96.47	96.50	96.61	96.67	96.70	96.69	96.74	96.67	96.58	96.65	96.63	96.65	96.53	96.58	96.53	95.59	95.93	95.68	96.62	96.53	96.58	96.65	96.05	96.43	95.87	96.25	96.11	96.16	96.06	94.49	94.51	94.13	94.37	94.49	94.39	95.84	95.16	85.52	85.64	85.98	81.55
NIAH11434	2	1 (1)	99.61	99.58	99.74	99.75	99.67	99.88	99.65	100	99.70	99.75	99.62	99.78	99.67	99.37	97.53	96.95	97.08	97.17	96.81	96.23	96.55	96.55	96.50	96.70	96.73	96.80	96.77	96.87	96.74	96.71	96.80	96.77	96.79	96.60	96.64	96.57	95.61	95.90	95.74	96.65	96.53	96.63	96.67	96.11	96.49	95.92	96.26	96.14	96.25	96.15	94.54	94.58	94.15	94.39	94.52	94.43	95.88	95.20	85.81	85.99	86.00	81.56
DAT273	2	1 (1)	99.60	99.57	99.74	99.71	99.68	99.86	100	99.65	99.66	99.72	99.64	99.75	99.65	99.37	97.50	96.95	97.08	97.19	96.78	96.21	96.57	96.57	96.50	96.71	96.69	96.78	96.76	96.76	96.72	96.64	96.77	96.76	96.78	96.60	96.64	96.57	95.58	95.83	95.71	96.65	96.53	96.61	96.67	96.14	96.52	95.95	96.22	96.17	96.14	96.12	94.54	94.52	94.16	94.43	94.48	94.40	95.88	95.20	86.01	86.31	86.16	81.61
P1/7	2	1 (1)	99.67	99.63	99.81	99.68	99.71	100	99.73	99.74	99.71	99.68	99.77	99.72	99.66	99.46	97.51	96.96	97.05	97.16	96.82	96.16	96.61	96.60	96.63	96.68	96.71	96.73	96.78	96.78	96.77	96.81	96.75	96.71	96.72	96.64	96.65	96.61	95.87	96.17	96.02	96.88	96.80	96.66	96.68	96.10	96.51	96.14	96.24	96.17	96.20	96.14	94.52	94.47	94.05	94.33	94.49	94.38	95.90	95.16	85.94	86.16	86.29	81.55
SS12	1/2	1 (1)	99.45	99.42	99.59	99.61	100	99.71	99.55	99.53	99.49	99.58	99.49	99.61	99.57	99.31	97.50	96.90	97.02	97.15	96.78	96.15	96.58	96.58	96.52	96.69	96.71	96.76	96.75	96.73	96.73	96.56	96.71	96.72	96.73	96.62	96.65	96.57	95.58	95.85	95.71	96.62	96.57	96.58	96.61	96.08	96.52	95.93	96.26	96.16	96.15	96.16	94.48	94.51	94.12	94.36	94.52	94.45	95.84	95.19	85.76	86.05	86.24	81.53
A7	2	7 (1)	99.54	99.52	99.68	100	99.61	99.68	99.60	99.62	99.60	99.55	99.65	99.59	99.54	99.35	97.44	96.90	97.00	97.12	96.77	96.11	96.57	96.57	96.58	96.63	96.67	96.68	96.63	96.74	96.71	96.75	96.68	96.67	96.68	96.49	96.49	96.45	95.75	96.13	95.89	96.86	96.78	96.59	96.64	96.01	96.49	96.12	96.23	96.14	96.19	96.10	94.51	94.48	94.08	94.44	94.59	94.44	95.87	95.14	85.77	86.02	86.25	81.57
SC84	2	7 (1)	99.68	99.65	100	99.68	99.59	99.81	99.61	99.61	99.59	99.67	99.66	99.71	99.66	99.35	97.51	96.91	97.05	97.16	96.81	96.07	96.52	96.52	96.53	96.61	96.71	96.73	96.70	96.71	96.70	96.56	96.67	96.63	96.65	96.55	96.58	96.53	95.53	95.83	95.66	96.65	96.56	96.62	96.63	96.01	96.42	95.86	96.24	96.11	96.11	96.11	94.53	94.44	94.03	94.35	94.45	94.36	95.83	95.15	86.33	86.33	86.51	82.03
05ZYH33	2	7 (1)	99.51	100	99.65	99.52	99.42	99.63	99.44	99.44	99.41	99.51	99.50	99.52	99.48	99.19	97.34	96.75	96.91	97.03	96.68	95.98	96.38	96.38	96.37	96.46	96.56	96.58	96.55	96.56	96.55	96.42	96.53	96.51	96.52	96.41	96.46	96.41	95.40	95.69	95.54	96.51	96.42	96.48	96.51	95.89	96.30	95.74	96.09	95.98	95.98	95.98	94.42	94.31	93.94	94.25	94.32	94.25	95.69	95.01	86.08	86.17	86.39	81.58
98HAH33	2	7 (1)	100	99.51	99.68	99.54	99.45	99.67	99.48	99.47	99.44	99.54	99.53	99.57	99.52	99.22	97.39	96.78	96.95	97.04	96.71	96.02	96.41	96.41	96.42	96.49	96.60	96.61	96.59	96.60	96.59	96.47	96.56	96.52	96.54	96.44	96.47	96.42	95.43	95.70	95.55	96.53	96.44	96.51	96.52	95.91	96.30	95.76	96.13	96.01	96.00	95.99	94.43	94.33	93.94	94.26	94.34	94.27	95.71	95.05	86.27	86.38	86.23	81.65

ANIm values;
- 100%
- 98.0-99.99%
- 96.0-97.99%
- 95.0-95.99%
- 93.0-94.99%
- 90% <

Table S5. Precence/absence of each HG among 57 *S. suis* strains

				1																13			25					28						104		94			76			53/54		Divergent *S. suis*																
Index[a]	ClutserID	Gene products	Members	98HAH33	05ZYH33	SC84	A7	SS12	P1/7	DAT273	NIAH11434	JS14	GZ1	BM407	S735	13730	MNCM07	NCTC 10446	14636	NCTC 10237	ST1	2651	89-1591	MNCM04	MNCM25	8074	D9	4961	ST3	MNCM43	DAT292	DAT260	NIAH11435	MNCM21	MNCM50	89-2479	DAT234	DAT290	93A	42A	NT77	6407	11538	8830	12814	92-1400	89-590	2726	2524	89-3576-3	4417	D12	10581	89-5259	88-5299A	R61	92-1191	14A	92-4172	22083
1	1	ABC-type multidrug transport system, ATPase component	57	+	+	+	+	+	+	+	+	+	+	+	+	+	+	+	+	+	+	+	+	+	+	+	+	+	+	+	+	+	+	+	+	+	+	+	+	+	+	+	+	+	+	+	+	+	+	+	+	+	+	+	+	+	+	+	+	+
2	3	Phosphate transport ATP-binding protein PstB (TC 3.A.1.7.1)	57	+	+	+	+	+	+	+	+	+	+	+	+	+	+	+	+	+	+	+	+	+	+	+	+	+	+	+	+	+	+	+	+	+	+	+	+	+	+	+	+	+	+	+	+	+	+	+	+	+	+	+	+	+	+	+	+	+
3	5	Ribose-phosphate pyrophosphokinase (EC 2.7.6.1)	57	+	+	+	+	+	+	+	+	+	+	+	+	+	+	+	+	+	+	+	+	+	+	+	+	+	+	+	+	+	+	+	+	+	+	+	+	+	+	+	+	+	+	+	+	+	+	+	+	+	+	+	+	+	+	+	+	+
4	6	DNA gyrase subunit B (EC 5.99.1.3)	57	+	+	+	+	+	+	+	+	+	+	+	+	+	+	+	+	+	+	+	+	+	+	+	+	+	+	+	+	+	+	+	+	+	+	+	+	+	+	+	+	+	+	+	+	+	+	+	+	+	+	+	+	+	+	+	+	+
5	8	Tagatose 1,6-bisphosphate aldolase (EC 4.1.2.40)	57	+	+	+	+	+	+	+	+	+	+	+	+	+	+	+	+	+	+	+	+	+	+	+	+	+	+	+	+	+	+	+	+	+	+	+	+	+	+	+	+	+	+	+	+	+	+	+	+	+	+	+	+	+	+	+	+	+
6	9	2-keto-3-deoxy-D-arabino-heptulosonate-7-phosphate synthase I alpha (EC 2.5.1.54)	57	+	+	+	+	+	+	+	+	+	+	+	+	+	+	+	+	+	+	+	+	+	+	+	+	+	+	+	+	+	+	+	+	+	+	+	+	+	+	+	+	+	+	+	+	+	+	+	+	+	+	+	+	+	+	+	+	+
7	11	ABC transporter ATP-binding protein	57	+	+	+	+	+	+	+	+	+	+	+	+	+	+	+	+	+	+	+	+	+	+	+	+	+	+	+	+	+	+	+	+	+	+	+	+	+	+	+	+	+	+	+	+	+	+	+	+	+	+	+	+	+	+	+	+	+
8	12	Maltodextrin phosphorylase (EC 2.4.1.1)	57	+	+	+	+	+	+	+	+	+	+	+	+	+	+	+	+	+	+	+	+	+	+	+	+	+	+	+	+	+	+	+	+	+	+	+	+	+	+	+	+	+	+	+	+	+	+	+	+	+	+	+	+	+	+	+	+	+
9	13	4-alpha-glucanotransferase (amylomaltase) (EC 2.4.1.25)	57	+	+	+	+	+	+	+	+	+	+	+	+	+	+	+	+	+	+	+	+	+	+	+	+	+	+	+	+	+	+	+	+	+	+	+	+	+	+	+	+	+	+	+	+	+	+	+	+	+	+	+	+	+	+	+	+	+
10	14	Transcriptional regulator, PadR family	57	+	+	+	+	+	+	+	+	+	+	+	+	+	+	+	+	+	+	+	+	+	+	+	+	+	+	+	+	+	+	+	+	+	+	+	+	+	+	+	+	+	+	+	+	+	+	+	+	+	+	+	+	+	+	+	+	+
11	16	Beta-glucosidase (EC 3.2.1.21)	57	+	+	+	+	+	+	+	+	+	+	+	+	+	+	+	+	+	+	+	+	+	+	+	+	+	+	+	+	+	+	+	+	+	+	+	+	+	+	+	+	+	+	+	+	+	+	+	+	+	+	+	+	+	+	+	+	+
12	18	Single-stranded DNA-binding protein	57	+	+	+	+	+	+	+	+	+	+	+	+	+	+	+	+	+	+	+	+	+	+	+	+	+	+	+	+	+	+	+	+	+	+	+	+	+	+	+	+	+	+	+	+	+	+	+	+	+	+	+	+	+	+	+	+	+
13	20	Alpha-D-GlcNAc alpha-1,2-L-rhamnosyltransferase (EC 2.4.1.-)	57	+	+	+	+	+	+	+	+	+	+	+	+	+	+	+	+	+	+	+	+	+	+	+	+	+	+	+	+	+	+	+	+	+	+	+	+	+	+	+	+	+	+	+	+	+	+	+	+	+	+	+	+	+	+	+	+	+
14	23	hypothetical protein	57	+	+	+	+	+	+	+	+	+	+	+	+	+	+	+	+	+	+	+	+	+	+	+	+	+	+	+	+	+	+	+	+	+	+	+	+	+	+	+	+	+	+	+	+	+	+	+	+	+	+	+	+	+	+	+	+	+
15	24	Formate--tetrahydrofolate ligase (EC 6.3.4.3)	57	+	+	+	+	+	+	+	+	+	+	+	+	+	+	+	+	+	+	+	+	+	+	+	+	+	+	+	+	+	+	+	+	+	+	+	+	+	+	+	+	+	+	+	+	+	+	+	+	+	+	+	+	+	+	+	+	+
16	26	Iron-sulfur cluster assembly protein SufB	57	+	+	+	+	+	+	+	+	+	+	+	+	+	+	+	+	+	+	+	+	+	+	+	+	+	+	+	+	+	+	+	+	+	+	+	+	+	+	+	+	+	+	+	+	+	+	+	+	+	+	+	+	+	+	+	+	+
17	29	Nucleoside-binding protein	57	+	+	+	+	+	+	+	+	+	+	+	+	+	+	+	+	+	+	+	+	+	+	+	+	+	+	+	+	+	+	+	+	+	+	+	+	+	+	+	+	+	+	+	+	+	+	+	+	+	+	+	+	+	+	+	+	+
18	30	Glucokinase (EC 2.7.1.2)	57	+	+	+	+	+	+	+	+	+	+	+	+	+	+	+	+	+	+	+	+	+	+	+	+	+	+	+	+	+	+	+	+	+	+	+	+	+	+	+	+	+	+	+	+	+	+	+	+	+	+	+	+	+	+	+	+	+
19	32	Aspartyl-tRNA synthetase (EC 6.1.1.12)	57	+	+	+	+	+	+	+	+	+	+	+	+	+	+	+	+	+	+	+	+	+	+	+	+	+	+	+	+	+	+	+	+	+	+	+	+	+	+	+	+	+	+	+	+	+	+	+	+	+	+	+	+	+	+	+	+	+
20	34	Hypothetical, related to broad specificity phosphatases COG0406	57	+	+	+	+	+	+	+	+	+	+	+	+	+	+	+	+	+	+	+	+	+	+	+	+	+	+	+	+	+	+	+	+	+	+	+	+	+	+	+	+	+	+	+	+	+	+	+	+	+	+	+	+	+	+	+	+	+
21	35	ABC transporter, ATP-binding protein	57	+	+	+	+	+	+	+	+	+	+	+	+	+	+	+	+	+	+	+	+	+	+	+	+	+	+	+	+	+	+	+	+	+	+	+	+	+	+	+	+	+	+	+	+	+	+	+	+	+	+	+	+	+	+	+	+	+
22	37	Thioredoxin	57	+	+	+	+	+	+	+	+	+	+	+	+	+	+	+	+	+	+	+	+	+	+	+	+	+	+	+	+	+	+	+	+	+	+	+	+	+	+	+	+	+	+	+	+	+	+	+	+	+	+	+	+	+	+	+	+	+
23	38	NAD-dependent glyceraldehyde-3-phosphate dehydrogenase (EC 1.2.1.12) / Plasmin(ogen) receptor	57	+	+	+	+	+	+	+	+	+	+	+	+	+	+	+	+	+	+	+	+	+	+	+	+	+	+	+	+	+	+	+	+	+	+	+	+	+	+	+	+	+	+	+	+	+	+	+	+	+	+	+	+	+	+	+	+	+
24	39	Multiple sugar ABC transporter, ATP-binding protein	57	+	+	+	+	+	+	+	+	+	+	+	+	+	+	+	+	+	+	+	+	+	+	+	+	+	+	+	+	+	+	+	+	+	+	+	+	+	+	+	+	+	+	+	+	+	+	+	+	+	+	+	+	+	+	+	+	+
25	40	Acetyltransferase (EC 2.3.1.-)	57	+	+	+	+	+	+	+	+	+	+	+	+	+	+	+	+	+	+	+	+	+	+	+	+	+	+	+	+	+	+	+	+	+	+	+	+	+	+	+	+	+	+	+	+	+	+	+	+	+	+	+	+	+	+	+	+	+
26	41	UTP--glucose-1-phosphate uridylyltransferase (EC 2.7.7.9)	57	+	+	+	+	+	+	+	+	+	+	+	+	+	+	+	+	+	+	+	+	+	+	+	+	+	+	+	+	+	+	+	+	+	+	+	+	+	+	+	+	+	+	+	+	+	+	+	+	+	+	+	+	+	+	+	+	+
27	43	Acetate kinase (EC 2.7.2.1)	57	+	+	+	+	+	+	+	+	+	+	+	+	+	+	+	+	+	+	+	+	+	+	+	+	+	+	+	+	+	+	+	+	+	+	+	+	+	+	+	+	+	+	+	+	+	+	+	+	+	+	+	+	+	+	+	+	+
28	44	Universal stress protein family	57	+	+	+	+	+	+	+	+	+	+	+	+	+	+	+	+	+	+	+	+	+	+	+	+	+	+	+	+	+	+	+	+	+	+	+	+	+	+	+	+	+	+	+	+	+	+	+	+	+	+	+	+	+	+	+	+	+
29	47	Ornithine carbamoyltransferase (EC 2.1.3.3)	57	+	+	+	+	+	+	+	+	+	+	+	+	+	+	+	+	+	+	+	+	+	+	+	+	+	+	+	+	+	+	+	+	+	+	+	+	+	+	+	+	+	+	+	+	+	+	+	+	+	+	+	+	+	+	+	+	+
30	48	S-ribosylhomocysteine lyase (EC 4.4.1.21) / Autoinducer-2 production protein LuxS	57	+	+	+	+	+	+	+	+	+	+	+	+	+	+	+	+	+	+	+	+	+	+	+	+	+	+	+	+	+	+	+	+	+	+	+	+	+	+	+	+	+	+	+	+	+	+	+	+	+	+	+	+	+	+	+	+	+
31	51	Phosphate regulon transcriptional regulatory protein PhoB (SphR)	57	+	+	+	+	+	+	+	+	+	+	+	+	+	+	+	+	+	+	+	+	+	+	+	+	+	+	+	+	+	+	+	+	+	+	+	+	+	+	+	+	+	+	+	+	+	+	+	+	+	+	+	+	+	+	+	+	+
32	52	Acyltransferase family	57	+	+	+	+	+	+	+	+	+	+	+	+	+	+	+	+	+	+	+	+	+	+	+	+	+	+	+	+	+	+	+	+	+	+	+	+	+	+	+	+	+	+	+	+	+	+	+	+	+	+	+	+	+	+	+	+	+
33	55	Bactoprenol glucosyl transferase (EC 2.4.1.-)	57	+	+	+	+	+	+	+	+	+	+	+	+	+	+	+	+	+	+	+	+	+	+	+	+	+	+	+	+	+	+	+	+	+	+	+	+	+	+	+	+	+	+	+	+	+	+	+	+	+	+	+	+	+	+	+	+	+
34	56	Hypothetical protein VC0266 (sugar utilization related?)	57	+	+	+	+	+	+	+	+	+	+	+	+	+	+	+	+	+	+	+	+	+	+	+	+	+	+	+	+	+	+	+	+	+	+	+	+	+	+	+	+	+	+	+	+	+	+	+	+	+	+	+	+	+	+	+	+	+
35	57	N-acetylglucosamine-1-phosphate uridyltransferase (EC 2.7.7.23) / Glucosamine-1-phosphate N-acetyltransferase (EC 2.3.1.157)	57	+	+	+	+	+	+	+	+	+	+	+	+	+	+	+	+	+	+	+	+	+	+	+	+	+	+	+	+	+	+	+	+	+	+	+	+	+	+	+	+	+	+	+	+	+	+	+	+	+	+	+	+	+	+	+	+	+
36	58	tRNA (Guanine37-N1) -methyltransferase (EC 2.1.1.31)	57	+	+	+	+	+	+	+	+	+	+	+	+	+	+	+	+	+	+	+	+	+	+	+	+	+	+	+	+	+	+	+	+	+	+	+	+	+	+	+	+	+	+	+	+	+	+	+	+	+	+	+	+	+	+	+	+	+
37	59	Fructokinase (EC 2.7.1.4)	57	+	+	+	+	+	+	+	+	+	+	+	+	+	+	+	+	+	+	+	+	+	+	+	+	+	+	+	+	+	+	+	+	+	+	+	+	+	+	+	+	+	+	+	+	+	+	+	+	+	+	+	+	+	+	+	+	+
38	60	Glutamine synthetase type I (EC 6.3.1.2)	57	+	+	+	+	+	+	+	+	+	+	+	+	+	+	+	+	+	+	+	+	+	+	+	+	+	+	+	+	+	+	+	+	+	+	+	+	+	+	+	+	+	+	+	+	+	+	+	+	+	+	+	+	+	+	+	+	+
39	62	23S rRNA (Uracil-5-) -methyltransferase RumA (EC 2.1.1.-) ## LSU rRNA m(5)U1939	57	+	+	+	+	+	+	+	+	+	+	+	+	+	+	+	+	+	+	+	+	+	+	+	+	+	+	+	+	+	+	+	+	+	+	+	+	+	+	+	+	+	+	+	+	+	+	+	+	+	+	+	+	+	+	+	+	+
40	63	bacterial seryl-tRNA synthetase related	57	+	+	+	+	+	+	+	+	+	+	+	+	+	+	+	+	+	+	+	+	+	+	+	+	+	+	+	+	+	+	+	+	+	+	+	+	+	+	+	+	+	+	+	+	+	+	+	+	+	+	+	+	+	+	+	+	+
41	64	Ribonucleotide reduction protein NrdI	57	+	+	+	+	+	+	+	+	+	+	+	+	+	+	+	+	+	+	+	+	+	+	+	+	+	+	+	+	+	+	+	+	+	+	+	+	+	+	+	+	+	+	+	+	+	+	+	+	+	+	+	+	+	+	+	+	+
42	65	Ribonucleotide reductase of class Ib (aerobic), beta subunit (EC 1.17.4.1)	57	+	+	+	+	+	+	+	+	+	+	+	+	+	+	+	+	+	+	+	+	+	+	+	+	+	+	+	+	+	+	+	+	+	+	+	+	+	+	+	+	+	+	+	+	+	+	+	+	+	+	+	+	+	+	+	+	+
43	66	Replicative DNA helicase (EC 3.6.1.-)	57	+	+	+	+	+	+	+	+	+	+	+	+	+	+	+	+	+	+	+	+	+	+	+	+	+	+	+	+	+	+	+	+	+	+	+	+	+	+	+	+	+	+	+	+	+	+	+	+	+	+	+	+	+	+	+	+	+
44	67	16S rRNA processing protein RimM	57	+	+	+	+	+	+	+	+	+	+	+	+	+	+	+	+	+	+	+	+	+	+	+	+	+	+	+	+	+	+	+	+	+	+	+	+	+	+	+	+	+	+	+	+	+	+	+	+	+	+	+	+	+	+	+	+	+
45	68	Sortase A, LPXTG specific	57	+	+	+	+	+	+	+	+	+	+	+	+	+	+	+	+	+	+	+	+	+	+	+	+	+	+	+	+	+	+	+	+	+	+	+	+	+	+	+	+	+	+	+	+	+	+	+	+	+	+	+	+	+	+	+	+	+
46	69	Hypothetical protein possible functionally linked with Alanyl-tRNA synthetase	57	+	+	+	+	+	+	+	+	+	+	+	+	+	+	+	+	+	+	+	+	+	+	+	+	+	+	+	+	+	+	+	+	+	+	+	+	+	+	+	+	+	+	+	+	+	+	+	+	+	+	+	+	+	+	+	+	+
47	70	Immunoreactive protein Se23.5 (Fragment)	57	+	+	+	+	+	+	+	+	+	+	+	+	+	+	+	+	+	+	+	+	+	+	+	+	+	+	+	+	+	+	+	+	+	+	+	+	+	+	+	+	+	+	+	+	+	+	+	+	+	+	+	+	+	+	+	+	+
48	71	hypothetical protein	57	+	+	+	+	+	+	+	+	+	+	+	+	+	+	+	+	+	+	+	+	+	+	+	+	+	+	+	+	+	+	+	+	+	+	+	+	+	+	+	+	+	+	+	+	+	+	+	+	+	+	+	+	+	+	+	+	+
49	72	L-lactate dehydrogenase (EC 1.1.1.27)	57	+	+	+	+	+	+	+	+	+	+	+	+	+	+	+	+	+	+	+	+	+	+	+	+	+	+	+	+	+	+	+	+	+	+	+	+	+	+	+	+	+	+	+	+	+	+	+	+	+	+	+	+	+	+	+	+	+
50	73	Methionine ABC transporter permease protein	57	+	+	+	+	+	+	+	+	+	+	+	+	+	+	+	+	+	+	+	+	+	+	+	+	+	+	+	+	+	+	+	+	+	+	+	+	+	+	+	+	+	+	+	+	+	+	+	+	+	+	+	+	+	+	+	+	+
51	74	Alanine racemase (EC 5.1.1.1)	57	+	+	+	+	+	+	+	+	+	+	+	+	+	+	+	+	+	+	+	+	+	+	+	+	+	+	+	+	+	+	+	+	+	+	+	+	+	+	+	+	+	+	+	+	+	+	+	+	+	+	+	+	+	+	+	+	+
52	76	Holo-[acyl-carrier protein] synthase (EC 2.7.8.7)	57	+	+	+	+	+	+	+	+	+	+	+	+	+	+	+	+	+	+	+	+	+	+	+	+	+	+	+	+	+	+	+	+	+	+	+	+	+	+	+	+	+	+	+	+	+	+	+	+	+	+	+	+	+	+	+	+	+
53	78	FIG107367: Single-stranded DNA-binding protein	57	+	+	+	+	+	+	+	+	+	+	+	+	+	+	+	+	+	+	+	+	+	+	+	+	+	+	+	+	+	+	+	+	+	+	+	+	+	+	+	+	+	+	+	+	+	+	+	+	+	+	+	+	+	+	+	+	+
54	79	Enoyl-[acyl-carrier-protein] reductase [FMN] (EC 1.3.1.9)	57	+	+	+	+	+	+	+	+	+	+	+	+	+	+	+	+	+	+	+	+	+	+	+	+	+	+	+	+	+	+	+	+	+	+	+	+	+	+	+	+	+	+	+	+	+	+	+	+	+	+	+	+	+	+	+	+	+
55	80	Cysteinyl-tRNA synthetase related protein	57	+	+	+	+	+	+	+	+	+	+	+	+	+	+	+	+	+	+	+	+	+	+	+	+	+	+	+	+	+	+	+	+	+	+	+	+	+	+	+	+	+	+	+	+	+	+	+	+	+	+	+	+	+	+	+	+	+
56	81	Methionine ABC transporter ATP-binding protein	57	+	+	+	+	+	+	+	+	+	+	+	+	+	+	+	+	+	+	+	+	+	+	+	+	+	+	+	+	+	+	+	+	+	+	+	+	+	+	+	+	+	+	+	+	+	+	+	+	+	+	+	+	+	+	+	+	+
57	82	tRNA (adenine37-N(6))-methyltransferase TrmN6 (EC 2.1.1.223)	57	+	+	+	+	+	+	+	+	+	+	+	+	+	+	+	+	+	+	+	+	+	+	+	+	+	+	+	+	+	+	+	+	+	+	+	+	+	+	+	+	+	+	+	+	+	+	+	+	+	+	+	+	+	+	+	+	+
58	83	Shikimate kinase I (EC 2.7.1.71)	57	+	+	+	+	+	+	+	+	+	+	+	+	+	+	+	+	+	+	+	+	+	+	+	+	+	+	+	+	+	+	+	+	+	+	+	+	+	+	+	+	+	+	+	+	+	+	+	+	+	+	+	+	+	+	+	+	+
59	84	3-oxoacyl-[acyl-carrier protein] reductase (EC 1.1.1.100)	57	+	+	+	+	+	+	+	+	+	+	+	+	+	+	+	+	+	+	+	+	+	+	+	+	+	+	+	+	+	+	+	+	+	+	+	+	+	+	+	+	+	+	+	+	+	+	+	+	+	+	+	+	+	+	+	+	+
60	85	Substrate-specific component PanT of predicted pantothenate ECF transporter	57	+	+	+	+	+	+	+	+	+	+	+	+	+	+	+	+	+	+	+	+	+	+	+	+	+	+	+	+	+	+	+	+	+	+	+	+	+	+	+	+	+	+	+	+	+	+	+	+	+	+	+	+	+	+	+	+	+
61	86	Prolipoprotein diacylglyceryl transferase (EC 2.4.99.-)	57	+	+	+	+	+	+	+	+	+	+	+	+	+	+	+	+	+	+	+	+	+	+	+	+	+	+	+	+	+	+	+	+	+	+	+	+	+	+	+	+	+	+	+	+	+	+	+	+	+	+	+	+	+	+	+	+	+
62	87	Adenylate kinase (EC 2.7.4.3)	57	+	+	+	+	+	+	+	+	+	+	+	+	+	+	+	+	+	+	+	+	+	+	+	+	+	+	+	+	+	+	+	+	+	+	+	+	+	+	+	+	+	+	+	+	+	+	+	+	+	+	+	+	+	+	+	+	+
63	88	SSU ribosomal protein S4p (S9e)	57	+	+	+	+	+	+	+	+	+	+	+	+	+	+	+	+	+	+	+	+	+	+	+	+	+	+	+	+	+	+	+	+	+	+	+	+	+	+	+	+	+	+	+	+	+	+	+	+	+	+	+	+	+	+	+	+	+
64	89	ATPase YjeE, predicted to have essential role in cell wall biosynthesis	57	+	+	+	+	+	+	+	+	+	+	+	+	+	+	+	+	+	+	+	+	+	+	+	+	+	+	+	+	+	+	+	+	+	+	+	+	+	+	+	+	+	+	+	+	+	+	+	+	+	+	+	+	+	+	+	+	+
65	90	Short chain dehydrogenase	57	+	+	+	+	+	+	+	+	+	+	+	+	+	+	+	+	+	+	+	+	+	+	+	+	+	+	+	+	+	+	+	+	+	+	+	+	+	+	+	+	+	+	+	+	+	+	+	+	+	+	+	+	+	+	+	+	+
66	91	DNA polymerase I (EC 2.7.7.7)	57	+	+	+	+	+	+	+	+	+	+	+	+	+	+	+	+	+	+	+	+	+	+	+	+	+	+	+	+	+	+	+	+	+	+	+	+	+	+	+	+	+	+	+	+	+	+	+	+	+	+	+	+	+	+	+	+	+
67	92	Excinuclease ABC subunit A	57	+	+	+	+	+	+	+	+	+	+	+	+	+	+	+	+	+	+	+	+	+	+	+	+	+	+	+	+	+	+	+	+	+	+	+	+	+	+	+	+	+	+	+	+	+	+	+	+	+	+	+	+	+	+	+	+	+
68	93	Integral membrane protein	57	+	+	+	+	+	+	+	+	+	+	+	+	+	+	+	+	+	+	+	+	+	+	+	+	+	+	+	+	+	+	+	+	+	+	+	+	+	+	+	+	+	+	+	+	+	+	+	+	+	+	+	+	+	+	+	+	+
69	94	Ribulose-phosphate 3-epimerase (EC 5.1.3.1)	57	+	+	+	+	+	+	+	+	+	+	+	+	+	+	+	+	+	+	+	+	+	+	+	+	+	+	+	+	+	+	+	+	+	+	+	+	+	+	+	+	+	+	+	+	+	+	+	+	+	+	+	+	+	+	+	+	+
70	95	tRNA (guanine46-N7-)-methyltransferase (EC 2.1.1.33)	57	+	+	+	+	+	+	+	+	+	+	+	+	+	+	+	+	+	+	+	+	+	+	+	+	+	+	+	+	+	+	+	+	+	+	+	+	+	+	+	+	+	+	+	+	+	+	+	+	+	+	+	+	+	+	+	+	+
71	96	Magnesium and cobalt transport protein CorA	57	+	+	+	+	+	+	+	+	+	+	+	+	+	+	+	+	+	+	+	+	+	+	+	+	+	+	+	+	+	+	+	+	+	+	+	+	+	+	+	+	+	+	+	+	+	+	+	+	+	+	+	+	+	+	+	+	+
72	97	Holliday junction DNA helicase RuvA	57	+	+	+	+	+	+	+	+	+	+	+	+	+	+	+	+	+	+	+	+	+	+	+	+	+	+	+	+	+	+	+	+	+	+	+	+	+	+	+	+	+	+	+	+	+	+	+	+	+	+	+	+	+	+	+	+	+
73	98	Hemolysin III	57	+	+	+	+	+	+	+	+	+	+	+	+	+	+	+	+	+	+	+	+	+	+	+	+	+	+	+	+	+	+	+	+	+	+	+	+	+	+	+	+	+	+	+	+	+	+	+	+	+	+	+	+	+	+	+	+	+
74	99	Protein-N(5)-glutamine methyltransferase PrmC, methylates polypeptide chain release factors RF1 and RF2	57	+	+	+	+	+	+	+	+	+	+	+	+	+	+	+	+	+	+	+	+	+	+	+	+	+	+	+	+	+	+	+	+	+	+	+	+	+	+	+	+	+	+	+	+	+	+	+	+	+	+	+	+	+	+	+	+	+
75	100	Prolyl-tRNA synthetase (EC 6.1.1.15)	57	+	+	+	+	+	+	+	+	+	+	+	+	+	+	+	+	+	+	+	+	+	+	+	+	+	+	+	+	+	+	+	+	+	+	+	+	+	+	+	+	+	+	+	+	+	+	+	+	+	+	+	+	+	+	+	+	+
76	101	Signal recognition particle associated protein	57	+	+	+	+	+	+	+	+	+	+	+	+	+	+	+	+	+	+	+	+	+	+	+	+	+	+	+	+	+	+	+	+	+	+	+	+	+	+	+	+	+	+	+	+	+	+	+	+	+	+	+	+	+	+	+	+	+
77	102	Transcriptional regulator SpxA2	57	+	+	+	+	+	+	+	+	+	+	+	+	+	+	+	+	+	+	+	+	+	+	+	+	+	+	+	+	+	+	+	+	+	+	+	+	+	+	+	+	+	+	+	+	+	+	+	+	+	+	+	+	+	+	+	+	+
78	103	Transcriptional regulator SpxA1	57	+	+	+	+	+	+	+	+	+	+	+	+	+	+	+	+	+	+	+	+	+	+	+	+	+	+	+	+	+	+	+	+	+	+	+	+	+	+	+	+	+	+	+	+	+	+	+	+	+	+	+	+	+	+	+	+	+
79	104	Ribonuclease III (EC 3.1.26.3)	57	+	+	+	+	+	+	+	+	+	+	+	+	+	+	+	+	+	+	+	+	+	+	+	+	+	+	+	+	+	+	+	+	+	+	+	+	+	+	+	+	+	+	+	+	+	+	+	+	+	+	+	+	+	+	+	+	+
80	105	Ribosome small subunit-stimulated GTPase EngC	57	+	+	+	+	+	+	+	+	+	+	+	+	+	+	+	+	+	+	+	+	+	+	+	+	+	+	+	+	+	+	+	+	+	+	+	+	+	+	+	+	+	+	+	+	+	+	+	+	+	+	+	+	+	+	+	+	+
81	106	ATP synthase gamma chain (EC 3.6.3.14)	57	+	+	+	+	+	+	+	+	+	+	+	+	+	+	+	+	+	+	+	+	+	+	+	+	+	+	+	+	+	+	+	+	+	+	+	+	+	+	+	+	+	+	+	+	+	+	+	+	+	+	+	+	+	+	+	+	+
82	107	Putative iron-sulfur cluster assembly scaffold protein for SUF system, SufE2	57	+	+	+	+	+	+	+	+	+	+	+	+	+	+	+	+	+	+	+	+	+	+	+	+	+	+	+	+	+	+	+	+	+	+	+	+	+	+	+	+	+	+	+	+	+	+	+	+	+	+	+	+	+	+	+	+	+
83	108	NAD synthetase (EC 6.3.1.5)	57	+	+	+	+	+	+	+	+	+	+	+	+	+	+	+	+	+	+	+	+	+	+	+	+	+	+	+	+	+	+	+	+	+	+	+	+	+	+	+	+	+	+	+	+	+	+	+	+	+	+	+	+	+	+	+	+	+
84	109	hypothetical protein	57	+	+	+	+	+	+	+	+	+	+	+	+	+	+	+	+	+	+	+	+	+	+	+	+	+	+	+	+	+	+	+	+	+	+	+	+	+	+	+	+	+	+	+	+	+	+	+	+	+	+	+	+	+	+	+	+	+
85	110	Pyruvate formate-lyase (EC 2.3.1.54)	57	+	+	+	+	+	+	+	+	+	+	+	+	+	+	+	+	+	+	+	+	+	+	+	+	+	+	+	+	+	+	+	+	+	+	+	+	+	+	+	+	+	+	+	+	+	+	+	+	+	+	+	+	+	+	+	+	+
86	111	ABC transporter ATP-binding protein	57	+	+	+	+	+	+	+	+	+	+	+	+	+	+	+	+	+	+	+	+	+	+	+	+	+	+	+	+	+	+	+	+	+	+	+	+	+	+	+	+	+	+	+	+	+	+	+	+	+	+	+	+	+	+	+	+	+
87	112	Candidate zinc-binding lipoprotein ZinT	57	+	+	+	+	+	+	+	+	+	+	+	+	+	+	+	+	+	+	+	+	+	+	+	+	+	+	+	+	+	+	+	+	+	+	+	+	+	+	+	+	+	+	+	+	+	+	+	+	+	+	+	+	+	+	+	+	+
88	113	Hydrolase (HAD superfamily), YqeK	57	+	+	+	+	+	+	+	+	+	+	+	+	+	+	+	+	+	+	+	+	+	+	+	+	+	+	+	+	+	+	+	+	+	+	+	+	+	+	+	+	+	+	+	+	+	+	+	+	+	+	+	+	+	+	+	+	+
89	114	Iojap protein	57	+	+	+	+	+	+	+	+	+	+	+	+	+	+	+	+	+	+	+	+	+	+	+	+	+	+	+	+	+	+	+	+	+	+	+	+	+	+	+	+	+	+	+	+	+	+	+	+	+	+	+	+	+	+	+	+	+
90	115	Ribonucleotide reductase transcriptional regulator NrdR	57	+	+	+	+	+	+	+	+	+	+	+	+	+	+	+	+	+	+	+	+	+	+	+	+	+	+	+	+	+	+	+	+	+	+	+	+	+	+	+	+	+	+	+	+	+	+	+	+	+	+	+	+	+	+	+	+	+
91	116	DNA-directed RNA polymerase omega subunit (EC 2.7.7.6)	57	+	+	+	+	+	+	+	+	+	+	+	+	+	+	+	+	+	+	+	+	+	+	+	+	+	+	+	+	+	+	+	+	+	+	+	+	+	+	+	+	+	+	+	+	+	+	+	+	+	+	+	+	+	+	+	+	+
92	117	Ribonucleotide reductase of class III (anaerobic), activating protein (EC 1.97.1.4)	57	+	+	+	+	+	+	+	+	+	+	+	+	+	+	+	+	+	+	+	+	+	+	+	+	+	+	+	+	+	+	+	+	+	+	+	+	+	+	+	+	+	+	+	+	+	+	+	+	+	+	+	+	+	+	+	+	+
93	118	Cell division protein FtsA	57	+	+	+	+	+	+	+	+	+	+	+	+	+	+	+	+	+	+	+	+	+	+	+	+	+	+	+	+	+	+	+	+	+	+	+	+	+	+	+	+	+	+	+	+	+	+	+	+	+	+	+	+	+	+	+	+	+
94	119	Sensor histidine kinase VraS	57	+	+	+	+	+	+	+	+	+	+	+	+	+	+	+	+	+	+	+	+	+	+	+	+	+	+	+	+	+	+	+	+	+	+	+	+	+	+	+	+	+	+	+	+	+	+	+	+	+	+	+	+	+	+	+	+	+
95	120	Cysteinyl-tRNA synthetase (EC 6.1.1.16)	57	+	+	+	+	+	+	+	+	+	+	+	+	+	+	+	+	+	+	+	+	+	+	+	+	+	+	+	+	+	+	+	+	+	+	+	+	+	+	+	+	+	+	+	+	+	+	+	+	+	+	+	+	+	+	+	+	+
96	121	RNA binding methyltransferase FtsJ like	57	+	+	+	+	+	+	+	+	+	+	+	+	+	+	+	+	+	+	+	+	+	+	+	+	+	+	+	+	+	+	+	+	+	+	+	+	+	+	+	+	+	+	+	+	+	+	+	+	+	+	+	+	+	+	+	+	+
97	122	Transmembrane component of general energizing module of ECF transporters	57	+	+	+	+	+	+	+	+	+	+	+	+	+	+	+	+	+	+	+	+	+	+	+	+	+	+	+	+	+	+	+	+	+	+	+	+	+	+	+	+	+	+	+	+	+	+	+	+	+	+	+	+	+	+	+	+	+
98	123	Thymidylate kinase (EC 2.7.4.9)	57	+	+	+	+	+	+	+	+	+	+	+	+	+	+	+	+	+	+	+	+	+	+	+	+	+	+	+	+	+	+	+	+	+	+	+	+	+	+	+	+	+	+	+	+	+	+	+	+	+	+	+	+	+	+	+	+	+
99	124	Membrane protein involved in the export of O-antigen, teichoic acid lipoteichoic acids	57	+	+	+	+	+	+	+	+	+	+	+	+	+	+	+	+	+	+	+	+	+	+	+	+	+	+	+	+	+	+	+	+	+	+	+	+	+	+	+	+	+	+	+	+	+	+	+	+	+	+	+	+	+	+	+	+	+
100	125	GTP-binding protein TypA/BipA	57	+	+	+	+	+	+	+	+	+	+	+	+	+	+	+	+	+	+	+	+	+	+	+	+	+	+	+	+	+	+	+	+	+	+	+	+	+	+	+	+	+	+	+	+	+	+	+	+	+	+	+	+	+	+	+	+	+
101	126	Malonyl CoA-acyl carrier protein transacylase (EC 2.3.1.39)	57	+	+	+	+	+	+	+	+	+	+	+	+	+	+	+	+	+	+	+	+	+	+	+	+	+	+	+	+	+	+	+	+	+	+	+	+	+	+	+	+	+	+	+	+	+	+	+	+	+	+	+	+	+	+	+	+	+
102	127	DNA-directed RNA polymerase beta' subunit (EC 2.7.7.6)	57	+	+	+	+	+	+	+	+	+	+	+	+	+	+	+	+	+	+	+	+	+	+	+	+	+	+	+	+	+	+	+	+	+	+	+	+	+	+	+	+	+	+	+	+	+	+	+	+	+	+	+	+	+	+	+	+	+
103	128	Phosphopantothenoylcysteine decarboxylase (EC 4.1.1.36)	57	+	+	+	+	+	+	+	+	+	+	+	+	+	+	+	+	+	+	+	+	+	+	+	+	+	+	+	+	+	+	+	+	+	+	+	+	+	+	+	+	+	+	+	+	+	+	+	+	+	+	+	+	+	+	+	+	+
104	129	FIG002813: LPPG:FO 2-phospho-L-lactate transferase like, CofD-like	57	+	+	+	+	+	+	+	+	+	+	+	+	+	+	+	+	+	+	+	+	+	+	+	+	+	+	+	+	+	+	+	+	+	+	+	+	+	+	+	+	+	+	+	+	+	+	+	+	+	+	+	+	+	+	+	+	+
105	130	Putative Holliday junction resolvase YggF	57	+	+	+	+	+	+	+	+	+	+	+	+	+	+	+	+	+	+	+	+	+	+	+	+	+	+	+	+	+	+	+	+	+	+	+	+	+	+	+	+	+	+	+	+	+	+	+	+	+	+	+	+	+	+	+	+	+
106	131	Substrate-specific component PdxU2 of predicted pyridoxin-related ECF transporter	57	+	+	+	+	+	+	+	+	+	+	+	+	+	+	+	+	+	+	+	+	+	+	+	+	+	+	+	+	+	+	+	+	+	+	+	+	+	+	+	+	+	+	+	+	+	+	+	+	+	+	+	+	+	+	+	+	+
107	133	LSU ribosomal protein L16p (L10e)	57	+	+	+	+	+	+	+	+	+	+	+	+	+	+	+	+	+	+	+	+	+	+	+	+	+	+	+	+	+	+	+	+	+	+	+	+	+	+	+	+	+	+	+	+	+	+	+	+	+	+	+	+	+	+	+	+	+
108	134	Chromosome replication initiation protein dnaD	57	+	+	+	+	+	+	+	+	+	+	+	+	+	+	+	+	+	+	+	+	+	+	+	+	+	+	+	+	+	+	+	+	+	+	+	+	+	+	+	+	+	+	+	+	+	+	+	+	+	+	+	+	+	+	+	+	+
109	135	Phosphate:acyl-ACP acyltransferase PlsX	57	+	+	+	+	+	+	+	+	+	+	+	+	+	+	+	+	+	+	+	+	+	+	+	+	+	+	+	+	+	+	+	+	+	+	+	+	+	+	+	+	+	+	+	+	+	+	+	+	+	+	+	+	+	+	+	+	+
110	136	DNA primase (EC 2.7.7.-)	57	+	+	+	+	+	+	+	+	+	+	+	+	+	+	+	+	+	+	+	+	+	+	+	+	+	+	+	+	+	+	+	+	+	+	+	+	+	+	+	+	+	+	+	+	+	+	+	+	+	+	+	+	+	+	+	+	+
111	137	Regulatory protein recX	57	+	+	+	+	+	+	+	+	+	+	+	+	+	+	+	+	+	+	+	+	+	+	+	+	+	+	+	+	+	+	+	+	+	+	+	+	+	+	+	+	+	+	+	+	+	+	+	+	+	+	+	+	+	+	+	+	+
112	138	Nicotinamidase (EC 3.5.1.19)	57	+	+	+	+	+	+	+	+	+	+	+	+	+	+	+	+	+	+	+	+	+	+	+	+	+	+	+	+	+	+	+	+	+	+	+	+	+	+	+	+	+	+	+	+	+	+	+	+	+	+	+	+	+	+	+	+	+
113	139	Cytidylate kinase (EC 2.7.4.14)	57	+	+	+	+	+	+	+	+	+	+	+	+	+	+	+	+	+	+	+	+	+	+	+	+	+	+	+	+	+	+	+	+	+	+	+	+	+	+	+	+	+	+	+	+	+	+	+	+	+	+	+	+	+	+	+	+	+
114	140	Glutaredoxin-like protein NrdH, required for reduction of Ribonucleotide reductase class Ib	57	+	+	+	+	+	+	+	+	+	+	+	+	+	+	+	+	+	+	+	+	+	+	+	+	+	+	+	+	+	+	+	+	+	+	+	+	+	+	+	+	+	+	+	+	+	+	+	+	+	+	+	+	+	+	+	+	+
115	141	Beta-lactamase, class C	57	+	+	+	+	+	+	+	+	+	+	+	+	+	+	+	+	+	+	+	+	+	+	+	+	+	+	+	+	+	+	+	+	+	+	+	+	+	+	+	+	+	+	+	+	+	+	+	+	+	+	+	+	+	+	+	+	+
116	142	FIG009886: phosphoesterase	57	+	+	+	+	+	+	+	+	+	+	+	+	+	+	+	+	+	+	+	+	+	+	+	+	+	+	+	+	+	+	+	+	+	+	+	+	+	+	+	+	+	+	+	+	+	+	+	+	+	+	+	+	+	+	+	+	+
117	143	Dihydrofolate reductase (EC 1.5.1.3)	57	+	+	+	+	+	+	+	+	+	+	+	+	+	+	+	+	+	+	+	+	+	+	+	+	+	+	+	+	+	+	+	+	+	+	+	+	+	+	+	+	+	+	+	+	+	+	+	+	+	+	+	+	+	+	+	+	+
118	144	ribosomal protein L7Ae family protein	57	+	+	+	+	+	+	+	+	+	+	+	+	+	+	+	+	+	+	+	+	+	+	+	+	+	+	+	+	+	+	+	+	+	+	+	+	+	+	+	+	+	+	+	+	+	+	+	+	+	+	+	+	+	+	+	+	+
119	145	Inner membrane protein translocase component YidC, OxaA protein	57	+	+	+	+	+	+	+	+	+	+	+	+	+	+	+	+	+	+	+	+	+	+	+	+	+	+	+	+	+	+	+	+	+	+	+	+	+	+	+	+	+	+	+	+	+	+	+	+	+	+	+	+	+	+	+	+	+
120	146	Phosphatidate cytidylyltransferase (EC 2.7.7.41)	57	+	+	+	+	+	+	+	+	+	+	+	+	+	+	+	+	+	+	+	+	+	+	+	+	+	+	+	+	+	+	+	+	+	+	+	+	+	+	+	+	+	+	+	+	+	+	+	+	+	+	+	+	+	+	+	+	+
121	147	FIG009688: Thioredoxin	57	+	+	+	+	+	+	+	+	+	+	+	+	+	+	+	+	+	+	+	+	+	+	+	+	+	+	+	+	+	+	+	+	+	+	+	+	+	+	+	+	+	+	+	+	+	+	+	+	+	+	+	+	+	+	+	+	+
122	148	Chromosome (plasmid) partitioning protein ParB / Stage 0 sporulation protein J	57	+	+	+	+	+	+	+	+	+	+	+	+	+	+	+	+	+	+	+	+	+	+	+	+	+	+	+	+	+	+	+	+	+	+	+	+	+	+	+	+	+	+	+	+	+	+	+	+	+	+	+	+	+	+	+	+	+
123	149	Phosphoglycerate kinase (EC 2.7.2.3)	57	+	+	+	+	+	+	+	+	+	+	+	+	+	+	+	+	+	+	+	+	+	+	+	+	+	+	+	+	+	+	+	+	+	+	+	+	+	+	+	+	+	+	+	+	+	+	+	+	+	+	+	+	+	+	+	+	+
124	150	Aspartate aminotransferase (EC 2.6.1.1)	57	+	+	+	+	+	+	+	+	+	+	+	+	+	+	+	+	+	+	+	+	+	+	+	+	+	+	+	+	+	+	+	+	+	+	+	+	+	+	+	+	+	+	+	+	+	+	+	+	+	+	+	+	+	+	+	+	+
125	151	Lipoprotein signal peptidase (EC 3.4.23.36)	57	+	+	+	+	+	+	+	+	+	+	+	+	+	+	+	+	+	+	+	+	+	+	+	+	+	+	+	+	+	+	+	+	+	+	+	+	+	+	+	+	+	+	+	+	+	+	+	+	+	+	+	+	+	+	+	+	+
126	152	Glycosyltransferase LafA, responsible for the formation of Glc-DAG	57	+	+	+	+	+	+	+	+	+	+	+	+	+	+	+	+	+	+	+	+	+	+	+	+	+	+	+	+	+	+	+	+	+	+	+	+	+	+	+	+	+	+	+	+	+	+	+	+	+	+	+	+	+	+	+	+	+
127	153	Polyribonucleotide nucleotidyltransferase (EC 2.7.7.8)	57	+	+	+	+	+	+	+	+	+	+	+	+	+	+	+	+	+	+	+	+	+	+	+	+	+	+	+	+	+	+	+	+	+	+	+	+	+	+	+	+	+	+	+	+	+	+	+	+	+	+	+	+	+	+	+	+	+
128	154	NAD kinase (EC 2.7.1.23)	57	+	+	+	+	+	+	+	+	+	+	+	+	+	+	+	+	+	+	+	+	+	+	+	+	+	+	+	+	+	+	+	+	+	+	+	+	+	+	+	+	+	+	+	+	+	+	+	+	+	+	+	+	+	+	+	+	+
129	155	hypothetical protein	57	+	+	+	+	+	+	+	+	+	+	+	+	+	+	+	+	+	+	+	+	+	+	+	+	+	+	+	+	+	+	+	+	+	+	+	+	+	+	+	+	+	+	+	+	+	+	+	+	+	+	+	+	+	+	+	+	+
130	156	Hypothetical protein YbbP, contains nucleotide-binding domain of DisA bacterial checkpoint controller	57	+	+	+	+	+	+	+	+	+	+	+	+	+	+	+	+	+	+	+	+	+	+	+	+	+	+	+	+	+	+	+	+	+	+	+	+	+	+	+	+	+	+	+	+	+	+	+	+	+	+	+	+	+	+	+	+	+
131	157	FIG004454: RNA binding protein	57	+	+	+	+	+	+	+	+	+	+	+	+	+	+	+	+	+	+	+	+	+	+	+	+	+	+	+	+	+	+	+	+	+	+	+	+	+	+	+	+	+	+	+	+	+	+	+	+	+	+	+	+	+	+	+	+	+
132	158	Methionyl-tRNA formyltransferase (EC 2.1.2.9)	57	+	+	+	+	+	+	+	+	+	+	+	+	+	+	+	+	+	+	+	+	+	+	+	+	+	+	+	+	+	+	+	+	+	+	+	+	+	+	+	+	+	+	+	+	+	+	+	+	+	+	+	+	+	+	+	+	+
133	159	Sodium/glycine symporter GlyP	57	+	+	+	+	+	+	+	+	+	+	+	+	+	+	+	+	+	+	+	+	+	+	+	+	+	+	+	+	+	+	+	+	+	+	+	+	+	+	+	+	+	+	+	+	+	+	+	+	+	+	+	+	+	+	+	+	+
134	160	5-Enolpyruvylshikimate-3-phosphate synthase (EC 2.5.1.19)	57	+	+	+	+	+	+	+	+	+	+	+	+	+	+	+	+	+	+	+	+	+	+	+	+	+	+	+	+	+	+	+	+	+	+	+	+	+	+	+	+	+	+	+	+	+	+	+	+	+	+	+	+	+	+	+	+	+
135	161	DNA polymerase IV (EC 2.7.7.7)	57	+	+	+	+	+	+	+	+	+	+	+	+	+	+	+	+	+	+	+	+	+	+	+	+	+	+	+	+	+	+	+	+	+	+	+	+	+	+	+	+	+	+	+	+	+	+	+	+	+	+	+	+	+	+	+	+	+
136	162	FIG001553: Hydrolase, HAD subfamily IIIA	57	+	+	+	+	+	+	+	+	+	+	+	+	+	+	+	+	+	+	+	+	+	+	+	+	+	+	+	+	+	+	+	+	+	+	+	+	+	+	+	+	+	+	+	+	+	+	+	+	+	+	+	+	+	+	+	+	+
137	163	Acetyl-coenzyme A carboxyl transferase beta chain (EC 6.4.1.2)	57	+	+	+	+	+	+	+	+	+	+	+	+	+	+	+	+	+	+	+	+	+	+	+	+	+	+	+	+	+	+	+	+	+	+	+	+	+	+	+	+	+	+	+	+	+	+	+	+	+	+	+	+	+	+	+	+	+
138	164	GTP-binding protein EngB	57	+	+	+	+	+	+	+	+	+	+	+	+	+	+	+	+	+	+	+	+	+	+	+	+	+	+	+	+	+	+	+	+	+	+	+	+	+	+	+	+	+	+	+	+	+	+	+	+	+	+	+	+	+	+	+	+	+
139	165	FIG004453: protein YceG like	57	+	+	+	+	+	+	+	+	+	+	+	+	+	+	+	+	+	+	+	+	+	+	+	+	+	+	+	+	+	+	+	+	+	+	+	+	+	+	+	+	+	+	+	+	+	+	+	+	+	+	+	+	+	+	+	+	+
140	166	LSU ribosomal protein L9p	57	+	+	+	+	+	+	+	+	+	+	+	+	+	+	+	+	+	+	+	+	+	+	+	+	+	+	+	+	+	+	+	+	+	+	+	+	+	+	+	+	+	+	+	+	+	+	+	+	+	+	+	+	+	+	+	+	+
141	167	LSU ribosomal protein L11p (L12e)	57	+	+	+	+	+	+	+	+	+	+	+	+	+	+	+	+	+	+	+	+	+	+	+	+	+	+	+	+	+	+	+	+	+	+	+	+	+	+	+	+	+	+	+	+	+	+	+	+	+	+	+	+	+	+	+	+	+
142	168	Recombination inhibitory protein MutS2	57	+	+	+	+	+	+	+	+	+	+	+	+	+	+	+	+	+	+	+	+	+	+	+	+	+	+	+	+	+	+	+	+	+	+	+	+	+	+	+	+	+	+	+	+	+	+	+	+	+	+	+	+	+	+	+	+	+
143	169	Ribosome recycling factor	57	+	+	+	+	+	+	+	+	+	+	+	+	+	+	+	+	+	+	+	+	+	+	+	+	+	+	+	+	+	+	+	+	+	+	+	+	+	+	+	+	+	+	+	+	+	+	+	+	+	+	+	+	+	+	+	+	+
144	170	SSU ribosomal protein S17p (S11e)	57	+	+	+	+	+	+	+	+	+	+	+	+	+	+	+	+	+	+	+	+	+	+	+	+	+	+	+	+	+	+	+	+	+	+	+	+	+	+	+	+	+	+	+	+	+	+	+	+	+	+	+	+	+	+	+	+	+
145	171	Chaperone protein DnaJ	57	+	+	+	+	+	+	+	+	+	+	+	+	+	+	+	+	+	+	+	+	+	+	+	+	+	+	+	+	+	+	+	+	+	+	+	+	+	+	+	+	+	+	+	+	+	+	+	+	+	+	+	+	+	+	+	+	+
146	172	probably aromatic ring hydroxylating enzyme, evidenced by COGnitor; PaaD-like protein (DUF59) involved in Fe-S cluster assembly	57	+	+	+	+	+	+	+	+	+	+	+	+	+	+	+	+	+	+	+	+	+	+	+	+	+	+	+	+	+	+	+	+	+	+	+	+	+	+	+	+	+	+	+	+	+	+	+	+	+	+	+	+	+	+	+	+	+
147	173	Phage lysin, glycosyl hydrolase, family 25	57	+	+	+	+	+	+	+	+	+	+	+	+	+	+	+	+	+	+	+	+	+	+	+	+	+	+	+	+	+	+	+	+	+	+	+	+	+	+	+	+	+	+	+	+	+	+	+	+	+	+	+	+	+	+	+	+	+
148	174	ATP-dependent DNA helicase UvrD/PcrA	57	+	+	+	+	+	+	+	+	+	+	+	+	+	+	+	+	+	+	+	+	+	+	+	+	+	+	+	+	+	+	+	+	+	+	+	+	+	+	+	+	+	+	+	+	+	+	+	+	+	+	+	+	+	+	+	+	+
149	175	ATPase component of general energizing module of ECF transporters	57	+	+	+	+	+	+	+	+	+	+	+	+	+	+	+	+	+	+	+	+	+	+	+	+	+	+	+	+	+	+	+	+	+	+	+	+	+	+	+	+	+	+	+	+	+	+	+	+	+	+	+	+	+	+	+	+	+

Table S5. Precence/absence of each HG among 57 *S. suis* strains

				1																13			25						28						104		94			76			53/54		Divergent *S. suis*															
Index[a]	ClusterID	Gene products	Members	98HAH33	05ZYH33	SC84	A7	SS12	P1/7	DAT273	NIAH11434	JS14	GZ1	BM407	S735	13730	MNCM07	NCTC 10446	14636	NCTC 10237	ST1	2651	89-1591	MNCM04	MNCM25	8074	D9	4961	ST3	MNCM43	DAT292	DAT260	NIAH11435	MNCM21	MNCM50	89-2479	DAT234	DAT290	93A	42A	NT77	6407	11538	8830	12814	92-1400	89-590	2726	2524	89-3576-3	4417	D12	10581	89-5259	88-5299A	R61	92-1191	14A	92-4172	22083
150	176	Phosphate ABC transporter, periplasmic phosphate-binding protein PstS (TC 3.A.1.7.1)	57	+	+	+	+	+	+	+	+	+	+	+	+	+	+	+	+	+	+	+	+	+	+	+	+	+	+	+	+	+	+	+	+	+	+	+	+	+	+	+	+	+	+	+	+	+	+	+	+	+	+	+	+	+
151	177	Lysyl-tRNA synthetase (class II) (EC 6.1.1.6)	57	+	+	+	+	+	+	+	+	+	+	+	+	+	+	+	+	+	+	+	+	+	+	+	+	+	+	+	+	+	+	+	+	+	+	+	+	+	+	+	+	+	+	+	+	+	+	+	+	+	+	+	+	+
152	178	ComF operon protein A, DNA transporter ATPase	57	+	+	+	+	+	+	+	+	+	+	+	+	+	+	+	+	+	+	+	+	+	+	+	+	+	+	+	+	+	+	+	+	+	+	+	+	+	+	+	+	+	+	+	+	+	+	+	+	+	+	+	+	+
153	179	Rossmann fold nucleotide-binding protein Smf possibly involved in DNA uptake	57	+	+	+	+	+	+	+	+	+	+	+	+	+	+	+	+	+	+	+	+	+	+	+	+	+	+	+	+	+	+	+	+	+	+	+	+	+	+	+	+	+	+	+	+	+	+	+	+	+	+	+	+	+
154	180	Alkaline shock protein	57	+	+	+	+	+	+	+	+	+	+	+	+	+	+	+	+	+	+	+	+	+	+	+	+	+	+	+	+	+	+	+	+	+	+	+	+	+	+	+	+	+	+	+	+	+	+	+	+	+	+	+	+	+
155	181	LSU ribosomal protein L2p (L8e)	57	+	+	+	+	+	+	+	+	+	+	+	+	+	+	+	+	+	+	+	+	+	+	+	+	+	+	+	+	+	+	+	+	+	+	+	+	+	+	+	+	+	+	+	+	+	+	+	+	+	+	+	+	+
156	182	hypothetical protein	57	+	+	+	+	+	+	+	+	+	+	+	+	+	+	+	+	+	+	+	+	+	+	+	+	+	+	+	+	+	+	+	+	+	+	+	+	+	+	+	+	+	+	+	+	+	+	+	+	+	+	+	+	+
157	183	DNA gyrase subunit A (EC 5.99.1.3)	57	+	+	+	+	+	+	+	+	+	+	+	+	+	+	+	+	+	+	+	+	+	+	+	+	+	+	+	+	+	+	+	+	+	+	+	+	+	+	+	+	+	+	+	+	+	+	+	+	+	+	+	+	+
158	184	ABC transporter, ATP-binding protein EcsA	57	+	+	+	+	+	+	+	+	+	+	+	+	+	+	+	+	+	+	+	+	+	+	+	+	+	+	+	+	+	+	+	+	+	+	+	+	+	+	+	+	+	+	+	+	+	+	+	+	+	+	+	+	+
159	185	FIG042801: CBS domain containing protein	57	+	+	+	+	+	+	+	+	+	+	+	+	+	+	+	+	+	+	+	+	+	+	+	+	+	+	+	+	+	+	+	+	+	+	+	+	+	+	+	+	+	+	+	+	+	+	+	+	+	+	+	+	+
160	186	Exodeoxyribonuclease VII large subunit (EC 3.1.11.6)	57	+	+	+	+	+	+	+	+	+	+	+	+	+	+	+	+	+	+	+	+	+	+	+	+	+	+	+	+	+	+	+	+	+	+	+	+	+	+	+	+	+	+	+	+	+	+	+	+	+	+	+	+	+
161	187	Adenylate cyclase	57	+	+	+	+	+	+	+	+	+	+	+	+	+	+	+	+	+	+	+	+	+	+	+	+	+	+	+	+	+	+	+	+	+	+	+	+	+	+	+	+	+	+	+	+	+	+	+	+	+	+	+	+	+
162	188	DNA recombination and repair protein RecO	57	+	+	+	+	+	+	+	+	+	+	+	+	+	+	+	+	+	+	+	+	+	+	+	+	+	+	+	+	+	+	+	+	+	+	+	+	+	+	+	+	+	+	+	+	+	+	+	+	+	+	+	+	+
163	189	Adenylosuccinate synthetase (EC 6.3.4.4)	57	+	+	+	+	+	+	+	+	+	+	+	+	+	+	+	+	+	+	+	+	+	+	+	+	+	+	+	+	+	+	+	+	+	+	+	+	+	+	+	+	+	+	+	+	+	+	+	+	+	+	+	+	+
164	190	Arsenate reductase (EC 1.20.4.1)	57	+	+	+	+	+	+	+	+	+	+	+	+	+	+	+	+	+	+	+	+	+	+	+	+	+	+	+	+	+	+	+	+	+	+	+	+	+	+	+	+	+	+	+	+	+	+	+	+	+	+	+	+	+
165	191	SAM-dependent methyltransferase, MraW methylase family (EC 2.1.1.-)	57	+	+	+	+	+	+	+	+	+	+	+	+	+	+	+	+	+	+	+	+	+	+	+	+	+	+	+	+	+	+	+	+	+	+	+	+	+	+	+	+	+	+	+	+	+	+	+	+	+	+	+	+	+
166	192	3-oxoacyl-[acyl-carrier-protein] synthase, KASIII (EC 2.3.1.41)	57	+	+	+	+	+	+	+	+	+	+	+	+	+	+	+	+	+	+	+	+	+	+	+	+	+	+	+	+	+	+	+	+	+	+	+	+	+	+	+	+	+	+	+	+	+	+	+	+	+	+	+	+	+
167	193	Hypothetical protein SP1558	57	+	+	+	+	+	+	+	+	+	+	+	+	+	+	+	+	+	+	+	+	+	+	+	+	+	+	+	+	+	+	+	+	+	+	+	+	+	+	+	+	+	+	+	+	+	+	+	+	+	+	+	+	+
168	194	possible permease	57	+	+	+	+	+	+	+	+	+	+	+	+	+	+	+	+	+	+	+	+	+	+	+	+	+	+	+	+	+	+	+	+	+	+	+	+	+	+	+	+	+	+	+	+	+	+	+	+	+	+	+	+	+
169	195	Cell division trigger factor (EC 5.2.1.8)	57	+	+	+	+	+	+	+	+	+	+	+	+	+	+	+	+	+	+	+	+	+	+	+	+	+	+	+	+	+	+	+	+	+	+	+	+	+	+	+	+	+	+	+	+	+	+	+	+	+	+	+	+	+
170	196	FIG005935: membrane protein	57	+	+	+	+	+	+	+	+	+	+	+	+	+	+	+	+	+	+	+	+	+	+	+	+	+	+	+	+	+	+	+	+	+	+	+	+	+	+	+	+	+	+	+	+	+	+	+	+	+	+	+	+	+
171	197	Multimodular transpeptidase-transglycosylase (EC 2.4.1.129) (EC 3.4.-.-) / Penicillin-binding protein 1A/1B (PBP1)	57	+	+	+	+	+	+	+	+	+	+	+	+	+	+	+	+	+	+	+	+	+	+	+	+	+	+	+	+	+	+	+	+	+	+	+	+	+	+	+	+	+	+	+	+	+	+	+	+	+	+	+	+	+
172	198	Ca ion P-type ATPase	57	+	+	+	+	+	+	+	+	+	+	+	+	+	+	+	+	+	+	+	+	+	+	+	+	+	+	+	+	+	+	+	+	+	+	+	+	+	+	+	+	+	+	+	+	+	+	+	+	+	+	+	+	+
173	199	Translation elongation factor G	57	+	+	+	+	+	+	+	+	+	+	+	+	+	+	+	+	+	+	+	+	+	+	+	+	+	+	+	+	+	+	+	+	+	+	+	+	+	+	+	+	+	+	+	+	+	+	+	+	+	+	+	+	+
174	200	GTP-binding protein EngA	57	+	+	+	+	+	+	+	+	+	+	+	+	+	+	+	+	+	+	+	+	+	+	+	+	+	+	+	+	+	+	+	+	+	+	+	+	+	+	+	+	+	+	+	+	+	+	+	+	+	+	+	+	+
175	201	tRNA uridine 5-carboxymethylaminomethyl modification enzyme GidA	57	+	+	+	+	+	+	+	+	+	+	+	+	+	+	+	+	+	+	+	+	+	+	+	+	+	+	+	+	+	+	+	+	+	+	+	+	+	+	+	+	+	+	+	+	+	+	+	+	+	+	+	+	+
176	202	peptidase, U32 family large subunit [C1]	57	+	+	+	+	+	+	+	+	+	+	+	+	+	+	+	+	+	+	+	+	+	+	+	+	+	+	+	+	+	+	+	+	+	+	+	+	+	+	+	+	+	+	+	+	+	+	+	+	+	+	+	+	+
177	203	Holliday junction DNA helicase RuvB	57	+	+	+	+	+	+	+	+	+	+	+	+	+	+	+	+	+	+	+	+	+	+	+	+	+	+	+	+	+	+	+	+	+	+	+	+	+	+	+	+	+	+	+	+	+	+	+	+	+	+	+	+	+
178	204	Ribosomal RNA large subunit methyltransferase N (EC 2.1.1.-)	57	+	+	+	+	+	+	+	+	+	+	+	+	+	+	+	+	+	+	+	+	+	+	+	+	+	+	+	+	+	+	+	+	+	+	+	+	+	+	+	+	+	+	+	+	+	+	+	+	+	+	+	+	+
179	205	FIG000506: Predicted P-loop-containing kinase	57	+	+	+	+	+	+	+	+	+	+	+	+	+	+	+	+	+	+	+	+	+	+	+	+	+	+	+	+	+	+	+	+	+	+	+	+	+	+	+	+	+	+	+	+	+	+	+	+	+	+	+	+	+
180	206	Ribosomal subunit interface protein	57	+	+	+	+	+	+	+	+	+	+	+	+	+	+	+	+	+	+	+	+	+	+	+	+	+	+	+	+	+	+	+	+	+	+	+	+	+	+	+	+	+	+	+	+	+	+	+	+	+	+	+	+	+
181	207	Arginine pathway regulatory protein ArgR, repressor of arg regulon	57	+	+	+	+	+	+	+	+	+	+	+	+	+	+	+	+	+	+	+	+	+	+	+	+	+	+	+	+	+	+	+	+	+	+	+	+	+	+	+	+	+	+	+	+	+	+	+	+	+	+	+	+	+
182	208	Undecaprenyl diphosphate synthase (EC 2.5.1.31)	57	+	+	+	+	+	+	+	+	+	+	+	+	+	+	+	+	+	+	+	+	+	+	+	+	+	+	+	+	+	+	+	+	+	+	+	+	+	+	+	+	+	+	+	+	+	+	+	+	+	+	+	+	+
183	209	ATP-dependent RNA helicase YqfR	57	+	+	+	+	+	+	+	+	+	+	+	+	+	+	+	+	+	+	+	+	+	+	+	+	+	+	+	+	+	+	+	+	+	+	+	+	+	+	+	+	+	+	+	+	+	+	+	+	+	+	+	+	+
184	210	SSU ribosomal protein S18p @ SSU ribosomal protein S18p, zinc-independent	57	+	+	+	+	+	+	+	+	+	+	+	+	+	+	+	+	+	+	+	+	+	+	+	+	+	+	+	+	+	+	+	+	+	+	+	+	+	+	+	+	+	+	+	+	+	+	+	+	+	+	+	+	+
185	211	LSU ribosomal protein L24p (L26e)	57	+	+	+	+	+	+	+	+	+	+	+	+	+	+	+	+	+	+	+	+	+	+	+	+	+	+	+	+	+	+	+	+	+	+	+	+	+	+	+	+	+	+	+	+	+	+	+	+	+	+	+	+	+
186	212	5'-methylthioadenosine nucleosidase (EC 3.2.2.16) / S-adenosylhomocysteine nucleosidase (EC 3.2.2.9)	57	+	+	+	+	+	+	+	+	+	+	+	+	+	+	+	+	+	+	+	+	+	+	+	+	+	+	+	+	+	+	+	+	+	+	+	+	+	+	+	+	+	+	+	+	+	+	+	+	+	+	+	+	+
187	213	UDP-N-acetylmuramate--alanine ligase (EC 6.3.2.8)	57	+	+	+	+	+	+	+	+	+	+	+	+	+	+	+	+	+	+	+	+	+	+	+	+	+	+	+	+	+	+	+	+	+	+	+	+	+	+	+	+	+	+	+	+	+	+	+	+	+	+	+	+	+
188	214	Fibronectin/fibrinogen-binding protein	57	+	+	+	+	+	+	+	+	+	+	+	+	+	+	+	+	+	+	+	+	+	+	+	+	+	+	+	+	+	+	+	+	+	+	+	+	+	+	+	+	+	+	+	+	+	+	+	+	+	+	+	+	+
189	215	COG1939: Ribonuclease III family protein	57	+	+	+	+	+	+	+	+	+	+	+	+	+	+	+	+	+	+	+	+	+	+	+	+	+	+	+	+	+	+	+	+	+	+	+	+	+	+	+	+	+	+	+	+	+	+	+	+	+	+	+	+	+
190	216	Predicted transcriptional regulator of pyridoxine metabolism	57	+	+	+	+	+	+	+	+	+	+	+	+	+	+	+	+	+	+	+	+	+	+	+	+	+	+	+	+	+	+	+	+	+	+	+	+	+	+	+	+	+	+	+	+	+	+	+	+	+	+	+	+	+
191	217	Zn-dependent hydrolase YycJ/WalJ, required for cell wall metabolism and coordination of cell division with DNA replication	57	+	+	+	+	+	+	+	+	+	+	+	+	+	+	+	+	+	+	+	+	+	+	+	+	+	+	+	+	+	+	+	+	+	+	+	+	+	+	+	+	+	+	+	+	+	+	+	+	+	+	+	+	+
192	218	Aspartyl-tRNA(Asn) amidotransferase subunit B (EC 6.3.5.6) @ Glutamyl-tRNA(Gln) amidotransferase subunit B (EC 6.3.5.7)	57	+	+	+	+	+	+	+	+	+	+	+	+	+	+	+	+	+	+	+	+	+	+	+	+	+	+	+	+	+	+	+	+	+	+	+	+	+	+	+	+	+	+	+	+	+	+	+	+	+	+	+	+	+
193	219	Catabolite control protein A	57	+	+	+	+	+	+	+	+	+	+	+	+	+	+	+	+	+	+	+	+	+	+	+	+	+	+	+	+	+	+	+	+	+	+	+	+	+	+	+	+	+	+	+	+	+	+	+	+	+	+	+	+	+
194	220	Segregation and condensation protein A	57	+	+	+	+	+	+	+	+	+	+	+	+	+	+	+	+	+	+	+	+	+	+	+	+	+	+	+	+	+	+	+	+	+	+	+	+	+	+	+	+	+	+	+	+	+	+	+	+	+	+	+	+	+
195	221	DNA repair protein RecN	57	+	+	+	+	+	+	+	+	+	+	+	+	+	+	+	+	+	+	+	+	+	+	+	+	+	+	+	+	+	+	+	+	+	+	+	+	+	+	+	+	+	+	+	+	+	+	+	+	+	+	+	+	+
196	222	LSU ribosomal protein L15p (L27Ae)	57	+	+	+	+	+	+	+	+	+	+	+	+	+	+	+	+	+	+	+	+	+	+	+	+	+	+	+	+	+	+	+	+	+	+	+	+	+	+	+	+	+	+	+	+	+	+	+	+	+	+	+	+	+
197	223	Protein of unknown function DUF1447	57	+	+	+	+	+	+	+	+	+	+	+	+	+	+	+	+	+	+	+	+	+	+	+	+	+	+	+	+	+	+	+	+	+	+	+	+	+	+	+	+	+	+	+	+	+	+	+	+	+	+	+	+	+
198	224	Helicase loader DnaI	57	+	+	+	+	+	+	+	+	+	+	+	+	+	+	+	+	+	+	+	+	+	+	+	+	+	+	+	+	+	+	+	+	+	+	+	+	+	+	+	+	+	+	+	+	+	+	+	+	+	+	+	+	+
199	225	hypothetical protein	57	+	+	+	+	+	+	+	+	+	+	+	+	+	+	+	+	+	+	+	+	+	+	+	+	+	+	+	+	+	+	+	+	+	+	+	+	+	+	+	+	+	+	+	+	+	+	+	+	+	+	+	+	+
200	226	3'-->5' exoribonuclease Bsu YhaM	57	+	+	+	+	+	+	+	+	+	+	+	+	+	+	+	+	+	+	+	+	+	+	+	+	+	+	+	+	+	+	+	+	+	+	+	+	+	+	+	+	+	+	+	+	+	+	+	+	+	+	+	+	+
201	227	SSU ribosomal protein S3p (S3e)	57	+	+	+	+	+	+	+	+	+	+	+	+	+	+	+	+	+	+	+	+	+	+	+	+	+	+	+	+	+	+	+	+	+	+	+	+	+	+	+	+	+	+	+	+	+	+	+	+	+	+	+	+	+
202	228	Aspartyl-tRNA(Asn) amidotransferase subunit A (EC 6.3.5.6) @ Glutamyl-tRNA(Gln) amidotransferase subunit A (EC 6.3.5.7)	57	+	+	+	+	+	+	+	+	+	+	+	+	+	+	+	+	+	+	+	+	+	+	+	+	+	+	+	+	+	+	+	+	+	+	+	+	+	+	+	+	+	+	+	+	+	+	+	+	+	+	+	+	+
203	229	Phenylalanyl-tRNA synthetase domain protein (Bsu YtpR)	57	+	+	+	+	+	+	+	+	+	+	+	+	+	+	+	+	+	+	+	+	+	+	+	+	+	+	+	+	+	+	+	+	+	+	+	+	+	+	+	+	+	+	+	+	+	+	+	+	+	+	+	+	+
204	230	COG2740: Predicted nucleic-acid-binding protein implicated in transcription termination	57	+	+	+	+	+	+	+	+	+	+	+	+	+	+	+	+	+	+	+	+	+	+	+	+	+	+	+	+	+	+	+	+	+	+	+	+	+	+	+	+	+	+	+	+	+	+	+	+	+	+	+	+	+
205	231	Phosphate starvation-inducible protein PhoH, predicted ATPase	57	+	+	+	+	+	+	+	+	+	+	+	+	+	+	+	+	+	+	+	+	+	+	+	+	+	+	+	+	+	+	+	+	+	+	+	+	+	+	+	+	+	+	+	+	+	+	+	+	+	+	+	+	+
206	232	Serine/threonine protein kinase PrkC, regulator of stationary phase	57	+	+	+	+	+	+	+	+	+	+	+	+	+	+	+	+	+	+	+	+	+	+	+	+	+	+	+	+	+	+	+	+	+	+	+	+	+	+	+	+	+	+	+	+	+	+	+	+	+	+	+	+	+
207	233	Ribosomal RNA small subunit methyltransferase D (EC 2.1.1.-)	57	+	+	+	+	+	+	+	+	+	+	+	+	+	+	+	+	+	+	+	+	+	+	+	+	+	+	+	+	+	+	+	+	+	+	+	+	+	+	+	+	+	+	+	+	+	+	+	+	+	+	+	+	+
208	234	GTP pyrophosphokinase (EC 2.7.6.5)	57	+	+	+	+	+	+	+	+	+	+	+	+	+	+	+	+	+	+	+	+	+	+	+	+	+	+	+	+	+	+	+	+	+	+	+	+	+	+	+	+	+	+	+	+	+	+	+	+	+	+	+	+	+
209	235	ABC transporter substrate-binding protein	57	+	+	+	+	+	+	+	+	+	+	+	+	+	+	+	+	+	+	+	+	+	+	+	+	+	+	+	+	+	+	+	+	+	+	+	+	+	+	+	+	+	+	+	+	+	+	+	+	+	+	+	+	+
210	236	Thioredoxin reductase (EC 1.8.1.9)	57	+	+	+	+	+	+	+	+	+	+	+	+	+	+	+	+	+	+	+	+	+	+	+	+	+	+	+	+	+	+	+	+	+	+	+	+	+	+	+	+	+	+	+	+	+	+	+	+	+	+	+	+	+
211	237	tRNA (cytosine34-2'-O-)-methyltransferase (EC 2.1.1.-)	57	+	+	+	+	+	+	+	+	+	+	+	+	+	+	+	+	+	+	+	+	+	+	+	+	+	+	+	+	+	+	+	+	+	+	+	+	+	+	+	+	+	+	+	+	+	+	+	+	+	+	+	+	+
212	238	DNA mismatch repair protein MutL	57	+	+	+	+	+	+	+	+	+	+	+	+	+	+	+	+	+	+	+	+	+	+	+	+	+	+	+	+	+	+	+	+	+	+	+	+	+	+	+	+	+	+	+	+	+	+	+	+	+	+	+	+	+
213	239	SSU ribosomal protein S6p	57	+	+	+	+	+	+	+	+	+	+	+	+	+	+	+	+	+	+	+	+	+	+	+	+	+	+	+	+	+	+	+	+	+	+	+	+	+	+	+	+	+	+	+	+	+	+	+	+	+	+	+	+	+
214	240	PROTEASE (EC 3.4.-.-)	57	+	+	+	+	+	+	+	+	+	+	+	+	+	+	+	+	+	+	+	+	+	+	+	+	+	+	+	+	+	+	+	+	+	+	+	+	+	+	+	+	+	+	+	+	+	+	+	+	+	+	+	+	+
215	241	DNA-entry nuclease (Competence-specific nuclease) (EC 3.1.30.-)	57	+	+	+	+	+	+	+	+	+	+	+	+	+	+	+	+	+	+	+	+	+	+	+	+	+	+	+	+	+	+	+	+	+	+	+	+	+	+	+	+	+	+	+	+	+	+	+	+	+	+	+	+	+
216	242	Permease of the drug/metabolite transporter (DMT) superfamily	57	+	+	+	+	+	+	+	+	+	+	+	+	+	+	+	+	+	+	+	+	+	+	+	+	+	+	+	+	+	+	+	+	+	+	+	+	+	+	+	+	+	+	+	+	+	+	+	+	+	+	+	+	+
217	243	DNA ligase (EC 6.5.1.2)	57	+	+	+	+	+	+	+	+	+	+	+	+	+	+	+	+	+	+	+	+	+	+	+	+	+	+	+	+	+	+	+	+	+	+	+	+	+	+	+	+	+	+	+	+	+	+	+	+	+	+	+	+	+
218	244	Cell division protein FtsZ (EC 3.4.24.-)	57	+	+	+	+	+	+	+	+	+	+	+	+	+	+	+	+	+	+	+	+	+	+	+	+	+	+	+	+	+	+	+	+	+	+	+	+	+	+	+	+	+	+	+	+	+	+	+	+	+	+	+	+	+
219	245	POSSIBLE SERINE/THREONINE PHOSPHATASE PPP (EC 3.1.3.16)	57	+	+	+	+	+	+	+	+	+	+	+	+	+	+	+	+	+	+	+	+	+	+	+	+	+	+	+	+	+	+	+	+	+	+	+	+	+	+	+	+	+	+	+	+	+	+	+	+	+	+	+	+	+
220	246	1-acyl-sn-glycerol-3-phosphate acyltransferase (EC 2.3.1.51)	57	+	+	+	+	+	+	+	+	+	+	+	+	+	+	+	+	+	+	+	+	+	+	+	+	+	+	+	+	+	+	+	+	+	+	+	+	+	+	+	+	+	+	+	+	+	+	+	+	+	+	+	+	+
221	247	Chaperone protein DnaK	57	+	+	+	+	+	+	+	+	+	+	+	+	+	+	+	+	+	+	+	+	+	+	+	+	+	+	+	+	+	+	+	+	+	+	+	+	+	+	+	+	+	+	+	+	+	+	+	+	+	+	+	+	+
222	248	DNA-3-methyladenine glycosylase (EC 3.2.2.20)	57	+	+	+	+	+	+	+	+	+	+	+	+	+	+	+	+	+	+	+	+	+	+	+	+	+	+	+	+	+	+	+	+	+	+	+	+	+	+	+	+	+	+	+	+	+	+	+	+	+	+	+	+	+
223	249	DNA polymerase III delta prime subunit (EC 2.7.7.7)	57	+	+	+	+	+	+	+	+	+	+	+	+	+	+	+	+	+	+	+	+	+	+	+	+	+	+	+	+	+	+	+	+	+	+	+	+	+	+	+	+	+	+	+	+	+	+	+	+	+	+	+	+	+
224	250	SWF/SNF family helicase	57	+	+	+	+	+	+	+	+	+	+	+	+	+	+	+	+	+	+	+	+	+	+	+	+	+	+	+	+	+	+	+	+	+	+	+	+	+	+	+	+	+	+	+	+	+	+	+	+	+	+	+	+	+
225	251	Ribonuclease M5 (EC 3.1.26.8)	57	+	+	+	+	+	+	+	+	+	+	+	+	+	+	+	+	+	+	+	+	+	+	+	+	+	+	+	+	+	+	+	+	+	+	+	+	+	+	+	+	+	+	+	+	+	+	+	+	+	+	+	+	+
226	252	Multimodular transpeptidase-transglycosylase (EC 2.4.1.129) (EC 3.4.-.-)	57	+	+	+	+	+	+	+	+	+	+	+	+	+	+	+	+	+	+	+	+	+	+	+	+	+	+	+	+	+	+	+	+	+	+	+	+	+	+	+	+	+	+	+	+	+	+	+	+	+	+	+	+	+
227	253	Ortholog of S. aureus MRSA252 (BX571856) SAR1694	57	+	+	+	+	+	+	+	+	+	+	+	+	+	+	+	+	+	+	+	+	+	+	+	+	+	+	+	+	+	+	+	+	+	+	+	+	+	+	+	+	+	+	+	+	+	+	+	+	+	+	+	+	+
228	254	Proline dipeptidase (EC 3.4.13.9)	57	+	+	+	+	+	+	+	+	+	+	+	+	+	+	+	+	+	+	+	+	+	+	+	+	+	+	+	+	+	+	+	+	+	+	+	+	+	+	+	+	+	+	+	+	+	+	+	+	+	+	+	+	+
229	255	Transcriptional repressor of the fructose operon, DeoR family	57	+	+	+	+	+	+	+	+	+	+	+	+	+	+	+	+	+	+	+	+	+	+	+	+	+	+	+	+	+	+	+	+	+	+	+	+	+	+	+	+	+	+	+	+	+	+	+	+	+	+	+	+	+
230	256	Conserved uncharacterized protein	57	+	+	+	+	+	+	+	+	+	+	+	+	+	+	+	+	+	+	+	+	+	+	+	+	+	+	+	+	+	+	+	+	+	+	+	+	+	+	+	+	+	+	+	+	+	+	+	+	+	+	+	+	+
231	257	S1 RNA binding domain	57	+	+	+	+	+	+	+	+	+	+	+	+	+	+	+	+	+	+	+	+	+	+	+	+	+	+	+	+	+	+	+	+	+	+	+	+	+	+	+	+	+	+	+	+	+	+	+	+	+	+	+	+	+
232	258	Thymidylate synthase (EC 2.1.1.45)	57	+	+	+	+	+	+	+	+	+	+	+	+	+	+	+	+	+	+	+	+	+	+	+	+	+	+	+	+	+	+	+	+	+	+	+	+	+	+	+	+	+	+	+	+	+	+	+	+	+	+	+	+	+
233	259	SSU ribosomal protein S13p (S18e)	57	+	+	+	+	+	+	+	+	+	+	+	+	+	+	+	+	+	+	+	+	+	+	+	+	+	+	+	+	+	+	+	+	+	+	+	+	+	+	+	+	+	+	+	+	+	+	+	+	+	+	+	+	+
234	260	Putative deoxyribose-specific ABC transporter, ATP-binding protein	57	+	+	+	+	+	+	+	+	+	+	+	+	+	+	+	+	+	+	+	+	+	+	+	+	+	+	+	+	+	+	+	+	+	+	+	+	+	+	+	+	+	+	+	+	+	+	+	+	+	+	+	+	+
235	261	Phosphate transport system regulatory protein PhoU	57	+	+	+	+	+	+	+	+	+	+	+	+	+	+	+	+	+	+	+	+	+	+	+	+	+	+	+	+	+	+	+	+	+	+	+	+	+	+	+	+	+	+	+	+	+	+	+	+	+	+	+	+	+
236	262	Iron-sulfur cluster assembly ATPase protein SufC	57	+	+	+	+	+	+	+	+	+	+	+	+	+	+	+	+	+	+	+	+	+	+	+	+	+	+	+	+	+	+	+	+	+	+	+	+	+	+	+	+	+	+	+	+	+	+	+	+	+	+	+	+	+
237	263	Cell division protein FtsK	57	+	+	+	+	+	+	+	+	+	+	+	+	+	+	+	+	+	+	+	+	+	+	+	+	+	+	+	+	+	+	+	+	+	+	+	+	+	+	+	+	+	+	+	+	+	+	+	+	+	+	+	+	+
238	264	Hydrolase (HAD superfamily)	57	+	+	+	+	+	+	+	+	+	+	+	+	+	+	+	+	+	+	+	+	+	+	+	+	+	+	+	+	+	+	+	+	+	+	+	+	+	+	+	+	+	+	+	+	+	+	+	+	+	+	+	+	+
239	265	ATP synthase beta chain (EC 3.6.3.14)	57	+	+	+	+	+	+	+	+	+	+	+	+	+	+	+	+	+	+	+	+	+	+	+	+	+	+	+	+	+	+	+	+	+	+	+	+	+	+	+	+	+	+	+	+	+	+	+	+	+	+	+	+	+
240	266	FIG001802: Putative alkaline-shock protein	57	+	+	+	+	+	+	+	+	+	+	+	+	+	+	+	+	+	+	+	+	+	+	+	+	+	+	+	+	+	+	+	+	+	+	+	+	+	+	+	+	+	+	+	+	+	+	+	+	+	+	+	+	+
241	267	ATP synthase alpha chain (EC 3.6.3.14)	57	+	+	+	+	+	+	+	+	+	+	+	+	+	+	+	+	+	+	+	+	+	+	+	+	+	+	+	+	+	+	+	+	+	+	+	+	+	+	+	+	+	+	+	+	+	+	+	+	+	+	+	+	+
242	268	Phosphoglycerate mutase (EC 5.4.2.1)	57	+	+	+	+	+	+	+	+	+	+	+	+	+	+	+	+	+	+	+	+	+	+	+	+	+	+	+	+	+	+	+	+	+	+	+	+	+	+	+	+	+	+	+	+	+	+	+	+	+	+	+	+	+
243	269	GTP-binding protein Era	57	+	+	+	+	+	+	+	+	+	+	+	+	+	+	+	+	+	+	+	+	+	+	+	+	+	+	+	+	+	+	+	+	+	+	+	+	+	+	+	+	+	+	+	+	+	+	+	+	+	+	+	+	+
244	270	Two-component sensor kinase SA14-24	57	+	+	+	+	+	+	+	+	+	+	+	+	+	+	+	+	+	+	+	+	+	+	+	+	+	+	+	+	+	+	+	+	+	+	+	+	+	+	+	+	+	+	+	+	+	+	+	+	+	+	+	+	+
245	271	Alanyl-tRNA synthetase (EC 6.1.1.7)	57	+	+	+	+	+	+	+	+	+	+	+	+	+	+	+	+	+	+	+	+	+	+	+	+	+	+	+	+	+	+	+	+	+	+	+	+	+	+	+	+	+	+	+	+	+	+	+	+	+	+	+	+	+
246	272	Cysteine desulfurase (EC 2.8.1.7), SufS subfamily	57	+	+	+	+	+	+	+	+	+	+	+	+	+	+	+	+	+	+	+	+	+	+	+	+	+	+	+	+	+	+	+	+	+	+	+	+	+	+	+	+	+	+	+	+	+	+	+	+	+	+	+	+	+
247	273	ATP-dependent DNA helicase RecG (EC 3.6.1.-)	57	+	+	+	+	+	+	+	+	+	+	+	+	+	+	+	+	+	+	+	+	+	+	+	+	+	+	+	+	+	+	+	+	+	+	+	+	+	+	+	+	+	+	+	+	+	+	+	+	+	+	+	+	+
248	274	LSU ribosomal protein L10p (P0)	57	+	+	+	+	+	+	+	+	+	+	+	+	+	+	+	+	+	+	+	+	+	+	+	+	+	+	+	+	+	+	+	+	+	+	+	+	+	+	+	+	+	+	+	+	+	+	+	+	+	+	+	+	+
249	275	Cysteine and methionine metabolism regulator CmbR, LysR family	57	+	+	+	+	+	+	+	+	+	+	+	+	+	+	+	+	+	+	+	+	+	+	+	+	+	+	+	+	+	+	+	+	+	+	+	+	+	+	+	+	+	+	+	+	+	+	+	+	+	+	+	+	+
250	276	Transcriptional regulatory protein	57	+	+	+	+	+	+	+	+	+	+	+	+	+	+	+	+	+	+	+	+	+	+	+	+	+	+	+	+	+	+	+	+	+	+	+	+	+	+	+	+	+	+	+	+	+	+	+	+	+	+	+	+	+
251	277	Ribosomal large subunit pseudouridine synthase B (EC 4.2.1.70)	57	+	+	+	+	+	+	+	+	+	+	+	+	+	+	+	+	+	+	+	+	+	+	+	+	+	+	+	+	+	+	+	+	+	+	+	+	+	+	+	+	+	+	+	+	+	+	+	+	+	+	+	+	+
252	278	Phosphoglucomutase (EC 5.4.2.2)	57	+	+	+	+	+	+	+	+	+	+	+	+	+	+	+	+	+	+	+	+	+	+	+	+	+	+	+	+	+	+	+	+	+	+	+	+	+	+	+	+	+	+	+	+	+	+	+	+	+	+	+	+	+
253	279	Mannose-6-phosphate isomerase (EC 5.3.1.8)	57	+	+	+	+	+	+	+	+	+	+	+	+	+	+	+	+	+	+	+	+	+	+	+	+	+	+	+	+	+	+	+	+	+	+	+	+	+	+	+	+	+	+	+	+	+	+	+	+	+	+	+	+	+
254	280	Histidyl-tRNA synthetase (EC 6.1.1.21)	57	+	+	+	+	+	+	+	+	+	+	+	+	+	+	+	+	+	+	+	+	+	+	+	+	+	+	+	+	+	+	+	+	+	+	+	+	+	+	+	+	+	+	+	+	+	+	+	+	+	+	+	+	+
255	281	(3R)-hydroxymyristoyl-[acyl carrier protein] dehydratase (EC 4.2.1.-)	57	+	+	+	+	+	+	+	+	+	+	+	+	+	+	+	+	+	+	+	+	+	+	+	+	+	+	+	+	+	+	+	+	+	+	+	+	+	+	+	+	+	+	+	+	+	+	+	+	+	+	+	+	+
256	282	S-adenosylmethionine:tRNA ribosyltransferase-isomerase (EC 5.-.-.-)	57	+	+	+	+	+	+	+	+	+	+	+	+	+	+	+	+	+	+	+	+	+	+	+	+	+	+	+	+	+	+	+	+	+	+	+	+	+	+	+	+	+	+	+	+	+	+	+	+	+	+	+	+	+
257	283	SSU ribosomal protein S19p (S15e)	57	+	+	+	+	+	+	+	+	+	+	+	+	+	+	+	+	+	+	+	+	+	+	+	+	+	+	+	+	+	+	+	+	+	+	+	+	+	+	+	+	+	+	+	+	+	+	+	+	+	+	+	+	+
258	284	FIG000325: clustered with transcription termination protein NusA	57	+	+	+	+	+	+	+	+	+	+	+	+	+	+	+	+	+	+	+	+	+	+	+	+	+	+	+	+	+	+	+	+	+	+	+	+	+	+	+	+	+	+	+	+	+	+	+	+	+	+	+	+	+
259	285	ATP-dependent Clp protease ATP-binding subunit ClpA	57	+	+	+	+	+	+	+	+	+	+	+	+	+	+	+	+	+	+	+	+	+	+	+	+	+	+	+	+	+	+	+	+	+	+	+	+	+	+	+	+	+	+	+	+	+	+	+	+	+	+	+	+	+
260	286	Thymidine kinase (EC 2.7.1.21)	57	+	+	+	+	+	+	+	+	+	+	+	+	+	+	+	+	+	+	+	+	+	+	+	+	+	+	+	+	+	+	+	+	+	+	+	+	+	+	+	+	+	+	+	+	+	+	+	+	+	+	+	+	+
261	287	Sucrose-6-phosphate hydrolase (EC 3.2.1.26)	57	+	+	+	+	+	+	+	+	+	+	+	+	+	+	+	+	+	+	+	+	+	+	+	+	+	+	+	+	+	+	+	+	+	+	+	+	+	+	+	+	+	+	+	+	+	+	+	+	+	+	+	+	+
262	288	Redox-sensitive transcriptional regulator (AT-rich DNA-binding protein)	57	+	+	+	+	+	+	+	+	+	+	+	+	+	+	+	+	+	+	+	+	+	+	+	+	+	+	+	+	+	+	+	+	+	+	+	+	+	+	+	+	+	+	+	+	+	+	+	+	+	+	+	+	+
263	289	Pyruvate kinase (EC 2.7.1.40)	57	+	+	+	+	+	+	+	+	+	+	+	+	+	+	+	+	+	+	+	+	+	+	+	+	+	+	+	+	+	+	+	+	+	+	+	+	+	+	+	+	+	+	+	+	+	+	+	+	+	+	+	+	+
264	290	Transcription termination protein NusB	57	+	+	+	+	+	+	+	+	+	+	+	+	+	+	+	+	+	+	+	+	+	+	+	+	+	+	+	+	+	+	+	+	+	+	+	+	+	+	+	+	+	+	+	+	+	+	+	+	+	+	+	+	+
265	291	PurR: transcription regulator associated with purine metabolism	57	+	+	+	+	+	+	+	+	+	+	+	+	+	+	+	+	+	+	+	+	+	+	+	+	+	+	+	+	+	+	+	+	+	+	+	+	+	+	+	+	+	+	+	+	+	+	+	+	+	+	+	+	+
266	292	Cell division protein GpsB, coordinates the switch between cylindrical and septal cell wall synthesis by re-localization of PBP1	57	+	+	+	+	+	+	+	+	+	+	+	+	+	+	+	+	+	+	+	+	+	+	+	+	+	+	+	+	+	+	+	+	+	+	+	+	+	+	+	+	+	+	+	+	+	+	+	+	+	+	+	+	+
267	293	Potassium efflux system KefA protein / Small-conductance mechanosensitive channel	57	+	+	+	+	+	+	+	+	+	+	+	+	+	+	+	+	+	+	+	+	+	+	+	+	+	+	+	+	+	+	+	+	+	+	+	+	+	+	+	+	+	+	+	+	+	+	+	+	+	+	+	+	+
268	294	Hypothetical protein SPy1011	57	+	+	+	+	+	+	+	+	+	+	+	+	+	+	+	+	+	+	+	+	+	+	+	+	+	+	+	+	+	+	+	+	+	+	+	+	+	+	+	+	+	+	+	+	+	+	+	+	+	+	+	+	+
269	295	COG0488: ATPase components of ABC transporters with duplicated ATPase domains	57	+	+	+	+	+	+	+	+	+	+	+	+	+	+	+	+	+	+	+	+	+	+	+	+	+	+	+	+	+	+	+	+	+	+	+	+	+	+	+	+	+	+	+	+	+	+	+	+	+	+	+	+	+
270	296	Methylenetetrahydrofolate dehydrogenase (NADP+) (EC 1.5.1.5) / Methenyltetrahydrofolate cyclohydrolase (EC 3.5.4.9)	57	+	+	+	+	+	+	+	+	+	+	+	+	+	+	+	+	+	+	+	+	+	+	+	+	+	+	+	+	+	+	+	+	+	+	+	+	+	+	+	+	+	+	+	+	+	+	+	+	+	+	+	+	+
271	297	RecA protein	57	+	+	+	+	+	+	+	+	+	+	+	+	+	+	+	+	+	+	+	+	+	+	+	+	+	+	+	+	+	+	+	+	+	+	+	+	+	+	+	+	+	+	+	+	+	+	+	+	+	+	+	+	+
272	298	Cell division initiation protein DivIVA	57	+	+	+	+	+	+	+	+	+	+	+	+	+	+	+	+	+	+	+	+	+	+	+	+	+	+	+	+	+	+	+	+	+	+	+	+	+	+	+	+	+	+	+	+	+	+	+	+	+	+	+	+	+
273	299	Nicotinate-nucleotide adenylyltransferase (EC 2.7.7.18)	57	+	+	+	+	+	+	+	+	+	+	+	+	+	+	+	+	+	+	+	+	+	+	+	+	+	+	+	+	+	+	+	+	+	+	+	+	+	+	+	+	+	+	+	+	+	+	+	+	+	+	+	+	+
274	300	Cell division protein FtsX	57	+	+	+	+	+	+	+	+	+	+	+	+	+	+	+	+	+	+	+	+	+	+	+	+	+	+	+	+	+	+	+	+	+	+	+	+	+	+	+	+	+	+	+	+	+	+	+	+	+	+	+	+	+
275	301	Phosphoenolpyruvate-protein phosphotransferase of PTS system (EC 2.7.3.9)	57	+	+	+	+	+	+	+	+	+	+	+	+	+	+	+	+	+	+	+	+	+	+	+	+	+	+	+	+	+	+	+	+	+	+	+	+	+	+	+	+	+	+	+	+	+	+	+	+	+	+	+	+	+
276	302	Octaprenyl diphosphate synthase (EC 2.5.1.90) / Dimethylallyltransferase (EC 2.5.1.1) / (2E,6E)-farnesyl diphosphate synthase (EC 2.5.1.10) / Geranylgeranyl diphosphate synthase (EC 2.5.1.29)	57	+	+	+	+	+	+	+	+	+	+	+	+	+	+	+	+	+	+	+	+	+	+	+	+	+	+	+	+	+	+	+	+	+	+	+	+	+	+	+	+	+	+	+	+	+	+	+	+	+	+	+	+	+	+	+
277	303	UDP-N-acetylglucosamine--N-acetylmuramyl-(pentapeptide) pyrophosphoryl-undecaprenol N-acetylglucosamine transferase (EC 2.4.1.227)	57	+	+	+	+	+	+	+	+	+	+	+	+	+	+	+	+	+	+	+	+	+	+	+	+	+	+	+	+	+	+	+	+	+	+	+	+	+	+	+	+	+	+	+	+	+	+	+	+	+	+	+	+	+
278	304	Preprotein translocase subunit SecY	57	+	+	+	+	+	+	+	+	+	+	+	+	+	+	+	+	+	+	+	+	+	+	+	+	+	+	+	+	+	+	+	+	+	+	+	+	+	+	+	+	+	+	+	+	+	+	+	+	+	+	+	+	+
279	305	Phosphate transport system permease protein PstA (TC 3.A.1.7.1)	57	+	+	+	+	+	+	+	+	+	+	+	+	+	+	+	+	+	+	+	+	+	+	+	+	+	+	+	+	+	+	+	+	+	+	+	+	+	+	+	+	+	+	+	+	+	+	+	+	+	+	+	+	+
280	306	proposed amino acid ligase found clustered with an amidotransferase	57	+	+	+	+	+	+	+	+	+	+	+	+	+	+	+	+	+	+	+	+	+	+	+	+	+	+	+	+	+	+	+	+	+	+	+	+	+	+	+	+	+	+	+	+	+	+	+	+	+	+	+	+	+
281	307	LSU m5C1962 methyltransferase RlmI	57	+	+	+	+	+	+	+	+	+	+	+	+	+	+	+	+	+	+	+	+	+	+	+	+	+	+	+	+	+	+	+	+	+	+	+	+	+	+	+	+	+	+	+	+	+	+	+	+	+	+	+	+	+
282	308	NADH peroxidase (EC 1.11.1.1)	57	+	+	+	+	+	+	+	+	+	+	+	+	+	+	+	+	+	+	+	+	+	+	+	+	+	+	+	+	+	+	+	+	+	+	+	+	+	+	+	+	+	+	+	+	+	+	+	+	+	+	+	+	+
283	309	LSU ribosomal protein L29p (L35e)	57	+	+	+	+	+	+	+	+	+	+	+	+	+	+	+	+	+	+	+	+	+	+	+	+	+	+	+	+	+	+	+	+	+	+	+	+	+	+	+	+	+	+	+	+	+	+	+	+	+	+	+	+	+

Table S5. Precence/absence of each HG among 57 *S. suis* strains

				1																13			25					28						104		94			76			53/54		Divergent *S. suis*																
Index[a]	ClutserID	Gene products	Members	98HAH33	05ZYH33	SC84	A7	SS12	P1/7	DAT273	NIAH11434	JS14	GZ1	BM407	S735	13730	MNCM07	NCTC 10446	14636	NCTC 10237	ST1	2651	89-1591	MNCM04	MNCM25	8074	D9	4961	ST3	MNCM43	DAT292	DAT260	NIAH11435	MNCM21	MNCM50	89-2479	DAT234	DAT290	93A	42A	NT77	6407	11538	8830	12814	92-1400	89-590	2726	2524	89-3576-3	4417	D12	10581	89-5259	88-5299A	R61	92-1191	14A	92-4172	22083
284	310	Seryl-tRNA synthetase (EC 6.1.1.11)	57	+	+	+	+	+	+	+	+	+	+	+	+	+	+	+	+	+	+	+	+	+	+	+	+	+	+	+	+	+	+	+	+	+	+	+	+	+	+	+	+	+	+	+	+	+	+	+	+	+	+	+	+	+	+	+	+	+
285	311	SSU ribosomal protein S8p (S15Ae)	57	+	+	+	+	+	+	+	+	+	+	+	+	+	+	+	+	+	+	+	+	+	+	+	+	+	+	+	+	+	+	+	+	+	+	+	+	+	+	+	+	+	+	+	+	+	+	+	+	+	+	+	+	+	+	+	+	+
286	312	Heat-inducible transcription repressor HrcA	57	+	+	+	+	+	+	+	+	+	+	+	+	+	+	+	+	+	+	+	+	+	+	+	+	+	+	+	+	+	+	+	+	+	+	+	+	+	+	+	+	+	+	+	+	+	+	+	+	+	+	+	+	+	+	+	+	+
287	313	Peptide chain release factor 2; programmed frameshift-containing	57	+	+	+	+	+	+	+	+	+	+	+	+	+	+	+	+	+	+	+	+	+	+	+	+	+	+	+	+	+	+	+	+	+	+	+	+	+	+	+	+	+	+	+	+	+	+	+	+	+	+	+	+	+	+	+	+	+
288	315	unknown domain / Nucleoside 5-triphosphatase RdgB (dHAPTP, dITP, XTP-specific) (EC 3.6.1.15)	57	+	+	+	+	+	+	+	+	+	+	+	+	+	+	+	+	+	+	+	+	+	+	+	+	+	+	+	+	+	+	+	+	+	+	+	+	+	+	+	+	+	+	+	+	+	+	+	+	+	+	+	+	+	+	+	+	+
289	316	LSU ribosomal protein L6p (L9e)	57	+	+	+	+	+	+	+	+	+	+	+	+	+	+	+	+	+	+	+	+	+	+	+	+	+	+	+	+	+	+	+	+	+	+	+	+	+	+	+	+	+	+	+	+	+	+	+	+	+	+	+	+	+	+	+	+	+
290	317	Dihydroxyacetone kinase family protein	57	+	+	+	+	+	+	+	+	+	+	+	+	+	+	+	+	+	+	+	+	+	+	+	+	+	+	+	+	+	+	+	+	+	+	+	+	+	+	+	+	+	+	+	+	+	+	+	+	+	+	+	+	+	+	+	+	+
291	318	Asparaginyl-tRNA synthetase (EC 6.1.1.22)	57	+	+	+	+	+	+	+	+	+	+	+	+	+	+	+	+	+	+	+	+	+	+	+	+	+	+	+	+	+	+	+	+	+	+	+	+	+	+	+	+	+	+	+	+	+	+	+	+	+	+	+	+	+	+	+	+	+
292	319	Hydroxymethylglutaryl-CoA synthase (EC 2.3.3.10)	57	+	+	+	+	+	+	+	+	+	+	+	+	+	+	+	+	+	+	+	+	+	+	+	+	+	+	+	+	+	+	+	+	+	+	+	+	+	+	+	+	+	+	+	+	+	+	+	+	+	+	+	+	+	+	+	+	+
293	320	Serine protease, DegP/HtrA, do-like (EC 3.4.21.-)	57	+	+	+	+	+	+	+	+	+	+	+	+	+	+	+	+	+	+	+	+	+	+	+	+	+	+	+	+	+	+	+	+	+	+	+	+	+	+	+	+	+	+	+	+	+	+	+	+	+	+	+	+	+	+	+	+	+
294	321	O-methyltransferase family protein [C1]	57	+	+	+	+	+	+	+	+	+	+	+	+	+	+	+	+	+	+	+	+	+	+	+	+	+	+	+	+	+	+	+	+	+	+	+	+	+	+	+	+	+	+	+	+	+	+	+	+	+	+	+	+	+	+	+	+	+
295	322	Nicotinate phosphoribosyltransferase (EC 2.4.2.11)	57	+	+	+	+	+	+	+	+	+	+	+	+	+	+	+	+	+	+	+	+	+	+	+	+	+	+	+	+	+	+	+	+	+	+	+	+	+	+	+	+	+	+	+	+	+	+	+	+	+	+	+	+	+	+	+	+	+
296	323	Zinc ABC transporter, inner membrane permease protein ZnuB	57	+	+	+	+	+	+	+	+	+	+	+	+	+	+	+	+	+	+	+	+	+	+	+	+	+	+	+	+	+	+	+	+	+	+	+	+	+	+	+	+	+	+	+	+	+	+	+	+	+	+	+	+	+	+	+	+	+
297	324	Xaa-Pro dipeptidyl-peptidase (EC 3.4.14.11)	57	+	+	+	+	+	+	+	+	+	+	+	+	+	+	+	+	+	+	+	+	+	+	+	+	+	+	+	+	+	+	+	+	+	+	+	+	+	+	+	+	+	+	+	+	+	+	+	+	+	+	+	+	+	+	+	+	+
298	325	ADP-ribose pyrophosphatase (EC 3.6.1.13)	57	+	+	+	+	+	+	+	+	+	+	+	+	+	+	+	+	+	+	+	+	+	+	+	+	+	+	+	+	+	+	+	+	+	+	+	+	+	+	+	+	+	+	+	+	+	+	+	+	+	+	+	+	+	+	+	+	+
299	326	tRNA nucleotidyltransferase (EC 2.7.7.21) (EC 2.7.7.25)	57	+	+	+	+	+	+	+	+	+	+	+	+	+	+	+	+	+	+	+	+	+	+	+	+	+	+	+	+	+	+	+	+	+	+	+	+	+	+	+	+	+	+	+	+	+	+	+	+	+	+	+	+	+	+	+	+	+
300	327	Acyl-phosphate:glycerol-3-phosphate O-acyltransferase PlsY	57	+	+	+	+	+	+	+	+	+	+	+	+	+	+	+	+	+	+	+	+	+	+	+	+	+	+	+	+	+	+	+	+	+	+	+	+	+	+	+	+	+	+	+	+	+	+	+	+	+	+	+	+	+	+	+	+	+
301	328	Septation ring formation regulator EzrA	57	+	+	+	+	+	+	+	+	+	+	+	+	+	+	+	+	+	+	+	+	+	+	+	+	+	+	+	+	+	+	+	+	+	+	+	+	+	+	+	+	+	+	+	+	+	+	+	+	+	+	+	+	+	+	+	+	+
302	329	General stress protein	57	+	+	+	+	+	+	+	+	+	+	+	+	+	+	+	+	+	+	+	+	+	+	+	+	+	+	+	+	+	+	+	+	+	+	+	+	+	+	+	+	+	+	+	+	+	+	+	+	+	+	+	+	+	+	+	+	+
303	330	Exodeoxyribonuclease VII small subunit (EC 3.1.11.6)	57	+	+	+	+	+	+	+	+	+	+	+	+	+	+	+	+	+	+	+	+	+	+	+	+	+	+	+	+	+	+	+	+	+	+	+	+	+	+	+	+	+	+	+	+	+	+	+	+	+	+	+	+	+	+	+	+	+
304	331	MORN motif family protein	57	+	+	+	+	+	+	+	+	+	+	+	+	+	+	+	+	+	+	+	+	+	+	+	+	+	+	+	+	+	+	+	+	+	+	+	+	+	+	+	+	+	+	+	+	+	+	+	+	+	+	+	+	+	+	+	+	+
305	332	GTP-binding protein YqeH, required for biogenesis of 30S ribosome subunit	57	+	+	+	+	+	+	+	+	+	+	+	+	+	+	+	+	+	+	+	+	+	+	+	+	+	+	+	+	+	+	+	+	+	+	+	+	+	+	+	+	+	+	+	+	+	+	+	+	+	+	+	+	+	+	+	+	+
306	333	Recombination protein RecR	57	+	+	+	+	+	+	+	+	+	+	+	+	+	+	+	+	+	+	+	+	+	+	+	+	+	+	+	+	+	+	+	+	+	+	+	+	+	+	+	+	+	+	+	+	+	+	+	+	+	+	+	+	+	+	+	+	+
307	334	Cell envelope-associated transcriptional attenuator LytR-CpsA-Psr, subfamily F1 (as in PMID19099556)	57	+	+	+	+	+	+	+	+	+	+	+	+	+	+	+	+	+	+	+	+	+	+	+	+	+	+	+	+	+	+	+	+	+	+	+	+	+	+	+	+	+	+	+	+	+	+	+	+	+	+	+	+	+	+	+	+	+
308	335	ATP synthase A chain (EC 3.6.3.14)	57	+	+	+	+	+	+	+	+	+	+	+	+	+	+	+	+	+	+	+	+	+	+	+	+	+	+	+	+	+	+	+	+	+	+	+	+	+	+	+	+	+	+	+	+	+	+	+	+	+	+	+	+	+	+	+	+	+
309	336	LSU ribosomal protein L18p (L5e)	57	+	+	+	+	+	+	+	+	+	+	+	+	+	+	+	+	+	+	+	+	+	+	+	+	+	+	+	+	+	+	+	+	+	+	+	+	+	+	+	+	+	+	+	+	+	+	+	+	+	+	+	+	+	+	+	+	+
310	337	Two component system response regulator CiaR	57	+	+	+	+	+	+	+	+	+	+	+	+	+	+	+	+	+	+	+	+	+	+	+	+	+	+	+	+	+	+	+	+	+	+	+	+	+	+	+	+	+	+	+	+	+	+	+	+	+	+	+	+	+	+	+	+	+
311	338	DinG family ATP-dependent helicase YoaA	57	+	+	+	+	+	+	+	+	+	+	+	+	+	+	+	+	+	+	+	+	+	+	+	+	+	+	+	+	+	+	+	+	+	+	+	+	+	+	+	+	+	+	+	+	+	+	+	+	+	+	+	+	+	+	+	+	+
312	339	S-adenosylmethionine synthetase (EC 2.5.1.6)	57	+	+	+	+	+	+	+	+	+	+	+	+	+	+	+	+	+	+	+	+	+	+	+	+	+	+	+	+	+	+	+	+	+	+	+	+	+	+	+	+	+	+	+	+	+	+	+	+	+	+	+	+	+	+	+	+	+
313	340	RecU Holliday junction resolvase	57	+	+	+	+	+	+	+	+	+	+	+	+	+	+	+	+	+	+	+	+	+	+	+	+	+	+	+	+	+	+	+	+	+	+	+	+	+	+	+	+	+	+	+	+	+	+	+	+	+	+	+	+	+	+	+	+	+
314	341	tRNA pseudouridine synthase A (EC 4.2.1.70)	57	+	+	+	+	+	+	+	+	+	+	+	+	+	+	+	+	+	+	+	+	+	+	+	+	+	+	+	+	+	+	+	+	+	+	+	+	+	+	+	+	+	+	+	+	+	+	+	+	+	+	+	+	+	+	+	+	+
315	342	Biotin carboxyl carrier protein of acetyl-CoA carboxylase	57	+	+	+	+	+	+	+	+	+	+	+	+	+	+	+	+	+	+	+	+	+	+	+	+	+	+	+	+	+	+	+	+	+	+	+	+	+	+	+	+	+	+	+	+	+	+	+	+	+	+	+	+	+	+	+	+	+
316	343	Uridine kinase (EC 2.7.1.48) [C1]	57	+	+	+	+	+	+	+	+	+	+	+	+	+	+	+	+	+	+	+	+	+	+	+	+	+	+	+	+	+	+	+	+	+	+	+	+	+	+	+	+	+	+	+	+	+	+	+	+	+	+	+	+	+	+	+	+	+
317	344	Ribonucleotide reductase of class III (anaerobic), large subunit (EC 1.17.4.2)	57	+	+	+	+	+	+	+	+	+	+	+	+	+	+	+	+	+	+	+	+	+	+	+	+	+	+	+	+	+	+	+	+	+	+	+	+	+	+	+	+	+	+	+	+	+	+	+	+	+	+	+	+	+	+	+	+	+
318	345	ABC transporter, substrate-binding protein	57	+	+	+	+	+	+	+	+	+	+	+	+	+	+	+	+	+	+	+	+	+	+	+	+	+	+	+	+	+	+	+	+	+	+	+	+	+	+	+	+	+	+	+	+	+	+	+	+	+	+	+	+	+	+	+	+	+
319	346	Transcriptional regulator	57	+	+	+	+	+	+	+	+	+	+	+	+	+	+	+	+	+	+	+	+	+	+	+	+	+	+	+	+	+	+	+	+	+	+	+	+	+	+	+	+	+	+	+	+	+	+	+	+	+	+	+	+	+	+	+	+	+
320	347	Neutral endopeptidase O (EC 3.4.24.-)	57	+	+	+	+	+	+	+	+	+	+	+	+	+	+	+	+	+	+	+	+	+	+	+	+	+	+	+	+	+	+	+	+	+	+	+	+	+	+	+	+	+	+	+	+	+	+	+	+	+	+	+	+	+	+	+	+	+
321	348	LSU ribosomal protein L14p (L23e)	57	+	+	+	+	+	+	+	+	+	+	+	+	+	+	+	+	+	+	+	+	+	+	+	+	+	+	+	+	+	+	+	+	+	+	+	+	+	+	+	+	+	+	+	+	+	+	+	+	+	+	+	+	+	+	+	+	+
322	349	Sucrose operon repressor ScrR, LacI family	57	+	+	+	+	+	+	+	+	+	+	+	+	+	+	+	+	+	+	+	+	+	+	+	+	+	+	+	+	+	+	+	+	+	+	+	+	+	+	+	+	+	+	+	+	+	+	+	+	+	+	+	+	+	+	+	+	+
323	350	Guanine-hypoxanthine permease	57	+	+	+	+	+	+	+	+	+	+	+	+	+	+	+	+	+	+	+	+	+	+	+	+	+	+	+	+	+	+	+	+	+	+	+	+	+	+	+	+	+	+	+	+	+	+	+	+	+	+	+	+	+	+	+	+	+
324	351	FMN adenylyltransferase (EC 2.7.7.2)	57	+	+	+	+	+	+	+	+	+	+	+	+	+	+	+	+	+	+	+	+	+	+	+	+	+	+	+	+	+	+	+	+	+	+	+	+	+	+	+	+	+	+	+	+	+	+	+	+	+	+	+	+	+	+	+	+	+
325	352	DNA mismatch repair protein MutS	57	+	+	+	+	+	+	+	+	+	+	+	+	+	+	+	+	+	+	+	+	+	+	+	+	+	+	+	+	+	+	+	+	+	+	+	+	+	+	+	+	+	+	+	+	+	+	+	+	+	+	+	+	+	+	+	+	+
326	353	Hydrolase (HAD superfamily)	57	+	+	+	+	+	+	+	+	+	+	+	+	+	+	+	+	+	+	+	+	+	+	+	+	+	+	+	+	+	+	+	+	+	+	+	+	+	+	+	+	+	+	+	+	+	+	+	+	+	+	+	+	+	+	+	+	+
327	354	Transcriptional regulator OrfX	57	+	+	+	+	+	+	+	+	+	+	+	+	+	+	+	+	+	+	+	+	+	+	+	+	+	+	+	+	+	+	+	+	+	+	+	+	+	+	+	+	+	+	+	+	+	+	+	+	+	+	+	+	+	+	+	+	+
328	355	RNA polymerase sigma factor RpoD	57	+	+	+	+	+	+	+	+	+	+	+	+	+	+	+	+	+	+	+	+	+	+	+	+	+	+	+	+	+	+	+	+	+	+	+	+	+	+	+	+	+	+	+	+	+	+	+	+	+	+	+	+	+	+	+	+	+
329	356	ATP-dependent Clp protease ATP-binding subunit ClpX	57	+	+	+	+	+	+	+	+	+	+	+	+	+	+	+	+	+	+	+	+	+	+	+	+	+	+	+	+	+	+	+	+	+	+	+	+	+	+	+	+	+	+	+	+	+	+	+	+	+	+	+	+	+	+	+	+	+
330	357	Translation initiation factor 2	57	+	+	+	+	+	+	+	+	+	+	+	+	+	+	+	+	+	+	+	+	+	+	+	+	+	+	+	+	+	+	+	+	+	+	+	+	+	+	+	+	+	+	+	+	+	+	+	+	+	+	+	+	+	+	+	+	+
331	358	Isoleucyl-tRNA synthetase (EC 6.1.1.5)	57	+	+	+	+	+	+	+	+	+	+	+	+	+	+	+	+	+	+	+	+	+	+	+	+	+	+	+	+	+	+	+	+	+	+	+	+	+	+	+	+	+	+	+	+	+	+	+	+	+	+	+	+	+	+	+	+	+
332	359	A/G-specific adenine glycosylase (EC 3.2.2.-)	57	+	+	+	+	+	+	+	+	+	+	+	+	+	+	+	+	+	+	+	+	+	+	+	+	+	+	+	+	+	+	+	+	+	+	+	+	+	+	+	+	+	+	+	+	+	+	+	+	+	+	+	+	+	+	+	+	+
333	360	Segregation and condensation protein B	57	+	+	+	+	+	+	+	+	+	+	+	+	+	+	+	+	+	+	+	+	+	+	+	+	+	+	+	+	+	+	+	+	+	+	+	+	+	+	+	+	+	+	+	+	+	+	+	+	+	+	+	+	+	+	+	+	+
334	361	Aspartate aminotransferase (EC 2.6.1.1)	57	+	+	+	+	+	+	+	+	+	+	+	+	+	+	+	+	+	+	+	+	+	+	+	+	+	+	+	+	+	+	+	+	+	+	+	+	+	+	+	+	+	+	+	+	+	+	+	+	+	+	+	+	+	+	+	+	+
335	362	DNA topoisomerase I (EC 5.99.1.2)	57	+	+	+	+	+	+	+	+	+	+	+	+	+	+	+	+	+	+	+	+	+	+	+	+	+	+	+	+	+	+	+	+	+	+	+	+	+	+	+	+	+	+	+	+	+	+	+	+	+	+	+	+	+	+	+	+	+
336	363	GTP-sensing transcriptional pleiotropic repressor codY	57	+	+	+	+	+	+	+	+	+	+	+	+	+	+	+	+	+	+	+	+	+	+	+	+	+	+	+	+	+	+	+	+	+	+	+	+	+	+	+	+	+	+	+	+	+	+	+	+	+	+	+	+	+	+	+	+	+
337	364	Molybdopterin binding motif, CinA N-terminal domain / C-terminal domain of CinA type S	57	+	+	+	+	+	+	+	+	+	+	+	+	+	+	+	+	+	+	+	+	+	+	+	+	+	+	+	+	+	+	+	+	+	+	+	+	+	+	+	+	+	+	+	+	+	+	+	+	+	+	+	+	+	+	+	+	+
338	365	Two component system sensor histidine kinase CiaH (EC 2.7.3.-)	57	+	+	+	+	+	+	+	+	+	+	+	+	+	+	+	+	+	+	+	+	+	+	+	+	+	+	+	+	+	+	+	+	+	+	+	+	+	+	+	+	+	+	+	+	+	+	+	+	+	+	+	+	+	+	+	+	+
339	366	Single-stranded-DNA-specific exonuclease RecJ (EC 3.1.-.-)	57	+	+	+	+	+	+	+	+	+	+	+	+	+	+	+	+	+	+	+	+	+	+	+	+	+	+	+	+	+	+	+	+	+	+	+	+	+	+	+	+	+	+	+	+	+	+	+	+	+	+	+	+	+	+	+	+	+
340	367	Glutathione reductase (EC 1.8.1.7)	57	+	+	+	+	+	+	+	+	+	+	+	+	+	+	+	+	+	+	+	+	+	+	+	+	+	+	+	+	+	+	+	+	+	+	+	+	+	+	+	+	+	+	+	+	+	+	+	+	+	+	+	+	+	+	+	+	+
341	368	rRNA small subunit methyltransferase I	57	+	+	+	+	+	+	+	+	+	+	+	+	+	+	+	+	+	+	+	+	+	+	+	+	+	+	+	+	+	+	+	+	+	+	+	+	+	+	+	+	+	+	+	+	+	+	+	+	+	+	+	+	+	+	+	+	+
342	369	ATP-dependent Clp protease proteolytic subunit (EC 3.4.21.92)	57	+	+	+	+	+	+	+	+	+	+	+	+	+	+	+	+	+	+	+	+	+	+	+	+	+	+	+	+	+	+	+	+	+	+	+	+	+	+	+	+	+	+	+	+	+	+	+	+	+	+	+	+	+	+	+	+	+
343	370	Adenine-specific methyltransferase (EC 2.1.1.72)	57	+	+	+	+	+	+	+	+	+	+	+	+	+	+	+	+	+	+	+	+	+	+	+	+	+	+	+	+	+	+	+	+	+	+	+	+	+	+	+	+	+	+	+	+	+	+	+	+	+	+	+	+	+	+	+	+	+
344	371	Ribonuclease J1 (endonuclease and 5' exonuclease)	57	+	+	+	+	+	+	+	+	+	+	+	+	+	+	+	+	+	+	+	+	+	+	+	+	+	+	+	+	+	+	+	+	+	+	+	+	+	+	+	+	+	+	+	+	+	+	+	+	+	+	+	+	+	+	+	+	+
345	372	LSU ribosomal protein L30p (L7e)	57	+	+	+	+	+	+	+	+	+	+	+	+	+	+	+	+	+	+	+	+	+	+	+	+	+	+	+	+	+	+	+	+	+	+	+	+	+	+	+	+	+	+	+	+	+	+	+	+	+	+	+	+	+	+	+	+	+
346	373	Transcriptional regulator of fatty acid biosynthesis FabT	57	+	+	+	+	+	+	+	+	+	+	+	+	+	+	+	+	+	+	+	+	+	+	+	+	+	+	+	+	+	+	+	+	+	+	+	+	+	+	+	+	+	+	+	+	+	+	+	+	+	+	+	+	+	+	+	+	+
347	374	Threonyl-tRNA synthetase (EC 6.1.1.3)	57	+	+	+	+	+	+	+	+	+	+	+	+	+	+	+	+	+	+	+	+	+	+	+	+	+	+	+	+	+	+	+	+	+	+	+	+	+	+	+	+	+	+	+	+	+	+	+	+	+	+	+	+	+	+	+	+	+
348	375	DegV family protein	57	+	+	+	+	+	+	+	+	+	+	+	+	+	+	+	+	+	+	+	+	+	+	+	+	+	+	+	+	+	+	+	+	+	+	+	+	+	+	+	+	+	+	+	+	+	+	+	+	+	+	+	+	+	+	+	+	+
349	376	DNA recombination protein RmuC	57	+	+	+	+	+	+	+	+	+	+	+	+	+	+	+	+	+	+	+	+	+	+	+	+	+	+	+	+	+	+	+	+	+	+	+	+	+	+	+	+	+	+	+	+	+	+	+	+	+	+	+	+	+	+	+	+	+
350	377	aminoglycoside phosphotransferase family protein	57	+	+	+	+	+	+	+	+	+	+	+	+	+	+	+	+	+	+	+	+	+	+	+	+	+	+	+	+	+	+	+	+	+	+	+	+	+	+	+	+	+	+	+	+	+	+	+	+	+	+	+	+	+	+	+	+	+
351	378	Hypothetical protein DUF194, DegV family	57	+	+	+	+	+	+	+	+	+	+	+	+	+	+	+	+	+	+	+	+	+	+	+	+	+	+	+	+	+	+	+	+	+	+	+	+	+	+	+	+	+	+	+	+	+	+	+	+	+	+	+	+	+	+	+	+	+
352	379	DNA polymerase III alpha subunit (EC 2.7.7.7)	57	+	+	+	+	+	+	+	+	+	+	+	+	+	+	+	+	+	+	+	+	+	+	+	+	+	+	+	+	+	+	+	+	+	+	+	+	+	+	+	+	+	+	+	+	+	+	+	+	+	+	+	+	+	+	+	+	+
353	380	Hypothetical protein YggS, proline synthase co-transcribed bacterial homolog PROSC	57	+	+	+	+	+	+	+	+	+	+	+	+	+	+	+	+	+	+	+	+	+	+	+	+	+	+	+	+	+	+	+	+	+	+	+	+	+	+	+	+	+	+	+	+	+	+	+	+	+	+	+	+	+	+	+	+	+
354	381	rRNA small subunit 7-methylguanosine (m7G) methyltransferase GidB	57	+	+	+	+	+	+	+	+	+	+	+	+	+	+	+	+	+	+	+	+	+	+	+	+	+	+	+	+	+	+	+	+	+	+	+	+	+	+	+	+	+	+	+	+	+	+	+	+	+	+	+	+	+	+	+	+	+
355	382	Phosphate acetyltransferase (EC 2.3.1.8)	57	+	+	+	+	+	+	+	+	+	+	+	+	+	+	+	+	+	+	+	+	+	+	+	+	+	+	+	+	+	+	+	+	+	+	+	+	+	+	+	+	+	+	+	+	+	+	+	+	+	+	+	+	+	+	+	+	+
356	383	PTS system, fructose-specific IIA component (EC 2.7.1.69) / PTS system, fructose-specific IIB component (EC 2.7.1.69) / PTS system, fructose-specific IIC component (EC 2.7.1.69)	57	+	+	+	+	+	+	+	+	+	+	+	+	+	+	+	+	+	+	+	+	+	+	+	+	+	+	+	+	+	+	+	+	+	+	+	+	+	+	+	+	+	+	+	+	+	+	+	+	+	+	+	+	+	+	+	+	+
357	384	Transcription termination protein NusA	57	+	+	+	+	+	+	+	+	+	+	+	+	+	+	+	+	+	+	+	+	+	+	+	+	+	+	+	+	+	+	+	+	+	+	+	+	+	+	+	+	+	+	+	+	+	+	+	+	+	+	+	+	+	+	+	+	+
358	385	LSU ribosomal protein L22p (L17e)	57	+	+	+	+	+	+	+	+	+	+	+	+	+	+	+	+	+	+	+	+	+	+	+	+	+	+	+	+	+	+	+	+	+	+	+	+	+	+	+	+	+	+	+	+	+	+	+	+	+	+	+	+	+	+	+	+	+
359	386	Transcription elongation factor GreA	57	+	+	+	+	+	+	+	+	+	+	+	+	+	+	+	+	+	+	+	+	+	+	+	+	+	+	+	+	+	+	+	+	+	+	+	+	+	+	+	+	+	+	+	+	+	+	+	+	+	+	+	+	+	+	+	+	+
360	387	Putative stomatin/prohibitin-family membrane protease subunit YbbK	57	+	+	+	+	+	+	+	+	+	+	+	+	+	+	+	+	+	+	+	+	+	+	+	+	+	+	+	+	+	+	+	+	+	+	+	+	+	+	+	+	+	+	+	+	+	+	+	+	+	+	+	+	+	+	+	+	+
361	388	UDP-N-acetylenolpyruvoylglucosamine reductase (EC 1.1.1.158)	57	+	+	+	+	+	+	+	+	+	+	+	+	+	+	+	+	+	+	+	+	+	+	+	+	+	+	+	+	+	+	+	+	+	+	+	+	+	+	+	+	+	+	+	+	+	+	+	+	+	+	+	+	+	+	+	+	+
362	389	Guanylate kinase (EC 2.7.4.8)	57	+	+	+	+	+	+	+	+	+	+	+	+	+	+	+	+	+	+	+	+	+	+	+	+	+	+	+	+	+	+	+	+	+	+	+	+	+	+	+	+	+	+	+	+	+	+	+	+	+	+	+	+	+	+	+	+	+
363	390	Phosphoesterase, DHH family protein	57	+	+	+	+	+	+	+	+	+	+	+	+	+	+	+	+	+	+	+	+	+	+	+	+	+	+	+	+	+	+	+	+	+	+	+	+	+	+	+	+	+	+	+	+	+	+	+	+	+	+	+	+	+	+	+	+	+
364	391	Uridylate kinase (EC 2.7.4.-)	57	+	+	+	+	+	+	+	+	+	+	+	+	+	+	+	+	+	+	+	+	+	+	+	+	+	+	+	+	+	+	+	+	+	+	+	+	+	+	+	+	+	+	+	+	+	+	+	+	+	+	+	+	+	+	+	+	+
365	392	Adenine phosphoribosyltransferase (EC 2.4.2.7)	57	+	+	+	+	+	+	+	+	+	+	+	+	+	+	+	+	+	+	+	+	+	+	+	+	+	+	+	+	+	+	+	+	+	+	+	+	+	+	+	+	+	+	+	+	+	+	+	+	+	+	+	+	+	+	+	+	+
366	393	Hypothetical NagD-like phosphatase	57	+	+	+	+	+	+	+	+	+	+	+	+	+	+	+	+	+	+	+	+	+	+	+	+	+	+	+	+	+	+	+	+	+	+	+	+	+	+	+	+	+	+	+	+	+	+	+	+	+	+	+	+	+	+	+	+	+
367	394	LSU ribosomal protein L5p (L11e)	57	+	+	+	+	+	+	+	+	+	+	+	+	+	+	+	+	+	+	+	+	+	+	+	+	+	+	+	+	+	+	+	+	+	+	+	+	+	+	+	+	+	+	+	+	+	+	+	+	+	+	+	+	+	+	+	+	+
368	395	Zinc ABC transporter, ATP-binding protein ZnuC	57	+	+	+	+	+	+	+	+	+	+	+	+	+	+	+	+	+	+	+	+	+	+	+	+	+	+	+	+	+	+	+	+	+	+	+	+	+	+	+	+	+	+	+	+	+	+	+	+	+	+	+	+	+	+	+	+	+
369	396	hypothetical protein	57	+	+	+	+	+	+	+	+	+	+	+	+	+	+	+	+	+	+	+	+	+	+	+	+	+	+	+	+	+	+	+	+	+	+	+	+	+	+	+	+	+	+	+	+	+	+	+	+	+	+	+	+	+	+	+	+	+
370	397	Cysteine desulfurase (EC 2.8.1.7)	57	+	+	+	+	+	+	+	+	+	+	+	+	+	+	+	+	+	+	+	+	+	+	+	+	+	+	+	+	+	+	+	+	+	+	+	+	+	+	+	+	+	+	+	+	+	+	+	+	+	+	+	+	+	+	+	+	+
371	399	Manganese-dependent inorganic pyrophosphatase (EC 3.6.1.1)	57	+	+	+	+	+	+	+	+	+	+	+	+	+	+	+	+	+	+	+	+	+	+	+	+	+	+	+	+	+	+	+	+	+	+	+	+	+	+	+	+	+	+	+	+	+	+	+	+	+	+	+	+	+	+	+	+	+
372	400	Translation initiation factor 1	57	+	+	+	+	+	+	+	+	+	+	+	+	+	+	+	+	+	+	+	+	+	+	+	+	+	+	+	+	+	+	+	+	+	+	+	+	+	+	+	+	+	+	+	+	+	+	+	+	+	+	+	+	+	+	+	+	+
373	401	Cell envelope-associated transcriptional attenuator LytR-CpsA-Psr, subfamily F2 (as in PMID19099556)	57	+	+	+	+	+	+	+	+	+	+	+	+	+	+	+	+	+	+	+	+	+	+	+	+	+	+	+	+	+	+	+	+	+	+	+	+	+	+	+	+	+	+	+	+	+	+	+	+	+	+	+	+	+	+	+	+	+
374	402	Methionyl-tRNA synthetase (EC 6.1.1.10)	57	+	+	+	+	+	+	+	+	+	+	+	+	+	+	+	+	+	+	+	+	+	+	+	+	+	+	+	+	+	+	+	+	+	+	+	+	+	+	+	+	+	+	+	+	+	+	+	+	+	+	+	+	+	+	+	+	+
375	403	hypothetical protein	57	+	+	+	+	+	+	+	+	+	+	+	+	+	+	+	+	+	+	+	+	+	+	+	+	+	+	+	+	+	+	+	+	+	+	+	+	+	+	+	+	+	+	+	+	+	+	+	+	+	+	+	+	+	+	+	+	+
376	404	Chromosome partition protein smc	57	+	+	+	+	+	+	+	+	+	+	+	+	+	+	+	+	+	+	+	+	+	+	+	+	+	+	+	+	+	+	+	+	+	+	+	+	+	+	+	+	+	+	+	+	+	+	+	+	+	+	+	+	+	+	+	+	+
377	405	Tyrosyl-tRNA synthetase (EC 6.1.1.1)	57	+	+	+	+	+	+	+	+	+	+	+	+	+	+	+	+	+	+	+	+	+	+	+	+	+	+	+	+	+	+	+	+	+	+	+	+	+	+	+	+	+	+	+	+	+	+	+	+	+	+	+	+	+	+	+	+	+
378	406	16S rRNA m(2)G 1207 methyltransferase (EC 2.1.1.52)	57	+	+	+	+	+	+	+	+	+	+	+	+	+	+	+	+	+	+	+	+	+	+	+	+	+	+	+	+	+	+	+	+	+	+	+	+	+	+	+	+	+	+	+	+	+	+	+	+	+	+	+	+	+	+	+	+	+
379	407	FIG000605: protein co-occurring with transport systems (COG1739)	57	+	+	+	+	+	+	+	+	+	+	+	+	+	+	+	+	+	+	+	+	+	+	+	+	+	+	+	+	+	+	+	+	+	+	+	+	+	+	+	+	+	+	+	+	+	+	+	+	+	+	+	+	+	+	+	+	+
380	408	Ribonuclease J2 (endoribonuclease in RNA processing)	57	+	+	+	+	+	+	+	+	+	+	+	+	+	+	+	+	+	+	+	+	+	+	+	+	+	+	+	+	+	+	+	+	+	+	+	+	+	+	+	+	+	+	+	+	+	+	+	+	+	+	+	+	+	+	+	+	+
381	409	Glutamine ABC transporter, glutamine-binding protein/permease protein	57	+	+	+	+	+	+	+	+	+	+	+	+	+	+	+	+	+	+	+	+	+	+	+	+	+	+	+	+	+	+	+	+	+	+	+	+	+	+	+	+	+	+	+	+	+	+	+	+	+	+	+	+	+	+	+	+	+
382	410	Phosphoenolpyruvate carboxylase (EC 4.1.1.31)	57	+	+	+	+	+	+	+	+	+	+	+	+	+	+	+	+	+	+	+	+	+	+	+	+	+	+	+	+	+	+	+	+	+	+	+	+	+	+	+	+	+	+	+	+	+	+	+	+	+	+	+	+	+	+	+	+	+
383	411	Biotin carboxylase of acetyl-CoA carboxylase (EC 6.3.4.14)	57	+	+	+	+	+	+	+	+	+	+	+	+	+	+	+	+	+	+	+	+	+	+	+	+	+	+	+	+	+	+	+	+	+	+	+	+	+	+	+	+	+	+	+	+	+	+	+	+	+	+	+	+	+	+	+	+	+
384	412	ATPase component of general energizing module of ECF transporters	57	+	+	+	+	+	+	+	+	+	+	+	+	+	+	+	+	+	+	+	+	+	+	+	+	+	+	+	+	+	+	+	+	+	+	+	+	+	+	+	+	+	+	+	+	+	+	+	+	+	+	+	+	+	+	+	+	+
385	413	Ribosome-binding factor A	57	+	+	+	+	+	+	+	+	+	+	+	+	+	+	+	+	+	+	+	+	+	+	+	+	+	+	+	+	+	+	+	+	+	+	+	+	+	+	+	+	+	+	+	+	+	+	+	+	+	+	+	+	+	+	+	+	+
386	415	Large-conductance mechanosensitive channel	57	+	+	+	+	+	+	+	+	+	+	+	+	+	+	+	+	+	+	+	+	+	+	+	+	+	+	+	+	+	+	+	+	+	+	+	+	+	+	+	+	+	+	+	+	+	+	+	+	+	+	+	+	+	+	+	+	+
387	416	Exodeoxyribonuclease III (EC 3.1.11.2)	57	+	+	+	+	+	+	+	+	+	+	+	+	+	+	+	+	+	+	+	+	+	+	+	+	+	+	+	+	+	+	+	+	+	+	+	+	+	+	+	+	+	+	+	+	+	+	+	+	+	+	+	+	+	+	+	+	+
388	417	Enolase (EC 4.2.1.11)	57	+	+	+	+	+	+	+	+	+	+	+	+	+	+	+	+	+	+	+	+	+	+	+	+	+	+	+	+	+	+	+	+	+	+	+	+	+	+	+	+	+	+	+	+	+	+	+	+	+	+	+	+	+	+	+	+	+
389	418	LSU ribosomal protein L1p (L10Ae)	57	+	+	+	+	+	+	+	+	+	+	+	+	+	+	+	+	+	+	+	+	+	+	+	+	+	+	+	+	+	+	+	+	+	+	+	+	+	+	+	+	+	+	+	+	+	+	+	+	+	+	+	+	+	+	+	+	+
390	419	ABC transporter, periplasmic spermidine putrescine-binding protein PotD (TC 3.A.1.11.1)	57	+	+	+	+	+	+	+	+	+	+	+	+	+	+	+	+	+	+	+	+	+	+	+	+	+	+	+	+	+	+	+	+	+	+	+	+	+	+	+	+	+	+	+	+	+	+	+	+	+	+	+	+	+	+	+	+	+
391	420	dCMP deaminase (EC 3.5.4.12); Late competence protein ComEB	57	+	+	+	+	+	+	+	+	+	+	+	+	+	+	+	+	+	+	+	+	+	+	+	+	+	+	+	+	+	+	+	+	+	+	+	+	+	+	+	+	+	+	+	+	+	+	+	+	+	+	+	+	+	+	+	+	+
392	421	Ribosomal RNA small subunit methyltransferase E (EC 2.1.1.-)	57	+	+	+	+	+	+	+	+	+	+	+	+	+	+	+	+	+	+	+	+	+	+	+	+	+	+	+	+	+	+	+	+	+	+	+	+	+	+	+	+	+	+	+	+	+	+	+	+	+	+	+	+	+	+	+	+	+
393	423	FIG011178: rRNA methylase	57	+	+	+	+	+	+	+	+	+	+	+	+	+	+	+	+	+	+	+	+	+	+	+	+	+	+	+	+	+	+	+	+	+	+	+	+	+	+	+	+	+	+	+	+	+	+	+	+	+	+	+	+	+	+	+	+	+
394	425	Transcriptional regulator CtsR	57	+	+	+	+	+	+	+	+	+	+	+	+	+	+	+	+	+	+	+	+	+	+	+	+	+	+	+	+	+	+	+	+	+	+	+	+	+	+	+	+	+	+	+	+	+	+	+	+	+	+	+	+	+	+	+	+	+
395	426	Phosphate transport system permease protein PstC (TC 3.A.1.7.1)	57	+	+	+	+	+	+	+	+	+	+	+	+	+	+	+	+	+	+	+	+	+	+	+	+	+	+	+	+	+	+	+	+	+	+	+	+	+	+	+	+	+	+	+	+	+	+	+	+	+	+	+	+	+	+	+	+	+
396	427	Protein of unknown function DUF28	57	+	+	+	+	+	+	+	+	+	+	+	+	+	+	+	+	+	+	+	+	+	+	+	+	+	+	+	+	+	+	+	+	+	+	+	+	+	+	+	+	+	+	+	+	+	+	+	+	+	+	+	+	+	+	+	+	+
397	428	PTS system, maltose and glucose-specific IIC component (EC 2.7.1.69) / PTS system, maltose and glucose-specific IIB component (EC 2.7.1.69) / PTS system, maltose and glucose-specific IIA component (EC 2.7.1.69)	57	+	+	+	+	+	+	+	+	+	+	+	+	+	+	+	+	+	+	+	+	+	+	+	+	+	+	+	+	+	+	+	+	+	+	+	+	+	+	+	+	+	+	+	+	+	+	+	+	+	+	+	+	+	+	+	+	+
398	429	GTP-binding and nucleic acid-binding protein YchF	57	+	+	+	+	+	+	+	+	+	+	+	+	+	+	+	+	+	+	+	+	+	+	+	+	+	+	+	+	+	+	+	+	+	+	+	+	+	+	+	+	+	+	+	+	+	+	+	+	+	+	+	+	+	+	+	+	+
399	430	DNA-directed RNA polymerase alpha subunit (EC 2.7.7.6)	57	+	+	+	+	+	+	+	+	+	+	+	+	+	+	+	+	+	+	+	+	+	+	+	+	+	+	+	+	+	+	+	+	+	+	+	+	+	+	+	+	+	+	+	+	+	+	+	+	+	+	+	+	+	+	+	+	+
400	431	Inner membrane protein translocase component YidC, short form OxaI-like	57	+	+	+	+	+	+	+	+	+	+	+	+	+	+	+	+	+	+	+	+	+	+	+	+	+	+	+	+	+	+	+	+	+	+	+	+	+	+	+	+	+	+	+	+	+	+	+	+	+	+	+	+	+	+	+	+	+
401	432	Glycerol-3-phosphate dehydrogenase [NAD(P)+] (EC 1.1.1.94)	57	+	+	+	+	+	+	+	+	+	+	+	+	+	+	+	+	+	+	+	+	+	+	+	+	+	+	+	+	+	+	+	+	+	+	+	+	+	+	+	+	+	+	+	+	+	+	+	+	+	+	+	+	+	+	+	+	+
402	433	Heat shock protein GrpE	57	+	+	+	+	+	+	+	+	+	+	+	+	+	+	+	+	+	+	+	+	+	+	+	+	+	+	+	+	+	+	+	+	+	+	+	+	+	+	+	+	+	+	+	+	+	+	+	+	+	+	+	+	+	+	+	+	+
403	434	Histidine triad (HIT) nucleotide-binding protein, similarity with At5g48545 and yeast YDL125C (HNT1)	57	+	+	+	+	+	+	+	+	+	+	+	+	+	+	+	+	+	+	+	+	+	+	+	+	+	+	+	+	+	+	+	+	+	+	+	+	+	+	+	+	+	+	+	+	+	+	+	+	+	+	+	+	+	+	+	+	+
404	435	Spermidine Putrescine ABC transporter permease component potC (TC_3.A.1.11.1)	57	+	+	+	+	+	+	+	+	+	+	+	+	+	+	+	+	+	+	+	+	+	+	+	+	+	+	+	+	+	+	+	+	+	+	+	+	+	+	+	+	+	+	+	+	+	+	+	+	+	+	+	+	+	+	+	+	+
405	436	DNA polymerase III subunits gamma and tau (EC 2.7.7.7)	57	+	+	+	+	+	+	+	+	+	+	+	+	+	+	+	+	+	+	+	+	+	+	+	+	+	+	+	+	+	+	+	+	+	+	+	+	+	+	+	+	+	+	+	+	+	+	+	+	+	+	+	+	+	+	+	+	+
406	437	Glutamyl aminopeptidase (EC 3.4.11.7)	57	+	+	+	+	+	+	+	+	+	+	+	+	+	+	+	+	+	+	+	+	+	+	+	+	+	+	+	+	+	+	+	+	+	+	+	+	+	+	+	+	+	+	+	+	+	+	+	+	+	+	+	+	+	+	+	+	+
407	438	SSU ribosomal protein S21p	57	+	+	+	+	+	+	+	+	+	+	+	+	+	+	+	+	+	+	+	+	+	+	+	+	+	+	+	+	+	+	+	+	+	+	+	+	+	+	+	+	+	+	+	+	+	+	+	+	+	+	+	+	+	+	+	+	+
408	441	Pseudouridylate synthases, 23S RNA-specific	57	+	+	+	+	+	+	+	+	+	+	+	+	+	+	+	+	+	+	+	+	+	+	+	+	+	+	+	+	+	+	+	+	+	+	+	+	+	+	+	+	+	+	+	+	+	+	+	+	+	+	+	+	+	+	+	+	+
409	442	HPr kinase/phosphorylase (EC 2.7.1.-) (EC 2.7.4.-)	57	+	+	+	+	+	+	+	+	+	+	+	+	+	+	+	+	+	+	+	+	+	+	+	+	+	+	+	+	+	+	+	+	+	+	+	+	+	+	+	+	+	+	+	+	+	+	+	+	+	+	+	+	+	+	+	+	+
410	443	Streptococcal lipoprotein rotamase A; Peptidyl-prolyl cis-trans isomerase (EC 5.2.1.8)	57	+	+	+	+	+	+	+	+	+	+	+	+	+	+	+	+	+	+	+	+	+	+	+	+	+	+	+	+	+	+	+	+	+	+	+	+	+	+	+	+	+	+	+	+	+	+	+	+	+	+	+	+	+	+	+	+	+
411	444	Metal-dependent hydrolase YbeY, involved in rRNA and/or ribosome maturation and assembly	57	+	+	+	+	+	+	+	+	+	+	+	+	+	+	+	+	+	+	+	+	+	+	+	+	+	+	+	+	+	+	+	+	+	+	+	+	+	+	+	+	+	+	+	+	+	+	+	+	+	+	+	+	+	+	+	+	+
412	445	UDP-N-acetylglucosamine 1-carboxyvinyltransferase (EC 2.5.1.7)	57	+	+	+	+	+	+	+	+	+	+	+	+	+	+	+	+	+	+	+	+	+	+	+	+	+	+	+	+	+	+	+	+	+	+	+	+	+	+	+	+	+	+	+	+	+	+	+	+	+	+	+	+	+	+	+	+	+
413	446	Transcription-repair coupling factor	57	+	+	+	+	+	+	+	+	+	+	+	+	+	+	+	+	+	+	+	+	+	+	+	+	+	+	+	+	+	+	+	+	+	+	+	+	+	+	+	+	+	+	+	+	+	+	+	+	+	+	+	+	+	+	+	+	+
414	447	FIG001721: Predicted N6-adenine-specific DNA methylase	57	+	+	+	+	+	+	+	+	+	+	+	+	+	+	+	+	+	+	+	+	+	+	+	+	+	+	+	+	+	+	+	+	+	+	+	+	+	+	+	+	+	+	+	+	+	+	+	+	+	+	+	+	+	+	+	+	+
415	449	6-phosphofructokinase (EC 2.7.1.11)	57	+	+	+	+	+	+	+	+	+	+	+	+	+	+	+	+	+	+	+	+	+	+	+	+	+	+	+	+	+	+	+	+	+	+	+	+	+	+	+	+	+	+	+	+	+	+	+	+	+	+	+	+	+	+	+	+	+
416	450	Cell division protein FtsI [Peptidoglycan synthetase] (EC 2.4.1.129)	57	+	+	+	+	+	+	+	+	+	+	+	+	+	+	+	+	+	+	+	+	+	+	+	+	+	+	+	+	+	+	+	+	+	+	+	+	+	+	+	+	+	+	+	+	+	+	+	+	+	+	+	+	+	+	+	+	+
417	452	Hemolysins and related proteins containing CBS domains	57	+	+	+	+	+	+	+	+	+	+	+	+	+	+	+	+	+	+	+	+	+	+	+	+	+	+	+	+	+	+	+	+	+	+	+	+	+	+	+	+	+	+	+	+	+	+	+	+	+	+	+	+	+	+	+	+	+
418	454	UDP-N-acetylmuramoylalanine--D-glutamate ligase (EC 6.3.2.9)	57	+	+	+	+	+	+	+	+	+	+	+	+	+	+	+	+	+	+	+	+	+	+	+	+	+	+	+	+	+	+	+	+	+	+	+	+	+	+	+	+	+	+	+	+	+	+	+	+	+	+	+	+	+	+	+	+	+
419	455	Dephospho-CoA kinase (EC 2.7.1.24)	57	+	+	+	+	+	+	+	+	+	+	+	+	+	+	+	+	+	+	+	+	+	+	+	+	+	+	+	+	+	+	+	+	+	+	+	+	+	+	+	+	+	+	+	+	+	+	+	+	+	+	+	+	+	+	+	+	+
420	456	Putative amidotransferase similar to cobyric acid synthase	57	+	+	+	+	+	+	+	+	+	+	+	+	+	+	+	+	+	+	+	+	+	+	+	+	+	+	+	+	+	+	+	+	+	+	+	+	+	+	+	+	+	+	+	+	+	+	+	+	+	+	+	+	+	+	+	+	+
421	457	Uracil phosphoribosyltransferase (EC 2.4.2.9) / Pyrimidine operon regulatory protein PyrR	57	+	+	+	+	+	+	+	+	+	+	+	+	+	+	+	+	+	+	+	+	+	+	+	+	+	+	+	+	+	+	+	+	+	+	+	+	+	+	+	+	+	+	+	+	+	+	+	+	+	+	+	+	+	+	+	+	+
422	458	NADP-specific glutamate dehydrogenase (EC 1.4.1.4)	57	+	+	+	+	+	+	+	+	+	+	+	+	+	+	+	+	+	+	+	+	+	+	+	+	+	+	+	+	+	+	+	+	+	+	+	+	+	+	+	+	+	+	+	+	+	+	+	+	+	+	+	+	+	+	+	+	+
423	460	Late competence protein ComGB, access of DNA to ComEA	57	+	+	+	+	+	+	+	+	+	+	+	+	+	+	+	+	+	+	+	+	+	+	+	+	+	+	+	+	+	+	+	+	+	+	+	+	+	+	+	+	+	+	+	+	+	+	+	+	+	+	+	+	+	+	+	+	+
424	461	Transcriptional regulator, MerR family	57	+	+	+	+	+	+	+	+	+	+	+	+	+	+	+	+	+	+	+	+	+	+	+	+	+	+	+	+	+	+	+	+	+	+	+	+	+	+	+	+	+	+	+	+	+	+	+	+	+	+	+	+	+	+	+	+	+
425	463	tmRNA-binding protein SmpB	57	+	+	+	+	+	+	+	+	+	+	+	+	+	+	+	+	+	+	+	+	+	+	+	+	+	+	+	+	+	+	+	+	+	+	+	+	+	+	+	+	+	+	+	+	+	+	+	+	+	+	+	+	+	+	+	+	+

Table S5. Precence/absence of each HG among 57 *S. suis* strains

				1																13			25					28						104		94			76			53/54		Divergent *S. suis*																
Index[a]	ClutserID	Gene products	Members	98HAH33	05ZYH33	SC84	A7	SS12	P1/7	DAT273	NIAH11434	JS14	GZ1	BM407	S735	13730	MNCM07	NCTC 10446	14636	NCTC 10237	ST1	2651	89-1591	MNCM04	MNCM25	8074	D9	4961	ST3	MNCM43	DAT292	DAT260	NIAH11435	MNCM21	MNCM50	89-2479	DAT234	DAT290	93A	42A	NT77	6407	11538	8830	12814	92-1400	89-590	2726	2524	89-3576-3	4417	D12	10581	89-5259	88-5299A	R61	92-1191	14A	92-4172	22083
426	464	50S ribosomal subunit maturation GTPase RbgA (B. subtilis YlqF)	57	+	+	+	+	+	+	+	+	+	+	+	+	+	+	+	+	+	+	+	+	+	+	+	+	+	+	+	+	+	+	+	+	+	+	+	+	+	+	+	+	+	+	+	+	+	+	+	+	+	+	+	+	+	+	+	+	+
427	468	ATP synthase C chain (EC 3.6.3.14)	57	+	+	+	+	+	+	+	+	+	+	+	+	+	+	+	+	+	+	+	+	+	+	+	+	+	+	+	+	+	+	+	+	+	+	+	+	+	+	+	+	+	+	+	+	+	+	+	+	+	+	+	+	+	+	+	+	+
428	469	Excinuclease ABC subunit B	57	+	+	+	+	+	+	+	+	+	+	+	+	+	+	+	+	+	+	+	+	+	+	+	+	+	+	+	+	+	+	+	+	+	+	+	+	+	+	+	+	+	+	+	+	+	+	+	+	+	+	+	+	+	+	+	+	+
429	470	Hydroxyacylglutathione hydrolase (EC 3.1.2.6)	57	+	+	+	+	+	+	+	+	+	+	+	+	+	+	+	+	+	+	+	+	+	+	+	+	+	+	+	+	+	+	+	+	+	+	+	+	+	+	+	+	+	+	+	+	+	+	+	+	+	+	+	+	+	+	+	+	+
430	472	Similar to ribosomal large subunit pseudouridine synthase D, Bacillus subtilis YjbO type	57	+	+	+	+	+	+	+	+	+	+	+	+	+	+	+	+	+	+	+	+	+	+	+	+	+	+	+	+	+	+	+	+	+	+	+	+	+	+	+	+	+	+	+	+	+	+	+	+	+	+	+	+	+	+	+	+	+
431	473	ATP-dependent RNA helicase YfmL	57	+	+	+	+	+	+	+	+	+	+	+	+	+	+	+	+	+	+	+	+	+	+	+	+	+	+	+	+	+	+	+	+	+	+	+	+	+	+	+	+	+	+	+	+	+	+	+	+	+	+	+	+	+	+	+	+	+
432	475	tRNA pseudouridine synthase B (EC 4.2.1.70)	57	+	+	+	+	+	+	+	+	+	+	+	+	+	+	+	+	+	+	+	+	+	+	+	+	+	+	+	+	+	+	+	+	+	+	+	+	+	+	+	+	+	+	+	+	+	+	+	+	+	+	+	+	+	+	+	+	+
433	476	Putative deoxyribose-specific ABC transporter, permease protein	57	+	+	+	+	+	+	+	+	+	+	+	+	+	+	+	+	+	+	+	+	+	+	+	+	+	+	+	+	+	+	+	+	+	+	+	+	+	+	+	+	+	+	+	+	+	+	+	+	+	+	+	+	+	+	+	+	+
434	477	Putrescine transport ATP-binding protein PotA (TC 3.A.1.11.1)	57	+	+	+	+	+	+	+	+	+	+	+	+	+	+	+	+	+	+	+	+	+	+	+	+	+	+	+	+	+	+	+	+	+	+	+	+	+	+	+	+	+	+	+	+	+	+	+	+	+	+	+	+	+	+	+	+	+
435	478	Adenylosuccinate lyase (EC 4.3.2.2)	57	+	+	+	+	+	+	+	+	+	+	+	+	+	+	+	+	+	+	+	+	+	+	+	+	+	+	+	+	+	+	+	+	+	+	+	+	+	+	+	+	+	+	+	+	+	+	+	+	+	+	+	+	+	+	+	+	+
436	479	Pantothenate kinase (EC 2.7.1.33)	57	+	+	+	+	+	+	+	+	+	+	+	+	+	+	+	+	+	+	+	+	+	+	+	+	+	+	+	+	+	+	+	+	+	+	+	+	+	+	+	+	+	+	+	+	+	+	+	+	+	+	+	+	+	+	+	+	+
437	481	Uracil-DNA glycosylase, family 1	57	+	+	+	+	+	+	+	+	+	+	+	+	+	+	+	+	+	+	+	+	+	+	+	+	+	+	+	+	+	+	+	+	+	+	+	+	+	+	+	+	+	+	+	+	+	+	+	+	+	+	+	+	+	+	+	+	+
438	482	Pneumococcal vaccine antigen A homolog	57	+	+	+	+	+	+	+	+	+	+	+	+	+	+	+	+	+	+	+	+	+	+	+	+	+	+	+	+	+	+	+	+	+	+	+	+	+	+	+	+	+	+	+	+	+	+	+	+	+	+	+	+	+	+	+	+	+
439	483	Diacylglycerol kinase (EC 2.7.1.107)	57	+	+	+	+	+	+	+	+	+	+	+	+	+	+	+	+	+	+	+	+	+	+	+	+	+	+	+	+	+	+	+	+	+	+	+	+	+	+	+	+	+	+	+	+	+	+	+	+	+	+	+	+	+	+	+	+	+
440	485	Phenylalanyl-tRNA synthetase alpha chain (EC 6.1.1.20)	57	+	+	+	+	+	+	+	+	+	+	+	+	+	+	+	+	+	+	+	+	+	+	+	+	+	+	+	+	+	+	+	+	+	+	+	+	+	+	+	+	+	+	+	+	+	+	+	+	+	+	+	+	+	+	+	+	+
441	487	tRNA delta(2)-isopentenylpyrophosphate transferase (EC 2.5.1.8)	57	+	+	+	+	+	+	+	+	+	+	+	+	+	+	+	+	+	+	+	+	+	+	+	+	+	+	+	+	+	+	+	+	+	+	+	+	+	+	+	+	+	+	+	+	+	+	+	+	+	+	+	+	+	+	+	+	+
442	489	1-phosphofructokinase (EC 2.7.1.56)	57	+	+	+	+	+	+	+	+	+	+	+	+	+	+	+	+	+	+	+	+	+	+	+	+	+	+	+	+	+	+	+	+	+	+	+	+	+	+	+	+	+	+	+	+	+	+	+	+	+	+	+	+	+	+	+	+	+
443	491	Transcriptional regulator in cluster with beta-lactamase, GntR family	57	+	+	+	+	+	+	+	+	+	+	+	+	+	+	+	+	+	+	+	+	+	+	+	+	+	+	+	+	+	+	+	+	+	+	+	+	+	+	+	+	+	+	+	+	+	+	+	+	+	+	+	+	+	+	+	+	+
444	492	Substrate-specific component RibU of riboflavin ECF transporter	57	+	+	+	+	+	+	+	+	+	+	+	+	+	+	+	+	+	+	+	+	+	+	+	+	+	+	+	+	+	+	+	+	+	+	+	+	+	+	+	+	+	+	+	+	+	+	+	+	+	+	+	+	+	+	+	+	+
445	493	DNA replication intiation control protein YabA	57	+	+	+	+	+	+	+	+	+	+	+	+	+	+	+	+	+	+	+	+	+	+	+	+	+	+	+	+	+	+	+	+	+	+	+	+	+	+	+	+	+	+	+	+	+	+	+	+	+	+	+	+	+	+	+	+	+
446	496	Hydrolase (HAD superfamily)	57	+	+	+	+	+	+	+	+	+	+	+	+	+	+	+	+	+	+	+	+	+	+	+	+	+	+	+	+	+	+	+	+	+	+	+	+	+	+	+	+	+	+	+	+	+	+	+	+	+	+	+	+	+	+	+	+	+
447	497	TrmH family tRNA/rRNA methyltransferase YacO (EC 2.1.1.-)	57	+	+	+	+	+	+	+	+	+	+	+	+	+	+	+	+	+	+	+	+	+	+	+	+	+	+	+	+	+	+	+	+	+	+	+	+	+	+	+	+	+	+	+	+	+	+	+	+	+	+	+	+	+	+	+	+	+
448	499	Chromosomal replication initiator protein DnaA	57	+	+	+	+	+	+	+	+	+	+	+	+	+	+	+	+	+	+	+	+	+	+	+	+	+	+	+	+	+	+	+	+	+	+	+	+	+	+	+	+	+	+	+	+	+	+	+	+	+	+	+	+	+	+	+	+	+
449	500	Purine nucleoside phosphorylase (EC 2.4.2.1)	57	+	+	+	+	+	+	+	+	+	+	+	+	+	+	+	+	+	+	+	+	+	+	+	+	+	+	+	+	+	+	+	+	+	+	+	+	+	+	+	+	+	+	+	+	+	+	+	+	+	+	+	+	+	+	+	+	+
450	501	SSU ribosomal protein S20p	57	+	+	+	+	+	+	+	+	+	+	+	+	+	+	+	+	+	+	+	+	+	+	+	+	+	+	+	+	+	+	+	+	+	+	+	+	+	+	+	+	+	+	+	+	+	+	+	+	+	+	+	+	+	+	+	+	+
451	502	Formamidopyrimidine-DNA glycosylase (EC 3.2.2.23)	57	+	+	+	+	+	+	+	+	+	+	+	+	+	+	+	+	+	+	+	+	+	+	+	+	+	+	+	+	+	+	+	+	+	+	+	+	+	+	+	+	+	+	+	+	+	+	+	+	+	+	+	+	+	+	+	+	+
452	504	DNA polymerase III beta subunit (EC 2.7.7.7)	57	+	+	+	+	+	+	+	+	+	+	+	+	+	+	+	+	+	+	+	+	+	+	+	+	+	+	+	+	+	+	+	+	+	+	+	+	+	+	+	+	+	+	+	+	+	+	+	+	+	+	+	+	+	+	+	+	+
453	505	Chorismate synthase (EC 4.2.3.5)	57	+	+	+	+	+	+	+	+	+	+	+	+	+	+	+	+	+	+	+	+	+	+	+	+	+	+	+	+	+	+	+	+	+	+	+	+	+	+	+	+	+	+	+	+	+	+	+	+	+	+	+	+	+	+	+	+	+
454	506	Glucosamine-6-phosphate deaminase (EC 3.5.99.6)	57	+	+	+	+	+	+	+	+	+	+	+	+	+	+	+	+	+	+	+	+	+	+	+	+	+	+	+	+	+	+	+	+	+	+	+	+	+	+	+	+	+	+	+	+	+	+	+	+	+	+	+	+	+	+	+	+	+
455	507	hypothetical protein	57	+	+	+	+	+	+	+	+	+	+	+	+	+	+	+	+	+	+	+	+	+	+	+	+	+	+	+	+	+	+	+	+	+	+	+	+	+	+	+	+	+	+	+	+	+	+	+	+	+	+	+	+	+	+	+	+	+
456	508	Thiamin pyrophosphokinase (EC 2.7.6.2)	57	+	+	+	+	+	+	+	+	+	+	+	+	+	+	+	+	+	+	+	+	+	+	+	+	+	+	+	+	+	+	+	+	+	+	+	+	+	+	+	+	+	+	+	+	+	+	+	+	+	+	+	+	+	+	+	+	+
457	509	phosphoribosyltransferase	57	+	+	+	+	+	+	+	+	+	+	+	+	+	+	+	+	+	+	+	+	+	+	+	+	+	+	+	+	+	+	+	+	+	+	+	+	+	+	+	+	+	+	+	+	+	+	+	+	+	+	+	+	+	+	+	+	+
458	510	Ribosomal protein L11 methyltransferase (EC 2.1.1.-)	57	+	+	+	+	+	+	+	+	+	+	+	+	+	+	+	+	+	+	+	+	+	+	+	+	+	+	+	+	+	+	+	+	+	+	+	+	+	+	+	+	+	+	+	+	+	+	+	+	+	+	+	+	+	+	+	+	+
459	512	Biotin-protein ligase (EC 6.3.4.15) / Biotin operon repressor	57	+	+	+	+	+	+	+	+	+	+	+	+	+	+	+	+	+	+	+	+	+	+	+	+	+	+	+	+	+	+	+	+	+	+	+	+	+	+	+	+	+	+	+	+	+	+	+	+	+	+	+	+	+	+	+	+	+
460	513	hypothetical protein	57	+	+	+	+	+	+	+	+	+	+	+	+	+	+	+	+	+	+	+	+	+	+	+	+	+	+	+	+	+	+	+	+	+	+	+	+	+	+	+	+	+	+	+	+	+	+	+	+	+	+	+	+	+	+	+	+	+
461	515	hypothetical protein	57	+	+	+	+	+	+	+	+	+	+	+	+	+	+	+	+	+	+	+	+	+	+	+	+	+	+	+	+	+	+	+	+	+	+	+	+	+	+	+	+	+	+	+	+	+	+	+	+	+	+	+	+	+	+	+	+	+
462	516	Acetyl-coenzyme A carboxyl transferase alpha chain (EC 6.4.1.2)	57	+	+	+	+	+	+	+	+	+	+	+	+	+	+	+	+	+	+	+	+	+	+	+	+	+	+	+	+	+	+	+	+	+	+	+	+	+	+	+	+	+	+	+	+	+	+	+	+	+	+	+	+	+	+	+	+	+
463	517	Peptide chain release factor 1	57	+	+	+	+	+	+	+	+	+	+	+	+	+	+	+	+	+	+	+	+	+	+	+	+	+	+	+	+	+	+	+	+	+	+	+	+	+	+	+	+	+	+	+	+	+	+	+	+	+	+	+	+	+	+	+	+	+
464	518	Cell division protein FtsW	57	+	+	+	+	+	+	+	+	+	+	+	+	+	+	+	+	+	+	+	+	+	+	+	+	+	+	+	+	+	+	+	+	+	+	+	+	+	+	+	+	+	+	+	+	+	+	+	+	+	+	+	+	+	+	+	+	+
465	519	Ribonuclease P protein component (EC 3.1.26.5)	57	+	+	+	+	+	+	+	+	+	+	+	+	+	+	+	+	+	+	+	+	+	+	+	+	+	+	+	+	+	+	+	+	+	+	+	+	+	+	+	+	+	+	+	+	+	+	+	+	+	+	+	+	+	+	+	+	+
466	520	General stress protein 13	57	+	+	+	+	+	+	+	+	+	+	+	+	+	+	+	+	+	+	+	+	+	+	+	+	+	+	+	+	+	+	+	+	+	+	+	+	+	+	+	+	+	+	+	+	+	+	+	+	+	+	+	+	+	+	+	+	+
467	521	tRNA-guanine transglycosylase (EC 2.4.2.29)	57	+	+	+	+	+	+	+	+	+	+	+	+	+	+	+	+	+	+	+	+	+	+	+	+	+	+	+	+	+	+	+	+	+	+	+	+	+	+	+	+	+	+	+	+	+	+	+	+	+	+	+	+	+	+	+	+	+
468	525	Cell division protein FtsI [Peptidoglycan synthetase] (EC 2.4.1.129)	57	+	+	+	+	+	+	+	+	+	+	+	+	+	+	+	+	+	+	+	+	+	+	+	+	+	+	+	+	+	+	+	+	+	+	+	+	+	+	+	+	+	+	+	+	+	+	+	+	+	+	+	+	+	+	+	+	+
469	527	GAF domain-containing proteins	57	+	+	+	+	+	+	+	+	+	+	+	+	+	+	+	+	+	+	+	+	+	+	+	+	+	+	+	+	+	+	+	+	+	+	+	+	+	+	+	+	+	+	+	+	+	+	+	+	+	+	+	+	+	+	+	+	+
470	529	Ribosomal RNA small subunit methyltransferase B (EC 2.1.1.-)	57	+	+	+	+	+	+	+	+	+	+	+	+	+	+	+	+	+	+	+	+	+	+	+	+	+	+	+	+	+	+	+	+	+	+	+	+	+	+	+	+	+	+	+	+	+	+	+	+	+	+	+	+	+	+	+	+	+
471	530	Ribonuclease HIII (EC 3.1.26.4)	57	+	+	+	+	+	+	+	+	+	+	+	+	+	+	+	+	+	+	+	+	+	+	+	+	+	+	+	+	+	+	+	+	+	+	+	+	+	+	+	+	+	+	+	+	+	+	+	+	+	+	+	+	+	+	+	+	+
472	531	SSU ribosomal protein S11p (S14e)	57	+	+	+	+	+	+	+	+	+	+	+	+	+	+	+	+	+	+	+	+	+	+	+	+	+	+	+	+	+	+	+	+	+	+	+	+	+	+	+	+	+	+	+	+	+	+	+	+	+	+	+	+	+	+	+	+	+
473	532	5-formyltetrahydrofolate cyclo-ligase (EC 6.3.3.2)	57	+	+	+	+	+	+	+	+	+	+	+	+	+	+	+	+	+	+	+	+	+	+	+	+	+	+	+	+	+	+	+	+	+	+	+	+	+	+	+	+	+	+	+	+	+	+	+	+	+	+	+	+	+	+	+	+	+
474	533	DNA-binding protein HBsu	57	+	+	+	+	+	+	+	+	+	+	+	+	+	+	+	+	+	+	+	+	+	+	+	+	+	+	+	+	+	+	+	+	+	+	+	+	+	+	+	+	+	+	+	+	+	+	+	+	+	+	+	+	+	+	+	+	+
475	536	Glucose-1-phosphate thymidylyltransferase (EC 2.7.7.24)	57	+	+	+	+	+	+	+	+	+	+	+	+	+	+	+	+	+	+	+	+	+	+	+	+	+	+	+	+	+	+	+	+	+	+	+	+	+	+	+	+	+	+	+	+	+	+	+	+	+	+	+	+	+	+	+	+	+
476	537	Ribosomal large subunit pseudouridine synthase D (EC 4.2.1.70)	57	+	+	+	+	+	+	+	+	+	+	+	+	+	+	+	+	+	+	+	+	+	+	+	+	+	+	+	+	+	+	+	+	+	+	+	+	+	+	+	+	+	+	+	+	+	+	+	+	+	+	+	+	+	+	+	+	+
477	538	UPF0135 protein Bsu YqfO	57	+	+	+	+	+	+	+	+	+	+	+	+	+	+	+	+	+	+	+	+	+	+	+	+	+	+	+	+	+	+	+	+	+	+	+	+	+	+	+	+	+	+	+	+	+	+	+	+	+	+	+	+	+	+	+	+	+
478	539	Spermidine Putrescine ABC transporter permease component PotB (TC 3.A.1.11.1)	57	+	+	+	+	+	+	+	+	+	+	+	+	+	+	+	+	+	+	+	+	+	+	+	+	+	+	+	+	+	+	+	+	+	+	+	+	+	+	+	+	+	+	+	+	+	+	+	+	+	+	+	+	+	+	+	+	+
479	541	Serine hydroxymethyltransferase (EC 2.1.2.1)	57	+	+	+	+	+	+	+	+	+	+	+	+	+	+	+	+	+	+	+	+	+	+	+	+	+	+	+	+	+	+	+	+	+	+	+	+	+	+	+	+	+	+	+	+	+	+	+	+	+	+	+	+	+	+	+	+	+
480	542	COG4478, integral membrane protein	57	+	+	+	+	+	+	+	+	+	+	+	+	+	+	+	+	+	+	+	+	+	+	+	+	+	+	+	+	+	+	+	+	+	+	+	+	+	+	+	+	+	+	+	+	+	+	+	+	+	+	+	+	+	+	+	+	+
481	543	Intramembrane protease RasP/YluC, implicated in cell division based on FtsL cleavage	57	+	+	+	+	+	+	+	+	+	+	+	+	+	+	+	+	+	+	+	+	+	+	+	+	+	+	+	+	+	+	+	+	+	+	+	+	+	+	+	+	+	+	+	+	+	+	+	+	+	+	+	+	+	+	+	+	+
482	545	Ribosomal-protein-S5p-alanine acetyltransferase	57	+	+	+	+	+	+	+	+	+	+	+	+	+	+	+	+	+	+	+	+	+	+	+	+	+	+	+	+	+	+	+	+	+	+	+	+	+	+	+	+	+	+	+	+	+	+	+	+	+	+	+	+	+	+	+	+	+
483	546	Deoxyuridine 5'-triphosphate nucleotidohydrolase (EC 3.6.1.23)	57	+	+	+	+	+	+	+	+	+	+	+	+	+	+	+	+	+	+	+	+	+	+	+	+	+	+	+	+	+	+	+	+	+	+	+	+	+	+	+	+	+	+	+	+	+	+	+	+	+	+	+	+	+	+	+	+	+
484	550	Heat shock protein 60 family chaperone GroEL	57	+	+	+	+	+	+	+	+	+	+	+	+	+	+	+	+	+	+	+	+	+	+	+	+	+	+	+	+	+	+	+	+	+	+	+	+	+	+	+	+	+	+	+	+	+	+	+	+	+	+	+	+	+	+	+	+	+
485	552	Peptidyl-tRNA hydrolase (EC 3.1.1.29)	57	+	+	+	+	+	+	+	+	+	+	+	+	+	+	+	+	+	+	+	+	+	+	+	+	+	+	+	+	+	+	+	+	+	+	+	+	+	+	+	+	+	+	+	+	+	+	+	+	+	+	+	+	+	+	+	+	+
486	553	Hydroxymethylglutaryl-CoA reductase (EC 1.1.1.34)	57	+	+	+	+	+	+	+	+	+	+	+	+	+	+	+	+	+	+	+	+	+	+	+	+	+	+	+	+	+	+	+	+	+	+	+	+	+	+	+	+	+	+	+	+	+	+	+	+	+	+	+	+	+	+	+	+	+
487	555	Phosphoglucosamine mutase (EC 5.4.2.10)	57	+	+	+	+	+	+	+	+	+	+	+	+	+	+	+	+	+	+	+	+	+	+	+	+	+	+	+	+	+	+	+	+	+	+	+	+	+	+	+	+	+	+	+	+	+	+	+	+	+	+	+	+	+	+	+	+	+
488	558	Aspartyl-tRNA(Asn) amidotransferase subunit C (EC 6.3.5.6) @ Glutamyl-tRNA(Gln) amidotransferase subunit C (EC 6.3.5.7)	57	+	+	+	+	+	+	+	+	+	+	+	+	+	+	+	+	+	+	+	+	+	+	+	+	+	+	+	+	+	+	+	+	+	+	+	+	+	+	+	+	+	+	+	+	+	+	+	+	+	+	+	+	+	+	+	+	+
489	559	Xaa-His dipeptidase (EC 3.4.13.3)	57	+	+	+	+	+	+	+	+	+	+	+	+	+	+	+	+	+	+	+	+	+	+	+	+	+	+	+	+	+	+	+	+	+	+	+	+	+	+	+	+	+	+	+	+	+	+	+	+	+	+	+	+	+	+	+	+	+
490	560	Glycyl-tRNA synthetase alpha chain (EC 6.1.1.14)	57	+	+	+	+	+	+	+	+	+	+	+	+	+	+	+	+	+	+	+	+	+	+	+	+	+	+	+	+	+	+	+	+	+	+	+	+	+	+	+	+	+	+	+	+	+	+	+	+	+	+	+	+	+	+	+	+	+
491	562	Phosphopantothenoylcysteine synthetase (EC 6.3.2.5)	57	+	+	+	+	+	+	+	+	+	+	+	+	+	+	+	+	+	+	+	+	+	+	+	+	+	+	+	+	+	+	+	+	+	+	+	+	+	+	+	+	+	+	+	+	+	+	+	+	+	+	+	+	+	+	+	+	+
492	563	4-oxalocrotonate tautomerase (EC 5.3.2.-); Xylose transport system permease protein xylH	57	+	+	+	+	+	+	+	+	+	+	+	+	+	+	+	+	+	+	+	+	+	+	+	+	+	+	+	+	+	+	+	+	+	+	+	+	+	+	+	+	+	+	+	+	+	+	+	+	+	+	+	+	+	+	+	+	+
493	566	Preprotein translocase subunit SecG (TC 3.A.5.1.1)	57	+	+	+	+	+	+	+	+	+	+	+	+	+	+	+	+	+	+	+	+	+	+	+	+	+	+	+	+	+	+	+	+	+	+	+	+	+	+	+	+	+	+	+	+	+	+	+	+	+	+	+	+	+	+	+	+	+
494	567	Translation elongation factor P	57	+	+	+	+	+	+	+	+	+	+	+	+	+	+	+	+	+	+	+	+	+	+	+	+	+	+	+	+	+	+	+	+	+	+	+	+	+	+	+	+	+	+	+	+	+	+	+	+	+	+	+	+	+	+	+	+	+
495	568	tRNA:m(5)U-54 MTase gid	57	+	+	+	+	+	+	+	+	+	+	+	+	+	+	+	+	+	+	+	+	+	+	+	+	+	+	+	+	+	+	+	+	+	+	+	+	+	+	+	+	+	+	+	+	+	+	+	+	+	+	+	+	+	+	+	+	+
496	569	Hypoxanthine-guanine phosphoribosyltransferase (EC 2.4.2.8)	57	+	+	+	+	+	+	+	+	+	+	+	+	+	+	+	+	+	+	+	+	+	+	+	+	+	+	+	+	+	+	+	+	+	+	+	+	+	+	+	+	+	+	+	+	+	+	+	+	+	+	+	+	+	+	+	+	+
497	570	Heat shock protein 60 family co-chaperone GroES	57	+	+	+	+	+	+	+	+	+	+	+	+	+	+	+	+	+	+	+	+	+	+	+	+	+	+	+	+	+	+	+	+	+	+	+	+	+	+	+	+	+	+	+	+	+	+	+	+	+	+	+	+	+	+	+	+	+
498	571	Two component transcriptional regulator VraR	57	+	+	+	+	+	+	+	+	+	+	+	+	+	+	+	+	+	+	+	+	+	+	+	+	+	+	+	+	+	+	+	+	+	+	+	+	+	+	+	+	+	+	+	+	+	+	+	+	+	+	+	+	+	+	+	+	+
499	572	Excinuclease ABC subunit C	57	+	+	+	+	+	+	+	+	+	+	+	+	+	+	+	+	+	+	+	+	+	+	+	+	+	+	+	+	+	+	+	+	+	+	+	+	+	+	+	+	+	+	+	+	+	+	+	+	+	+	+	+	+	+	+	+	+
500	573	LSU ribosomal protein L7/L12 (P1/P2)	57	+	+	+	+	+	+	+	+	+	+	+	+	+	+	+	+	+	+	+	+	+	+	+	+	+	+	+	+	+	+	+	+	+	+	+	+	+	+	+	+	+	+	+	+	+	+	+	+	+	+	+	+	+	+	+	+	+
501	574	Acyl carrier protein	57	+	+	+	+	+	+	+	+	+	+	+	+	+	+	+	+	+	+	+	+	+	+	+	+	+	+	+	+	+	+	+	+	+	+	+	+	+	+	+	+	+	+	+	+	+	+	+	+	+	+	+	+	+	+	+	+	+
502	577	FIG145533: Methyltransferase (EC 2.1.1.-)	57	+	+	+	+	+	+	+	+	+	+	+	+	+	+	+	+	+	+	+	+	+	+	+	+	+	+	+	+	+	+	+	+	+	+	+	+	+	+	+	+	+	+	+	+	+	+	+	+	+	+	+	+	+	+	+	+	+
503	578	Mutator mutT protein (7,8-dihydro-8-oxoguanine-triphosphatase) (EC 3.6.1.-)	57	+	+	+	+	+	+	+	+	+	+	+	+	+	+	+	+	+	+	+	+	+	+	+	+	+	+	+	+	+	+	+	+	+	+	+	+	+	+	+	+	+	+	+	+	+	+	+	+	+	+	+	+	+	+	+	+	+
504	579	D-alanine--D-alanine ligase A (EC 6.3.2.4)	57	+	+	+	+	+	+	+	+	+	+	+	+	+	+	+	+	+	+	+	+	+	+	+	+	+	+	+	+	+	+	+	+	+	+	+	+	+	+	+	+	+	+	+	+	+	+	+	+	+	+	+	+	+	+	+	+	+
505	580	L-asparaginase (EC 3.5.1.1)	57	+	+	+	+	+	+	+	+	+	+	+	+	+	+	+	+	+	+	+	+	+	+	+	+	+	+	+	+	+	+	+	+	+	+	+	+	+	+	+	+	+	+	+	+	+	+	+	+	+	+	+	+	+	+	+	+	+
506	581	Ribose 5-phosphate isomerase A (EC 5.3.1.6)	57	+	+	+	+	+	+	+	+	+	+	+	+	+	+	+	+	+	+	+	+	+	+	+	+	+	+	+	+	+	+	+	+	+	+	+	+	+	+	+	+	+	+	+	+	+	+	+	+	+	+	+	+	+	+	+	+	+
507	582	Arginyl-tRNA synthetase (EC 6.1.1.19)	57	+	+	+	+	+	+	+	+	+	+	+	+	+	+	+	+	+	+	+	+	+	+	+	+	+	+	+	+	+	+	+	+	+	+	+	+	+	+	+	+	+	+	+	+	+	+	+	+	+	+	+	+	+	+	+	+	+
508	584	DNA recombination and repair protein RecF	57	+	+	+	+	+	+	+	+	+	+	+	+	+	+	+	+	+	+	+	+	+	+	+	+	+	+	+	+	+	+	+	+	+	+	+	+	+	+	+	+	+	+	+	+	+	+	+	+	+	+	+	+	+	+	+	+	+
509	585	Phosphotransferase system, phosphocarrier protein HPr	57	+	+	+	+	+	+	+	+	+	+	+	+	+	+	+	+	+	+	+	+	+	+	+	+	+	+	+	+	+	+	+	+	+	+	+	+	+	+	+	+	+	+	+	+	+	+	+	+	+	+	+	+	+	+	+	+	+
510	589	Ribosome-associated heat shock protein implicated in the recycling of the 50S subunit (S4 paralog)	57	+	+	+	+	+	+	+	+	+	+	+	+	+	+	+	+	+	+	+	+	+	+	+	+	+	+	+	+	+	+	+	+	+	+	+	+	+	+	+	+	+	+	+	+	+	+	+	+	+	+	+	+	+	+	+	+	+
511	591	Lipoteichoic acid synthase LtaS Type IIc	57	+	+	+	+	+	+	+	+	+	+	+	+	+	+	+	+	+	+	+	+	+	+	+	+	+	+	+	+	+	+	+	+	+	+	+	+	+	+	+	+	+	+	+	+	+	+	+	+	+	+	+	+	+	+	+	+	+
512	593	Ribonuclease Z (EC 3.1.26.11)	57	+	+	+	+	+	+	+	+	+	+	+	+	+	+	+	+	+	+	+	+	+	+	+	+	+	+	+	+	+	+	+	+	+	+	+	+	+	+	+	+	+	+	+	+	+	+	+	+	+	+	+	+	+	+	+	+	+
513	595	Substrate-specific component QueT (COG4708) of predicted queuosine-regulated ECF transporter	57	+	+	+	+	+	+	+	+	+	+	+	+	+	+	+	+	+	+	+	+	+	+	+	+	+	+	+	+	+	+	+	+	+	+	+	+	+	+	+	+	+	+	+	+	+	+	+	+	+	+	+	+	+	+	+	+	+
514	596	SSU ribosomal protein S10p (S20e)	57	+	+	+	+	+	+	+	+	+	+	+	+	+	+	+	+	+	+	+	+	+	+	+	+	+	+	+	+	+	+	+	+	+	+	+	+	+	+	+	+	+	+	+	+	+	+	+	+	+	+	+	+	+	+	+	+	+
515	597	Purine nucleoside phosphorylase (EC 2.4.2.1)	57	+	+	+	+	+	+	+	+	+	+	+	+	+	+	+	+	+	+	+	+	+	+	+	+	+	+	+	+	+	+	+	+	+	+	+	+	+	+	+	+	+	+	+	+	+	+	+	+	+	+	+	+	+	+	+	+	+
516	598	Peptidyl-prolyl cis-trans isomerase (EC 5.2.1.8)	57	+	+	+	+	+	+	+	+	+	+	+	+	+	+	+	+	+	+	+	+	+	+	+	+	+	+	+	+	+	+	+	+	+	+	+	+	+	+	+	+	+	+	+	+	+	+	+	+	+	+	+	+	+	+	+	+	+
517	600	Protein YidD	57	+	+	+	+	+	+	+	+	+	+	+	+	+	+	+	+	+	+	+	+	+	+	+	+	+	+	+	+	+	+	+	+	+	+	+	+	+	+	+	+	+	+	+	+	+	+	+	+	+	+	+	+	+	+	+	+	+
518	604	Succinyl-CoA synthetase, alpha subunit-related enzymes	57	+	+	+	+	+	+	+	+	+	+	+	+	+	+	+	+	+	+	+	+	+	+	+	+	+	+	+	+	+	+	+	+	+	+	+	+	+	+	+	+	+	+	+	+	+	+	+	+	+	+	+	+	+	+	+	+	+
519	607	LSU ribosomal protein L17p	57	+	+	+	+	+	+	+	+	+	+	+	+	+	+	+	+	+	+	+	+	+	+	+	+	+	+	+	+	+	+	+	+	+	+	+	+	+	+	+	+	+	+	+	+	+	+	+	+	+	+	+	+	+	+	+	+	+
520	608	Translation elongation factor Ts	57	+	+	+	+	+	+	+	+	+	+	+	+	+	+	+	+	+	+	+	+	+	+	+	+	+	+	+	+	+	+	+	+	+	+	+	+	+	+	+	+	+	+	+	+	+	+	+	+	+	+	+	+	+	+	+	+	+
521	609	Capsule biosynthesis protein capA	57	+	+	+	+	+	+	+	+	+	+	+	+	+	+	+	+	+	+	+	+	+	+	+	+	+	+	+	+	+	+	+	+	+	+	+	+	+	+	+	+	+	+	+	+	+	+	+	+	+	+	+	+	+	+	+	+	+
522	613	ATP synthase epsilon chain (EC 3.6.3.14)	57	+	+	+	+	+	+	+	+	+	+	+	+	+	+	+	+	+	+	+	+	+	+	+	+	+	+	+	+	+	+	+	+	+	+	+	+	+	+	+	+	+	+	+	+	+	+	+	+	+	+	+	+	+	+	+	+	+
523	614	Oligoendopeptidase F	57	+	+	+	+	+	+	+	+	+	+	+	+	+	+	+	+	+	+	+	+	+	+	+	+	+	+	+	+	+	+	+	+	+	+	+	+	+	+	+	+	+	+	+	+	+	+	+	+	+	+	+	+	+	+	+	+	+
524	622	hypothetical protein	57	+	+	+	+	+	+	+	+	+	+	+	+	+	+	+	+	+	+	+	+	+	+	+	+	+	+	+	+	+	+	+	+	+	+	+	+	+	+	+	+	+	+	+	+	+	+	+	+	+	+	+	+	+	+	+	+	+
525	624	Ribosomal small subunit pseudouridine synthase A (EC 4.2.1.70)	57	+	+	+	+	+	+	+	+	+	+	+	+	+	+	+	+	+	+	+	+	+	+	+	+	+	+	+	+	+	+	+	+	+	+	+	+	+	+	+	+	+	+	+	+	+	+	+	+	+	+	+	+	+	+	+	+	+
526	625	Cell division protein FtsH (EC 3.4.24.-)	57	+	+	+	+	+	+	+	+	+	+	+	+	+	+	+	+	+	+	+	+	+	+	+	+	+	+	+	+	+	+	+	+	+	+	+	+	+	+	+	+	+	+	+	+	+	+	+	+	+	+	+	+	+	+	+	+	+
527	626	Hydrolase (HAD superfamily)	57	+	+	+	+	+	+	+	+	+	+	+	+	+	+	+	+	+	+	+	+	+	+	+	+	+	+	+	+	+	+	+	+	+	+	+	+	+	+	+	+	+	+	+	+	+	+	+	+	+	+	+	+	+	+	+	+	+
528	627	Putative signal peptidase I (EC 3.4.21.89)	57	+	+	+	+	+	+	+	+	+	+	+	+	+	+	+	+	+	+	+	+	+	+	+	+	+	+	+	+	+	+	+	+	+	+	+	+	+	+	+	+	+	+	+	+	+	+	+	+	+	+	+	+	+	+	+	+	+
529	631	Putative deoxyribonuclease YcfH	57	+	+	+	+	+	+	+	+	+	+	+	+	+	+	+	+	+	+	+	+	+	+	+	+	+	+	+	+	+	+	+	+	+	+	+	+	+	+	+	+	+	+	+	+	+	+	+	+	+	+	+	+	+	+	+	+	+
530	632	Methionine aminopeptidase (EC 3.4.11.18)	57	+	+	+	+	+	+	+	+	+	+	+	+	+	+	+	+	+	+	+	+	+	+	+	+	+	+	+	+	+	+	+	+	+	+	+	+	+	+	+	+	+	+	+	+	+	+	+	+	+	+	+	+	+	+	+	+	+
531	633	hypothetical protein	57	+	+	+	+	+	+	+	+	+	+	+	+	+	+	+	+	+	+	+	+	+	+	+	+	+	+	+	+	+	+	+	+	+	+	+	+	+	+	+	+	+	+	+	+	+	+	+	+	+	+	+	+	+	+	+	+	+
532	641	SSU ribosomal protein S15p (S13e)	57	+	+	+	+	+	+	+	+	+	+	+	+	+	+	+	+	+	+	+	+	+	+	+	+	+	+	+	+	+	+	+	+	+	+	+	+	+	+	+	+	+	+	+	+	+	+	+	+	+	+	+	+	+	+	+	+	+
533	647	Putative deoxyribose-specific ABC transporter, permease protein	57	+	+	+	+	+	+	+	+	+	+	+	+	+	+	+	+	+	+	+	+	+	+	+	+	+	+	+	+	+	+	+	+	+	+	+	+	+	+	+	+	+	+	+	+	+	+	+	+	+	+	+	+	+	+	+	+	+
534	649	Dihydroorotate dehydrogenase, catalytic subunit (EC 1.3.3.1)	57	+	+	+	+	+	+	+	+	+	+	+	+	+	+	+	+	+	+	+	+	+	+	+	+	+	+	+	+	+	+	+	+	+	+	+	+	+	+	+	+	+	+	+	+	+	+	+	+	+	+	+	+	+	+	+	+	+
535	652	SSU ribosomal protein S2p (SAe)	57	+	+	+	+	+	+	+	+	+	+	+	+	+	+	+	+	+	+	+	+	+	+	+	+	+	+	+	+	+	+	+	+	+	+	+	+	+	+	+	+	+	+	+	+	+	+	+	+	+	+	+	+	+	+	+	+	+
536	654	Predicted nucleoside phosphatase; Acylphosphate phosphohydrolase (EC 3.6.1.7)	57	+	+	+	+	+	+	+	+	+	+	+	+	+	+	+	+	+	+	+	+	+	+	+	+	+	+	+	+	+	+	+	+	+	+	+	+	+	+	+	+	+	+	+	+	+	+	+	+	+	+	+	+	+	+	+	+	+
537	657	dTDP-4-dehydrorhamnose 3,5-epimerase (EC 5.1.3.13)	57	+	+	+	+	+	+	+	+	+	+	+	+	+	+	+	+	+	+	+	+	+	+	+	+	+	+	+	+	+	+	+	+	+	+	+	+	+	+	+	+	+	+	+	+	+	+	+	+	+	+	+	+	+	+	+	+	+
538	659	GMP synthase [glutamine-hydrolyzing], amidotransferase subunit (EC 6.3.5.2) / GMP synthase [glutamine-hydrolyzing], ATP pyrophosphatase subunit (EC 6.3.5.2)	57	+	+	+	+	+	+	+	+	+	+	+	+	+	+	+	+	+	+	+	+	+	+	+	+	+	+	+	+	+	+	+	+	+	+	+	+	+	+	+	+	+	+	+	+	+	+	+	+	+	+	+	+	+	+	+	+	+
539	660	TsaC protein (YrdC domain) required for threonylcarbamoyladenosine t(6)A37 modification in tRNA	57	+	+	+	+	+	+	+	+	+	+	+	+	+	+	+	+	+	+	+	+	+	+	+	+	+	+	+	+	+	+	+	+	+	+	+	+	+	+	+	+	+	+	+	+	+	+	+	+	+	+	+	+	+	+	+	+	+
540	662	ABC transporter ATP-binding protein	57	+	+	+	+	+	+	+	+	+	+	+	+	+	+	+	+	+	+	+	+	+	+	+	+	+	+	+	+	+	+	+	+	+	+	+	+	+	+	+	+	+	+	+	+	+	+	+	+	+	+	+	+	+	+	+	+	+
541	667	putative ATP-dependent Clp proteinase (ATP-binding subunit)	57	+	+	+	+	+	+	+	+	+	+	+	+	+	+	+	+	+	+	+	+	+	+	+	+	+	+	+	+	+	+	+	+	+	+	+	+	+	+	+	+	+	+	+	+	+	+	+	+	+	+	+	+	+	+	+	+	+
542	668	Phosphoribosylformylglycinamidine cyclo-ligase (EC 6.3.3.1)	57	+	+	+	+	+	+	+	+	+	+	+	+	+	+	+	+	+	+	+	+	+	+	+	+	+	+	+	+	+	+	+	+	+	+	+	+	+	+	+	+	+	+	+	+	+	+	+	+	+	+	+	+	+	+	+	+	+
543	669	3-dehydroquinate dehydratase I (EC 4.2.1.10)	57	+	+	+	+	+	+	+	+	+	+	+	+	+	+	+	+	+	+	+	+	+	+	+	+	+	+	+	+	+	+	+	+	+	+	+	+	+	+	+	+	+	+	+	+	+	+	+	+	+	+	+	+	+	+	+	+	+
544	671	dNTP triphosphohydrolase, putative	57	+	+	+	+	+	+	+	+	+	+	+	+	+	+	+	+	+	+	+	+	+	+	+	+	+	+	+	+	+	+	+	+	+	+	+	+	+	+	+	+	+	+	+	+	+	+	+	+	+	+	+	+	+	+	+	+	+
545	675	Phosphoribosylformylglycinamidine synthase, synthetase subunit (EC 6.3.5.3) / Phosphoribosylformylglycinamidine synthase, glutamine amidotransferase subunit (EC 6.3.5.3)	57	+	+	+	+	+	+	+	+	+	+	+	+	+	+	+	+	+	+	+	+	+	+	+	+	+	+	+	+	+	+	+	+	+	+	+	+	+	+	+	+	+	+	+	+	+	+	+	+	+	+	+	+	+	+	+	+	+
546	677	LSU ribosomal protein L32p	57	+	+	+	+	+	+	+	+	+	+	+	+	+	+	+	+	+	+	+	+	+	+	+	+	+	+	+	+	+	+	+	+	+	+	+	+	+	+	+	+	+	+	+	+	+	+	+	+	+	+	+	+	+	+	+	+	+
547	678	Acetoin dehydrogenase E1 component beta-subunit (EC 1.2.4.-)	57	+	+	+	+	+	+	+	+	+	+	+	+	+	+	+	+	+	+	+	+	+	+	+	+	+	+	+	+	+	+	+	+	+	+	+	+	+	+	+	+	+	+	+	+	+	+	+	+	+	+	+	+	+	+	+	+	+
548	680	Dihydrolipoamide acetyltransferase component (E2) of acetoin dehydrogenase complex (EC 2.3.1.-)	57	+	+	+	+	+	+	+	+	+	+	+	+	+	+	+	+	+	+	+	+	+	+	+	+	+	+	+	+	+	+	+	+	+	+	+	+	+	+	+	+	+	+	+	+	+	+	+	+	+	+	+	+	+	+	+	+	+
549	686	Tripeptide aminopeptidase (EC 3.4.11.4)	57	+	+	+	+	+	+	+	+	+	+	+	+	+	+	+	+	+	+	+	+	+	+	+	+	+	+	+	+	+	+	+	+	+	+	+	+	+	+	+	+	+	+	+	+	+	+	+	+	+	+	+	+	+	+	+	+	+
550	687	Dihydrolipoamide dehydrogenase of acetoin dehydrogenase (EC 1.8.1.4)	57	+	+	+	+	+	+	+	+	+	+	+	+	+	+	+	+	+	+	+	+	+	+	+	+	+	+	+	+	+	+	+	+	+	+	+	+	+	+	+	+	+	+	+	+	+	+	+	+	+	+	+	+	+	+	+	+	+
551	689	Deoxyribose-phosphate aldolase (EC 4.1.2.4)	57	+	+	+	+	+	+	+	+	+	+	+	+	+	+	+	+	+	+	+	+	+	+	+	+	+	+	+	+	+	+	+	+	+	+	+	+	+	+	+	+	+	+	+	+	+	+	+	+	+	+	+	+	+	+	+	+	+
552	692	Phosphopentomutase (EC 5.4.2.7)	57	+	+	+	+	+	+	+	+	+	+	+	+	+	+	+	+	+	+	+	+	+	+	+	+	+	+	+	+	+	+	+	+	+	+	+	+	+	+	+	+	+	+	+	+	+	+	+	+	+	+	+	+	+	+	+	+	+
553	693	Phosphoribosylaminoimidazole-succinocarboxamide synthase (EC 6.3.2.6)	57	+	+	+	+	+	+	+	+	+	+	+	+	+	+	+	+	+	+	+	+	+	+	+	+	+	+	+	+	+	+	+	+	+	+	+	+	+	+	+	+	+	+	+	+	+	+	+	+	+	+	+	+	+	+	+	+	+
554	694	Dihydroorotase (EC 3.5.2.3)	57	+	+	+	+	+	+	+	+	+	+	+	+	+	+	+	+	+	+	+	+	+	+	+	+	+	+	+	+	+	+	+	+	+	+	+	+	+	+	+	+	+	+	+	+	+	+	+	+	+	+	+	+	+	+	+	+	+
555	695	RNA-binding protein Jag	57	+	+	+	+	+	+	+	+	+	+	+	+	+	+	+	+	+	+	+	+	+	+	+	+	+	+	+	+	+	+	+	+	+	+	+	+	+	+	+	+	+	+	+	+	+	+	+	+	+	+	+	+	+	+	+	+	+
556	696	Aspartate carbamoyltransferase (EC 2.1.3.2)	57	+	+	+	+	+	+	+	+	+	+	+	+	+	+	+	+	+	+	+	+	+	+	+	+	+	+	+	+	+	+	+	+	+	+	+	+	+	+	+	+	+	+	+	+	+	+	+	+	+	+	+	+	+	+	+	+	+
557	697	ABC-type multidrug transport system, ATPase component	57	+	+	+	+	+	+	+	+	+	+	+	+	+	+	+	+	+	+	+	+	+	+	+	+	+	+	+	+	+	+	+	+	+	+	+	+	+	+	+	+	+	+	+	+	+	+	+	+	+	+	+	+	+	+	+	+	+
558	699	GTP cyclohydrolase I (EC 3.5.4.16) type 1	57	+	+	+	+	+	+	+	+	+	+	+	+	+	+	+	+	+	+	+	+	+	+	+	+	+	+	+	+	+	+	+	+	+	+	+	+	+	+	+	+	+	+	+	+	+	+	+	+	+	+	+	+	+	+	+	+	+
559	700	Amidophosphoribosyltransferase (EC 2.4.2.14)	57	+	+	+	+	+	+	+	+	+	+	+	+	+	+	+	+	+	+	+	+	+	+	+	+	+	+	+	+	+	+	+	+	+	+	+	+	+	+	+	+	+	+	+	+	+	+	+	+	+	+	+	+	+	+	+	+	+
560	701	hypothetical protein	57	+	+	+	+	+	+	+	+	+	+	+	+	+	+	+	+	+	+	+	+	+	+	+	+	+	+	+	+	+	+	+	+	+	+	+	+	+	+	+	+	+	+	+	+	+	+	+	+	+	+	+	+	+	+	+	+	+
561	702	Phosphoribosylglycinamide formyltransferase (EC 2.1.2.2)	57	+	+	+	+	+	+	+	+	+	+	+	+	+	+	+	+	+	+	+	+	+	+	+	+	+	+	+	+	+	+	+	+	+	+	+	+	+	+	+	+	+	+	+	+	+	+	+	+	+	+	+	+	+	+	+	+	+
562	703	ABC transporter ATP-binding protein	57	+	+	+	+	+	+	+	+	+	+	+	+	+	+	+	+	+	+	+	+	+	+	+	+	+	+	+	+	+	+	+	+	+	+	+	+	+	+	+	+	+	+	+	+	+	+	+	+	+	+	+	+	+	+	+	+	+
563	704	LSU ribosomal protein L34p	57	+	+	+	+	+	+	+	+	+	+	+	+	+	+	+	+	+	+	+	+	+	+	+	+	+	+	+	+	+	+	+	+	+	+	+	+	+	+	+	+	+	+	+	+	+	+	+	+	+	+	+	+	+	+	+	+	+
564	706	Rhodanese-like domain protein	57	+	+	+	+	+	+	+	+	+	+	+	+	+	+	+	+	+	+	+	+	+	+	+	+	+	+	+	+	+	+	+	+	+	+	+	+	+	+	+	+	+	+	+	+	+	+	+	+	+	+	+	+	+	+	+	+	+
565	707	Ribonucleotide reductase of class Ib (aerobic), alpha subunit (EC 1.17.4.1)	57	+	+	+	+	+	+	+	+	+	+	+	+	+	+	+	+	+	+	+	+	+	+	+	+	+	+	+	+	+	+	+	+	+	+	+	+	+	+	+	+	+	+	+	+	+	+	+	+	+	+	+	+	+	+	+	+	+
566	710	Aspartokinase (EC 2.7.2.4)	57	+	+	+	+	+	+	+	+	+	+	+	+	+	+	+	+	+	+	+	+	+	+	+	+	+	+	+	+	+	+	+	+	+	+	+	+	+	+	+	+	+	+	+	+	+	+	+	+	+	+	+	+	+	+	+	+	+
567	712	Phosphoglycerate mutase family 5	57	+	+	+	+	+	+	+	+	+	+	+	+	+	+	+	+	+	+	+	+	+	+	+	+	+	+	+	+	+	+	+	+	+	+	+	+	+	+	+	+	+	+	+	+	+	+	+	+	+	+	+	+	+	+	+	+	+
568	713	hypothetical protein	57	+	+	+	+	+	+	+	+	+	+	+	+	+	+	+	+	+	+	+	+	+	+	+	+	+	+	+	+	+	+	+	+	+	+	+	+	+	+	+	+	+	+	+	+	+	+	+	+	+	+	+	+	+	+	+	+	+
569	715	Phosphoribosylaminoimidazole carboxylase ATPase subunit (EC 4.1.1.21)	57	+	+	+	+	+	+	+	+	+	+	+	+	+	+	+	+	+	+	+	+	+	+	+	+	+	+	+	+	+	+	+	+	+	+	+	+	+	+	+	+	+	+	+	+	+	+	+	+	+	+	+	+	+	+	+	+	+

Table S5. Precence/absence of each HG among 57 *S. suis* strains

				1																13			25					28						104		94			76			53/54		Divergent *S. suis*																
Index[a]	ClusterID	Gene products	Members	98HAH33	05ZYH33	SC84	A7	SS12	P1/7	DAT273	NIAH11434	JS14	GZ1	BM407	S735	13730	MNCM07	NCTC 10446	14636	NCTC 10237	ST1	2651	89-1591	MNCM04	MNCM25	8074	D9	4961	ST3	MNCM43	DAT292	DAT260	NIAH11435	MNCM21	MNCM50	89-2479	DAT234	DAT290	93A	42A	NT77	6407	11538	8830	12814	92-1400	89-590	2726	2524	89-3576-3	4417	D12	10581	89-5259	88-5299A	R61	92-1191	14A	92-4172	22083
570	716	Aspartate-semialdehyde dehydrogenase (EC 1.2.1.11)	57	+	+	+	+	+	+	+	+	+	+	+	+	+	+	+	+	+	+	+	+	+	+	+	+	+	+	+	+	+	+	+	+	+	+	+	+	+	+	+	+	+	+	+	+	+	+	+	+	+	+	+	+	+
571	719	Homoserine dehydrogenase (EC 1.1.1.3)	57	+	+	+	+	+	+	+	+	+	+	+	+	+	+	+	+	+	+	+	+	+	+	+	+	+	+	+	+	+	+	+	+	+	+	+	+	+	+	+	+	+	+	+	+	+	+	+	+	+	+	+	+	+
572	722	dTDP-4-dehydrorhamnose reductase (EC 1.1.1.133)	57	+	+	+	+	+	+	+	+	+	+	+	+	+	+	+	+	+	+	+	+	+	+	+	+	+	+	+	+	+	+	+	+	+	+	+	+	+	+	+	+	+	+	+	+	+	+	+	+	+	+	+	+	+
573	724	Acetoin dehydrogenase E1 component alpha-subunit (EC 1.2.4.-)	57	+	+	+	+	+	+	+	+	+	+	+	+	+	+	+	+	+	+	+	+	+	+	+	+	+	+	+	+	+	+	+	+	+	+	+	+	+	+	+	+	+	+	+	+	+	+	+	+	+	+	+	+	+
574	728	Tributyrin esterase	57	+	+	+	+	+	+	+	+	+	+	+	+	+	+	+	+	+	+	+	+	+	+	+	+	+	+	+	+	+	+	+	+	+	+	+	+	+	+	+	+	+	+	+	+	+	+	+	+	+	+	+	+	+
575	730	tRNA and rRNA cytosine-C5-methylases	57	+	+	+	+	+	+	+	+	+	+	+	+	+	+	+	+	+	+	+	+	+	+	+	+	+	+	+	+	+	+	+	+	+	+	+	+	+	+	+	+	+	+	+	+	+	+	+	+	+	+	+	+	+
576	731	Ribosomal small subunit pseudouridine synthase A (EC 4.2.1.70)	57	+	+	+	+	+	+	+	+	+	+	+	+	+	+	+	+	+	+	+	+	+	+	+	+	+	+	+	+	+	+	+	+	+	+	+	+	+	+	+	+	+	+	+	+	+	+	+	+	+	+	+	+	+
577	734	L-serine dehydratase, beta subunit (EC 4.3.1.17)	57	+	+	+	+	+	+	+	+	+	+	+	+	+	+	+	+	+	+	+	+	+	+	+	+	+	+	+	+	+	+	+	+	+	+	+	+	+	+	+	+	+	+	+	+	+	+	+	+	+	+	+	+	+
578	735	Transcriptional regulator, GntR family	57	+	+	+	+	+	+	+	+	+	+	+	+	+	+	+	+	+	+	+	+	+	+	+	+	+	+	+	+	+	+	+	+	+	+	+	+	+	+	+	+	+	+	+	+	+	+	+	+	+	+	+	+	+
579	736	Glucose-1-phosphate adenylyltransferase (EC 2.7.7.27)	57	+	+	+	+	+	+	+	+	+	+	+	+	+	+	+	+	+	+	+	+	+	+	+	+	+	+	+	+	+	+	+	+	+	+	+	+	+	+	+	+	+	+	+	+	+	+	+	+	+	+	+	+	+
580	738	Peptide deformylase (EC 3.5.1.88)	57	+	+	+	+	+	+	+	+	+	+	+	+	+	+	+	+	+	+	+	+	+	+	+	+	+	+	+	+	+	+	+	+	+	+	+	+	+	+	+	+	+	+	+	+	+	+	+	+	+	+	+	+	+
581	740	ABC transporter, permease protein	57	+	+	+	+	+	+	+	+	+	+	+	+	+	+	+	+	+	+	+	+	+	+	+	+	+	+	+	+	+	+	+	+	+	+	+	+	+	+	+	+	+	+	+	+	+	+	+	+	+	+	+	+	+
582	742	Foldase protein prsA 1 precursor (EC 5.2.1.8)	57	+	+	+	+	+	+	+	+	+	+	+	+	+	+	+	+	+	+	+	+	+	+	+	+	+	+	+	+	+	+	+	+	+	+	+	+	+	+	+	+	+	+	+	+	+	+	+	+	+	+	+	+	+
583	743	hypothetical protein	57	+	+	+	+	+	+	+	+	+	+	+	+	+	+	+	+	+	+	+	+	+	+	+	+	+	+	+	+	+	+	+	+	+	+	+	+	+	+	+	+	+	+	+	+	+	+	+	+	+	+	+	+	+
584	746	PTS system	57	+	+	+	+	+	+	+	+	+	+	+	+	+	+	+	+	+	+	+	+	+	+	+	+	+	+	+	+	+	+	+	+	+	+	+	+	+	+	+	+	+	+	+	+	+	+	+	+	+	+	+	+	+
585	747	Non-specific DNA-binding protein Dps / Iron-binding ferritin-like antioxidant protein / Ferroxidase (EC 1.16.3.1)	57	+	+	+	+	+	+	+	+	+	+	+	+	+	+	+	+	+	+	+	+	+	+	+	+	+	+	+	+	+	+	+	+	+	+	+	+	+	+	+	+	+	+	+	+	+	+	+	+	+	+	+	+	+
586	749	Transcription regulator [contains diacylglycerol kinase catalytic domain]	57	+	+	+	+	+	+	+	+	+	+	+	+	+	+	+	+	+	+	+	+	+	+	+	+	+	+	+	+	+	+	+	+	+	+	+	+	+	+	+	+	+	+	+	+	+	+	+	+	+	+	+	+	+
587	750	High-affinity branched-chain amino acid transport system permease protein LivH (TC 3.A.1.4.1)	57	+	+	+	+	+	+	+	+	+	+	+	+	+	+	+	+	+	+	+	+	+	+	+	+	+	+	+	+	+	+	+	+	+	+	+	+	+	+	+	+	+	+	+	+	+	+	+	+	+	+	+	+	+
588	751	Anaerobic ribonucleoside-triphosphate reductase (EC 1.17.4.2)	57	+	+	+	+	+	+	+	+	+	+	+	+	+	+	+	+	+	+	+	+	+	+	+	+	+	+	+	+	+	+	+	+	+	+	+	+	+	+	+	+	+	+	+	+	+	+	+	+	+	+	+	+	+
589	752	Branched-chain amino acid transport ATP-binding protein LivG (TC 3.A.1.4.1)	57	+	+	+	+	+	+	+	+	+	+	+	+	+	+	+	+	+	+	+	+	+	+	+	+	+	+	+	+	+	+	+	+	+	+	+	+	+	+	+	+	+	+	+	+	+	+	+	+	+	+	+	+	+
590	754	Polysaccharide deacetylase	57	+	+	+	+	+	+	+	+	+	+	+	+	+	+	+	+	+	+	+	+	+	+	+	+	+	+	+	+	+	+	+	+	+	+	+	+	+	+	+	+	+	+	+	+	+	+	+	+	+	+	+	+	+
591	755	ABC transporter ATP-binding protein	57	+	+	+	+	+	+	+	+	+	+	+	+	+	+	+	+	+	+	+	+	+	+	+	+	+	+	+	+	+	+	+	+	+	+	+	+	+	+	+	+	+	+	+	+	+	+	+	+	+	+	+	+	+
592	756	Methionine ABC transporter ATP-binding protein	57	+	+	+	+	+	+	+	+	+	+	+	+	+	+	+	+	+	+	+	+	+	+	+	+	+	+	+	+	+	+	+	+	+	+	+	+	+	+	+	+	+	+	+	+	+	+	+	+	+	+	+	+	+
593	758	Pore forming protein ebsA	57	+	+	+	+	+	+	+	+	+	+	+	+	+	+	+	+	+	+	+	+	+	+	+	+	+	+	+	+	+	+	+	+	+	+	+	+	+	+	+	+	+	+	+	+	+	+	+	+	+	+	+	+	+
594	760	Undecaprenyl-phosphate N-acetylglucosaminyl 1-phosphate transferase (EC 2.7.8.-)	57	+	+	+	+	+	+	+	+	+	+	+	+	+	+	+	+	+	+	+	+	+	+	+	+	+	+	+	+	+	+	+	+	+	+	+	+	+	+	+	+	+	+	+	+	+	+	+	+	+	+	+	+	+
595	762	Branched-chain amino acid transport system permease protein LivM (TC 3.A.1.4.1)	57	+	+	+	+	+	+	+	+	+	+	+	+	+	+	+	+	+	+	+	+	+	+	+	+	+	+	+	+	+	+	+	+	+	+	+	+	+	+	+	+	+	+	+	+	+	+	+	+	+	+	+	+	+
596	765	Acetoin utilization CBS domain-containing protein; Inosine-5'-monophosphate dehydrogenase (EC 1.1.1.205)	57	+	+	+	+	+	+	+	+	+	+	+	+	+	+	+	+	+	+	+	+	+	+	+	+	+	+	+	+	+	+	+	+	+	+	+	+	+	+	+	+	+	+	+	+	+	+	+	+	+	+	+	+	+
597	766	Multidrug resistance efflux pump PmrA	57	+	+	+	+	+	+	+	+	+	+	+	+	+	+	+	+	+	+	+	+	+	+	+	+	+	+	+	+	+	+	+	+	+	+	+	+	+	+	+	+	+	+	+	+	+	+	+	+	+	+	+	+	+
598	767	LSU ribosomal protein L33p @ LSU ribosomal protein L33p, zinc-independent	57	+	+	+	+	+	+	+	+	+	+	+	+	+	+	+	+	+	+	+	+	+	+	+	+	+	+	+	+	+	+	+	+	+	+	+	+	+	+	+	+	+	+	+	+	+	+	+	+	+	+	+	+	+
599	768	Putative metallopeptidase (Zinc) SprT family	57	+	+	+	+	+	+	+	+	+	+	+	+	+	+	+	+	+	+	+	+	+	+	+	+	+	+	+	+	+	+	+	+	+	+	+	+	+	+	+	+	+	+	+	+	+	+	+	+	+	+	+	+	+
600	770	Cytidine deaminase (EC 3.5.4.5)	57	+	+	+	+	+	+	+	+	+	+	+	+	+	+	+	+	+	+	+	+	+	+	+	+	+	+	+	+	+	+	+	+	+	+	+	+	+	+	+	+	+	+	+	+	+	+	+	+	+	+	+	+	+
601	771	Laminin-binding surface protein	57	+	+	+	+	+	+	+	+	+	+	+	+	+	+	+	+	+	+	+	+	+	+	+	+	+	+	+	+	+	+	+	+	+	+	+	+	+	+	+	+	+	+	+	+	+	+	+	+	+	+	+	+	+
602	772	Homoserine kinase (EC 2.7.1.39)	57	+	+	+	+	+	+	+	+	+	+	+	+	+	+	+	+	+	+	+	+	+	+	+	+	+	+	+	+	+	+	+	+	+	+	+	+	+	+	+	+	+	+	+	+	+	+	+	+	+	+	+	+	+
603	775	Glucan 1,6-alpha-glucosidase (EC 3.2.1.70)	57	+	+	+	+	+	+	+	+	+	+	+	+	+	+	+	+	+	+	+	+	+	+	+	+	+	+	+	+	+	+	+	+	+	+	+	+	+	+	+	+	+	+	+	+	+	+	+	+	+	+	+	+	+
604	776	Branched-chain amino acid ABC transporter, amino acid-binding protein (TC 3.A.1.4.1)	57	+	+	+	+	+	+	+	+	+	+	+	+	+	+	+	+	+	+	+	+	+	+	+	+	+	+	+	+	+	+	+	+	+	+	+	+	+	+	+	+	+	+	+	+	+	+	+	+	+	+	+	+	+
605	778	Cytoplasmic copper homeostasis protein CutC	57	+	+	+	+	+	+	+	+	+	+	+	+	+	+	+	+	+	+	+	+	+	+	+	+	+	+	+	+	+	+	+	+	+	+	+	+	+	+	+	+	+	+	+	+	+	+	+	+	+	+	+	+	+
606	779	dTDP-glucose 4,6-dehydratase (EC 4.2.1.46)	57	+	+	+	+	+	+	+	+	+	+	+	+	+	+	+	+	+	+	+	+	+	+	+	+	+	+	+	+	+	+	+	+	+	+	+	+	+	+	+	+	+	+	+	+	+	+	+	+	+	+	+	+	+
607	781	ABC transporter ATP-binding protein; Branched-chain amino acid transport ATP-binding protein LivF (TC 3.A.1.4.1)	57	+	+	+	+	+	+	+	+	+	+	+	+	+	+	+	+	+	+	+	+	+	+	+	+	+	+	+	+	+	+	+	+	+	+	+	+	+	+	+	+	+	+	+	+	+	+	+	+	+	+	+	+	+
608	785	Carbamoyl-phosphate synthase large chain (EC 6.3.5.5)	57	+	+	+	+	+	+	+	+	+	+	+	+	+	+	+	+	+	+	+	+	+	+	+	+	+	+	+	+	+	+	+	+	+	+	+	+	+	+	+	+	+	+	+	+	+	+	+	+	+	+	+	+	+
609	786	Galactose-1-phosphate uridylyltransferase (EC 2.7.7.10)	57	+	+	+	+	+	+	+	+	+	+	+	+	+	+	+	+	+	+	+	+	+	+	+	+	+	+	+	+	+	+	+	+	+	+	+	+	+	+	+	+	+	+	+	+	+	+	+	+	+	+	+	+	+
610	789	Aromatic amino acid aminotransferase gamma (EC 2.6.1.57) @ N-acetyl-L,L-diaminopimelate aminotransferase (EC 2.6.1.-)	57	+	+	+	+	+	+	+	+	+	+	+	+	+	+	+	+	+	+	+	+	+	+	+	+	+	+	+	+	+	+	+	+	+	+	+	+	+	+	+	+	+	+	+	+	+	+	+	+	+	+	+	+	+
611	790	DegV family protein	57	+	+	+	+	+	+	+	+	+	+	+	+	+	+	+	+	+	+	+	+	+	+	+	+	+	+	+	+	+	+	+	+	+	+	+	+	+	+	+	+	+	+	+	+	+	+	+	+	+	+	+	+	+
612	791	FIG005986: HD family hydrolase	57	+	+	+	+	+	+	+	+	+	+	+	+	+	+	+	+	+	+	+	+	+	+	+	+	+	+	+	+	+	+	+	+	+	+	+	+	+	+	+	+	+	+	+	+	+	+	+	+	+	+	+	+	+
613	793	Galactose operon repressor, GalR-LacI family of transcriptional regulators	57	+	+	+	+	+	+	+	+	+	+	+	+	+	+	+	+	+	+	+	+	+	+	+	+	+	+	+	+	+	+	+	+	+	+	+	+	+	+	+	+	+	+	+	+	+	+	+	+	+	+	+	+	+
614	794	Peroxide stress regulator PerR, FUR family	57	+	+	+	+	+	+	+	+	+	+	+	+	+	+	+	+	+	+	+	+	+	+	+	+	+	+	+	+	+	+	+	+	+	+	+	+	+	+	+	+	+	+	+	+	+	+	+	+	+	+	+	+	+
615	795	Carbamate kinase (EC 2.7.2.2)	57	+	+	+	+	+	+	+	+	+	+	+	+	+	+	+	+	+	+	+	+	+	+	+	+	+	+	+	+	+	+	+	+	+	+	+	+	+	+	+	+	+	+	+	+	+	+	+	+	+	+	+	+	+
616	796	Methylated-DNA--protein-cysteine methyltransferase (EC 2.1.1.63)	57	+	+	+	+	+	+	+	+	+	+	+	+	+	+	+	+	+	+	+	+	+	+	+	+	+	+	+	+	+	+	+	+	+	+	+	+	+	+	+	+	+	+	+	+	+	+	+	+	+	+	+	+	+
617	797	Cationic amino acid transporter - APC Superfamily	57	+	+	+	+	+	+	+	+	+	+	+	+	+	+	+	+	+	+	+	+	+	+	+	+	+	+	+	+	+	+	+	+	+	+	+	+	+	+	+	+	+	+	+	+	+	+	+	+	+	+	+	+	+
618	804	Galactokinase (EC 2.7.1.6)	57	+	+	+	+	+	+	+	+	+	+	+	+	+	+	+	+	+	+	+	+	+	+	+	+	+	+	+	+	+	+	+	+	+	+	+	+	+	+	+	+	+	+	+	+	+	+	+	+	+	+	+	+	+
619	805	ABC transporter, ATP-binding/permease protein	57	+	+	+	+	+	+	+	+	+	+	+	+	+	+	+	+	+	+	+	+	+	+	+	+	+	+	+	+	+	+	+	+	+	+	+	+	+	+	+	+	+	+	+	+	+	+	+	+	+	+	+	+	+
620	806	Ferredoxin	57	+	+	+	+	+	+	+	+	+	+	+	+	+	+	+	+	+	+	+	+	+	+	+	+	+	+	+	+	+	+	+	+	+	+	+	+	+	+	+	+	+	+	+	+	+	+	+	+	+	+	+	+	+
621	808	ABC transporter, ATP-binding/permease protein	57	+	+	+	+	+	+	+	+	+	+	+	+	+	+	+	+	+	+	+	+	+	+	+	+	+	+	+	+	+	+	+	+	+	+	+	+	+	+	+	+	+	+	+	+	+	+	+	+	+	+	+	+	+
622	809	DegV family protein in cluster with TrmH family tRNA/rRNA methyltransferase YacO	57	+	+	+	+	+	+	+	+	+	+	+	+	+	+	+	+	+	+	+	+	+	+	+	+	+	+	+	+	+	+	+	+	+	+	+	+	+	+	+	+	+	+	+	+	+	+	+	+	+	+	+	+	+
623	813	Substrate-specific component MtsA of methionine-regulated ECF transporter	57	+	+	+	+	+	+	+	+	+	+	+	+	+	+	+	+	+	+	+	+	+	+	+	+	+	+	+	+	+	+	+	+	+	+	+	+	+	+	+	+	+	+	+	+	+	+	+	+	+	+	+	+	+
624	814	Transmembrane component MtsC of energizing module of methionine-regulated ECF transporter	57	+	+	+	+	+	+	+	+	+	+	+	+	+	+	+	+	+	+	+	+	+	+	+	+	+	+	+	+	+	+	+	+	+	+	+	+	+	+	+	+	+	+	+	+	+	+	+	+	+	+	+	+	+
625	817	Duplicated ATPase component MtsB of energizing module of methionine-regulated ECF transporter	57	+	+	+	+	+	+	+	+	+	+	+	+	+	+	+	+	+	+	+	+	+	+	+	+	+	+	+	+	+	+	+	+	+	+	+	+	+	+	+	+	+	+	+	+	+	+	+	+	+	+	+	+	+
626	818	Alcohol dehydrogenase (EC 1.1.1.1); Acetaldehyde dehydrogenase (EC 1.2.1.10)	57	+	+	+	+	+	+	+	+	+	+	+	+	+	+	+	+	+	+	+	+	+	+	+	+	+	+	+	+	+	+	+	+	+	+	+	+	+	+	+	+	+	+	+	+	+	+	+	+	+	+	+	+	+
627	819	Alpha-acetolactate decarboxylase (EC 4.1.1.5)	57	+	+	+	+	+	+	+	+	+	+	+	+	+	+	+	+	+	+	+	+	+	+	+	+	+	+	+	+	+	+	+	+	+	+	+	+	+	+	+	+	+	+	+	+	+	+	+	+	+	+	+	+	+
628	821	Glycosyltransferase LafB, responsible for the formation of Gal-Glc-DAG	57	+	+	+	+	+	+	+	+	+	+	+	+	+	+	+	+	+	+	+	+	+	+	+	+	+	+	+	+	+	+	+	+	+	+	+	+	+	+	+	+	+	+	+	+	+	+	+	+	+	+	+	+	+
629	823	Lactoylglutathione lyase (EC 4.4.1.5)	57	+	+	+	+	+	+	+	+	+	+	+	+	+	+	+	+	+	+	+	+	+	+	+	+	+	+	+	+	+	+	+	+	+	+	+	+	+	+	+	+	+	+	+	+	+	+	+	+	+	+	+	+	+
630	824	hypothetical protein	57	+	+	+	+	+	+	+	+	+	+	+	+	+	+	+	+	+	+	+	+	+	+	+	+	+	+	+	+	+	+	+	+	+	+	+	+	+	+	+	+	+	+	+	+	+	+	+	+	+	+	+	+	+
631	826	5'-nucleotidase YjjG (EC 3.1.3.5)	57	+	+	+	+	+	+	+	+	+	+	+	+	+	+	+	+	+	+	+	+	+	+	+	+	+	+	+	+	+	+	+	+	+	+	+	+	+	+	+	+	+	+	+	+	+	+	+	+	+	+	+	+	+
632	828	Acetyltransferase (EC 2.3.1.-)	57	+	+	+	+	+	+	+	+	+	+	+	+	+	+	+	+	+	+	+	+	+	+	+	+	+	+	+	+	+	+	+	+	+	+	+	+	+	+	+	+	+	+	+	+	+	+	+	+	+	+	+	+	+
633	829	ABC transporter membrane-spanning permease - glutamine transport	57	+	+	+	+	+	+	+	+	+	+	+	+	+	+	+	+	+	+	+	+	+	+	+	+	+	+	+	+	+	+	+	+	+	+	+	+	+	+	+	+	+	+	+	+	+	+	+	+	+	+	+	+	+
634	830	Phenylacetic acid degradation protein PaaD, thioesterase	57	+	+	+	+	+	+	+	+	+	+	+	+	+	+	+	+	+	+	+	+	+	+	+	+	+	+	+	+	+	+	+	+	+	+	+	+	+	+	+	+	+	+	+	+	+	+	+	+	+	+	+	+	+
635	831	Phosphoserine phosphatase (EC 3.1.3.3)	57	+	+	+	+	+	+	+	+	+	+	+	+	+	+	+	+	+	+	+	+	+	+	+	+	+	+	+	+	+	+	+	+	+	+	+	+	+	+	+	+	+	+	+	+	+	+	+	+	+	+	+	+	+
636	833	Hypothetical protein YaeR with similarity to glyoxylase family	57	+	+	+	+	+	+	+	+	+	+	+	+	+	+	+	+	+	+	+	+	+	+	+	+	+	+	+	+	+	+	+	+	+	+	+	+	+	+	+	+	+	+	+	+	+	+	+	+	+	+	+	+	+
637	836	Amino acid ABC transporter, amino acid-binding protein	57	+	+	+	+	+	+	+	+	+	+	+	+	+	+	+	+	+	+	+	+	+	+	+	+	+	+	+	+	+	+	+	+	+	+	+	+	+	+	+	+	+	+	+	+	+	+	+	+	+	+	+	+	+
638	837	Trehalose-6-phosphate hydrolase (EC 3.2.1.93)	57	+	+	+	+	+	+	+	+	+	+	+	+	+	+	+	+	+	+	+	+	+	+	+	+	+	+	+	+	+	+	+	+	+	+	+	+	+	+	+	+	+	+	+	+	+	+	+	+	+	+	+	+	+
639	838	uncharacterized protein	57	+	+	+	+	+	+	+	+	+	+	+	+	+	+	+	+	+	+	+	+	+	+	+	+	+	+	+	+	+	+	+	+	+	+	+	+	+	+	+	+	+	+	+	+	+	+	+	+	+	+	+	+	+
640	839	Valyl-tRNA synthetase (EC 6.1.1.9)	57	+	+	+	+	+	+	+	+	+	+	+	+	+	+	+	+	+	+	+	+	+	+	+	+	+	+	+	+	+	+	+	+	+	+	+	+	+	+	+	+	+	+	+	+	+	+	+	+	+	+	+	+	+
641	840	Cold shock protein CspA	57	+	+	+	+	+	+	+	+	+	+	+	+	+	+	+	+	+	+	+	+	+	+	+	+	+	+	+	+	+	+	+	+	+	+	+	+	+	+	+	+	+	+	+	+	+	+	+	+	+	+	+	+	+
642	841	Nucleoside diphosphate kinase (EC 2.7.4.6)	57	+	+	+	+	+	+	+	+	+	+	+	+	+	+	+	+	+	+	+	+	+	+	+	+	+	+	+	+	+	+	+	+	+	+	+	+	+	+	+	+	+	+	+	+	+	+	+	+	+	+	+	+	+
643	843	putative Zn-dependent protease	57	+	+	+	+	+	+	+	+	+	+	+	+	+	+	+	+	+	+	+	+	+	+	+	+	+	+	+	+	+	+	+	+	+	+	+	+	+	+	+	+	+	+	+	+	+	+	+	+	+	+	+	+	+
644	844	GtrA family protein; MesH protein	57	+	+	+	+	+	+	+	+	+	+	+	+	+	+	+	+	+	+	+	+	+	+	+	+	+	+	+	+	+	+	+	+	+	+	+	+	+	+	+	+	+	+	+	+	+	+	+	+	+	+	+	+	+
645	845	Maltose O-acetyltransferase (EC 2.3.1.79)	57	+	+	+	+	+	+	+	+	+	+	+	+	+	+	+	+	+	+	+	+	+	+	+	+	+	+	+	+	+	+	+	+	+	+	+	+	+	+	+	+	+	+	+	+	+	+	+	+	+	+	+	+	+
646	846	Two-component response regulator	57	+	+	+	+	+	+	+	+	+	+	+	+	+	+	+	+	+	+	+	+	+	+	+	+	+	+	+	+	+	+	+	+	+	+	+	+	+	+	+	+	+	+	+	+	+	+	+	+	+	+	+	+	+
647	847	L-proline glycine betaine ABC transport system permease protein ProV (TC 3.A.1.12.1)	57	+	+	+	+	+	+	+	+	+	+	+	+	+	+	+	+	+	+	+	+	+	+	+	+	+	+	+	+	+	+	+	+	+	+	+	+	+	+	+	+	+	+	+	+	+	+	+	+	+	+	+	+	+
648	848	COG2827: putative endonuclease containing a URI domain	57	+	+	+	+	+	+	+	+	+	+	+	+	+	+	+	+	+	+	+	+	+	+	+	+	+	+	+	+	+	+	+	+	+	+	+	+	+	+	+	+	+	+	+	+	+	+	+	+	+	+	+	+	+
649	850	oxidoreductase of aldo/keto reductase family, subgroup 1	57	+	+	+	+	+	+	+	+	+	+	+	+	+	+	+	+	+	+	+	+	+	+	+	+	+	+	+	+	+	+	+	+	+	+	+	+	+	+	+	+	+	+	+	+	+	+	+	+	+	+	+	+	+
650	851	N-acetyl-L,L-diaminopimelate deacetylase (EC 3.5.1.47)	57	+	+	+	+	+	+	+	+	+	+	+	+	+	+	+	+	+	+	+	+	+	+	+	+	+	+	+	+	+	+	+	+	+	+	+	+	+	+	+	+	+	+	+	+	+	+	+	+	+	+	+	+	+
651	852	Tyrosine-protein phosphatase CpsB (EC 3.1.3.48)	57	+	+	+	+	+	+	+	+	+	+	+	+	+	+	+	+	+	+	+	+	+	+	+	+	+	+	+	+	+	+	+	+	+	+	+	+	+	+	+	+	+	+	+	+	+	+	+	+	+	+	+	+	+
652	853	Aspartate aminotransferase (EC 2.6.1.1)	57	+	+	+	+	+	+	+	+	+	+	+	+	+	+	+	+	+	+	+	+	+	+	+	+	+	+	+	+	+	+	+	+	+	+	+	+	+	+	+	+	+	+	+	+	+	+	+	+	+	+	+	+	+
653	854	PTS system, trehalose-specific IIB component (EC 2.7.1.69) / PTS system, trehalose-specific IIC component (EC 2.7.1.69)	57	+	+	+	+	+	+	+	+	+	+	+	+	+	+	+	+	+	+	+	+	+	+	+	+	+	+	+	+	+	+	+	+	+	+	+	+	+	+	+	+	+	+	+	+	+	+	+	+	+	+	+	+	+
654	855	Dihydrodipicolinate reductase (EC 1.3.1.26)	57	+	+	+	+	+	+	+	+	+	+	+	+	+	+	+	+	+	+	+	+	+	+	+	+	+	+	+	+	+	+	+	+	+	+	+	+	+	+	+	+	+	+	+	+	+	+	+	+	+	+	+	+	+
655	856	L-proline glycine betaine binding ABC transporter protein ProX (TC 3.A.1.12.1) / Osmotic adaptation	57	+	+	+	+	+	+	+	+	+	+	+	+	+	+	+	+	+	+	+	+	+	+	+	+	+	+	+	+	+	+	+	+	+	+	+	+	+	+	+	+	+	+	+	+	+	+	+	+	+	+	+	+	+
656	857	Trehalose operon transcriptional repressor	57	+	+	+	+	+	+	+	+	+	+	+	+	+	+	+	+	+	+	+	+	+	+	+	+	+	+	+	+	+	+	+	+	+	+	+	+	+	+	+	+	+	+	+	+	+	+	+	+	+	+	+	+	+
657	858	Thiol peroxidase, Tpx-type (EC 1.11.1.15)	57	+	+	+	+	+	+	+	+	+	+	+	+	+	+	+	+	+	+	+	+	+	+	+	+	+	+	+	+	+	+	+	+	+	+	+	+	+	+	+	+	+	+	+	+	+	+	+	+	+	+	+	+	+
658	860	Dihydrodipicolinate synthase (EC 4.2.1.52)	57	+	+	+	+	+	+	+	+	+	+	+	+	+	+	+	+	+	+	+	+	+	+	+	+	+	+	+	+	+	+	+	+	+	+	+	+	+	+	+	+	+	+	+	+	+	+	+	+	+	+	+	+	+
659	861	ABC transporter permease protein	57	+	+	+	+	+	+	+	+	+	+	+	+	+	+	+	+	+	+	+	+	+	+	+	+	+	+	+	+	+	+	+	+	+	+	+	+	+	+	+	+	+	+	+	+	+	+	+	+	+	+	+	+	+
660	862	FIG006988: Lipase/Acylhydrolase with GDSL-like motif	57	+	+	+	+	+	+	+	+	+	+	+	+	+	+	+	+	+	+	+	+	+	+	+	+	+	+	+	+	+	+	+	+	+	+	+	+	+	+	+	+	+	+	+	+	+	+	+	+	+	+	+	+	+
661	863	Aminopeptidase S (Leu, Val, Phe, Tyr preference) (EC 3.4.11.24)	57	+	+	+	+	+	+	+	+	+	+	+	+	+	+	+	+	+	+	+	+	+	+	+	+	+	+	+	+	+	+	+	+	+	+	+	+	+	+	+	+	+	+	+	+	+	+	+	+	+	+	+	+	+
662	864	1,3-propanediol dehydrogenase (EC 1.1.1.202)	57	+	+	+	+	+	+	+	+	+	+	+	+	+	+	+	+	+	+	+	+	+	+	+	+	+	+	+	+	+	+	+	+	+	+	+	+	+	+	+	+	+	+	+	+	+	+	+	+	+	+	+	+	+
663	867	Glutamine amidotransferase, class I	57	+	+	+	+	+	+	+	+	+	+	+	+	+	+	+	+	+	+	+	+	+	+	+	+	+	+	+	+	+	+	+	+	+	+	+	+	+	+	+	+	+	+	+	+	+	+	+	+	+	+	+	+	+
664	868	Prephenate dehydratase (EC 4.2.1.51)	57	+	+	+	+	+	+	+	+	+	+	+	+	+	+	+	+	+	+	+	+	+	+	+	+	+	+	+	+	+	+	+	+	+	+	+	+	+	+	+	+	+	+	+	+	+	+	+	+	+	+	+	+	+
665	870	Arginine pathway regulatory protein ArgR, repressor of arg regulon	57	+	+	+	+	+	+	+	+	+	+	+	+	+	+	+	+	+	+	+	+	+	+	+	+	+	+	+	+	+	+	+	+	+	+	+	+	+	+	+	+	+	+	+	+	+	+	+	+	+	+	+	+	+
666	871	Integral membrane protein	57	+	+	+	+	+	+	+	+	+	+	+	+	+	+	+	+	+	+	+	+	+	+	+	+	+	+	+	+	+	+	+	+	+	+	+	+	+	+	+	+	+	+	+	+	+	+	+	+	+	+	+	+	+
667	873	UDP-glucose 4-epimerase (EC 5.1.3.2)	57	+	+	+	+	+	+	+	+	+	+	+	+	+	+	+	+	+	+	+	+	+	+	+	+	+	+	+	+	+	+	+	+	+	+	+	+	+	+	+	+	+	+	+	+	+	+	+	+	+	+	+	+	+
668	874	Tyrosine-protein kinase EpsD (EC 2.7.10.2)	57	+	+	+	+	+	+	+	+	+	+	+	+	+	+	+	+	+	+	+	+	+	+	+	+	+	+	+	+	+	+	+	+	+	+	+	+	+	+	+	+	+	+	+	+	+	+	+	+	+	+	+	+	+
669	875	Rhodanese domain protein UPF0176, Firmicutes subgroup	57	+	+	+	+	+	+	+	+	+	+	+	+	+	+	+	+	+	+	+	+	+	+	+	+	+	+	+	+	+	+	+	+	+	+	+	+	+	+	+	+	+	+	+	+	+	+	+	+	+	+	+	+	+
670	876	hypothetical protein	57	+	+	+	+	+	+	+	+	+	+	+	+	+	+	+	+	+	+	+	+	+	+	+	+	+	+	+	+	+	+	+	+	+	+	+	+	+	+	+	+	+	+	+	+	+	+	+	+	+	+	+	+	+
671	877	3-dehydroquinate synthase (EC 4.2.3.4)	57	+	+	+	+	+	+	+	+	+	+	+	+	+	+	+	+	+	+	+	+	+	+	+	+	+	+	+	+	+	+	+	+	+	+	+	+	+	+	+	+	+	+	+	+	+	+	+	+	+	+	+	+	+
672	878	6-phosphogluconolactonase (EC 3.1.1.31)	57	+	+	+	+	+	+	+	+	+	+	+	+	+	+	+	+	+	+	+	+	+	+	+	+	+	+	+	+	+	+	+	+	+	+	+	+	+	+	+	+	+	+	+	+	+	+	+	+	+	+	+	+	+
673	880	Glutamate transport permease protein	57	+	+	+	+	+	+	+	+	+	+	+	+	+	+	+	+	+	+	+	+	+	+	+	+	+	+	+	+	+	+	+	+	+	+	+	+	+	+	+	+	+	+	+	+	+	+	+	+	+	+	+	+	+
674	883	Dicarboxylate/amino acid:cation (Na+ or H+) symporter	57	+	+	+	+	+	+	+	+	+	+	+	+	+	+	+	+	+	+	+	+	+	+	+	+	+	+	+	+	+	+	+	+	+	+	+	+	+	+	+	+	+	+	+	+	+	+	+	+	+	+	+	+	+
675	884	Glutamate transport membrane-spanning protein	57	+	+	+	+	+	+	+	+	+	+	+	+	+	+	+	+	+	+	+	+	+	+	+	+	+	+	+	+	+	+	+	+	+	+	+	+	+	+	+	+	+	+	+	+	+	+	+	+	+	+	+	+	+
676	886	membrane protein, putative	57	+	+	+	+	+	+	+	+	+	+	+	+	+	+	+	+	+	+	+	+	+	+	+	+	+	+	+	+	+	+	+	+	+	+	+	+	+	+	+	+	+	+	+	+	+	+	+	+	+	+	+	+	+
677	887	Secreted antigen GbpB/SagA/PcsB, putative peptidoglycan hydrolase	57	+	+	+	+	+	+	+	+	+	+	+	+	+	+	+	+	+	+	+	+	+	+	+	+	+	+	+	+	+	+	+	+	+	+	+	+	+	+	+	+	+	+	+	+	+	+	+	+	+	+	+	+	+
678	888	Branched-chain amino acid transport system carrier protein	57	+	+	+	+	+	+	+	+	+	+	+	+	+	+	+	+	+	+	+	+	+	+	+	+	+	+	+	+	+	+	+	+	+	+	+	+	+	+	+	+	+	+	+	+	+	+	+	+	+	+	+	+	+
679	890	Pullulanase (EC 3.2.1.41)	57	+	+	+	+	+	+	+	+	+	+	+	+	+	+	+	+	+	+	+	+	+	+	+	+	+	+	+	+	+	+	+	+	+	+	+	+	+	+	+	+	+	+	+	+	+	+	+	+	+	+	+	+	+
680	891	Galactosamine-6-phosphate isomerase (EC 5.3.1.-)	57	+	+	+	+	+	+	+	+	+	+	+	+	+	+	+	+	+	+	+	+	+	+	+	+	+	+	+	+	+	+	+	+	+	+	+	+	+	+	+	+	+	+	+	+	+	+	+	+	+	+	+	+	+
681	894	Rrf2-linked NADH-flavin reductase	57	+	+	+	+	+	+	+	+	+	+	+	+	+	+	+	+	+	+	+	+	+	+	+	+	+	+	+	+	+	+	+	+	+	+	+	+	+	+	+	+	+	+	+	+	+	+	+	+	+	+	+	+	+
682	897	Glutamine ABC transporter, periplasmic glutamine-binding protein (TC 3.A.1.3.2)	57	+	+	+	+	+	+	+	+	+	+	+	+	+	+	+	+	+	+	+	+	+	+	+	+	+	+	+	+	+	+	+	+	+	+	+	+	+	+	+	+	+	+	+	+	+	+	+	+	+	+	+	+	+
683	898	Homoserine O-succinyltransferase (EC 2.3.1.46)	57	+	+	+	+	+	+	+	+	+	+	+	+	+	+	+	+	+	+	+	+	+	+	+	+	+	+	+	+	+	+	+	+	+	+	+	+	+	+	+	+	+	+	+	+	+	+	+	+	+	+	+	+	+
684	900	Oxidoreductase, aldo/keto reductase family	57	+	+	+	+	+	+	+	+	+	+	+	+	+	+	+	+	+	+	+	+	+	+	+	+	+	+	+	+	+	+	+	+	+	+	+	+	+	+	+	+	+	+	+	+	+	+	+	+	+	+	+	+	+
685	902	Shikimate/quinate 5-dehydrogenase I beta (EC 1.1.1.282)	57	+	+	+	+	+	+	+	+	+	+	+	+	+	+	+	+	+	+	+	+	+	+	+	+	+	+	+	+	+	+	+	+	+	+	+	+	+	+	+	+	+	+	+	+	+	+	+	+	+	+	+	+	+
686	904	Beta-galactosidase 3	57	+	+	+	+	+	+	+	+	+	+	+	+	+	+	+	+	+	+	+	+	+	+	+	+	+	+	+	+	+	+	+	+	+	+	+	+	+	+	+	+	+	+	+	+	+	+	+	+	+	+	+	+	+
687	905	Endoglucanase M (EC 3.2.1.4)	57	+	+	+	+	+	+	+	+	+	+	+	+	+	+	+	+	+	+	+	+	+	+	+	+	+	+	+	+	+	+	+	+	+	+	+	+	+	+	+	+	+	+	+	+	+	+	+	+	+	+	+	+	+
688	911	ABC-type Fe3+-siderophore transport system, permease 2 component	57	+	+	+	+	+	+	+	+	+	+	+	+	+	+	+	+	+	+	+	+	+	+	+	+	+	+	+	+	+	+	+	+	+	+	+	+	+	+	+	+	+	+	+	+	+	+	+	+	+	+	+	+	+
689	912	ATP-dependent Zn protease	57	+	+	+	+	+	+	+	+	+	+	+	+	+	+	+	+	+	+	+	+	+	+	+	+	+	+	+	+	+	+	+	+	+	+	+	+	+	+	+	+	+	+	+	+	+	+	+	+	+	+	+	+	+
690	914	Phosphoserine aminotransferase (EC 2.6.1.52)	57	+	+	+	+	+	+	+	+	+	+	+	+	+	+	+	+	+	+	+	+	+	+	+	+	+	+	+	+	+	+	+	+	+	+	+	+	+	+	+	+	+	+	+	+	+	+	+	+	+	+	+	+	+
691	917	Ferrous iron transport protein A	57	+	+	+	+	+	+	+	+	+	+	+	+	+	+	+	+	+	+	+	+	+	+	+	+	+	+	+	+	+	+	+	+	+	+	+	+	+	+	+	+	+	+	+	+	+	+	+	+	+	+	+	+	+
692	918	ABC-type Fe3+-siderophore transport system, permease component	57	+	+	+	+	+	+	+	+	+	+	+	+	+	+	+	+	+	+	+	+	+	+	+	+	+	+	+	+	+	+	+	+	+	+	+	+	+	+	+	+	+	+	+	+	+	+	+	+	+	+	+	+	+
693	919	Ferrichrome transport ATP-binding protein FhuC (TC 3.A.1.14.3)	57	+	+	+	+	+	+	+	+	+	+	+	+	+	+	+	+	+	+	+	+	+	+	+	+	+	+	+	+	+	+	+	+	+	+	+	+	+	+	+	+	+	+	+	+	+	+	+	+	+	+	+	+	+
694	920	Glutathione peroxidase (EC 1.11.1.9)	57	+	+	+	+	+	+	+	+	+	+	+	+	+	+	+	+	+	+	+	+	+	+	+	+	+	+	+	+	+	+	+	+	+	+	+	+	+	+	+	+	+	+	+	+	+	+	+	+	+	+	+	+	+
695	921	membrane protein	57	+	+	+	+	+	+	+	+	+	+	+	+	+	+	+	+	+	+	+	+	+	+	+	+	+	+	+	+	+	+	+	+	+	+	+	+	+	+	+	+	+	+	+	+	+	+	+	+	+	+	+	+	+
696	922	Glutathione biosynthesis bifunctional protein gshF (EC 6.3.2.2)(EC 6.3.2.3)	57	+	+	+	+	+	+	+	+	+	+	+	+	+	+	+	+	+	+	+	+	+	+	+	+	+	+	+	+	+	+	+	+	+	+	+	+	+	+	+	+	+	+	+	+	+	+	+	+	+	+	+	+	+
697	927	Iron-sulfur cluster-binding protein	57	+	+	+	+	+	+	+	+	+	+	+	+	+	+	+	+	+	+	+	+	+	+	+	+	+	+	+	+	+	+	+	+	+	+	+	+	+	+	+	+	+	+	+	+	+	+	+	+	+	+	+	+	+
698	928	Acetolactate synthase small subunit (EC 2.2.1.6)	57	+	+	+	+	+	+	+	+	+	+	+	+	+	+	+	+	+	+	+	+	+	+	+	+	+	+	+	+	+	+	+	+	+	+	+	+	+	+	+	+	+	+	+	+	+	+	+	+	+	+	+	+	+
699	930	Arginine deiminase (EC 3.5.3.6)	57	+	+	+	+	+	+	+	+	+	+	+	+	+	+	+	+	+	+	+	+	+	+	+	+	+	+	+	+	+	+	+	+	+	+	+	+	+	+	+	+	+	+	+	+	+	+	+	+	+	+	+	+	+
700	931	Two-component system histidine kinase	57	+	+	+	+	+	+	+	+	+	+	+	+	+	+	+	+	+	+	+	+	+	+	+	+	+	+	+	+	+	+	+	+	+	+	+	+	+	+	+	+	+	+	+	+	+	+	+	+	+	+	+	+	+
701	933	Methionine ABC transporter ATP-binding protein	57	+	+	+	+	+	+	+	+	+	+	+	+	+	+	+	+	+	+	+	+	+	+	+	+	+	+	+	+	+	+	+	+	+	+	+	+	+	+	+	+	+	+	+	+	+	+	+	+	+	+	+	+	+
702	934	PTS system, mannose-specific IIB component (EC 2.7.1.69) / PTS system, mannose-specific IIA component (EC 2.7.1.69)	57	+	+	+	+	+	+	+	+	+	+	+	+	+	+	+	+	+	+	+	+	+	+	+	+	+	+	+	+	+	+	+	+	+	+	+	+	+	+	+	+	+	+	+	+	+	+	+	+	+	+	+	+	+
703	935	Ketol-acid reductoisomerase (EC 1.1.1.86)	57	+	+	+	+	+	+	+	+	+	+	+	+	+	+	+	+	+	+	+	+	+	+	+	+	+	+	+	+	+	+	+	+	+	+	+	+	+	+	+	+	+	+	+	+	+	+	+	+	+	+	+	+	+
704	936	DNA-binding response regulator	57	+	+	+	+	+	+	+	+	+	+	+	+	+	+	+	+	+	+	+	+	+	+	+	+	+	+	+	+	+	+	+	+	+	+	+	+	+	+	+	+	+	+	+	+	+	+	+	+	+	+	+	+	+
705	937	PTS system, mannose-specific IIC component (EC 2.7.1.69)	57	+	+	+	+	+	+	+	+	+	+	+	+	+	+	+	+	+	+	+	+	+	+	+	+	+	+	+	+	+	+	+	+	+	+	+	+	+	+	+	+	+	+	+	+	+	+	+	+	+	+	+	+	+
706	938	Acetolactate synthase large subunit (EC 2.2.1.6)	57	+	+	+	+	+	+	+	+	+	+	+	+	+	+	+	+	+	+	+	+	+	+	+	+	+	+	+	+	+	+	+	+	+	+	+	+	+	+	+	+	+	+	+	+	+	+	+	+	+	+	+	+	+
707	939	PTS system, mannose-specific IIB component (EC 2.7.1.69)	57	+	+	+	+	+	+	+	+	+	+	+	+	+	+	+	+	+	+	+	+	+	+	+	+	+	+	+	+	+	+	+	+	+	+	+	+	+	+	+	+	+	+	+	+	+	+	+	+	+	+	+	+	+
708	941	PTS system, N-acetylgalactosamine-specific IID component (EC 2.7.1.69)	57	+	+	+	+	+	+	+	+	+	+	+	+	+	+	+	+	+	+	+	+	+	+	+	+	+	+	+	+	+	+	+	+	+	+	+	+	+	+	+	+	+	+	+	+	+	+	+	+	+	+	+	+	+
709	942	Threonine dehydratase (EC 4.3.1.19)	57	+	+	+	+	+	+	+	+	+	+	+	+	+	+	+	+	+	+	+	+	+	+	+	+	+	+	+	+	+	+	+	+	+	+	+	+	+	+	+	+	+	+	+	+	+	+	+	+	+	+	+	+	+
710	946	hypothetical protein	57	+	+	+	+	+	+	+	+	+	+	+	+	+	+	+	+	+	+	+	+	+	+	+	+	+	+	+	+	+	+	+	+	+	+	+	+	+	+	+	+	+	+	+	+	+	+	+	+	+	+	+	+	+

Table S5. Precence/absence of each HG among 57 *S. suis* strains

				1																13			25					28						104		94			76			53/54		Divergent *S. suis*																
Index[a]	ClusterID	Gene products	Members	98HAH33	05ZYH33	SC84	A7	SS12	P1/7	DAT273	NIAH11434	JS14	GZ1	BM407	S735	13730	MNCM07	NCTC 10446	14636	NCTC 10237	ST1	2651	89-1591	MNCM04	MNCM25	8074	D9	4961	ST3	MNCM43	DAT292	DAT260	NIAH11435	MNCM21	MNCM50	89-2479	DAT234	DAT290	93A	42A	NT77	6407	11538	8830	12814	92-1400	89-590	2726	2524	89-3576-3	4417	D12	10581	89-5259	88-5299A	R61	92-1191	14A	92-4172	22083
711	949	Tellurite resistance protein	57	+	+	+	+	+	+	+	+	+	+	+	+	+	+	+	+	+	+	+	+	+	+	+	+	+	+	+	+	+	+	+	+	+	+	+	+	+	+	+	+	+	+	+	+	+	+	+	+	+	+	+	+	+	+	+	+	+
712	950	Endoribonuclease L-PSP	57	+	+	+	+	+	+	+	+	+	+	+	+	+	+	+	+	+	+	+	+	+	+	+	+	+	+	+	+	+	+	+	+	+	+	+	+	+	+	+	+	+	+	+	+	+	+	+	+	+	+	+	+	+	+	+	+	+
713	952	Predicted divalent heavy-metal cations transporter	57	+	+	+	+	+	+	+	+	+	+	+	+	+	+	+	+	+	+	+	+	+	+	+	+	+	+	+	+	+	+	+	+	+	+	+	+	+	+	+	+	+	+	+	+	+	+	+	+	+	+	+	+	+	+	+	+	+
714	955	Ferrous iron transport protein B	57	+	+	+	+	+	+	+	+	+	+	+	+	+	+	+	+	+	+	+	+	+	+	+	+	+	+	+	+	+	+	+	+	+	+	+	+	+	+	+	+	+	+	+	+	+	+	+	+	+	+	+	+	+	+	+	+	+
715	956	2-isopropylmalate synthase (EC 2.3.3.13)	57	+	+	+	+	+	+	+	+	+	+	+	+	+	+	+	+	+	+	+	+	+	+	+	+	+	+	+	+	+	+	+	+	+	+	+	+	+	+	+	+	+	+	+	+	+	+	+	+	+	+	+	+	+	+	+	+	+
716	959	Argininosuccinate lyase (EC 4.3.2.1)	57	+	+	+	+	+	+	+	+	+	+	+	+	+	+	+	+	+	+	+	+	+	+	+	+	+	+	+	+	+	+	+	+	+	+	+	+	+	+	+	+	+	+	+	+	+	+	+	+	+	+	+	+	+	+	+	+	+
717	960	Cell division protein YlmG/Ycf19 (putative), YggT family	57	+	+	+	+	+	+	+	+	+	+	+	+	+	+	+	+	+	+	+	+	+	+	+	+	+	+	+	+	+	+	+	+	+	+	+	+	+	+	+	+	+	+	+	+	+	+	+	+	+	+	+	+	+	+	+	+	+
718	961	Glutathione S-transferase, Streptococcal type (EC 2.5.1.18)	57	+	+	+	+	+	+	+	+	+	+	+	+	+	+	+	+	+	+	+	+	+	+	+	+	+	+	+	+	+	+	+	+	+	+	+	+	+	+	+	+	+	+	+	+	+	+	+	+	+	+	+	+	+	+	+	+	+
719	963	3-isopropylmalate dehydratase small subunit (EC 4.2.1.33)	57	+	+	+	+	+	+	+	+	+	+	+	+	+	+	+	+	+	+	+	+	+	+	+	+	+	+	+	+	+	+	+	+	+	+	+	+	+	+	+	+	+	+	+	+	+	+	+	+	+	+	+	+	+	+	+	+	+	+	+
720	965	D-3-phosphoglycerate dehydrogenase (EC 1.1.1.95)	57	+	+	+	+	+	+	+	+	+	+	+	+	+	+	+	+	+	+	+	+	+	+	+	+	+	+	+	+	+	+	+	+	+	+	+	+	+	+	+	+	+	+	+	+	+	+	+	+	+	+	+	+	+	+	+	+	+	+	+
721	967	Substrate-specific component BioY of biotin ECF transporter	57	+	+	+	+	+	+	+	+	+	+	+	+	+	+	+	+	+	+	+	+	+	+	+	+	+	+	+	+	+	+	+	+	+	+	+	+	+	+	+	+	+	+	+	+	+	+	+	+	+	+	+	+	+	+	+	+	+
722	968	Adenosine deaminase (EC 3.5.4.4)	57	+	+	+	+	+	+	+	+	+	+	+	+	+	+	+	+	+	+	+	+	+	+	+	+	+	+	+	+	+	+	+	+	+	+	+	+	+	+	+	+	+	+	+	+	+	+	+	+	+	+	+	+	+	+	+	+	+
723	971	Arginine/ornithine antiporter ArcD	57	+	+	+	+	+	+	+	+	+	+	+	+	+	+	+	+	+	+	+	+	+	+	+	+	+	+	+	+	+	+	+	+	+	+	+	+	+	+	+	+	+	+	+	+	+	+	+	+	+	+	+	+	+	+	+	+	+
724	972	1,3-propanediol dehydrogenase (EC 1.1.1.202)	57	+	+	+	+	+	+	+	+	+	+	+	+	+	+	+	+	+	+	+	+	+	+	+	+	+	+	+	+	+	+	+	+	+	+	+	+	+	+	+	+	+	+	+	+	+	+	+	+	+	+	+	+	+	+	+	+	+
725	976	Aggregation promoting factor	57	+	+	+	+	+	+	+	+	+	+	+	+	+	+	+	+	+	+	+	+	+	+	+	+	+	+	+	+	+	+	+	+	+	+	+	+	+	+	+	+	+	+	+	+	+	+	+	+	+	+	+	+	+	+	+	+	+
726	977	ThiJ/PfpI family protein	57	+	+	+	+	+	+	+	+	+	+	+	+	+	+	+	+	+	+	+	+	+	+	+	+	+	+	+	+	+	+	+	+	+	+	+	+	+	+	+	+	+	+	+	+	+	+	+	+	+	+	+	+	+	+	+	+	+
727	979	3-isopropylmalate dehydrogenase (EC 1.1.1.85)	57	+	+	+	+	+	+	+	+	+	+	+	+	+	+	+	+	+	+	+	+	+	+	+	+	+	+	+	+	+	+	+	+	+	+	+	+	+	+	+	+	+	+	+	+	+	+	+	+	+	+	+	+	+	+	+	+	+
728	982	Aspartate aminotransferase (EC 2.6.1.1)	57	+	+	+	+	+	+	+	+	+	+	+	+	+	+	+	+	+	+	+	+	+	+	+	+	+	+	+	+	+	+	+	+	+	+	+	+	+	+	+	+	+	+	+	+	+	+	+	+	+	+	+	+	+	+	+	+	+
729	983	Lipid A export ATP-binding/permease protein MsbA	57	+	+	+	+	+	+	+	+	+	+	+	+	+	+	+	+	+	+	+	+	+	+	+	+	+	+	+	+	+	+	+	+	+	+	+	+	+	+	+	+	+	+	+	+	+	+	+	+	+	+	+	+	+	+	+	+	+
730	984	Late competence protein ComGF, access of DNA to ComEA, FIG012620	57	+	+	+	+	+	+	+	+	+	+	+	+	+	+	+	+	+	+	+	+	+	+	+	+	+	+	+	+	+	+	+	+	+	+	+	+	+	+	+	+	+	+	+	+	+	+	+	+	+	+	+	+	+	+	+	+	+
731	986	Integral membrane protein	57	+	+	+	+	+	+	+	+	+	+	+	+	+	+	+	+	+	+	+	+	+	+	+	+	+	+	+	+	+	+	+	+	+	+	+	+	+	+	+	+	+	+	+	+	+	+	+	+	+	+	+	+	+	+	+	+	+
732	989	Lipid A export ATP-binding/permease protein MsbA	57	+	+	+	+	+	+	+	+	+	+	+	+	+	+	+	+	+	+	+	+	+	+	+	+	+	+	+	+	+	+	+	+	+	+	+	+	+	+	+	+	+	+	+	+	+	+	+	+	+	+	+	+	+	+	+	+	+
733	991	Maltose/maltodextrin ABC transporter, permease protein MalG	57	+	+	+	+	+	+	+	+	+	+	+	+	+	+	+	+	+	+	+	+	+	+	+	+	+	+	+	+	+	+	+	+	+	+	+	+	+	+	+	+	+	+	+	+	+	+	+	+	+	+	+	+	+	+	+	+	+
734	993	Endonuclease III (EC 4.2.99.18)	57	+	+	+	+	+	+	+	+	+	+	+	+	+	+	+	+	+	+	+	+	+	+	+	+	+	+	+	+	+	+	+	+	+	+	+	+	+	+	+	+	+	+	+	+	+	+	+	+	+	+	+	+	+	+	+	+	+
735	994	Maltose/maltodextrin ABC transporter, permease protein MalF	57	+	+	+	+	+	+	+	+	+	+	+	+	+	+	+	+	+	+	+	+	+	+	+	+	+	+	+	+	+	+	+	+	+	+	+	+	+	+	+	+	+	+	+	+	+	+	+	+	+	+	+	+	+	+	+	+	+
736	995	RNA polymerase sigma factor, ECF subfamily	57	+	+	+	+	+	+	+	+	+	+	+	+	+	+	+	+	+	+	+	+	+	+	+	+	+	+	+	+	+	+	+	+	+	+	+	+	+	+	+	+	+	+	+	+	+	+	+	+	+	+	+	+	+	+	+	+	+
737	996	Substrate-specific component PdxU of predicted pyridoxine ECF transporter	57	+	+	+	+	+	+	+	+	+	+	+	+	+	+	+	+	+	+	+	+	+	+	+	+	+	+	+	+	+	+	+	+	+	+	+	+	+	+	+	+	+	+	+	+	+	+	+	+	+	+	+	+	+	+	+	+	+
738	997	D-alanyl-D-alanine carboxypeptidase (EC 3.4.16.4)#penicillin-binding protein 6b	57	+	+	+	+	+	+	+	+	+	+	+	+	+	+	+	+	+	+	+	+	+	+	+	+	+	+	+	+	+	+	+	+	+	+	+	+	+	+	+	+	+	+	+	+	+	+	+	+	+	+	+	+	+	+	+	+	+
739	998	Acetyltransferase, GNAT family	57	+	+	+	+	+	+	+	+	+	+	+	+	+	+	+	+	+	+	+	+	+	+	+	+	+	+	+	+	+	+	+	+	+	+	+	+	+	+	+	+	+	+	+	+	+	+	+	+	+	+	+	+	+	+	+	+	+
740	1001	Transcriptional regulator, MerR family	57	+	+	+	+	+	+	+	+	+	+	+	+	+	+	+	+	+	+	+	+	+	+	+	+	+	+	+	+	+	+	+	+	+	+	+	+	+	+	+	+	+	+	+	+	+	+	+	+	+	+	+	+	+	+	+	+	+
741	1002	probably aromatic ring hydroxylating enzyme, evidenced by COGnitor; PaaD-like protein (DUF59) involved in Fe-S cluster assembly	57	+	+	+	+	+	+	+	+	+	+	+	+	+	+	+	+	+	+	+	+	+	+	+	+	+	+	+	+	+	+	+	+	+	+	+	+	+	+	+	+	+	+	+	+	+	+	+	+	+	+	+	+	+	+	+	+	+
742	1003	Dihydroxy-acid dehydratase (EC 4.2.1.9)	57	+	+	+	+	+	+	+	+	+	+	+	+	+	+	+	+	+	+	+	+	+	+	+	+	+	+	+	+	+	+	+	+	+	+	+	+	+	+	+	+	+	+	+	+	+	+	+	+	+	+	+	+	+	+	+	+	+
743	1006	Maltose/maltodextrin ABC transporter, substrate binding periplasmic protein MalE	57	+	+	+	+	+	+	+	+	+	+	+	+	+	+	+	+	+	+	+	+	+	+	+	+	+	+	+	+	+	+	+	+	+	+	+	+	+	+	+	+	+	+	+	+	+	+	+	+	+	+	+	+	+	+	+	+	+
744	1008	O-acetylhomoserine sulfhydrylase (EC 2.5.1.49) / O-succinylhomoserine sulfhydrylase (EC 2.5.1.48)	57	+	+	+	+	+	+	+	+	+	+	+	+	+	+	+	+	+	+	+	+	+	+	+	+	+	+	+	+	+	+	+	+	+	+	+	+	+	+	+	+	+	+	+	+	+	+	+	+	+	+	+	+	+	+	+	+	+
745	1010	ABC transporter, ATP-binding/permease protein	57	+	+	+	+	+	+	+	+	+	+	+	+	+	+	+	+	+	+	+	+	+	+	+	+	+	+	+	+	+	+	+	+	+	+	+	+	+	+	+	+	+	+	+	+	+	+	+	+	+	+	+	+	+	+	+	+	+
746	1011	putative membrane protein	57	+	+	+	+	+	+	+	+	+	+	+	+	+	+	+	+	+	+	+	+	+	+	+	+	+	+	+	+	+	+	+	+	+	+	+	+	+	+	+	+	+	+	+	+	+	+	+	+	+	+	+	+	+	+	+	+	+
747	1012	ABC transporter, ATP-binding/permease protein	57	+	+	+	+	+	+	+	+	+	+	+	+	+	+	+	+	+	+	+	+	+	+	+	+	+	+	+	+	+	+	+	+	+	+	+	+	+	+	+	+	+	+	+	+	+	+	+	+	+	+	+	+	+	+	+	+	+
748	1013	Phosphinothricin N-acetyltransferase (EC 2.3.1.-)	57	+	+	+	+	+	+	+	+	+	+	+	+	+	+	+	+	+	+	+	+	+	+	+	+	+	+	+	+	+	+	+	+	+	+	+	+	+	+	+	+	+	+	+	+	+	+	+	+	+	+	+	+	+	+	+	+	+
749	1015	Cystine transport system permease protein	57	+	+	+	+	+	+	+	+	+	+	+	+	+	+	+	+	+	+	+	+	+	+	+	+	+	+	+	+	+	+	+	+	+	+	+	+	+	+	+	+	+	+	+	+	+	+	+	+	+	+	+	+	+	+	+	+	+
750	1016	Sialic acid utilization regulator, RpiR family	57	+	+	+	+	+	+	+	+	+	+	+	+	+	+	+	+	+	+	+	+	+	+	+	+	+	+	+	+	+	+	+	+	+	+	+	+	+	+	+	+	+	+	+	+	+	+	+	+	+	+	+	+	+	+	+	+	+
751	1017	Phosphohydrolase (MutT/nudix family protein)	57	+	+	+	+	+	+	+	+	+	+	+	+	+	+	+	+	+	+	+	+	+	+	+	+	+	+	+	+	+	+	+	+	+	+	+	+	+	+	+	+	+	+	+	+	+	+	+	+	+	+	+	+	+	+	+	+	+
752	1019	3-ketoacyl-CoA thiolase (EC 2.3.1.16) @ Acetyl-CoA acetyltransferase (EC 2.3.1.9)	57	+	+	+	+	+	+	+	+	+	+	+	+	+	+	+	+	+	+	+	+	+	+	+	+	+	+	+	+	+	+	+	+	+	+	+	+	+	+	+	+	+	+	+	+	+	+	+	+	+	+	+	+	+	+	+	+	+
753	1022	6-aminohexanoate-cyclic-dimer hydrolase (EC 3.5.2.12)	57	+	+	+	+	+	+	+	+	+	+	+	+	+	+	+	+	+	+	+	+	+	+	+	+	+	+	+	+	+	+	+	+	+	+	+	+	+	+	+	+	+	+	+	+	+	+	+	+	+	+	+	+	+	+	+	+	+
754	1023	Ribosomal RNA large subunit methyltransferase A (EC 2.1.1.51)	57	+	+	+	+	+	+	+	+	+	+	+	+	+	+	+	+	+	+	+	+	+	+	+	+	+	+	+	+	+	+	+	+	+	+	+	+	+	+	+	+	+	+	+	+	+	+	+	+	+	+	+	+	+	+	+	+	+
755	1024	Predicted dehydrogenase and related proteins	57	+	+	+	+	+	+	+	+	+	+	+	+	+	+	+	+	+	+	+	+	+	+	+	+	+	+	+	+	+	+	+	+	+	+	+	+	+	+	+	+	+	+	+	+	+	+	+	+	+	+	+	+	+	+	+	+	+
756	1028	D-alanyl-D-alanine carboxypeptidase (EC 3.4.16.4)#penicillin-binding protein 6b	57	+	+	+	+	+	+	+	+	+	+	+	+	+	+	+	+	+	+	+	+	+	+	+	+	+	+	+	+	+	+	+	+	+	+	+	+	+	+	+	+	+	+	+	+	+	+	+	+	+	+	+	+	+	+	+	+	+
757	1030	Predicted dehydrogenase and related proteins	57	+	+	+	+	+	+	+	+	+	+	+	+	+	+	+	+	+	+	+	+	+	+	+	+	+	+	+	+	+	+	+	+	+	+	+	+	+	+	+	+	+	+	+	+	+	+	+	+	+	+	+	+	+	+	+	+	+
758	1031	Rrf2 family transcriptional regulator, group III	57	+	+	+	+	+	+	+	+	+	+	+	+	+	+	+	+	+	+	+	+	+	+	+	+	+	+	+	+	+	+	+	+	+	+	+	+	+	+	+	+	+	+	+	+	+	+	+	+	+	+	+	+	+	+	+	+	+
759	1038	hypothetical protein	57	+	+	+	+	+	+	+	+	+	+	+	+	+	+	+	+	+	+	+	+	+	+	+	+	+	+	+	+	+	+	+	+	+	+	+	+	+	+	+	+	+	+	+	+	+	+	+	+	+	+	+	+	+	+	+	+	+
760	1042	hypothetical protein	57	+	+	+	+	+	+	+	+	+	+	+	+	+	+	+	+	+	+	+	+	+	+	+	+	+	+	+	+	+	+	+	+	+	+	+	+	+	+	+	+	+	+	+	+	+	+	+	+	+	+	+	+	+	+	+	+	+
761	1043	Predicted amidophosphoribosyltransferase	57	+	+	+	+	+	+	+	+	+	+	+	+	+	+	+	+	+	+	+	+	+	+	+	+	+	+	+	+	+	+	+	+	+	+	+	+	+	+	+	+	+	+	+	+	+	+	+	+	+	+	+	+	+	+	+	+	+
762	1045	Transcriptional regulator, GntR family	57	+	+	+	+	+	+	+	+	+	+	+	+	+	+	+	+	+	+	+	+	+	+	+	+	+	+	+	+	+	+	+	+	+	+	+	+	+	+	+	+	+	+	+	+	+	+	+	+	+	+	+	+	+	+	+	+	+
763	1047	Isocitrate dehydrogenase [NADP] (EC 1.1.1.42)	57	+	+	+	+	+	+	+	+	+	+	+	+	+	+	+	+	+	+	+	+	+	+	+	+	+	+	+	+	+	+	+	+	+	+	+	+	+	+	+	+	+	+	+	+	+	+	+	+	+	+	+	+	+	+	+	+	+
764	1048	Competence protein CoiA	57	+	+	+	+	+	+	+	+	+	+	+	+	+	+	+	+	+	+	+	+	+	+	+	+	+	+	+	+	+	+	+	+	+	+	+	+	+	+	+	+	+	+	+	+	+	+	+	+	+	+	+	+	+	+	+	+	+
765	1049	hypothetical protein	57	+	+	+	+	+	+	+	+	+	+	+	+	+	+	+	+	+	+	+	+	+	+	+	+	+	+	+	+	+	+	+	+	+	+	+	+	+	+	+	+	+	+	+	+	+	+	+	+	+	+	+	+	+	+	+	+	+
766	1050	transcriptional regulator, MerR family	57	+	+	+	+	+	+	+	+	+	+	+	+	+	+	+	+	+	+	+	+	+	+	+	+	+	+	+	+	+	+	+	+	+	+	+	+	+	+	+	+	+	+	+	+	+	+	+	+	+	+	+	+	+	+	+	+	+
767	1051	Hydrolase, haloacid dehalogenase-like family	57	+	+	+	+	+	+	+	+	+	+	+	+	+	+	+	+	+	+	+	+	+	+	+	+	+	+	+	+	+	+	+	+	+	+	+	+	+	+	+	+	+	+	+	+	+	+	+	+	+	+	+	+	+	+	+	+	+
768	1056	Transcriptional regulator, PBSX family	57	+	+	+	+	+	+	+	+	+	+	+	+	+	+	+	+	+	+	+	+	+	+	+	+	+	+	+	+	+	+	+	+	+	+	+	+	+	+	+	+	+	+	+	+	+	+	+	+	+	+	+	+	+	+	+	+	+
769	1057	Citrate synthase (si) (EC 2.3.3.1)	57	+	+	+	+	+	+	+	+	+	+	+	+	+	+	+	+	+	+	+	+	+	+	+	+	+	+	+	+	+	+	+	+	+	+	+	+	+	+	+	+	+	+	+	+	+	+	+	+	+	+	+	+	+	+	+	+	+
770	1059	DUF1696 domain-containing protein	57	+	+	+	+	+	+	+	+	+	+	+	+	+	+	+	+	+	+	+	+	+	+	+	+	+	+	+	+	+	+	+	+	+	+	+	+	+	+	+	+	+	+	+	+	+	+	+	+	+	+	+	+	+	+	+	+	+
771	1060	FIG009210: peptidase, M16 family	57	+	+	+	+	+	+	+	+	+	+	+	+	+	+	+	+	+	+	+	+	+	+	+	+	+	+	+	+	+	+	+	+	+	+	+	+	+	+	+	+	+	+	+	+	+	+	+	+	+	+	+	+	+	+	+	+	+
772	1061	hypothetical protein	57	+	+	+	+	+	+	+	+	+	+	+	+	+	+	+	+	+	+	+	+	+	+	+	+	+	+	+	+	+	+	+	+	+	+	+	+	+	+	+	+	+	+	+	+	+	+	+	+	+	+	+	+	+	+	+	+	+
773	1062	Glucose-6-phosphate 1-dehydrogenase (EC 1.1.1.49)	57	+	+	+	+	+	+	+	+	+	+	+	+	+	+	+	+	+	+	+	+	+	+	+	+	+	+	+	+	+	+	+	+	+	+	+	+	+	+	+	+	+	+	+	+	+	+	+	+	+	+	+	+	+	+	+	+	+
774	1063	Integral membrane protein	57	+	+	+	+	+	+	+	+	+	+	+	+	+	+	+	+	+	+	+	+	+	+	+	+	+	+	+	+	+	+	+	+	+	+	+	+	+	+	+	+	+	+	+	+	+	+	+	+	+	+	+	+	+	+	+	+	+
775	1064	Hydrolase (HAD superfamily)	57	+	+	+	+	+	+	+	+	+	+	+	+	+	+	+	+	+	+	+	+	+	+	+	+	+	+	+	+	+	+	+	+	+	+	+	+	+	+	+	+	+	+	+	+	+	+	+	+	+	+	+	+	+	+	+	+	+
776	1066	Potassium uptake protein TrkH	57	+	+	+	+	+	+	+	+	+	+	+	+	+	+	+	+	+	+	+	+	+	+	+	+	+	+	+	+	+	+	+	+	+	+	+	+	+	+	+	+	+	+	+	+	+	+	+	+	+	+	+	+	+	+	+	+	+
777	1067	Transporter	57	+	+	+	+	+	+	+	+	+	+	+	+	+	+	+	+	+	+	+	+	+	+	+	+	+	+	+	+	+	+	+	+	+	+	+	+	+	+	+	+	+	+	+	+	+	+	+	+	+	+	+	+	+	+	+	+	+
778	1068	uncharacterized secreted protein, YBBR Bacillus subtilis homolog	57	+	+	+	+	+	+	+	+	+	+	+	+	+	+	+	+	+	+	+	+	+	+	+	+	+	+	+	+	+	+	+	+	+	+	+	+	+	+	+	+	+	+	+	+	+	+	+	+	+	+	+	+	+	+	+	+	+
779	1069	Maltose operon transcriptional repressor MalR, LacI family	57	+	+	+	+	+	+	+	+	+	+	+	+	+	+	+	+	+	+	+	+	+	+	+	+	+	+	+	+	+	+	+	+	+	+	+	+	+	+	+	+	+	+	+	+	+	+	+	+	+	+	+	+	+	+	+	+	+
780	1071	Phosphoribosylanthranilate isomerase like (EC 5.3.1.24)	57	+	+	+	+	+	+	+	+	+	+	+	+	+	+	+	+	+	+	+	+	+	+	+	+	+	+	+	+	+	+	+	+	+	+	+	+	+	+	+	+	+	+	+	+	+	+	+	+	+	+	+	+	+	+	+	+	+
781	1072	Predicted phosphatase	57	+	+	+	+	+	+	+	+	+	+	+	+	+	+	+	+	+	+	+	+	+	+	+	+	+	+	+	+	+	+	+	+	+	+	+	+	+	+	+	+	+	+	+	+	+	+	+	+	+	+	+	+	+	+	+	+	+
782	1073	VanZF-related protein	57	+	+	+	+	+	+	+	+	+	+	+	+	+	+	+	+	+	+	+	+	+	+	+	+	+	+	+	+	+	+	+	+	+	+	+	+	+	+	+	+	+	+	+	+	+	+	+	+	+	+	+	+	+	+	+	+	+
783	1074	Putative uncharacterized protein gbs2121	57	+	+	+	+	+	+	+	+	+	+	+	+	+	+	+	+	+	+	+	+	+	+	+	+	+	+	+	+	+	+	+	+	+	+	+	+	+	+	+	+	+	+	+	+	+	+	+	+	+	+	+	+	+	+	+	+	+
784	1075	Trk system potassium uptake protein TrkA	57	+	+	+	+	+	+	+	+	+	+	+	+	+	+	+	+	+	+	+	+	+	+	+	+	+	+	+	+	+	+	+	+	+	+	+	+	+	+	+	+	+	+	+	+	+	+	+	+	+	+	+	+	+	+	+	+	+
785	1077	Negative regulator of genetic competence MecA	57	+	+	+	+	+	+	+	+	+	+	+	+	+	+	+	+	+	+	+	+	+	+	+	+	+	+	+	+	+	+	+	+	+	+	+	+	+	+	+	+	+	+	+	+	+	+	+	+	+	+	+	+	+	+	+	+	+
786	1083	2-dehydropantoate 2-reductase (EC 1.1.1.169)	57	+	+	+	+	+	+	+	+	+	+	+	+	+	+	+	+	+	+	+	+	+	+	+	+	+	+	+	+	+	+	+	+	+	+	+	+	+	+	+	+	+	+	+	+	+	+	+	+	+	+	+	+	+	+	+	+	+
787	1084	membrane protein	57	+	+	+	+	+	+	+	+	+	+	+	+	+	+	+	+	+	+	+	+	+	+	+	+	+	+	+	+	+	+	+	+	+	+	+	+	+	+	+	+	+	+	+	+	+	+	+	+	+	+	+	+	+	+	+	+	+
788	1086	5,10-methylenetetrahydrofolate reductase (EC 1.5.1.20)	57	+	+	+	+	+	+	+	+	+	+	+	+	+	+	+	+	+	+	+	+	+	+	+	+	+	+	+	+	+	+	+	+	+	+	+	+	+	+	+	+	+	+	+	+	+	+	+	+	+	+	+	+	+	+	+	+	+
789	1090	Transcriptional regulator, MarR family	57	+	+	+	+	+	+	+	+	+	+	+	+	+	+	+	+	+	+	+	+	+	+	+	+	+	+	+	+	+	+	+	+	+	+	+	+	+	+	+	+	+	+	+	+	+	+	+	+	+	+	+	+	+	+	+	+	+
790	1091	Transcriptional regulator, GntR family	57	+	+	+	+	+	+	+	+	+	+	+	+	+	+	+	+	+	+	+	+	+	+	+	+	+	+	+	+	+	+	+	+	+	+	+	+	+	+	+	+	+	+	+	+	+	+	+	+	+	+	+	+	+	+	+	+	+
791	1092	Aconitate hydratase (EC 4.2.1.3)	57	+	+	+	+	+	+	+	+	+	+	+	+	+	+	+	+	+	+	+	+	+	+	+	+	+	+	+	+	+	+	+	+	+	+	+	+	+	+	+	+	+	+	+	+	+	+	+	+	+	+	+	+	+	+	+	+	+
792	1093	Membrane spanning protein	57	+	+	+	+	+	+	+	+	+	+	+	+	+	+	+	+	+	+	+	+	+	+	+	+	+	+	+	+	+	+	+	+	+	+	+	+	+	+	+	+	+	+	+	+	+	+	+	+	+	+	+	+	+	+	+	+	+
793	1095	Membrane protein, putative	57	+	+	+	+	+	+	+	+	+	+	+	+	+	+	+	+	+	+	+	+	+	+	+	+	+	+	+	+	+	+	+	+	+	+	+	+	+	+	+	+	+	+	+	+	+	+	+	+	+	+	+	+	+	+	+	+	+
794	1098	putative transcriptional regulator	57	+	+	+	+	+	+	+	+	+	+	+	+	+	+	+	+	+	+	+	+	+	+	+	+	+	+	+	+	+	+	+	+	+	+	+	+	+	+	+	+	+	+	+	+	+	+	+	+	+	+	+	+	+	+	+	+	+
795	1101	hypothetical protein	57	+	+	+	+	+	+	+	+	+	+	+	+	+	+	+	+	+	+	+	+	+	+	+	+	+	+	+	+	+	+	+	+	+	+	+	+	+	+	+	+	+	+	+	+	+	+	+	+	+	+	+	+	+	+	+	+	+
796	1102	5-methyltetrahydropteroyltriglutamate--homocysteine methyltransferase (EC 2.1.1.14)	57	+	+	+	+	+	+	+	+	+	+	+	+	+	+	+	+	+	+	+	+	+	+	+	+	+	+	+	+	+	+	+	+	+	+	+	+	+	+	+	+	+	+	+	+	+	+	+	+	+	+	+	+	+	+	+	+	+
797	1107	Histone acetyltransferase HPA2 and related acetyltransferases	57	+	+	+	+	+	+	+	+	+	+	+	+	+	+	+	+	+	+	+	+	+	+	+	+	+	+	+	+	+	+	+	+	+	+	+	+	+	+	+	+	+	+	+	+	+	+	+	+	+	+	+	+	+	+	+	+	+
798	1108	ABC transporter permease protein	57	+	+	+	+	+	+	+	+	+	+	+	+	+	+	+	+	+	+	+	+	+	+	+	+	+	+	+	+	+	+	+	+	+	+	+	+	+	+	+	+	+	+	+	+	+	+	+	+	+	+	+	+	+	+	+	+	+
799	1109	oxidoreductase	57	+	+	+	+	+	+	+	+	+	+	+	+	+	+	+	+	+	+	+	+	+	+	+	+	+	+	+	+	+	+	+	+	+	+	+	+	+	+	+	+	+	+	+	+	+	+	+	+	+	+	+	+	+	+	+	+	+
800	1121	5'-nucleotidase (EC 3.1.3.5)	57	+	+	+	+	+	+	+	+	+	+	+	+	+	+	+	+	+	+	+	+	+	+	+	+	+	+	+	+	+	+	+	+	+	+	+	+	+	+	+	+	+	+	+	+	+	+	+	+	+	+	+	+	+	+	+	+	+
801	1123	Membrane-associated phospholipid phosphatase	57	+	+	+	+	+	+	+	+	+	+	+	+	+	+	+	+	+	+	+	+	+	+	+	+	+	+	+	+	+	+	+	+	+	+	+	+	+	+	+	+	+	+	+	+	+	+	+	+	+	+	+	+	+	+	+	+	+
802	1127	periplasmic component of efflux system	57	+	+	+	+	+	+	+	+	+	+	+	+	+	+	+	+	+	+	+	+	+	+	+	+	+	+	+	+	+	+	+	+	+	+	+	+	+	+	+	+	+	+	+	+	+	+	+	+	+	+	+	+	+	+	+	+	+
803	1128	Cystine ABC transporter, periplasmic cystine-binding protein FliY	57	+	+	+	+	+	+	+	+	+	+	+	+	+	+	+	+	+	+	+	+	+	+	+	+	+	+	+	+	+	+	+	+	+	+	+	+	+	+	+	+	+	+	+	+	+	+	+	+	+	+	+	+	+	+	+	+	+
804	1130	Acetyltransferase (EC 2.3.1.-)	57	+	+	+	+	+	+	+	+	+	+	+	+	+	+	+	+	+	+	+	+	+	+	+	+	+	+	+	+	+	+	+	+	+	+	+	+	+	+	+	+	+	+	+	+	+	+	+	+	+	+	+	+	+	+	+	+	+
805	1132	PhnB protein	57	+	+	+	+	+	+	+	+	+	+	+	+	+	+	+	+	+	+	+	+	+	+	+	+	+	+	+	+	+	+	+	+	+	+	+	+	+	+	+	+	+	+	+	+	+	+	+	+	+	+	+	+	+	+	+	+	+
806	1133	2',3'-cyclic-nucleotide 2'-phosphodiesterase (EC 3.1.4.16)	57	+	+	+	+	+	+	+	+	+	+	+	+	+	+	+	+	+	+	+	+	+	+	+	+	+	+	+	+	+	+	+	+	+	+	+	+	+	+	+	+	+	+	+	+	+	+	+	+	+	+	+	+	+	+	+	+	+
807	1139	Ferrichrome-binding periplasmic protein precursor (TC 3.A.1.14.3)	57	+	+	+	+	+	+	+	+	+	+	+	+	+	+	+	+	+	+	+	+	+	+	+	+	+	+	+	+	+	+	+	+	+	+	+	+	+	+	+	+	+	+	+	+	+	+	+	+	+	+	+	+	+	+	+	+	+
808	1140	Inositol-1-monophosphatase (EC 3.1.3.25)	57	+	+	+	+	+	+	+	+	+	+	+	+	+	+	+	+	+	+	+	+	+	+	+	+	+	+	+	+	+	+	+	+	+	+	+	+	+	+	+	+	+	+	+	+	+	+	+	+	+	+	+	+	+	+	+	+	+
809	1143	Hypothetical protein SPy1643	57	+	+	+	+	+	+	+	+	+	+	+	+	+	+	+	+	+	+	+	+	+	+	+	+	+	+	+	+	+	+	+	+	+	+	+	+	+	+	+	+	+	+	+	+	+	+	+	+	+	+	+	+	+	+	+	+	+
810	1145	Protein of unknown function DUF1349	57	+	+	+	+	+	+	+	+	+	+	+	+	+	+	+	+	+	+	+	+	+	+	+	+	+	+	+	+	+	+	+	+	+	+	+	+	+	+	+	+	+	+	+	+	+	+	+	+	+	+	+	+	+	+	+	+	+
811	1147	Oxidoreductase, short-chain dehydrogenase/reductase family (EC 1.1.1.-)	57	+	+	+	+	+	+	+	+	+	+	+	+	+	+	+	+	+	+	+	+	+	+	+	+	+	+	+	+	+	+	+	+	+	+	+	+	+	+	+	+	+	+	+	+	+	+	+	+	+	+	+	+	+	+	+	+	+
812	1151	Nitroreductase family protein	57	+	+	+	+	+	+	+	+	+	+	+	+	+	+	+	+	+	+	+	+	+	+	+	+	+	+	+	+	+	+	+	+	+	+	+	+	+	+	+	+	+	+	+	+	+	+	+	+	+	+	+	+	+	+	+	+	+
813	1157	Quinone oxidoreductase (EC 1.6.5.5)	57	+	+	+	+	+	+	+	+	+	+	+	+	+	+	+	+	+	+	+	+	+	+	+	+	+	+	+	+	+	+	+	+	+	+	+	+	+	+	+	+	+	+	+	+	+	+	+	+	+	+	+	+	+	+	+	+	+
814	1159	Transcriptional regulator ArcR essential for anaerobic expression of the ADI pathway, Crp/Fnr family	57	+	+	+	+	+	+	+	+	+	+	+	+	+	+	+	+	+	+	+	+	+	+	+	+	+	+	+	+	+	+	+	+	+	+	+	+	+	+	+	+	+	+	+	+	+	+	+	+	+	+	+	+	+	+	+	+	+
815	1160	hypothetical protein	57	+	+	+	+	+	+	+	+	+	+	+	+	+	+	+	+	+	+	+	+	+	+	+	+	+	+	+	+	+	+	+	+	+	+	+	+	+	+	+	+	+	+	+	+	+	+	+	+	+	+	+	+	+	+	+	+	+
816	1162	Competence-specific sigma factor ComX	57	+	+	+	+	+	+	+	+	+	+	+	+	+	+	+	+	+	+	+	+	+	+	+	+	+	+	+	+	+	+	+	+	+	+	+	+	+	+	+	+	+	+	+	+	+	+	+	+	+	+	+	+	+	+	+	+	+
817	1164	Pullulanase (EC 3.2.1.41)	57	+	+	+	+	+	+	+	+	+	+	+	+	+	+	+	+	+	+	+	+	+	+	+	+	+	+	+	+	+	+	+	+	+	+	+	+	+	+	+	+	+	+	+	+	+	+	+	+	+	+	+	+	+	+	+	+	+
818	1166	Muramoyltetrapeptide carboxypeptidase (EC 3.4.17.13)	57	+	+	+	+	+	+	+	+	+	+	+	+	+	+	+	+	+	+	+	+	+	+	+	+	+	+	+	+	+	+	+	+	+	+	+	+	+	+	+	+	+	+	+	+	+	+	+	+	+	+	+	+	+	+	+	+	+
819	1168	Phosphoglycolate phosphatase (EC 3.1.3.18)	57	+	+	+	+	+	+	+	+	+	+	+	+	+	+	+	+	+	+	+	+	+	+	+	+	+	+	+	+	+	+	+	+	+	+	+	+	+	+	+	+	+	+	+	+	+	+	+	+	+	+	+	+	+	+	+	+	+
820	1169	FIG001960: FtsZ-interacting protein related to cell division	57	+	+	+	+	+	+	+	+	+	+	+	+	+	+	+	+	+	+	+	+	+	+	+	+	+	+	+	+	+	+	+	+	+	+	+	+	+	+	+	+	+	+	+	+	+	+	+	+	+	+	+	+	+	+	+	+	+
821	1170	Isochorismatase (EC 3.3.2.1)	57	+	+	+	+	+	+	+	+	+	+	+	+	+	+	+	+	+	+	+	+	+	+	+	+	+	+	+	+	+	+	+	+	+	+	+	+	+	+	+	+	+	+	+	+	+	+	+	+	+	+	+	+	+	+	+	+	+
822	1176	Transcriptional regulator, MarR family	57	+	+	+	+	+	+	+	+	+	+	+	+	+	+	+	+	+	+	+	+	+	+	+	+	+	+	+	+	+	+	+	+	+	+	+	+	+	+	+	+	+	+	+	+	+	+	+	+	+	+	+	+	+	+	+	+	+
823	1184	Glutathione S-transferase, omega (EC 2.5.1.18)	57	+	+	+	+	+	+	+	+	+	+	+	+	+	+	+	+	+	+	+	+	+	+	+	+	+	+	+	+	+	+	+	+	+	+	+	+	+	+	+	+	+	+	+	+	+	+	+	+	+	+	+	+	+	+	+	+	+
824	1187	EpiH/GdmH-related protein	57	+	+	+	+	+	+	+	+	+	+	+	+	+	+	+	+	+	+	+	+	+	+	+	+	+	+	+	+	+	+	+	+	+	+	+	+	+	+	+	+	+	+	+	+	+	+	+	+	+	+	+	+	+	+	+	+	+
825	1188	ADP-ribose pyrophosphatase	57	+	+	+	+	+	+	+	+	+	+	+	+	+	+	+	+	+	+	+	+	+	+	+	+	+	+	+	+	+	+	+	+	+	+	+	+	+	+	+	+	+	+	+	+	+	+	+	+	+	+	+	+	+	+	+	+	+
826	1190	Copper chaperone	57	+	+	+	+	+	+	+	+	+	+	+	+	+	+	+	+	+	+	+	+	+	+	+	+	+	+	+	+	+	+	+	+	+	+	+	+	+	+	+	+	+	+	+	+	+	+	+	+	+	+	+	+	+	+	+	+	+
827	1191	hypothetical protein	57	+	+	+	+	+	+	+	+	+	+	+	+	+	+	+	+	+	+	+	+	+	+	+	+	+	+	+	+	+	+	+	+	+	+	+	+	+	+	+	+	+	+	+	+	+	+	+	+	+	+	+	+	+	+	+	+	+
828	1194	Transcriptional regulator, PadR family	57	+	+	+	+	+	+	+	+	+	+	+	+	+	+	+	+	+	+	+	+	+	+	+	+	+	+	+	+	+	+	+	+	+	+	+	+	+	+	+	+	+	+	+	+	+	+	+	+	+	+	+	+	+	+	+	+	+
829	1198	Cell division protein DivIC (FtsB), stabilizes FtsL against RasP cleavage	57	+	+	+	+	+	+	+	+	+	+	+	+	+	+	+	+	+	+	+	+	+	+	+	+	+	+	+	+	+	+	+	+	+	+	+	+	+	+	+	+	+	+	+	+	+	+	+	+	+	+	+	+	+	+	+	+	+
830	1199	hypothetical protein	57	+	+	+	+	+	+	+	+	+	+	+	+	+	+	+	+	+	+	+	+	+	+	+	+	+	+	+	+	+	+	+	+	+	+	+	+	+	+	+	+	+	+	+	+	+	+	+	+	+	+	+	+	+	+	+	+	+
831	1201	lantibiotic efflux protein	57	+	+	+	+	+	+	+	+	+	+	+	+	+	+	+	+	+	+	+	+	+	+	+	+	+	+	+	+	+	+	+	+	+	+	+	+	+	+	+	+	+	+	+	+	+	+	+	+	+	+	+	+	+	+	+	+	+
832	1202	Rrf2 family transcriptional regulator, group III	57	+	+	+	+	+	+	+	+	+	+	+	+	+	+	+	+	+	+	+	+	+	+	+	+	+	+	+	+	+	+	+	+	+	+	+	+	+	+	+	+	+	+	+	+	+	+	+	+	+	+	+	+	+	+	+	+	+
833	1204	Low-specificity L-threonine aldolase (EC 4.1.2.5)	57	+	+	+	+	+	+	+	+	+	+	+	+	+	+	+	+	+	+	+	+	+	+	+	+	+	+	+	+	+	+	+	+	+	+	+	+	+	+	+	+	+	+	+	+	+	+	+	+	+	+	+	+	+	+	+	+	+
834	1212	Putative prenyltransferase, contains 1,4-dihydroxy-2-naphthoate octaprenyltransferase domain	57	+	+	+	+	+	+	+	+	+	+	+	+	+	+	+	+	+	+	+	+	+	+	+	+	+	+	+	+	+	+	+	+	+	+	+	+	+	+	+	+	+	+	+	+	+	+	+	+	+	+	+	+	+	+	+	+	+
835	1213	Glyoxalase/bleomycin resistance protein/dioxygenase	57	+	+	+	+	+	+	+	+	+	+	+	+	+	+	+	+	+	+	+	+	+	+	+	+	+	+	+	+	+	+	+	+	+	+	+	+	+	+	+	+	+	+	+	+	+	+	+	+	+	+	+	+	+	+	+	+	+
836	1214	Hydrolase, alpha/beta fold family	57	+	+	+	+	+	+	+	+	+	+	+	+	+	+	+	+	+	+	+	+	+	+	+	+	+	+	+	+	+	+	+	+	+	+	+	+	+	+	+	+	+	+	+	+	+	+	+	+	+	+	+	+	+	+	+	+	+
837	1215	Predicted membrane protein	57	+	+	+	+	+	+	+	+	+	+	+	+	+	+	+	+	+	+	+	+	+	+	+	+	+	+	+	+	+	+	+	+	+	+	+	+	+	+	+	+	+	+	+	+	+	+	+	+	+	+	+	+	+	+	+	+	+
838	1216	putative bacterocin transport accessory protein, Bta	57	+	+	+	+	+	+	+	+	+	+	+	+	+	+	+	+	+	+	+	+	+	+	+	+	+	+	+	+	+	+	+	+	+	+	+	+	+	+	+	+	+	+	+	+	+	+	+	+	+	+	+	+	+	+	+	+	+
839	1217	tRNA(Ile)-lysidine synthetase	57	+	+	+	+	+	+	+	+	+	+	+	+	+	+	+	+	+	+	+	+	+	+	+	+	+	+	+	+	+	+	+	+	+	+	+	+	+	+	+	+	+	+	+	+	+	+	+	+	+	+	+	+	+	+	+	+	+
840	1218	ATP synthase B chain (EC 3.6.3.14)	57	+	+	+	+	+	+	+	+	+	+	+	+	+	+	+	+	+	+	+	+	+	+	+	+	+	+	+	+	+	+	+	+	+	+	+	+	+	+	+	+	+	+	+	+	+	+	+	+	+	+	+	+	+	+	+	+	+
841	1225	Glycosyl transferase, family 2	57	+	+	+	+	+	+	+	+	+	+	+	+	+	+	+	+	+	+	+	+	+	+	+	+	+	+	+	+	+	+	+	+	+	+	+	+	+	+	+	+	+	+	+	+	+	+	+	+	+	+	+	+	+	+	+	+	+
842	1226	hypothetical protein	57	+	+	+	+	+	+	+	+	+	+	+	+	+	+	+	+	+	+	+	+	+	+	+	+	+	+	+	+	+	+	+	+	+	+	+	+	+	+	+	+	+	+	+	+	+	+	+	+	+	+	+	+	+	+	+	+	+
843	1230	Cell division protein FtsL	57	+	+	+	+	+	+	+	+	+	+	+	+	+	+	+	+	+	+	+	+	+	+	+	+	+	+	+	+	+	+	+	+	+	+	+	+	+	+	+	+	+	+	+	+	+	+	+	+	+	+	+	+	+	+	+	+	+
844	1231	Hypothetical similar to thiamin biosynthesis lipoprotein ApbE	57	+	+	+	+	+	+	+	+	+	+	+	+	+	+	+	+	+	+	+	+	+	+	+	+	+	+	+	+	+	+	+	+	+	+	+	+	+	+	+	+	+	+	+	+	+	+	+	+	+	+	+	+	+	+	+	+	+
845	1233	LemA family protein	57	+	+	+	+	+	+	+	+	+	+	+	+	+	+	+	+	+	+	+	+	+	+	+	+	+	+	+	+	+	+	+	+	+	+	+	+	+	+	+	+	+	+	+	+	+	+	+	+	+	+	+	+	+	+	+	+	+
846	1234	NADH dehydrogenase (EC 1.6.99.3) in cluster with putative pheromone precursor	57	+	+	+	+	+	+	+	+	+	+	+	+	+	+	+	+	+	+	+	+	+	+	+	+	+	+	+	+	+	+	+	+	+	+	+	+	+	+	+	+	+	+	+	+	+	+	+	+	+	+	+	+	+	+	+	+	+
847	1238	Putative pheromone precursor lipoprotein	57	+	+	+	+	+	+	+	+	+	+	+	+	+	+	+	+	+	+	+	+	+	+	+	+	+	+	+	+	+	+	+	+	+	+	+	+	+	+	+	+	+	+	+	+	+	+	+	+	+	+	+	+	+	+	+	+	+
848	1239	Arginine pathway regulatory protein ArgR, repressor of arg regulon	57	+	+	+	+	+	+	+	+	+	+	+	+	+	+	+	+	+	+	+	+	+	+	+	+	+	+	+	+	+	+	+	+	+	+	+	+	+	+	+	+	+	+	+	+	+	+	+	+	+	+	+	+	+	+	+	+	+
849	1245	Heptaprenyl diphosphate synthase component I (EC 2.5.1.30)	57	+	+	+	+	+	+	+	+	+	+	+	+	+	+	+	+	+	+	+	+	+	+	+	+	+	+	+	+	+	+	+	+	+	+	+	+	+	+	+	+	+	+	+	+	+	+	+	+	+	+	+	+	+	+	+	+	+
850	1249	Heptaprenyl diphosphate synthase component II (EC 2.5.1.30)	57	+	+	+	+	+	+	+	+	+	+	+	+	+	+	+	+	+	+	+	+	+	+	+	+	+	+	+	+	+	+	+	+	+	+	+	+	+	+	+	+	+	+	+	+	+	+	+	+	+	+	+	+	+	+	+	+	+
851	1251	Glycerophosphoryl diester phosphodiesterase (EC 3.1.4.46)	57	+	+	+	+	+	+	+	+	+	+	+	+	+	+	+	+	+	+	+	+	+	+	+	+	+	+	+	+	+	+	+	+	+	+	+	+	+	+	+	+	+	+	+	+	+	+	+	+	+	+	+	+	+	+	+	+	+
852	1254	hypothetical protein	57	+	+	+	+	+	+	+	+	+	+	+	+	+	+	+	+	+	+	+	+	+	+	+	+	+	+	+	+	+	+	+	+	+	+	+	+	+	+	+	+	+	+	+	+	+	+	+	+	+	+	+	+	+	+	+	+	+
853	1259	HIT family protein	57	+	+	+	+	+	+	+	+	+	+	+	+	+	+	+	+	+	+	+	+	+	+	+	+	+	+	+	+	+	+	+	+	+	+	+	+	+	+	+	+	+	+	+	+	+	+	+	+	+	+	+	+	+	+	+	+	+
854	1261	FIG001621: Zinc protease	57	+	+	+	+	+	+	+	+	+	+	+	+	+	+	+	+	+	+	+	+	+	+	+	+	+	+	+	+	+	+	+	+	+	+	+	+	+	+	+	+	+	+	+	+	+	+	+	+	+	+	+	+	+	+	+	+	+
855	1272	Acyl carrier protein	57	+	+	+	+	+	+	+	+	+	+	+	+	+	+	+	+	+	+	+	+	+	+	+	+	+	+	+	+	+	+	+	+	+	+	+	+	+	+	+	+	+	+	+	+	+	+	+	+	+	+	+	+	+	+	+	+	+
856	1273	putative transcriptional regulator	57	+	+	+	+	+	+	+	+	+	+	+	+	+	+	+	+	+	+	+	+	+	+	+	+	+	+	+	+	+	+	+	+	+	+	+	+	+	+	+	+	+	+	+	+	+	+	+	+	+	+	+	+	+	+	+	+	+
857	1274	Histone acetyltransferase HPA2 and related acetyltransferases	57	+	+	+	+	+	+	+	+	+	+	+	+	+	+	+	+	+	+	+	+	+	+	+	+	+	+	+	+	+	+	+	+	+	+	+	+	+	+	+	+	+	+	+	+	+	+	+	+	+	+	+	+	+	+	+	+	+
858	1280	hypothetical protein	57	+	+	+	+	+	+	+	+	+	+	+	+	+	+	+	+	+	+	+	+	+	+	+	+	+	+	+	+	+	+	+	+	+	+	+	+	+	+	+	+	+	+	+	+	+	+	+	+	+	+	+	+	+	+	+	+	+
859	1282	Colicin V production protein	57	+	+	+	+	+	+	+	+	+	+	+	+	+	+	+	+	+	+	+	+	+	+	+	+	+	+	+	+	+	+	+	+	+	+	+	+	+	+	+	+	+	+	+	+	+	+	+	+	+	+	+	+	+	+	+	+	+
860	1283	Trehalose operon transcriptional repressor	57	+	+	+	+	+	+	+	+	+	+	+	+	+	+	+	+	+	+	+	+	+	+	+	+	+	+	+	+	+	+	+	+	+	+	+	+	+	+	+	+	+	+	+	+	+	+	+	+	+	+	+	+	+	+	+	+	+
861	1285	Hypothetical protein SPy1656	57	+	+	+	+	+	+	+	+	+	+	+	+	+	+	+	+	+	+	+	+	+	+	+	+	+	+	+	+	+	+	+	+	+	+	+	+	+	+	+	+	+	+	+	+	+	+	+	+	+	+	+	+	+	+	+	+	+
862	1286	Predicted hydrolase (HAD superfamily)	57	+	+	+	+	+	+	+	+	+	+	+	+	+	+	+	+	+	+	+	+	+	+	+	+	+	+	+	+	+	+	+	+	+	+	+	+	+	+	+	+	+	+	+	+	+	+	+	+	+	+	+	+	+	+	+	+	+
863	1287	Cardiolipin synthetase (EC 2.7.8.-)	57	+	+	+	+	+	+	+	+	+	+	+	+	+	+	+	+	+	+	+	+	+	+	+	+	+	+	+	+	+	+	+	+	+	+	+	+	+	+	+	+	+	+	+	+	+	+	+	+	+	+	+	+	+	+	+	+	+
864	1290	Septum formation protein Maf	57	+	+	+	+	+	+	+	+	+	+	+	+	+	+	+	+	+	+	+	+	+	+	+	+	+	+	+	+	+	+	+	+	+	+	+	+	+	+	+	+	+	+	+	+	+	+	+	+	+	+	+	+	+	+	+	+	+
865	1291	SatD	57	+	+	+	+	+	+	+	+	+	+	+	+	+	+	+	+	+	+	+	+	+	+	+	+	+	+	+	+	+	+	+	+	+	+	+	+	+	+	+	+	+	+	+	+	+	+	+	+	+	+	+	+	+	+	+	+	+
866	1293	Exodeoxyribonuclease III (EC 3.1.11.2)	57	+	+	+	+	+	+	+	+	+	+	+	+	+	+	+	+	+	+	+	+	+	+	+	+	+	+	+	+	+	+	+	+	+	+	+	+	+	+	+	+	+	+	+	+	+	+	+	+	+	+	+	+	+	+	+	+	+

Table S5. Precence/absence of each HG among 57 *S. suis* strains

Index[a]	ClusterID	Gene products	Members	1																13			25					28						104		94			76			53/54		Divergent *S. suis*																
				98HAH33	05ZYH33	SC84	A7	SS12	P1/7	DAT273	NIAH11434	JS14	GZ1	BM407	S735	13730	MNCM07	NCTC 10446	14636	NCTC 10237	ST1	2651	89-1591	MNCM04	MNCM25	8074	D9	4961	ST3	MNCM43	DAT292	DAT260	NIAH11435	MNCM21	MNCM50	89-2479	DAT234	DAT290	93A	42A	NT77	6407	11538	8830	12814	92-1400	89-590	2726	2524	89-3576-3	4417	D12	10581	89-5259	88-5299A	R61	92-1191	14A	92-4172	22083
867	1297	Acetyltransferase (EC 2.3.1.-)	57	+	+	+	+	+	+	+	+	+	+	+	+	+	+	+	+	+	+	+	+	+	+	+	+	+	+	+	+	+	+	+	+	+	+	+	+	+	+	+	+	+	+	+	+	+	+	+	+	+	+	+	+	+
868	1298	Transcription regulator [contains diacylglycerol kinase catalytic domain]	57	+	+	+	+	+	+	+	+	+	+	+	+	+	+	+	+	+	+	+	+	+	+	+	+	+	+	+	+	+	+	+	+	+	+	+	+	+	+	+	+	+	+	+	+	+	+	+	+	+	+	+	+	+
869	1302	Substrate-specific component ThiT of thiamin ECF transporter	57	+	+	+	+	+	+	+	+	+	+	+	+	+	+	+	+	+	+	+	+	+	+	+	+	+	+	+	+	+	+	+	+	+	+	+	+	+	+	+	+	+	+	+	+	+	+	+	+	+	+	+	+	+
870	1303	Transcriptional regulator, MarR family	57	+	+	+	+	+	+	+	+	+	+	+	+	+	+	+	+	+	+	+	+	+	+	+	+	+	+	+	+	+	+	+	+	+	+	+	+	+	+	+	+	+	+	+	+	+	+	+	+	+	+	+	+	+
871	1307	Signal peptidase I (EC 3.4.21.89)	57	+	+	+	+	+	+	+	+	+	+	+	+	+	+	+	+	+	+	+	+	+	+	+	+	+	+	+	+	+	+	+	+	+	+	+	+	+	+	+	+	+	+	+	+	+	+	+	+	+	+	+	+	+
872	1308	Substrate-specific component FolT of folate ECF transporter	57	+	+	+	+	+	+	+	+	+	+	+	+	+	+	+	+	+	+	+	+	+	+	+	+	+	+	+	+	+	+	+	+	+	+	+	+	+	+	+	+	+	+	+	+	+	+	+	+	+	+	+	+	+
873	1309	Predicted permease	57	+	+	+	+	+	+	+	+	+	+	+	+	+	+	+	+	+	+	+	+	+	+	+	+	+	+	+	+	+	+	+	+	+	+	+	+	+	+	+	+	+	+	+	+	+	+	+	+	+	+	+	+	+
874	1310	Glycine/D-amino acid oxidases (deaminating)	57	+	+	+	+	+	+	+	+	+	+	+	+	+	+	+	+	+	+	+	+	+	+	+	+	+	+	+	+	+	+	+	+	+	+	+	+	+	+	+	+	+	+	+	+	+	+	+	+	+	+	+	+	+
875	1311	FMN-dependent NADH-azoreductase	57	+	+	+	+	+	+	+	+	+	+	+	+	+	+	+	+	+	+	+	+	+	+	+	+	+	+	+	+	+	+	+	+	+	+	+	+	+	+	+	+	+	+	+	+	+	+	+	+	+	+	+	+	+
876	1313	hypothetical protein	57	+	+	+	+	+	+	+	+	+	+	+	+	+	+	+	+	+	+	+	+	+	+	+	+	+	+	+	+	+	+	+	+	+	+	+	+	+	+	+	+	+	+	+	+	+	+	+	+	+	+	+	+	+
877	1314	VanZ like protein	57	+	+	+	+	+	+	+	+	+	+	+	+	+	+	+	+	+	+	+	+	+	+	+	+	+	+	+	+	+	+	+	+	+	+	+	+	+	+	+	+	+	+	+	+	+	+	+	+	+	+	+	+	+
878	1315	putative arylalkylamine n-acetyltransferase	57	+	+	+	+	+	+	+	+	+	+	+	+	+	+	+	+	+	+	+	+	+	+	+	+	+	+	+	+	+	+	+	+	+	+	+	+	+	+	+	+	+	+	+	+	+	+	+	+	+	+	+	+	+
879	1316	membrane protein, putative	57	+	+	+	+	+	+	+	+	+	+	+	+	+	+	+	+	+	+	+	+	+	+	+	+	+	+	+	+	+	+	+	+	+	+	+	+	+	+	+	+	+	+	+	+	+	+	+	+	+	+	+	+	+
880	1317	ATP synthase delta chain (EC 3.6.3.14)	57	+	+	+	+	+	+	+	+	+	+	+	+	+	+	+	+	+	+	+	+	+	+	+	+	+	+	+	+	+	+	+	+	+	+	+	+	+	+	+	+	+	+	+	+	+	+	+	+	+	+	+	+	+
881	1318	hypothetical protein	57	+	+	+	+	+	+	+	+	+	+	+	+	+	+	+	+	+	+	+	+	+	+	+	+	+	+	+	+	+	+	+	+	+	+	+	+	+	+	+	+	+	+	+	+	+	+	+	+	+	+	+	+	+
882	1319	Late competence protein ComC, processing protease	57	+	+	+	+	+	+	+	+	+	+	+	+	+	+	+	+	+	+	+	+	+	+	+	+	+	+	+	+	+	+	+	+	+	+	+	+	+	+	+	+	+	+	+	+	+	+	+	+	+	+	+	+	+
883	1320	Lysophospholipase L1 and related esterase	57	+	+	+	+	+	+	+	+	+	+	+	+	+	+	+	+	+	+	+	+	+	+	+	+	+	+	+	+	+	+	+	+	+	+	+	+	+	+	+	+	+	+	+	+	+	+	+	+	+	+	+	+	+
884	1321	hypothetical protein	57	+	+	+	+	+	+	+	+	+	+	+	+	+	+	+	+	+	+	+	+	+	+	+	+	+	+	+	+	+	+	+	+	+	+	+	+	+	+	+	+	+	+	+	+	+	+	+	+	+	+	+	+	+
885	1322	1,4-alpha-glucan (glycogen) branching enzyme, GH-13-type (EC 2.4.1.18)	57	+	+	+	+	+	+	+	+	+	+	+	+	+	+	+	+	+	+	+	+	+	+	+	+	+	+	+	+	+	+	+	+	+	+	+	+	+	+	+	+	+	+	+	+	+	+	+	+	+	+	+	+	+
886	1323	hypothetical protein	57	+	+	+	+	+	+	+	+	+	+	+	+	+	+	+	+	+	+	+	+	+	+	+	+	+	+	+	+	+	+	+	+	+	+	+	+	+	+	+	+	+	+	+	+	+	+	+	+	+	+	+	+	+
887	1324	Negative transcriptional regulator-copper transport operon	57	+	+	+	+	+	+	+	+	+	+	+	+	+	+	+	+	+	+	+	+	+	+	+	+	+	+	+	+	+	+	+	+	+	+	+	+	+	+	+	+	+	+	+	+	+	+	+	+	+	+	+	+	+
888	1325	Predicted aminoglycoside phosphotransferase	57	+	+	+	+	+	+	+	+	+	+	+	+	+	+	+	+	+	+	+	+	+	+	+	+	+	+	+	+	+	+	+	+	+	+	+	+	+	+	+	+	+	+	+	+	+	+	+	+	+	+	+	+	+
889	1326	hypothetical protein	57	+	+	+	+	+	+	+	+	+	+	+	+	+	+	+	+	+	+	+	+	+	+	+	+	+	+	+	+	+	+	+	+	+	+	+	+	+	+	+	+	+	+	+	+	+	+	+	+	+	+	+	+	+
890	1327	Glycogen synthase, ADP-glucose transglucosylase (EC 2.4.1.21)	57	+	+	+	+	+	+	+	+	+	+	+	+	+	+	+	+	+	+	+	+	+	+	+	+	+	+	+	+	+	+	+	+	+	+	+	+	+	+	+	+	+	+	+	+	+	+	+	+	+	+	+	+	+
891	1328	Hydrolase (HAD superfamily)	57	+	+	+	+	+	+	+	+	+	+	+	+	+	+	+	+	+	+	+	+	+	+	+	+	+	+	+	+	+	+	+	+	+	+	+	+	+	+	+	+	+	+	+	+	+	+	+	+	+	+	+	+	+
892	1329	hypothetical protein	57	+	+	+	+	+	+	+	+	+	+	+	+	+	+	+	+	+	+	+	+	+	+	+	+	+	+	+	+	+	+	+	+	+	+	+	+	+	+	+	+	+	+	+	+	+	+	+	+	+	+	+	+	+
893	1330	Permease of the major facilitator superfamily	57	+	+	+	+	+	+	+	+	+	+	+	+	+	+	+	+	+	+	+	+	+	+	+	+	+	+	+	+	+	+	+	+	+	+	+	+	+	+	+	+	+	+	+	+	+	+	+	+	+	+	+	+	+
894	1331	Helicase loader DnaB	57	+	+	+	+	+	+	+	+	+	+	+	+	+	+	+	+	+	+	+	+	+	+	+	+	+	+	+	+	+	+	+	+	+	+	+	+	+	+	+	+	+	+	+	+	+	+	+	+	+	+	+	+	+
895	1332	Glucose-6-phosphate isomerase	57	+	+	+	+	+	+	+	+	+	+	+	+	+	+	+	+	+	+	+	+	+	+	+	+	+	+	+	+	+	+	+	+	+	+	+	+	+	+	+	+	+	+	+	+	+	+	+	+	+	+	+	+	+
896	1333	Membrane-bound protease, CAAX family	57	+	+	+	+	+	+	+	+	+	+	+	+	+	+	+	+	+	+	+	+	+	+	+	+	+	+	+	+	+	+	+	+	+	+	+	+	+	+	+	+	+	+	+	+	+	+	+	+	+	+	+	+	+
897	1334	Oligoendopeptidase F	57	+	+	+	+	+	+	+	+	+	+	+	+	+	+	+	+	+	+	+	+	+	+	+	+	+	+	+	+	+	+	+	+	+	+	+	+	+	+	+	+	+	+	+	+	+	+	+	+	+	+	+	+	+
898	1335	C3-degrading proteinase	57	+	+	+	+	+	+	+	+	+	+	+	+	+	+	+	+	+	+	+	+	+	+	+	+	+	+	+	+	+	+	+	+	+	+	+	+	+	+	+	+	+	+	+	+	+	+	+	+	+	+	+	+	+
899	1336	hypothetical protein	57	+	+	+	+	+	+	+	+	+	+	+	+	+	+	+	+	+	+	+	+	+	+	+	+	+	+	+	+	+	+	+	+	+	+	+	+	+	+	+	+	+	+	+	+	+	+	+	+	+	+	+	+	+
900	1337	hypothetical protein	57	+	+	+	+	+	+	+	+	+	+	+	+	+	+	+	+	+	+	+	+	+	+	+	+	+	+	+	+	+	+	+	+	+	+	+	+	+	+	+	+	+	+	+	+	+	+	+	+	+	+	+	+	+
901	1338	hypothetical protein	57	+	+	+	+	+	+	+	+	+	+	+	+	+	+	+	+	+	+	+	+	+	+	+	+	+	+	+	+	+	+	+	+	+	+	+	+	+	+	+	+	+	+	+	+	+	+	+	+	+	+	+	+	+
902	1339	hypothetical protein	57	+	+	+	+	+	+	+	+	+	+	+	+	+	+	+	+	+	+	+	+	+	+	+	+	+	+	+	+	+	+	+	+	+	+	+	+	+	+	+	+	+	+	+	+	+	+	+	+	+	+	+	+	+
903	1340	Transporter associated with VraSR	57	+	+	+	+	+	+	+	+	+	+	+	+	+	+	+	+	+	+	+	+	+	+	+	+	+	+	+	+	+	+	+	+	+	+	+	+	+	+	+	+	+	+	+	+	+	+	+	+	+	+	+	+	+
904	1341	ABC transporter permease protein	57	+	+	+	+	+	+	+	+	+	+	+	+	+	+	+	+	+	+	+	+	+	+	+	+	+	+	+	+	+	+	+	+	+	+	+	+	+	+	+	+	+	+	+	+	+	+	+	+	+	+	+	+	+
905	1342	Glycogen biosynthesis protein GlgD, glucose-1-phosphate adenylyltransferase family	57	+	+	+	+	+	+	+	+	+	+	+	+	+	+	+	+	+	+	+	+	+	+	+	+	+	+	+	+	+	+	+	+	+	+	+	+	+	+	+	+	+	+	+	+	+	+	+	+	+	+	+	+	+
906	1343	Dihydrofolate synthase (EC 6.3.2.12) / Folylpolyglutamate synthase (EC 6.3.2.17)	57	+	+	+	+	+	+	+	+	+	+	+	+	+	+	+	+	+	+	+	+	+	+	+	+	+	+	+	+	+	+	+	+	+	+	+	+	+	+	+	+	+	+	+	+	+	+	+	+	+	+	+	+	+
907	1345	Transcriptional regulator in cluster with unspecified monosaccharide ABC transport system	57	+	+	+	+	+	+	+	+	+	+	+	+	+	+	+	+	+	+	+	+	+	+	+	+	+	+	+	+	+	+	+	+	+	+	+	+	+	+	+	+	+	+	+	+	+	+	+	+	+	+	+	+	+
908	1347	membrane protein	57	+	+	+	+	+	+	+	+	+	+	+	+	+	+	+	+	+	+	+	+	+	+	+	+	+	+	+	+	+	+	+	+	+	+	+	+	+	+	+	+	+	+	+	+	+	+	+	+	+	+	+	+	+
909	1348	hypothetical protein	57	+	+	+	+	+	+	+	+	+	+	+	+	+	+	+	+	+	+	+	+	+	+	+	+	+	+	+	+	+	+	+	+	+	+	+	+	+	+	+	+	+	+	+	+	+	+	+	+	+	+	+	+	+
910	1349	hypothetical protein	57	+	+	+	+	+	+	+	+	+	+	+	+	+	+	+	+	+	+	+	+	+	+	+	+	+	+	+	+	+	+	+	+	+	+	+	+	+	+	+	+	+	+	+	+	+	+	+	+	+	+	+	+	+
911	1350	hypothetical protein	57	+	+	+	+	+	+	+	+	+	+	+	+	+	+	+	+	+	+	+	+	+	+	+	+	+	+	+	+	+	+	+	+	+	+	+	+	+	+	+	+	+	+	+	+	+	+	+	+	+	+	+	+	+
912	1351	Esterase/lipase	57	+	+	+	+	+	+	+	+	+	+	+	+	+	+	+	+	+	+	+	+	+	+	+	+	+	+	+	+	+	+	+	+	+	+	+	+	+	+	+	+	+	+	+	+	+	+	+	+	+	+	+	+	+
913	1352	hypothetical protein	57	+	+	+	+	+	+	+	+	+	+	+	+	+	+	+	+	+	+	+	+	+	+	+	+	+	+	+	+	+	+	+	+	+	+	+	+	+	+	+	+	+	+	+	+	+	+	+	+	+	+	+	+	+
914	1353	Hypothetical protein in cluster with Ecs transporter (in Streptococci)	57	+	+	+	+	+	+	+	+	+	+	+	+	+	+	+	+	+	+	+	+	+	+	+	+	+	+	+	+	+	+	+	+	+	+	+	+	+	+	+	+	+	+	+	+	+	+	+	+	+	+	+	+	+
915	1354	Tyrosine recombinase XerD	57	+	+	+	+	+	+	+	+	+	+	+	+	+	+	+	+	+	+	+	+	+	+	+	+	+	+	+	+	+	+	+	+	+	+	+	+	+	+	+	+	+	+	+	+	+	+	+	+	+	+	+	+	+
916	1355	Maltodextrose utilization protein MalA	57	+	+	+	+	+	+	+	+	+	+	+	+	+	+	+	+	+	+	+	+	+	+	+	+	+	+	+	+	+	+	+	+	+	+	+	+	+	+	+	+	+	+	+	+	+	+	+	+	+	+	+	+	+
917	1356	Permease of the major facilitator superfamily	57	+	+	+	+	+	+	+	+	+	+	+	+	+	+	+	+	+	+	+	+	+	+	+	+	+	+	+	+	+	+	+	+	+	+	+	+	+	+	+	+	+	+	+	+	+	+	+	+	+	+	+	+	+
918	1357	hypothetical protein	57	+	+	+	+	+	+	+	+	+	+	+	+	+	+	+	+	+	+	+	+	+	+	+	+	+	+	+	+	+	+	+	+	+	+	+	+	+	+	+	+	+	+	+	+	+	+	+	+	+	+	+	+	+
919	1358	Translation initiation factor 1 (IF-1)	57	+	+	+	+	+	+	+	+	+	+	+	+	+	+	+	+	+	+	+	+	+	+	+	+	+	+	+	+	+	+	+	+	+	+	+	+	+	+	+	+	+	+	+	+	+	+	+	+	+	+	+	+	+
920	1359	Rod shape-determining protein MreC	57	+	+	+	+	+	+	+	+	+	+	+	+	+	+	+	+	+	+	+	+	+	+	+	+	+	+	+	+	+	+	+	+	+	+	+	+	+	+	+	+	+	+	+	+	+	+	+	+	+	+	+	+	+
921	1360	Alcohol dehydrogenase class III (EC 1.1.1.1); Glutathione-dependent formaldehyde dehydrogenase	57	+	+	+	+	+	+	+	+	+	+	+	+	+	+	+	+	+	+	+	+	+	+	+	+	+	+	+	+	+	+	+	+	+	+	+	+	+	+	+	+	+	+	+	+	+	+	+	+	+	+	+	+	+
922	1361	Permease of the major facilitator superfamily	57	+	+	+	+	+	+	+	+	+	+	+	+	+	+	+	+	+	+	+	+	+	+	+	+	+	+	+	+	+	+	+	+	+	+	+	+	+	+	+	+	+	+	+	+	+	+	+	+	+	+	+	+	+
923	1362	D-beta-hydroxybutyrate permease	57	+	+	+	+	+	+	+	+	+	+	+	+	+	+	+	+	+	+	+	+	+	+	+	+	+	+	+	+	+	+	+	+	+	+	+	+	+	+	+	+	+	+	+	+	+	+	+	+	+	+	+	+	+
924	1363	hypothetical protein	57	+	+	+	+	+	+	+	+	+	+	+	+	+	+	+	+	+	+	+	+	+	+	+	+	+	+	+	+	+	+	+	+	+	+	+	+	+	+	+	+	+	+	+	+	+	+	+	+	+	+	+	+	+
925	1364	Acetyltransferase	57	+	+	+	+	+	+	+	+	+	+	+	+	+	+	+	+	+	+	+	+	+	+	+	+	+	+	+	+	+	+	+	+	+	+	+	+	+	+	+	+	+	+	+	+	+	+	+	+	+	+	+	+	+
926	1365	Permease of the major facilitator superfamily	57	+	+	+	+	+	+	+	+	+	+	+	+	+	+	+	+	+	+	+	+	+	+	+	+	+	+	+	+	+	+	+	+	+	+	+	+	+	+	+	+	+	+	+	+	+	+	+	+	+	+	+	+	+
927	1370	Oligopeptide transport system permease protein OppB (TC 3.A.1.5.1)	57	+	+	+	+	+	+	+	+	+	+	+	+	+	+	+	+	+	+	+	+	+	+	+	+	+	+	+	+	+	+	+	+	+	+	+	+	+	+	+	+	+	+	+	+	+	+	+	+	+	+	+	+	+
928	1371	hypothetical protein	57	+	+	+	+	+	+	+	+	+	+	+	+	+	+	+	+	+	+	+	+	+	+	+	+	+	+	+	+	+	+	+	+	+	+	+	+	+	+	+	+	+	+	+	+	+	+	+	+	+	+	+	+	+
929	1372	hypothetical protein	57	+	+	+	+	+	+	+	+	+	+	+	+	+	+	+	+	+	+	+	+	+	+	+	+	+	+	+	+	+	+	+	+	+	+	+	+	+	+	+	+	+	+	+	+	+	+	+	+	+	+	+	+	+
930	1374	hypothetical protein	57	+	+	+	+	+	+	+	+	+	+	+	+	+	+	+	+	+	+	+	+	+	+	+	+	+	+	+	+	+	+	+	+	+	+	+	+	+	+	+	+	+	+	+	+	+	+	+	+	+	+	+	+	+
931	1375	Additional lipoprotein component of predicted cobalamin ECF transporter	57	+	+	+	+	+	+	+	+	+	+	+	+	+	+	+	+	+	+	+	+	+	+	+	+	+	+	+	+	+	+	+	+	+	+	+	+	+	+	+	+	+	+	+	+	+	+	+	+	+	+	+	+	+
932	1377	Inner membrane protein YihY, formerly thought to be RNase BN	57	+	+	+	+	+	+	+	+	+	+	+	+	+	+	+	+	+	+	+	+	+	+	+	+	+	+	+	+	+	+	+	+	+	+	+	+	+	+	+	+	+	+	+	+	+	+	+	+	+	+	+	+	+
933	1378	Histidinol-phosphate aminotransferase (EC 2.6.1.9)	57	+	+	+	+	+	+	+	+	+	+	+	+	+	+	+	+	+	+	+	+	+	+	+	+	+	+	+	+	+	+	+	+	+	+	+	+	+	+	+	+	+	+	+	+	+	+	+	+	+	+	+	+	+
934	1380	hypothetical protein	57	+	+	+	+	+	+	+	+	+	+	+	+	+	+	+	+	+	+	+	+	+	+	+	+	+	+	+	+	+	+	+	+	+	+	+	+	+	+	+	+	+	+	+	+	+	+	+	+	+	+	+	+	+
935	1381	YfaA	57	+	+	+	+	+	+	+	+	+	+	+	+	+	+	+	+	+	+	+	+	+	+	+	+	+	+	+	+	+	+	+	+	+	+	+	+	+	+	+	+	+	+	+	+	+	+	+	+	+	+	+	+	+
936	1382	Aldose 1-epimerase (EC 5.1.3.3)	57	+	+	+	+	+	+	+	+	+	+	+	+	+	+	+	+	+	+	+	+	+	+	+	+	+	+	+	+	+	+	+	+	+	+	+	+	+	+	+	+	+	+	+	+	+	+	+	+	+	+	+	+	+
937	1383	hypothetical protein	57	+	+	+	+	+	+	+	+	+	+	+	+	+	+	+	+	+	+	+	+	+	+	+	+	+	+	+	+	+	+	+	+	+	+	+	+	+	+	+	+	+	+	+	+	+	+	+	+	+	+	+	+	+
938	1385	hypothetical protein	57	+	+	+	+	+	+	+	+	+	+	+	+	+	+	+	+	+	+	+	+	+	+	+	+	+	+	+	+	+	+	+	+	+	+	+	+	+	+	+	+	+	+	+	+	+	+	+	+	+	+	+	+	+
939	1386	Transcriptional regulator, TetR family	57	+	+	+	+	+	+	+	+	+	+	+	+	+	+	+	+	+	+	+	+	+	+	+	+	+	+	+	+	+	+	+	+	+	+	+	+	+	+	+	+	+	+	+	+	+	+	+	+	+	+	+	+	+
940	1387	Histone acetyltransferase HPA2 and related acetyltransferases	57	+	+	+	+	+	+	+	+	+	+	+	+	+	+	+	+	+	+	+	+	+	+	+	+	+	+	+	+	+	+	+	+	+	+	+	+	+	+	+	+	+	+	+	+	+	+	+	+	+	+	+	+	+
941	1388	hypothetical protein	57	+	+	+	+	+	+	+	+	+	+	+	+	+	+	+	+	+	+	+	+	+	+	+	+	+	+	+	+	+	+	+	+	+	+	+	+	+	+	+	+	+	+	+	+	+	+	+	+	+	+	+	+	+
942	1389	hypothetical protein	57	+	+	+	+	+	+	+	+	+	+	+	+	+	+	+	+	+	+	+	+	+	+	+	+	+	+	+	+	+	+	+	+	+	+	+	+	+	+	+	+	+	+	+	+	+	+	+	+	+	+	+	+	+
943	1390	Predicted secreted protein	57	+	+	+	+	+	+	+	+	+	+	+	+	+	+	+	+	+	+	+	+	+	+	+	+	+	+	+	+	+	+	+	+	+	+	+	+	+	+	+	+	+	+	+	+	+	+	+	+	+	+	+	+	+
944	1391	hypothetical protein	57	+	+	+	+	+	+	+	+	+	+	+	+	+	+	+	+	+	+	+	+	+	+	+	+	+	+	+	+	+	+	+	+	+	+	+	+	+	+	+	+	+	+	+	+	+	+	+	+	+	+	+	+	+
945	1392	Dihydrofolate synthase (EC 6.3.2.12) / Folylpolyglutamate synthase (EC 6.3.2.17)	57	+	+	+	+	+	+	+	+	+	+	+	+	+	+	+	+	+	+	+	+	+	+	+	+	+	+	+	+	+	+	+	+	+	+	+	+	+	+	+	+	+	+	+	+	+	+	+	+	+	+	+	+	+
946	1393	Oligopeptide transport ATP-binding protein OppD (TC 3.A.1.5.1)	57	+	+	+	+	+	+	+	+	+	+	+	+	+	+	+	+	+	+	+	+	+	+	+	+	+	+	+	+	+	+	+	+	+	+	+	+	+	+	+	+	+	+	+	+	+	+	+	+	+	+	+	+	+
947	1395	ABC transporter, ATP-binding protein	57	+	+	+	+	+	+	+	+	+	+	+	+	+	+	+	+	+	+	+	+	+	+	+	+	+	+	+	+	+	+	+	+	+	+	+	+	+	+	+	+	+	+	+	+	+	+	+	+	+	+	+	+	+
948	1399	Acetyltransferase, GNAT family	57	+	+	+	+	+	+	+	+	+	+	+	+	+	+	+	+	+	+	+	+	+	+	+	+	+	+	+	+	+	+	+	+	+	+	+	+	+	+	+	+	+	+	+	+	+	+	+	+	+	+	+	+	+
949	1402	hypothetical protein	57	+	+	+	+	+	+	+	+	+	+	+	+	+	+	+	+	+	+	+	+	+	+	+	+	+	+	+	+	+	+	+	+	+	+	+	+	+	+	+	+	+	+	+	+	+	+	+	+	+	+	+	+	+
950	1403	Protein of unknown function DUF975	57	+	+	+	+	+	+	+	+	+	+	+	+	+	+	+	+	+	+	+	+	+	+	+	+	+	+	+	+	+	+	+	+	+	+	+	+	+	+	+	+	+	+	+	+	+	+	+	+	+	+	+	+	+
951	1404	hypothetical protein	57	+	+	+	+	+	+	+	+	+	+	+	+	+	+	+	+	+	+	+	+	+	+	+	+	+	+	+	+	+	+	+	+	+	+	+	+	+	+	+	+	+	+	+	+	+	+	+	+	+	+	+	+	+
952	1405	DUF1801 domain-containing protein	57	+	+	+	+	+	+	+	+	+	+	+	+	+	+	+	+	+	+	+	+	+	+	+	+	+	+	+	+	+	+	+	+	+	+	+	+	+	+	+	+	+	+	+	+	+	+	+	+	+	+	+	+	+
953	1406	Acetyltransferase	57	+	+	+	+	+	+	+	+	+	+	+	+	+	+	+	+	+	+	+	+	+	+	+	+	+	+	+	+	+	+	+	+	+	+	+	+	+	+	+	+	+	+	+	+	+	+	+	+	+	+	+	+	+
954	1407	Predicted membrane protein	57	+	+	+	+	+	+	+	+	+	+	+	+	+	+	+	+	+	+	+	+	+	+	+	+	+	+	+	+	+	+	+	+	+	+	+	+	+	+	+	+	+	+	+	+	+	+	+	+	+	+	+	+	+
955	1410	Histone acetyltransferase HPA2 and related acetyltransferases	57	+	+	+	+	+	+	+	+	+	+	+	+	+	+	+	+	+	+	+	+	+	+	+	+	+	+	+	+	+	+	+	+	+	+	+	+	+	+	+	+	+	+	+	+	+	+	+	+	+	+	+	+	+
956	1411	Beta-propeller domains of methanol dehydrogenase type	57	+	+	+	+	+	+	+	+	+	+	+	+	+	+	+	+	+	+	+	+	+	+	+	+	+	+	+	+	+	+	+	+	+	+	+	+	+	+	+	+	+	+	+	+	+	+	+	+	+	+	+	+	+
957	1413	hypothetical protein	57	+	+	+	+	+	+	+	+	+	+	+	+	+	+	+	+	+	+	+	+	+	+	+	+	+	+	+	+	+	+	+	+	+	+	+	+	+	+	+	+	+	+	+	+	+	+	+	+	+	+	+	+	+
958	1415	Oligopeptide transport system permease protein OppC (TC 3.A.1.5.1)	57	+	+	+	+	+	+	+	+	+	+	+	+	+	+	+	+	+	+	+	+	+	+	+	+	+	+	+	+	+	+	+	+	+	+	+	+	+	+	+	+	+	+	+	+	+	+	+	+	+	+	+	+	+
959	1416	Competence-induced protein Ccs4	57	+	+	+	+	+	+	+	+	+	+	+	+	+	+	+	+	+	+	+	+	+	+	+	+	+	+	+	+	+	+	+	+	+	+	+	+	+	+	+	+	+	+	+	+	+	+	+	+	+	+	+	+	+
960	1417	Colicin lysis protein	57	+	+	+	+	+	+	+	+	+	+	+	+	+	+	+	+	+	+	+	+	+	+	+	+	+	+	+	+	+	+	+	+	+	+	+	+	+	+	+	+	+	+	+	+	+	+	+	+	+	+	+	+	+
961	1418	hypothetical protein	57	+	+	+	+	+	+	+	+	+	+	+	+	+	+	+	+	+	+	+	+	+	+	+	+	+	+	+	+	+	+	+	+	+	+	+	+	+	+	+	+	+	+	+	+	+	+	+	+	+	+	+	+	+
962	1419	Oligopeptide transport ATP-binding protein OppF (TC 3.A.1.5.1)	57	+	+	+	+	+	+	+	+	+	+	+	+	+	+	+	+	+	+	+	+	+	+	+	+	+	+	+	+	+	+	+	+	+	+	+	+	+	+	+	+	+	+	+	+	+	+	+	+	+	+	+	+	+
963	1420	putative sensor histidine kinase	57	+	+	+	+	+	+	+	+	+	+	+	+	+	+	+	+	+	+	+	+	+	+	+	+	+	+	+	+	+	+	+	+	+	+	+	+	+	+	+	+	+	+	+	+	+	+	+	+	+	+	+	+	+
964	1421	Competence-associated EpuA protein	57	+	+	+	+	+	+	+	+	+	+	+	+	+	+	+	+	+	+	+	+	+	+	+	+	+	+	+	+	+	+	+	+	+	+	+	+	+	+	+	+	+	+	+	+	+	+	+	+	+	+	+	+	+
965	1422	Conserved domain protein, histidine-rich	57	+	+	+	+	+	+	+	+	+	+	+	+	+	+	+	+	+	+	+	+	+	+	+	+	+	+	+	+	+	+	+	+	+	+	+	+	+	+	+	+	+	+	+	+	+	+	+	+	+	+	+	+	+
966	1424	Streptococcal histidine triad protein	57	+	+	+	+	+	+	+	+	+	+	+	+	+	+	+	+	+	+	+	+	+	+	+	+	+	+	+	+	+	+	+	+	+	+	+	+	+	+	+	+	+	+	+	+	+	+	+	+	+	+	+	+	+
967	1425	Pyridoxal kinase (EC 2.7.1.35)	57	+	+	+	+	+	+	+	+	+	+	+	+	+	+	+	+	+	+	+	+	+	+	+	+	+	+	+	+	+	+	+	+	+	+	+	+	+	+	+	+	+	+	+	+	+	+	+	+	+	+	+	+	+
968	1426	hypothetical protein	57	+	+	+	+	+	+	+	+	+	+	+	+	+	+	+	+	+	+	+	+	+	+	+	+	+	+	+	+	+	+	+	+	+	+	+	+	+	+	+	+	+	+	+	+	+	+	+	+	+	+	+	+	+
969	1427	hypothetical protein	57	+	+	+	+	+	+	+	+	+	+	+	+	+	+	+	+	+	+	+	+	+	+	+	+	+	+	+	+	+	+	+	+	+	+	+	+	+	+	+	+	+	+	+	+	+	+	+	+	+	+	+	+	+
970	1428	hypothetical protein	57	+	+	+	+	+	+	+	+	+	+	+	+	+	+	+	+	+	+	+	+	+	+	+	+	+	+	+	+	+	+	+	+	+	+	+	+	+	+	+	+	+	+	+	+	+	+	+	+	+	+	+	+	+
971	1430	hypothetical protein	57	+	+	+	+	+	+	+	+	+	+	+	+	+	+	+	+	+	+	+	+	+	+	+	+	+	+	+	+	+	+	+	+	+	+	+	+	+	+	+	+	+	+	+	+	+	+	+	+	+	+	+	+	+
972	1432	hypothetical protein	57	+	+	+	+	+	+	+	+	+	+	+	+	+	+	+	+	+	+	+	+	+	+	+	+	+	+	+	+	+	+	+	+	+	+	+	+	+	+	+	+	+	+	+	+	+	+	+	+	+	+	+	+	+
973	1435	Serine/threonine protein phosphatase family protein	57	+	+	+	+	+	+	+	+	+	+	+	+	+	+	+	+	+	+	+	+	+	+	+	+	+	+	+	+	+	+	+	+	+	+	+	+	+	+	+	+	+	+	+	+	+	+	+	+	+	+	+	+	+
974	1436	hypothetical protein	57	+	+	+	+	+	+	+	+	+	+	+	+	+	+	+	+	+	+	+	+	+	+	+	+	+	+	+	+	+	+	+	+	+	+	+	+	+	+	+	+	+	+	+	+	+	+	+	+	+	+	+	+	+
975	1437	hypothetical protein	57	+	+	+	+	+	+	+	+	+	+	+	+	+	+	+	+	+	+	+	+	+	+	+	+	+	+	+	+	+	+	+	+	+	+	+	+	+	+	+	+	+	+	+	+	+	+	+	+	+	+	+	+	+
976	1439	Lactoylglutathione lyase and related lyases	57	+	+	+	+	+	+	+	+	+	+	+	+	+	+	+	+	+	+	+	+	+	+	+	+	+	+	+	+	+	+	+	+	+	+	+	+	+	+	+	+	+	+	+	+	+	+	+	+	+	+	+	+	+
977	1449	hypothetical protein	57	+	+	+	+	+	+	+	+	+	+	+	+	+	+	+	+	+	+	+	+	+	+	+	+	+	+	+	+	+	+	+	+	+	+	+	+	+	+	+	+	+	+	+	+	+	+	+	+	+	+	+	+	+
978	1450	hypothetical protein	57	+	+	+	+	+	+	+	+	+	+	+	+	+	+	+	+	+	+	+	+	+	+	+	+	+	+	+	+	+	+	+	+	+	+	+	+	+	+	+	+	+	+	+	+	+	+	+	+	+	+	+	+	+
979	1451	Arsenate reductase (EC 1.20.4.1)	57	+	+	+	+	+	+	+	+	+	+	+	+	+	+	+	+	+	+	+	+	+	+	+	+	+	+	+	+	+	+	+	+	+	+	+	+	+	+	+	+	+	+	+	+	+	+	+	+	+	+	+	+	+
980	1455	Substrate-specific component CbrT of predicted cobalamin ECF transporter	57	+	+	+	+	+	+	+	+	+	+	+	+	+	+	+	+	+	+	+	+	+	+	+	+	+	+	+	+	+	+	+	+	+	+	+	+	+	+	+	+	+	+	+	+	+	+	+	+	+	+	+	+	+
981	1458	hypothetical protein	57	+	+	+	+	+	+	+	+	+	+	+	+	+	+	+	+	+	+	+	+	+	+	+	+	+	+	+	+	+	+	+	+	+	+	+	+	+	+	+	+	+	+	+	+	+	+	+	+	+	+	+	+	+
982	1463	extracellular serine protease	57	+	+	+	+	+	+	+	+	+	+	+	+	+	+	+	+	+	+	+	+	+	+	+	+	+	+	+	+	+	+	+	+	+	+	+	+	+	+	+	+	+	+	+	+	+	+	+	+	+	+	+	+	+
983	1464	Phosphoglycerol transferase and related proteins, alkaline phosphatase superfamily	57	+	+	+	+	+	+	+	+	+	+	+	+	+	+	+	+	+	+	+	+	+	+	+	+	+	+	+	+	+	+	+	+	+	+	+	+	+	+	+	+	+	+	+	+	+	+	+	+	+	+	+	+	+
984	1467	Rod shape-determining protein MreD	57	+	+	+	+	+	+	+	+	+	+	+	+	+	+	+	+	+	+	+	+	+	+	+	+	+	+	+	+	+	+	+	+	+	+	+	+	+	+	+	+	+	+	+	+	+	+	+	+	+	+	+	+	+
985	1468	regulatory protein, ArsR	57	+	+	+	+	+	+	+	+	+	+	+	+	+	+	+	+	+	+	+	+	+	+	+	+	+	+	+	+	+	+	+	+	+	+	+	+	+	+	+	+	+	+	+	+	+	+	+	+	+	+	+	+	+
986	1471	Late competence protein ComGG, FIG068335	57	+	+	+	+	+	+	+	+	+	+	+	+	+	+	+	+	+	+	+	+	+	+	+	+	+	+	+	+	+	+	+	+	+	+	+	+	+	+	+	+	+	+	+	+	+	+	+	+	+	+	+	+	+
987	1473	hypothetical protein	57	+	+	+	+	+	+	+	+	+	+	+	+	+	+	+	+	+	+	+	+	+	+	+	+	+	+	+	+	+	+	+	+	+	+	+	+	+	+	+	+	+	+	+	+	+	+	+	+	+	+	+	+	+
988	1482	hypothetical protein	57	+	+	+	+	+	+	+	+	+	+	+	+	+	+	+	+	+	+	+	+	+	+	+	+	+	+	+	+	+	+	+	+	+	+	+	+	+	+	+	+	+	+	+	+	+	+	+	+	+	+	+	+	+
989	1487	Peptidoglycan N-acetylglucosamine deacetylase (EC 3.5.1.-)	57	+	+	+	+	+	+	+	+	+	+	+	+	+	+	+	+	+	+	+	+	+	+	+	+	+	+	+	+	+	+	+	+	+	+	+	+	+	+	+	+	+	+	+	+	+	+	+	+	+	+	+	+	+
990	1492	similar to Uncharacterized protein conserved in bacteria	57	+	+	+	+	+	+	+	+	+	+	+	+	+	+	+	+	+	+	+	+	+	+	+	+	+	+	+	+	+	+	+	+	+	+	+	+	+	+	+	+	+	+	+	+	+	+	+	+	+	+	+	+	+
991	1495	hypothetical protein	57	+	+	+	+	+	+	+	+	+	+	+	+	+	+	+	+	+	+	+	+	+	+	+	+	+	+	+	+	+	+	+	+	+	+	+	+	+	+	+	+	+	+	+	+	+	+	+	+	+	+	+	+	+
992	1497	hypothetical protein	57	+	+	+	+	+	+	+	+	+	+	+	+	+	+	+	+	+	+	+	+	+	+	+	+	+	+	+	+	+	+	+	+	+	+	+	+	+	+	+	+	+	+	+	+	+	+	+	+	+	+	+	+	+
993	1500	hypothetical protein	57	+	+	+	+	+	+	+	+	+	+	+	+	+	+	+	+	+	+	+	+	+	+	+	+	+	+	+	+	+	+	+	+	+	+	+	+	+	+	+	+	+	+	+	+	+	+	+	+	+	+	+	+	+
994	1502	Potassium voltage-gated channel subfamily KQT; possible potassium channel, VIC family	57	+	+	+	+	+	+	+	+	+	+	+	+	+	+	+	+	+	+	+	+	+	+	+	+	+	+	+	+	+	+	+	+	+	+	+	+	+	+	+	+	+	+	+	+	+	+	+	+	+	+	+	+	+
995	1509	hypothetical protein	57	+	+	+	+	+	+	+	+	+	+	+	+	+	+	+	+	+	+	+	+	+	+	+	+	+	+	+	+	+	+	+	+	+	+	+	+	+	+	+	+	+	+	+	+	+	+	+	+	+	+	+	+	+
996	1510	hypothetical protein	57	+	+	+	+	+	+	+	+	+	+	+	+	+	+	+	+	+	+	+	+	+	+	+	+	+	+	+	+	+	+	+	+	+	+	+	+	+	+	+	+	+	+	+	+	+	+	+	+	+	+	+	+	+
997	1511	hypothetical protein	57	+	+	+	+	+	+	+	+	+	+	+	+	+	+	+	+	+	+	+	+	+	+	+	+	+	+	+	+	+	+	+	+	+	+	+	+	+	+	+	+	+	+	+	+	+	+	+	+	+	+	+	+	+
998	1520	BlpT protein, fusion	57	+	+	+	+	+	+	+	+	+	+	+	+	+	+	+	+	+	+	+	+	+	+	+	+	+	+	+	+	+	+	+	+	+	+	+	+	+	+	+	+	+	+	+	+	+	+	+	+	+	+	+	+	+
999	17	ABC transporter permease protein	56	+	+	+	+	+	+	+	+	+	+	+	+	+	+	+	+	+	+	+	−	+	+	+	+	+	+	+	+	+	+	+	+	+	+	+	+	+	+	+	+	+	+	+	+	+	+	+	+	+	+	+	+	+	+	+	+	+
1000	21	Glycerol uptake facilitator protein	56	+	+	+	+	+	+	+	+	+	+	+	+	+	+	+	+	+	+	+	+	+	+	+	+	+	+	+	+	+	+	+	+	+	+	+	+	+	+	+	+	+	+	+	+	+	−	+	+	+	+	+	+	+
1001	25	Galactose-6-phosphate isomerase, LacB subunit (EC 5.3.1.26)	56	+	+	+	+	+	+	+	+	+	+	+	+	+	+	+	+	+	+	+	+	+	+	+	+	+	+	+	+	+	+	+	+	+	+	+	+	+	+	+	+	+	+	+	+	−	+	+	+	+	+	+	+	+
1002	33	Galactose-6-phosphate isomerase, LacA subunit (EC 5.3.1.26)	56	+	+	+	+	+	+	+	+	+	+	+	+	+	+	+	+	+	+	+	+	+	+	+	+	+	+	+	+	+	+	+	+	+	+	+	+	+	+	+	+	+	+	+	+	−	+	+	+	+	+	+	+	+
1003	42	6-phospho-beta-galactosidase (EC 3.2.1.85)	56	+	+	+	+	+	+	+	+	+	+	+	+	+	+	+	+	+	+	+	+	+	+	+	+	+	+	+	+	+	+	+	+	+	+	+	+	+	+	+	+	+	+	+	+	−	+	+	+	+	+	+	+	+
1004	45	Tagatose-6-phosphate kinase (EC 2.7.1.144) / 1-phosphofructokinase (EC 2.7.1.56)	56	+	+	+	+	+	+	+	+	+	+	+	+	+	+	+	+	+	+	+	+	+	+	+	+	+	+	+	+	+	+	+	+	+	+	+	+	+	+	+	+	+	+	+	+	−	+	+	+	+	+	+	+	+
1005	46	Methionine ABC transporter substrate-binding protein	56	+	+	+	+	+	+	+	+	+	−	+	+	+	+	+	+	+	+	+	+	+	+	+	+	+	+	+	+	+	+	+	+	+	+	+	+	+	+	+	+	+	+	+	+	+	+	+	+	+	+	+	+	+	+	+	+	+
1006	49	PTS system, mannose-specific IIB component (EC 2.7.1.69) / PTS system, mannose-specific IIA component (EC 2.7.1.69)	56	+	+	+	+	+	+	+	+	+	+	+	+	+	+	+	+	+	+	+	−	+	+	+	+	+	+	+	+	+	+	+	+	+	+	+	+	+	+	+	+	+	+	+	+	+	+	+	+	+	+	+	+	+	+	+	+	+
1007	50	PTS system, mannose-specific IIC component (EC 2.7.1.69)	56	+	+	+	+	+	+	+	+	+	+	+	+	+	+	+	+	+	+	+	−	+	+	+	+	+	+	+	+	+	+	+	+	+	+	+	+	+	+	+	+	+	+	+	+	+	+	+	+	+	+	+	+	+	+	+	+	+
1008	54	PTS system, mannose-specific IID component (EC 2.7.1.69)	56	+	+	+	+	+	+	+	+	+	+	+	+	+	+	+	+	+	+	+	−	+	+	+	+	+	+	+	+	+	+	+	+	+	+	+	+	+	+	+	+	+	+	+	+	+	+	+	+	+	+	+	+	+	+	+	+	+
1009	61	PTS system, lactose-specific IIB component (EC 2.7.1.69) / PTS system, lactose-specific IIC component (EC 2.7.1.69)	56	+	+	+	+	+	+	+	+	+	+	+	+	+	+	+	+	+	+	+	+	+	+	+	+	+	+	+	+	+	+	+	+	+	+	+	+	+	+	+	+	+	+	+	+	−	+	+	+	+	+	+	+	+
1010	75	3-oxoacyl-[acyl-carrier-protein] synthase, KASII (EC 2.3.1.41)	56	+	+	+	+	+	+	+	+	+	+	+	+	+	+	+	+	+	+	+	+	+	+	+	+	+	+	+	+	+	+	+	+	+	+	+	+	+	+	+	+	+	+	+	+	+	+	+	+	+	+	+	−	+
1011	77	Cobalt-zinc-cadmium resistance protein	56	+	+	+	+	+	+	+	+	+	+	+	+	+	+	+	+	+	+	+	−	+	+	+	+	+	+	+	+	+	+	+	+	+	+	+	+	+	+	+	+	+	+	+	+	+	+	+	+	+	+	+	+	+	+	+	+	+
1012	132	Glutamyl-tRNA synthetase (EC 6.1.1.17) @ Glutamyl-tRNA(Gln) synthetase (EC 6.1.1.24)	56	+	+	+	+	+	+	+	+	+	+	+	+	+	+	+	+	+	+	+	+	+	+	+	+	+	+	+	+	+	+	+	+	+	+	+	+	+	+	+	+	+	+	+	+	+	−	+	+	+	+	+	+	+
1013	314	PTS system, lactose-specific IIA component (EC 2.7.1.69)	56	+	+	+	+	+	+	+	+	+	+	+	+	+	+	+	+	+	+	+	+	+	+	+	+	+	+	+	+	+	+	+	+	+	+	+	+	+	+	+	+	+	+	+	+	−	+	+	+	+	+	+	+	+
1014	398	Manganese superoxide dismutase (EC 1.15.1.1)	56	+	+	+	+	+	+	+	+	+	+	+	+	+	+	+	+	+	+	+	−	+	+	+	+	+	+	+	+	+	+	+	+	+	+	+	+	+	+	+	+	+	+	+	+	+	+	+	+	+	+	+	+	+	+	+	+	+
1015	414	TPR-repeat-containing protein	56	+	+	+	+	+	+	+	+	+	+	+	+	+	+	+	+	+	+	+	+	+	+	+	+	+	+	+	+	+	+	+	+	+	+	+	+	+	+	+	+	+	+	+	+	+	+	+	+	+	+	+	−	+

Table S5. Precence/absence of each HG among 57 *S. suis* strains

				1																13			25					28						104		94			76			53/54		Divergent *S. suis*																
Index[a]	ClusterID	Gene products	Members	98HAH33	05ZYH33	SC84	A7	SS12	P1/7	DAT273	NIAH11434	JS14	GZ1	BM407	S735	13730	MNCM07	NCTC 10446	14636	NCTC 10237	ST1	2651	89-1591	MNCM04	MNCM25	8074	D9	4961	ST3	MNCM43	DAT292	DAT260	NIAH11435	MNCM21	MNCM50	89-2479	DAT234	DAT290	93A	42A	NT77	6407	11538	8830	12814	92-1400	89-590	2726	2524	89-3576-3	4417	D12	10581	89-5259	88-5299A	R61	92-1191	14A	92-4172	22083
1016	422	SSU ribosomal protein S12p (S23e)	56	+	+	+	+	+	+	+	+	+	+	+	+	+	+	+	+	+	+	+	–	+	+	+	+	+	+	+	+	+	+	+	+	+	+	+	+	+	+	+	+	+	+	+	+	+	+	+	+	+	+	+	+	+	+	+	+	+
1017	424	ATP-dependent Clp protease, ATP-binding subunit ClpE	56	+	+	+	+	+	+	+	+	+	+	+	–	+	+	+	+	+	+	+	+	+	+	+	+	+	+	+	+	+	+	+	+	+	+	+	+	+	+	+	+	+	+	+	+	+	+	+	+	+	+	+	+	+	+	+	+	+
1018	439	Dimethyladenosine transferase (EC 2.1.1.-)	56	+	+	+	+	+	+	+	+	+	+	+	+	+	+	+	+	+	+	+	+	+	+	+	+	+	+	+	+	+	+	+	+	+	+	+	+	+	+	+	+	+	+	+	+	+	+	+	+	+	+	+	–	+
1019	440	GTP pyrophosphokinase (EC 2.7.6.5), (p)ppGpp synthetase I	56	+	+	+	+	+	+	+	+	+	+	+	+	+	+	+	+	+	+	+	–	+	+	+	+	+	+	+	+	+	+	+	+	+	+	+	+	+	+	+	+	+	+	+	+	+	+	+	+	+	+	+	+	+	+	+	+	+
1020	448	Phosphopantetheine adenylyltransferase (EC 2.7.7.3)	56	+	+	+	+	+	+	+	+	+	+	+	+	+	+	+	+	+	+	+	–	+	+	+	+	+	+	+	+	+	+	+	+	+	+	+	+	+	+	+	+	+	+	+	+	+	+	+	+	+	+	+	+	+	+	+	+	+
1021	451	Transketolase (EC 2.2.1.1)	56	+	+	+	+	+	+	+	+	+	+	+	+	+	+	+	+	+	+	+	–	+	+	+	+	+	+	+	+	+	+	+	+	+	+	+	+	+	+	+	+	+	+	+	+	+	+	+	+	+	+	+	+	+	+	+	+	+
1022	453	Glutamate racemase (EC 5.1.1.3)	56	+	+	+	+	+	+	+	+	+	+	+	+	+	+	+	+	+	+	+	+	+	+	+	+	+	+	+	+	+	+	+	+	+	+	+	+	+	–	+	+	+	+	+	+	+	+	+	+	+	+	+	+	+	+	+	+	+
1023	459	Lon-like protease with PDZ domain	56	+	+	+	+	+	+	+	+	+	+	+	+	+	+	+	+	+	+	+	+	+	+	+	+	+	+	+	+	+	+	+	+	+	+	+	+	–	+	+	+	+	+	+	+	+	+	+	+	+	+	+	+	+	+	+	+	+
1024	465	Triosephosphate isomerase (EC 5.3.1.1)	56	+	+	+	+	+	+	+	+	+	+	+	+	+	+	+	+	+	+	+	–	+	+	+	+	+	+	+	+	+	+	+	+	+	+	+	+	+	+	+	+	+	+	+	+	+	+	+	+	+	+	+	+	+	+	+	+	+
1025	466	LSU ribosomal protein L21p	56	+	+	+	+	+	+	+	+	+	+	+	+	+	+	+	+	+	+	+	–	+	+	+	+	+	+	+	+	+	+	+	+	+	+	+	+	+	+	+	+	+	+	+	+	+	+	+	+	+	+	+	+	+	+	+	+	+
1026	467	Cystathionine beta-lyase (EC 4.4.1.8)	56	+	+	+	+	+	+	+	+	+	+	+	+	+	+	+	+	+	+	+	+	+	+	+	+	+	+	+	+	+	+	+	+	+	+	+	+	+	+	–	+	+	+	+	+	+	+	+	+	+	+	+	+	+	+	+	+	+
1027	471	SSU ribosomal protein S5p (S2e)	56	+	+	+	+	+	+	+	+	+	+	+	+	+	+	+	+	+	+	+	+	+	+	+	+	+	+	+	+	+	+	+	+	+	+	+	+	+	+	+	+	+	+	+	+	+	+	+	+	+	+	+	–	+
1028	474	Iron-sulfur cluster assembly protein SufD	56	+	+	+	+	+	+	+	+	+	+	+	+	+	+	+	+	+	+	+	+	+	+	+	+	+	+	+	+	+	+	+	+	+	+	+	+	+	+	+	+	+	+	+	+	+	+	+	+	+	+	+	–	+
1029	480	tRNA S(4)U 4-thiouridine synthase (former ThiI)	56	+	+	+	+	+	+	+	+	+	+	+	+	+	+	+	+	+	+	+	+	+	+	+	+	+	+	+	+	+	+	+	+	+	+	+	+	+	+	+	+	+	+	+	+	+	+	+	+	+	+	+	–	+
1030	484	Low molecular weight protein tyrosine phosphatase (EC 3.1.3.48)	56	+	+	+	+	+	+	+	+	+	+	+	+	+	+	+	+	+	+	+	–	+	+	+	+	+	+	+	+	+	+	+	+	+	+	+	+	+	+	+	+	+	+	+	+	+	+	+	+	+	+	+	+	+	+	+	+	+
1031	486	ATP-dependent nuclease, subunit A	56	+	+	+	+	+	+	+	+	+	+	–	+	+	+	+	+	+	+	+	+	+	+	+	+	+	+	+	+	+	+	+	+	+	+	+	+	+	+	+	+	+	+	+	+	+	+	+	+	+	+	+	+	+	+	+	+	+
1032	488	Trans-2,cis-3-Decenoyl-ACP isomerase	56	+	+	+	+	+	+	+	+	+	+	+	+	+	+	+	+	+	+	+	–	+	+	+	+	+	+	+	+	+	+	+	+	+	+	+	+	+	+	+	+	+	+	+	+	+	+	+	+	+	+	+	+	+	+	+	+	+
1033	490	Glucose-6-phosphate isomerase (EC 5.3.1.9)	56	+	+	+	+	+	+	+	+	+	+	+	+	+	+	+	+	+	+	+	–	+	+	+	+	+	+	+	+	+	+	+	+	+	+	+	+	+	+	+	+	+	+	+	+	+	+	+	+	+	+	+	+	+	+	+	+	+
1034	494	Cysteine desulfurase (EC 2.8.1.7)	56	+	+	+	+	+	+	+	+	+	+	+	+	+	+	+	+	+	+	+	+	+	+	+	+	+	+	+	+	+	+	+	+	+	+	+	+	+	–	+	+	+	+	+	+	+	+	+	+	+	+	+	+	+	+	+	+	+
1035	495	Amino acid ABC transporter, glutamine-binding protein/permease protein	56	+	+	+	+	+	+	+	+	+	+	+	+	+	+	+	+	+	+	+	–	+	+	+	+	+	+	+	+	+	+	+	+	+	+	+	+	+	+	+	+	+	+	+	+	+	+	+	+	+	+	+	+	+	+	+	+	+
1036	498	Helicase PriA essential for oriC/DnaA-independent DNA replication	56	+	+	+	+	+	+	+	+	+	+	+	+	+	+	+	+	+	+	+	+	+	+	+	+	+	+	+	+	+	+	+	+	+	+	+	+	+	+	+	+	+	+	+	+	+	+	+	+	+	+	+	–	+
1037	503	Serine acetyltransferase (EC 2.3.1.30)	56	+	+	+	+	+	+	+	+	+	+	+	+	+	+	+	+	+	+	+	+	+	+	+	+	+	+	+	+	+	+	+	+	+	+	+	+	+	+	+	+	+	+	+	+	+	+	+	+	+	+	+	–	+
1038	511	Acyl-ACP thioesterase	56	+	+	+	+	+	+	+	+	+	+	+	+	+	+	+	+	+	+	+	+	+	+	+	+	+	+	+	+	+	+	+	+	+	+	+	+	+	+	+	+	+	+	+	+	+	+	+	+	–	+	+	+	+
1039	514	COG0536: GTP-binding protein Obg	56	+	+	+	+	+	+	+	+	+	+	+	+	+	+	+	+	+	+	+	+	+	+	+	+	+	+	+	+	+	+	+	+	+	+	+	+	+	+	+	+	+	+	+	+	+	+	+	+	+	+	+	–	+
1040	522	DNA polymerase III polC-type (EC 2.7.7.7)	56	+	+	+	+	+	+	+	+	+	+	+	+	+	+	+	+	+	+	+	+	+	+	+	+	+	+	+	+	+	+	+	+	+	+	+	+	+	+	+	+	+	+	+	+	+	+	+	+	+	+	+	–	+
1041	523	LSU ribosomal protein L27p	56	+	+	+	+	+	+	+	+	+	+	+	+	+	+	+	+	+	+	+	–	+	+	+	+	+	+	+	+	+	+	+	+	+	+	+	+	+	+	+	+	+	+	+	+	+	+	+	+	+	+	+	+	+	+	+	+	+
1042	524	SSU ribosomal protein S9p (S16e)	56	+	+	+	+	+	+	+	+	+	+	+	+	+	+	+	+	+	+	+	–	+	+	+	+	+	+	+	+	+	+	+	+	+	+	+	+	+	+	+	+	+	+	+	+	+	+	+	+	+	+	+	+	+	+	+	+	+
1043	526	Peptide chain release factor 3	56	+	+	+	+	+	+	+	+	+	+	+	+	+	+	+	+	+	+	+	+	+	+	+	+	+	+	+	+	+	+	+	+	+	+	+	+	+	+	+	+	+	+	+	+	+	+	+	+	+	+	+	–	+
1044	528	hypothetical protein	56	+	+	+	+	+	+	+	+	+	+	+	+	+	+	+	+	+	+	+	+	+	+	+	+	+	+	+	+	+	+	+	+	–	+	+	+	+	+	+	+	+	+	+	+	+	+	+	+	+	+	+	+	+	+	+	+	+
1045	534	Peptide deformylase (EC 3.5.1.88)	56	+	+	+	+	+	+	+	+	+	+	+	+	+	+	+	+	+	+	+	+	+	+	+	+	+	+	+	+	+	+	+	+	+	+	+	+	+	+	+	+	+	+	+	+	+	+	+	+	+	+	+	–	+
1046	535	LSU ribosomal protein L19p	56	+	+	+	+	+	+	+	+	+	+	+	+	+	+	+	+	+	+	+	–	+	+	+	+	+	+	+	+	+	+	+	+	+	+	+	+	+	+	+	+	+	+	+	+	+	+	+	+	+	+	+	+	+	+	+	+	+
1047	540	DNA-directed RNA polymerase beta subunit (EC 2.7.7.6)	56	+	+	+	+	+	+	+	+	+	+	+	+	+	+	+	+	+	+	+	+	+	+	+	+	+	+	+	+	+	+	+	+	+	+	+	+	+	+	+	+	+	+	+	+	+	+	+	+	+	+	+	–	+
1048	544	Multi antimicrobial extrusion (MATE) family transporter	56	+	+	+	+	+	+	+	+	+	+	+	+	+	+	+	+	+	+	+	+	+	+	+	+	+	+	+	+	+	+	+	+	+	+	+	+	+	+	+	+	+	–	+	+	+	+	+	+	+	+	+	+	+
1049	547	UDP-N-acetylglucosamine 1-carboxyvinyltransferase (EC 2.5.1.7)	56	+	+	+	+	+	+	+	+	+	+	+	+	+	+	+	–	+	+	+	+	+	+	+	+	+	+	+	+	+	+	+	+	+	+	+	+	+	+	+	+	+	+	+	+	+	+	+	+	+	+	+	+	+	+	+	+	+
1050	548	rRNA small subunit methyltransferase H	56	+	+	+	+	+	+	+	+	+	+	+	+	+	+	+	+	+	+	+	+	+	+	+	+	+	+	+	+	+	+	+	+	+	+	+	+	+	+	+	+	+	+	+	+	+	+	+	+	+	+	+	–	+
1051	549	Pyruvate formate-lyase activating enzyme (EC 1.97.1.4)	56	+	+	+	+	+	+	+	+	+	+	+	+	+	+	+	+	+	+	+	–	+	+	+	+	+	+	+	+	+	+	+	+	+	+	+	+	+	+	+	+	+	+	+	+	+	+	+	+	+	+	+	+	+	+	+	+	+
1052	551	LSU ribosomal protein L28p	56	+	+	+	+	+	+	+	+	+	+	+	+	+	+	+	+	+	+	+	–	+	+	+	+	+	+	+	+	+	+	+	+	+	+	+	+	+	+	+	+	+	+	+	+	+	+	+	+	+	+	+	+	+	+	+	+	+
1053	554	Mutator mutT protein (7,8-dihydro-8-oxoguanine-triphosphatase) (EC 3.6.1.-)	56	+	+	+	+	+	+	+	+	+	+	+	–	+	+	+	+	+	+	+	+	+	+	+	+	+	+	+	+	+	+	+	+	+	+	+	+	+	+	+	+	+	+	+	+	+	+	+	+	+	+	+	+	+	+	+	+	+
1054	556	SSU ribosomal protein S7p (S5e)	56	+	+	+	+	+	+	+	+	+	+	+	+	+	+	+	+	+	+	+	–	+	+	+	+	+	+	+	+	+	+	+	+	+	+	+	+	+	+	+	+	+	+	+	+	+	+	+	+	+	+	+	+	+	+	+	+	+
1055	557	tRNA (5-methylaminomethyl-2-thiouridylate)-methyltransferase (EC 2.1.1.61)	56	+	+	+	+	+	+	+	+	+	+	+	+	+	+	+	+	+	+	+	+	+	+	+	+	+	+	+	+	+	+	+	+	+	+	+	+	+	+	+	+	+	+	+	+	+	+	+	+	+	+	+	–	+
1056	561	D-tyrosyl-tRNA(Tyr) deacylase	56	+	+	+	+	+	+	+	+	+	+	+	+	+	+	+	+	+	+	+	–	+	+	+	+	+	+	+	+	+	+	+	+	+	+	+	+	+	+	+	+	+	+	+	+	+	+	+	+	+	+	+	+	+	+	+	+	+
1057	564	Ribonuclease HII (EC 3.1.26.4)	56	+	+	+	+	+	+	+	+	+	+	+	+	+	+	+	+	+	+	+	+	+	+	+	+	+	+	+	+	+	+	+	+	+	+	+	+	+	+	+	+	+	+	+	+	+	+	+	+	+	+	+	–	+
1058	565	CDP-diacylglycerol--glycerol-3-phosphate 3-phosphatidyltransferase (EC 2.7.8.5)	56	+	+	+	+	+	+	+	+	+	+	+	+	+	+	+	+	+	+	+	–	+	+	+	+	+	+	+	+	+	+	+	+	+	+	+	+	+	+	+	+	+	+	+	+	+	+	+	+	+	+	+	+	+	+	+	+	+
1059	575	Phosphatase YidA	56	+	+	+	+	+	+	+	+	+	+	+	+	+	+	+	+	+	+	+	–	+	+	+	+	+	+	+	+	+	+	+	+	+	+	+	+	+	+	+	+	+	+	+	+	+	+	+	+	+	+	+	+	+	+	+	+	+
1060	576	Signal recognition particle, subunit Ffh SRP54 (TC 3.A.5.1.1)	56	+	+	+	+	+	+	+	+	+	+	+	+	+	+	+	+	+	+	+	+	+	+	+	+	+	+	+	+	+	+	+	+	+	+	+	+	–	+	+	+	+	+	+	+	+	+	+	+	+	+	+	+	+	+	+	+	+
1061	583	Multimodular transpeptidase-transglycosylase (EC 2.4.1.129) (EC 3.4.-.-)	56	+	+	+	+	+	+	+	+	+	+	+	+	+	+	+	+	+	+	+	–	+	+	+	+	+	+	+	+	+	+	+	+	+	+	+	+	+	+	+	+	+	+	+	+	+	+	+	+	+	+	+	+	+	+	+	+	+
1062	586	LSU ribosomal protein L23p (L23Ae)	56	+	+	+	+	+	+	+	+	+	+	+	+	+	+	+	+	+	+	+	–	+	+	+	+	+	+	+	+	+	+	+	+	+	+	+	+	+	+	+	+	+	+	+	+	+	+	+	+	+	+	+	+	+	+	+	+	+
1063	587	Glycyl-tRNA synthetase beta chain (EC 6.1.1.14)	56	+	+	+	+	+	+	+	+	+	+	+	+	+	+	+	+	+	+	+	+	+	+	+	+	+	+	+	+	+	+	+	+	+	+	+	+	+	+	+	+	+	+	+	+	+	+	+	+	+	+	+	–	+
1064	588	Protein export cytoplasm protein SecA ATPase RNA helicase (TC 3.A.5.1.1)	56	+	+	+	+	+	+	+	+	+	+	+	+	+	+	+	+	+	+	+	+	+	+	+	+	+	+	+	+	+	+	+	+	+	+	+	+	+	+	+	+	+	+	+	+	+	+	+	+	+	+	+	–	+
1065	590	Similar to ribosomal large subunit pseudouridine synthase D, Bacillus subtilis YhcT type	56	+	+	+	+	+	+	+	+	+	+	+	+	+	+	+	+	+	+	+	+	+	+	+	+	+	+	+	+	+	+	+	+	+	+	+	+	+	–	+	+	+	+	+	+	+	+	+	+	+	+	+	+	+	+	+	+	+
1066	592	Translation elongation factor Tu	56	+	+	+	+	+	+	+	+	+	+	+	+	+	+	+	+	+	+	+	–	+	+	+	+	+	+	+	+	+	+	+	+	+	+	+	+	+	+	+	+	+	+	+	+	+	+	+	+	+	+	+	+	+	+	+	+	+
1067	594	R801 tRNA-Arg	56	+	+	+	+	+	+	+	+	+	+	+	+	+	+	+	+	+	+	+	–	+	+	+	+	+	+	+	+	+	+	+	+	+	+	+	+	+	+	+	+	+	+	+	+	+	+	+	+	+	+	+	+	+	+	+	+	+
1068	599	Translation elongation factor LepA	56	+	+	+	+	+	+	+	+	+	+	+	+	+	+	+	+	+	+	+	+	+	+	+	+	+	+	+	+	+	+	+	+	+	+	+	+	+	+	+	+	+	+	+	+	+	+	+	+	+	+	+	–	+
1069	601	Inosine-5'-monophosphate dehydrogenase (EC 1.1.1.205)	56	+	+	+	+	+	+	+	+	+	+	+	+	+	+	+	+	+	+	+	–	+	+	+	+	+	+	+	+	+	+	+	+	+	+	+	+	+	+	+	+	+	+	+	+	+	+	+	+	+	+	+	+	+	+	+	+	+
1070	602	hypothetical protein	56	+	+	+	+	+	+	+	+	+	+	+	–	+	+	+	+	+	+	+	+	+	+	+	+	+	+	+	+	+	+	+	+	+	+	+	+	+	+	+	+	+	+	+	+	+	+	+	+	+	+	+	+	+	+	+	+	+
1071	603	Orotidine 5'-phosphate decarboxylase (EC 4.1.1.23)	56	+	+	+	+	+	+	+	+	+	+	+	+	+	+	+	+	+	+	+	–	+	+	+	+	+	+	+	+	+	+	+	+	+	+	+	+	+	+	+	+	+	+	+	+	+	+	+	+	+	+	+	+	+	+	+	+	+
1072	605	Transcription antitermination protein NusG	56	+	+	+	+	+	+	+	+	+	+	+	+	+	+	+	+	+	+	+	–	+	+	+	+	+	+	+	+	+	+	+	+	+	+	+	+	+	+	+	+	+	+	+	+	+	+	+	+	+	+	+	+	+	+	+	+	+
1073	606	Leucyl-tRNA synthetase (EC 6.1.1.4)	56	+	+	+	+	+	+	+	+	+	+	+	+	+	+	+	+	+	+	+	+	+	+	+	+	+	+	+	+	+	+	+	+	+	+	+	+	+	+	+	+	+	+	+	+	+	+	+	+	+	+	+	–	+
1074	610	Orotate phosphoribosyltransferase (EC 2.4.2.10)	56	+	+	+	+	+	+	+	+	+	+	+	+	+	+	+	+	+	+	+	–	+	+	+	+	+	+	+	+	+	+	+	+	+	+	+	+	+	+	+	+	+	+	+	+	+	+	+	+	+	+	+	+	+	+	+	+	+
1075	612	Hypothetical protein ywlG	56	+	+	+	+	+	+	+	+	+	+	+	+	+	+	+	+	+	+	+	+	+	+	+	+	+	+	+	+	+	+	+	+	+	+	+	+	+	+	+	+	+	+	+	+	+	+	+	+	+	+	+	–	+
1076	615	Topoisomerase IV subunit A (EC 5.99.1.-)	56	+	+	+	+	+	+	+	+	+	+	+	+	+	+	+	+	+	+	+	+	+	+	+	+	+	+	+	+	+	+	+	+	+	+	+	+	+	+	+	+	+	+	+	+	+	+	+	+	+	+	+	–	+
1077	616	UDP-N-acetylmuramoylalanyl-D-glutamyl-2,6-diaminopimelate--D-alanyl-D-alanine ligase (EC 6.3.2.10)	56	+	+	+	+	+	+	+	+	+	–	+	+	+	+	+	+	+	+	+	+	+	+	+	+	+	+	+	+	+	+	+	+	+	+	+	+	+	+	+	+	+	+	+	+	+	+	+	+	+	+	+	+	+	+	+	+	+
1078	617	Fructose-bisphosphate aldolase class II (EC 4.1.2.13)	56	+	+	+	+	+	+	+	+	+	+	+	+	+	+	+	+	+	+	+	–	+	+	+	+	+	+	+	+	+	+	+	+	+	+	+	+	+	+	+	+	+	+	+	+	+	+	+	+	+	+	+	+	+	+	+	+	+
1079	618	Tryptophanyl-tRNA synthetase (EC 6.1.1.2)	56	+	+	+	+	+	+	+	+	+	+	+	+	+	+	+	+	+	+	+	–	+	+	+	+	+	+	+	+	+	+	+	+	+	+	+	+	+	+	+	+	+	+	+	+	+	+	+	+	+	+	+	+	+	+	+	+	+
1080	619	Putative regulator of the mannose operon, ManO	56	+	+	+	+	+	+	+	+	+	+	+	+	+	+	+	+	+	+	+	–	+	+	+	+	+	+	+	+	+	+	+	+	+	+	+	+	+	+	+	+	+	+	+	+	+	+	+	+	+	+	+	+	+	+	+	+	+
1081	620	Translation initiation factor 3	56	+	+	+	+	+	+	+	+	+	+	+	+	+	+	+	+	+	+	+	–	+	+	+	+	+	+	+	+	+	+	+	+	+	+	+	+	+	+	+	+	+	+	+	+	+	+	+	+	+	+	+	+	+	+	+	+	+
1082	621	KH domain RNA binding protein YlqC	56	+	+	+	+	+	+	+	+	+	+	+	+	+	+	+	+	+	+	+	–	+	+	+	+	+	+	+	+	+	+	+	+	+	+	+	+	+	+	+	+	+	+	+	+	+	+	+	+	+	+	+	+	+	+	+	+	+
1083	623	Branched-chain amino acid aminotransferase (EC 2.6.1.42)	56	+	+	+	+	+	+	+	+	+	+	+	+	+	+	+	+	+	+	+	–	+	+	+	+	+	+	+	+	+	+	+	+	+	+	+	+	+	+	+	+	+	+	+	+	+	+	+	+	+	+	+	+	+	+	+	+	+
1084	628	LSU ribosomal protein L20p	56	+	+	+	+	+	+	+	+	+	+	+	+	+	+	+	+	+	+	+	–	+	+	+	+	+	+	+	+	+	+	+	+	+	+	+	+	+	+	+	+	+	+	+	+	+	+	+	+	+	+	+	+	+	+	+	+	+
1085	629	N-acetylglucosamine-6-phosphate deacetylase (EC 3.5.1.25)	56	+	+	+	+	+	+	+	+	+	+	+	+	+	+	+	+	+	+	+	–	+	+	+	+	+	+	+	+	+	+	+	+	+	+	+	+	+	+	+	+	+	+	+	+	+	+	+	+	+	+	+	+	+	+	+	+	+
1086	630	DNA-directed RNA polymerase delta subunit (EC 2.7.7.6)	56	+	+	+	+	+	+	+	+	+	+	+	+	+	+	+	+	+	+	+	–	+	+	+	+	+	+	+	+	+	+	+	+	+	+	+	+	+	+	+	+	+	+	+	+	+	+	+	+	+	+	+	+	+	+	+	+	+
1087	634	Peptide methionine sulfoxide reductase MsrA (EC 1.8.4.11) / Peptide methionine sulfoxide reductase MsrB (EC 1.8.4.12)	56	+	+	+	+	+	+	+	+	+	+	+	+	+	+	+	+	+	+	+	+	+	+	+	+	+	+	+	+	+	+	+	+	+	+	+	+	+	+	+	+	+	+	+	+	+	+	+	+	+	+	+	–	+
1088	635	LSU ribosomal protein L3p (L3e)	56	+	+	+	+	+	+	+	+	+	+	+	+	+	+	+	+	+	+	+	–	+	+	+	+	+	+	+	+	+	+	+	+	+	+	+	+	+	+	+	+	+	+	+	+	+	+	+	+	+	+	+	+	+	+	+	+	+
1089	636	hypothetical protein	56	+	+	+	+	+	+	+	+	+	+	+	+	+	+	+	+	+	+	+	–	+	+	+	+	+	+	+	+	+	+	+	+	+	+	+	+	+	+	+	+	+	+	+	+	+	+	+	+	+	+	+	+	+	+	+	+	+
1090	637	Tyrosine recombinase XerC	56	+	+	+	+	+	+	+	+	+	+	+	+	+	+	+	+	+	+	+	+	+	+	+	+	+	+	+	+	+	+	+	+	+	+	+	+	+	+	+	+	+	+	+	+	+	+	+	+	+	+	+	–	+
1091	639	RNA methyltransferase, TrmA family	56	+	+	+	+	+	+	+	+	+	+	+	+	+	+	+	+	+	+	+	+	+	+	+	+	+	+	+	+	+	+	+	+	+	+	+	+	+	+	+	+	+	+	+	+	+	+	+	+	+	+	–	+	+
1092	640	Alkylphosphonate utilization operon protein PhnA	56	+	+	+	+	+	+	+	+	+	+	+	+	+	+	+	+	+	+	+	–	+	+	+	+	+	+	+	+	+	+	+	+	+	+	+	+	+	+	+	+	+	+	+	+	+	+	+	+	+	+	+	+	+	+	+	+	+
1093	643	Hydroxymethylpyrimidine phosphate kinase ThiD (EC 2.7.4.7) / Thiamin-phosphate pyrophosphorylase (EC 2.5.1.3) / Hydroxymethylpyrimidine kinase (EC 2.7.1.49)	56	+	+	+	+	+	+	+	+	+	+	+	+	+	+	+	+	+	+	+	+	+	+	+	+	+	+	+	+	+	+	+	+	+	+	+	+	+	+	+	+	+	+	+	+	+	+	+	+	+	+	+	–	+
1094	644	Aminopeptidase C (EC 3.4.22.40)	56	+	+	+	+	+	+	+	+	+	+	+	+	+	+	+	+	+	+	+	–	+	+	+	+	+	+	+	+	+	+	+	+	+	+	+	+	+	+	+	+	+	+	+	+	+	+	+	+	+	+	+	+	+	+	+	+	+
1095	645	Glucosamine--fructose-6-phosphate aminotransferase [isomerizing] (EC 2.6.1.16)	56	+	+	+	+	+	+	+	+	+	+	+	+	+	+	+	+	+	+	+	–	+	+	+	+	+	+	+	+	+	+	+	+	+	+	+	+	+	+	+	+	+	+	+	+	+	+	+	+	+	+	+	+	+	+	+	+	+
1096	646	TsaD/Kae1/Qri7 protein, required for threonylcarbamoyladenosine t(6)A37 formation in tRNA	56	+	+	+	+	+	+	+	+	+	+	+	+	+	+	+	+	+	+	+	+	+	+	+	+	+	+	+	+	+	+	+	+	+	+	+	+	+	+	+	+	+	+	+	+	+	+	+	+	+	+	+	–	+
1097	648	LSU ribosomal protein L35p	56	+	+	+	+	+	+	+	+	+	+	+	+	+	+	+	+	+	+	+	–	+	+	+	+	+	+	+	+	+	+	+	+	+	+	+	+	+	+	+	+	+	+	+	+	+	+	+	+	+	+	+	+	+	+	+	+	+
1098	650	Hypothetical DUF1027 domain protein	56	+	+	+	+	+	+	+	+	+	+	+	+	–	+	+	+	+	+	+	+	+	+	+	+	+	+	+	+	+	+	+	+	+	+	+	+	+	+	+	+	+	+	+	+	+	+	+	+	+	+	+	+	+	+	+	+	+
1099	651	Late competence protein ComGC, access of DNA to ComEA, FIG007487	56	+	+	+	+	+	+	+	+	+	+	+	+	+	+	+	–	+	+	+	+	+	+	+	+	+	+	+	+	+	+	+	+	+	+	+	+	+	+	+	+	+	+	+	+	+	+	+	+	+	+	+	+	+	+	+	+	+
1100	653	3'-to-5' exoribonuclease RNase R	56	+	+	+	+	+	+	+	+	+	+	+	+	+	+	+	+	+	+	+	+	+	+	+	+	+	+	+	+	+	+	+	+	+	+	+	+	+	+	+	+	+	+	+	+	+	+	+	+	+	+	+	–	+
1101	655	N-acetylmannosamine-6-phosphate 2-epimerase (EC 5.1.3.9)	56	+	+	+	+	+	+	+	+	+	+	+	+	+	+	+	+	+	+	+	–	+	+	+	+	+	+	+	+	+	+	+	+	+	+	+	+	+	+	+	+	+	+	+	+	+	+	+	+	+	+	+	+	+	+	+	+	+
1102	656	Aquaporin (Major Intrinsic Protein Family)	56	+	+	+	+	+	+	+	+	+	+	+	+	+	+	+	+	+	+	+	+	+	+	+	+	+	+	+	+	+	+	+	+	+	+	+	+	+	+	+	+	+	+	+	+	+	+	–	+	+	+	+	+	+	+	+
1103	658	Flavodoxin	56	+	+	+	+	+	+	+	+	+	+	+	+	+	+	+	+	+	+	+	–	+	+	+	+	+	+	+	+	+	+	+	+	+	+	+	+	+	+	+	+	+	+	+	+	+	+	+	+	+	+	+	+	+	+	+	+	+
1104	661	SSU ribosomal protein S16p	56	+	+	+	+	+	+	+	+	+	+	+	+	+	+	+	+	+	+	+	–	+	+	+	+	+	+	+	+	+	+	+	+	+	+	+	+	+	+	+	+	+	+	+	+	+	+	+	+	+	+	+	+	+	+	+	+	+
1105	663	Thioredoxin reductase (EC 1.8.1.9)	56	+	+	+	+	+	+	+	+	+	+	+	+	+	+	+	+	+	+	+	+	+	+	+	+	+	+	+	+	+	+	+	+	+	+	+	+	+	+	+	+	+	+	+	+	+	+	+	+	+	+	+	–	+
1106	664	UDP-N-acetylmuramoylalanyl-D-glutamate--L-lysine ligase (EC 6.3.2.7)	56	+	+	+	+	+	+	+	+	+	+	+	+	+	+	+	+	+	+	+	–	+	+	+	+	+	+	+	+	+	+	+	+	+	+	+	+	+	+	+	+	+	+	+	+	+	+	+	+	+	+	+	+	+	+	+	+	+
1107	670	Transcriptional regulator, TetR family	56	+	+	+	+	+	+	+	+	+	+	+	+	+	+	+	+	+	+	+	+	+	+	+	+	+	+	+	+	+	+	+	+	+	+	+	+	+	+	+	+	+	+	+	+	+	+	+	+	+	+	+	–	+	+	+
1108	673	Cold-shock DEAD-box protein A	56	+	+	+	+	+	+	+	+	+	+	+	+	+	+	+	+	+	+	+	+	+	+	+	+	+	+	+	+	+	+	+	+	+	+	+	+	+	+	+	+	+	+	+	+	+	+	+	+	+	+	+	–	+
1109	676	FIG146085: 3'-to-5' oligoribonuclease A, Bacillus type	56	+	+	+	+	+	+	+	+	+	+	+	+	+	+	+	+	+	+	+	–	+	+	+	+	+	+	+	+	+	+	+	+	+	+	+	+	+	+	+	+	+	+	+	+	+	+	+	+	+	+	+	+	+	+	+	+	+
1110	679	Methionine biosynthesis and transport regulator MtaR, LysR family	56	+	+	+	+	+	+	+	+	+	+	+	+	+	+	+	+	+	+	+	–	+	+	+	+	+	+	+	+	+	+	+	+	+	+	+	+	+	+	+	+	+	+	+	+	+	+	+	+	+	+	+	+	+	+	+	+	+
1111	681	Late competence protein ComEC, DNA transport	56	+	+	+	+	+	+	+	+	+	+	+	+	–	+	+	+	+	+	+	+	+	+	+	+	+	+	+	+	+	+	+	+	+	+	+	+	+	+	+	+	+	+	+	+	+	+	+	+	+	+	+	+	+	+	+	+	+
1112	682	Inactive homolog of metal-dependent proteases, putative molecular chaperone	56	+	+	+	+	+	+	+	+	+	+	+	+	+	+	+	+	+	+	+	+	+	+	+	+	+	+	+	+	+	+	+	+	+	+	+	+	+	+	+	+	+	+	+	+	+	+	+	+	+	+	+	–	+
1113	683	Mn-dependent transcriptional regulator MntR	56	+	+	+	+	+	+	+	+	+	+	+	+	+	+	+	+	+	+	+	–	+	+	+	+	+	+	+	+	+	+	+	+	+	+	+	+	+	+	+	+	+	+	+	+	+	+	+	+	+	+	+	+	+	+	+	+	+
1114	685	tRNA-specific adenosine-34 deaminase (EC 3.5.4.-)	56	+	+	+	+	+	+	+	+	+	+	+	+	+	+	+	+	+	+	+	–	+	+	+	+	+	+	+	+	+	+	+	+	+	+	+	+	+	+	+	+	+	+	+	+	+	+	+	+	+	+	+	+	+	+	+	+	+
1115	688	CTP synthase (EC 6.3.4.2)	56	+	+	+	+	+	+	+	+	+	+	+	+	+	+	+	+	+	+	+	–	+	+	+	+	+	+	+	+	+	+	+	+	+	+	+	+	+	+	+	+	+	+	+	+	+	+	+	+	+	+	+	+	+	+	+	+	+
1116	691	Signal recognition particle receptor protein FtsY (=alpha subunit) (TC 3.A.5.1.1)	56	+	+	+	+	+	+	+	+	+	+	+	+	+	+	+	+	+	+	+	+	+	+	+	+	+	+	+	+	+	+	+	+	+	+	+	+	+	–	+	+	+	+	+	+	+	+	+	+	+	+	+	+	+	+	+	+	+
1117	698	Xaa-Pro dipeptidase (EC 3.4.13.9)	56	+	+	+	+	+	+	+	+	+	+	+	+	+	+	+	+	+	+	+	–	+	+	+	+	+	+	+	+	+	+	+	+	+	+	+	+	+	+	+	+	+	+	+	+	+	+	+	+	+	+	+	+	+	+	+	+	+
1118	705	Phenylalanyl-tRNA synthetase beta chain (EC 6.1.1.20)	56	+	+	+	+	+	+	+	+	+	+	+	+	–	+	+	+	+	+	+	+	+	+	+	+	+	+	+	+	+	+	+	+	+	+	+	+	+	+	+	+	+	+	+	+	+	+	+	+	+	+	+	+	+	+	+	+	+
1119	711	LSU ribosomal protein L4p (L1e)	56	+	+	+	+	+	+	+	+	+	+	+	+	+	+	+	+	+	+	+	–	+	+	+	+	+	+	+	+	+	+	+	+	+	+	+	+	+	+	+	+	+	+	+	+	+	+	+	+	+	+	+	+	+	+	+	+	+
1120	714	Chaperonin (heat shock protein 33)	56	+	+	+	+	+	+	+	+	+	+	+	+	+	+	+	+	+	+	+	+	+	+	+	+	+	+	+	+	+	+	+	+	+	+	+	+	+	+	+	+	+	+	+	+	+	+	+	+	+	+	+	–	+
1121	717	Preprotein translocase subunit SecE (TC 3.A.5.1.1)	56	+	+	+	+	+	+	+	+	+	+	+	+	+	+	+	+	+	+	+	–	+	+	+	+	+	+	+	+	+	+	+	+	+	+	+	+	+	+	+	+	+	+	+	+	+	+	+	+	+	+	+	+	+	+	+	+	+
1122	718	IMP cyclohydrolase (EC 3.5.4.10) / Phosphoribosylaminoimidazolecarboxamide formyltransferase (EC 2.1.2.3)	56	+	+	+	+	+	+	+	+	+	+	+	+	+	+	+	+	+	+	+	+	+	+	+	+	+	+	+	+	+	+	+	+	+	+	+	+	+	+	+	+	+	+	+	+	+	+	+	+	+	+	+	–	+
1123	720	RecD-like DNA helicase YrrC	56	+	+	+	+	+	+	+	+	+	+	+	+	+	+	+	+	+	+	+	+	+	+	+	+	+	+	+	+	+	+	+	+	+	+	+	+	+	+	+	+	+	+	+	+	+	+	+	+	+	+	+	–	+
1124	723	tRNA dihydrouridine synthase B (EC 1.-.-.-)	56	+	+	+	+	+	+	+	+	+	+	+	+	+	+	+	+	+	+	+	+	+	+	+	+	+	+	+	+	+	+	+	+	+	+	+	+	+	+	+	+	+	+	+	+	+	+	+	+	+	+	+	–	+
1125	726	Threonine synthase (EC 4.2.3.1)	56	+	+	+	+	+	+	+	+	+	+	+	+	+	+	+	+	+	+	+	+	+	+	+	+	+	+	+	+	+	+	+	+	+	+	+	+	+	+	+	+	+	+	+	+	+	+	+	+	+	+	+	–	+
1126	727	Cell division protein FtsW	56	+	+	+	+	+	+	+	+	+	+	+	+	+	+	+	+	+	+	+	+	+	+	+	+	+	+	+	+	+	+	+	+	+	+	+	+	+	+	+	+	+	+	+	+	+	+	+	+	+	+	+	–	+
1127	733	Lipoate-protein ligase A	56	+	+	+	+	+	+	+	+	+	+	+	+	+	+	+	+	+	+	+	–	+	+	+	+	+	+	+	+	+	+	+	+	+	+	+	+	+	+	+	+	+	+	+	+	+	+	+	+	+	+	+	+	+	+	+	+	+
1128	739	ABC-type polar amino acid transport system, ATPase component	56	+	+	+	+	+	+	+	+	+	+	+	+	+	+	+	+	+	+	+	–	+	+	+	+	+	+	+	+	+	+	+	+	+	+	+	+	+	+	+	+	+	+	+	+	+	+	+	+	+	+	+	+	+	+	+	+	+
1129	741	Phosphoribosylaminoimidazole carboxylase catalytic subunit (EC 4.1.1.21)	56	+	+	+	+	+	+	+	+	+	+	+	+	+	+	+	+	+	+	+	+	+	+	+	+	+	+	+	+	+	+	+	+	+	+	+	+	+	+	+	+	+	+	+	+	+	+	+	+	+	+	+	–	+
1130	745	Amino acid ABC transporter, amino acid-binding protein	56	+	+	+	+	+	+	+	+	+	+	+	+	+	+	+	+	+	+	+	–	+	+	+	+	+	+	+	+	+	+	+	+	+	+	+	+	+	+	+	+	+	+	+	+	+	+	+	+	+	+	+	+	+	+	+	+	+
1131	748	Hypothetical similar to thiamin biosynthesis lipoprotein ApbE	56	+	+	+	+	+	+	+	+	+	+	+	+	+	+	+	+	+	+	+	–	+	+	+	+	+	+	+	+	+	+	+	+	+	+	+	+	+	+	+	+	+	+	+	+	+	+	+	+	+	+	+	+	+	+	+	+	+
1132	753	LacX protein, plasmid	56	+	+	+	+	+	+	+	+	+	+	+	+	+	+	+	+	+	+	+	+	+	+	+	+	+	+	+	+	+	+	+	+	+	+	+	+	+	+	+	+	+	+	+	+	–	+	+	+	+	+	+	+	+
1133	757	L-serine dehydratase, alpha subunit (EC 4.3.1.17)	56	+	+	+	+	+	+	+	+	+	+	+	+	+	+	+	+	+	+	+	+	+	+	+	+	+	+	+	+	+	+	+	+	+	+	+	+	+	+	+	+	+	+	+	+	+	+	+	+	+	+	+	–	+
1134	759	Transcriptional regulator, MarR family	56	+	+	+	+	+	+	+	+	+	+	+	+	+	+	+	+	+	+	+	+	+	+	+	+	+	+	+	+	+	+	+	+	+	+	+	+	+	+	+	+	+	+	+	+	+	–	+	+	+	+	+	+	+	+	+
1135	761	Carbamoyl-phosphate synthase small chain (EC 6.3.5.5)	56	+	+	+	+	+	+	+	+	+	+	+	+	–	+	+	+	+	+	+	+	+	+	+	+	+	+	+	+	+	+	+	+	+	+	+	+	+	+	+	+	+	+	+	+	+	+	+	+	+	+	+	+	+	+	+	+	+
1136	764	Carbonic anhydrase (EC 4.2.1.1)	56	+	+	+	+	+	+	+	+	+	+	+	+	+	+	+	+	+	+	+	–	+	+	+	+	+	+	+	+	+	+	+	+	+	+	+	+	+	+	+	+	+	+	+	+	+	+	+	+	+	+	+	+	+	+	+	+	+
1137	769	Phosphoribosylamine--glycine ligase (EC 6.3.4.13)	56	+	+	+	+	+	+	+	+	+	+	+	+	+	+	+	+	+	+	+	+	+	+	+	+	+	+	+	+	+	+	+	+	+	+	+	+	+	+	+	+	+	+	+	+	+	+	+	+	+	+	+	–	+
1138	773	Chloride channel protein	56	+	+	+	+	+	+	+	+	+	+	+	+	+	+	+	+	+	+	+	–	+	+	+	+	+	+	+	+	+	+	+	+	+	+	+	+	+	+	+	+	+	+	+	+	+	+	+	+	+	+	+	+	+	+	+	+	+
1139	780	Chorismate mutase I (EC 5.4.99.5)	56	+	+	+	+	+	+	+	+	+	+	+	+	+	+	+	+	+	+	+	–	+	+	+	+	+	+	+	+	+	+	+	+	+	+	+	+	+	+	+	+	+	+	+	+	+	+	+	+	+	+	+	+	+	+	+	+	+
1140	782	Preprotein translocase subunit YajC (TC 3.A.5.1.1)	56	+	+	+	+	+	+	+	+	+	+	+	+	+	+	+	+	+	+	+	–	+	+	+	+	+	+	+	+	+	+	+	+	+	+	+	+	+	+	+	+	+	+	+	+	+	+	+	+	+	+	+	+	+	+	+	+	+
1141	783	Transcriptional regulator, HxlR family	56	+	+	+	+	+	+	+	+	+	+	+	+	+	+	+	+	+	+	–	+	+	+	+	+	+	+	+	+	+	+	+	+	+	+	+	+	+	+	+	+	+	+	+	+	+	+	+	+	+	+	+	+	+	+	+	+	+
1142	784	LSU ribosomal protein L13p (L13Ae)	56	+	+	+	+	+	+	+	+	+	+	+	+	+	+	+	+	+	+	+	–	+	+	+	+	+	+	+	+	+	+	+	+	+	+	+	+	+	+	+	+	+	+	+	+	+	+	+	+	+	+	+	+	+	+	+	+	+
1143	788	N-acyl-L-amino acid amidohydrolase (EC 3.5.1.14)	56	+	+	+	+	+	+	+	+	+	–	+	+	+	+	+	+	+	+	+	+	+	+	+	+	+	+	+	+	+	+	+	+	+	+	+	+	+	+	+	+	+	+	+	+	+	+	+	+	+	+	+	+	+	+	+	+	+
1144	798	Gamma-glutamyl phosphate reductase (EC 1.2.1.41)	56	+	+	+	+	+	+	+	+	+	+	+	+	+	+	+	+	+	+	+	+	+	+	+	+	+	+	+	+	+	+	+	+	+	+	+	+	+	+	+	+	+	+	+	+	+	+	+	+	+	+	+	–	+
1145	799	hypothetical protein	56	+	+	+	+	+	+	+	+	+	+	+	+	+	+	+	+	+	+	+	+	+	+	+	+	+	+	+	+	+	+	+	+	+	+	+	+	+	+	+	+	+	+	+	+	+	+	+	+	+	+	+	–	+
1146	803	Pyrimidine-nucleoside phosphorylase (EC 2.4.2.2)	56	+	+	+	+	+	+	+	+	+	+	+	+	+	+	+	+	+	+	+	+	+	+	+	+	+	+	+	+	+	+	+	+	+	+	+	+	+	–	+	+	+	+	+	+	+	+	+	+	+	+	+	+	+	+	+	+	+
1147	811	rhamnose-containing polysacharide translocation permease	56	+	+	+	+	+	+	+	+	+	+	+	+	+	+	+	+	+	+	+	+	+	+	+	+	+	+	+	+	+	+	+	+	+	+	+	+	–	+	+	+	+	+	+	+	+	+	+	+	+	+	+	+	+	+	+	+	+

Table S5. Precence/absence of each HG among 57 *S. suis* strains

				1																13			25					28						104		94			76			53/54		Divergent *S. suis*																
Index[a]	ClusterID	Gene products	Members	98HAH33	05ZYH33	SC84	A7	SS12	P1/7	DAT273	NIAH11434	JS14	GZ1	BM407	S735	13730	MNCM07	NCTC 10446	14636	NCTC 10237	ST1	2651	89-1591	MNCM04	MNCM25	8074	D9	4961	ST3	MNCM43	DAT292	DAT260	NIAH11435	MNCM21	MNCM50	89-2479	DAT234	DAT290	93A	42A	NT77	6407	11538	8830	12814	92-1400	89-590	2726	2524	89-3576-3	4417	D12	10581	89-5259	88-5299A	R61	92-1191	14A	92-4172	22083
1148	815	3-oxoacyl-[acyl-carrier protein] reductase (EC 1.1.1.100)	56	+	+	+	+	+	+	+	+	+	+	+	+	+	+	+	+	+	+	+	+	+	+	+	+	+	+	+	+	+	+	+	+	+	+	+	+	+	+	+	+	+	+	+	+	+	+	+	+	+	+	+	–	+
1149	816	ABC-transporter (ATP-binding protein) -possibly involved in cell wall localization and side chain formation of rhamnose-glucose polysaccharide	56	+	+	+	+	+	+	+	+	+	+	+	+	+	+	+	+	+	+	+	+	+	+	+	+	+	+	+	+	+	+	+	+	–	+	+	+	+	+	+	+	+	+	+	+	+	+	+	+	+	+	+	+	+
1150	822	GMP reductase (EC 1.7.1.7)	56	+	+	+	+	+	+	+	+	+	+	+	+	+	+	+	+	+	+	+	–	+	+	+	+	+	+	+	+	+	+	+	+	+	+	+	+	+	+	+	+	+	+	+	+	+	+	+	+	+	+	+	+	+
1151	825	ABC transporter, ATP-binding/permease protein	56	+	+	+	+	+	+	+	+	+	+	+	+	+	+	+	+	+	+	+	–	+	+	+	+	+	+	+	+	+	+	+	+	+	+	+	+	+	+	+	+	+	+	+	+	+	+	+	+	+	+	+	+	+
1152	827	ACT domain containing transcriptional regulators, related to gcvR of E.coli	56	+	+	+	+	+	+	+	+	+	+	+	+	+	+	+	+	+	+	+	–	+	+	+	+	+	+	+	+	+	+	+	+	+	+	+	+	+	+	+	+	+	+	+	+	+	+	+	+	+	+	+	+	+
1153	835	Putative membrane protein YeiH	56	+	+	+	+	+	+	+	+	+	+	+	+	+	+	+	+	+	+	+	+	+	+	+	+	+	+	+	+	+	+	+	+	+	+	+	+	+	+	+	+	+	+	+	+	+	+	+	+	+	+	+	–	+
1154	865	Hypothetical protein DUF901, similar to C-terminal domain of ribosome protection-type Tc-resistance proteins	56	+	+	+	+	+	+	+	+	+	+	+	+	+	+	+	+	+	+	+	+	+	+	+	+	+	+	+	+	+	+	+	+	+	+	+	+	+	+	+	+	+	+	+	+	+	+	–	+	+	+	+	+	+
1155	866	Diaminopimelate decarboxylase (EC 4.1.1.20)	56	+	+	+	+	+	+	+	+	+	+	+	+	+	+	+	+	+	+	+	–	+	+	+	+	+	+	+	+	+	+	+	+	+	+	+	+	+	+	+	+	+	+	+	+	+	+	+	+	+	+	+	+	+
1156	869	Tyrosine-protein kinase transmembrane modulator EpsC	56	+	+	+	+	+	+	+	+	+	+	+	+	–	+	+	+	+	+	+	+	+	+	+	+	+	+	+	+	+	+	+	+	+	+	+	+	+	+	+	+	+	+	+	+	+	+	+	+	+	+	+	+	+	+	+
1157	879	Transcriptional regulator, Cro/CI family	56	+	+	+	+	+	+	+	+	+	+	+	+	+	+	+	+	+	+	+	–	+	+	+	+	+	+	+	+	+	+	+	+	+	+	+	+	+	+	+	+	+	+	+	+	+	+	+	+	+	+	+	+	+
1158	881	Branched-chain amino acid transport protein azlC	56	+	+	+	+	+	+	+	+	+	+	+	+	+	+	+	+	+	+	+	–	+	+	+	+	+	+	+	+	+	+	+	+	+	+	+	+	+	+	+	+	+	+	+	+	+	+	+	+	+	+	+	+	+
1159	882	Predicted membrane protein	56	+	+	+	+	+	+	+	+	+	+	+	+	+	+	+	+	+	+	+	+	+	+	+	+	+	+	+	+	+	+	+	+	–	+	+	+	+	+	+	+	+	+	+	+	+	+	+	+	+	+	+	+	+
1160	885	Dihydroorotate dehydrogenase, catalytic subunit (EC 1.3.3.1)	56	+	+	+	+	+	+	+	+	+	+	+	+	+	+	+	+	+	+	+	–	+	+	+	+	+	+	+	+	+	+	+	+	+	+	+	+	+	+	+	+	+	+	+	+	+	+	+	+	+	+	+	+	+
1161	899	Beta-glucoside bgl operon antiterminator, BglG family	56	+	+	+	+	+	+	+	+	+	+	+	+	+	+	+	+	+	+	+	+	+	+	+	+	+	+	+	+	+	+	+	+	+	+	+	+	+	+	+	+	+	+	+	+	–	+	+	+	+	+	+	+	+
1162	915	Cystathionine gamma-synthase (EC 2.5.1.48)	56	+	+	+	+	+	+	+	+	+	+	+	+	+	+	+	+	+	+	–	+	+	+	+	+	+	+	+	+	+	+	+	+	+	+	+	+	+	+	+	+	+	+	+	+	+	+	+	+	+	+	+	+	+	+	+
1163	945	isoleucyl-tRNA synthetase	56	+	+	+	+	+	+	+	+	+	+	+	+	+	+	+	+	+	+	+	+	+	+	+	+	+	+	+	+	+	+	+	+	+	+	+	+	+	+	+	+	+	+	+	+	+	+	+	+	+	+	–	+	+
1164	951	hypothetical protein	56	+	+	+	+	+	+	+	+	+	+	+	+	+	+	+	+	+	+	+	+	+	+	+	+	+	+	+	+	+	+	+	+	–	+	+	+	+	+	+	+	+	+	+	+	+	+	+	+	+	+	+	+	+
1165	957	Methionine ABC transporter ATP-binding protein	56	+	+	+	+	+	+	+	+	+	+	+	+	+	+	+	+	+	+	+	+	+	+	+	+	+	+	+	+	+	+	+	+	+	+	+	+	+	+	+	+	+	+	+	+	+	+	+	+	+	+	+	–	+
1166	966	DUF124 domain-containing protein	56	+	+	+	+	+	+	+	+	+	+	+	+	+	+	+	+	+	+	+	–	+	+	+	+	+	+	+	+	+	+	+	+	+	+	+	+	+	+	+	+	+	+	+	+	+	+	+	+	+	+	+	+	+
1167	969	Response regulator of the LytR/AlgR family	56	+	+	+	+	+	+	+	+	+	+	+	+	+	+	+	+	+	+	+	–	+	+	+	+	+	+	+	+	+	+	+	+	+	+	+	+	+	+	+	+	+	+	+	+	+	+	+	+	+	+	+	+	+
1168	973	hypothetical protein	56	+	+	+	+	+	+	+	+	+	+	+	+	+	+	+	+	+	+	+	–	+	+	+	+	+	+	+	+	+	+	+	+	+	+	+	+	+	+	+	+	+	+	+	+	+	+	+	+	+	+	+	+	+
1169	985	ABC transporter permease protein	56	+	+	+	+	+	+	+	+	+	+	+	+	–	+	+	+	+	+	+	+	+	+	+	+	+	+	+	+	+	+	+	+	+	+	+	+	+	+	+	+	+	+	+	+	+	+	+	+	+	+	+	+	+	+	+
1170	987	ABC transporter permease protein	56	+	+	+	+	+	+	+	+	+	+	+	+	+	+	+	+	+	+	+	+	+	+	+	+	+	+	+	+	+	+	+	+	+	+	+	–	+	+	+	+	+	+	+	+	+	+	+	+	+	+	+	+	+
1171	988	SSU ribosomal protein S14p (S29e)	56	+	+	+	+	+	+	+	+	+	+	+	+	+	+	+	+	+	+	+	–	+	+	+	+	+	+	+	+	+	+	+	+	+	+	+	+	+	+	+	+	+	+	+	+	+	+	+	+	+	+	+	+	+
1172	1000	Cyanate permease	56	+	+	+	+	+	+	+	+	+	+	+	+	+	+	+	+	+	+	+	–	+	+	+	+	+	+	+	+	+	+	+	+	+	+	+	+	+	+	+	+	+	+	+	+	+	+	+	+	+	+	+	+	+
1173	1004	UDP-galactopyranose mutase (EC 5.4.99.9)	56	+	+	+	+	+	+	+	+	+	+	+	+	+	+	+	+	+	+	+	+	+	+	+	+	+	+	+	+	+	+	+	+	+	+	+	+	+	+	+	+	+	+	+	–	+	+	+	+	+	+	+	+	+
1174	1033	response regulator (homolog to RR01Spn & Sth)	56	+	+	+	+	+	+	+	+	+	+	+	+	+	+	+	+	+	+	+	+	+	+	+	+	+	+	+	+	+	+	+	+	+	+	+	+	+	+	+	+	+	–	+	+	+	+	+	+	+	+	+	+	+
1175	1034	Two-component system histidine kinase	56	+	+	+	+	+	+	+	+	+	+	+	+	+	+	+	+	+	+	+	+	+	+	+	+	+	+	+	+	+	+	+	+	+	+	+	+	+	+	+	+	+	–	+	+	+	+	+	+	+	+	+	+	+
1176	1044	Predicted hydrolase of the alpha/beta superfamily	56	+	+	+	+	+	+	+	+	+	+	+	+	+	+	+	+	+	+	+	+	+	+	+	+	+	+	+	+	+	+	+	+	–	+	+	+	+	+	+	+	+	+	+	+	+	+	+	+	+	+	+	+	+
1177	1046	Carbamoylphosphate synthase large subunit (split gene in MJ)	56	+	+	+	+	+	+	+	+	+	+	+	+	+	+	+	+	+	+	+	+	+	+	+	+	+	+	+	+	+	+	+	+	–	+	+	+	+	+	+	+	+	+	+	+	+	+	+	+	+	+	+	+	+
1178	1052	5-methyltetrahydropteroyltriglutamate--homocysteine methyltransferase (EC 2.1.1.14)	56	+	+	+	+	+	+	+	+	+	+	+	+	+	+	+	+	+	+	+	–	+	+	+	+	+	+	+	+	+	+	+	+	+	+	+	+	+	+	+	+	+	+	+	+	+	+	+	+	+	+	+	+	+
1179	1058	ABC transporter ATP-binding protein	56	+	+	+	+	+	+	+	+	+	+	+	+	+	+	–	+	+	+	+	+	+	+	+	+	+	+	+	+	+	+	+	+	+	+	+	+	+	+	+	+	+	+	+	+	+	+	+	+	+	+	+	+	+	+	+
1180	1076	Glycosyl transferase, family 2	56	+	+	+	+	+	+	+	+	+	+	+	+	+	+	+	+	+	+	+	+	+	+	+	+	+	+	+	+	+	+	+	+	+	+	+	+	+	+	+	+	+	+	+	+	+	+	+	–	+	+	+	+	+
1181	1085	6-phosphogluconate dehydrogenase, decarboxylating (EC 1.1.1.44)	56	+	+	+	+	+	+	+	+	+	+	+	+	+	+	+	+	+	+	+	–	+	+	+	+	+	+	+	+	+	+	+	+	+	+	+	+	+	+	+	+	+	+	+	+	+	+	+	+	+	+	+	+	+
1182	1088	hypothetical protein	56	+	+	+	+	+	+	+	+	+	+	+	+	+	+	+	+	+	+	+	–	+	+	+	+	+	+	+	+	+	+	+	+	+	+	+	+	+	+	+	+	+	+	+	+	+	+	+	+	+	+	+	+	+
1183	1089	Cation-transporting ATPase, E1-E2 family	56	+	+	+	+	+	+	+	+	+	+	+	+	+	+	+	+	+	+	+	+	+	+	+	+	+	+	+	+	+	+	+	+	+	+	+	+	+	+	+	+	+	+	+	+	+	+	+	+	+	+	+	–	+
1184	1094	Response regulator CsrR	56	+	+	+	+	+	+	+	+	+	+	+	+	+	+	+	+	+	+	+	–	+	+	+	+	+	+	+	+	+	+	+	+	+	+	+	+	+	+	+	+	+	+	+	+	+	+	+	+	+	+	+	+	+
1185	1097	hypothetical protein	56	+	+	+	+	+	+	+	+	+	+	+	+	+	+	+	+	+	+	+	+	+	+	+	+	+	–	+	+	+	+	+	+	+	+	+	+	+	+	+	+	+	+	+	+	+	+	+	+	+	+	+	+	+
1186	1110	Serine/threonine protein phosphatase (EC 3.1.3.16)	56	+	+	+	+	+	+	+	+	+	+	+	+	+	+	+	+	+	+	+	–	+	+	+	+	+	+	+	+	+	+	+	+	+	+	+	+	+	+	+	+	+	+	+	+	+	+	+	+	+	+	+	+	+
1187	1119	Phosphinothricin N-acetyltransferase	56	+	+	+	+	+	+	+	+	+	+	+	+	+	+	+	+	+	+	+	+	+	+	+	+	+	+	+	+	+	+	+	+	+	+	+	+	+	+	+	+	+	+	+	+	+	+	+	+	+	+	+	–	+
1188	1122	COG1399 protein in cluster with ribosomal protein L32p, Firmicutes subfamily	56	+	+	+	+	+	+	+	+	+	+	+	+	+	+	+	+	+	+	+	–	+	+	+	+	+	+	+	+	+	+	+	+	+	+	+	+	+	+	+	+	+	+	+	+	+	+	+	+	+	+	+	+	+
1189	1129	Glutamine amidotransferase, class I	56	+	+	+	+	+	+	+	+	+	–	+	+	+	+	+	+	+	+	+	+	+	+	+	+	+	+	+	+	+	+	+	+	+	+	+	+	+	+	+	+	+	+	+	+	+	+	+	+	+	+	+	+	+	+	+
1190	1131	Putative ABC transporter (ATP-binding protein), spy1791 homolog	56	+	+	+	+	+	+	+	+	+	+	+	+	+	+	+	+	+	+	+	+	+	+	+	+	+	+	+	+	+	+	+	+	+	+	+	+	+	+	+	+	+	+	+	+	+	+	+	+	+	–	+	+	+
1191	1142	Protein-L-isoD(D-D) O-methyltransferase	56	+	+	+	+	+	+	+	+	+	+	+	+	+	+	+	+	+	+	+	–	+	+	+	+	+	+	+	+	+	+	+	+	+	+	+	+	+	+	+	+	+	+	+	+	+	+	+	+	+	+	+	+	+
1192	1146	MutT/nudix family protein	56	+	+	+	+	+	+	+	+	+	+	+	+	+	+	+	+	+	+	+	+	+	+	+	+	+	+	+	+	+	+	+	+	+	+	+	+	+	+	+	+	+	+	+	+	–	+	+	+	+	+	+	+	+
1193	1148	Putative ABC transporter ATP-binding protein, spy1790 homolog	56	+	+	+	+	+	+	+	+	+	+	+	+	+	+	+	+	+	+	+	+	+	+	+	+	+	+	+	+	+	+	+	+	+	+	+	+	+	+	+	+	+	+	+	+	+	+	+	+	+	–	+	+	+
1194	1158	Pyrrolidone-carboxylate peptidase (EC 3.4.19.3)	56	+	+	+	+	+	+	+	+	+	+	+	+	+	+	+	+	+	+	+	–	+	+	+	+	+	+	+	+	+	+	+	+	+	+	+	+	+	+	+	+	+	+	+	+	+	+	+	+	+	+	+	+	+
1195	1163	Oligopeptide ABC transporter, periplasmic oligopeptide-binding protein OppA (TC 3.A.1.5.1)	56	+	+	+	+	+	+	+	+	+	–	+	+	+	+	+	+	+	+	+	+	+	+	+	+	+	+	+	+	+	+	+	+	+	+	+	+	+	+	+	+	+	+	+	+	+	+	+	+	+	+	+	+	+	+	+
1196	1172	Lactose phosphotransferase system repressor	56	+	+	+	+	+	+	+	+	+	+	+	+	+	+	+	+	+	+	+	+	+	+	+	+	+	+	+	+	+	+	+	+	+	+	+	+	+	+	+	+	+	+	+	+	–	+	+	+	+	+	+	+	+
1197	1180	Membrane protein, putative	56	+	+	+	+	+	+	+	+	+	+	+	+	+	+	+	+	+	+	+	–	+	+	+	+	+	+	+	+	+	+	+	+	+	+	+	+	+	+	+	+	+	+	+	+	+	+	+	+	+	+	+	+	+
1198	1182	YefM protein (antitoxin to YoeB)	56	+	+	+	+	+	+	+	+	+	+	+	+	+	+	+	+	+	+	+	+	+	+	+	+	+	+	+	+	+	+	+	+	+	+	+	+	+	+	+	+	+	+	+	+	–	+	+	+	+	+	+	+	+
1199	1183	Glutamate 5-kinase (EC 2.7.2.11) / RNA-binding C-terminal domain PUA	56	+	+	+	+	+	+	+	+	+	+	+	+	+	+	+	+	+	+	+	+	+	+	+	+	+	+	+	+	+	+	+	+	+	+	+	+	+	+	+	+	+	+	+	+	+	+	+	+	+	+	+	–	+
1200	1185	transcriptional regulator	56	+	+	+	+	+	+	+	+	+	+	+	+	+	+	+	+	+	+	+	–	+	+	+	+	+	+	+	+	+	+	+	+	+	+	+	+	+	+	+	+	+	+	+	+	+	+	+	+	+	+	+	+	+
1201	1186	probable enzyme with TIM-barrel fold	56	+	+	+	+	+	+	+	+	+	+	+	+	+	+	+	+	+	+	+	–	+	+	+	+	+	+	+	+	+	+	+	+	+	+	+	+	+	+	+	+	+	+	+	+	+	+	+	+	+	+	+	+	+
1202	1189	hypothetical protein	56	+	+	+	+	+	+	+	+	+	+	+	+	+	+	+	+	+	+	+	+	+	+	+	+	+	+	+	+	+	+	+	+	+	+	+	+	+	+	+	+	+	+	+	+	+	+	+	–	+	+	+	+	+
1203	1197	Multiple sugar ABC transporter, membrane-spanning permease protein MsmF	56	+	+	+	+	+	+	+	+	+	+	+	+	+	+	+	+	+	+	+	+	+	+	+	+	+	+	+	+	+	+	+	+	+	+	+	+	+	+	+	+	+	+	+	+	+	+	+	+	+	+	+	+	–
1204	1200	Ribosomal-protein-S18p-alanine acetyltransferase (EC 2.3.1.-)	56	+	+	+	+	+	+	+	+	+	+	+	+	+	+	+	+	+	+	+	+	+	+	+	+	+	+	+	+	+	+	+	+	+	+	+	+	+	+	+	+	+	+	+	+	+	+	+	+	+	+	+	–	+
1205	1203	Inorganic pyrophosphatase	56	+	+	+	+	+	+	+	+	+	+	+	+	+	+	+	+	+	+	+	–	+	+	+	+	+	+	+	+	+	+	+	+	+	+	+	+	+	+	+	+	+	+	+	+	+	+	+	+	+	+	+	+	+
1206	1206	Uracil permease	56	+	+	+	+	+	+	+	+	+	+	+	+	+	+	+	+	+	+	+	+	+	+	+	+	+	+	+	+	+	+	+	+	+	+	+	+	+	+	+	+	+	+	+	+	+	+	+	+	+	+	+	–	+
1207	1207	YoeB toxin protein	56	+	+	+	+	+	+	+	+	+	+	+	+	+	+	+	+	+	+	+	+	+	+	+	+	+	+	+	+	+	+	+	+	+	+	+	+	+	+	+	+	+	+	+	+	–	+	+	+	+	+	+	+	+
1208	1220	Sucrose operon repressor ScrR, LacI family	56	+	+	+	+	+	+	+	+	+	+	+	+	+	+	+	+	+	+	+	+	+	+	+	+	+	+	+	+	+	+	+	+	+	+	+	+	+	+	+	+	+	+	+	+	+	+	+	+	+	+	+	+	–
1209	1223	Sucrose-6-phosphate hydrolase (EC 3.2.1.26)	56	+	+	+	+	+	+	+	+	+	+	+	+	+	+	+	+	+	+	+	+	+	+	+	+	+	+	+	+	+	+	+	+	+	+	+	+	+	+	+	+	+	+	+	+	+	+	+	+	+	+	+	+	–
1210	1227	Endonuclease/Exonuclease/phosphatase family protein	56	+	+	+	+	+	+	+	+	+	+	+	+	+	+	+	+	+	+	+	+	+	+	+	+	+	+	+	+	+	+	+	+	+	–	+	+	+	+	+	+	+	+	+	+	+	+	+	+	+	+	+	+	+
1211	1229	UPF0246 protein YaaA	56	+	+	+	+	+	+	+	+	+	+	+	+	+	+	+	+	+	+	+	–	+	+	+	+	+	+	+	+	+	+	+	+	+	+	+	+	+	+	+	+	+	+	+	+	+	+	+	+	+	+	+	+	+
1212	1241	Phage infection protein	56	+	+	+	+	+	+	+	+	+	+	+	+	+	+	+	+	+	+	+	+	+	+	+	+	+	+	+	+	+	+	+	+	+	+	+	+	+	+	+	+	+	+	+	+	+	+	+	+	+	–	+	+	+
1213	1243	late competence protein ComGE, FIG075573	56	+	+	+	+	+	+	+	+	+	+	+	+	+	+	+	–	+	+	+	+	+	+	+	+	+	+	+	+	+	+	+	+	+	+	+	+	+	+	+	+	+	+	+	+	+	+	+	+	+	+	+	+	+	+	+
1214	1247	ABC transporter, substrate-binding protein	56	+	+	+	+	+	+	+	+	+	+	+	+	+	+	+	+	+	+	+	+	+	+	+	+	+	+	+	+	+	+	+	+	+	+	+	+	+	+	+	+	+	+	+	+	+	+	+	+	+	+	+	+	–
1215	1248	ABC-type sugar transport system, permease component	56	+	+	+	+	+	+	+	+	+	+	+	+	+	+	+	+	+	+	+	+	+	+	+	+	+	+	+	+	+	+	+	+	+	+	+	+	+	+	+	+	+	+	+	+	+	+	+	+	+	+	+	+	–
1216	1252	Choline binding protein D	56	+	+	+	+	+	+	+	+	+	+	+	+	+	+	+	+	+	+	+	+	+	+	+	+	+	+	+	+	+	+	+	+	+	+	+	+	+	+	+	+	+	+	+	+	+	+	+	–	+	+	+	+	+
1217	1255	Daunorubicin resistance transmembrane protein	56	+	+	+	+	+	+	+	+	+	+	+	+	+	+	+	+	+	+	+	+	+	+	+	+	+	+	+	+	+	+	+	+	+	+	+	+	+	+	+	+	+	+	+	+	+	+	+	+	+	–	+	+	+
1218	1258	hypothetical protein	56	+	+	+	+	+	+	+	+	+	+	+	+	+	+	+	+	+	+	–	+	+	+	+	+	+	+	+	+	+	+	+	+	+	+	+	+	+	+	+	+	+	+	+	+	+	+	+	+	+	+	+	+	+	+	+
1219	1260	Serine endopeptidase ScpC (EC 3.4.21.-)	56	+	+	+	+	+	+	+	+	+	+	+	+	+	+	+	+	+	+	+	+	+	+	+	+	+	+	+	+	+	+	+	+	–	+	+	+	+	+	+	+	+	+	+	+	+	+	+	+	+	+	+	+	+	+	+
1220	1263	Daunorubicin resistance ATP-binding protein drrA	56	+	+	+	+	+	+	+	+	+	+	+	+	+	+	+	+	+	+	+	+	+	+	+	+	+	+	+	+	+	+	+	+	+	+	+	+	+	+	+	+	+	+	+	+	+	+	+	+	+	–	+	+	+
1221	1264	Probable dipeptidase B (EC 3.4.-.-)	56	+	+	+	+	+	+	+	+	+	+	+	+	+	+	+	+	+	+	+	+	+	+	+	+	+	+	+	+	+	+	+	+	+	+	+	+	+	+	+	+	+	+	+	+	+	+	–	+	+	+	+	+	+
1222	1265	ABC transporter, permease protein EscB	56	+	+	+	+	+	+	+	+	+	+	+	+	+	+	+	+	+	+	+	–	+	+	+	+	+	+	+	+	+	+	+	+	+	+	+	+	+	+	+	+	+	+	+	+	+	+	+	+	+	+	+	+	+
1223	1276	ABC-type uncharacterized transport system, permease component	56	+	+	+	+	+	+	+	+	+	+	+	+	+	+	+	+	+	+	+	+	+	+	+	+	+	+	+	+	+	+	+	+	+	+	+	+	+	+	+	+	+	+	+	+	+	+	+	+	+	–	+	+	+
1224	1292	NTP pyrophosphohydrolase including oxidative damage repair enzymes	56	+	+	+	+	+	+	+	+	+	+	+	+	+	+	+	+	+	+	+	+	+	+	+	+	+	+	+	+	+	+	+	+	+	+	+	+	+	+	+	+	+	+	+	+	+	+	+	+	+	+	+	–	+
1225	1294	putative low temperature requirement A protein	56	+	+	+	+	+	+	+	+	+	+	+	+	+	+	+	+	+	+	+	+	+	+	+	+	+	+	+	+	+	+	+	+	+	+	+	+	+	+	+	–	+	+	+	+	+	+	+	+	+	+	+	+	+
1226	1295	Multi antimicrobial extrusion protein MatE	56	+	+	+	+	+	+	+	+	+	+	+	+	+	+	+	+	+	+	+	+	+	+	+	+	+	+	+	+	+	+	+	+	+	+	+	+	+	+	+	+	+	+	+	+	+	+	+	+	+	+	+	–	+
1227	1296	hypothetical protein	56	+	+	+	+	+	+	+	+	+	+	+	+	+	+	+	+	+	+	+	–	+	+	+	+	+	+	+	+	+	+	+	+	+	+	+	+	+	+	+	+	+	+	+	+	+	+	+	+	+	+	+	+	+
1228	1300	probable permease	56	+	+	+	+	+	+	+	+	+	+	+	+	+	+	+	+	+	+	+	–	+	+	+	+	+	+	+	+	+	+	+	+	+	+	+	+	+	+	+	+	+	+	+	+	+	+	+	+	+	+	+	+	+
1229	1304	internalin, putative	56	+	+	+	+	+	+	+	+	+	+	+	+	+	+	+	+	+	+	+	+	+	+	+	+	+	+	+	+	+	+	+	+	+	+	+	+	+	+	+	+	+	+	+	+	+	+	–	+	+	+	+	+	+
1230	1312	hypothetical protein	56	+	+	+	+	+	+	+	+	+	+	+	+	+	+	+	+	+	+	+	–	+	+	+	+	+	+	+	+	+	+	+	+	+	+	+	+	+	+	+	+	+	+	+	+	+	+	+	+	+	+	+	+	+
1231	1344	hypothetical protein	56	+	+	+	+	+	+	+	+	+	+	+	+	+	+	+	+	+	+	+	–	+	+	+	+	+	+	+	+	+	+	+	+	+	+	+	+	+	+	+	+	+	+	+	+	+	+	+	+	+	+	+	+	+
1232	1373	Transporter	56	+	+	+	+	+	+	+	+	+	+	+	+	+	+	+	+	+	+	+	+	+	+	+	+	+	+	+	+	+	+	+	+	+	+	+	+	+	+	+	+	+	+	+	+	+	+	–	+	+	+	+	+	+
1233	1376	hypothetical protein	56	+	+	+	+	+	+	+	+	+	+	+	+	+	+	+	+	+	+	+	+	+	+	+	+	+	+	+	+	+	+	+	+	+	+	+	+	+	+	+	+	+	+	+	+	+	+	+	+	+	–	+	+	+
1234	1379	Fumarate reductase flavoprotein subunit (EC 1.3.99.1)	56	+	+	+	+	+	+	+	+	+	–	+	+	+	+	+	+	+	+	+	+	+	+	+	+	+	+	+	+	+	+	+	+	+	+	+	+	+	+	+	+	+	+	+	+	+	+	+	+	+	+	+	+	+	+	+
1235	1384	Late competence protein ComGD, access of DNA to ComEA, FIG038316	56	+	+	+	+	+	+	+	+	+	+	+	+	+	+	+	–	+	+	+	+	+	+	+	+	+	+	+	+	+	+	+	+	+	+	+	+	+	+	+	+	+	+	+	+	+	+	+	+	+	+	+	+	+	+	+
1236	1394	Diphosphomevalonate decarboxylase (EC 4.1.1.33)	56	+	+	+	+	+	+	+	+	+	+	+	+	+	+	+	+	+	+	+	+	+	+	+	+	+	+	+	+	+	+	+	+	+	+	+	+	+	+	+	+	+	+	+	+	+	+	+	+	+	+	+	–	+
1237	1396	ABC transporter, ATP-binding protein	56	+	+	+	+	+	+	+	+	+	+	+	+	+	+	+	+	+	+	+	–	+	+	+	+	+	+	+	+	+	+	+	+	+	+	+	+	+	+	+	+	+	+	+	+	+	+	+	+	+	+	+	+	+
1238	1397	hypothetical protein	56	+	+	+	+	+	+	+	+	+	+	+	+	+	+	+	+	+	+	+	+	+	+	+	+	+	+	+	+	+	+	+	+	+	+	+	+	+	+	–	+	+	+	+	+	+	+	+	+	+	+	+	+	+
1239	1398	Manganese ABC transporter, periplasmic-binding protein SitA	56	+	+	+	+	+	+	+	+	+	+	+	+	+	+	+	+	+	+	+	–	+	+	+	+	+	+	+	+	+	+	+	+	+	+	+	+	+	+	+	+	+	+	+	+	+	+	+	+	+	+	+	+	+
1240	1400	ABC-type multidrug transport system, ATPase component	56	+	+	+	+	+	+	+	+	+	+	+	+	+	+	+	+	+	+	+	+	+	+	+	+	+	+	+	+	+	+	+	+	+	+	+	+	+	+	+	+	+	+	+	+	+	+	+	+	+	–	+	+	+
1241	1401	Isopentenyl-diphosphate delta-isomerase, FMN-dependent (EC 5.3.3.2)	56	+	+	+	+	+	+	+	+	+	+	+	+	+	+	+	+	+	+	+	+	+	+	+	+	+	+	+	+	+	+	+	+	+	+	+	+	+	+	+	+	+	+	+	+	+	+	+	+	+	+	+	–	+
1242	1408	lantibiotic efflux protein	56	+	+	+	+	+	+	+	+	+	+	+	+	+	+	+	+	+	+	+	+	+	+	+	+	+	+	+	+	+	+	+	+	+	+	+	+	+	+	+	+	+	+	+	+	+	+	+	+	+	+	+	–	+
1243	1409	hypothetical protein	56	+	+	+	+	+	+	+	+	+	+	+	+	+	+	+	+	+	+	+	+	+	+	+	+	+	+	+	+	+	+	+	+	+	+	+	+	+	–	+	+	+	+	+	+	+	+	+	+	+	+	+	+	+
1244	1412	hypothetical protein	56	+	+	+	+	+	+	+	+	+	+	+	+	+	+	+	+	+	+	+	+	+	+	+	+	+	+	+	+	+	+	+	+	+	+	+	+	+	+	+	+	+	+	+	+	+	+	+	+	+	+	+	–	+
1245	1414	Formate/nitrite family of transporters	56	+	+	+	+	–	+	+	+	+	+	+	+	+	+	+	+	+	+	+	+	+	+	+	+	+	+	+	+	+	+	+	+	+	+	+	+	+	+	+	+	+	+	+	+	+	+	+	+	+	+	+	+	+	+	+
1246	1429	FIG04612: Integral membrane protein (putative)	56	+	+	+	+	+	+	+	+	+	+	+	+	+	+	+	+	+	+	+	+	+	+	+	+	+	+	+	+	+	+	+	+	+	+	+	+	+	+	+	+	+	+	+	+	+	+	+	+	+	–	+	+	+
1247	1431	Manganese ABC transporter, inner membrane permease protein SitC	56	+	+	+	+	+	+	+	+	+	+	+	+	+	+	+	+	+	+	+	–	+	+	+	+	+	+	+	+	+	+	+	+	+	+	+	+	+	+	+	+	+	+	+	+	+	+	+	+	+	+	+	+	+
1248	1433	Phosphomevalonate kinase (EC 2.7.4.2)	56	+	+	+	+	+	+	+	+	+	+	+	+	+	+	+	+	+	+	+	+	+	+	+	+	+	+	+	+	+	+	+	+	+	+	+	+	+	+	+	+	+	+	+	+	+	+	+	+	+	+	+	–	+
1249	1440	Uncharacterized protein involved in cytokinesis, contains TGc (transglutaminase/protease-like) domain	56	+	+	+	+	+	+	+	+	+	+	+	+	–	+	+	+	+	+	+	+	+	+	+	+	+	+	+	+	+	+	+	+	+	+	+	+	+	+	+	+	+	+	+	+	+	+	+	+	+	+	+	+	+	+	+
1250	1441	Predicted transcriptional regulator	56	+	+	+	+	+	+	+	+	+	+	+	+	+	+	+	+	+	+	+	+	+	+	+	+	+	+	+	+	+	+	+	+	+	+	+	+	+	+	+	+	+	+	+	+	+	+	+	+	–	+	+	+	+
1251	1442	Manganese ABC transporter, inner membrane permease protein SitD	56	+	+	+	+	+	+	+	+	+	+	+	+	+	+	+	+	+	+	+	–	+	+	+	+	+	+	+	+	+	+	+	+	+	+	+	+	+	+	+	+	+	+	+	+	+	+	+	+	+	+	+	+	+
1252	1443	Transcriptional regulator	56	+	+	+	+	+	+	+	+	+	+	+	+	+	+	+	+	+	+	+	–	+	+	+	+	+	+	+	+	+	+	+	+	+	+	+	+	+	+	+	+	+	+	+	+	+	+	+	+	+	+	+	+	+
1253	1446	DNA binding protein, FIG046916	56	+	+	+	+	+	+	+	+	+	+	+	+	+	+	+	+	+	+	+	+	+	+	+	+	+	+	+	+	+	+	+	+	+	+	+	+	+	+	+	+	+	+	+	+	+	+	+	+	+	+	–	+	+
1254	1448	hypothetical protein	56	+	+	+	+	+	+	+	+	+	+	+	+	+	+	+	+	+	+	+	+	+	+	+	+	+	+	+	+	+	+	+	+	+	+	+	+	+	+	+	+	+	+	+	+	+	+	+	+	+	–	+	+	+
1255	1453	Hydrolase (HAD superfamily)	56	+	+	+	+	+	+	+	+	+	+	+	+	+	+	+	+	+	+	+	+	+	+	+	+	+	+	+	+	+	+	+	+	–	+	+	+	+	+	+	+	+	+	+	+	+	+	+	+	+	+	+	+	+
1256	1454	hypothetical protein	56	+	+	+	+	+	+	+	+	+	+	+	+	+	+	+	+	+	+	+	+	+	+	+	+	+	+	+	+	+	+	+	+	–	+	+	+	+	+	+	+	+	+	+	+	+	+	+	+	+	+	+	+	+
1257	1461	ATPase component of ABC transporters with duplicated ATPase domains	56	+	+	+	+	+	+	+	+	+	+	+	+	+	+	+	+	+	+	+	+	+	+	+	+	+	+	+	+	+	+	+	+	+	+	+	+	+	+	+	+	+	+	+	+	+	+	+	+	+	+	+	+	–
1258	1465	hypothetical protein	56	+	+	+	+	+	+	+	+	+	+	+	+	+	+	+	+	+	+	+	–	+	+	+	+	+	+	+	+	+	+	+	+	+	+	+	+	+	+	+	+	+	+	+	+	+	+	+	+	+	+	+	+	+
1259	1469	hypothetical protein	56	+	+	+	+	+	+	+	+	+	+	+	+	+	+	+	+	+	+	+	–	+	+	+	+	+	+	+	+	+	+	+	+	+	+	+	+	+	+	+	+	+	+	+	+	+	+	+	+	+	+	+	+	+
1260	1476	Negative regulator of phenolic acid metabolism PadR	56	+	+	+	+	+	+	+	+	+	+	+	+	+	+	+	+	+	+	+	–	+	+	+	+	+	+	+	+	+	+	+	+	+	+	+	+	+	+	+	+	+	+	+	+	+	+	+	+	+	+	+	+	+
1261	1477	hypothetical protein	56	+	+	+	+	+	+	+	+	+	+	+	+	+	+	+	+	+	+	+	+	+	+	+	+	+	+	+	+	+	+	+	+	+	+	+	+	+	+	+	+	+	+	+	+	+	+	+	+	+	–	+	+	+
1262	1479	hypothetical protein	56	+	+	+	+	+	+	+	+	+	+	+	+	+	+	+	+	+	+	+	+	+	+	+	+	+	+	+	+	+	+	+	+	+	–	+	+	+	+	+	+	+	+	+	+	+	+	+	+	+	+	+	+	+
1263	1480	hypothetical protein	56	+	+	+	+	+	+	+	+	+	+	+	+	+	+	+	+	+	+	+	–	+	+	+	+	+	+	+	+	+	+	+	+	+	+	+	+	+	+	+	+	+	+	+	+	+	+	+	+	+	+	+	+	+
1264	1483	Thiol-disulfide isomerase and thioredoxin	56	+	+	+	+	+	+	+	+	+	–	+	+	+	+	+	+	+	+	+	+	+	+	+	+	+	+	+	+	+	+	+	+	+	+	+	+	+	+	+	+	+	+	+	+	+	+	+	+	+	+	+	+	+	+	+
1265	1491	Acyl-coenzyme A synthetases/AMP-(fatty) acid ligase	56	+	+	+	+	+	+	+	+	+	+	+	+	+	+	+	+	+	+	+	+	+	+	+	+	+	+	+	+	+	+	+	+	+	+	+	+	+	+	+	+	+	+	+	+	+	+	–	+	+	+	+	+	+
1266	1521	hypothetical protein	56	+	+	+	+	+	+	+	+	+	+	+	+	+	+	+	+	+	+	+	+	+	+	+	+	+	+	+	–	+	+	+	+	+	+	+	+	+	+	+	+	+	+	+	+	+	+	+	+	+	+	+	+	+	+	+
1267	462	DNA polymerase III delta subunit (EC 2.7.7.7)	55	+	+	+	+	+	+	+	+	+	+	+	+	+	+	+	+	+	+	+	–	+	+	+	+	+	+	+	+	+	+	+	+	+	+	+	+	+	–	+	+	+	+	+	+	+	+	+	+	+	+	+	+	+	+	+
1268	638	DNA repair protein RadC	55	–	–	+	+	+	+	+	+	+	+	+	+	+	+	+	+	+	+	+	+	+	+	+	+	+	+	+	+	+	+	+	+	+	+	+	+	+	+	+	+	+	+	+	+	+	+	+	+	+	+	+	+	+	+	+
1269	642	GTPase and tRNA-U34 5-formylation enzyme TrmE	55	+	+	+	+	+	+	+	+	+	+	+	+	+	+	+	+	+	+	+	+	+	+	+	+	+	+	+	+	+	+	+	+	+	–	+	+	+	+	+	+	+	+	+	+	+	+	+	+	+	+	+	–	+
1270	666	Cysteine synthase (EC 2.5.1.47)	55	+	+	+	+	+	+	+	+	+	+	+	+	+	+	+	+	+	+	+	+	+	+	–	+	+	+	+	+	+	+	+	+	+	+	+	+	+	–	+	+	+	+	+	+	+	+	+	+	+	+	+	+	+	+	+
1271	674	LSU m3Psi1915 methyltransferase RlmH	55	–	–	+	+	+	+	+	+	+	+	+	+	+	+	+	+	+	+	+	+	+	+	+	+	+	+	+	+	+	+	+	+	+	+	+	+	+	+	+	+	+	+	+	+	+	+	+	+	+	+	+	+	+	+	+
1272	684	Transcriptional repressor AdcR for Zn(2+)-responsive expression	55	–	–	+	+	+	+	+	+	+	+	+	+	+	+	+	+	+	+	+	+	+	+	+	+	+	+	+	+	+	+	+	+	+	+	+	+	+	+	+	+	+	+	+	+	+	+	+	+	+	+	+	+	+	+	+
1273	690	SSU ribosomal protein S1p	55	+	+	+	+	+	+	+	+	+	+	+	+	+	+	+	+	+	+	+	–	+	+	+	+	+	+	+	+	+	+	+	+	+	+	+	–	+	+	+	+	+	+	+	+	+	+	+	+	+	+	+	+	+	+	+
1274	721	Late competence protein ComGA, access of DNA to ComEA	55	–	–	+	+	+	+	+	+	+	+	+	+	+	+	+	+	+	+	+	+	+	+	+	+	+	+	+	+	+	+	+	+	+	+	+	+	+	+	+	+	+	+	+	+	+	+	+	+	+	+	+	+	+	+	+
1275	725	DNA repair protein RadA	55	+	+	+	+	+	+	+	+	+	+	+	+	–	+	+	+	+	+	+	+	+	+	+	+	+	+	+	+	+	+	+	+	+	+	+	+	+	+	+	+	+	+	+	+	+	+	+	+	+	+	+	–	+
1276	729	LSU ribosomal protein L31p @ LSU ribosomal protein L31p, zinc-independent	55	+	+	+	+	+	+	+	+	+	+	+	+	+	+	+	+	+	+	+	–	+	+	+	+	+	+	+	+	+	+	+	+	+	+	+	+	+	+	+	+	+	+	+	+	+	+	+	+	+	+	–	+	+	+	+
1277	792	ABC transporter membrane-spanning permease - glutamine transport	55	+	+	+	+	+	+	+	+	+	+	+	+	+	+	+	+	+	+	+	–	+	+	+	+	+	+	+	+	+	+	+	+	+	+	+	–	+	+	+	+	+	+	+	+	+	+	+	+	+	+	+	+	+	+	+
1278	800	NAD(P)HX dehydratase	55	+	+	+	+	+	+	+	+	+	+	+	+	+	+	+	+	+	+	+	+	+	+	+	+	+	+	+	+	+	+	+	+	+	+	+	–	–	+	+	+	+	+	+	+	+	+	+	+	+	+	+	+	+	+	+	+	+	+	+
1279	812	Glycerol-3-phosphate dehydrogenase (NAD(P)+) (EC 1.1.1.94)	55	+	+	+	+	+	+	+	+	+	+	+	+	+	+	+	+	+	+	+	–	+	+	+	+	+	+	+	+	+	+	+	+	+	+	+	+	+	+	+	+	+	+	+	+	+	+	+	+	+	+	+	+	+	–	+
1280	842	hypothetical protein	55	+	+	+	+	+	+	+	+	+	+	+	+	+	+	+	+	+	+	+	+	+	+	+	+	+	+	+	+	+	+	+	+	+	+	+	+	+	+	+	+	+	+	+	–	+	+	+	+	+	–	+	+	+
1281	849	Aspartate--ammonia ligase (EC 6.3.1.1)	55	+	+	+	+	+	+	+	+	+	+	+	+	+	+	+	+	+	+	+	–	+	+	+	+	+	+	+	+	+	+	+	+	+	+	+	+	+	+	+	+	+	+	+	+	+	+	+	+	+	+	+	+	+	–	+

Table S5. Precence/absence of each HG among 57 *S. suis* strains

Index[a]	ClusterID	Gene products	Members	1																13			25					28						104		94			76			53/54		Divergent *S. suis*																
				98HAH33	05ZYH33	SC84	A7	SS12	P1/7	DAT273	NIAH11434	JS14	GZ1	BM407	S735	13730	MNCM07	NCTC 10446	14636	NCTC 10237	ST1	2651	89-1591	MNCM04	MNCM25	8074	D9	4961	ST3	MNCM43	DAT292	DAT260	NIAH11435	MNCM21	MNCM50	89-2479	DAT234	DAT290	93A	42A	NT77	6407	11538	8830	12814	92-1400	89-590	2726	2524	89-3576-3	4417	D12	10581	89-5259	88-5299A	R61	92-1191	14A	92-4172	22083
1282	872	Exopolysaccharide biosynthesis transcriptional activator EpsA	55	+	+	+	+	+	+	+	+	–	+	+	+	+	–	+	+	+	+	+	+	+	+	+	+	+	+	+	+	+	+	+	+	+	+	+	+	+	+	+	+	+	+	+	+	+	+	+	+	+	+	+	+	+	+	+	+	+
1283	889	ABC transporter, ATP-binding/permease protein	55	+	+	+	+	+	+	+	+	+	–	+	+	+	+	+	+	+	+	+	–	+	+	+	+	+	+	+	+	+	+	+	+	+	+	+	+	+	+	+	+	+	+	+	+	+	+	+	+	+	+	+	+	+	+	+	+	+
1284	892	FIG053235: Diacylglucosamine hydrolase like	55	+	+	+	+	+	+	+	+	+	+	+	+	+	+	+	+	+	+	+	+	+	+	+	+	+	+	+	+	+	+	+	+	+	+	+	+	+	+	+	+	+	+	+	+	+	+	+	+	+	–	+	–	+	+	+	+	+
1285	895	Prephenate dehydrogenase (EC 1.3.1.12)	55	+	+	+	+	+	+	+	+	+	+	+	+	+	+	+	+	+	+	+	+	–	–	+	+	+	+	+	+	+	+	+	+	+	+	+	+	+	+	+	+	+	+	+	+	+	+	+	+	+	+	+	+	+	+	+	+	+
1286	907	ATP-dependent nuclease, subunit B	55	+	+	+	+	+	+	+	+	+	+	–	+	+	+	+	+	+	+	+	+	+	+	+	+	+	+	+	+	+	+	+	+	+	+	+	+	+	+	+	+	+	+	+	+	+	+	+	+	+	+	+	+	+	+	+	–	+
1287	916	Dihydropteroate synthase (EC 2.5.1.15)	55	+	+	+	+	+	+	+	+	+	+	+	+	+	+	+	+	+	+	+	+	+	+	+	+	+	+	+	+	+	+	+	+	+	+	+	+	+	+	+	+	+	+	+	+	+	–	+	+	–	+	+	+	+
1288	924	2,3,4,5-tetrahydropyridine-2,6-dicarboxylate N-acetyltransferase (EC 2.3.1.89)	55	+	+	+	+	+	+	+	+	+	+	+	+	+	+	+	+	+	+	+	–	+	+	+	+	+	+	+	+	+	+	+	+	+	+	+	+	+	+	+	+	+	+	+	+	+	+	+	+	+	+	+	+	+	–	+
1289	925	Dihydroorotate dehydrogenase electron transfer subunit (EC 1.3.3.1)	55	+	+	+	+	+	+	+	+	+	+	+	+	+	+	+	+	+	+	+	–	+	+	+	+	+	+	+	+	+	+	+	+	+	+	+	+	+	+	+	+	+	+	+	+	+	+	+	+	+	+	+	+	+	–	+
1290	947	MutT/nudix family protein	55	+	+	+	+	+	+	+	+	+	+	+	+	+	+	+	+	+	+	+	+	+	+	+	+	+	+	+	+	+	+	+	+	+	+	+	+	+	+	+	+	+	+	+	+	+	+	–	+	–	+	+	+	+
1291	953	Ascorbate-specific PTS system, EIIC component	55	+	+	+	+	+	+	+	+	+	+	+	+	+	+	+	+	+	+	+	+	+	+	+	+	+	+	+	+	+	+	+	+	+	+	+	+	+	+	+	+	+	–	+	+	+	+	+	+	+	+	–	+	+	+	+	+	+
1292	954	L-xylulose 5-phosphate 3-epimerase (EC 5.1.3.-)	55	+	+	+	+	+	+	+	+	+	+	+	+	+	+	+	+	+	+	+	+	+	+	+	+	+	+	+	+	+	+	+	+	+	+	+	+	+	+	+	+	+	–	+	+	+	+	+	+	+	+	–	+	+	+	+	+	+
1293	958	PTS system, IIA component, possible mannitol permease (EC 2.7.1.69)	55	+	+	+	+	+	+	+	+	+	+	+	+	+	+	+	+	+	+	+	+	+	+	+	+	+	+	+	+	+	+	+	+	+	+	+	+	+	+	+	–	+	+	+	+	+	+	+	+	–	+	+	+	+	+	+
1294	962	Ascorbate-specific PTS system, EIIB component (EC 2.7.1.69)	55	+	+	+	+	+	+	+	+	+	+	+	+	+	+	+	+	+	+	+	+	+	+	+	+	+	+	+	+	+	+	+	+	+	+	+	+	+	+	+	–	+	+	+	+	+	+	+	+	–	+	+	+	+	+	+
1295	964	Probable L-ascorbate-6-phosphate lactonase UlaG (EC 3.1.1.-) (L-ascorbate utilization protein G)	55	+	+	+	+	+	+	+	+	+	+	+	+	+	+	+	+	+	+	+	+	+	+	+	+	+	+	+	+	+	+	+	+	+	+	+	+	+	+	+	–	+	+	+	+	+	+	+	+	–	+	+	+	+	+	+
1296	974	L-ribulose-5-phosphate 4-epimerase (EC 5.1.3.4)	55	+	+	+	+	+	+	+	+	+	+	+	+	+	+	+	+	+	+	+	+	+	+	+	+	+	+	+	+	+	+	+	+	+	+	+	+	+	+	+	–	+	+	+	+	+	+	+	+	–	+	+	+	+	+	+
1297	981	Argininosuccinate synthase (EC 6.3.4.5)	55	+	+	+	+	+	+	+	+	+	+	+	+	+	+	+	+	+	+	+	+	+	+	+	+	+	+	+	+	+	+	+	+	+	+	+	+	+	+	–	–	+	+	+	+	+	+	+	+	+	+	+	+	+	+	+	+	+
1298	990	3-isopropylmalate dehydratase large subunit (EC 4.2.1.33)	55	+	+	+	+	+	+	+	+	+	+	+	+	+	+	+	+	+	+	+	+	+	+	+	+	+	+	+	+	+	+	+	+	+	+	+	+	–	–	+	+	+	+	+	+	+	+	+	+	+	+	+	+	+	+	+
1299	992	Phosphoenolpyruvate-dihydroxyacetone phosphotransferase (EC 2.7.1.121), subunit DhaM; DHA-specific IIA component	55	+	+	+	+	+	+	+	+	+	+	+	+	+	+	+	+	+	+	+	+	+	+	+	+	+	+	+	+	+	+	+	+	+	+	+	+	+	+	+	+	+	+	+	+	+	+	+	+	–	–	+	+	+
1300	999	Xaa-His dipeptidase (EC 3.4.13.3)	55	+	+	+	+	+	+	+	+	+	+	+	+	+	+	+	+	+	+	+	+	+	+	+	+	+	+	+	+	+	+	+	+	+	+	+	–	+	–	+	+	+	+	+	+	+	+	+	+	+	+	+	+	+	+	+	+	+
1301	1009	Phosphoenolpyruvate-dihydroxyacetone phosphotransferase (EC 2.7.1.121), ADP-binding subunit DhaL	55	+	+	+	+	+	+	+	+	+	+	+	+	+	+	+	+	+	+	+	+	+	+	+	+	+	+	+	+	+	+	+	+	+	+	+	+	+	+	+	+	+	+	+	+	+	+	+	+	–	–	+	+	+
1302	1021	FIG042921: similarity to aminoacyl-tRNA editing enzymes YbaK, ProX	55	+	+	+	+	+	+	+	+	+	+	+	+	+	+	+	+	+	+	+	+	+	+	+	+	+	+	+	+	+	+	+	+	+	+	+	–	–	+	+	+	+	+	+	+	+	+	+	+	+	+	+	+	+	+	+	+	+
1303	1025	ABC transporter ATP binding protein	55	+	+	+	+	+	+	+	+	+	+	+	+	+	+	+	+	+	+	+	+	+	+	+	+	+	+	+	+	+	+	+	+	+	+	+	+	+	+	–	+	+	+	+	+	+	+	+	+	+	+	+	–	+
1304	1026	UPF0337 protein M6_Spy1542	55	+	+	+	+	+	+	+	+	+	+	+	+	+	+	+	+	+	+	+	–	+	+	+	+	+	+	+	+	+	+	+	+	+	+	+	+	+	+	+	+	+	+	+	+	+	+	+	+	+	+	–	+	+	+	+	+	+
1305	1027	Phosphoenolpyruvate-dihydroxyacetone phosphotransferase (EC 2.7.1.121), dihydroxyacetone binding subunit DhaK	55	+	+	+	+	+	+	+	+	+	+	+	+	+	+	+	+	+	+	+	+	+	+	+	+	+	+	+	+	+	+	+	+	+	+	+	+	+	+	+	+	+	+	+	+	+	+	+	+	–	–	+	+	+
1306	1029	Dihydrolipoamide acetyltransferase	55	+	+	+	+	+	+	+	+	+	+	+	+	+	+	+	+	+	+	+	–	+	+	+	+	+	+	+	+	+	+	+	+	+	+	+	+	+	+	+	+	+	+	+	+	+	+	+	+	+	+	+	–	+
1307	1104	Imidazole glycerol phosphate synthase cyclase subunit (EC 4.1.3.-)	55	+	+	+	+	+	+	+	+	+	+	+	+	+	+	+	+	+	+	+	+	+	+	+	+	+	+	+	+	+	+	+	+	+	+	+	+	–	–	+	+	+	+	+	+	+	+	+	+	+	+	+	+	+	+	+
1308	1105	Transcriptional regulator, TetR family	55	+	+	+	+	+	+	+	+	+	+	+	+	+	+	+	+	+	+	+	+	+	+	+	+	+	+	+	+	+	+	+	+	+	+	+	+	+	+	+	+	+	+	+	+	+	+	+	+	–	–	+	+	+
1309	1111	dihydroxyacetone kinase family protein	55	+	+	+	+	+	+	+	+	+	+	+	+	+	+	+	+	+	+	+	+	+	+	+	+	+	+	+	+	+	+	+	+	+	+	+	+	+	+	+	+	+	+	+	+	+	+	+	+	–	–	+	+	+
1310	1141	hypothetical protein	55	+	+	+	+	+	+	+	+	+	+	+	+	+	+	+	+	+	+	+	+	+	+	+	+	+	+	+	+	+	+	+	+	+	+	+	+	+	–	+	+	+	+	+	+	+	+	–	+	+	+	+	+	+
1311	1154	Cu+ P-type ATPase	55	+	+	+	+	+	+	+	+	+	+	+	+	+	–	+	+	+	+	+	+	+	+	+	+	+	+	+	+	+	+	+	+	+	+	+	+	+	+	+	+	+	+	+	+	+	+	+	+	+	+	+	–	+
1312	1179	Pyrroline-5-carboxylate reductase (EC 1.5.1.2)	55	+	+	+	+	+	+	+	+	+	+	+	+	+	+	+	+	+	+	+	+	+	+	+	+	+	+	+	+	+	+	+	+	+	+	+	–	+	+	+	+	+	+	+	+	+	+	+	+	+	+	+	+	+	–	+
1313	1256	PTS system, N-acetylglucosamine-specific IIA component (EC 2.7.1.69) / PTS system, N-acetylglucosamine-specific IIB component (EC 2.7.1.69) / PTS system, N-acetylglucosamine-specific IIC component (EC 2.7.1.69)	55	+	+	+	+	+	+	+	+	+	+	+	+	+	+	+	+	+	+	+	+	+	+	+	+	+	+	+	+	+	+	+	+	+	+	+	+	+	+	+	+	+	+	+	+	+	+	+	–	+	+	–	+	+
1314	1278	Transcriptional antiterminator with PTS regulation domain, SPy0181 ortholog	55	+	+	+	+	+	+	+	+	+	+	+	+	+	+	+	+	+	+	+	+	+	+	+	+	+	+	+	+	+	+	+	+	+	+	+	+	+	–	+	+	+	+	+	+	+	+	–	+	+	+	+	+	+
1315	1288	Multi antimicrobial extrusion protein (Na(+)/drug antiporter), MATE family of MDR efflux pumps	55	+	+	+	+	+	+	+	+	+	+	+	+	+	+	+	+	+	+	+	–	+	+	+	+	+	+	+	+	+	+	+	+	+	+	+	+	+	+	+	+	+	+	+	+	+	+	+	+	+	+	+	–	+
1316	1289	SMT0608 replicon stabilization toxin	55	+	+	+	+	+	+	+	+	+	+	+	+	+	+	+	+	+	+	+	+	+	+	+	+	+	+	+	+	+	+	+	+	+	+	+	+	+	+	+	–	+	+	–	+	+	+	+	+	+	+	+	+	+	+	+
1317	1301	Mevalonate kinase (EC 2.7.1.36)	55	+	+	+	+	+	+	+	+	+	+	+	+	–	+	+	+	+	+	+	+	+	+	+	+	+	+	+	+	+	+	+	+	+	+	+	+	+	+	+	+	+	+	+	+	+	+	+	+	+	+	+	–	+
1318	1366	hypothetical protein	55	+	+	+	+	+	+	+	+	+	+	+	+	+	+	+	+	–	–	+	+	+	+	+	+	+	+	+	+	+	+	+	+	+	+	+	+	+	+	+	+	+	+	+	+	+	+	+	+	+	+	+	+	+	+	+
1319	1438	Oligoendopeptidase F	55	+	+	+	+	+	+	+	+	+	+	+	+	+	+	+	+	+	+	+	–	+	+	+	+	+	+	+	+	+	+	+	+	+	+	+	+	+	–	+	+	+	+	+	+	+	+	+	+	+	+	+	+	+	+	+	+	+
1320	1445	hypothetical protein	55	+	+	+	+	+	+	+	+	+	+	+	+	+	+	+	+	+	+	+	+	+	+	+	+	–	–	+	+	+	+	+	+	+	+	+	+	+	+	+	+	+	+	+	+	+	+	+	+	+	+	+	+	+	+	+
1321	1447	Predicted metal-dependent membrane protease	55	–	–	+	+	+	+	+	+	+	+	+	+	+	+	+	+	+	+	+	+	+	+	+	+	+	+	+	+	+	+	+	+	+	+	+	+	+	+	+	+	+	+	+	+	+	+	+	+	+	+	+	+	+	+	+
1322	1456	hypothetical protein	55	+	+	+	+	+	+	+	+	+	+	+	+	+	+	+	+	+	+	+	+	+	+	+	+	+	+	+	+	+	+	+	+	+	+	+	+	+	+	+	+	+	+	+	+	+	+	+	+	+	+	–	–	+
1323	1462	Phosphomannomutase	55	+	+	+	+	+	+	+	+	+	+	+	+	+	+	+	+	+	+	+	+	+	+	+	+	+	+	+	+	+	+	+	+	+	+	+	+	+	+	+	+	+	+	+	+	+	+	+	+	+	+	–	–	+
1324	1472	hypothetical protein	55	+	+	+	+	+	+	+	+	+	+	+	+	+	+	+	+	+	+	+	+	+	+	+	+	+	+	+	+	+	+	+	+	+	+	+	+	+	+	+	+	+	+	+	+	+	–	+	–	+	+	+	+	+
1325	1474	hypothetical protein	55	+	+	+	+	+	+	+	+	+	+	+	+	+	+	+	+	+	+	+	+	+	+	+	+	+	+	+	+	+	–	+	+	+	+	+	+	+	+	+	+	+	+	+	+	+	+	+	+	+	+	–	+	+	+	+
1326	1485	hypothetical protein	55	+	+	+	+	+	+	+	+	+	+	+	+	+	+	+	+	+	+	+	+	+	+	+	+	+	+	+	+	+	+	+	+	+	+	+	+	+	+	+	+	+	+	+	+	+	–	+	–	+	+	+	+	+
1327	1496	hypothetical protein	55	+	+	+	+	+	+	+	+	+	+	+	+	+	+	+	+	+	+	+	–	+	+	+	+	+	+	+	+	+	+	+	+	+	+	+	+	+	+	+	+	+	+	+	+	+	–	+	+	+	+	+	+	+	+	+	+	+
1328	1525	similar to membrane protein	55	+	+	+	+	+	+	+	+	+	+	+	+	+	+	+	+	+	+	+	+	+	+	+	+	+	+	+	–	+	+	+	+	+	–	+	+	+	+	+	+	+	+	+	+	+	+	+	+	+	+	+	+	+	+	+
1329	1532	Predicted lactoylglutathione lyase	55	+	+	+	+	+	+	+	+	+	+	+	+	+	+	+	+	+	+	+	+	+	+	+	+	+	+	+	+	+	+	+	+	+	+	+	+	+	+	+	+	+	+	–	+	+	+	+	+	+	–	+	+	+
1330	1549	hypothetical protein	55	+	+	+	+	+	+	+	+	+	+	+	+	+	+	+	+	+	+	–	+	+	+	+	+	+	–	+	+	+	+	+	+	+	+	+	+	+	+	+	+	+	+	+	+	+	+	+	+	+	+	+	+	+	+	+
1331	36	MutT/nudix family protein	54	+	+	+	+	+	+	+	+	+	+	+	+	+	+	+	+	+	+	+	+	+	+	+	+	+	+	+	+	+	+	+	+	–	–	–	+	+	+	+	+	+	+	+	+	+	+	+	+	+	+	+	+	+	+	+	+	+
1332	665	Late competence protein ComEA, DNA receptor	54	+	+	+	+	+	+	+	+	+	+	+	+	+	+	+	+	–	–	+	+	+	+	+	+	+	+	+	+	+	+	+	+	+	+	+	+	+	+	+	+	+	+	+	+	+	+	+	+	+	+	+	+	+	–	+
1333	672	Putative coproporphyrinogen III oxidase of BS HemN-type, oxygen-independent (EC 1.3.99.22), in heat shock gene cluster	54	+	+	+	+	+	+	+	+	+	+	+	+	+	+	+	+	+	+	+	+	+	+	+	+	–	+	+	+	+	+	+	+	+	+	+	+	–	+	+	+	+	+	+	+	+	+	+	+	+	+	+	+	–	+	+	+	+
1334	737	ABC transporter permease protein	54	+	+	+	–	+	+	+	+	+	+	+	+	+	+	+	+	+	+	+	+	+	+	+	+	+	+	+	+	+	+	+	+	+	+	+	+	+	+	+	+	+	+	+	+	+	+	+	+	+	+	–	+	+	+	–	+	+
1335	763	FIG007079: UPF0348 protein family	54	+	+	+	+	+	+	+	+	+	+	+	+	+	+	+	+	+	+	+	+	+	–	+	+	–	+	+	+	+	+	+	+	+	+	+	+	+	+	+	+	+	+	+	+	+	+	+	+	+	+	+	+	+	–	+
1336	774	D-alanine--poly(phosphoribitol) ligase subunit 1 (EC 6.1.1.13)	54	+	+	+	+	+	+	+	+	+	+	+	+	+	+	+	+	+	+	+	+	+	+	+	+	+	+	+	+	+	+	+	+	+	+	+	+	+	+	+	+	+	+	+	+	+	+	+	+	+	+	+	–	+	–	–
1337	777	D-alanyl transfer protein DltB	54	+	+	+	+	+	+	+	+	+	+	+	+	+	+	+	+	+	+	+	+	+	+	+	+	+	+	+	+	+	+	+	+	+	+	+	+	+	+	+	+	+	+	+	+	+	+	+	+	+	+	+	–	+	–	–
1338	787	D-alanine--poly(phosphoribitol) ligase subunit 2 (EC 6.1.1.13)	54	+	+	+	+	+	+	+	+	+	+	+	+	+	+	+	+	+	+	+	+	+	+	+	+	+	+	+	+	+	+	+	+	+	+	+	+	+	+	+	+	+	+	+	+	+	+	+	+	+	+	+	–	+	–	–
1339	801	ABC transporter, ATP-binding/permease protein	54	+	+	+	+	+	+	+	+	+	+	+	+	+	+	+	+	+	+	+	+	+	+	+	+	+	+	+	+	+	+	+	+	+	+	+	+	+	+	+	+	+	+	+	+	+	+	+	+	+	+	–	–	+	+	–
1340	859	Putative Dihydrolipoamide dehydrogenase (EC 1.8.1.4); Mercuric ion reductase (EC 1.16.1.1); PF00070 family, FAD-dependent NAD(P)-disulphide oxidoreductase	54	+	+	+	+	+	+	+	+	+	+	+	+	+	+	+	+	+	+	+	+	+	+	+	+	+	+	+	+	+	+	+	+	+	+	+	+	+	+	–	–	+	+	–	+	+	+	+	+	+	+	+	+	+	+	+	+	+
1341	908	ABC transporter ATP-binding protein uup	54	+	+	+	+	+	+	+	+	+	+	+	+	+	–	+	+	+	+	+	+	+	+	–	+	+	+	–	+	+	+	+	+	+	+	+	+	+	+	+	+	+	+	+	+	+	+	+	+	+	+	+	+	+	+	+	+	+
1342	940	integral membrane protein	54	+	+	–	+	+	+	+	+	+	+	+	+	+	+	+	+	+	+	+	–	+	+	+	+	+	+	+	+	+	+	+	+	+	+	+	+	+	+	+	+	–	+	+	+	+	+	+	+	+	+	+	+	+	+	+	+	+
1343	944	Glycerate kinase (EC 2.7.1.31)	54	+	+	+	+	+	+	+	+	+	+	+	+	+	+	+	+	+	+	+	+	+	+	+	+	+	+	+	+	+	+	+	+	+	+	+	–	–	–	+	+	+	+	+	+	+	+	+	+	+	+	+	+	+	+	+	+	+
1344	948	Poly(glycerophosphate chain) D-alanine transfer protein DltD	54	+	+	+	+	+	+	+	+	+	+	+	+	+	+	+	+	+	+	+	+	+	+	+	+	+	+	+	+	+	+	+	+	+	+	+	+	+	+	+	+	+	+	+	+	+	+	+	+	+	+	+	–	+	–	–
1345	978	3-keto-L-gulonate 6-phosphate decarboxylase	54	+	+	+	+	+	+	+	+	+	+	+	+	+	+	+	+	+	+	+	+	+	+	+	+	+	+	+	+	+	+	+	+	+	+	+	+	+	+	+	–	+	+	+	+	+	+	+	+	–	+	+	+	+	–	+
1346	1078	Multiple sugar ABC transporter, substrate-binding protein	54	+	+	+	+	+	+	+	+	+	+	+	+	+	+	+	+	–	–	–	+	+	+	+	+	+	+	+	+	+	+	+	+	+	+	+	+	+	+	+	+	+	+	+	+	+	+	+	+	+	+	+	+	+	+	+	+	+
1347	1099	Tellurite resistance protein TehB	54	+	+	+	+	+	+	+	+	+	+	+	+	+	+	+	+	+	+	+	+	+	–	+	+	+	+	+	+	+	+	+	–	+	+	+	+	+	+	+	+	+	+	+	+	+	+	+	+	+	+	+	–	+	+	+	+	+
1348	1103	YpfJ protein, zinc metalloprotease superfamily	54	+	+	+	+	+	+	+	+	+	+	+	+	+	+	+	+	+	+	+	+	+	+	+	+	+	+	+	+	+	+	+	+	+	+	+	+	+	+	+	+	+	+	+	+	+	–	–	+	–	+	+	+	+
1349	1124	Sucrose phosphorylase (EC 2.4.1.7)	54	+	+	+	+	+	+	+	+	+	+	+	+	+	+	+	+	–	–	–	+	+	+	+	+	+	+	+	+	+	+	+	+	+	+	+	+	+	+	+	+	+	+	+	+	+	+	+	+	+	+	+	+	+	+	+	+	+
1350	1134	Multiple sugar ABC transporter, membrane-spanning permease protein MsmG	54	+	+	+	+	+	+	+	+	+	+	+	+	+	+	+	+	–	–	–	+	+	+	+	+	+	+	+	+	+	+	+	+	+	+	+	+	+	+	+	+	+	+	+	+	+	+	+	+	+	+	+	+	+	+	+	+	+
1351	1136	Multiple sugar ABC transporter, membrane-spanning permease protein MsmF	54	+	+	+	+	+	+	+	+	+	+	+	+	+	+	+	+	–	–	–	+	+	+	+	+	+	+	+	+	+	+	+	+	+	+	+	+	+	+	+	+	+	+	+	+	+	+	+	+	+	+	+	+	+	+	+	+	+
1352	1144	5'-nucleotidase (EC 3.1.3.5)	54	+	+	+	+	+	+	+	+	+	+	+	+	+	+	+	+	+	+	+	+	+	+	+	+	+	+	+	+	+	+	+	+	+	+	+	+	–	+	+	–	+	+	+	+	+	+	+	+	+	–	+	+	+	+	+	+	+
1353	1173	Glycosyltransferases involved in cell wall biogenesis	54	+	+	+	+	+	+	+	+	+	+	+	+	+	+	+	+	+	+	+	+	+	+	+	+	+	+	+	+	+	+	+	+	+	+	+	+	+	+	+	+	+	+	+	+	+	+	+	+	–	–	–	+	+	+	+
1354	1181	Acetyltransferase (EC 2.3.1.-)	54	+	+	+	+	+	+	+	+	+	+	+	+	–	+	+	+	+	+	+	+	+	+	+	+	+	+	+	+	+	+	+	+	+	+	+	+	+	+	+	+	+	+	+	+	+	+	+	+	+	+	–	+	+	+	–	+	+
1355	1192	MSM (multiple sugar metabolism) operon regulatory protein	54	+	+	+	+	+	+	+	+	+	+	+	+	+	+	+	+	–	–	–	+	+	+	+	+	+	+	+	+	+	+	+	+	+	+	+	+	+	+	+	+	+	+	+	+	+	+	+	+	+	+	+	+	+	+	+	+	+
1356	1195	hypothetical protein	54	+	+	+	+	+	+	+	+	+	+	+	+	+	+	+	+	+	+	+	+	+	+	+	+	+	+	+	+	+	+	+	+	+	+	+	+	+	+	+	+	–	+	+	+	+	+	+	+	+	+	+	+	+	–	–
1357	1228	Duplicated ATPase component BL0693 of energizing module of predicted ECF transporter	54	+	+	+	+	+	+	+	+	+	+	+	+	+	+	+	+	+	+	+	+	+	+	+	+	+	+	+	+	–	–	+	+	+	+	+	+	+	+	+	+	+	+	+	+	+	+	+	+	+	+	+	–	+	+	+
1358	1271	ABC-type multidrug transport system, ATPase component	54	+	+	+	+	+	+	+	+	+	+	+	+	+	+	+	–	+	+	+	+	+	+	+	+	+	+	+	+	+	+	+	+	+	+	+	+	+	+	+	+	+	+	+	+	+	+	+	+	+	+	+	+	+	–	+	–	+
1359	1277	hypothetical protein	54	+	+	+	+	+	+	+	+	+	+	+	+	+	+	–	+	+	+	+	+	+	+	+	+	+	+	+	+	+	+	+	+	+	+	+	+	+	+	+	+	+	+	–	+	+	+	+	+	+	+	–	+	+	+	+	+	+
1360	1279	ATPase (PilT family)	54	+	+	+	+	+	+	+	+	+	+	+	+	+	+	+	+	+	+	+	–	+	+	+	+	+	–	+	+	+	+	+	+	+	+	+	+	+	+	+	+	+	+	+	+	+	+	+	+	+	–	+	+	+	+	+	+	+
1361	1470	Membrane protein with 3 strong transmembrane helices	54	+	+	+	+	+	+	+	+	+	+	+	+	+	+	+	+	+	+	+	+	+	+	+	+	+	+	+	+	+	+	+	+	+	+	+	+	+	+	+	+	+	+	+	+	+	+	+	+	–	–	–	+	+	+	+
1362	1481	2-haloalkanoic acid dehalogenase (EC 3.8.1.2)	54	+	+	+	+	+	+	+	+	+	+	+	+	+	+	+	+	+	+	+	+	+	+	+	+	+	+	+	+	+	+	+	+	+	+	+	+	+	+	+	+	+	+	–	+	+	+	+	+	+	–	+	–	+	+	+	+	+
1363	1488	Alpha-galactosidase (EC 3.2.1.22)	54	+	+	+	+	+	+	+	+	+	+	+	+	+	+	+	+	–	–	–	+	+	+	+	+	+	+	+	+	+	+	+	+	+	+	+	+	+	+	+	+	+	+	+	+	+	+	+	+	+	+	+	+	+	+	+	+	+
1364	1503	hypothetical protein	54	+	+	+	+	+	+	+	+	+	+	+	+	+	+	+	+	+	+	+	+	+	+	+	+	+	+	+	+	+	+	+	+	+	+	+	+	+	+	+	+	+	+	+	+	+	+	+	+	+	+	–	–	–	+	+
1365	1517	hypothetical protein	54	+	+	+	+	+	+	+	+	+	+	+	+	+	+	+	+	–	–	+	+	+	+	+	+	+	+	+	+	+	+	+	+	+	+	+	+	–	+	+	+	+	+	+	+	+	+	+	+	+	+	+	+	+	+	+	+	+
1366	1522	hypothetical protein	54	+	+	+	+	+	+	+	+	+	+	+	+	+	+	+	+	+	+	+	+	+	+	+	+	+	+	+	+	+	+	+	+	+	+	+	+	+	+	+	+	+	+	+	+	+	+	+	+	–	–	–	+	+	+	+
1367	1523	Mannosyl-glycoprotein endo-beta-N-acetylglucosamidase	54	+	+	+	+	+	+	+	+	+	+	+	+	+	+	+	+	+	+	+	+	+	+	+	+	+	+	+	+	+	+	+	+	+	+	+	+	+	+	+	+	+	+	+	+	+	+	+	+	–	–	–	+	+	+	+
1368	1530	Transcriptional regulator, TetR family	54	+	+	+	+	+	+	+	+	+	+	+	+	+	+	+	+	+	+	+	+	+	+	+	+	+	+	+	+	+	+	+	+	+	+	+	+	+	+	+	+	+	+	+	+	+	+	+	+	+	–	+	+	+	–	–
1369	1533	hypothetical protein	54	+	+	+	+	+	+	+	+	+	+	+	+	+	+	+	+	+	+	+	+	+	+	+	+	+	+	+	+	+	+	+	+	+	+	+	–	+	–	+	+	+	+	+	+	+	+	+	+	+	+	+	+	+	–	+	+	+
1370	1536	Transmembrane component BL0694 of energizing module of predicted ECF transporter	54	+	+	+	+	+	+	+	+	+	+	+	+	+	+	+	+	+	+	+	+	+	+	+	+	+	+	+	+	–	–	+	+	+	+	+	+	+	+	+	+	+	+	+	+	+	+	+	+	+	+	+	–	+	+	+
1371	1547	ABC-type multidrug/protein/lipid transport system, ATPase component	54	+	+	+	+	+	+	+	+	+	+	+	+	+	+	+	+	+	+	+	–	+	+	+	+	+	+	+	+	+	+	–	–	+	+	+	+	+	+	+	+	+	+	+	+	+	+	+	+	+	+	+	+	+	+	+	+	+
1372	1558	McrBC 5-methylcytosine restriction system component	54	+	+	+	+	+	+	+	+	+	+	+	+	+	+	+	+	+	+	–	+	+	+	+	+	+	+	+	+	+	+	+	+	+	+	+	+	+	+	+	+	+	+	+	+	+	+	+	+	+	+	–	–	+	+	+	+	+
1373	1567	similar to other proteins, putative transmembrane protein	54	+	+	+	+	+	+	+	+	+	+	+	+	+	+	+	+	+	+	+	+	+	+	+	+	+	+	+	+	+	+	–	–	+	+	+	+	+	+	+	+	–	+	+	+	+	+	+	+	+	+	+	+	+	+	+	+	+
1374	708	Phospho-N-acetylmuramoyl-pentapeptide-transferase (EC 2.7.8.13)	53	+	+	+	+	+	+	+	–	+	+	+	+	+	+	+	+	+	+	+	+	+	+	+	+	+	+	+	+	+	+	+	+	+	+	–	+	+	+	–	+	+	+	+	+	–	+	+	+	+	+	+	+	+	+	+	+	+
1375	709	Undecaprenyl-diphosphatase (EC 3.6.1.27)	53	+	+	+	+	+	+	+	+	+	+	+	+	+	+	+	+	+	+	+	+	+	+	+	+	–	+	+	+	+	–	–	+	+	+	+	+	+	+	+	+	+	+	+	+	+	+	+	+	+	+	+	+	+	+	–	+	+
1376	732	NAD(FAD)-utilizing dehydrogenases	53	+	+	+	+	+	+	+	+	+	+	+	+	+	+	+	+	+	+	+	+	+	+	+	+	+	+	+	+	+	+	+	+	+	+	+	+	–	+	+	–	+	+	+	+	+	+	+	+	+	–	+	+	+	–	+	+	+
1377	744	GTP-binding protein HflX	53	+	+	+	+	+	+	+	+	+	+	+	+	+	+	+	+	+	–	+	–	+	+	+	+	+	–	+	+	+	+	+	+	+	+	+	+	+	+	+	+	+	+	+	+	+	+	+	+	+	–	+	+	+	+	+	+	+
1378	820	ABC transporter, ATP-binding/permease protein	53	+	+	+	+	+	+	+	+	+	+	+	+	+	+	+	+	+	+	+	–	+	+	+	+	+	+	+	+	+	+	+	+	+	+	+	+	+	+	+	+	+	+	+	+	+	+	+	+	+	+	+	+	–	–	+	+	–
1379	834	Copper-translocating P-type ATPase (EC 3.6.3.4)	53	+	+	+	+	+	+	+	+	+	+	+	+	–	+	+	+	+	+	+	+	+	+	+	+	+	+	+	–	+	+	+	+	+	+	+	+	+	–	+	+	+	+	+	+	+	+	+	+	+	+	+	+	+	+	+	–	+
1380	923	ABC transporter ATP binding protein	53	+	+	+	+	+	+	+	+	+	+	+	+	+	+	+	+	+	+	+	+	+	+	+	+	–	+	+	+	+	+	+	+	+	+	+	+	–	+	+	+	+	+	+	+	+	+	+	+	+	–	+	+	+	+	+	–	+
1381	943	LrgA-associated membrane protein LrgB	53	+	+	+	+	+	+	+	+	+	+	+	+	+	+	+	+	+	+	+	+	+	+	+	+	+	+	+	+	+	+	+	+	+	+	+	+	+	+	+	+	+	+	+	+	–	+	–	–	–	+	+	+	+	+	+	+	+
1382	1005	Dihydrofolate reductase (EC 1.5.1.3)	53	+	+	+	+	+	+	+	+	+	+	+	+	+	+	+	+	+	+	+	+	+	+	+	+	+	+	+	+	+	+	+	+	+	+	+	–	–	–	+	+	+	+	+	+	+	+	+	+	+	+	+	+	+	–	+	+	+
1383	1035	hypothetical protein	53	+	+	+	+	+	+	+	+	+	+	+	+	+	+	+	+	+	+	+	+	+	+	+	+	+	+	+	+	+	+	+	+	+	+	+	+	+	+	+	–	+	+	+	+	+	+	+	+	+	–	+	+	–	–	+
1384	1037	INTEGRAL MEMBRANE PROTEIN (Rhomboid family)	53	+	+	+	+	+	+	+	+	+	+	+	+	+	+	+	+	+	+	+	+	+	+	+	+	+	+	+	+	+	+	+	+	+	+	+	+	+	+	+	+	+	+	+	+	+	+	+	–	–	–	–	+	+	+	+
1385	1175	Transcriptional regulator, GntR family	53	+	+	+	+	+	+	+	+	+	+	+	+	+	+	+	+	+	+	+	+	+	+	+	+	+	+	+	+	+	+	+	+	+	+	+	+	+	+	+	+	+	+	+	–	+	+	+	+	+	+	+	–	+	+	+	–	–
1386	1177	Substrate-specific component BL0695 of predicted ECF transporter	53	+	+	+	+	+	+	+	+	+	+	+	+	+	+	+	+	+	+	+	–	+	+	+	+	+	+	+	+	–	–	+	+	+	+	+	+	+	+	+	+	+	+	+	+	+	+	+	+	+	+	+	+	+	–	+	+	+
1387	1208	Two-component system response regulator	53	+	+	+	+	+	+	+	+	+	+	+	+	+	+	+	+	+	+	+	+	+	+	+	+	+	+	+	+	+	+	+	+	+	+	+	+	+	+	+	+	+	+	+	+	+	+	+	–	–	–	–	+	+	+	+
1388	1222	D-mannonate oxidoreductase (EC 1.1.1.57)	53	+	+	+	+	+	+	+	+	+	+	+	+	+	+	+	+	+	+	+	+	+	+	+	+	+	+	+	+	+	+	+	+	+	+	+	+	+	+	+	+	+	+	+	–	+	+	+	+	+	+	+	–	+	+	+	–	–
1389	1232	2-dehydro-3-deoxygluconate kinase (EC 2.7.1.45)	53	+	+	+	+	+	+	+	+	+	+	+	+	+	+	+	+	+	+	+	+	+	+	+	+	+	+	+	+	+	+	+	+	+	+	+	+	+	+	+	+	+	+	+	–	+	+	+	+	+	+	+	–	+	+	+	–	–
1390	1236	Hydrolase, haloacid dehalogenase-like family	53	+	+	+	+	+	+	+	+	+	+	+	+	+	+	+	+	+	+	+	+	+	+	+	+	+	+	+	+	+	+	+	+	+	+	+	+	+	+	+	+	+	+	+	–	+	+	+	+	+	+	+	–	+	+	+	–	–
1391	1240	Beta-hexosaminidase (EC 3.2.1.52)	53	+	+	+	+	+	+	+	+	+	+	+	+	+	+	+	+	+	+	+	+	+	+	+	+	+	+	+	+	+	+	+	+	+	+	+	+	+	+	+	+	+	+	+	–	+	+	+	+	+	+	+	–	+	+	+	–	–
1392	1244	4-Hydroxy-2-oxoglutarate aldolase (EC 4.1.3.16) / 2-dehydro-3-deoxyphosphogluconate aldolase (EC 4.1.2.14)	53	+	+	+	+	+	+	+	+	+	+	+	+	+	+	+	+	+	+	+	+	+	+	+	+	+	+	+	+	+	+	+	+	+	+	+	+	+	+	+	+	+	+	+	–	+	+	+	+	+	+	+	–	+	+	+	–	–
1393	1246	Uronate isomerase (EC 5.3.1.12)	53	+	+	+	+	+	+	+	+	+	+	+	+	+	+	+	+	+	+	+	+	+	+	+	+	+	+	+	+	+	+	+	+	+	+	+	+	+	+	+	+	+	+	+	–	+	+	+	+	+	+	+	–	+	+	+	–	–
1394	1266	Beta-glucuronidase (EC 3.2.1.31)	53	+	+	+	+	+	+	+	+	+	+	+	+	+	+	+	+	+	+	+	+	+	+	+	+	+	+	+	+	+	+	+	+	+	+	+	+	+	+	+	+	+	+	+	–	+	+	+	+	+	+	+	–	+	+	+	–	–
1395	1444	SMT0609 replicon stabilization protein (antitoxin to SMT0608)	53	–	–	+	+	+	+	+	+	+	+	+	+	+	+	+	+	+	+	+	+	+	+	+	+	+	+	+	+	+	+	+	+	+	+	+	+	+	+	+	+	+	–	+	+	–	+	+	+	+	+	+	+	+	+	+	+	+
1396	1466	hypothetical protein	53	+	+	+	+	+	+	+	+	+	+	+	+	+	+	+	+	+	+	+	+	+	+	+	+	+	+	+	+	+	+	+	+	+	+	+	+	+	+	+	+	+	+	+	+	+	+	+	–	–	–	–	+	+	+	+
1397	1501	Two-component system histidine kinase	53	+	+	+	+	+	+	+	+	+	+	+	+	+	+	+	+	+	+	+	+	+	+	+	+	+	+	+	+	+	+	+	+	+	+	+	+	+	+	+	+	+	+	+	+	+	+	+	–	–	–	–	+	+	+	+
1398	1512	ABC-type uncharacterized transport system, ATPase component	53	+	+	+	+	+	+	+	+	+	+	+	+	+	+	+	+	+	+	+	+	+	+	+	+	+	+	+	+	+	+	+	+	+	+	+	+	+	+	+	+	–	+	–	+	+	+	+	+	+	–	+	+	+	+	–
1399	1516	Permease of the major facilitator superfamily	53	+	+	+	+	+	+	+	+	+	+	+	+	+	+	+	–	–	+	+	+	+	+	+	+	–	+	+	+	+	+	+	+	+	+	+	+	+	+	+	+	+	+	+	+	+	+	+	+	+	+	+	+	–	+	+	+	+
1400	1529	hypothetical protein	53	+	+	+	+	+	+	+	+	+	+	+	+	+	+	+	+	+	+	+	+	+	+	+	+	+	+	+	+	+	+	+	+	+	+	+	–	+	+	+	+	+	+	+	+	+	+	+	–	–	+	+	+	+	+	–
1401	1534	Beta-hexosaminidase (EC 3.2.1.52)	53	+	+	+	+	+	+	+	+	+	+	+	+	+	+	+	+	+	+	+	+	+	+	+	+	+	+	+	+	+	+	+	+	+	+	+	+	+	+	+	+	+	–	+	+	+	+	+	+	+	–	+	+	+	–	–
1402	1541	Phosphoglycerol transferase and related proteins, alkaline phosphatase superfamily	53	+	+	+	+	+	+	+	+	+	+	+	+	+	+	+	+	+	+	+	+	+	+	+	+	+	+	+	+	+	+	+	+	+	+	+	+	+	+	+	–	+	+	+	+	+	+	+	+	–	–	–	+	+	+	+
1403	1555	glycosyl transferase family 8	53	+	+	+	+	+	+	+	+	+	+	+	+	+	+	+	+	+	+	+	+	+	+	+	+	+	+	+	+	+	+	+	+	+	+	+	+	+	+	+	+	+	+	+	+	+	+	+	–	–	–	–	+	+	+	+
1404	1566	hypothetical protein	53	+	+	+	+	+	+	+	+	+	+	+	+	+	+	+	+	+	+	+	+	+	+	+	+	+	+	+	+	+	+	+	+	–	+	–	+	+	+	+	+	–	+	+	+	+	+	+	+	+	+	+	+	+	+	+	+	–
1405	1575	Subtilisin-like serine protease	53	+	+	+	+	+	+	+	+	+	+	+	+	+	+	+	+	+	+	+	+	+	+	+	+	+	+	+	+	+	+	+	+	+	+	+	+	+	+	+	+	+	+	+	+	+	+	+	–	–	–	–	+	+	+	+
1406	1578	Amino acid transporter	53	+	+	+	+	+	+	+	+	+	+	+	+	+	+	+	+	+	+	+	+	+	+	+	+	+	+	+	+	+	+	+	+	+	+	+	+	+	+	+	+	+	+	+	+	+	+	+	–	+	–	–	–	+	+	+
1407	1593	hypothetical protein	53	+	+	+	+	+	+	+	+	+	+	+	+	+	+	+	+	+	+	+	+	–	+	+	+	–	+	+	+	+	+	+	+	+	+	+	+	–	+	+	+	+	+	+	+	+	+	+	+	+	+	–	+	+	+	+	+	+
1408	932	hemerythrin HHE cation binding domain subfamily protein	52	+	+	+	+	+	+	+	+	+	+	+	+	+	+	+	+	+	+	+	+	+	+	+	+	+	+	+	+	+	+	+	+	+	+	+	–	+	+	+	+	+	+	+	+	+	+	+	+	–	+	–	–	–	+	+

Table S5. Precence/absence of each HG among 57 *S. suis* strains

Index[a]	ClusterID	Gene products	Members
1409	970	conserved domain protein	52
1410	975	Antiholin-like protein LrgA	52
1411	1112	Transcriptional regulator, ArsR family	52
1412	1257	Mannonate dehydratase (EC 4.2.1.8)	52
1413	1269	Transcriptional regulator, XRE family	52
1414	1299	Predicted hydrolase (HAD superfamily)	52
1415	1486	Lipopolysaccharide biosynthesis protein	52
1416	1535	Glycosyltransferase	52
1417	1539	Putative CDP-glycosylpolyol phosphate:glycosylpolyol glycosylpolyolphosphotransferase	52
1418	1542	ATP-binding membrane protein	52
1419	1543	Phage envelope protein	52
1420	1548	hypothetical protein	52
1421	1556	hypothetical protein	52
1422	1580	hypothetical protein	52
1423	1583	YxkD	52
1424	1591	ATPas	52
1425	1592	Predicted integral membrane protein	52
1426	1594	hypothetical protein	52
1427	2	6-phospho-beta-glucosidase (EC 3.2.1.86)	51
1428	1020	Transcriptional regulator, Cro/CI family	51
1429	1032	Substrate-specific component NiaX of predicted niacin ECF transporter	51
1430	1174	Uridine phosphorylase (EC 2.4.2.3)	51
1431	1205	Glycosyltransferases involved in cell wall biogenesis	51
1432	1219	Glucan-binding protein	51
1433	1423	Alpha-galactosidase (EC 3.2.1.22)	51
1434	1457	Pyridoxamine 5'-phosphate oxidase-related	51
1435	1475	Heteropolysaccharide repeat unit export protein	51
1436	1493	Dehydrogenase with different specificities (related to short-chain alcohol dehydrogenase)	51
1437	1494	N-Acetyl-D-glucosamine ABC transport system, sugar-binding protein	51
1438	1499	MSM (multiple sugar metabolism) operon regulatory protein	51
1439	1506	Transcriptional regulator, PadR family	51
1440	1524	ABC-type sugar transport system, permease component	51
1441	1540	Cobalt-zinc-cadmium resistance protein	51
1442	1550	Beta-hexosaminidase (EC 3.2.1.52)	51
1443	1557	Predicted metal-dependent membrane protease	51
1444	1559	hypothetical protein	51
1445	1563	hypothetical protein	51
1446	1570	Transcriptional repressor for NAD biosynthesis in gram-positives	51
1447	1571	hypothetical protein	51
1448	1577	hypothetical protein	51
1449	1579	Predicted integral membrane protein	51
1450	1581	hypothetical protein	51
1451	1582	hypothetical protein	51
1452	1596	Amino acid ABC transporter, permease protein	51
1453	1598	Lipoate-protein ligase A	51
1454	1602	amino acid ABC transporter (amino acid-binding protein)	51
1455	1604	hypothetical protein	51
1456	1605	membrane protein, related to Actinobacillus protein (1944168)	51
1457	1606	hypothetical protein	51
1458	832	GlpG protein (membrane protein of glp regulon)	50
1459	1065	PTS system, cellobiose-specific IIB component (EC 2.7.1.69)	50
1460	1070	PTS system, cellobiose-specific IIA component (EC 2.7.1.69)	50
1461	1120	Predicted membrane protein	50
1462	1346	hypothetical protein	50
1463	1504	Transcriptional antiterminator of lichenan operon, BglG family	50
1464	1505	PTS system, cellobiose-specific IIC component (EC 2.7.1.69)	50
1465	1507	putative protease	50
1466	1528	N-Acetyl-D-glucosamine ABC transport system, permease protein 1	50
1467	1552	hypothetical protein	50
1468	1576	6-phospho-beta-glucosidase (EC 3.2.1.86)	50
1469	1584	hypothetical protein	50
1470	1597	hypothetical protein	50
1471	1600	Putative uncharacterized protein SPs0857	50
1472	1611	YSIRK Gram-positive signal peptide	50
1473	1614	hypothetical protein	50
1474	1615	C-P lyase regulatory protein	50
1475	1619	hypothetical protein	50
1476	1620	Alpha-L-Rha alpha-1,2-L-rhamnosyltransferase/alpha-L-Rha alpha-1,3-L- rhamnosyltransferase (EC 2.4.1.-)	50
1477	7	putative transposase	49
1478	31	Sortase A, LPXTG specific	49
1479	53	Signal peptidase I (EC 3.4.21.89)	49
1480	611	IS66 family element, Orf1	49
1481	1014	acetyltransferase, GNAT family	49
1482	1156	hypothetical protein	49
1483	1452	SAM-dependent methyltransferase	49
1484	1490	LSU ribosomal protein L33p	49
1485	1498	Histone acetyltransferase HPA2 and related acetyltransferases	49
1486	1513	Protein involved in cell division	49
1487	1527	Outer surface protein of unknown function	49
1488	1590	hypothetical protein	49
1489	1601	Putative NADPH-quinone reductase (modulator of drug activity B)	49
1490	1603	Predicted membrane protein	49
1491	1622	NADH:flavin oxidoreductase, Old Yellow Enzyme family	49
1492	1629	Endothelin-converting enzyme 1(EC:3.4.24.71)	49
1493	1634	Zinc metalloprotease zmpC precursor (EC 3.4.24.-) (MMP-9 protease)	49
1494	1642	hypothetical protein	49
1495	1644	hypothetical protein	49
1496	802	Transposase	48
1497	1237	ATPase	48
1498	1612	hypothetical protein	48
1499	1617	hypothetical protein	48
1500	1627	hypothetical protein	48
1501	1630	ABC-type sugar transport system, permease component	48
1502	1632	hypothetical protein	48
1503	1640	Integrase	48
1504	1645	hypothetical protein	48
1505	1036	DNA alkylation repair enzyme	47
1506	1196	Mobile element protein	47
1507	1613	hypothetical protein	47
1508	1616	Fibronectin-binding protein	47
1509	1623	5'-nucleotidase (EC 3.1.3.5)	47
1510	1650	hypothetical protein	47
1511	903	SSU ribosomal protein S14p (S29e)	46
1512	1039	Dihydroneopterin aldolase (EC 4.1.2.25)	46
1513	1087	hypothetical protein	46
1514	1478	Putative integral membrane protein	46
1515	1508	Phosphotransferase system	46
1516	1514	Transketolase, C-terminal section (EC 2.2.1.1)	46
1517	1515	Transketolase, N-terminal section (EC 2.2.1.1)	46
1518	1538	internalin, putative	46
1519	1618	Hyaluronan synthase (EC 2.4.1.212)	46
1520	1636	hypothetical protein	46
1521	1649	integrase family protein	46
1522	1658	Predicted galactitol operon regulator (Transcriptional antiterminator), BglG family / PTS system, mannitol/fructose-specific IIA component (EC 2.7.1.69)	46
1523	1663	hypothetical protein	46
1524	1664	hypothetical protein	46
1525	1106	Amino acid ABC transporter, amino acid-binding/permease protein	45
1526	1628	Methyl-accepting chemotaxis protein	45
1527	1648	Type I restriction-modification system, DNA-methyltransferase subunit M (EC 2.1.1.72)	45
1528	1659	Predicted metal-dependent membrane protease	45
1529	1662	hypothetical protein	45
1530	1667	Acetyltransferase, including N-acetylases of ribosomal proteins	45
1531	1672	hypothetical protein	45
1532	1674	Restriction endonuclease S subunits	45
1533	1676	hypothetical protein	45
1534	4	Mobile element protein	44
1535	807	DNA-cytosine methyltransferase (EC 2.1.1.37)	44
1536	896	Voltage-gated chloride channel family protein	44
1537	1080	conserved domain protein	44
1538	1081	TrsE-like protein	44
1539	1082	Tn5252, Orf23	44
1540	1114	hypothetical protein	44
1541	1115	hypothetical protein	44
1542	1117	hypothetical protein	44
1543	1153	Mobile element protein	44
1544	1209	ATPases with chaperone activity, ATP-binding subunit	44
1545	1554	PTS system, beta-glucoside-specific IIB component (EC 2.7.1.69) / PTS system, beta-glucoside-specific IIC component (EC 2.7.1.69) / PTS system, beta-glucoside-specific IIA component (EC 2.7.1.69)	44

Table S5. Precence/absence of each HG among 57 *S. suis* strains

Strain columns (grouped by serotype): 1: 98HAH33, 05ZYH33, SC84, A7, SS12, P1/7, DAT273, NIAH11434, JS14, GZ1, BM407, S735, 13730, MNCM07; NCTC 10446, 14636; 13: NCTC 10237, ST1; 2651; 25: 89-1591, MNCM04, MNCM25, 8074, D9; 28: 4961, ST3, MNCM43, DAT292, DAT260, NIAH11435; 104: MNCM21, MNCM50; 94: 89-2479, DAT234, DAT290; 76: 93A, 42A; NT77; 53/54: 6407, 11538; Divergent *S. suis*: 8830, 12814, 92-1400, 89-590, 2726, 2524, 89-3576-3, 4417, D12, 10581, 89-5259, 88-5299A, R61, 92-1191, 14A, 92-4172, 22083

Index[a]	ClusterID	Gene products	Members
1546	1564	Beta-glucoside bgl operon antiterminator, BglG family	44
1547	1673	Type I restriction-modification system, restriction subunit R (EC 3.1.21.3)	44
1548	1704	hypothetical protein	44
1549	980	hypothetical protein	43
1550	1053	hypothetical protein	43
1551	1113	hypothetical protein	43
1552	1116	Zn-finger	43
1553	1125	hypothetical protein	43
1554	1126	hypothetical protein	43
1555	1138	Tn5252, Orf 9 protein	43
1556	1150	agglutinin receptor	43
1557	1152	Pyruvate formate-lyase (EC 2.3.1.54)	43
1558	1161	Transaldolase (EC 2.2.1.2)	43
1559	1167	Pyruvate formate-lyase activating enzyme (EC 1.97.1.4)	43
1560	1171	Transcriptional regulator, DeoR family	43
1561	1221	unknown	43
1562	1224	transcriptional regulator, putative	43
1563	1235	PTS system, cellobiose-specific IIB component (EC 2.7.1.69)	43
1564	1253	Glycerol dehydrogenase (EC 1.1.1.6)	43
1565	1262	PTS system, cellobiose-specific IIA component (EC 2.7.1.69)	43
1566	1267	PTS system, cellobiose-specific IIC component (EC 2.7.1.69)	43
1567	1284	calcium-binding protein, putative	43
1568	1635	Mobile element protein	43
1569	1681	endonuclease III domain protein	43
1570	1683	Glycerol dehydrogenase (EC 1.1.1.6)	43
1571	1702	hypothetical protein	43
1572	1703	immunity protein, probable	43
1573	929	hypothetical protein	42
1574	1054	hypothetical protein	42
1575	1137	unknown	42
1576	1242	Alpha-glycerophosphate oxidase (EC 1.1.3.21)	42
1577	1654	Inner membrane protein YqiK	42
1578	1677	Ribonucleases G and E	42
1579	1682	hypothetical protein	42
1580	1684	Glyoxalase family protein	42
1581	1697	Mobile element protein	42
1582	1716	hypothetical protein	42
1583	906	Transcription accessory protein (S1 RNA-binding domain)	41
1584	1018	unknown	41
1585	1193	Tn5252, Orf 10 protein	41
1586	1587	hypothetical protein	41
1587	1660	GCN5-related N-acetyltransferase	41
1588	1687	Phage shock protein C, putative; stress-responsive transcriptional regulator	41
1589	893	Cell division transporter, ATP-binding protein FtsE (TC 3.A.5.1.1)	40
1590	913	Putative tRNA-m1A22 methylase	40
1591	1210	Tn5252, relaxase	40
1592	1250	unknown	40
1593	1572	Mobile element protein	40
1594	1639	hypothetical protein	40
1595	1652	Cell wall surface anchor family protein	40
1596	1675	Sakacin A production response regulator	40
1597	1706	hypothetical protein	40
1598	1729	Nicotinamide-nucleotide adenylyltransferase, NadR family (EC 2.7.7.1) / Ribosylnicotinamide kinase (EC 2.7.1.22)	40
1599	1735	hypothetical protein	40
1600	1744	hypothetical protein	40
1601	901	Mobile element protein	39
1602	1155	Glycerol kinase (EC 2.7.1.30)	39
1603	1165	Mobile element protein	39
1604	1211	SNF2 family protein	39
1605	1270	hypothetical protein	39
1606	1526	2-deoxy-D-gluconate 3-dehydrogenase (EC 1.1.1.125)	39
1607	1551	PTS system, N-acetylgalactosamine-specific IIB component (EC 2.7.1.69)	39
1608	1553	PTS system, N-acetylgalactosamine-specific IID component (EC 2.7.1.69)	39
1609	1560	predicted 4-deoxy-L-threo-5-hexosulose-uronate ketol-isomerase (EC 5.3.1.17)	39
1610	1561	PTS system, N-acetylgalactosamine-specific IIC component (EC 2.7.1.69)	39
1611	1565	4-hydroxy-2-oxoglutarate aldolase (EC 4.1.3.16) @ 2-dehydro-3-deoxyphosphogluconate aldolase (EC 4.1.2.14)	39
1612	1568	Unsaturated glucuronyl hydrolase (EC 3.2.1.-)	39
1613	1569	Transcriptional regulator RegR, rpressor of hyaluronate and KDG utilization	39
1614	1641	Uncharacterized membrane protein, possible Na+ channel or pump	39
1615	1665	2-dehydro-3-deoxygluconate kinase (EC 2.7.1.45)	39
1616	1685	Preprotein translocase subunit YajC (TC 3.A.5.1.1)	39
1617	1699	acetyltransferase, GNAT family family	39
1618	1700	PTS system, N-acetylgalactosamine- and galactosamine-specific IIA component (EC 2.7.1.69)	39
1619	1705	hypothetical protein	39
1620	1715	Predicted metal-dependent membrane protease	39
1621	1725	Trans-acting positive regulator	39
1622	1728	NADH peroxidase (EC 1.11.1.1)	39
1623	1731	Hyaluronate lyase precursor (EC 4.2.2.1)	39
1624	1732	Heparinase II/III-like	39
1625	1741	hypothetical protein	39
1626	1743	Translation initiation factor 2 (IF-2; GTPase)	39
1627	1764	hypothetical protein	39
1628	1100	Plasmid addiction system poison protein	38
1629	1305	CoA-disulfide reductase (EC 1.8.1.14) / Disulfide bond regulator	38
1630	1368	Protein of unknown function DUF156	38
1631	1434	transcription regulator, probable	38
1632	1459	Zeta toxin	38
1633	1608	Transcriptional regulator, AraC family	38
1634	1661	Rhodanese-related sulfurtransferase	38
1635	1749	hypothetical protein	38
1636	1765	hypothetical protein	38
1637	1774	hypothetical protein	38
1638	1537	Multiple sugar ABC transporter, membrane-spanning permease protein MsmG	37
1639	1544	Multiple sugar ABC transporter, membrane-spanning permease protein MsmF	37
1640	1545	hypothetical protein	37
1641	1546	Multiple sugar ABC transporter, substrate-binding protein	37
1642	1562	ABC transporter permease protein	37
1643	1651	Alpha-1,2-mannosidase	37
1644	1653	Alpha-mannosidase (EC 3.2.1.24)	37
1645	1655	Glycosyl hydrolase-related protein	37
1646	1657	N-acetylmannosamine kinase (EC 2.7.1.60)	37
1647	1739	Leucine rich protein	37
1648	1775	Two-component sensor kinase YesM (EC 2.7.3.-)	37
1649	1777	hypothetical protein	37
1650	1781	Type I restriction-modification system, specificity subunit S (EC 3.1.21.3)	37
1651	1784	DNA-binding response regulator, AraC family	37
1652	1787	hypothetical protein	37
1653	1799	Putative uncharacterized protein SPs0573	37
1654	1041	Alpha-L-Rha alpha-1,3-L-rhamnosyltransferase (EC 2.4.1.-)	36
1655	1588	Tetracycline resistance protein TetM	36
1656	1666	hypothetical protein	36
1657	1678	Endo-beta-N-acetylglucosaminidase (EC 3.2.1.96)	36
1658	1698	Chromosome segregation helicase	36
1659	1727	Multidrug resistance ABC transporter ATP-binding and permease protein	36
1660	1738	tagatose-6-phosphate kinase	36
1661	1773	hypothetical protein	36
1662	1780	hypothetical protein	36
1663	1782	Glucuronide permease	36
1664	1802	hypothetical protein	36
1665	1803	Xylose repressor protein	36
1666	1804	PTS system, cellobiose-specific IIC component (EC 2.7.1.69)	36
1667	1816	ABC-type nitrate/sulfonate/bicarbonate transport system, periplasmic component	36
1668	1817	putative ABC-transport protein, membrane component	36
1669	1820	Glucosamine-6-phosphate deaminase (EC 3.5.99.6)	36
1670	1821	hypothetical protein	36
1671	1822	ABC-type nitrate/sulfonate/bicarbonate transport system, ATPase component	36
1672	1823	N-acetylmuramoyl-L-alanine amidase (EC 3.5.1.28)	36
1673	27	Mobile element protein	35
1674	28	Mobile element protein	35
1675	1656	Transposase and inactivated derivative	35
1676	1761	Transposase	35
1677	1806	hypothetical protein	35
1678	1819	hypothetical protein	35
1679	1840	Cu+ P-type ATPase fragment	35
1680	1842	Parallel beta-helix repeat	35
1681	1610	Arginine permease RocE	34
1682	1709	RRNA (Adenine-N(6)-) -methyltransferase (EC 2.1.1.48)	34
1683	1745	hypothetical protein	34

Table S5. Precence/absence of each HG among 57 *S. suis* strains

				1																13			25					28						104		94			76			53/54		Divergent *S. suis*																
Index[a]	ClusterID	Gene products	Members	98HAH33	05ZYH33	SC84	A7	SS12	P1/7	DAT273	NIAH11434	JS14	GZ1	BM407	S735	13730	MNCM07	NCTC 10446	14636	NCTC 10237	ST1	2651	89-1591	MNCM04	MNCM25	8074	D9	4961	ST3	MNCM43	DAT292	DAT260	NIAH11435	MNCM21	MNCM50	89-2479	DAT234	DAT290	93A	42A	NT77	6407	11538	8830	12814	92-1400	89-590	2726	2524	89-3576-3	4417	D12	10581	89-5259	88-5299A	R61	92-1191	14A	92-4172	22083
1684	1760	Cell division protein FtsQ	34	+	+	–	+	–	–	+	–	–	–	–	–	–	–	+	+	+	+	+	+	+	+	+	+	+	+	+	+	+	+	–	–	+	+	+	+	+	–	–	–	+	–	+	+	–	–	+	+	+	–	+	–	–	–	+	–	+
1685	1766	Phage integrase	34	–	–	–	–	–	–	–	–	–	–	+	–	–	+	–	+	+	+	+	+	+	+	+	–	+	+	+	+	+	+	+	+	+	+	+	+	+	+	+	+	+	+	+	+	–	–	+	+	–	+	–	+	–	–	–	–	–
1686	1633	ABC transporter, ATP-binding protein	33	–	–	–	–	–	–	–	–	–	–	–	–	–	–	–	+	+	+	+	+	+	+	+	+	+	+	+	+	+	+	+	+	+	+	+	+	+	+	–	–	+	+	+	–	+	+	–	+	–	–	+	–	+	–	–	+	+
1687	1686	Integrase	33	+	+	+	+	+	+	+	+	+	+	+	–	–	–	+	+	–	–	+	–	–	–	+	+	+	+	+	+	+	+	+	+	+	+	+	+	+	+	–	–	+	+	–	–	–	–	–	–	–	–	–	–	+	–	–	–	–
1688	1740	Transposase and inactivated derivative, IS5 family	33	+	+	+	+	+	+	+	+	+	+	+	+	+	+	–	–	+	+	+	+	–	+	+	+	–	–	+	+	+	+	+	+	–	+	+	–	–	–	–	–	–	–	–	–	–	–	–	–	–	+	+	+	+	–	–	–	–
1689	1778	cell filamentation protein Fic-related protein	33	+	+	+	+	+	+	+	+	+	+	+	+	+	+	+	+	+	+	–	–	–	–	–	–	–	–	–	–	–	–	–	–	+	+	+	+	+	+	+	+	+	+	+	+	–	–	–	–	–	–	–	–	–	+	–	+	+
1690	1839	hypothetical protein	33	+	+	+	–	+	+	–	–	+	+	+	–	–	–	+	–	+	–	+	+	+	+	+	–	–	–	–	–	+	–	+	+	+	–	–	–	–	–	+	+	+	+	+	+	+	–	+	+	+	–	+	+	–	–	–	+	+
1691	1841	Muramidase-released protein precursor (136 kDa surface protein)	33	+	+	+	+	+	+	–	+	+	+	+	+	+	+	–	+	–	–	+	+	–	–	+	+	+	+	+	+	+	+	–	–	+	+	+	+	+	+	+	+	–	–	–	+	–	–	–	–	–	–	–	–	–	–	–	–	–
1692	1844	hypothetical protein	33	+	+	+	+	+	+	+	+	+	+	+	+	+	+	+	+	–	–	+	–	–	–	–	–	–	–	–	–	–	–	–	–	+	+	+	+	+	+	–	–	+	+	+	+	+	–	–	+	–	–	–	–	+	+	–	+	+
1693	1854	similar to Uncharacterized conserved protein	33	+	+	+	+	+	+	+	+	+	+	+	+	+	+	+	+	+	+	–	+	+	+	+	+	–	–	+	+	+	+	+	+	–	–	–	–	–	–	+	+	+	–	–	+	–	–	–	–	–	–	–	–	–	–	–	–	–
1694	1862	hypothetical protein	33	–	–	–	–	+	–	–	–	–	–	–	–	–	–	–	+	+	+	–	+	+	+	+	+	+	+	+	+	+	+	+	+	+	+	+	+	+	+	–	–	+	+	+	–	+	+	+	+	+	–	–	–	–	–	–	–	–
1695	1863	Putative membrane spanning protein	33	–	–	–	–	–	–	–	–	–	–	–	–	–	–	–	+	+	+	+	+	+	+	+	+	+	+	+	+	+	+	+	+	+	+	+	+	+	+	–	–	+	+	+	–	+	+	–	+	–	–	+	–	+	–	–	+	+
1696	1867	copper-translocating P-type ATPase(EC:3.6.3.4)	33	+	+	+	+	+	+	+	+	+	+	+	+	+	+	–	+	–	–	–	+	+	+	+	+	+	+	+	–	+	+	+	–	+	+	+	+	+	–	+	+	–	–	–	–	–	–	–	–	–	–	–	–	–	–	–	–	–
1697	1643	Galactosyl transferase CpsE (EC 2.7.8.-)	32	+	+	+	+	+	+	+	+	+	+	+	+	+	+	+	+	+	+	+	+	+	+	–	–	–	–	+	+	+	+	+	+	–	–	–	–	–	–	–	–	–	–	–	–	+	+	+	–	–	–	+	–	–	–	–	–	–
1698	1769	Type III restriction enzyme, res subunit:DEAD/DEAH box helicase, N-terminal	32	+	+	+	+	+	+	+	+	+	+	+	+	+	+	+	+	–	–	+	–	+	+	–	–	–	–	–	–	–	–	–	–	+	+	+	+	+	+	–	–	–	–	+	–	–	–	–	–	–	–	+	+	+	–	+	+	+
1699	1770	Type III restriction-modification system methylation subunit (EC 2.1.1.72)	32	+	+	+	+	+	+	+	+	+	+	+	+	+	+	+	+	–	–	+	–	+	+	–	–	–	–	–	–	–	–	–	–	+	+	+	+	+	+	–	–	–	–	+	–	–	–	–	–	–	–	+	+	+	–	+	+	+
1700	1785	RelB/StbD replicon stabilization protein (antitoxin to RelE/StbE)	32	+	+	+	+	+	+	+	+	+	+	+	+	+	+	+	+	–	–	–	+	+	+	+	+	+	+	+	+	+	+	–	–	+	–	–	–	–	–	+	+	+	–	–	–	–	–	–	–	–	–	–	–	–	–	–	–	+
1701	1789	hypothetical protein	32	+	+	+	+	+	+	+	+	+	+	+	+	+	+	+	+	–	–	–	+	–	+	–	+	–	–	+	–	–	–	+	+	–	–	–	–	–	+	+	+	–	–	–	–	+	–	–	–	+	+	+	–	+	+	–	+	–
1702	1800	NADPH-flavin oxidoreductase, putative(EC:1.-.-,EC:1.6.99.-)	32	–	–	–	–	–	–	–	–	–	+	–	–	–	–	–	+	–	–	+	+	+	+	+	+	+	+	+	–	–	+	–	–	–	–	–	+	+	+	+	+	+	+	+	+	+	+	+	+	–	+	+	–	+	+	+	+	+
1703	1805	hypothetical protein	32	+	+	+	+	+	+	+	+	+	+	+	–	–	–	–	+	–	–	+	–	–	–	+	+	+	+	+	+	+	+	+	+	+	+	+	+	+	+	+	–	+	+	–	–	–	–	–	–	–	–	–	–	–	–	–	–	–
1704	1813	Transcriptional regulator, XRE family	32	+	+	+	+	+	+	+	+	+	+	+	+	+	+	–	–	+	+	–	–	–	–	–	–	–	–	–	–	–	–	–	–	–	–	–	+	+	+	+	+	–	–	+	+	–	+	+	–	–	+	+	+	+	+	+	–	+
1705	1814	hypothetical protein	32	–	–	–	–	–	–	–	–	–	–	–	–	–	+	+	–	+	+	+	+	+	+	+	+	+	+	+	+	+	+	+	+	+	+	+	–	–	–	+	+	+	–	–	+	+	+	–	+	+	+	–	–	–	–	–	+	+
1706	1815	hypothetical protein	32	+	+	+	+	+	+	+	+	+	+	+	–	–	–	–	+	–	–	+	–	–	–	+	+	+	+	+	+	+	+	+	+	+	+	+	+	+	+	+	–	+	+	–	–	–	–	–	–	–	–	–	–	–	–	–	–	–
1707	1855	hypothetical protein	32	+	+	+	+	+	+	+	+	+	+	+	+	+	+	+	–	–	–	–	+	–	–	+	+	+	+	+	–	+	+	+	+	+	+	+	–	–	–	+	+	–	–	–	+	–	–	–	–	–	–	–	–	–	–	–	–	+
1708	1007	Mobile element protein	31	+	+	+	+	+	+	+	+	+	+	+	+	+	+	+	–	–	+	–	+	+	+	–	+	–	–	+	+	–	–	+	+	+	–	–	+	+	–	+	+	–	–	–	–	–	–	–	–	–	–	–	–	+	–	–	+	–
1709	1178	PTS system, sucrose-specific IIB component (EC 2.7.1.69) / PTS system, sucrose-specific IIC component (EC 2.7.1.69) / PTS system, sucrose-specific IIA component (EC 2.7.1.69)	31	+	+	+	+	+	+	+	+	+	+	+	+	+	+	+	–	+	+	–	–	–	–	–	–	–	–	–	–	–	–	–	–	–	–	–	–	–	–	+	+	+	+	–	+	+	+	–	–	–	+	+	+	+	+	–	+	+
1710	1625	Homocysteine S-methyltransferase (EC 2.1.1.10)	31	+	+	+	+	+	+	+	+	+	+	+	+	+	+	+	–	–	–	–	–	–	–	–	–	–	–	–	–	–	–	–	–	+	+	+	+	+	+	–	–	–	–	–	+	+	–	+	+	+	+	+	+	+	–	+	–	–
1711	1717	MutT/NudX family protein (putative)	31	+	+	+	+	+	+	+	+	+	+	+	+	+	+	+	+	+	+	+	–	–	–	–	–	–	–	–	–	–	–	–	–	–	–	–	–	–	–	+	+	+	–	+	–	+	+	–	–	+	+	–	+	+	+	+	–	–
1712	1790	ABC-type multidrug transport system, ATPase component	31	+	+	+	+	+	+	+	+	+	+	+	+	+	+	–	+	–	–	–	+	+	+	–	–	–	–	–	–	+	–	–	–	+	–	+	+	+	+	–	–	–	–	–	+	–	–	–	–	–	+	+	+	+	–	+	+	–
1713	1826	putative bacteriocin operon protein	31	+	+	+	+	+	+	+	+	+	+	+	+	+	+	–	+	–	–	–	+	+	+	–	–	–	–	–	–	+	–	–	–	+	–	+	+	+	+	–	–	–	–	–	+	–	–	–	–	–	+	+	+	+	–	+	+	–
1714	1832	Beta-glucosidase (EC 3.2.1.21)	31	+	+	+	+	+	+	+	+	+	+	+	+	+	+	+	+	+	+	+	–	–	–	–	–	–	–	–	–	–	–	–	–	+	+	+	+	+	–	–	–	+	+	+	–	–	–	–	–	–	+	+	+	–	–	+	–	–
1715	1833	hypothetical protein	31	+	+	+	+	+	+	+	+	+	+	+	+	+	+	+	+	+	+	+	–	–	–	–	–	–	–	–	–	–	–	–	–	+	+	+	+	+	–	–	–	+	+	+	–	–	–	–	–	–	+	+	+	–	–	+	–	–
1716	1834	hypothetical protein	31	+	+	+	+	+	+	+	+	+	+	+	+	+	+	+	+	+	+	+	–	–	–	–	–	–	–	–	–	–	–	–	–	+	+	+	+	+	–	–	–	+	+	+	–	–	–	–	–	–	+	+	+	–	–	+	–	–
1717	1836	hypothetical protein	31	+	+	+	+	+	+	+	+	+	+	+	+	+	+	+	+	+	+	+	–	–	–	–	–	–	–	–	–	–	–	–	–	+	+	+	+	+	–	–	–	+	+	+	–	–	–	–	–	–	+	+	+	–	–	+	–	–
1718	1851	hypothetical protein	31	–	–	–	+	–	–	+	–	–	+	–	–	+	–	+	–	+	+	+	–	–	–	–	–	–	–	–	+	+	–	–	–	+	+	+	+	+	+	+	–	+	+	+	+	+	+	+	+	+	+	+	–	+	+	–	–	+
1719	1864	Beta-glucosidase (EC 3.2.1.21)	31	+	+	+	+	+	+	+	+	+	+	+	+	+	+	+	+	+	+	+	–	–	–	–	–	–	–	–	–	–	–	–	–	+	+	+	+	+	–	–	–	+	+	+	–	–	–	–	–	–	+	+	+	–	–	+	–	–
1720	1872	Rrf2 family transcriptional regulator, group III	31	+	+	+	+	+	+	+	+	+	+	+	+	+	+	+	–	–	–	–	–	–	–	–	–	–	–	–	–	–	–	–	–	+	+	+	+	+	+	–	–	+	+	+	+	+	–	+	+	+	–	+	–	+	–	–	–	–
1721	1882	hypothetical protein	31	+	+	+	+	+	+	+	+	+	+	+	+	+	+	+	+	+	+	+	–	–	–	–	–	+	+	+	+	+	+	+	+	–	–	–	–	–	–	+	+	+	–	+	–	–	–	–	–	–	–	–	–	–	–	–	–	–
1722	1896	hypothetical protein	31	–	–	–	–	–	–	–	–	–	–	–	–	–	–	+	+	+	+	+	+	+	+	+	+	+	+	+	+	+	+	–	–	+	+	+	–	+	–	+	+	+	+	+	+	+	+	+	+	–	–	–	–	–	–	–	+	–
1723	1897	hypothetical protein	31	–	–	–	–	–	–	–	–	–	–	–	–	–	+	+	–	+	+	–	+	+	+	+	+	+	+	+	+	+	+	+	+	–	+	+	–	–	–	–	–	–	+	+	–	+	+	+	+	+	–	–	–	+	+	+	+	+
1724	1901	hypothetical protein	31	+	+	+	+	+	+	+	+	+	+	+	–	+	–	+	–	–	–	–	+	+	+	+	+	+	+	–	–	+	+	–	–	–	–	–	–	–	–	+	+	–	–	–	+	–	+	+	–	+	–	+	–	–	+	–	+	–
1725	1367	hypothetical protein	30	–	–	–	–	–	–	–	–	–	–	–	–	–	–	+	–	–	–	+	+	+	+	–	+	+	–	+	+	+	+	+	+	+	+	+	+	–	+	+	+	+	+	+	+	+	–	–	–	+	–	–	+	+	+	+	–	–
1726	1631	Conserved domain protein	30	+	+	+	+	+	+	+	+	+	+	+	+	+	+	+	+	+	+	+	–	–	–	–	–	–	–	–	–	–	–	–	–	–	–	–	–	–	+	–	–	–	–	+	+	–	–	–	+	–	+	+	+	+	+	+	+	–
1727	1829	Chromosome (plasmid) partitioning protein ParB / Stage 0 sporulation protein J	30	–	–	–	–	+	–	+	+	+	+	–	–	–	+	–	–	–	–	–	+	+	+	–	+	–	–	+	+	–	+	+	+	+	+	+	+	+	+	–	–	–	–	+	+	–	–	+	+	+	–	+	+	–	+	+	–	–
1728	1838	Thiol-activated cytolysin # pneumolysin	30	+	+	+	+	+	+	+	+	+	+	+	+	+	+	+	+	+	+	+	–	–	–	–	–	–	–	–	–	–	–	+	+	+	+	+	+	+	+	+	+	–	–	–	+	–	–	–	–	–	–	–	–	–	–	–	–	–
1729	1865	Conserved domain protein SP0160	30	+	+	+	+	+	+	+	+	+	+	+	+	+	+	+	+	+	+	+	–	–	–	–	–	–	–	–	–	–	–	–	–	–	–	–	–	–	+	–	–	–	–	+	+	–	–	–	+	–	+	+	+	+	+	+	+	–
1730	1866	N-acetylneuraminate synthase (EC 2.5.1.56)	30	+	+	+	+	+	+	+	+	+	+	+	+	+	+	–	–	+	+	+	+	+	+	–	–	–	–	+	+	+	+	+	+	–	–	–	–	–	–	–	–	–	–	–	–	+	+	–	–	–	+	+	–	–	–	–	–	–
1731	1900	hypothetical protein	30	+	+	+	+	+	+	+	+	+	+	+	+	+	+	+	+	–	–	–	+	+	+	+	+	+	+	+	+	–	+	–	–	+	–	+	+	–	–	–	–	–	–	–	+	–	–	–	–	–	–	–	–	–	–	–	–	–
1732	1923	hypothetical protein	30	+	+	+	–	+	+	+	+	+	+	+	+	+	+	–	–	–	+	–	–	+	+	–	+	+	+	+	–	–	+	+	+	+	–	–	+	–	+	+	–	+	–	–	–	–	–	–	+	–	–	–	–	–	–	–	+	–
1733	1927	hypothetical protein	30	–	–	–	–	–	–	–	–	–	–	–	–	–	–	–	+	+	+	+	+	+	+	+	+	+	+	+	+	+	+	+	+	+	+	+	+	+	+	–	–	+	+	+	–	+	–	–	–	–	–	–	–	+	–	–	+	+
1734	910	Mobile element protein	29	+	+	+	+	+	+	+	+	+	+	+	+	–	+	+	–	+	+	–	+	–	–	–	+	–	+	+	+	–	–	+	+	–	–	–	+	+	+	+	+	–	–	–	–	–	–	–	–	–	–	–	–	–	–	–	+	–
1735	1734	hypothetical protein	29	+	+	+	+	+	–	+	+	+	–	+	–	+	+	+	–	+	+	+	–	+	–	–	+	–	–	+	–	–	–	–	–	+	–	–	+	+	+	+	–	–	–	–	–	+	–	–	+	–	–	–	+	+	–	+	+	–
1736	1736	hypothetical protein	29	+	+	+	–	+	+	+	+	+	+	+	–	–	–	+	+	–	–	+	–	–	–	+	+	+	+	+	+	+	+	–	–	+	+	+	+	–	+	–	–	+	+	–	–	–	–	–	–	–	–	–	–	+	–	–	–	–
1737	1846	hypothetical protein	29	–	–	–	–	+	–	+	+	+	–	–	–	–	+	–	–	–	–	–	+	+	+	–	+	–	–	+	+	–	+	+	+	+	+	+	+	+	+	–	–	–	–	+	+	–	–	+	+	+	–	+	+	–	+	+	–	–
1738	1847	bacterial seryl-tRNA synthetase related	29	–	–	–	–	+	–	+	+	+	–	–	–	–	+	–	–	–	–	–	+	+	+	–	+	–	–	+	+	–	+	+	+	+	+	+	+	+	+	–	–	–	–	+	+	–	–	+	+	+	–	+	+	–	+	+	–	–
1739	1852	FIG048485: Phage protein	29	–	–	–	–	+	–	–	–	–	–	–	–	–	–	+	+	+	+	–	+	+	+	+	–	+	+	+	+	+	+	+	+	–	+	+	–	–	–	+	–	+	–	–	+	–	–	+	–	–	+	+	+	–	+	+	+	–
1740	1857	hypothetical protein	29	–	–	–	–	+	–	+	+	+	–	–	–	–	+	–	–	–	–	–	+	+	+	–	+	–	–	+	+	–	+	+	+	+	+	+	+	+	+	–	–	–	–	+	+	–	–	+	+	+	–	+	+	–	+	+	–	–
1741	1868	hypothetical protein	29	–	–	–	–	–	–	–	–	–	–	–	–	–	–	+	+	–	–	+	+	+	+	–	+	+	+	+	+	+	+	+	+	–	–	–	–	+	+	+	+	+	+	–	+	+	+	+	–	–	+	+	–	+	–	+	–	–
1742	1873	hypothetical protein	29	–	–	–	–	+	–	+	+	+	–	–	–	–	+	–	–	–	–	–	+	+	+	–	+	–	–	+	+	–	+	+	+	+	+	+	+	+	+	–	–	–	–	+	+	–	–	+	+	+	–	+	+	–	+	+	–	–
1743	1878	UDP-N-acetylglucosamine 2-epimerase (EC 5.1.3.14)	29	+	+	+	+	+	+	+	+	+	+	+	+	+	+	–	–	+	+	+	+	+	+	–	–	–	–	+	+	+	+	+	+	–	–	–	–	–	–	–	–	–	–	–	–	+	+	–	–	–	–	+	–	–	–	–	–	–
1744	1881	Sialic acid biosynthesis protein NeuD, O-acetyltransferase	29	+	+	+	+	+	+	+	+	+	+	+	+	+	+	–	–	+	+	+	+	+	+	–	–	–	–	+	+	+	+	+	+	–	–	–	–	–	–	–	–	–	–	–	–	+	+	–	–	–	–	+	–	–	–	–	–	–
1745	1884	N-Acetylneuraminate cytidylyltransferase (EC 2.7.7.43)	29	+	+	+	+	+	+	+	+	+	+	+	+	+	+	–	–	+	+	+	+	+	+	–	–	–	–	+	+	+	+	+	+	–	–	–	–	–	–	–	–	–	–	–	–	+	+	–	–	–	–	+	–	–	–	–	–	–
1746	1918	Capsular polysaccharide biosynthesis protein CpsK(V)	29	+	+	+	+	+	+	+	+	+	+	+	+	+	+	–	–	+	+	+	+	+	+	–	–	–	–	+	+	+	+	+	+	–	–	–	–	–	–	–	–	–	–	–	–	+	+	–	–	–	–	+	–	–	–	–	–	–
1747	1919	Glycosyltransferases involved in cell wall biogenesis	29	+	+	+	+	+	+	+	+	+	+	+	+	+	+	–	–	+	+	+	+	+	+	–	–	–	–	+	+	+	+	+	+	–	–	–	–	–	–	–	–	–	–	–	–	+	+	–	–	–	–	+	–	–	–	–	–	–
1748	1921	Capsular polysaccharide repeat unit transporter CpsL	29	+	+	+	+	+	+	+	+	+	+	+	+	+	+	–	–	+	+	+	+	+	+	–	–	–	–	+	+	+	+	+	+	–	–	–	–	–	–	–	–	–	–	–	–	+	+	–	–	–	–	+	–	–	–	–	–	–
1749	1930	hypothetical protein	29	+	+	+	+	+	+	+	+	+	+	+	+	+	+	–	+	–	–	–	+	+	+	–	–	–	–	–	–	+	–	–	–	+	–	+	+	+	+	–	–	–	–	–	+	–	–	–	–	–	–	+	–	+	–	+	+	–
1750	1949	hypothetical protein	29	–	–	–	–	–	–	–	–	–	–	–	–	–	–	–	+	+	+	+	–	–	–	–	–	–	–	–	–	–	–	+	+	+	+	+	+	+	+	+	+	–	+	+	+	+	+	+	+	+	+	+	+	+	+	+	–	+
1751	1135	Cysteine ABC transporter, permease protein	28	–	–	–	–	–	–	–	–	–	–	–	–	–	–	–	–	–	–	–	+	+	+	+	+	+	+	+	+	+	+	+	+	+	+	+	+	+	–	–	–	+	+	+	–	+	+	+	+	+	–	–	–	–	–	–	+	+
1752	1647	Integrase/recombinase, phage associated	28	–	–	–	–	–	–	–	–	–	–	–	–	–	–	–	–	–	–	–	+	+	+	+	+	+	+	+	+	+	+	+	+	+	–	+	+	+	+	+	+	+	+	+	–	+	+	–	–	+	–	–	+	+	–	–	–	–
1753	1669	hypothetical protein	28	+	+	+	–	+	–	+	–	–	–	+	–	–	+	–	+	–	–	–	+	+	+	–	+	–	–	–	–	+	+	–	–	+	+	–	+	+	+	–	–	–	+	+	+	–	–	–	+	–	+	–	–	+	–	+	+	+
1754	1670	Integrase	28	+	+	+	–	+	–	+	–	–	–	+	–	–	+	–	+	–	–	–	+	+	+	–	+	–	–	–	–	+	+	–	–	+	+	–	+	+	+	–	–	–	+	+	+	–	–	–	+	–	+	–	–	+	–	+	+	+
1755	1810	abortive infection protein AbiGI	28	+	+	+	–	–	–	–	+	+	–	+	–	–	–	–	–	–	–	–	+	+	+	–	–	–	–	+	+	+	+	+	+	+	–	+	–	–	+	–	–	–	+	+	+	+	–	–	+	+	+	+	+	–	–	–	+	–
1756	1837	DNA/RNA HELICASE (DEAD/DEAH BOX FAMILY)	28	+	+	+	+	+	+	+	+	+	+	+	+	+	+	+	–	+	+	–	–	–	–	–	–	–	–	–	–	–	–	–	–	+	+	+	+	+	+	+	+	–	–	+	–	–	–	–	–	–	–	–	–	–	+	–	–	+
1757	1887	hypothetical protein	28	–	–	–	–	+	–	+	+	+	–	–	–	–	+	–	–	–	–	–	+	+	+	–	+	–	–	+	+	–	+	+	+	+	+	+	+	+	+	–	–	–	–	+	+	–	–	+	+	+	–	–	+	–	+	+	–	–
1758	1924	putative esterase	28	–	–	–	–	–	–	–	–	–	–	–	–	–	–	–	+	–	–	+	+	+	+	+	+	+	+	+	+	+	+	+	+	–	–	–	+	+	+	+	+	+	+	–	–	–	+	–	–	–	+	+	–	+	+	+	–	–
1759	1950	Protein of unknown function DUF11:Surface protein from Gram-positive cocci, anchor region	28	+	+	+	+	+	+	+	+	+	+	+	+	–	–	–	–	+	+	–	+	–	+	–	+	–	+	–	–	–	+	+	–	–	+	+	–	+	–	+	+	–	+	–	–	–	–	–	–	+	–	–	–	–	–	+	–	–
1760	1955	hypothetical protein	28	–	–	–	–	–	–	–	–	–	–	–	–	–	–	–	–	–	–	–	+	+	+	+	+	+	+	+	+	+	+	+	+	+	+	+	+	+	+	–	–	+	+	–	–	+	+	+	+	+	–	–	–	–	–	+	+	–
1761	1958	hypothetical protein	28	+	+	+	+	+	+	+	+	+	–	+	+	+	+	+	+	–	–	–	–	+	+	–	+	–	+	+	+	+	+	–	–	+	–	–	–	–	–	–	+	–	–	–	–	–	–	–	+	–	–	–	–	–	–	–	+	+
1762	1977	hypothetical protein	28	–	–	–	–	–	+	–	–	+	+	+	+	–	+	+	+	+	+	+	–	+	–	+	–	+	–	+	+	–	–	–	–	+	–	+	+	–	+	+	–	–	–	+	–	+	+	+	–	+	+	–	–	–	–	–	+	–
1763	1986	hypothetical protein	28	+	+	+	+	+	+	+	+	+	+	+	+	+	+	–	–	–	–	–	–	–	–	–	–	–	–	–	–	–	–	–	–	+	+	+	+	+	+	–	–	–	–	–	–	–	–	–	+	–	+	+	+	+	+	+	–	+
1764	1638	ABC transporter ATP-binding protein	27	–	–	–	–	–	–	–	–	–	–	–	–	–	–	–	–	–	–	+	+	+	+	+	+	+	+	+	+	+	+	+	+	–	–	–	–	–	–	–	–	–	–	–	–	+	+	+	+	+	+	+	+	+	+	+	+	+
1765	1689	prophage ps3 protein 13	27	–	–	–	–	–	–	–	–	–	–	–	–	–	–	+	–	–	–	+	+	+	+	+	+	+	+	+	+	+	+	+	+	+	+	+	+	+	+	–	–	+	+	+	–	–	–	–	–	+	–	–	+	–	+	–	–	–
1766	1707	Macrophage infectivity potentiator protein	27	–	–	–	–	–	–	–	–	–	–	–	–	–	–	–	–	–	–	–	+	+	+	+	+	+	+	+	+	+	+	–	–	–	–	–	–	–	–	–	–	+	+	+	–	+	+	+	+	+	+	+	+	+	+	+	+	+
1767	1708	ABC transporter permease protein	27	–	–	–	–	–	–	–	–	–	–	–	–	–	–	–	–	–	–	+	+	+	+	+	+	+	+	+	+	+	+	+	+	–	–	–	–	–	–	–	–	–	–	–	–	+	+	+	+	+	+	+	+	+	+	+	+	+
1768	1750	Thiamin-phosphate pyrophosphorylase (EC 2.5.1.3)	27	+	+	+	+	+	+	+	+	+	+	+	+	+	+	+	–	–	–	–	–	+	+	–	–	–	–	–	–	–	–	–	–	+	+	+	+	+	+	+	+	–	–	–	+	–	–	–	–	–	–	–	–	–	–	–	+	–
1769	1752	Hydroxymethylpyrimidine phosphate kinase ThiD (EC 2.7.4.7) / Thiamin-phosphate pyrophosphorylase (EC 2.5.1.3) / Hydroxyethylthiazole kinase (EC 2.7.1.49)	27	+	+	+	+	+	+	+	+	+	+	+	+	+	+	+	–	–	–	–	–	+	+	–	–	–	–	–	–	–	–	–	–	+	+	+	+	+	+	+	+	–	–	–	+	–	–	–	–	–	–	–	–	–	–	–	+	–
1770	1768	Hydroxyethylthiazole kinase (EC 2.7.1.50)	27	+	+	+	+	+	+	+	+	+	+	+	+	+	+	+	–	–	–	–	–	+	+	–	–	–	–	–	–	–	–	–	–	+	+	+	+	+	+	+	+	–	–	–	+	–	–	–	–	–	–	–	–	–	–	–	+	–
1771	1828	Abortive infection protein AbiGII	27	+	+	+	–	–	–	–	+	+	–	+	–	–	–	–	–	–	–	–	+	+	+	–	–	–	–	+	+	+	+	+	+	+	–	+	–	–	+	–	–	–	+	+	+	+	–	–	+	+	+	+	–	–	–	–	+	–
1772	1871	Site-specific recombinase	27	–	–	–	–	+	–	+	+	+	–	–	–	–	+	–	–	–	–	–	+	+	+	–	+	–	–	+	+	–	+	+	+	+	+	+	+	+	+	–	–	–	–	+	+	–	–	+	+	–	–	–	+	–	+	+	–	–
1773	1880	Transcriptional regulator	27	+	+	+	+	+	+	+	+	+	+	+	+	+	+	+	–	+	+	–	–	–	–	–	–	–	–	–	–	–	–	–	–	+	+	+	+	+	+	+	+	–	–	+	–	–	–	–	–	–	–	–	–	–	–	–	–	+
1774	1885	hypothetical protein	27	–	–	–	–	–	–	–	–	–	–	–	–	–	–	–	–	–	–	+	+	+	–	+	+	+	+	+	+	+	+	+	+	+	+	+	–	+	+	+	+	+	–	+	+	+	–	–	–	+	–	–	–	+	–	+	–	–
1775	1915	hypothetical protein	27	–	–	–	–	+	–	–	–	+	–	–	–	–	–	–	–	+	–	–	+	+	+	+	–	–	–	+	+	+	+	+	+	+	+	–	+	+	–	+	–	–	–	–	–	+	–	–	+	+	+	+	–	+	+	–	+	+
1776	1917	hypothetical protein	27	–	–	–	–	–	–	–	–	–	–	–	–	–	–	+	+	+	+	–	+	+	+	+	+	+	+	+	+	+	+	–	–	–	–	–	+	+	–	+	+	+	+	–	+	+	–	–	+	–	–	–	–	–	+	–	+	+
1777	1926	hypothetical protein	27	–	–	–	–	–	–	–	–	–	–	–	–	–	–	+	–	–	–	+	+	–	–	+	+	+	+	+	+	+	+	+	+	+	+	+	+	+	+	+	+	+	–	+	+	+	–	–	–	–	–	–	–	+	–	+	–	–
1778	1929	predicted phosphatase/phosphohexomutase	27	+	+	+	+	+	+	+	+	+	+	+	+	+	+	+	–	–	–	–	–	+	+	–	–	–	–	–	–	–	–	–	–	+	+	+	+	+	+	+	+	–	–	–	+	–	–	–	–	–	–	–	–	–	–	–	+	–
1779	1953	hypothetical protein	27	+	+	+	+	+	+	+	+	+	+	+	+	+	+	+	–	+	+	–	–	–	–	–	–	–	–	–	–	–	–	–	–	+	+	+	+	+	+	+	+	–	–	+	–	–	–	–	–	–	–	–	–	–	–	–	–	+
1780	1975	Glycosyl transferase, family 2	27	+	+	+	+	+	+	+	+	+	+	+	+	+	+	–	–	+	+	+	+	+	+	–	–	–	–	+	+	+	+	+	+	–	–	–	–	–	–	–	–	–	–	–	–	–	+	–	–	–	–	–	–	–	–	–	–	–
1781	1979	Potassium voltage-gated channel subfamily KQT; possible potassium channel, VIC family	27	+	+	+	+	+	+	+	+	+	+	+	+	+	+	–	–	+	+	–	–	–	–	–	–	–	–	–	–	–	–	+	+	+	+	+	+	+	+	–	–	–	–	+	+	–	–	–	–	+	–	–	–	–	–	–	–	–
1782	1980	hypothetical protein	27	–	–	–	–	–	–	–	–	–	–	–	–	–	–	–	–	–	–	+	+	+	+	+	+	+	+	+	+	+	+	+	+	+	+	+	–	+	–	+	+	+	–	+	+	+	–	+	+	+	–	–	–	–	–	–	–	–
1783	1268	Transcriptional regulator, AraC family	26	–	–	–	–	–	–	–	–	–	–	–	–	–	–	–	–	–	–	–	+	+	+	+	+	+	+	+	+	+	+	+	+	+	+	+	+	+	–	–	–	–	–	–	–	+	+	+	+	+	+	–	+	–	–	–	+	–
1784	1369	Response regulator of the competence regulon ComE	26	+	+	+	+	+	+	+	+	+	+	+	+	+	+	+	–	–	–	–	–	–	–	–	–	–	–	–	–	–	–	–	–	–	–	–	+	+	+	–	–	–	–	–	+	–	–	–	–	+	+	+	+	+	+	+	–	–
1785	1595	Acetyltransferase, GNAT family	26	–	–	–	–	–	–	–	–	–	–	–	–	–	–	–	–	+	+	–	+	+	+	+	+	+	+	+	+	+	+	+	+	–	–	–	–	–	–	–	–	+	–	–	+	+	–	+	+	+	+	+	–	+	–	+	+	–
1786	1621	PTS system, beta-glucoside-specific IIB component (EC 2.7.1.69) / PTS system, beta-glucoside-specific IIC component (EC 2.7.1.69) / PTS system, beta-glucoside-specific IIA component (EC 2.7.1.69)	26	–	–	–	–	–	–	–	–	–	–	–	–	–	–	–	–	–	–	–	+	+	+	+	+	+	+	+	+	+	+	+	+	+	+	+	+	+	–	–	–	–	–	–	–	+	+	+	+	+	+	–	+	–	–	–	+	–
1787	1679	Phosphoglycerate mutase family	26	–	–	–	–	–	–	–	–	–	–	–	–	–	–	–	–	–	–	–	+	+	+	+	+	+	+	+	+	+	+	+	+	+	+	+	+	+	–	–	–	–	–	–	–	+	+	+	+	+	+	–	+	–	–	–	+	–
1788	1688	Beta-glucoside bgl operon antiterminator, BglG family	26	–	–	–	–	–	–	–	–	–	–	–	–	–	–	–	–	–	–	–	+	+	+	+	+	+	+	+	+	+	+	+	+	+	+	+	+	+	–	–	–	–	–	–	–	+	+	+	+	+	+	–	+	–	–	–	+	–
1789	1726	Integrase/recombinase, putative, truncated	26	–	–	–	–	–	–	–	–	–	–	–	–	–	–	–	–	+	+	+	+	+	+	+	–	+	+	+	+	+	+	+	+	+	+	+	–	–	–	–	–	+	–	+	–	–	+	+	+	–	+	–	+	–	+	–	–	–
1790	1733	Cytidine/deoxycytidylate deaminase family protein	26	–	–	–	–	–	–	–	–	–	–	–	–	–	–	–	–	+	+	–	+	+	+	+	+	+	+	+	+	+	+	+	+	–	+	–	–	–	–	+	+	+	+	+	–	–	–	–	–	–	+	+	+	+	+	–	–	–
1791	1747	PTS system, sucrose-specific IIB component (EC 2.7.1.69) / PTS system, sucrose-specific IIC component (EC 2.7.1.69) / PTS system, sucrose-specific IIA component (EC 2.7.1.69)	26	–	–	–	–	–	–	–	–	–	–	–	–	–	–	–	+	–	–	+	+	+	+	+	+	+	+	+	+	+	+	+	+	+	+	+	+	+	+	–	–	–	–	+	–	–	–	+	+	+	–	–	–	–	–	+	–	–
1792	1818	Conserved domain protein	26	–	–	–	–	–	–	–	–	–	–	–	–	–	–	–	–	–	–	–	+	+	+	+	+	+	+	+	+	+	+	+	+	+	+	+	+	+	–	–	–	–	–	–	–	+	+	+	+	+	+	–	+	–	–	–	+	–
1793	1853	RelB/StbD replicon stabilization protein (antitoxin to RelE/StbE)	26	+	+	+	+	+	+	+	+	+	+	+	+	+	+	–	–	+	+	–	–	–	–	+	+	+	+	+	+	+	+	+	+	–	–	–	–	–	–	–	–	–	–	–	–	–	–	–	–	–	–	–	–	–	–	–
1794	1905	Substrate-specific component STY3230 of queuosine-regulated ECF transporter	26	+	+	+	+	+	+	+	+	+	+	+	+	+	+	+	–	–	–	–	–	+	+	–	–	–	–	–	–	–	–	–	–	+	+	+	+	+	+	+	+	–	–	–	+	–	–	–	–	–	–	–	–	–	–	–	–	–
1795	1928	Tn916, transcriptional regulator, putative	26	–	–	–	–	+	–	–	–	–	–	–	–	–	–	–	–	–	–	–	+	–	–	–	+	+	–	–	+	+	+	+	+	+	+	+	+	+	+	–	–	–	+	+	+	+	–	+	–	+	–	+	+	+	+	+	–	–
1796	1932	hypothetical protein	26	–	–	–	–	–	–	+	+	+	–	–	–	–	+	–	–	–	–	–	+	+	+	–	+	–	–	+	+	–	+	+	+	+	+	+	+	+	+	–	–	–	–	+	+	–	–	+	+	+	–	–	–	–	+	+	–	–
1797	1933	conserved domain protein	26	–	–	–	–	+	–	+	+	+	–	–	–	–	+	–	–	–	–	–	+	+	+	–	+	–	–	+	+	+	+	+	+	+	+	+	–	–	–	–	–	–	–	+	+	+	–	–	+	+	–	+	+	–	–	+	+	–
1798	1978	hypothetical protein	26	+	+	–	–	+	+	+	+	+	+	+	+	+	+	–	+	+	–	+	+	+	–	+	+	–	–	–	–	–	–	–	+	–	–	–	–	+	–	+	+	–	–	+	–	–	–	–	–	–	–	+	–	–	–	–	+	–
1799	2006	hypothetical protein	26	+	+	+	+	+	+	+	+	+	+	+	+	+	+	–	–	–	–	–	+	+	+	+	+	+	+	+	+	+	+	–	–	–	–	–	–	–	–	–	–	+	–	–	–	–	–	–	–	–	–	–	–	–	–	–	–	–
1800	2012	Predicted transcriptional regulator	26	+	+	+	+	+	+	+	+	+	+	+	+	+	+	+	–	–	–	–	+	–	–	+	+	–	–	–	–	–	–	–	–	–	–	–	–	+	–	–	–	–	+	–	–	–	–	–	–	–	+	+	+	–	+	–	+	+
1801	2042	hypothetical protein	26	+	+	+	+	+	+	+	+	+	+	+	+	+	+	–	–	+	+	–	–	–	–	–	–	–	–	–	–	–	–	–	–	+	+	+	+	+	+	–	–	+	–	–	+	–	–	–	–	–	–	–	–	+	–	+	–	–
1802	2048	hypothetical protein	26	–	–	+	–	–	–	+	+	–	–	+	–	–	+	+	–	–	–	+	+	+	+	+	–	–	–	–	–	–	+	+	+	+	–	–	+	+	+	+	+	+	–	+	+	+	+	+	–	–	–	–	–	–	–	–	–	–
1803	2049	cobalt ABC transporter ATP-binding protein	26	+	+	+	+	+	+	+	+	+	+	+	+	+	+	+	–	–	–	–	–	+	+	–	–	–	–	–	–	–	–	–	–	+	+	+	+	+	+	+	+	–	–	–	+	–	–	–	–	–	–	–	–	–	–	–	–	–
1804	2050	Transmembrane component STY3231 of energizing module of queuosine-regulated ECF transporter	26	+	+	+	+	+	+	+	+	+	+	+	+	+	+	+	–	–	–	–	–	+	+	–	–	–	–	–	–	–	–	–	–	+	+	+	+	+	+	+	+	–	–	–	+	–	–	–	–	–	–	–	–	–	–	–	–	–
1805	2051	Predicted N-ribosylNicotinamide CRP-like regulator	26	+	+	+	+	+	+	+	+	+	+	+	+	+	+	+	–	–	–	–	–	+	+	–	–	–	–	–	–	–	–	–	–	+	+	+	+	+	+	+	+	–	–	–	+	–	–	–	–	–	–	–	–	–	–	–	–	–
1806	2054	N-Ribosylnicotinamide phosphorylase (EC 2.4.2.1)	26	+	+	+	+	+	+	+	+	+	+	+	+	+	+	+	–	–	–	–	–	+	+	–	–	–	–	–	–	–	–	–	–	+	+	+	+	+	+	+	+	–	–	–	+	–	–	–	–	–	–	–	–	–	–	–	–	–
1807	2055	ATPase component STY3233 of energizing module of queuosine-regulated ECF transporter	26	+	+	+	+	+	+	+	+	+	+	+	+	+	+	+	–	–	–	–	–	+	+	–	–	–	–	–	–	–	–	–	–	+	+	+	+	+	+	+	+	–	–	–	+	–	–	–	–	–	–	–	–	–	–	–	–	–
1808	2061	deoxyguanosinetriphosphate triphosphohydrolase-related protein	26	+	+	+	+	+	+	+	+	+	+	+	+	+	+	+	–	–	–	+	–	–	–	–	–	–	–	–	–	–	–	–	–	+	+	+	+	+	+	–	–	+	+	–	+	+	–	–	–	–	–	–	–	–	–	–	–	–
1809	15	Mobile element protein	25	+	+	+	+	+	+	–	–	+	+	+	+	–	+	+	+	–	+	+	+	–	–	–	+	–	+	–	–	–	–	+	+	–	+	–	–	–	–	–	+	–	–	+	–	–	–	–	–	+	–	–	–	+	–	–	–	–
1810	19	transposase of IS200 family	25	–	–	+	+	+	+	–	–	+	–	+	–	–	–	–	–	+	+	–	–	–	–	+	–	–	–	+	–	–	–	–	+	+	+	–	+	+	+	+	+	–	+	+	+	–	–	–	–	+	+	–	–	+	–	+	–	–
1811	1079	Sortase A, LPXTG specific	25	+	+	+	+	–	+	+	+	+	+	+	+	+	+	+	–	–	–	+	–	–	–	–	–	–	–	–	–	–	–	–	–	–	–	–	–	–	–	+	+	+	–	–	+	–	–	–	–	–	+	+	+	+	+	+	–	–
1812	1149	Cysteine ABC transporter, substrate-binding protein	25	–	–	–	–	–	–	–	–	–	–	–	–	–	–	–	–	–	–	–	–	–	–	+	+	+	+	+	+	+	+	+	+	+	+	+	+	+	–	–	–	+	+	+	–	+	+	+	+	+	–	–	–	–	–	–	+	+
1813	1637	Haloacid dehalogenase-like hydrolase	25	–	–	–	–	–	–	–	–	–	–	–	–	–	–	–	–	–	–	–	+	+	+	+	–	+	+	+	+	+	+	+	+	+	+	+	+	+	–	–	–	–	–	–	–	+	+	+	+	+	+	–	+	–	–	–	+	–
1814	1751	SNF2 family protein	25	–	–	–	–	+	–	–	+	+	–	–	–	–	–	–	+	–	–	–	+	+	+	–	–	–	–	+	+	–	+	+	+	–	–	–	+	+	–	+	+	–	+	–	+	–	–	+	+	–	+	+	–	+	–	+	+	–
1815	1783	Oxygen-insensitive NADPH nitroreductase (EC 1.-.-.-)	25	+	+	+	+	+	+	+	+	+	–	+	+	+	+	+	–	+	+	–	–	–	–	–	–	–	–	–	+	+	–	+	+	+	+	+	–	–	–	–	–	–	–	–	–	–	–	–	–	+	–	–	+	–	–	–	–	–
1816	1879	Glutamine amidotransferase, class I	25	–	–	–	–	–	–	–	–	–	–	–	–	–	–	–	–	–	–	–	–	–	–	+	+	+	+	+	+	+	+	+	+	+	+	+	+	+	–	–	–	+	+	+	–	+	+	+	+	+	–	–	–	–	–	–	+	+
1817	1925	Ribosomal-protein-S5p-alanine acetyltransferase	25	–	–	–	–	–	–	–	–	–	–	–	–	–	–	+	–	–	–	–	+	+	+	+	+	+	+	+	+	+	+	+	+	–	–	–	–	–	–	–	–	–	+	+	–	–	+	+	+	+	+	+	+	–	–	–	+	+
1818	1961	hypothetical protein	25	+	+	+	+	+	+	+	+	+	+	+	+	+	+	+	+	+	–	–	–	–	–	–	–	–	–	–	–	–	–	–	–	–	–	–	–	–	–	+	+	–	–	–	–	–	–	–	–	+	+	+	+	+	+	+	–	–
1819	1985	hypothetical protein	25	+	+	+	+	+	+	+	+	+	+	+	+	+	+	+	+	+	–	–	–	–	–	–	–	–	–	–	–	–	–	–	–	–	–	–	–	–	–	+	+	–	–	–	–	–	–	–	–	+	+	+	+	+	+	+	–	–

Table S5. Precence/absence of each HG among 57 *S. suis* strains

				1																13			25					28						104		94			76			53/54		Divergent *S. suis*																
Index[a]	ClutserID	Gene products	Members	98HAH33	05ZYH33	SC84	A7	SS12	P1/7	DAT273	NIAH11434	JS14	GZ1	BM407	S735	13730	MNCM07	NCTC 10446	14636	NCTC 10237	ST1	2651	89-1591	MNCM04	MNCM25	8074	D9	4961	ST3	MNCM43	DAT292	DAT260	NIAH11435	MNCM21	MNCM50	89-2479	DAT234	DAT290	93A	42A	NT77	6407	11538	8830	12814	92-1400	89-590	2726	2524	89-3576-3	4417	D12	10581	89-5259	88-5299A	R61	92-1191	14A	92-4172	22083
1820	2071	lipase	25	+	+	+	+	+	+	+	+	+	+	+	+	+	+	+	–	+	+	–	–	–	–	–	–	–	–	–	–	–	–	–	–	+	+	+	–	–	–	–	–	–	–	–	+	+	–	+	+	+	–	–	–	–	–	–	–	–
1821	2078	ABC transporter, ATP-binding protein	25	+	+	+	+	+	+	+	+	+	+	+	+	+	+	+	–	–	–	+	–	–	–	–	–	–	–	–	–	–	–	–	–	–	–	–	–	–	–	+	+	+	–	+	–	+	+	–	+	–	+	–	–	–	+	–	–	–
1822	2085	hypothetical protein	25	+	+	+	+	+	–	+	+	–	–	–	–	+	–	–	–	–	–	–	+	–	+	–	+	–	+	–	–	+	+	+	+	–	+	–	–	+	–	–	–	+	+	–	+	–	–	–	+	–	–	–	+	+	–	–	–	+
1823	1748	Epimerase/dehydratase WbkD	24	–	–	–	–	–	–	–	–	–	–	–	–	–	–	–	–	–	–	–	–	–	–	+	+	+	+	–	–	–	–	–	–	+	–	+	+	+	+	+	+	+	+	+	+	–	–	–	+	+	+	–	+	+	+	+	+	+
1824	1883	N-acetylmuramoyl-L-alanine amidase (EC 3.5.1.28)	24	+	+	+	+	+	+	–	–	+	+	+	+	–	–	–	–	+	+	–	+	–	–	–	+	–	+	–	–	–	–	+	+	–	+	+	–	+	–	+	+	–	–	–	–	–	–	–	–	+	–	–	+	–	–	–	–	–
1825	1954	Pli0008 protein	24	–	–	–	–	–	–	–	–	–	–	–	–	–	–	–	–	–	–	–	+	+	+	+	+	+	–	+	+	+	+	+	+	+	+	+	+	+	+	–	–	+	+	–	–	–	–	–	–	–	–	+	–	+	+	+	–	–
1826	1981	hypothetical protein	24	–	–	–	–	+	–	–	–	–	–	–	–	–	–	+	–	–	–	–	+	+	+	+	–	+	+	+	+	+	+	+	+	+	+	–	+	+	+	+	–	–	–	–	–	–	–	–	–	–	–	+	–	+	+	+	+	–
1827	1984	Sortase A, LPXTG specific	24	+	+	+	+	–	+	+	+	+	+	+	+	+	+	+	–	–	–	+	–	–	–	–	–	–	–	–	–	–	–	–	–	–	–	–	–	–	–	+	+	–	–	–	+	–	–	–	–	–	+	+	+	+	+	+	–	–
1828	2007	Cell wall surface anchor family protein	24	+	+	+	+	–	+	+	+	+	+	+	+	+	+	+	–	–	–	+	–	–	–	–	–	–	–	–	–	–	–	–	–	–	–	–	–	–	–	+	+	–	–	–	+	–	–	–	–	–	+	+	+	+	+	+	–	–
1829	2009	L-fuculose phosphate aldolase (EC 4.1.2.17)	24	–	–	–	+	–	–	–	–	–	–	–	–	–	+	–	+	–	–	–	+	+	+	+	+	–	–	+	–	+	+	+	+	+	+	+	+	–	+	+	+	–	–	–	–	–	–	–	–	–	–	–	+	+	+	+	–	–
1830	2040	hypothetical protein	24	+	+	+	+	+	+	+	+	+	+	+	+	+	+	+	+	+	+	+	–	–	–	–	–	–	–	–	–	–	–	–	–	–	–	–	–	–	–	+	+	+	+	–	+	–	–	–	–	–	–	–	–	–	–	–	–	–
1831	2056	L-fucose operon activator	24	–	–	–	+	–	–	–	–	–	–	–	–	–	+	–	+	–	–	–	+	+	+	+	+	–	–	+	–	+	+	+	+	+	+	+	+	–	+	+	+	–	–	–	–	–	–	–	–	–	–	–	+	+	+	+	–	–
1832	2057	hypothetical protein	24	+	+	+	+	+	+	+	+	+	+	+	–	–	–	–	+	–	–	+	–	–	–	–	–	–	–	+	+	+	+	+	+	+	+	–	–	+	+	–	–	–	+	–	–	–	–	–	–	–	–	–	–	–	–	–	–	–
1833	2076	ABC-type uncharacterized transport system, ATPase component	24	–	–	–	–	–	–	–	–	–	–	–	–	–	–	–	–	–	–	–	+	+	+	+	+	+	+	+	+	+	+	+	+	+	+	+	–	–	–	–	–	–	+	–	+	+	–	+	+	+	–	+	–	–	–	–	+	–
1834	2077	Conserved domain protein SP0160	24	–	–	–	–	–	–	–	–	–	–	–	–	–	–	–	–	–	–	–	+	+	+	+	+	+	+	+	+	+	+	+	+	+	+	+	+	+	–	–	–	+	+	–	–	–	+	+	–	+	–	–	–	–	–	–	–	+
1835	2084	hypothetical protein	24	+	+	+	+	+	+	+	+	+	+	+	+	+	+	+	+	–	–	–	–	–	–	–	–	–	–	–	–	–	–	–	–	+	+	+	+	+	+	–	–	–	–	–	–	–	–	–	–	–	+	–	+	–	–	–	–	–
1836	2102	hypothetical protein	24	–	–	–	–	–	–	–	–	–	–	–	–	–	–	–	–	+	+	–	+	+	+	+	+	+	+	+	+	+	+	+	+	–	–	–	–	–	–	+	+	–	–	–	–	–	+	+	–	+	+	+	+	–	–	+	–	–
1837	2103	hypothetical protein	24	+	+	–	–	+	–	+	+	+	+	+	+	–	+	+	–	+	–	–	+	+	+	–	+	–	+	+	+	–	+	+	+	–	–	–	–	–	–	–	–	+	–	–	+	–	–	–	–	–	–	–	–	–	–	–	–	–
1838	2105	hypothetical protein	24	+	+	+	+	+	+	+	+	+	+	+	+	+	+	+	–	+	+	+	–	+	+	+	+	–	–	–	–	–	–	–	–	–	–	–	–	–	–	–	–	+	+	–	–	–	–	–	–	–	–	–	–	–	–	–	–	–
1839	2125	hypothetical protein	24	+	+	+	+	+	+	+	+	+	+	–	+	+	+	+	–	–	–	+	–	+	+	–	–	–	–	–	–	–	–	–	–	+	+	+	+	+	+	–	–	–	–	+	–	–	–	–	–	–	–	–	–	–	–	–	–	–
1840	22	Transposase	23	+	+	+	+	+	+	+	+	+	+	+	+	+	+	+	+	–	–	–	–	–	–	–	+	–	–	–	–	–	–	–	–	–	–	–	–	+	–	–	–	+	+	–	+	–	–	–	–	–	+	–	–	+	–	–	–	–
1841	1599	Hypothetical, related to broad specificity phosphatases COG0406	23	–	–	–	–	–	–	–	–	–	–	–	–	–	–	–	–	–	–	–	+	+	+	+	+	+	+	+	+	+	+	–	–	+	+	–	+	+	–	–	–	–	–	–	–	+	+	+	+	+	+	–	+	–	–	–	+	–
1842	2005	hypothetical protein	23	–	–	–	–	+	–	–	–	–	–	–	–	–	–	+	–	–	–	+	+	+	+	+	–	+	–	–	+	–	–	–	–	+	+	–	+	+	+	+	–	+	–	–	–	–	–	+	–	–	+	+	+	+	+	+	–	–
1843	2013	Type I restriction-modification system, restriction subunit R (EC 3.1.21.3)	23	+	+	+	+	+	+	+	+	+	+	+	+	+	+	+	+	–	–	–	–	+	+	–	–	–	–	–	–	–	–	–	–	+	+	+	–	+	–	–	–	–	–	–	–	–	–	–	–	–	–	–	+	–	–	–	–	–
1844	2053	L-fucose isomerase (EC 5.3.1.25)	23	–	–	–	+	–	–	–	–	–	–	–	–	–	+	–	+	–	–	–	+	+	+	+	+	–	–	+	–	+	+	+	+	+	+	+	+	–	–	+	+	–	–	–	–	–	–	–	–	–	–	–	+	+	+	+	–	–
1845	2070	Mobile element protein	23	+	+	+	+	+	+	+	+	+	+	+	+	+	+	–	–	+	+	+	–	–	–	–	–	–	–	+	+	+	+	+	+	–	–	–	–	–	–	–	–	–	–	–	–	–	–	–	–	–	–	–	–	–	–	–	–	–
1846	2072	hypothetical protein	23	+	+	+	+	+	+	+	+	+	+	+	+	+	–	–	–	+	+	+	–	+	–	–	–	–	–	+	+	+	+	+	+	–	–	–	–	–	–	–	–	–	–	–	–	–	–	–	–	–	–	–	–	–	–	–	–	–
1847	2074	hypothetical protein	23	–	–	–	–	–	–	–	–	–	–	–	–	–	–	+	+	–	–	+	+	+	+	+	+	+	+	+	+	+	+	+	+	+	+	+	–	–	–	–	–	+	+	–	–	–	–	–	+	+	–	–	–	–	–	–	–	–
1848	2083	FtsK protein	23	+	+	+	+	+	+	+	+	+	+	+	–	–	–	+	+	–	–	+	–	–	–	–	–	–	–	+	+	+	+	–	–	+	–	+	+	–	+	–	–	–	+	–	–	–	–	–	–	–	–	–	–	–	–	–	–	–
1849	2090	RNA polymerase ECF-type sigma factor	23	–	–	–	–	+	–	+	–	–	–	–	–	–	+	–	–	–	–	–	–	+	+	–	+	–	–	+	–	–	+	+	+	+	+	+	+	+	+	–	–	–	–	+	+	–	–	+	–	+	–	–	+	–	+	+	–	–
1850	2091	hypothetical protein	23	–	–	–	–	–	–	+	–	–	–	–	–	–	+	–	–	–	–	–	+	+	+	+	+	–	–	+	–	–	+	+	+	+	+	+	+	+	+	–	–	–	–	+	+	–	–	+	–	+	–	–	+	–	+	+	–	–
1851	2108	hypothetical protein	23	–	–	–	–	–	–	–	–	–	–	–	–	–	–	–	–	–	–	–	+	+	+	+	+	+	+	+	+	+	+	+	+	+	+	+	–	–	–	+	+	+	+	–	–	+	–	–	–	+	–	–	–	+	+	–	–	–
1852	2109	hypothetical protein	23	+	+	+	–	+	+	–	+	+	–	+	+	–	+	–	+	–	–	–	–	+	+	+	+	+	+	–	–	–	+	+	+	–	–	–	–	–	–	–	+	–	–	–	–	–	–	–	–	–	–	–	–	–	+	–
1853	2110	Competence-specific sigma factor ComX	23	+	+	+	+	+	+	+	+	+	–	+	–	–	–	+	–	–	–	–	+	–	+	+	+	+	+	–	–	–	+	+	+	–	–	–	–	–	–	–	–	–	+	–	–	–	–	–	–	–	–	+	–	–	–	–	–	–
1854	2115	Cell wall surface anchor family protein, LPXTG motif	23	+	+	+	+	–	+	+	+	+	+	+	+	+	+	+	–	–	–	+	–	–	–	–	–	–	–	–	–	–	–	–	–	–	–	–	–	–	–	+	+	–	–	–	+	–	–	–	–	–	+	+	+	+	–	+	–	–
1855	2143	hypothetical protein	23	–	–	–	–	–	–	–	–	–	–	–	–	–	–	+	–	–	–	–	+	+	+	+	+	+	+	+	+	+	+	+	+	+	+	+	+	+	+	–	–	+	–	+	–	–	–	–	–	–	–	–	–	–	–	+	–	–
1856	2146	Cell division protein FtsQ	23	–	–	+	–	+	+	–	+	+	+	+	+	+	–	–	–	–	–	–	–	–	–	–	–	–	–	–	–	–	–	+	+	–	–	–	–	–	+	+	+	–	+	–	–	+	+	–	–	–	+	–	+	+	+	–	+	–
1857	2153	hypothetical protein	23	+	+	+	+	+	+	+	+	+	+	+	+	+	+	+	+	–	–	–	–	+	+	–	–	–	–	–	–	–	–	–	–	+	+	+	–	–	+	–	–	–	–	+	–	–	–	–	–	–	–	–	–	–	–	–	–	–
1858	1585	PTS system, cellobiose-specific IIB component (EC 2.7.1.69)	22	–	–	–	–	–	–	–	–	–	–	–	–	–	–	–	–	–	–	–	+	+	+	+	+	+	+	+	+	+	+	+	+	+	+	+	+	+	–	–	–	–	–	–	–	–	–	+	+	–	+	–	+	–	–	–	–	–
1859	1607	PTS system, cellobiose-specific IIA component (EC 2.7.1.69)	22	–	–	–	–	–	–	–	–	–	–	–	–	–	–	–	–	–	–	–	+	+	+	+	+	+	+	+	+	+	+	+	+	+	+	+	+	+	–	–	–	–	–	–	–	–	–	+	+	–	+	–	+	–	–	–	–	–
1860	1680	PTS system, cellobiose-specific IIC component (EC 2.7.1.69)	22	–	–	–	–	–	–	–	–	–	–	–	–	–	–	–	–	–	–	–	+	+	+	+	+	+	+	+	+	+	+	+	+	+	+	+	+	+	–	–	–	–	–	–	–	–	–	+	+	–	+	–	+	–	–	–	–	–
1861	1808	hypothetical protein	22	+	+	+	+	+	+	+	+	+	+	+	+	+	+	+	–	–	–	–	–	–	–	–	–	–	–	–	–	–	–	+	+	–	–	–	–	–	–	+	+	–	–	–	+	–	–	–	–	–	–	+	–	–	–	–	+	–
1862	1824	hypothetical protein	22	–	–	–	–	–	–	–	–	–	–	–	–	–	–	–	–	–	–	–	+	+	+	+	+	+	+	+	+	+	+	+	+	+	+	+	+	+	–	–	–	–	–	–	–	–	–	+	+	–	+	–	+	–	–	–	–	–
1863	1835	ABC transporter protein BldKB	22	–	–	–	–	–	–	–	–	–	–	–	–	–	–	–	–	+	+	+	+	+	+	–	–	–	–	+	–	+	+	+	+	+	+	+	–	–	–	–	–	+	–	+	–	–	+	+	–	–	–	+	+	–	+	–	–	–
1864	1902	Transcriptional antiterminator of lichenan operon, BglG family	22	–	–	–	–	–	–	–	–	–	–	–	–	–	–	–	–	–	–	–	+	+	+	+	+	+	+	+	+	+	+	+	+	+	+	+	+	+	–	–	–	–	–	–	–	–	–	+	+	–	+	–	+	–	–	–	–	–
1865	1963	Glycosyltransferase	22	+	+	+	+	+	+	+	+	–	+	+	+	–	–	+	–	–	–	+	+	+	+	–	–	–	–	+	+	+	+	+	+	–	–	–	–	–	–	–	–	–	–	–	–	–	–	–	–	–	–	–	–	–	–	–
1866	2003	conserved domain protein	22	–	–	–	–	+	–	–	–	–	–	–	–	–	–	–	–	+	–	+	+	+	+	+	–	–	–	+	+	+	+	+	+	+	+	–	+	+	+	–	–	–	–	–	–	–	–	–	–	–	–	+	–	–	+	–	+	+
1867	2010	prophage LambdaSa2, site-specific recombinase, phage integrase family	22	+	+	+	+	+	+	+	–	+	+	+	–	+	+	–	–	–	–	–	–	–	–	–	–	–	–	–	–	–	–	–	–	–	–	+	+	–	+	–	+	–	–	–	–	–	–	+	–	–	+	+	+	–	–	+	–	+
1868	2046	replisome organizer	22	–	–	–	–	–	–	–	–	–	–	–	–	–	–	–	–	–	–	–	–	+	+	+	+	+	+	+	+	+	+	–	–	–	–	–	+	+	+	+	+	+	–	–	–	+	–	+	–	+	+	–	+	–	–	–	–	+
1869	2106	Methyl-accepting chemotaxis protein	22	–	–	–	–	–	–	–	–	–	–	–	–	–	–	–	+	+	+	+	+	–	–	+	+	+	+	+	+	+	+	+	+	–	–	–	–	–	–	–	–	–	–	–	–	+	+	+	+	+	–	+	–	–	–	–	+	+
1870	2113	hypothetical protein	22	–	–	–	–	–	–	–	–	–	–	–	–	–	–	–	+	–	–	+	–	–	–	–	–	–	–	–	–	–	–	–	–	+	+	+	+	+	+	+	+	+	+	+	+	+	–	+	+	+	–	–	+	+	+	+	–	–
1871	2114	hypothetical protein	22	–	–	–	–	–	–	–	–	–	–	–	–	–	–	–	–	–	–	–	+	+	+	+	+	+	+	+	+	+	+	+	+	+	+	+	+	+	–	–	–	–	–	–	–	–	–	+	+	–	+	–	+	–	–	–	–	–
1872	2120	hypothetical protein	22	+	+	+	+	+	+	+	+	+	+	+	–	–	–	–	+	–	–	+	–	–	–	–	–	–	–	–	–	–	–	+	+	+	+	+	+	–	+	–	–	+	+	–	–	–	–	–	–	–	–	–	–	–	–	–	–	–
1873	2145	hypothetical protein	22	+	+	+	+	+	+	+	+	+	+	+	+	+	+	–	–	–	–	–	–	–	–	–	–	+	+	+	+	+	+	+	+	–	–	–	–	–	–	–	–	–	–	–	–	–	–	–	–	–	–	–	–	–	–	–
1874	2152	Type I restriction-modification system, DNA-methyltransferase subunit M (EC 2.1.1.72)	22	+	+	+	+	+	+	+	+	+	+	+	+	+	+	+	+	–	–	–	–	+	+	–	–	–	–	–	–	–	–	–	–	–	–	–	+	+	+	–	–	–	–	–	–	–	–	–	–	–	–	–	+	–	–	–	–	–
1875	2181	Cu+ P-type ATPase	22	+	+	+	+	+	+	+	+	+	+	+	+	+	+	–	+	–	–	–	+	–	–	–	+	–	+	+	–	–	+	–	+	–	–	–	–	–	–	+	–	–	–	–	–	–	–	–	–	–	–	–	–	–	–	–
1876	2182	hypothetical protein	22	+	+	+	+	+	+	+	+	+	+	+	+	+	+	+	–	–	–	+	–	–	–	–	–	–	–	–	–	–	–	–	–	–	–	–	+	–	+	+	+	–	–	–	–	–	–	–	–	+	–	–	–	–	–	–	–	+
1877	2183	hypothetical protein	22	+	+	+	+	+	+	+	+	+	+	+	+	–	+	+	–	–	–	–	+	–	–	–	+	–	+	–	–	–	–	+	+	–	–	–	+	–	–	+	+	–	–	–	–	–	–	–	–	–	–	–	–	–	–	–
1878	1306	Chromosome segregation helicase	21	–	–	–	–	–	–	–	–	–	–	–	–	+	–	+	–	–	–	+	–	–	–	–	–	–	–	–	+	+	–	–	–	+	+	+	–	+	–	–	+	+	+	–	–	–	+	+	+	+	+	+	–	+	+	+	–	–
1879	1586	hypothetical protein	21	–	–	–	–	–	–	–	–	+	–	+	–	–	–	–	+	–	–	–	–	–	–	–	+	–	–	+	–	+	+	–	–	+	+	+	+	+	+	+	+	–	–	+	–	–	–	–	–	+	+	+	+	–	+	–	–	–
1880	1960	Phage protein	21	+	+	+	+	+	+	+	–	+	+	+	–	+	+	–	–	–	–	+	–	–	–	–	–	–	–	–	–	–	–	–	–	–	+	–	+	+	–	+	+	+	–	+	–	–	–	–	–	–	–	–	–	–	–	–	–	+
1881	1982	ATP-dependent DNA helicase recG (EC 3.6.1.-)	21	–	–	–	–	–	–	–	–	–	–	–	–	–	–	–	–	–	–	–	+	+	+	+	+	+	+	+	+	+	+	+	+	–	–	–	+	+	+	–	–	–	–	–	+	–	+	–	–	–	+	–	–	+	+	–	–	–
1882	2001	Antirepressor	21	–	–	–	–	+	–	–	–	–	–	–	–	–	–	–	–	+	–	–	+	–	–	+	–	–	–	–	+	–	–	+	+	+	+	+	–	–	+	+	+	–	–	–	+	–	–	+	–	–	+	+	+	–	+	+	–	+
1883	2047	ABC transporter, ATP-binding protein	21	–	–	–	–	–	–	–	–	–	–	–	–	–	–	–	–	–	–	–	+	+	+	+	+	+	+	+	+	+	+	+	+	+	+	+	+	+	+	+	+	–	–	–	–	–	–	–	–	–	–	–	–	–	–	–	–	–
1884	2112	hypothetical protein	21	–	–	–	–	–	–	–	–	–	–	–	–	–	–	–	–	–	–	–	+	+	+	+	+	+	+	+	+	+	+	+	+	+	+	+	+	+	+	+	+	–	–	–	–	–	–	–	–	–	–	–	–	–	–	–	–	–
1885	2123	prophage Lp3 protein 1, integrase	21	+	+	+	+	+	+	+	–	+	+	+	–	+	+	–	–	–	–	–	–	–	–	–	–	–	–	–	–	–	–	–	–	–	+	–	–	+	+	–	–	–	–	–	–	–	–	–	–	–	–	+	+	+	+	+	–	+
1886	2171	Fibronectin-binding protein	21	–	–	–	–	–	–	–	–	–	–	–	–	–	–	–	–	+	+	+	–	–	–	+	+	+	+	+	+	+	+	+	+	+	+	+	+	+	+	+	+	–	–	–	–	–	–	–	–	–	–	–	–	–	–	–	–	–
1887	2174	hypothetical protein	21	–	–	–	–	–	–	–	–	–	–	–	–	–	–	–	–	–	–	–	+	+	+	+	+	+	+	+	+	+	+	+	+	+	+	+	+	+	+	+	+	–	–	–	–	–	–	–	–	–	–	–	–	–	–	–	–	–
1888	2175	Endo-beta-N-acetylglucosaminidase (EC 3.2.1.96)	21	–	–	–	–	–	–	–	–	–	–	–	–	–	–	+	–	–	–	–	+	+	+	+	+	+	+	+	+	+	+	–	–	+	+	+	+	+	+	–	–	–	–	–	+	–	–	–	–	–	–	–	+	–	–	+	–	–
1889	2179	hypothetical protein	21	–	–	–	–	–	–	–	–	–	–	–	–	–	–	–	–	–	–	–	+	+	+	+	+	+	+	+	+	+	+	+	+	+	+	+	+	+	+	+	+	–	–	–	–	–	–	–	–	–	–	–	–	–	–	–	–	–
1890	2190	hypothetical protein	21	+	+	+	+	+	+	+	+	+	+	+	–	–	–	–	+	–	–	+	–	–	–	–	–	–	–	+	+	+	+	–	–	+	+	–	–	–	+	–	–	–	+	–	–	–	–	–	–	–	–	–	–	–	–	–	–	–
1891	2217	Polysaccharide biosynthesis protein CpsH(V)	21	+	+	+	+	+	+	+	+	–	+	+	+	–	–	–	–	–	–	+	+	+	+	–	–	–	–	+	+	+	+	+	+	–	–	–	–	–	–	–	–	–	–	–	–	–	–	–	–	–	–	–	–	–	–	–
1892	2218	Cps2H	21	+	+	+	+	+	+	+	+	–	+	+	+	–	–	–	–	–	–	+	+	+	+	–	–	–	–	+	+	+	+	+	+	–	–	–	–	–	–	–	–	–	–	–	–	–	–	–	–	–	–	–	–	–	–	–
1893	2222	hypothetical protein	21	–	–	+	+	+	–	–	+	–	+	–	+	+	+	+	+	+	–	–	–	–	–	–	–	–	–	–	–	–	–	–	–	–	–	–	–	–	–	–	–	–	–	+	+	+	–	–	–	+	+	+	+	–	+	+	+	–
1894	2236	hypothetical protein	21	–	–	–	–	–	–	–	–	–	–	–	–	–	–	–	–	–	–	–	+	+	+	+	+	+	+	+	+	+	+	+	+	+	+	+	+	+	+	+	+	–	–	–	–	–	–	–	–	–	–	–	–	–	–	–	–	–
1895	2238	tagatose-6-phosphate kinase	21	+	+	+	+	+	+	+	+	+	+	+	+	+	+	+	+	–	–	–	–	–	–	–	–	–	–	–	–	–	–	–	–	–	–	–	–	–	–	+	+	+	+	–	–	–	–	–	–	–	–	–	–	–	–	+	–	–
1896	2239	Putative RTX family exoprotein A gene	21	+	+	+	+	+	+	+	+	+	+	+	+	+	+	+	–	–	–	+	–	–	–	–	–	–	–	–	–	–	–	–	–	–	–	–	+	–	–	+	–	–	–	–	–	–	+	–	–	–	–	+	–	+	–	–	–	–
1897	2240	hypothetical protein	21	+	+	+	+	+	+	+	+	+	+	+	+	+	+	+	–	–	–	–	–	–	–	–	–	–	–	–	–	–	–	–	–	–	–	–	–	–	–	+	+	–	–	+	+	+	–	–	–	+	–	–	–	–	–	–	–	–
1898	2251	hypothetical protein	21	+	+	+	+	+	+	+	+	+	+	+	+	+	+	+	–	–	–	–	–	–	–	–	–	–	–	–	–	–	–	–	–	–	–	–	–	–	–	–	–	–	–	–	+	–	–	–	–	–	+	+	–	+	+	+	–	–
1899	1573	tRNA-dependent lipid II--L-alanine ligase	20	–	–	–	–	–	–	–	–	–	–	–	–	–	–	+	+	–	–	–	–	–	–	–	–	–	–	–	–	–	–	+	+	+	+	+	+	+	+	–	–	–	–	+	+	–	–	–	+	+	+	+	+	+	+	+	–	–
1900	1786	Possible glyoxylase family protein (Lactoylglutathione lyase) (EC 4.4.1.5)	20	–	–	–	–	–	–	–	–	–	–	–	–	–	–	–	–	–	–	–	+	+	+	+	+	+	+	+	+	+	+	+	+	+	+	+	+	+	–	–	–	–	–	–	–	–	–	+	+	–	–	–	–	–	–	–	–	–
1901	1801	hypothetical protein	20	–	–	–	–	–	–	–	–	–	–	–	–	–	–	–	–	–	–	–	+	+	+	+	+	+	+	+	+	+	+	+	+	+	+	+	–	–	–	–	–	–	–	+	–	+	–	–	–	–	–	–	–	+	+	–	–	–
1902	1807	Pleiotropic regulator of exopolysaccharide synthesis, competence and biofilm formation Ftr, XRE family	20	–	–	–	–	–	–	–	–	+	–	+	–	–	–	–	+	–	–	–	–	–	–	–	+	–	–	+	–	+	+	–	–	+	+	+	+	–	+	+	+	–	–	+	–	+	–	–	–	+	+	+	–	–	+	–	–	–
1903	1825	hypothetical protein	20	–	–	–	–	–	–	–	–	+	–	+	–	–	–	–	+	–	–	–	–	–	–	–	+	–	–	+	–	+	+	–	–	+	+	+	+	–	+	+	+	–	–	+	–	–	–	–	–	+	+	+	+	–	+	–	–	–
1904	2144	hypothetical protein	20	–	–	–	–	–	–	–	–	–	–	–	–	–	–	–	+	–	–	–	+	+	+	+	+	+	+	+	+	+	+	+	+	–	–	–	–	–	–	–	–	–	–	–	–	–	–	–	–	–	+	+	+	+	–	+	+	–
1905	2148	hypothetical protein	20	–	–	–	–	–	–	–	–	–	–	–	–	–	–	–	–	–	–	+	–	–	–	–	–	–	–	–	–	–	–	–	–	+	–	+	+	+	+	–	–	–	–	–	+	+	+	+	+	+	+	+	+	+	+	+	+	+
1906	2154	hypothetical protein	20	+	+	+	+	+	+	+	+	+	+	+	+	+	+	–	–	–	–	–	–	–	–	–	–	–	–	–	–	–	–	–	–	–	–	–	–	–	+	–	–	–	–	–	–	+	–	–	–	–	–	+	–	+	+	+	–	–
1907	2173	hypothetical protein	20	–	–	–	–	–	–	–	–	–	–	–	–	–	–	–	+	+	+	–	–	–	–	–	–	–	–	–	–	–	–	–	–	+	+	+	+	+	+	–	–	+	+	–	–	+	+	–	+	–	+	+	+	+	+	+	–	–
1908	2228	Alpha-L-Rha alpha-1,3-L-rhamnosyltransferase (EC 2.4.1.-)	20	–	–	–	–	+	–	–	+	+	–	–	–	–	+	–	+	+	–	–	–	+	+	+	+	+	–	+	–	–	+	+	+	–	–	–	–	–	–	–	–	–	–	–	–	+	+	–	–	+	–	–	–	+	–	–	+	–
1909	2231	hypothetical protein	20	–	–	–	–	–	–	–	–	–	–	–	–	–	–	–	–	+	+	–	+	+	+	+	+	+	+	+	+	+	+	+	+	–	–	–	–	–	–	–	–	–	–	–	–	–	–	–	–	–	+	+	–	+	+	+	–	–
1910	2233	Leucine rich protein	20	–	–	–	–	–	–	–	–	–	–	–	–	–	–	–	–	–	–	–	+	+	+	+	+	+	+	+	+	+	+	+	+	–	–	–	–	–	–	–	–	+	+	+	–	–	–	–	–	–	–	–	+	+	+	+	–	–
1911	2248	hypothetical protein	20	+	+	+	+	+	+	+	+	+	+	+	+	+	+	+	–	–	–	–	–	–	–	–	–	–	–	–	–	–	–	–	–	–	–	–	–	–	–	–	–	–	+	–	–	–	–	+	–	+	–	+	–	–	–	–	+	–
1912	2276	ATPase involved in DNA repair	20	+	+	+	+	+	+	+	+	+	+	+	+	+	+	–	–	+	+	+	+	+	+	–	–	–	–	–	–	–	–	–	–	–	–	–	–	–	–	–	–	–	–	–	–	–	–	–	–	–	–	–	–	–	–	–	–	–
1913	2278	hypothetical protein	20	+	+	+	+	+	+	+	+	+	+	+	+	+	+	–	–	+	+	+	+	+	+	–	–	–	–	–	–	–	–	–	–	–	–	–	–	–	–	–	–	–	–	–	–	–	–	–	–	–	–	–	–	–	–	–	–	–
1914	2280	hypothetical protein	20	–	–	–	–	–	–	–	–	–	–	–	–	–	–	–	–	+	+	+	+	+	+	+	+	+	+	+	+	+	+	–	–	–	–	–	–	–	–	–	–	+	+	–	–	+	–	+	–	–	–	–	–	–	–	–	+	+
1915	2287	hypothetical protein	20	–	–	–	–	–	–	–	–	–	–	–	–	–	–	–	–	–	–	–	–	+	+	+	+	+	+	+	+	+	+	+	+	–	–	–	–	–	–	–	–	+	+	+	+	+	+	–	+	+	–	–	–	–	–	–	–	–
1916	2288	hypothetical protein	20	+	+	+	+	+	+	+	+	+	+	+	+	+	+	–	–	–	–	+	–	–	–	–	–	–	–	–	–	–	–	+	+	–	–	–	–	–	–	+	+	–	–	+	–	–	–	–	–	–	–	–	–	–	–	–	–	–
1917	2289	hypothetical protein	20	+	+	+	+	+	+	+	+	+	+	+	+	+	+	+	–	–	–	–	–	–	–	–	–	–	–	–	–	–	–	–	–	–	–	–	–	–	–	+	+	–	–	–	–	–	–	+	–	–	–	–	+	–	+	–	–	–
1918	2292	hypothetical protein	20	+	+	+	+	+	+	–	+	+	+	–	+	–	+	–	–	–	–	+	+	+	+	+	+	–	–	–	–	–	–	–	–	–	–	–	–	–	–	–	–	–	–	–	–	–	–	–	–	–	–	–	–	+	+	–	+	–
1919	2293	Hemolysins and related proteins containing CBS domains	20	+	+	+	+	+	+	+	+	+	+	+	+	+	+	+	+	–	–	+	–	–	–	–	–	–	–	–	–	–	–	–	–	–	–	–	–	–	–	–	–	–	–	–	–	–	–	–	–	–	–	+	–	+	–	+	–	–
1920	1668	ImpB/MucB/SamB family protein	19	–	–	–	–	–	–	–	–	+	–	+	–	–	–	–	+	–	–	–	–	–	–	–	+	–	–	+	–	+	+	–	–	+	+	+	+	–	+	+	+	–	–	+	–	–	–	–	–	+	+	+	–	–	+	–	–	–
1921	1959	Phage transcriptional repressor	19	–	–	–	–	+	–	–	–	–	–	–	–	–	–	–	–	–	–	–	+	+	–	+	–	–	–	–	–	–	–	+	+	+	+	–	–	–	+	+	–	–	+	–	–	–	–	+	+	+	+	+	+	–	+	+	+	–
1922	2014	unknown phage protein	19	+	+	+	+	+	+	+	–	+	+	+	–	+	+	–	–	–	–	–	–	–	–	–	–	–	–	–	–	–	–	–	–	–	+	–	+	+	+	–	–	–	–	–	–	–	–	–	–	–	–	–	+	+	–	–	–	+
1923	2044	Methyl-directed repair DNA adenine methylase (EC 2.1.1.72)	19	–	–	–	–	–	–	–	–	–	–	–	–	–	–	–	–	–	–	–	+	+	+	+	+	+	+	+	+	+	+	+	+	+	+	+	–	–	–	–	–	–	–	–	–	–	–	–	–	+	–	–	–	+	+	–	–	–
1924	2075	DNA modification methyltransferase (EC 2.1.1.-)	19	–	–	–	–	–	–	–	–	–	–	–	–	–	–	–	–	–	–	–	+	+	+	+	+	+	+	+	+	+	+	+	+	+	+	+	–	–	–	–	–	–	–	–	–	–	–	–	–	+	–	–	–	+	+	–	–	–
1925	2117	hypothetical protein	19	–	–	–	–	–	–	–	–	–	–	–	–	–	–	–	+	–	–	–	+	+	+	+	+	–	–	–	–	–	–	+	+	+	+	+	+	+	+	–	–	+	–	–	–	–	–	–	–	–	–	–	–	+	+	+	–	+
1926	2124	hypothetical protein	19	+	+	+	–	+	–	+	–	–	–	+	–	–	+	–	–	–	–	–	+	+	+	–	+	–	–	–	–	+	–	–	–	+	–	–	–	–	–	–	–	–	+	–	+	–	–	–	+	–	+	–	–	+	–	+	–	–
1927	2141	hypothetical protein	19	–	–	–	–	–	–	–	–	–	–	–	–	–	–	+	–	+	–	+	+	+	+	+	–	–	–	–	+	–	–	–	–	+	–	+	+	+	+	+	–	–	–	–	–	+	–	–	–	–	+	–	–	+	+	+	–	–
1928	2188	Sialic acid utilization regulator, RpiR family	19	+	+	+	+	+	+	+	+	+	+	+	+	+	+	+	+	–	–	–	–	–	–	–	–	–	–	–	–	–	–	–	–	–	–	–	–	–	–	–	–	+	–	+	+	–	–	–	–	–	–	–	–	–	–	–	–	–
1929	2189	N-acetylmuramic acid 6-phosphate etherase (EC 4.2.-.-)	19	+	+	+	+	+	+	+	+	+	+	+	+	+	+	+	+	–	–	–	–	–	–	–	–	–	–	–	–	–	–	–	–	–	–	–	–	–	–	–	–	+	–	+	+	–	–	–	–	–	–	–	–	–	–	–	–	–
1930	2192	PTS system, sucrose-specific IIB component (EC 2.7.1.69) / PTS system, sucrose-specific IIC component (EC 2.7.1.69)	19	+	+	+	+	+	+	+	+	+	+	+	+	+	+	+	+	–	–	–	–	–	–	–	–	–	–	–	–	–	–	–	–	–	–	–	–	–	–	–	–	+	–	+	+	–	–	–	–	–	–	–	–	–	–	–	–	–
1931	2193	hypothetical protein	19	+	+	+	+	+	+	+	+	+	+	+	+	+	+	+	+	–	–	–	–	–	–	–	–	–	–	–	–	–	–	–	–	–	–	–	–	–	–	–	–	+	–	+	+	–	–	–	–	–	–	–	–	–	–	–	–	–
1932	2221	unknown	19	–	–	–	–	–	–	–	–	–	–	–	–	–	–	+	+	+	+	–	–	–	–	–	–	–	–	–	–	–	–	+	+	+	+	+	+	+	+	–	–	–	–	+	+	–	–	–	–	–	–	+	+	+	+	+	–	–
1933	2229	hypothetical protein	19	–	–	–	–	–	–	–	–	–	–	–	–	–	–	+	+	+	+	–	–	–	–	–	–	–	–	–	–	–	–	+	+	+	+	+	+	+	+	–	–	–	–	+	+	–	–	–	–	–	–	+	+	+	+	+	–	–
1934	2237	hypothetical protein	19	–	–	–	–	–	–	–	–	–	–	–	–	–	–	–	–	–	–	–	+	–	–	+	+	+	+	+	+	+	+	+	+	–	–	–	–	–	–	–	–	–	–	–	–	–	–	–	–	–	+	+	+	+	+	+	+	+
1935	2294	protein with N-terminal chaperone domain,C-terminal 9 TM helices predicted	19	+	+	+	+	+	+	+	+	+	+	+	+	+	+	+	+	–	–	+	–	–	–	–	–	–	–	–	–	–	–	–	–	–	–	–	–	–	–	–	–	–	–	–	–	–	–	–	+	–	–	–	–	–	–	–	+	–
1936	2323	Rhodanese-like	19	–	–	–	+	–	–	–	–	–	–	–	–	–	–	–	–	+	+	–	–	–	–	–	–	–	–	–	–	–	–	–	–	+	+	+	+	+	+	–	–	–	+	–	–	+	–	+	+	+	–	+	–	+	+	+	–	+
1937	2333	hypothetical protein	19	–	–	–	–	–	–	+	+	–	–	–	–	+	+	+	–	–	–	–	–	–	–	+	–	+	–	+	+	+	+	+	+	+	–	+	–	–	+	–	–	–	–	–	+	–	–	+	+	–	–	–	–	–	–	–	–	–
1938	2338	hypothetical protein	19	–	–	–	–	–	–	–	–	–	–	–	–	–	–	–	–	–	–	–	+	+	+	+	+	+	+	+	+	+	+	+	+	+	+	+	+	+	+	–	–	–	–	–	–	–	–	–	–	–	–	–	–	–	–	–	–	–
1939	2339	hypothetical protein	19	–	–	–	–	–	–	–	–	–	–	–	–	–	–	–	–	–	–	–	+	+	+	+	+	+	+	+	+	+	+	+	+	+	+	+	+	+	+	–	–	–	–	–	–	–	–	–	–	–	–	–	–	–	–	–	–	–
1940	2340	Protein of unknown function DUF11:Surface protein from Gram-positive cocci, anchor region	19	–	–	–	+	–	–	–	–	–	–	–	–	+	+	+	+	–	–	+	–	+	–	+	–	+	–	+	+	+	–	–	+	+	–	–	+	–	+	–	–	+	–	–	+	–	–	–	–	–	–	–	–	–	+	–	–	–
1941	2341	N-Acetyl-D-glucosamine ABC transport system, permease protein 1	19	–	–	–	+	–	–	–	–	–	–	–	–	–	+	–	+	–	–	–	–	–	–	–	–	–	–	+	–	+	+	+	+	+	+	+	+	–	+	+	+	–	–	–	–	–	–	–	–	–	–	–	+	+	+	+	–	–
1942	2342	N-acetylglucosamine kinase bacterial type predicted (EC 2.7.1.59) / Transcriptional regulator	19	–	–	–	+	–	–	–	–	–	–	–	–	–	+	–	+	–	–	–	–	–	–	–	–	–	–	+	–	+	+	+	+	+	+	+	+	–	+	+	+	–	–	–	–	–	–	–	–	–	–	–	+	+	+	+	–	–
1943	2343	Multiple sugar ABC transporter, substrate-binding protein	19	–	–	–	+	–	–	–	–	–	–	–	–	–	+	–	+	–	–	–	–	–	–	–	–	–	–	+	–	+	+	+	+	+	+	+	+	–	+	+	+	–	–	–	–	–	–	–	–	–	–	–	+	+	+	+	–	–
1944	2344	Enterotoxin	19	–	–	–	+	–	–	–	–	–	–	–	–	–	+	–	+	–	–	–	–	–	–	–	–	–	–	+	–	+	+	+	+	+	+	+	+	–	+	+	+	–	–	–	–	–	–	–	–	–	–	–	+	+	+	+	–	–
1945	2345	Alpha-L-fucosidase (EC 3.2.1.51)	19	–	–	–	+	–	–	–	–	–	–	–	–	–	+	–	+	–	–	–	–	–	–	–	–	–	–	+	–	+	+	+	+	+	+	+	+	–	+	+	+	–	–	–	–	–	–	–	–	–	–	–	+	+	+	+	–	–
1946	2347	Multiple sugar ABC transporter, membrane-spanning permease protein MsmG	19	–	–	–	+	–	–	–	–	–	–	–	–	–	+	–	+	–	–	–	–	–	–	–	–	–	–	+	–	+	+	+	+	+	+	+	+	–	+	+	+	–	–	–	–	–	–	–	–	–	–	–	+	+	+	+	–	–
1947	1484	Xanthine phosphoribosyltransferase (EC 2.4.2.22)	18	–	–	–	–	–	–	–	–	–	–	–	–	–	–	–	–	+	+	–	–	–	–	–	–	–	–	–	–	–	–	–	–	–	–	–	–	–	–	+	+	+	–	+	+	+	–	+	+	+	–	+	+	+	+	+	+	+
1948	1762	hypothetical protein	18	–	–	–	–	–	–	–	–	–	–	–	–	–	–	+	+	+	+	–	–	–	–	–	–	–	+	+	+	+	+	+	+	–	–	–	–	–	–	–	–	–	+	–	–	+	+	+	+	+	–	–	–	–	–	–	–	–
1949	1983	Conserved Membrane Protein (Archaea)	18	–	–	–	–	–	–	–	–	–	–	–	–	–	–	+	–	–	–	+	+	+	+	+	–	+	+	+	+	+	+	–	–	–	+	–	–	–	–	–	–	+	+	+	–	–	+	–	–	+	–	–	–	–	–	–	–	–
1950	2000	Phage transcriptional regulator, Cro/CI family	18	–	–	–	–	–	–	–	–	–	–	–	–	–	–	+	–	+	+	+	–	–	–	–	–	–	–	–	+	–	–	–	–	–	–	+	+	+	–	+	+	–	–	–	+	+	–	+	–	–	–	+	+	–	+	+	–	+
1951	2058	hypothetical protein	18	+	+	+	+	+	+	+	+	+	+	+	+	+	+	+	+	–	–	–	–	–	–	–	–	–	–	–	–	–	–	–	–	–	–	–	–	–	–	–	–	+	–	+	–	–	–	–	–	–	–	–	–	–	–	–	–	–
1952	2176	Type II restriction enzyme MjaIII (EC 3.1.21.4)	18	–	–	–	–	–	–	–	–	–	–	–	–	–	–	–	–	–	–	–	+	+	+	+	+	+	+	+	+	+	+	+	+	+	+	+	–	–	–	–	–	–	–	–	–	–	–	–	–	+	–	–	–	+	–	–	–	–
1953	2180	Helicase loader DnaI	18	–	–	–	–	–	–	–	–	–	–	–	–	–	–	+	–	–	–	+	–	+	+	–	–	–	–	+	+	+	–	–	–	–	–	–	+	+	+	+	+	+	–	+	+	–	–	–	–	+	–	–	+	–	–	+	–	–
1954	2225	Uracil-xanthine permease	18	–	–	–	–	–	–	–	–	–	–	–	–	–	–	–	–	+	+	–	–	–	–	–	–	–	–	–	–	–	–	–	–	–	–	–	–	–	–	+	+	+	–	+	+	+	–	+	+	+	–	+	+	+	+	+	+	+
1955	2232	hypothetical protein	18	–	–	–	–	–	–	–	–	–	–	–	–	–	–	–	+	–	–	–	+	+	+	+	–	+	+	+	+	+	+	+	+	–	–	–	–	–	–	–	–	–	–	–	–	–	–	–	–	–	+	+	+	+	–	+	–	–
1956	2274	Phage integrase	18	–	–	–	–	+	–	–	–	–	–	–	–	–	–	–	–	+	–	–	+	–	–	+	–	–	–	–	+	–	–	+	+	+	+	–	–	–	+	–	–	–	+	–	–	+	–	–	–	–	+	+	+	–	+	+	+	–
1957	2298	hypothetical protein	18	–	–	–	–	–	–	–	–	–	–	–	–	–	–	–	–	–	–	+	–	–	–	–	–	–	–	–	–	–	–	–	–	+	–	+	+	+	+	–	–	–	–	–	+	+	–	+	+	–	+	+	+	+	+	+	+	+
1958	2299	Virulence-associated protein E	18	+	+	+	+	+	+	+	–	+	+	+	–	+	+	–	–	–	–	–	–	–	–	–	–	–	–	–	–	–	–	–	–	–	+	–	+	+	+	–	–	–	–	–	–	–	–	–	–	–	–	–	+	–	–	–	–	+
1959	2300	Phage protein	18	+	+	+	+	+	+	+	–	+	+	+	–	+	+	–	–	–	–	–	–	–	–	–	–	–	–	–	–	–	–	–	–	–	+	–	+	+	+	–	–	–	–	–	–	–	–	–	–	–	–	–	+	–	–	–	–	+

Table S5. Precence/absence of each HG among 57 *S. suis* strains

Index[a]	ClutserID	Gene products	Members	98HAH33	05ZYH33	SC84	A7	SS12	P1/7	DAT273	NIAH11434	JS14	GZ1	BM407	S735	13730	MNCM07	NCTC 10446	14636	NCTC 10237	ST1	2651	89-1591	MNCM04	MNCM25	8074	D9	4961	ST3	MNCM43	DAT292	DAT260	NIAH11435	MNCM21	MNCM50	89-2479	DAT234	DAT290	93A	42A	NT77	6407	11538	8830	12814	92-1400	89-590	2726	2524	89-3576-3	4417	D12	10581	89-5259	88-5299A	R61	92-1191	14A	92-4172	22083
				1																13			25					28						104		94			76			53/54		Divergent *S. suis*																
1960	2301	hypothetical protein	18	+	+	+	+	+	+	+	–	+	+	+	–	+	+	–	–	–	–	–	–	–	–	–	–	–	–	–	–	–	–	–	–	–	+	–	+	+	+	–	–	–	–	–	–	–	–	–	–	–	–	–	+	–	–	–	–	+
1961	2302	hypothetical protein	18	+	+	+	+	+	+	+	–	+	+	+	–	+	+	–	–	–	–	–	–	–	–	–	–	–	–	–	–	–	–	–	–	–	+	–	+	+	+	–	–	–	–	–	–	–	–	–	–	–	–	–	+	–	–	–	–	+
1962	2321	hypothetical protein	18	–	–	–	–	–	–	–	–	–	–	–	–	–	–	+	–	+	+	–	+	+	+	+	+	+	+	+	+	+	+	–	–	–	–	–	–	–	–	–	–	–	–	–	–	+	–	+	+	+	–	–	–	–	–	–	–	–
1963	2335	Type II restriction enzyme MjaIII (EC 3.1.21.4)	18	–	–	–	–	–	–	–	–	–	–	–	–	–	–	–	–	–	–	–	+	+	+	+	+	+	+	+	+	+	+	+	+	+	+	+	–	–	–	–	–	–	–	–	–	–	–	–	–	+	–	–	–	+	–	–	–	–
1964	2351	hypothetical protein	18	+	+	+	+	+	+	+	+	+	+	+	+	+	+	+	+	–	–	+	–	–	–	–	–	–	–	–	–	–	–	–	–	–	–	–	–	–	–	–	–	–	–	–	–	–	–	–	–	–	–	–	+	–	–	–	–	–
1965	2352	hypothetical protein	18	+	+	+	+	+	+	+	–	+	+	+	–	+	+	–	–	–	–	–	–	–	–	–	–	–	–	–	–	–	–	–	–	–	+	–	+	+	+	–	–	–	–	–	–	–	–	–	–	–	–	–	+	–	–	–	–	+
1966	2358	putative plasmid replication protein	18	–	–	–	–	+	–	–	–	–	–	–	–	–	–	–	–	–	–	–	–	+	+	–	+	–	–	–	–	+	+	+	+	+	–	+	+	+	+	–	–	–	–	+	+	+	–	–	–	–	–	–	–	–	+	+	–	–
1967	2385	hypothetical protein	18	–	–	–	–	–	–	–	–	–	–	–	–	–	–	–	–	–	–	–	–	–	–	+	+	+	+	+	+	+	+	+	+	+	+	+	+	+	+	+	+	–	–	–	–	–	–	–	–	–	–	–	–	–	–	–	–	–
1968	2387	L-fuculokinase (EC 2.7.1.51)	18	–	–	–	+	–	–	–	–	–	–	–	–	–	+	–	+	–	–	–	–	–	–	–	–	–	–	+	–	+	+	+	+	+	+	+	+	–	–	+	+	–	–	–	–	–	–	–	–	–	–	–	+	+	+	+	–	–
1969	2388	putative tail protein	18	–	–	–	–	+	–	–	–	–	–	–	–	–	–	–	–	–	–	–	+	+	+	+	–	–	–	+	+	+	+	+	+	–	+	–	–	–	–	+	–	+	–	–	+	–	–	+	–	–	–	–	+	–	–	+	–	–
1970	2392	hypothetical protein	18	+	+	+	+	+	+	+	+	+	+	+	+	+	+	+	+	–	–	–	–	–	–	–	–	–	–	–	–	–	–	–	–	–	–	–	–	–	–	–	–	–	+	–	–	–	+	–	–	–	–	–	–	–	–	–	–	–
1971	2400	hypothetical protein	18	+	+	+	+	+	+	+	+	+	+	+	+	+	+	+	–	–	–	–	–	–	–	–	–	–	–	–	–	–	–	–	–	–	–	–	–	–	–	–	–	+	+	–	+	–	–	–	–	–	–	–	–	–	–	–	–	–
1972	2407	hypothetical protein	18	+	+	+	+	+	+	+	+	+	+	+	+	+	+	–	–	–	–	–	–	–	–	–	–	–	–	–	–	–	–	–	–	+	+	–	–	+	+	–	–	–	–	–	–	–	–	–	–	–	–	–	–	–	–	–	–	–
1973	1788	Integrase/recombinase, phage associated	17	–	–	–	–	–	–	–	–	–	–	–	–	–	–	+	–	–	–	+	–	+	+	+	–	–	–	–	–	–	–	+	+	–	+	+	+	+	–	–	–	+	–	+	+	–	–	–	–	–	–	–	+	–	+	+	–	–
1974	1798	Ribosyl nicotinamide transporter, PnuC-like	17	+	+	+	+	+	+	+	+	+	+	+	+	+	+	–	–	+	+	+	–	–	–	–	–	–	–	–	–	–	–	–	–	–	–	–	–	–	–	–	–	–	–	–	–	–	–	–	–	–	–	–	–	–	–	–	–	–
1975	1899	protein of unknown function	17	–	–	–	–	–	–	–	–	–	–	–	–	–	–	–	+	–	–	–	+	+	+	+	+	+	–	+	+	+	+	–	–	–	–	–	–	–	–	–	–	+	+	+	–	–	–	+	+	+	–	–	–	–	–	–	–	–
1976	1916	Glyoxalase family protein	17	–	–	–	–	–	–	–	–	–	–	–	–	–	–	–	–	+	+	–	–	–	–	–	–	–	–	–	–	–	–	–	–	–	+	–	+	+	+	–	–	–	–	–	–	+	–	+	–	+	+	+	+	+	+	+	+	+
1977	1951	Nicotinamide-nucleotide adenylyltransferase, NadR family (EC 2.7.7.1) / Ribosylnicotinamide kinase (EC 2.7.1.22)	17	+	+	+	+	+	+	+	+	+	+	+	+	+	+	–	–	+	+	+	–	–	–	–	–	–	–	–	–	–	–	–	–	–	–	–	–	–	–	–	–	–	–	–	–	–	–	–	–	–	–	–	–	–	–	–	–	–
1978	1998	Nudix-related transcriptional regulator NrtR	17	+	+	+	+	+	+	+	+	+	+	+	+	+	+	–	–	+	+	+	–	–	–	–	–	–	–	–	–	–	–	–	–	–	–	–	–	–	–	–	–	–	–	–	–	–	–	–	–	–	–	–	–	–	–	–	–	–
1979	2002	hypothetical protein	17	–	–	–	–	–	–	–	–	–	–	–	–	–	–	–	–	+	+	–	–	–	–	–	–	–	–	–	–	–	–	–	–	–	–	–	–	–	–	–	–	+	+	+	+	+	+	+	+	+	+	+	+	+	+	+	–	–
1980	2052	transcriptional regulator, putative	17	–	–	–	–	–	–	–	–	–	–	–	–	–	–	–	–	–	–	–	+	+	+	–	–	–	–	–	–	–	–	+	+	+	–	–	–	+	+	+	+	+	+	+	+	+	–	–	–	+	–	–	+	–	–	–	–	–
1981	2227	hypothetical protein	17	–	–	–	–	–	–	–	–	–	–	–	–	–	–	+	–	+	–	+	+	+	+	–	–	–	–	–	+	–	–	–	–	+	+	–	+	+	+	–	+	–	–	–	+	–	–	–	–	–	–	–	–	–	+	+	–	+
1982	2230	Secreted antigen GbpB/SagA/PcsB, putative peptidoglycan hydrolase	17	–	–	–	–	–	–	–	–	–	–	–	–	–	–	–	+	+	+	–	–	–	–	–	–	+	+	+	+	+	+	+	+	–	–	–	–	–	–	–	–	–	+	–	–	+	+	+	+	+	–	–	–	–	–	–	–	–
1983	2297	hypothetical protein	17	+	+	+	+	+	+	+	+	+	+	+	+	+	+	+	+	–	–	+	–	–	–	–	–	–	–	–	–	–	–	–	–	–	–	–	–	–	–	–	–	–	–	–	–	–	–	–	–	–	–	–	–	–	–	–	–	–
1984	2371	hypothetical protein	17	+	+	+	+	+	+	+	+	+	+	+	+	+	+	+	–	+	+	–	–	–	–	–	–	–	–	–	–	–	–	–	–	–	–	–	–	–	–	–	–	–	–	–	–	–	–	–	–	–	–	–	–	–	–	–	–	–
1985	2374	hypothetical protein	17	+	+	+	+	+	+	+	+	+	+	+	+	+	+	–	–	+	+	–	–	–	–	–	–	–	–	–	–	–	–	–	–	–	–	–	–	–	–	–	–	–	–	–	–	–	–	–	–	–	–	+	–	–	–	–	–	–
1986	2381	hypothetical protein	17	–	–	–	–	–	–	–	–	–	–	–	–	–	–	+	–	–	–	–	+	+	+	+	+	+	+	+	+	+	+	–	–	–	–	–	–	–	–	–	–	–	–	–	+	–	+	–	–	–	+	–	–	+	–	+	–	–
1987	2393	integrase family protein	17	+	+	+	+	+	–	–	–	+	–	–	–	–	+	–	–	–	–	–	+	+	+	+	+	–	–	–	–	–	–	–	–	–	–	–	–	–	–	–	–	+	–	+	+	+	–	–	+	–	–	–	–	–	–	–	–	–
1988	2394	Type I restriction-modification system, specificity subunit S (EC 3.1.21.3)	17	+	+	+	+	+	+	+	+	+	+	+	+	+	+	+	+	–	–	–	–	–	–	–	–	–	–	–	–	–	–	–	–	–	–	–	–	–	–	–	–	–	–	–	–	–	–	–	–	–	–	–	+	–	–	–	–	–
1989	2404	hypothetical protein	17	+	+	+	–	+	+	+	+	+	+	+	+	+	+	+	–	–	–	–	–	–	–	–	–	–	–	–	–	–	–	–	+	–	–	–	–	–	–	–	–	+	–	–	–	–	–	–	–	–	+	–	–	–	–	–	–	–
1990	2432	Methyl-accepting chemotaxis protein	17	+	+	+	+	+	+	+	+	+	+	+	+	+	+	–	+	–	–	+	–	–	–	–	–	–	–	–	–	–	–	–	–	–	–	–	–	–	–	–	–	–	–	–	–	–	+	–	–	–	–	–	–	–	–	–	–	–
1991	2437	hypothetical protein	17	+	+	+	+	+	+	+	+	+	+	+	+	+	+	–	–	+	+	–	–	–	–	–	–	–	–	–	–	–	–	–	–	–	–	–	–	–	–	–	–	–	–	–	+	–	–	–	–	–	–	–	–	–	–	–
1992	2440	Acylneuraminate cytidylyltransferase(EC:2.7.7.43)	17	+	+	+	+	+	–	+	+	+	+	+	–	+	–	–	–	+	–	–	+	+	+	–	–	–	–	+	–	–	+	–	–	–	–	–	–	–	–	–	–	–	–	–	–	–	–	–	–	–	–	–	–	–	–	–	–	–
1993	2443	hypothetical protein	17	–	–	–	–	–	–	–	–	–	–	–	–	–	–	–	+	–	–	–	+	+	+	+	+	+	–	+	+	+	+	–	–	–	–	–	–	–	–	–	–	+	+	+	–	–	–	+	+	+	–	–	–	–	–	–	–	–
1994	2447	hypothetical protein	17	–	–	–	–	–	–	–	–	–	–	–	–	–	–	–	+	–	–	–	+	+	+	+	+	+	–	+	+	+	+	–	–	–	–	–	–	–	–	–	–	+	+	+	–	–	–	+	+	+	–	–	–	–	–	–	–	–
1995	2448	Pyridoxamine 5'-phosphate oxidase-related	17	+	+	+	+	+	+	+	+	+	+	+	+	+	+	–	–	–	–	–	–	–	–	–	–	–	–	–	–	–	–	–	–	–	–	–	–	–	–	+	+	–	–	–	–	–	–	–	–	–	–	–	–	–	–	+	–	–	–	–
1996	2450	hypothetical protein	17	+	+	+	+	+	+	+	+	–	+	+	+	+	+	–	–	–	–	–	–	–	–	–	–	–	–	–	–	–	–	–	–	–	–	–	–	–	–	+	+	+	–	–	–	–	+	–	–	–	–	–	–	–	–	–	–	–
1997	2451	Uncharacterized membrane protein, possible Na+ channel or pump	17	+	+	+	+	–	+	+	+	+	+	+	+	+	+	+	–	–	–	+	–	–	–	–	–	–	–	–	–	–	–	–	–	–	–	–	–	–	–	+	+	–	–	–	–	–	–	–	–	–	–	–	–	–	–	–	–	–
1998	2453	hypothetical protein	17	+	+	+	+	–	–	+	+	+	+	+	+	+	+	–	–	–	–	–	–	–	–	–	–	–	–	–	–	–	–	–	–	–	–	–	–	–	–	–	–	+	–	–	+	–	+	+	–	+	–	–	–	–	–	–	–	–
1999	2478	transcriptional regulator PlcR, putative	17	–	–	+	+	+	+	+	+	+	+	+	+	+	+	+	–	–	+	+	–	–	–	–	–	–	–	–	–	–	–	–	–	–	–	–	–	–	+	–	–	–	–	–	–	–	–	–	–	–	+	–	–	–	–	–	–	–
2000	10	Mobile element protein	16	–	–	–	–	–	–	–	–	–	–	–	–	–	–	–	–	–	–	–	+	–	–	–	+	–	+	–	–	+	–	+	+	–	+	+	+	–	+	–	–	–	–	–	+	+	+	+	+	+	–	–	–	–	–	–	–	–
2001	1987	Glucuronide permease	16	+	+	–	–	–	–	–	–	–	–	–	–	–	–	–	–	–	–	+	–	+	+	–	–	–	–	–	+	+	–	–	–	+	+	+	+	–	–	–	–	–	–	–	–	+	–	–	+	+	+	–	–	–	–	+	–	–
2002	2011	Phage holin	16	–	–	–	–	+	–	–	–	–	–	–	–	–	–	–	–	–	–	–	+	+	+	+	–	–	–	–	+	–	–	+	+	+	+	–	+	+	–	–	–	+	–	–	–	–	–	+	–	–	+	–	+	–	–	–	–	–
2003	2142	Conserved uncharacterized protein	16	–	–	–	–	–	–	–	–	–	–	–	–	–	–	–	+	–	–	–	–	+	+	+	+	+	–	+	+	+	+	–	–	–	–	–	–	–	–	–	–	+	+	+	–	–	–	+	+	+	–	–	–	–	–	–	–	–
2004	2191	tRNA-dependent lipid II-Ala--L-alanine ligase	16	–	–	–	–	–	–	–	–	–	–	–	–	–	–	+	+	–	–	–	–	–	–	–	–	–	–	–	–	–	–	–	–	+	+	+	–	+	+	–	–	–	–	+	+	–	–	–	+	+	+	+	+	+	–	+	–	–
2005	2330	kilA protein, putative phage-related DNA binding protein	16	–	–	–	–	–	–	–	–	–	–	–	–	–	–	–	–	–	–	–	–	+	+	+	+	+	+	+	+	+	+	+	+	+	+	–	–	–	–	–	–	–	+	+	–	–	–	–	–	–	–	–	+	–	–	–	–	–
2006	2356	Type I restriction-modification system, DNA-methyltransferase subunit M (EC 2.1.1.72)	16	+	+	+	+	+	+	+	+	+	+	+	+	+	+	–	–	–	–	–	–	–	–	–	–	–	–	–	–	–	–	–	–	–	+	+	–	–	–	–	–	–	–	–	–	–	–	–	–	–	–	–	–	–	–	–	–	–
2007	2357	Type I restriction-modification system, restriction subunit R (EC 3.1.21.3)	16	+	+	+	+	+	+	+	+	+	+	+	+	+	+	–	–	–	–	–	–	–	–	–	–	–	–	–	–	–	–	–	–	–	+	+	–	–	–	–	–	–	–	–	–	–	–	–	–	–	–	–	–	–	–	–	–	–
2008	2375	hypothetical protein	16	–	–	–	–	–	–	–	–	–	–	–	–	–	–	–	–	–	–	–	–	+	+	+	+	+	+	+	+	+	+	+	+	+	–	+	–	–	–	–	–	–	–	–	+	–	–	–	–	+	–	–	–	–	–	–	–	–
2009	2390	Retron-type RNA-directed DNA polymerase (EC 2.7.7.49)	16	–	–	–	–	–	–	–	–	–	–	–	–	–	–	–	+	–	–	–	–	+	+	–	–	–	–	+	–	–	+	+	+	–	–	–	–	–	–	+	+	–	+	–	–	–	–	–	+	–	–	+	+	–	+	+	+	–
2010	2435	hypothetical protein	16	–	–	–	–	–	–	–	–	–	–	–	–	–	–	+	+	+	+	–	–	–	–	–	–	+	+	+	+	+	+	–	–	–	–	–	–	–	–	+	+	–	–	+	+	+	–	–	–	+	–	–	–	–	–	–	–	–
2011	2441	hypothetical protein	16	–	–	–	–	–	–	–	–	–	–	–	–	–	–	–	–	–	–	–	+	+	+	+	+	+	+	+	+	+	+	+	+	–	–	–	–	–	–	–	–	+	–	–	–	–	–	+	+	–	–	–	–	–	–	–	–	–
2012	2444	hypothetical protein	16	–	–	–	–	–	–	–	–	–	–	–	–	–	–	–	–	–	–	–	+	+	+	+	+	+	+	+	+	+	+	+	+	–	–	–	–	–	–	–	–	+	–	–	–	–	–	+	+	–	–	–	–	–	–	–	–	–
2013	2446	hypothetical protein	16	–	–	–	–	–	–	–	–	–	–	–	–	–	–	–	–	–	–	–	+	–	–	+	+	+	+	+	+	+	+	+	+	–	–	–	–	–	–	–	–	–	–	–	–	–	–	–	–	–	–	–	+	+	–	+	+	+
2014	2452	hypothetical protein	16	–	–	–	–	–	–	–	–	–	–	–	–	–	–	–	–	–	–	–	+	+	+	+	+	–	–	+	+	+	+	–	–	–	+	–	–	–	–	+	+	+	+	+	–	–	–	–	–	–	–	–	–	–	+	–	–	–
2015	2517	Acetyltransferase	16	+	+	+	+	+	+	+	+	+	+	+	+	+	+	–	–	+	+	–	–	–	–	–	–	–	–	–	–	–	–	–	–	–	–	–	–	–	–	–	–	–	–	–	–	–	–	–	–	–	–	–	–	–	–	–	–	–
2016	2519	hypothetical protein	16	–	–	–	–	–	–	+	–	–	–	–	–	–	–	–	–	+	+	–	–	–	–	–	–	–	–	–	–	–	–	–	–	–	–	–	–	–	–	–	–	–	–	–	–	+	+	+	+	+	+	+	+	+	+	+	+	+
2017	2522	hypothetical protein	16	+	+	+	+	+	+	+	+	+	–	+	+	–	+	–	–	+	–	+	–	–	–	–	–	–	–	–	–	–	–	–	–	–	–	–	–	–	–	–	–	–	–	+	–	–	–	–	–	–	–	+	–	–	–	–	–	–
2018	2525	Transcription accessory protein (S1 RNA-binding domain)	16	+	+	+	+	+	+	+	+	+	+	+	+	+	+	–	–	+	+	–	–	–	–	–	–	–	–	–	–	–	–	–	–	–	–	–	–	–	–	–	–	–	–	–	–	–	–	–	–	–	–	–	–	–	–	–	–	–
2019	2528	Helix-turn-helix motif:Protein of unknown function DUF955	16	–	–	–	–	–	–	–	–	–	–	–	–	–	–	–	–	–	–	–	+	+	+	+	+	+	+	+	+	+	+	+	+	–	–	–	–	–	–	–	–	–	–	–	–	–	+	–	–	–	+	–	–	–	+	–	–	–
2020	2531	hypothetical protein	16	–	–	–	–	–	–	–	–	–	–	–	–	–	–	–	–	–	–	–	+	+	+	+	+	+	+	+	+	+	+	+	+	–	–	–	–	–	–	–	–	–	–	–	–	–	+	–	–	–	+	–	–	–	+	–	–	–
2021	2534	DNA-binding protein	16	+	+	+	+	+	+	+	+	+	+	+	+	+	+	–	–	–	–	–	–	–	–	–	–	–	–	–	–	–	–	–	–	–	–	–	–	–	–	+	+	–	–	–	–	–	–	–	–	–	–	–	–	–	–	–	–	–
2022	2537	LPXTG-motif cell wall anchor domain protein	16	+	+	+	+	–	+	+	+	+	+	+	+	+	+	+	–	–	–	+	–	–	–	–	–	–	–	–	–	–	–	–	–	–	–	–	–	–	–	–	–	–	–	–	+	–	–	–	–	–	–	–	–	–	–	–	–	–
2023	2538	Muramidase-released protein precursor (136 kDa surface protein)	16	–	–	–	–	–	–	–	–	+	–	–	–	–	–	+	–	–	–	–	–	+	+	+	+	–	+	–	–	–	–	–	–	–	–	–	–	–	–	+	–	+	+	+	–	–	–	–	–	–	+	+	+	–	–	+	+	–
2024	2540	Fucolectin-related protein	16	–	–	–	+	–	–	–	–	–	–	–	–	–	+	–	+	–	–	–	–	–	–	–	–	–	–	+	–	+	+	–	+	+	+	+	+	–	+	+	+	–	–	–	–	–	–	–	–	–	–	–	–	–	+	+	–	–
2025	2544	acetyltransferase, GNAT family	16	–	–	–	–	–	–	–	–	–	–	–	–	–	–	–	–	–	–	+	–	–	–	–	–	–	–	–	–	–	–	+	+	+	+	+	+	+	+	–	–	–	–	+	–	+	+	+	+	+	–	–	–	–	–	–	–	+
2026	2547	hypothetical protein	16	+	+	+	+	+	+	+	+	+	+	+	+	+	+	+	+	–	–	–	–	–	–	–	–	–	–	–	–	–	–	–	–	–	–	–	–	–	–	–	–	–	–	–	–	–	–	–	–	–	–	–	–	–	–	–	–	–
2027	2549	hypothetical protein	16	+	+	+	+	+	+	+	+	+	+	+	+	+	+	+	+	–	–	–	–	–	–	–	–	–	–	–	–	–	–	–	–	–	–	–	–	–	–	–	–	–	–	–	–	–	–	–	–	–	–	–	–	–	–	–	–	–
2028	2550	hypothetical protein	16	+	+	+	+	+	+	+	+	+	+	+	+	+	+	+	+	–	–	–	–	–	–	–	–	–	–	–	–	–	–	–	–	–	–	–	–	–	–	–	–	–	–	–	–	–	–	–	–	–	–	–	–	–	–	–	–	–
2029	2551	hypothetical protein	16	+	+	+	+	+	+	+	+	+	+	+	+	+	+	+	+	–	–	–	–	–	–	–	–	–	–	–	–	–	–	–	–	–	–	–	–	–	–	–	–	–	–	–	–	–	–	–	–	–	–	–	–	–	–	–	–	–
2030	2552	hypothetical protein	16	+	+	+	+	+	+	+	+	+	+	+	+	+	–	–	+	–	–	–	–	–	–	–	–	–	–	–	–	–	–	–	–	–	–	–	–	–	–	–	–	–	+	–	–	–	–	+	–	–	–	–	–	–	–	–	–	–
2031	2571	hypothetical protein	16	+	+	+	+	+	+	+	+	+	+	+	+	+	+	+	–	–	–	–	–	–	–	–	–	–	–	–	–	–	–	–	–	–	–	–	–	–	–	–	–	–	–	–	–	–	–	–	–	–	–	–	+	–	–	–	–	–
2032	2572	hypothetical protein	16	+	+	+	+	+	+	+	+	+	+	+	+	+	+	+	–	–	–	–	–	–	–	–	–	–	–	–	–	–	–	–	–	–	–	–	–	–	–	–	–	–	–	+	–	–	–	–	–	–	–	–	–	–	–	–	–	–
2033	2573	hypothetical protein	16	+	+	+	+	+	+	+	+	+	+	+	+	+	+	+	–	–	–	–	–	–	–	–	–	–	–	–	–	–	–	–	–	–	–	–	–	–	–	–	–	–	–	+	–	–	–	–	–	–	–	–	–	–	–	–	–	–
2034	909	IS861, transposase (orf1), IS3 family, truncated	15	–	–	–	–	–	–	–	–	–	–	–	–	–	–	–	–	–	–	–	+	–	–	–	+	–	+	–	+	+	+	–	–	–	–	–	+	+	+	–	–	–	–	–	+	+	+	+	+	+	–	–	–	–	–	–	–	–
2035	1609	Tryptophan synthase beta chain (EC 4.2.1.20)	15	–	–	–	–	–	–	–	–	–	–	–	–	–	–	–	+	–	–	–	–	–	–	–	–	–	–	–	–	–	–	–	–	–	–	–	+	+	–	–	–	+	+	–	–	+	+	+	–	+	–	+	+	+	+	+	–	+
2036	2147	Acetyltransferase (EC 2.3.1.-)	15	–	–	–	–	–	–	–	–	–	+	–	–	–	–	–	–	–	–	+	–	–	–	–	–	–	–	–	–	–	–	–	–	–	–	–	+	+	–	–	–	–	+	+	+	–	+	–	–	–	+	+	+	+	+	+	–	+
2037	2196	Site-specific recombinase	15	–	–	–	–	–	–	–	+	+	–	–	–	–	–	–	–	–	–	–	+	+	+	–	–	–	–	+	+	–	+	+	+	+	–	+	–	–	–	–	–	–	–	–	–	–	–	–	–	+	+	–	–	–	+	–	–	–
2038	2327	Beta-hexosaminidase (EC 3.2.1.52)	15	–	–	–	–	–	–	–	–	–	–	–	–	–	–	–	–	+	+	–	–	–	–	–	–	–	–	–	–	–	–	–	–	–	+	–	+	+	+	–	–	–	–	–	–	+	–	+	–	+	+	–	+	+	–	+	+	+
2039	2334	putative transposase	15	–	–	–	–	–	–	–	–	–	–	–	–	–	–	–	–	–	–	–	+	+	+	+	+	+	–	–	–	–	–	–	–	–	+	+	–	–	–	+	–	–	+	–	+	+	–	–	–	–	+	–	+	+	–	–	–	–
2040	2372	Phage protein	15	–	–	–	–	+	–	–	–	–	–	–	–	–	–	–	+	+	+	–	+	+	+	+	–	+	–	–	+	–	–	–	–	+	–	–	–	–	–	+	–	–	–	–	+	–	–	–	–	–	–	–	+	–	–	+	–	–
2041	2438	3,4-dihydroxy-2-butanone 4-phosphate synthase (EC 4.1.99.12) / GTP cyclohydrolase II (EC 3.5.4.25)	15	–	–	–	–	–	–	–	–	–	–	–	–	–	–	–	–	+	+	–	–	–	–	–	–	–	–	–	–	–	–	–	–	–	+	–	+	+	+	–	–	–	–	–	–	+	–	+	–	+	+	+	+	+	+	+	–	–
2042	2527	hypothetical protein	15	–	–	–	–	–	–	–	–	–	–	–	–	–	–	–	–	–	–	–	+	+	+	+	+	+	+	+	+	+	+	+	+	–	–	–	–	–	–	–	–	–	–	–	+	–	+	–	–	–	–	–	–	–	–	–	–	–
2043	2529	hypothetical protein	15	–	–	–	–	–	–	–	–	–	–	–	–	–	–	+	–	–	–	–	–	–	–	+	+	+	+	+	+	+	+	–	–	–	–	–	–	–	–	+	+	+	–	–	–	+	–	–	–	–	+	–	–	+	–	–
2044	2530	hypothetical protein	15	–	–	–	–	–	–	–	–	–	–	–	–	–	–	–	–	–	–	–	+	+	+	+	+	+	+	+	+	+	+	+	+	–	–	–	–	–	–	–	–	–	–	–	+	–	+	–	–	–	–	–	–	–	–	–	–	–
2045	2532	hypothetical protein	15	–	–	–	–	–	–	–	–	–	–	–	–	–	–	–	–	–	–	–	+	+	+	–	–	+	+	+	+	+	+	+	+	+	+	+	–	–	–	–	–	–	–	–	–	–	–	–	–	–	–	–	–	–	–	–	–	+
2046	2533	hypothetical protein	15	–	–	–	–	+	–	–	–	–	–	–	–	–	–	–	–	–	–	–	+	–	–	–	–	+	–	–	–	–	–	+	+	–	+	+	+	+	+	–	–	–	–	–	–	–	–	–	–	–	–	+	–	+	–	+	+	+
2047	2546	transposase	15	–	–	–	–	+	–	–	+	–	+	–	+	+	–	+	–	–	–	–	+	–	–	+	+	–	+	+	–	–	+	–	–	–	–	–	–	–	–	–	–	–	–	+	–	+	–	–	+	–	–	–	–	–	–	–	–	–
2048	2553	hypothetical protein	15	+	+	+	+	+	+	+	–	+	+	+	–	–	+	–	–	–	–	–	–	–	–	–	–	–	–	–	–	–	–	–	–	–	+	–	–	–	+	–	–	–	–	–	–	–	–	–	–	–	–	–	+	–	–	–	–	+
2049	2554	hypothetical protein	15	+	+	+	+	+	+	+	–	+	+	+	–	+	+	–	–	–	–	–	–	–	–	–	–	–	–	–	–	–	–	–	–	–	+	–	–	–	+	–	–	–	–	–	–	–	–	–	–	–	–	–	–	–	–	–	–	+
2050	2570	Type I restriction-modification system, specificity subunit S (EC 3.1.21.3)	15	+	+	+	+	–	+	+	+	+	+	+	+	+	+	–	–	–	–	–	–	–	–	–	–	–	–	–	–	–	–	–	–	–	+	+	–	–	–	–	–	–	–	–	–	–	–	–	–	–	–	–	–	–	–	–	–	–
2051	2580	hypothetical protein	15	+	+	+	+	+	+	+	–	+	+	+	–	+	+	–	–	–	–	–	–	–	–	–	–	–	–	–	–	–	–	–	–	–	+	–	+	–	–	–	–	–	–	–	–	–	–	–	–	–	–	–	+	–	–	–	–	–
2052	2616	Thioredoxin	15	–	–	–	–	–	–	–	–	–	–	–	–	–	–	–	–	+	+	–	–	–	–	–	–	–	–	–	–	–	–	–	–	–	+	–	+	+	+	–	–	–	–	–	–	+	–	+	–	+	+	+	+	+	+	+	–	–
2053	2633	hypothetical protein	15	+	+	+	+	+	+	+	+	+	–	+	+	+	+	–	–	–	–	–	–	–	–	–	–	–	–	–	–	–	–	–	–	–	–	–	–	–	–	+	+	–	–	–	–	–	–	–	–	–	–	–	–	–	–	–
2054	2637	hypothetical protein	15	+	+	+	+	–	+	+	+	+	+	+	+	+	+	–	–	–	–	–	–	–	–	–	–	–	–	–	–	–	–	–	–	–	–	–	–	–	–	+	+	–	–	–	–	–	–	–	–	–	–	–	–	–	–	–
2055	2644	hypothetical protein	15	+	+	+	+	+	+	+	–	+	+	+	–	+	+	–	–	–	–	–	–	–	–	–	–	–	–	–	–	–	–	–	–	–	+	–	–	–	+	–	–	–	–	–	–	–	–	–	–	–	–	–	–	–	–	–	–	+
2056	2659	hypothetical protein	15	+	+	+	+	+	+	+	+	+	+	–	+	+	+	+	–	–	–	–	–	–	–	–	–	–	–	–	–	–	–	–	–	–	–	–	–	–	–	–	–	–	–	–	–	–	–	–	+	–	–	–	–	–	–	–	–	–
2057	2660	Type III restriction-modification system methylation subunit (EC 2.1.1.72)	15	+	+	+	+	+	+	+	+	+	+	+	+	+	+	–	–	–	–	+	–	–	–	–	–	–	–	–	–	–	–	–	–	–	–	–	–	–	–	–	–	–	–	–	–	–	–	–	–	–	–	–	–	–	–	–	–	–
2058	2662	hypothetical protein	15	+	+	+	+	+	+	+	+	+	+	+	+	+	+	+	–	–	–	–	–	–	–	–	–	–	–	–	–	–	–	–	–	–	–	–	–	–	–	–	–	–	–	–	–	–	–	–	–	–	–	–	–	–	–	–	–	–
2059	2664	hypothetical protein	15	+	+	+	+	+	+	+	–	+	+	+	–	+	+	–	–	–	–	–	–	–	–	–	–	–	–	–	–	–	–	–	–	–	+	–	+	+	–	–	–	–	–	–	–	–	–	–	–	–	–	–	–	–	–	–	–	–
2060	2665	metallo cofactor biosynthesis protein	15	+	+	+	+	+	+	+	+	+	+	+	+	+	+	+	–	–	–	–	–	–	–	–	–	–	–	–	–	–	–	–	–	–	–	–	–	–	–	–	–	–	–	–	–	–	–	–	–	–	–	–	–	–	–	–	–	–
2061	2666	Transcriptional regulator, XRE family	15	+	+	+	+	+	+	+	–	+	+	+	–	+	+	–	–	–	–	–	–	–	–	–	–	–	–	–	–	–	–	–	–	–	+	–	+	+	–	–	–	–	–	–	–	–	–	–	–	–	–	–	–	–	–	–	–	–
2062	1624	Non-phosphorylating glyceraldehyde-3-phosphate dehydrogenase (NADP) (EC 1.2.1.9)	14	–	–	–	–	–	–	–	–	–	–	–	–	–	–	–	–	–	–	+	–	–	–	–	–	–	–	–	–	–	–	–	–	–	–	–	–	–	–	+	+	–	–	–	+	+	+	–	–	+	+	+	+	+	+	+	–	+
2063	1791	Cadmium resistance protein	14	–	–	–	–	–	–	–	–	–	–	–	–	–	–	–	–	–	–	–	–	–	–	–	–	–	–	–	–	–	–	+	+	+	–	–	–	–	+	–	–	+	+	+	+	–	–	–	+	–	+	+	–	–	+	+	+	–
2064	1792	Cadmium efflux system accessory protein	14	–	–	–	–	–	–	–	–	–	–	–	–	–	–	–	–	–	–	–	–	–	–	–	–	–	–	–	–	–	–	+	+	+	–	–	–	–	+	–	–	+	+	+	+	–	–	–	+	–	+	+	–	–	+	+	+	–
2065	1957	UDP-glucose dehydrogenase in hyaluronic acid synthesis (EC 1.1.1.22)	14	–	–	–	–	–	–	–	–	–	–	–	–	–	–	–	–	–	–	–	–	–	–	+	+	+	+	–	–	–	–	–	–	+	–	+	+	+	+	+	+	–	–	–	–	–	–	–	–	–	+	–	+	+	–	–	–	–
2066	2080	Transcriptional regulator, Cro/CI family	14	+	+	+	–	–	–	–	–	–	–	+	–	–	–	–	–	–	–	–	–	–	–	+	–	–	–	–	–	–	–	–	–	+	+	+	–	–	+	+	+	–	–	+	–	–	–	–	+	–	+	–	–	–	–	–	–	–
2067	2177	phage encoded transcriptional regulator, ArpU family	14	–	–	–	–	–	–	–	–	–	–	–	–	–	–	–	–	–	–	+	+	+	+	–	–	+	–	–	+	–	–	–	–	+	–	–	–	–	+	+	–	–	–	–	+	–	–	–	–	–	+	+	–	–	–	+	–	+
2068	2178	Glycosyl transferase, family 2	14	–	–	–	–	–	–	–	–	–	–	–	–	–	–	–	–	–	–	–	+	–	–	+	+	+	+	–	–	–	–	–	–	–	–	–	–	–	–	–	–	+	+	+	+	+	+	+	+	+	–	–	–	–	–	–	–	–
2069	2195	hypothetical protein	14	–	–	–	–	–	–	+	+	+	–	–	–	–	–	–	–	–	–	–	–	–	–	–	–	–	–	+	+	–	+	+	+	–	–	–	–	–	–	–	–	–	+	–	–	–	–	–	–	–	+	+	+	+	–	–	–	–
2070	2234	putative transmembrane protein	14	–	–	–	–	–	–	–	–	–	–	–	–	–	–	–	–	–	–	–	+	–	–	+	+	+	+	–	–	–	–	–	–	–	–	–	–	–	–	–	–	+	+	+	+	+	+	+	+	+	–	–	–	–	–	–	–	–
2071	2279	Protein of unknown function DUF955	14	–	–	–	–	–	–	–	–	–	–	–	–	–	–	–	–	+	+	–	+	+	+	–	–	+	+	+	+	+	+	–	–	–	–	–	–	–	–	+	–	–	–	–	–	–	–	–	–	–	–	–	–	+	–	–	+
2072	2283	DNA/RNA helicase of DEAD/DEAH box family	14	–	–	–	–	–	–	–	–	–	–	–	–	–	–	–	–	–	–	–	+	+	+	+	+	+	+	+	+	+	+	+	+	–	–	–	–	–	–	–	–	–	–	–	–	–	–	–	–	–	–	–	–	–	–	–	+	–
2073	2286	Mutator mutT protein (7,8-dihydro-8-oxoguanine-triphosphatase) (EC 3.6.1.-)	14	–	–	–	–	–	–	–	–	–	–	–	–	–	–	–	–	–	–	–	+	+	+	+	+	+	+	+	+	+	+	+	+	–	–	–	–	–	–	–	–	–	–	–	–	–	–	–	–	–	–	–	–	–	–	–	+	–
2074	2332	Sortase A, LPXTG specific	14	–	–	–	–	–	–	–	–	–	–	–	–	–	–	–	–	–	–	–	+	+	+	–	–	+	+	+	+	+	+	+	+	–	–	–	–	–	–	–	–	+	+	+	–	–	–	–	–	–	–	–	–	–	–	–	–	–
2075	2336	hypothetical protein	14	–	–	–	–	–	–	–	–	–	–	–	–	–	–	–	–	–	–	–	+	+	+	+	–	+	–	–	+	–	–	+	+	+	–	+	–	–	+	+	+	–	–	–	–	–	–	–	–	–	–	–	–	–	–	–	+	–
2076	2354	Lantibiotic transport permease protein	14	–	–	–	–	–	–	+	+	+	–	–	–	–	–	–	–	–	–	–	–	–	–	–	–	–	–	+	+	–	+	+	+	–	–	–	–	–	–	–	–	–	+	+	–	–	–	–	–	–	+	+	+	+	–	–	–	–
2077	2379	Cell wall surface anchor family protein	14	–	–	–	–	–	–	–	–	–	–	–	–	–	–	–	–	–	–	–	+	+	+	–	–	+	+	+	+	+	+	+	+	–	–	–	–	–	–	–	–	+	+	+	–	–	–	–	–	–	–	–	–	–	–	–	–	–
2078	2408	hypothetical protein	14	+	+	+	+	+	+	+	+	+	+	+	+	+	+	–	–	–	–	–	–	–	–	–	–	–	–	–	–	–	–	–	–	–	–	–	–	–	–	–	–	–	–	–	–	–	–	–	–	–	–	–	–	–	–	–	–	–
2079	2536	phage encoded transcriptional regulator	14	–	–	–	–	–	–	–	–	–	–	–	–	–	–	–	–	–	–	–	–	+	+	+	–	–	–	+	+	+	+	+	+	–	–	+	–	–	–	+	+	–	+	–	+	–	–	–	–	–	–	–	–	–	–	–	–	–
2080	2610	hypothetical protein	14	–	–	–	–	–	–	–	–	–	–	–	–	–	–	+	–	+	+	–	–	–	–	+	–	+	+	+	–	–	+	–	–	–	–	–	–	–	–	+	+	–	–	+	–	+	–	–	+	+	–	–	–	–	–	–	–	–
2081	2612	multidrug ABC transporter, ATP-binding protein	14	–	–	–	–	–	–	–	–	–	–	–	–	–	–	–	–	+	+	–	–	+	+	+	+	–	–	–	–	–	–	–	–	–	–	–	–	+	–	–	–	–	–	–	–	–	–	–	–	+	+	+	+	–	+	+	+	–
2082	2623	hypothetical protein	14	–	–	–	–	–	–	–	–	–	–	–	–	–	–	–	–	–	–	–	–	–	–	+	+	+	+	+	+	+	+	–	–	–	–	–	–	–	+	–	–	+	–	–	–	+	–	–	–	–	+	–	+	–	+	–	–	–
2083	2625	hypothetical protein	14	–	–	–	–	–	–	–	–	–	–	–	–	–	–	–	–	–	–	–	+	+	+	+	+	+	+	+	+	+	+	+	+	–	–	–	–	–	–	–	–	–	–	+	–	–	–	–	–	–	–	–	–	–	–	–	–	–
2084	2630	Lj965 prophage protein	14	–	–	–	–	+	–	–	–	–	–	–	–	–	–	–	–	–	–	+	+	+	+	+	–	–	–	–	+	–	–	–	–	+	–	–	–	–	+	+	–	–	–	–	–	–	–	–	–	–	–	+	–	+	+	–	+	–
2085	2631	Phage portal	14	–	–	–	–	+	–	–	–	–	–	–	–	–	–	–	–	–	–	+	+	+	+	+	–	–	–	–	+	–	–	–	–	+	–	–	–	–	+	+	–	–	–	–	–	–	–	–	–	–	–	+	–	+	+	–	+	–
2086	2632	putative major tail protein	14	–	–	–	–	+	–	–	–	–	–	–	–	–	–	–	–	–	–	+	+	+	+	+	–	–	–	–	+	–	–	–	–	+	–	–	–	–	+	+	–	–	–	–	–	–	–	–	–	–	–	+	–	+	+	–	+	–
2087	2634	Phage capsid and scaffold	14	–	–	–	–	+	–	–	–	–	–	–	–	–	–	–	–	–	–	+	+	+	+	+	–	–	–	–	+	–	–	–	–	+	–	–	–	–	+	+	–	–	–	–	–	–	–	–	–	–	–	+	–	+	+	–	+	–
2088	2639	Phage protein, HK97, gp10	14	–	–	–	–	+	–	–	–	–	–	–	–	–	–	–	–	–	–	+	+	+	+	+	–	–	–	–	+	–	–	–	–	+	–	–	–	–	+	+	–	–	–	–	–	–	–	–	–	–	–	+	–	+	+	–	+	–
2089	2640	MAFB alternative C-terminus	14	–	–	–	–	+	–	–	–	–	–	–	–	–	–	–	–	–	–	+	+	+	+	+	–	–	–	–	+	–	–	–	–	+	–	–	–	–	+	+	–	–	–	–	–	–	–	–	–	–	–	+	–	+	+	–	+	–
2090	2641	Lj965 prophage protein	14	–	–	–	–	+	–	–	–	–	–	–	–	–	–	–	–	–	–	+	+	+	+	+	–	–	–	–	+	–	–	–	–	+	–	–	–	–	+	+	–	–	–	–	–	–	–	–	–	–	–	+	–	+	+	–	+	–
2091	2642	hypothetical protein	14	–	–	–	–	+	–	–	–	–	–	–	–	–	–	–	–	–	–	+	+	+	+	+	–	–	–	–	+	–	–	–	–	+	–	–	–	–	+	+	–	–	–	–	–	–	–	–	–	–	–	+	–	+	+	–	+	–
2092	2657	hypothetical protein	14	+	+	+	+	+	+	+	+	+	+	+	+	+	+	–	–	–	–	–	–	–	–	–	–	–	–	–	–	–	–	–	–	–	–	–	–	–	–	–	–	–	–	–	–	–	–	–	–	–	–	–	–	–	–	–	–	–
2093	2658	Bipolar DNA helicase	14	+	+	+	+	+	+	+	+	+	+	+	+	+	+	–	–	–	–	–	–	–	–	–	–	–	–	–	–	–	–	–	–	–	–	–	–	–	–	–	–	–	–	–	–	–	–	–	–	–	–	–	–	–	–	–	–	–
2094	2661	hypothetical protein	14	+	+	+	+	+	+	+	+	+	+	+	+	+	+	–	–	–	–	–	–	–	–	–	–	–	–	–	–	–	–	–	–	–	–	–	–	–	–	–	–	–	–	–	–	–	–	–	–	–	–	–	–	–	–	–	–	–
2095	2663	hypothetical protein	14	+	+	+	+	+	+	+	+	+	+	+	+	+	+	–	–	–	–	–	–	–	–	–	–	–	–	–	–	–	–	–	–	–	–	–	–	–	–	–	–	–	–	–	–	–	–	–	–	–	–	–	–	–	–	–	–	–
2096	2743	Cu+ P-type ATPase	14	–	–	–	–	–	–	–	–	–	–	–	–	–	–	–	–	–	–	–	–	+	+	+	–	+	–	–	+	+	–	+	–	+	+	+	+	+	+	–	+	–	–	–	–	–	–	–	–	–	–	–	–	–	–	–	–	–
2097	2744	Collagen adhesion protein	14	–	–	–	–	–	–	–	–	–	–	–	–	–	–	–	–	–	–	–	+	+	+	–	–	+	+	+	+	+	+	+	+	–	–	–	–	–	–	–	–	+	+	+	–	–	–	–	–	–	–	–	–	–	–	–	–	–
2098	2747	Cu+ P-type ATPase	14	–	–	–	–	–	–	–	–	–	–	–	–	–	–	–	–	–	–	–	–	+	+	+	–	+	–	–	+	+	–	+	–	+	+	+	+	+	+	–	+	–	–	–	–	–	–	–	–	–	–	–	–	–	–	–	–	–
2099	2751	hypothetical protein	14	–	–	–	–	+	–	–	–	–	–	–	–	–	–	–	–	–	–	+	+	+	+	+	–	–	–	–	+	–	–	–	–	+	–	–	–	–	+	+	–	–	–	–	–	–	–	–	–	–	–	+	–	+	+	–	+	–
2100	2756	hypothetical protein	14	+	+	+	+	–	+	+	+	–	+	+	+	+	+	+	–	–	–	–	–	–	–	–	–	–	–	–	–	–	–	–	–	–	–	–	–	–	–	–	+	–	–	–	–	–	–	–	–	–	–	–	–	–	–	–	–	–
2101	2757	Hyaluronate lyase precursor (EC 4.2.2.1)	14	+	+	+	+	+	+	+	+	+	+	+	–	–	–	–	–	–	–	–	–	–	–	–	–	–	–	–	–	–	–	+	+	–	–	–	–	–	–	–	+	–	–	–	–	–	–	–	–	–	–	–	–	–	–	–	–	–
2102	2759	hypothetical protein	14	–	–	–	–	–	–	–	–	–	–	–	–	–	–	–	–	–	–	–	+	+	+	+	+	–	–	+	+	+	+	+	+	–	–	–	–	–	–	–	–	–	–	–	–	–	–	+	–	+	+	–	–	–	–	–	–	–
2103	2781	hypothetical protein	14	+	+	+	+	+	+	+	+	+	+	+	+	+	+	–	–	–	–	–	–	–	–	–	–	–	–	–	–	–	–	–	–	–	–	–	–	–	–	–	–	–	–	–	–	–	–	–	–	–	–	–	–	–	–	–	–	–

Table S5. Precence/absence of each HG among 57 *S. suis* strains

				1																13			25					28						104		94			76			53/54		Divergent *S. suis*																
Index[a]	ClutserID	Gene products	Members	98HAH33	05ZYH33	SC84	A7	SS12	P1/7	DAT273	NIAH11434	JS14	GZ1	BM407	S735	13730	MNCM07	NCTC 10446	14636	NCTC 10237	ST1	2651	89-1591	MNCM04	MNCM25	8074	D9	4961	ST3	MNCM43	DAT292	DAT260	NIAH11435	MNCM21	MNCM50	89-2479	DAT234	DAT290	93A	42A	NT77	6407	11538	8830	12814	92-1400	89-590	2726	2524	89-3576-3	4417	D12	10581	89-5259	88-5299A	R61	92-1191	14A	92-4172	22083
2104	2782	hyaluronidase	14	+	+	+	+	+	+	+	+	+	+	+	+	+	+	–	–	–	–	–	–	–	–	–	–	–	–	–	–	–	–	–	–	–	–	–	–	–	–	–	–	–	–	–	–	–	–	–	–	–	–	–	–	–	–	–	–	–
2105	2783	Ribonucleases G and E	14	+	+	+	+	+	+	+	+	+	+	+	+	+	+	–	–	–	–	–	–	–	–	–	–	–	–	–	–	–	–	–	–	–	–	–	–	–	–	–	–	–	–	–	–	–	–	–	–	–	–	–	–	–	–	–	–	–
2106	2785	Group B streptococcal surface immunogenic protein	14	+	+	+	–	–	+	+	+	+	+	+	–	+	+	+	–	–	–	+	–	–	–	–	–	–	–	–	–	–	–	–	–	–	–	–	–	–	–	–	–	–	–	–	–	–	–	–	–	–	–	–	+	–	–	–
2107	2786	Protein involved in cell division	14	+	+	+	+	+	+	+	+	+	+	+	+	+	+	–	–	–	–	–	–	–	–	–	–	–	–	–	–	–	–	–	–	–	–	–	–	–	–	–	–	–	–	–	–	–	–	–	–	–	–	–	–	–	–	–	–	–
2108	2787	Histone acetyltransferase HPA2 and related acetyltransferases	14	+	+	+	+	+	+	+	+	+	+	+	+	+	+	–	–	–	–	–	–	–	–	–	–	–	–	–	–	–	–	–	–	–	–	–	–	–	–	–	–	–	–	–	–	–	–	–	–	–	–	–	–	–	–	–	–	–
2109	2788	hypothetical protein	14	+	+	+	+	+	+	+	+	+	+	+	+	+	+	–	–	–	–	–	–	–	–	–	–	–	–	–	–	–	–	–	–	–	–	–	–	–	–	–	–	–	–	–	–	–	–	–	–	–	–	–	–	–	–	–	–	–
2110	2798	Site-specific recombinase	14	–	–	–	–	–	–	–	–	–	–	–	–	–	–	–	–	–	–	–	+	+	+	–	–	–	–	–	–	–	+	+	+	+	–	+	–	+	+	–	–	–	–	–	+	–	–	–	–	–	–	+	–	–	+	+	–	–
2111	1701	Replication protein repB	13	–	–	–	–	–	–	–	–	–	–	–	–	–	–	–	–	+	+	+	–	–	–	–	–	–	–	–	–	–	–	–	–	+	+	+	–	–	–	–	–	+	+	+	–	–	–	–	+	–	–	–	+	+	–	–	+	–
2112	1714	Cobalt-zinc-cadmium resistance protein CzcD	13	–	–	–	–	–	–	–	–	–	–	–	–	–	–	–	–	+	+	–	–	–	–	–	–	–	–	–	–	–	–	–	–	–	–	–	–	–	–	–	–	–	–	+	+	–	–	+	+	+	+	+	+	+	–	–	+	+
2113	1746	Transcriptional regulator SczA, TetR family	13	–	–	–	–	–	–	–	–	–	–	–	–	–	–	–	–	+	+	–	–	–	–	–	–	–	–	–	–	–	–	–	–	–	–	–	–	–	–	–	–	–	–	+	+	–	–	+	+	+	+	+	+	+	–	–	+	+
2114	1767	hypothetical protein	13	–	–	–	–	–	–	–	–	–	–	–	–	–	–	–	–	–	–	–	+	+	+	+	+	+	+	+	+	+	+	–	–	–	–	–	–	–	–	–	–	+	+	–	–	–	–	–	–	–	–	–	–	–	–	–	–	–
2115	2045	hypothetical protein	13	–	–	–	–	–	–	–	–	–	–	–	–	–	–	–	–	–	–	–	+	+	+	+	+	+	+	+	+	+	+	–	–	–	–	–	–	–	–	–	–	+	+	–	–	–	–	–	–	–	–	–	–	–	–	–	–	–
2116	2111	ESAT-6/Esx family secreted protein EsxA/YukE	13	–	–	–	–	–	–	–	–	–	–	–	–	–	–	–	–	–	–	–	–	–	–	–	–	+	+	+	+	+	+	–	–	–	–	–	–	–	–	–	–	+	+	+	–	+	–	+	+	+	–	–	–	–	–	–	–	–
2117	2243	Superfamily II DNA and RNA helicase	13	–	–	–	–	–	–	–	–	–	+	–	–	–	–	–	+	–	–	–	–	–	–	–	+	–	–	+	–	+	+	–	–	+	+	+	+	–	+	–	–	–	–	+	–	–	–	–	–	–	+	–	–	–	–	–	–	–
2118	2249	Two-component response regulator, SrtR, lantibiotic-associated	13	–	–	–	–	–	–	+	+	+	–	–	–	–	–	–	–	–	–	–	–	–	–	–	–	–	–	+	+	–	+	+	+	–	–	–	–	–	–	–	–	–	+	+	–	–	–	–	–	–	+	–	+	+	–	–	–	–
2119	2275	transcriptional regulator	13	–	–	–	–	–	–	–	–	–	–	–	–	–	–	–	+	+	+	–	–	–	–	–	–	–	–	–	–	–	–	–	–	–	–	–	–	–	–	–	–	+	+	–	+	–	–	–	+	–	+	+	+	+	+	–	–	+
2120	2281	Putative secretion accessory protein EsaB/YukD	13	–	–	–	–	–	–	–	–	–	–	–	–	–	–	–	–	–	–	–	–	–	–	–	–	+	+	+	+	+	+	–	–	–	–	–	–	–	–	–	–	+	+	+	–	+	–	+	+	+	–	–	–	–	–	–	–	–
2121	2295	hypothetical protein	13	–	–	–	–	–	–	–	–	–	–	–	–	–	–	–	+	–	–	–	+	+	+	–	–	–	–	–	–	–	–	–	–	+	+	+	+	+	+	–	–	–	–	–	–	–	–	–	–	–	–	–	–	+	+	+	–	–
2122	2348	HTH DNA-binding protein	13	–	–	–	–	–	–	–	–	–	–	–	–	–	–	–	–	–	–	–	+	–	–	–	–	–	–	–	–	–	–	+	+	–	+	+	+	–	+	–	–	–	–	–	+	+	+	–	–	–	–	–	+	+	–	+	–	–
2123	2353	Epidermin leader peptide processing serine protease epiP precursor (EC 3.4.21.-)	13	–	–	–	–	–	–	+	+	+	–	–	–	–	–	–	–	–	–	–	–	–	–	–	–	–	–	+	+	–	+	+	+	–	–	–	–	–	–	–	–	–	+	+	–	–	–	–	–	–	+	–	+	+	–	–	–	–
2124	2383	FIG021474: Phage protein	13	–	–	–	–	+	–	–	–	–	–	–	–	–	–	–	–	–	–	–	+	–	–	–	–	+	+	+	+	+	+	+	+	–	+	–	+	+	–	–	–	–	–	–	–	–	–	–	–	–	–	–	–	–	–	–	–	–
2125	2402	Sensor-receptor histidine kinase NisK	13	–	–	–	–	–	–	–	+	+	–	–	–	–	–	–	–	–	–	–	–	–	–	–	–	–	–	+	+	–	+	+	+	–	–	–	–	–	–	–	–	–	+	+	–	–	–	–	–	–	+	+	+	+	–	–	–	–
2126	2436	lysis protein	13	–	–	–	–	–	–	–	–	–	–	–	–	–	–	–	–	+	–	–	–	–	–	–	–	–	–	+	+	+	+	–	–	–	–	–	–	–	–	–	+	–	–	–	+	+	–	+	–	–	–	+	–	–	–	+	+	+
2127	2469	Transcriptional regulator, TetR family	13	–	–	–	–	–	–	–	–	–	–	–	–	–	–	+	–	–	–	+	–	–	–	–	–	–	–	–	–	–	–	–	–	–	–	–	+	+	+	–	–	–	+	+	+	+	–	+	+	+	–	+	–	–	–	–	–	–
2128	2474	Nisin immunity protein precursor	13	–	–	–	–	–	–	+	+	+	–	–	–	–	–	–	–	–	–	–	–	–	–	–	–	–	–	+	+	–	+	+	+	–	–	–	–	–	–	–	–	–	+	+	–	–	–	–	–	–	+	+	–	+	–	–	–	–
2129	2518	Phage capsid and scaffold	13	–	–	–	–	–	–	–	–	–	–	–	–	–	–	+	+	+	+	–	–	–	–	–	–	+	–	–	–	–	–	–	–	–	+	+	–	–	–	+	–	–	–	–	+	+	–	+	–	–	+	+	–	–	+	–	–	–
2130	2555	hypothetical protein	13	–	–	–	–	–	–	+	+	+	–	–	–	–	–	–	–	–	–	–	–	–	–	–	+	–	–	+	+	–	+	+	+	–	–	–	–	–	–	–	–	–	+	+	–	–	–	–	–	–	+	–	–	+	–	–	–	–
2131	2556	membrane protein	13	–	–	–	–	–	–	+	+	+	–	–	–	–	–	–	–	–	–	–	–	–	–	–	–	–	–	+	+	–	+	+	+	–	–	–	–	–	–	–	–	–	+	+	–	–	–	–	–	–	+	–	+	+	–	–	–	–
2132	2607	Putative DNA-binding protein in cluster with Type I restriction-modification system	13	–	–	–	–	–	–	–	–	–	–	–	–	–	–	–	–	+	+	–	+	–	–	–	–	–	–	–	–	–	–	–	–	+	–	–	–	–	–	+	+	+	–	+	–	+	–	–	–	–	+	–	+	–	+	–	–	+
2133	2627	GtrA family protein	13	–	–	–	–	–	–	–	–	–	–	–	–	–	–	–	–	–	–	–	+	–	–	+	+	+	+	–	–	–	–	–	–	–	–	–	–	–	–	–	–	+	–	+	+	+	+	+	+	+	–	–	–	–	–	–	–	–
2134	2735	hypothetical protein	13	–	–	–	–	–	–	–	–	–	–	–	–	–	–	+	–	+	+	–	–	–	–	–	–	–	–	–	–	–	–	–	–	–	–	–	–	–	–	–	–	–	–	+	–	–	+	+	+	+	+	+	–	+	+	+	–	–
2135	2746	hypothetical protein	13	–	–	–	–	–	–	–	–	–	–	–	–	–	–	+	–	–	–	+	–	–	–	+	–	+	+	+	+	+	+	–	–	+	–	–	–	–	–	+	–	–	–	–	–	–	–	–	–	–	–	+	–	–	+	–	–	–
2136	2752	hypothetical protein	13	–	–	–	–	–	–	–	–	–	–	–	–	–	–	–	+	–	–	+	–	+	+	–	–	–	–	–	–	–	–	–	–	+	+	+	–	–	–	+	+	–	–	+	–	–	–	–	–	–	–	–	–	+	+	+	–	–
2137	2754	hypothetical protein	13	–	–	–	+	–	–	+	–	–	+	–	–	+	–	+	–	–	–	–	–	–	–	–	–	–	–	–	+	+	–	–	–	–	+	–	–	–	–	+	–	+	+	+	–	–	+	–	–	–	–	–	–	–	–	–	–	–
2138	2755	hypothetical protein	13	–	–	–	–	+	–	–	–	–	–	–	–	–	–	–	–	–	–	–	+	+	+	+	–	–	–	–	–	–	–	–	–	+	–	–	+	+	+	–	+	–	–	–	–	–	–	–	–	–	+	–	–	–	+	+	–	–
2139	2762	hypothetical protein	13	+	+	+	+	+	+	+	–	+	–	–	–	–	–	–	–	–	–	–	–	–	–	–	–	–	–	–	–	–	–	–	–	–	+	–	+	+	+	–	–	–	–	–	–	–	–	–	–	–	–	–	–	–	–	–	–	+
2140	2772	hypothetical protein	13	+	+	+	+	+	+	–	+	–	–	–	–	–	+	–	–	–	–	–	–	–	–	–	–	–	–	–	–	–	–	+	+	–	–	–	–	–	–	–	–	+	–	–	–	–	–	–	–	–	+	+	–	–	–	–	–	–
2141	2773	hypothetical protein	13	+	+	+	+	+	+	–	+	–	–	–	–	–	+	–	–	–	–	–	–	–	–	–	–	–	–	–	–	–	–	+	+	–	–	–	–	–	–	–	–	+	–	–	–	–	–	–	–	–	+	+	–	–	–	–	–	–
2142	2774	hypothetical protein	13	+	+	+	+	+	+	–	+	–	–	–	–	–	+	–	–	–	–	–	–	–	–	–	–	–	–	–	–	–	–	+	+	–	–	–	–	–	–	–	–	+	–	–	–	–	–	–	–	–	+	+	–	–	–	–	–	–
2143	2784	hypothetical protein	13	+	+	+	+	+	+	+	–	+	+	+	–	+	+	–	–	–	–	–	–	–	–	–	–	–	–	–	–	–	–	–	–	–	+	–	–	–	–	–	–	–	–	–	–	–	–	–	–	–	–	–	–	–	–	–	–	–
2144	2851	transcriptional regulator PlcR, putative	13	+	+	–	–	–	–	–	–	–	–	–	–	–	–	–	–	+	–	–	–	–	–	–	–	–	–	–	–	–	–	–	–	+	+	+	+	+	–	+	+	–	–	–	–	–	–	+	+	+	–	–	–	–	–	–	–	–
2145	2857	Putative secretion accessory protein EsaA/YueB	13	–	–	–	–	–	–	–	–	–	–	–	–	–	–	–	–	–	–	–	–	–	–	–	–	+	+	+	+	+	+	–	–	–	–	–	–	–	–	–	–	+	+	+	–	+	–	+	+	+	–	–	–	–	–	–	–	–
2146	2859	hypothetical protein	13	–	–	–	–	–	–	–	–	–	–	–	–	–	–	–	–	–	–	–	+	+	+	+	+	+	+	+	+	+	+	+	+	–	–	–	–	–	–	–	–	–	–	–	–	–	–	–	–	–	–	–	–	–	–	–	–	–
2147	2861	hypothetical protein	13	–	–	–	–	–	–	–	–	–	–	–	–	–	–	–	–	–	–	–	+	+	+	+	+	+	+	+	+	+	+	–	–	–	–	–	–	–	–	–	–	+	+	–	–	–	–	–	–	–	–	–	–	–	–	–	–	–
2148	2866	hypothetical protein	13	–	–	–	–	–	–	–	–	–	–	–	–	–	–	–	–	–	–	–	–	–	–	–	–	+	+	+	+	+	+	–	–	–	–	–	–	–	–	–	–	+	+	+	–	+	–	+	+	+	–	–	–	–	–	–	–	–
2149	2867	hypothetical protein	13	–	–	–	–	–	–	–	–	–	–	–	–	–	–	–	–	–	–	–	+	+	+	+	+	+	+	+	+	+	+	–	–	–	–	–	–	–	–	–	–	+	+	–	–	–	–	–	–	–	–	–	–	–	–	–	–	–
2150	2868	hypothetical protein	13	–	–	–	–	–	–	–	–	–	–	–	–	–	–	–	–	–	–	–	+	+	+	+	+	+	+	+	+	+	+	–	–	–	–	–	–	–	–	–	–	+	+	–	–	–	–	–	–	–	–	–	–	–	–	–	–	–
2151	2881	ABC-type multidrug transport system, ATPase and permease component	13	+	+	+	–	+	+	–	+	+	–	+	+	–	+	–	–	–	–	–	–	–	–	–	–	–	–	–	–	–	–	–	–	–	–	–	–	–	–	–	+	–	–	–	–	+	–	–	–	+	–	–	–	–	–	–	–	–
2152	2882	hypothetical protein	13	+	+	+	+	+	+	+	+	+	+	–	–	–	–	–	–	–	–	–	–	–	–	–	–	–	–	–	–	–	–	–	–	–	–	–	–	–	–	–	–	–	–	–	–	+	+	–	–	+	–	–	–	–	–	–	–	–
2153	2893	hypothetical protein	13	–	–	–	–	–	–	–	–	–	–	–	–	–	–	–	–	–	–	+	–	–	–	–	–	–	–	–	–	–	–	–	–	+	+	+	+	+	+	–	–	–	–	–	–	–	–	–	+	–	+	+	+	–	–	+	+	–
2154	2913	hypothetical protein	13	+	+	+	+	–	+	+	+	+	+	+	+	+	+	–	–	–	–	–	–	–	–	–	–	–	–	–	–	–	–	–	–	–	–	–	–	–	–	–	–	–	–	–	–	–	–	–	–	–	–	–	–	–	–	–	–	–
2155	2914	von Willebrand factor, type A	13	+	+	+	+	+	+	+	+	+	+	+	+	+	–	–	–	–	–	–	–	–	–	–	–	–	–	–	–	–	–	–	–	–	–	–	–	–	–	–	–	–	–	–	–	–	–	–	–	–	–	–	–	–	–	–	–	–
2156	2936	hypothetical protein	13	+	+	+	+	+	+	+	+	+	+	+	+	–	+	–	–	–	–	–	–	–	–	–	–	–	–	–	–	–	–	–	–	–	–	–	–	–	–	–	–	–	–	–	–	–	–	–	–	–	–	–	–	–	–	–	–	–
2157	1869	TrsE-like protein	12	–	–	–	–	–	–	–	–	–	–	–	–	–	–	–	+	–	–	–	–	–	–	–	+	–	–	+	–	+	+	–	–	+	+	+	+	–	+	–	–	–	–	+	–	–	–	–	–	–	+	–	–	–	–	–	–	–
2158	2121	hypothetical protein	12	–	–	–	–	–	–	–	–	–	–	–	–	–	–	–	+	–	–	–	–	–	–	–	+	–	–	+	–	+	+	–	–	+	+	+	+	–	+	–	–	–	–	+	–	–	–	–	–	–	+	–	–	–	–	–	–	–
2159	2122	Tn5252, Orf23	12	–	–	–	–	–	–	–	–	–	–	–	–	–	–	–	+	–	–	–	–	–	–	–	+	–	–	+	–	+	+	–	–	+	+	+	+	–	+	–	–	–	–	+	–	–	–	–	–	–	+	–	–	–	–	–	–	–
2160	2151	hypothetical protein	12	–	–	–	–	–	–	–	–	–	–	–	–	–	–	–	+	–	–	–	–	–	–	–	+	–	–	+	–	+	+	–	–	+	+	+	+	–	+	–	–	–	–	+	–	–	–	–	–	–	+	–	–	–	–	–	–	–
2161	2187	Lactaldehyde dehydrogenase involved in fucose or rhamnose utilization (EC 1.2.1.22)	12	–	–	–	–	–	–	–	–	–	–	–	–	–	–	–	–	–	–	–	+	+	+	+	+	–	–	–	–	–	–	–	–	–	+	+	–	–	+	–	–	–	–	–	–	–	–	–	–	–	+	–	+	+	–	+	–	–
2162	2245	TrsK-like protein	12	–	–	–	–	–	–	–	–	–	–	–	–	–	–	–	+	–	–	–	–	–	–	–	+	–	–	+	–	+	+	–	–	+	+	+	+	–	+	–	–	–	–	+	–	–	–	–	–	–	+	–	–	–	–	–	–	–
2163	2296	Relaxase	12	–	–	–	–	–	–	–	–	–	–	–	–	–	–	–	+	–	–	–	–	–	–	–	+	–	–	+	–	+	+	–	–	+	+	+	+	–	+	–	–	–	–	+	–	–	–	–	–	–	+	–	–	–	–	–	–	–
2164	2322	prophage pi2 protein 40	12	–	–	–	–	–	–	–	–	+	–	–	–	–	–	–	–	+	–	–	–	–	–	–	–	–	–	–	–	–	–	–	–	–	–	–	+	+	–	+	+	–	–	–	–	+	–	+	–	+	–	+	+	–	+	–	–	–
2165	2331	Phage protein	12	–	–	–	–	–	–	–	–	–	–	–	–	–	–	–	–	–	–	–	+	–	–	–	–	+	+	+	+	+	+	+	+	–	+	–	–	–	–	–	–	–	–	–	–	–	–	–	–	–	+	–	–	–	–	–	–	+
2166	2337	Phage portal protein; Phage capsid and scaffold	12	–	–	–	–	–	–	–	–	–	–	–	–	–	–	–	–	–	–	–	+	–	–	–	–	+	+	+	+	+	+	+	+	–	+	–	–	–	–	–	–	–	–	–	–	–	–	–	–	–	+	–	–	–	–	–	–	+
2167	2376	CRISPR-associated protein Cas1	12	–	–	–	–	–	–	–	–	–	–	–	–	–	–	–	–	–	–	–	+	+	+	+	+	+	+	+	+	+	+	–	–	–	–	–	–	–	–	–	–	+	–	–	–	–	–	–	–	–	–	–	–	–	–	–	–	–
2168	2377	Putative secretion system component EssB/YukC	12	–	–	–	–	–	–	–	–	–	–	–	–	–	–	–	–	–	–	–	–	–	–	–	–	+	+	+	+	+	+	–	–	–	–	–	–	–	–	–	–	+	+	–	–	+	–	+	+	+	–	–	–	–	–	–	–	–
2169	2384	CRISPR-associated protein, Csn1 family	12	–	–	–	–	–	–	–	–	–	–	–	–	–	–	–	–	–	–	–	+	+	+	+	+	+	+	+	+	+	+	–	–	–	–	–	–	–	–	–	–	+	–	–	–	–	–	–	–	–	–	–	–	–	–	–	–	–
2170	2399	site-specific recombinase, phage integrase family	12	+	+	+	+	+	+	–	+	–	–	–	–	–	+	–	–	–	–	–	–	–	–	–	–	–	–	–	–	–	–	+	+	–	–	–	–	–	–	–	–	–	–	–	–	–	–	–	+	–	–	–	–	+	–	–	–	–
2171	2434	CRISPR-associated protein Cas2	12	–	–	–	–	–	–	–	–	–	–	–	–	–	–	–	–	–	–	–	+	+	+	+	+	+	+	+	+	+	+	–	–	–	–	–	–	–	–	–	–	+	–	–	–	–	–	–	–	–	–	–	–	–	–	–	–	–
2172	2439	Mobile element protein	12	+	+	+	–	+	+	–	–	–	–	+	–	–	–	–	–	+	+	–	–	–	–	+	–	–	+	–	–	–	–	–	–	–	–	+	–	–	+	–	–	–	–	–	–	–	–	–	–	–	–	–	–	–	–	–	–	–	–	–
2173	2456	putative conjugative transposon membrane protein	12	–	–	–	–	–	–	–	–	–	–	–	–	–	–	–	+	–	–	–	–	–	–	–	+	–	–	+	–	+	+	–	–	+	+	+	+	–	+	–	–	–	–	+	–	–	–	–	–	–	+	–	–	–	–	–	–	–
2174	2471	hypothetical protein	12	–	–	–	–	–	–	–	–	–	–	–	–	–	–	+	–	–	–	–	–	–	–	–	–	–	–	–	–	–	–	–	–	–	–	–	+	+	+	–	–	–	+	+	+	+	–	+	+	+	–	+	–	–	–	–	–	–
2175	2473	hypothetical protein	12	–	–	–	–	–	–	–	–	–	–	–	–	–	–	+	–	–	–	+	–	–	–	–	–	–	–	–	–	–	–	–	–	–	–	–	+	+	+	–	–	–	+	+	+	+	–	–	+	+	–	+	–	–	–	–	–	–
2176	2545	Carboxylesterase, type B	12	–	–	–	–	–	–	–	–	–	–	–	–	–	–	–	–	–	–	–	+	+	+	+	+	–	–	–	–	–	–	–	–	–	+	+	+	+	–	–	–	–	–	–	–	–	–	–	–	–	+	–	+	–	–	–	+	–
2177	2622	hypothetical protein	12	–	–	–	–	–	–	–	–	–	–	–	–	–	–	+	–	–	–	+	–	–	–	–	–	+	–	–	–	–	–	–	–	–	+	–	+	+	+	+	–	–	–	–	–	–	–	–	–	–	–	+	–	+	+	+	–	–
2178	2629	ORF014	12	–	–	–	–	–	–	–	–	–	–	–	–	–	–	–	–	–	–	–	–	+	+	+	+	+	+	+	+	+	+	–	–	–	–	–	–	–	–	–	–	–	–	+	–	–	–	–	–	+	–	–	–	–	–	–	–	–
2179	2635	hypothetical protein	12	–	–	–	–	–	–	–	–	–	–	–	–	–	–	–	–	–	–	–	+	+	+	+	+	–	–	–	–	–	–	–	–	–	–	–	+	+	+	+	+	–	–	–	–	+	+	–	–	–	–	–	–	–	–	–	–	–
2180	2638	hypothetical protein	12	–	–	–	–	–	–	–	–	–	–	–	–	–	–	–	–	–	–	–	+	+	+	+	+	–	–	–	–	–	–	–	–	–	–	–	+	+	+	+	+	–	–	–	–	+	+	–	–	–	–	–	–	–	–	–	–	–
2181	2646	hypothetical protein	12	–	–	–	–	–	–	–	–	–	–	–	–	–	–	–	–	–	–	–	–	+	–	–	–	–	–	–	+	–	–	+	–	+	+	+	+	+	–	–	–	–	–	–	–	–	–	+	+	–	+	–	+	–	–	–	–	–
2182	2698	Type I restriction-modification system, specificity subunit S (EC 3.1.21.3)	12	+	+	+	+	+	+	–	–	+	+	+	+	–	–	–	–	–	–	–	–	–	–	–	–	–	–	–	–	–	–	–	–	–	+	+	–	–	–	–	–	–	–	–	–	–	–	–	–	–	–	–	–	–	–	–	–	–
2183	2740	Phage capsid and scaffold	12	–	–	–	–	–	–	–	–	–	–	–	–	–	–	+	+	+	+	–	–	–	–	–	–	+	–	–	–	–	–	–	–	–	+	+	–	–	–	+	–	–	–	–	+	–	–	+	–	–	+	–	–	–	–	+	–	–
2184	2753	hypothetical protein	12	–	–	–	–	–	–	–	–	–	–	–	–	–	–	–	+	–	–	+	–	–	–	–	–	–	–	–	–	–	–	+	+	–	–	–	–	–	–	+	+	+	+	+	–	–	–	–	–	–	–	+	+	+	–	–	–	–
2185	2760	hypothetical protein	12	–	–	–	–	+	–	–	–	–	–	–	–	–	–	–	–	–	–	–	+	+	+	+	–	–	–	–	+	–	–	+	+	+	+	–	–	–	–	–	–	+	–	–	–	–	–	–	–	–	–	–	+	–	–	–	–	–
2186	2761	Signal transduction histidine kinase	12	–	–	–	–	–	–	–	–	–	–	–	–	–	–	–	–	–	–	+	–	–	–	–	–	–	–	–	–	–	–	–	–	–	–	–	–	–	–	–	–	–	+	–	–	+	–	–	–	+	+	+	+	+	+	+	+	+
2187	2848	hypothetical protein	12	–	–	–	–	–	–	–	–	–	–	–	–	–	–	–	–	+	–	–	–	–	–	–	–	–	–	–	–	–	–	–	–	–	–	+	+	+	–	+	–	–	–	–	–	+	–	+	–	–	+	+	+	–	+	+	–	–
2188	2852	hypothetical protein	12	–	–	–	–	–	–	–	–	–	–	–	–	–	–	–	–	+	+	+	–	–	–	–	–	–	–	–	–	–	–	+	+	–	–	–	–	–	–	+	+	+	–	+	–	–	–	–	–	–	–	+	+	+	–	–	–	–
2189	2855	hypothetical protein	12	–	–	–	–	–	–	–	–	–	–	–	–	–	–	–	–	+	–	–	–	–	–	–	–	–	–	–	–	–	–	–	–	–	–	+	+	+	–	+	–	–	–	–	–	+	–	+	–	–	+	+	+	–	+	+	–	–
2190	2856	hypothetical protein	12	–	–	–	–	–	–	–	–	–	–	–	–	–	–	–	–	–	–	–	+	+	+	+	–	+	+	+	+	+	+	–	–	–	–	–	–	–	–	+	+	–	–	–	–	–	–	–	–	–	–	–	–	–	–	–
2191	2864	hypothetical protein	12	–	–	–	–	–	–	–	–	–	–	–	–	–	–	–	–	–	–	–	–	–	–	–	–	+	+	+	+	+	+	–	–	–	–	–	–	–	–	–	–	+	+	+	–	–	–	+	+	+	–	–	–	–	–	–	–	–
2192	2871	CRISPR-associated protein Cas7	12	–	–	–	–	–	–	–	–	–	–	–	–	–	–	–	–	–	–	–	+	+	+	+	+	+	+	+	+	+	+	–	–	–	–	–	–	–	–	–	–	+	–	–	–	–	–	–	–	–	–	–	–	–	–	–	–	–
2193	2886	hypothetical protein	12	–	–	–	–	–	–	–	–	–	–	–	–	–	–	–	+	–	–	–	–	–	–	–	+	–	–	+	–	+	+	–	–	+	+	+	+	–	+	–	–	–	–	+	–	–	–	–	–	–	+	–	–	–	–	–	–	–
2194	2889	Two-component response regulator SA14-24	12	–	–	–	–	–	–	–	–	–	–	–	–	–	–	–	–	–	–	+	–	–	–	–	–	–	–	–	–	–	–	–	–	–	–	–	–	–	–	–	–	–	+	–	–	+	–	–	–	+	+	+	+	+	+	+	+	+
2195	2890	Predicted cell-wall-anchored protein SasA (LPXTG motif)	12	–	–	–	–	–	–	–	–	–	–	–	–	–	–	–	–	–	–	+	–	–	–	–	–	–	–	–	–	–	–	–	–	–	–	–	–	–	–	–	–	–	+	–	–	+	–	–	–	+	+	+	+	+	+	+	+	+
2196	2912	Epf-like protein	12	+	+	+	+	+	+	+	+	+	+	–	–	+	–	–	–	–	–	–	–	–	–	–	–	–	–	–	–	–	–	–	–	–	–	–	–	–	–	–	–	–	–	–	–	–	–	–	–	–	–	–	–	+	–	–	–	–
2197	2989	hypothetical protein	12	–	–	–	–	–	–	–	–	–	–	–	–	–	–	–	–	–	–	–	+	–	–	–	+	–	+	+	+	+	+	–	–	+	+	+	–	–	–	–	–	–	+	–	–	–	–	–	–	–	–	–	–	–	+	–	–	–
2198	2991	hypothetical protein	12	–	–	–	–	–	–	–	–	–	–	–	–	–	–	–	–	–	–	–	–	–	–	+	+	–	–	–	–	–	–	+	+	+	+	+	+	+	+	+	+	–	–	–	–	–	–	–	–	–	–	–	–	–	–	–	–	–
2199	2992	hypothetical protein	12	–	–	–	–	–	–	–	–	–	–	–	–	–	–	–	–	–	–	–	+	+	+	+	+	–	–	–	–	–	–	–	–	–	–	–	+	+	+	+	+	–	–	–	–	+	+	–	–	–	–	–	–	–	–	–	–	–
2200	2993	Serine hydroxymethyltransferase (EC 2.1.2.1)	12	–	–	–	–	–	–	–	–	–	–	–	–	–	–	–	–	–	–	–	–	–	–	+	+	–	–	–	–	–	–	+	+	+	+	+	+	+	+	+	+	–	–	–	–	–	–	–	–	–	–	–	–	–	–	–	–	–
2201	2994	hypothetical protein	12	–	–	–	–	–	–	–	–	–	–	–	–	–	–	–	+	–	–	–	–	–	–	–	–	–	–	–	–	–	–	–	–	+	+	+	+	+	+	+	+	+	+	–	–	–	–	–	–	–	–	–	–	–	–	–	+	–
2202	2999	hypothetical protein	12	–	–	–	–	–	–	–	–	–	–	–	–	–	–	–	+	–	–	–	–	–	–	–	–	–	–	–	–	–	–	–	–	+	+	+	+	+	+	+	+	+	+	–	–	–	–	–	–	–	–	–	–	–	–	–	+	–
2203	3001	hypothetical protein	12	+	+	+	–	+	–	–	+	+	+	+	–	–	+	–	–	–	–	–	–	–	–	–	+	–	–	–	–	–	+	–	–	–	–	–	–	–	–	+	–	–	–	–	–	–	–	–	–	–	–	–	–	–	–	–	–	–
2204	3002	hypothetical protein	12	–	–	–	–	–	–	–	–	–	–	–	–	–	–	–	+	–	–	–	–	–	–	–	+	–	–	+	–	+	+	–	–	+	+	+	+	–	+	–	–	–	–	+	–	–	–	–	–	–	+	–	–	–	–	–	–	–
2205	3004	Phage tail length tape-measure protein	12	–	–	–	–	+	–	–	–	–	–	–	–	–	–	–	–	–	–	–	+	+	+	+	–	–	–	–	+	–	–	–	–	+	–	–	–	–	+	+	–	–	–	–	–	–	–	–	–	–	–	–	–	+	+	–	+	–
2206	3009	hypothetical protein	12	–	–	–	–	–	–	–	–	–	–	–	–	–	–	–	+	–	–	–	–	–	–	–	+	–	–	+	–	+	+	–	–	+	+	+	+	–	+	–	–	–	–	+	–	–	–	–	–	–	+	–	–	–	–	–	–	–
2207	3010	hypothetical protein	12	–	–	–	–	–	–	–	–	–	–	–	–	–	–	–	+	–	–	–	–	–	–	–	+	–	–	+	–	+	+	–	–	+	+	+	+	–	+	–	–	–	–	+	–	–	–	–	–	–	+	–	–	–	–	–	–	–
2208	3013	N-acetylmuramoyl-L-alanine amidase	12	–	–	–	–	–	–	–	–	–	–	–	–	–	–	–	+	–	–	–	–	–	–	–	+	–	–	+	–	+	+	–	–	+	+	+	+	–	+	–	–	–	–	+	–	–	–	–	–	–	+	–	–	–	–	–	–	–
2209	3014	hypothetical protein	12	–	–	–	–	–	–	–	–	–	–	–	–	–	–	–	+	–	–	–	–	–	–	–	+	–	–	+	–	+	+	–	–	+	+	+	+	–	+	–	–	–	–	+	–	–	–	–	–	–	+	–	–	–	–	–	–	–
2210	3015	Mature parasite-infected erythrocyte surface antigen (MESA) or PfEMP2	12	–	–	–	–	–	–	–	–	–	–	–	–	–	–	–	+	–	–	–	–	–	–	–	+	–	–	+	–	+	+	–	–	+	+	+	+	–	+	–	–	–	–	+	–	–	–	–	–	–	+	–	–	–	–	–	–	–
2211	3016	hypothetical protein	12	–	–	–	–	–	–	–	–	–	–	–	–	–	–	–	+	–	–	–	–	–	–	–	+	–	–	+	–	+	+	–	–	+	+	+	+	–	+	–	–	–	–	+	–	–	–	–	–	–	+	–	–	–	–	–	–	–
2212	3017	hypothetical protein	12	–	–	–	–	–	–	–	–	–	–	–	–	–	–	–	+	–	–	–	–	–	–	–	+	–	–	+	–	+	+	–	–	+	+	+	+	–	+	–	–	–	–	+	–	–	–	–	–	–	+	–	–	–	–	–	–	–
2213	3021	hypothetical protein	12	–	–	–	–	–	–	–	–	–	–	–	–	–	–	–	–	–	–	–	–	–	–	–	–	–	–	–	–	–	–	–	–	+	+	+	+	+	+	–	–	–	–	–	–	+	–	+	+	+	–	–	–	–	–	–	+	+
2214	3028	hypothetical protein	12	–	–	–	–	–	–	–	–	–	–	–	–	–	–	–	–	–	–	+	–	–	–	–	–	–	–	–	–	–	–	–	–	+	+	+	+	+	+	–	–	–	–	–	+	+	–	+	+	+	–	–	–	–	–	–	–	–
2215	3037	hypothetical protein	12	+	+	+	+	–	+	+	+	+	+	+	+	+	–	–	–	–	–	–	–	–	–	–	–	–	–	–	–	–	–	–	–	–	–	–	–	–	–	–	–	–	–	–	–	–	–	–	–	–	–	–	–	–	–	–	–	–
2216	3038	Protein of unknown function DUF975	12	+	+	+	–	+	+	+	+	+	–	+	+	+	–	+	–	–	–	–	–	–	–	–	–	–	–	–	–	–	–	–	–	–	–	–	–	–	–	–	–	–	–	–	–	–	–	–	–	–	–	–	–	–	–	–	–	–
2217	3039	Type I restriction-modification system, DNA-methyltransferase subunit M (EC 2.1.1.72)	12	+	+	+	–	–	+	+	+	+	+	+	+	+	+	–	–	–	–	–	–	–	–	–	–	–	–	–	–	–	–	–	–	–	–	–	–	–	–	–	–	–	–	–	–	–	–	–	–	–	–	–	–	–	–	–	–	–
2218	3058	hypothetical protein	12	+	+	+	+	+	+	+	+	+	–	–	–	–	–	–	–	–	–	–	–	–	–	–	–	–	–	–	–	–	–	+	+	–	+	–	–	–	–	–	–	–	–	–	–	–	–	–	–	–	–	–	–	–	–	–	–	–
2219	1671	Fumarate reductase, flavoprotein subunit precursor (EC 1.3.99.1)	11	–	–	–	–	–	–	–	–	–	–	–	–	–	–	–	–	–	–	–	–	–	–	–	–	–	–	–	–	–	–	–	–	–	–	–	–	–	–	–	–	–	–	+	–	+	–	+	+	–	+	+	+	+	+	+	–	+
2220	1763	FtsK/SpoIIIE family	11	–	–	–	–	–	–	–	–	–	–	–	–	–	–	–	–	+	+	+	–	–	–	–	–	–	–	–	–	–	–	–	–	+	+	+	–	–	–	–	–	+	–	–	–	–	–	–	+	–	–	–	+	+	–	–	+	–
2221	1776	hypothetical protein	11	–	–	–	–	–	–	–	–	–	–	–	–	–	–	–	–	+	+	+	–	–	–	–	–	–	–	–	–	–	–	–	–	+	+	+	–	–	–	–	–	+	–	–	–	–	–	–	+	–	–	–	+	+	–	–	+	–
2222	1845	Formate-nitrate transporter	11	–	–	–	–	–	–	–	–	–	–	–	–	–	–	–	–	–	–	–	–	–	–	–	–	–	–	–	–	–	–	–	–	–	–	–	–	–	–	–	–	–	–	+	–	+	–	+	+	–	+	+	+	+	+	+	–	+
2223	2016	Transcriptional regulator, Cro/CI family	11	–	–	–	–	–	–	–	–	–	–	–	–	–	–	–	–	–	–	–	–	–	–	–	–	–	–	–	–	–	–	+	+	+	+	+	+	+	–	–	–	–	+	+	+	–	–	–	–	–	–	+	–	–	–	–	–	–
2224	2184	Mobile element protein	11	–	–	–	–	–	–	–	–	–	–	–	–	–	–	–	–	–	–	–	+	–	+	–	–	–	–	+	+	+	+	–	–	–	–	–	–	+	–	–	–	–	+	–	–	+	+	+	–	–	–	–	–	–	–	–	–	–
2225	2235	Undecaprenyl-phosphate galactosephosphotransferase (EC 2.7.8.6)	11	–	–	–	–	–	–	–	–	–	–	–	–	–	–	–	–	–	–	–	–	–	–	–	–	–	–	–	–	–	–	–	–	–	–	–	–	–	–	–	–	+	+	+	+	–	–	–	+	+	–	–	+	+	+	+	–	+
2226	2284	Phage lysin, N-acetylmuramoyl-L-alanine amidase (EC 3.5.1.28)	11	–	–	–	–	–	–	–	–	–	–	–	–	–	–	+	–	–	–	–	–	–	–	–	–	+	–	–	–	–	–	–	–	–	+	+	–	–	–	+	+	–	–	–	–	+	–	+	–	–	–	–	+	–	+	+	–	–
2227	2378	Phage capsid and scaffold # FIG020433	11	–	–	–	–	–	–	–	–	–	–	–	–	–	–	–	–	–	–	–	+	–	–	–	–	+	–	+	+	+	+	+	+	–	+	–	–	–	–	–	–	–	–	–	–	–	–	–	–	–	+	–	–	–	–	–	–	+
2228	2382	Phage terminase	11	–	–	–	–	–	–	–	–	–	–	–	–	–	–	–	–	–	–	–	+	–	–	–	–	+	–	+	+	+	+	+	+	–	+	–	–	–	–	–	–	–	–	–	–	–	–	–	–	–	+	–	–	–	–	–	–	+
2229	2389	Resolvase, N-terminal domain	11	–	–	–	–	–	–	–	–	–	–	–	–	–	–	–	–	–	–	–	–	–	–	–	–	–	–	–	–	–	–	–	–	+	–	–	+	+	–	+	+	–	–	–	–	–	–	–	–	–	+	+	+	+	+	–	–	+
2230	2431	hypothetical protein	11	–	–	–	–	–	–	–	–	–	–	–	–	–	–	–	–	+	+	–	–	–	–	–	–	–	–	–	–	–	–	–	–	–	–	–	–	–	–	+	+	–	–	+	–	–	–	+	+	+	–	–	+	–	–	–	+	+
2231	2449	Site-specific recombinase	11	–	–	–	–	–	–	–	–	–	–	+	–	–	–	–	+	–	–	–	–	–	–	+	–	–	–	–	–	–	–	–	–	–	–	–	–	–	+	+	+	+	–	+	–	+	–	+	+	–	–	–	–	–	–	–	–	–
2232	2516	putative integrase	11	–	–	–	–	–	–	–	–	–	–	–	–	–	–	–	–	+	+	–	–	–	–	–	–	–	–	–	–	–	–	–	–	–	–	–	–	–	–	+	+	–	–	+	–	–	–	+	+	+	+	–	+	–	–	–	–	+
2233	2526	Phage major tail shaft protein	11	–	–	–	–	–	–	–	–	–	–	–	–	–	–	+	+	+	+	–	–	–	–	–	–	+	–	–	–	–	–	–	–	–	+	–	–	–	–	+	–	–	–	–	+	–	–	+	–	–	+	–	–	–	–	+	–	–
2234	2557	hypothetical protein	11	–	–	–	–	–	–	–	–	–	–	–	–	–	–	–	–	–	–	–	–	–	–	–	–	–	–	+	+	+	–	+	+	–	–	–	–	–	–	–	–	+	–	–	–	–	–	–	–	+	–	+	+	+	–	+	–	–
2235	2563	Integrase	11	–	–	–	–	–	–	–	–	–	–	–	–	–	–	–	–	–	–	–	–	–	–	–	+	–	–	+	–	–	–	–	–	+	+	+	+	–	+	–	–	–	–	–	–	+	–	–	–	–	+	+	–	+	–	–	–	–
2236	2620	Phage Holliday junction resolvase	11	–	–	–	–	–	–	–	–	–	–	–	–	–	–	+	–	–	–	–	–	–	–	+	–	+	–	–	–	–	–	–	–	+	+	+	+	+	–	–	–	–	–	–	–	–	–	–	–	–	–	–	–	+	–	+	–	+
2237	2624	FIG006126: DNA helicase, restriction/modification system component YeeB	11	–	–	–	–	–	–	–	–	–	–	–	–	–	–	–	–	–	–	–	+	–	–	+	+	+	+	+	+	+	+	+	+	–	–	–	–	–	–	–	–	–	–	–	–	–	–	–	–	–	–	–	–	–	–	–	–	–
2238	2628	FIG045374: Type II restriction enzyme, methylase subunit YeeA	11	–	–	–	–	–	–	–	–	–	–	–	–	–	–	–	–	–	–	–	+	–	–	+	+	+	+	+	+	+	+	+	+	–	–	–	–	–	–	–	–	–	–	–	–	–	–	–	–	–	–	–	–	–	–	–	–	–
2239	2649	membrane protein, putative	11	–	–	–	–	–	–	–	–	–	–	–	–	–	–	–	–	–	–	–	–	–	–	–	–	–	–	–	–	–	–	+	+	+	+	+	+	+	–	–	–	–	+	+	+	–	–	–	–	–	–	+	–	–	–	–	–	–
2240	2739	hypothetical protein	11	–	–	+	+	–	–	–	–	–	–	–	–	–	–	–	–	+	+	–	–	–	–	–	–	–	–	–	–	–	–	+	+	–	–	–	–	–	–	+	+	–	–	+	–	–	–	+	+	–	–	–	–	–	–	–	–	–
2241	2742	Cro-like phage transcriptional repressor protein	11	–	–	–	–	–	–	–	–	–	–	–	–	–	–	–	+	–	–	–	+	+	+	–	–	+	+	+	+	+	+	–	–	–	–	–	–	–	–	–	–	–	–	–	–	–	–	–	–	–	–	+	–	–	–	–	–	–
2242	2745	Phage-associated HNH homing endonuclease	11	–	–	–	–	–	–	–	–	–	–	–	–	–	–	–	–	–	–	–	+	–	–	–	–	+	+	+	+	+	+	+	+	–	+	–	–	–	–	–	–	–	–	–	–	–	–	–	–	–	+	–	–	–	–	–	–	–
2243	2792	putative aminoglycoside 6-adenylyltransferase	11	+	+	+	–	–	–	–	+	+	–	+	–	–	–	–	–	–	–	–	–	–	–	–	–	–	–	–	–	–	–	–	–	–	–	–	–	–	–	–	–	–	–	–	+	+	–	–	–	+	–	–	–	+	–	–	+	–
2244	2796	hypothetical protein	11	–	–	–	–	+	–	–	–	–	–	–	–	–	–	–	–	–	–	–	+	–	–	–	–	–	–	–	–	–	–	+	+	+	+	–	+	+	+	–	–	–	–	–	–	–	–	–	–	–	–	+	–	+	–	–	–	–
2245	2860	YeeC-like protein	11	–	–	–	–	–	–	–	–	–	–	–	–	–	–	–	–	–	–	–	+	–	–	+	+	+	+	+	+	+	+	+	+	–	–	–	–	–	–	–	–	–	–	–	–	–	–	–	–	–	–	–	–	–	–	–	–	–
2246	2870	YeeC-like protein	11	–	–	–	–	–	–	–	–	–	–	–	–	–	–	–	–	–	–	–	+	–	–	+	+	+	+	+	+	+	+	+	+	–	–	–	–	–	–	–	–	–	–	–	–	–	–	–	–	–	–	–	–	–	–	–	–	–
2247	2976	hypothetical protein	11	–	–	–	–	–	–	–	–	–	–	–	–	–	–	–	+	+	+	+	–	–	–	–	–	–	–	–	–	–	–	–	–	–	–	–	–	–	–	+	+	+	–	–	+	–	–	+	–	–	–	+	–	–	–	+	–

Table S5. Precence/absence of each HG among 57 *S. suis* strains

				1																13			25					28						104		94			76			53/54		Divergent *S. suis*																
Index[a]	ClutserID	Gene products	Members	98HAH33	05ZYH33	SC84	A7	SS12	P1/7	DAT273	NIAH11434	JS14	GZ1	BM407	S735	13730	MNCM07	NCTC 10446	14636	NCTC 10237	ST1	2651	89-1591	MNCM04	MNCM25	8074	D9	4961	ST3	MNCM43	DAT292	DAT260	NIAH11435	MNCM21	MNCM50	89-2479	DAT234	DAT290	93A	42A	NT77	6407	11538	8830	12814	92-1400	89-590	2726	2524	89-3576-3	4417	D12	10581	89-5259	88-5299A	R61	92-1191	14A	92-4172	22083
2248	2978	hypothetical protein	11	-	-	-	-	-	-	-	-	-	-	-	-	-	-	-	-	+	+	-	-	+	+	+	+	+	+	-	-	-	-	-	-	-	-	-	-	-	-	-	-	-	-	+	+	+	-	-	-	-	-	-	-	-
2249	2979	Phage-related protein	11	-	-	-	-	-	-	-	-	-	-	-	-	-	-	-	+	+	+	+	-	-	-	-	-	-	-	-	-	-	-	-	-	-	-	-	-	-	-	+	+	+	-	-	+	-	-	+	-	-	-	-	+	-	-	-	+	-
2250	2980	putative low temperature requirement A protein	11	-	-	-	-	-	-	-	-	-	-	-	-	-	-	-	-	-	-	-	-	+	+	-	-	+	+	+	+	+	+	-	-	+	+	+	-	-	-	-	-	-	-	-	-	-	-	-	-	-	-	-	-	-	-	-	-	-
2251	2981	putative low temperature requirement A protein	11	-	-	-	-	-	-	-	-	-	-	-	-	-	-	-	-	-	-	-	-	+	+	-	-	+	+	+	+	+	+	-	-	+	+	+	-	-	-	-	-	-	-	-	-	-	-	-	-	-	-	-	-	-	-	-	-	-
2252	2985	hypothetical protein	11	-	-	-	-	-	-	-	-	-	-	-	-	-	-	-	-	-	-	-	-	-	-	-	-	+	+	+	+	+	+	-	-	-	-	-	-	-	-	-	-	+	+	+	-	-	-	+	+	-	-	-	-	-	-	-	-	-
2253	2986	Mobile element protein	11	-	-	-	-	-	-	-	-	-	-	-	-	-	-	-	-	-	-	-	-	-	-	-	-	+	+	+	-	-	+	+	+	-	-	-	-	-	-	-	-	+	+	+	-	+	-	-	-	-	-	+	-	-	-	-	-	-
2254	3007	Phage hyaluronidase	11	-	-	-	-	-	-	-	-	-	-	-	-	-	-	-	-	-	-	-	+	-	-	-	-	-	-	+	+	+	+	+	+	+	+	-	-	-	-	-	-	-	-	-	-	-	-	-	-	-	+	-	-	-	-	-	-	+
2255	3012	Modification methylase ScrFIB (EC 2.1.1.37)	11	-	-	-	-	-	-	-	-	-	-	-	-	-	-	-	+	-	-	-	-	-	-	-	-	-	-	+	-	+	+	-	-	+	+	+	+	-	+	-	-	-	-	+	-	-	-	-	-	-	+	-	-	-	-	-	-	-
2256	3020	Salt-stress induced protein	11	+	+	-	-	-	-	-	-	-	-	-	-	-	-	-	-	-	-	-	-	-	-	-	-	-	-	-	+	+	-	-	-	+	+	+	-	-	-	-	-	-	-	+	-	-	-	-	-	-	+	-	-	-	+	-	-	+
2257	3100	hypothetical protein	11	-	-	-	-	-	-	-	-	-	-	-	-	-	-	-	-	+	-	-	+	-	-	-	+	+	-	+	-	-	+	+	+	-	+	+	-	-	-	-	-	+	-	-	-	-	-	-	-	-	-	-	-	-	-	-	-	-
2258	3103	hypothetical protein	11	-	-	-	+	+	-	+	-	+	-	+	-	+	-	+	-	+	+	-	-	-	-	-	-	-	-	-	-	-	-	-	-	-	-	-	-	-	-	-	-	-	-	-	+	-	-	-	-	-	+	-	-	-	-	-	-	-
2259	3105	FIG045374: Type II restriction enzyme, methylase subunit YeeA	11	-	-	-	-	-	-	-	-	-	-	-	-	-	-	-	-	-	-	-	+	-	-	+	+	+	+	+	+	+	+	+	+	-	-	-	-	-	-	-	-	-	-	-	-	-	-	-	-	-	-	-	-	-	-	-	-	-
2260	3106	unknown protein	11	-	-	-	-	-	-	-	-	-	-	-	-	-	-	-	-	-	-	-	-	-	-	+	+	+	+	+	+	+	+	-	-	-	-	-	-	-	-	-	-	-	+	-	-	-	+	+	-	-	-	-	-	-	-	-	-	-
2261	3110	hypothetical protein	11	-	-	-	-	-	-	-	-	-	-	-	-	-	-	-	-	-	-	-	-	-	-	-	-	+	+	+	+	+	+	-	-	-	-	-	-	-	-	-	-	+	+	+	-	-	-	+	+	-	-	-	-	-	-	-	-	-
2262	3111	hypothetical protein	11	-	-	-	-	-	-	-	-	-	-	-	-	-	-	-	+	-	-	-	-	+	+	+	+	+	-	+	+	+	+	-	-	-	-	-	-	-	-	-	-	-	-	+	-	-	-	-	-	-	-	-	-	-	-	-	-	-
2263	3113	hypothetical protein	11	-	-	-	-	-	-	-	-	-	-	-	-	-	-	-	-	-	-	-	-	-	-	+	+	+	+	+	+	+	+	-	-	-	-	-	-	-	-	-	-	-	+	-	-	-	+	+	-	-	-	-	-	-	-	-	-	-
2264	3114	hypothetical protein	11	-	-	-	-	-	-	-	-	-	-	-	-	-	-	-	-	-	-	-	-	-	-	-	-	+	+	+	+	+	+	-	-	-	-	-	-	-	-	-	-	+	+	+	-	-	-	+	+	-	-	-	-	-	-	-	-	-
2265	3120	Phage uncharacterized protein, C-terminal	11	-	-	-	-	+	-	-	-	-	-	-	-	-	-	-	-	-	-	-	+	+	+	+	-	-	-	-	+	-	-	-	-	+	-	-	-	-	+	+	-	-	-	-	-	-	-	-	-	-	-	-	-	+	-	-	+	-
2266	3127	putative amino acid transporter, amino acid-binding protein	11	-	-	-	-	-	-	-	-	-	-	-	-	-	-	-	-	-	-	-	+	+	+	+	+	-	-	-	-	-	-	-	-	+	+	+	+	+	+	-	-	-	-	-	-	-	-	-	-	-	-	-	-	-	-	-	-	-
2267	3128	hypothetical protein	11	-	-	-	-	-	-	-	-	-	-	-	-	-	+	-	-	-	-	-	+	+	+	+	+	-	-	-	-	-	-	+	+	+	+	+	-	-	-	-	-	-	-	-	-	-	-	-	-	-	-	-	-	-	-	-	-	-
2268	3130	putative amino acid transporter, amino acid-binding protein	11	-	-	-	-	-	-	-	-	-	-	-	-	-	-	-	-	-	-	-	+	+	+	+	+	-	-	-	-	-	-	-	-	+	+	+	+	+	+	-	-	-	-	-	-	-	-	-	-	-	-	-	-	-	-	-	-	-
2269	3131	Zinc metalloprotease zmpB precursor (EC 3.4.24.-)	11	-	-	-	-	-	-	-	-	-	-	-	-	-	-	-	+	-	-	-	-	-	-	-	+	-	-	+	-	+	+	-	-	+	+	+	+	-	+	-	-	-	-	+	-	-	-	-	-	-	-	-	-	-	-	-	-	-
2270	3134	Ser/Thr protein phosphatase family protein	11	-	-	-	-	-	-	-	-	-	-	-	-	-	-	-	-	-	-	-	-	-	-	-	-	-	-	-	-	-	-	+	+	-	-	-	-	-	+	-	-	-	-	-	-	-	-	-	-	-	+	+	+	+	+	+	+	+
2271	3149	Dihydrodipicolinate synthase (EC 4.2.1.52)	11	-	-	-	-	-	-	-	-	-	-	-	-	-	-	-	-	-	-	-	-	-	-	-	-	-	-	-	-	-	-	-	-	-	-	-	+	+	+	-	-	+	+	-	-	-	-	-	-	-	+	+	+	+	+	+	-	-
2272	3155	hypothetical protein	11	+	+	+	+	+	+	+	-	+	-	+	+	+	-	-	-	-	-	-	-	-	-	-	-	-	-	-	-	-	-	-	-	-	-	-	-	-	-	-	-	-	-	-	-	-	-	-	-	-	-	-	-	-	-	-	-	-
2273	3156	Polyribonucleotide nucleotidyltransferase (polynucleotide phosphorylase)	11	-	-	+	+	+	-	+	+	+	+	+	+	+	+	-	-	-	-	-	-	-	-	-	-	-	-	-	-	-	-	-	-	-	-	-	-	-	-	-	-	-	-	-	-	-	-	-	-	-	-	-	-	-	-	-	-	-
2274	3175	hypothetical protein	11	-	-	-	-	-	-	-	-	+	-	-	-	-	-	-	-	-	-	-	+	-	-	-	-	-	-	-	-	-	+	+	+	-	+	+	-	-	-	-	-	-	-	+	-	-	-	-	-	+	-	+	-	-	+	-	-	-
2275	1489	Aldose 1-epimerase (EC 5.1.3.3)	10	-	-	-	-	-	-	-	-	-	-	-	-	-	-	-	-	-	-	-	-	-	-	-	-	-	-	-	+	+	-	-	-	+	+	+	-	-	-	-	-	-	-	-	-	-	-	-	-	-	+	+	+	+	-	+	-	-
2276	1518	Fumarate reductase, flavoprotein subunit precursor (EC 1.3.99.1)	10	-	-	-	-	-	-	-	-	-	-	-	-	-	-	-	-	-	-	-	-	-	-	-	-	-	-	-	-	-	-	-	-	-	-	-	-	-	-	-	-	-	-	+	-	+	-	+	+	-	+	+	+	+	-	+	-	+
2277	1519	Mobile element protein	10	-	-	-	-	-	-	-	-	-	-	-	-	-	-	-	-	-	-	-	-	-	-	-	-	-	-	-	-	-	-	-	-	-	-	-	+	+	+	-	-	-	-	-	-	-	-	+	-	-	-	+	-	+	-	+	+	+	+	-
2278	1531	Competence-stimulating peptide ABC transporter ATP-binding protein ComA	10	-	-	-	-	-	-	-	-	-	-	-	-	-	-	-	-	-	-	-	+	+	+	-	-	-	-	-	-	-	-	-	-	+	-	+	-	-	-	+	+	-	-	-	-	-	-	-	-	+	+	+	-	-	-	-	-	-
2279	1574	Mobile element protein	10	-	-	-	-	-	-	-	-	-	-	-	-	-	-	-	-	-	-	-	-	-	-	-	-	-	-	-	-	-	-	-	-	-	-	-	+	+	+	-	-	-	-	-	-	-	-	+	-	-	-	+	-	+	-	+	+	+	+	-
2280	1856	Hypothetical protein DUF454	10	-	-	-	-	-	-	-	-	-	-	-	-	-	-	-	-	-	-	-	-	-	-	-	-	-	-	-	-	-	-	-	-	-	-	-	-	-	-	-	-	-	-	+	-	+	-	+	+	-	+	+	+	+	-	+	-	+
2281	1962	Phage protein	10	-	-	-	-	-	-	-	-	-	-	-	-	-	-	-	-	-	-	-	+	-	-	-	-	-	-	-	-	-	-	+	+	+	+	+	-	-	-	-	-	+	-	-	-	+	-	-	-	-	-	-	+	+	-	-	-	-
2282	1964	Positive transcriptional regulator, MutR family	10	-	-	-	-	-	-	-	-	-	-	-	-	-	-	-	-	-	-	-	-	-	-	-	-	-	-	-	-	-	-	+	+	+	+	+	+	+	+	-	-	-	-	-	-	-	-	-	-	-	-	-	-	-	+	+	-	-
2283	2149	Transposase	10	-	-	-	-	-	-	-	-	-	-	-	-	-	-	-	-	-	-	+	-	-	-	-	-	-	-	-	-	-	-	-	-	-	-	-	-	+	+	-	-	-	-	-	-	+	+	-	-	-	+	+	+	-	+	-	+	-
2284	2150	Acetyltransferase, GNAT family	10	-	-	-	-	-	-	-	-	-	-	-	-	-	-	-	-	-	-	+	-	-	-	+	+	-	-	-	-	-	-	-	-	-	-	-	-	-	-	-	-	-	-	-	-	-	-	-	+	-	+	-	+	+	+	+	-	+
2285	2185	Spermidine synthase (EC 2.5.1.16)	10	-	-	-	-	-	-	-	-	-	-	-	-	-	-	-	-	-	-	-	-	-	-	-	-	-	-	-	-	-	-	-	-	-	-	-	-	-	-	-	-	-	-	-	-	+	+	+	+	+	+	-	+	-	+	-	+	+
2286	2186	Arginine decarboxylase (EC 4.1.1.19)	10	-	-	-	-	-	-	-	-	-	-	-	-	-	-	-	-	-	-	-	-	-	-	-	-	-	-	-	-	-	-	-	-	-	-	-	-	-	-	-	-	-	-	-	-	+	+	+	+	+	+	-	+	-	+	-	+	+
2287	2241	Carboxynorspermidine dehydrogenase	10	-	-	-	-	-	-	-	-	-	-	-	-	-	-	-	-	-	-	-	-	-	-	-	-	-	-	-	-	-	-	-	-	-	-	-	-	-	-	-	-	-	-	-	-	+	+	+	+	+	+	-	+	-	+	-	+	+
2288	2242	Carboxynorspermidine decarboxylase	10	-	-	-	-	-	-	-	-	-	-	-	-	-	-	-	-	-	-	-	-	-	-	-	-	-	-	-	-	-	-	-	-	-	-	-	-	-	-	-	-	-	-	-	-	+	+	+	+	+	+	-	+	-	+	-	+	+
2289	2285	FtsK/SpoIIIE family protein, putative secretion system component EssC/YukA	10	-	-	-	-	-	-	-	-	-	-	-	-	-	-	-	-	-	-	-	-	-	-	-	-	+	+	+	+	+	+	-	-	-	-	-	-	-	-	-	-	+	+	-	-	-	-	+	-	+	-	-	-	-	-	-	-	-
2290	2324	NAD-dependent protein deacetylase of SIR2 family	10	-	-	-	-	-	-	-	-	-	-	-	-	-	-	-	+	+	+	+	-	-	-	-	-	-	-	-	-	-	-	-	-	-	-	-	-	-	-	-	-	-	-	-	+	+	+	+	+	-	-	-	-	-	+	-	-	-
2291	2325	Biotinyl-lipoyl attachment domain protein, GcvH-like	10	-	-	-	-	-	-	-	-	-	-	-	-	-	-	-	+	+	+	+	-	-	-	-	-	-	-	-	-	-	-	-	-	-	-	-	-	-	-	-	-	-	-	-	+	+	+	+	+	-	-	-	-	-	+	-	-	-
2292	2328	Probable NADH-dependent flavin oxidoreductase in cluster with COG2110	10	-	-	-	-	-	-	-	-	-	-	-	-	-	-	-	+	+	+	+	-	-	-	-	-	-	-	-	-	-	-	-	-	-	-	-	-	-	-	-	-	-	-	-	+	+	+	+	+	-	-	-	-	-	+	-	-	-
2293	2329	Lipoate-protein ligase A	10	-	-	-	-	-	-	-	-	-	-	-	-	-	-	-	+	+	+	+	-	-	-	-	-	-	-	-	-	-	-	-	-	-	-	-	-	-	-	-	-	-	-	-	+	+	+	+	+	-	-	-	-	-	+	-	-	-
2294	2380	hypothetical protein	10	-	-	-	-	-	-	-	-	-	-	-	-	-	-	-	-	-	-	-	-	+	+	+	-	+	-	+	+	-	+	+	-	+	-	-	-	-	+	-	-	-	-	-	-	-	-	-	-	-	-	-	-	-	-	-	-	-
2295	2442	FIG018599: Phage-associated protein	10	-	-	-	-	-	-	-	-	-	-	-	-	-	-	-	-	-	-	-	+	-	-	-	-	+	+	+	+	+	+	+	+	-	+	-	-	-	-	-	-	-	-	-	-	-	-	-	-	-	-	-	-	-	-	-	-	-
2296	2458	Phage protein	10	-	-	-	-	-	-	-	-	-	-	-	-	-	-	-	-	-	-	-	+	-	-	-	-	-	-	+	+	+	+	+	+	-	+	-	-	-	-	-	-	-	-	-	-	-	-	-	-	-	+	-	-	-	-	-	-	+
2297	2460	FIG018632: Phage-associated protein	10	-	-	-	-	-	-	-	-	-	-	-	-	-	-	-	-	-	-	-	+	-	-	-	-	-	-	+	+	+	+	+	+	-	+	-	-	-	-	-	-	-	-	-	-	-	-	-	-	-	+	-	-	-	-	-	-	+
2298	2462	FIG020717: Phage-associated protein	10	-	-	-	-	-	-	-	-	-	-	-	-	-	-	-	-	-	-	-	+	-	-	-	-	-	-	+	+	+	+	+	+	-	+	-	-	-	-	-	-	-	-	-	-	-	-	-	-	-	+	-	-	-	-	-	-	+
2299	2463	Phage major tail protein	10	-	-	-	-	-	-	-	-	-	-	-	-	-	-	-	-	-	-	-	+	-	-	-	-	-	-	+	+	+	+	+	+	-	+	-	-	-	-	-	-	-	-	-	-	-	-	-	-	-	+	-	-	-	-	-	-	+
2300	2465	FIG018217: Phage-associated protein	10	-	-	-	-	-	-	-	-	-	-	-	-	-	-	-	-	-	-	-	+	-	-	-	-	-	-	+	+	+	+	+	+	-	+	-	-	-	-	-	-	-	-	-	-	-	-	-	-	-	+	-	-	-	-	-	-	+
2301	2466	hypothetical protein	10	-	-	-	-	-	-	-	-	-	-	-	-	-	-	-	-	-	-	-	+	-	-	-	-	-	-	+	+	+	+	+	+	-	+	-	-	-	-	-	-	-	-	-	-	-	-	-	-	-	+	-	-	-	-	-	-	+
2302	2467	FIG017897: Phage protein	10	-	-	-	-	-	-	-	-	-	-	-	-	-	-	-	-	-	-	-	+	-	-	-	-	-	-	+	+	+	+	+	+	-	+	-	-	-	-	-	-	-	-	-	-	-	-	-	-	-	+	-	-	-	-	-	-	+
2303	2521	hypothetical protein	10	-	-	-	-	-	-	-	-	-	-	-	-	-	-	-	-	+	+	+	-	-	-	-	-	-	-	-	-	-	-	-	-	-	-	-	+	+	+	-	-	+	+	-	-	-	-	-	-	-	-	-	-	+	-	-	+	-
2304	2524	hypothetical protein	10	-	-	-	-	-	-	-	-	-	-	-	-	-	-	-	-	+	+	-	-	-	-	-	-	-	-	-	-	-	-	+	+	-	-	-	-	-	+	-	-	-	-	-	-	-	-	-	-	-	+	-	+	+	+	+	-	-
2305	2543	CDS_ID OB3334	10	-	-	-	-	-	-	-	-	-	-	-	-	-	-	-	+	-	-	-	-	-	-	-	-	-	-	-	-	-	-	-	-	-	-	-	-	-	-	-	-	+	+	-	-	-	+	+	-	+	+	-	+	+	-	+	-	-
2306	2609	Phage minor capsid protein	10	-	-	-	-	-	-	-	-	-	-	-	-	-	-	+	+	+	+	-	-	-	-	-	-	+	-	-	-	-	-	-	-	-	+	-	-	-	-	+	-	-	-	-	+	-	-	-	-	-	+	-	-	-	-	+	-	-
2307	2611	Phage minor capsid protein	10	-	-	-	-	-	-	-	-	-	-	-	-	-	-	+	+	+	+	-	-	-	-	-	-	+	-	-	-	-	-	-	-	-	+	-	-	-	-	+	-	-	-	-	+	-	-	-	-	-	+	-	-	-	-	+	-	-
2308	2737	conserved domain protein	10	-	-	-	-	-	-	-	-	-	-	-	-	-	-	-	-	+	-	-	-	-	-	-	-	-	-	-	+	-	-	-	-	-	-	-	+	+	-	+	+	-	-	-	-	-	-	-	-	-	-	-	-	+	+	-	+	-	-	+
2309	2741	hypothetical protein	10	-	-	-	-	-	-	-	-	-	-	-	-	-	-	-	-	+	+	-	-	-	-	-	-	-	-	-	-	-	-	-	-	-	-	-	-	-	-	-	-	-	-	-	-	-	+	-	-	+	-	+	+	+	-	+	+	+
2310	2748	Site-specific recombinase	10	-	-	-	-	-	-	-	-	-	-	+	-	-	-	-	+	-	-	-	-	-	-	+	-	-	-	-	-	-	-	-	-	-	-	-	-	-	+	+	+	+	-	-	-	+	-	+	+	-	-	-	-	-	-	-	-	-
2311	2858	Unknown	10	-	-	-	-	-	-	-	-	-	-	-	-	-	-	-	+	-	-	-	+	+	+	-	+	+	-	+	+	+	+	-	-	-	-	-	-	-	-	-	-	-	-	-	-	-	-	-	-	-	-	-	-	-	-	-	-	-
2312	2862	Zinc metalloprotease zmpC precursor (EC 3.4.24.-) (MMP-9 protease)	10	-	-	-	-	-	-	-	-	-	-	-	-	-	-	-	-	-	-	-	-	-	-	-	-	+	+	-	-	+	+	+	+	-	-	-	-	-	-	-	-	-	-	-	-	-	-	-	-	-	+	-	-	-	+	+	+	-
2313	2869	FtsK protein	10	-	-	-	-	-	-	-	-	-	-	-	-	-	-	-	-	-	-	-	-	-	-	+	+	+	+	-	-	-	-	+	+	-	+	-	-	+	-	+	-	+	-	-	-	-	-	-	-	-	-	-	-	-	-	-	-	-
2314	2877	hypothetical protein	10	-	-	-	-	-	-	-	-	-	-	-	-	-	-	-	-	-	-	-	-	-	-	-	-	-	-	-	-	-	-	-	-	-	-	-	+	+	+	+	+	-	-	-	-	+	-	-	-	-	-	-	+	+	+	-	-	+
2315	2879	Phage integrase	10	-	-	-	-	-	-	-	-	-	-	-	-	-	-	-	-	-	-	-	-	-	-	-	-	-	-	-	-	-	-	-	-	-	-	+	+	+	-	+	+	-	-	-	-	-	-	+	-	-	+	-	+	-	+	+	-	-
2316	2919	hypothetical protein	10	-	-	-	-	-	-	-	-	-	-	-	-	-	-	-	-	-	-	-	+	+	+	+	-	+	+	+	-	+	+	-	-	+	-	-	-	-	-	-	-	-	-	-	-	-	-	-	-	-	-	-	-	-	-	-	-	-
2317	2975	hypothetical protein	10	-	-	-	-	-	-	-	-	-	-	-	-	-	-	-	+	+	+	-	-	-	-	-	-	-	-	-	-	-	-	-	-	-	-	-	-	-	-	-	-	+	+	-	+	-	-	-	-	-	-	-	+	+	+	-	-	+
2318	2977	RloF	10	-	-	-	-	-	-	-	-	-	-	-	-	-	-	-	+	+	+	-	-	-	-	-	-	-	-	-	-	-	-	-	-	-	-	-	-	-	-	-	-	+	+	-	+	-	-	-	-	-	-	-	+	+	+	-	-	+
2319	2987	Hypothetical protein	10	-	-	-	-	-	-	-	-	-	-	-	-	-	-	+	-	-	-	-	-	-	-	-	-	+	-	-	-	-	-	-	-	-	+	+	-	-	-	+	-	-	-	-	-	+	-	+	-	-	+	-	-	-	+	+	-	-
2320	2998	hypothetical protein	10	-	-	-	-	-	-	-	-	-	-	+	-	-	-	-	+	-	-	-	-	-	-	+	-	-	-	-	-	-	-	-	-	-	-	-	-	-	+	+	+	+	-	-	-	+	-	+	+	-	-	-	-	-	-	-	-	-
2321	3005	Translation initiation factor 2 (IF-2; GTPase)	10	-	-	-	+	-	-	-	-	+	-	-	-	-	-	-	-	-	-	-	-	-	-	-	-	-	-	-	-	+	-	-	-	-	-	-	+	-	-	-	+	+	+	-	-	-	-	-	+	+	-	-	-	-	-	-	-	+
2322	3023	Glutamate transport ATP-binding protein	10	-	-	-	-	-	-	-	-	-	-	-	-	-	-	-	-	-	-	-	-	-	-	-	-	-	-	-	-	-	-	-	-	-	-	-	-	-	-	-	-	+	-	-	-	-	-	+	-	-	+	+	+	+	+	+	+	+
2323	3024	FIG022125: Phage-associated protein	10	-	-	-	-	-	-	-	-	-	-	-	-	-	-	-	-	-	-	-	+	-	-	-	-	-	-	+	+	+	+	+	+	-	+	-	-	-	-	-	-	-	-	-	-	-	-	-	-	-	+	-	-	-	-	-	-	+
2324	3044	hypothetical protein	10	-	-	-	-	+	-	-	-	-	-	-	-	-	-	-	-	-	-	-	-	-	-	-	-	-	-	-	-	-	-	+	+	-	+	-	+	-	-	-	-	-	-	-	+	-	-	-	-	-	-	+	+	+	-	+	-	-
2325	3045	hypothetical protein	10	-	-	-	-	+	-	-	-	-	-	-	-	-	-	-	-	-	-	-	+	-	-	-	-	-	-	-	-	-	-	+	+	-	+	-	+	-	-	-	-	-	-	-	-	-	-	-	-	-	-	+	+	+	-	+	-	-
2326	3051	DNA alkylation repair enzyme	10	+	+	+	-	+	+	-	+	+	-	+	+	-	+	-	-	-	-	-	-	-	-	-	-	-	-	-	-	-	-	-	-	-	-	-	-	-	-	-	-	-	-	-	-	-	-	-	-	-	-	-	-	-	-	-	-	-
2327	3096	Luciferase-like monooxygenase (EC 1.14.-.-) in cluster with COG2110	10	-	-	-	-	-	-	-	-	-	-	-	-	-	-	-	+	+	+	+	-	-	-	-	-	-	-	-	-	-	-	-	-	-	-	-	-	-	-	-	-	-	-	-	+	+	+	+	+	-	-	-	-	-	+	-	-	-
2328	3101	putative sporulation protein (partial match)	10	-	-	-	-	-	-	-	-	-	-	-	-	-	-	-	+	+	+	-	-	-	-	-	-	-	-	-	-	-	-	-	-	-	-	-	-	-	-	-	-	+	+	-	+	-	-	-	-	-	-	-	+	+	+	-	-	-	+
2329	3102	COG2110, Macro domain, possibly ADP-ribose binding module	10	-	-	-	-	-	-	-	-	-	-	-	-	-	-	-	+	+	+	+	-	-	-	-	-	-	-	-	-	-	-	-	-	-	-	-	-	-	-	-	-	-	-	-	+	+	+	+	+	-	-	-	-	-	+	-	-	-
2330	3108	ORF049	10	-	-	-	-	-	-	-	-	-	-	-	-	-	-	-	-	-	-	-	+	-	-	+	-	+	-	-	-	-	-	+	+	-	+	-	-	-	+	-	-	+	+	-	-	-	-	-	-	-	-	-	-	-	-	-	+	-
2331	3112	Helicase loader DnaI	10	-	-	-	-	-	-	-	-	-	-	-	-	-	-	+	-	-	-	-	-	-	-	+	-	+	-	-	-	-	-	-	-	+	-	+	-	+	+	+	+	-	-	-	-	-	-	-	-	-	-	-	-	-	-	-	-	+
2332	3115	Oligopeptide ABC transporter, periplasmic oligopeptide-binding protein OppA (TC 3.A.1.5.1)	10	-	-	-	-	-	-	-	-	-	-	-	-	-	-	-	-	-	-	-	-	-	-	-	-	+	+	-	-	-	-	-	-	-	-	-	+	+	+	-	-	-	-	-	-	-	-	-	-	-	+	-	+	+	+	+	-	-
2333	3121	hypothetical protein	10	-	-	-	-	+	-	-	-	-	-	-	-	-	-	-	-	-	-	+	+	+	+	+	-	-	-	-	+	-	-	-	-	+	-	-	-	-	-	+	-	-	-	-	-	-	-	-	-	-	-	-	-	-	-	-	+	-
2334	3123	hypothetical protein	10	-	-	-	-	-	-	-	-	-	-	-	-	-	-	-	-	-	-	-	-	-	-	-	-	-	-	-	-	-	-	-	-	-	-	-	+	+	-	+	+	-	+	-	-	+	+	-	+	-	-	-	-	-	+	+	-	-
2335	3150	hypothetical protein	10	-	-	-	-	-	-	-	-	-	-	-	-	-	-	-	-	-	-	-	-	-	-	-	-	-	-	+	+	+	-	+	+	-	-	-	+	+	+	-	-	+	-	-	-	-	-	-	-	-	-	-	+	-	-	-	-	-
2336	3170	spbA protein	10	-	-	-	-	+	-	-	-	-	-	-	-	-	-	-	-	-	-	-	-	-	-	-	-	-	-	+	+	+	+	+	+	-	+	-	-	-	-	-	-	-	-	-	-	-	-	-	-	-	-	-	+	+	-	-	-	-
2337	3239	Metallo-beta-lactamase superfamily protein PA0057	10	-	-	-	-	-	-	-	-	-	-	-	-	-	-	-	-	+	+	-	-	-	-	-	-	-	-	-	-	-	-	-	-	-	-	-	-	-	-	-	-	-	-	-	-	-	-	+	-	+	+	+	+	+	+	+	-	-
2338	3246	hypothetical protein	10	-	-	-	-	-	-	-	-	-	-	-	-	-	-	-	-	+	-	-	-	-	-	-	-	-	-	-	-	-	-	-	-	-	-	+	+	+	-	-	-	-	-	-	-	+	-	-	-	-	+	+	+	-	+	+	-	-
2339	3249	hypothetical protein	10	-	-	-	-	-	-	-	-	-	-	-	-	-	-	-	-	+	+	-	-	-	-	-	-	-	-	-	-	-	-	-	-	-	-	-	-	-	-	-	-	+	+	-	+	-	-	-	+	-	-	-	+	+	+	-	-	+
2340	3250	hypothetical protein	10	-	-	-	-	-	-	-	-	-	-	-	-	-	-	-	-	-	-	-	-	-	-	-	-	+	+	+	+	+	+	-	-	-	-	-	-	-	-	-	-	-	-	+	-	+	-	+	+	-	-	-	-	-	-	-	-	-
2341	3252	hypothetical protein	10	-	-	-	-	-	-	-	-	-	-	-	-	-	-	-	-	-	-	-	+	+	+	-	-	+	+	+	+	+	+	-	-	-	-	-	-	-	-	-	-	-	-	-	-	-	-	-	+	-	-	-	-	-	-	-
2342	3258	Phage integrase: site-specific recombinase	10	-	-	-	-	-	-	-	-	-	-	-	-	-	-	+	-	-	-	-	+	+	+	-	-	+	+	+	+	+	+	-	-	-	-	-	-	-	-	-	-	-	-	-	-	-	-	-	-	-	-	-	-	-	-	-
2343	3261	hypothetical protein	10	-	-	-	-	-	-	-	-	-	-	-	-	-	-	-	-	-	-	-	-	-	-	-	-	+	+	+	+	+	+	-	-	-	+	-	-	-	-	+	-	-	-	-	-	-	-	-	-	-	+	-	-	+	-	-	-
2344	3263	hypothetical protein	10	-	-	-	-	-	-	-	-	-	-	-	-	-	-	-	-	-	-	-	-	-	-	-	+	-	-	+	-	-	-	-	-	+	+	+	+	-	+	+	+	-	-	-	-	-	-	-	-	-	+	-	-	-	-	-	-	-
2345	3265	hypothetical protein	10	-	-	-	-	-	-	-	+	-	-	-	-	-	-	+	-	-	-	-	+	+	+	+	-	-	-	-	-	-	-	-	-	+	-	+	-	-	-	+	+	-	-	-	-	-	-	-	-	-	-	-	-	-	-	-	-	-
2346	3268	ORF26	10	-	-	-	-	-	-	-	-	-	-	-	-	-	-	-	-	-	-	-	+	+	+	-	-	-	-	-	+	-	-	-	-	+	-	+	-	-	+	-	-	-	-	-	-	+	-	-	-	-	-	+	-	-	-	-	+	-
2347	3281	Periaxin	10	-	-	-	+	+	-	-	-	-	-	-	-	+	-	-	-	-	-	-	-	-	-	+	-	-	-	-	-	-	-	-	-	+	+	+	+	+	-	-	-	-	-	+	-	-	-	-	-	-	-	-	-	-	-	-	-	-
2348	3282	ORF012	10	-	-	-	-	+	-	-	-	-	-	-	-	-	-	-	-	-	-	-	+	+	+	+	-	-	-	-	+	-	-	-	-	+	-	-	-	-	+	-	-	-	-	-	-	-	-	-	-	-	-	-	-	-	+	-	+	-
2349	3283	putative conjugative transposon mobilization protein	10	-	-	-	-	-	-	-	-	-	-	-	-	-	-	-	+	-	-	-	-	-	-	-	+	-	-	+	-	+	+	-	-	+	-	-	+	-	+	-	-	-	-	+	-	-	-	-	-	-	+	-	-	-	-	-	-	-
2350	3284	hypothetical protein	10	-	-	-	-	-	-	-	-	-	-	-	-	-	-	-	+	-	-	-	-	-	-	-	-	-	-	-	-	-	-	-	-	-	-	-	-	-	-	-	-	+	+	-	+	-	-	-	+	-	-	+	+	+	+	-	-	+
2351	3297	hypothetical protein	10	+	+	-	-	-	-	-	-	-	-	-	-	-	-	-	-	-	-	+	-	-	-	-	-	-	-	-	-	-	-	-	-	-	-	-	+	-	-	-	-	-	-	+	+	-	-	-	+	-	+	+	-	+	-	-	-	-
2352	3318	hypothetical protein	10	-	-	-	-	-	-	-	-	-	-	-	-	-	-	-	-	-	-	-	-	-	-	-	-	-	-	-	-	-	-	+	+	+	+	+	+	+	+	-	-	-	-	-	-	-	-	-	-	-	-	-	-	-	+	+	-	-
2353	3349	hypothetical protein	10	+	+	+	+	-	+	+	+	+	+	+	-	-	-	-	-	-	-	-	-	-	-	-	-	-	-	-	-	-	-	-	-	-	-	-	-	-	-	-	-	-	-	-	-	-	-	-	-	-	-	-	-	-	-	-	-	-
2354	3350	extracellular factor protein	10	+	+	+	+	+	+	+	+	+	+	-	-	-	-	-	-	-	-	-	-	-	-	-	-	-	-	-	-	-	-	-	-	-	-	-	-	-	-	-	-	-	-	-	-	-	-	-	-	-	-	-	-	-	-	-	-	-
2355	3351	Hyaluronate lyase precursor (EC 4.2.2.1)	10	+	+	+	+	+	+	+	+	+	+	-	-	-	-	-	-	-	-	-	-	-	-	-	-	-	-	-	-	-	-	-	-	-	-	-	-	-	-	-	-	-	-	-	-	-	-	-	-	-	-	-	-	-	-	-	-	-
2356	3352	hypothetical protein	10	+	+	+	+	+	+	-	+	-	-	-	-	-	+	-	-	-	-	-	-	-	-	-	-	-	-	-	-	-	-	+	+	-	-	-	-	-	-	-	-	-	-	-	-	-	-	-	-	-	-	-	-	-	-	-	-	-
2357	3353	Hypothetical cytosolic protein	10	+	+	+	+	+	+	-	+	-	-	-	-	-	+	-	-	-	-	-	-	-	-	-	-	-	-	-	-	-	-	+	+	-	-	-	-	-	-	-	-	-	-	-	-	-	-	-	-	-	-	-	-	-	-	-	-	-
2358	3354	putative replication initiator protein	10	+	+	+	+	+	+	-	+	-	-	-	-	-	+	-	-	-	-	-	-	-	-	-	-	-	-	-	-	-	-	+	+	-	-	-	-	-	-	-	-	-	-	-	-	-	-	-	-	-	-	-	-	-	-	-	-	-
2359	3355	DNA alkylation repair enzyme	10	+	+	+	-	+	+	-	+	+	-	+	+	-	+	-	-	-	-	-	-	-	-	-	-	-	-	-	-	-	-	-	-	-	-	-	-	-	-	-	-	-	-	-	-	-	-	-	-	-	-	-	-	-	-	-	-	-
2360	3356	hypothetical protein	10	+	+	+	+	+	+	-	+	-	-	-	-	-	+	-	-	-	-	-	-	-	-	-	-	-	-	-	-	-	-	+	+	-	-	-	-	-	-	-	-	-	-	-	-	-	-	-	-	-	-	-	-	-	-	-	-	-
2361	3357	hypothetical protein	10	+	+	+	+	+	+	-	+	-	-	-	-	-	+	-	-	-	-	-	-	-	-	-	-	-	-	-	-	-	-	+	+	-	-	-	-	-	-	-	-	-	-	-	-	-	-	-	-	-	-	-	-	-	-	-	-	-
2362	3358	Predicted dehydrogenase and related proteins	10	+	+	+	-	+	+	-	+	+	-	+	+	-	+	-	-	-	-	-	-	-	-	-	-	-	-	-	-	-	-	-	-	-	-	-	-	-	-	-	-	-	-	-	-	-	-	-	-	-	-	-	-	-	-	-	-	-
2363	3359	hypothetical protein	10	+	+	+	+	+	+	-	+	-	-	-	-	-	+	-	-	-	-	-	-	-	-	-	-	-	-	-	-	-	-	+	+	-	-	-	-	-	-	-	-	-	-	-	-	-	-	-	-	-	-	-	-	-	-	-	-	-
2364	1719	Lactose phosphotransferase system repressor	9	-	-	-	-	-	-	-	-	-	-	+	-	-	-	-	-	-	-	-	+	+	+	-	-	-	-	-	-	-	-	-	-	+	-	+	-	-	-	+	+	-	-	-	-	+	-	-	-	-	-	-	-	-	-	-	-	-
2365	1870	Poly(glycerol-phosphate) alpha-glucosyltransferase GftA (EC 2.4.1.52)	9	-	-	-	-	-	-	-	-	-	-	-	-	-	-	-	-	-	-	-	-	-	-	-	-	-	-	-	-	-	-	-	-	-	-	-	+	+	+	-	-	-	-	-	-	-	-	-	-	-	-	+	-	+	+	+	+	+
2366	1904	Mobile element protein	9	-	-	-	-	-	-	-	-	-	-	-	-	-	-	-	-	-	-	-	-	+	+	+	-	+	-	+	+	-	+	-	-	+	-	-	-	-	+	-	-	-	-	-	-	-	-	-	-	-	-	-	-	-	-	-	-	-
2367	1922	Protein export cytoplasm protein SecA2 ATPase RNA helicase (TC 3.A.5.1.1)	9	-	-	-	-	-	-	-	-	-	-	-	-	-	-	-	-	-	-	-	-	-	-	-	-	-	-	-	-	-	-	-	-	-	-	-	+	+	+	-	-	-	-	-	-	-	-	-	-	-	-	+	-	+	+	+	+	+
2368	2244	Transcriptional regulator, PadR family	9	-	-	-	-	-	-	-	-	-	-	-	-	-	-	-	+	-	-	-	-	-	-	-	-	-	-	-	-	-	-	-	-	-	-	-	-	-	-	-	-	-	-	-	+	+	-	-	-	+	+	+	+	-	+	+	-	-
2369	2290	Agmatine deiminase (EC 3.5.3.12)	9	-	-	-	-	-	-	-	-	-	-	-	-	-	-	-	-	-	-	-	-	-	-	-	-	-	-	-	-	-	-	-	-	-	-	-	-	-	-	-	-	-	-	-	-	+	+	+	+	+	+	-	+	-	+	-	-	+
2370	2291	N-carbamoylputrescine amidase (3.5.1.53) / Omega amidase (Nit2 homolog)	9	-	-	-	-	-	-	-	-	-	-	-	-	-	-	-	-	-	-	-	-	-	-	-	-	-	-	-	-	-	-	-	-	-	-	-	-	-	-	-	-	-	-	-	-	+	+	+	+	+	+	-	+	-	+	-	-	+
2371	2350	Mobile element protein	9	-	-	-	-	-	-	-	-	-	-	-	-	-	-	-	-	-	-	-	+	+	+	+	+	-	-	-	-	-	-	-	-	-	+	+	-	-	-	-	-	+	+	-	-	-	-	-	-	-	-	-	-	-	-	-	-	-
2372	2430	hypothetical protein	9	-	-	-	-	-	-	-	-	+	-	-	-	-	-	-	-	+	-	-	-	-	-	-	-	-	-	-	-	-	-	-	-	-	-	-	+	+	-	+	-	-	-	-	-	+	-	-	-	+	-	+	-	-	+	-	-	-
2373	2457	Unknown	9	-	-	-	-	-	-	-	-	-	-	-	-	-	-	-	+	-	-	-	+	-	-	-	+	-	-	-	+	+	-	-	-	-	-	-	-	-	-	-	-	-	+	-	-	-	-	+	+	+	-	-	-	-	-	-	-	-
2374	2520	Prophage pi2 protein 39	9	-	-	-	-	-	-	-	-	+	-	-	-	-	-	-	-	+	-	-	-	-	-	-	-	-	-	-	-	-	-	-	-	-	-	-	+	+	-	+	-	-	-	-	-	+	-	-	-	+	-	+	-	-	+	-	-	-
2375	2558	Two-component response regulator	9	-	-	-	-	-	-	-	-	-	-	-	-	-	-	-	-	-	-	-	+	+	+	-	-	-	-	-	-	+	-	-	-	+	-	-	-	-	-	-	-	-	-	-	-	-	-	-	-	-	+	-	+	-	-	+	+	-
2376	2693	Microcin C7 self-immunity protein mccF	9	+	+	+	-	+	+	-	+	+	-	+	+	-	-	-	-	-	-	-	-	-	-	-	-	-	-	-	-	-	-	-	-	-	-	-	-	-	-	-	-	-	-	-	-	-	-	-	-	-	-	-	-	-	-	-	-	-
2377	2736	Cell wall surface anchor family protein	9	-	-	-	-	-	-	-	-	-	-	-	-	-	-	+	+	+	+	+	-	-	-	-	-	-	-	-	-	-	-	-	-	-	-	-	-	-	-	+	+	-	-	-	+	+	-	-	-	-	-	-	-	-	-	-	-	-
2378	2749	hypothetical protein	9	-	-	-	-	-	-	-	-	-	-	+	-	-	-	-	-	-	-	-	+	+	+	-	-	-	-	-	-	-	-	-	-	+	-	+	-	-	-	+	+	-	-	-	-	+	-	-	-	-	-	-	-	-	-	-	-	-
2379	2863	hypothetical protein	9	-	-	-	-	-	-	-	-	-	-	-	-	-	-	-	-	-	-	-	-	-	-	+	+	+	+	+	+	+	+	-	-	-	-	+	-	-	-	-	-	-	-	-	-	-	-	-	-	-	-	-	-	-	-	-
2380	2865	Phage holin	9	-	-	-	-	-	-	-	-	-	-	-	-	-	-	+	-	-	-	+	-	-	-	-	-	+	-	-	-	-	-	-	-	-	+	+	-	-	-	+	-	-	-	-	-	-	-	-	-	-	-	+	-	-	+	+	-	-
2381	2885	hypothetical protein	9	-	-	-	-	-	-	-	-	-	-	-	-	-	-	-	-	-	-	-	-	-	-	+	-	-	-	-	-	-	-	+	+	+	+	-	+	+	+	-	-	-	-	-	-	-	-	+	-	-	-	-	-	-	-	-	-	-
2382	2904	hypothetical protein	9	-	-	-	-	-	-	-	-	-	-	-	-	-	-	-	-	-	-	-	-	-	-	-	-	-	-	+	+	+	-	+	+	-	-	-	-	-	-	-	-	+	-	-	-	-	-	-	-	-	-	+	+	-	-	+	-	-
2383	2982	Hypothetical protein	9	-	-	-	-	-	-	-	-	-	-	-	-	-	-	-	-	-	-	-	-	-	-	-	-	+	+	+	+	+	+	-	-	-	-	-	-	-	-	+	-	-	-	-	-	-	+	-	-	+	-	-	-	-	-	-	-	-
2384	2983	hypothetical protein	9	-	-	-	-	-	-	-	-	-	-	-	-	-	-	-	-	-	-	-	-	-	-	-	-	+	+	+	+	+	+	-	-	-	-	-	-	-	-	+	-	-	-	-	-	-	+	-	-	+	-	-	-	-	-	-	-	-
2385	2984	putative replication protein	9	-	-	-	-	-	-	-	-	-	-	-	-	-	-	-	-	-	-	-	-	-	-	-	-	+	+	+	+	+	+	-	-	-	-	-	-	-	-	+	-	-	-	-	-	-	+	-	-	+	-	-	-	-	-	-	-	-
2386	2988	Phage infection protein	9	-	-	-	-	-	-	-	-	-	-	-	-	-	-	-	-	-	-	-	-	-	-	-	-	+	+	+	+	+	+	-	-	-	-	+	-	-	-	+	+	-	-	-	-	-	-	-	-	-	-	-	-	-	-	-	-	-
2387	2990	hypothetical protein	9	-	-	-	-	-	-	-	-	-	-	-	-	-	-	-	-	-	-	-	-	-	-	-	-	+	+	+	+	+	+	-	-	-	-	-	-	-	-	+	-	-	-	-	-	-	+	-	-	+	-	-	-	-	-	-	-	-
2388	2995	phage protein	9	-	-	-	-	-	-	-	-	-	-	-	-	-	-	-	-	-	-	-	-	-	-	-	-	-	-	-	-	-	-	-	-	-	-	-	+	+	-	+	+	-	-	-	-	-	-	-	-	-	+	+	-	+	+	+	-	-
2389	3022	MutR	9	-	-	-	-	-	-	-	-	-	-	-	-	-	-	-	-	-	-	-	-	-	-	-	-	-	-	-	-	-	-	-	-	-	-	-	-	-	-	-	-	-	-	-	-	+	-	-	-	-	+	+	+	+	+	+	+	+
2390	3025	ABC transporter, ATP-binding protein	9	-	-	-	-	-	-	-	-	-	-	-	-	-	-	-	-	-	-	-	-	-	-	-	-	-	-	-	-	-	-	-	-	-	-	-	-	-	-	-	-	-	-	-	-	+	-	-	-	-	+	+	+	+	+	+	+	+
2391	3104	Multi antimicrobial extrusion protein MatE	9	-	-	-	-	-	-	-	-	-	-	-	-	-	-	-	-	+	+	-	-	-	-	+	-	-	-	-	-	-	-	-	-	-	-	-	-	+	+	-	-	-	-	-	-	-	-	-	-	-	-	+	+	+	-	+	-	-
2392	3119	Site-specific recombinase	9	-	-	-	-	-	-	-	-	-	-	+	-	-	-	-	+	-	-	-	-	-	-	+	-	-	-	-	-	-	-	-	-	-	-	-	-	-	+	+	-	+	-	-	-	+	-	+	+	-	-	-	-	-	-	-	-	-
2393	3124	FIG021474: Phage protein	9	-	-	-	-	-	-	-	-	-	-	-	-	-	-	+	-	-	-	-	-	-	-	-	-	-	-	-	-	-	-	-	-	-	-	-	-	-	-	+	-	-	-	-	-	-	+	-	-	+	+	+	-	+	+	+	-
2394	3133	hypothetical protein	9	-	-	-	-	-	-	-	-	-	-	-	-	-	-	-	-	-	-	-	-	-	-	-	-	-	-	-	-	-	-	-	-	-	-	-	-	-	-	-	-	-	-	-	-	+	-	-	-	-	+	+	+	+	+	+	+	+
2395	3168	hypothetical protein	9	-	-	-	-	-	-	-	-	-	-	-	-	-	-	-	-	-	-	-	+	-	-	-	-	-	-	-	-	-	-	+	+	-	+	-	-	-	-	-	-	-	-	-	+	-	-	-	-	-	-	+	+	+	-	+	-	-
2396	3240	hypothetical protein	9	-	-	-	-	-	-	-	-	-	-	-	-	-	-	-	-	+	-	+	+	+	+	-	-	-	-	-	+	-	-	-	-	+	-	+	-	-	-	-	-	-	-	-	-	+	-	-	-	-	-	-	-	-	-	-	-	-
2397	3244	putative transposase	9	-	-	-	-	-	-	-	-	-	-	-	-	-	-	-	-	-	-	-	-	-	-	+	-	-	-	-	+	+	-	+	+	-	-	-	-	-	+	-	-	+	-	-	-	-	-	-	-	-	-	-	+	-	+	-	-	-
2398	3247	putative minor capsid protein 3	9	-	-	-	-	-	-	-	-	-	-	-	-	-	-	-	+	+	+	-	-	-	-	-	-	+	-	-	-	-	-	-	-	-	+	-	-	-	-	+	-	-	-	+	-	-	-	-	+	-	-	-	-	+	-	-

Table S5. Precence/absence of each HG among 57 *S. suis* strains

				1																13			25					28						104		94			76			53/54		Divergent *S. suis*																
Index[a]	ClutserID	Gene products	Members	98HAH33	05ZYH33	SC84	A7	SS12	P1/7	DAT273	NIAH11434	JS14	GZ1	BM407	S735	13730	MNCM07	NCTC 10446	14636	NCTC 10237	ST1	2651	89-1591	MNCM04	MNCM25	8074	D9	4961	ST3	MNCM43	DAT292	DAT260	NIAH11435	MNCM21	MNCM50	89-2479	DAT234	DAT290	93A	42A	NT77	6407	11538	8830	12814	92-1400	89-590	2726	2524	89-3576-3	4417	D12	10581	89-5259	88-5299A	R61	92-1191	14A	92-4172	22083
2399	3251	hypothetical protein	9	-	-	-	-	-	-	-	-	-	-	-	-	-	-	+	-	-	-	-	-	-	-	-	-	+	-	-	-	-	-	-	-	-	+	+	-	-	-	+	-	-	-	-	-	+	-	-	-	-	+	-	-	-	+	+	-	-
2400	3262	hypothetical protein	9	-	-	-	-	-	-	-	-	-	-	-	-	-	-	-	-	-	-	-	+	-	-	+	+	+	+	+	+	-	+	-	-	-	-	-	-	-	-	-	-	-	-	-	-	-	-	-	-	-	-	+	-	-	-	-	-	-
2401	3266	2-haloalkanoic acid dehalogenase (EC 3.8.1.2)	9	-	-	-	-	-	-	-	-	-	-	+	-	-	-	-	-	-	-	-	+	+	+	-	-	-	-	-	-	-	-	-	-	+	-	+	-	-	-	+	+	-	-	-	-	+	-	-	-	-	-	-	-	-	-	-	-	-
2402	3277	transcriptional regulator PlcR, putative	9	-	-	-	-	-	-	-	-	-	-	-	-	-	-	-	-	-	-	-	+	+	+	+	+	-	-	-	-	-	-	-	-	-	-	-	-	-	-	-	-	-	-	-	-	-	-	-	-	-	-	-	-	-	+	+	-	-
2403	3280	hypothetical protein	9	-	-	-	-	-	-	-	-	-	-	-	-	-	-	-	-	-	-	-	+	+	+	+	+	-	-	-	-	-	-	-	-	-	-	-	+	+	-	-	-	-	-	-	-	-	-	-	-	-	-	-	-	-	+	+	-	-
2404	3285	Glycosyl transferase, family 8	9	-	-	-	-	-	-	-	-	-	-	-	-	-	-	-	-	-	-	-	-	-	-	-	-	-	-	-	-	-	-	-	-	-	-	-	+	+	+	-	-	-	-	-	-	-	-	-	-	+	+	+	-	-	+	+	-	+
2405	3287	hypothetical protein	9	-	-	-	-	-	-	-	-	-	-	-	-	-	-	-	-	-	-	-	-	-	-	-	-	-	-	-	-	-	-	-	-	-	-	-	-	-	-	-	-	-	-	-	-	-	-	-	+	-	+	+	+	+	+	+	+	+
2406	3288	ABC-type multidrug transport system	9	-	-	-	-	-	-	-	-	-	-	-	-	-	-	-	-	-	-	-	-	-	-	-	-	-	-	-	-	-	-	-	-	-	-	-	-	-	-	-	-	-	-	-	-	-	-	-	+	-	+	+	+	+	+	+	+	+
2407	3289	hypothetical protein	9	-	-	-	-	-	-	-	-	-	-	-	-	-	-	-	-	-	-	-	-	-	-	-	-	-	-	-	-	-	-	-	-	-	-	-	-	-	-	-	-	-	-	-	-	-	-	-	+	-	+	+	+	+	+	+	+	+
2408	3303	similar to Abortive infection bacteriophage resistance protein	9	-	-	-	-	-	-	-	-	-	-	-	-	-	-	-	-	-	-	-	+	+	+	-	-	-	-	-	-	-	-	-	-	+	-	+	-	-	+	-	-	+	-	-	-	-	-	+	-	-	-	-	-	+	-	-	-	-
2409	3420	Fibronectin-binding protein	9	-	-	-	-	-	-	-	-	-	-	-	-	-	-	+	+	+	+	+	-	-	-	-	-	-	-	-	-	-	-	-	-	-	-	-	-	-	-	+	+	-	-	-	+	+	-	-	-	-	-	-	-	-	-	-	-	-
2410	3422	5'-nucleotidase (EC 3.1.3.5)	9	-	-	-	-	-	-	-	-	-	-	-	-	-	-	-	-	+	+	-	-	-	-	-	-	-	-	-	-	-	-	-	-	-	-	-	-	-	-	-	-	-	-	+	+	-	-	-	-	-	+	-	+	+	-	+	-	+
2411	3424	hypothetical protein	9	-	-	-	-	-	-	-	-	-	-	-	-	-	-	+	-	+	+	-	-	-	-	-	-	-	-	-	-	-	-	-	-	+	+	+	+	-	+	-	-	-	-	-	-	+	-	-	-	-	-	-	-	-	-	-	-	-
2412	3427	putative DNA-binding phage protein	9	-	-	-	-	-	-	-	-	-	-	-	-	-	-	-	-	-	-	-	+	+	+	-	-	+	+	+	+	+	+	-	-	-	-	-	-	-	-	-	-	-	-	-	-	-	-	-	-	-	-	-	-	-	-	-	-	-
2413	3429	Glycerol uptake facilitator protein	9	-	-	-	-	-	-	-	-	-	-	-	-	-	-	-	-	-	-	-	+	-	-	+	+	+	+	+	+	+	+	-	-	-	-	-	-	-	-	-	-	-	-	-	-	-	-	-	-	-	-	-	-	-	-	-	-	-
2414	3430	hypothetical protein	9	-	-	-	-	-	-	-	-	-	-	-	-	-	-	-	-	-	-	-	-	-	-	-	-	+	+	+	+	+	+	-	-	-	-	-	-	-	-	-	-	-	-	+	-	-	-	+	+	-	-	-	-	-	-	-	-	-
2415	3433	hypothetical protein	9	-	-	-	-	-	-	-	-	-	-	-	-	-	-	+	-	-	-	-	-	-	-	+	-	+	-	-	-	-	-	-	-	+	-	+	-	+	+	-	-	-	-	-	-	-	-	+	-	-	-	-	-	-	-	-	-	+
2416	3434	cell wall surface anchor family protein	9	-	-	-	-	-	-	-	-	-	-	-	-	-	-	-	+	-	-	-	-	-	-	-	-	+	+	+	+	+	+	+	+	-	-	-	-	-	-	-	-	-	-	-	-	-	-	-	-	-	-	-	-	-	-	-	-	-
2417	3442	hypothetical protein	9	-	-	-	-	+	-	-	-	-	-	-	-	-	-	-	-	-	-	+	+	+	+	+	-	-	-	-	+	-	-	-	-	+	-	-	-	-	-	+	-	-	-	-	-	-	-	-	-	-	-	-	-	-	-	-	-	-
2418	3446	hypothetical protein	9	-	-	-	-	-	-	-	-	-	-	-	-	-	-	-	-	-	-	-	-	-	-	-	-	-	-	-	-	-	-	-	-	-	-	-	+	+	-	-	-	+	-	-	-	+	+	-	-	-	-	-	+	+	+	+	-	-
2419	3450	hypothetical protein	9	-	-	-	-	-	-	-	-	-	-	-	-	-	-	-	-	-	-	-	+	+	+	+	+	-	-	-	-	-	-	-	-	-	+	+	+	+	-	-	-	-	-	-	-	-	-	-	-	-	-	-	-	-	-	-	-	-
2420	3454	ABC transporter	9	-	-	-	-	-	-	-	-	-	-	-	-	-	-	-	+	-	-	-	-	+	+	-	-	-	-	-	-	-	-	-	-	+	+	+	+	+	+	-	-	-	-	-	-	-	-	-	-	-	-	-	-	-	-	-	-	-
2421	3459	hypothetical protein	9	-	-	-	-	-	-	-	-	-	-	-	-	-	-	-	-	-	-	-	-	-	-	-	-	-	-	-	-	-	-	+	+	-	-	-	-	-	-	-	-	+	+	+	+	+	-	-	-	-	-	-	-	-	-	-	+	+
2422	3470	hypothetical protein	9	-	-	-	-	-	-	-	-	-	-	-	-	-	-	-	-	-	-	-	+	+	+	-	-	-	-	-	-	-	-	-	-	+	-	+	-	-	+	-	-	+	-	-	-	-	-	+	-	-	-	-	-	+	-	-	-	-
2423	3475	ATPase	9	-	-	-	-	-	-	-	-	-	-	-	-	-	-	-	-	-	-	-	-	-	-	-	-	-	-	-	-	-	-	+	+	-	-	-	-	-	+	-	-	-	-	-	-	-	-	-	-	-	+	+	+	+	+	+	-	-
2424	3485	transcriptional regulator, MerR family	9	-	-	-	-	-	-	-	-	-	-	-	-	-	-	-	-	-	-	-	-	-	-	-	-	-	-	-	-	-	-	+	+	-	-	-	-	-	-	-	-	-	-	-	+	-	-	-	-	-	+	+	-	+	+	+	+	-
2425	3486	hypothetical protein	9	-	-	+	-	+	+	-	-	+	+	+	+	+	-	+	-	-	-	-	-	-	-	-	-	-	-	-	-	-	-	-	-	-	-	-	-	-	-	-	-	-	-	-	-	-	-	-	-	-	-	-	-	-	-	-	-	-
2426	3489	probable membrane protein b2001	9	-	-	-	-	-	-	-	-	-	-	-	-	-	-	-	-	-	-	-	+	+	+	-	-	-	-	-	-	-	-	-	-	+	+	+	+	+	+	-	-	-	-	-	-	-	-	-	-	-	-	-	-	-	-	-	-	-
2427	3490	extracellular factor protein	9	-	-	-	-	-	-	-	-	-	-	+	+	-	+	-	-	-	-	-	-	-	-	-	-	-	-	-	-	-	-	-	-	+	+	+	+	+	+	-	-	-	-	-	-	-	-	-	-	-	-	-	-	-	-	-	-	-
2428	1646	Mobile element protein	8	-	-	-	-	-	-	-	-	-	-	-	-	-	-	-	-	-	-	-	-	-	-	-	-	+	+	-	+	-	-	-	-	-	-	-	-	+	+	-	-	-	-	-	-	-	-	-	-	-	+	-	-	+	-	+	-	-
2429	1886	hypothetical protein	8	-	-	-	-	-	-	-	-	-	-	-	-	-	-	-	-	-	-	-	-	-	-	-	-	-	-	-	-	-	-	-	-	-	-	-	-	-	-	-	-	-	-	+	+	-	-	+	+	+	+	-	-	-	-	+	+	-
2430	2008	Retron-type reverse transcriptase	8	-	-	-	-	-	-	-	-	-	-	-	-	-	-	-	-	-	-	-	-	+	+	-	-	-	-	-	-	+	-	-	-	-	-	-	-	-	+	+	+	-	-	-	-	-	-	+	-	-	-	-	-	-	-	+	-	-
2431	2079	TrsE-like protein	8	-	-	-	-	-	-	-	-	-	-	-	-	-	-	-	-	-	-	-	-	+	-	-	-	-	-	-	-	+	-	-	-	-	-	-	-	-	-	+	+	-	-	-	-	-	-	+	+	-	+	-	-	-	-	+	-	-
2432	2081	Predicted esterase of the alpha-beta hydrolase superfamily	8	-	-	-	-	-	-	-	-	-	-	-	-	-	-	-	+	-	-	-	-	-	-	-	-	-	-	-	-	-	-	-	-	-	-	-	-	-	-	-	-	-	+	-	-	+	+	+	-	+	-	-	+	-	-	+	-	-
2433	2119	Transposase	8	-	-	-	-	-	-	-	-	-	-	-	-	-	-	-	-	-	-	-	-	-	-	-	-	-	-	-	-	-	-	-	-	+	+	+	-	+	+	-	-	-	-	-	-	-	-	-	-	-	-	+	+	+	-	-	-	-
2434	2253	unknown phage protein	8	-	-	-	-	-	-	-	-	-	-	-	-	-	-	-	-	-	-	-	+	+	+	-	-	-	-	-	-	-	-	+	+	-	-	-	-	-	-	-	-	-	-	+	-	+	-	-	-	-	-	-	-	+	-	-	-	-
2435	2282	hypothetical protein	8	-	-	-	-	-	-	-	-	-	-	-	-	-	-	-	-	-	-	-	-	-	-	-	-	+	+	+	+	+	+	-	-	-	-	-	-	-	-	-	-	-	+	+	-	-	-	-	-	-	-	-	-	+	-	-	-	-
2436	2386	Sugar kinase and transcription regulator (EC 2.7.1.-)	8	-	-	-	-	-	-	-	-	-	-	-	-	-	-	-	-	-	-	-	-	-	-	+	+	-	-	-	-	-	-	-	-	-	-	-	-	-	-	+	+	+	-	-	-	-	-	-	-	-	-	+	-	-	-	+	+	-
2437	2391	transcriptional regulator PlcR, putative	8	-	-	-	-	-	-	-	-	-	-	-	-	-	-	-	-	-	-	-	-	-	-	+	-	-	-	-	-	-	-	-	-	-	-	-	-	-	+	+	+	-	-	-	-	-	-	-	+	-	+	-	+	-	-	+	-	-
2438	2397	hypothetical protein	8	-	-	-	-	-	-	-	-	-	-	-	-	-	-	-	-	-	-	-	-	-	-	-	-	-	-	-	-	-	-	-	-	-	-	-	-	-	-	-	-	-	-	-	-	-	-	-	-	-	+	+	+	+	+	+	+	+
2439	2405	Dihydrodipicolinate synthase (EC 4.2.1.52)	8	-	-	-	-	-	-	-	-	-	-	-	-	-	-	-	-	-	-	-	-	-	-	-	-	-	-	+	+	+	+	+	+	-	-	-	-	-	-	-	-	-	-	-	-	-	-	-	-	-	+	-	-	-	+	-	-	-
2440	2459	Transcriptional regulator, ArsR family	8	-	-	-	-	-	-	-	-	-	-	-	-	-	-	-	-	-	-	-	-	-	-	-	-	-	-	-	-	-	-	-	-	-	-	-	-	-	-	-	-	+	-	+	-	-	-	-	-	+	+	-	+	+	-	-	+	+
2441	2539	UDP-4-amino-4-deoxy-L-arabinose--oxoglutarate aminotransferase (EC 2.6.1.-)	8	-	-	-	-	-	-	-	-	-	-	-	-	-	-	-	-	-	-	-	-	-	-	+	+	-	-	-	-	-	-	-	-	+	-	+	+	+	-	+	+	-	-	-	-	-	-	-	-	-	-	-	-	-	-	-	-	-
2442	2626	Recombinational DNA repair protein RecT (prophage associated)	8	-	-	-	-	-	-	-	-	-	-	-	-	-	-	+	-	-	-	-	-	-	-	+	-	+	-	-	-	-	-	-	-	-	-	+	+	+	+	-	-	-	-	-	-	-	-	-	-	-	-	-	-	-	-	-	-	+
2443	2636	Undecaprenyl-phosphate galactosephosphotransferase (EC 2.7.8.6)	8	-	-	-	-	-	-	-	-	-	-	-	-	-	-	-	-	-	-	-	-	-	-	+	+	-	-	-	-	-	-	-	-	+	-	+	+	+	-	+	+	-	-	-	-	-	-	-	-	-	-	-	-	-	-	-	-	-
2444	2770	Phage integrase	8	-	-	-	-	-	-	-	-	-	-	-	-	-	-	-	-	-	-	-	-	-	-	-	-	-	-	-	-	-	-	-	-	-	-	-	-	-	-	-	-	+	-	-	-	-	-	+	+	+	-	+	+	+	-	+	-	-
2445	2817	Phage minor tail protein	8	-	-	-	-	-	-	-	-	-	-	-	-	-	-	-	-	-	-	-	+	-	-	-	-	-	-	+	+	+	+	+	+	-	+	-	-	-	-	-	-	-	-	-	-	-	-	-	-	-	-	-	-	-	-	-	-	-
2446	3118	Phage lysin	8	-	-	-	-	-	-	-	-	-	-	-	-	-	-	-	-	-	-	+	-	-	-	-	-	-	-	-	-	-	-	-	-	+	-	-	-	+	+	+	-	-	-	-	-	-	-	-	-	-	+	+	-	+	-	-	-	-
2447	3256	Essential recombination function protein	8	-	-	-	-	-	-	-	-	-	-	-	-	-	-	-	-	-	-	-	-	-	-	-	-	+	+	+	+	+	+	-	-	-	-	-	-	-	-	-	-	-	-	-	-	-	-	+	-	-	+	-	-	-	-	-	-	-
2448	3257	Type I restriction-modification system, specificity subunit S (EC 3.1.21.3)	8	-	-	-	-	-	-	-	-	-	-	-	-	-	-	-	-	-	-	+	+	+	+	+	+	+	-	-	-	+	-	-	-	-	-	-	-	-	-	-	-	-	-	-	-	-	-	-	-	-	-	-	-	-	-	-	-	-
2449	3260	hypothetical protein	8	-	-	-	-	-	-	-	-	-	-	-	-	-	-	-	-	-	-	-	-	-	-	-	-	+	+	+	+	+	+	-	-	-	-	-	-	-	-	-	-	-	-	-	-	-	-	+	-	-	+	-	-	-	-	-	-	-
2450	3264	salavaricin two-component response regulator	8	+	+	+	-	-	-	-	-	-	-	-	-	-	-	-	-	-	-	-	-	-	-	-	-	-	-	+	-	-	-	-	-	-	-	-	-	-	+	+	+	-	-	-	-	-	-	-	-	+	-	-	-	-	-	-	-	-
2451	3273	cdd4	8	+	+	+	-	-	-	-	-	-	-	-	-	-	-	-	-	-	-	-	-	-	-	-	-	-	-	+	-	-	-	-	-	-	-	-	-	-	+	+	+	-	-	-	-	-	-	-	-	+	-	-	-	-	-	-	-	-
2452	3274	hypothetical protein	8	-	-	-	-	-	-	+	-	-	-	-	-	-	-	-	-	-	-	-	-	-	-	-	-	-	-	-	-	-	-	-	-	-	-	-	-	-	-	+	+	-	-	+	-	+	+	+	+	-	-	-	-	-	-	-	-	-
2453	3276	Peptide ABC transporter ATP binding/permease protein	8	-	-	-	-	-	-	-	-	-	-	-	-	-	-	-	-	-	-	-	+	+	+	+	+	-	-	-	-	-	-	-	-	-	-	-	+	+	-	-	-	-	-	-	-	-	-	-	-	-	-	-	-	-	-	-	-	-
2454	3278	hypothetical protein	8	-	-	-	-	-	-	-	-	-	-	-	-	-	-	-	-	-	-	-	+	+	+	+	+	-	-	-	-	-	-	-	-	-	-	-	+	+	-	-	-	-	-	-	-	-	-	-	-	-	-	-	-	-	+	-	-	-
2455	3417	hypothetical protein	8	-	-	-	-	-	-	-	-	+	-	-	-	-	-	-	-	+	-	-	-	+	+	-	-	-	-	+	+	+	+	-	-	-	-	-	-	-	-	-	-	-	-	-	-	-	-	-	-	-	-	-	-	-	-	-	-	-
2456	3426	hypothetical protein	8	-	-	-	-	-	-	-	-	-	-	-	-	-	-	-	-	+	-	-	-	-	-	-	-	-	-	-	-	-	-	-	-	-	+	-	-	-	-	+	-	-	-	-	-	+	-	-	-	-	-	+	+	-	+	+	-	-
2457	3440	Phage tail length tape-measure protein	8	-	-	-	-	-	-	-	-	+	-	-	-	-	-	-	-	-	-	-	-	-	-	-	-	-	-	-	-	-	-	-	-	-	-	-	+	+	-	+	-	-	-	-	-	+	-	-	-	+	-	+	-	-	+	-	-	-
2458	3441	DNA-damage-inducible protein J	8	-	-	-	-	-	-	-	-	-	-	-	-	-	-	-	-	-	-	-	-	-	-	-	-	-	-	-	-	-	-	-	-	-	-	-	-	-	-	+	+	-	+	-	-	+	+	-	+	-	-	+	-	-	-	-	+	-
2459	3445	hypothetical protein	8	+	+	+	-	-	+	-	-	-	-	+	+	-	-	-	-	-	-	-	-	-	-	-	-	-	-	-	-	-	-	-	-	-	-	-	-	-	-	-	+	-	-	-	-	-	-	-	-	-	+	-	-	-	-	-	-	-
2460	3448	Aminoglycoside 6'-N-acetyltransferase	8	-	-	-	-	-	-	-	-	-	-	-	-	-	-	-	-	-	-	-	-	-	-	-	-	-	-	-	-	-	-	-	-	-	-	-	-	-	-	-	-	-	-	-	-	+	+	-	+	+	-	-	+	+	+	+	-	-
2461	3449	HlyD family secretion protein	8	-	-	-	-	-	-	-	-	-	-	-	-	-	-	-	-	-	-	-	+	+	+	+	+	-	-	-	-	-	-	-	-	-	-	-	+	+	-	-	-	-	-	-	-	-	-	-	-	-	-	-	-	-	+	-	-	-
2462	3458	hypothetical protein	8	-	-	-	-	-	-	-	-	-	-	-	-	-	-	-	-	-	-	-	-	-	-	-	-	-	-	-	-	-	-	-	-	-	-	-	+	+	-	-	-	-	-	-	-	-	-	-	-	-	-	+	+	+	+	+	-	+
2463	3463	hypothetical protein	8	-	-	-	-	-	-	-	-	-	-	-	-	-	-	-	-	-	-	-	-	-	-	-	-	-	-	-	-	-	-	-	-	-	-	-	-	-	-	-	-	-	-	+	-	-	-	+	+	+	-	+	-	+	+	+	-	-
2464	3466	Beta-hexosaminidase (EC 3.2.1.52)	8	-	-	-	-	-	-	-	-	-	-	-	-	-	-	-	-	-	-	-	-	-	-	-	-	-	-	-	-	-	-	-	-	-	-	-	-	-	-	-	-	-	-	+	-	-	-	+	+	+	-	+	-	+	+	+	-	-
2465	3500	hypothetical protein	8	+	+	+	-	-	-	-	-	-	-	+	-	-	-	-	-	-	-	-	-	-	-	-	-	-	-	-	-	-	-	-	-	-	-	-	-	-	-	-	-	-	-	-	+	-	-	-	-	-	-	+	-	+	-	-	+	-
2466	3501	Ubiquinone/menaquinone biosynthesis methyltransferase UBIE (EC 2.1.1.-)	8	+	+	+	-	-	-	-	-	-	-	+	-	-	-	-	-	-	-	-	-	-	-	-	-	-	-	-	-	-	-	-	-	-	-	-	-	-	-	-	-	-	-	-	+	-	-	-	-	-	-	+	-	+	-	-	+	-
2467	3627	hypothetical protein	8	-	-	-	-	-	-	-	-	-	-	-	-	-	-	-	-	+	+	+	-	-	-	-	-	-	-	-	-	-	-	-	-	-	-	-	-	-	-	-	-	-	-	-	-	+	+	+	+	+	-	-	-	-	-	-	-	-
2468	3628	hypothetical protein	8	-	-	-	-	-	-	-	-	-	-	-	-	-	-	-	-	+	-	-	-	-	-	-	-	-	-	-	-	-	-	-	-	-	-	-	+	+	-	-	+	-	-	-	-	+	-	-	-	-	-	+	+	-	-	+	-	-
2469	3631	hypothetical protein	8	-	-	-	-	-	-	-	-	-	-	-	-	-	-	-	-	+	-	-	+	+	+	+	-	-	-	-	+	-	-	-	-	+	-	-	-	-	+	-	-	-	-	-	-	-	-	-	-	-	-	-	-	-	-	-	-	-
2470	3632	hypothetical protein	8	-	-	-	-	-	-	-	-	+	+	-	-	-	-	+	-	+	-	-	-	-	-	-	-	-	-	-	-	-	-	-	-	+	-	-	-	-	-	+	-	-	-	-	-	-	+	-	+	-	-	-	-	-	-	-	-	-
2471	3636	hypothetical protein	8	-	-	-	-	-	-	-	-	-	-	-	-	-	-	-	-	-	-	-	-	-	-	-	-	+	+	+	+	+	+	-	-	-	+	-	-	-	-	-	-	-	-	-	-	-	-	-	-	-	-	+	-	-	-	-	-	-
2472	3638	Phage transcriptional repressor	8	-	-	-	-	-	-	-	-	-	-	-	-	-	-	-	-	-	-	-	-	-	-	+	+	+	+	+	+	+	+	-	-	-	-	-	-	-	-	-	-	-	-	-	-	-	-	-	-	-	-	-	-	-	-	-	-	-
2473	3639	hypothetical protein	8	-	-	-	-	-	-	-	-	-	-	-	-	-	-	+	-	-	-	-	-	-	-	+	-	+	-	-	-	-	-	-	-	+	-	+	-	+	+	-	-	-	-	-	-	-	-	-	-	-	-	-	-	-	-	-	-	+
2474	3640	FtsK/SpoIIIE family protein, putative secretion system component EssC/YukA	8	-	-	-	-	-	-	-	-	-	-	-	-	-	-	-	-	-	-	-	-	-	-	-	-	+	+	+	+	+	+	-	-	-	-	-	-	-	-	-	-	-	-	-	-	-	-	+	+	-	-	-	-	-	-	-	-	-
2475	3644	hypothetical protein	8	-	-	-	-	-	-	-	-	-	-	-	-	-	-	-	-	-	-	-	-	-	-	-	-	+	-	-	-	-	-	-	-	-	-	-	-	-	-	-	-	+	-	+	+	+	+	+	+	-	-	-	-	-	-	-	-	-
2476	3648	hypothetical protein	8	-	-	-	-	-	-	-	-	-	-	-	-	-	-	-	-	-	-	-	-	-	-	-	-	-	-	-	-	-	-	-	-	-	-	-	-	-	-	+	+	-	+	-	-	+	+	-	+	-	-	+	-	-	-	-	+	-
2477	3649	hypothetical protein	8	-	-	-	-	-	-	-	-	-	-	-	-	-	-	-	+	-	-	-	-	-	-	-	-	-	-	-	-	-	-	-	-	+	+	-	+	-	+	+	+	-	-	-	-	-	-	-	-	+	-	-	-	-	-	-	-	-
2478	3650	Integrase	8	-	-	-	+	-	-	-	-	-	-	-	-	-	+	-	-	-	-	-	-	-	-	-	-	-	-	-	-	-	-	+	+	-	-	-	-	-	-	+	+	-	-	-	-	-	-	-	-	-	-	-	-	-	+	+	-	-
2479	3660	hypothetical protein	8	-	-	-	-	+	-	-	-	-	-	-	-	-	-	-	-	-	-	+	+	+	+	+	-	-	-	-	-	-	-	-	-	-	-	-	-	-	+	-	-	-	-	-	-	-	-	-	-	-	-	-	-	-	-	-	+	-
2480	3663	hypothetical protein	8	-	-	-	-	+	-	-	-	-	-	-	-	-	-	-	-	-	-	-	+	+	+	+	-	-	-	-	+	-	-	-	-	+	-	-	-	-	-	-	-	-	-	-	-	-	-	-	-	-	-	-	-	+	-	-	-	-
2481	3665	Excinuclease ABC, C subunit, N-terminal	8	-	-	-	-	-	-	-	-	-	-	-	-	-	-	-	-	-	-	-	+	+	+	+	+	-	-	-	-	-	-	+	+	-	-	-	-	-	-	-	-	-	-	-	-	-	-	-	-	+	-	-	-	-	-	-	-	-
2482	3669	transcriptional regulator PlcR, putative	8	-	-	-	-	-	-	-	-	-	-	-	-	-	-	-	-	-	-	-	-	-	-	-	-	-	-	-	-	-	-	-	-	-	-	-	-	-	-	-	-	-	-	-	-	-	-	-	-	-	+	+	+	+	+	+	+	+
2483	3672	hypothetical protein	8	-	-	-	-	-	-	-	-	-	-	-	-	-	-	-	-	-	-	-	-	-	-	-	-	-	-	-	-	-	-	-	-	-	-	-	-	-	-	-	-	-	-	-	-	-	-	-	-	-	+	+	+	+	+	+	+	+
2484	3681	hypothetical protein	8	-	-	-	-	-	-	-	-	-	-	-	-	-	-	-	-	-	-	-	-	-	-	-	-	-	-	-	-	-	-	-	-	-	-	-	-	-	-	-	-	-	-	+	-	-	-	+	+	+	-	+	-	+	+	+	-	-
2485	3687	hypothetical protein	8	-	-	-	-	-	-	-	-	-	-	-	-	-	-	-	-	-	-	-	-	-	-	-	-	-	-	-	-	-	-	-	-	-	-	-	-	-	-	-	-	+	+	+	-	-	-	-	-	-	+	+	+	-	+	+	-	-
2486	3694	Type I restriction-modification system, DNA-methyltransferase subunit M (EC 2.1.1.72)	8	-	-	-	-	-	-	-	-	-	-	-	-	-	-	-	-	-	-	-	+	+	+	-	-	-	-	-	+	-	-	-	-	+	-	-	-	-	+	-	-	-	-	-	-	-	-	-	-	-	+	+	-	-	-	-	-	-
2487	3701	hypothetical protein	8	-	-	-	-	-	-	-	-	-	-	-	-	-	-	-	-	-	-	-	-	-	-	-	-	-	-	-	-	-	-	-	-	-	-	-	+	+	-	-	-	-	-	-	-	-	-	-	-	-	+	+	+	+	+	+	-	-
2488	3703	hypothetical protein	8	-	-	-	-	-	-	+	+	-	-	-	-	-	+	+	-	-	+	-	-	-	-	-	-	-	-	-	-	-	-	-	-	+	+	-	-	+	-	-	-	-	-	-	-	-	-	-	-	-	-	-	-	-	-	-	-	-
2489	3710	Type I restriction-modification system	8	-	-	-	-	-	-	-	-	-	-	-	-	-	-	-	-	-	-	-	-	+	+	-	-	-	-	-	-	-	-	-	-	+	+	+	+	+	+	-	-	-	-	-	-	-	-	-	-	-	-	-	-	-	-	-	-	-
2490	3714	hypothetical protein	8	-	-	-	-	-	-	-	-	-	-	-	-	-	-	-	-	-	-	-	-	-	-	-	-	-	-	-	-	-	-	-	-	+	-	-	+	+	+	-	-	-	-	-	+	-	-	+	-	+	-	-	-	-	-	-	+	-
2491	3715	replication protein	8	-	-	-	-	-	-	-	-	-	-	-	-	-	-	-	-	-	-	-	+	+	+	-	-	-	-	-	-	-	-	+	+	-	-	-	+	+	-	-	-	-	-	-	+	-	-	-	-	-	-	-	-	-	-	-	-	-
2492	3717	hypothetical protein	8	-	-	-	-	-	-	-	-	-	-	-	-	-	-	-	-	-	-	-	-	-	-	-	-	-	-	-	-	-	-	-	-	+	+	+	+	+	+	-	-	-	-	-	-	-	-	-	-	-	-	-	-	-	+	+	-	-
2493	3718	hypothetical protein	8	-	-	-	-	-	-	-	-	-	-	-	-	-	-	-	-	-	-	-	-	-	-	-	-	-	-	-	-	-	-	-	-	+	+	+	+	+	+	-	-	-	-	-	-	-	-	-	-	-	-	-	-	-	+	+	-	-
2494	3733	hypothetical protein	8	-	-	-	-	-	-	-	-	-	-	-	-	-	-	-	-	-	-	-	-	+	+	-	-	-	-	+	+	+	-	-	-	+	+	+	-	-	-	-	-	-	-	-	-	-	-	-	-	-	-	-	-	-	-	-	-	-
2495	3736	hypothetical protein	8	-	-	-	-	+	-	-	-	-	-	-	-	-	-	-	-	-	-	-	+	-	-	-	-	-	-	-	-	-	-	+	+	-	+	-	-	-	-	-	-	-	-	-	-	-	-	+	-	-	-	-	+	-	-	-	+	-
2496	1589	Mobile element protein	7	-	-	-	-	-	-	-	-	-	-	-	-	-	+	-	-	-	-	-	-	-	-	-	-	-	-	-	-	-	-	-	-	-	-	-	-	+	-	-	-	-	-	+	+	-	-	-	-	-	+	-	-	+	-	-	+	-
2497	1720	N-acetylneuraminate lyase (EC 4.1.3.3)	7	-	-	-	+	-	-	-	-	-	-	-	-	-	-	-	-	-	-	-	-	-	-	-	-	-	-	-	-	-	-	-	-	-	-	-	-	-	-	-	-	-	-	+	-	-	-	-	-	-	+	-	+	+	+	+	-	-
2498	1793	N-acetylmannosamine kinase (EC 2.7.1.60)	7	-	-	-	+	-	-	-	-	-	-	-	-	-	-	-	-	-	-	-	-	-	-	-	-	-	-	-	-	-	-	-	-	-	-	-	-	-	-	-	-	-	-	+	-	-	-	-	-	-	+	-	+	+	+	+	-	-
2499	1809	2-amino-4-hydroxy-6-hydroxymethyldihydropteridine pyrophosphokinase (EC 2.7.6.3)	7	+	+	-	-	-	-	-	-	-	-	-	-	-	-	-	-	-	-	-	-	-	-	-	-	-	-	-	+	+	-	-	-	+	+	+	-	-	-	-	-	-	-	-	-	-	-	-	-	-	-	-	-	-	-	-	-	-
2500	1827	LSU ribosomal protein L36p	7	-	-	-	-	-	-	-	-	-	-	-	-	-	-	-	+	-	-	-	+	-	-	-	+	-	-	-	-	-	-	-	-	-	+	-	-	-	-	-	-	+	+	-	-	-	-	-	-	-	-	-	-	-	-	-	+	-
2501	1843	Transcriptional regulator, Cro/CI family	7	-	-	-	-	-	-	-	-	-	-	-	-	-	-	-	-	-	-	-	-	-	-	-	-	-	-	-	-	-	-	-	-	-	-	-	-	-	-	+	+	-	-	+	-	+	-	+	-	+	-	-	-	-	-	-	+	-
2502	2060	conserved domain protein	7	-	-	-	-	-	-	-	-	-	-	-	-	-	-	-	-	-	-	-	-	-	-	-	-	-	-	-	-	-	-	-	-	-	-	-	-	-	-	-	-	-	-	+	+	-	-	+	+	+	-	-	-	-	-	+	+	-
2503	2086	hypothetical protein	7	-	-	-	-	-	-	-	-	-	-	-	-	-	-	-	-	-	-	-	-	-	-	-	-	-	-	-	-	-	-	-	-	-	-	-	-	-	-	-	-	-	-	+	+	-	-	+	+	+	-	-	-	-	-	+	+	-
2504	2220	Type I restriction-modification system, DNA-methyltransferase subunit M (EC 2.1.1.72)	7	-	-	-	-	-	-	-	-	-	-	-	-	-	-	-	-	-	-	-	-	-	-	-	-	-	-	-	-	-	-	-	-	-	-	-	-	-	-	-	-	-	-	-	-	-	-	-	-	+	+	+	+	+	+	-	+	-
2505	2250	acetyl xylan esterase, putative	7	-	-	-	+	-	-	-	-	-	-	-	-	-	-	-	-	-	-	-	-	-	-	-	-	-	-	-	-	-	-	-	-	-	-	-	-	-	-	-	-	-	-	+	-	-	-	-	-	-	+	-	+	+	+	+	-	-
2506	2304	Type I restriction-modification system, restriction subunit R (EC 3.1.21.3)	7	-	-	-	-	-	-	-	-	-	-	-	-	-	-	-	-	-	-	-	-	-	-	-	-	-	-	-	-	-	-	-	-	-	-	-	-	-	-	-	-	-	-	-	-	-	-	-	-	+	+	+	+	+	+	-	+	-
2507	2535	Potassium voltage-gated channel subfamily KQT; possible potassium channel, VIC family	7	-	-	-	-	-	-	-	-	-	-	-	-	-	-	-	-	-	-	+	-	-	-	-	-	-	-	-	-	-	-	-	-	-	-	-	-	-	-	+	+	-	+	-	-	+	-	-	-	-	+	-	-	-	-	-	+	-
2508	2548	Superfamily II DNA and RNA helicase	7	-	-	-	-	-	-	-	-	-	-	-	-	-	-	-	-	-	-	-	-	-	-	-	-	-	-	-	-	-	+	-	-	+	+	+	+	-	+	-	-	-	-	+	-	-	-	-	-	-	-	-	-	-	-	-	-	-
2509	2565	putative ABC transporter (ATP-binding protein)	7	-	-	-	-	-	-	-	-	-	-	-	-	-	-	-	-	-	-	-	-	-	-	-	-	-	-	-	-	-	-	-	-	-	-	-	-	-	-	-	-	-	-	-	+	-	-	-	-	+	+	+	+	+	+	-	-	-
2510	2566	Phage terminase large subunit	7	-	-	-	-	-	-	-	-	+	-	-	-	-	-	-	-	-	-	-	-	-	-	-	-	-	-	-	-	-	-	-	-	-	-	-	-	-	-	-	-	-	-	-	-	+	-	-	-	+	+	+	-	-	+	-	+	-
2511	2578	Phage-related protein	7	-	-	-	-	-	-	-	-	-	-	-	-	-	-	-	-	-	-	-	-	-	-	-	-	-	-	-	-	-	-	-	-	-	-	-	+	+	-	-	-	-	-	-	-	-	-	-	-	-	-	+	+	+	+	+	-	-
2512	2579	hypothetical protein	7	-	-	-	-	-	-	-	-	-	-	-	-	-	-	-	-	-	-	-	-	-	-	-	-	-	-	-	-	-	-	-	-	-	-	-	+	+	-	-	-	-	-	-	-	-	-	-	-	-	-	+	+	+	+	+	-	-
2513	2618	COG2110, Macro domain, possibly ADP-ribose binding module	7	-	-	-	-	-	-	-	-	-	-	-	-	-	-	-	-	-	-	-	-	-	-	-	-	-	-	-	-	-	-	-	-	-	-	-	-	-	-	-	-	-	-	+	-	+	-	-	-	-	-	+	+	-	+	-	+	+
2514	2672	GftB: Glycosyl transferase, family 8	7	-	-	-	-	-	-	-	-	-	-	-	-	-	-	-	-	-	-	-	-	-	-	-	-	-	-	-	-	-	-	-	-	-	-	-	+	+	+	-	-	-	-	-	-	-	-	-	-	-	-	+	-	+	+	+	-	-
2515	2675	FIG040428: Phage-associated protein	7	-	-	-	-	+	-	-	-	-	-	-	-	-	-	-	-	-	-	-	+	-	-	-	-	-	-	-	-	-	-	+	+	-	+	-	-	-	-	-	-	-	-	-	-	-	-	-	-	-	-	-	+	-	-	+	-	-
2516	2883	DNA-cytosine methyltransferase (EC 2.1.1.37)	7	-	-	-	-	-	-	-	-	-	-	-	-	-	-	-	-	-	-	-	-	-	-	-	-	-	-	-	-	-	-	-	-	-	-	-	-	-	-	-	-	-	-	-	-	-	+	+	-	+	+	-	+	+	-	+	-	-
2517	2896	UDP-glucose 4-epimerase (EC 5.1.3.2)	7	-	-	-	-	-	-	-	-	-	-	-	-	-	-	-	-	-	-	-	-	-	-	-	-	-	-	-	-	-	-	-	-	-	-	-	-	-	-	-	-	+	+	-	+	-	-	-	+	-	-	-	-	+	+	-	-	-
2518	2908	transcriptional regulator PlcR, putative	7	-	-	-	-	+	-	-	-	-	-	-	-	-	-	-	-	-	-	-	-	-	-	-	-	-	-	-	-	-	-	-	-	-	-	-	-	-	-	-	-	-	-	-	+	-	-	-	-	-	+	+	+	+	+	-	-	-
2519	2917	Holin	7	-	-	-	-	-	-	-	-	+	-	-	-	-	-	-	-	-	-	-	-	-	-	-	-	-	-	-	-	-	-	-	-	-	-	-	-	-	-	-	-	-	-	-	-	+	-	-	-	+	-	+	-	+	+	-	+	-
2520	3000	Chitobiose phosphorylase (EC 2.4.1.-)	7	-	-	-	-	-	-	-	-	-	-	-	-	-	-	-	-	-	-	-	-	-	-	+	+	-	-	-	-	-	-	-	-	-	-	-	-	-	-	+	+	+	-	-	-	-	-	-	-	-	-	-	-	-	-	+	+	-
2521	3003	Endo-beta-1,3-1,4 glucanase (Licheninase) (EC 3.2.1.73)	7	-	-	-	-	-	-	-	-	-	-	-	-	-	-	-	-	-	-	-	-	-	-	+	+	-	-	-	-	-	-	-	-	-	-	-	-	-	-	+	+	+	-	-	-	-	-	-	-	-	-	-	-	-	-	+	+	-
2522	3046	Nucleotide sugar synthetase-like protein	7	-	-	-	-	-	-	-	-	-	-	-	-	-	-	-	-	-	-	-	-	-	-	-	-	-	-	-	-	-	-	-	-	-	-	-	+	+	+	-	-	-	-	-	-	-	-	-	-	-	-	+	-	+	+	+	-	-
2523	3116	hypothetical protein	7	-	-	-	-	-	-	-	-	-	-	-	-	-	-	-	-	-	-	-	-	-	-	-	-	-	-	-	-	-	-	-	-	-	-	-	-	-	-	+	+	+	+	+	-	-	+	+	-	-	-	-	-	-	-	-	-	-
2524	3117	Cyclic beta-1,2-glucan synthase (EC 2.4.1.-)	7	-	-	-	-	-	-	-	-	-	-	-	-	-	-	-	-	-	-	-	-	-	-	+	+	-	-	-	-	-	-	-	-	-	-	-	-	-	-	+	+	+	-	-	-	-	-	-	-	-	-	-	-	-	-	+	+	-
2525	3122	Transcriptional regulator, AraC family	7	-	-	-	-	-	-	-	-	-	-	-	-	-	-	-	-	-	-	-	-	-	-	+	+	-	-	-	-	-	-	-	-	-	-	-	-	-	-	+	+	+	-	-	-	-	-	-	-	-	-	-	-	-	-	+	+	-
2526	3129	Probable site-specific serine recombinase-resolvase family protein	7	-	-	-	-	-	-	-	-	-	-	-	-	-	-	-	-	-	-	-	+	+	+	+	+	-	-	-	-	-	-	-	-	-	+	+	-	-	-	-	-	-	-	-	-	-	-	-	-	-	-	-	-	-	-	-	-	-
2527	3154	hypothetical protein	7	-	-	-	-	-	-	-	-	-	-	-	-	-	-	-	-	-	-	-	-	-	-	-	-	-	-	-	-	-	-	-	-	-	-	+	-	-	-	-	-	-	-	-	-	+	-	-	-	-	+	+	+	-	+	+	-	-
2528	3161	Methylase	7	-	-	-	-	-	-	-	-	-	-	-	-	-	-	-	-	-	-	-	-	-	-	-	-	-	-	-	-	-	-	-	-	-	-	-	+	+	+	-	-	-	-	-	+	-	-	-	-	-	-	-	+	+	-	+	-	-
2529	3169	ATP-binding region, ATPase-like:Histidine kinase A, N-terminal	7	-	-	-	-	-	-	-	-	-	-	-	-	-	-	-	-	-	-	-	+	+	+	-	-	-	-	-	-	+	-	-	-	+	-	-	-	-	-	-	-	-	-	-	-	-	-	-	-	-	-	-	-	-	-	+	+	-
2530	3253	Phage major capsid protein	7	-	-	-	-	-	-	-	-	-	-	-	-	-	-	-	-	-	-	-	-	-	-	-	-	+	-	+	+	+	+	-	-	-	-	-	-	-	-	-	-	-	-	-	-	-	-	-	-	-	+	-	-	-	-	-	-	+
2531	3255	hypothetical protein	7	-	-	-	-	-	-	-	-	-	-	-	-	-	-	-	-	-	-	-	-	-	-	-	-	+	+	+	+	+	+	-	-	-	-	-	-	-	-	+	-	-	-	-	-	-	-	-	-	-	-	-	-	-	-	-	-	-
2532	3267	Possible alpha-xyloside ABC transporter, permease component	7	-	-	-	-	-	-	-	-	-	-	-	-	-	-	-	-	-	-	-	-	-	-	+	+	-	-	-	-	-	-	-	-	-	-	-	-	-	-	+	+	+	-	-	-	-	-	-	-	-	-	-	-	-	-	+	+	-
2533	3271	hypothetical protein	7	-	-	-	-	-	-	-	-	-	-	-	-	-	-	-	-	-	-	-	-	-	-	+	+	-	-	-	-	-	-	-	-	-	-	-	-	-	-	+	+	+	-	-	-	-	-	-	-	-	-	-	-	-	-	+	+	-
2534	3272	phage protein	7	-	-	-	-	-	-	-	-	-	-	-	-	-	-	-	-	-	-	-	-	-	-	-	-	-	-	+	+	+	-	-	-	-	-	-	-	-	-	+	+	-	-	-	-	+	-	-	-	+	-	-	-	-	-	-	-	-
2535	3275	Possible alpha-xyloside ABC transporter, permease component	7	-	-	-	-	-	-	-	-	-	-	-	-	-	-	-	-	-	-	-	-	-	-	+	+	-	-	-	-	-	-	-	-	-	-	-	-	-	-	+	+	+	-	-	-	-	-	-	-	-	-	-	-	-	-	+	+	-
2536	3301	DNA primase, phage associated	7	-	-	-	-	-	-	-	-	-	-	-	-	-	-	-	-	-	-	-	-	-	-	-	-	-	-	-	-	-	-	+	+	+	+	+	-	-	-	-	-	+	-	-	-	-	-	-	-	-	-	-	+	-	-	-	-	-
2537	3305	Oligopeptide transport ATP-binding protein OppD (TC 3.A.1.5.1)	7	-	-	-	-	-	-	-	-	-	-	-	-	-	-	-	-	-	-	-	-	-	-	-	-	-	-	-	-	-	-	-	-	-	-	-	+	-	-	-	-	-	-	-	-	-	-	-	-	-	+	+	+	+	+	+	-	-
2538	3306	Oligopeptide transport system permease protein OppB (TC 3.A.1.5.1)	7	-	-	-	-	-	-	-	-	-	-	-	-	-	-	-	-	-	-	-	-	-	-	-	-	-	-	-	-	-	-	-	-	-	-	-	+	-	-	-	-	-	-	-	-	-	-	-	-	-	+	+	+	+	+	+	-	-
2539	3307	Octaprenyl-diphosphate synthase (EC 2.5.1.-) / Dimethylallyltransferase (EC 2.5.1.1) / Geranyltranstransferase (farnesyldiphosphate synthase) (EC 2.5.1.10) / Geranylgeranyl pyrophosphate synthetase (EC 2.5.1.29)	7	-	-	-	-	-	-	-	-	-	-	-	-	-	-	-	-	-	-	-	-	-	-	-	-	-	-	-	-	-	-	-	-	-	-	-	+	-	-	-	-	-	-	-	-	-	-	-	-	-	+	+	+	+	+	+	-	-
2540	3313	Oligopeptide/dipeptide ABC transporter, ATP-binding protein, C-terminal	7	-	-	-	-	-	-	-	-	-	-	-	-	-	-	-	-	-	-	-	-	-	-	-	-	-	-	-	-	-	-	-	-	-	-	-	+	-	-	-	-	-	-	-	-	-	-	-	-	-	+	+	+	+	+	+	-	-
2541	3314	Oligopeptide ABC transporter, periplasmic oligopeptide-binding protein OppA (TC 3.A.1.5.1)	7	-	-	-	-	-	-	-	-	-	-	-	-	-	-	-	-	-	-	-	-	-	-	-	-	-	-	-	-	-	-	-	-	-	-	-	+	-	-	-	-	-	-	-	-	-	-	-	-	-	+	+	+	+	+	+	-	-
2542	3326	Transport ATP Binding Protein	7	-	-	-	-	-	-	-	-	-	-	-	-	-	-	-	-	-	-	-	+	+	+	-	-	-	-	-	-	+	-	-	-	+	-	-	-	-	-	-	-	-	-	-	-	-	-	-	-	-	-	-	-	-	-	+	+	-
2543	3418	Positive transcriptional regulator, MutR family	7	-	-	-	-	-	-	-	-	-	-	-	-	-	-	-	-	+	+	-	-	-	-	-	-	-	-	-	-	-	-	-	-	-	-	-	-	-	-	-	-	-	-	-	-	-	-	-	-	-	+	+	+	-	+	+	-	-
2544	3421	Glycosyl transferase CpsG	7	-	-	-	-	-	-	-	-	+	-	-	-	+	+	-	-	+	+	-	-	-	-	-	-	-	-	-	-	-	-	-	-	-	-	-	-	-	-	-	-	-	-	-	-	+	-	+	-	-	-	-	-	-	-	-	-	-
2545	3437	integrase	7	-	-	-	-	-	-	-	-	-	-	-	-	-	-	-	-	-	-	-	-	-	-	-	-	+	-	-	-	-	-	-	-	-	+	+	+	+	-	+	-	-	-	-	-	-	-	-	-	-	-	-	-	-	-	-	-	+
2546	3438	Endo-beta-1,3-1,4 glucanase (Licheninase) (EC 3.2.1.73)	7	-	-	-	-	-	-	-	-	-	-	-	-	-	-	-	-	-	-	-	-	-	-	+	+	-	-	-	-	-	-	-	-	-	-	-	-	-	-	+	+	+	-	-	-	-	-	-	-	-	-	-	-	-	-	+	+	-
2547	3439	hypothetical protein	7	-	-	-	-	-	-	-	-	-	-	-	-	-	-	-	-	-	-	-	-	-	-	-	-	-	-	-	-	-	-	-	-	-	-	-	-	-	-	+	+	-	-	+	-	+	-	+	-	+	-	-	-	-	-	-	+	-
2548	3443	dihydroneopterin aldolase	7	-	-	-	-	-	-	-	-	-	-	-	-	-	-	+	-	-	-	+	-	-	-	-	-	-	-	-	-	-	-	-	-	-	-	-	-	-	+	+	-	-	-	-	-	-	-	-	+	-	-	+	-	-	-	-	-	+
2549	3453	hypothetical protein	7	-	-	-	-	-	-	-	-	-	-	-	-	-	-	-	+	-	-	-	-	-	-	-	-	-	-	-	-	-	-	-	-	-	-	-	-	-	-	-	-	-	-	-	+	+	-	-	-	-	-	-	+	+	+	+	-	-

Table S5. Precence/absence of each HG among 57 *S. suis* strains

				1																13			25					28						104		94			76			53/54		Divergent *S. suis*																
Index[a]	ClutserID	Gene products	Members	98HAH33	05ZYH33	SC84	A7	SS12	P1/7	DAT273	NIAH11434	JS14	GZ1	BM407	S735	13730	MNCM07	NCTC 10446	14636	NCTC 10237	ST1	2651	89-1591	MNCM04	MNCM25	8074	D9	4961	ST3	MNCM43	DAT292	DAT260	NIAH11435	MNCM21	MNCM50	89-2479	DAT234	DAT290	93A	42A	NT77	6407	11538	8830	12814	92-1400	89-590	2726	2524	89-3576-3	4417	D12	10581	89-5259	88-5299A	R61	92-1191	14A	92-4172	22083
2550	3457	hypothetical protein	7	-	-	-	-	-	-	-	-	-	-	-	-	-	-	-	-	-	-	-	-	-	-	-	-	-	-	-	-	-	-	-	-	-	-	-	-	-	-	-	-	-	-	-	-	+	-	-	-	-	+	+	-	-	+	+	+	+
2551	3482	ABC transporter permease protein	7	-	-	-	-	-	-	-	-	-	-	-	-	-	-	-	-	-	-	-	-	-	-	-	-	-	-	-	-	-	-	-	-	-	-	-	-	-	-	-	-	-	-	-	+	-	-	-	-	+	+	+	+	+	+	-	-	-
2552	3484	HTH-type transcriptional regulator rgg	7	-	-	-	-	-	-	-	-	-	-	-	-	-	-	-	-	-	-	-	-	-	-	-	-	-	-	-	-	-	-	-	-	-	-	-	-	-	-	-	-	-	-	-	+	-	-	-	-	+	+	+	+	+	+	-	-	-
2553	3491	hypothetical protein	7	-	-	-	-	-	-	-	-	-	-	-	-	-	-	-	-	-	+	-	-	+	+	-	-	-	-	-	-	-	-	-	-	+	+	-	-	+	-	-	-	-	-	-	-	-	-	-	-	-	-	-	-	-	-	-	+	-
2554	3622	hypothetical protein	7	-	-	-	-	-	-	-	-	-	-	-	-	-	-	-	-	+	-	-	-	-	-	-	-	-	-	-	-	-	-	-	-	-	-	+	+	+	-	-	-	-	-	-	-	+	-	-	-	-	-	-	+	-	+	-	-	-
2555	3626	hypothetical protein	7	-	-	-	-	-	-	-	-	-	-	-	-	-	-	-	-	+	-	-	-	-	-	-	-	-	-	-	-	-	-	-	-	-	-	-	-	-	-	+	+	-	-	-	-	+	-	-	-	-	+	+	-	-	+	-	-	-
2556	3634	hypothetical protein	7	-	-	-	-	-	-	-	-	-	-	-	-	-	-	-	-	-	-	-	-	-	-	-	-	+	+	+	+	+	+	-	-	-	-	-	-	-	-	-	-	-	-	-	-	-	-	+	-	-	-	-	-	-	-	-	-	-
2557	3641	hypothetical protein	7	-	-	-	-	-	-	-	-	-	-	-	-	-	-	-	-	-	-	-	-	-	-	-	-	+	-	-	-	-	-	-	-	-	+	-	-	-	-	+	+	-	-	-	-	-	-	-	-	+	+	-	-	-	-	+	-	-
2558	3643	hypothetical protein	7	-	-	-	-	-	-	-	-	-	-	-	-	-	-	-	-	-	-	-	+	-	-	+	+	+	+	+	-	-	+	-	-	-	-	-	-	-	-	-	-	-	-	-	-	-	-	-	-	-	-	-	-	-	-	-	-	-
2559	3645	FtsK/SpoIIIE family protein, putative secretion system component EssC/YukA	7	-	-	-	-	-	-	-	-	-	-	-	-	-	-	-	-	-	-	-	-	-	-	-	-	+	-	+	+	+	+	-	-	-	-	-	-	-	-	-	-	-	-	+	-	+	-	-	-	-	-	-	-	-	-	-	-	-
2560	3651	hypothetical protein	7	+	+	+	-	-	-	-	-	-	-	-	-	-	-	-	-	-	-	-	-	-	-	-	-	-	-	+	-	-	-	-	-	-	-	-	-	-	-	+	+	-	-	-	-	-	-	-	-	+	-	-	-	-	-	-	-	-
2561	3652	possible two-component sensor histidine kinase	7	+	+	+	-	-	-	-	-	-	-	-	-	-	-	-	-	-	-	-	-	-	-	-	-	-	-	+	-	-	-	-	-	-	-	-	-	-	-	+	+	-	-	-	-	-	-	-	-	+	-	-	-	-	-	-	-	-
2562	3655	Lactaldehyde dehydrogenase involved in fucose or rhamnose utilization (EC 1.2.1.22)	7	-	-	-	-	-	-	-	-	-	-	-	-	-	-	-	-	-	-	-	+	+	+	+	+	-	-	-	-	-	-	-	-	-	+	+	-	-	-	-	-	-	-	-	-	-	-	-	-	-	-	-	-	-	-	-	-	-
2563	3656	putative large secreted protein	7	-	-	-	-	-	-	-	-	-	-	-	-	-	-	-	-	-	-	-	+	+	+	+	+	-	-	-	-	-	-	-	-	-	+	+	-	-	-	-	-	-	-	-	-	-	-	-	-	-	-	-	-	-	-	-	-	-
2564	3657	PTS system, mannose-specific IID component (EC 2.7.1.69)	7	-	-	-	-	-	-	-	-	-	-	-	-	-	-	-	-	-	-	-	+	+	+	+	+	-	-	-	-	-	-	-	-	-	+	+	-	-	-	-	-	-	-	-	-	-	-	-	-	-	-	-	-	-	-	-	-	-
2565	3658	PTS system, N-acetylgalactosamine-specific IIC component (EC 2.7.1.69)	7	-	-	-	-	-	-	-	-	-	-	-	-	-	-	-	-	-	-	-	+	+	+	+	+	-	-	-	-	-	-	-	-	-	+	+	-	-	-	-	-	-	-	-	-	-	-	-	-	-	-	-	-	-	-	-	-	-
2566	3661	Predicted L-fucose-specific phosphotransferase system, EIIB component	7	-	-	-	-	-	-	-	-	-	-	-	-	-	-	-	-	-	-	-	+	+	+	+	+	-	-	-	-	-	-	-	-	-	+	+	-	-	-	-	-	-	-	-	-	-	-	-	-	-	-	-	-	-	-	-	-	-
2567	3662	L-fuculokinase (EC 2.7.1.51)	7	-	-	-	-	-	-	-	-	-	-	-	-	-	-	-	-	-	-	-	+	+	+	+	+	-	-	-	-	-	-	-	-	-	+	+	-	-	-	-	-	-	-	-	-	-	-	-	-	-	-	-	-	-	-	-	-	-
2568	3664	PTS system, L-fucose-specific IIA component (EC 2.7.1.69)	7	-	-	-	-	-	-	-	-	-	-	-	-	-	-	-	-	-	-	-	+	+	+	+	+	-	-	-	-	-	-	-	-	-	+	+	-	-	-	-	-	-	-	-	-	-	-	-	-	-	-	-	-	-	-	-	-	-
2569	3666	L-fucose mutarotase	7	-	-	-	-	-	-	-	-	-	-	-	-	-	-	-	-	-	-	-	+	+	+	+	+	-	-	-	-	-	-	-	-	-	+	+	-	-	-	-	-	-	-	-	-	-	-	-	-	-	-	-	-	-	-	-	-	-
2570	3677	hypothetical protein	7	-	-	-	-	-	-	-	-	-	-	-	-	-	-	-	-	-	-	-	-	-	-	-	-	-	-	-	-	-	-	-	-	-	-	-	-	-	-	-	-	+	-	-	-	-	-	+	+	+	+	-	+	-	-	+	-	-
2571	3686	hypothetical protein	7	-	-	-	-	-	-	-	-	-	-	-	-	-	-	-	-	-	-	-	-	-	-	-	-	-	-	+	+	+	+	-	-	-	-	-	-	-	-	-	-	+	+	+	-	-	-	-	-	-	-	-	-	-	-	-	-	-
2572	3689	hypothetical protein	7	-	-	-	-	-	-	-	-	-	-	-	-	-	-	-	-	-	-	-	-	-	-	-	-	-	-	+	+	+	-	+	+	-	-	-	-	-	-	-	-	+	-	-	-	-	-	-	-	-	-	-	+	-	-	-	-	-
2573	3693	hypothetical protein	7	-	-	-	-	-	-	-	-	-	-	-	-	-	-	-	-	-	-	-	-	-	-	-	-	-	-	+	+	+	-	+	+	-	-	-	-	-	-	-	-	+	-	-	-	-	-	-	-	-	-	-	+	-	-	-	-	-
2574	3704	hypothetical protein	7	-	-	-	-	-	-	-	-	-	-	-	-	-	-	-	-	-	-	-	-	-	-	-	-	-	-	-	-	-	-	+	+	+	+	+	-	-	-	-	-	-	-	-	-	+	-	-	-	+	-	-	-	-	-	-	-	-
2575	3709	hypothetical protein	7	-	-	-	-	-	-	-	-	-	-	-	-	-	-	-	-	-	-	-	-	-	-	-	-	-	-	-	-	-	-	-	-	-	-	-	+	+	+	-	-	-	-	-	-	-	-	-	-	-	-	+	-	+	+	+	-	-
2576	3712	Predicted transcriptional regulator	7	-	-	-	-	-	-	-	-	-	-	-	-	-	-	-	-	-	-	-	+	-	-	-	-	-	-	-	-	-	-	+	+	-	+	+	+	-	+	-	-	-	-	-	-	-	-	-	-	-	-	-	-	-	-	-	-	-
2577	3724	hypothetical protein	7	-	-	-	-	+	-	-	-	-	-	-	-	-	-	-	-	-	-	-	+	-	-	-	-	-	-	-	-	-	-	+	+	-	+	-	-	-	-	-	-	-	-	-	-	-	-	-	-	-	-	+	-	-	-	+	-	-
2578	3734	hypothetical protein	7	-	-	-	-	-	-	-	-	-	-	-	-	-	-	-	-	-	-	-	+	+	+	-	-	-	-	+	-	-	-	-	-	+	-	+	-	-	-	-	-	-	-	-	-	-	-	-	-	-	-	-	-	-	+	-	-	-
2579	3735	unknown	7	-	-	-	-	-	-	-	-	+	-	-	-	-	-	-	-	-	-	-	-	-	-	-	-	-	-	-	-	-	-	-	-	+	-	-	-	-	-	-	-	-	-	-	+	-	-	-	-	+	-	+	-	+	+	-	-	-
2580	3739	hypothetical protein	7	-	-	-	-	+	-	-	-	-	-	-	-	-	-	-	-	-	-	-	+	-	-	-	-	-	-	-	-	-	-	+	+	-	+	-	-	-	-	-	-	-	-	-	+	-	-	-	-	-	-	+	-	-	-	-	-	-
2581	3856	hypothetical protein	7	-	-	-	-	-	-	-	-	-	-	-	-	-	-	-	-	+	+	-	-	-	-	-	-	-	-	-	-	-	-	-	-	-	-	-	-	-	-	+	+	-	-	+	-	-	-	+	+	-	-	-	-	-	-	-	-	-
2582	3857	putative transcriptional activator - phage associated	7	-	-	-	-	-	-	-	-	-	-	-	-	-	-	-	-	+	-	-	-	-	-	-	-	-	-	-	-	-	-	-	-	-	-	-	+	+	-	+	+	-	-	-	-	-	-	+	-	-	-	-	+	-	-	-	-	-
2583	3858	hypothetical protein	7	-	-	-	-	-	-	-	-	-	-	-	-	-	-	-	-	+	+	-	-	-	-	-	-	-	-	-	-	-	-	-	-	-	-	-	-	-	-	+	+	-	-	+	-	-	-	+	+	-	-	-	-	-	-	-	-	-
2584	3859	hypothetical protein	7	-	-	-	-	-	-	-	-	-	-	-	-	-	-	-	-	+	-	+	-	-	-	-	-	-	-	-	-	-	-	-	-	-	-	-	+	+	-	-	-	-	-	-	-	-	-	-	-	-	-	-	-	-	+	+	-	+
2585	3860	Phage protein	7	-	-	-	-	-	-	-	-	-	-	-	-	-	-	-	-	+	-	-	-	-	-	-	-	-	-	-	-	-	-	-	-	-	-	-	+	+	-	+	+	-	-	-	-	-	-	+	-	-	-	-	+	-	-	-	-	-
2586	3862	hypothetical protein	7	-	-	-	-	-	-	-	-	-	-	-	-	-	-	-	-	+	+	-	-	-	-	-	-	-	-	-	-	-	-	-	-	-	-	-	-	-	-	-	-	+	-	+	-	-	-	-	-	+	-	+	+	-	-	-	-	-
2587	3865	Phage portal protein	7	-	-	-	-	-	-	-	-	-	-	-	-	-	-	-	-	+	-	-	-	-	-	-	-	-	-	-	-	-	-	-	-	-	-	-	+	+	-	+	+	-	-	-	-	-	-	+	-	-	-	-	+	-	-	-	-	-
2588	3866	hypothetical protein	7	-	-	-	-	+	-	-	-	-	-	-	-	-	-	-	-	+	-	-	-	-	-	-	-	-	-	-	-	-	-	+	+	+	+	-	-	-	+	-	-	-	-	-	-	-	-	-	-	-	-	-	-	-	-	-	-	-
2589	3869	putative replication initiator protein	7	-	-	-	-	-	-	-	-	-	-	-	-	-	-	-	-	+	+	-	-	-	-	-	-	-	-	-	-	-	-	-	-	-	-	-	-	-	-	+	+	-	-	+	-	-	-	+	+	-	-	-	-	-	-	-	-	-
2590	3870	Phage terminase	7	-	-	-	-	-	-	-	-	-	-	-	-	-	-	-	-	+	-	-	-	-	-	-	-	-	-	-	-	-	-	-	-	-	-	-	+	+	-	+	+	-	-	-	-	-	-	+	-	-	-	-	+	-	-	-	-	-
2591	3873	hypothetical protein	7	-	-	-	-	+	-	-	-	-	-	-	-	-	-	-	-	+	-	-	-	-	-	-	-	-	-	-	-	-	-	+	+	+	+	-	-	-	+	-	-	-	-	-	-	-	-	-	-	-	-	-	-	-	-	-	-	-
2592	3874	hypothetical protein	7	-	-	-	-	-	-	-	-	-	-	-	-	-	-	-	-	-	-	-	-	-	-	-	-	+	+	+	+	+	+	-	-	-	-	-	-	-	-	-	-	-	-	-	-	-	-	-	+	-	-	-	-	-	-	-	-	-
2593	3875	hypothetical protein	7	-	-	-	-	-	-	-	-	-	-	-	-	-	-	-	+	-	-	-	-	-	-	-	-	+	-	+	+	-	+	+	+	-	-	-	-	-	-	-	-	-	-	-	-	-	-	-	-	-	-	-	-	-	-	-	-	-
2594	3877	phage protein	7	-	-	-	-	-	-	-	-	-	-	-	-	-	-	+	+	-	-	-	-	-	-	-	-	+	-	-	-	-	-	-	-	-	+	-	-	-	-	-	-	-	-	-	+	-	-	+	-	-	+	-	-	-	-	-	-	-
2595	3879	hypothetical protein	7	-	-	-	-	-	-	-	-	-	-	-	-	-	-	-	-	-	-	-	-	-	-	-	-	+	+	+	+	+	+	-	-	-	-	-	-	-	-	-	-	-	-	-	-	-	-	-	-	-	-	-	-	-	-	-	-	+
2596	3880	hypothetical protein	7	-	-	-	-	-	-	-	-	-	-	-	-	-	-	-	-	-	-	-	-	-	-	-	-	+	+	+	+	+	+	-	-	-	+	-	-	-	-	-	-	-	-	-	-	-	-	-	-	-	-	-	-	-	-	-	-	-
2597	3882	hypothetical protein	7	-	-	-	-	-	-	-	-	-	-	-	-	-	-	-	-	-	-	-	-	-	-	-	-	+	+	+	+	+	+	-	-	-	-	-	-	-	-	-	-	-	-	-	-	-	-	-	+	-	-	-	-	-	-	-	-	-
2598	3883	hypothetical protein	7	-	-	-	-	-	-	-	-	-	-	-	-	-	-	-	-	-	-	-	-	-	-	-	-	+	+	+	+	+	+	-	-	-	-	-	-	-	-	-	-	-	-	-	-	-	-	+	-	-	-	-	-	-	-	-	-	-
2599	3884	prophage Lp1 protein 19	7	-	-	-	-	-	-	-	-	-	-	-	-	-	-	+	-	-	-	-	-	-	-	+	-	+	-	-	-	-	-	-	-	-	-	+	-	-	+	-	-	-	-	-	+	-	-	-	-	-	-	-	-	-	-	-	-	+
2600	3886	hypothetical protein	7	-	-	-	-	-	-	-	-	-	-	-	-	-	-	-	-	-	-	-	-	-	-	-	-	+	+	+	+	+	+	-	-	-	-	-	-	-	-	+	-	-	-	-	-	-	-	-	-	-	-	-	-	-	-	-	-	-
2601	3888	hypothetical protein	7	-	-	-	-	-	-	-	-	-	-	-	-	-	-	-	-	-	-	-	-	-	-	-	-	+	-	-	-	-	-	-	-	-	+	+	+	+	-	+	-	-	-	-	-	-	-	-	-	-	-	-	-	-	-	-	-	+
2602	3895	response regulator	7	-	-	-	-	-	-	-	-	-	-	-	-	-	-	-	-	-	-	-	+	+	+	-	-	-	-	-	-	-	-	-	-	+	-	+	-	-	-	+	+	-	-	-	-	-	-	-	-	-	-	-	-	-	-	-	-	-
2603	3896	hypothetical protein	7	-	-	-	-	+	-	-	-	-	-	-	-	-	-	-	-	-	-	-	-	-	-	-	-	-	-	-	-	-	-	-	-	-	-	-	-	-	-	+	-	-	-	-	-	-	-	-	-	-	+	+	+	+	-	+	-	-
2604	3897	glycosyl transferase, family 2	7	-	-	-	-	-	-	-	-	-	-	-	-	-	-	-	-	-	-	-	+	+	+	-	-	-	-	-	-	-	-	-	-	+	-	+	-	-	-	+	+	-	-	-	-	-	-	-	-	-	-	-	-	-	-	-	-	-
2605	3899	hypothetical protein	7	-	-	-	-	-	-	-	-	+	-	-	-	-	-	-	-	-	-	-	+	-	-	-	-	-	-	-	-	-	-	-	-	-	-	-	-	-	-	+	+	+	-	-	-	-	-	-	-	-	-	+	-	+	-	-
2606	3901	agglutinin receptor	7	-	-	-	-	-	-	-	-	-	-	-	-	-	-	-	-	-	-	-	+	+	+	-	-	-	-	-	-	-	-	-	-	+	-	+	-	-	-	+	+	-	-	-	-	-	-	-	-	-	-	-	-	-	-	-	-	-
2607	3902	MutT/nudix family protein	7	-	-	-	-	-	-	-	-	-	-	-	-	-	-	-	+	-	-	-	-	-	-	-	-	-	-	-	-	-	-	-	-	-	-	-	-	-	+	+	+	+	-	-	-	-	-	+	-	-	-	-	-	+	-	-	-	-
2608	3904	Signal transduction protein with a C-terminal ATPase/histidine kinase domain, putative	7	-	-	-	-	-	-	-	-	-	-	-	-	-	-	-	-	-	-	-	+	+	+	-	-	-	-	-	-	-	-	-	-	+	-	+	-	-	-	+	+	-	-	-	-	-	-	-	-	-	-	-	-	-	-	-	-	-
2609	3913	GlpG protein (membrane protein of glp regulon)	7	-	-	-	-	-	-	-	-	-	-	-	-	-	-	-	-	-	-	-	-	+	+	-	+	-	+	+	-	-	-	-	-	-	-	-	-	-	-	-	-	-	-	-	-	+	+	-	-	-	-	-	-	-	-	-	-	-
2610	3916	putative transposase	7	-	-	-	-	-	-	-	-	-	-	-	-	-	-	-	-	-	+	-	+	+	+	+	+	-	+	-	-	-	-	-	-	-	-	-	-	-	-	-	-	-	-	-	-	-	-	-	-	-	-	-	-	-	-	-	-	-
2611	3918	hypothetical protein	7	-	-	-	-	-	-	-	-	-	-	-	-	-	-	-	+	-	-	-	-	-	-	-	-	-	-	-	-	-	-	-	-	+	+	+	+	+	+	-	-	-	-	-	-	-	-	-	-	-	-	-	-	-	-	-	-	-
2612	3919	Type I restriction-modification system	7	-	-	-	-	-	-	-	-	-	-	-	-	-	-	-	-	-	-	-	-	+	+	-	-	-	-	-	-	-	-	-	-	+	+	+	+	-	+	-	-	-	-	-	-	-	-	-	-	-	-	-	-	-	-	-	-	-
2613	3935	hypothetical protein	7	-	-	-	-	-	-	-	-	-	-	-	-	-	-	-	-	-	-	-	-	-	-	-	-	-	+	+	+	+	+	-	-	-	-	-	-	-	-	-	-	-	+	-	-	-	-	-	+	-	-	-	-	-	-	-	-	-
2614	3942	hypothetical protein	7	-	-	-	-	-	-	-	-	-	-	-	-	-	-	-	-	-	-	-	+	+	+	-	-	-	-	+	-	+	-	-	-	+	-	-	-	-	-	-	-	-	-	-	-	-	-	-	+	-	-	-	-	-	-	-	-	-
2615	3948	hypothetical protein	7	-	-	-	-	-	-	-	-	-	-	-	-	-	-	-	-	-	-	-	-	-	-	-	-	-	-	-	-	-	-	-	-	-	-	-	-	-	-	-	-	+	+	+	+	-	-	+	+	+	-	-	-	-	-	-	-	-
2616	3955	Helix-turn-helix motif	7	-	-	-	-	-	-	-	-	-	-	-	-	-	-	-	-	-	-	-	+	+	+	-	-	-	-	-	-	-	-	-	-	-	-	-	-	-	-	-	-	-	-	-	-	-	-	+	+	+	-	-	+	-	-	-	-	-
2617	3959	hypothetical protein	7	-	-	-	-	-	-	-	-	-	-	-	-	-	-	-	-	-	-	-	-	+	+	-	-	-	-	-	-	-	-	-	-	+	+	+	-	-	-	-	-	-	+	-	-	-	-	-	-	+	-	-	-	-	-	-	-	-
2618	3969	Predicted Zn-dependent protease	7	-	-	-	-	-	-	-	-	-	-	-	-	-	-	-	-	-	-	-	-	-	-	-	-	-	-	-	-	-	-	-	-	-	-	-	-	-	+	-	-	-	-	-	-	-	-	-	-	-	+	+	+	+	+	+	-	-
2619	3971	hypothetical protein	7	-	-	-	-	-	-	-	-	-	-	-	-	-	-	-	-	-	-	-	-	+	+	-	-	-	-	-	+	-	-	-	-	+	-	-	-	-	+	-	-	-	-	-	-	-	-	-	-	-	+	+	-	-	-	-	-	-
2620	3974	hypothetical protein	7	-	-	-	-	-	-	-	-	-	-	-	-	-	-	-	-	-	-	-	-	-	-	-	-	-	-	-	-	-	-	-	-	+	+	+	+	-	+	-	-	-	-	-	-	+	-	-	-	+	-	-	-	-	-	-	-	-
2621	3978	Accessory secretory protein Asp1	7	-	-	-	-	-	-	-	-	-	-	-	-	-	-	-	-	-	-	-	-	-	-	-	-	-	-	-	-	-	-	-	-	-	-	-	+	+	+	-	-	-	-	-	-	-	-	-	-	-	-	+	-	+	+	+	-	-
2622	3981	extracellular factor protein	7	-	-	-	-	-	-	-	-	-	-	-	-	-	-	-	-	-	-	-	-	-	-	-	-	-	-	-	-	-	-	-	-	+	+	+	+	+	+	-	-	-	-	-	-	-	-	-	-	-	-	-	-	-	-	+	-	-
2623	3987	ABC transporter	7	-	-	-	-	-	-	-	-	-	-	-	-	-	-	-	-	-	-	-	-	-	-	-	-	-	-	-	-	-	-	+	+	+	+	+	-	+	-	-	-	-	-	-	-	-	-	-	-	-	-	-	-	+	-	-
2624	3996	hypothetical protein	7	-	-	-	-	+	-	-	-	-	-	-	-	-	-	-	-	-	-	-	+	-	-	-	-	-	-	-	-	-	-	+	+	-	+	-	-	-	-	-	-	-	-	-	-	-	-	-	-	-	+	-	-	+	-	-
2625	4002	hypothetical protein	7	-	-	-	-	+	-	-	-	-	-	-	-	-	-	-	-	-	-	-	+	-	-	-	-	-	-	-	-	-	-	+	+	-	+	-	-	-	-	-	-	-	-	-	-	-	-	-	-	-	+	-	-	-	+	-
2626	4004	hypothetical protein	7	-	-	-	-	+	-	-	-	-	-	-	-	-	-	-	-	-	-	-	+	-	-	-	-	-	-	-	-	-	-	+	+	-	+	-	-	-	-	-	-	-	-	-	-	+	-	-	-	-	+	-	-	-	-	-
2627	4044	Na+-driven multidrug efflux pump	7	-	-	-	-	+	-	-	+	+	-	-	-	-	+	-	-	-	-	-	+	-	-	-	-	-	-	-	-	-	-	+	+	-	-	-	-	-	-	-	-	-	-	-	-	-	-	-	-	-	-	-	-	-	-	-
2628	4045	hypothetical protein	7	+	+	+	+	+	+	-	+	-	-	-	-	-	-	-	-	-	-	-	-	-	-	-	-	-	-	-	-	-	-	-	-	-	-	-	-	-	-	-	-	-	-	-	-	-	-	-	-	-	-	-	-	-	-	-
2629	2140	CRISPR-associated protein, Csn2 family	6	-	-	-	-	-	-	-	-	-	-	-	-	-	-	-	-	+	+	-	-	-	-	-	-	-	-	-	-	-	-	-	-	+	-	-	-	-	-	+	+	-	-	-	-	-	-	-	-	-	-	-	-	-	-	-	-	+
2630	2170	CRISPR-associated protein, Csn1 family	6	-	-	-	-	-	-	-	-	-	-	-	-	-	-	-	-	+	+	-	-	-	-	-	-	-	-	-	-	-	-	-	-	+	-	-	-	-	-	+	+	-	-	-	-	-	-	-	-	-	-	-	-	-	-	-	-	+
2631	2172	CRISPR-associated protein Cas1	6	-	-	-	-	-	-	-	-	-	-	-	-	-	-	-	-	+	+	-	-	-	-	-	-	-	-	-	-	-	-	-	-	+	-	-	-	-	-	+	+	-	-	-	-	-	-	-	-	-	-	-	-	-	-	-	-	+
2632	2194	hypothetical protein	6	-	-	-	-	-	-	-	-	-	-	-	-	-	-	-	-	-	-	-	-	-	-	-	-	-	-	-	-	-	-	+	+	-	-	-	-	-	-	-	-	+	+	-	-	+	-	-	-	-	-	-	+	-	-	-	-	-
2633	2223	CRISPR-associated protein Cas2	6	-	-	-	-	-	-	-	-	-	-	-	-	-	-	-	-	+	+	-	-	-	-	-	-	-	-	-	-	-	-	-	-	+	-	-	-	-	-	+	+	-	-	-	-	-	-	-	-	-	-	-	-	-	-	-	-	+
2634	2303	hypothetical protein	6	-	-	-	-	-	-	-	-	-	-	-	-	-	-	-	-	-	-	+	-	-	-	-	-	-	-	-	-	-	-	-	-	+	+	+	-	-	-	-	-	-	-	-	-	-	-	-	+	-	-	-	-	-	-	-	+	-
2635	2349	LSU ribosomal protein L33p @ LSU ribosomal protein L33p, zinc-dependent	6	-	-	-	-	-	-	-	-	-	-	-	-	-	-	-	-	-	-	-	-	-	-	+	-	-	-	+	+	+	-	-	-	-	-	+	-	-	-	-	-	-	-	-	-	-	-	-	-	-	-	-	-	-	-	+	-	-
2636	2406	hypothetical protein	6	+	+	+	-	-	-	-	-	-	-	+	-	-	-	-	-	-	-	-	-	-	-	-	-	-	-	-	-	-	-	-	-	-	-	-	-	-	-	-	-	-	-	-	-	+	-	-	-	-	+	-	-	-	-	-	-	-
2637	2470	hypothetical protein	6	-	-	-	-	-	-	-	-	-	-	-	-	-	-	-	-	-	-	-	-	-	-	-	-	-	-	-	-	-	-	-	-	-	-	-	-	-	-	-	-	-	-	+	+	-	-	+	+	+	+	-	-	-	-	-	-	-
2638	2477	FIG086557: Conjugation related protein	6	+	+	+	-	-	-	-	-	-	-	+	-	-	-	-	-	-	-	-	-	-	-	-	-	-	-	-	-	-	-	-	-	-	-	-	-	-	-	-	-	-	-	-	-	+	-	-	-	-	+	-	-	-	-	-	-	-
2639	2479	hypothetical protein	6	+	+	+	-	-	-	-	-	-	-	+	-	-	-	-	-	-	-	-	-	-	-	-	-	-	-	-	-	-	-	-	-	-	-	-	-	-	-	-	-	-	-	-	-	+	-	-	-	-	+	-	-	-	-	-	-	-
2640	2480	lipoprotein, NLP/P60 family	6	+	+	+	-	-	-	-	-	-	-	+	-	-	-	-	-	-	-	-	-	-	-	-	-	-	-	-	-	-	-	-	-	-	-	-	-	-	-	-	-	-	-	-	-	+	-	-	-	-	+	-	-	-	-	-	-	-
2641	2541	hypothetical protein	6	-	-	-	-	-	-	-	-	-	-	-	-	-	-	-	-	-	-	-	-	-	-	-	-	-	-	-	-	-	-	-	-	-	-	-	+	+	+	+	+	-	-	-	-	+	-	-	-	-	-	-	-	-	-	-	-	-
2642	2559	membrane protein, putative	6	+	+	+	-	-	-	-	-	-	-	+	-	-	-	-	-	-	-	-	-	-	-	-	-	-	-	-	-	-	-	-	-	-	-	-	-	-	-	-	-	-	-	-	-	+	-	-	-	-	+	-	-	-	-	-	-	-
2643	2562	hypothetical protein	6	+	+	+	-	-	-	-	-	-	-	+	-	-	-	-	-	-	-	-	-	-	-	-	-	-	-	-	-	-	-	-	-	-	-	-	-	-	-	-	-	-	-	-	-	+	-	-	-	-	+	-	-	-	-	-	-	-
2644	2564	hypothetical protein	6	+	+	+	-	-	-	-	-	-	-	+	-	-	-	-	-	-	-	-	-	-	-	-	-	-	-	-	-	-	-	-	-	-	-	-	-	-	-	-	-	-	-	-	-	+	-	-	-	-	+	-	-	-	-	-	-	-
2645	2567	hypothetical protein	6	+	+	+	-	-	-	-	-	-	-	+	-	-	-	-	-	-	-	-	-	-	-	-	-	-	-	-	-	-	-	-	-	-	-	-	-	-	-	-	-	-	-	-	-	+	-	-	-	-	+	-	-	-	-	-	-	-
2646	2568	Antirestriction protein	6	+	+	+	-	-	-	-	-	-	-	+	-	-	-	-	-	-	-	-	-	-	-	-	-	-	-	-	-	-	-	-	-	-	-	-	-	-	-	-	-	-	-	-	-	+	-	-	-	-	+	-	-	-	-	-	-	-
2647	2569	hypothetical protein	6	+	+	+	-	-	-	-	-	-	-	+	-	-	-	-	-	-	-	-	-	-	-	-	-	-	-	-	-	-	-	-	-	-	-	-	-	-	-	-	-	-	-	-	-	+	-	-	-	-	+	-	-	-	-	-	-	-
2648	2577	Prophage Clp protease-like protein	6	-	-	-	-	-	-	-	-	+	-	-	-	-	-	-	-	-	-	-	-	-	-	-	-	-	-	-	-	-	-	-	-	-	-	-	-	-	-	-	-	-	-	-	-	+	-	-	-	+	-	+	-	-	+	-	+	-
2649	2645	replication initiator protein A	6	-	-	-	-	-	-	-	-	-	-	-	-	-	-	-	-	-	-	-	-	-	-	-	-	-	-	-	-	-	-	-	-	-	-	-	-	-	-	-	-	-	-	-	+	-	-	+	+	+	+	-	-	-	-	-	+	-
2650	2656	conjugative transposon membrane protein	6	+	+	+	-	-	-	-	-	-	-	+	-	-	-	-	-	-	-	-	-	-	-	-	-	-	-	-	-	-	-	-	-	-	-	-	-	-	-	-	-	-	-	-	-	+	-	-	-	-	+	-	-	-	-	-	-	-
2651	2669	Phage major capsid protein	6	-	-	-	-	-	-	-	-	+	-	-	-	-	-	-	-	-	-	-	-	-	-	-	-	-	-	-	-	-	-	-	-	-	-	-	-	-	-	-	-	-	-	-	-	+	-	-	-	+	-	+	-	-	+	-	+	-
2652	2670	Phage portal protein	6	-	-	-	-	-	-	-	-	+	-	-	-	-	-	-	-	-	-	-	-	-	-	-	-	-	-	-	-	-	-	-	-	-	-	-	-	-	-	-	-	-	-	-	-	+	-	-	-	+	-	+	-	-	+	-	+	-
2653	2738	ABC transporter ATP-binding protein	6	-	-	-	-	-	-	-	-	-	-	-	-	-	-	-	-	+	+	-	-	-	-	-	-	-	-	-	-	-	-	-	-	-	-	-	-	-	-	-	-	-	-	-	-	-	+	-	-	+	-	-	-	-	-	+	-	+
2654	2790	Phage protein	6	-	-	-	-	-	-	-	-	+	-	-	-	-	-	-	-	-	-	-	-	-	-	-	-	-	-	-	-	-	-	-	-	-	-	-	-	-	-	-	-	-	-	-	-	+	-	-	-	+	-	+	-	-	+	-	+	-
2655	2793	Phage-associated cell wall hydrolase	6	-	-	-	-	-	-	-	-	+	-	-	-	-	-	-	-	-	-	-	-	-	-	-	-	-	-	-	-	-	-	-	-	-	-	-	-	-	-	-	-	-	-	-	-	+	-	-	-	+	-	+	-	+	+	-	-	-
2656	2795	Phage protein	6	-	-	-	-	-	-	-	-	+	-	-	-	-	-	-	-	-	-	-	-	-	-	-	-	-	-	-	-	-	-	-	-	-	-	-	-	-	-	-	-	-	-	-	-	+	-	-	-	+	-	+	-	-	+	-	+	-
2657	2873	FIG006036: phage encoded DNA polymerase I (EC 2.7.7.7)	6	-	-	-	-	-	-	-	-	+	-	-	-	-	-	-	-	-	-	-	-	-	-	-	-	-	-	-	-	-	-	-	-	-	-	-	-	-	-	-	-	-	-	-	-	+	-	-	-	+	-	+	-	-	+	-	+	-
2658	2875	DNA helicase, phage-associated	6	-	-	-	-	-	-	-	-	+	-	-	-	-	-	-	-	-	-	-	-	-	-	-	-	-	-	-	-	-	-	-	-	-	-	-	-	-	-	-	-	-	-	-	-	+	-	-	-	+	-	+	-	-	+	-	+	-
2659	2887	NAD-dependent protein deacetylase of SIR2 family	6	-	-	-	-	-	-	-	-	-	-	-	-	-	-	-	-	-	-	-	-	-	-	-	-	-	-	-	-	-	-	-	-	-	-	-	-	-	-	-	-	-	-	+	-	+	-	-	-	-	-	+	+	-	-	-	+	+
2660	2888	ISSpo3, transposase	6	-	-	-	-	-	-	-	-	-	-	-	-	-	-	-	-	-	-	-	-	-	-	-	-	-	-	-	-	-	-	-	-	-	-	-	-	-	-	-	-	+	+	-	-	+	-	+	+	-	-	-	-	-	-	-	-	+
2661	2910	Phage terminase small subunit	6	-	-	-	-	-	-	-	-	+	-	-	-	-	-	-	-	-	-	-	-	-	-	-	-	-	-	-	-	-	-	-	-	-	-	-	-	-	-	-	-	-	-	-	-	+	-	-	-	+	+	+	-	-	+	-	-	-
2662	2911	Tetratricopeptide TPR_4	6	-	-	-	-	-	-	-	-	-	-	-	-	-	-	-	-	-	-	-	-	-	-	-	-	-	-	-	-	-	-	-	-	-	-	-	-	+	-	-	-	-	-	-	-	-	-	-	-	-	+	+	+	-	+	+	-	-
2663	2916	Phage protein	6	-	-	-	-	-	-	-	-	+	-	-	-	-	-	-	-	-	-	-	-	-	-	-	-	-	-	-	-	-	-	-	-	-	-	-	-	-	-	-	-	-	-	-	-	+	-	-	-	+	-	+	-	-	+	-	+	-
2664	3029	hypothetical protein	6	-	-	-	-	-	-	-	-	-	-	-	-	-	-	-	-	-	-	-	-	-	-	-	-	-	-	-	-	-	-	-	-	-	-	-	-	-	-	-	-	-	-	+	+	-	-	+	+	+	+	-	-	-	-	-	-	-
2665	3030	hypothetical protein	6	-	-	-	-	-	-	-	-	-	-	-	-	-	-	-	-	-	-	-	-	-	-	-	-	-	-	-	-	-	-	-	-	-	-	-	-	-	-	-	-	-	-	+	+	-	-	+	+	+	+	-	-	-	-	-	-	-
2666	3031	hypothetical protein	6	-	-	-	-	-	-	-	-	-	-	-	-	-	-	-	-	-	-	-	-	-	-	-	-	-	-	-	-	-	-	-	-	-	-	-	-	-	-	-	-	-	-	+	+	-	-	+	+	+	+	-	-	-	-	-	-	-
2667	3032	hypothetical protein	6	-	-	-	-	-	-	-	-	-	-	-	-	-	-	-	-	-	-	-	-	-	-	-	-	-	-	-	-	-	-	-	-	-	-	-	-	-	-	-	-	-	-	+	+	-	-	+	+	+	+	-	-	-	-	-	-	-
2668	3036	hypothetical protein	6	-	-	-	-	-	-	-	-	+	-	-	-	-	-	-	-	-	-	-	-	-	-	-	-	-	-	-	-	-	-	-	-	-	-	-	-	-	-	-	-	-	-	-	-	+	-	-	-	+	+	+	-	-	+	-	-	-
2669	3107	hypothetical protein	6	-	-	-	-	-	-	-	-	-	-	-	-	-	-	+	-	-	-	+	-	-	-	-	-	+	-	-	-	-	-	-	-	+	-	-	-	-	-	-	-	+	-	+	-	-	-	-	-	-	-	-	-	-	-	-	-	-
2670	3136	Phage excisionase	6	-	-	-	-	-	-	-	-	-	-	-	-	-	-	-	-	-	-	-	-	-	-	-	-	-	-	-	-	-	-	-	-	-	-	-	-	-	-	-	-	+	-	-	-	-	-	+	+	+	-	-	+	-	-	+	-	-
2671	3137	hypothetical protein	6	-	-	-	-	-	-	-	-	-	-	-	-	-	-	-	-	-	-	-	-	-	-	-	-	-	-	-	-	-	-	-	-	-	-	-	-	-	-	-	-	-	-	+	+	-	-	+	+	+	+	-	-	-	-	-	-	-
2672	3248	Mobile element protein	6	-	-	-	-	-	-	-	+	-	-	-	-	-	-	-	-	-	-	-	-	-	-	-	-	-	-	-	-	-	-	-	-	+	-	-	-	+	-	-	-	-	-	-	+	-	-	-	-	-	-	+	-	+	-	-
2673	3259	Adenine-specific methyltransferase (EC 2.1.1.72)	6	-	-	-	-	-	-	-	-	-	-	-	-	-	-	+	-	-	-	-	-	-	-	-	-	+	-	-	-	-	-	-	-	-	-	+	-	-	-	-	-	-	-	-	+	+	-	-	-	-	-	-	-	+	-	-
2674	3309	hypothetical protein	6	-	-	-	-	-	-	-	-	-	-	-	-	-	-	-	-	-	-	-	-	-	-	-	-	-	-	-	-	-	-	-	-	-	-	-	-	-	-	-	-	-	-	+	-	-	-	-	-	-	+	-	+	+	+	+	-	-
2675	3310	Thiol:disulfide oxidoreductase related to ResA	6	-	-	-	-	-	-	-	-	-	-	-	-	-	-	-	-	-	-	-	-	-	-	-	-	-	-	-	-	-	-	-	-	-	-	-	-	-	-	-	-	-	-	-	-	-	-	-	-	-	+	+	+	+	+	+	-	-
2676	3319	Type II restriction endonuclease	6	-	-	-	-	-	-	-	-	-	-	-	-	-	-	-	-	-	-	-	-	-	-	-	-	-	-	-	-	-	-	-	-	-	-	-	+	+	+	-	-	-	-	-	+	-	-	-	-	-	-	-	+	+	-	-	-	-	-	-
2677	3461	Conjugative transfer protein TrbI	6	-	-	-	-	-	-	-	-	-	-	-	-	-	-	-	-	-	-	+	-	-	-	-	-	-	-	-	-	-	-	-	-	+	+	+	-	-	-	-	-	-	-	-	-	-	-	-	+	-	-	-	-	-	-	-	+	-
2678	3468	Mobile element protein	6	-	-	-	-	-	-	-	-	-	-	-	-	-	-	-	-	-	-	-	-	-	-	-	-	-	-	-	+	-	-	-	-	-	-	-	-	-	+	-	-	+	+	-	-	-	-	-	-	-	-	-	+	+	-	-	-	-
2679	3483	Cytochrome c-type biogenesis protein CcdA (DsbD analog)	6	-	-	-	-	-	-	-	-	-	-	-	-	-	-	-	-	-	-	-	-	-	-	-	-	-	-	-	-	-	-	-	-	-	-	-	-	-	-	-	-	-	-	-	-	-	-	-	-	-	+	+	+	+	+	+	-	-
2680	3495	Lantibiotic mersacidin modifying enzyme	6	-	-	-	-	-	-	-	-	-	-	-	-	-	-	-	-	-	-	-	-	+	+	-	-	-	-	-	-	+	-	-	-	+	-	-	-	-	-	-	-	-	-	-	-	-	-	-	-	-	-	-	-	-	-	+	+	-
2681	3623	Phage CI-like repressor	6	-	-	-	-	-	-	-	-	-	-	-	-	-	-	-	-	-	-	-	-	-	-	-	-	+	-	-	-	-	-	-	-	-	+	-	+	+	-	-	-	-	-	-	-	-	-	-	+	-	-	-	-	-	+	-	-	-
2682	3624	Polysaccharide biosynthesis protein CpsF	6	-	-	-	-	-	-	-	-	+	-	-	-	+	+	-	-	+	+	-	-	-	-	-	-	-	-	-	-	-	-	-	-	-	-	-	-	-	-	-	-	-	-	-	-	-	-	+	-	-	-	-	-	-	-	-	-	-
2683	3630	Phage terminase, large subunit	6	-	-	-	-	-	-	-	-	-	-	-	-	-	-	+	+	+	+	-	-	-	-	-	-	-	-	-	-	-	-	-	-	-	-	-	-	-	-	-	-	-	-	-	+	-	-	+	-	-	-	-	-	-	-	-	-	-
2684	3653	CAAX amino terminal protease family protein	6	-	-	-	-	-	-	-	-	-	-	-	-	-	-	+	+	-	-	-	-	-	-	-	-	-	-	-	-	-	-	-	-	-	-	-	-	-	-	+	+	-	+	+	-	-	-	-	-	-	-	-	-	-	-	-	-	-
2685	3654	putative PTS system, IID component	6	-	-	-	-	-	-	-	-	+	-	-	-	-	-	-	-	-	-	-	-	-	-	-	-	-	-	-	-	-	-	-	-	-	-	-	+	+	-	+	+	-	-	-	-	-	-	-	-	-	-	-	-	-	+	-	-	-
2686	3674	Cu+ P-type ATPase	6	-	-	-	-	-	-	-	-	-	-	-	-	-	-	-	-	-	-	-	-	-	-	-	-	-	-	-	-	-	-	-	-	-	-	-	-	-	-	-	-	-	-	-	-	-	-	-	-	+	+	-	+	+	-	+	-	+
2687	3678	Transcriptional regulator, MerR family	6	-	-	-	-	-	-	-	-	-	-	-	-	-	-	-	-	-	-	-	-	-	-	-	-	-	-	-	-	-	-	-	-	-	-	-	-	-	-	-	-	-	-	-	-	-	-	-	+	-	-	+	+	+	+	+	-	-
2688	3685	Nisin biosynthesis protein nisC	6	-	-	-	-	-	-	+	-	+	-	-	-	-	-	-	-	-	-	-	-	-	-	-	-	-	-	-	-	-	-	-	-	-	-	-	-	-	-	-	-	-	+	-	-	-	-	-	-	-	+	-	+	+	-	-	-	-
2689	3688	Subtilin transport ATP-binding protein spaT	6	-	-	-	-	-	-	+	-	+	-	-	-	-	-	-	-	-	-	-	-	-	-	-	-	-	-	-	-	-	-	-	-	-	-	-	-	-	-	-	-	-	+	-	-	-	-	-	-	-	+	-	+	+	-	-	-	-
2690	3696	hypothetical protein	6	-	-	-	-	-	-	-	-	-	-	-	-	-	-	-	-	-	-	-	-	-	-	-	-	-	-	-	-	-	-	-	-	-	-	-	-	-	-	-	-	-	-	-	+	-	-	-	-	+	+	+	+	-	+	-	-	-
2691	3861	ABC transporter, ATP-binding protein	6	-	-	-	-	-	-	-	-	-	-	-	-	-	-	-	-	+	+	+	-	-	-	-	-	-	-	-	-	-	-	-	-	-	-	-	-	-	-	+	+	-	-	-	-	-	-	-	-	-	-	+	-	-	-	-	-	-
2692	3863	hypothetical protein	6	-	-	-	-	-	-	-	-	-	-	-	-	-	-	-	-	-	-	-	-	-	-	-	-	-	-	-	-	-	-	-	-	-	-	-	-	-	-	-	-	-	-	-	-	-	-	-	-	-	+	+	+	+	+	+	-	-
2693	3868	hypothetical protein	6	-	-	-	-	-	-	-	-	-	-	-	-	-	-	-	-	-	-	-	-	-	-	-	-	-	-	-	-	-	-	-	-	-	-	-	+	+	-	-	+	-	-	-	+	-	-	-	-	-	-	+	-	-	-	+	-	-
2694	3872	hypothetical protein	6	-	-	-	-	-	-	-	-	-	-	-	-	-	-	+	-	+	+	+	-	-	-	-	-	-	-	-	-	-	-	-	-	-	-	+	-	-	-	-	-	-	-	-	+	-	-	-	-	-	-	-	-	-	-	-	-	-
2695	3876	Negative regulator of phenolic acid metabolism PadR	6	-	-	-	-	-	-	-	-	-	-	-	-	-	-	-	-	-	-	-	-	-	-	-	-	+	+	+	+	+	+	-	-	-	-	-	-	-	-	-	-	-	-	-	-	-	-	-	-	-	-	-	-	-	-	-	-	-
2696	3878	hypothetical protein	6	-	-	-	-	-	-	-	-	-	-	-	-	-	-	-	-	-	-	-	-	-	-	-	-	+	-	+	+	+	+	-	-	-	-	+	-	-	-	-	-	-	-	-	-	-	-	-	-	-	-	-	-	-	-	-	-	-
2697	3885	Phage protein # Prophage Lp1 Protein 19	6	-	-	-	-	-	-	-	-	-	-	-	-	-	-	-	-	-	-	-	-	-	-	-	-	-	-	-	-	-	-	-	-	-	+	-	+	+	-	-	-	-	-	-	-	-	-	-	-	-	-	-	+	+	-	+	-	-
2698	3887	hypothetical protein	6	-	-	-	-	-	-	-	-	-	-	-	-	-	-	-	-	-	-	-	-	-	-	-	-	+	-	-	+	+	-	-	-	+	+	+	-	-	-	-	-	-	-	-	-	-	-	-	-	-	-	-	-	-	-	-	-	-
2699	3889	hypothetical protein	6	-	-	-	-	-	-	-	-	-	-	-	-	-	-	-	-	-	-	-	-	-	-	-	-	+	+	+	+	+	+	-	-	-	-	-	-	-	-	-	-	-	-	-	-	-	-	-	-	-	-	-	-	-	-	-	-	-
2700	3890	Transcriptional regulator	6	-	-	-	-	-	-	-	-	-	-	-	-	-	-	+	+	-	-	-	-	-	-	-	-	-	-	-	-	-	-	-	-	-	-	-	-	-	-	+	+	-	+	+	-	-	-	-	-	-	-	-	-	-	-	-	-	-
2701	3893	hypothetical protein	6	-	-	-	-	-	-	-	-	-	-	-	-	-	-	+	+	-	-	-	-	-	-	-	-	-	-	-	-	-	-	-	-	-	-	-	-	-	-	+	+	-	-	-	-	-	-	-	+	-	-	-	-	-	-	-	+	-
2702	3894	phosphoenolpyruvate-dependent sugar phosphotransferase system EIIC, probable sorbose specific	6	-	-	-	-	-	-	-	-	+	-	-	-	-	-	-	-	-	-	-	-	-	-	-	-	-	-	-	-	-	-	-	-	-	-	-	+	+	-	+	+	-	-	-	-	-	-	-	-	-	-	-	-	-	+	-	-	-
2703	3898	phosphoenolpyruvate-dependent sugar phosphotransferase system EIIB, probable sorbose specific	6	-	-	-	-	-	-	-	-	+	-	-	-	-	-	-	-	-	-	-	-	-	-	-	-	-	-	-	-	-	-	-	-	-	-	-	+	+	-	+	+	-	-	-	-	-	-	-	-	-	-	-	-	-	+	-	-	-
2704	3909	hypothetical protein	6	-	-	-	-	-	-	-	-	-	-	-	-	-	-	-	-	-	-	-	+	-	-	-	+	-	+	+	-	-	-	-	-	-	-	-	-	-	-	-	+	-	-	-	-	-	-	-	+	-	-	-	-	-	-	-	-	-
2705	3911	hypothetical protein	6	-	-	-	-	-	-	-	-	-	-	-	-	-	-	-	-	-	-	-	-	-	-	-	-	-	-	-	-	-	-	-	-	-	-	-	-	-	-	-	-	-	-	-	-	+	+	+	-	+	-	-	-	-	-	-	+	+
2706	3912	thiol:disulfide interchange protein (thioredoxin)	6	-	-	-	-	-	-	-	-	-	-	-	-	-	-	-	-	-	-	-	-	-	-	-	-	-	-	-	-	-	-	-	-	-	-	-	-	-	-	-	-	-	-	-	+	+	+	+	+	+	-	-	-	-	-	-	-	-
2707	3914	hypothetical protein	6	-	-	-	-	-	-	-	-	-	-	-	-	-	-	-	-	-	-	-	+	+	+	+	-	-	-	-	-	-	-	-	-	-	-	-	+	-	-	-	-	-	-	-	-	-	-	-	-	-	-	-	-	-	+	-	-	-
2708	3915	hypothetical protein	6	-	-	-	-	-	-	-	-	-	-	-	-	-	-	-	+	-	-	-	-	-	-	+	+	-	-	+	-	-	+	-	-	-	-	-	-	-	+	-	-	-	-	-	-	-	-	-	-	-	-	-	-	-	-	-	-	-
2709	3917	hypothetical protein	6	-	-	-	-	-	-	-	-	-	-	-	-	-	-	-	-	-	-	-	-	-	-	+	-	-	-	-	-	-	-	-	-	-	-	+	-	-	+	-	-	-	-	-	-	+	-	-	-	-	-	+	-	-	-	+	-	-
2710	3925	hypothetical protein	6	-	-	-	-	-	-	-	-	-	-	-	-	-	-	-	-	-	-	-	-	-	-	-	-	-	-	-	-	-	-	-	-	-	-	-	-	-	-	-	-	+	+	-	-	+	-	+	+	-	-	-	-	-	-	-	-	+
2711	3934	hypothetical protein	6	-	-	-	-	-	-	-	-	-	-	-	-	-	-	-	-	-	-	-	-	-	-	-	-	-	-	-	-	-	-	-	-	-	-	-	-	-	-	-	-	-	-	-	+	-	-	+	+	+	+	-	-	-	-	-	+	-
2712	3937	hypothetical protein	6	-	-	-	-	-	-	-	-	-	-	-	-	-	-	-	-	-	-	-	-	-	-	-	-	-	-	-	-	-	-	-	-	-	-	-	-	-	-	-	-	-	-	+	+	-	-	+	+	+	+	-	-	-	-	-	-	-
2713	3945	hypothetical protein	6	-	-	-	-	-	-	-	-	-	-	-	-	-	-	-	-	-	-	-	-	-	-	-	-	-	-	-	-	-	-	+	+	-	+	-	-	-	+	-	-	-	-	-	-	-	-	-	+	-	-	-	-	-	+	-	-	-
2714	3947	Retron-type RNA-directed DNA polymerase (EC 2.7.7.49)	6	-	-	-	-	-	-	-	-	-	-	-	-	-	-	-	-	-	-	-	+	-	-	-	-	-	-	-	-	-	+	+	+	+	-	-	-	-	-	-	-	-	-	-	-	-	-	-	+	-	-	-	-	-	-	-	-	-

Table S5. Precence/absence of each HG among 57 *S. suis* strains

				1																13			25					28						104		94			76			53/54		Divergent *S. suis*																
Index[a]	ClusterID	Gene products	Members	98HAH33	05ZYH33	SC84	A7	SS12	P1/7	DAT273	NIAH11434	JS14	GZ1	BM407	S735	13730	MNCM07	NCTC 10446	14636	NCTC 10237	ST1	2651	89-1591	MNCM04	MNCM25	8074	D9	4961	ST3	MNCM43	DAT292	DAT260	NIAH11435	MNCM21	MNCM50	89-2479	DAT234	DAT290	93A	42A	NT77	6407	11538	8830	12814	92-1400	89-590	2726	2524	89-3576-3	4417	D12	10581	89-5259	88-5299A	R61	92-1191	14A	92-4172	22083
2715	3949	hypothetical protein	6	-	-	-	-	-	-	-	-	-	-	-	-	-	-	-	-	-	-	-	+	-	-	-	-	-	+	-	-	+	-	-	-	-	-	-	-	-	-	-	-	-	-	-	+	-	-	+	+	-	-	-	-	-	-	-	-	-
2716	3963	hypothetical protein	6	-	-	-	-	-	-	-	-	-	-	-	-	-	-	-	-	-	-	-	-	-	-	-	-	-	-	-	-	-	-	-	-	-	-	-	-	-	+	-	-	-	-	-	-	-	-	+	-	-	+	+	-	+	+	-	-	-
2717	3964	putative amino acid ABC transporter, ATP-binding protein	6	-	-	-	-	-	-	-	-	-	-	-	-	-	-	-	-	-	-	-	-	-	-	-	-	-	-	-	-	-	-	-	-	-	-	-	-	-	-	-	-	-	-	-	-	-	-	-	-	-	+	+	+	+	+	+	-	-
2718	3983	DNA/RNA HELICASE (DEAD/DEAH BOX FAMILY)	6	-	-	-	-	-	-	-	-	-	-	-	-	-	-	-	-	-	-	-	-	-	-	-	-	-	-	-	-	-	-	-	-	+	+	+	+	+	+	-	-	-	-	-	-	-	-	-	-	-	-	-	-	-	-	-	-	-
2719	4161	Phage minor tail protein	6	-	-	-	-	-	-	-	-	-	-	-	-	-	-	+	+	+	+	-	-	-	-	-	-	-	-	-	-	-	-	-	-	-	-	-	-	-	-	-	-	-	-	-	+	-	-	+	-	-	-	-	-	-	-	-	-	-
2720	4162	hypothetical protein	6	-	-	-	-	-	-	-	-	+	-	-	-	-	-	-	-	-	-	-	-	-	-	-	-	-	-	-	-	-	-	-	-	-	-	-	-	-	-	-	-	-	-	-	-	+	-	-	-	+	-	+	-	+	+	-	-	-
2721	4163	hypothetical protein	6	-	-	-	-	-	-	-	-	-	-	-	-	-	-	-	-	+	+	+	-	-	-	-	-	-	-	-	-	-	-	-	-	-	-	-	-	-	-	+	+	-	-	-	-	-	-	-	-	-	-	+	-	-	-	-	-	-
2722	4164	B-cell receptor associated protein-related protein	6	-	-	-	-	-	-	-	-	-	-	-	-	-	-	-	+	+	+	-	-	-	-	-	-	-	-	-	-	-	-	-	-	-	-	-	-	-	-	-	-	-	-	-	+	-	-	-	-	-	-	-	-	-	-	+	+	-
2723	4166	hypothetical protein	6	-	-	-	-	-	-	-	-	-	-	-	-	-	-	-	-	+	-	-	-	-	-	-	-	-	-	-	-	-	-	-	-	-	-	+	-	-	-	-	-	-	-	-	-	+	-	-	-	-	+	+	-	-	+	-	-	-
2724	4167	hypothetical protein	6	-	-	-	-	-	-	-	-	-	-	-	-	-	-	-	-	+	+	-	-	-	-	-	-	-	-	-	-	-	-	-	-	-	-	-	-	-	-	+	+	-	-	+	-	-	-	+	-	-	-	-	-	-	-	-	-	-
2725	4169	hypothetical protein	6	-	-	-	-	-	-	-	-	+	-	+	+	+	+	-	-	+	-	-	-	-	-	-	-	-	-	-	-	-	-	-	-	-	-	-	-	-	-	-	-	-	-	-	-	-	-	-	-	-	-	-	-	-	-	-	-	-
2726	4171	hypothetical protein	6	-	-	-	-	-	-	-	-	-	-	-	-	-	-	-	-	+	+	-	-	-	-	-	-	-	-	-	-	-	-	-	-	-	-	-	-	-	-	-	-	-	-	-	-	-	-	-	-	-	+	+	-	+	-	-	-	+
2727	4173	ABC transporter membrane-spanning permease - amino acid transport	6	-	-	-	-	-	-	-	-	-	-	-	-	-	-	-	-	+	+	-	-	-	-	-	-	-	-	-	-	-	-	-	-	-	-	-	-	-	-	-	-	-	-	-	-	-	+	-	-	+	-	-	-	-	-	+	-	+
2728	4175	oxidoreductase	6	-	-	-	-	-	-	-	-	-	-	+	-	-	-	-	-	+	+	-	-	-	-	-	-	-	-	-	-	-	-	-	-	-	-	-	-	-	-	+	+	-	-	-	-	+	-	-	-	-	-	-	-	-	-	-	-	-
2729	4176	RecX	6	-	-	-	-	-	-	-	-	-	-	-	-	-	-	-	-	+	+	-	-	-	-	-	-	-	-	-	-	-	-	-	-	-	-	-	-	-	-	-	-	-	+	-	-	+	-	+	-	+	-	-	-	-	-	-	-	-
2730	4177	lipoprotein	6	-	-	-	-	-	-	-	-	-	-	-	-	-	-	-	-	-	-	-	-	-	-	-	-	+	-	+	+	+	+	-	-	-	-	-	-	-	-	-	-	-	-	+	-	-	-	-	-	-	-	-	-	-	-	-	-	-
2731	4179	D-3-phosphoglycerate dehydrogenase	6	-	-	-	-	-	-	-	-	-	-	-	-	-	-	-	-	-	-	-	-	-	-	-	-	+	-	+	+	+	+	-	-	-	-	-	-	-	-	-	-	-	-	+	-	-	-	-	-	-	-	-	-	-	-	-	-	-
2732	4180	hypothetical protein	6	-	-	-	-	-	-	-	-	-	-	-	-	-	-	-	-	-	-	-	-	-	-	-	-	+	-	+	+	+	+	-	-	-	-	-	-	-	-	-	-	-	-	+	-	-	-	-	-	-	-	-	-	-	-	-	-	-
2733	4181	hypothetical protein	6	-	-	-	-	-	-	-	-	-	-	-	-	-	-	-	-	-	-	-	-	+	+	+	+	+	+	-	-	-	-	-	-	-	-	-	-	-	-	-	-	-	-	-	-	-	-	-	-	-	-	-	-	-	-	-	-	-
2734	4182	hypothetical protein	6	-	-	-	-	-	-	-	-	-	-	-	-	-	-	-	-	-	-	-	-	-	-	-	-	+	-	+	+	+	+	-	-	-	-	-	-	-	-	-	-	-	-	+	-	-	-	-	-	-	-	-	-	-	-	-	-	-
2735	4184	hypothetical protein	6	-	-	-	-	-	-	-	-	-	-	-	-	-	-	-	-	-	-	-	-	-	-	-	-	+	-	-	-	-	-	-	-	-	+	-	+	-	-	-	-	-	-	-	-	-	-	-	-	-	-	-	+	+	-	+	-	-
2736	4185	merozoite surface protein 1	6	-	-	-	-	-	-	-	-	-	-	-	-	-	-	-	-	-	-	-	-	-	-	-	-	+	-	+	+	+	+	-	-	-	-	-	-	-	-	-	-	-	-	+	-	-	-	-	-	-	-	-	-	-	-	-	-	-
2737	4186	protein with prophage function domain	6	-	-	-	-	-	-	-	-	-	-	-	-	-	-	-	-	-	-	-	-	-	-	-	-	+	-	+	+	+	+	-	-	-	-	-	-	-	-	-	-	-	-	+	-	-	-	-	-	-	-	-	-	-	-	-	-	-
2738	4187	hypothetical protein	6	-	-	-	-	-	-	-	-	-	-	-	-	-	-	-	-	-	-	-	-	-	-	-	-	+	-	-	-	-	-	-	-	+	-	-	+	+	-	-	-	-	-	-	-	-	-	-	-	-	-	-	+	-	-	+	-	-
2739	4189	hypothetical protein	6	-	-	-	-	-	-	-	-	-	-	-	-	-	-	-	-	-	-	-	-	-	-	-	-	+	+	+	+	+	+	-	-	-	-	-	-	-	-	-	-	-	-	-	-	-	-	-	-	-	-	-	-	-	-	-	-	-
2740	4190	hypothetical protein	6	-	-	-	-	-	-	-	-	-	-	-	-	-	-	-	-	-	-	-	-	-	-	-	-	+	+	+	+	+	+	-	-	-	-	-	-	-	-	-	-	-	-	-	-	-	-	-	-	-	-	-	-	-	-	-	-	-
2741	4191	hypothetical protein	6	-	-	-	-	-	-	-	-	-	-	-	-	-	-	-	-	-	-	-	-	-	-	-	-	+	-	+	+	+	+	-	-	-	-	-	-	-	-	-	-	-	-	-	-	-	-	+	-	-	-	-	-	-	-	-	-	-
2742	4192	Inner membrane protein YqiK	6	-	-	-	-	-	-	-	-	-	-	-	-	-	-	-	-	-	-	-	-	+	+	-	-	+	-	+	+	-	-	-	-	-	-	-	-	-	-	-	-	-	-	-	-	-	-	-	-	-	-	-	-	-	-	-	+	-
2743	4193	hypothetical protein	6	-	-	-	-	-	-	-	-	-	-	-	-	-	-	-	-	-	-	-	-	-	-	-	-	+	-	+	+	+	+	-	-	-	-	-	-	-	-	-	-	-	-	+	-	-	-	-	-	-	-	-	-	-	-	-	-	-
2744	4194	hypothetical protein	6	-	-	-	-	-	-	-	-	-	-	-	-	-	-	+	-	-	-	-	-	-	-	-	-	+	-	-	-	-	-	-	-	-	-	+	+	+	-	-	-	-	-	-	-	-	-	-	-	-	-	-	-	-	-	-	-	+
2745	4196	Xylose isomerase (EC 5.3.1.5)	6	-	-	-	-	-	-	-	-	+	-	-	-	-	-	-	-	-	-	-	-	-	-	-	-	-	-	-	-	-	-	-	-	-	-	-	+	+	-	+	+	-	-	-	-	-	-	-	-	-	-	-	-	-	+	-	-	-
2746	4200	Phage-related protein	6	-	-	-	-	-	-	-	-	-	-	-	-	-	-	-	-	-	-	-	-	-	-	-	-	-	-	-	-	-	-	-	-	-	-	-	+	+	-	+	+	-	-	-	-	-	-	+	-	-	-	-	+	-	-	-	-	-
2747	4201	Xylulose kinase (EC 2.7.1.17)	6	-	-	-	-	-	-	-	-	+	-	-	-	-	-	-	-	-	-	-	-	-	-	-	-	-	-	-	-	-	-	-	-	-	-	-	+	+	-	+	+	-	-	-	-	-	-	-	-	-	-	-	-	-	+	-	-	-
2748	4204	hypothetical protein	6	-	-	-	-	+	-	-	-	-	-	-	-	-	-	-	-	-	-	-	+	-	-	-	-	-	-	-	-	-	-	+	+	-	+	-	-	-	-	+	-	-	-	-	-	-	-	-	-	-	-	-	-	-	-	-	-	-
2749	4207	Alpha-xylosidase (EC 3.2.1.-)	6	-	-	-	-	-	-	-	-	+	-	-	-	-	-	-	-	-	-	-	-	-	-	-	-	-	-	-	-	-	-	-	-	-	-	-	+	+	-	+	+	-	-	-	-	-	-	-	-	-	-	-	-	-	+	-	-	-
2750	4209	Alpha-xylosidase (EC 3.2.1.-)	6	-	-	-	-	-	-	-	-	+	-	-	-	-	-	-	-	-	-	-	-	-	-	-	-	-	-	-	-	-	-	-	-	-	-	-	+	+	-	+	+	-	-	-	-	-	-	-	-	-	-	-	-	-	+	-	-	-
2751	4210	PTS system, mannose-specific IIA component (EC 2.7.1.69)	6	-	-	-	-	-	-	-	-	+	-	-	-	-	-	-	-	-	-	-	-	-	-	-	-	-	-	-	-	-	-	-	-	-	-	-	+	+	-	+	+	-	-	-	-	-	-	-	-	-	-	-	-	-	+	-	-	-
2752	4216	xylose repressor, putative	6	-	-	-	-	-	-	-	-	+	-	-	-	-	-	-	-	-	-	-	-	-	-	-	-	-	-	-	-	-	-	-	-	-	-	-	+	+	-	+	+	-	-	-	-	-	-	-	-	-	-	-	-	-	+	-	-	-
2753	4218	hypothetical protein	6	-	-	-	-	-	-	-	-	-	-	-	-	-	-	+	-	-	-	-	-	-	-	-	-	-	+	-	-	-	-	-	-	-	-	-	-	-	-	-	+	-	-	+	-	+	-	-	-	+	-	-	-	-	-	-	-	-
2754	4223	Cell wall surface anchor family protein	6	-	-	-	-	-	-	-	-	-	-	-	-	-	-	-	-	-	-	-	-	-	-	-	-	-	-	-	-	-	-	-	-	-	-	-	-	-	-	-	-	-	-	-	-	+	+	+	+	-	-	-	-	-	-	-	+	+
2755	4224	Ribonucleases G and E	6	-	-	-	-	-	-	-	-	-	-	-	-	-	-	-	-	-	-	-	-	+	-	+	+	-	-	-	+	+	-	-	-	-	-	-	-	-	-	-	-	-	+	-	-	-	-	-	-	-	-	-	-	-	-	-	-	-
2756	4225	hypothetical protein	6	-	-	-	-	-	-	-	-	-	-	-	-	-	-	-	-	-	-	-	+	+	+	+	+	-	-	-	-	-	-	-	-	-	-	-	-	+	-	-	-	-	-	-	-	-	-	-	-	-	-	-	-	-	-	-	-	-
2757	4230	hypothetical protein	6	+	+	+	-	-	-	-	-	-	+	+	-	-	-	-	+	-	-	-	-	-	-	-	-	-	-	-	-	-	-	-	-	-	-	-	-	-	-	-	-	-	-	-	-	-	-	-	-	-	-	-	-	-	-	-	-	-
2758	4236	hypothetical protein	6	-	-	-	-	-	-	-	-	-	-	-	-	-	-	-	-	-	-	-	-	-	-	-	-	-	-	-	-	-	-	-	-	-	-	-	-	-	-	-	-	+	+	+	+	+	-	-	-	-	-	-	-	-	-	-	+	-
2759	4247	hypothetical protein	6	-	-	-	-	-	-	-	-	-	-	-	-	-	-	-	-	-	-	-	+	-	-	-	-	-	+	+	-	-	-	-	-	-	-	-	+	+	-	-	-	-	-	-	-	-	-	-	+	-	-	-	-	-	-	-	-	-
2760	4248	SNF2 family protein	6	-	-	-	-	-	-	-	-	-	-	-	-	-	-	-	-	-	-	-	-	-	-	-	-	-	-	-	-	-	+	+	+	+	-	-	-	-	-	-	-	-	-	-	-	-	-	-	+	-	-	-	+	-	-	-	-	-
2761	4252	hypothetical protein	6	-	-	-	-	-	-	-	-	-	-	-	-	-	-	-	-	-	-	-	-	-	-	-	-	-	-	-	-	-	-	-	-	-	-	-	-	-	-	-	-	-	-	+	+	-	-	+	+	+	+	-	-	-	-	-	-	-
2762	4259	hypothetical protein	6	-	-	-	-	-	-	-	-	-	-	-	-	-	-	-	-	-	-	-	-	-	-	-	-	-	-	+	+	+	-	+	+	-	-	-	-	-	-	-	-	+	-	-	-	-	-	-	-	-	-	-	-	-	-	-	-	-
2763	4261	phage protein	6	-	-	-	-	-	-	-	-	-	-	-	-	-	-	+	-	-	-	+	-	-	-	-	-	-	-	-	-	-	-	-	-	-	-	-	-	-	-	-	-	+	-	+	+	-	-	-	-	-	-	-	-	-	-	+	-	-
2764	4266	DNA-damage-inducible protein D	6	-	-	-	-	-	-	-	-	-	-	-	-	-	-	-	-	-	-	-	-	-	-	-	-	-	-	-	-	-	-	-	-	-	-	-	+	+	+	-	-	-	-	-	-	-	-	-	-	-	+	-	-	-	+	+	-	-
2765	4274	hypothetical protein	6	-	-	-	-	-	-	-	-	-	-	-	-	-	-	-	-	-	-	-	-	-	-	-	-	-	-	-	-	-	-	-	-	-	-	-	+	+	-	-	-	-	-	-	-	-	-	-	-	-	+	+	-	-	+	+	-	-
2766	4275	ABC-type uncharacterized transport system	6	-	-	-	-	-	-	-	-	-	-	-	-	-	-	-	-	-	-	-	-	-	-	-	-	-	-	-	-	-	-	-	-	-	-	-	+	+	+	-	-	-	-	-	-	-	-	-	-	-	+	-	-	-	+	+	-	-
2767	4276	hypothetical protein	6	-	-	-	-	-	-	-	-	-	-	-	-	-	-	-	-	-	-	-	-	-	-	-	-	-	-	-	-	-	-	-	-	-	-	+	+	+	-	-	-	-	-	-	-	-	-	-	-	-	+	-	-	-	+	+	-	-
2768	4278	Daunorubicin resistance transmembrane protein	6	-	-	-	-	-	-	-	-	-	-	-	-	-	-	-	-	-	-	-	-	-	-	-	-	-	-	-	-	-	-	-	-	-	-	-	+	+	+	-	-	-	-	-	-	-	-	-	-	-	+	-	-	-	+	+	-	-
2769	4280	Type I restriction-modification system	6	-	-	-	-	-	-	+	+	-	-	-	-	+	+	-	-	-	-	-	-	-	-	-	-	-	-	-	-	-	-	-	-	-	+	+	-	-	-	-	-	-	-	-	-	-	-	-	-	-	-	-	-	-	-	-	-	-
2770	4282	hypothetical protein	6	-	-	-	-	-	-	-	-	-	-	-	-	-	-	+	-	-	-	-	-	-	-	-	-	-	-	-	-	-	-	-	-	-	-	-	-	+	-	-	-	-	-	+	-	+	-	-	-	-	-	+	-	-	-	+	-	-
2771	4283	conserved domain protein	6	-	-	-	-	-	-	-	-	+	-	-	-	-	-	-	-	-	-	-	-	-	-	-	-	-	-	-	-	-	-	-	-	-	-	-	-	-	-	-	-	-	-	-	-	+	-	-	-	+	-	+	-	+	+	-	-	-
2772	4291	hypothetical protein	6	-	-	-	-	-	-	-	-	-	-	-	-	-	-	-	-	-	-	-	-	-	-	-	-	-	-	-	-	-	-	-	-	+	+	+	+	+	+	-	-	-	-	-	-	-	-	-	-	-	-	-	-	-	-	-	-	-
2773	4292	hypothetical protein	6	-	-	-	-	-	-	-	-	-	-	-	-	-	-	-	-	-	-	-	-	-	-	-	-	-	-	-	-	-	-	-	-	+	+	+	+	+	+	-	-	-	-	-	-	-	-	-	-	-	-	-	-	-	-	-	-	-
2774	4293	hypothetical protein	6	-	-	-	-	-	-	-	-	-	-	-	-	-	-	-	-	-	-	-	-	-	-	-	-	-	-	+	-	-	-	-	-	+	+	+	+	-	+	-	-	-	-	-	-	-	-	-	-	-	-	-	-	-	-	-	-	-
2775	4294	hypothetical protein	6	-	-	-	-	-	-	-	-	-	-	-	-	-	-	-	-	-	-	-	-	-	-	-	-	-	-	-	-	-	-	-	-	-	+	-	+	+	-	-	-	-	-	-	-	-	-	-	-	-	-	-	+	+	-	+	-	-
2776	4297	hypothetical protein	6	-	-	-	-	-	-	-	-	-	-	-	-	-	-	-	-	-	-	-	-	-	-	-	-	-	-	-	-	-	-	-	-	+	+	+	+	+	+	-	-	-	-	-	-	-	-	-	-	-	-	-	-	-	-	-	-	-
2777	4299	type II restriction endonuclease	6	-	-	-	-	-	-	-	-	-	-	-	-	-	-	-	-	-	-	-	-	-	-	-	-	-	-	-	-	-	-	-	-	+	+	+	+	+	+	-	-	-	-	-	-	-	-	-	-	-	-	-	-	-	-	-	-	-
2778	4300	hypothetical protein	6	-	-	-	-	-	-	-	-	-	-	-	-	-	-	-	-	-	-	-	-	-	-	-	-	-	-	-	-	-	-	-	-	+	+	+	+	+	+	-	-	-	-	-	-	-	-	-	-	-	-	-	-	-	-	-	-	-
2779	4301	Glycosyltransferase involved in cell wall biogenesis	6	-	-	-	-	-	-	-	-	-	-	-	-	-	-	-	-	-	-	-	-	-	-	-	-	-	-	-	-	-	-	-	-	-	-	-	+	+	-	-	-	-	-	-	-	-	-	-	-	-	-	+	-	+	+	+	-	-
2780	4302	exopolysaccharide biosynthesis protein	6	-	-	-	-	-	-	-	-	-	-	-	-	-	-	-	-	-	-	-	-	-	-	-	-	-	-	-	-	-	-	-	-	-	-	-	+	+	+	-	-	-	-	-	-	-	-	-	-	-	-	+	-	-	+	+	-	-
2781	4303	hypothetical protein	6	-	-	-	-	-	-	-	-	-	-	-	-	-	-	-	-	-	-	-	-	+	+	-	-	-	-	-	-	-	-	-	-	+	+	+	+	-	-	-	-	-	-	-	-	-	-	-	-	-	-	-	-	-	-	-	-	-
2782	4304	hypothetical protein	6	-	-	-	-	-	-	-	-	-	-	-	-	-	-	-	-	-	-	-	-	-	-	-	-	-	-	-	-	-	-	-	-	+	+	+	+	+	+	-	-	-	-	-	-	-	-	-	-	-	-	-	-	-	-	-	-	-
2783	4305	Surface antigen	6	-	-	-	-	-	-	-	-	-	-	-	-	-	-	-	-	-	-	-	-	-	-	-	-	-	-	-	-	-	-	-	-	+	+	+	+	+	+	-	-	-	-	-	-	-	-	-	-	-	-	-	-	-	-	-	-	-
2784	4306	hypothetical protein	6	-	-	-	-	-	-	-	-	-	-	-	-	-	-	-	-	-	-	-	+	+	+	-	-	-	-	-	+	-	-	-	-	+	-	-	-	-	+	-	-	-	-	-	-	-	-	-	-	-	-	-	-	-	-	-	-	-
2785	4327	Type I restriction-modification system	6	-	-	-	-	-	-	-	-	-	-	-	-	-	-	-	-	-	-	-	-	-	-	-	-	-	-	+	-	-	-	-	-	+	+	-	-	-	-	-	-	-	-	-	-	-	-	-	-	-	-	+	-	-	+	+	-	-
2786	4345	hypothetical protein	6	-	-	-	-	+	-	-	-	-	-	-	-	-	-	-	-	-	-	-	+	-	-	-	-	-	-	-	-	-	-	+	+	-	+	-	-	-	-	-	-	-	-	-	-	-	-	-	-	-	-	+	-	-	-	-	-	-
2787	4348	Adenine phosphoribosyltransferase (EC 2.4.2.7)	6	+	+	+	-	-	-	-	-	-	-	-	-	-	-	-	-	-	-	-	-	-	-	-	-	-	-	-	-	-	-	-	-	-	-	-	-	-	-	-	-	-	-	-	+	-	-	-	-	+	-	-	-	+	-	-	-	-
2788	4380	Transcriptional regulator	6	-	-	-	-	-	-	-	-	-	-	-	-	-	-	-	-	-	-	-	-	-	-	-	-	-	-	+	+	+	+	+	+	-	-	-	-	-	-	-	-	-	-	-	-	-	-	-	-	-	-	-	-	-	-	-	-	-
2789	4381	hypothetical protein	6	-	-	-	-	-	-	-	-	-	-	-	-	-	-	-	-	-	-	-	-	-	-	-	-	-	-	+	+	+	+	+	+	-	-	-	-	-	-	-	-	-	-	-	-	-	-	-	-	-	-	-	-	-	-	-	-	-
2790	4382	PTS system	6	-	-	-	-	-	-	-	-	-	-	-	-	-	-	-	-	-	-	-	-	-	-	-	-	-	-	+	+	+	+	+	+	-	-	-	-	-	-	-	-	-	-	-	-	-	-	-	-	-	-	-	-	-	-	-	-	-
2791	4587	hypothetical protein	6	+	+	+	-	-	+	-	-	-	+	-	+	-	-	-	-	-	-	-	-	-	-	-	-	-	-	-	-	-	-	-	-	-	-	-	-	-	-	-	-	-	-	-	-	-	-	-	-	-	-	-	-	-	-	-	-	-
2792	2015	Ethanolamine utilization protein similar to PduA/PduJ	5	-	-	-	-	-	-	-	-	-	-	-	-	-	-	-	-	-	-	-	-	-	-	-	-	-	-	-	-	-	-	-	-	-	-	-	-	-	-	-	-	-	-	+	-	-	-	-	+	-	+	-	+	-	-	-	+	-
2793	2062	Malolactic enzyme (EC 1.-.-.-)	5	-	-	-	-	-	-	-	-	-	-	-	-	-	-	-	-	-	-	-	-	-	-	-	-	-	-	-	-	-	-	-	-	-	-	-	-	-	-	-	-	-	-	-	-	-	-	-	-	-	+	+	+	+	-	+	-	-
2794	2087	Integrase	5	-	-	-	-	-	-	-	-	-	-	-	-	-	-	-	-	-	-	-	-	-	-	-	-	-	-	-	-	-	-	-	-	-	-	-	-	-	-	-	-	+	-	+	-	-	-	-	-	-	+	-	+	+	-	-	-	-
2795	2247	hypothetical protein	5	-	-	-	-	-	-	-	-	-	-	-	-	-	-	-	-	-	-	-	-	-	-	-	-	-	-	-	-	-	-	-	-	-	-	-	-	-	-	-	-	-	-	-	+	-	-	+	-	+	+	-	-	-	-	-	-	+
2796	2346	Integrase/recombinase, putative, truncated	5	-	-	-	-	-	-	-	-	-	-	-	-	-	-	-	-	-	-	-	-	-	-	-	+	-	-	-	-	-	-	-	-	-	-	-	-	-	-	+	+	-	-	-	-	-	-	-	-	-	-	+	-	-	+	-	-	-
2797	2355	Dihydroxy-acid dehydratase (EC 4.2.1.9)	5	-	-	-	-	-	-	-	-	-	-	-	-	-	-	-	-	-	-	-	-	-	-	-	-	-	-	-	-	-	-	-	-	-	-	-	-	-	-	-	-	-	-	-	-	-	-	-	-	-	+	+	+	+	-	+	-	-
2798	2429	Paratox	5	-	-	-	-	-	-	-	-	-	-	-	-	-	-	+	-	+	-	-	-	-	-	-	-	-	-	-	-	-	-	-	-	+	-	-	-	-	-	-	-	-	-	-	+	-	-	+	-	-	-	-	-	-	-	-	-	-
2799	2560	predicted 4-deoxy-L-threo-5-hexosulose-uronate ketol-isomerase (EC 5.3.1.17)	5	-	-	-	-	-	-	-	-	-	-	-	-	-	-	-	-	-	-	-	-	-	-	-	-	-	-	-	-	-	-	-	-	-	-	-	-	-	-	-	-	-	-	-	+	-	-	-	-	-	+	+	-	+	-	+	-	-
2800	2650	Mobile element protein	5	+	+	+	-	-	-	-	-	-	-	+	-	-	-	-	-	-	-	-	-	-	-	-	-	-	-	-	-	-	-	-	-	-	-	-	-	-	-	-	-	-	-	-	-	-	-	-	-	-	+	-	-	-	-	-	-	-
2801	2651	Malate permease	5	-	-	-	-	-	-	-	-	-	-	-	-	-	-	-	-	-	-	-	-	-	-	-	-	-	-	-	-	-	-	-	-	-	-	-	-	-	-	-	-	-	-	-	-	-	-	-	-	-	+	+	+	+	-	+	-	-
2802	2652	hypothetical protein	5	+	+	+	-	-	-	-	-	-	-	+	-	-	-	-	-	-	-	-	-	-	-	-	-	-	-	-	-	-	-	-	-	-	-	-	-	-	-	-	-	-	-	-	-	-	-	-	-	-	+	-	-	-	-	-	-	-
2803	2654	putative transposon excisionase; Tn916 ORF1-like	5	+	+	+	-	-	-	-	-	-	-	+	-	-	-	-	-	-	-	-	-	-	-	-	-	-	-	-	-	-	-	-	-	-	-	-	-	-	-	-	-	-	-	-	-	-	-	-	-	-	+	-	-	-	-	-	-	-
2804	2655	Tn916, transcriptional regulator, putative	5	+	+	+	-	-	-	-	-	-	-	+	-	-	-	-	-	-	-	-	-	-	-	-	-	-	-	-	-	-	-	-	-	-	-	-	-	-	-	-	-	-	-	-	-	-	-	-	-	-	+	-	-	-	-	-	-	-
2805	2776	hypothetical protein	5	-	-	-	-	-	-	-	-	-	-	-	-	-	-	-	-	-	-	-	-	-	-	-	-	-	-	-	-	-	-	-	-	-	-	-	-	+	-	-	-	-	-	-	-	-	-	-	-	-	+	+	+	-	+	-	-	-
2806	2779	hypothetical protein	5	-	-	-	-	-	-	-	-	-	-	-	-	-	-	-	-	-	-	-	-	-	-	-	-	-	-	-	-	-	-	-	-	-	-	-	-	-	-	-	-	-	-	+	+	-	-	+	-	+	+	-	-	-	-	-	-	-
2807	2780	putative transcriptional regulator	5	-	-	-	-	-	-	-	-	-	-	-	-	-	-	-	-	-	-	-	-	-	-	-	-	-	-	-	-	-	-	-	-	-	-	-	-	-	-	-	-	-	-	-	-	-	-	-	-	-	+	+	+	+	-	+	-	-
2808	2878	Programmed cell death antitoxin MazE	5	-	-	-	-	-	-	-	-	-	-	-	-	-	-	-	-	-	-	+	-	-	-	-	-	-	-	-	-	-	-	-	-	-	-	-	-	-	-	+	-	-	-	-	-	-	-	-	-	-	+	+	-	+	-	-	-	-
2809	2900	Ethanolamine utilization protein similar to PduU	5	-	-	-	-	-	-	-	-	-	-	-	-	-	-	-	-	-	-	-	-	-	-	-	-	-	-	-	-	-	-	-	-	-	-	-	-	-	-	-	-	-	-	+	-	-	-	-	+	-	+	-	+	-	-	-	+	-
2810	2997	Programmed cell death toxin MazF	5	-	-	-	-	-	-	-	-	-	-	-	-	-	-	-	-	-	-	+	-	-	-	-	-	-	-	-	-	-	-	-	-	-	-	-	-	-	-	+	-	-	-	-	-	-	-	-	-	-	+	+	-	+	-	-	-	-
2811	3027	Ethanolamine utilization protein similar to PduL	5	-	-	-	-	-	-	-	-	-	-	-	-	-	-	-	-	-	-	-	-	-	-	-	-	-	-	-	-	-	-	-	-	-	-	-	-	-	-	-	-	-	-	+	-	-	-	-	+	-	+	-	+	-	-	-	+	-
2812	3040	hypothetical protein	5	-	-	-	-	-	-	-	-	+	-	-	-	-	-	-	-	-	-	-	-	-	-	-	-	-	-	-	-	-	-	-	-	-	-	-	-	-	-	-	-	-	-	-	-	+	-	-	-	+	-	+	-	-	+	-	-	-
2813	3041	prophage pi2 protein 37	5	-	-	-	-	-	-	-	-	+	-	-	-	-	-	-	-	-	-	-	-	-	-	-	-	-	-	-	-	-	-	-	-	-	-	-	-	-	-	-	-	-	-	-	-	+	-	-	-	+	-	+	-	-	+	-	-	-
2814	3042	DNA primase/helicase, phage-associated	5	-	-	-	-	-	-	-	-	+	-	-	-	-	-	-	-	-	-	-	-	-	-	-	-	-	-	-	-	-	-	-	-	-	-	-	-	-	-	-	-	-	-	-	-	+	-	-	-	+	-	+	-	-	+	-	-	-
2815	3043	prophage LambdaSa04, head-tail adaptor, putative	5	-	-	-	-	-	-	-	-	+	-	-	-	-	-	-	-	-	-	-	-	-	-	-	-	-	-	-	-	-	-	-	-	-	-	-	-	-	-	-	-	-	-	-	-	+	-	-	-	+	-	+	-	-	+	-	-	-
2816	3050	hypothetical protein	5	-	-	-	-	-	-	-	-	-	-	-	-	-	-	-	-	-	-	-	+	+	+	-	-	-	-	-	-	-	-	-	-	+	-	+	-	-	-	-	-	-	-	-	-	-	-	-	-	-	-	-	-	-	-	-	-	-
2817	3143	hypothetical protein	5	-	-	-	-	-	-	-	-	-	-	-	-	-	-	-	-	-	-	-	-	-	-	-	-	-	-	-	-	-	-	-	-	-	-	-	-	-	-	-	-	-	-	-	+	-	-	+	+	+	+	-	-	-	-	-	-	-
2818	3153	hypothetical protein	5	-	-	-	-	-	-	-	-	-	-	-	-	-	-	-	-	-	-	-	-	-	-	-	-	-	-	-	-	-	-	-	-	-	-	-	-	+	-	-	-	-	-	-	-	-	-	-	-	-	+	+	+	-	+	-	-	-
2819	3157	prophage pi2 protein 38	5	-	-	-	-	-	-	-	-	+	-	-	-	-	-	-	-	-	-	-	-	-	-	-	-	-	-	-	-	-	-	-	-	-	-	-	-	-	-	-	-	-	-	-	-	+	-	-	-	+	-	+	-	-	+	-	-	-
2820	3160	unknown phage protein	5	-	-	-	-	-	-	-	-	+	-	-	-	-	-	-	-	-	-	-	-	-	-	-	-	-	-	-	-	-	-	-	-	-	-	-	-	-	-	-	-	-	-	-	-	+	-	-	-	+	-	+	-	-	+	-	-	-
2821	3167	Lantibiotic streptococcin A-M49 precursor (Antibacterial peptide A-M49)	5	-	-	-	-	-	-	-	-	-	-	-	-	-	-	-	-	-	-	-	-	+	+	-	-	-	-	-	-	+	-	-	-	+	-	-	-	-	-	-	-	-	-	-	-	-	-	-	-	-	-	-	-	-	-	+	-	-
2822	3291	hypothetical protein	5	-	-	-	-	-	-	-	-	-	-	-	-	-	-	-	-	-	-	-	-	-	-	-	-	-	-	-	-	-	-	-	-	-	-	-	-	-	-	-	-	-	-	-	+	-	-	+	+	+	+	-	-	-	-	-	-	-
2823	3304	hypothetical protein	5	-	-	-	-	-	-	-	-	+	-	-	-	-	-	-	-	-	-	-	-	-	-	-	-	-	-	-	-	-	-	-	-	-	-	-	-	-	-	-	-	-	-	-	-	+	-	-	-	+	+	+	-	-	-	-	-	-
2824	3311	hypothetical protein	5	+	+	+	-	-	-	-	-	-	-	+	-	-	-	-	-	-	-	-	-	-	-	-	-	-	-	-	-	-	-	-	-	-	-	-	-	-	-	-	-	-	-	-	-	-	-	-	-	-	+	-	-	-	-	-	-	-
2825	3320	ImpB/MucB/SamB family protein	5	-	-	-	-	-	-	-	-	-	-	-	-	-	-	-	-	-	-	-	-	-	-	-	-	-	-	-	-	-	-	-	-	+	+	+	+	-	+	-	-	-	-	-	-	-	-	-	-	-	-	-	-	-	-	-	-	-
2826	3462	Tn5252, relaxase	5	-	-	-	-	-	-	-	-	-	-	-	-	-	-	-	-	-	-	-	-	-	-	-	-	-	-	-	-	-	-	-	-	-	-	-	-	-	-	-	-	-	-	-	+	-	-	+	+	+	+	-	-	-	-	-	-	-
2827	3471	hypothetical protein	5	-	-	-	-	-	-	-	-	-	-	-	-	-	-	-	-	-	-	+	-	-	-	-	-	-	-	-	-	-	-	-	-	-	-	-	-	-	-	-	-	+	-	-	-	-	-	-	-	-	-	-	+	-	+	+	-	-
2828	3474	hypothetical protein	5	-	-	-	-	-	-	-	-	-	-	-	-	-	-	+	-	-	-	+	-	-	-	-	-	-	-	-	-	-	-	-	-	-	-	-	-	-	-	-	-	+	-	+	+	-	-	-	-	-	-	-	-	-	-	-	-	-
2829	3478	Histidine kinase of the competence regulon ComD	5	-	-	-	-	-	-	-	-	-	-	-	-	-	-	-	-	-	-	-	-	-	-	-	-	-	-	-	-	-	-	-	-	-	-	-	-	-	-	-	-	-	-	-	-	-	-	-	-	-	+	+	-	+	+	+	-	-
2830	3487	DNA-cytosine methyltransferase	5	-	-	-	-	-	-	-	-	+	-	-	-	-	-	-	-	-	-	-	-	-	-	-	-	-	-	-	-	-	-	-	-	-	-	-	-	-	-	-	-	-	-	-	-	+	-	-	-	+	-	+	-	-	+	-	-	-
2831	3488	S-adenosylmethionine synthetase (EC 2.5.1.6)	5	-	-	-	-	-	-	-	-	+	-	-	-	-	-	-	-	-	-	-	-	-	-	-	-	-	-	-	-	-	-	-	-	-	-	-	-	-	-	-	-	-	-	-	-	+	-	-	-	+	-	+	-	-	+	-	-	-
2832	3633	Phage protein	5	-	-	-	-	-	-	-	-	-	-	-	-	-	-	-	-	-	-	-	-	-	-	-	-	+	-	-	-	-	-	-	-	-	+	-	-	-	-	+	-	-	-	-	-	-	-	-	-	-	+	-	-	-	-	+	-	-
2833	3635	terminase large subunit	5	-	-	-	-	-	-	-	-	-	-	-	-	-	-	-	-	-	-	-	-	-	-	-	-	+	-	-	-	-	-	-	-	-	+	-	-	-	-	+	-	-	-	-	-	-	-	-	-	-	+	-	-	-	-	+	-	-
2834	3637	Phage tail length tape-measure protein	5	-	-	-	-	-	-	-	-	-	-	-	-	-	-	-	-	-	-	-	-	-	-	-	-	+	-	-	-	-	-	-	-	-	+	-	-	-	-	+	-	-	-	-	-	-	-	-	-	-	+	-	-	-	-	+	-	-
2835	3642	Phage protein	5	-	-	-	-	-	-	-	-	-	-	-	-	-	-	-	-	-	-	-	-	-	-	-	-	+	-	-	-	-	-	-	-	-	+	-	-	-	-	+	-	-	-	-	-	-	-	-	-	-	+	-	-	-	-	+	-	-
2836	3646	Phage protein	5	-	-	-	-	-	-	-	-	-	-	-	-	-	-	-	-	-	-	-	-	-	-	-	-	+	-	-	-	-	-	-	-	-	+	-	-	-	-	+	-	-	-	-	-	-	-	-	-	-	+	-	-	-	-	+	-	-
2837	3667	hypothetical protein	5	-	-	-	-	-	-	-	-	-	-	-	-	-	-	-	-	-	-	-	-	-	-	-	-	-	-	-	-	-	-	-	-	-	-	-	-	-	+	-	-	-	-	-	-	-	-	-	-	-	+	+	+	+	-	-	-	-
2838	3675	putative transcriptional regulator, XRE family protein	5	-	-	-	-	-	-	-	-	-	-	-	-	-	-	-	-	-	-	-	-	-	-	-	-	-	-	-	-	-	-	-	-	+	+	+	-	-	-	-	-	-	-	-	-	-	-	-	+	-	-	-	+	-	-	-	-	-
2839	3676	hypothetical protein	5	-	-	-	-	-	-	-	-	-	-	-	-	-	-	-	-	-	-	-	-	-	-	-	-	-	-	-	-	-	-	-	-	-	-	-	-	-	-	-	-	-	-	+	-	-	-	+	+	+	-	-	+	-	-	-	-	-
2840	3695	NisR	5	+	+	+	-	-	-	-	-	-	-	+	-	-	-	-	-	-	-	-	-	-	-	-	-	-	-	-	-	-	-	-	-	-	-	-	-	-	-	-	-	-	-	-	-	-	-	-	-	-	+	-	-	-	-	-	-	-
2841	3699	ThiJ/PfpI family protein	5	-	-	-	-	-	-	-	-	-	-	-	-	-	-	-	-	-	-	-	-	-	-	-	-	-	-	-	-	-	-	-	-	-	-	-	-	-	-	-	-	-	-	-	-	-	-	-	-	-	+	+	+	+	+	-	-	-
2842	3700	similar to glutathione reductase	5	-	-	-	-	-	-	-	-	-	-	-	-	-	-	-	-	-	-	-	-	-	-	-	-	-	-	-	-	-	-	-	-	-	-	-	-	-	-	-	-	-	-	-	-	-	-	-	-	-	+	+	+	+	+	-	-	-
2843	3705	hypothetical protein	5	-	-	-	-	-	-	-	-	+	-	-	-	-	-	-	-	-	-	-	-	-	-	-	-	-	-	-	-	-	-	-	-	-	-	-	-	-	-	-	-	-	-	-	-	+	-	-	-	+	-	+	-	-	+	-	-	-
2844	3707	Phage protein	5	-	-	-	-	-	-	-	-	-	-	-	-	-	-	-	-	-	-	-	+	+	+	-	-	-	-	-	-	-	-	-	-	-	-	-	-	-	-	-	-	-	-	-	-	+	-	-	-	-	-	-	-	-	+	-	-	-
2845	3711	hypothetical protein	5	-	-	-	-	-	-	-	-	-	-	-	-	-	-	-	-	-	-	-	-	-	-	-	-	-	-	-	-	-	-	-	-	-	-	+	+	+	-	-	-	-	-	-	-	-	-	-	-	-	-	-	+	-	+	-	-	-
2846	3725	truncated transcriptional regulator	5	-	-	-	-	-	-	-	-	-	-	-	-	-	-	-	-	-	-	-	-	-	-	-	-	-	-	-	-	-	-	-	-	-	-	-	-	-	-	-	-	-	-	-	-	-	-	-	-	-	-	+	+	+	-	+	+	-
2847	3881	Phage major capsid protein	5	-	-	-	-	-	-	-	-	-	-	-	-	-	-	-	-	-	-	-	-	-	-	-	-	+	-	-	-	-	-	-	-	-	+	-	-	-	-	+	-	-	-	-	-	-	-	-	-	-	+	-	-	-	-	+	-	-
2848	3900	prophage Lp3 protein 1, integrase	5	-	-	-	-	-	-	-	-	-	-	-	-	-	-	-	-	-	-	+	-	-	-	-	-	-	-	-	-	-	-	-	-	-	-	-	-	-	-	+	-	-	-	-	-	-	-	+	-	-	-	+	-	+	-	-	-	-
2849	3905	putative transcriptional regulator	5	-	-	-	-	-	-	-	-	-	-	-	-	-	-	-	-	-	-	+	-	-	-	-	-	-	-	-	-	-	-	-	-	-	-	-	-	-	-	+	+	-	-	-	+	-	-	-	-	-	-	-	+	-	-	-	-	-
2850	3906	hypothetical protein	5	-	-	-	-	-	-	-	-	-	-	-	-	-	-	-	-	-	-	-	-	-	-	-	-	-	-	-	-	-	-	-	-	+	-	-	-	-	-	+	+	-	+	-	-	-	-	-	-	-	-	-	-	-	-	-	-	+
2851	3957	nisin biosynthesis protein NisB	5	-	-	-	-	-	-	-	-	+	-	-	-	-	-	-	-	-	-	-	-	-	-	-	-	-	-	-	-	-	-	-	-	-	-	-	-	-	-	-	-	-	+	-	-	-	-	-	-	-	+	-	+	+	-	-	-	-
2852	3966	Organic hydroperoxide resistance transcriptional regulator	5	-	-	-	-	-	-	-	-	-	-	-	-	-	-	-	-	-	-	-	-	-	-	-	-	-	-	-	-	-	-	-	-	-	-	-	-	-	-	-	-	-	-	-	-	-	-	-	-	-	+	+	+	+	+	-	-	-
2853	3975	UDP-N-acetylglucosamine 2-epimerase (EC 5.1.3.14)	5	-	-	-	-	-	-	-	-	-	-	-	-	-	-	-	-	-	-	-	-	-	-	-	-	-	-	-	-	-	-	-	-	-	-	-	+	-	-	-	-	-	-	+	+	-	-	-	-	-	-	-	-	-	+	+	-	-
2854	3976	Epimerase/dehydratase WbkD	5	-	-	-	-	-	-	-	-	-	-	-	-	-	-	-	-	-	-	-	-	-	-	-	-	-	-	-	-	-	-	-	-	-	-	-	+	-	-	-	-	-	-	+	+	-	-	-	-	-	-	-	-	-	+	+	-	-
2855	3980	Capsular polysaccharide synthesis enzyme Cap5F	5	-	-	-	-	-	-	-	-	-	-	-	-	-	-	-	-	-	-	-	-	-	-	-	-	-	-	-	-	-	-	-	-	-	-	-	+	-	-	-	-	-	-	+	+	-	-	-	-	-	-	-	-	-	+	+	-	-
2856	3994	ScnA1	5	-	-	-	-	-	-	-	-	-	-	-	-	-	-	-	-	-	-	-	-	+	+	-	-	-	-	-	-	+	-	-	-	+	-	-	-	-	-	-	-	-	-	-	-	-	-	-	-	-	-	-	-	-	-	+	-	-
2857	3999	Spectinomycin 9-O-adenylyltransferase	5	-	-	-	-	-	-	-	-	-	-	-	-	-	-	-	-	-	-	-	-	-	-	-	-	-	-	-	-	-	-	-	-	+	-	-	-	-	-	-	-	-	-	+	+	-	-	-	-	+	-	+	-	-	-	-	-	-
2858	4168	Phage protein	5	-	-	-	-	-	-	-	-	-	-	-	-	-	-	-	+	+	+	-	-	-	-	-	-	-	-	-	-	-	-	-	-	-	-	-	-	-	-	-	-	-	-	-	+	-	-	+	-	-	-	-	-	-	-	-	-	-
2859	4170	Dihydrofolate reductase (EC 1.5.1.3)	5	-	-	-	-	-	-	-	-	-	-	-	-	-	-	-	-	-	-	-	+	-	-	-	+	-	+	-	-	-	-	-	-	-	-	-	-	-	-	+	+	-	-	-	-	-	-	-	-	-	-	-	-	-	-	-	-	-
2860	4197	hypothetical protein	5	-	-	+	-	-	-	-	-	-	-	-	-	-	-	-	-	-	-	-	-	-	-	-	-	-	-	+	-	-	-	-	-	-	-	-	-	-	-	+	+	-	-	-	-	-	-	-	-	+	-	-	-	-	-	-	-	-
2861	4208	hypothetical protein	5	-	-	-	-	-	-	-	-	-	-	-	-	-	-	-	-	-	-	-	-	-	-	-	-	-	-	-	-	-	-	-	-	-	-	-	+	+	+	+	+	-	-	-	-	-	-	-	-	-	-	-	-	-	-	-	-	-
2862	4213	LPXTG-motif cell wall anchor domain protein	5	-	-	-	-	-	-	-	-	-	-	-	-	-	-	-	-	-	-	-	-	-	-	-	-	-	-	-	-	-	-	-	-	-	-	-	-	-	-	+	+	-	-	-	-	-	-	-	-	-	-	+	-	+	+	-	-	-
2863	4214	hypothetical protein	5	-	-	-	-	-	-	-	-	-	-	-	-	-	-	-	-	-	-	-	-	-	-	-	-	-	-	-	-	-	-	-	-	-	-	-	+	+	+	+	+	-	-	-	-	-	-	-	-	-	-	-	-	-	-	-	-	-
2864	4215	hypothetical protein	5	-	-	-	-	-	-	-	-	-	-	-	-	-	-	-	-	-	-	-	-	-	-	-	-	-	-	-	-	-	-	-	-	-	+	+	-	-	-	+	-	-	-	-	-	-	-	-	-	-	-	-	-	+	-	+	-	-
2865	4219	hypothetical protein	5	-	-	-	-	-	-	-	-	-	-	-	-	-	-	-	-	-	-	-	-	-	-	-	-	-	-	-	-	-	-	-	-	-	-	-	-	-	-	-	-	-	-	-	-	+	+	+	+	+	-	-	-	-	-	-	-	-
2866	4220	Mobile element protein	5	-	-	-	-	-	-	-	-	-	-	-	-	-	-	-	-	-	-	-	-	-	-	-	-	-	-	-	-	-	-	-	-	-	-	-	-	-	-	-	-	-	-	-	-	+	+	-	+	+	-	-	-	+	-	-	-	-
2867	4222	Type III restriction-modification system DNA endonuclease res (EC 3.1.21.5)	5	-	-	-	-	-	-	-	-	-	-	-	-	-	-	-	-	-	-	+	-	-	-	-	-	-	-	-	-	-	-	-	-	-	-	-	-	-	-	-	-	-	+	-	-	+	+	+	-	-	-	-	-	-	-	-	-	-
2868	4226	hypothetical protein	5	-	-	-	-	-	-	-	-	-	-	-	-	-	-	-	-	-	-	-	+	+	-	+	-	-	-	-	+	-	-	-	-	-	-	-	-	-	-	-	-	-	-	-	+	-	-	-	-	-	-	-	-	-	-	-	-	-
2869	4228	putative replication protein - phage associated	5	-	-	-	-	-	-	-	-	-	-	-	-	-	-	-	-	-	-	-	-	-	-	-	-	-	-	-	-	-	-	-	-	-	+	-	+	-	-	-	-	-	-	-	+	-	-	-	-	-	-	+	-	-	-	+	-	-
2870	4232	hypothetical protein	5	-	-	-	-	-	-	-	-	-	-	-	-	-	-	-	-	-	-	-	-	-	-	-	-	-	-	-	-	-	-	-	-	-	-	-	-	-	-	-	-	-	-	-	-	-	-	-	-	-	-	+	+	-	+	+	-	+
2871	4238	DNA topology modulation protein	5	-	-	-	-	-	-	-	-	-	-	-	-	-	-	-	-	-	-	-	-	-	-	-	-	-	-	-	-	-	-	-	-	-	-	-	-	-	-	-	-	-	-	-	-	-	-	-	-	-	+	+	+	-	-	-	+	+
2872	4241	hypothetical protein	5	-	-	-	-	-	-	-	-	-	-	-	-	-	-	-	-	-	-	-	-	-	-	-	-	-	-	-	-	-	-	-	-	-	-	-	-	-	-	-	-	-	-	+	+	-	-	-	+	-	+	-	-	-	-	-	+	-
2873	4253	Type I restriction-modification system, DNA-methyltransferase subunit M (EC 2.1.1.72)	5	-	-	-	-	-	-	-	-	-	-	-	-	-	-	-	-	-	-	-	-	-	-	-	-	-	-	-	-	-	-	-	-	-	-	-	-	-	-	-	-	-	-	+	-	-	-	+	+	-	+	-	+	-	-	-	-	-
2874	4254	hypothetical protein	5	-	-	-	-	-	-	-	-	-	-	-	-	-	-	-	-	-	-	-	-	-	-	-	-	-	-	-	-	-	-	-	-	-	-	-	-	-	-	-	-	-	-	-	+	-	-	-	+	+	+	-	-	-	-	-	+	-
2875	4264	copper amine oxidase-like	5	-	-	-	-	-	-	-	-	-	-	-	-	-	-	-	-	-	-	-	-	-	-	-	-	-	-	-	-	-	-	-	-	-	-	-	-	-	-	-	-	-	-	-	+	-	-	+	-	+	+	-	-	-	-	-	+	-
2876	4270	glycerophosphoryl diester phosphodiesterase	5	-	-	-	-	-	-	-	-	-	-	-	-	-	-	-	-	-	-	-	-	-	-	-	-	-	-	-	-	-	-	-	-	-	-	-	+	+	-	-	-	-	-	-	-	-	-	-	-	-	+	-	-	-	+	+	-	-
2877	4284	hypothetical protein	5	-	-	-	-	-	-	-	-	+	-	-	-	-	-	-	-	-	-	-	-	-	-	-	-	-	-	-	-	-	-	-	-	-	-	-	-	-	-	-	-	-	-	-	-	+	-	-	-	+	-	+	-	-	+	-	-	-
2878	4288	hypothetical protein	5	-	-	-	-	-	-	-	-	+	-	-	-	-	-	-	-	-	-	-	-	-	-	-	-	-	-	-	-	-	-	-	-	-	-	-	-	-	-	-	-	-	-	-	-	+	-	-	-	+	-	+	-	-	+	-	-	-
2879	4296	ORF49	5	-	-	-	-	-	-	-	-	-	-	-	-	-	-	-	-	-	-	+	-	-	-	-	-	-	-	-	-	-	-	-	-	-	-	-	+	+	+	-	-	-	-	-	-	-	-	-	-	-	-	-	-	-	+	-	-	-
2880	4298	Toll-Interleukin receptor	5	-	-	-	-	-	-	-	-	-	-	-	-	-	-	-	-	-	-	-	-	-	-	-	-	-	-	-	-	-	-	-	-	-	-	-	+	+	+	-	-	-	-	-	-	-	-	-	-	-	-	-	-	+	-	+	-	-
2881	4308	DNA helicase	5	-	-	-	-	-	-	-	-	-	-	-	-	-	-	-	-	-	-	-	-	-	-	-	-	-	-	-	-	-	-	-	-	-	-	-	-	-	+	-	-	-	-	-	-	-	-	-	-	-	-	+	-	+	+	+	-	-
2882	4310	hypothetical protein	5	-	-	-	-	-	-	-	-	-	-	-	-	-	-	-	-	-	-	+	+	+	+	-	-	-	-	-	-	-	-	-	-	-	-	-	-	+	-	-	-	-	-	-	-	-	-	-	-	-	-	-	-	-	-	-	-	-
2883	4324	Recombinational DNA repair protein RecT (prophage associated)	5	-	-	-	-	-	-	-	-	-	-	-	-	-	-	-	-	-	-	-	-	-	-	-	-	-	-	-	-	-	-	-	-	-	+	-	-	-	-	-	-	-	-	-	+	-	-	-	-	-	-	-	+	+	-	+	-	-
2884	4325	ABC transporter ATP-binding/membrane spanning protein - multidrug resistance	5	-	-	-	-	-	-	-	-	-	-	-	-	-	-	-	-	-	-	-	-	-	-	-	-	-	-	-	-	-	-	-	-	-	-	-	-	-	-	-	-	-	-	-	-	-	-	-	-	+	-	-	+	-	+	+	+	-
2885	4330	Transcriptional activator PlcR	5	-	-	-	-	-	-	-	-	-	-	-	-	-	-	-	-	-	-	-	-	-	-	-	-	-	-	-	-	-	-	-	-	-	-	-	-	-	-	-	-	-	-	-	-	-	-	-	-	+	-	-	+	-	+	+	+	-
2886	4347	some similarities to phage related proteins	5	-	-	-	-	+	-	-	-	-	-	-	-	-	-	-	-	-	-	-	-	-	-	-	-	-	-	-	-	-	-	+	+	-	+	-	-	-	-	-	-	-	-	-	-	-	-	-	-	-	-	+	-	-	-	-	-	-

Table S5. Precence/absence of each HG among 57 *S. suis* strains

				1																13			25					28						104		94			76			53/54		Divergent *S. suis*																
Index[a]	ClutserID	Gene products	Members	98HAH33	05ZYH33	SC84	A7	SS12	P1/7	DAT273	NIAH11434	JS14	GZ1	BM407	S735	13730	MNCM07	NCTC 10446	14636	NCTC 10237	ST1	2651	89-1591	MNCM04	MNCM25	8074	D9	4961	ST3	MNCM43	DAT292	DAT260	NIAH11435	MNCM21	MNCM50	89-2479	DAT234	DAT290	93A	42A	NT77	6407	11538	8830	12814	92-1400	89-590	2726	2524	89-3576-3	4417	D12	10581	89-5259	88-5299A	R61	92-1191	14A	92-4172	22083
2887	4593	sepS9	5	-	-	-	-	-	-	-	-	-	-	-	-	-	-	-	-	+	+	-	-	-	-	-	-	-	-	-	-	-	-	-	-	-	-	-	-	-	-	+	+	-	-	-	-	-	-	-	-	-	-	-	-	-	-	-	-	+
2888	4594	glycosyl transferase group 2 family protein(EC:2.4.1.-)	5	-	-	-	-	-	-	-	-	+	-	-	-	+	+	-	-	+	+	-	-	-	-	-	-	-	-	-	-	-	-	-	-	-	-	-	-	-	-	-	-	-	-	-	-	-	-	-	-	-	-	-	-	-	-	-	-	-
2889	4602	Fibronectin-binding protein	5	-	-	-	-	-	-	-	-	-	-	-	-	-	-	-	+	+	+	-	-	-	-	-	-	-	-	-	-	-	-	-	-	-	-	-	-	-	-	-	-	-	-	-	-	-	+	+	-	-	-	-	-	-	-	-	-	-
2890	4603	hypothetical protein	5	-	-	-	-	-	-	-	-	+	-	-	-	+	+	-	-	+	+	-	-	-	-	-	-	-	-	-	-	-	-	-	-	-	-	-	-	-	-	-	-	-	-	-	-	-	-	-	-	-	-	-	-	-	-	-	-	-
2891	4608	DNA modification methylase	5	-	-	-	-	-	-	-	-	-	-	-	-	-	-	-	+	+	+	-	-	-	-	-	-	-	-	-	-	-	-	-	-	-	-	-	-	-	-	-	-	-	-	-	+	-	-	-	-	-	-	+	-	-	-	-	-	-
2892	4609	hypothetical protein	5	-	-	-	-	-	-	-	-	-	-	-	-	-	-	-	-	+	-	-	-	-	-	-	-	-	-	-	-	-	-	-	-	-	-	-	-	-	-	-	-	-	-	-	-	-	-	+	-	-	-	-	+	-	-	-	-	-
2893	4610	transporter accessory protein, putative	5	-	-	-	-	-	-	-	-	-	-	-	-	-	-	-	-	+	+	+	-	-	-	-	-	-	-	-	-	-	-	-	-	-	-	-	-	-	-	+	+	-	-	-	-	-	-	-	-	-	-	-	-	-	-	-	-	-
2894	4611	Capsular polysaccharide repeating-unit polymerase CpsH	5	-	-	-	-	-	-	-	-	+	-	-	-	+	+	-	-	+	+	-	-	-	-	-	-	-	-	-	-	-	-	-	-	-	-	-	-	-	-	+	+	-	-	-	-	-	-	-	-	-	-	-	-	-	-	-	-	-
2895	4612	hypothetical protein	5	-	-	-	-	-	-	-	-	-	-	-	-	-	-	-	-	-	-	-	-	-	-	-	-	+	-	+	+	+	+	-	-	-	-	-	-	-	-	-	-	-	-	-	-	-	-	-	-	-	-	-	-	-	-	-	-	-
2896	4613	hypothetical protein	5	-	-	-	-	-	-	-	-	-	-	-	-	-	-	+	-	-	-	-	-	-	-	-	-	+	-	-	-	-	-	-	-	-	-	+	-	+	+	-	-	-	-	-	-	-	-	-	-	-	-	-	-	-	-	-	-	-
2897	4614	Phage minor capsid protein #Fam0018	5	-	-	-	-	-	-	-	-	-	-	-	-	-	-	-	-	-	-	-	-	-	-	-	-	+	-	-	-	-	-	-	-	-	+	-	-	-	-	+	-	-	-	-	-	-	-	-	-	-	+	-	-	-	-	+	-	-
2898	4615	Type I restriction-modification system, specificity subunit S (EC 3.1.21.3)	5	-	-	-	-	-	-	-	-	-	-	-	-	-	-	-	-	-	-	-	-	-	-	-	+	-	-	-	+	-	-	-	-	-	-	-	+	-	-	-	-	+	-	-	-	-	-	-	-	-	-	-	-	-	-	+	-	-
2899	4617	hypothetical protein	5	-	-	-	-	-	-	-	-	-	-	-	-	-	-	-	-	-	-	-	-	-	-	-	-	+	-	-	-	-	-	-	-	-	+	-	-	-	-	+	-	-	-	-	-	-	-	-	-	-	+	-	-	-	-	+	-	-
2900	4618	hypothetical protein	5	-	-	-	-	-	-	-	-	-	-	-	-	-	-	-	-	-	-	-	-	-	-	-	-	+	-	-	-	-	-	-	-	-	+	-	-	-	-	+	-	-	-	-	-	-	-	-	-	-	+	-	-	-	-	+	-	-
2901	4619	hypothetical protein	5	-	-	-	-	-	-	-	-	-	-	-	-	-	-	-	-	-	-	-	+	+	+	-	-	-	-	-	+	-	-	-	-	-	-	-	-	-	-	-	-	-	-	-	-	-	-	-	-	-	-	-	-	-	+	-	-	-
2902	4621	hypothetical protein	5	-	-	-	-	-	-	-	-	-	-	-	-	-	-	-	-	-	-	-	-	+	+	-	-	+	+	-	-	-	+	-	-	-	-	-	-	-	-	-	-	-	-	-	-	-	-	-	-	-	-	-	-	-	-	-	-	-
2903	4625	hypothetical protein	5	-	-	-	-	-	-	-	-	-	-	-	-	-	-	-	-	-	-	-	-	-	-	-	-	+	-	-	-	-	-	-	-	-	+	-	-	-	-	+	-	-	-	-	-	-	-	-	-	-	+	-	-	-	-	+	-	-
2904	4626	terminase small subunit	5	-	-	-	-	-	-	-	-	-	-	-	-	-	-	-	-	-	-	-	-	-	-	-	-	+	-	-	-	-	-	-	-	-	+	-	-	-	-	+	-	-	-	-	-	-	-	-	-	-	+	-	-	-	-	+	-	-
2905	4631	hypothetical protein	5	-	-	-	-	-	-	-	-	-	-	-	-	-	-	-	-	-	-	-	-	-	-	-	-	-	-	-	-	-	-	-	-	-	-	-	+	+	-	+	-	-	+	-	-	-	-	-	-	-	-	+	-	+	-	-	-	-
2906	4634	hypothetical protein	5	-	-	-	-	-	-	-	-	-	-	-	-	-	-	-	-	-	-	-	-	-	-	-	-	-	-	+	+	+	+	-	-	-	-	-	-	-	-	+	-	-	-	-	-	-	-	-	-	-	-	-	-	-	-	-	-	-
2907	4636	conserved domain protein	5	-	-	-	-	-	-	-	-	-	-	-	-	-	-	-	-	-	-	-	-	-	-	-	-	-	-	-	-	-	-	-	-	-	-	+	-	-	-	+	-	-	-	-	-	+	-	+	-	-	-	-	-	-	-	+	-	-
2908	4643	Putative Dihydrolipoamide dehydrogenase (EC 1.8.1.4); Mercuric ion reductase (EC 1.16.1.1); PF00070 family	5	-	-	-	-	-	-	-	-	-	-	-	-	-	-	-	-	-	-	-	-	-	-	-	-	-	-	-	+	+	-	-	-	-	-	-	-	-	-	+	+	-	-	+	-	-	-	-	-	-	-	-	-	-	-	-	-	-
2909	4648	hypothetical protein	5	-	-	-	-	-	-	-	-	-	-	-	-	-	-	-	-	-	-	+	-	-	-	-	-	-	-	-	-	-	-	-	-	-	-	-	-	-	-	+	-	-	-	-	-	-	-	-	-	-	-	+	-	-	+	-	+	-
2910	4652	1,3-propanediol dehydrogenase (EC 1.1.1.202)	5	-	-	-	-	-	-	-	-	-	-	-	-	-	-	-	-	-	-	-	-	-	-	-	-	-	-	-	-	-	-	-	-	-	-	-	-	-	-	-	-	-	-	-	-	+	+	+	+	+	-	-	-	-	-	-	-	-
2911	4653	Transcriptional regulator, MerR family	5	-	-	-	-	-	-	-	-	-	-	-	-	-	-	-	-	-	-	-	-	-	-	-	-	-	-	-	-	-	-	-	-	-	-	-	-	-	-	-	-	-	-	-	-	+	+	+	+	+	-	-	-	-	-	-	-	-
2912	4655	hypothetical protein	5	-	-	-	-	-	-	-	-	-	-	-	-	-	-	-	-	-	-	-	-	-	-	-	-	-	-	-	-	-	-	-	-	-	-	-	-	-	-	-	-	-	-	-	-	+	+	+	+	+	-	-	-	-	-	-	-	-
2913	4658	hypothetical protein	5	-	-	-	-	-	-	-	-	-	-	-	-	-	-	-	-	-	-	-	-	-	-	-	-	-	-	-	-	-	-	-	-	-	-	-	-	-	-	-	-	-	-	-	-	+	+	+	+	+	-	-	-	-	-	-	-	-
2914	4659	hypothetical protein	5	-	-	-	-	-	-	-	-	-	-	-	-	-	-	-	-	-	-	-	-	-	-	-	-	-	-	-	-	-	-	-	-	-	-	-	-	-	-	-	-	-	-	-	-	+	+	+	+	+	-	-	-	-	-	-	-	-
2915	4660	hypothetical protein	5	-	-	-	-	-	-	-	-	-	-	-	-	-	-	-	-	-	-	-	-	-	-	-	-	-	-	-	-	-	-	-	-	-	-	-	-	-	-	-	-	-	-	-	-	+	+	+	+	+	-	-	-	-	-	-	-	-
2916	4661	S-layer-like domain, putative	5	-	-	-	-	-	-	-	-	-	-	-	-	-	-	-	-	-	-	-	-	-	-	-	-	-	-	-	-	-	-	-	-	-	-	-	-	-	-	-	-	-	-	-	-	+	+	+	+	+	-	-	-	-	-	-	-	-
2917	4663	hypothetical protein	5	-	-	-	-	-	-	-	-	-	-	-	-	-	-	-	-	-	-	-	-	-	-	-	-	-	-	-	-	-	-	-	-	-	-	-	-	-	-	-	-	-	-	-	-	+	+	+	+	+	-	-	-	-	-	-	-	-
2918	4664	hypothetical protein	5	-	-	-	-	-	-	-	-	-	-	-	-	-	-	-	-	-	-	-	-	-	-	-	-	-	-	-	-	-	-	-	-	-	-	-	-	-	-	-	-	-	-	-	-	+	+	+	+	+	-	-	-	-	-	-	-	-
2919	4665	Type III restriction-modification system methylation subunit (EC 2.1.1.72)	5	-	-	-	-	-	-	-	-	-	-	-	-	-	-	-	-	-	-	+	-	-	-	-	-	-	-	-	-	-	-	-	-	-	-	-	-	-	-	-	-	-	+	-	-	+	+	+	-	-	-	-	-	-	-	-	-	-
2920	4666	hypothetical protein	5	-	-	-	-	-	-	-	-	-	-	-	-	-	-	-	-	-	-	-	+	+	+	+	-	-	-	-	+	-	-	-	-	-	-	-	-	-	-	-	-	-	-	-	-	-	-	-	-	-	-	-	-	-	-	-	-	-
2921	4667	hypothetical protein	5	-	-	-	-	-	-	-	-	-	-	-	-	-	-	-	+	-	-	-	+	-	-	+	+	-	-	-	-	-	-	-	-	-	-	-	-	-	-	-	-	-	-	-	-	-	-	-	-	-	-	-	-	-	-	-	+	-
2922	4669	hypothetical protein	5	-	-	-	-	-	-	-	-	-	-	-	-	-	-	-	-	-	-	-	+	-	-	+	+	-	+	-	+	-	-	-	-	-	-	-	-	-	-	-	-	-	-	-	-	-	-	-	-	-	-	-	-	-	-	-	-	-
2923	4670	hypothetical protein	5	-	-	-	-	-	-	-	-	-	-	-	-	-	-	-	-	-	-	-	+	+	+	+	+	-	-	-	-	-	-	-	-	-	-	-	-	-	-	-	-	-	-	-	-	-	-	-	-	-	-	-	-	-	-	-	-	-
2924	4671	putative transposase	5	-	-	-	-	-	-	-	-	-	-	-	-	-	-	-	-	-	-	-	-	+	+	+	-	-	-	-	-	-	-	-	-	-	-	-	+	-	-	-	-	-	+	-	-	-	-	-	-	-	-	-	-	-	-	-	-	-
2925	4676	hypothetical protein	5	-	-	-	-	-	-	-	-	-	-	+	-	-	-	-	+	-	-	-	-	-	-	-	-	-	-	-	-	+	+	-	-	-	-	-	-	-	-	-	-	-	-	-	-	-	-	-	-	-	-	-	-	-	-	-	-	-
2926	4680	hypothetical protein	5	-	-	-	-	-	-	-	-	-	-	-	-	-	-	-	-	-	-	-	-	-	-	-	-	-	-	-	-	-	-	-	-	-	-	-	-	-	-	-	-	+	-	-	-	+	-	-	-	-	+	-	-	-	-	-	+	+
2927	4693	hypothetical protein	5	-	-	-	-	-	-	-	-	-	-	-	-	-	-	-	-	-	-	-	-	-	-	-	-	-	-	-	-	-	-	-	-	-	-	-	-	-	-	-	-	-	-	-	-	+	-	-	+	+	-	-	+	-	-	-	+	-
2928	4700	hypothetical protein	5	-	-	-	-	-	-	-	-	-	-	-	-	-	-	-	-	-	-	+	-	-	-	-	-	-	-	-	-	-	-	-	-	-	-	-	-	-	-	-	-	+	-	+	-	+	-	-	+	-	-	-	-	-	-	-	-	-
2929	4708	MT-S_A70 family protein	5	-	-	-	-	-	-	-	-	-	-	-	-	-	-	-	-	-	-	-	-	-	-	-	-	-	-	-	-	-	-	-	-	-	-	-	+	+	-	-	-	-	+	-	-	-	-	-	-	-	-	+	-	+	-	-	-	-
2930	4717	Helix-turn-helix motif	5	-	-	-	-	-	-	-	-	-	-	-	-	-	-	-	-	-	-	-	+	+	+	-	-	-	-	-	-	-	-	-	-	-	-	-	-	-	-	+	-	-	-	-	-	-	-	+	-	-	-	-	-	-	-	-	-	-
2931	4721	hypothetical protein	5	-	-	-	-	-	-	-	-	-	-	-	-	-	-	+	-	-	-	+	-	-	-	-	-	-	-	-	-	-	-	-	-	-	-	-	-	-	-	+	-	+	-	-	-	-	-	-	-	-	-	-	-	-	-	+	-	-
2932	4729	hypothetical protein	5	-	-	-	-	-	-	-	-	-	-	-	-	-	-	-	-	-	-	-	-	-	-	-	-	-	-	-	-	-	-	-	-	-	-	-	-	-	-	-	-	-	-	-	-	-	+	+	-	+	+	+	-	-
2933	4732	hypothetical protein	5	-	-	-	-	-	-	-	-	-	-	-	-	-	-	-	-	-	-	-	-	-	-	-	-	-	-	-	-	-	-	-	-	-	-	-	-	-	-	-	-	-	-	-	-	-	+	+	+	+	+	-	-	-
2934	4733	hypothetical protein	5	-	-	-	-	-	-	-	-	-	-	-	-	-	-	-	-	-	-	-	-	-	-	-	-	-	-	-	-	-	-	-	-	-	+	-	-	-	-	-	-	-	-	-	-	-	+	+	+	-	-	+	-	-
2935	4736	ORF4	5	-	-	-	-	-	-	-	-	-	-	-	-	-	-	-	-	-	-	-	+	+	+	-	-	-	-	-	-	-	-	-	-	-	-	-	-	-	-	-	-	-	+	-	-	-	-	-	+	-	-	-	-	-	-	-
2936	4738	hypothetical protein	5	-	-	-	-	-	-	-	-	-	-	-	-	-	-	-	-	-	-	-	-	-	-	-	-	-	-	-	-	-	-	+	-	-	-	-	-	-	-	-	-	+	-	-	-	-	+	-	-	-	+	+	-	-
2937	4742	LPXTG-motif cell wall anchor domain protein	5	-	-	-	-	-	-	-	-	-	-	-	-	-	-	-	-	-	-	-	-	-	-	-	-	-	-	-	-	-	-	-	-	-	-	-	-	-	-	-	-	-	-	-	-	-	+	+	+	+	-	+	-	-
2938	4743	membrane protein, putative	5	-	-	-	-	-	-	-	-	-	-	-	-	-	-	-	-	-	-	-	-	-	-	-	-	-	-	-	-	-	-	-	-	-	-	-	-	-	-	-	-	-	-	-	-	-	+	+	+	+	-	+	-	-
2939	4744	Positive transcriptional regulator, MutR family	5	-	-	-	-	-	-	-	-	-	-	-	-	-	-	-	-	-	-	-	-	-	-	-	-	-	-	-	-	-	-	-	-	-	-	-	-	-	-	-	-	-	-	-	-	-	+	+	+	+	-	+	-	-
2940	4747	Alpha/beta hydrolase fold (EC 3.8.1.5)	5	-	-	-	-	-	-	-	-	-	-	-	-	-	-	-	-	-	-	-	-	-	-	-	-	-	-	-	-	-	-	-	-	-	-	-	-	-	-	-	-	-	-	-	-	-	+	+	+	+	-	+	-	-
2941	4750	hypothetical protein	5	-	-	-	-	-	-	-	-	-	-	-	-	-	-	-	-	-	-	-	-	-	-	-	-	-	-	-	-	-	-	-	-	-	-	-	-	-	-	-	-	-	-	-	-	-	+	+	+	+	+	-	-	-
2942	4751	ABC transporter membrane-spanning permease - Na+ export	5	-	-	-	-	-	-	-	-	-	-	-	-	-	-	-	-	-	-	-	-	-	-	-	-	-	-	-	-	-	-	-	-	-	-	-	-	-	-	-	-	-	-	-	-	-	+	+	+	+	-	+	-	-
2943	4752	hypothetical protein	5	-	-	-	-	-	-	-	-	-	-	-	-	-	-	-	-	-	-	-	-	-	-	-	-	-	-	-	-	-	-	-	-	-	-	-	-	-	-	+	-	+	-	-	-	+	+	-	-	-	+	-	-	-
2944	4753	ABC transporter permease protein	5	-	-	-	-	-	-	-	-	-	-	-	-	-	-	-	-	-	-	-	-	-	-	-	-	-	-	-	-	-	-	-	-	-	-	-	-	-	-	-	-	-	-	-	-	-	+	+	+	+	+	-	-	-
2945	4759	hypothetical protein	5	-	-	-	-	-	-	-	-	-	-	-	-	-	-	-	-	-	-	-	-	-	-	-	-	-	-	-	-	-	-	-	+	+	+	-	-	-	-	-	-	-	-	-	-	-	+	-	-	-	+	-	-	-
2946	4761	hypothetical protein	5	-	-	-	-	-	-	-	-	-	+	+	-	+	+	-	-	-	-	-	-	-	-	-	-	-	-	-	-	-	-	-	-	-	-	-	-	-	-	-	-	-	-	-	-	-	-	-	+	-	-	-	-	-
2947	4762	hypothetical protein	5	-	-	-	-	-	-	-	-	-	-	-	-	-	-	+	-	-	-	-	-	-	-	-	-	-	-	-	-	-	-	-	-	-	-	-	+	+	-	-	-	-	-	+	-	-	-	-	+	-	-	-	-	-	-	-	-	-
2948	4770	hypothetical protein	5	-	-	-	-	-	-	-	-	-	-	-	-	-	-	-	-	-	-	-	+	+	+	-	-	-	-	-	-	-	-	-	-	-	-	-	-	-	-	-	-	-	-	-	-	+	-	-	-	-	-	-	-	+	-	-	-	-
2949	4771	Glycosyltransferases involved in cell wall biogenesis	5	-	-	-	-	-	-	-	-	-	-	-	-	-	-	-	-	-	-	-	-	-	-	-	-	-	-	-	-	-	-	-	-	+	-	+	+	+	-	-	-	-	-	+	-	-	-	-	-	-	-	-	-	-	-	-	-	-
2950	4772	PTS system	5	-	-	-	-	-	-	-	-	-	-	-	-	-	-	-	-	-	-	-	-	-	-	-	-	-	-	-	-	-	-	-	-	+	+	+	+	-	+	-	-	-	-	-	-	-	-	-	-	-	-	-	-	-	-	-	-	-
2951	4773	glycosyltransferase family 2 protein	5	-	-	-	-	-	-	-	-	-	-	-	-	-	-	-	-	-	-	-	-	-	-	-	-	-	-	-	-	-	-	-	-	-	-	-	+	+	+	-	-	-	-	-	-	-	-	-	-	-	-	-	-	-	+	+	-	-
2952	4774	hypothetical protein	5	-	-	-	-	-	-	-	-	-	-	-	-	-	-	-	-	-	-	-	-	-	-	-	-	-	-	-	-	-	-	-	-	+	+	+	+	+	-	-	-	-	-	-	-	-	-	-	-	-	-	-	-	-	-	-	-	-
2953	4775	hypothetical protein	5	-	-	-	-	-	-	-	-	-	-	-	-	-	-	-	-	-	-	-	-	-	-	-	-	-	-	-	-	-	-	-	-	+	+	+	+	-	+	-	-	-	-	-	-	-	-	-	-	-	-	-	-	-	-	-	-	-
2954	4776	ThiJ/PfpI family protein	5	-	-	-	-	-	-	-	-	-	-	-	-	-	-	-	-	-	-	-	-	-	-	-	-	-	-	-	-	-	-	-	-	+	+	+	+	-	+	-	-	-	-	-	-	-	-	-	-	-	-	-	-	-	-	-	-	-
2955	4778	IgA1 protease (EC 3.4.24.13)	5	-	-	-	-	-	-	-	-	-	-	-	-	-	-	-	-	-	-	-	-	-	-	-	-	-	-	-	-	-	-	-	-	-	-	-	+	+	+	-	-	-	-	-	-	-	-	-	-	-	-	-	-	-	+	+	-	-
2956	4779	Beta-1	5	-	-	-	-	-	-	-	-	-	-	-	-	-	-	-	-	-	-	-	-	-	-	-	-	-	-	-	-	-	-	-	-	-	-	-	+	+	+	-	-	-	-	-	-	-	-	-	-	-	-	-	-	-	+	+	-	-
2957	4783	hypothetical protein	5	-	-	-	-	-	-	-	-	-	-	-	-	-	-	-	-	-	-	-	-	-	-	-	-	-	-	-	-	-	-	-	-	-	+	+	+	+	+	-	-	-	-	-	-	-	-	-	-	-	-	-	-	-	-	-	-	-
2958	4784	hypothetical protein	5	-	-	-	-	-	-	-	-	-	-	-	-	-	-	-	-	-	-	-	-	-	-	-	-	-	-	-	-	-	-	-	-	+	+	+	+	+	+	-	-	-	-	-	-	-	-	-	-	-	-	-	-	-	-	-	-	-
2959	4785	DNA methylase	5	-	-	-	-	-	-	-	-	-	-	-	-	-	-	-	-	-	-	-	-	-	-	-	-	-	-	-	-	-	-	-	-	+	+	+	+	-	+	-	-	-	-	-	-	-	-	-	-	-	-	-	-	-	-	-	-	-
2960	4786	Helix-turn-helix motif	5	-	-	-	-	-	-	-	-	-	-	-	-	-	-	-	-	-	-	-	-	-	+	+	-	-	-	-	-	-	-	-	-	-	-	-	+	+	-	-	-	-	-	-	-	-	-	-	+	-	-	-	-	-	-	-	-	-
2961	4787	hypothetical protein	5	-	-	-	-	-	-	-	-	-	-	-	-	-	-	-	-	-	-	-	-	-	-	-	-	-	-	-	-	-	-	-	-	-	-	-	+	+	+	-	-	-	-	-	-	-	-	-	-	-	-	-	-	-	+	+	-	-
2962	4789	hypothetical protein	5	-	-	-	-	-	-	-	-	-	-	-	-	-	-	-	-	-	-	-	-	-	-	-	-	-	-	-	-	-	-	-	-	-	-	-	-	-	+	-	-	-	-	-	-	-	-	-	-	-	-	-	+	+	+	+	-	-
2963	4791	hypothetical protein	5	-	-	-	-	-	-	-	-	-	-	-	-	-	-	-	-	-	-	-	-	-	-	-	-	-	-	-	-	-	-	-	-	-	-	-	-	+	+	-	-	-	-	-	-	-	-	-	-	-	-	+	-	+	+	-	-	-
2964	4792	hypothetical protein	5	-	-	-	-	-	-	-	-	-	-	-	-	-	-	-	-	-	-	-	-	-	-	-	-	-	-	-	-	-	-	+	+	-	-	+	-	+	+	-	-	-	-	-	-	-	-	-	-	-	-	-	-	-	-	-	-	-
2965	4803	maltose O-acetyltransferase(EC:2.3.1.79)	5	-	-	-	-	-	-	-	-	-	-	-	-	-	-	-	-	-	-	-	-	-	-	-	-	-	-	-	-	-	-	-	-	-	-	-	-	-	-	-	-	-	-	-	-	-	-	-	-	-	-	+	+	+	-	+	+	-
2966	4804	hypothetical protein	5	-	-	-	-	-	-	-	-	-	-	-	-	-	-	-	-	-	-	-	+	+	+	-	-	-	-	-	+	-	-	-	-	-	-	-	-	-	-	-	-	-	-	-	-	-	-	-	-	-	-	-	-	-	-	+	-	-
2967	4814	Hydrolase (HAD superfamily)	5	-	-	-	-	-	-	-	-	-	-	-	-	-	-	-	-	-	-	-	-	-	-	-	-	-	-	-	+	+	-	-	-	-	+	+	+	-	-	-	-	-	-	-	-	-	-	-	-	-	-	-	-	-	-	-	-	-
2968	4815	Fructose-1-phosphate kinase and related fructose-6-phosphate kinase (PfkB)	5	-	-	-	-	-	-	-	-	-	-	-	-	-	-	-	-	-	-	-	-	-	-	-	-	-	-	-	+	+	-	-	-	-	+	+	+	-	-	-	-	-	-	-	-	-	-	-	-	-	-	-	-	-	-	-	-	-
2969	4816	hypothetical protein	5	-	-	-	-	-	-	-	-	-	-	-	-	-	-	-	-	-	-	-	-	-	-	-	-	-	-	-	+	+	-	-	-	-	+	+	+	-	-	-	-	-	-	-	-	-	-	-	-	-	-	-	-	-	-	-	-	-
2970	4825	hypothetical protein	5	-	-	-	-	-	-	-	-	+	-	-	-	-	-	-	-	-	-	-	-	-	-	-	-	-	-	-	-	-	-	-	-	-	-	-	-	-	-	-	-	-	-	-	-	+	-	-	-	+	-	+	-	-	+	-	-	-
2971	4878	hypothetical protein	5	-	-	-	-	+	-	-	-	-	-	-	-	-	-	-	-	-	-	-	+	-	-	-	-	-	-	-	-	-	-	+	+	-	+	-	-	-	-	-	-	-	-	-	-	-	-	-	-	-	-	-	-	-	-	-	-	-
2972	4880	hypothetical protein	5	-	-	-	-	+	-	-	-	-	-	-	-	-	-	-	-	-	-	-	+	-	-	-	-	-	-	-	-	-	-	+	+	-	+	-	-	-	-	-	-	-	-	-	-	-	-	-	-	-	-	-	-	-	-	-	-	-
2973	4883	hypothetical protein	5	-	-	-	-	-	-	-	-	-	-	-	-	-	-	-	-	-	-	-	+	+	+	-	+	-	-	-	-	-	+	-	-	-	-	-	-	-	-	-	-	-	-	-	-	-	-	-	-	-	-	-	-	-	-	-	-	-
2974	4884	hypothetical protein	5	+	+	-	-	-	+	-	+	-	-	-	-	-	+	-	-	-	-	-	-	-	-	-	-	-	-	-	-	-	-	-	-	-	-	-	-	-	-	-	-	-	-	-	-	-	-	-	-	-	-	-	-	-	-	-	-	-
2975	2041	Magnesium and cobalt transport protein CorA	4	-	-	-	-	-	-	-	-	-	-	-	-	-	-	-	+	+	+	-	-	-	-	-	-	-	-	-	-	-	-	-	-	-	-	-	-	-	-	-	-	-	-	-	-	-	+	-	-	-	-	-	-	-	-	-	-	-
2976	2059	putative oxidoreductase	4	-	-	-	-	-	-	-	-	-	-	-	-	-	-	-	-	-	-	-	-	-	-	-	-	-	-	-	-	-	-	-	-	-	-	-	-	-	-	-	-	-	-	-	-	-	-	+	+	-	+	-	-	+
2977	2073	Streptococcal extracellular nuclease 3; Mitogenic factor 3	4	-	-	-	-	-	-	-	-	-	-	-	-	-	-	-	+	+	+	+	-	-	-	-	-	-	-	-	-	-	-	-	-	-	-	-	-	-	-	-	-	-	-	-	-	-	-	-	-	-	-	-	-	-	-	-	-	-
2978	2309	ABC transporter, ATP-binding protein	4	-	-	-	-	-	-	-	-	-	-	-	-	-	-	-	-	-	-	-	-	-	-	-	-	-	-	-	-	-	-	-	-	-	-	-	-	-	-	-	-	-	-	-	-	-	-	+	+	+	-	+	-	-
2979	2409	PTS system, mannitol-specific IIB component (EC 2.7.1.69) / PTS system, mannitol-specific IIC component (EC 2.7.1.69)	4	-	-	-	-	-	-	-	-	-	-	-	-	-	-	-	-	-	-	-	-	-	-	-	-	-	-	-	-	-	-	-	-	-	-	-	+	+	-	-	-	-	-	-	-	-	-	-	-	-	-	-	-	-	+	+	-	-
2980	2445	Transcriptional regulator, LacI family	4	-	-	-	-	-	-	-	-	-	-	-	-	-	-	-	+	-	-	-	-	-	-	-	-	+	+	-	-	-	-	-	-	-	-	-	-	-	-	-	-	+	-	-	-	-	-	-	-	-	-	-	-	-	-	-	-	-
2981	2468	hypothetical protein	4	-	-	-	-	-	-	-	-	-	-	-	-	-	-	-	-	-	-	-	-	-	-	-	-	-	-	-	-	-	-	-	-	-	-	-	-	-	-	-	-	-	-	-	+	-	+	-	+	-	-	-	+	-
2982	2472	hypothetical protein	4	-	-	-	-	-	-	-	-	-	-	-	-	-	-	-	-	-	-	-	-	-	-	-	-	-	-	-	-	-	-	-	-	-	-	-	-	-	-	-	-	-	-	-	+	-	+	-	+	+	-	-	-	-
2983	2475	hypothetical protein	4	-	-	-	-	-	-	-	-	-	-	-	-	-	-	-	-	-	-	-	-	-	-	-	-	-	-	-	-	-	-	-	-	-	-	-	-	+	-	+	-	-	-	-	-	-	+	-	-	+	-	-	-	-
2984	2481	PTS system, mannitol-specific IIA component (EC 2.7.1.69)	4	-	-	-	-	-	-	-	-	-	-	-	-	-	-	-	-	-	-	-	-	-	-	-	-	-	-	-	-	-	-	-	-	-	-	-	+	+	-	-	-	-	-	-	-	-	-	-	-	-	-	-	-	-	+	+	-	-
2985	2482	Mannitol operon activator, BglG family	4	-	-	-	-	-	-	-	-	-	-	-	-	-	-	-	-	-	-	-	-	-	-	-	-	-	-	-	-	-	-	-	-	-	-	-	+	+	-	-	-	-	-	-	-	-	-	-	-	-	-	-	-	-	+	+	-	-
2986	2483	Mannitol-1-phosphate 5-dehydrogenase (EC 1.1.1.17)	4	-	-	-	-	-	-	-	-	-	-	-	-	-	-	-	-	-	-	-	-	-	-	-	-	-	-	-	-	-	-	-	-	-	-	-	+	+	-	-	-	-	-	-	-	-	-	-	-	-	-	-	-	-	+	+	-	-
2987	2615	Phage lysin	4	-	-	-	-	-	-	-	-	-	-	-	-	-	-	-	+	+	+	-	-	-	-	-	-	-	-	-	-	-	-	-	-	-	-	-	-	-	-	-	-	-	-	-	+	-	-	-	-	-	-	-	-	-	-	-	-	-
2988	2621	Neopullulanase (EC 3.2.1.135)	4	-	-	-	-	-	-	-	-	-	-	-	-	-	-	-	+	-	-	-	-	-	-	-	-	+	+	-	-	-	-	-	-	-	-	-	-	-	-	-	-	+	-	-	-	-	-	-	-	-	-	-	-	-	-	-	-	-
2989	2671	Histidine kinase of the competence regulon ComD	4	-	-	-	-	-	-	-	-	-	-	-	-	-	-	-	-	-	-	-	-	-	-	-	-	-	-	-	-	-	-	-	-	-	-	-	+	+	+	-	-	-	-	-	-	-	-	-	-	-	-	+	-	-	-	-	-	-
2990	2673	Transposase	4	-	-	-	-	-	-	-	-	-	-	-	-	-	-	-	-	-	-	-	-	-	-	-	-	-	-	-	-	-	-	-	-	-	-	-	-	+	-	-	-	-	-	-	-	-	-	-	-	-	-	-	+	+	+	-	-	-
2991	2777	Putative trimethylamine dehydrogenase (EC 1.5.8.2)	4	-	-	-	-	-	-	-	-	-	-	-	-	-	-	-	-	-	-	-	-	-	-	-	-	-	-	-	-	-	-	-	-	-	-	-	-	-	-	-	-	-	-	-	-	-	-	+	-	-	+	+	-	+	-	-	-	-
2992	2801	Oxidoreductase (EC 1.1.1.-)	4	-	-	-	+	-	-	-	-	-	-	-	-	-	-	-	-	-	-	-	-	-	-	-	-	-	-	-	-	-	-	-	-	-	-	-	-	-	-	-	-	-	-	+	-	-	-	-	-	-	-	-	-	+	+	-	-	-
2993	2872	putative transcriptional regulator	4	-	-	-	-	-	-	-	-	-	-	-	-	-	-	-	-	-	-	-	-	-	-	-	-	-	-	-	-	-	-	-	-	-	-	-	-	-	-	+	+	+	-	-	-	-	-	-	-	-	-	-	+	-	-	-	-	-
2994	2902	hypothetical protein	4	-	-	-	-	-	-	-	-	-	-	-	-	-	-	-	-	-	-	-	-	-	-	-	-	-	-	-	-	-	-	-	-	+	-	-	-	-	-	-	-	-	+	-	+	-	-	-	-	-	+	-	-	-	-	-	-	-
2995	2903	Integrase	4	-	-	-	-	-	-	-	-	-	-	-	-	-	-	-	-	-	-	-	-	-	-	-	-	-	-	-	-	-	-	-	-	+	-	-	-	-	-	-	-	-	+	-	+	-	-	-	-	-	+	-	-	-	-	-	-	-
2996	2905	Adenine-specific methyltransferase (EC 2.1.1.72)	4	-	-	-	-	-	-	-	-	+	-	-	-	-	-	-	-	-	-	-	-	-	-	-	-	-	-	-	-	-	-	-	-	-	-	-	-	-	-	-	-	-	-	-	-	-	-	-	-	-	+	-	-	-	+	-	+	-
2997	2920	hypothetical protein	4	-	-	-	-	-	-	-	-	-	-	-	-	-	-	-	-	-	-	-	-	-	-	-	-	-	-	-	-	-	-	-	-	-	-	-	-	-	-	-	-	-	-	-	-	-	-	-	-	-	-	+	-	+	+	+	-	-
2998	2924	probable beta-D-galactosidase	4	-	-	-	+	-	-	-	-	-	-	-	-	-	-	-	-	-	-	-	-	-	-	-	-	-	-	-	-	-	-	-	-	-	-	-	-	-	-	-	-	-	-	+	-	-	-	-	-	-	-	-	-	+	+	-	-	-
2999	3047	Type I restriction-modification system, DNA-methyltransferase subunit M (EC 2.1.1.72)	4	-	-	-	-	-	-	-	-	-	-	-	-	-	-	-	-	-	-	-	-	-	-	-	-	-	-	-	-	-	-	-	-	-	-	-	-	-	+	-	-	-	-	-	+	-	-	-	-	-	-	+	-	-	-	+	-	-
3000	3148	Mobile element protein	4	-	-	-	-	-	-	-	-	-	-	-	-	-	-	-	-	-	-	-	-	-	-	-	-	-	-	-	-	-	-	-	-	-	-	-	-	-	-	-	-	+	+	-	-	-	-	+	-	+	-	-	-	-	-	-	-	-
3001	3152	Resolvase	4	-	-	-	-	-	-	-	-	-	-	-	-	-	-	-	-	-	-	-	-	-	-	-	-	-	-	-	-	+	-	-	-	-	-	-	-	-	-	-	-	-	-	-	-	-	-	-	-	+	-	-	+	-	-	+	-
3002	3162	Type I restriction-modification system, restriction subunit R (EC 3.1.21.3)	4	-	-	-	-	-	-	-	-	-	-	-	-	-	-	-	-	-	-	-	-	-	-	-	-	-	-	-	-	-	-	-	-	-	-	-	-	-	+	-	-	-	-	-	+	-	-	-	-	-	-	+	-	-	-	+	-	-
3003	3166	Dienelactone hydrolase and related enzymes	4	-	-	-	-	-	-	-	-	-	-	-	-	-	-	-	-	-	-	-	-	-	-	-	-	-	-	-	-	-	-	-	-	-	-	-	-	-	-	-	-	-	-	-	-	-	-	-	-	-	-	+	+	-	-	+	+	-
3004	3243	Phage tail length tape-measure protein	4	-	-	-	-	-	-	-	-	-	-	-	-	-	-	-	-	+	-	-	-	-	-	-	-	-	-	-	-	-	-	-	-	-	-	-	-	-	-	-	-	+	-	-	-	-	-	-	-	+	-	-	-	-	+	-	-	-	-	-
3005	3254	Cyclomaltodextrin glucanotransferase (EC 2.4.1.19); Maltogenic alpha-amylase (EC 3.2.1.133)	4	-	-	-	-	-	-	-	-	-	-	-	-	-	-	-	+	-	-	-	-	-	-	-	-	+	+	-	-	-	-	-	-	-	-	-	-	-	-	-	-	+	-	-	-	-	-	-	-	-	-	-	-	-	-	-	-	-
3006	3270	hypothetical protein	4	-	-	-	-	-	-	-	-	-	-	-	-	-	-	-	-	-	-	-	-	-	-	-	-	-	-	-	-	-	-	-	-	-	-	-	-	-	-	+	+	+	-	-	-	-	-	-	-	-	-	-	+	-	-	-	-	-
3007	3333	Degenerative transposase	4	-	-	-	-	-	-	-	-	-	-	-	-	-	-	-	-	-	-	-	-	-	-	-	-	-	-	-	-	-	-	-	-	-	-	-	-	-	-	-	-	-	-	-	-	-	-	-	-	-	-	+	-	+	+	-	+	-
3008	3335	Multiple sugar ABC transporter, membrane-spanning permease protein MsmF	4	-	-	-	+	-	-	-	-	-	-	-	-	-	-	-	-	-	-	-	-	-	-	-	-	-	-	-	-	-	-	-	-	-	-	-	-	-	-	-	-	+	-	-	-	-	-	-	-	-	-	+	+	-	-	-
3009	3336	ABC transporter substrate-binding protein - sugar transport	4	-	-	-	+	-	-	-	-	-	-	-	-	-	-	-	-	-	-	-	-	-	-	-	-	-	-	-	-	-	-	-	-	-	-	-	-	-	-	-	-	+	-	-	-	-	-	-	-	-	-	+	+	-	-	-
3010	3337	ABC-type sugar transport systems, permease component	4	-	-	-	+	-	-	-	-	-	-	-	-	-	-	-	-	-	-	-	-	-	-	-	-	-	-	-	-	-	-	-	-	-	-	-	-	-	-	-	-	+	-	-	-	-	-	-	-	-	-	+	+	-	-	-
3011	3428	hypothetical protein	4	-	-	-	-	-	-	-	-	-	-	-	-	-	-	-	+	-	-	-	-	-	-	-	-	+	+	-	-	-	-	-	-	-	-	-	-	-	-	-	-	+	-	-	-	-	-	-	-	-	-	-	-	-	-	-	-	-
3012	3431	Phage CI-like repressor	4	-	-	-	-	-	-	-	-	-	-	-	-	-	-	-	+	-	-	-	-	-	-	-	-	-	-	-	-	-	-	-	-	-	-	-	-	-	-	-	-	-	-	-	-	+	-	-	-	-	+	-	-	+	-	-
3013	3432	Maltose/maltodextrin ABC transporter, permease protein MalF	4	-	-	-	-	-	-	-	-	-	-	-	-	-	-	-	+	-	-	-	-	-	-	-	-	+	+	-	-	-	-	-	-	-	-	-	-	-	-	-	-	+	-	-	-	-	-	-	-	-	-	-	-	-	-	-	-	-
3014	3435	Maltose/maltodextrin ABC transporter, substrate binding periplasmic protein MalE	4	-	-	-	-	-	-	-	-	-	-	-	-	-	-	-	+	-	-	-	-	-	-	-	-	+	+	-	-	-	-	-	-	-	-	-	-	-	-	-	-	+	-	-	-	-	-	-	-	-	-	-	-	-	-	-	-	-
3015	3436	Maltose/maltodextrin ABC transporter, permease protein MalG	4	-	-	-	-	-	-	-	-	-	-	-	-	-	-	-	+	-	-	-	-	-	-	-	-	+	+	-	-	-	-	-	-	-	-	-	-	-	-	-	-	+	-	-	-	-	-	-	-	-	-	-	-	-	-	-	-	-
3016	3444	Helix-turn-helix domain protein	4	-	-	-	-	-	-	-	-	-	-	-	-	-	-	-	-	-	-	-	-	-	-	-	-	-	-	-	-	-	-	-	-	-	-	-	-	-	-	+	+	-	-	-	-	+	-	-	-	-	-	-	-	-	-	-	-	+
3017	3456	hypothetical protein	4	-	-	-	-	-	-	-	-	-	-	-	-	-	-	-	-	-	-	-	-	-	-	-	-	-	-	-	-	-	-	-	-	-	-	-	-	-	-	-	-	-	-	+	-	+	+	-	-	-	-	-	-	-	-	+
3018	3469	hypothetical protein	4	-	-	-	-	-	-	-	-	-	-	-	-	-	-	-	-	-	-	-	-	-	-	-	-	-	-	-	-	-	-	-	-	+	-	-	-	-	-	-	-	-	+	-	+	-	-	-	-	-	+	-	-	-	-	-	-	-
3019	3472	DNA primase, phage associated	4	-	-	-	-	-	-	-	-	-	-	-	-	-	-	-	-	-	-	-	+	-	-	-	-	-	-	-	-	-	-	+	+	-	-	-	-	-	-	-	-	-	-	-	-	+	-	-	-	-	-	-	-	-	-	-	-	-
3020	3477	Ribokinase (EC 2.7.1.15)	4	-	-	-	-	-	-	-	-	-	-	-	-	-	-	-	-	-	-	-	-	-	-	-	-	-	-	-	-	-	-	-	-	-	-	-	-	-	-	-	-	-	-	-	-	-	+	+	+	+	-	-	-	-
3021	3480	GNAT family acetyltransferase BA3010	4	-	-	-	-	-	-	-	-	-	-	-	-	-	-	-	-	-	-	-	-	-	-	-	-	-	-	-	-	-	-	-	-	-	-	-	-	-	-	-	-	-	-	-	-	-	+	+	-	+	-	+	-	-
3022	3505	Sialidase (EC 3.2.1.18)	4	-	-	-	+	-	-	-	-	-	-	-	-	-	-	-	-	-	-	-	-	-	-	-	-	-	-	-	-	-	-	-	-	-	-	-	-	-	-	-	-	+	-	-	-	-	-	-	-	-	-	+	+	-	-	-
3023	3597	putative Abi-alpha protein	4	+	+	+	-	-	-	-	-	-	-	+	-	-	-	-	-	-	-	-	-	-	-	-	-	-	-	-	-	-	-	-	-	-	-	-	-	-	-	-	-	-	-	-	-	-	-	-	-	-	-	-	-	-	-	-	-	-
3024	3612	putative transport ATP-binding protein	4	+	+	+	-	-	-	-	-	-	-	+	-	-	-	-	-	-	-	-	-	-	-	-	-	-	-	-	-	-	-	-	-	-	-	-	-	-	-	-	-	-	-	-	-	-	-	-	-	-	-	-	-	-	-	-	-	-
3025	3625	Phage terminase, small subunit	4	-	-	-	-	-	-	-	-	-	-	-	-	-	-	-	+	+	+	-	-	-	-	-	-	-	-	-	-	-	-	-	-	-	-	-	-	-	-	-	-	-	+	-	-	-	-	-	-	-	-	-	-	-	-	-
3026	3647	hypothetical protein	4	-	-	-	-	-	-	-	-	-	-	-	-	-	+	-	-	-	-	-	-	-	-	-	+	-	-	-	-	-	-	-	-	-	+	-	-	-	-	+	-	-	-	-	-	-	-	-	-	-	-	-	-	-	-	-	-	-
3027	3659	putative phage replication protein	4	-	-	-	-	-	-	-	-	-	-	-	-	-	-	+	-	-	-	-	-	-	-	+	-	-	-	-	-	-	-	-	-	-	-	-	+	-	-	-	-	-	-	-	-	-	-	-	-	-	+	-	-	-	-	-
3028	3680	hypothetical protein	4	-	-	-	-	-	-	-	-	-	-	-	-	-	-	-	-	-	-	-	-	-	-	-	-	-	-	-	-	-	-	-	-	-	-	-	-	-	-	-	-	+	+	-	-	-	+	-	+	-	-	-	-	-	-	-
3029	3682	hypothetical protein	4	-	-	-	-	-	-	-	-	-	-	-	-	-	-	-	-	-	-	-	-	-	-	-	-	-	-	-	-	-	-	+	+	+	-	-	-	-	-	-	-	-	-	-	-	-	+	-	-	-	-	-	-	-	-	-
3030	3692	Phage Terminase Small Subunit	4	-	-	-	-	-	-	-	-	-	-	-	-	-	-	-	-	-	-	-	-	-	-	-	-	-	-	-	-	-	-	-	-	-	-	-	-	-	-	+	-	-	+	-	-	-	-	-	-	-	+	-	-	+	-	-
3031	3702	Cation transport ATPase	4	-	-	-	-	-	-	-	-	-	-	-	-	+	-	-	-	-	-	-	-	-	-	-	-	-	-	-	+	-	-	-	-	-	-	-	-	-	+	-	-	-	-	-	-	-	-	-	-	-	-	-	-	-	+	-
3032	3722	hypothetical protein	4	-	-	-	-	-	-	-	-	-	-	-	-	-	-	-	-	-	-	-	-	-	-	-	-	-	-	-	-	-	-	-	-	-	-	-	-	-	-	-	-	-	-	-	-	-	-	-	-	+	-	+	+	+	-	-
3033	3723	helix-turn-helix domain protein	4	+	+	+	-	-	-	-	-	-	-	+	-	-	-	-	-	-	-	-	-	-	-	-	-	-	-	-	-	-	-	-	-	-	-	-	-	-	-	-	-	-	-	-	-	-	-	-	-	-	-	-	-	-	-	-	-	-
3034	3766	Phage major capsid protein	4	-	-	-	-	-	-	-	-	-	-	-	-	-	-	-	-	-	-	-	+	-	-	-	-	-	-	-	-	-	-	+	+	-	+	-	-	-	-	-	-	-	-	-	-	-	-	-	-	-	-	-	-	-	-	-	-	-
3035	3767	Protein of unknown function DUF994	4	-	-	-	-	-	-	-	-	-	-	-	-	-	-	-	-	-	-	-	+	+	+	-	-	-	-	-	+	-	-	-	-	-	-	-	-	-	-	-	-	-	-	-	-	-	-	-	-	-	-	-	-	-	-	-	-	-
3036	3864	hypothetical protein	4	-	-	-	-	-	-	-	-	-	-	-	-	-	-	-	+	+	+	-	-	-	-	-	-	-	-	-	-	-	-	-	-	-	-	-	-	-	-	-	-	-	+	-	-	-	-	-	-	-	-	-	-	-	-	-
3037	3867	Single-stranded DNA-binding protein	4	-	-	-	-	-	-	-	-	-	-	-	-	-	-	-	+	+	+	-	-	-	-	-	-	-	-	-	-	-	-	-	-	-	-	-	-	-	-	-	-	-	+	-	-	-	-	-	-	-	-	-	-	-	-	-
3038	3892	hypothetical protein	4	-	-	-	-	-	-	-	-	-	-	-	-	-	-	-	-	-	-	-	-	-	-	-	-	-	-	-	-	-	-	-	-	-	-	-	-	+	+	-	-	-	-	-	-	-	-	+	-	-	+	-	-	-	-	-
3039	3908	dTDP-glucose 4,6-dehydratase (EC 4.2.1.46)	4	-	-	-	-	-	-	-	-	-	-	-	-	-	-	-	-	-	-	-	-	-	-	-	-	-	-	-	-	-	-	-	+	-	+	-	+	-	-	-	-	-	-	-	-	-	-	-	-	+	-	-	-	-
3040	3924	hypothetical protein	4	-	-	-	-	-	-	-	-	-	-	-	-	-	-	-	-	-	-	-	-	-	-	-	-	-	-	-	-	-	-	-	-	-	-	-	-	-	-	-	-	-	-	+	-	-	+	-	+	-	-	-	-	+
3041	3928	Acetyltransferase (EC 2.3.1.-)	4	-	-	-	-	-	-	-	-	-	-	-	-	-	-	-	-	-	-	-	-	-	-	-	-	-	-	-	-	-	-	-	-	-	-	-	-	-	-	-	-	-	-	-	-	-	-	+	-	+	-	-	+	+
3042	3933	LLAKR2I restriction enzyme	4	-	-	-	-	-	-	-	-	-	-	-	-	-	-	-	-	-	-	-	-	-	-	-	-	-	-	-	-	-	-	-	-	-	-	-	-	-	-	-	+	+	-	-	+	-	-	+	-	-	-	-	-	-
3043	3940	hypothetical protein	4	-	-	-	-	-	-	-	-	-	-	-	-	-	-	-	-	-	-	-	-	-	-	-	-	-	-	-	-	-	-	-	-	-	-	-	-	+	-	-	-	-	-	+	+	-	+	-	-	-	-	-	-	-
3044	3943	DNA-cytosine methyltransferase (EC 2.1.1.37)	4	-	-	-	-	-	-	-	-	-	-	-	-	-	-	-	-	-	-	-	-	-	-	-	-	-	-	-	-	-	-	-	-	-	-	-	-	-	-	-	+	+	-	-	+	-	-	+	-	-	-	-	-	-
3045	3958	Tn5252, Orf 9 protein	4	-	-	-	-	-	-	-	-	-	-	-	-	-	-	-	-	-	-	-	-	-	-	-	-	-	-	-	-	-	-	-	-	+	-	-	-	-	-	-	-	-	+	-	+	-	-	-	-	-	+	-	-	-	-	-	-	-
3046	3960	Tn5252, Orf 9 protein	4	-	-	-	-	-	-	-	-	-	-	-	-	-	-	-	-	-	-	-	-	-	-	-	-	-	-	-	-	-	-	-	-	+	-	-	-	-	-	-	-	-	+	-	+	-	-	-	-	-	+	-	-	-	-	-	-	-
3047	3965	hypothetical protein	4	-	-	-	-	-	-	-	-	-	-	-	-	-	-	-	-	-	-	-	-	-	-	-	-	-	-	-	-	-	-	-	-	-	-	-	-	-	-	-	-	-	+	-	-	-	-	+	+	-	-	+	-	-	-
3048	3984	Transposase and inactivated derivative	4	-	-	-	-	-	-	-	-	-	-	-	-	-	-	-	-	-	-	-	-	-	-	-	-	-	-	-	-	-	-	-	-	-	-	-	-	-	+	+	-	-	-	-	-	-	-	-	-	-	-	-	-	-	-	-	+	+	-	-
3049	3986	Putative DNA-binding protein in cluster with Type I restriction-modification system	4	-	-	-	-	-	-	-	-	-	-	-	-	-	-	-	-	-	-	-	-	-	-	-	-	-	-	-	-	-	-	-	-	-	-	-	-	-	+	-	-	-	-	-	+	-	-	-	-	-	-	+	-	-	-	+	-	-
3050	3990	hypothetical protein	4	-	-	-	-	-	-	-	-	-	-	-	-	-	-	-	-	-	-	-	-	-	-	-	-	-	-	-	-	-	-	-	-	-	-	-	-	-	-	-	-	-	-	-	-	-	-	-	-	+	-	+	+	+	-	-

Table S5. Precence/absence of each HG among 57 *S. suis* strains

				1														13			25					28						104		94			76			53/54		Divergent *S. suis*																
Index[a]	ClutserID	Gene products	Members	98HAH33	05ZYH33	SC84	A7	SS12	P1/7	DAT273	NIAH11434	JS14	GZ1	BM407	S735	13730	MNCM07	NCTC 10446	14636	NCTC 10237	ST1	2651	89-1591	MNCM04	MNCM25	8074	D9	4961	ST3	MNCM43	DAT292	DAT260	NIAH11435	MNCM21	MNCM50	89-2479	DAT234	DAT290	93A	42A	NT77	6407	11538	8830	12814	92-1400	89-590	2726	2524	89-3576-3	4417	D12	10581	89-5259	88-5299A	R61	92-1191	14A	92-4172	22083
3051	3995	Membrane-bound protease, CAAX family	4	-	-	-	-	-	-	-	-	-	-	-	-	-	-	-	-	-	-	-	-	-	-	-	-	-	-	-	-	-	-	-	-	-	-	-	-	-	-	-	-	-	-	-	-	-	-	-	-	-	-	+	-	+	+	+	-	-
3052	4001	hypothetical protein	4	-	-	-	-	-	-	-	-	-	-	-	-	-	-	-	-	-	-	-	+	+	+	-	-	-	-	-	-	-	-	-	-	+	-	-	-	-	-	-	-	-	-	-	-	-	-	-	-	-	-	-	-	-	-	-	-	-
3053	4008	Recombinase	4	-	-	-	-	-	-	-	-	+	-	-	-	-	-	-	-	-	-	-	-	-	-	-	-	-	-	-	-	-	-	-	-	-	-	-	-	-	-	-	-	-	-	-	-	-	-	-	-	-	-	+	-	+	+	-	-	-
3054	4013	Site-specific recombinase	4	+	+	+	-	-	-	-	-	-	-	-	-	-	-	-	-	-	-	-	-	-	-	-	-	-	-	-	-	-	-	-	-	-	-	-	-	-	-	-	-	-	-	-	-	-	-	-	-	-	-	-	-	-	-	-	+	-
3055	4094	UvrD/REP helicase family protein	4	+	+	+	-	-	-	-	-	-	-	+	-	-	-	-	-	-	-	-	-	-	-	-	-	-	-	-	-	-	-	-	-	-	-	-	-	-	-	-	-	-	-	-	-	-	-	-	-	-	-	-	-	-	-	-	-	-
3056	4117	FIG131328: Predicted ATP-dependent endonuclease of the OLD family	4	+	+	+	-	-	-	-	-	-	-	+	-	-	-	-	-	-	-	-	-	-	-	-	-	-	-	-	-	-	-	-	-	-	-	-	-	-	-	-	-	-	-	-	-	-	-	-	-	-	-	-	-	-	-	-	-	-
3057	4178	replication protein B	4	-	-	-	-	-	-	-	-	-	-	-	-	-	-	-	-	-	-	-	-	-	-	-	-	+	-	-	-	-	-	-	-	-	-	-	-	-	-	-	-	-	+	-	-	-	-	-	-	-	+	-	-	-	-	-	-	+
3058	4188	Phage major capsid protein	4	-	-	-	-	-	-	-	-	-	-	-	-	-	-	-	-	-	-	+	-	-	-	-	-	-	-	-	-	-	-	-	-	-	-	-	-	-	-	+	-	-	-	-	-	-	-	-	-	-	-	+	-	+	-	-	-	-
3059	4198	hypothetical protein	4	-	-	-	-	-	-	-	-	-	-	-	-	-	-	-	-	-	-	-	-	-	-	-	-	-	-	-	-	-	-	-	-	+	-	-	-	-	-	+	+	-	-	-	-	-	-	-	-	-	-	-	-	-	-	-	-	+
3060	4202	Site-specific recombinase	4	-	-	-	-	-	-	-	-	-	-	-	-	-	-	-	-	-	-	-	-	-	-	-	-	-	-	-	-	-	-	+	-	-	-	-	-	+	+	-	-	-	-	-	-	-	-	-	-	-	-	-	-	-	-	+
3061	4203	hypothetical protein	4	-	-	-	-	-	-	-	-	-	-	-	-	-	-	-	-	-	-	-	-	-	-	-	-	-	-	-	-	-	-	-	-	-	-	-	-	-	+	-	-	-	-	-	-	-	-	-	-	-	-	+	-	+	+	-	-	-
3062	4205	hypothetical protein	4	-	-	-	-	-	-	-	-	-	-	-	-	-	-	-	-	-	-	-	-	-	-	-	-	-	-	-	-	-	-	+	-	-	-	-	-	+	+	-	-	-	-	-	-	-	-	-	-	-	-	-	-	-	-	+
3063	4206	Unknown	4	-	-	-	-	-	-	-	-	-	-	-	-	-	+	-	-	-	-	-	-	-	-	-	+	-	-	-	-	-	-	-	-	-	+	-	-	-	-	+	-	-	-	-	-	-	-	-	-	-	-	-	-	-	-	-	-	-
3064	4217	ATP-dependent DNA helicase recG (EC 3.6.1.-)	4	-	-	-	-	-	-	-	-	-	-	-	-	-	-	-	-	-	-	-	-	-	-	-	-	-	-	-	-	-	-	-	-	-	-	-	-	+	+	-	-	-	-	-	-	-	-	-	-	-	+	+	-	-	-	-
3065	4221	ABC transporter related	4	-	-	-	-	-	-	-	-	-	-	-	-	-	-	-	-	-	-	-	-	-	-	-	-	-	-	-	-	-	-	-	-	-	-	-	-	-	-	-	-	-	+	+	+	+	-	-	-	-	-	-	-	-
3066	4227	Tn5252, Orf 10 protein	4	-	-	-	-	-	-	-	-	-	-	-	-	-	+	-	-	-	-	-	-	-	-	-	+	-	-	-	-	-	-	-	-	-	+	-	-	-	-	-	-	-	-	-	-	-	+	-	-	-	-	-	-	-	-	-
3067	4237	DNA replication protein dnaD	4	-	-	-	-	-	-	-	-	-	-	-	-	-	-	-	-	-	-	-	-	-	-	-	-	-	-	-	-	-	-	-	-	-	-	+	+	+	-	-	-	-	-	-	-	-	-	-	-	-	-	-	-	+	-	-
3068	4240	hypothetical protein	4	-	-	-	-	-	-	-	-	-	-	-	-	-	-	-	-	-	-	-	-	-	-	-	-	-	-	-	-	+	+	+	-	-	-	-	-	-	-	-	-	-	-	-	+	-	-	-	-	-	-	-	-	-
3069	4249	Mobile element protein	4	-	-	-	-	-	-	-	-	-	-	-	-	-	-	-	-	-	-	-	-	-	-	-	-	-	-	-	-	-	-	-	+	+	+	-	-	-	-	-	-	-	-	-	-	-	-	-	-	-	-	+	-	-
3070	4250	hypothetical protein	4	-	-	-	-	-	-	-	-	-	-	-	-	-	-	-	-	-	-	-	-	-	-	-	-	-	-	-	-	-	-	-	-	-	-	-	-	+	-	-	-	-	-	+	+	-	+	-	-	-	-	-	-	-
3071	4269	hypothetical protein	4	-	-	-	-	-	-	-	-	-	-	-	-	-	-	-	-	-	-	-	-	-	-	-	-	-	-	-	-	-	-	-	-	-	-	-	-	-	-	-	-	-	-	-	-	-	+	+	+	+	-	-	-	-
3072	4272	ABC-type sugar transport system, permease component	4	-	-	-	-	-	-	-	-	-	-	-	-	-	-	-	-	-	-	-	-	-	-	-	-	-	-	-	-	-	-	-	-	-	-	-	-	-	-	-	-	-	-	-	-	-	+	+	+	+	-	-	-	-
3073	4273	Mobile element protein	4	-	-	-	-	-	-	-	-	-	-	-	-	-	-	-	-	-	-	-	-	-	-	-	-	-	-	-	-	-	-	-	-	-	-	-	-	-	-	-	-	-	-	-	-	-	+	+	-	-	+	+	-	-
3074	4277	Histidine kinase of the competence regulon ComD	4	-	-	-	-	-	-	-	-	-	-	-	-	-	-	-	-	-	-	-	-	-	-	-	-	-	-	-	-	-	-	-	-	-	-	-	-	-	-	-	-	-	-	-	-	-	+	+	+	+	-	-	-	-
3075	4279	Amino acid transporter	4	-	-	-	-	-	-	-	-	-	-	-	-	-	-	-	-	-	-	-	-	-	-	-	-	-	-	-	-	-	-	-	-	-	-	-	-	-	-	-	-	-	-	-	-	-	+	+	+	+	-	-	-	-
3076	4285	hypothetical protein	4	-	-	-	-	-	-	-	-	-	-	-	-	-	-	-	-	-	-	+	-	-	-	-	-	-	-	-	-	-	-	-	-	-	-	-	-	-	-	-	-	-	-	+	-	-	-	-	-	-	-	+	+	-	-
3077	4289	hypothetical protein	4	-	-	-	-	-	-	-	-	-	-	-	-	-	-	-	-	-	-	-	-	-	-	-	-	-	-	-	-	-	-	-	+	+	-	-	-	-	-	-	-	-	-	-	-	-	-	+	+	-	-	-	-	-
3078	4295	restriction enzyme BcgI alpha chain-like protein(EC:2.1.1.72)	4	-	-	-	-	-	-	-	-	-	-	-	-	-	-	-	-	-	-	-	-	-	-	-	-	-	-	-	-	-	-	-	+	+	+	-	-	-	-	-	-	-	-	-	-	-	-	-	-	+	-	-	-	-
3079	4328	hypothetical protein	4	-	-	-	-	-	-	-	-	-	-	-	-	-	-	-	-	-	-	-	-	-	-	-	-	-	-	-	-	-	-	-	-	-	-	-	-	-	-	-	+	-	-	-	-	-	-	+	-	+	-	+	-	-
3080	4344	Site-specific recombinase	4	-	-	-	-	-	-	-	-	+	-	-	-	-	-	-	-	-	-	-	-	-	-	-	-	-	-	-	-	-	-	-	-	-	-	-	-	-	-	-	-	-	-	-	-	-	-	+	-	+	+	-	-	-
3081	4346	hypothetical protein	4	-	-	-	-	-	-	-	-	+	-	-	-	-	-	-	-	-	-	-	-	-	-	-	-	-	-	-	-	-	-	-	-	-	-	-	-	-	-	-	-	-	-	-	-	+	-	+	-	-	+	-	-	-
3082	4383	NTP-binding protein	4	-	-	-	-	-	-	-	-	-	-	-	-	-	-	-	-	-	-	-	+	+	+	-	-	-	-	-	+	-	-	-	-	-	-	-	-	-	-	-	-	-	-	-	-	-	-	-	-	-	-	-	-	-
3083	4384	DNA helicase, phage-associated	4	-	-	-	-	-	-	-	-	-	-	-	-	-	-	-	-	-	-	-	+	+	+	-	-	-	-	-	+	-	-	-	-	-	-	-	-	-	-	-	-	-	-	-	-	-	-	-	-	-	-	-	-	-
3084	4385	putative replication protein	4	-	-	-	-	-	-	-	-	-	-	-	-	-	-	-	-	-	-	-	+	+	+	-	-	-	-	-	+	-	-	-	-	-	-	-	-	-	-	-	-	-	-	-	-	-	-	-	-	-	-	-	-	-
3085	4590	putative replication protein - phage associated	4	-	-	-	-	-	-	-	-	-	-	-	-	-	-	-	+	+	+	-	-	-	-	-	-	-	-	-	-	-	-	-	-	-	-	-	-	-	-	-	-	-	-	-	-	-	-	-	-	+	-	-	-	-
3086	4591	Phage capsid and scaffold	4	-	-	-	-	-	-	-	-	-	-	-	-	-	-	-	+	+	+	-	-	-	-	-	-	-	-	-	-	-	-	-	-	-	-	-	-	-	-	-	-	-	+	-	-	-	-	-	-	-	-	-	-	-	-	-
3087	4592	Holin	4	-	-	-	-	-	-	-	-	-	-	-	-	-	-	-	+	+	+	-	-	-	-	-	-	-	-	-	-	-	-	-	-	-	-	-	-	-	-	-	-	-	+	-	-	-	-	-	-	-	-	-	-	-	-	-
3088	4596	hypothetical protein	4	-	-	-	-	-	-	-	-	-	-	-	-	-	-	-	+	+	+	-	-	-	-	-	-	-	-	-	-	-	-	-	-	-	-	-	-	-	-	-	-	-	+	-	-	-	-	-	-	-	-	-	-	-	-	-
3089	4598	Phage minor capsid protein #Fam0018	4	-	-	-	-	-	-	-	-	-	-	-	-	-	-	-	+	+	+	-	-	-	-	-	-	-	-	-	-	-	-	-	-	-	-	-	-	-	-	-	-	-	+	-	-	-	-	-	-	-	-	-	-	-	-	-
3090	4600	hypothetical protein	4	-	-	-	-	-	-	-	-	-	-	-	-	-	-	-	+	+	+	-	-	-	-	-	-	-	-	-	-	-	-	-	-	-	-	-	-	-	-	-	-	-	+	-	-	-	-	-	-	-	-	-	-	-	-	-
3091	4601	Integrase	4	-	-	-	-	-	-	-	-	-	-	-	-	-	-	-	-	+	+	-	-	-	-	-	-	-	-	-	-	-	-	-	-	-	-	-	-	-	-	-	-	-	+	-	-	-	+	-	-	-	-	-	-	-	-	-
3092	4604	putative minor capsid protein 2	4	-	-	-	-	-	-	-	-	-	-	-	-	-	-	-	+	+	+	-	-	-	-	-	-	-	-	-	-	-	-	-	-	-	-	-	-	-	-	-	-	-	+	-	-	-	-	-	-	-	-	-	-	-	-	-
3093	4606	hypothetical protein	4	-	-	-	-	-	-	-	-	-	-	-	-	-	-	-	+	+	+	-	-	-	-	-	-	-	-	-	-	-	-	-	-	-	-	-	-	-	-	-	-	-	+	-	-	-	-	-	-	-	-	-	-	-	-	-
3094	4607	Phage protein	4	-	-	-	-	-	-	-	-	-	-	-	-	-	-	-	+	+	+	-	-	-	-	-	-	-	-	-	-	-	-	-	-	-	-	-	-	-	-	-	-	-	+	-	-	-	-	-	-	-	-	-	-	-	-	-
3095	4616	hypothetical protein	4	-	-	-	-	-	-	-	-	-	-	-	-	-	-	-	-	-	-	-	-	-	-	+	+	+	+	-	-	-	-	-	-	-	-	-	-	-	-	-	-	-	-	-	-	-	-	-	-	-	-	-	-	-	-	-
3096	4620	4-keto-6-deoxy-N-Acetyl-D-hexosaminyl-(Lipid carrier) aminotransferase	4	-	-	-	-	-	-	-	-	-	-	-	-	-	-	-	-	-	-	-	-	-	-	-	-	+	+	-	-	-	-	-	-	-	-	-	-	-	+	-	-	-	-	-	-	-	-	-	+	-	-	-	-	-	-	-
3097	4624	Lipid carrier : UDP-N-acetylgalactosaminyltransferase (EC 2.4.1.-)	4	-	-	-	-	-	-	-	-	-	-	-	-	-	-	-	-	-	-	-	-	-	-	-	-	+	+	-	-	-	-	-	-	-	-	-	-	-	+	-	-	-	-	-	-	-	-	-	+	-	-	-	-	-	-	-
3098	4628	hypothetical protein	4	-	-	-	-	-	-	-	-	-	-	-	-	-	-	-	-	-	-	-	-	-	-	-	-	-	-	-	-	-	-	-	-	-	-	-	-	-	-	+	-	-	-	-	-	-	-	-	-	+	-	-	-	+	+	-	-
3099	4629	hypothetical protein	4	-	-	-	-	-	-	-	-	-	-	-	-	-	-	-	-	-	-	-	-	-	-	-	-	-	-	-	-	-	-	-	-	-	-	-	-	-	-	+	-	-	-	-	-	-	-	-	-	+	-	-	-	+	+	-	-
3100	4630	hypothetical protein	4	-	-	-	-	-	-	-	-	-	-	-	-	-	-	-	-	-	-	-	-	-	-	-	-	-	-	-	-	+	-	-	-	-	-	+	+	-	-	-	-	-	-	-	-	-	-	-	-	-	-	-	-	+
3101	4633	hypothetical protein	4	-	-	-	-	-	-	-	-	-	-	-	-	-	-	-	-	-	-	-	-	-	-	-	-	-	-	-	-	+	-	-	-	-	-	+	+	-	-	-	-	-	-	-	-	-	-	-	-	-	-	-	-	+
3102	4635	Chromosome segregation ATPase	4	-	-	-	-	-	-	-	-	-	-	-	-	-	-	-	-	-	-	-	-	-	-	-	-	-	-	-	+	-	-	-	-	-	-	-	-	-	-	+	-	-	-	-	-	-	-	-	-	-	-	-	+	-	-	+
3103	4637	hypothetical protein	4	-	-	-	-	-	-	-	-	-	-	-	-	-	-	-	-	-	-	-	-	-	-	-	-	-	-	-	-	-	-	-	-	-	-	+	+	-	+	-	-	-	-	-	-	-	-	-	-	-	-	-	-	+
3104	4641	hypothetical protein	4	-	-	-	-	-	-	-	-	-	-	-	-	-	-	-	-	-	-	+	-	-	-	-	-	-	-	-	-	-	-	-	-	-	-	-	-	+	-	-	-	-	-	-	+	-	-	-	+	-	-	-	-	-	-
3105	4642	hypothetical protein	4	-	-	-	-	-	-	-	-	-	-	-	-	-	-	-	+	-	-	+	-	-	-	-	-	-	-	-	-	-	-	-	-	-	-	-	-	+	+	-	-	-	-	-	-	-	-	-	-	-	-	-	-	-
3106	4644	hypothetical protein	4	-	-	-	-	-	-	-	-	-	-	-	-	-	-	-	-	-	-	-	-	-	-	-	-	-	-	-	-	-	-	+	+	-	-	+	-	+	-	-	-	-	-	-	-	-	-	-	-	-	-	-	-	-
3107	4650	ORF101	4	-	-	-	-	-	-	-	-	-	-	-	-	-	-	-	-	-	-	-	-	-	-	-	-	-	-	-	-	-	-	+	-	-	-	-	+	-	-	-	-	-	-	-	-	-	-	+	+	-	-	-	-	-
3108	4656	Enoyl-[acyl-carrier-protein] reductase [FMN] (EC 1.3.1.9)	4	-	-	-	-	-	-	-	-	-	-	-	-	-	-	-	+	-	-	+	-	-	-	-	-	-	-	-	-	-	-	-	-	-	-	-	-	-	-	-	-	-	+	-	-	-	-	-	-	-	+	-	-	-
3109	4657	hypothetical protein	4	-	-	-	-	-	-	-	-	-	-	-	-	-	-	-	-	-	-	+	-	-	-	-	-	-	-	-	-	-	-	-	-	-	-	-	-	+	-	-	-	+	+	-	-	-	-	-	-	-	-	-	-	-
3110	4662	monooxygenase	4	-	-	-	-	-	-	-	-	-	-	-	-	-	-	-	+	-	-	+	-	-	-	-	-	-	-	-	-	-	-	-	-	-	-	-	-	-	-	-	-	-	+	-	-	-	-	-	-	-	+	-	-	-
3111	4674	hypothetical protein	4	-	-	-	+	-	-	-	-	-	-	-	-	-	-	-	+	-	+	-	-	-	-	-	-	-	-	-	-	-	-	-	-	-	-	+	-	-	-	-	-	-	-	-	-	-	-	-	-	-	-	-	-	-	-	-
3112	4690	Lipid A export ATP-binding/permease protein MsbA	4	-	-	-	-	-	-	-	-	-	-	-	-	-	-	-	-	-	-	-	-	-	-	-	-	-	-	-	-	-	-	-	-	-	-	-	-	-	-	-	-	+	-	-	+	-	-	+	-	-	-	+	-	-
3113	4703	Cell surface protein	4	-	-	-	-	-	-	-	-	-	-	-	-	-	-	-	-	-	-	-	-	-	-	-	-	-	-	-	-	-	-	-	-	-	-	-	-	-	-	-	+	+	-	+	+	-	-	-	-	-	-	-	-	-
3114	4706	Type I restriction-modification system, restriction subunit R (EC 3.1.21.3)	4	-	-	-	-	-	-	-	-	-	-	-	-	-	-	-	-	-	-	-	-	-	-	-	-	-	-	-	-	-	-	-	-	-	-	-	-	-	-	+	-	-	-	-	+	-	+	-	+	-	-	-	-	-
3115	4711	putative DNA replication protein	4	-	-	-	-	-	-	-	-	-	-	-	-	-	-	-	-	-	-	-	-	-	-	-	-	-	-	-	-	+	-	-	-	-	-	-	-	-	+	-	+	-	-	-	-	-	+	-	-	-	-	-	-	-
3116	4712	hypothetical protein	4	-	-	-	-	-	-	-	-	-	-	-	-	-	-	-	-	-	-	-	-	-	-	-	-	-	-	-	-	-	-	-	-	-	-	-	-	+	+	+	-	-	-	-	-	-	-	-	-	-	-	-	+	-
3117	4716	hypothetical protein	4	-	-	-	-	-	-	-	-	-	-	-	-	-	-	-	-	-	-	-	-	-	-	-	-	-	-	-	-	-	-	-	-	-	-	-	-	+	-	+	-	-	-	-	-	+	-	-	+	-	-	-	-	-
3118	4718	hypothetical protein	4	-	-	-	-	-	-	-	-	-	-	-	-	-	-	-	-	-	-	-	-	-	-	-	-	-	-	-	-	-	-	-	+	+	-	-	-	+	-	-	-	-	-	-	-	-	-	-	-	-	+	-	-	-
3119	4719	phage protein	4	-	-	-	-	-	-	-	-	-	-	-	-	-	-	-	-	-	-	-	-	-	-	-	-	-	-	-	-	-	+	-	+	+	-	-	-	+	-	-	-	-	-	-	-	-	-	-	-	-	-	-	-	-
3120	4731	hypothetical protein	4	-	-	-	-	-	-	-	-	-	-	-	-	-	-	-	-	-	-	-	-	-	-	-	-	-	-	-	-	-	-	-	-	-	-	-	-	-	-	-	-	-	-	-	-	-	+	-	+	-	+	+	-	-
3121	4746	Phage protein	4	-	-	-	-	-	-	-	-	-	-	-	-	-	-	-	-	-	-	-	-	-	-	-	-	-	-	-	-	-	-	-	-	-	-	-	-	-	-	-	-	-	-	-	-	-	+	+	-	-	+	-	+	-
3122	4769	hypothetical protein	4	-	-	-	-	-	-	-	-	-	-	-	-	-	-	-	-	-	-	-	-	-	-	-	-	-	-	-	-	-	-	-	-	-	-	-	-	-	-	-	-	+	-	-	-	-	-	+	-	-	+	+	-	-
3123	4780	DNA-binding protein	4	-	-	-	-	-	-	-	-	-	-	-	-	-	-	-	-	-	-	-	-	-	-	-	-	-	-	-	-	-	-	-	+	+	+	-	-	-	-	-	-	-	-	-	-	-	-	-	-	-	-	+	-	-
3124	4782	hypothetical protein	4	-	-	-	-	-	-	-	-	-	-	-	-	-	-	-	-	-	-	-	-	-	-	-	-	-	-	-	-	-	-	-	+	+	+	-	-	-	-	+	-	-	-	-	-	-	-	-	-	-	-	-	-	-
3125	4799	prophage LambdaSa1, antirepressor, putative	4	-	-	-	-	-	-	-	-	-	-	-	-	-	-	-	-	-	-	-	-	-	-	-	-	-	-	-	-	-	-	-	-	-	-	-	-	-	-	-	-	-	-	-	-	-	-	-	+	+	-	+	+	-
3126	4801	Accessory secretory protein Asp2	4	-	-	-	-	-	-	-	-	-	-	-	-	-	-	-	-	-	-	-	-	-	-	-	-	-	-	-	-	-	-	-	-	-	-	-	-	-	-	-	-	-	-	-	-	-	-	+	-	+	+	+	-	-
3127	4802	ORF032	4	-	-	-	-	-	-	-	-	-	-	-	-	-	-	-	-	-	-	-	-	-	-	-	-	-	-	-	-	+	-	-	-	-	-	-	-	-	-	-	+	-	-	+	-	-	-	-	-	-	-	+	-	-
3128	4805	hypothetical protein	4	-	-	-	-	-	-	-	-	-	-	-	-	-	-	-	-	-	-	-	-	-	-	-	-	-	-	-	-	-	-	-	-	-	-	-	-	-	-	-	-	-	-	-	-	-	-	+	-	+	+	+	-	-
3129	4806	hypothetical protein	4	-	-	-	-	-	-	-	-	-	-	-	-	-	-	-	-	-	-	-	-	-	-	-	-	-	-	-	-	-	-	-	-	-	-	-	-	-	-	-	-	-	-	-	-	-	-	+	-	+	+	+	-	-
3130	4863	ABC transporter, ATP-binding/permease protein	4	+	+	+	-	-	-	-	-	-	-	-	-	-	-	-	-	-	-	-	-	-	-	-	-	-	-	-	-	-	-	-	-	-	-	-	-	-	-	-	-	-	-	-	-	+	-	-	-	-	-	-	-	-
3131	4866	lantibiotic mersacidin modifying enzyme	4	+	+	+	-	-	-	-	-	-	-	-	-	-	-	-	-	-	-	-	-	-	-	-	-	-	-	-	-	-	-	-	-	-	-	-	-	-	-	-	-	-	-	-	-	+	-	-	-	-	-	-	-	-
3132	4881	DNA primase/helicase, phage-associated	4	-	-	-	-	-	-	-	-	-	-	-	-	-	-	-	-	-	-	-	+	+	+	-	-	-	-	-	+	-	-	-	-	-	-	-	-	-	-	-	-	-	-	-	-	-	-	-	-	-	-	-	-	-
3133	4882	Hypothetical protein	4	-	-	-	-	-	-	-	-	-	-	-	-	-	-	-	-	-	-	-	+	+	+	-	-	-	-	-	+	-	-	-	-	-	-	-	-	-	-	-	-	-	-	-	-	-	-	-	-	-	-	-	-	-
3134	5131	Phage capsid protein	4	-	-	-	-	-	-	-	-	-	-	-	-	-	-	-	-	+	-	-	-	-	-	-	-	-	-	-	-	-	-	-	-	-	-	-	+	+	-	+	-	-	-	-	-	-	-	-	-	-	-	-	-	-	-	-
3135	5132	Orf46	4	-	-	-	-	-	-	-	-	-	-	-	-	-	-	-	-	+	-	-	-	-	-	-	-	-	-	-	-	-	-	-	-	-	-	-	+	+	-	+	-	-	-	-	-	-	-	-	-	-	-	-	-	-	-	-
3136	5134	hypothetical protein	4	-	-	-	-	-	-	-	-	-	-	-	-	-	-	-	+	+	+	-	-	-	-	-	-	-	-	-	-	-	-	-	-	-	-	-	-	-	-	-	-	-	+	-	-	-	-	-	-	-	-	-	-	-	-	-
3137	5135	putative transcriptional regulator	4	-	-	-	-	-	-	-	-	-	-	-	-	-	-	-	+	+	+	-	-	-	-	-	-	-	-	-	-	-	-	-	-	-	-	-	-	-	-	-	-	-	-	-	+	-	-	-	-	-	-	-	-	-
3138	5136	hypothetical protein	4	-	-	-	-	-	-	-	-	-	-	-	-	-	-	-	+	+	+	-	-	-	-	-	-	-	-	-	-	-	-	-	-	-	-	-	-	-	-	-	-	-	+	-	-	-	-	-	-	-	-	-	-	-	-	-
3139	5138	hypothetical protein	4	-	-	-	-	-	-	-	-	-	-	-	-	-	-	-	+	+	+	-	-	-	-	-	-	-	-	-	-	-	-	-	-	-	-	-	-	-	-	-	-	-	+	-	-	-	-	-	-	-	-	-	-	-	-	-
3140	5140	hypothetical protein	4	-	-	-	-	-	-	-	-	-	-	-	-	-	-	-	-	+	-	-	-	-	-	-	-	-	-	-	-	-	-	-	-	-	-	-	+	+	-	+	-	-	-	-	-	-	-	-	-	-	-	-	-	-	-	-
3141	5141	Abortive infection bacteriophage resistance protein	4	-	-	-	-	-	-	-	-	-	-	-	-	-	-	-	-	+	-	-	-	-	-	-	-	-	-	-	-	-	-	-	-	-	-	+	-	-	-	-	-	-	+	-	-	-	-	-	-	-	-	-	-	-	-	+	-
3142	5142	hypothetical protein	4	-	-	-	-	-	-	-	-	-	-	-	-	-	-	-	-	-	-	-	-	-	-	-	-	-	-	-	-	-	-	-	+	+	+	-	-	-	-	-	-	-	-	-	-	-	-	+	-	-	-	-	-	-
3143	5143	hypothetical protein	4	-	-	-	-	-	-	-	-	-	-	-	-	-	-	-	+	+	+	-	-	-	-	-	-	-	-	-	-	-	-	-	-	-	-	-	-	-	-	-	-	-	+	-	-	-	-	-	-	-	-	-	-	-	-	-
3144	5144	Membrane proteins related to metalloendopeptidases	4	-	-	-	-	-	-	-	-	-	-	-	-	-	-	-	-	+	-	-	-	-	-	-	-	-	-	-	-	-	-	-	-	-	-	-	+	+	-	+	-	-	-	-	-	-	-	-	-	-	-	-	-	-	-	-
3145	5147	Ribonucleases G and E	4	-	-	-	-	-	-	-	-	-	-	-	-	-	-	-	-	+	+	-	-	-	-	-	-	-	-	-	-	-	-	+	+	-	-	-	-	-	-	-	-	-	-	-	-	-	-	-	-	-	-	-	-	-	-	-
3146	5149	hypothetical protein	4	-	-	-	-	-	-	-	-	-	-	-	-	-	-	-	+	+	+	-	-	-	-	-	-	-	-	-	-	-	-	-	-	-	-	-	-	-	-	-	-	-	+	-	-	-	-	-	-	-	-	-	-	-	-	-
3147	5150	hypothetical protein	4	-	-	-	-	-	-	-	-	-	-	-	-	-	-	-	-	+	+	-	-	-	-	-	-	-	-	-	-	-	-	-	-	-	-	-	-	-	-	-	-	-	-	-	-	-	-	+	-	+	-	-	-	-
3148	5151	hypothetical protein	4	-	-	-	-	-	-	-	-	-	-	-	-	-	-	-	-	+	-	+	-	-	-	-	-	-	-	-	-	-	-	-	-	-	-	-	+	-	+	-	-	-	-	-	-	-	-	-	-	-	-	-	-	-	-	-
3149	5153	hypothetical protein	4	-	-	-	-	-	-	-	-	-	-	-	-	-	-	-	-	+	-	-	-	-	-	-	-	-	-	-	-	-	-	-	-	-	-	-	+	+	-	+	-	-	-	-	-	-	-	-	-	-	-	-	-	-	-	-
3150	5154	hypothetical protein	4	-	-	-	-	-	-	-	-	-	-	-	-	-	-	-	+	+	+	-	-	-	-	-	-	-	-	-	-	-	-	-	-	-	-	-	-	-	-	-	-	-	+	-	-	-	-	-	-	-	-	-	-	-	-	-
3151	5155	prophage pi2 protein 38	4	-	-	-	-	-	-	-	-	-	-	-	-	-	-	-	-	+	-	-	-	-	-	-	-	-	-	-	-	-	-	-	-	-	-	-	+	+	-	+	-	-	-	-	-	-	-	-	-	-	-	-	-	-	-	-
3152	5157	N-acetylmuramoyl-L-alanine amidase xlyB precursor (EC 3.5.1.28) (Cell wall hydrolase) (Autolysin)	4	-	-	-	-	-	-	-	-	-	-	-	-	-	-	-	-	+	-	-	-	-	-	-	-	-	-	-	-	-	-	-	-	-	-	-	-	-	-	-	-	-	-	-	-	-	-	-	-	-	-	+	-	-	-	-	+	+
3153	5158	hypothetical protein	4	-	-	-	-	-	-	-	-	-	-	-	-	-	-	-	-	+	+	-	-	-	-	-	-	-	-	-	-	-	-	-	-	-	-	-	-	-	-	+	-	-	-	-	-	-	-	-	-	-	+	-	-	-	-	-
3154	5159	phage replication initiation protein	4	-	-	-	-	-	-	-	-	-	-	-	-	-	-	-	-	-	-	-	-	-	-	-	-	+	-	-	-	-	-	-	-	+	-	+	-	+	-	-	-	-	-	-	-	-	-	-	-	-	-	-	-	-	-	-
3155	5160	hypothetical protein	4	-	-	-	-	-	-	-	-	-	-	-	-	-	-	-	-	-	-	-	-	-	-	-	-	+	-	-	-	-	-	-	-	-	+	-	-	+	+	-	-	-	-	-	-	-	-	-	-	-	-	-	-	-	-	-
3156	5163	Abortive infection bacteriophage resistance protein	4	-	-	-	-	-	-	-	-	-	-	-	-	-	-	-	-	-	-	-	-	-	-	-	-	+	-	-	-	-	-	-	-	-	-	+	-	-	-	-	-	-	-	-	-	-	-	-	-	-	+	-	-	-	-	+
3157	5164	Undecaprenyl-diphosphatase (EC 3.6.1.27)	4	-	-	-	-	-	-	-	-	-	-	-	-	-	-	-	-	-	-	-	-	-	-	-	-	+	-	-	-	-	+	+	-	-	-	-	-	-	-	-	-	-	-	-	-	-	-	-	-	-	-	+	-	-
3158	5167	Zinc metalloprotease zmpC precursor (EC 3.4.24.-) (MMP-9 protease)	4	-	-	-	-	-	-	-	-	-	-	-	-	-	-	-	-	-	-	-	-	-	-	-	+	+	+	-	-	-	-	-	-	-	-	-	-	-	-	-	-	-	-	-	-	+	-	-	-	-	-	-	-	-	-	-	-	-
3159	5169	hypothetical protein	4	-	-	-	-	-	-	-	-	-	-	-	-	-	-	-	-	-	-	-	-	-	-	-	-	+	+	-	-	-	-	-	-	-	-	-	-	-	-	-	-	-	+	+	-	-	-	-	-	-	-	-	-	-	-	-	-	-
3160	5176	hypothetical protein	4	-	-	-	-	-	-	-	-	-	-	-	-	-	-	-	-	-	-	-	-	-	-	-	-	-	-	-	-	-	-	-	-	-	-	-	+	+	-	-	-	-	-	-	+	-	-	-	-	-	-	-	+	-
3161	5180	hypothetical protein	4	-	-	-	-	-	-	-	-	-	-	-	-	-	-	-	-	-	-	-	-	-	-	-	-	-	-	-	-	-	-	-	-	-	-	-	+	-	-	-	-	+	-	-	-	-	-	-	+	-	-	-	+	-	-
3162	5182	hypothetical protein	4	-	-	-	-	-	-	-	-	-	-	-	-	-	-	-	-	-	-	-	-	-	-	-	-	-	-	-	-	-	-	-	+	+	-	+	+	-	-	-	-	-	-	-	-	-	-	-	-	-	-	-	-	-
3163	5183	hypothetical protein	4	-	-	-	-	-	-	-	-	-	-	-	-	-	-	-	-	-	-	-	-	-	-	-	-	-	-	-	-	-	-	-	-	-	-	+	-	-	-	-	-	-	-	-	-	-	+	-	-	+	-	+	-	-
3164	5185	hypothetical protein	4	-	-	-	-	-	-	-	-	-	-	-	-	-	-	-	-	-	-	-	-	-	-	-	-	-	-	-	-	-	-	-	-	-	-	+	-	-	-	-	-	-	-	+	-	-	-	-	+	-	-	+	-	-
3165	5186	hypothetical protein	4	-	-	-	-	-	-	-	-	-	-	-	-	-	-	-	-	-	-	-	-	-	-	-	-	-	-	-	-	-	-	-	-	-	-	+	+	-	-	-	+	-	-	-	-	+	-	-	-	-	-	-	-	-
3166	5187	hypothetical protein	4	-	-	-	-	-	-	-	-	-	-	-	-	-	-	-	-	-	-	-	-	-	-	-	-	-	-	-	-	-	-	-	-	-	-	+	+	-	-	-	-	-	-	+	-	-	-	-	+	-	-	-	-	-
3167	5188	hypothetical protein	4	-	-	-	-	-	-	-	-	-	-	-	-	-	-	-	-	-	-	+	-	-	-	-	-	-	-	-	-	-	-	-	-	-	-	-	-	+	-	-	-	-	-	-	-	-	-	-	-	+	-	+	-	-	-	-
3168	5189	hypothetical protein	4	-	-	-	-	-	-	-	-	-	-	-	-	-	-	-	-	-	-	-	+	-	-	-	-	-	-	-	-	-	-	-	-	+	-	-	-	+	-	-	-	-	-	-	-	-	-	-	-	-	-	-	+	-	-	-
3169	5190	hypothetical protein	4	-	-	-	-	-	-	-	-	-	-	-	-	-	-	-	-	-	-	-	-	-	-	-	-	-	-	-	-	-	-	-	+	+	-	-	-	-	-	-	-	-	-	-	-	-	-	+	-	+	-	-	-	-
3170	5192	hypothetical protein	4	-	-	-	-	-	-	-	-	-	-	-	-	-	-	-	-	-	-	-	-	-	-	-	-	-	-	-	-	-	-	-	-	-	-	+	+	-	-	-	-	-	-	+	-	-	-	-	+	-	-	-	-	-
3171	5193	hypothetical protein	4	-	-	-	-	-	-	-	-	-	-	-	-	-	-	-	-	-	-	-	-	-	-	-	-	-	-	-	-	-	-	-	-	-	-	+	-	-	+	-	-	-	-	-	-	-	-	+	+	-	-	-	-	-
3172	5194	hypothetical protein	4	-	-	-	-	-	-	-	-	-	-	-	-	-	-	-	-	-	-	-	-	-	-	-	-	-	-	-	-	-	-	-	-	-	-	+	-	-	-	-	-	-	-	+	-	-	-	-	+	-	-	+	-	-
3173	5195	hypothetical protein	4	-	-	-	-	-	-	-	-	-	-	-	-	-	-	-	-	-	-	-	+	-	-	-	-	-	-	-	-	-	-	-	-	+	-	-	-	+	-	-	-	-	-	-	-	-	-	-	-	-	-	-	+	-	-	-
3174	5198	putative protein	4	-	-	-	-	-	-	-	-	-	-	-	-	-	-	-	-	-	-	-	-	-	-	-	-	-	-	-	-	-	-	-	-	-	-	-	+	-	-	-	+	-	-	+	-	-	-	-	+	-	-	-	-	-
3175	5200	putative protein	4	-	-	-	-	-	-	-	-	-	-	-	-	-	-	-	-	-	-	-	-	-	-	-	-	-	-	-	-	-	-	-	-	-	-	-	+	-	-	-	+	-	-	+	-	-	-	-	+	-	-	-	-	-
3176	5201	hypothetical protein	4	-	-	-	-	-	-	-	-	-	-	-	-	-	-	-	-	-	-	-	-	-	-	-	-	-	-	-	-	-	-	-	-	-	-	-	-	-	+	-	-	-	+	+	-	+	-	-	-	-	-	-	-	-
3177	5202	putative transcriptional regulator	4	-	-	-	-	-	-	-	-	-	-	-	-	-	-	-	+	-	-	+	-	-	-	-	-	-	-	-	-	-	-	-	-	-	-	-	-	-	-	-	-	-	+	-	-	-	-	-	-	-	+	-	-	-
3178	5204	hypothetical protein	4	-	-	-	-	-	-	-	-	-	-	-	-	-	-	-	-	-	-	-	-	-	-	-	-	-	-	-	-	-	-	-	-	-	-	-	-	-	+	+	+	-	+	-	-	-	-	-	-	-	-	-	-	-
3179	5205	hypothetical protein	4	-	-	-	-	-	-	-	-	-	-	-	-	-	-	-	-	-	-	-	-	-	-	-	-	-	-	-	-	-	-	-	-	-	-	-	-	-	-	-	-	+	+	+	-	+	-	-	-	-	-	-	-	-
3180	5207	hypothetical protein	4	-	-	-	-	-	-	-	-	-	-	-	-	-	-	-	-	-	-	-	-	-	-	-	-	-	-	-	-	-	-	-	-	-	-	-	-	-	-	-	-	-	+	+	+	+	-	-	-	-	-	-	-	-
3181	5208	hypothetical protein	4	-	-	-	-	-	-	-	-	-	-	-	-	-	-	-	-	-	-	-	-	-	-	-	-	-	-	-	-	-	-	+	-	-	-	-	-	-	-	-	-	+	+	-	-	-	-	-	-	-	-	+	-	-
3182	5209	hypothetical protein	4	-	-	-	-	-	-	-	-	-	-	-	-	-	-	-	-	-	-	-	-	-	-	-	-	-	-	-	-	-	-	-	-	-	-	-	-	-	-	-	-	+	+	+	-	+	-	-	-	-	-	-	-	-
3183	5210	hypothetical protein	4	-	-	-	-	-	-	-	-	-	-	-	-	-	-	-	-	-	-	-	-	-	-	-	-	-	-	-	-	-	-	-	+	+	+	-	-	-	-	-	-	-	+	-	-	-	-	-	-	-	-	-	-	-
3184	5212	hypothetical protein	4	-	-	-	-	-	-	-	-	-	-	-	-	-	-	-	-	-	-	-	-	-	-	+	-	-	-	-	-	-	-	-	-	-	-	-	+	+	+	-	-	-	-	-	-	-	-	-	-	-	-	-	-	-	-	-
3185	5214	hypothetical protein	4	-	-	-	-	-	-	-	-	-	-	-	-	-	-	-	-	-	-	-	-	-	-	+	-	-	-	-	-	-	-	-	-	-	-	-	+	+	+	-	-	-	-	-	-	-	-	-	-	-	-	-	-	-	-	-
3186	5215	calcium-binding protein	4	-	-	-	-	-	-	-	-	-	-	-	-	-	-	-	-	-	-	-	-	-	-	+	-	-	-	-	-	-	-	-	-	-	-	-	+	+	+	-	-	-	-	-	-	-	-	-	-	-	-	-	-	-	-	-
3187	5217	Sucrose operon repressor ScrR, LacI family	4	-	-	-	-	-	-	-	-	-	-	-	-	-	-	-	-	-	-	-	-	-	-	+	+	-	-	-	-	-	-	-	-	-	-	-	-	-	-	-	-	-	-	-	-	-	-	-	-	+	-	+	-	-
3188	5218	hypothetical protein	4	-	-	-	-	-	-	-	-	-	-	-	-	-	-	-	-	-	-	-	-	-	-	+	-	-	-	-	-	-	-	+	-	-	-	+	+	-	-	-	-	-	-	-	-	-	-	-	-	-	-	-	-	-
3189	5219	hypothetical protein	4	-	-	-	-	-	-	-	-	-	-	-	-	-	-	-	-	-	-	-	-	-	-	+	-	-	-	-	-	-	-	-	-	-	-	-	+	+	+	-	-	-	-	-	-	-	-	-	-	-	-	-	-	-	-	-
3190	5220	hypothetical protein	4	-	-	-	-	-	-	-	-	-	-	-	-	-	-	-	-	-	-	-	-	-	-	+	-	-	-	-	-	-	-	-	-	-	-	-	+	+	+	-	-	-	-	-	-	-	-	-	-	-	-	-	-	-	-	-
3191	5223	protease	4	-	-	-	-	-	-	-	-	-	-	-	-	-	-	-	-	-	-	-	-	-	-	+	-	-	-	-	-	-	-	-	-	-	-	-	+	+	+	-	-	-	-	-	-	-	-	-	-	-	-	-	-	-	-	-
3192	5227	hypothetical protein	4	-	-	-	-	-	-	-	-	-	-	-	-	-	-	-	+	-	-	-	-	-	-	-	-	-	-	-	-	+	+	-	-	-	-	-	-	-	-	-	-	-	-	+	-	-	-	-	-	-	-	-	-	-	-	-	-	-
3193	5235	hypothetical protein	4	-	-	-	-	-	-	-	-	-	-	-	-	-	-	-	-	-	-	-	-	-	-	-	-	-	-	-	-	-	-	-	-	-	-	-	-	-	-	-	-	+	-	+	+	-	-	-	-	-	-	-	-	+
3194	5237	hypothetical protein	4	-	-	-	-	-	-	-	-	-	-	-	-	-	-	-	-	-	-	-	-	-	-	-	-	-	-	-	-	-	-	-	-	-	-	-	-	+	-	-	-	-	-	-	-	-	+	-	-	-	-	-	+	+
3195	5238	hypothetical protein	4	-	-	-	-	-	-	-	-	-	-	-	-	-	-	-	-	-	-	-	-	-	-	-	-	-	-	-	-	-	-	-	-	-	-	-	-	-	-	-	-	-	-	-	-	-	-	+	-	+	-	+	-	+
3196	5242	Methyl-accepting chemotaxis protein	4	-	-	-	-	-	-	-	-	-	-	-	-	-	-	-	-	-	-	-	-	-	-	-	-	-	-	-	-	-	-	-	-	-	-	-	-	-	-	-	-	-	-	-	-	-	-	-	-	-	+	+	+	+
3197	5251	hypothetical protein	4	-	-	-	-	-	-	-	-	-	-	-	-	-	-	-	-	-	-	-	+	+	+	-	-	-	-	-	-	-	-	-	-	-	-	-	-	-	-	-	-	-	-	-	+	-	-	-	-	-	-	-	-	-
3198	5252	hypothetical protein	4	-	-	-	-	-	-	-	-	-	-	-	-	-	-	-	-	-	-	-	-	-	-	-	-	-	-	+	+	-	-	-	-	-	-	-	-	-	-	-	-	-	-	-	+	-	-	-	-	-	-	-	-	+
3199	5255	hypothetical protein	4	-	-	-	-	-	-	-	-	-	-	-	-	-	-	-	-	-	-	-	-	-	-	-	-	-	-	-	-	-	-	-	-	-	+	-	-	-	-	-	-	-	-	-	+	-	+	-	+	-	-	-	-	-
3200	5256	hypothetical protein	4	-	-	-	-	-	-	-	-	-	-	-	-	-	-	-	-	-	-	-	-	-	-	-	-	-	-	-	-	-	-	-	-	-	-	-	-	-	-	-	+	-	-	-	+	+	-	-	-	-	-	+	-	-
3201	5262	probable glycine hydroxymethyltransferase	4	-	-	-	-	-	-	-	-	-	-	-	-	-	-	-	-	-	-	-	-	-	-	-	-	-	-	-	-	-	-	-	-	-	+	-	-	-	-	-	-	-	-	-	+	-	+	-	+	-	-	-	-	-
3202	5263	hypothetical protein	4	-	-	-	-	-	-	-	-	-	-	-	-	-	-	-	-	-	-	-	-	-	-	-	-	-	-	-	-	-	-	-	-	-	-	-	-	-	-	-	-	-	-	+	+	+	+	-	-	-	-	-	-	-
3203	5264	hypothetical protein	4	-	-	-	-	-	-	+	-	+	-	-	-	-	-	-	-	-	-	-	-	-	-	-	-	-	-	-	-	-	-	-	-	-	+	-	-	-	-	-	-	-	-	-	+	-	-	-	-	-	-	-	-	-
3204	5266	hypothetical protein	4	-	-	-	-	-	-	-	-	-	-	-	-	-	-	-	-	-	-	-	-	-	-	-	-	-	-	-	-	-	-	-	-	-	-	-	-	-	-	+	-	-	-	-	+	-	+	-	-	-	-	-	+	-
3205	5267	Beta-1,3-glucosyltransferase	4	-	-	-	-	-	-	-	-	-	-	-	-	-	-	-	-	-	-	-	-	-	-	-	-	-	-	-	-	-	-	-	-	-	-	-	-	+	+	-	-	-	-	-	+	-	-	-	-	+	-	-	-	-
3206	5271	hypothetical protein	4	-	-	-	-	-	-	-	-	-	-	-	-	-	-	-	-	-	-	-	-	-	-	-	-	-	-	+	+	-	-	-	-	-	-	-	-	+	+	-	-	-	-	-	-	-	-	-	-	-	-	-	-	-
3207	5272	hypothetical protein	4	-	-	-	-	-	-	-	-	-	-	-	-	-	-	-	-	-	-	-	-	-	-	-	-	-	-	-	-	-	-	-	-	-	-	-	-	-	+	+	-	-	-	-	-	-	+	-	+	-	-	-	-	-
3208	5274	Phage transcriptional activator RinA	4	-	-	-	-	-	-	-	-	-	-	-	-	-	-	-	-	-	-	-	+	-	-	-	-	-	-	-	-	-	-	-	-	-	-	-	-	-	+	-	-	-	-	-	-	-	-	-	-	+	+	-	-	-
3209	5278	hypothetical protein	4	-	-	-	-	-	-	-	-	-	-	-	-	-	-	-	-	-	-	-	-	-	-	-	-	-	-	-	-	-	-	-	+	-	-	-	-	-	+	-	-	-	-	-	-	-	-	+	-	+	-	-	-	-
3210	5284	hypothetical protein	4	-	-	-	-	-	-	-	-	-	-	-	-	-	-	+	-	-	-	+	-	-	-	-	-	-	-	-	-	-	-	-	-	-	-	-	-	+	-	-	+	-	-	-	-	-	-	-	-	-	-	-	-	-
3211	5291	hypothetical protein	4	-	-	-	-	-	-	-	-	-	-	-	-	-	-	-	-	-	-	-	-	-	-	-	-	-	-	+	+	+	-	-	-	-	-	-	-	-	+	-	-	-	-	-	-	-	-	-	-	-	-	-	-	-
3212	5298	hypothetical protein	4	-	-	-	-	-	-	-	-	-	-	-	-	-	-	-	-	-	-	-	-	-	-	-	-	-	-	-	-	-	-	-	-	-	+	-	-	-	-	-	-	+	-	-	-	+	-	-	+	-	-	-	-	-
3213	5310	Mobile element protein	4	-	-	-	-	-	-	-	-	-	-	-	-	-	-	-	-	-	-	-	-	-	-	-	-	-	-	-	-	-	-	-	-	-	-	-	-	-	-	-	-	-	-	-	-	-	+	+	-	+	-	+	-	-
3214	5315	hypothetical protein	4	-	-	-	-	-	-	-	-	-	-	-	-	-	-	-	-	-	-	-	-	-	-	-	-	-	-	-	-	-	-	-	-	-	-	-	-	-	-	-	-	-	-	-	-	-	+	+	+	+	-	-	-	-
3215	5324	hypothetical protein	4	-	-	-	-	-	-	-	-	-	-	-	-	-	-	-	-	-	-	-	-	-	-	-	-	-	-	-	-	-	-	-	-	-	-	-	-	-	-	-	+	-	-	+	-	+	+	-	-	-	-	-	-	-
3216	5331	Glycosyl transferase family protein	4	-	-	-	-	-	-	-	-	-	-	-	-	-	-	-	-	-	-	-	-	-	-	-	-	-	-	-	-	-	-	-	-	-	-	-	-	-	-	-	-	-	-	-	-	-	+	+	-	+	-	+	-	-
3217	5332	hypothetical protein	4	-	-	-	-	-	-	-	-	-	-	-	-	-	-	-	-	-	-	+	-	-	-	-	-	-	-	-	-	-	-	-	-	-	-	-	-	-	-	-	+	-	-	-	-	-	+	-	+	-	-	-	-	-
3218	5342	hypothetical protein	4	-	-	-	-	-	-	-	-	-	-	-	-	-	-	-	-	-	-	-	-	-	-	-	-	-	-	-	-	-	-	+	+	+	-	-	-	-	-	-	-	-	-	-	-	-	-	-	-	-	-	+	-	-
3219	5347	HNH endonuclease	4	-	-	-	-	-	-	-	-	+	-	-	-	-	-	-	-	-	-	-	-	-	-	-	-	-	-	-	-	-	-	-	-	-	-	-	-	-	-	-	-	+	-	-	-	-	+	-	-	-	+	-	-	-
3220	5351	Abortive infection protein	4	-	-	-	-	-	-	-	-	-	-	-	-	-	-	-	-	-	-	-	-	-	-	-	-	-	-	-	-	-	-	-	-	-	-	-	-	-	-	-	-	-	-	-	-	-	+	+	+	+	-	-	-	-
3221	5357	hypothetical protein	4	-	-	-	-	-	-	-	-	-	-	-	-	-	-	-	-	-	-	-	-	-	-	-	-	-	-	-	-	-	-	-	-	-	-	-	-	-	-	-	-	-	-	-	-	-	+	-	+	+	+	-	-	-
3222	5360	hypothetical protein	4	-	-	-	-	-	-	-	-	-	-	-	-	-	-	-	-	-	-	-	-	-	-	-	-	-	-	-	-	-	-	-	-	-	-	-	-	-	-	-	-	-	-	-	-	-	+	+	+	-	-	+	-	-
3223	5364	UDP-glucose 4-epimerase (EC 5.1.3.2)	4	-	-	+	-	-	+	-	-	-	-	-	+	+	-	-	-	-	-	-	-	-	-	-	-	-	-	-	-	-	-	-	-	-	-	-	-	-	-	-	-	-	-	-	-	-	-	-	-	-	-	-	-	-
3224	5365	YSIRK Gram-positive signal peptide	4	-	-	-	-	-	-	+	+	-	-	-	-	+	-	-	-	-	-	-	-	-	-	-	-	-	-	-	-	-	-	-	-	-	-	-	-	-	-	-	-	-	-	-	-	+	-	-	-	-	-	-	-	-
3225	5377	hypothetical protein	4	-	-	-	-	-	-	-	-	-	-	-	-	-	-	-	-	-	-	-	-	-	-	-	-	-	-	+	+	-	-	-	-	-	-	-	-	-	-	-	+	+	-	-	-	-	-	-	-	-	-	-	-	-
3226	5388	hypothetical protein	4	-	-	-	-	-	-	-	-	-	-	-	-	-	-	-	-	-	-	-	-	-	-	-	-	-	-	-	-	+	+	+	-	-	-	-	-	-	-	-	-	-	-	-	-	-	-	-	-	-	+	-	-	-
3227	5391	hypothetical protein	4	-	-	-	-	-	-	-	-	-	-	-	-	-	-	-	-	-	-	-	-	-	-	-	-	-	-	-	-	-	-	-	-	-	-	-	-	-	-	-	-	-	-	-	-	-	-	-	+	+	+	+	-	-
3228	5392	hypothetical protein	4	-	-	-	-	-	-	-	-	-	-	-	-	-	-	-	-	-	-	-	-	-	-	-	-	-	-	-	-	-	-	-	+	+	-	-	-	-	-	-	-	-	-	-	-	-	-	+	-	+	-	-	-	-

Table S5. Precence/absence of each HG among 57 *S. suis* strains

				1																13			25					28						104		94			76			53/54		Divergent *S. suis*																
Index[a]	ClusterID	Gene products	Members	98HAH33	05ZYH33	SC84	A7	SS12	P1/7	DAT273	NIAH11434	JS14	GZ1	BM407	S735	13730	MNCM07	NCTC 10446	14636	NCTC 10237	ST1	2651	89-1591	MNCM04	MNCM25	8074	D9	4961	ST3	MNCM43	DAT292	DAT260	NIAH11435	MNCM21	MNCM50	89-2479	DAT234	DAT290	93A	42A	NT77	6407	11538	8830	12814	92-1400	89-590	2726	2524	89-3576-3	4417	D12	10581	89-5259	88-5299A	R61	92-1191	14A	92-4172	22083
3229	5394	Transposase and inactivated derivative	4	-	-	-	-	-	-	-	-	-	-	-	-	-	-	-	-	-	-	-	-	-	-	-	-	-	-	-	-	-	-	-	-	-	-	-	+	+	+	-	-	-	-	-	-	-	-	-	-	-	-	-	-	-	-	+	-	-
3230	5398	hypothetical protein	4	-	-	-	-	-	-	-	-	+	-	-	-	-	-	-	-	-	-	-	-	-	-	-	-	-	-	-	-	-	-	-	-	-	-	-	+	+	-	-	-	-	-	-	-	-	-	-	-	-	-	-	-	-	+	-	-	-
3231	5402	hypothetical protein	4	-	-	-	-	-	-	-	-	-	-	-	-	-	-	-	-	-	-	-	-	-	-	-	-	-	-	-	-	-	-	+	+	-	-	-	+	+	-	-	-	-	-	-	-	-	-	-	-	-	-	-	-	-	-	-	-	-
3232	5403	hypothetical protein	4	-	-	-	-	-	-	-	-	-	-	-	-	-	-	-	-	-	-	-	-	-	-	-	-	-	-	-	-	-	-	+	+	-	-	-	+	+	-	-	-	-	-	-	-	-	-	-	-	-	-	-	-	-	-	-	-	-
3233	5407	hypothetical protein	4	-	-	-	-	-	-	-	-	-	-	-	-	-	-	-	-	-	-	-	-	-	-	-	-	-	-	-	-	-	-	-	-	-	-	-	+	-	+	-	-	-	-	-	-	-	-	-	-	-	-	+	-	+	-	-	-	-
3234	5408	hypothetical protein	4	-	-	-	-	-	-	-	-	-	-	-	-	-	-	-	-	-	-	-	-	-	-	-	-	-	-	-	-	-	-	+	+	-	-	-	+	+	-	-	-	-	-	-	-	-	-	-	-	-	-	-	-	-	-	-	-	-
3235	5409	hypothetical protein	4	-	-	-	-	-	-	-	-	-	-	-	-	-	-	-	-	-	-	-	-	-	-	-	-	-	-	-	-	-	-	+	+	-	-	-	+	+	-	-	-	-	-	-	-	-	-	-	-	-	-	-	-	-	-	-	-	-
3236	5410	putative helicase	4	-	-	-	-	-	-	-	-	-	-	-	-	-	-	-	-	-	-	-	-	-	-	-	-	-	-	-	-	-	-	-	-	-	+	+	+	-	+	-	-	-	-	-	-	-	-	-	-	-	-	-	-	-	-	-	-	-
3237	5411	Mobile element protein	4	-	-	-	-	-	-	-	-	-	-	-	-	-	-	-	-	-	-	-	-	-	-	-	-	-	-	-	-	-	-	-	-	-	-	-	+	+	+	-	-	-	-	-	-	-	-	-	-	-	-	-	-	-	-	+	-	-
3238	5412	hypothetical protein	4	-	-	-	-	-	-	-	-	-	-	-	-	-	-	-	-	-	-	-	-	-	-	-	-	-	-	-	-	-	-	-	-	-	-	-	+	+	-	-	-	-	-	-	-	-	-	-	-	-	-	+	-	+	-	-	-	-
3239	5417	hypothetical protein	4	-	-	-	-	-	-	-	-	-	-	-	-	-	-	-	-	-	-	-	-	-	-	-	-	-	-	-	-	-	-	+	+	-	-	-	+	+	-	-	-	-	-	-	-	-	-	-	-	-	-	-	-	-	-	-	-	-
3240	5420	hypothetical protein	4	-	-	-	-	-	-	-	-	-	-	-	-	-	-	-	-	-	-	-	-	-	-	-	-	-	-	-	-	-	-	-	-	+	+	+	-	-	+	-	-	-	-	-	-	-	-	-	-	-	-	-	-	-	-	-	-	-
3241	5421	hypothetical protein	4	-	-	-	-	-	-	-	-	-	-	-	-	-	-	-	-	-	-	-	-	-	-	-	-	-	-	-	-	-	-	-	-	-	-	-	-	+	+	-	-	-	-	-	-	-	-	-	-	-	-	-	-	+	-	-	+	-
3242	5422	hypothetical protein	4	-	-	-	-	-	-	-	-	-	-	-	-	-	-	-	-	-	-	-	-	-	-	-	-	-	-	-	-	-	-	-	-	-	-	-	-	+	-	-	-	-	-	-	-	-	-	-	-	-	-	+	+	-	+	-	-	-
3243	5443	preprotein translocase SecY family protein	4	-	-	-	-	-	-	-	-	-	-	-	-	-	-	-	-	-	-	-	-	-	-	-	-	-	-	-	-	-	-	-	-	-	-	-	-	-	-	-	-	-	-	-	-	-	-	-	-	-	-	+	-	+	+	+	-	-
3244	5445	Accessory secretory protein Asp3	4	-	-	-	-	-	-	-	-	-	-	-	-	-	-	-	-	-	-	-	-	-	-	-	-	-	-	-	-	-	-	-	-	-	-	-	-	-	-	-	-	-	-	-	-	-	-	-	-	-	-	+	-	+	+	+	-	-
3245	5446	Acetyltransferase(EC:2.3.1.-)	4	-	-	-	-	-	-	-	-	-	-	-	-	-	-	-	-	-	-	-	-	-	-	-	-	-	-	-	-	-	-	-	-	-	-	-	-	-	-	-	-	-	-	-	-	-	-	-	-	-	-	-	-	+	+	+	+	-
3246	5447	hypothetical protein	4	-	-	-	-	-	-	-	-	-	-	-	-	-	-	-	-	-	-	-	-	-	-	-	-	-	-	-	-	-	-	-	-	-	-	-	-	-	-	-	-	-	-	-	-	-	-	-	-	-	-	+	-	+	-	+	+	-
3247	5450	Histone acetyltransferase HPA2 and related acetyltransferases	4	-	-	-	-	-	-	-	-	-	-	-	-	-	-	-	-	-	-	-	-	-	-	-	-	-	-	-	-	-	-	-	-	-	-	-	-	-	-	-	-	-	-	-	-	-	-	-	-	-	-	-	-	+	+	+	+	-
3248	5471	hypothetical protein	4	-	-	-	-	-	-	-	-	-	-	-	-	-	-	-	-	-	-	-	-	-	-	-	-	-	-	-	-	-	-	-	-	-	-	-	-	-	-	-	-	-	-	-	-	-	-	-	-	-	-	+	+	+	+	-	-	-
3249	5491	Phosphatidylinositol-specific phospholipase C (EC 4.6.1.13)	4	-	-	-	-	-	-	-	-	-	-	-	-	-	-	-	-	-	-	-	-	-	-	-	-	-	-	-	-	-	-	-	-	-	-	-	-	-	-	-	-	+	-	-	-	-	-	-	-	+	-	+	-	-	+	-
3250	5498	Sialic acid utilization regulator, RpiR family	4	-	-	-	+	-	-	-	-	-	-	-	-	-	-	-	-	-	-	-	-	-	-	-	-	-	-	-	-	-	-	-	-	-	-	-	-	-	-	-	-	-	-	+	-	-	-	-	-	-	-	-	-	+	+	-	-	-
3251	5500	hypothetical protein	4	-	-	-	+	-	-	-	-	-	-	-	-	-	-	-	-	-	-	-	-	-	-	-	-	-	-	-	-	-	-	-	-	-	-	-	-	-	-	-	-	-	-	+	-	-	-	-	-	-	-	-	-	+	+	-	-	-
3252	5576	Ribonucleases G and E	4	-	-	-	-	-	-	-	-	-	-	-	-	-	-	-	-	-	-	+	+	+	+	-	-	-	-	-	-	-	-	-	-	-	-	-	-	-	-	-	-	-	-	-	-	-	-	-	-	-	-	-	-	-	-	-	-	-
3253	5577	FIG021300: Phage-associated protein	4	-	-	-	-	-	-	-	-	-	-	-	-	-	-	-	-	-	-	-	+	-	-	-	-	-	-	-	-	-	-	+	+	-	+	-	-	-	-	-	-	-	-	-	-	-	-	-	-	-	-	-	-	-	-	-	-	-
3254	5578	hypothetical protein	4	-	-	-	-	-	-	-	-	-	-	-	-	-	-	-	-	-	-	-	+	-	-	-	-	-	-	-	-	-	-	+	+	-	+	-	-	-	-	-	-	-	-	-	-	-	-	-	-	-	-	-	-	-	-	-	-	-
3255	5580	FIG021300: Phage-associated protein	4	-	-	-	-	-	-	-	-	-	-	-	-	-	-	-	-	-	-	-	-	-	-	-	-	-	-	+	+	+	+	-	-	-	-	-	-	-	-	-	-	-	-	-	-	-	-	-	-	-	-	-	-	-	-	-	-	-
3256	5581	hypothetical protein	4	-	-	-	-	-	-	-	-	-	-	-	-	-	-	-	-	-	-	-	-	-	-	-	-	-	-	+	+	+	+	-	-	-	-	-	-	-	-	-	-	-	-	-	-	-	-	-	-	-	-	-	-	-	-	-	-	-
3257	5583	hypothetical protein	4	-	-	-	-	-	-	-	-	-	-	-	-	-	-	-	-	-	-	-	+	+	+	-	-	-	-	-	+	-	-	-	-	-	-	-	-	-	-	-	-	-	-	-	-	-	-	-	-	-	-	-	-	-	-	-	-	-
3258	5930	hypothetical protein	4	+	+	+	-	-	-	-	-	-	-	+	-	-	-	-	-	-	-	-	-	-	-	-	-	-	-	-	-	-	-	-	-	-	-	-	-	-	-	-	-	-	-	-	-	-	-	-	-	-	-	-	-	-	-	-	-	-
3259	5962	hypothetical protein	4	+	+	+	-	-	-	-	-	-	-	+	-	-	-	-	-	-	-	-	-	-	-	-	-	-	-	-	-	-	-	-	-	-	-	-	-	-	-	-	-	-	-	-	-	-	-	-	-	-	-	-	-	-	-	-	-	-
3260	5963	hypothetical protein	4	+	+	+	-	-	-	-	-	-	-	+	-	-	-	-	-	-	-	-	-	-	-	-	-	-	-	-	-	-	-	-	-	-	-	-	-	-	-	-	-	-	-	-	-	-	-	-	-	-	-	-	-	-	-	-	-	-
3261	5964	ABC transporter, NBP/MSD fusion protein	4	+	+	+	-	-	-	-	-	-	-	+	-	-	-	-	-	-	-	-	-	-	-	-	-	-	-	-	-	-	-	-	-	-	-	-	-	-	-	-	-	-	-	-	-	-	-	-	-	-	-	-	-	-	-	-	-	-
3262	5965	Sensor-receptor histidine kinase NisK	4	+	+	+	-	-	-	-	-	-	-	+	-	-	-	-	-	-	-	-	-	-	-	-	-	-	-	-	-	-	-	-	-	-	-	-	-	-	-	-	-	-	-	-	-	-	-	-	-	-	-	-	-	-	-	-	-	-
3263	5966	transposase (24)	4	+	+	+	-	-	-	-	-	-	-	-	-	-	-	-	-	-	-	-	-	-	-	-	-	-	-	-	-	-	-	-	-	-	-	-	-	-	-	-	-	-	-	-	-	-	-	-	-	-	-	-	-	+	-	-	-	-
3264	5967	columbicin A	4	+	+	+	-	-	-	-	-	-	-	-	-	-	-	-	-	-	-	-	-	-	-	-	-	-	-	-	-	-	-	-	-	-	-	-	-	-	-	-	-	-	-	-	-	-	-	-	-	+	-	-	-	-	-	-	-	-
3265	1118	Transposase	3	-	-	-	-	-	-	-	-	-	-	-	-	-	-	-	-	-	-	-	-	-	-	-	-	-	-	+	-	-	-	-	-	-	-	-	-	-	-	-	-	-	-	-	+	-	-	-	-	-	-	-	-	-	-	-	+	-
3266	1934	Alpha-L-Rha alpha-1,2-L-rhamnosyltransferase/alpha-L-Rha alpha-1,3-L- rhamnosyltransferase (EC 2.4.1.-)	3	-	-	-	-	-	-	-	-	-	-	-	-	-	-	-	-	-	-	-	-	-	-	-	-	-	-	-	-	-	-	-	-	-	-	-	-	-	-	-	-	-	+	-	-	-	-	-	-	-	-	+	-	+	-	-	-	-
3267	2025	oxidoreductase, aldo/keto reductase family	3	-	-	-	-	-	-	-	-	-	-	-	-	-	-	-	-	-	-	-	-	-	-	-	-	-	-	-	-	-	-	+	+	-	-	-	-	-	-	-	-	-	-	-	-	-	-	-	-	-	-	-	-	-	+	-	-	-
3268	2063	ABC transporter, predicted N-acetylneuraminate-binding protein	3	-	-	-	-	-	-	-	-	-	-	-	-	-	-	-	-	-	-	-	-	-	-	-	-	-	-	-	-	-	-	-	-	-	-	-	-	-	-	-	-	-	-	-	-	-	-	-	-	-	+	-	+	-	-	+	-	-
3269	2064	ABC transporter, predicted N-acetylneuraminate transport system permease protein 1	3	-	-	-	-	-	-	-	-	-	-	-	-	-	-	-	-	-	-	-	-	-	-	-	-	-	-	-	-	-	-	-	-	-	-	-	-	-	-	-	-	-	-	-	-	-	-	-	-	-	+	-	+	-	-	+	-	-
3270	2088	ABC transporter, predicted N-acetylneuraminate transport system permease protein 2	3	-	-	-	-	-	-	-	-	-	-	-	-	-	-	-	-	-	-	-	-	-	-	-	-	-	-	-	-	-	-	-	-	-	-	-	-	-	-	-	-	-	-	-	-	-	-	-	-	-	+	-	+	-	-	+	-	-
3271	2401	hypothetical protein	3	-	-	-	-	-	-	-	-	-	-	-	-	-	-	-	-	-	-	-	-	-	-	-	-	-	-	-	-	-	-	-	-	-	-	-	-	-	-	-	-	+	+	-	-	-	-	-	-	+	-	-	-	-	-	-	-	-
3272	2574	CRISPR-associated protein, CT1133 family	3	-	-	-	-	-	-	-	-	-	-	-	-	-	-	+	-	-	-	-	-	-	-	-	-	-	-	-	-	-	-	+	+	-	-	-	-	-	-	-	-	-	-	-	-	-	-	-	-	-	-	-	-	-	-	-	-	-
3273	2575	CRISPR-associated protein, CT1134 family	3	-	-	-	-	-	-	-	-	-	-	-	-	-	-	+	-	-	-	-	-	-	-	-	-	-	-	-	-	-	-	+	+	-	-	-	-	-	-	-	-	-	-	-	-	-	-	-	-	-	-	-	-	-	-	-	-	-
3274	2576	CRISPR-associated helicase Cas3	3	-	-	-	-	-	-	-	-	-	-	-	-	-	-	+	-	-	-	-	-	-	-	-	-	-	-	-	-	-	-	+	+	-	-	-	-	-	-	-	-	-	-	-	-	-	-	-	-	-	-	-	-	-	-	-	-	-
3275	2647	Cu+ P-type ATPase	3	-	-	-	-	-	-	-	-	-	-	-	-	-	-	-	-	-	-	-	-	-	-	-	-	-	-	-	-	-	-	-	-	-	-	-	-	-	-	-	-	-	-	+	-	-	-	-	+	-	-	+	-	-	-	-	-	-
3276	2667	CRISPR-associated protein, Csd2 family	3	-	-	-	-	-	-	-	-	-	-	-	-	-	-	+	-	-	-	-	-	-	-	-	-	-	-	-	-	-	-	+	+	-	-	-	-	-	-	-	-	-	-	-	-	-	-	-	-	-	-	-	-	-	-	-	-	-
3277	2668	CRISPR-associated RecB family exonuclease Cas4b	3	-	-	-	-	-	-	-	-	-	-	-	-	-	-	+	-	-	-	-	-	-	-	-	-	-	-	-	-	-	-	+	+	-	-	-	-	-	-	-	-	-	-	-	-	-	-	-	-	-	-	-	-	-	-	-	-	-
3278	2681	DNA primase, phage associated	3	-	-	-	-	-	-	-	-	-	-	-	-	-	-	-	-	-	-	-	-	-	-	-	-	-	-	-	-	-	-	-	-	-	-	-	-	-	-	-	-	-	-	+	+	-	-	-	-	-	-	-	-	+	-	-	-	-
3279	2769	Negative transcriptional regulator-copper transport operon	3	-	-	-	-	-	-	-	-	-	-	-	-	-	-	-	-	-	-	-	-	-	-	-	-	-	-	-	-	-	-	-	-	-	-	-	-	-	-	-	-	-	-	+	-	-	-	-	+	-	-	+	-	-	-	-	-	-
3280	2789	CRISPR-associated protein Cas2	3	-	-	-	-	-	-	-	-	-	-	-	-	-	-	+	-	-	-	-	-	-	-	-	-	-	-	-	-	-	-	+	+	-	-	-	-	-	-	-	-	-	-	-	-	-	-	-	-	-	-	-	-	-	-	-	-	-
3281	2797	putative role in replication	3	-	-	-	-	-	-	-	-	-	-	-	-	-	-	-	-	-	-	-	-	-	-	-	-	-	-	-	-	-	-	-	-	-	-	-	+	+	+	-	-	-	-	-	-	-	-	-	-	-	-	-	-	-	-	-	-	-
3282	2906	Bacteriocin ABC-type exporter, ATP binding/permease protein, putative	3	-	-	-	-	-	-	-	-	-	-	-	-	-	-	-	-	-	-	-	-	-	-	-	-	-	-	-	-	-	-	-	-	-	-	-	-	-	-	-	-	-	-	-	-	-	-	-	-	-	+	-	-	+	-	+	-	-
3283	2915	CRISPR-associated protein Cas1	3	-	-	-	-	-	-	-	-	-	-	-	-	-	-	+	-	-	-	-	-	-	-	-	-	-	-	-	-	-	-	+	+	-	-	-	-	-	-	-	-	-	-	-	-	-	-	-	-	-	-	-	-	-	-	-	-	-
3284	3048	putative plasmid partition protein	3	-	-	-	-	-	-	-	-	-	-	-	-	-	-	-	-	-	-	-	-	-	-	-	-	-	-	-	-	-	-	-	-	-	-	-	-	-	-	-	-	-	-	-	+	-	-	-	-	-	-	-	-	-	-	+	+	-
3285	3097	Purine nucleoside phosphorylase (EC 2.4.2.1)	3	-	-	-	-	-	-	-	-	-	-	-	-	-	-	-	-	+	+	-	-	-	-	-	-	-	-	-	-	-	-	-	-	-	-	-	-	-	-	-	-	-	-	-	-	-	-	-	-	-	+	-	-	-	-	-	-	-
3286	3125	Mobile element protein	3	-	-	-	-	-	-	-	-	-	-	-	-	-	-	-	-	-	-	-	+	-	-	-	-	-	-	-	-	-	-	-	-	-	-	-	-	-	-	-	+	-	-	-	-	-	-	-	-	-	-	-	-	-	-	+	-	-
3287	3145	hypothetical protein	3	-	-	-	-	-	-	-	-	-	-	-	-	-	-	-	-	-	-	-	-	-	-	-	-	-	-	-	-	-	-	-	-	-	-	-	-	-	-	-	-	-	-	-	-	-	-	-	+	-	-	-	+	+	-	-	-	-
3288	3269	immunity protein, probable	3	-	-	-	-	-	-	-	-	-	-	-	-	-	-	-	-	-	-	-	-	-	-	+	-	-	-	-	-	-	-	-	-	-	-	-	-	-	-	+	+	-	-	-	-	-	-	-	-	-	-	-	-	-	-	-	-	-
3289	3286	hypothetical protein	3	-	-	-	-	-	-	-	-	-	-	-	-	-	-	-	-	-	-	-	-	-	-	-	-	-	-	-	-	-	-	-	-	-	-	-	-	-	-	-	-	+	+	-	-	-	-	-	-	-	-	-	-	-	-	-	-	+
3290	3296	Retron-type RNA-directed DNA polymerase (EC 2.7.7.49)	3	-	-	-	-	-	-	-	-	-	-	-	-	-	-	-	-	-	-	-	-	-	-	-	-	-	-	-	-	-	-	-	-	-	-	-	-	-	-	-	-	+	-	-	-	-	-	+	-	-	-	-	+	-	-	-	-	-
3291	3316	Rep protein	3	-	-	-	-	-	-	-	-	-	-	-	-	-	-	+	-	-	-	-	-	-	-	-	-	-	-	-	-	-	-	-	-	-	-	-	-	-	-	-	-	-	-	-	-	-	-	+	-	-	-	-	-	+	-	-	-	-
3292	3334	hypothetical protein	3	-	-	-	-	-	-	-	-	-	-	-	-	-	-	-	-	-	-	-	-	-	-	-	-	-	-	-	-	-	-	-	-	-	-	-	-	-	-	-	-	-	-	-	-	-	-	-	-	-	-	+	-	+	+	-	-	-
3293	3460	Putative EsaC protein analog (Listeria type 3)	3	-	-	-	-	-	-	-	-	-	-	-	-	-	-	-	-	-	-	-	-	-	-	-	-	-	-	-	-	-	-	-	-	-	-	-	-	-	-	-	-	-	+	-	-	+	-	-	+	-	-	-	-	-	-	-	-	-
3294	3697	hypothetical protein	3	-	-	-	-	-	-	-	-	-	-	-	-	-	-	-	-	-	-	-	-	-	-	-	-	-	-	-	-	-	-	-	-	-	-	-	-	-	-	-	-	-	-	-	-	-	-	-	-	-	+	+	-	-	-	+	-	-
3295	3719	Type I restriction-modification system, specificity subunit S (EC 3.1.21.3)	3	-	-	-	-	-	-	-	-	-	-	-	-	-	-	-	-	-	-	-	-	-	-	-	-	-	-	-	-	-	-	-	-	-	-	-	-	-	+	-	-	-	-	-	-	-	-	-	-	-	-	+	-	-	-	+	-	-
3296	3740	Rrf2 family transcriptional regulator, group III	3	-	-	-	-	-	-	-	-	-	-	-	-	-	-	-	-	-	-	-	-	-	-	-	-	-	-	-	-	-	-	-	-	-	-	-	-	-	-	-	-	-	-	+	-	-	-	-	-	-	-	+	-	-	-	-	+	-
3297	3922	DEAD/DEAH box helicase-like protein	3	-	-	-	-	-	-	-	-	-	-	-	-	-	-	-	-	-	-	-	-	-	-	-	-	-	-	-	-	-	-	-	-	-	-	-	-	-	-	-	-	-	-	-	-	-	-	-	-	-	-	-	+	-	-	+	-	+
3298	3929	Ethanolamine utilization protein EutJ	3	-	-	-	-	-	-	-	-	-	-	-	-	-	-	-	-	-	-	-	-	-	-	-	-	-	-	-	-	-	-	-	-	-	-	-	-	-	-	-	-	-	-	+	-	-	-	-	+	-	-	-	-	-	-	-	+	-
3299	3930	Ethanolamine utilization polyhedral-body-like protein EutN	3	-	-	-	-	-	-	-	-	-	-	-	-	-	-	-	-	-	-	-	-	-	-	-	-	-	-	-	-	-	-	-	-	-	-	-	-	-	-	-	-	-	-	+	-	-	-	-	+	-	-	-	-	-	-	-	+	-
3300	3931	Carbon dioxide concentrating mechanism/carboxysome shell protein	3	-	-	-	-	-	-	-	-	-	-	-	-	-	-	-	-	-	-	-	-	-	-	-	-	-	-	-	-	-	-	-	-	-	-	-	-	-	-	-	-	-	-	+	-	-	-	-	+	-	-	-	-	-	-	-	+	-
3301	3932	hypothetical protein	3	-	-	-	-	-	-	-	-	-	-	-	-	-	-	-	-	-	-	-	-	-	-	-	-	-	-	-	-	-	-	-	-	-	-	-	-	-	-	-	-	+	-	-	-	-	-	-	+	+	-	-	-	-	-	-	-	-
3302	3938	Pyruvate formate-lyase activating enzyme (EC 1.97.1.4)	3	-	-	-	-	-	-	-	-	-	-	-	-	-	-	-	-	-	-	-	-	-	-	-	-	-	-	-	-	-	-	-	-	-	-	-	-	-	-	-	-	-	-	+	-	-	-	-	+	-	-	-	-	-	-	-	+	-
3303	3939	hypothetical protein	3	-	-	-	-	-	-	-	-	-	-	-	-	-	-	-	-	-	-	-	-	-	-	-	-	-	-	-	-	-	-	-	-	-	-	-	-	-	-	-	-	-	-	+	-	-	-	-	+	-	-	-	-	-	-	-	+	-
3304	3941	Acetaldehyde dehydrogenase (acetylating) (EC 1.2.1.10)	3	-	-	-	-	-	-	-	-	-	-	-	-	-	-	-	-	-	-	-	-	-	-	-	-	-	-	-	-	-	-	-	-	-	-	-	-	-	-	-	-	-	-	+	-	-	-	-	+	-	-	-	-	-	-	-	+	-
3305	3944	hypothetical protein	3	-	-	-	-	-	-	-	-	-	-	-	-	-	-	-	-	-	-	-	-	-	-	-	-	-	-	-	-	-	-	-	-	-	-	-	-	-	-	-	-	-	-	+	-	-	-	-	+	-	-	-	-	-	-	-	+	-
3306	3946	Pyruvate formate-lyase (EC 2.3.1.54)	3	-	-	-	-	-	-	-	-	-	-	-	-	-	-	-	-	-	-	-	-	-	-	-	-	-	-	-	-	-	-	-	-	-	-	-	-	-	-	-	-	-	-	+	-	-	-	-	+	-	-	-	-	-	-	-	+	-
3307	3950	Iron-containing alcohol dehydrogenase	3	-	-	-	-	-	-	-	-	-	-	-	-	-	-	-	-	-	-	-	-	-	-	-	-	-	-	-	-	-	-	-	-	-	-	-	-	-	-	-	-	-	-	+	-	-	-	-	+	-	-	-	-	-	-	-	+	-
3308	3951	Propanediol utilization protein PduA	3	-	-	-	-	-	-	-	-	-	-	-	-	-	-	-	-	-	-	-	-	-	-	-	-	-	-	-	-	-	-	-	-	-	-	-	-	-	-	-	-	-	-	+	-	-	-	-	+	-	-	-	-	-	-	-	+	-
3309	3952	Choline permease LicB	3	-	-	-	-	-	-	-	-	-	-	-	-	-	-	-	-	-	-	-	-	-	-	-	-	-	-	-	-	-	-	-	-	-	-	-	-	-	-	-	-	-	-	+	-	-	-	-	+	-	-	-	-	-	-	-	+	-
3310	3953	Ethanolamine utilization protein EutQ	3	-	-	-	-	-	-	-	-	-	-	-	-	-	-	-	-	-	-	-	-	-	-	-	-	-	-	-	-	-	-	-	-	-	-	-	-	-	-	-	-	-	-	+	-	-	-	-	+	-	-	-	-	-	-	-	+	-
3311	3954	Acetaldehyde dehydrogenase, ethanolamine utilization cluster	3	-	-	-	-	-	-	-	-	-	-	-	-	-	-	-	-	-	-	-	-	-	-	-	-	-	-	-	-	-	-	-	-	-	-	-	-	-	-	-	-	-	-	+	-	-	-	-	+	-	-	-	-	-	-	-	+	-
3312	3956	Pyruvate-flavodoxin oxidoreductase (EC 1.2.7.-)	3	-	-	-	-	-	-	-	-	-	-	-	-	-	-	-	-	-	-	-	-	-	-	-	-	-	-	-	-	-	-	-	-	-	-	-	-	-	-	-	-	-	-	+	-	-	-	-	+	-	-	-	-	-	-	-	+	-
3313	3962	putative replication protein RepA	3	-	-	-	-	-	-	-	-	-	-	-	-	-	-	-	-	-	-	-	-	-	-	-	-	-	-	-	-	-	-	-	-	-	-	-	-	-	-	-	-	+	-	-	-	-	-	-	-	-	+	-	+	-	-	-	-	-
3314	3967	conserved domain protein	3	-	-	-	-	-	-	-	-	-	-	-	-	-	-	-	-	-	-	-	-	-	-	-	-	-	-	-	-	-	-	-	-	-	-	-	-	-	-	-	-	-	-	-	-	-	-	-	-	+	+	-	+	-	-	-	-	-
3315	3973	hypothetical protein	3	-	-	-	-	-	-	-	-	-	-	-	-	-	-	+	-	-	-	-	-	-	-	-	-	-	-	-	-	-	-	-	-	-	-	-	-	-	-	-	-	-	-	-	-	-	-	+	-	-	-	-	-	+	-	-	-	-
3316	4195	hypothetical protein	3	-	-	-	-	-	-	-	-	-	-	-	-	-	-	-	-	-	-	-	-	-	-	-	-	-	-	-	-	-	-	-	-	-	-	-	-	-	-	+	+	-	-	-	-	-	-	-	-	+	-	-	-	-	-	-	-	-
3317	4199	hypothetical protein	3	-	-	-	-	-	-	-	-	-	-	-	-	-	-	-	-	-	-	-	-	-	-	-	-	-	-	-	-	-	-	-	-	-	-	-	-	-	+	+	+	-	-	-	-	-	-	-	-	-	-	-	-	-	-	-	-	-
3318	4211	hypothetical protein	3	-	-	-	-	-	-	-	-	-	-	-	-	-	-	-	-	-	-	-	-	-	-	+	-	-	-	-	-	-	-	-	-	-	-	-	-	-	-	+	+	-	-	-	-	-	-	-	-	-	-	-	-	-	-	-	-	-
3319	4229	phi5005.3 integrase	3	-	-	-	-	-	-	-	-	-	-	-	-	-	-	-	+	-	+	+	-	-	-	-	-	-	-	-	-	-	-	-	-	-	-	-	-	-	-	-	-	-	-	-	-	-	-	-	-	-	-	-	-	-	-	-	-	-
3320	4231	Possible polysaccharide transport protein, putative	3	-	-	-	-	-	-	-	-	-	-	-	-	-	-	-	+	-	-	-	-	-	-	-	-	-	-	-	-	-	-	-	-	-	-	-	-	-	-	-	-	-	-	-	-	-	-	-	-	+	-	-	-	-	-	-	-	+
3321	4233	bacteriocin, putative	3	-	-	-	-	-	-	-	-	-	-	-	-	-	-	-	-	-	-	-	-	-	-	-	-	-	-	-	-	-	-	-	-	-	-	-	-	-	-	-	-	-	-	-	-	-	-	-	-	+	-	-	-	-	-	+	-	+
3322	4234	putative glycosyltransferase	3	-	-	-	-	-	-	-	-	-	-	-	-	-	-	-	-	-	-	-	-	-	-	-	-	-	-	-	-	-	-	-	-	-	-	-	-	-	-	-	-	-	-	-	-	-	-	-	-	+	-	-	+	-	-	-	-	+
3323	4244	UDP-glucose 4-epimerase (EC 5.1.3.2)	3	-	-	-	-	-	-	-	-	-	-	-	-	-	-	-	-	-	-	-	-	-	-	-	-	-	-	-	-	-	-	-	-	-	-	-	-	-	-	-	-	-	+	-	-	-	-	-	+	-	-	-	+	-	-	-	-	-
3324	4251	Phage-related protein	3	-	-	-	-	-	-	-	-	-	-	-	-	-	-	-	-	-	-	-	-	-	-	-	-	-	-	-	-	-	-	-	-	-	-	-	-	-	-	-	-	-	-	-	-	-	-	+	+	+	-	-	-	-	-	-	-	-
3325	4255	NPQTN specific sortase B	3	-	-	-	-	-	-	-	-	-	-	-	-	-	-	-	-	-	-	-	-	-	-	-	-	-	-	-	-	-	-	-	-	-	-	-	-	-	-	-	-	+	+	+	-	-	-	-	-	-	-	-	-	-	-	-	-	-
3326	4256	hypothetical protein	3	-	-	-	-	-	-	-	-	-	-	-	-	-	-	-	-	-	-	-	-	-	-	-	-	-	-	-	-	-	-	-	-	+	-	-	-	-	-	-	-	-	+	-	+	-	-	-	-	-	-	-	-	-	-	-	-	-
3327	4257	major tail protein	3	-	-	-	-	-	-	-	-	-	-	-	-	-	-	-	-	-	-	-	-	-	-	-	-	-	-	-	-	-	-	-	-	-	-	-	-	-	-	-	-	+	-	-	-	-	-	-	-	-	-	-	+	-	-	+	-	-
3328	4258	Transcriptional regulator DegU, LuxR family	3	-	-	-	-	-	-	-	-	-	-	-	-	-	-	-	-	-	-	-	-	-	-	-	-	-	-	-	-	-	-	-	-	-	-	-	-	-	-	-	-	+	-	-	-	-	-	+	-	-	-	-	-	-	+	-	-	-
3329	4265	Death on curing protein, Doc toxin	3	-	-	-	-	-	-	-	-	-	-	-	-	-	-	-	-	-	-	-	-	-	-	-	-	-	-	-	-	-	-	-	-	-	-	-	-	-	-	-	-	-	-	-	-	-	-	-	-	-	+	+	-	-	+	-	-	-
3330	4268	Arylsulfatase regulator	3	-	-	-	-	-	-	-	-	-	-	-	-	-	-	-	-	-	-	-	-	-	-	-	-	-	-	-	-	-	-	-	-	-	-	-	-	-	-	-	-	-	-	-	-	-	-	-	-	-	+	-	-	+	-	+	-	-
3331	4271	hypothetical protein	3	-	-	-	-	-	-	-	-	-	-	-	-	-	-	-	-	-	-	-	-	-	-	-	-	-	-	-	-	-	-	-	-	-	-	-	-	-	-	-	-	-	-	-	-	-	-	-	-	-	+	+	-	-	+	-	-	-
3332	4290	Mobile element protein	3	-	-	-	-	-	-	-	-	-	-	-	-	-	-	-	-	-	-	-	-	-	-	-	-	-	-	-	-	-	-	-	-	-	-	-	+	-	-	-	-	-	-	-	-	-	-	-	-	-	-	+	-	+	-	-	-	-
3333	4331	replication initiator protein A	3	-	-	-	-	-	-	-	-	-	-	-	-	-	-	-	-	-	-	-	-	-	-	-	-	-	-	-	-	-	-	-	-	-	-	-	-	-	-	-	-	-	-	-	+	-	-	-	-	-	-	-	-	-	-	+	+	-
3334	4335	Type I restriction-modification system, DNA-methyltransferase subunit M (EC 2.1.1.72)	3	-	-	-	-	-	-	-	-	-	-	-	-	-	-	-	-	-	-	-	-	-	-	-	-	-	-	-	-	-	-	-	-	+	+	+	-	-	-	-	-	-	-	-	-	-	-	-	-	-	-	-	-	-	-	-	-	-
3335	4336	Metal transporter, ZIP family	3	-	-	-	-	-	-	-	-	-	-	-	-	-	-	-	-	-	-	-	-	-	-	-	-	-	-	-	-	-	-	-	-	+	+	+	-	-	-	-	-	-	-	-	-	-	-	-	-	-	-	-	-	-	-	-	-	-
3336	4605	hypothetical protein	3	-	-	-	-	-	-	-	-	-	-	-	-	-	-	-	-	+	+	-	-	-	-	-	-	-	-	-	-	-	-	-	-	-	-	-	-	-	-	-	-	-	-	-	-	-	-	-	-	+	-	-	-	-	-	-	-	-
3337	4632	DNA polymerase I (EC 2.7.7.7)	3	-	-	-	-	-	-	-	-	-	-	-	-	-	-	-	-	-	-	-	-	-	-	-	-	-	-	-	-	-	-	-	-	-	-	-	-	-	-	+	-	-	-	-	-	-	-	-	-	-	-	+	-	-	+	-	-	-
3338	4638	DNA primase, phage associated	3	-	-	-	-	-	-	-	-	-	-	-	-	-	-	-	-	-	-	-	-	-	-	-	-	-	-	-	-	-	-	-	-	-	-	-	-	-	-	+	-	-	-	-	-	-	-	-	-	-	-	+	-	-	+	-	-	-
3339	4639	Phage protein	3	-	-	-	-	-	-	-	-	-	-	-	-	-	-	-	-	-	-	-	-	-	-	-	-	-	-	-	-	-	-	-	-	-	-	-	-	-	-	+	-	-	-	-	-	-	-	-	-	-	-	+	-	-	+	-	-	-
3340	4640	DNA helicase, phage-associated	3	-	-	-	-	-	-	-	-	-	-	-	-	-	-	-	-	-	-	-	-	-	-	-	-	-	-	-	-	-	-	-	-	-	-	-	-	-	-	+	-	-	-	-	-	-	-	-	-	-	-	+	-	-	+	-	-	-
3341	4646	Phage protein	3	-	-	-	-	-	-	-	-	-	-	-	-	-	-	-	-	-	-	-	-	-	-	-	-	-	-	-	-	-	-	-	-	-	-	-	-	-	-	+	-	-	-	-	-	-	-	-	-	-	-	+	-	-	+	-	-	-
3342	4647	Type III restriction-modification system methylation subunit (EC 2.1.1.72)	3	-	-	-	-	-	-	-	-	-	-	-	-	-	-	-	-	-	-	-	-	-	-	-	-	-	-	-	-	-	-	-	-	-	-	-	-	-	+	+	+	-	-	-	-	-	-	-	-	-	-	-	-	-	-	-	-	-
3343	4649	Adenine-specific methyltransferase (EC 2.1.1.72)	3	-	-	-	-	-	-	-	-	-	-	-	-	-	-	-	-	-	-	-	-	-	-	-	-	-	-	-	-	-	-	-	-	-	-	+	-	-	-	-	+	-	-	-	-	-	-	-	-	-	-	-	-	-	-	+	-	-
3344	4654	Mobile element protein	3	-	-	-	-	-	-	-	-	-	-	-	-	-	-	-	+	-	-	-	-	-	-	-	-	-	-	-	-	-	-	-	-	-	-	-	-	-	-	-	-	-	+	-	+	-	-	-	-	-	-	-	-	-	-	-	-	-
3345	4668	hypothetical protein	3	-	-	-	-	-	-	-	-	-	-	-	-	-	-	-	-	-	-	-	-	-	-	+	-	-	-	-	-	-	-	-	-	-	-	-	-	-	-	-	+	-	-	+	-	-	-	-	-	-	-	-	-	-	-	-	-	-
3346	4672	Mobile element protein	3	-	-	-	-	-	-	-	-	-	-	-	-	-	-	-	+	-	-	-	-	-	-	-	-	-	-	-	-	-	-	-	-	-	-	-	-	-	-	-	-	-	+	-	-	-	-	-	-	+	-	-	-	-	-	-	-	-
3347	4683	hypothetical protein	3	-	-	-	-	-	-	-	-	-	-	-	-	-	-	-	-	-	-	-	-	-	-	-	-	-	-	-	-	-	-	-	-	-	-	-	-	-	-	-	-	-	-	-	-	-	+	-	-	+	-	-	-	-	-	+
3348	4685	Cadmium resistance protein	3	-	-	-	-	-	-	-	-	-	-	-	-	-	-	-	-	-	-	-	-	-	-	-	-	-	-	-	-	-	-	-	-	-	-	-	-	-	-	-	-	-	-	-	-	-	+	+	-	-	-	-	-	-	-	+
3349	4688	hypothetical protein	3	-	-	-	-	-	-	-	-	-	-	-	-	-	-	-	-	-	-	-	-	-	-	-	-	-	-	-	-	-	-	-	-	-	-	-	-	-	-	-	-	+	+	-	-	-	-	-	-	-	-	-	-	-	-	-	-	+
3350	4691	mobilization protein Mob 14-3	3	-	-	-	-	-	-	-	-	-	-	-	-	-	-	-	-	-	-	-	-	-	-	-	-	-	-	-	-	-	-	-	-	-	-	-	-	-	-	-	-	-	-	+	-	-	-	-	+	-	-	-	-	-	-	-	+	-
3351	4692	agglutinin receptor	3	-	-	-	-	-	-	-	-	-	-	-	-	-	-	-	-	-	-	-	+	-	-	-	-	-	-	-	-	-	-	-	-	-	-	-	-	-	-	-	-	-	-	-	-	-	-	-	+	-	+	-	-	-	-	-	-	-
3352	4694	Multicopper oxidase	3	-	-	-	-	-	-	-	-	-	-	-	-	-	-	-	-	-	-	-	-	-	-	-	-	-	-	-	-	-	-	-	-	-	-	-	-	-	-	-	-	-	-	+	-	-	-	-	+	-	-	+	-	-	-	-	-	-
3353	4696	hypothetical protein	3	-	-	-	-	-	-	-	-	-	-	-	-	-	-	-	-	-	-	-	-	-	-	-	-	-	-	-	-	-	-	-	-	-	-	-	-	-	-	-	-	-	-	+	-	-	-	-	+	-	-	-	-	-	-	-	+	-
3354	4702	Ethanolamine utilization protein similar to PduA/PduJ	3	-	-	-	-	-	-	-	-	-	-	-	-	-	-	-	-	-	-	-	-	-	-	-	-	-	-	-	-	-	-	-	-	-	-	-	-	-	-	-	-	-	-	+	-	-	-	-	+	-	-	-	-	-	-	-	+	-
3355	4705	Mobile element protein	3	-	-	-	-	-	-	-	-	-	-	-	-	-	-	-	-	-	-	-	-	-	-	-	-	-	-	-	-	-	-	-	-	-	-	-	-	-	-	-	-	-	-	-	-	-	-	-	+	+	-	-	-	+	-	-	-	-
3356	4707	Ethanolamine utilization protein similar to PduV	3	-	-	-	-	-	-	-	-	-	-	-	-	-	-	-	-	-	-	-	-	-	-	-	-	-	-	-	-	-	-	-	-	-	-	-	-	-	-	-	-	-	-	+	-	-	-	-	+	-	-	-	-	-	-	-	+	-
3357	4713	hypothetical protein	3	-	-	-	-	-	-	-	-	-	-	-	-	-	-	-	-	-	-	-	-	-	-	-	-	-	-	-	-	-	-	-	-	-	-	-	-	-	-	-	-	+	+	+	-	-	-	-	-	-	-	-	-	-	-	-	-	-
3358	4714	ADA regulatory protein / Methylated-DNA--protein-cysteine methyltransferase (EC 2.1.1.63)	3	-	-	-	-	-	-	-	-	-	-	-	-	-	-	-	-	-	-	-	-	-	-	-	-	-	-	-	-	-	-	-	-	-	-	-	-	-	-	+	+	+	-	-	-	-	-	-	-	-	-	-	-	-	-	-
3359	4720	hypothetical protein	3	-	-	-	-	-	-	-	-	-	-	-	-	-	-	-	-	-	-	-	-	-	-	-	-	-	-	-	-	-	-	-	-	-	-	-	-	-	-	-	-	+	-	-	-	-	-	+	-	-	-	-	-	-	+	-	-	-
3360	4722	Phage terminase, large subunit	3	-	-	-	-	-	-	-	-	-	-	-	-	-	-	-	-	-	-	-	-	-	-	-	-	-	-	-	-	-	-	-	-	-	-	-	+	+	-	-	-	+	-	-	-	-	-	-	-	-	-	-	-	-	-	-	-	-
3361	4723	ABC transporter, ATP-binding protein	3	-	-	-	-	-	-	-	-	-	-	-	-	-	-	-	-	-	-	-	-	-	-	-	-	-	-	-	-	-	-	-	-	-	-	-	-	-	-	-	-	+	-	-	-	-	-	+	-	-	-	-	-	-	+	-	-	-
3362	4754	Oxidoreductase ucpA (EC 1.-.-.-)	3	-	-	-	-	-	-	-	-	-	-	-	-	-	-	-	-	-	-	-	-	-	-	-	-	-	-	-	-	-	-	-	-	-	-	-	-	-	-	-	-	-	-	-	-	-	-	-	-	-	+	+	-	-	-	+	-	-
3363	4760	Late competence protein ComEC, DNA transport	3	-	-	-	-	-	-	-	-	-	-	-	-	+	-	-	-	-	-	-	-	-	-	-	-	-	-	-	-	-	-	-	-	-	-	-	-	-	-	-	-	-	-	-	-	+	-	-	-	+	-	-	-	-	-	-	-	-
3364	4765	NPQTN specific sortase B	3	-	-	-	-	-	-	-	-	-	-	-	-	-	-	+	-	-	-	-	-	-	-	-	-	-	-	-	-	-	-	-	-	-	-	-	-	-	-	-	-	-	-	-	+	+	-	-	-	-	-	-	-	-	-	-	-	-
3365	4767	Death on curing protein, Doc toxin	3	-	-	-	-	-	-	-	-	-	-	-	-	-	-	-	-	-	-	-	-	-	-	-	-	-	-	-	-	-	-	-	-	-	-	-	-	-	-	-	-	-	-	-	-	+	-	-	-	-	-	+	-	-	+	-	-	-
3366	4788	Anticodon nuclease	3	-	-	-	-	-	-	-	-	-	-	-	-	-	-	-	-	-	-	-	-	-	-	-	-	-	-	-	-	-	-	-	-	-	-	-	-	-	+	-	-	-	-	-	+	-	-	-	-	-	-	-	-	-	-	+	-	-
3367	4817	Phage protein	3	-	-	-	-	-	-	-	-	-	-	-	-	-	-	-	-	-	-	-	-	-	-	-	-	-	-	-	-	-	-	-	-	-	-	-	-	-	-	-	-	-	-	-	-	-	-	-	-	-	-	-	+	-	+	-	+	-
3368	4819	cell wall surface anchor family protein	3	-	-	-	-	-	-	-	-	-	-	-	-	-	-	-	-	-	-	-	-	-	-	-	-	-	-	-	-	-	-	-	-	-	-	-	-	-	-	-	-	-	-	-	+	-	-	+	-	+	-	-	-	-	-	-	-	-
3369	4820	L-rhamnose isomerase (EC 5.3.1.14)	3	-	-	-	-	+	-	-	-	-	-	-	-	-	-	-	-	-	-	-	-	-	-	-	-	-	-	-	-	-	-	-	-	-	-	-	-	-	-	-	-	-	-	-	+	-	-	-	-	-	-	+	-	-	-	-	-	-
3370	4822	DNA topoisomerase III (EC 5.99.1.2)	3	-	-	-	-	-	-	-	+	-	-	-	-	-	-	-	-	-	-	-	-	-	-	-	-	-	-	-	+	-	-	-	-	-	+	-	-	-	-	-	-	-	-	-	-	-	-	-	-	-	-	-	-	-	-	-	-	-
3371	5130	hypothetical protein	3	-	-	-	-	-	-	-	-	-	-	-	-	-	-	-	-	+	-	-	-	-	-	-	-	-	-	-	-	-	-	-	-	-	-	-	-	-	-	-	-	-	-	-	-	+	-	-	-	-	-	+	-	-	-	-	-	-
3372	5133	hypothetical protein	3	-	-	-	-	-	-	-	-	-	-	-	-	-	-	-	-	+	-	-	-	-	-	-	-	-	-	-	-	-	-	-	-	-	-	-	+	+	-	-	-	-	-	-	-	-	-	-	-	-	-	-	-	-	-	-	-	-
3373	5137	acetyltransferase	3	-	-	-	-	-	-	-	-	-	-	-	-	-	-	-	-	+	+	-	-	-	-	-	-	-	-	-	-	-	-	-	-	-	-	-	-	-	-	-	-	-	-	-	-	-	-	-	-	-	-	-	-	-	-	-	-	+
3374	5139	Hypothetical protein	3	-	-	-	-	-	-	-	-	-	-	-	-	-	-	-	-	+	+	-	-	-	-	-	-	-	-	-	-	-	-	-	-	-	-	-	-	-	-	-	-	-	-	-	+	-	-	-	-	-	-	-	-	-	-	-	-	-
3375	5146	hypothetical protein	3	-	-	-	-	-	-	-	-	-	-	-	-	-	-	-	+	+	+	-	-	-	-	-	-	-	-	-	-	-	-	-	-	-	-	-	-	-	-	-	-	-	-	-	-	-	-	-	-	-	-	-	-	-	-	-	-	-
3376	5156	Cell wall surface anchor family protein	3	-	-	-	-	-	-	-	-	-	-	-	-	-	-	-	-	+	+	-	-	-	-	-	-	-	-	-	-	-	-	-	-	-	-	-	-	-	-	-	-	-	-	-	-	+	-	-	-	-	-	-	-	-	-	-	-	-
3377	5165	putative ATPase	3	-	-	-	-	-	-	-	-	-	-	-	-	-	-	-	-	-	-	-	-	-	-	-	-	+	-	-	-	-	-	-	-	-	-	-	-	-	-	-	-	-	-	-	-	-	-	-	-	-	+	-	-	-	-	-	+	-
3378	5168	FIG007079: UPF0348 protein family	3	-	-	-	-	-	-	-	-	-	-	-	-	-	-	-	-	-	-	-	-	-	+	-	-	+	-	-	-	-	-	-	-	-	-	-	-	-	-	-	-	-	-	-	-	-	-	-	-	-	-	-	-	-	-	-	+	-
3379	5171	unknown phage protein	3	-	-	-	-	-	-	-	-	-	-	-	-	-	-	-	-	-	-	-	-	-	-	-	-	-	-	-	-	-	-	-	-	-	-	+	-	-	-	+	+	-	-	-	-	-	-	-	-	-	-	-	-	-	-	-	-	-
3380	5172	hypothetical protein	3	-	-	-	-	-	-	-	-	-	-	-	-	-	-	-	-	-	-	-	-	-	-	-	-	-	-	-	-	-	-	-	-	-	-	-	-	-	-	+	+	-	-	-	-	-	-	-	-	-	-	-	-	-	-	-	+	-
3381	5174	putative DNA replication protein	3	-	-	-	-	-	-	-	-	-	-	-	-	-	-	-	-	-	-	-	-	-	-	-	-	-	-	-	-	-	-	-	-	-	-	-	-	-	-	+	+	-	-	-	-	-	-	-	-	+	-	-	-	-	-	-	-	-
3382	5177	hypothetical protein	3	-	-	-	-	-	-	-	-	-	-	-	-	-	-	-	-	-	-	-	-	-	-	-	-	-	-	-	-	-	-	-	-	-	-	-	-	-	-	+	+	-	-	-	-	-	-	-	-	-	-	-	-	-	-	-	-	+
3383	5178	Helicase conserved C-terminal domain protein	3	-	-	-	-	-	-	-	-	-	-	-	-	-	-	-	-	-	-	-	-	-	-	-	-	-	-	-	-	-	-	-	-	-	-	-	-	-	-	+	+	-	-	-	-	-	-	-	-	-	-	-	-	-	-	-	+	-
3384	5179	Phage protein	3	-	-	-	-	-	-	-	-	-	-	-	-	-	-	-	-	-	-	-	-	-	-	-	-	-	-	-	-	-	-	-	-	-	-	-	-	-	-	+	-	-	-	-	-	-	-	+	-	-	+	-	-	-	-	-	-	-
3385	5181	Phage protein	3	-	-	-	-	-	-	-	-	-	-	-	-	-	-	-	-	-	-	-	-	-	-	-	-	-	-	-	-	-	-	-	-	-	+	-	-	-	-	+	-	-	-	-	-	-	-	-	-	-	-	-	-	-	+	-	-	-
3386	5197	hypothetical protein	3	-	-	-	-	-	-	-	-	-	-	-	-	-	-	-	-	-	-	-	-	-	-	-	-	-	-	-	-	-	-	-	-	-	-	-	-	-	-	-	+	-	-	-	-	-	-	+	-	-	-	-	+	-	-	-	-	-
3387	5199	NAD(FAD)-utilizing dehydrogenases	3	-	-	-	-	-	-	-	-	-	-	-	-	-	-	-	-	-	-	-	-	-	-	-	-	-	-	-	-	-	-	-	-	-	-	-	-	+	-	-	+	-	-	-	-	-	-	-	-	-	+	-	-	-	-	-	-	-
3388	5203	antirepressor	3	-	-	-	-	-	-	-	-	-	-	-	-	-	-	-	-	-	-	-	-	-	-	-	-	-	-	-	-	-	-	-	-	-	-	-	+	-	-	-	-	-	-	-	-	+	+	-	-	-	-	-	-	-	-	-	-	-
3389	5206	Integrase/recombinase, putative, truncated	3	-	-	-	-	-	-	-	-	-	-	-	-	-	-	-	-	-	-	+	+	-	-	-	-	-	-	-	-	-	-	-	-	-	-	-	-	-	-	-	-	-	-	-	-	-	+	-	-	-	-	-	-	-	-	-	-	-
3390	5211	Beta-hexosaminidase (EC 3.2.1.52)	3	-	-	-	-	-	-	-	-	-	-	-	-	-	-	-	-	-	-	-	-	-	-	-	-	-	-	-	-	-	-	-	-	-	-	-	-	-	-	-	-	-	-	-	-	+	+	-	-	+	-	-	-	-	-	-	-	-
3391	5222	Putative N-acetylgalactosaminyl-diphosphoundecaprenol glucuronosyltransferase	3	-	-	-	-	-	-	-	-	-	-	-	-	-	-	-	-	-	-	-	-	-	-	+	+	-	-	-	-	-	-	-	-	-	-	-	-	-	-	-	-	-	-	-	-	-	-	-	+	-	-	-	-	-	-	-
3392	5224	Heptaprenyl diphosphate synthase component II (EC 2.5.1.30)	3	-	-	-	-	-	-	-	-	-	-	-	-	-	-	-	+	-	-	-	-	-	-	-	-	-	-	-	-	-	-	-	-	-	-	-	-	-	-	-	-	-	+	+	-	-	-	-	-	-	-	-	-	-	-	-	-	-
3393	5225	hypothetical protein	3	-	-	-	-	-	-	-	-	-	-	-	-	-	-	-	+	-	-	-	-	-	-	-	-	-	-	-	-	-	-	-	-	-	-	-	-	-	-	-	-	-	+	+	-	-	-	-	-	-	-	-	-	-	-	-	-	-
3394	5229	77ORF017	3	-	-	-	-	-	-	-	-	-	-	-	-	-	-	-	+	-	-	-	-	-	-	-	-	-	-	-	-	-	-	-	-	-	+	-	-	-	-	-	-	-	-	-	-	-	-	-	-	-	-	+	-	-	-	-	-	-

Table S5. Precence/absence of each HG among 57 *S. suis* strains

				1																13			25					28						104		94			76			53/54		Divergent *S. suis*																
Index[a]	ClusterID	Gene products	Members	98HAH33	05ZYH33	SC84	A7	SS12	P1/7	DAT273	NIAH11434	JS14	GZ1	BM407	S735	13730	MNCM07	NCTC 10446	14636	NCTC 10237	ST1	2651	89-1591	MNCM04	MNCM25	8074	D9	4961	ST3	MNCM43	DAT292	DAT260	NIAH11435	MNCM21	MNCM50	89-2479	DAT234	DAT290	93A	42A	NT77	6407	11538	8830	12814	92-1400	89-590	2726	2524	89-3576-3	4417	D12	10581	89-5259	88-5299A	R61	92-1191	14A	92-4172	22083
3395	5236	hypothetical protein	3	-	-	-	-	-	-	-	-	-	-	-	-	-	-	-	-	-	-	+	-	-	-	-	-	-	-	-	-	-	-	-	-	-	-	-	-	-	-	-	-	-	-	-	-	-	-	-	-	-	+	-	-	+
3396	5241	Competence protein CoiA	3	-	-	-	-	-	-	-	-	-	-	-	-	-	-	-	-	-	-	-	-	-	-	-	-	-	-	-	-	-	-	-	-	-	-	-	-	+	+	-	-	-	-	-	-	-	-	-	-	-	-	-	-	+
3397	5244	hypothetical protein	3	-	-	-	-	-	-	-	-	-	-	-	-	-	-	-	-	-	-	-	-	-	-	-	-	-	-	-	-	-	-	-	-	-	-	-	-	-	-	-	-	-	-	-	-	-	-	-	+	-	-	+	-	+
3398	5247	Mobile element protein	3	-	-	-	-	-	-	-	-	-	-	-	-	-	-	-	-	-	-	-	-	-	-	-	-	-	-	-	-	-	-	-	-	-	-	-	-	-	-	-	-	-	-	+	+	+	-	-	-	-	-	-	-	-
3399	5248	hypothetical protein	3	-	-	-	-	-	-	-	-	-	-	-	-	-	-	-	-	-	-	-	-	-	-	-	-	-	-	-	-	-	-	-	-	-	-	-	-	-	-	+	-	-	-	-	+	-	-	-	+	-	-	-	-	-
3400	5254	hypothetical protein	3	-	-	-	-	-	-	-	-	-	-	-	-	-	-	-	-	-	-	-	-	+	+	-	-	-	-	-	-	-	-	-	-	-	-	-	-	-	-	-	-	-	-	-	+	-	-	-	-	-	-	-	-	-
3401	5257	hypothetical protein	3	-	-	-	-	-	-	-	-	-	-	-	-	-	-	-	-	-	-	-	-	-	-	-	-	-	-	-	-	-	-	-	-	-	-	-	-	-	-	+	+	-	-	-	+	-	-	-	-	-	-	-	-	-
3402	5260	hypothetical protein	3	-	-	-	-	-	-	-	-	-	-	-	-	-	-	-	-	-	-	-	-	-	-	-	-	-	-	-	-	-	-	-	-	-	-	-	-	-	-	-	+	-	-	-	+	-	-	-	-	-	-	-	+	-
3403	5273	putative transcriptional regulator	3	-	-	-	-	-	-	-	-	-	-	-	-	-	-	-	-	-	-	-	-	-	-	-	-	-	-	-	-	+	-	-	-	-	-	-	-	-	+	-	+	-	-	-	-	-	-	-	-	-	-	-	-	-
3404	5275	hypothetical protein	3	-	-	-	-	-	-	-	-	-	-	-	-	-	-	-	-	-	-	-	-	-	-	-	-	-	-	-	-	-	-	-	-	-	-	-	-	-	+	-	-	-	-	+	-	-	+	-	-	-	-	-	-	-
3405	5279	hypothetical protein	3	-	-	-	-	-	-	-	-	-	-	-	-	-	-	-	-	-	-	-	-	-	-	-	-	-	-	-	-	-	-	-	-	-	-	-	-	+	-	-	-	-	-	-	-	-	-	-	+	-	-	+	-	-
3406	5280	unknown	3	-	-	-	-	-	-	-	-	-	-	-	-	-	-	-	-	-	-	-	-	-	-	-	-	-	-	-	-	-	-	-	-	-	-	-	-	+	-	-	-	-	-	-	-	-	-	-	+	-	-	+	-	-
3407	5282	Phage resolvase	3	-	-	-	-	-	-	-	-	-	-	-	-	-	-	-	-	-	-	+	-	-	-	-	-	-	-	-	-	-	-	-	-	-	-	-	-	+	-	-	-	-	-	-	-	-	-	-	-	-	-	-	+	-
3408	5283	hypothetical protein	3	-	-	-	-	-	-	-	-	-	-	-	-	-	-	-	-	-	-	-	-	-	-	-	-	-	-	-	-	-	-	-	-	-	-	-	-	+	-	-	-	-	-	-	-	-	-	-	+	-	-	+	-	-
3409	5285	hypothetical protein	3	-	-	-	-	-	-	-	-	-	-	-	-	-	-	-	-	-	-	-	-	-	-	-	-	-	-	-	-	-	-	-	-	-	-	-	-	+	-	-	-	-	-	-	-	-	-	-	+	-	-	+	-	-
3410	5286	Predicted membrane metalloendopeptidase	3	-	-	-	-	-	-	-	-	-	-	-	-	-	-	-	-	-	-	-	-	-	-	-	-	-	-	-	-	-	-	-	-	-	-	-	-	+	-	-	-	-	-	-	-	-	-	-	+	-	-	+	-	-
3411	5287	Helix-turn-helix domain protein	3	-	-	-	-	-	-	-	-	-	-	-	-	-	-	-	-	-	-	-	-	-	-	-	-	-	-	-	-	-	-	-	-	+	+	-	-	-	-	-	-	-	-	-	-	-	-	-	+	-	-	-	-	-
3412	5289	hypothetical protein	3	-	-	-	-	-	-	-	-	-	-	-	-	-	-	-	-	-	-	+	-	-	-	-	-	-	-	-	-	-	-	-	-	-	-	-	-	+	-	-	-	-	-	-	-	-	-	-	-	-	-	-	+	-
3413	5290	hypothetical protein	3	-	-	-	-	-	-	-	-	-	-	-	-	-	-	-	-	-	-	-	-	-	-	-	-	-	-	-	-	-	-	-	-	-	-	-	-	+	-	-	-	-	-	+	-	-	-	-	-	-	+	-	-	-
3414	5294	hypothetical protein	3	-	-	-	-	-	-	-	-	-	-	-	-	-	-	-	-	-	-	-	-	-	-	-	-	-	-	-	-	-	-	-	-	-	-	-	-	+	-	-	-	-	-	-	-	-	-	-	+	-	-	+	-	-
3415	5295	Phage replicative DNA helicase, repA	3	-	-	-	-	-	-	-	-	-	-	-	-	-	-	-	-	-	-	+	-	-	-	-	-	-	-	-	-	-	-	-	-	-	-	-	-	+	-	-	-	-	-	-	-	-	-	-	-	-	-	-	+	-
3416	5296	prophage Lp1 protein 51	3	-	-	-	-	-	-	-	-	-	-	-	-	-	-	-	-	-	-	-	-	-	-	-	-	-	-	-	-	-	-	-	-	-	-	-	-	+	-	-	-	-	-	-	-	-	-	-	+	-	-	+	-	-
3417	5299	DNA helicase, phage-associated; Type III restriction enzme	3	-	-	-	-	-	-	-	-	-	-	-	-	-	-	-	-	-	-	+	-	-	-	-	-	-	-	-	-	-	-	-	-	-	-	-	-	+	-	-	-	-	-	-	-	-	-	-	-	-	-	-	+	-
3418	5301	hypothetical protein	3	-	-	-	-	-	-	-	-	-	-	-	-	-	-	-	-	-	-	+	-	-	-	-	-	-	-	-	-	-	-	-	-	-	-	-	-	+	-	-	-	-	-	-	-	-	-	-	-	-	-	-	+	-
3419	5303	hypothetical protein	3	-	-	-	-	-	-	-	-	-	-	-	-	-	-	-	-	-	-	-	-	-	-	-	-	-	-	-	-	-	-	-	-	-	-	-	-	+	-	-	-	-	-	-	-	-	-	-	+	-	-	+	-	-
3420	5304	putative tape measure protein	3	-	-	-	-	-	-	-	-	-	-	-	-	-	-	-	-	-	-	-	-	-	-	-	-	-	-	-	-	-	-	-	-	-	-	-	-	+	-	-	-	-	-	-	-	-	-	-	+	-	-	+	-	-
3421	5305	hypothetical protein	3	-	-	-	-	-	-	-	-	-	-	-	-	-	-	-	-	-	-	-	-	-	-	-	-	-	-	-	-	-	-	-	-	-	-	-	-	+	-	-	-	-	-	-	-	-	-	-	+	-	-	+	-	-
3422	5306	Phage portal	3	-	-	-	-	-	-	-	-	-	-	-	-	-	-	-	-	-	-	-	-	-	-	-	-	-	-	-	-	-	-	-	-	-	-	-	-	+	-	-	-	-	-	-	-	-	-	-	+	-	-	+	-	-
3423	5307	hypothetical protein	3	-	-	-	-	-	-	-	-	-	-	-	-	-	-	-	-	-	-	+	-	-	-	-	-	-	-	-	-	-	-	-	-	-	-	-	-	+	-	-	-	-	-	-	-	-	-	-	-	-	-	-	+	-
3424	5308	ORF23	3	-	-	-	-	-	-	-	-	-	-	-	-	-	-	-	-	-	-	+	-	-	-	-	-	-	-	-	-	-	-	-	-	-	-	-	-	+	-	-	-	-	-	-	-	-	-	-	-	-	+	-	-	-
3425	5314	hypothetical protein	3	-	-	-	-	-	-	-	-	-	-	-	-	-	-	-	-	-	-	-	-	-	-	-	-	-	-	-	-	-	-	+	-	-	-	-	-	-	-	-	-	-	-	-	-	-	+	+	-	-	-	-	-	-
3426	5335	Protein yjgK	3	-	-	-	-	-	-	-	-	-	-	-	-	-	-	-	-	-	-	-	-	-	-	-	-	-	-	-	-	-	-	-	-	-	-	-	-	-	-	-	-	-	-	-	-	-	+	-	+	-	-	+	-	-
3427	5336	hypothetical protein	3	-	-	-	-	-	-	-	-	-	-	-	-	-	-	-	-	-	-	-	-	-	-	-	-	-	-	-	-	-	-	-	-	-	-	-	-	-	-	-	-	-	-	-	-	-	+	-	+	-	-	+	-	-
3428	5358	hypothetical protein	3	-	-	-	-	-	-	-	-	-	-	-	-	-	-	-	-	-	-	-	-	-	-	-	-	-	-	-	-	-	-	-	-	-	-	-	-	-	-	-	-	+	-	-	-	-	+	+	-	-	-	-	-	-
3429	5367	Thiol peroxidase, Tpx-type (EC 1.11.1.15)	3	-	-	-	-	-	-	-	-	-	-	-	-	-	-	-	-	-	-	-	-	-	-	-	-	-	-	-	+	-	-	-	+	-	-	-	-	-	+	-	-	-	-	-	-	-	-	-	-	-	-	-	-	-
3430	5369	hypothetical protein	3	-	-	-	-	-	-	-	-	-	-	-	-	-	-	+	-	-	-	+	-	-	-	-	-	-	-	-	-	-	-	-	-	-	-	-	-	-	-	-	-	-	-	-	-	-	-	-	-	-	-	+	-	-
3431	5370	Signal peptidase I (EC 3.4.21.89)	3	-	-	-	-	-	-	-	-	-	-	-	-	-	-	+	-	-	-	-	-	-	-	-	-	-	-	-	-	-	-	-	-	-	-	-	-	-	-	-	+	+	-	-	-	-	-	-	-	-	-	-	-	-
3432	5372	Phage protein	3	-	-	-	-	-	-	-	-	-	-	-	-	-	-	+	-	-	-	+	-	-	-	-	-	-	-	-	-	-	-	+	-	-	-	-	-	-	-	-	-	-	-	-	-	-	-	-	-	-	-	-	-	-
3433	5373	Fibronectin-binding protein	3	-	-	-	-	-	-	-	-	-	-	-	-	-	-	+	-	-	-	-	-	-	-	-	-	-	-	-	-	-	-	-	-	-	-	-	-	-	-	-	+	+	-	-	-	-	-	-	-	-	-	-	-	-
3434	5374	Phage terminase large subunit	3	-	-	-	-	-	-	-	-	-	-	-	-	-	-	-	-	-	-	-	-	-	-	-	-	-	-	-	-	-	-	-	-	-	-	-	-	-	-	-	-	+	-	-	-	-	-	-	-	-	+	+	-	-
3435	5375	Gp9 protein	3	-	-	-	-	-	-	-	-	-	-	-	-	-	-	-	-	-	-	-	-	-	-	-	-	-	-	-	-	-	-	-	-	-	-	-	-	-	-	-	-	+	-	-	-	-	-	-	-	-	+	+	-	-
3436	5378	Phage major capsid protein #Fam0013	3	-	-	-	-	-	-	-	-	-	-	-	-	-	-	-	-	-	-	-	-	-	-	-	-	-	-	-	-	-	-	-	-	-	-	-	-	-	-	-	-	+	-	-	-	-	-	-	-	-	+	+	-	-
3437	5379	Mobile element protein	3	-	-	-	-	-	-	-	-	-	-	-	-	-	-	-	-	-	-	-	-	-	-	-	-	-	-	-	-	-	-	-	-	-	-	-	-	-	-	-	-	+	-	-	-	+	-	-	-	+	-	-	-	-
3438	5380	hypothetical protein	3	-	-	-	-	-	-	-	-	-	-	-	-	-	-	-	-	-	-	-	-	-	-	-	-	-	-	-	-	-	-	-	-	-	-	-	-	-	-	-	-	+	-	-	-	-	-	-	-	-	+	+	-	-
3439	5381	hypothetical protein	3	-	-	-	-	-	-	-	-	-	-	-	-	-	-	-	-	-	-	-	-	-	-	-	-	-	-	-	-	-	-	-	-	-	-	-	-	-	-	-	-	+	-	-	-	-	-	-	-	-	+	+	-	-
3440	5384	hypothetical protein	3	-	-	-	-	-	-	-	-	-	-	-	-	-	-	-	-	-	-	-	-	-	-	-	-	-	-	-	-	-	-	-	-	-	-	-	-	-	-	-	-	+	-	-	-	-	-	-	-	-	+	+	-	-
3441	5385	Prophage Clp protease-like protein	3	-	-	-	-	-	-	-	-	-	-	-	-	-	-	-	-	-	-	-	-	-	-	-	-	-	-	-	-	-	-	-	-	-	-	-	-	-	-	-	-	+	-	-	-	-	-	-	-	-	+	+	-	-
3442	5386	hypothetical protein	3	-	-	-	-	-	-	-	-	-	-	-	-	-	-	-	-	-	-	-	-	-	-	-	-	-	-	-	-	-	-	-	-	-	-	-	-	-	-	-	-	+	-	-	-	-	-	-	-	-	+	+	-	-
3443	5389	major tail protein b	3	-	-	-	-	-	-	-	-	-	-	-	-	-	-	-	-	-	-	-	-	-	-	-	-	-	-	-	-	-	-	-	-	-	-	-	-	-	-	-	-	+	-	-	-	-	-	-	-	-	+	+	-	-
3444	5390	Portal protein	3	-	-	-	-	-	-	-	-	-	-	-	-	-	-	-	-	-	-	-	-	-	-	-	-	-	-	-	-	-	-	-	-	-	-	-	-	-	-	-	-	+	-	-	-	-	-	-	-	-	+	+	-	-
3445	5393	scaffold protein	3	-	-	-	-	-	-	-	-	-	-	-	-	-	-	-	-	-	-	-	-	-	-	-	-	-	-	-	-	-	-	-	+	+	-	-	-	-	-	-	+	-	-	-	-	-	-	-	-	-	-	-	-	-
3446	5395	hypothetical protein	3	-	-	-	-	-	-	-	-	-	-	-	-	-	-	-	-	-	-	-	-	-	-	-	-	-	-	-	-	-	-	-	+	+	-	-	-	-	-	-	+	-	-	-	-	-	-	-	-	-	-	-	-	-
3447	5396	hypothetical protein	3	-	-	-	-	-	-	-	-	-	-	-	-	-	-	-	-	-	-	-	-	-	-	-	-	-	-	-	-	-	-	-	+	+	-	-	-	-	-	-	-	-	-	-	-	-	-	-	-	-	+	-	-	-
3448	5400	hypothetical protein	3	-	-	-	-	-	-	-	-	-	-	-	-	-	-	-	-	-	-	-	-	-	-	-	-	-	-	-	-	-	-	-	+	+	-	-	-	-	-	-	-	-	-	-	-	-	-	-	-	-	+	-	-	-
3449	5401	hypothetical protein	3	-	-	-	-	-	-	-	-	-	-	-	-	-	-	-	-	-	-	-	-	-	-	-	-	-	-	-	-	-	-	-	+	+	+	-	-	-	-	-	-	-	-	-	-	-	-	-	-	-	-	-	-	-
3450	5404	hypothetical protein	3	-	-	-	-	-	-	-	-	-	-	-	-	-	-	-	-	-	-	-	-	-	-	-	-	-	-	-	-	-	-	-	+	+	-	-	-	-	-	-	+	-	-	-	-	-	-	-	-	-	-	-	-	-
3451	5405	hypothetical protein	3	-	-	-	-	-	-	-	-	-	-	-	-	-	-	-	-	-	-	-	-	-	-	-	-	-	-	-	-	-	-	-	+	+	-	-	-	-	-	-	+	-	-	-	-	-	-	-	-	-	-	-	-	-
3452	5406	hypothetical protein	3	-	-	-	-	-	-	-	-	-	-	-	-	-	-	-	-	-	-	-	-	-	-	-	-	-	-	-	-	-	+	+	+	-	-	-	-	-	-	-	-	-	-	-	-	-	-	-	-	-	-	-	-	-
3453	5413	Putative type II restriction enzyme	3	-	-	-	-	-	-	-	-	-	-	-	-	-	-	-	-	-	-	-	-	-	-	-	-	-	-	-	-	-	-	-	+	+	+	-	-	-	-	-	-	-	-	-	-	-	-	-	-	-	-	-	-	-
3454	5414	hypothetical protein	3	-	-	-	-	-	-	-	-	-	-	-	-	-	-	-	-	-	-	-	-	-	-	-	-	-	-	-	-	-	+	-	+	-	+	-	-	-	-	-	-	-	-	-	-	-	-	-	-	-	-	-	-	-
3455	5416	hypothetical protein	3	-	-	-	-	-	-	-	-	-	-	-	-	-	-	-	-	-	-	-	-	-	-	-	-	-	-	-	-	-	-	-	+	+	-	-	-	-	-	-	-	-	-	-	-	-	-	-	+	-	-	-	-	-
3456	5418	hypothetical protein	3	-	-	-	-	-	-	-	-	-	-	-	-	-	-	-	-	-	-	-	-	-	-	-	-	-	-	-	-	-	-	-	+	+	+	-	-	-	-	-	-	-	-	-	-	-	-	-	-	-	-	-	-	-
3457	5444	Type I restriction-modification system	3	-	-	-	-	-	-	-	-	-	-	-	-	-	-	-	-	-	-	-	-	-	-	-	-	-	-	-	-	-	-	-	-	-	-	-	-	-	-	-	+	-	-	-	-	-	-	+	-	-	-	+	-	-
3458	5448	hypothetical protein	3	-	-	-	-	-	-	-	-	-	-	-	-	-	-	-	-	-	-	-	-	-	-	-	-	-	-	-	-	-	-	-	-	-	-	-	-	-	-	-	-	-	-	-	-	-	-	+	-	+	-	+	-	-
3459	5449	hypothetical protein	3	-	-	-	-	-	-	-	-	-	-	-	-	-	-	-	-	-	-	-	-	-	-	-	-	-	-	-	-	-	-	-	-	-	-	-	-	-	-	-	-	-	-	-	-	-	-	+	-	+	-	+	-	-
3460	5466	Predicted hydrolase (HAD superfamily)	3	-	-	-	-	-	-	-	-	-	-	-	-	-	-	-	-	-	-	-	-	-	-	-	-	-	-	-	-	+	+	+	-	-	-	-	-	-	-	-	-	-	-	-	-	-	-	-	-	-	-	-	-	-
3461	5469	hypothetical protein	3	-	-	-	-	-	-	-	-	-	-	-	-	-	-	-	-	-	-	-	-	-	-	-	-	-	-	-	-	-	-	-	-	-	-	-	-	-	-	-	-	-	-	+	-	-	-	-	+	-	-	-	+	-
3462	5492	Type I restriction-modification system, specificity subunit S (EC 3.1.21.3)	3	-	-	-	-	-	-	-	-	-	-	-	-	-	-	-	-	-	-	-	-	-	-	-	-	-	-	-	-	-	-	-	-	-	-	-	-	-	-	-	-	-	-	-	-	-	-	+	-	+	-	-	+	-
3463	5493	Macrolide-efflux protein	3	-	-	-	-	-	-	-	-	+	-	-	-	-	-	-	-	-	-	-	-	-	-	-	-	-	-	-	-	-	-	-	-	-	-	-	-	-	-	-	-	-	-	-	-	-	-	+	-	+	-	-	-	-
3464	5494	Tetracycline resistence protein, metal-tetracycline/H+ antiporter	3	-	-	-	-	-	-	-	-	-	-	+	-	-	-	-	-	-	-	-	-	-	-	-	-	-	-	-	-	-	-	-	-	-	-	-	-	-	-	-	-	-	-	-	-	+	-	+	-	-	-	-	-	-
3465	5499	Aminoglycoside 3'-phosphotransferase (EC 2.7.1.95)	3	-	-	-	-	-	-	-	+	+	-	-	-	-	-	-	-	-	-	-	-	-	-	-	-	-	-	-	-	-	-	-	-	-	-	-	-	-	-	-	-	-	-	-	-	-	-	-	-	-	+	-	-	-
3466	5503	Phage tail length tape-measure protein	3	-	-	-	-	-	-	-	-	-	-	-	-	-	-	-	-	-	-	+	-	-	-	-	-	-	-	-	-	-	-	-	-	-	-	-	-	-	-	-	-	-	-	-	-	-	-	-	-	+	+	-	-	-
3467	5552	hypothetical protein	3	-	-	-	-	-	-	-	-	-	-	-	-	-	-	-	+	-	-	-	-	-	-	-	-	-	-	-	-	-	-	-	-	-	-	-	-	-	-	-	-	+	-	-	+	-	-	-	-	-	-	-	-	-
3468	5553	hypothetical protein	3	-	-	-	-	-	-	-	-	-	-	-	-	-	-	-	-	-	-	-	+	+	+	-	-	-	-	-	-	-	-	-	-	-	-	-	-	-	-	-	-	-	-	-	-	-	-	-	-	-	-	-	-	-
3469	5582	Phage protein	3	-	-	-	-	-	-	-	-	-	-	-	-	-	-	-	-	-	-	-	-	-	-	-	-	-	-	+	+	+	-	-	-	-	-	-	-	-	-	-	-	-	-	-	-	-	-	-	-	-	-	-	-	-
3470	5584	pXO1-38	3	-	-	-	-	-	-	-	-	-	-	-	-	-	-	-	-	-	-	-	+	+	+	-	-	-	-	-	-	-	-	-	-	-	-	-	-	-	-	-	-	-	-	-	-	-	-	-	-	-	-	-	-	-
3471	5936	1,3-propanediol dehydrogenase (EC 1.1.1.202)	3	-	-	-	-	-	-	-	-	-	-	-	-	-	-	-	-	-	-	-	+	-	-	-	+	-	+	-	-	-	-	-	-	-	-	-	-	-	-	-	-	-	-	-	-	-	-	-	-	-	-	-	-	-
3472	5961	hypothetical protein	3	+	+	-	-	-	-	-	-	-	-	+	-	-	-	-	-	-	-	-	-	-	-	-	-	-	-	-	-	-	-	-	-	-	-	-	-	-	-	-	-	-	-	-	-	-	-	-	-	-	-	-	-	-
3473	5969	hypothetical protein	3	-	-	-	-	-	-	-	-	-	-	-	-	-	-	-	-	+	-	-	-	-	-	-	-	-	-	-	-	-	-	-	-	-	-	-	-	-	-	-	-	-	-	-	-	-	-	+	-	-	+	-	-	-
3474	5970	hypothetical protein	3	-	-	-	-	-	-	-	-	-	-	-	-	-	-	-	-	+	+	-	-	-	-	-	-	-	-	-	-	-	-	-	-	-	-	-	-	-	-	-	+	-	-	-	-	-	-	-	-	-	-	-	-	-
3475	5971	hypothetical protein	3	-	-	-	-	-	-	-	-	-	-	-	-	-	-	-	-	+	+	-	-	-	-	-	-	-	-	-	-	-	-	-	-	-	-	-	-	-	-	-	-	-	-	-	-	-	-	-	+	-	-	-	-	-
3476	5972	hypothetical protein	3	-	-	-	-	-	-	-	-	-	-	-	-	-	-	-	-	+	+	-	-	-	-	-	-	-	-	-	-	-	-	-	-	-	-	-	-	-	-	-	+	-	-	-	-	-	-	-	-	-	-	-	-	-
3477	5973	hypothetical protein	3	-	-	-	-	-	-	-	-	-	-	-	-	-	-	-	-	+	+	-	-	-	-	-	-	-	-	-	-	+	-	-	-	-	-	-	-	-	-	-	-	-	-	-	-	-	-	-	-	-	-	-	-	-
3478	5975	hypothetical protein	3	-	-	-	-	-	-	-	-	-	-	-	-	-	-	-	-	+	+	-	-	-	-	-	-	-	-	-	-	-	-	-	-	-	-	-	-	-	-	+	-	-	-	-	-	-	-	-	-	-	-	-	-	-
3479	5976	hypothetical protein	3	-	-	-	-	-	-	-	-	-	-	-	-	-	-	-	-	+	+	-	-	-	-	-	-	-	-	-	-	-	-	-	-	-	-	-	-	-	-	-	+	-	-	-	-	-	-	-	-	-	-	-	-	-
3480	5977	hypothetical protein	3	-	-	-	-	-	-	-	-	-	-	-	-	-	-	-	-	+	+	-	-	-	-	-	-	-	-	-	-	-	-	-	-	-	-	-	-	-	-	-	-	-	-	-	+	-	-	-	-	-	-	-	-	-
3481	5978	Phage major tail protein	3	-	-	-	-	-	-	-	-	-	-	-	-	-	-	-	-	-	-	-	-	-	-	-	-	-	-	-	-	-	-	-	-	-	-	-	+	-	-	-	-	-	-	+	-	-	-	-	+	-	-	-	-	-
3482	5979	hypothetical protein	3	-	-	-	-	-	-	-	-	-	-	-	-	-	-	-	-	+	+	-	-	-	-	-	-	-	-	-	-	-	-	-	-	-	-	-	-	-	-	-	+	-	-	-	-	-	-	-	-	-	-	-	-	-
3483	5981	hypothetical protein	3	-	-	-	-	-	-	-	-	-	-	-	-	-	-	-	-	+	+	-	-	-	-	-	-	-	-	-	-	-	-	-	-	-	-	-	-	-	-	-	-	-	-	-	-	+	-	-	-	-	-	-	-	-
3484	5983	hypothetical protein	3	-	-	-	-	-	-	-	-	-	-	-	-	-	-	-	+	+	+	-	-	-	-	-	-	-	-	-	-	-	-	-	-	-	-	-	-	-	-	-	-	-	-	-	-	-	-	-	-	-	-	-	-	-
3485	5984	Glycerophosphoryl diester phosphodiesterase (EC 3.1.4.46)	3	-	-	-	-	-	-	-	-	-	-	-	-	-	-	-	-	+	+	-	-	-	-	-	-	-	-	-	-	-	-	-	-	-	-	-	-	-	-	-	-	-	-	-	-	-	+	-	-	-	-	-	-	-
3486	5985	hypothetical protein	3	-	-	-	-	-	-	-	-	-	-	-	-	-	-	-	-	+	+	-	-	-	-	-	-	-	-	-	-	-	-	-	-	-	-	-	-	+	-	-	-	-	-	-	-	-	-	-	-	-	-	-	-	-
3487	5986	hypothetical protein	3	-	-	-	-	-	-	-	-	-	-	-	-	-	-	-	-	+	+	-	-	-	-	-	-	-	-	-	-	-	-	-	-	-	-	-	-	-	-	-	-	-	-	-	-	-	-	-	-	-	-	+	-	-
3488	5987	Transcriptional regulator, XRE family	3	-	-	-	-	-	-	-	-	-	-	-	-	-	-	-	-	+	+	-	-	-	-	-	-	-	-	-	-	-	-	-	-	-	-	-	-	-	-	+	-	-	-	-	-	-	-	-	-	-	-	-	-	-
3489	5988	hypothetical protein	3	-	-	-	-	-	-	-	-	-	-	-	-	-	-	-	-	+	+	-	-	-	-	-	-	-	-	-	-	-	-	-	-	-	-	-	-	+	-	-	-	-	-	-	-	-	-	-	-	-	-	-	-	-
3490	5990	hypothetical protein	3	-	-	-	-	-	-	-	-	-	-	-	-	-	-	-	+	+	+	-	-	-	-	-	-	-	-	-	-	-	-	-	-	-	-	-	-	-	-	-	-	-	-	-	-	-	-	-	-	-	-	-	-	-
3491	5991	hypothetical protein	3	-	-	-	-	-	-	-	-	-	-	-	-	-	-	-	-	+	+	-	-	-	-	-	-	-	-	-	-	-	-	-	-	-	-	-	-	-	-	-	-	-	-	-	-	-	-	+	-	-	-	-	-	-
3492	5992	ORF149	3	-	-	-	-	-	-	-	-	-	-	-	-	-	-	-	-	+	+	-	-	-	-	-	-	-	-	-	-	-	-	-	-	-	-	-	-	-	-	-	+	-	-	-	-	-	-	-	-	-	-	-	-	-
3493	5993	Acetyltransferase, GNAT family	3	-	-	-	-	-	-	+	-	-	-	-	-	-	-	-	-	+	+	-	-	-	-	-	-	-	-	-	-	-	-	-	-	-	-	-	-	-	-	-	-	-	-	-	-	-	-	-	-	-	-	-	-	-
3494	5994	hypothetical protein	3	-	-	-	-	-	-	-	-	-	-	-	-	-	-	-	-	+	+	-	-	-	-	-	-	-	-	-	-	-	-	-	-	-	-	-	-	+	-	-	-	-	-	-	-	-	-	-	-	-	-	-	-	-
3495	5995	hypothetical protein	3	-	-	-	-	-	-	-	-	-	-	-	-	-	-	-	-	+	+	-	-	-	-	-	-	-	-	-	-	-	-	-	-	-	-	-	-	-	-	-	+	-	-	-	-	-	-	-	-	-	-	-	-	-
3496	5997	Acetyltransferase	3	-	-	-	-	-	-	-	-	-	-	-	-	-	-	-	-	-	-	-	-	-	-	-	-	+	+	-	-	-	-	-	-	-	+	-	-	-	-	-	-	-	-	-	-	-	-	-	-	-	-	-	-	-
3497	5998	hypothetical protein	3	-	-	-	-	-	-	-	-	-	-	-	-	-	-	-	-	-	-	-	-	-	-	-	-	+	-	-	-	-	-	-	-	-	-	-	-	-	+	-	-	-	-	-	-	-	-	-	-	-	-	-	-	+
3498	6000	hypothetical protein	3	-	-	-	-	-	-	-	-	-	-	-	-	-	-	-	-	-	-	-	-	-	-	-	-	+	-	-	-	-	-	-	-	-	-	-	-	-	+	-	-	-	-	-	-	-	-	-	-	-	-	-	-	+
3499	6001	hypothetical protein	3	-	-	-	-	-	-	-	-	-	-	-	-	-	-	-	-	-	-	-	-	-	-	-	-	+	-	-	-	-	-	-	-	-	-	-	-	-	+	-	-	-	-	-	-	-	-	-	-	-	-	-	-	+
3500	6002	hypothetical protein	3	-	-	-	-	-	-	-	-	-	-	-	-	-	-	-	-	-	-	-	-	-	-	-	-	+	-	-	-	-	-	-	-	+	-	-	-	+	-	-	-	-	-	-	-	-	-	-	-	-	-	-	-	-
3501	6003	hypothetical protein	3	-	-	-	-	-	-	-	-	-	-	-	-	-	-	-	-	-	-	-	-	+	-	-	-	+	-	-	-	-	-	-	-	-	-	-	-	+	-	-	-	-	-	-	-	-	-	-	-	-	-	-	-	-
3502	6004	hypothetical protein	3	-	-	-	-	-	-	-	-	-	-	-	-	-	-	-	-	-	-	-	-	-	-	-	-	+	+	-	-	-	-	-	-	-	-	-	+	-	-	-	-	-	-	-	-	-	-	-	-	-	-	-	-	-
3503	6005	hypothetical protein	3	-	-	-	-	-	-	-	-	-	-	-	-	-	-	-	-	-	-	-	-	-	-	-	-	-	-	-	-	-	-	-	-	-	-	+	-	-	-	-	-	-	-	-	-	-	-	+	-	-	+	-	-	-
3504	6008	hypothetical protein	3	-	-	-	-	-	-	-	-	-	-	-	-	-	-	-	-	-	-	+	-	-	-	-	-	-	-	-	-	-	-	-	-	-	-	+	+	-	-	-	-	-	-	-	-	-	-	-	-	-	-	-	-	-
3505	6009	hypothetical protein	3	-	-	-	-	-	-	-	-	-	-	-	-	-	-	-	-	-	-	-	-	-	-	-	-	-	-	-	-	-	-	-	-	-	-	+	+	-	-	-	+	-	-	-	-	-	-	-	-	-	-	-	-	-
3506	6014	hypothetical protein	3	-	-	-	-	-	-	-	-	-	-	-	-	-	-	+	-	-	-	-	-	-	-	-	-	-	-	-	-	-	-	-	-	-	-	+	+	-	-	-	-	-	-	-	-	-	-	-	-	-	-	-	-	-
3507	6015	hypothetical protein	3	-	-	-	-	-	-	-	-	-	-	-	-	-	-	-	-	-	-	-	-	-	-	-	-	-	-	-	-	-	-	-	-	-	-	+	-	-	-	-	-	-	-	+	-	-	-	-	+	-	-	-	-	-
3508	6017	hypothetical protein	3	-	-	-	-	-	-	-	-	-	-	-	-	-	-	-	-	-	-	-	-	-	-	-	-	-	-	-	-	-	-	+	-	-	-	+	-	-	-	-	+	-	-	-	-	-	-	-	-	-	-	-	-	-
3509	6018	hypothetical protein	3	-	-	-	-	-	-	-	-	-	-	-	-	-	-	-	+	-	-	-	-	-	-	-	-	-	-	-	-	-	-	-	-	-	-	+	+	-	-	-	-	-	-	-	-	-	-	-	-	-	-	-	-	-
3510	6021	hypothetical protein	3	-	-	-	-	-	-	-	-	-	-	-	-	-	-	-	-	-	-	-	-	-	-	-	-	-	-	-	-	-	-	-	-	-	-	+	+	-	-	-	-	-	-	-	-	+	-	-	-	-	-	-	-	-
3511	6023	Multi antimicrobial extrusion protein MatE	3	-	-	-	-	-	-	-	-	-	-	-	-	-	-	-	-	-	-	-	-	-	-	-	-	-	-	-	-	+	-	-	-	-	-	+	+	-	-	-	-	-	-	-	-	-	-	-	-	-	-	-	-	-
3512	6024	hypothetical protein	3	-	-	-	-	-	-	-	-	-	-	-	-	-	-	-	-	-	-	-	-	-	-	-	-	-	-	-	-	-	-	-	-	-	-	+	+	-	-	-	-	-	-	-	-	-	-	-	+	-	-	-	-	-
3513	6025	hypothetical protein	3	-	-	-	-	-	-	-	-	-	-	-	-	-	-	-	-	-	-	-	-	-	-	-	-	-	-	-	-	-	-	-	-	-	-	+	+	-	-	-	-	-	+	-	-	-	-	-	-	-	-	-	-	-
3514	6027	hypothetical protein	3	-	-	-	-	-	-	-	-	-	-	-	-	-	-	+	-	-	-	-	-	-	-	-	-	-	-	-	-	-	-	-	-	-	-	+	-	-	-	-	-	-	+	-	-	-	-	-	-	-	-	-	-	-
3515	6028	hypothetical protein	3	-	-	-	-	-	-	-	-	-	-	-	-	-	-	-	-	-	-	-	-	-	-	-	-	-	-	-	-	-	-	-	-	-	-	+	+	+	-	-	-	-	-	-	-	-	-	-	-	-	-	-	-	-
3516	6030	hypothetical protein	3	-	-	-	-	-	-	-	-	-	-	-	-	-	-	-	-	-	-	-	-	-	-	-	-	-	-	-	-	-	-	-	-	-	-	+	+	-	-	-	-	-	-	-	-	+	-	-	-	-	-	-	-	-
3517	6031	hypothetical protein	3	-	-	-	-	-	-	-	-	-	-	-	-	-	-	-	-	-	-	-	-	-	-	-	-	-	-	-	-	-	-	-	-	-	-	+	+	-	-	-	-	-	-	-	-	+	-	-	-	-	-	-	-	-
3518	6032	Phage protein	3	-	-	-	-	-	-	-	-	-	-	-	-	-	-	-	-	-	-	-	-	-	-	-	-	-	-	-	-	-	-	-	-	-	-	+	-	-	-	-	-	-	-	-	-	-	-	+	-	-	+	-	-	-
3519	6033	hypothetical protein	3	-	-	-	-	-	-	-	-	-	-	-	-	-	-	-	-	-	-	+	-	-	-	-	-	-	-	-	-	-	-	-	-	-	-	+	-	-	-	-	-	-	-	-	-	-	-	+	-	-	-	-	-	-
3520	6034	hypothetical protein	3	-	-	-	-	-	-	-	-	-	-	-	-	-	-	-	-	-	-	-	-	-	-	-	-	-	-	-	-	-	-	-	-	-	-	+	+	-	-	-	-	-	-	-	-	+	-	-	-	-	-	-	-	-
3521	6035	hypothetical protein	3	-	-	-	-	-	-	-	-	-	-	-	-	-	-	-	-	-	-	-	-	-	-	-	-	-	-	-	-	-	-	-	-	-	-	-	+	-	-	-	-	-	-	+	-	-	-	-	+	-	-	-	-	-
3522	6036	Phage protein	3	-	-	-	-	-	-	-	-	-	-	-	-	-	-	-	-	-	-	-	-	-	-	-	-	-	-	-	-	-	-	-	-	-	-	-	+	-	-	-	-	-	-	+	-	-	-	-	+	-	-	-	-	-
3523	6037	5'-nucleotidase (EC 3.1.3.5)	3	-	-	-	-	-	-	-	-	-	-	-	-	-	-	-	-	-	-	-	-	-	-	-	-	-	-	-	-	-	-	-	-	+	-	-	+	-	-	-	-	-	-	-	-	-	+	-	-	-	-	-	-	-
3524	6038	hypothetical protein	3	-	-	-	-	-	-	-	-	-	-	-	-	-	-	-	-	-	-	-	-	-	-	-	-	-	-	-	-	-	-	-	-	-	-	-	+	-	-	-	-	-	-	+	-	-	-	-	+	-	-	-	-	-
3525	6039	hypothetical protein	3	-	-	-	-	-	-	-	-	-	-	-	-	-	-	-	-	-	-	-	-	-	-	-	-	-	-	-	-	-	-	-	-	-	-	-	+	-	-	-	-	-	-	+	-	-	-	-	+	-	-	-	-	-
3526	6040	hypothetical protein	3	-	-	-	-	-	-	-	-	-	-	-	-	-	-	-	-	-	-	-	-	-	-	-	-	-	-	-	-	-	-	-	-	-	-	-	+	-	-	-	-	-	-	+	-	-	-	-	+	-	-	-	-	-
3527	6041	SNF2 family protein	3	-	-	-	-	-	-	-	-	-	-	-	-	-	-	-	-	-	-	-	-	+	+	-	-	-	-	-	-	-	-	-	-	-	-	-	+	-	-	-	-	-	-	-	-	-	-	-	-	-	-	-	-	-
3528	6042	phage capsid protein	3	-	-	-	-	-	-	-	-	-	-	-	-	-	-	-	-	-	-	-	-	-	-	-	-	-	-	-	-	-	-	-	-	-	-	-	+	-	-	-	-	-	-	+	-	-	-	-	+	-	-	-	-	-
3529	6043	DNA-cytosine methyltransferase (EC 2.1.1.37)	3	-	-	-	-	-	-	-	-	-	-	-	-	-	-	-	-	-	-	-	-	+	-	-	-	-	-	-	-	-	-	-	-	-	-	-	+	-	-	-	-	-	-	-	-	-	-	-	-	-	+	-	-	-
3530	6047	Phage head maturation protease	3	-	-	-	-	-	-	-	-	-	-	-	-	-	-	-	-	-	-	-	-	-	-	-	-	-	-	-	-	-	-	-	-	-	-	-	+	-	-	-	-	-	-	+	-	-	-	-	+	-	-	-	-	-
3531	6051	hypothetical protein	3	-	-	-	-	-	-	-	-	-	-	-	-	-	-	-	-	-	-	-	-	-	-	-	-	-	-	-	-	-	-	-	-	-	-	-	-	-	-	-	-	-	+	-	+	+	-	-	-	-	-	-	-	-
3532	6053	Sucrose-6-phosphate hydrolase (EC 3.2.1.26)	3	-	-	-	-	-	-	-	-	-	-	-	-	-	-	-	-	-	-	-	-	-	-	+	+	-	-	-	-	-	-	-	-	-	-	-	-	-	-	-	-	-	-	-	-	-	-	-	-	+	-	-	-	-
3533	6059	Cysteine synthase (EC 2.5.1.47)	3	-	-	-	-	-	-	-	-	-	-	-	-	-	-	-	-	-	-	-	-	-	-	+	-	-	-	-	-	-	-	+	-	-	-	-	+	-	-	-	-	-	-	-	-	-	-	-	-	-	-	-	-	-
3534	6063	Muramidase-released protein precursor (136 kDa surface protein)	3	-	-	-	-	-	-	-	-	+	-	-	-	-	-	-	+	-	-	-	+	-	-	-	-	-	-	-	-	-	-	-	-	-	-	-	-	-	-	-	-	-	-	-	-	-	-	-	-	-	-	-	-	-
3535	6065	hypothetical protein	3	-	-	-	-	-	-	-	-	-	-	-	-	-	-	-	+	-	-	-	-	-	-	-	-	-	-	-	-	-	-	-	-	-	-	-	-	+	+	-	-	-	-	-	-	-	-	-	-	-	-	-	-	-
3536	6066	hypothetical protein	3	-	-	-	-	-	-	-	-	-	-	-	-	-	-	-	+	-	-	-	-	-	-	-	-	-	-	-	-	-	-	+	+	-	-	-	-	-	-	-	-	-	-	-	-	-	-	-	-	-	-	-	-	-
3537	6067	hypothetical protein	3	-	-	-	-	-	-	-	-	-	-	-	-	-	-	-	+	-	-	-	-	-	-	-	-	-	-	-	-	-	-	-	-	-	-	-	-	-	-	-	-	-	-	-	-	-	-	+	-	-	+	-	-	-
3538	6068	hypothetical protein	3	-	-	-	-	-	-	-	-	-	-	-	-	-	-	-	+	-	-	-	-	-	-	-	-	-	-	-	-	-	-	-	-	-	-	-	-	+	+	-	-	-	-	-	-	-	-	-	-	-	-	-	-	-
3539	6069	hypothetical protein	3	-	-	-	-	-	-	-	-	-	-	-	-	-	-	-	+	-	-	-	-	-	-	-	-	-	-	-	-	-	-	-	-	-	-	-	-	+	+	-	-	-	-	-	-	-	-	-	-	-	-	-	-	-
3540	6070	hypothetical protein	3	-	-	-	-	-	-	-	-	-	-	-	-	-	-	+	+	-	-	+	-	-	-	-	-	-	-	-	-	-	-	-	-	-	-	-	-	-	-	-	-	-	-	-	-	-	-	-	-	-	-	-	-	-
3541	6071	hypothetical protein	3	-	-	-	-	-	-	-	-	-	-	-	-	-	-	-	+	-	-	-	-	-	-	-	-	-	-	-	-	-	-	-	-	-	-	-	-	-	-	-	-	-	-	-	+	+	-	-	-	-	-	-	-	-
3542	6073	Export ABC transporter ATP-binding protein	3	-	-	-	-	-	-	-	-	-	-	-	-	-	-	-	-	-	-	-	-	-	-	-	-	-	-	-	-	-	-	-	-	-	-	-	-	-	-	-	-	+	-	-	-	-	+	-	+	-	-	-	-	-
3543	6077	5'-nucleotidase (EC 3.1.3.5)	3	-	-	-	-	-	-	-	-	-	-	-	-	-	-	-	-	-	-	-	-	-	-	-	-	-	-	-	-	-	-	-	-	-	-	-	-	-	-	-	+	-	-	-	-	-	-	-	-	-	+	-	-	+
3544	6081	hypothetical protein	3	-	-	-	-	-	-	-	-	-	-	-	-	-	-	-	-	-	-	-	-	-	-	-	-	-	-	+	+	-	-	-	-	-	-	-	-	-	-	-	-	-	-	-	-	-	-	-	-	-	-	-	-	+
3545	6082	hypothetical protein	3	-	-	-	-	-	-	-	-	-	-	-	-	-	-	-	-	-	-	-	-	-	-	-	-	-	-	-	-	-	-	-	-	-	-	-	-	-	+	+	-	-	-	-	-	-	-	-	-	-	-	-	-	+
3546	6085	DNA-cytosine methyltransferase (EC 2.1.1.37)	3	-	-	-	-	-	-	-	-	-	-	-	-	-	-	-	-	-	-	-	-	-	-	-	-	-	-	-	-	-	-	-	-	-	-	-	-	+	-	-	-	+	-	-	-	-	-	-	-	-	-	-	-	+
3547	6092	hypothetical protein	3	-	-	-	-	-	-	-	-	-	-	-	-	-	-	-	-	-	-	-	-	-	-	-	-	-	-	-	-	-	-	-	-	-	-	-	-	-	-	-	-	-	-	-	+	-	-	-	+	-	-	+	-	-
3548	6095	hypothetical protein	3	-	-	-	-	-	-	-	-	-	-	-	-	-	-	-	-	-	-	-	-	-	-	-	-	-	-	-	-	-	-	-	-	-	-	-	-	-	-	-	-	-	-	-	+	+	-	-	-	-	-	-	+	-
3549	6100	hypothetical protein	3	-	-	-	-	-	-	-	-	-	-	-	-	-	-	-	-	-	-	-	-	-	-	-	-	-	-	-	-	-	-	-	-	-	-	-	-	-	-	-	-	-	-	+	+	+	-	-	-	-	-	-	-	-
3550	6101	cell surface protein precursor	3	-	-	-	-	-	-	-	-	-	-	-	-	-	-	-	-	-	-	-	-	-	-	-	-	-	-	-	-	-	-	-	-	-	-	-	-	-	-	-	-	-	-	-	+	-	-	-	+	-	-	+	-	-
3551	6102	prophage Lp2 protein 14	3	-	-	-	-	-	-	-	-	-	-	-	-	-	-	-	-	-	-	-	-	-	-	-	-	-	-	-	-	-	-	-	-	-	-	-	-	-	-	-	-	-	-	+	+	+	-	-	-	-	-	-	-	-
3552	6103	hypothetical protein	3	-	-	-	-	-	-	-	-	-	-	-	-	-	-	-	-	-	-	-	-	-	-	-	-	-	-	-	-	-	-	-	-	-	-	-	-	-	-	+	-	-	-	-	+	-	-	-	-	-	-	-	+	-
3553	6104	hypothetical protein	3	-	-	-	+	-	-	-	-	-	-	-	-	-	-	-	-	-	-	-	-	-	-	-	-	-	-	-	-	-	-	-	-	-	-	-	-	-	-	-	+	-	-	-	+	-	-	-	-	-	-	-	-	-
3554	6105	hypothetical protein	3	-	-	-	-	-	-	-	-	-	-	-	-	-	-	-	-	-	-	-	-	-	-	-	-	-	-	-	-	-	-	-	-	-	-	-	-	-	-	-	-	-	-	+	+	+	-	-	-	-	-	-	-	-
3555	6106	hypothetical protein	3	-	-	-	-	-	-	-	-	-	-	-	-	-	-	-	-	-	-	-	-	-	-	-	-	-	-	-	-	-	-	-	-	-	-	-	-	+	-	+	-	-	-	+	-	-	-	-	-	-	-	-	-	-
3556	6107	hypothetical protein	3	-	-	-	-	-	-	-	-	-	-	-	-	-	-	-	-	-	-	-	-	-	-	-	-	-	-	-	-	-	-	-	-	-	-	-	-	-	-	-	-	-	-	-	+	-	-	-	+	-	-	+	-	-
3557	6109	hypothetical protein	3	-	-	-	-	-	-	-	-	-	-	-	-	-	-	-	-	-	-	-	-	-	-	-	-	-	-	-	-	-	-	-	-	-	-	-	-	-	-	-	-	-	-	-	+	-	-	-	+	-	-	+	-	-
3558	6111	hypothetical protein	3	-	-	-	-	-	-	-	-	-	-	-	-	-	-	-	-	-	-	+	-	-	-	-	-	-	-	-	-	-	-	-	-	-	-	-	-	-	-	-	-	-	-	-	+	-	-	-	+	-	-	-	-	-
3559	6112	hypothetical protein	3	-	-	-	-	-	-	-	-	-	-	-	-	-	-	-	-	-	-	-	-	-	-	-	-	-	-	-	-	-	-	-	-	-	-	-	-	-	-	-	-	-	-	+	+	+	-	-	-	-	-	-	-	-
3560	6113	hypothetical protein	3	-	-	-	-	-	-	-	-	-	-	-	-	-	-	-	-	-	-	-	-	-	-	-	-	-	-	-	-	-	-	-	-	-	-	-	-	-	-	-	-	-	-	-	+	-	-	-	+	-	-	+	-	-
3561	6114	ATP-binding region, ATPase-like	3	-	-	-	-	-	-	-	-	-	-	-	-	-	-	-	-	-	-	-	-	-	-	-	-	-	-	-	-	-	-	-	-	-	-	-	-	-	-	-	-	-	-	+	+	+	-	-	-	-	-	-	-	-
3562	6118	hypothetical protein	3	-	-	-	-	-	-	-	-	-	-	-	-	-	-	-	-	-	-	-	-	-	-	-	-	-	-	-	-	-	-	-	-	-	-	-	-	-	+	-	-	-	-	-	-	-	+	-	-	-	-	-	+	-
3563	6119	Site-specific recombinase	3	-	-	-	-	-	-	-	-	-	-	-	-	-	-	-	-	-	-	-	-	-	-	-	-	-	-	-	-	-	-	-	-	-	-	-	-	-	+	-	-	-	-	-	-	-	-	+	-	+	-	-	-	-
3564	6120	hypothetical protein	3	-	-	-	-	-	-	-	-	-	-	-	-	-	-	-	-	-	-	-	-	-	-	-	-	-	-	-	-	-	-	-	-	-	-	-	-	-	+	+	-	+	-	-	-	-	-	-	-	-	-	-	-	-
3565	6122	hypothetical protein	3	-	-	-	-	-	-	-	-	-	-	-	-	-	-	-	-	-	-	-	-	-	-	-	-	-	-	-	-	-	-	-	-	-	-	-	-	-	+	+	-	+	-	-	-	-	-	-	-	-	-	-	-	-
3566	6123	hypothetical protein	3	-	-	-	-	-	-	-	-	-	-	-	-	-	-	-	-	-	-	-	-	-	-	-	-	-	-	-	-	-	-	-	-	-	-	-	-	-	+	-	-	-	-	+	-	-	-	+	-	-	-	-	-	-
3567	6132	hypothetical protein	3	-	-	-	-	-	-	-	-	-	-	-	-	-	-	-	-	-	-	-	-	-	-	-	-	-	-	-	-	-	-	-	+	+	-	-	-	-	+	-	-	-	-	-	-	-	-	-	-	-	-	-	-	-
3568	6133	transcriptional regulator, Cro/CI family, putative	3	-	-	-	-	-	-	-	-	-	-	-	-	-	-	-	-	-	-	-	-	-	-	-	-	-	-	-	-	-	-	-	-	-	-	-	-	-	+	-	-	-	-	-	-	-	-	-	-	+	-	+	-	-
3569	6134	hypothetical protein	3	-	-	-	-	-	-	-	-	-	-	-	-	-	-	-	-	-	-	-	-	-	-	-	-	-	-	-	-	+	-	-	-	-	-	-	-	-	-	+	+	-	-	-	-	-	-	-	-	-	-	-	-	-
3570	6137	Arsenate reductase and related proteins	3	-	-	-	-	-	-	-	-	-	-	-	-	-	-	-	-	-	-	-	-	-	-	-	-	-	-	-	-	-	-	-	-	-	-	-	-	-	+	-	-	-	-	-	-	-	+	-	-	-	-	-	+	-
3571	6138	Cobalt-zinc-cadmium resistance protein CzcD	3	-	-	-	-	-	-	-	-	-	-	-	-	-	-	-	-	-	-	-	-	-	-	-	-	-	-	-	-	+	-	-	-	-	-	-	-	-	+	-	+	-	-	-	-	-	-	-	-	-	-	-	-	-
3572	6144	hypothetical protein	3	-	-	-	-	-	-	-	-	-	-	-	-	-	-	-	-	-	-	-	-	-	-	-	-	-	-	-	-	-	-	-	-	-	-	-	-	+	-	-	-	-	-	-	-	-	+	-	-	-	-	-	+	-
3573	6145	hypothetical protein	3	-	-	-	-	-	-	-	-	-	-	-	-	-	-	-	-	-	-	-	-	-	-	-	-	-	-	-	-	-	-	-	-	-	-	-	-	+	-	-	-	-	-	+	-	-	-	-	-	-	+	-	-	-
3574	6146	hypothetical protein	3	-	-	-	-	-	-	-	-	-	-	-	-	-	-	-	-	-	-	-	-	-	-	-	-	-	-	-	-	-	-	-	-	-	-	-	-	+	-	-	-	-	-	+	-	-	+	-	-	-	-	-	-	-
3575	6148	hypothetical protein	3	-	-	-	-	-	-	-	-	-	-	-	-	-	-	-	-	-	-	-	-	-	-	-	-	-	-	-	-	-	-	-	+	+	-	-	-	+	-	-	-	-	-	-	-	-	-	-	-	-	-	-	-	-
3576	6151	hypothetical protein	3	-	-	-	-	-	-	-	-	-	-	-	-	-	-	-	-	-	-	-	-	-	-	-	-	-	-	-	-	-	-	-	-	-	-	-	-	+	-	-	-	-	-	-	-	-	+	-	-	-	-	-	+	-

Table S5. Precence/absence of each HG among 57 *S. suis* strains

				1																13			25					28						104		94			76			53/54		Divergent *S. suis*																
Index[a]	ClutserID	Gene products	Members	98HAH33	05ZYH33	SC84	A7	SS12	P1/7	DAT273	NIAH11434	JS14	GZ1	BM407	S735	13730	MNCM07	NCTC 10446	14636	NCTC 10237	ST1	2651	89-1591	MNCM04	MNCM25	8074	D9	4961	ST3	MNCM43	DAT292	DAT260	NIAH11435	MNCM21	MNCM50	89-2479	DAT234	DAT290	93A	42A	NT77	6407	11538	8830	12814	92-1400	89-590	2726	2524	89-3576-3	4417	D12	10581	89-5259	88-5299A	R61	92-1191	14A	92-4172	22083
3577	6159	hypothetical protein	3	-	-	-	-	-	-	-	-	-	-	-	-	-	-	-	-	-	-	-	-	-	-	-	-	-	-	-	-	-	-	-	-	-	-	-	-	-	-	-	-	-	-	-	-	-	-	-	-	+	-	-	-	-	-	-	+	-	+	-	-	-	-	-
3578	6161	Mobilization protein MobM	3	-	-	-	-	-	-	-	-	-	-	-	-	-	-	-	-	-	-	-	+	-	+	-	-	-	-	-	-	-	-	-	-	-	-	-	-	-	-	-	-	-	-	-	-	-	+	-	-	-	-	-	-	-
3579	6164	transcription regulator	3	-	-	-	-	-	-	-	-	-	-	-	-	-	-	-	-	-	-	-	-	-	-	-	-	-	-	-	-	-	-	-	-	-	-	-	-	-	-	-	-	-	-	-	-	-	+	+	-	+	-	-	-	-
3580	6166	Macrolide-efflux protein	3	-	-	-	-	-	-	-	-	-	-	-	-	-	-	-	-	-	-	-	-	-	-	-	-	-	-	-	-	-	-	-	-	-	-	-	-	-	-	-	-	-	-	-	-	-	+	-	+	-	+	-	-	-
3581	6167	peptide ABC transporter ATP binding/permease protein	3	-	-	-	-	-	-	-	-	-	-	-	-	-	-	-	-	-	-	-	-	-	-	-	-	-	-	-	-	-	-	-	-	-	-	-	-	-	-	-	-	-	-	-	-	-	+	-	+	-	-	+	-	-
3582	6168	Muramidase-released protein precursor (136 kDa surface protein)	3	-	-	-	-	-	-	-	-	-	-	-	-	-	-	-	-	-	-	-	-	-	-	-	-	-	-	-	-	-	-	+	+	-	-	-	-	-	-	-	-	-	-	-	-	-	+	-	-	-	-	-	-	-
3583	6169	CAAX amino terminal protease family	3	-	-	-	-	-	-	-	-	-	-	-	-	-	-	-	-	-	-	-	-	-	-	-	-	-	-	-	-	-	-	-	-	-	-	-	-	-	-	-	-	-	-	-	-	-	+	+	+	-	-	-	-	-
3584	6178	hypothetical protein	3	-	-	-	-	-	-	-	-	-	-	-	-	-	-	-	-	-	-	-	-	-	-	-	-	-	-	-	-	-	-	-	-	-	-	-	-	-	-	-	-	-	-	-	-	-	+	-	+	-	+	-	-	-
3585	6179	Transcriptional regulator	3	-	-	-	-	-	-	-	-	-	-	-	-	-	-	-	-	-	-	-	-	-	-	-	-	-	-	-	-	-	-	-	-	-	-	-	-	-	-	-	-	-	-	-	-	-	+	+	+	-	-	-	-	-
3586	6188	Type I restriction-modification system	3	-	-	-	-	-	-	-	-	-	-	-	-	-	-	-	-	-	-	-	-	-	-	-	-	-	-	-	-	-	-	-	-	-	-	-	-	-	-	-	-	-	-	-	-	-	+	-	+	-	+	-	-	-
3587	6191	hypothetical protein	3	-	-	-	-	-	-	-	-	-	-	-	-	-	-	-	-	-	-	-	-	-	-	-	-	-	-	-	-	-	-	-	-	-	-	-	-	-	-	+	-	-	-	-	-	-	+	-	+	-	-	-	-	-
3588	6193	hypothetical protein	3	-	-	-	-	-	-	-	-	-	-	-	-	-	-	-	-	-	-	-	-	-	-	-	-	-	-	-	-	-	-	-	-	-	-	-	-	-	-	-	-	-	-	-	-	-	+	+	-	+	-	-	-	-
3589	6196	hypothetical protein	3	-	-	-	-	-	-	-	-	-	-	-	-	-	-	-	-	-	-	-	-	-	-	-	+	-	-	+	-	-	-	-	-	-	-	-	-	-	-	-	-	-	-	-	-	-	+	-	-	-	-	-	-	-
3590	6198	Putative ABC transporter (ATP-binding protein)	3	-	-	-	-	-	-	-	-	-	-	-	-	+	-	-	-	-	-	-	-	-	-	-	-	-	-	-	+	+	-	-	-	-	-	-	-	-	-	-	-	-	-	-	-	-	-	-	-	-	-	-	-	-
3591	6199	hypothetical protein	3	-	-	-	-	-	+	-	-	-	+	-	-	-	-	+	-	-	-	-	-	-	-	-	-	-	-	-	-	-	-	-	-	-	-	-	-	-	-	-	-	-	-	-	-	-	-	-	-	-	-	-	-	-
3592	6200	fibronectin-binding protein	3	-	-	-	-	-	-	-	-	-	-	-	-	-	-	+	-	-	-	-	-	-	-	-	-	-	-	-	-	-	-	-	-	-	-	-	-	-	-	-	-	-	-	-	-	-	-	-	-	-	-	-	-	-
3593	6201	hypothetical protein	3	-	-	-	-	-	-	-	-	-	-	-	-	-	-	+	-	-	-	-	-	-	-	-	-	-	-	-	-	-	-	-	-	-	-	-	-	-	-	-	+	+	-	-	-	-	-	-	-	-	-	-	-	-
3594	6202	hypothetical protein	3	-	-	-	-	-	-	-	-	-	-	-	-	-	-	-	-	-	-	-	-	-	-	-	-	-	-	-	-	-	-	-	-	-	-	+	-	-	-	-	-	-	+	-	-	-	-	-	-	+	-	-	-	-
3595	6203	hypothetical protein	3	-	-	-	-	-	-	-	-	-	-	-	-	-	-	-	-	-	-	-	-	-	-	-	-	-	-	-	-	-	-	-	-	-	-	-	-	-	-	-	-	+	-	-	-	-	-	-	-	-	+	+	-	-
3596	6204	hypothetical protein	3	-	-	-	-	-	-	-	-	-	-	+	-	-	-	-	-	-	-	-	-	-	-	-	-	-	-	-	-	-	-	-	-	-	-	-	-	-	-	-	-	+	-	-	-	+	-	-	-	-	-	-	-	-
3597	6205	hypothetical protein	3	-	-	-	-	-	-	-	-	-	-	-	-	-	-	-	-	-	-	-	-	-	-	-	-	-	-	-	-	-	-	-	-	-	-	-	-	-	-	-	-	+	-	-	-	-	-	-	-	-	+	+	-	-
3598	6207	hypothetical protein	3	-	-	-	-	-	-	-	-	-	-	-	-	-	-	-	-	-	-	-	-	-	-	-	-	-	-	-	-	-	-	-	-	-	-	-	-	-	-	-	-	+	-	-	-	-	-	-	-	-	+	+	-	-
3599	6212	hypothetical protein	3	-	-	-	-	-	-	-	-	-	-	-	-	-	-	-	-	-	-	-	-	-	-	-	-	-	-	-	-	-	-	-	-	-	-	-	-	-	-	+	-	+	-	+	-	-	-	-	-	-	-	-	-	-
3600	6214	Adenine-specific methyltransferase (EC 2.1.1.72)	3	-	-	-	-	-	-	-	-	-	-	-	-	-	-	-	-	-	-	-	-	-	-	-	-	-	-	-	-	-	-	-	-	-	-	-	-	-	-	-	-	+	-	-	-	+	-	+	-	-	-	-	-	-
3601	6216	Phage protein	3	-	-	-	-	-	-	-	-	-	-	-	-	-	-	-	-	-	-	-	-	-	-	-	-	-	-	-	-	-	-	-	-	-	-	-	-	-	-	-	-	+	-	-	-	-	-	-	-	-	+	+	-	-
3602	6217	unknown phage protein	3	-	-	-	-	-	-	-	-	-	-	-	-	-	-	-	-	-	-	-	-	-	-	-	-	-	-	-	-	-	-	-	-	-	-	-	-	-	-	-	-	+	-	-	-	+	-	+	-	-	-	-	-	-
3603	6219	hypothetical protein	3	-	-	-	-	-	-	-	-	-	-	-	-	-	-	-	-	-	-	-	-	-	-	-	-	-	-	-	-	-	-	-	-	-	-	-	-	-	-	-	-	+	-	-	-	-	-	-	-	-	+	+	-	-
3604	6220	Phage tail length tape-measure protein	3	-	-	-	-	-	-	-	-	-	-	-	-	-	-	-	-	-	-	-	-	-	-	-	-	-	-	-	-	-	-	-	-	-	-	-	-	-	-	-	-	+	-	-	-	-	-	-	-	-	+	+	-	-
3605	6222	hypothetical protein	3	-	-	-	-	-	-	-	-	-	-	-	-	-	-	-	-	-	-	-	-	-	-	-	-	-	-	-	-	-	-	-	-	-	-	-	-	-	-	-	-	+	-	+	-	-	-	-	-	-	-	-	-	+
3606	6223	hypothetical protein	3	-	-	-	-	-	-	-	-	-	-	-	-	-	-	-	-	-	-	-	-	-	-	-	-	-	-	-	-	+	-	-	-	-	-	-	-	-	-	-	-	+	-	-	-	-	-	-	-	-	-	+	-	-
3607	6225	Predicted ATP-binding protein involved in virulence	3	-	-	-	-	-	-	-	-	-	-	-	-	-	-	-	-	-	-	-	-	-	-	-	-	-	-	-	-	-	-	-	-	-	-	-	-	-	-	+	-	+	-	+	-	-	-	-	-	-	-	-	-	-
3608	6228	Accessory secretory protein Asp2	3	-	-	-	-	-	-	-	-	-	-	-	-	-	-	-	-	-	-	-	-	-	-	-	-	-	-	-	-	-	-	-	+	+	+	-	-	-	-	-	-	-	-	-	-	-	-	-	-	-	-	-	-	-
3609	6229	hypothetical protein	3	-	-	-	-	-	-	-	-	-	-	-	-	-	-	-	-	-	-	-	-	-	-	-	-	-	-	-	-	-	-	-	+	-	-	-	-	-	-	-	-	-	-	-	-	-	-	-	-	-	+	+	-	-
3610	6232	hypothetical protein	3	-	-	-	-	-	-	-	-	-	-	-	-	-	-	-	-	-	-	-	-	-	-	-	-	-	-	-	-	-	-	-	+	+	-	-	-	-	-	-	-	-	-	-	-	-	-	-	-	+	-	-	-	-
3611	6233	putative HsdR	3	-	-	-	-	-	-	-	-	-	-	-	-	-	-	-	-	-	-	-	-	-	-	-	-	-	-	-	-	-	-	-	+	+	+	-	-	-	-	-	-	-	-	-	-	-	-	-	-	-	-	-	-	-
3612	6235	Glycosyltransferases involved in cell wall biogenesis	3	-	-	-	-	-	-	-	-	-	-	-	-	-	-	-	-	-	-	-	-	-	-	-	-	-	-	-	-	-	-	-	+	+	+	-	-	-	-	-	-	-	-	-	-	-	-	-	-	-	-	-	-	-
3613	6236	phage portal protein	3	-	-	-	-	-	-	-	-	-	-	-	-	-	-	-	-	-	-	-	-	-	-	-	-	-	-	-	-	-	-	-	+	+	-	-	-	-	-	-	+	-	-	-	-	-	-	-	-	-	-	-	-	-
3614	6237	hypothetical protein	3	-	-	-	-	-	-	-	-	-	-	-	-	-	-	-	-	-	-	-	-	-	-	-	-	-	-	-	-	-	-	-	+	+	-	-	-	-	-	-	-	-	-	-	-	-	-	+	-	-	-	-	-	-
3615	6239	hypothetical protein	3	-	-	-	-	-	-	-	-	-	-	-	-	-	-	-	-	-	-	-	-	-	-	-	-	-	-	-	-	-	-	-	+	+	+	-	-	-	-	-	-	-	-	-	-	-	-	-	-	-	-	-	-	-
3616	6240	hypothetical protein	3	-	-	-	-	-	-	-	-	-	-	-	-	-	-	-	-	-	-	-	-	-	-	-	-	-	-	-	-	-	-	-	+	+	+	-	-	-	-	-	-	-	-	-	-	-	-	-	-	-	-	-	-	-
3617	6242	hypothetical protein	3	-	-	-	-	-	-	-	-	-	-	-	-	-	-	-	-	-	-	-	-	-	-	-	-	-	-	-	-	-	-	-	+	+	-	-	-	-	-	-	-	-	-	-	-	-	-	-	-	+	-	-	-	-
3618	6243	hypothetical protein	3	-	-	-	-	-	-	-	-	-	-	-	-	-	-	-	-	-	-	-	-	-	-	-	-	-	-	-	-	-	-	-	+	+	+	-	-	-	-	-	-	-	-	-	-	-	-	-	-	-	-	-	-	-
3619	6245	YbbL ABC transporter ATP-binding protein	3	-	-	-	-	-	-	-	-	-	-	-	-	-	-	-	-	-	-	-	-	-	-	-	-	-	-	-	-	-	-	-	+	-	+	-	-	-	-	-	-	-	-	-	-	-	-	-	-	-	+	-	-	-
3620	6246	hypothetical protein	3	-	-	-	-	-	-	-	-	-	-	-	-	-	-	-	-	-	-	-	-	-	-	-	-	-	-	-	-	-	+	-	+	+	-	-	-	-	-	-	-	-	-	-	-	-	-	-	-	-	-	-	-	-
3621	6249	preprotein translocase SecY family protein	3	-	-	-	-	-	-	-	-	-	-	-	-	-	-	-	-	-	-	-	-	-	-	-	-	-	-	-	-	-	-	-	+	+	+	-	-	-	-	-	-	-	-	-	-	-	-	-	-	-	-	-	-	-
3622	6250	Beta-1	3	-	-	-	-	-	-	-	-	-	-	-	-	-	-	-	-	-	-	-	-	-	-	-	-	-	-	-	-	-	-	-	+	+	+	-	-	-	-	-	-	-	-	-	-	-	-	-	-	-	-	-	-	-
3623	6251	hypothetical protein	3	-	-	-	-	-	-	-	-	-	-	-	-	-	-	-	-	-	-	+	-	-	-	-	-	-	-	-	-	-	-	-	-	-	-	-	+	+	-	-	-	-	-	-	-	-	-	-	-	-	-	-	-	-
3624	6253	Pathogenicity island SaPIn2	3	-	-	-	-	-	-	-	-	-	-	-	-	-	-	-	-	-	-	-	-	-	-	-	-	-	-	-	-	-	-	-	+	+	-	-	-	-	-	-	-	-	-	-	-	-	-	+	-	-	-	-	-	-
3625	6254	hypothetical protein	3	-	-	-	-	-	-	-	-	-	-	-	-	-	-	-	-	-	-	-	-	-	-	-	-	-	-	-	-	-	-	-	+	+	+	-	-	-	-	-	-	-	-	-	-	-	-	-	-	-	-	-	-	-
3626	6255	Pleiotropic regulator of exopolysaccharide synthesis	3	-	-	-	-	-	-	-	-	-	-	-	-	-	-	-	-	-	-	-	-	-	-	-	-	-	-	-	-	-	-	-	+	+	+	-	-	-	-	-	-	-	-	-	-	-	-	-	-	-	-	-	-	-
3627	6256	hypothetical protein	3	-	-	-	-	-	-	-	-	-	-	-	-	-	-	-	-	-	-	-	-	-	-	-	-	-	-	-	-	-	-	-	+	+	+	-	-	-	-	-	-	-	-	-	-	-	-	-	-	-	-	-	-	-
3628	6257	hypothetical protein	3	-	-	-	-	-	-	-	-	-	-	-	-	-	-	-	-	-	-	-	-	-	-	-	-	-	-	-	-	-	-	-	+	+	-	-	-	-	-	-	-	-	-	-	-	-	-	-	-	+	-	-	-	-
3629	6258	D-alanine--D-alanine ligase (EC 6.3.2.4)	3	-	-	-	-	-	-	-	-	-	-	-	-	-	-	-	-	-	-	-	-	-	-	-	-	-	-	-	-	-	-	-	+	+	+	-	-	-	-	-	-	-	-	-	-	-	-	-	-	-	-	-	-	-
3630	6259	Glycerate kinase (EC 2.7.1.31)	3	-	-	-	-	-	-	-	-	-	-	-	-	-	-	-	-	-	-	-	-	-	-	-	-	-	-	-	-	-	-	-	+	+	+	-	-	-	-	-	-	-	-	-	-	-	-	-	-	-	-	-	-	-
3631	6260	Phage protein	3	-	-	-	-	-	-	-	-	-	-	-	-	-	-	-	-	-	-	-	-	-	-	-	-	-	-	-	-	-	-	-	+	+	-	-	-	-	-	-	-	-	-	-	-	-	-	+	-	-	-	-	-	-
3632	6262	Accessory secretory protein Asp3	3	-	-	-	-	-	-	-	-	-	-	-	-	-	-	-	-	-	-	-	-	-	-	-	-	-	-	-	-	-	-	-	+	+	+	-	-	-	-	-	-	-	-	-	-	-	-	-	-	-	-	-	-	-
3633	6263	hypothetical protein	3	-	-	-	-	-	-	-	-	-	-	-	-	-	-	-	-	-	-	-	-	-	-	-	-	-	-	-	-	-	-	-	-	+	+	-	-	-	-	-	-	-	-	-	-	-	-	-	-	-	+	-	-	-
3634	6266	Uracil-xanthine permease	3	-	-	-	-	-	-	-	-	-	-	-	-	-	-	-	-	-	-	-	-	-	-	-	-	-	-	-	-	-	-	-	+	+	-	-	-	-	-	+	-	-	-	-	-	-	-	-	-	-	-	-	-	-
3635	6269	hypothetical protein	3	-	-	-	-	-	-	-	-	-	-	-	-	-	-	-	-	-	-	-	-	-	-	-	-	-	-	-	-	+	-	+	-	-	+	-	-	-	-	-	-	-	-	-	-	-	-	-	-	-	-	-	-	-
3636	6270	hypothetical protein	3	-	-	-	-	-	-	-	-	-	-	-	-	-	-	-	-	-	-	-	-	-	-	-	-	-	-	-	-	-	-	-	-	-	+	-	-	-	-	-	-	-	-	-	-	-	-	-	-	+	-	+	-	-
3637	6272	hypothetical protein	3	-	-	-	-	-	-	-	-	-	-	-	-	-	-	-	-	-	-	-	-	-	+	-	-	-	-	-	-	-	-	+	-	-	-	-	+	-	-	-	-	-	-	-	-	-	-	-	-	-	-	-	-	-
3638	6279	Phosphoglycerate/bisphosphoglycerate mutase	3	-	-	-	-	-	-	-	-	-	-	-	-	-	-	-	-	-	-	-	-	-	-	-	-	-	-	-	-	+	+	-	-	+	-	-	-	-	-	-	-	-	-	-	-	-	-	-	-	-	-	-	-	-
3639	6338	Neutral endopeptidase O (EC 3.4.24.-)	3	-	-	-	-	-	-	-	-	-	-	-	-	-	-	-	-	-	-	-	-	-	-	-	-	-	-	-	-	-	-	-	-	-	-	-	-	-	-	-	-	-	-	-	-	-	-	-	+	-	+	+	-	-
3640	6339	hypothetical protein	3	-	-	-	-	-	-	-	-	-	-	-	-	-	-	-	-	-	-	-	-	-	-	-	-	-	-	-	-	-	-	-	-	-	-	-	-	-	-	-	-	-	-	-	-	-	-	+	-	-	+	+	-	-
3641	6341	hypothetical protein	3	-	-	-	-	-	-	-	-	-	-	-	-	-	-	-	-	-	-	-	-	-	-	-	-	-	-	-	-	-	-	-	-	-	-	-	-	-	-	-	-	-	-	-	-	-	-	+	-	-	+	+	-	-
3642	6343	putative glycosyltransferase	3	-	-	-	-	-	-	-	-	-	-	-	-	-	-	-	-	-	-	-	-	-	-	-	-	-	-	-	-	-	-	-	-	-	-	-	-	-	-	-	-	-	-	-	-	-	-	+	-	-	+	+	-	-
3643	6344	hypothetical protein	3	-	-	-	-	-	-	-	-	-	-	-	-	-	-	-	-	-	-	-	-	-	-	-	-	-	-	-	-	-	-	-	-	-	-	-	-	-	-	-	-	-	-	-	-	-	-	+	-	-	+	+	-	-
3644	6345	Beta-1	3	-	-	-	-	-	-	-	-	-	-	-	-	-	-	-	-	-	-	-	-	-	-	-	-	-	-	-	-	-	-	-	-	-	-	-	-	-	-	-	-	-	-	-	-	-	-	+	-	-	+	+	-	-
3645	6346	hypothetical protein	3	-	-	-	-	-	-	-	-	-	-	-	-	-	-	-	-	-	-	+	-	-	-	-	-	-	-	-	-	-	-	-	-	-	-	-	-	-	-	+	-	-	-	-	-	-	-	-	-	-	-	+	-	-
3646	6348	hypothetical protein	3	-	-	-	-	-	-	-	-	-	-	-	-	-	-	-	-	-	-	-	-	-	-	-	-	-	-	-	-	-	-	-	-	-	-	-	-	-	-	-	-	-	-	-	-	-	-	+	-	+	-	+	-	-
3647	6349	Glycosyltransferases involved in cell wall biogenesis	3	-	-	-	-	-	-	-	-	-	-	-	-	-	-	-	-	-	-	-	-	-	-	-	-	-	-	-	-	-	-	-	-	-	-	-	-	-	-	-	-	-	-	-	-	-	-	+	-	-	+	+	-	-
3648	6350	Acetyltransferase	3	-	-	-	-	-	-	-	-	-	-	-	-	-	-	-	-	-	-	-	-	-	-	-	-	-	-	-	-	-	-	-	-	-	-	-	-	-	-	-	-	-	-	-	-	-	-	-	+	-	+	+	-	-
3649	6351	glycosyltransferase	3	-	-	-	-	-	-	-	-	-	-	-	-	-	-	-	-	-	-	-	-	-	-	-	-	-	-	-	-	-	-	-	-	-	-	-	-	-	-	-	-	-	-	-	-	-	-	-	-	+	+	+	-	-
3650	6373	Abortive infection protein AbiGII	3	-	-	-	-	-	-	-	-	+	-	-	-	-	-	-	-	-	-	-	-	-	-	-	-	-	-	-	-	-	-	-	-	-	-	-	-	-	-	-	-	-	-	-	-	-	-	+	+	-	-	-	-	-
3651	6375	Phage DNA-binding protein	3	-	-	-	-	-	-	-	-	-	-	-	-	-	-	-	-	-	-	-	-	-	-	-	-	-	-	-	-	+	+	-	-	-	-	-	-	-	-	-	-	-	-	-	-	-	-	+	-	-	-	-	-	-
3652	6376	hypothetical protein	3	-	-	-	-	-	-	-	-	-	-	-	-	-	-	-	-	-	-	-	-	-	-	-	-	-	-	-	-	+	+	+	-	-	-	-	-	-	-	-	-	-	-	-	-	-	-	-	-	-	-	-	-	-
3653	6377	6-phospho-beta-glucosidase (EC 3.2.1.86)	3	-	-	-	-	-	-	-	-	-	-	-	-	-	-	-	-	-	-	-	-	-	-	-	-	-	-	-	-	+	+	+	-	-	-	-	-	-	-	-	-	-	-	-	-	-	-	-	-	-	-	-	-	-
3654	6379	Predicted hydrolase (HAD superfamily)	3	-	-	-	-	-	-	-	-	-	-	-	-	-	-	-	-	-	-	-	-	-	-	-	-	-	-	-	-	+	+	+	-	-	-	-	-	-	-	-	-	-	-	-	-	-	-	-	-	-	-	-	-	-
3655	6380	hypothetical protein	3	-	-	-	-	-	-	-	-	-	-	-	-	-	-	-	-	-	-	-	-	+	+	-	-	-	-	-	-	-	-	+	-	-	-	-	-	-	-	-	-	-	-	-	-	-	-	-	-	-	-	-	-	-
3656	6381	immunity protein	3	-	-	-	-	-	-	-	-	-	-	-	-	-	-	-	-	-	-	-	-	-	-	-	-	-	-	-	-	+	+	+	-	-	-	-	-	-	-	-	-	-	-	-	-	-	-	-	-	-	-	-	-	-
3657	6382	hypothetical protein	3	-	-	-	-	-	-	-	-	-	-	-	-	-	-	-	-	-	-	-	-	-	-	-	-	-	-	-	-	+	+	+	-	-	-	-	-	-	-	-	-	-	-	-	-	-	-	-	-	-	-	-	-	-
3658	6383	hypothetical protein	3	-	-	-	-	-	-	-	-	-	-	-	-	-	-	-	-	-	-	-	-	-	-	-	-	-	-	-	-	+	+	+	-	-	-	-	-	-	-	-	-	-	-	-	-	-	-	-	-	-	-	-	-	-
3659	6384	SNF2 family protein	3	-	-	-	-	-	-	-	-	-	-	-	-	-	-	-	-	-	-	-	-	+	+	-	-	-	-	-	-	-	-	+	-	-	-	-	-	-	-	-	-	-	-	-	-	-	-	-	-	-	-	-	-	-
3660	6386	NUDIX hydrolase	3	-	-	-	-	-	-	-	-	-	-	-	-	-	-	-	-	-	-	-	-	-	-	-	-	-	-	-	-	+	+	+	-	-	-	-	-	-	-	-	-	-	-	-	-	-	-	-	-	-	-	-	-	-
3661	6387	Predicted N-ribosylNicotinamide CRP-like regulator	3	-	-	-	-	-	-	-	-	-	-	-	-	-	-	-	-	-	-	-	-	-	-	-	-	-	-	-	-	+	+	+	-	-	-	-	-	-	-	-	-	-	-	-	-	-	-	-	-	-	-	-	-	-
3662	6388	Transposase	3	-	-	-	-	-	-	-	-	-	-	-	-	-	-	-	-	-	-	-	-	-	-	-	-	-	-	-	-	+	+	+	-	-	-	-	-	-	-	-	-	-	-	-	-	-	-	-	-	-	-	-	-	-
3663	6390	hypothetical protein	3	-	-	-	-	-	-	-	-	-	-	-	-	-	-	-	-	-	-	-	-	-	-	-	-	-	-	-	-	+	+	+	-	-	-	-	-	-	-	-	-	-	-	-	-	-	-	-	-	-	-	-	-	-
3664	6391	Tn5252	3	-	-	-	-	-	-	-	-	-	-	-	-	-	-	-	-	-	-	-	-	+	+	-	-	-	-	-	-	-	-	+	-	-	-	-	-	-	-	-	-	-	-	-	-	-	-	-	-	-	-	-	-	-
3665	6392	Glycerophosphoryl diester phosphodiesterase (EC 3.1.4.46)	3	-	-	-	-	-	-	-	-	-	-	-	-	-	-	-	-	-	-	-	-	-	-	-	-	-	-	-	-	-	-	-	-	-	-	-	-	-	-	-	-	-	-	-	-	-	-	+	+	+	-	-	-	-
3666	6393	hypothetical protein	3	-	-	-	-	-	-	-	-	-	-	-	-	-	-	-	-	-	-	-	-	-	-	-	-	-	-	-	-	-	-	-	-	-	-	-	-	-	-	-	-	-	-	-	-	-	-	+	+	+	-	-	-	-
3667	6395	hypothetical protein	3	-	-	-	-	-	-	-	-	-	-	-	-	-	-	-	-	-	-	-	-	-	-	-	-	-	-	+	+	-	-	-	-	-	-	-	-	-	-	-	-	-	-	-	-	-	-	-	+	-	-	-	-	-
3668	6398	Tellurite resistance protein TehB	3	-	-	-	-	-	-	-	-	-	-	-	-	-	-	-	-	-	-	-	-	-	+	-	-	-	-	-	-	-	-	-	+	-	-	-	-	-	-	-	-	-	-	-	-	-	-	-	+	-	-	-	-	-
3669	6402	hypothetical protein	3	-	-	-	-	-	-	-	-	-	-	-	-	-	-	-	-	-	+	-	-	-	-	-	-	-	-	-	-	-	-	-	-	-	-	-	-	-	-	-	-	-	-	+	-	-	-	-	+	-	-	-	-	-
3670	6405	hypothetical protein	3	-	-	-	-	-	-	-	-	-	-	-	-	-	-	-	-	-	-	-	-	-	-	-	-	-	-	-	-	-	-	-	-	-	-	-	-	-	-	+	-	-	-	+	-	+	-	-	-	-	-	-	-	-
3671	6408	hypothetical protein	3	-	-	-	-	-	-	-	-	-	-	-	-	-	-	-	-	-	-	-	-	-	-	-	-	-	-	-	-	-	-	-	-	-	-	-	-	-	-	+	-	-	-	+	-	+	-	-	-	-	-	-	-	-
3672	6409	hypothetical protein	3	-	-	-	-	-	-	-	-	-	-	-	-	-	-	-	-	-	-	-	-	-	-	-	-	-	-	-	-	-	-	-	-	-	-	-	-	-	-	-	-	-	-	+	-	-	-	-	-	+	-	-	+	-
3673	6441	hypothetical protein	3	-	-	-	-	-	-	-	-	-	-	-	-	-	-	-	-	-	-	-	-	-	-	-	-	-	-	-	-	-	-	-	-	-	-	-	-	-	-	-	-	-	-	-	-	-	-	+	-	+	+	-	-	-
3674	6448	unknown	3	-	-	-	-	-	-	-	-	+	-	-	-	-	-	-	-	-	-	-	-	-	-	-	-	-	-	-	-	-	-	-	-	-	-	-	-	-	-	-	-	-	-	-	-	-	-	-	-	-	+	-	+	-
3675	6456	hypothetical protein	3	-	-	-	-	-	-	-	-	-	-	-	-	-	-	-	-	-	-	-	-	-	-	-	-	-	-	+	+	-	-	-	-	-	-	-	-	-	-	-	-	-	-	-	-	-	-	-	-	-	-	-	+	-
3676	6457	transcriptional regulator, putative	3	-	-	-	-	-	-	-	+	+	-	-	-	-	-	-	-	-	-	-	-	-	-	-	-	-	-	-	-	-	-	-	-	-	-	-	-	-	-	-	-	-	-	-	-	-	-	-	-	-	-	-	+	-
3677	6529	hypothetical protein	3	-	-	-	-	-	-	-	-	-	-	-	-	-	-	-	-	-	-	+	-	-	-	-	-	-	-	-	-	-	-	+	+	-	-	-	-	-	-	-	-	-	-	-	-	-	-	-	-	-	-	-	-	-
3678	6531	hypothetical protein	3	-	-	-	-	-	-	-	-	-	-	-	-	-	-	-	-	-	-	+	-	-	-	-	-	-	-	-	-	-	-	+	+	-	-	-	-	-	-	-	-	-	-	-	-	-	-	-	-	-	-	-	-	-
3679	6535	hypothetical protein	3	-	-	-	-	-	-	-	-	-	-	-	-	-	-	-	-	-	-	-	-	-	-	-	-	-	-	+	+	+	-	-	-	-	-	-	-	-	-	-	-	-	-	-	-	-	-	-	-	-	-	-	-	-
3680	6536	hypothetical protein	3	-	-	-	-	-	-	-	-	-	-	-	-	-	-	-	-	-	-	-	-	-	-	-	-	-	-	+	+	+	-	-	-	-	-	-	-	-	-	-	-	-	-	-	-	-	-	-	-	-	-	-	-	-
3681	6538	hypothetical protein	3	-	-	-	-	-	-	-	-	-	-	-	-	-	-	-	-	-	-	-	-	-	-	-	-	-	-	+	+	+	-	-	-	-	-	-	-	-	-	-	-	-	-	-	-	-	-	-	-	-	-	-	-	-
3682	6539	hypothetical protein	3	-	-	-	-	-	-	-	-	-	-	-	-	-	-	-	-	-	-	-	-	-	-	-	-	-	-	+	+	+	-	-	-	-	-	-	-	-	-	-	-	-	-	-	-	-	-	-	-	-	-	-	-	-
3683	6540	hypothetical protein	3	-	-	-	-	-	-	-	-	-	-	-	-	-	-	-	-	-	-	-	-	-	-	-	-	-	-	+	+	+	-	-	-	-	-	-	-	-	-	-	-	-	-	-	-	-	-	-	-	-	-	-	-	-
3684	6547	Type I restriction-modification system	3	-	-	-	-	-	-	-	-	-	-	-	-	-	-	-	-	-	-	-	-	-	-	-	-	-	-	-	-	-	-	+	+	-	-	+	-	-	-	-	-	-	-	-	-	-	-	-	-	-	-	-	-	-
3685	6551	hypothetical protein	3	-	-	-	-	-	-	-	-	-	-	-	-	-	-	-	-	-	-	-	-	-	-	-	-	-	-	+	+	-	+	-	-	-	-	-	-	-	-	-	-	-	-	-	-	-	-	-	-	-	-	-	-	-
3686	6552	RNA polymerase ECF-type sigma factor	3	-	-	-	-	-	-	-	+	+	-	-	-	-	-	-	-	-	-	-	-	-	-	-	-	-	-	-	+	-	-	-	-	-	-	-	-	-	-	-	-	-	-	-	-	-	-	-	-	-	-	-	-	-
3687	6553	hypothetical protein	3	-	-	-	-	-	-	-	-	-	-	-	-	-	-	-	-	-	-	-	+	+	+	-	-	-	-	-	-	-	-	-	-	-	-	-	-	-	-	-	-	-	-	-	-	-	-	-	-	-	-	-	-	-
3688	6554	ORF3	3	-	-	-	-	-	-	-	-	-	-	-	-	-	-	-	-	-	-	-	+	+	+	-	-	-	-	-	-	-	-	-	-	-	-	-	-	-	-	-	-	-	-	-	-	-	-	-	-	-	-	-	-	-
3689	6555	hypothetical protein	3	-	-	-	-	-	-	-	-	-	-	-	-	-	-	-	-	-	-	-	+	+	+	-	-	-	-	-	-	-	-	-	-	-	-	-	-	-	-	-	-	-	-	-	-	-	-	-	-	-	-	-	-	-
3690	6556	Periaxin	3	-	-	-	-	-	-	-	-	-	-	-	-	-	-	-	-	-	-	-	+	+	+	-	-	-	-	-	-	-	-	-	-	-	-	-	-	-	-	-	-	-	-	-	-	-	-	-	-	-	-	-	-	-
3691	6557	hypothetical protein	3	-	-	-	-	-	-	-	-	-	-	-	-	-	-	-	-	-	-	-	+	+	+	-	-	-	-	-	-	-	-	-	-	-	-	-	-	-	-	-	-	-	-	-	-	-	-	-	-	-	-	-	-	-
3692	6558	internalin, putative	3	-	-	-	-	-	-	-	-	-	-	-	-	-	-	-	-	-	-	-	+	+	+	-	-	-	-	-	-	-	-	-	-	-	-	-	-	-	-	-	-	-	-	-	-	-	-	-	-	-	-	-	-	-
3693	6559	internalin, putative	3	-	-	-	-	-	-	-	-	-	-	-	-	-	-	-	-	-	-	-	+	+	+	-	-	-	-	-	-	-	-	-	-	-	-	-	-	-	-	-	-	-	-	-	-	-	-	-	-	-	-	-	-	-
3694	6560	hypothetical protein	3	-	-	-	-	-	-	-	-	-	-	-	-	-	+	-	-	-	-	-	-	-	-	-	-	-	-	-	-	-	-	+	+	-	-	-	-	-	-	-	-	-	-	-	-	-	-	-	-	-	-	-	-	-
3695	6562	LacX protein, plasmid	3	-	-	-	-	-	-	-	-	-	-	-	-	-	-	-	-	-	-	-	-	-	-	-	-	-	-	+	+	-	-	-	-	-	-	-	-	-	-	-	-	-	-	-	-	-	-	-	-	+	-	-	-	-
3696	6565	hypothetical protein	3	-	-	-	-	-	-	-	-	-	-	-	-	-	-	-	-	-	-	-	-	-	-	+	-	-	-	+	+	-	-	-	-	-	-	-	-	-	-	-	-	-	-	-	-	-	-	-	-	-	-	-	-	-
3697	7329	DNA recombinase, putative	3	+	+	+	-	-	-	-	-	-	-	-	-	-	-	-	-	-	-	-	-	-	-	-	-	-	-	-	-	-	-	-	-	-	-	-	-	-	-	-	-	-	-	-	-	-	-	-	-	-	-	-	-	-
3698	7330	putative asparagine synthetase	3	+	+	+	-	-	-	-	-	-	-	-	-	-	-	-	-	-	-	-	-	-	-	-	-	-	-	-	-	-	-	-	-	-	-	-	-	-	-	-	-	-	-	-	-	-	-	-	-	-	-	-	-	-
3699	7331	hypothetical protein	3	+	+	+	-	-	-	-	-	-	-	-	-	-	-	-	-	-	-	-	-	-	-	-	-	-	-	-	-	-	-	-	-	-	-	-	-	-	-	-	-	-	-	-	-	-	-	-	-	-	-	-	-	-
3700	7332	hypothetical protein	3	+	+	+	-	-	-	-	-	-	-	-	-	-	-	-	-	-	-	-	-	-	-	-	-	-	-	-	-	-	-	-	-	-	-	-	-	-	-	-	-	-	-	-	-	-	-	-	-	-	-	-	-	-
3701	7333	Recombinase	3	+	+	+	-	-	-	-	-	-	-	-	-	-	-	-	-	-	-	-	-	-	-	-	-	-	-	-	-	-	-	-	-	-	-	-	-	-	-	-	-	-	-	-	-	-	-	-	-	-	-	-	-	-
3702	7334	hypothetical protein	3	+	+	+	-	-	-	-	-	-	-	-	-	-	-	-	-	-	-	-	-	-	-	-	-	-	-	-	-	-	-	-	-	-	-	-	-	-	-	-	-	-	-	-	-	-	-	-	-	-	-	-	-	-
3703	7335	MutM protein	3	+	+	+	-	-	-	-	-	-	-	-	-	-	-	-	-	-	-	-	-	-	-	-	-	-	-	-	-	-	-	-	-	-	-	-	-	-	-	-	-	-	-	-	-	-	-	-	-	-	-	-	-	-
3704	1055	Mobile element protein	2	-	-	-	-	-	-	-	-	-	-	-	-	-	-	-	-	-	-	-	-	-	-	-	-	-	-	-	-	-	-	-	-	-	-	-	-	-	-	-	-	-	-	-	-	-	+	-	+	-	-	-	-	-
3705	1096	Mobile element protein	2	-	-	-	-	-	-	-	-	-	-	-	-	-	-	-	-	-	-	-	-	-	-	-	-	-	-	-	-	-	-	-	-	-	-	-	-	-	-	-	-	-	-	-	-	-	+	-	+	-	-	-	-	-
3706	1908	PTS system, galactose-specific IIB component (EC 2.7.1.69)	2	-	-	-	-	-	-	-	-	-	-	-	-	-	-	-	-	-	-	-	-	-	-	-	-	-	-	-	-	-	-	-	-	-	-	-	-	-	-	+	-	-	-	-	-	-	-	-	-	-	-	+	-	-
3707	1935	PTS system, galactose-specific IIC component (EC 2.7.1.69)	2	-	-	-	-	-	-	-	-	-	-	-	-	-	-	-	-	-	-	-	-	-	-	-	-	-	-	-	-	-	-	-	-	-	-	-	-	-	-	+	-	-	-	-	-	-	-	-	-	-	-	+	-	-
3708	2204	N-acetyl-gamma-glutamyl-phosphate reductase (EC 1.2.1.38)	2	-	-	-	-	-	-	-	-	-	-	-	-	-	-	-	-	-	-	-	-	-	-	-	-	-	-	-	-	-	-	-	-	+	-	-	-	-	-	-	-	-	-	-	-	-	-	-	-	+	-	-	-	-
3709	2205	Acetylornithine aminotransferase (EC 2.6.1.11)	2	-	-	-	-	-	-	-	-	-	-	-	-	-	-	-	-	-	-	-	-	-	-	-	-	-	-	-	-	-	-	-	-	+	-	-	-	-	-	-	-	-	-	-	-	-	-	-	-	+	-	-	-	-
3710	2206	Acetylglutamate kinase (EC 2.7.2.8)	2	-	-	-	-	-	-	-	-	-	-	-	-	-	-	-	-	-	-	-	-	-	-	-	-	-	-	-	-	-	-	-	-	+	-	-	-	-	-	-	-	-	-	-	-	-	-	-	-	+	-	-	-	-
3711	2208	putative glycosyltransferase - possibly involved in cell wall localization and side chain formation of rhamnose-glucose polysaccharide	2	-	-	-	-	-	-	-	-	-	-	-	-	-	-	-	-	-	-	-	-	-	-	-	-	-	-	-	-	-	-	-	-	-	-	-	-	-	-	-	-	-	-	-	-	-	-	+	-	+	-	-	-	-
3712	2254	Glutamate N-acetyltransferase (EC 2.3.1.35) / N-acetylglutamate synthase (EC 2.3.1.1)	2	-	-	-	-	-	-	-	-	-	-	-	-	-	-	-	-	-	-	-	-	-	-	-	-	-	-	-	-	-	-	-	-	+	-	-	-	-	-	-	-	-	-	-	-	-	-	-	-	+	-	-	-	-
3713	2485	Glycosyl transferase, group 2 family protein	2	-	-	-	-	-	-	-	-	-	-	-	-	-	-	-	-	-	-	-	-	-	-	-	-	-	-	-	-	-	-	-	-	-	-	-	-	-	-	-	-	-	-	-	-	-	-	+	-	+	-	-	-	-
3714	2680	Positive transcriptional regulator MutR family	2	-	-	-	-	-	-	-	-	-	-	-	-	-	-	-	-	-	-	-	-	-	-	-	-	-	-	-	-	-	-	-	-	-	-	-	-	-	-	-	-	-	-	-	-	-	-	-	+	-	-	-	+	-
3715	2763	hypothetical protein	2	-	-	-	-	-	-	-	-	-	-	-	-	-	-	-	-	-	-	-	-	-	-	-	-	-	-	-	-	-	-	-	-	-	-	-	-	-	-	-	-	-	-	-	+	-	-	-	+	-	-	-	-	-
3716	2764	ATP-dependent Clp protease, ATP-binding subunit ClpC	2	-	-	-	-	-	-	-	-	-	-	-	-	-	-	-	-	-	-	-	-	-	-	-	-	-	-	-	-	-	-	-	-	-	-	-	-	-	-	-	-	-	-	-	+	-	-	-	+	-	-	-	-	-
3717	2765	putative ATPase TraE	2	-	-	-	-	-	-	-	-	-	-	-	-	-	-	-	-	-	-	-	-	-	-	-	-	-	-	-	-	-	-	-	-	-	-	-	-	-	-	-	-	-	-	-	+	-	-	-	+	-	-	-	-	-
3718	2766	hypothetical protein	2	-	-	-	-	-	-	-	-	-	-	-	-	-	-	-	-	-	-	-	-	-	-	-	-	-	-	-	-	-	-	-	-	-	-	-	-	-	-	-	-	-	-	-	+	-	-	-	+	-	-	-	-	-
3719	2767	hypothetical protein	2	-	-	-	-	-	-	-	-	-	-	-	-	-	-	-	-	-	-	-	-	-	-	-	-	-	-	-	-	-	-	-	-	-	-	-	-	-	-	-	-	-	-	-	+	-	-	-	+	-	-	-	-	-
3720	2768	conserved domain protein	2	-	-	-	-	-	-	-	-	-	-	-	-	-	-	-	-	-	-	-	-	-	-	-	-	-	-	-	-	-	-	-	-	-	-	-	-	-	-	-	-	-	-	-	+	-	-	-	+	-	-	-	-	-
3721	2891	hypothetical protein	2	-	-	-	-	-	-	-	-	-	-	-	-	-	-	-	-	-	-	-	-	-	-	-	-	-	-	-	-	-	-	-	-	-	-	-	-	-	-	-	-	-	-	-	+	-	-	-	+	-	-	-	-	-
3722	2892	hypothetical protein	2	-	-	-	-	-	-	-	-	-	-	-	-	-	-	-	-	-	-	-	-	-	-	-	-	-	-	-	-	-	-	-	-	-	-	-	-	-	-	-	-	-	-	-	+	-	-	-	+	-	-	-	-	-
3723	2894	hypothetical protein	2	-	-	-	-	-	-	-	-	-	-	-	-	-	-	-	-	-	-	-	-	-	-	-	-	-	-	-	-	-	-	-	-	-	-	-	-	-	-	-	-	-	-	-	+	-	-	-	+	-	-	-	-	-
3724	2895	DNA-entry nuclease (Competence-specific nuclease) (EC 3.1.30.-)	2	-	-	-	-	-	-	-	-	-	-	-	-	-	-	-	-	-	-	-	-	-	-	-	-	-	-	-	-	-	-	-	-	-	-	-	-	-	-	-	-	-	-	-	+	-	-	-	+	-	-	-	-	-
3725	2897	hypothetical protein	2	-	-	-	-	-	-	-	-	-	-	-	-	-	-	-	-	-	-	-	-	-	-	-	-	-	-	-	-	-	-	-	-	-	-	-	-	-	-	-	-	-	-	-	+	-	-	-	+	-	-	-	-	-
3726	2898	hypothetical protein	2	-	-	-	-	-	-	-	-	-	-	-	-	-	-	-	-	-	-	-	-	-	-	-	-	-	-	-	-	-	-	-	-	-	-	-	-	-	-	-	-	-	-	-	+	-	-	-	+	-	-	-	-	-
3727	2899	hypothetical protein	2	-	-	-	-	-	-	-	-	-	-	-	-	-	-	-	-	-	-	-	-	-	-	-	-	-	-	-	-	-	-	-	-	-	-	-	-	-	-	-	-	-	-	-	+	-	-	-	+	-	-	-	-	-
3728	2901	DNA topoisomerase III (EC 5.99.1.2)	2	-	-	-	-	-	-	-	-	-	-	-	-	-	-	-	-	-	-	-	-	-	-	-	-	-	-	-	-	-	-	-	-	-	-	-	-	-	-	-	-	-	-	-	+	-	-	-	+	-	-	-	-	-
3729	2907	Mannose-6-phosphate isomerase	2	-	-	-	-	-	-	-	-	-	-	-	-	-	-	-	-	-	-	-	-	-	-	-	-	-	-	-	-	-	-	-	-	-	-	-	-	-	-	-	-	-	-	-	-	-	+	-	+	-	-	-	-	-
3730	2922	Competence-stimulating peptide ABC transporter permease protein ComB	2	-	-	-	-	-	-	-	-	-	-	-	-	-	-	-	-	-	-	-	-	-	-	-	-	-	-	-	-	-	-	-	-	-	-	-	-	-	-	-	-	-	-	-	-	+	-	+	-	-	-	-	-	-
3731	2996	Mobile element protein	2	-	-	-	-	-	-	-	-	-	-	-	-	-	-	-	-	-	-	-	-	-	-	-	-	-	-	-	-	-	-	-	-	-	-	+	+	-	-	-	-	-	-	-	-	-	-	-	-	-	-	-	-	-
3732	3033	hypothetical protein	2	-	-	-	-	-	-	-	-	-	-	-	-	-	-	-	-	-	-	-	-	-	-	-	-	-	-	-	-	-	-	-	-	-	-	-	-	-	-	-	-	-	-	-	+	-	-	-	+	-	-	-	-	-
3733	3035	putative transposase	2	-	-	-	-	-	-	-	-	-	-	-	-	-	-	-	-	-	-	-	-	-	-	-	-	-	-	-	-	-	-	-	-	-	-	-	-	-	-	-	-	-	-	-	-	-	+	-	+	-	-	-	-	-
3734	3135	hypothetical protein	2	-	-	-	-	-	-	-	-	-	-	-	-	-	-	-	-	-	-	-	-	-	-	-	-	-	-	-	-	-	-	-	-	-	-	-	-	-	-	-	-	-	-	-	+	-	-	-	+	-	-	-	-	-
3735	3138	hypothetical protein	2	-	-	-	-	-	-	-	-	-	-	-	-	-	-	-	-	-	-	-	-	-	-	-	-	-	-	-	-	-	-	-	-	-	-	-	-	-	-	-	-	-	-	-	+	-	-	-	+	-	-	-	-	-
3736	3139	hypothetical protein	2	-	-	-	-	-	-	-	-	-	-	-	-	-	-	-	-	-	-	-	-	-	-	-	-	-	-	-	-	-	-	-	-	-	-	-	-	-	-	-	-	-	-	-	+	-	-	-	+	-	-	-	-	-
3737	3140	hypothetical protein	2	-	-	-	-	-	-	-	-	-	-	-	-	-	-	-	-	-	-	-	-	-	-	-	-	-	-	-	-	-	-	-	-	-	-	-	-	-	-	-	-	-	-	-	+	-	-	-	+	-	-	-	-	-
3738	3141	ATPase involved in DNA repair	2	-	-	-	-	-	-	-	-	-	-	-	-	-	-	-	-	-	-	-	-	-	-	-	-	-	-	-	-	-	-	-	-	-	-	-	-	-	-	-	-	-	-	-	+	-	-	-	+	-	-	-	-	-
3739	3142	hypothetical protein	2	-	-	-	-	-	-	-	-	-	-	-	-	-	-	-	-	-	-	-	-	-	-	-	-	-	-	-	-	-	-	-	-	-	-	-	-	-	-	-	-	-	-	-	+	-	-	-	+	-	-	-	-	-
3740	3144	hypothetical protein	2	-	-	-	-	-	-	-	-	-	-	-	-	-	-	-	-	-	-	-	-	-	-	-	-	-	-	-	-	-	-	-	-	-	-	-	-	-	-	-	-	-	-	-	+	-	-	-	+	-	-	-	-	-
3741	3146	hypothetical protein	2	-	-	-	-	-	-	-	-	-	-	-	-	-	-	-	-	-	-	-	-	-	-	-	-	-	-	-	-	-	-	-	-	-	-	-	-	-	-	-	-	-	-	-	+	-	-	-	+	-	-	-	-	-
3742	3147	hypothetical protein	2	-	-	-	-	-	-	-	-	-	-	-	-	-	-	-	-	-	-	-	-	-	-	-	-	-	-	-	-	-	-	-	-	-	-	-	-	-	-	-	-	-	-	-	+	-	-	-	+	-	-	-	-	-
3743	3171	abortive infection protein AbiGI	2	-	-	-	-	-	-	+	-	-	-	-	-	-	-	-	-	-	-	-	-	-	-	-	-	-	-	-	-	-	-	-	-	-	-	-	-	-	-	-	-	-	-	-	-	-	-	-	-	+	-	-	-	-
3744	3292	hypothetical protein	2	-	-	-	-	-	-	-	-	-	-	-	-	-	-	-	-	-	-	-	-	-	-	-	-	-	-	-	-	-	-	-	-	-	-	-	-	-	-	-	-	-	-	-	+	-	-	-	+	-	-	-	-	-
3745	3293	hypothetical protein	2	-	-	-	-	-	-	-	-	-	-	-	-	-	-	-	-	-	-	-	-	-	-	-	-	-	-	-	-	-	-	-	-	-	-	-	-	-	-	-	-	-	-	-	+	-	-	-	+	-	-	-	-	-
3746	3294	hypothetical protein	2	-	-	-	-	-	-	-	-	-	-	-	-	-	-	-	-	-	-	-	-	-	-	-	-	-	-	-	-	-	-	-	-	-	-	-	-	-	-	-	-	-	-	-	+	-	-	-	+	-	-	-	-	-

Table S5. Precence/absence of each HG among 57 *S. suis* strains

				1																13				25					28						104		94			76			53/54		Divergent *S. suis*																
Index[a]	ClusterID	Gene products	Members	98HAH33	05ZYH33	SC84	A7	SS12	P1/7	DAT273	NIAH11434	JS14	GZ1	BM407	S735	13730	MNCM07	NCTC 10446	14636	NCTC 10237	ST1	2651	89-1591	MNCM04	MNCM25	8074	D9	4961	ST3	MNCM43	DAT292	DAT260	NIAH11435	MNCM21	MNCM50	89-2479	DAT234	DAT290	93A	42A	NT77	6407	11538	8830	12814	92-1400	89-590	2726	2524	89-3576-3	4417	D12	10581	89-5259	88-5299A	R61	92-1191	14A	92-4172	22083
3747	3295	hypothetical protein	2	-	-	-	-	-	-	-	-	-	-	-	-	-	-	-	-	-	-	-	-	-	-	-	-	-	-	-	-	-	-	-	-	-	-	-	-	-	-	-	-	-	-	-	-	-	-	-	+	-	-	-	+	-	-	-	-	-
3748	3300	Cholinephosphate cytidylyltransferase (EC 2.7.7.15)	2	-	-	-	-	-	-	-	-	-	-	-	-	-	-	-	-	-	-	-	-	-	-	-	-	-	-	-	-	-	-	-	-	-	-	-	-	-	-	-	-	+	-	-	-	-	-	-	-	-	-	-	-	+	-	-	-	-
3749	3302	hypothetical protein	2	-	-	-	-	-	-	-	-	-	-	-	-	-	-	-	-	-	-	-	-	-	-	-	-	-	-	-	-	-	-	-	-	-	-	-	-	-	-	-	-	+	-	-	-	-	-	-	-	-	-	-	-	+	-	-	-	-
3750	3315	glyoxalase family protein	2	-	-	-	-	-	-	-	-	-	-	-	-	-	-	-	-	-	-	-	-	-	-	-	-	-	-	-	-	-	-	-	-	-	-	-	-	-	-	-	-	-	-	-	-	-	-	-	-	-	+	-	+	-	-	-	-	-
3751	3327	Acetyltransferase (EC 2.3.1.-)	2	-	-	-	-	-	-	-	-	-	-	-	-	-	-	-	-	-	-	-	-	-	-	-	-	-	-	-	-	-	-	-	-	-	-	-	-	-	-	-	-	-	-	-	-	-	-	-	-	-	-	-	+	-	-	+	-	-
3752	3329	Abortive infection protein AbiGII	2	-	-	-	-	-	-	+	-	-	-	-	-	-	-	-	-	-	-	-	-	-	-	-	-	-	-	-	-	-	-	-	-	-	-	-	-	-	-	-	-	-	-	-	-	-	-	-	-	-	-	-	-	+	-	-	-	-
3753	3338	Putative EsaC protein analog (Listeria type 3)	2	-	-	-	-	-	-	-	-	-	-	-	-	-	-	-	-	-	-	-	-	-	-	-	-	-	-	-	-	-	-	-	-	-	-	-	-	-	-	-	-	-	-	+	-	-	-	-	-	-	-	-	-	+	-	-	-	-
3754	3452	thiJ/pfpI family protein	2	-	-	-	-	-	-	-	-	-	-	-	-	-	-	-	+	-	-	-	-	-	-	-	-	-	-	-	-	-	-	-	-	-	-	-	-	-	-	-	-	-	-	-	-	-	-	-	-	-	+	-	-	-	-	-	-	-
3755	3464	hypothetical protein	2	-	-	-	-	-	-	-	-	-	-	-	-	-	-	-	-	-	-	-	-	-	-	-	-	-	-	-	-	-	-	-	-	-	-	-	-	-	-	-	-	-	-	-	-	-	-	-	+	-	-	-	+	-	-	-	-	-
3756	3465	hypothetical protein	2	-	-	-	-	-	-	-	-	-	-	-	-	-	-	-	-	-	-	-	-	-	-	-	-	-	-	-	-	-	-	-	-	-	-	-	-	-	-	-	-	-	-	-	-	-	-	-	+	-	-	-	+	-	-	-	-	-
3757	3476	Cob(I)alamin adenosyltransferase PduO (EC 2.5.1.17)	2	-	-	-	-	-	-	-	-	-	-	-	-	-	-	-	-	-	-	-	-	-	-	-	-	-	-	-	-	-	-	-	-	-	-	-	-	-	-	-	-	-	-	-	-	-	-	-	-	-	+	-	+	-	-	-	-	-
3758	3479	rhamnosyltransferase	2	-	-	-	-	-	-	-	-	-	-	-	-	-	-	-	-	-	-	-	-	-	-	-	-	-	-	-	-	-	-	-	-	-	-	-	-	-	-	-	-	-	-	-	-	-	-	-	-	-	+	-	+	-	-	-	-	-
3759	3481	Dolichol-phosphate mannosyltransferase (EC 2.4.1.83) in lipid-linked oligosaccharide synthesis cluster	2	-	-	-	-	-	-	-	-	-	-	-	-	-	-	-	-	-	-	-	-	-	-	-	-	-	-	-	-	-	-	-	-	-	-	-	-	-	-	-	-	-	-	-	-	-	-	-	-	-	+	-	+	-	-	-	-	-
3760	3503	Heteropolysaccharide repeat unit export protein	2	-	-	-	-	-	-	-	-	-	-	-	-	-	-	-	-	-	-	-	-	-	-	-	-	-	-	-	-	-	-	-	-	-	-	-	-	-	-	-	-	-	-	-	-	-	-	-	-	-	-	+	-	+	-	-	-	-
3761	3671	2-C-methyl-D-erythritol 4-phosphate cytidylyltransferase (EC 2.7.7.60)	2	-	-	-	-	-	-	-	-	-	-	-	-	-	-	-	-	-	-	-	-	-	-	-	-	-	-	-	-	-	-	-	-	-	-	-	-	-	-	-	-	-	-	-	-	-	-	-	-	+	-	-	-	-	-	-	-	+
3762	3684	Membrane protein involved in the export of O-antigen, teichoic acid lipoteichoic acids	2	-	-	-	-	-	-	-	-	-	-	-	-	-	-	-	-	-	-	-	-	-	-	-	-	-	-	-	-	-	-	-	-	-	-	-	-	-	-	-	-	-	+	-	-	-	-	-	-	-	+	-	-	-	-	-	-	-
3763	3698	Cobalt-precorrin-8x methylmutase (EC 5.4.1.2)	2	-	-	-	-	-	-	-	-	-	-	-	-	-	-	-	-	-	-	-	-	-	-	-	-	-	-	-	-	-	-	-	-	-	-	-	-	-	-	-	-	-	-	-	-	-	-	-	-	-	+	-	+	-	-	-	-	-
3764	3706	Site-specific recombinase	2	-	-	-	-	-	-	-	-	-	-	-	-	-	-	-	-	-	-	-	-	-	-	-	-	-	-	-	-	-	-	-	-	-	-	-	-	-	-	-	-	-	-	-	-	+	-	-	-	+	-	-	-	-	-	-	-	-
3765	3716	Huntingtin interacting protein E-like protein	2	-	-	-	-	-	-	-	-	-	-	-	-	-	-	-	-	-	-	-	-	-	-	-	-	-	-	-	-	-	-	-	-	-	-	-	+	+	-	-	-	-	-	-	-	-	-	-	-	-	-	-	-	-	-	-	-	-
3766	3726	Phosphosugar-binding transcriptional repressor, RpiR family	2	-	-	-	-	-	-	-	-	-	-	-	-	-	-	-	-	-	-	-	-	-	-	-	-	-	-	-	-	-	-	-	-	-	-	-	-	-	-	-	-	-	-	-	-	-	-	-	-	+	-	-	+	-	-	-	-	-
3767	3728	1-phosphofructokinase	2	-	-	-	-	-	-	-	-	-	-	-	-	-	-	-	-	-	-	-	-	-	-	-	-	-	-	-	-	-	-	-	-	-	-	-	-	-	-	-	-	-	-	-	-	-	-	-	-	+	-	-	+	-	-	-	-	-
3768	3731	PTS system, galactose-inducible IIB component (EC 2.7.1.69) / PTS system, galactose-inducible IIC component (EC 2.7.1.69)	2	-	-	-	-	-	-	-	-	-	-	-	-	-	-	-	-	-	-	-	-	-	-	-	-	-	-	-	-	-	-	-	-	-	-	-	-	-	-	-	-	-	-	-	-	-	-	-	-	+	-	-	+	-	-	-	-	-
3769	3732	putative PTS system, fructose-specific IIA component	2	-	-	-	-	-	-	-	-	-	-	-	-	-	-	-	-	-	-	-	-	-	-	-	-	-	-	-	-	-	-	-	-	-	-	-	-	-	-	-	-	-	-	-	-	-	-	-	-	+	-	-	+	-	-	-	-	-
3770	3741	unknown phage protein	2	-	-	-	-	-	-	-	-	+	-	-	-	-	-	-	-	-	-	-	-	-	-	-	-	-	-	-	-	-	-	-	-	-	-	-	-	-	-	-	-	-	-	-	-	-	-	-	-	-	-	-	-	-	+	-	-	-
3771	3891	Transposase, uncharacterized, truncation	2	-	-	-	-	-	-	-	-	-	-	-	-	-	-	-	-	-	-	-	-	-	-	-	-	-	-	-	-	-	-	-	-	-	-	-	-	-	-	+	+	-	-	-	-	-	-	-	-	-	-	-	-	-	-	-	-	-
3772	3903	hypothetical protein	2	-	-	-	-	-	-	-	-	-	-	-	-	-	-	-	-	-	-	-	-	-	-	-	-	-	-	-	-	-	-	-	-	-	-	-	-	-	-	+	+	-	-	-	-	-	-	-	-	-	-	-	-	-	-	-	-	-
3773	3907	ABC transporter ATP-binding protein	2	-	-	-	-	-	-	-	-	-	-	-	-	-	-	-	-	-	-	-	-	-	-	-	-	-	-	-	-	-	-	-	-	-	-	-	-	-	-	+	+	-	-	-	-	-	-	-	-	-	-	-	-	-	-	-	-	-
3774	3910	Phage replication initiation	2	-	-	-	-	-	-	-	-	-	-	-	-	-	-	-	-	-	-	-	-	-	-	-	-	-	-	-	-	-	-	-	-	-	-	-	-	-	-	-	+	-	+	-	-	-	-	-	-	-	-	-	-	-	-	-	-	-
3775	3920	Unknown	2	-	-	-	-	-	-	-	-	-	-	-	-	-	-	-	-	-	-	-	-	-	-	-	-	-	-	-	-	-	-	-	-	-	-	-	-	-	-	-	-	-	-	-	-	-	-	-	-	-	-	-	-	-	-	-	+	+
3776	3921	LPXTG-motif containing, cell wall anchor domain protein	2	-	-	-	-	-	-	-	-	-	-	-	-	-	-	-	-	-	-	-	-	-	-	-	-	-	-	-	-	-	-	-	-	-	-	-	-	-	-	-	-	-	-	-	-	-	-	-	-	-	-	-	-	-	-	-	+	+
3777	3923	hypothetical protein	2	-	-	-	-	-	-	-	-	-	-	-	-	-	-	-	-	-	-	-	-	-	-	-	-	-	-	-	-	-	-	-	-	-	-	-	-	-	-	-	-	-	-	-	-	-	-	-	-	-	-	-	-	-	-	-	+	+
3778	3926	Sortase A, LPXTG specific	2	-	-	-	-	-	-	-	-	-	-	-	-	-	-	-	-	-	-	-	-	-	-	-	-	-	-	-	-	-	-	-	-	-	-	-	-	-	-	-	-	-	-	-	-	-	-	-	-	-	-	-	-	-	-	-	+	+
3779	3927	Unknown	2	-	-	-	-	-	-	-	-	-	-	-	-	-	-	-	-	-	-	-	-	-	-	-	-	-	-	-	-	-	-	-	-	-	-	-	-	-	-	-	-	-	-	-	-	-	-	-	-	-	-	-	-	-	-	-	+	+
3780	3968	hypothetical protein	2	-	-	-	-	-	-	-	-	-	-	-	-	-	-	-	-	-	-	-	-	-	-	-	-	-	-	-	-	-	-	-	-	-	-	-	-	-	-	-	-	-	-	-	-	-	-	-	-	-	+	-	-	-	+	-	-	-
3781	3977	Type I restriction-modification system, DNA-methyltransferase subunit M (EC 2.1.1.72)	2	-	-	-	-	-	-	-	-	-	-	-	-	-	-	-	-	-	-	-	-	-	-	-	-	-	-	-	-	-	-	-	-	-	-	-	+	+	-	-	-	-	-	-	-	-	-	-	-	-	-	-	-	-	-	-	-	-
3782	3979	Phage antirepressor protein	2	-	-	-	-	-	-	-	-	-	-	-	-	-	-	-	-	-	-	-	-	-	-	-	-	-	-	-	-	-	-	-	-	-	-	-	+	+	-	-	-	-	-	-	-	-	-	-	-	-	-	-	-	-	-	-	-	-
3783	3982	Type I restriction-modification system, restriction subunit R (EC 3.1.21.3)	2	-	-	-	-	-	-	-	-	-	-	-	-	-	-	-	-	-	-	-	-	-	-	-	-	-	-	-	-	-	-	-	-	-	-	-	+	+	-	-	-	-	-	-	-	-	-	-	-	-	-	-	-	-	-	-	-	-
3784	4212	hypothetical protein	2	-	-	-	-	-	-	-	-	-	-	-	-	-	-	-	-	-	-	-	-	-	-	-	-	-	-	-	-	-	-	-	-	-	-	-	-	-	-	+	+	-	-	-	-	-	-	-	-	-	-	-	-	-	-	-	-	-
3785	4239	Beta-1,3-glucosyltransferase	2	-	-	-	-	-	-	-	-	-	-	-	-	-	-	-	-	-	-	-	-	-	-	-	-	-	-	-	-	-	-	-	-	-	-	-	-	-	-	-	-	-	-	-	-	-	-	-	-	-	-	-	-	-	-	-	+	+
3786	4243	hypothetical protein	2	-	-	-	-	-	-	-	-	-	-	-	-	-	-	-	-	-	-	-	-	-	-	-	-	-	-	-	-	-	-	-	-	-	-	-	-	-	-	-	-	-	-	-	-	-	-	-	+	-	+	-	-	-	-	-	-	-
3787	4245	Mobile element protein	2	-	-	-	-	-	-	-	-	-	-	-	-	-	-	-	-	-	-	-	-	-	-	-	-	-	-	-	-	-	-	-	-	-	-	-	-	+	-	-	-	-	-	-	-	-	-	-	+	-	-	-	-	-	-	-	-	-
3788	4246	Transcriptional regulator, MerR family	2	-	-	-	-	-	-	-	-	-	-	-	-	-	-	-	-	-	-	-	-	-	-	-	-	-	-	-	-	-	-	-	-	-	-	-	-	-	-	-	-	-	-	+	-	-	-	-	+	-	-	-	-	-	-	-	-	-
3789	4262	NADH dehydrogenase (EC 1.6.99.3)	2	-	-	-	-	-	-	-	-	-	-	-	-	-	-	-	-	-	-	-	-	-	-	-	-	-	-	-	-	-	-	-	-	-	-	-	-	-	-	-	-	-	-	-	-	-	-	-	-	-	+	+	-	-	-	-	-	-
3790	4281	serine/threonine phosphatase, putative	2	-	-	-	-	-	-	-	-	-	-	-	-	-	-	+	-	-	-	-	-	-	-	-	-	-	-	-	-	-	-	-	-	-	-	-	-	-	-	-	-	-	-	-	-	-	-	-	-	-	-	-	-	+	-	-	-	-
3791	4287	hypothetical protein	2	-	-	-	-	-	-	-	-	-	-	-	-	-	-	-	-	-	-	-	-	-	-	-	-	-	-	-	-	-	-	-	-	-	-	-	-	-	-	-	-	-	-	+	-	+	-	-	-	-	-	-	-	-	-	-	-	-
3792	4326	hypothetical protein	2	-	-	-	-	-	-	-	-	-	-	-	-	-	-	-	-	-	-	-	-	-	-	-	-	-	-	-	-	-	-	-	-	-	-	-	-	-	-	-	-	-	-	-	-	-	-	-	-	-	-	-	+	-	-	+	-	-
3793	4329	PTS system, galactose-specific IIA component (EC 2.7.1.69)	2	-	-	-	-	-	-	-	-	-	-	-	-	-	-	-	-	-	-	-	-	-	-	-	-	-	-	-	-	-	-	-	-	-	-	-	-	-	-	-	-	-	-	+	-	-	-	-	-	-	-	-	-	-	-	+	-	-
3794	4332	Tn5252, relaxase	2	-	-	-	-	-	-	+	-	-	-	-	-	-	-	-	-	-	-	-	-	-	-	-	-	-	-	-	-	-	-	-	-	-	-	-	-	-	-	-	-	-	-	-	-	-	-	-	-	-	-	-	-	+	-	-	-	-
3795	4337	putative methyltransferase	2	-	-	-	-	-	-	-	-	-	-	-	-	-	-	-	-	-	-	-	-	-	-	-	-	-	-	-	-	-	-	-	-	-	-	-	-	-	-	-	-	-	-	-	-	-	-	+	-	-	-	-	+	-	-	-	-	-
3796	4338	FtsK/SpoIIIE family protein, putative	2	-	-	-	-	-	-	-	-	-	-	-	-	-	-	-	-	-	-	-	-	-	-	-	-	-	-	-	-	-	-	-	-	-	-	-	-	-	-	-	-	-	-	-	-	-	-	-	-	-	-	-	+	+	-	-	-	-
3797	4339	FtsK/SpoIIIE family	2	-	-	-	-	-	-	-	-	-	-	-	-	-	-	-	-	-	-	-	-	-	-	-	-	-	-	-	-	-	-	-	-	-	-	-	-	-	-	-	-	-	-	-	-	-	-	+	-	-	-	-	-	+	-	-	-	-
3798	4341	Transposase, IS630-Spn1 related, Orf2	2	-	-	-	-	-	-	-	-	-	-	-	-	-	-	-	-	-	-	-	-	-	-	-	-	-	-	-	-	-	-	-	-	-	-	-	-	-	-	-	-	-	-	-	-	-	-	-	-	-	-	+	-	+	-	-	-	-
3799	4627	putative UDP-galactose--lipooligosaccharide galactosyltransferase	2	-	-	-	-	-	-	-	-	-	-	-	-	-	-	-	-	-	-	-	-	-	-	-	-	-	-	-	-	-	-	-	-	-	-	+	-	-	-	+	-	-	-	-	-	-	-	-	-	-	-	-	-	-	-	-	-	-
3800	4645	hypothetical protein	2	-	-	-	-	-	-	-	-	-	-	-	-	-	-	-	-	-	-	-	-	-	-	-	-	-	-	-	-	-	-	-	-	-	-	-	-	-	-	+	+	-	-	-	-	-	-	-	-	-	-	-	-	-	-	-	-	-
3801	4651	Hypothetical protein	2	-	-	-	-	-	-	-	-	-	-	-	-	-	-	-	-	-	-	-	-	-	-	-	-	-	-	-	-	-	-	-	-	-	-	-	-	-	-	-	-	-	-	-	-	+	+	-	-	-	-	-	-	-	-	-	-	-
3802	4679	Cystathionine beta-lyase (EC 4.4.1.8)	2	-	-	-	-	-	-	-	-	-	-	-	-	-	-	-	-	-	-	-	-	-	-	-	-	-	-	-	-	-	-	-	-	-	-	-	-	-	-	-	-	-	-	-	-	-	-	-	-	-	-	-	-	-	-	-	+	+
3803	4681	unknown protein	2	-	-	-	-	-	-	-	-	-	-	-	-	-	-	-	-	-	-	-	-	-	-	-	-	-	-	-	-	-	-	-	-	-	-	-	-	-	-	-	-	+	-	-	-	-	-	-	-	-	-	-	-	-	-	-	-	-
3804	4682	Lipopolysaccharide cholinephosphotransferase LicD1 (EC 2.7.8.-)	2	-	-	-	-	-	-	-	-	-	-	-	-	-	-	-	-	-	-	-	-	-	-	-	-	-	-	-	-	-	-	-	-	-	-	-	-	-	-	-	-	-	-	-	-	-	-	-	-	+	-	-	-	-	-	-	-	+
3805	4684	dTDP-glucose 4,6-dehydratase (EC 4.2.1.46)	2	-	-	-	-	-	-	-	-	-	-	-	-	-	-	-	-	-	-	-	-	-	-	-	-	-	-	-	-	-	-	-	-	-	-	-	-	-	-	-	-	-	-	-	-	-	-	-	-	+	-	-	-	-	-	-	-	+
3806	4686	GftB: Glycosyl transferase, family 8	2	-	-	-	-	-	-	-	-	-	-	-	-	-	-	-	-	-	-	-	-	-	-	-	-	-	-	-	-	-	-	-	-	-	-	-	-	-	-	-	-	-	-	-	-	-	-	-	-	-	-	-	-	-	-	-	+	+
3807	4687	Accessory secretory protein Asp2	2	-	-	-	-	-	-	-	-	-	-	-	-	-	-	-	-	-	-	-	-	-	-	-	-	-	-	-	-	-	-	-	-	-	-	-	-	-	-	-	-	-	-	-	-	-	-	-	-	-	-	-	-	-	-	-	+	+
3808	4695	Acetaldehyde dehydrogenase (acetylating) (EC 1.2.1.10)	2	-	-	-	-	-	-	-	-	-	-	-	-	-	-	-	-	-	-	-	-	-	-	-	-	-	-	-	-	-	-	-	-	-	-	-	-	-	-	-	-	-	-	-	-	-	-	-	-	-	+	-	+	-	-	-	-	-
3809	4698	unknown protein	2	-	-	-	-	-	-	-	-	-	-	-	-	-	-	-	-	-	-	-	-	-	-	-	-	-	-	-	-	-	-	-	-	-	-	-	-	-	-	-	-	-	-	-	-	-	-	+	+	-	-	-	-	-	-	-	-	-
3810	4699	Tn5252, Orf23	2	-	-	-	-	-	-	-	-	-	-	-	-	-	-	-	-	-	-	-	-	-	-	-	-	-	-	-	-	-	-	-	-	-	-	-	-	-	-	-	-	-	-	-	-	-	-	-	+	-	-	-	-	-	-	-	+	-
3811	4701	TnpV	2	-	-	-	-	-	-	+	-	-	-	-	-	-	-	-	-	-	-	-	-	-	-	-	-	-	-	-	-	-	-	-	-	-	-	-	-	-	-	-	-	-	-	-	-	-	-	-	+	-	-	-	-	-	-	-	-	-
3812	4704	Methionine ABC transporter substrate-binding protein	2	-	-	-	-	-	-	-	-	-	-	-	-	-	-	-	-	-	-	-	-	-	-	-	-	-	-	-	-	-	-	-	-	-	-	-	-	-	-	-	-	-	-	-	-	-	-	-	+	-	-	-	+	-	-	-	-	-
3813	4724	YbbM seven transmembrane helix protein	2	-	-	-	-	-	-	-	-	-	-	-	-	-	-	-	-	-	-	-	-	-	-	-	-	-	-	-	-	-	-	-	-	-	-	-	-	-	-	-	-	-	-	-	-	-	-	-	-	-	+	-	+	-	-	-	-	-
3814	4725	Predicted membrane protein	2	-	-	-	-	-	-	-	-	-	-	-	-	-	-	-	-	-	-	-	-	-	-	-	-	-	-	-	-	-	-	-	-	-	-	-	-	-	-	-	-	-	-	-	-	-	-	-	-	-	+	-	+	-	-	-	-	-
3815	4726	Substrate-specific component CbiM of cobalt ECF transporter	2	-	-	-	-	-	-	-	-	-	-	-	-	-	-	-	-	-	-	-	-	-	-	-	-	-	-	-	-	-	-	-	-	-	-	-	-	-	-	-	-	-	-	-	-	-	-	-	-	-	+	-	+	-	-	-	-	-
3816	4727	Ethanolamine utilization protein EutA	2	-	-	-	-	-	-	-	-	-	-	-	-	-	-	-	-	-	-	-	-	-	-	-	-	-	-	-	-	-	-	-	-	-	-	-	-	-	-	-	-	-	-	-	-	-	-	-	-	-	+	-	+	-	-	-	-	-
3817	4728	Ethanolamine utilization polyhedral-body-like protein EutL	2	-	-	-	-	-	-	-	-	-	-	-	-	-	-	-	-	-	-	-	-	-	-	-	-	-	-	-	-	-	-	-	-	-	-	-	-	-	-	-	-	-	-	-	-	-	-	-	-	-	+	-	+	-	-	-	-	-
3818	4730	Transcriptional regulator	2	-	-	-	-	-	-	-	-	-	-	-	-	-	-	-	-	-	-	-	-	-	-	-	-	-	-	-	-	-	-	-	-	-	-	-	-	-	-	-	-	-	-	-	-	-	-	-	-	-	+	-	-	+	-	-	-	-
3819	4734	Additional substrate-specific component CbiN of cobalt ECF transporter	2	-	-	-	-	-	-	-	-	-	-	-	-	-	-	-	-	-	-	-	-	-	-	-	-	-	-	-	-	-	-	-	-	-	-	-	-	-	-	-	-	-	-	-	-	-	-	-	-	-	+	-	+	-	-	-	-	-
3820	4735	Ethanolamine permease	2	-	-	-	-	-	-	-	-	-	-	-	-	-	-	-	-	-	-	-	-	-	-	-	-	-	-	-	-	-	-	-	-	-	-	-	-	-	-	-	-	-	-	-	-	-	-	-	-	-	+	-	+	-	-	-	-	-
3821	4737	putative Cro-like protein, phage associated	2	-	-	-	-	-	-	-	-	-	-	-	-	-	-	-	-	-	-	-	-	-	-	-	-	-	-	-	-	-	-	-	-	-	-	-	-	-	-	-	-	-	-	-	-	-	-	-	-	-	+	-	+	-	-	-	-	-
3822	4740	Ethanolamine ammonia-lyase heavy chain (EC 4.3.1.7)	2	-	-	-	-	-	-	-	-	-	-	-	-	-	-	-	-	-	-	-	-	-	-	-	-	-	-	-	-	-	-	-	-	-	-	-	-	-	-	-	-	-	-	-	-	-	-	-	-	-	+	-	+	-	-	-	-	-
3823	4741	Ethanolamine ammonia-lyase light chain (EC 4.3.1.7)	2	-	-	-	-	-	-	-	-	-	-	-	-	-	-	-	-	-	-	-	-	-	-	-	-	-	-	-	-	-	-	-	-	-	-	-	-	-	-	-	-	-	-	-	-	-	-	-	-	-	+	-	+	-	-	-	-	-
3824	4748	ATPase component CbiO of energizing module of cobalt ECF transporter	2	-	-	-	-	-	-	-	-	-	-	-	-	-	-	-	-	-	-	-	-	-	-	-	-	-	-	-	-	-	-	-	-	-	-	-	-	-	-	-	-	-	-	-	-	-	-	-	-	-	+	-	+	-	-	-	-	-
3825	4749	immunity protein, probable	2	-	-	-	-	-	-	-	-	-	-	-	-	-	-	-	-	-	-	-	-	-	-	-	-	-	-	-	-	-	-	-	-	-	-	-	-	-	-	-	-	-	-	-	-	-	-	-	-	-	+	-	-	+	-	-	-	-
3826	4756	1,3-propanediol dehydrogenase (EC 1.1.1.202)	2	-	-	-	-	-	-	-	-	-	-	-	-	-	-	-	-	-	-	-	-	-	-	-	-	-	-	-	-	-	-	-	-	-	-	-	-	-	-	-	-	-	-	-	-	-	-	-	-	-	+	-	+	-	-	-	-	-
3827	4757	YbbL ABC transporter ATP-binding protein	2	-	-	-	-	-	-	-	-	-	-	-	-	-	-	-	-	-	-	-	-	-	-	-	-	-	-	-	-	-	-	-	-	-	-	-	-	-	-	-	-	-	-	-	-	-	-	-	-	-	+	-	+	-	-	-	-	-
3828	4758	Ethanolamine utilization protein EutQ	2	-	-	-	-	-	-	-	-	-	-	-	-	-	-	-	-	-	-	-	-	-	-	-	-	-	-	-	-	-	-	-	-	-	-	-	-	-	-	-	-	-	-	-	-	-	-	-	-	-	+	-	+	-	-	-	-	-
3829	4777	putative antirepressor - phage associated	2	-	-	-	-	-	-	-	-	-	-	-	-	-	-	-	-	-	-	-	-	-	-	-	-	-	-	-	-	-	-	+	-	-	-	-	-	-	-	-	-	-	-	-	-	-	-	-	-	-	-	-	-	-	+	-
3830	4781	conserved domain protein	2	-	-	-	-	-	-	-	-	-	-	-	-	-	-	-	-	-	-	-	-	+	+	-	-	-	-	-	-	-	-	-	-	-	-	-	-	-	-	-	-	-	-	-	-	-	-	-	-	-	-	-	-	-	-	-	-	-
3831	4793	hypothetical protein	2	-	-	-	-	-	-	-	-	-	-	-	-	-	-	-	-	-	-	-	-	-	-	-	-	-	-	-	-	-	-	-	-	-	-	-	-	-	-	-	-	-	-	-	-	-	-	-	-	-	-	+	+	-	-	-	-	-
3832	4795	hypothetical protein	2	-	-	-	-	-	-	-	-	-	-	-	-	-	-	-	-	-	-	-	-	-	-	-	-	-	-	-	-	-	-	-	-	-	-	-	-	-	-	-	-	-	+	-	-	-	-	-	-	-	-	+	-	-	-	-
3833	4800	Phage terminase, large subunit	2	-	-	-	-	-	-	-	-	-	-	-	-	-	-	-	-	-	-	-	-	-	-	-	-	-	-	-	-	-	-	-	-	-	-	-	-	-	-	-	-	-	-	-	-	-	-	-	-	-	-	-	+	-	-	+	-	-
3834	4823	hypothetical protein	2	-	-	-	-	-	-	-	-	-	-	-	-	-	-	-	-	-	-	-	-	-	-	-	-	-	-	-	-	-	-	-	-	-	-	-	-	-	-	-	-	-	+	-	-	-	-	-	-	-	-	-	-	-	+	-
3835	4879	Mobile element protein	2	-	-	-	-	-	-	-	-	-	-	-	-	-	-	-	-	-	-	-	-	-	-	-	-	-	-	-	-	-	+	+	-	-	-	-	-	-	-	-	-	-	-	-	-	-	-	-	-	-	-	-	-	-	-	-
3836	5128	hypothetical protein	2	-	-	-	-	-	-	-	-	+	-	-	-	-	-	-	-	-	-	-	-	-	-	-	-	-	-	-	-	-	-	-	-	-	-	-	-	-	-	-	-	-	-	-	-	-	-	+	-	-	-	-	-	-	-	-
3837	5161	hypothetical protein	2	-	-	-	-	-	-	-	-	-	-	-	-	-	-	-	-	-	-	-	-	-	-	-	-	+	+	-	-	-	-	-	-	-	-	-	-	-	-	-	-	-	-	-	-	-	-	-	-	-	-	-	-	-	-	-
3838	5162	hypothetical protein	2	-	-	-	-	-	-	-	-	-	-	-	-	-	-	-	-	-	-	-	-	-	-	-	-	+	-	-	-	-	-	-	-	-	-	-	-	-	-	-	-	-	-	-	-	-	-	-	-	-	-	-	-	-	+	-
3839	5170	hypothetical protein	2	-	-	-	-	-	-	-	-	-	-	-	-	-	-	-	-	-	-	-	-	-	-	-	-	-	-	-	-	-	-	-	-	-	-	-	-	-	-	+	-	-	-	-	-	-	-	-	-	-	-	-	-	-	+	-
3840	5173	site-specific recombinase, phage integrase family	2	-	-	-	-	-	-	-	-	-	-	-	-	-	-	-	-	-	-	-	-	-	-	-	-	-	-	-	-	-	-	-	-	-	-	-	-	-	-	+	-	-	-	-	-	-	-	-	-	-	-	-	-	-	+	-
3841	5175	Argininosuccinate synthase (EC 6.3.4.5)	2	-	-	-	-	-	-	-	-	-	-	-	-	-	-	-	-	-	-	-	-	-	-	-	-	-	-	-	-	-	-	-	-	-	-	-	-	-	-	+	+	-	-	-	-	-	-	-	-	-	-	-	-	-	-	-
3842	5191	Cell wall surface anchor family protein	2	-	-	-	-	-	-	-	-	-	-	-	-	-	-	-	-	-	-	-	-	-	-	-	-	-	-	-	-	-	-	-	-	-	-	-	-	-	-	+	+	-	-	-	-	-	-	-	-	-	-	-	-	-	-	-
3843	5213	ATP-dependent DNA helicase pcrA (EC 3.6.1.-)	2	-	-	-	-	-	-	-	-	-	-	-	-	-	-	-	-	-	-	-	-	-	-	+	-	-	-	-	-	-	-	-	-	-	-	-	-	-	-	-	-	-	-	-	-	+	-	-	-	-	-	-	-	-	-	-
3844	5216	ABC transporter permease protein	2	-	-	-	-	-	-	-	-	-	-	-	-	-	-	-	-	-	-	-	-	-	-	+	+	-	-	-	-	-	-	-	-	-	-	-	-	-	-	-	-	-	-	-	-	-	-	-	-	-	-	-	-	-	-	-
3845	5221	hypothetical protein	2	-	-	-	-	-	-	-	-	-	-	-	-	-	-	-	-	-	-	-	-	-	-	+	-	-	-	-	-	-	-	-	-	-	-	-	-	-	-	-	-	-	-	-	-	+	-	-	-	-	-	-	-	-	-	-
3846	5231	putative transcriptional regulator	2	-	-	-	-	-	-	-	-	-	-	-	-	-	-	-	-	-	-	-	-	-	-	-	-	-	-	-	-	-	-	-	-	-	-	-	-	-	-	-	-	-	-	-	-	-	-	-	-	-	-	-	-	+	-	-	+
3847	5232	Glutamine amidotransferase, class I	2	-	-	-	-	-	-	-	-	-	-	-	-	-	-	-	-	-	-	-	-	-	-	-	-	-	-	-	-	-	-	-	-	-	-	-	-	-	-	-	-	-	-	-	-	-	-	-	-	-	-	-	-	-	-	-	+	+
3848	5234	COG0459: Chaperonin GroEL (HSP60 family)	2	-	-	-	-	-	-	-	-	-	-	-	-	-	-	-	-	-	-	-	-	-	-	-	-	-	-	-	-	-	-	-	-	-	-	-	-	-	-	-	-	-	-	-	-	-	-	-	-	-	-	-	-	-	-	-	+	+
3849	5239	hypothetical protein	2	-	-	-	-	-	-	-	-	-	-	-	-	-	-	-	-	-	-	-	-	-	-	-	-	-	-	-	-	-	-	-	-	-	-	-	-	-	-	-	-	-	-	-	-	-	-	-	-	-	-	-	-	-	-	-	+	+
3850	5240	Endoribonuclease L-PSP	2	-	-	-	-	-	-	-	-	-	-	-	-	-	-	-	-	-	-	-	-	-	-	-	-	-	-	-	-	-	-	-	-	-	-	-	-	-	-	-	-	-	-	-	-	-	-	-	-	-	-	-	-	-	-	-	+	+
3851	5243	contains amino acid permease domain	2	-	-	-	-	-	-	-	-	-	-	-	-	-	-	-	-	-	-	-	-	-	-	-	-	-	-	-	-	-	-	-	-	-	-	-	-	-	-	-	-	-	-	-	-	-	-	-	-	-	-	-	-	-	-	-	+	+
3852	5246	YheO-like PAS domain	2	-	-	-	-	-	-	-	-	-	-	-	-	-	-	-	-	-	-	-	-	-	-	-	-	-	-	-	-	-	-	-	-	-	-	-	-	-	-	-	-	-	-	-	-	-	-	-	-	-	-	-	-	-	-	-	+	+
3853	5249	hypothetical protein	2	-	-	-	-	-	-	-	-	-	-	-	-	-	-	-	-	-	-	-	-	-	-	-	-	-	-	-	-	-	-	-	-	-	-	-	-	-	-	-	-	-	-	-	-	-	-	-	+	+	-	-	-	-	-	-	-	-
3854	5250	Phage integrase (Site-specific recombinase)	2	-	-	-	-	-	-	-	-	-	-	-	-	-	-	-	-	-	-	-	-	-	-	-	-	-	-	-	-	-	-	-	-	-	-	-	-	-	-	-	-	-	-	+	-	-	-	-	+	-	-	-	-	-	-	-	-	-
3855	5261	hypothetical protein	2	-	-	-	-	-	-	-	-	-	-	-	-	-	-	-	-	-	-	-	-	-	-	-	-	-	-	-	-	-	-	-	-	-	-	-	-	-	-	-	-	-	-	-	-	-	-	-	+	-	-	-	-	-	-	-	+	-
3856	5265	Replication protein	2	-	-	-	-	-	-	-	-	-	-	-	-	-	-	-	-	-	-	-	-	-	-	-	-	-	-	-	-	-	-	-	-	-	-	-	-	-	-	-	-	-	-	-	-	-	-	-	+	-	-	+	-	-	-	-	-	-
3857	5270	Hypothetical protein, CF-38 family	2	-	-	-	-	-	-	-	-	-	-	-	-	-	-	-	-	-	-	-	-	-	-	-	-	-	-	-	-	-	-	-	-	-	-	-	-	-	-	-	-	+	+	-	-	-	-	-	-	-	-	-	-	-	-	-	-	-
3858	5276	hypothetical protein	2	-	-	-	-	-	-	-	-	-	-	-	-	-	-	-	-	-	-	-	-	-	-	-	-	-	-	-	-	-	-	-	-	-	-	-	-	-	-	-	-	+	+	-	-	-	-	-	-	-	-	-	-	-	-	-	-	-
3859	5281	IS1381	2	-	-	-	-	-	-	-	-	-	-	-	-	-	-	-	-	-	-	-	-	-	-	-	-	-	-	-	-	-	-	-	-	-	-	+	-	-	-	-	-	+	-	-	-	-	-	-	-	-	-	-	-	-	-	-	-	-
3860	5292	Choline permease LicB	2	-	-	-	-	-	-	-	-	-	-	-	-	-	-	-	-	-	-	-	-	-	-	-	-	-	-	-	-	-	-	-	-	-	-	-	-	-	-	-	-	+	-	-	-	-	-	-	-	-	-	-	-	+	-	-	-	-
3861	5293	Sortase A, LPXTG specific	2	-	-	-	-	-	-	-	-	-	-	-	-	-	-	+	-	-	-	-	-	-	-	-	-	-	-	-	-	-	-	-	-	-	-	-	-	-	-	-	-	+	-	-	-	-	-	-	-	-	-	-	-	-	-	-	-	-
3862	5297	Type I restriction-modification system, restriction subunit R (EC 3.1.21.3)	2	-	-	-	-	-	-	-	-	-	-	-	-	-	-	-	-	-	-	-	-	-	-	-	-	-	-	-	-	-	-	-	-	-	-	-	-	-	-	-	-	+	-	-	-	-	-	-	-	-	+	-	-	-	-	-	-	-
3863	5300	hypothetical protein	2	-	-	-	-	-	-	-	-	-	-	-	-	-	-	-	-	-	-	-	-	-	-	-	-	-	-	-	-	-	-	-	-	-	-	-	-	-	-	-	-	+	-	-	-	-	-	+	-	-	-	-	-	-	-	-	-	-
3864	5311	Propanediol utilization protein PduV	2	-	-	-	-	-	-	-	-	-	-	-	-	-	-	-	-	-	-	-	-	-	-	-	-	-	-	-	-	-	-	-	-	-	-	-	-	-	-	-	-	-	-	-	-	-	-	-	-	-	+	-	+	-	-	-	-	-
3865	5312	Cobalamin synthase	2	-	-	-	-	-	-	-	-	-	-	-	-	-	-	-	-	-	-	-	-	-	-	-	-	-	-	-	-	-	-	-	-	-	-	-	-	-	-	-	-	-	-	-	-	-	-	-	-	-	+	-	+	-	-	-	-	-
3866	5313	Cobalt-precorrin-6y C5-methyltransferase (EC 2.1.1.-)	2	-	-	-	-	-	-	-	-	-	-	-	-	-	-	-	-	-	-	-	-	-	-	-	-	-	-	-	-	-	-	-	-	-	-	-	-	-	-	-	-	-	-	-	-	-	-	-	-	-	+	-	+	-	-	-	-	-
3867	5316	Hypothetical Cytosolic Protein	2	-	-	-	-	-	-	-	-	-	-	-	-	-	-	-	-	-	-	-	-	-	-	-	-	-	-	-	-	-	-	-	-	-	-	-	-	-	-	-	-	-	-	-	-	-	-	-	-	-	+	-	+	-	-	-	-	-
3868	5317	Propanediol dehydratase reactivation factor large subunit	2	-	-	-	-	-	-	-	-	-	-	-	-	-	-	-	-	-	-	-	-	-	-	-	-	-	-	-	-	-	-	-	-	-	-	-	-	-	-	-	-	-	-	-	-	-	-	-	-	-	+	-	+	-	-	-	-	-
3869	5318	Cobalt-precorrin-6x reductase (EC 1.3.1.54)	2	-	-	-	-	-	-	-	-	-	-	-	-	-	-	-	-	-	-	-	-	-	-	-	-	-	-	-	-	-	-	-	-	-	-	-	-	-	-	-	-	-	-	-	-	-	-	-	-	-	+	-	+	-	-	-	-	-
3870	5319	Siroheme synthase / Precorrin-2 oxidase (EC 1.3.1.76)	2	-	-	-	-	-	-	-	-	-	-	-	-	-	-	-	-	-	-	-	-	-	-	-	-	-	-	-	-	-	-	-	-	-	-	-	-	-	-	-	-	-	-	-	-	-	-	-	-	-	+	-	+	-	-	-	-	-
3871	5320	Adenosylcobinamide-phosphate synthase	2	-	-	-	-	-	-	-	-	-	-	-	-	-	-	-	-	-	-	-	-	-	-	-	-	-	-	-	-	-	-	-	-	-	-	-	-	-	-	-	-	-	-	-	-	-	-	-	-	-	+	-	+	-	-	-	-	-
3872	5321	Porphobilinogen deaminase (EC 2.5.1.61)	2	-	-	-	-	-	-	-	-	-	-	-	-	-	-	-	-	-	-	-	-	-	-	-	-	-	-	-	-	-	-	-	-	-	-	-	-	-	-	-	-	-	-	-	-	-	-	-	-	-	+	-	+	-	-	-	-	-
3873	5322	Cobalt-precorrin-6 synthase, anaerobic	2	-	-	-	-	-	-	-	-	-	-	-	-	-	-	-	-	-	-	-	-	-	-	-	-	-	-	-	-	-	-	-	-	-	-	-	-	-	-	-	-	-	-	-	-	-	-	-	-	-	+	-	+	-	-	-	-	-
3874	5323	hypothetical protein	2	-	-	-	-	-	-	-	-	-	-	-	-	-	-	-	-	-	-	-	-	-	-	-	-	-	-	-	-	-	-	-	-	-	-	-	-	-	-	-	-	-	-	-	-	-	-	-	-	-	+	-	+	-	-	-	-	-
3875	5325	Cobalt-precorrin-2 C20-methyltransferase (EC 2.1.1.130)	2	-	-	-	-	-	-	-	-	-	-	-	-	-	-	-	-	-	-	-	-	-	-	-	-	-	-	-	-	-	-	-	-	-	-	-	-	-	-	-	-	-	-	-	-	-	-	-	-	-	+	-	+	-	-	-	-	-
3876	5326	Glycerol uptake facilitator protein @ Propanediol diffusion facilitator	2	-	-	-	-	-	-	-	-	-	-	-	-	-	-	-	-	-	-	-	-	-	-	-	-	-	-	-	-	-	-	-	-	-	-	-	-	-	-	-	-	-	-	-	-	-	-	-	-	-	+	-	+	-	-	-	-	-
3877	5327	Cobalt-precorrin-3b C17-methyltransferase	2	-	-	-	-	-	-	-	-	-	-	-	-	-	-	-	-	-	-	-	-	-	-	-	-	-	-	-	-	-	-	-	-	-	-	-	-	-	-	-	-	-	-	-	-	-	-	-	-	-	+	-	+	-	-	-	-	-
3878	5328	Porphobilinogen synthase (EC 4.2.1.24)	2	-	-	-	-	-	-	-	-	-	-	-	-	-	-	-	-	-	-	-	-	-	-	-	-	-	-	-	-	-	-	-	-	-	-	-	-	-	-	-	-	-	-	-	-	-	-	-	-	-	+	-	+	-	-	-	-	-
3879	5329	Propanediol dehydratase small subunit (EC 4.2.1.28)	2	-	-	-	-	-	-	-	-	-	-	-	-	-	-	-	-	-	-	-	-	-	-	-	-	-	-	-	-	-	-	-	-	-	-	-	-	-	-	-	-	-	-	-	-	-	-	-	-	-	+	-	+	-	-	-	-	-
3880	5333	Cobyric acid synthase	2	-	-	-	-	-	-	-	-	-	-	-	-	-	-	-	-	-	-	-	-	-	-	-	-	-	-	-	-	-	-	-	-	-	-	-	-	-	-	-	-	-	-	-	-	-	-	-	-	-	+	-	+	-	-	-	-	-
3881	5334	Cobyrinic acid A,C-diamide synthase	2	-	-	-	-	-	-	-	-	-	-	-	-	-	-	-	-	-	-	-	-	-	-	-	-	-	-	-	-	-	-	-	-	-	-	-	-	-	-	-	-	-	-	-	-	-	-	-	-	-	+	-	+	-	-	-	-	-
3882	5337	Glutamyl-tRNA reductase (EC 1.2.1.70)	2	-	-	-	-	-	-	-	-	-	-	-	-	-	-	-	-	-	-	-	-	-	-	-	-	-	-	-	-	-	-	-	-	-	-	-	-	-	-	-	-	-	-	-	-	-	-	-	-	-	+	-	+	-	-	-	-	-
3883	5338	Alpha-ribazole-5'-phosphate phosphatase (EC 3.1.3.73)	2	-	-	-	-	-	-	-	-	-	-	-	-	-	-	-	-	-	-	-	-	-	-	-	-	-	-	-	-	-	-	-	-	-	-	-	-	-	-	-	-	-	-	-	-	-	-	-	-	-	+	-	+	-	-	-	-	-
3884	5340	Cob(I)alamin adenosyltransferase PduO (EC 2.5.1.17)	2	-	-	-	-	-	-	-	-	-	-	-	-	-	-	-	-	-	-	-	-	-	-	-	-	-	-	-	-	-	-	-	-	-	-	-	-	-	-	-	-	-	-	-	-	-	-	-	-	-	+	-	+	-	-	-	-	-
3885	5341	Ethanolamine two-component response regulator	2	-	-	-	-	-	-	-	-	-	-	-	-	-	-	-	-	-	-	-	-	-	-	-	-	-	-	-	-	-	-	-	-	-	-	-	-	-	-	-	-	-	-	-	-	-	-	-	-	-	+	-	+	-	-	-	-	-
3886	5343	Glutamate-1-semialdehyde aminotransferase (EC 5.4.3.8)	2	-	-	-	-	-	-	-	-	-	-	-	-	-	-	-	-	-	-	-	-	-	-	-	-	-	-	-	-	-	-	-	-	-	-	-	-	-	-	-	-	-	-	-	-	-	-	-	-	-	+	-	+	-	-	-	-	-
3887	5344	Sirohydrochlorin cobaltochelatase CbiK (EC 4.99.1.3)	2	-	-	-	-	-	-	-	-	-	-	-	-	-	-	-	-	-	-	-	-	-	-	-	-	-	-	-	-	-	-	-	-	-	-	-	-	-	-	-	-	-	-	-	-	-	-	-	-	-	+	-	+	-	-	-	-	-
3888	5345	Cobalt-precorrin-4 C11-methyltransferase (EC 2.1.1.133)	2	-	-	-	-	-	-	-	-	-	-	-	-	-	-	-	-	-	-	-	-	-	-	-	-	-	-	-	-	-	-	-	-	-	-	-	-	-	-	-	-	-	-	-	-	-	-	-	-	-	+	-	+	-	-	-	-	-
3889	5346	Cobalamin biosynthesis protein CbiG	2	-	-	-	-	-	-	-	-	-	-	-	-	-	-	-	-	-	-	-	-	-	-	-	-	-	-	-	-	-	-	-	-	-	-	-	-	-	-	-	-	-	-	-	-	-	-	-	-	-	+	-	+	-	-	-	-	-
3890	5348	Ethanolamine utilization polyhedral-body-like protein EutN	2	-	-	-	-	-	-	-	-	-	-	-	-	-	-	-	-	-	-	-	-	-	-	-	-	-	-	-	-	-	-	-	-	-	-	-	-	-	-	-	-	-	-	-	-	-	-	-	-	-	+	-	+	-	-	-	-	-
3891	5349	Propanediol dehydratase medium subunit (EC 4.2.1.28)	2	-	-	-	-	-	-	-	-	-	-	-	-	-	-	-	-	-	-	-	-	-	-	-	-	-	-	-	-	-	-	-	-	-	-	-	-	-	-	-	-	-	-	-	-	-	-	-	-	-	+	-	+	-	-	-	-	-
3892	5350	Propanediol utilization polyhedral body protein PduB	2	-	-	-	-	-	-	-	-	-	-	-	-	-	-	-	-	-	-	-	-	-	-	-	-	-	-	-	-	-	-	-	-	-	-	-	-	-	-	-	-	-	-	-	-	-	-	-	-	-	+	-	+	-	-	-	-	-
3893	5352	Cobalt-precorrin-6y C15-methyltransferase [decarboxylating] (EC 2.1.1.-)	2	-	-	-	-	-	-	-	-	-	-	-	-	-	-	-	-	-	-	-	-	-	-	-	-	-	-	-	-	-	-	-	-	-	-	-	-	-	-	-	-	-	-	-	-	-	-	-	-	-	+	-	+	-	-	-	-	-
3894	5353	protein with N-terminal chaperone domain	2	-	-	-	-	-	-	-	-	-	-	-	-	-	-	-	-	-	-	-	-	-	-	-	-	-	-	-	-	-	-	-	-	-	-	-	-	-	-	-	-	-	-	-	-	-	-	-	-	-	+	-	+	-	-	-	-	-
3895	5354	Nicotinate-nucleotide--dimethylbenzimidazole phosphoribosyltransferase (EC 2.4.2.21)	2	-	-	-	-	-	-	-	-	-	-	-	-	-	-	-	-	-	-	-	-	-	-	-	-	-	-	-	-	-	-	-	-	-	-	-	-	-	-	-	-	-	-	-	-	-	-	-	-	-	+	-	+	-	-	-	-	-
3896	5355	Adenosylcobinamide-phosphate guanylyltransferase (EC 2.7.7.62)	2	-	-	-	-	-	-	-	-	-	-	-	-	-	-	-	-	-	-	-	-	-	-	-	-	-	-	-	-	-	-	-	-	-	-	-	-	-	-	-	-	-	-	-	-	-	-	-	-	-	+	-	+	-	-	-	-	-
3897	5356	Uroporphyrinogen-III methyltransferase (EC 2.1.1.107) / Uroporphyrinogen-III synthase (EC 4.2.1.75)	2	-	-	-	-	-	-	-	-	-	-	-	-	-	-	-	-	-	-	-	-	-	-	-	-	-	-	-	-	-	-	-	-	-	-	-	-	-	-	-	-	-	-	-	-	-	-	-	-	-	+	-	+	-	-	-	-	-
3898	5359	hypothetical protein	2	-	-	-	-	-	-	-	-	-	-	-	-	-	-	-	-	-	-	-	-	-	-	-	-	-	-	-	-	-	-	-	-	-	-	-	-	-	-	-	-	-	-	-	-	-	-	-	-	-	+	-	-	+	-	-	-	-
3899	5361	Propanediol dehydratase large subunit (EC 4.2.1.28)	2	-	-	-	-	-	-	-	-	-	-	-	-	-	-	-	-	-	-	-	-	-	-	-	-	-	-	-	-	-	-	-	-	-	-	-	-	-	-	-	-	-	-	-	-	-	-	-	-	-	+	-	+	-	-	-	-	-
3900	5363	L-threonine 3-O-phosphate decarboxylase (EC 4.1.1.81)	2	-	-	-	-	-	-	-	-	-	-	-	-	-	-	-	-	-	-	-	-	-	-	-	-	-	-	-	-	-	-	-	-	-	-	-	-	-	-	-	-	-	-	-	-	-	-	-	-	-	+	-	+	-	-	-	-	-
3901	5366	DNA repair protein RadA	2	-	-	-	-	-	-	-	-	-	-	-	-	+	-	-	-	-	-	-	-	-	-	-	-	-	-	-	-	-	-	-	-	-	-	-	-	-	-	-	-	-	-	-	-	-	-	-	-	-	-	-	-	-	+	-
3902	5368	repressor-related protein	2	-	-	-	-	-	-	-	-	-	-	-	-	-	-	+	-	-	-	-	-	-	-	-	-	-	-	-	-	-	-	-	-	-	-	-	-	-	-	-	-	-	-	-	-	-	-	+	-	-	-	-	-	-	-	-
3903	5376	hypothetical protein	2	-	-	-	-	-	-	-	-	-	-	-	-	-	-	-	-	-	-	-	-	-	-	-	-	-	-	-	-	-	-	-	-	-	-	-	-	-	-	-	-	+	-	+	-	-	-	-	-	-	-	-	-	-	-	-
3904	5382	hypothetical protein	2	-	-	-	-	-	-	-	-	-	-	-	-	-	-	-	-	-	-	-	+	-	-	-	-	-	-	-	-	-	-	-	-	-	-	-	-	-	-	-	-	-	-	+	-	-	-	-	-	-	-	-	-	-	-	-
3905	5383	putative excisionase	2	-	-	-	-	-	-	-	-	-	-	-	-	-	-	-	-	-	-	-	-	-	-	-	-	-	-	-	-	-	-	-	-	-	-	-	-	-	-	-	-	-	-	+	-	-	-	-	-	+	-	-	-	-	-	-
3906	5387	Zinc-finger protein	2	-	-	-	-	-	-	-	-	+	-	-	-	-	-	-	-	-	-	-	-	-	-	-	-	-	-	-	-	-	-	-	-	-	-	-	-	-	-	-	-	-	-	+	-	-	-	-	-	-	-	-	-	-	-	-
3907	5397	Transposase, IS4	2	-	-	-	-	-	-	-	-	-	-	-	-	-	-	-	-	-	-	-	-	-	-	-	-	-	-	-	-	-	-	-	-	-	+	+	-	-	-	-	-	-	-	-	-	-	-	-	-	-	-	-	-	-	-	-
3908	5468	IFN-response binding factor 1	2	-	-	-	-	-	-	-	-	-	-	-	-	-	-	-	-	-	-	-	-	-	-	-	-	-	-	-	-	-	-	+	-	-	-	-	-	-	-	-	-	-	-	-	-	+	-	-	-	-	-	-	-	-	-	-
3909	5495	SNF2 family protein	2	-	-	-	-	-	-	+	-	-	-	-	-	-	-	-	-	-	-	-	-	-	-	-	-	-	-	-	-	-	-	-	-	-	-	-	-	-	-	-	-	-	+	-	-	-	-	-	-	-	-	-	-	-	-	-
3910	5496	hypothetical protein	2	-	-	-	-	-	-	-	-	-	-	-	-	-	-	-	-	-	-	-	-	-	-	-	-	-	-	-	-	-	-	-	-	-	-	-	-	-	-	-	-	+	+	-	-	-	-	-	-	-	-	-	-	-	-	-

Table S5. Precence/absence of each HG among 57 *S. suis* strains

				1																13			25					28						104		94			76			53/54		Divergent *S. suis*																
Index[a]	ClusterID	Gene products	Members	98HAH33	05ZYH33	SC84	A7	SS12	P1/7	DAT273	NIAH11434	JS14	GZ1	BM407	S735	13730	MNCM07	NCTC 10446	14636	NCTC 10237	ST1	2651	89-1591	MNCM04	MNCM25	8074	D9	4961	ST3	MNCM43	DAT292	DAT260	NIAH11435	MNCM21	MNCM50	89-2479	DAT234	DAT290	93A	42A	NT77	6407	11538	8830	12814	92-1400	89-590	2726	2524	89-3576-3	4417	D12	10581	89-5259	88-5299A	R61	92-1191	14A	92-4172	22083
3911	5497	hypothetical protein	2	-	-	-	-	-	-	-	-	-	-	-	-	-	-	-	-	-	-	-	-	-	-	-	-	-	-	-	-	-	-	-	-	-	-	-	-	-	-	-	-	-	-	+	+	-	-	-	-	-	-	-	-	-	-	-	-	-
3912	5505	replication initiation protein A	2	-	-	-	-	-	-	-	-	-	-	-	-	-	-	-	-	-	-	-	-	-	-	-	-	-	-	-	-	-	-	-	-	-	-	-	-	-	-	-	-	-	-	+	-	-	-	-	-	-	-	-	-	-	-	-	+	-
3913	5579	Phage transcriptional activator	2	-	-	-	-	-	-	-	-	-	-	-	-	-	-	-	-	-	-	-	-	-	-	-	-	-	-	-	-	-	-	-	-	-	+	-	-	-	-	-	-	-	-	-	-	-	-	-	-	-	-	-	-	+	-	-	-	-
3914	5959	hypothetical protein	2	-	-	-	-	-	-	-	-	-	-	-	-	-	-	-	-	-	-	-	-	-	-	-	-	-	-	-	-	-	-	-	-	-	-	-	-	-	-	-	-	-	-	-	-	-	-	-	-	-	-	+	-	-	+	-	-	-
3915	5980	Phage replication initiation	2	-	-	-	-	-	-	-	-	-	-	-	-	-	-	-	-	+	-	-	-	-	-	-	-	-	-	-	-	-	-	-	-	-	-	-	-	-	-	-	-	-	-	-	-	-	-	-	-	-	-	-	-	-	+	-	-	-
3916	5982	hypothetical protein	2	-	-	-	-	-	-	-	-	-	-	-	-	-	-	-	-	-	-	-	-	-	-	-	-	-	-	-	-	-	-	-	-	-	-	-	-	-	-	-	-	+	-	-	-	-	-	-	-	-	+	-	-	-	-	-	-	-
3917	6006	hypothetical protein	2	-	-	-	-	-	-	-	-	-	-	-	-	-	-	-	-	-	-	-	-	-	-	-	-	-	-	-	-	-	-	-	-	-	-	-	-	-	-	+	+	-	-	-	-	-	-	-	-	-	-	-	-	-	-	-	-	-
3918	6007	hypothetical protein	2	-	-	-	-	-	-	-	-	-	-	-	-	-	-	-	-	-	-	-	-	-	-	-	-	-	-	-	-	-	-	-	-	-	-	-	-	-	-	+	+	-	-	-	-	-	-	-	-	-	-	-	-	-	-	-	-	-
3919	6010	hypothetical protein	2	-	-	-	-	-	-	-	-	-	-	-	-	-	-	-	-	-	-	-	-	-	-	-	-	-	-	-	-	-	-	-	-	-	-	-	-	-	-	+	+	-	-	-	-	-	-	-	-	-	-	-	-	-	-	-	-	-
3920	6011	FIG131328: Predicted ATP-dependent endonuclease of the OLD family	2	-	-	-	-	-	-	-	-	-	-	-	-	-	-	-	-	-	-	-	-	-	-	-	-	-	-	-	-	-	-	-	-	-	-	-	-	-	-	+	+	-	-	-	-	-	-	-	-	-	-	-	-	-	-	-	-	-
3921	6012	hypothetical protein	2	-	-	-	-	-	-	-	-	-	-	-	-	-	-	-	-	-	-	-	-	-	-	-	-	-	-	-	-	-	-	-	-	-	-	-	-	-	-	+	+	-	-	-	-	-	-	-	-	-	-	-	-	-	-	-	-	-
3922	6016	hypothetical protein	2	-	-	-	-	-	-	-	-	-	-	-	-	-	-	-	-	-	-	-	-	-	-	-	-	-	-	-	-	-	-	-	-	-	-	-	-	-	-	+	+	-	-	-	-	-	-	-	-	-	-	-	-	-	-	-	-	-
3923	6019	abortive infection bacteriophage resistance protein	2	-	-	-	-	-	-	-	-	-	-	-	-	-	-	-	-	-	-	-	-	-	-	-	-	-	-	-	-	-	-	-	-	-	-	-	-	-	-	+	-	-	-	-	-	-	-	-	-	-	-	-	-	-	-	-	+	-
3924	6020	no hits	2	-	-	-	-	-	-	-	-	-	-	-	-	-	-	-	-	-	-	-	-	-	-	-	-	-	-	-	-	-	-	-	-	-	-	-	-	-	-	+	+	-	-	-	-	-	-	-	-	-	-	-	-	-	-	-	-	-
3925	6022	hypothetical protein	2	-	-	-	-	-	-	-	-	-	-	-	-	-	-	-	-	-	-	-	-	-	-	-	-	-	-	-	-	-	-	-	-	-	-	-	-	-	-	+	+	-	-	-	-	-	-	-	-	-	-	-	-	-	-	-	-	-
3926	6026	hypothetical protein	2	-	-	-	-	-	-	-	-	-	-	-	-	-	-	-	-	-	-	-	-	-	-	-	-	-	-	-	-	-	-	-	-	-	-	-	-	-	-	+	+	-	-	-	-	-	-	-	-	-	-	-	-	-	-	-	-	-
3927	6029	hypothetical protein	2	-	-	-	-	-	-	-	-	-	-	-	-	-	-	-	-	-	-	-	-	-	-	-	-	-	-	-	-	-	-	-	-	-	-	-	-	-	-	+	+	-	-	-	-	-	-	-	-	-	-	-	-	-	-	-	-	-
3928	6048	sepS6	2	-	-	-	-	-	-	-	-	-	-	-	-	-	-	-	-	-	-	-	-	-	-	-	-	-	-	-	-	-	-	-	-	-	-	-	-	-	-	-	-	-	-	-	-	-	+	-	-	-	-	-	-	-	-	-	+	-
3929	6049	hypothetical protein	2	-	-	-	-	-	-	-	-	-	-	-	-	-	-	-	-	-	-	+	-	-	-	-	-	-	-	-	-	-	-	-	-	-	-	-	-	-	-	-	-	-	-	-	-	-	+	-	-	-	-	-	-	-	-	-	-	-
3930	6050	Capsular polysaccharide repeat unit transporter CpsL	2	-	-	-	-	-	-	-	-	-	-	-	-	-	-	-	-	-	-	-	-	-	-	-	-	-	-	-	-	-	-	-	-	-	-	-	-	-	-	-	-	-	-	-	-	+	+	-	-	-	-	-	-	-	-	-	-	-
3931	6052	hypothetical protein	2	-	-	-	-	-	-	-	-	-	-	-	-	-	-	-	-	-	-	-	-	-	-	-	-	-	-	-	-	-	-	-	-	-	-	-	-	-	-	-	-	-	-	-	-	-	-	-	-	-	+	-	-	-	-	-	+	-
3932	6054	glycosyl transferase, group 1	2	-	-	-	-	-	-	-	-	-	-	-	-	-	-	-	-	-	-	-	-	-	-	+	+	-	-	-	-	-	-	-	-	-	-	-	-	-	-	-	-	-	-	-	-	-	-	-	-	-	-	-	-	-	-	-	-	-
3933	6055	polysaccharide polymerase	2	-	-	-	-	-	-	-	-	-	-	-	-	-	-	-	-	-	-	-	-	-	-	+	+	-	-	-	-	-	-	-	-	-	-	-	-	-	-	-	-	-	-	-	-	-	-	-	-	-	-	-	-	-	-	-	-	-
3934	6056	ADP-ribose pyrophosphatase	2	-	-	-	-	-	-	-	-	-	-	-	-	-	-	-	-	-	-	-	-	-	-	+	-	-	-	-	-	-	-	-	-	-	-	-	-	-	-	-	-	-	-	-	-	-	-	-	+	-	-	-	-	-	-	-	-	-
3935	6057	Transposase and inactivated derivative	2	-	-	-	-	-	-	-	-	-	-	-	-	-	-	-	-	-	-	-	-	-	-	-	-	-	-	-	-	-	-	-	-	-	-	-	-	-	-	-	-	-	-	-	-	-	-	-	-	-	-	-	-	+	-	+	-	-
3936	6058	putative glycosyl transferase	2	-	-	-	-	-	-	-	-	-	-	-	-	-	-	-	-	-	-	-	-	-	-	+	+	-	-	-	-	-	-	-	-	-	-	-	-	-	-	-	-	-	-	-	-	-	-	-	-	-	-	-	-	-	-	-	-	-
3937	6061	Membrane protein involved in the export of O-antigen, teichoic acid lipoteichoic acids	2	-	-	-	-	-	-	-	-	-	-	-	-	-	-	-	-	-	-	-	-	-	-	+	+	-	-	-	-	-	-	-	-	-	-	-	-	-	-	-	-	-	-	-	-	-	-	-	-	-	-	-	-	-	-	-	-	-
3938	6064	Lactobacillus delbrueckii phage mv4 main capsid protein Gp34 homolog lin2390	2	-	-	-	-	-	-	-	-	-	-	-	-	-	-	-	+	-	-	-	-	-	-	-	-	-	-	-	-	-	-	-	-	-	-	-	-	-	-	-	-	-	-	-	+	-	-	-	-	-	-	-	-	-	-	-	-	-
3939	6076	Beta-1,3-glucosyltransferase	2	-	-	-	-	-	-	-	-	-	-	-	-	-	-	-	-	-	-	-	-	-	-	-	-	-	-	-	-	-	-	-	-	-	-	-	-	-	-	-	-	-	-	-	-	-	-	-	-	-	-	-	-	-	-	-	+	+
3940	6079	glycosyl transferase, group 2 family protein	2	-	-	-	-	-	-	-	-	-	-	-	-	-	-	-	-	-	-	-	-	-	-	-	-	-	-	-	-	-	-	-	-	-	-	-	-	-	-	-	-	-	-	-	-	-	-	-	-	+	-	-	-	-	-	-	-	+
3941	6083	Nucleotide sugar synthetase-like protein	2	-	-	-	-	-	-	-	-	-	-	-	-	-	-	-	-	-	-	-	-	-	-	-	-	-	-	-	-	-	-	-	-	-	-	-	-	-	-	-	-	-	-	-	-	-	-	-	-	-	-	-	-	-	-	-	+	+
3942	6084	Plasmid replication protein Rep and AAA-class ATPase domain	2	-	-	-	-	-	-	-	-	-	-	-	-	-	-	-	-	-	-	-	-	-	-	-	-	-	-	-	-	-	-	-	-	-	-	-	-	-	-	-	-	-	-	+	-	-	-	-	-	-	-	-	-	-	-	-	-	+
3943	6086	Surface protein from Gram-positive cocci	2	-	-	-	-	-	-	-	-	-	-	-	-	-	-	-	-	-	-	-	-	-	-	-	-	-	-	-	-	-	-	-	-	-	-	-	-	-	-	-	-	-	-	-	-	-	-	-	-	-	-	-	-	-	-	-	+	+
3944	6087	hypothetical protein	2	-	-	-	-	-	-	-	-	-	-	-	-	-	-	-	-	-	-	-	-	-	-	-	-	-	-	-	-	-	-	-	-	-	-	-	-	-	-	-	-	-	-	-	-	-	-	-	-	-	-	-	-	-	-	-	+	+
3945	6088	Glycosyl transferase family protein	2	-	-	-	-	-	-	-	-	-	-	-	-	-	-	-	-	-	-	-	-	-	-	-	-	-	-	-	-	-	-	-	-	-	-	-	-	-	-	-	-	-	-	-	-	-	-	-	-	-	-	-	-	-	-	-	+	+
3946	6089	putative glycosyl transferase	2	-	-	-	-	-	-	-	-	-	-	-	-	-	-	-	-	-	-	-	-	-	-	-	-	-	-	-	-	-	-	-	-	-	-	-	-	-	-	-	-	-	-	-	-	-	-	-	-	+	-	-	-	-	-	-	-	+
3947	6091	Bipolar DNA helicase	2	-	-	-	-	-	-	-	-	-	-	-	-	-	-	-	-	-	-	-	-	-	-	-	-	-	-	-	-	-	-	-	-	-	-	-	-	-	-	-	-	-	-	-	-	-	-	-	+	-	+	-	-	-	-	-	-	-
3948	6096	hypothetical protein	2	-	-	-	-	-	-	-	-	-	-	-	-	-	-	-	-	-	-	-	-	-	-	-	-	-	-	-	-	-	-	-	-	-	-	-	-	-	-	-	-	-	-	-	-	-	-	-	+	-	-	-	-	-	-	-	+	-
3949	6098	hypothetical protein	2	-	-	-	-	-	-	-	-	-	-	-	-	-	-	-	-	-	-	-	-	-	-	-	-	-	-	-	-	-	-	-	-	-	-	-	-	-	-	-	-	-	-	-	-	-	-	-	+	-	+	-	-	-	-	-	-	-
3950	6099	hypothetical protein	2	-	-	-	-	-	-	-	-	-	-	-	-	-	-	-	-	-	-	-	-	-	-	-	-	-	-	-	-	-	-	-	-	-	-	-	-	-	-	-	-	-	-	-	-	-	-	-	+	-	-	-	-	-	-	-	+	-
3951	6108	hypothetical protein	2	-	-	-	-	-	-	-	-	-	-	-	-	-	-	-	-	-	-	-	-	-	-	-	-	-	-	-	-	-	-	-	-	-	-	-	-	-	-	-	-	-	-	-	-	-	-	-	+	-	-	+	-	-	-	-	-	-
3952	6115	Helicase conserved C-terminal domain protein	2	-	-	-	-	-	-	-	-	-	-	-	-	-	-	-	-	-	-	-	-	-	-	-	-	-	-	-	-	-	-	-	-	-	-	-	-	-	-	-	-	-	-	+	-	-	-	-	+	-	-	-	-	-	-	-	-	-
3953	6121	hypothetical protein	2	-	-	-	-	-	-	-	-	-	-	-	-	-	-	-	-	-	-	-	-	-	-	-	-	-	-	-	-	-	-	-	-	-	-	-	-	-	-	-	-	+	+	-	-	-	-	-	-	-	-	-	-	-	-	-	-	-
3954	6125	Hypothetical protein, CF-38 family	2	-	-	-	-	-	-	-	-	-	-	-	-	-	-	-	-	-	-	-	-	-	-	-	-	-	-	-	-	-	-	-	-	-	-	-	-	-	-	-	-	+	+	-	-	-	-	-	-	-	-	-	-	-	-	-	-	-
3955	6126	Hypothetical protein	2	-	-	-	-	-	-	-	-	-	-	-	-	-	-	-	-	-	-	-	-	-	-	-	-	-	-	-	-	-	-	-	-	-	-	-	-	-	-	-	-	+	+	-	-	-	-	-	-	-	-	-	-	-	-	-	-	-
3956	6128	hypothetical protein	2	-	-	-	-	-	-	-	-	-	-	-	-	-	-	-	-	-	-	-	-	-	-	-	-	-	-	-	-	-	-	-	-	-	-	-	-	-	-	-	-	-	+	-	-	-	-	-	-	-	-	-	-	-	-	-	+	-
3957	6129	Glycosyltransferase	2	-	-	-	-	-	-	-	-	-	-	-	-	-	-	+	-	-	-	-	-	-	-	-	-	-	-	-	-	-	-	-	-	-	-	-	-	-	-	-	-	-	+	-	-	-	-	-	-	-	-	-	-	-	-	-	-	-
3958	6130	hypothetical protein	2	-	-	-	-	-	-	-	-	-	-	-	-	-	-	-	-	-	-	-	-	-	-	-	-	-	-	-	-	-	-	-	-	-	-	-	-	-	-	-	-	+	+	-	-	-	-	-	-	-	-	-	-	-	-	-	-	-
3959	6136	1,3-propanediol dehydrogenase (EC 1.1.1.202)	2	-	-	-	-	-	-	-	-	-	-	-	-	-	-	-	-	-	-	-	-	-	-	-	-	-	-	-	-	-	-	-	-	-	-	-	-	-	-	-	-	-	+	-	-	-	-	-	-	+	-	-	-	-	-	-	-	-
3960	6139	hypothetical protein	2	-	-	-	-	-	-	-	-	-	-	-	-	-	-	-	-	-	-	-	-	-	-	-	-	-	-	-	-	-	-	-	-	-	-	-	-	-	-	-	-	+	+	-	-	-	-	-	-	-	-	-	-	-	-	-	-	-
3961	6140	hypothetical protein	2	-	-	-	-	-	-	-	-	-	-	-	-	-	-	-	-	-	-	-	-	-	-	-	-	-	-	-	-	-	-	-	-	-	-	-	-	-	-	-	-	+	+	-	-	-	-	-	-	-	-	-	-	-	-	-	-	-
3962	6141	extracellular protein	2	-	-	-	-	-	-	-	-	-	-	-	-	-	-	-	-	-	-	-	-	-	-	-	-	-	-	-	-	-	-	-	-	-	-	-	-	-	-	-	-	+	+	-	-	-	-	-	-	-	-	-	-	-	-	-	-	-
3963	6142	hypothetical protein	2	-	-	-	-	-	-	-	-	-	-	-	-	-	-	-	-	-	-	-	-	-	-	-	-	-	-	-	-	-	-	-	-	-	-	-	-	-	-	-	-	+	-	-	-	-	-	-	-	-	-	-	-	+	-	-	-	-
3964	6147	hypothetical protein	2	-	-	-	-	-	-	-	-	-	-	-	-	-	-	-	-	-	-	-	-	-	-	-	-	-	-	-	-	-	-	-	-	-	-	-	-	-	-	-	-	+	-	-	-	-	-	-	-	-	+	-	-	-	-	-	-	-
3965	6149	hypothetical protein	2	-	-	-	-	-	-	-	-	-	-	-	-	-	-	-	-	-	-	-	-	-	-	-	-	-	-	-	-	-	-	-	-	-	-	-	-	-	-	-	-	+	-	-	-	-	-	+	-	-	-	-	-	-	-	-	-	-
3966	6150	hypothetical protein	2	-	-	-	-	-	-	-	-	-	-	-	-	-	-	-	-	-	-	+	-	-	-	-	-	-	-	-	-	-	-	-	-	-	-	-	-	-	-	-	-	+	-	-	-	-	-	-	-	-	-	-	-	-	-	-	-	-
3967	6153	hypothetical protein	2	-	-	-	-	-	-	-	-	-	-	-	-	-	-	-	-	-	-	-	-	-	-	-	-	-	-	-	-	-	-	-	-	-	-	-	-	-	-	-	-	+	-	-	-	-	-	-	-	-	+	-	-	-	-	-	-	-
3968	6155	capsular polysaccharide biosynthesis protein	2	-	-	-	-	-	-	-	-	-	-	-	-	-	-	-	-	-	-	-	-	-	-	-	-	-	-	-	-	-	-	-	-	-	-	-	-	-	-	-	-	+	-	-	-	-	-	-	-	-	-	-	-	+	-	-	-	-
3969	6156	putative cI-like repressor, phage associated	2	-	-	-	-	-	-	-	-	-	-	-	-	-	-	-	-	-	-	-	-	-	-	-	-	-	-	-	-	-	-	-	-	-	-	-	-	-	-	-	-	+	-	-	-	-	-	-	-	-	+	-	-	-	-	-	-	-
3970	6157	Cholinephosphate cytidylyltransferase (EC 2.7.7.15) / Choline kinase (EC 2.7.1.32)	2	-	-	-	-	-	-	-	-	-	-	-	-	-	-	-	-	-	-	-	-	-	-	-	-	-	-	-	-	-	-	-	-	-	-	-	-	-	-	-	-	+	-	-	-	-	-	-	-	-	-	-	-	+	-	-	-	-
3971	6160	hypothetical protein	2	-	-	-	-	-	-	-	-	-	-	-	-	-	-	-	-	-	-	-	-	-	-	-	-	-	-	-	-	-	-	-	-	-	-	-	-	-	-	-	-	-	-	-	-	-	-	+	-	-	+	-	-	-	-	-	-	-
3972	6171	SivA	2	-	-	-	-	-	-	-	-	-	-	-	-	-	-	-	-	-	-	-	-	-	-	-	-	-	-	-	-	-	-	-	-	-	-	-	-	-	-	-	-	-	-	-	-	-	-	-	-	-	+	-	+	-	-	-	-	-
3973	6172	putative cro protein	2	-	-	-	-	+	-	-	-	-	-	-	-	-	-	-	-	-	-	-	-	-	-	-	-	-	-	-	-	-	-	-	-	-	-	-	-	-	-	-	-	-	-	-	-	-	-	-	-	-	+	-	-	-	-	-	-	-
3974	6174	hypothetical protein	2	-	-	-	-	-	-	-	-	-	-	-	-	-	-	-	-	-	-	-	-	-	-	-	-	-	-	-	-	-	-	-	-	-	-	-	-	-	-	-	-	-	-	-	-	-	-	-	-	-	+	-	+	-	-	-	-	-
3975	6176	Uroporphyrinogen-III synthase (EC 4.2.1.75)	2	-	-	-	-	-	-	-	-	-	-	-	-	-	-	-	-	-	-	-	-	-	-	-	-	-	-	-	-	-	-	-	-	-	-	-	-	-	-	-	-	-	-	-	-	-	-	-	-	-	+	-	+	-	-	-	-	-
3976	6181	hypothetical protein	2	-	-	-	-	-	-	-	-	-	-	-	-	-	-	-	-	-	-	-	-	-	-	-	-	-	-	-	-	-	-	-	-	-	-	-	-	-	-	-	-	-	-	-	-	-	-	-	-	-	+	-	+	-	-	-	-	-
3977	6182	hypothetical protein	2	-	-	-	-	-	-	-	-	-	-	-	-	-	-	-	-	-	-	-	-	-	-	-	-	-	-	-	-	-	-	-	-	-	-	-	-	-	-	-	-	-	-	-	-	-	-	-	-	-	+	-	-	+	-	-	-	-
3978	6184	hypothetical protein	2	-	-	-	-	-	-	-	-	-	-	-	-	-	-	-	-	-	-	-	-	-	-	-	-	-	-	-	-	-	-	-	-	-	-	-	-	-	-	-	-	-	-	-	-	-	-	-	-	-	+	-	+	-	-	-	-	-
3979	6186	conserved domain protein	2	-	-	-	-	-	-	-	-	-	-	-	-	-	-	-	-	-	-	-	-	-	-	-	-	-	-	-	-	-	-	-	-	-	-	-	-	-	-	-	-	-	-	-	-	-	-	+	-	-	+	-	-	-	-	-	-	-
3980	6189	hypothetical protein	2	-	-	-	-	-	-	-	-	-	-	-	-	-	-	-	-	-	-	-	-	-	-	-	-	-	-	-	-	-	-	-	-	-	-	-	-	-	-	-	-	-	-	-	-	-	-	-	-	-	+	-	-	-	-	+	-	-
3981	6192	Threonine kinase in B12 biosynthesis	2	-	-	-	-	-	-	-	-	-	-	-	-	-	-	-	-	-	-	-	-	-	-	-	-	-	-	-	-	-	-	-	-	-	-	-	-	-	-	-	-	-	-	-	-	-	-	-	-	-	+	-	+	-	-	-	-	-
3982	6208	hypothetical protein	2	-	-	-	-	-	-	-	-	-	-	-	-	-	-	-	-	-	-	-	-	-	-	-	-	-	-	-	-	-	-	-	-	-	-	-	-	-	-	-	-	-	-	-	-	+	-	+	-	-	-	-	-	-	-	-	-	-
3983	6209	Mobile element protein	2	-	-	-	-	-	-	-	-	-	-	-	-	-	-	-	-	-	-	-	-	-	-	-	-	-	-	-	-	-	-	-	-	-	-	-	-	-	-	-	-	-	-	-	-	+	-	-	-	-	-	-	-	-	+	-	-	-
3984	6210	Epsilon antitoxin	2	-	-	-	-	-	-	-	-	-	-	-	-	-	-	-	-	-	-	-	-	-	-	-	-	-	-	-	-	-	-	-	-	-	-	-	-	-	-	-	-	-	-	-	-	+	-	-	-	-	-	-	-	+	-	-	-	-
3985	6213	hypothetical protein	2	-	-	-	-	-	-	-	-	-	-	-	-	-	-	-	-	-	-	-	-	-	-	-	-	-	-	-	-	-	-	-	-	-	-	-	-	-	-	-	-	-	-	-	+	+	-	-	-	-	-	-	-	-	-	-	-	-
3986	6221	Site-specific recombinase	2	-	-	-	-	-	-	-	-	-	-	-	-	-	-	-	-	-	-	-	-	-	-	-	-	-	-	-	-	-	-	-	-	-	-	-	-	-	-	-	-	-	-	-	-	+	-	-	-	+	-	-	-	-	-	-	-	-
3987	6224	hypothetical protein	2	-	-	-	-	-	-	-	-	-	-	-	-	-	-	-	-	-	-	-	-	-	-	-	-	-	-	-	-	-	-	-	-	-	-	-	-	-	-	-	-	-	-	-	-	-	-	-	-	+	-	+	-	-	-	-	-	-
3988	6226	Oxidoreductase, putative(EC:1.-.-,EC:1.6.5.5)	2	-	-	-	-	-	-	-	-	-	-	-	-	-	-	-	-	-	-	-	-	-	-	-	-	-	-	-	-	-	-	-	-	-	-	-	-	-	-	-	-	-	-	-	-	+	-	-	-	-	-	-	-	-	-	-	+	-
3989	6230	hypothetical protein	2	-	-	-	-	-	-	-	-	-	-	-	-	-	-	-	-	-	-	-	-	-	-	-	-	-	-	-	-	-	-	-	-	-	-	-	-	+	+	-	-	-	-	-	-	-	-	-	-	-	-	-	-	-	-	-	-	-
3990	6234	hypothetical protein	2	-	-	-	-	-	-	-	-	-	-	-	-	-	-	-	-	-	-	-	-	-	-	-	-	-	-	-	-	-	-	-	-	-	-	-	-	+	+	-	-	-	-	-	-	-	-	-	-	-	-	-	-	-	-	-	-	-
3991	6238	capsular polysaccharide biosynthesis protein	2	-	-	-	-	-	-	-	-	-	-	-	-	-	-	-	-	-	-	-	-	-	-	-	-	-	-	-	-	-	-	-	-	-	-	-	-	+	+	-	-	-	-	-	-	-	-	-	-	-	-	-	-	-	-	-	-	-
3992	6241	minor head protein	2	-	-	-	-	-	-	-	-	-	-	-	-	-	-	-	-	-	-	-	-	-	-	-	-	-	-	-	-	-	-	-	-	-	-	-	-	+	+	-	-	-	-	-	-	-	-	-	-	-	-	-	-	-	-	-	-	-
3993	6244	hypothetical protein	2	-	-	-	-	-	-	-	-	-	-	-	-	-	-	-	-	-	-	-	-	-	-	-	-	-	-	-	-	-	-	-	-	-	-	-	-	+	+	-	-	-	-	-	-	-	-	-	-	-	-	-	-	-	-	-	-	-
3994	6247	ClpB protein	2	-	-	-	-	-	-	-	-	-	-	-	-	-	-	-	-	-	-	-	-	-	-	-	-	-	-	-	-	-	-	-	-	-	-	-	-	+	-	+	-	-	-	-	-	-	-	-	-	-	-	-	-	-	-	-	-	-
3995	6248	hypothetical protein	2	-	-	-	-	-	-	-	-	-	-	-	-	-	-	-	-	-	-	-	-	-	-	-	-	-	-	-	-	-	-	-	-	-	-	-	-	+	+	-	-	-	-	-	-	-	-	-	-	-	-	-	-	-	-	-	-	-
3996	6252	hypothetical protein	2	-	-	-	-	-	-	-	-	-	-	-	-	-	-	-	-	-	-	-	-	-	-	-	-	-	-	-	-	-	-	-	-	-	-	-	-	+	+	-	-	-	-	-	-	-	-	-	-	-	-	-	-	-	-	-	-	-
3997	6261	Phage minor tail protein	2	-	-	-	-	-	-	-	-	-	-	-	-	-	-	-	-	-	-	-	-	-	-	-	-	-	-	-	-	-	-	-	-	-	-	-	-	+	+	-	-	-	-	-	-	-	-	-	-	-	-	-	-	-	-	-	-	-
3998	6264	hypothetical protein	2	-	-	-	-	-	-	-	-	-	-	-	-	-	-	-	-	-	-	-	-	-	-	-	-	-	-	-	-	-	-	-	-	-	-	-	-	+	+	-	-	-	-	-	-	-	-	-	-	-	-	-	-	-	-	-	-	-
3999	6265	permease, major facilitator superfamily	2	-	-	-	-	-	-	-	-	-	-	-	-	-	-	-	-	-	-	-	-	-	-	-	-	-	-	-	-	-	-	-	-	-	-	-	-	+	-	+	-	-	-	-	-	-	-	-	-	-	-	-	-	-	-	-	-	-
4000	6267	Modification methylase EcoRI (EC 2.1.1.72)	2	-	-	-	-	-	-	-	-	-	-	-	-	-	-	-	-	-	-	-	-	-	-	-	-	-	-	-	-	-	-	-	-	-	-	-	-	-	-	+	-	-	-	-	-	-	-	-	-	-	-	-	-	-	+	-	-	-
4001	6271	Type II restriction enzyme EcoRI	2	-	-	-	-	-	-	-	-	-	-	-	-	-	-	-	-	-	-	-	-	-	-	-	-	-	-	-	-	-	-	-	-	-	-	-	-	-	-	+	-	-	-	-	-	-	-	-	-	-	-	-	-	-	+	-	-	-
4002	6273	hypothetical protein	2	-	-	-	-	-	-	-	-	-	-	-	-	-	-	-	-	-	-	+	-	-	-	-	-	-	-	-	-	-	-	-	-	-	-	-	-	-	-	+	-	-	-	-	-	-	-	-	-	-	-	-	-	-	-	-	-	-
4003	6277	hypothetical protein	2	-	-	-	-	-	-	-	-	-	-	-	-	-	-	-	-	-	-	-	-	-	-	-	-	-	-	-	-	-	-	-	-	-	-	-	-	-	+	-	-	-	-	-	+	-	-	-	-	-	-	-	-	-	-	-	-	-
4004	6278	hypothetical protein	2	-	-	-	-	-	-	-	-	-	-	-	-	-	-	-	-	-	-	-	-	-	-	-	-	-	-	-	-	-	-	-	-	-	-	-	-	-	+	-	-	-	-	-	-	-	-	-	-	-	-	-	-	+	-	-	-	-
4005	6298	hypothetical protein	2	-	-	-	-	-	-	-	-	-	-	-	-	-	-	-	-	-	-	-	-	-	-	-	-	-	-	-	-	-	-	-	-	-	-	-	+	-	-	-	-	-	-	-	-	-	-	-	-	-	-	-	-	-	+	-	-	-
4006	6334	hypothetical protein	2	-	-	-	-	-	-	-	-	-	-	-	-	-	-	-	-	-	-	-	-	-	-	-	-	-	-	-	-	-	-	-	-	-	-	-	-	-	-	-	-	-	-	-	-	-	-	-	-	-	-	+	-	-	+	-	-	-
4007	6337	hypothetical protein	2	-	-	-	-	-	-	-	-	-	-	-	-	-	-	-	-	-	-	-	-	-	-	-	-	-	-	-	-	-	-	-	-	-	-	-	-	-	-	-	-	-	-	-	-	-	-	-	-	-	-	+	-	-	-	+	-	-
4008	6340	hypothetical protein	2	-	-	-	-	-	-	-	-	-	-	-	-	-	-	-	-	-	-	-	-	-	-	-	-	-	-	-	-	-	-	-	-	-	-	-	-	-	-	-	-	-	-	-	-	-	-	-	-	-	-	-	-	-	+	+	-	-
4009	6347	hypothetical protein	2	-	-	-	-	-	-	-	-	-	-	-	-	-	-	-	-	-	-	-	-	-	-	-	-	-	-	-	-	-	-	-	-	-	-	-	-	-	-	-	-	-	-	-	-	-	-	-	-	-	-	-	-	-	+	+	-	-
4010	6352	phage protein	2	-	-	-	-	-	-	-	-	-	-	-	-	-	-	-	-	-	-	-	-	-	-	-	-	-	-	-	-	-	-	-	-	-	-	-	-	-	-	-	-	-	-	-	-	-	-	-	-	-	-	+	-	-	-	+	-	-
4011	6354	hypothetical protein	2	-	-	-	-	-	-	-	-	-	-	-	-	-	-	-	-	-	-	-	-	-	-	-	-	-	-	-	-	-	-	-	-	-	-	-	-	-	-	-	-	-	-	-	-	-	-	-	-	-	-	+	+	-	-	-	-	-
4012	6358	hypothetical protein	2	-	-	-	-	-	-	-	-	-	-	-	-	-	-	-	-	-	-	-	-	-	-	-	-	-	-	-	-	-	-	-	-	-	-	-	-	-	-	-	-	-	-	-	-	-	-	-	-	-	-	-	-	+	+	-	-	-
4013	6378	hypothetical protein	2	-	-	-	-	-	-	-	-	-	-	-	-	-	-	-	-	-	-	-	-	-	-	-	-	-	-	-	-	+	-	-	-	-	-	-	-	-	-	-	-	-	-	-	-	-	-	-	-	-	-	-	+	-	-	-	-	-
4014	6385	acetyltransferase, GNAT family family	2	-	-	-	-	-	-	-	-	-	-	-	-	-	-	-	-	-	-	-	-	-	-	-	-	-	-	-	-	+	-	-	-	-	-	-	-	-	-	-	-	-	-	-	-	-	-	-	-	-	-	-	+	-	-	-	-	-
4015	6394	ABC-type antimicrobial peptide transport system, permease component	2	-	-	-	-	-	-	-	-	-	-	-	-	-	-	-	-	-	-	-	-	-	-	-	-	-	-	-	-	-	-	-	-	-	-	-	-	-	-	-	-	-	-	-	-	-	-	-	-	-	-	-	+	+	-	-	-	-
4016	6396	Lj965 prophage protein	2	-	-	-	-	-	-	-	-	-	-	-	-	-	-	-	-	-	-	-	-	-	-	-	-	-	-	-	-	-	-	-	-	-	-	-	-	-	-	-	-	-	-	-	-	-	-	-	-	-	-	+	+	-	-	-	-	-
4017	6399	hypothetical protein	2	-	-	-	-	-	-	-	-	-	-	-	-	-	-	-	-	-	-	-	-	-	-	-	-	-	-	-	-	-	-	-	-	-	-	-	-	-	-	-	-	-	-	-	-	-	-	-	-	-	-	-	+	+	-	-	-	-
4018	6400	hypothetical protein	2	-	-	-	-	-	-	-	-	-	-	-	-	-	-	-	-	-	-	-	-	-	-	-	-	-	-	-	-	-	-	-	-	-	-	-	-	-	-	-	-	-	-	-	-	-	-	-	-	-	-	+	+	-	-	-	-	-
4019	6401	hypothetical protein	2	-	-	-	-	-	-	-	-	-	-	-	-	-	-	-	-	-	-	-	-	-	-	-	-	-	-	-	-	-	-	-	-	-	-	-	-	-	-	-	-	-	-	-	-	-	-	-	-	-	-	-	+	+	-	-	-	-
4020	6403	transcription regulator, probable	2	-	-	-	-	-	-	-	-	-	-	-	-	-	-	-	-	-	-	-	-	-	-	-	-	-	-	-	-	-	-	-	-	-	-	-	-	-	-	-	-	-	-	-	-	-	-	+	-	+	-	-	-	-	-	-	-	-
4021	6413	hypothetical protein	2	-	-	-	-	+	-	-	-	-	-	-	-	-	-	-	-	-	-	-	-	-	-	-	-	-	-	-	-	-	-	-	-	-	-	-	-	-	-	-	-	-	-	-	+	-	-	-	-	-	-	-	-	-	-	-	-	-
4022	6414	hypothetical protein	2	-	-	-	-	+	-	-	-	-	-	-	-	-	-	-	-	-	-	-	-	-	-	-	-	-	-	-	-	-	-	-	-	-	-	-	-	-	-	-	-	-	-	-	+	-	-	-	-	-	-	-	-	-	-	-	-	-
4023	6415	hypothetical protein	2	-	-	-	-	+	-	-	-	-	-	-	-	-	-	-	-	-	-	-	-	-	-	-	-	-	-	-	-	-	-	-	-	-	-	-	-	-	-	-	-	-	-	-	+	-	-	-	-	-	-	-	-	-	-	-	-	-
4024	6419	hypothetical protein	2	-	-	-	-	+	-	-	-	-	-	-	-	-	-	-	-	-	-	-	-	-	-	-	-	-	-	-	-	-	-	-	-	-	-	-	-	-	-	-	-	-	-	-	+	-	-	-	-	-	-	-	-	-	-	-	-	-
4025	6422	hypothetical protein	2	-	-	-	-	+	-	-	-	-	-	-	-	-	-	-	-	-	-	-	-	-	-	-	-	-	-	-	-	-	-	-	-	-	-	-	-	-	-	-	-	-	-	-	+	-	-	-	-	-	-	-	-	-	-	-	-	-
4026	6423	hypothetical protein	2	-	-	-	-	+	-	-	-	-	-	-	-	-	-	-	-	-	-	-	-	-	-	-	-	-	-	-	-	-	-	-	-	-	-	-	-	-	-	-	-	-	-	-	+	-	-	-	-	-	-	-	-	-	-	-	-	-
4027	6425	Rhamnulose-1-phosphate aldolase (EC 4.1.2.19)	2	-	-	-	-	+	-	-	-	-	-	-	-	-	-	-	-	-	-	-	-	-	-	-	-	-	-	-	-	-	-	-	-	-	-	-	-	-	-	-	-	-	-	-	+	-	-	-	-	-	-	-	-	-	-	-	-	-
4028	6426	glycosyl transferase, family 2	2	-	-	-	-	+	-	-	-	-	-	-	-	-	-	-	-	-	-	-	-	-	-	-	-	-	-	-	-	-	-	-	-	-	-	-	-	-	-	-	-	-	-	-	+	-	-	-	-	-	-	-	-	-	-	-	-	-
4029	6427	hypothetical protein	2	-	-	+	-	-	-	-	-	-	-	-	-	-	-	-	-	-	-	-	-	-	-	-	-	-	-	-	-	-	-	-	-	-	-	-	-	-	-	-	-	-	-	-	-	-	-	-	-	-	-	-	-	+	-	-	-	-
4030	6429	ABC-type multidrug transport system	2	-	-	-	-	+	-	-	-	-	-	-	-	-	-	-	-	-	-	-	-	-	-	-	-	-	-	-	-	-	-	-	-	-	-	-	-	-	-	-	-	-	-	-	+	-	-	-	-	-	-	-	-	-	-	-	-	-
4031	6432	Phosphoenolpyruvate synthase (EC 2.7.9.2)	2	-	-	-	-	+	-	-	-	-	-	-	-	-	-	-	-	-	-	-	-	-	-	-	-	-	-	-	-	-	-	-	-	-	-	-	-	-	-	-	-	-	-	-	+	-	-	-	-	-	-	-	-	-	-	-	-	-
4032	6434	hypothetical protein	2	-	-	-	-	+	-	-	-	-	-	-	-	-	-	-	-	-	-	-	-	-	-	-	-	-	-	-	-	-	-	-	-	-	-	-	-	-	-	-	-	-	-	-	+	-	-	-	-	-	-	-	-	-	-	-	-	-
4033	6436	Rhamnulose-1-phosphate aldolase (EC 4.1.2.19)	2	-	-	-	-	+	-	-	-	-	-	-	-	-	-	-	-	-	-	-	-	-	-	-	-	-	-	-	-	-	-	-	-	-	-	-	-	-	-	-	-	-	-	-	+	-	-	-	-	-	-	-	-	-	-	-	-	-
4034	6437	hypothetical protein	2	-	-	-	-	-	-	-	-	-	-	-	-	-	-	-	-	-	-	-	-	-	-	-	-	-	-	-	-	-	-	-	-	-	-	-	-	-	-	-	-	-	-	-	-	-	-	-	-	+	-	+	-	-	-	-	-	-
4035	6438	Phage portal	2	-	-	-	-	-	-	-	-	-	-	-	-	-	-	-	-	-	-	-	-	-	-	-	-	-	-	-	-	-	-	-	-	-	-	-	-	-	-	-	-	-	-	-	-	-	-	-	-	-	-	+	-	+	-	-	-	-
4036	6439	Cell wall surface anchor family protein, LPXTG motif	2	-	-	-	-	-	-	-	-	-	-	-	-	-	-	-	-	-	-	-	-	-	-	-	-	-	-	-	-	-	-	-	-	-	-	-	-	-	-	-	-	-	-	-	-	-	-	-	-	-	-	+	-	+	-	-	-	-
4037	6440	hypothetical protein	2	-	-	-	-	-	-	-	-	-	-	-	-	-	-	-	-	-	-	-	-	-	-	-	-	-	-	-	-	-	-	-	-	-	-	-	-	-	-	-	-	-	-	-	-	-	-	-	-	-	-	+	-	+	-	-	-	-
4038	6447	hypothetical protein	2	-	-	-	-	-	-	-	-	-	-	-	-	-	-	-	-	-	+	-	-	-	-	-	-	-	-	-	-	-	-	-	-	-	-	-	-	-	-	-	-	-	-	-	+	-	-	-	-	-	-	-	-	-	-	-	-	-
4039	6449	Phage terminase, large subunit	2	-	-	-	-	-	-	-	-	-	-	-	-	-	-	-	-	-	-	+	-	-	-	-	-	-	-	-	-	-	-	-	-	-	-	-	-	-	-	-	-	-	-	-	-	-	-	-	-	-	-	-	-	-	+	-	-	-
4040	6452	hypothetical protein	2	-	-	-	-	-	-	-	-	-	-	-	-	-	-	-	-	-	-	+	-	-	-	-	-	-	-	-	-	-	-	-	-	-	-	-	-	-	-	-	-	-	-	+	-	-	-	-	-	-	-	-	-	-	-	-	-	-
4041	6501	hypothetical protein	2	-	-	-	-	-	-	-	-	-	-	-	-	-	-	-	-	-	-	-	-	-	-	-	-	-	-	-	-	-	-	+	+	-	-	-	-	-	-	-	-	-	-	-	-	-	-	-	-	-	-	-	-	-	-	-	-	-
4042	6509	hypothetical protein	2	-	-	-	-	-	-	-	-	-	-	-	-	-	-	-	-	-	-	-	-	-	-	-	-	-	-	-	-	-	-	+	+	-	-	-	-	-	-	-	-	-	-	-	-	-	-	-	-	-	-	-	-	-	-	-	-	-
4043	6537	hypothetical protein	2	-	-	-	-	-	-	-	-	-	-	-	-	-	-	-	-	-	-	-	-	-	-	-	-	-	-	-	-	+	+	-	-	-	-	-	-	-	-	-	-	-	-	-	-	-	-	-	-	-	-	-	-	-	-	-	-	-
4044	6561	Helix-turn-helix domain protein	2	-	-	-	-	-	-	-	-	-	-	-	-	-	-	-	-	-	-	-	-	-	-	-	-	-	-	-	-	-	-	+	+	-	-	-	-	-	-	-	-	-	-	-	-	-	-	-	-	-	-	-	-	-	-	-	-	-
4045	6563	serine/threonine-protein kinase	2	-	-	-	-	-	-	-	-	-	-	-	-	-	-	-	-	-	-	-	-	-	-	-	-	-	-	-	-	-	-	+	+	-	-	-	-	-	-	-	-	-	-	-	-	-	-	-	-	-	-	-	-	-	-	-	-	-
4046	6564	Transporter	2	-	-	-	-	-	-	-	-	-	-	-	-	-	-	-	-	-	-	-	-	-	-	-	-	-	-	-	-	-	-	+	+	-	-	-	-	-	-	-	-	-	-	-	-	-	-	-	-	-	-	-	-	-	-	-	-	-
4047	7336	hypothetical protein	2	-	-	-	-	-	-	-	-	-	-	-	-	-	-	-	-	-	-	-	-	-	-	-	+	-	+	-	-	-	-	-	-	-	-	-	-	-	-	-	-	-	-	-	-	-	-	-	-	-	-	-	-	-	-	-	-	-
4048	7337	Aspartate aminotransferase (EC 2.6.1.1)	2	+	+	-	-	-	-	-	-	-	-	-	-	-	-	-	-	-	-	-	-	-	-	-	-	-	-	-	-	-	-	-	-	-	-	-	-	-	-	-	-	-	-	-	-	-	-	-	-	-	-	-	-	-	-	-	-	-
4049	7342	hypothetical protein	2	-	-	-	-	-	-	-	-	-	-	-	-	-	-	-	-	+	+	-	-	-	-	-	-	-	-	-	-	-	-	-	-	-	-	-	-	-	-	-	-	-	-	-	-	-	-	-	-	-	-	-	-	-	-	-	-	-
4050	7343	hypothetical protein	2	-	-	-	-	-	-	-	-	-	-	-	-	-	-	-	-	+	+	-	-	-	-	-	-	-	-	-	-	-	-	-	-	-	-	-	-	-	-	-	-	-	-	-	-	-	-	-	-	-	-	-	-	-	-	-	-	-
4051	7344	hypothetical protein	2	-	-	-	-	-	-	-	-	-	-	-	-	-	-	-	+	+	-	-	-	-	-	-	-	-	-	-	-	-	-	-	-	-	-	-	-	-	-	-	-	-	-	-	-	-	-	-	-	-	-	-	-	-	-	-	-	-
4052	7345	hypothetical protein	2	-	-	-	-	-	-	-	-	-	-	-	-	-	-	-	-	+	-	-	-	-	-	-	-	-	-	-	-	-	-	-	-	-	-	-	-	-	-	-	+	-	-	-	-	-	-	-	-	-	-	-	-	-	-	-	-	-
4053	7346	hypothetical protein	2	-	-	-	-	-	-	-	-	-	-	-	-	-	-	-	-	+	-	-	-	-	-	-	-	-	-	-	-	-	-	-	-	-	-	-	-	-	-	-	-	-	-	-	+	-	-	-	-	-	-	-	-	-	-	-	-	-
4054	7347	hypothetical protein	2	-	-	-	-	-	-	-	-	-	-	-	-	-	-	-	-	+	+	-	-	-	-	-	-	-	-	-	-	-	-	-	-	-	-	-	-	-	-	-	-	-	-	-	-	-	-	-	-	-	-	-	-	-	-	-	-	-
4055	7348	hypothetical protein	2	-	-	-	-	-	-	-	-	-	-	-	-	-	-	-	-	+	+	-	-	-	-	-	-	-	-	-	-	-	-	-	-	-	-	-	-	-	-	-	-	-	-	-	-	-	-	-	-	-	-	-	-	-	-	-	-	-
4056	7349	hypothetical protein	2	-	-	-	-	-	-	-	-	-	-	-	-	-	-	-	-	+	-	-	-	-	-	-	-	-	-	-	-	-	-	-	-	-	-	-	-	-	-	-	+	-	-	-	-	-	-	-	-	-	-	-	-	-	-	-	-	-
4057	7350	hypothetical protein	2	-	-	-	-	-	-	-	-	-	-	-	-	-	-	-	-	+	+	-	-	-	-	-	-	-	-	-	-	-	-	-	-	-	-	-	-	-	-	-	-	-	-	-	-	-	-	-	-	-	-	-	-	-	-	-	-	-
4058	7351	hypothetical protein	2	-	-	-	-	-	-	-	-	-	-	-	-	-	-	-	-	+	-	-	-	-	-	-	-	-	-	-	-	-	-	-	-	-	-	-	-	-	-	-	-	-	-	-	-	+	-	-	-	-	-	-	-	-	-	-	-	-
4059	7352	hypothetical protein	2	-	-	-	-	-	-	-	-	-	-	-	-	-	-	-	-	+	+	-	-	-	-	-	-	-	-	-	-	-	-	-	-	-	-	-	-	-	-	-	-	-	-	-	-	-	-	-	-	-	-	-	-	-	-	-	-	-
4060	7353	hypothetical protein	2	-	-	-	-	-	-	-	-	-	-	-	-	-	-	-	-	+	-	+	-	-	-	-	-	-	-	-	-	-	-	-	-	-	-	-	-	-	-	-	-	-	-	-	-	-	-	-	-	-	-	-	-	-	-	-	-	-
4061	7355	Predicted by GeneMark	2	-	-	-	-	-	-	-	-	-	-	-	-	-	-	-	-	+	-	-	-	-	-	+	-	-	-	-	-	-	-	-	-	-	-	-	-	-	-	-	-	-	-	-	-	-	-	-	-	-	-	-	-	-	-	-	-	-
4062	7356	hypothetical protein	2	-	-	-	-	-	-	-	-	-	-	-	-	-	-	-	-	+	+	-	-	-	-	-	-	-	-	-	-	-	-	-	-	-	-	-	-	-	-	-	-	-	-	-	-	-	-	-	-	-	-	-	-	-	-	-	-	-
4063	7357	hypothetical protein	2	-	-	-	-	-	-	-	-	-	-	-	-	-	-	-	-	+	+	-	-	-	-	-	-	-	-	-	-	-	-	-	-	-	-	-	-	-	-	-	-	-	-	-	-	-	-	-	-	-	-	-	-	-	-	-	-	-
4064	7358	hypothetical protein	2	-	-	-	-	-	-	-	-	-	-	-	-	-	-	-	-	+	+	-	-	-	-	-	-	-	-	-	-	-	-	-	-	-	-	-	-	-	-	-	-	-	-	-	-	-	-	-	-	-	-	-	-	-	-	-	-	-
4065	7359	Phage major capsid protein	2	-	-	-	-	-	-	-	-	-	-	-	-	-	-	-	-	+	+	-	-	-	-	-	-	-	-	-	-	-	-	-	-	-	-	-	-	-	-	-	-	-	-	-	-	-	-	-	-	-	-	-	-	-	-	-	-	-
4066	7360	hypothetical protein	2	-	-	-	-	-	-	-	-	-	-	-	-	-	-	-	-	+	-	-	-	-	-	-	-	-	-	-	-	-	-	-	-	-	-	-	-	-	-	+	-	-	-	-	-	-	-	-	-	-	-	-	-	-	-	-	-	-
4067	7361	Phage tail assembly	2	-	-	-	-	-	-	-	-	-	-	-	-	-	-	-	-	+	+	-	-	-	-	-	-	-	-	-	-	-	-	-	-	-	-	-	-	-	-	-	-	-	-	-	-	-	-	-	-	-	-	-	-	-	-	-	-	-
4068	7362	hypothetical protein	2	-	-	-	-	-	-	-	-	-	-	-	-	-	-	-	-	-	-	-	-	-	-	-	-	-	-	-	-	-	-	-	-	-	-	-	-	-	-	-	-	-	-	-	-	-	-	+	-	-	+	-	-	-	-	-	-	-
4069	7364	Late competence protein ComEA, DNA receptor	2	-	-	-	-	-	-	-	-	-	-	-	-	-	-	-	-	+	+	-	-	-	-	-	-	-	-	-	-	-	-	-	-	-	-	-	-	-	-	-	-	-	-	-	-	-	-	-	-	-	-	-	-	-	-	-	-	-
4070	7365	hypothetical protein	2	-	-	-	-	-	-	-	-	-	-	-	-	-	-	-	-	+	+	-	-	-	-	-	-	-	-	-	-	-	-	-	-	-	-	-	-	-	-	-	-	-	-	-	-	-	-	-	-	-	-	-	-	-	-	-	-	-
4071	7366	hypothetical protein	2	-	-	-	-	-	-	-	-	-	-	-	-	-	-	-	-	+	+	-	-	-	-	-	-	-	-	-	-	-	-	-	-	-	-	-	-	-	-	-	-	-	-	-	-	-	-	-	-	-	-	-	-	-	-	-	-	-
4072	7367	prophage LambdaSa2	2	-	-	-	-	-	-	-	-	-	-	-	-	-	-	-	+	+	-	-	-	-	-	-	-	-	-	-	-	-	-	-	-	-	-	-	-	-	-	-	-	-	-	-	-	-	-	-	-	-	-	-	-	-	-	-	-	-
4073	7368	hypothetical protein	2	-	-	-	-	-	-	-	-	-	-	-	-	-	-	-	-	+	+	-	-	-	-	-	-	-	-	-	-	-	-	-	-	-	-	-	-	-	-	-	-	-	-	-	-	-	-	-	-	-	-	-	-	-	-	-	-	-
4074	7369	hypothetical protein	2	-	-	-	-	-	-	-	-	-	-	-	-	-	-	-	-	+	+	-	-	-	-	-	-	-	-	-	-	-	-	-	-	-	-	-	-	-	-	-	-	-	-	-	-	-	-	-	-	-	-	-	-	-	-	-	-	-
4075	7370	hypothetical protein	2	-	-	-	-	-	-	-	-	-	-	-	-	-	-	-	-	-	-	-	-	-	-	-	-	+	+	-	-	-	-	-	-	-	-	-	-	-	-	-	-	-	-	-	-	-	-	-	-	-	-	-	-	-	-	-	-	-
4076	7371	hypothetical protein	2	-	-	-	-	-	-	-	-	-	-	-	-	-	-	-	-	-	-	-	-	-	-	-	-	+	+	-	-	-	-	-	-	-	-	-	-	-	-	-	-	-	-	-	-	-	-	-	-	-	-	-	-	-	-	-	-	-
4077	7372	O-Antigen Polymerase family	2	-	-	-	-	-	-	-	-	-	-	-	-	-	-	-	-	-	-	-	-	-	-	-	-	+	+	-	-	-	-	-	-	-	-	-	-	-	-	-	-	-	-	-	-	-	-	-	-	-	-	-	-	-	-	-	-	-
4078	7373	hypothetical protein	2	-	-	-	-	-	-	-	-	-	-	-	-	-	-	-	-	-	-	-	-	-	-	-	-	+	+	-	-	-	-	-	-	-	-	-	-	-	-	-	-	-	-	-	-	-	-	-	-	-	-	-	-	-	-	-	-	-
4079	7374	Putative secretion accessory protein EsaA/YueB	2	-	-	-	-	-	-	-	-	-	-	-	-	-	-	-	-	-	-	-	-	-	-	-	-	+	+	-	-	-	-	-	-	-	-	-	-	-	-	-	-	-	-	-	-	-	-	-	-	-	-	-	-	-	-	-	-	-
4080	7375	hypothetical protein	2	-	-	-	-	-	-	-	-	-	-	-	-	-	-	-	-	-	-	-	-	-	-	-	-	+	-	-	-	-	-	-	-	+	-	-	-	-	-	-	-	-	-	-	-	-	-	-	-	-	-	-	-	-	-	-	-	-
4081	7376	hypothetical protein	2	-	-	-	-	-	-	-	-	-	-	-	-	-	-	-	-	-	-	-	-	-	-	-	-	+	-	+	-	-	-	-	-	-	-	-	-	-	-	-	-	-	-	-	-	-	-	-	-	-	-	-	-	-	-	-	-	-
4082	7377	hypothetical protein	2	-	-	-	-	-	-	-	-	-	-	-	-	-	-	-	-	-	-	-	-	-	-	-	-	+	-	-	-	-	-	-	-	-	+	-	-	-	-	-	-	-	-	-	-	-	-	-	-	-	-	-	-	-	-	-	-	-
4083	7378	glycosyl transferase, family 2	2	-	-	-	-	-	-	-	-	-	-	-	-	-	-	-	-	-	-	-	-	-	-	-	-	+	+	-	-	-	-	-	-	-	-	-	-	-	-	-	-	-	-	-	-	-	-	-	-	-	-	-	-	-	-	-	-	-
4084	7379	Putative coproporphyrinogen III oxidase of BS HemN-type	2	-	-	-	-	-	-	-	-	-	-	-	-	-	-	-	-	-	-	-	-	-	-	-	-	+	-	-	-	-	-	-	-	-	-	-	-	+	-	-	-	-	-	-	-	-	-	-	-	-	-	-	-	-	-	-	-	-
4085	7380	hypothetical protein	2	-	-	-	-	-	-	-	-	-	-	-	-	-	-	-	-	-	-	-	-	-	-	-	-	+	+	-	-	-	-	-	-	-	-	-	-	-	-	-	-	-	-	-	-	-	-	-	-	-	-	-	-	-	-	-	-	-
4086	7382	hypothetical protein	2	-	-	-	-	-	-	-	-	-	-	-	-	-	-	-	-	-	-	-	-	-	-	-	-	+	-	-	-	-	-	-	-	-	-	-	-	-	-	-	-	-	-	+	-	-	-	-	-	-	-	-	-	-	-	-	-	-
4087	7383	ABC transporter	2	-	-	-	-	-	-	-	-	-	-	-	-	-	-	-	-	-	-	-	-	-	-	-	-	-	-	-	-	-	-	-	-	-	-	-	-	-	-	+	+	-	-	-	-	-	-	-	-	-	-	-	-	-	-	-	-	-
4088	7385	hypothetical protein	2	-	-	-	-	-	-	-	-	-	-	-	-	-	-	-	-	-	-	-	-	-	-	-	-	-	-	-	-	-	-	-	-	-	-	-	-	-	-	+	-	-	+	-	-	-	-	-	-	-	-	-	-	-	-	-	-	-
4089	7386	hypothetical protein	2	-	-	-	-	-	-	-	-	-	-	-	-	-	-	+	-	-	-	-	-	-	-	-	-	-	-	-	-	-	-	-	-	-	-	-	-	-	-	+	-	-	-	-	-	-	-	-	-	-	-	-	-	-	-	-	-	-

Table S5. Precence/absence of each HG among 57 *S. suis* strains

				1																13			25					28							104		94			76			53/54		Divergent *S. suis*																
Index[a]	ClusterID	Gene products	Members	98HAH33	05ZYH33	SC84	A7	SS12	P1/7	DAT273	NIAH11434	JS14	GZ1	BM407	S735	13730	MNCM07	NCTC 10446	14636	NCTC 10237	ST1	2651	89-1591	MNCM04	MNCM25	8074	D9	4961	ST3	MNCM43	DAT292	DAT260	NIAH11435	MNCM21	MNCM50	89-2479	DAT234	DAT290	93A	42A	NT77	6407	11538	8830	12814	92-1400	89-590	2726	2524	89-3576-3	4417	D12	10581	89-5259	88-5299A	R61	92-1191	14A	92-4172	22083
4090	7387	Integrase/recombinase	2	-	-	-	-	-	-	-	-	-	-	-	-	-	-	-	-	-	-	-	-	-	-	-	-	-	-	-	-	-	-	-	-	-	-	+	+	-	-	-	-	-	-	-	-	-	-	-	-	-	-	-	-	-
4091	7388	hypothetical protein	2	-	-	-	-	-	-	-	-	-	-	-	-	-	-	-	-	-	-	-	-	-	-	-	-	-	-	-	-	-	-	-	-	-	-	+	+	-	-	-	-	-	-	-	-	-	-	-	-	-	-	-	-	-
4092	7389	Paratox	2	-	-	-	-	-	-	-	-	-	-	-	-	-	-	-	-	-	-	-	-	-	-	+	-	-	-	-	-	-	-	-	-	-	-	-	-	-	-	-	-	-	-	+	-	-	-	-	-	-	-	-	-	-	-	-	-	-	-	-	-	-
4093	7390	Glycosyltransferase	2	-	-	-	-	-	-	-	-	-	-	-	-	-	-	-	-	-	-	-	-	-	-	-	-	-	-	-	-	-	-	+	-	-	-	+	-	-	-	-	-	-	-	-	-	-	-	-	-	-	-	-	-	-	-	-
4094	7391	hypothetical protein	2	-	-	-	-	-	-	-	-	-	-	-	-	-	-	-	-	-	-	-	-	-	-	-	-	-	-	-	-	-	-	-	-	-	-	+	+	-	-	-	-	-	-	-	-	-	-	-	-	-	-	-	-	-
4095	7392	hypothetical protein	2	-	-	-	-	-	-	-	-	-	-	-	-	-	-	-	-	-	-	-	-	-	-	-	-	-	-	-	-	-	-	-	-	-	-	+	+	-	-	-	-	-	-	-	-	-	-	-	-	-	-	-	-	-
4096	7393	hypothetical protein	2	-	-	-	-	-	-	-	-	-	-	-	-	-	-	-	-	-	-	-	-	-	-	-	-	-	-	-	-	-	-	-	-	-	-	+	-	-	-	-	-	-	-	+	-	-	-	-	-	-	-	-	-	-
4097	7395	hypothetical protein	2	-	-	-	-	-	-	-	-	-	-	-	-	-	-	-	-	-	-	-	-	-	-	-	-	-	-	-	-	-	-	-	-	-	-	+	-	-	-	-	-	-	-	-	-	-	-	+	-	-	-	-	-	-	-
4098	7396	extracellular factor protein	2	-	-	-	-	-	-	-	-	-	-	-	-	-	-	-	-	-	-	-	-	-	-	-	-	-	-	-	-	-	-	-	-	-	-	+	+	-	-	-	-	-	-	-	-	-	-	-	-	-	-	-	-	-
4099	7397	hypothetical protein	2	-	-	-	-	-	-	-	-	-	-	-	-	-	-	+	-	-	-	-	-	-	-	-	-	-	-	-	-	-	-	-	-	-	-	+	-	-	-	-	-	-	-	-	-	-	-	-	-	-	-	-	-	-
4100	7398	exopolysaccharide biosynthesis protein	2	-	-	-	-	-	-	-	-	-	-	-	-	-	-	-	-	-	-	-	-	-	-	-	-	-	-	-	-	-	-	+	-	-	-	+	-	-	-	-	-	-	-	-	-	-	-	-	-	-	-	-	-	-
4101	7399	hypothetical protein	2	-	-	-	-	-	-	-	-	-	-	-	-	-	-	-	-	-	-	-	-	-	-	-	-	-	-	-	-	-	-	-	-	-	-	+	-	-	-	-	-	-	-	-	-	-	-	+	-	-	-	-	-	-	-
4102	7401	hypothetical protein	2	-	-	-	-	-	-	-	-	-	-	-	-	-	-	-	-	-	-	-	-	-	-	-	-	-	-	-	-	-	-	-	-	-	-	+	-	-	-	-	-	-	-	-	-	-	-	-	+	-	-	-	-	-	-
4103	7402	hypothetical protein	2	-	-	-	-	-	-	-	-	-	-	-	-	-	-	-	-	-	-	-	-	-	-	-	-	-	-	-	-	-	-	-	-	-	-	+	+	-	-	-	-	-	-	-	-	-	-	-	-	-	-	-	-	-
4104	7403	Phage tail assembly	2	-	-	-	-	-	-	-	-	-	-	-	-	-	-	-	-	-	-	-	-	-	-	-	-	-	-	-	-	-	-	+	-	-	-	+	-	-	-	-	-	-	-	-	-	-	-	-	-	-	-	-	-	-
4105	7404	Membrane protein involved in the export of O-antigen	2	-	-	-	-	-	-	-	-	-	-	-	-	-	-	-	-	-	-	-	-	-	-	-	-	-	-	-	-	-	-	+	-	-	-	+	-	-	-	-	-	-	-	-	-	-	-	-	-	-	-	-	-	-
4106	7405	hypothetical protein	2	-	-	-	-	-	-	-	-	-	-	-	-	-	-	-	-	-	-	-	-	-	-	-	-	-	-	-	-	-	-	-	-	-	-	+	+	-	-	-	-	-	-	-	-	-	-	-	-	-	-	-	-	-
4107	7406	hypothetical protein	2	-	-	-	-	-	-	-	-	-	-	-	-	-	-	-	-	-	-	-	-	-	-	-	-	-	-	-	-	-	-	-	-	-	-	+	+	-	-	-	-	-	-	-	-	-	-	-	-	-	-	-	-	-
4108	7407	hypothetical protein	2	-	-	-	-	-	-	-	-	-	-	-	-	-	-	-	-	-	-	-	-	-	-	-	-	-	-	-	-	-	-	-	-	-	-	+	+	-	-	-	-	-	-	-	-	-	-	-	-	-	-	-	-	-
4109	7408	Mobile element protein	2	-	-	-	-	-	-	-	-	-	-	-	-	-	-	-	-	-	-	-	-	-	-	-	-	-	-	-	-	-	-	-	-	-	-	+	+	-	-	-	-	-	-	-	-	-	-	-	-	-	-	-	-	-
4110	7409	hypothetical protein	2	-	-	-	-	-	-	-	-	-	-	-	-	-	-	-	-	-	-	-	-	-	-	-	-	-	-	-	-	-	-	-	-	-	-	+	-	-	+	-	-	-	-	-	-	-	-	-	-	-	-	-	-	-
4111	7410	3-isopropylmalate dehydratase large subunit (EC 4.2.1.33)	2	-	-	-	-	-	-	-	-	-	-	-	-	-	-	-	-	-	-	-	-	-	-	-	-	-	-	-	-	-	-	-	-	-	-	+	+	-	-	-	-	-	-	-	-	-	-	-	-	-	-	-	-	-
4112	7411	hypothetical protein	2	-	-	-	-	-	-	-	-	-	-	-	-	-	-	-	-	-	-	-	-	-	-	-	-	-	-	-	-	-	-	-	-	-	-	+	+	-	-	-	-	-	-	-	-	-	-	-	-	-	-	-	-	-
4113	7412	hypothetical protein	2	-	-	-	-	-	-	-	-	-	-	-	-	-	-	-	-	-	-	-	-	-	-	-	-	-	-	-	-	-	-	+	-	-	-	+	-	-	-	-	-	-	-	-	-	-	-	-	-	-	-	-	-	-
4114	7413	phage protein	2	-	-	-	-	-	-	-	-	-	-	-	-	-	-	-	-	-	-	-	-	-	-	-	-	-	-	-	-	-	-	-	-	-	-	+	+	-	-	-	-	-	-	-	-	-	-	-	-	-	-	-	-	-
4115	7416	hypothetical protein	2	-	-	-	-	-	-	-	-	-	-	-	-	-	-	-	-	-	-	-	-	-	-	-	-	-	-	-	-	-	-	-	-	-	-	+	-	-	-	-	-	-	-	-	-	-	-	-	+	-	-	-	-	-	-
4116	7417	hypothetical protein	2	-	-	-	-	-	-	-	-	-	-	-	-	-	-	-	-	-	-	-	-	-	-	-	-	-	-	-	-	-	-	-	-	-	-	+	-	-	-	-	-	-	-	+	-	-	-	-	-	-	-	-	-	-
4117	7418	hypothetical protein	2	-	-	-	-	-	-	-	-	-	-	-	-	-	-	-	-	-	-	-	-	-	-	-	-	-	-	-	-	-	-	-	-	-	-	+	+	-	-	-	-	-	-	-	-	-	-	-	-	-	-	-	-	-
4118	7419	Helicase loader DnaI	2	-	-	-	-	-	-	-	-	-	-	-	-	-	-	-	-	-	-	-	-	-	-	-	-	-	-	-	-	-	-	-	-	-	-	+	+	-	-	-	-	-	-	-	-	-	-	-	-	-	-	-	-	-
4119	7420	hypothetical protein	2	-	-	-	-	-	-	-	-	-	-	-	-	-	-	-	-	-	-	-	-	-	-	-	-	-	-	-	-	-	-	-	-	-	-	+	+	-	-	-	-	-	-	-	-	-	-	-	-	-	-	-	-	-
4120	7421	hypothetical protein	2	-	-	-	-	-	-	-	-	-	-	-	-	-	-	-	-	-	-	-	-	-	-	-	-	-	-	-	-	-	-	-	-	-	-	+	+	-	-	-	-	-	-	-	-	-	-	-	-	-	-	-	-	-
4121	7422	hypothetical protein	2	-	-	-	-	-	-	-	-	-	-	-	-	-	-	-	-	-	-	-	-	-	-	-	-	-	-	-	-	-	-	-	-	-	-	+	+	-	-	-	-	-	-	-	-	-	-	-	-	-	-	-	-	-
4122	7423	Site-specific recombinase	2	-	-	-	-	-	-	-	-	-	-	-	-	-	-	-	-	-	-	-	-	-	-	-	-	-	-	-	-	-	-	-	-	-	-	+	+	-	-	-	-	-	-	-	-	-	-	-	-	-	-	-	-	-
4123	7424	hypothetical protein	2	-	-	-	-	-	-	-	-	-	-	-	-	-	-	-	-	-	-	-	-	-	-	-	-	-	-	-	-	-	-	-	-	-	-	+	-	-	-	-	-	-	-	-	-	-	-	-	-	-	-	+	-	-	-
4124	7425	hypothetical protein	2	-	-	-	-	-	-	-	-	-	-	-	-	-	-	-	-	-	-	-	-	-	-	-	-	-	-	-	-	-	-	-	-	-	-	+	+	-	-	-	-	-	-	-	-	-	-	-	-	-	-	-	-	-
4125	7426	hypothetical protein	2	-	-	-	-	-	-	-	-	-	-	-	-	-	-	-	-	-	-	-	-	-	-	-	-	-	-	-	-	-	-	+	-	-	-	+	-	-	-	-	-	-	-	-	-	-	-	-	-	-	-	-	-	-
4126	7428	hypothetical protein	2	-	-	-	-	-	-	-	-	-	-	-	-	-	-	-	-	-	-	-	-	-	-	-	-	-	-	-	-	-	-	-	-	-	-	+	+	-	-	-	-	-	-	-	-	-	-	-	-	-	-	-	-	-
4127	7429	conserved domain protein	2	-	-	-	-	-	-	-	-	-	-	-	-	-	-	-	-	-	-	-	-	-	-	-	-	-	-	-	-	-	-	-	-	-	-	+	-	-	-	-	-	-	-	-	-	-	-	+	-	-	-	-	-	-	-
4128	7430	hypothetical protein	2	-	-	-	-	-	-	-	-	-	-	-	-	-	-	-	-	-	-	-	-	-	-	-	-	-	-	-	-	-	-	-	-	-	-	+	+	-	-	-	-	-	-	-	-	-	-	-	-	-	-	-	-	-
4129	7431	hypothetical protein	2	-	-	-	-	-	-	-	-	-	-	-	-	-	-	-	-	-	-	-	-	-	-	-	-	-	-	-	-	-	-	-	-	-	-	+	+	-	-	-	-	-	-	-	-	-	-	-	-	-	-	-	-	-
4130	7433	COG1396: Predicted transcriptional regulators	2	-	-	-	-	-	-	-	-	-	-	-	-	-	-	-	-	-	-	-	-	-	-	-	-	-	-	-	-	-	-	-	-	-	-	+	+	-	-	-	-	-	-	-	-	-	-	-	-	-	-	-	-	-
4131	7434	hydrolase	2	-	-	-	-	-	-	-	-	-	-	-	-	-	-	-	-	-	-	-	-	-	-	-	-	-	-	-	-	-	-	-	-	-	-	+	+	-	-	-	-	-	-	-	-	-	-	-	-	-	-	-	-	-
4132	7436	DNA-cytosine methyltransferase (EC 2.1.1.37)	2	-	-	-	-	-	-	-	-	-	-	-	-	-	-	-	-	-	-	-	-	-	-	-	-	-	-	-	-	-	-	-	-	-	-	-	+	-	-	-	-	-	-	-	-	-	-	-	-	-	+	-	-	-
4133	7437	hypothetical protein	2	-	-	-	-	-	-	-	-	-	-	-	-	-	-	-	-	-	-	-	-	-	-	-	-	-	-	-	-	-	-	-	-	-	-	-	+	-	-	-	-	-	-	-	-	-	+	-	-	-	-	-	-	-
4134	7438	hypothetical protein	2	-	-	-	-	-	-	-	-	-	-	-	-	-	-	-	-	-	-	-	-	-	-	-	-	-	-	-	-	-	-	-	-	-	-	-	+	-	-	-	-	-	-	+	-	-	-	-	-	-	-	-	-	-
4135	7439	hypothetical protein	2	-	-	-	-	-	-	-	-	-	-	-	-	-	-	-	-	-	-	-	-	-	-	-	-	-	-	-	-	-	-	-	-	-	-	-	+	-	-	-	-	-	-	+	-	-	-	-	-	-	-	-	-	-
4136	7440	hypothetical protein	2	-	-	-	-	-	-	-	-	-	-	-	-	-	-	-	-	-	-	-	-	-	-	-	-	-	-	-	-	-	-	-	-	-	-	-	+	-	-	-	-	-	-	-	-	-	-	-	+	-	-	-	-	-
4137	7442	hypothetical protein	2	-	-	-	-	-	-	-	-	-	-	-	-	-	-	-	-	-	-	-	-	-	-	-	-	-	-	-	-	-	-	-	-	-	-	-	-	-	+	-	-	-	+	-	-	-	-	-	-	-	-	-	-	-
4138	7444	Capsular polysaccharide biosynthesis protein CpsK(V)	2	-	-	-	-	-	-	-	-	-	-	-	-	-	-	-	-	-	-	-	-	-	-	-	-	-	-	-	-	-	-	-	-	-	-	-	-	-	-	-	-	+	+	-	-	-	-	-	-	-	-	-	-	-
4139	7445	hypothetical protein	2	-	-	-	-	-	-	-	-	-	-	-	-	-	-	-	-	-	-	-	-	-	-	-	-	-	-	-	-	-	-	-	-	-	-	-	-	-	-	-	-	+	+	-	-	-	-	-	-	-	-	-	-	-
4140	7446	hypothetical protein	2	-	-	-	-	-	-	-	-	-	-	-	-	-	-	-	-	-	-	+	-	-	-	-	-	-	-	-	-	-	-	-	-	-	-	-	-	-	-	-	-	-	+	-	-	-	-	-	-	-	-	-	-	-
4141	7447	hypothetical protein	2	-	-	-	-	-	-	-	-	-	-	-	-	-	-	-	-	-	-	-	-	-	-	-	-	-	-	-	-	-	-	-	-	-	-	-	-	-	-	-	-	+	+	-	-	-	-	-	-	-	-	-	-	-
4142	7448	hypothetical protein	2	-	-	-	-	-	-	-	-	-	-	-	-	-	-	-	-	-	-	-	-	-	-	-	-	-	-	-	-	-	-	-	-	-	-	-	-	-	-	-	-	+	+	-	-	-	-	-	-	-	-	-	-	-
4143	7449	hypothetical protein	2	-	-	-	-	-	-	-	-	-	-	-	-	-	-	-	-	-	-	-	-	-	-	-	-	-	-	-	-	-	-	-	-	-	-	-	-	-	-	-	-	+	+	-	-	-	-	-	-	-	-	-	-	-
4144	7450	hypothetical protein	2	-	-	-	-	-	-	-	-	-	-	-	-	-	-	-	-	-	-	-	-	-	-	-	-	-	-	-	-	-	-	-	-	-	-	-	-	-	+	-	-	-	+	-	-	-	-	-	-	-	-	-	-	-
4145	7451	hypothetical protein	2	-	-	-	-	-	-	-	-	-	-	-	-	-	-	-	-	-	-	-	-	-	-	-	-	-	-	-	-	-	-	-	-	-	-	-	-	-	-	-	-	+	+	-	-	-	-	-	-	-	-	-	-	-
4146	7452	hypothetical protein	2	-	-	-	-	-	-	-	-	-	-	-	-	-	-	-	-	-	-	-	+	-	-	-	-	-	-	-	-	-	-	-	-	-	-	-	-	-	-	-	-	-	+	-	-	-	-	-	-	-	-	-	-	-
4147	7453	hypothetical protein	2	-	-	-	-	-	-	-	-	-	-	-	-	-	-	-	-	-	-	-	-	-	-	-	-	-	-	-	-	-	-	-	-	-	-	-	-	-	-	-	-	+	+	-	-	-	-	-	-	-	-	-	-	-
4148	7454	Cell surface protein	2	-	-	-	-	-	-	-	-	-	-	-	-	-	-	-	-	-	-	-	-	-	-	-	-	-	-	-	-	-	-	-	-	-	-	-	-	-	-	-	-	-	+	-	-	+	-	-	-	-	-	-	-	-
4149	7455	hypothetical protein	2	-	-	-	-	-	-	-	-	-	-	-	-	-	-	-	+	-	-	-	-	-	-	-	-	-	-	-	-	-	-	-	-	-	-	-	-	-	-	-	-	-	+	-	-	-	-	-	-	-	-	-	-	-
4150	7456	hypothetical protein	2	-	-	-	-	-	-	-	-	-	-	-	-	-	-	-	-	-	-	-	-	-	-	-	-	-	-	-	-	-	-	-	-	-	-	-	-	-	-	-	-	+	+	-	-	-	-	-	-	-	-	-	-	-
4151	7457	hypothetical protein	2	-	-	-	-	-	-	-	-	-	-	-	-	-	-	-	-	-	-	-	-	-	-	+	-	-	-	-	+	-	-	-	-	-	-	-	-	-	-	-	-	-	-	-	-	-	-	-	-	-	-	-	-	-
4152	7458	hypothetical protein	2	-	-	-	-	-	-	-	-	-	-	-	-	-	-	-	-	-	-	-	-	-	-	+	+	-	-	-	-	-	-	-	-	-	-	-	-	-	-	-	-	-	-	-	-	-	-	-	-	-	-	-	-	-
4153	7459	Cysteine synthase (EC 2.5.1.47)	2	-	-	-	-	-	-	-	-	-	-	-	-	-	-	-	-	-	-	-	-	-	-	+	-	-	-	-	-	-	-	-	-	-	-	-	-	-	+	-	-	-	-	-	-	-	-	-	-	-	-	-	-	-	-	-	-
4154	7460	hypothetical protein	2	-	-	-	-	-	-	-	-	-	-	-	-	-	-	-	-	-	-	-	-	-	-	+	-	-	-	-	+	-	-	-	-	-	-	-	-	-	-	-	-	-	-	-	-	-	-	-	-	-	-	-	-	-
4155	7462	Surface protein from Gram-positive cocci, anchor region	2	-	-	-	-	-	-	-	-	-	-	-	-	-	-	-	-	-	-	-	-	-	-	+	+	-	-	-	-	-	-	-	-	-	-	-	-	-	-	-	-	-	-	-	-	-	-	-	-	-	-	-	-	-
4156	7463	hypothetical protein	2	-	-	-	-	-	-	-	-	-	-	-	-	-	-	-	-	-	-	-	-	-	-	+	+	-	-	-	-	-	-	-	-	-	-	-	-	-	-	-	-	-	-	-	-	-	-	-	-	-	-	-	-	-
4157	7465	hypothetical protein	2	-	-	-	-	-	-	-	-	-	-	-	-	-	-	-	-	-	-	-	-	-	-	+	-	-	+	-	-	-	-	-	-	-	-	-	-	-	-	-	-	-	-	-	-	-	-	-	-	-	-	-	-	-
4158	7466	unknown phage protein	2	-	-	-	-	-	-	-	-	-	-	-	-	-	-	-	-	-	-	-	-	-	-	+	-	-	-	-	-	-	-	-	-	-	-	-	-	-	-	-	-	-	-	-	-	-	-	-	-	+	-	-	-	-
4159	7467	DNA segregation ATPase FtsK/SpoIIIE or related protein	2	-	-	-	-	-	-	-	-	-	-	-	-	-	-	-	-	-	-	-	-	-	-	+	-	-	-	-	+	-	-	-	-	-	-	-	-	-	-	-	-	-	-	-	-	-	-	-	-	-	-	-	-	-
4160	7468	hypothetical protein	2	-	-	-	-	-	-	-	-	-	-	-	-	-	-	-	-	-	-	-	-	-	-	+	+	-	-	-	-	-	-	-	-	-	-	-	-	-	-	-	-	-	-	-	-	-	-	-	-	-	-	-	-	-
4161	7469	hypothetical protein	2	-	-	-	-	-	-	-	-	-	-	-	-	-	-	-	-	-	-	-	-	-	-	+	-	-	-	-	+	-	-	-	-	-	-	-	-	-	-	-	-	-	-	-	-	-	-	-	-	-	-	-	-	-
4162	7472	hypothetical protein	2	-	-	-	-	-	-	-	-	-	-	-	-	-	-	-	-	-	-	-	-	-	-	+	-	-	-	-	-	-	+	-	-	-	-	-	-	-	-	-	-	-	-	-	-	-	-	-	-	-	-	-	-	-
4163	7473	Hypothetical SAR0365 homolog in superantigen-encoding pathogenicity islands SaPI	2	-	-	-	-	-	-	-	-	-	-	-	-	-	-	-	-	-	-	-	-	-	-	+	-	-	-	-	-	-	-	-	+	-	-	-	-	-	-	-	-	-	-	-	-	-	-	-	-	-	-	-	-	-
4164	7474	hypothetical protein	2	-	-	-	-	-	-	-	-	-	-	-	-	-	-	-	-	-	-	-	-	-	-	+	-	-	-	-	+	-	-	-	-	-	-	-	-	-	-	-	-	-	-	-	-	-	-	-	-	-	-	-	-	-
4165	7475	hypothetical protein	2	-	-	-	-	-	-	-	-	-	-	-	-	-	-	-	-	-	-	-	-	-	-	+	-	-	-	-	-	-	-	-	-	-	-	-	-	-	-	-	-	-	-	-	-	-	-	-	-	+	-	-	-	-
4166	7476	hypothetical protein	2	-	-	-	-	-	-	-	-	-	-	-	-	-	-	-	+	-	-	-	-	-	-	-	-	-	-	-	-	-	-	-	-	-	-	-	-	-	-	-	-	-	-	-	-	-	-	+	-	-	-	-	-	-
4167	7477	Surface protein Rib	2	-	-	-	-	-	-	-	-	-	-	-	-	-	-	+	+	-	-	-	-	-	-	-	-	-	-	-	-	-	-	-	-	-	-	-	-	-	-	-	-	-	-	-	-	-	-	-	-	-	-	-	-	-
4168	7478	hypothetical protein	2	-	-	-	-	-	-	-	-	-	-	-	-	-	-	-	-	-	-	-	-	-	-	-	-	-	-	-	-	-	-	-	-	-	-	-	-	-	-	-	-	-	-	-	-	-	-	+	-	-	-	-	+	-
4169	7480	hypothetical protein	2	-	-	-	-	-	-	-	-	-	-	-	-	-	-	-	+	-	-	-	-	-	-	-	-	-	-	-	-	-	-	-	-	-	-	-	-	-	-	-	-	-	-	-	+	-	-	-	-	-	-	-	-	-
4170	7482	Putative C5 methylase (MAV1virus-like)	2	-	-	-	-	-	-	-	-	-	-	-	-	-	-	-	+	-	-	-	-	-	-	-	-	-	-	-	-	-	-	-	-	-	-	-	-	-	-	-	+	-	-	-	-	-	-	-	-	-	-	-	-	-
4171	7483	hypothetical protein	2	-	-	-	-	-	-	-	-	-	-	-	-	-	-	-	+	-	-	+	-	-	-	-	-	-	-	-	-	-	-	-	-	-	-	-	-	-	-	-	-	-	-	-	-	-	-	-	-	-	-	-	-	-
4172	7484	hypothetical protein	2	-	-	-	-	-	-	-	-	-	-	-	-	-	-	-	+	-	-	-	-	-	-	-	-	-	-	-	-	-	-	-	-	-	-	-	-	-	-	-	-	-	-	-	-	-	+	-	-	-	-	-	-	-
4173	7485	hypothetical protein	2	-	-	-	-	-	-	-	-	-	-	-	-	-	-	-	+	-	-	-	-	-	-	-	-	-	-	-	-	-	-	-	-	-	-	-	-	-	-	-	+	-	-	-	-	-	-	-	-	-	-	-	-	-
4174	7486	hypothetical protein	2	-	-	-	-	-	-	-	-	-	-	-	-	-	-	-	+	-	-	-	-	-	-	-	-	-	-	-	-	-	-	-	-	-	-	-	-	-	+	-	-	-	-	-	-	-	-	-	-	-	-	-	-	-
4175	7487	UDP-N-acetylglucosamine 1-carboxyvinyltransferase (EC 2.5.1.7)	2	-	-	-	-	-	-	-	-	-	-	+	-	-	-	-	+	-	-	-	-	-	-	-	-	-	-	-	-	-	-	-	-	-	-	-	-	-	-	-	-	-	-	-	-	-	-	-	-	-	-	-	-	-
4176	7488	hypothetical protein	2	-	-	-	-	-	-	-	-	-	-	-	-	-	-	-	+	-	-	-	-	-	-	-	-	-	-	-	-	-	-	-	-	-	-	-	-	-	+	-	-	-	-	-	-	-	-	-	-	-	-	-	-	-
4177	7489	hypothetical protein	2	-	-	-	-	-	-	-	-	-	-	-	-	-	-	-	+	-	-	+	-	-	-	-	-	-	-	-	-	-	-	-	-	-	-	-	-	-	-	-	-	-	-	-	-	-	-	-	-	-	-	-	-	-
4178	7491	hypothetical protein	2	-	-	-	-	-	-	-	-	-	-	-	-	-	-	-	+	-	-	-	-	-	-	-	-	-	-	-	-	-	-	-	-	-	-	-	-	-	-	-	+	-	-	-	-	-	-	-	-	-	-	-	-	-
4179	7493	hypothetical protein	2	-	-	-	-	-	-	-	-	-	-	-	-	-	-	+	+	-	-	-	-	-	-	-	-	-	-	-	-	-	-	-	-	-	-	-	-	-	-	-	-	-	-	-	-	-	-	-	-	-	-	-	-	-
4180	7494	hypothetical protein	2	-	-	-	-	-	-	-	-	-	-	-	-	-	-	+	+	-	-	-	-	-	-	-	-	-	-	-	-	-	-	-	-	-	-	-	-	-	-	-	-	-	-	-	-	-	-	-	-	-	-	-	-	-
4181	7495	Integrase	2	-	-	-	-	-	-	-	-	-	-	-	-	+	-	-	+	-	-	-	-	-	-	-	-	-	-	-	-	-	-	-	-	-	-	-	-	-	-	-	-	-	-	-	-	-	-	-	-	-	-	-	-	-
4182	7497	hypothetical protein	2	-	-	-	-	-	-	-	-	-	-	-	-	-	-	-	-	-	-	-	-	-	-	-	-	-	-	-	-	-	-	-	-	-	-	-	-	-	-	-	-	-	-	-	-	-	+	-	-	-	-	-	-	+
4183	7498	hypothetical protein	2	-	-	-	-	-	-	-	-	-	-	-	-	-	-	-	-	-	-	-	-	-	-	-	-	-	-	-	-	-	-	-	-	-	-	-	-	-	-	-	-	-	-	-	-	-	-	-	-	-	-	-	+	+
4184	7503	hypothetical protein	2	-	-	-	-	-	-	-	-	-	-	-	-	-	-	-	-	-	-	-	-	-	-	-	-	-	-	-	-	-	-	-	-	-	-	-	-	-	-	-	-	-	-	-	-	-	-	-	-	-	-	-	+	+
4185	7504	Preprotein translocase SecY2 subunit (TC 3.A.5.1.1)	2	-	-	-	-	-	-	-	-	-	-	-	-	-	-	-	-	-	-	-	-	-	-	-	-	-	-	-	-	-	-	-	-	-	-	-	-	-	-	-	-	-	-	-	-	-	-	-	-	-	-	-	+	+
4186	7505	Glycosyl transferase	2	-	-	-	-	-	-	-	-	-	-	-	-	-	-	-	-	-	-	-	-	-	-	-	-	-	-	-	-	-	-	-	-	-	-	-	-	-	-	-	-	-	-	-	-	-	-	-	-	-	-	-	+	+
4187	7506	hypothetical protein	2	-	-	-	-	-	-	-	-	-	-	-	-	-	-	-	-	-	-	-	-	-	-	-	-	-	-	-	-	-	-	-	-	-	-	-	-	-	-	-	-	-	-	-	-	-	-	-	-	-	-	+	-	+
4188	7507	Muramidase-released protein precursor (136 kDa surface protein)	2	-	-	-	-	-	-	-	-	-	-	-	-	-	-	-	-	-	-	-	-	-	-	-	-	-	-	-	-	-	-	-	-	-	-	-	-	-	-	-	-	-	-	-	-	-	-	-	-	-	+	-	-	+
4189	7509	Accessory secretory protein Asp3	2	-	-	-	-	-	-	-	-	-	-	-	-	-	-	-	-	-	-	-	-	-	-	-	-	-	-	-	-	-	-	-	-	-	-	-	-	-	-	-	-	-	-	-	-	-	-	-	-	-	-	-	+	+
4190	7510	Xaa-His dipeptidase (EC 3.4.13.3)	2	-	-	-	-	-	-	-	-	-	-	-	-	-	-	-	-	-	-	-	-	-	-	-	-	-	-	-	-	-	-	-	-	-	-	-	-	-	-	-	-	-	-	-	-	-	-	-	-	-	-	-	+	+
4191	7511	Phage minor tail protein	2	-	-	-	-	-	-	-	-	-	-	-	-	-	-	-	-	-	-	-	-	-	-	-	-	-	-	-	-	-	-	-	-	-	-	-	-	-	-	-	-	-	-	-	-	-	+	-	-	-	-	-	-	+
4192	7512	type II restriction endonuclease	2	-	-	-	-	-	-	-	-	-	-	-	-	-	-	-	-	-	-	-	-	-	-	-	-	-	-	-	-	-	-	-	-	-	-	-	-	+	-	-	-	-	-	-	-	-	-	-	-	-	-	-	-	+
4193	7513	hypothetical protein	2	-	-	-	-	-	-	-	-	-	-	-	-	-	-	-	-	-	-	-	-	-	-	-	-	-	-	-	-	+	-	-	-	-	-	-	-	-	-	-	-	-	-	-	-	-	-	-	-	-	-	-	-	+
4194	7516	Isoprenylcysteine carboxyl methyltransferase (ICMT) family superfamily	2	-	-	-	-	-	-	-	-	-	-	-	-	-	-	-	-	-	-	-	-	-	-	-	-	-	-	-	-	-	-	-	-	-	-	-	-	-	-	-	-	-	-	-	-	-	-	-	-	-	-	-	+	+
4195	7518	hypothetical protein	2	-	-	-	-	-	-	-	-	-	-	-	-	-	-	-	-	-	-	-	-	-	-	-	-	-	-	-	-	-	-	-	-	-	-	-	-	-	-	+	-	-	-	-	-	-	-	-	-	-	-	-	-	+
4196	7519	hypothetical protein	2	-	-	-	-	-	-	-	-	-	-	-	-	-	-	-	-	-	-	-	-	-	-	-	-	-	-	-	-	-	-	-	-	-	-	-	-	-	-	-	-	-	-	-	-	-	-	-	-	-	-	-	+	+
4197	7520	hypothetical protein	2	-	-	-	-	-	-	-	-	-	-	-	-	-	-	-	-	-	-	-	-	-	-	-	-	-	-	-	-	-	-	-	-	-	-	-	-	-	-	-	-	-	-	-	-	-	-	-	-	-	-	-	+	+
4198	7521	hypothetical protein	2	-	-	-	-	-	-	-	-	-	-	-	-	-	-	-	-	-	-	-	-	-	-	-	-	-	-	-	-	-	-	-	-	-	-	-	-	-	-	-	-	-	-	-	-	-	-	-	-	-	-	-	+	+
4199	7522	hypothetical protein	2	-	-	-	-	-	-	-	-	-	-	-	-	-	-	-	-	-	-	-	-	-	-	-	-	-	-	-	-	-	-	-	-	-	-	-	-	-	-	-	-	-	-	-	-	+	-	-	-	-	-	-	-	+
4200	7523	hypothetical protein	2	-	-	-	-	-	-	-	-	-	-	-	-	-	-	-	-	-	-	-	-	-	-	-	-	-	-	-	-	-	-	+	-	-	-	-	-	-	-	-	-	-	-	-	-	-	-	-	-	-	-	-	-	+
4201	7524	hypothetical protein	2	-	-	-	-	-	-	-	-	-	-	-	-	-	-	-	-	-	-	-	-	-	-	-	-	-	-	-	-	-	-	-	-	-	-	-	-	-	-	+	-	-	-	-	-	-	-	-	-	-	-	-	-	+
4202	7525	putative glycosyltransferase	2	-	-	-	-	-	-	-	-	-	-	-	-	-	-	-	-	-	-	-	-	-	-	-	-	-	-	-	-	-	-	-	-	-	-	-	-	-	-	-	-	-	-	-	-	-	-	-	-	-	-	-	+	+
4203	7526	hypothetical protein	2	-	-	-	-	-	-	-	-	-	-	-	-	-	-	-	-	-	-	-	-	-	-	-	-	-	-	-	-	-	-	-	-	-	-	-	-	-	-	-	-	-	-	-	-	-	-	-	-	-	-	-	+	+
4204	7527	hypothetical protein	2	-	-	-	-	-	-	-	-	-	-	-	-	-	-	-	-	-	-	-	-	-	-	-	-	-	-	-	-	-	-	-	-	-	-	-	-	-	+	-	-	-	-	-	-	-	-	-	-	-	-	-	-	+
4205	7528	ftsK-like DNA segregation ATPase	2	-	-	-	-	-	-	-	-	-	-	-	-	-	-	-	-	-	-	-	-	-	-	-	-	-	-	-	-	-	-	-	-	-	-	-	-	-	-	-	-	-	-	-	-	-	-	-	-	-	-	-	+	+
4206	7529	hypothetical protein	2	-	-	-	-	-	-	-	-	-	-	-	-	-	-	-	-	-	-	-	-	-	-	-	-	-	-	-	-	-	-	-	-	-	-	-	-	-	-	-	-	-	-	-	-	-	-	-	-	-	-	-	+	+
4207	7530	hypothetical protein	2	-	-	-	-	-	-	-	-	-	-	-	-	-	-	-	-	-	-	-	-	-	-	-	-	-	-	-	-	-	-	-	-	-	-	-	-	-	-	-	-	-	-	-	-	-	-	-	+	-	-	-	-	+
4208	7531	hypothetical protein	2	-	-	-	-	-	-	-	-	-	-	-	-	-	-	-	-	-	-	-	-	-	-	-	-	-	-	-	-	-	-	-	-	-	-	-	-	-	-	+	-	-	-	-	-	-	-	-	-	-	-	-	-	+
4209	7532	Accessory secretory protein Asp1	2	-	-	-	-	-	-	-	-	-	-	-	-	-	-	-	-	-	-	-	-	-	-	-	-	-	-	-	-	-	-	-	-	-	-	-	-	-	-	-	-	-	-	-	-	-	-	-	-	-	-	-	+	+
4210	7533	hypothetical protein	2	-	-	-	-	-	-	-	-	-	-	-	-	-	-	-	-	-	-	-	-	-	-	-	-	-	-	-	-	-	-	-	-	-	-	-	-	+	-	-	-	-	-	-	-	-	-	-	-	-	-	-	-	+
4211	7534	hypothetical protein	2	-	-	-	-	-	-	-	-	-	-	-	-	-	-	-	-	-	-	-	-	-	-	-	-	-	-	-	-	-	-	-	-	-	-	-	-	-	-	-	-	-	-	-	-	-	-	-	-	-	-	-	+	+
4212	7536	hypothetical protein	2	-	-	-	-	-	-	-	-	-	-	-	-	-	-	-	-	-	-	-	-	-	-	-	-	-	-	-	-	-	-	-	-	-	-	-	-	+	-	-	-	-	-	-	+	-	-	-	-	-	-	-	-	-
4213	7537	Glycosyl transferase	2	-	-	-	-	-	-	-	-	-	-	-	-	-	-	-	-	-	-	-	-	-	-	-	-	-	-	-	-	-	-	-	-	-	-	-	-	-	-	-	-	-	-	-	-	-	-	-	-	-	-	-	-	-	-	-	-	-	-	-
4214	7539	hypothetical protein	2	-	-	-	-	-	-	-	-	-	-	-	-	-	-	-	-	-	-	-	-	-	-	-	-	-	-	-	-	-	-	-	-	-	-	-	-	-	+	-	-	-	-	-	+	-	-	-	-	-	-	-	-	-
4215	7540	O-acetyltransferase	2	-	-	-	-	-	-	-	-	-	-	-	-	-	-	-	-	-	-	-	-	-	-	-	-	-	-	-	-	-	-	-	-	-	-	-	-	-	+	-	-	-	-	-	+	-	-	-	-	-	-	-	-	-
4216	7542	hypothetical protein	2	-	-	-	-	-	-	-	-	-	-	-	-	-	-	-	-	-	-	-	-	-	-	-	-	-	-	-	-	-	-	-	-	-	-	-	-	-	-	-	-	-	-	-	+	-	-	-	-	-	-	-	+	-
4217	7544	hypothetical protein	2	-	-	-	-	-	-	-	-	-	-	-	-	-	-	-	-	-	-	-	-	-	-	-	-	-	-	-	-	-	-	-	-	-	-	-	-	-	-	-	-	-	-	+	+	-	-	-	-	-	-	-	-	-
4218	7545	Hemagglutinin/hemolysin-related protein	2	-	-	-	-	-	-	-	-	-	-	-	-	-	-	-	-	-	-	-	-	-	-	-	-	-	-	-	-	-	-	-	-	-	-	-	-	+	-	-	-	-	-	+	-	-	-	-	-	-	-	-	-	-
4219	7546	hypothetical protein	2	-	-	-	-	-	-	-	-	-	-	-	-	-	-	-	-	-	-	-	-	-	-	-	-	-	-	-	-	-	-	-	-	-	-	-	-	-	-	-	-	-	-	+	+	-	-	-	-	-	-	-	-	-
4220	7547	unknown protein	2	-	-	-	-	-	-	-	-	-	-	-	-	-	-	-	-	-	-	-	-	-	-	-	-	-	-	-	-	-	-	-	-	-	-	-	-	-	-	-	-	+	-	-	+	-	-	-	-	-	-	-	-	-
4221	7548	DNA segregation ATPase FtsK/SpoIIIE family protein	2	-	-	-	-	-	-	-	-	-	-	-	-	-	-	-	-	-	-	-	-	-	-	-	-	-	-	-	-	-	-	-	-	-	-	-	-	-	+	-	-	-	-	-	+	-	-	-	-	-	-	-	-	-
4222	7549	hypothetical protein	2	-	-	-	-	-	-	-	-	-	-	-	-	-	-	-	-	-	-	-	-	-	-	-	-	-	-	-	-	-	-	-	-	-	-	-	-	-	-	-	-	-	-	-	+	-	-	-	+	-	-	-	-	-
4223	7550	hypothetical protein	2	-	-	-	-	-	-	-	-	-	-	-	-	-	-	-	-	-	-	-	-	-	-	-	-	-	-	-	-	-	-	-	-	-	-	-	-	-	-	-	-	+	-	-	+	-	-	-	-	-	-	-	-	-
4224	7551	hypothetical protein	2	-	-	-	-	-	-	-	-	-	-	-	-	-	-	-	-	-	-	-	-	-	-	-	-	-	-	-	-	-	-	-	-	-	-	-	-	-	-	-	-	-	-	-	+	-	-	-	+	-	-	-	-	-
4225	7553	Orf9	2	-	-	-	-	-	-	-	-	-	-	-	-	-	-	-	-	-	-	-	-	-	-	-	-	-	-	-	-	-	-	-	-	-	-	-	-	-	-	-	+	-	-	-	-	-	-	-	-	-	-	-	+	-
4226	7554	Transcriptional regulator	2	-	-	-	-	-	-	-	-	-	-	-	-	-	-	-	-	-	-	-	-	-	-	-	-	-	-	-	-	-	-	-	-	-	-	-	-	-	-	-	-	+	-	-	+	-	-	-	-	-	-	-	-	-
4227	7555	hypothetical protein	2	-	-	-	-	-	-	-	-	-	-	-	-	-	-	-	-	-	-	-	-	-	-	-	-	-	-	-	-	-	-	-	-	-	-	-	-	-	-	-	-	-	-	-	+	-	-	-	-	-	-	-	+	-
4228	7557	Type I restriction-modification system	2	-	-	-	-	-	-	-	-	-	-	-	-	-	-	-	-	-	-	-	-	-	-	-	-	-	-	-	-	-	-	-	-	-	-	-	-	-	-	-	-	-	-	-	+	-	-	-	+	-	-	-	-	-
4229	7559	hypothetical protein	2	-	-	-	-	-	-	-	-	-	-	-	-	-	-	-	-	-	-	-	-	-	-	-	-	-	-	-	-	-	-	-	-	-	-	-	-	-	-	-	-	-	-	-	+	-	-	-	+	-	-	-	-	-
4230	7560	hypothetical protein	2	-	-	-	-	-	-	-	-	-	-	-	-	-	-	-	-	-	-	-	-	-	-	-	-	-	-	-	-	-	-	-	-	-	-	-	-	-	-	-	-	-	-	-	+	-	-	+	-	-	-	-	-	-
4231	7563	hypothetical protein	2	-	-	-	-	-	-	-	-	-	-	-	-	-	-	-	-	-	-	-	-	-	-	-	-	-	-	-	-	-	-	-	-	-	-	-	-	-	-	-	-	-	-	+	+	-	-	-	-	-	-	-	-	-
4232	7566	hypothetical protein	2	-	-	-	-	-	-	-	-	-	-	-	-	-	-	-	-	-	-	-	-	-	-	-	-	-	-	-	-	-	-	-	-	-	-	-	-	-	-	-	-	-	-	-	+	-	-	-	+	-	-	-	-	-
4233	7567	hypothetical protein	2	-	-	-	-	-	-	-	-	-	-	-	-	-	-	-	-	-	-	-	-	-	-	-	-	-	-	-	-	-	-	-	-	-	-	-	-	-	-	-	-	-	-	+	+	-	-	-	-	-	-	-	-	-
4234	7568	glycosyltransferase	2	-	-	-	-	-	-	-	-	-	-	-	-	-	-	-	-	-	-	-	-	-	-	-	-	-	-	-	-	-	-	-	-	-	-	-	-	-	+	-	-	-	-	-	+	-	-	-	-	-	-	-	-	-
4235	7569	hypothetical protein	2	-	-	-	-	-	-	-	-	-	-	-	-	-	-	-	-	-	-	-	-	-	-	-	-	-	-	-	-	-	-	-	-	-	-	-	-	-	-	-	-	-	-	-	+	-	-	-	+	-	-	-	-	-
4236	7570	Spermine/spermidine acetyltransferase (EC 2.3.1.57)	2	-	-	-	-	-	-	-	-	-	-	-	-	-	-	-	-	-	-	-	-	-	-	-	-	-	-	-	-	-	-	-	-	-	-	-	-	-	-	-	-	-	-	-	+	-	-	-	-	-	-	+	-	-
4237	7572	hypothetical protein	2	-	-	-	-	-	-	-	-	-	-	-	-	-	-	-	-	-	-	-	-	-	-	-	-	-	-	-	-	-	-	-	-	-	-	-	-	-	+	-	-	-	-	-	+	-	-	-	-	-	-	-	-	-
4238	7573	hypothetical protein	2	-	-	-	-	-	-	-	-	-	-	-	-	-	-	-	-	-	-	-	-	-	-	-	-	-	-	-	-	-	-	-	-	-	-	-	-	-	-	-	-	-	-	-	+	-	-	-	-	-	-	-	+	-
4239	7574	hypothetical protein	2	-	-	-	-	-	-	-	-	-	-	-	-	-	-	-	-	-	-	-	-	-	-	-	-	-	-	-	-	-	-	-	-	-	-	-	-	-	-	-	-	-	-	+	+	-	-	-	-	-	-	-	-	-
4240	7575	hypothetical protein	2	-	-	-	-	-	-	-	-	-	-	-	-	-	-	-	-	-	-	-	-	-	-	-	-	-	-	-	-	-	-	-	-	-	-	-	-	-	-	-	-	-	-	-	+	-	+	-	-	-	-	-	-	-
4241	7576	Glycosyl transferase	2	-	-	-	-	-	-	-	-	-	-	-	-	-	-	-	-	-	-	-	-	-	-	-	-	-	-	-	-	-	-	-	-	-	-	-	-	-	-	-	-	-	-	-	-	-	-	-	-	-	-	-	-	-	-	-	-	-	-	-
4242	7578	hypothetical protein	2	-	-	-	-	-	-	-	-	-	-	-	-	-	-	-	-	-	-	-	-	-	-	-	-	-	-	-	-	-	-	-	-	-	-	-	-	+	-	-	-	-	-	-	+	-	-	-	-	-	-	-	-	-
4243	7579	hypothetical protein	2	-	-	-	-	-	-	-	-	-	-	-	-	-	-	-	-	-	-	-	-	-	-	-	-	-	-	-	-	-	-	-	-	-	-	-	-	-	+	-	-	-	-	-	+	-	-	-	-	-	-	-	-	-
4244	7580	lipopolysaccharide biosynthesis protein	2	-	-	-	-	-	-	-	-	-	-	-	-	-	-	-	-	-	-	-	-	-	-	-	-	-	-	-	-	-	-	-	-	-	-	-	-	-	+	-	-	-	-	-	+	-	-	-	-	-	-	-	-	-
4245	7581	hypothetical protein	2	-	-	-	-	-	-	-	-	-	-	-	-	-	-	-	-	-	-	-	-	-	-	-	-	-	-	-	-	-	-	-	-	-	-	-	-	+	+	-	-	-	-	-	-	-	-	-	-	-	-	-	-	-
4246	7583	hypothetical protein	2	-	-	-	-	-	-	-	-	-	-	-	-	-	-	-	-	-	-	-	-	-	-	-	-	-	-	-	-	-	-	-	-	-	-	-	-	+	+	-	-	-	-	-	-	-	-	-	-	-	-	-	-	-
4247	7585	hypothetical protein	2	-	-	-	-	-	-	-	-	-	-	-	-	-	-	-	-	-	-	-	-	-	-	-	-	-	-	-	-	-	-	-	-	-	-	-	-	+	+	-	-	-	-	-	-	-	-	-	-	-	-	-	-	-
4248	7587	hypothetical protein	2	-	-	-	-	-	-	-	-	-	-	-	-	-	-	-	-	-	-	-	-	-	-	-	-	-	-	-	-	-	-	-	-	-	-	-	-	-	+	-	-	+	-	-	-	-	-	-	-	-	-	-	-	-
4249	7590	hypothetical protein	2	-	-	-	-	-	-	-	-	-	-	-	-	-	-	-	-	-	-	-	-	-	-	-	-	-	-	-	-	-	-	-	-	-	-	-	-	+	+	-	-	-	-	-	-	-	-	-	-	-	-	-	-	-
4250	7591	hypothetical protein	2	-	-	-	-	-	-	-	-	-	-	-	-	-	-	-	-	-	-	-	-	-	-	-	-	-	-	-	-	-	-	-	-	-	-	-	-	-	+	-	-	-	-	-	-	-	-	-	-	-	-	-	+	-
4251	7592	Modification methylase EcoRI (EC 2.1.1.72)	2	-	-	-	-	-	-	-	-	-	-	-	-	-	-	-	-	-	-	-	-	-	-	-	-	-	-	-	-	-	-	-	-	-	-	-	-	+	+	-	-	-	-	-	-	-	-	-	-	-	-	-	-	-
4252	7595	Putative toxin component near putative ESAT-related proteins	2	-	-	-	-	-	-	-	-	-	-	-	-	-	-	-	-	-	-	-	-	-	-	-	-	-	-	-	-	-	-	-	-	-	-	-	-	+	+	-	-	-	-	-	-	-	-	-	-	-	-	-	-	-
4253	7596	Glycosyltransferase (EC 2.4.1.-)	2	-	-	-	-	-	-	-	-	-	-	-	-	-	-	-	-	-	-	-	-	-	-	-	-	-	-	-	-	-	-	-	-	-	-	-	-	-	+	-	-	-	-	-	-	-	+	-	-	-	-	-	-	-
4254	7597	hypothetical protein	2	-	-	-	-	-	-	-	-	-	-	-	-	-	-	-	-	-	-	-	-	-	-	-	-	-	-	-	-	-	-	-	-	-	-	-	-	-	+	-	-	-	-	+	-	-	-	-	-	-	-	-	-	-
4255	7598	hypothetical protein	2	-	-	-	-	-	-	-	-	-	-	-	-	-	-	-	-	-	-	-	-	-	-	-	-	-	-	-	-	-	-	-	-	-	-	-	-	+	+	-	-	-	-	-	-	-	-	-	-	-	-	-	-	-
4256	7601	hypothetical protein	2	-	-	-	-	-	-	-	-	-	-	-	-	-	-	-	-	-	-	-	-	-	-	-	-	-	-	-	-	-	-	-	-	-	-	-	-	-	+	-	-	-	-	-	-	-	-	-	-	-	-	-	+	-
4257	7604	hypothetical protein	2	-	-	-	-	-	-	-	-	-	-	-	-	-	-	-	-	-	-	-	-	-	-	-	-	-	-	-	-	-	-	-	-	-	-	-	-	+	+	-	-	-	-	-	-	-	-	-	-	-	-	-	-	-
4258	7606	hypothetical protein	2	-	-	-	-	-	-	-	-	-	-	-	-	-	-	-	-	-	-	-	-	-	-	-	-	-	-	-	-	-	-	-	-	-	-	-	-	+	+	-	-	-	-	-	-	-	-	-	-	-	-	-	-	-
4259	7607	hypothetical protein	2	-	-	-	-	-	-	-	-	-	-	-	-	-	-	-	-	-	-	-	-	-	-	-	-	-	-	-	-	-	-	-	-	-	-	-	-	+	+	-	-	-	-	-	-	-	-	-	-	-	-	-	-	-
4260	7608	hypothetical protein	2	-	-	-	-	-	-	-	-	-	-	-	-	-	-	-	-	-	-	-	-	-	-	-	-	-	-	-	-	-	-	-	-	-	-	-	-	+	-	+	-	-	-	-	-	-	-	-	-	-	-	-	-	-
4261	7609	hypothetical protein	2	-	-	-	-	-	-	-	-	-	-	-	-	-	-	-	-	-	-	-	-	-	-	-	-	-	-	-	-	-	-	-	-	-	-	-	-	-	-	+	-	-	-	+	-	-	-	-	-	-	-	-	-	-
4262	7611	hypothetical protein	2	-	-	-	-	-	-	-	-	-	-	-	-	-	-	-	-	-	-	-	-	-	-	-	-	-	-	-	-	-	-	-	-	-	-	-	-	-	+	-	-	-	-	-	-	-	-	+	-	-	-	-	-	-
4263	7612	hypothetical protein	2	-	-	-	-	-	-	-	-	-	-	-	-	-	-	-	-	-	-	-	-	-	-	-	-	-	-	-	-	-	-	-	-	-	-	-	-	+	+	-	-	-	-	-	-	-	-	-	-	-	-	-	-	-
4264	7613	77ORF009	2	-	-	-	-	-	-	-	-	-	-	-	-	-	-	-	-	-	-	-	-	-	-	-	-	-	-	-	-	-	-	-	-	-	-	-	-	-	+	-	-	-	-	-	-	-	-	-	-	-	-	-	+	-
4265	7615	lantibiotic mersacidin modifying enzyme	2	-	-	-	-	-	-	-	-	-	-	-	-	-	-	-	-	-	-	-	-	-	-	-	-	-	-	-	-	-	-	-	-	-	-	-	-	+	-	-	-	-	-	+	-	-	-	-	-	-	-	-	-	-
4266	7617	Phage major capsid protein	2	-	-	-	-	-	-	-	-	-	-	-	-	-	-	-	-	-	-	-	-	-	-	-	-	-	-	-	-	-	-	-	-	-	-	-	-	+	-	-	-	-	-	-	-	-	-	-	-	-	-	+	-	-
4267	7619	Phage capsid and scaffold	2	-	-	-	-	-	-	-	-	-	-	-	-	-	-	-	-	-	-	-	-	-	-	-	-	-	-	-	-	-	-	-	-	-	-	-	-	+	-	-	-	-	-	-	-	-	-	-	-	-	-	+	-	-
4268	7620	hypothetical protein	2	-	-	-	-	-	-	-	-	-	-	-	-	-	-	-	-	-	-	-	-	-	-	-	-	-	-	-	-	-	-	-	-	-	-	-	-	+	-	-	-	-	-	-	-	-	-	-	+	-	-	-	-	-
4269	7623	hypothetical protein	2	-	-	-	-	-	-	-	-	-	-	-	-	-	-	-	-	-	-	-	-	-	-	-	-	-	-	-	-	-	-	-	-	-	-	-	-	+	-	-	-	-	-	+	-	-	-	-	-	-	-	-	-	-

Table S5. Precence/absence of each HG among 57 *S. suis* strains

				1																13			25					28						104		94			76			53/54		Divergent *S. suis*																
Index[a]	ClutserID	Gene products	Members	98HAH33	05ZYH33	SC84	A7	SS12	P1/7	DAT273	NIAH11434	JS14	GZ1	BM407	S735	13730	MNCM07	NCTC 10446	14636	NCTC 10237	ST1	2651	89-1591	MNCM04	MNCM25	8074	D9	4961	ST3	MNCM43	DAT292	DAT260	NIAH11435	MNCM21	MNCM50	89-2479	DAT234	DAT290	93A	42A	NT77	6407	11538	8830	12814	92-1400	89-590	2726	2524	89-3576-3	4417	D12	10581	89-5259	88-5299A	R61	92-1191	14A	92-4172	22083
4270	7624	hypothetical protein	2	-	-	-	-	-	-	-	-	-	-	-	-	-	-	-	-	-	-	-	-	-	-	-	-	-	-	-	-	-	-	-	-	-	-	-	-	-	-	-	-	+	-	+	-	-	-	-	-	-	-	-	-	-	-	-	-	-
4271	7625	Lipopolysaccharide biosynthesis protein WzxC	2	-	-	-	-	-	-	-	-	-	-	-	-	-	-	-	-	-	-	-	-	-	-	-	-	-	-	-	-	-	-	-	-	-	-	-	-	-	-	-	-	+	-	-	-	-	-	-	-	-	-	-	-	+	-	-	-	-
4272	7627	Type I restriction-modification system	2	-	-	-	-	-	-	-	-	-	-	-	-	-	-	-	-	-	-	-	-	-	-	-	-	-	-	-	-	-	-	-	-	-	-	-	-	-	-	-	-	+	-	-	-	-	-	-	-	-	-	-	+	-	-	-	-	-
4273	7629	lantibiotic mersacidin transporter system	2	-	-	-	-	-	-	-	-	-	-	-	-	-	-	-	-	-	-	-	-	-	-	-	-	-	-	-	-	-	-	-	-	-	-	-	-	-	-	-	-	+	-	-	-	-	-	+	-	-	-	-	-	-	-	-	-	-
4274	7631	hypothetical protein	2	-	-	-	-	-	-	-	-	-	-	-	-	-	-	-	-	-	+	-	-	-	-	-	-	-	-	-	-	-	-	-	-	-	-	-	-	-	-	-	-	+	-	-	-	-	-	-	-	-	-	-	-	-	-	-	-	-
4275	7632	hypothetical protein	2	-	-	-	-	-	-	-	-	-	-	-	-	-	-	-	-	-	-	-	-	-	-	-	-	-	-	-	-	-	-	-	-	-	-	-	-	-	-	-	-	+	-	-	-	-	-	-	-	-	-	+	-	-	-	-	-	-
4276	7633	Transcriptional regulator	2	-	-	-	-	-	-	-	-	-	-	-	-	-	-	-	-	-	-	-	-	-	-	-	-	-	-	-	-	-	-	-	-	-	-	-	-	-	-	-	-	+	-	+	-	-	-	-	-	-	-	-	-	-	-	-	-	-
4277	7634	Orf32	2	-	-	-	-	-	-	-	-	-	-	-	-	-	-	-	-	-	-	-	-	-	-	-	-	-	-	-	-	-	-	-	-	-	-	-	-	-	-	-	-	+	-	+	-	-	-	-	-	-	-	-	-	-	-	-	-	-
4278	7635	hypothetical protein	2	-	-	-	-	-	-	-	-	-	-	-	-	-	-	-	-	-	-	+	-	-	-	-	-	-	-	-	-	-	-	-	-	-	-	-	-	-	-	-	-	+	-	-	-	-	-	-	-	-	-	-	-	-	-	-	-	-
4279	7636	hypothetical protein	2	-	-	-	-	-	-	-	-	-	-	-	-	-	-	-	-	-	-	-	-	-	-	-	-	-	-	-	-	-	-	-	-	-	-	-	-	-	-	-	-	+	-	-	-	-	-	-	-	+	-	-	-	-	-	-	-	-
4280	7638	hypothetical protein	2	-	-	-	-	-	-	-	-	-	-	-	-	-	-	-	-	-	-	-	-	-	-	-	-	-	-	-	-	-	-	-	-	-	-	-	-	-	-	-	-	+	-	-	-	-	-	+	-	-	-	-	-	-	-	-	-	-
4281	7639	hypothetical protein	2	-	-	-	-	-	-	-	-	-	-	-	-	-	-	-	-	-	-	+	-	-	-	-	-	-	-	-	-	-	-	-	-	-	-	-	-	-	-	-	-	+	-	-	-	-	-	-	-	-	-	-	-	-	-	-	-	-
4282	7641	hypothetical protein	2	-	-	-	-	-	-	-	-	-	-	-	-	-	-	-	-	-	-	-	-	-	-	-	-	-	-	-	-	-	-	+	-	-	-	-	-	-	-	-	-	+	-	-	-	-	-	-	-	-	-	-	-	-	-	-	-	-
4283	7642	glycosyltransferase	2	-	-	-	-	-	-	-	-	-	-	-	-	-	-	-	-	-	-	-	-	-	-	-	-	-	-	-	-	-	-	-	-	-	-	-	-	-	-	-	-	+	-	-	-	-	-	-	-	-	-	-	-	+	-	-	-	-
4284	7643	hypothetical protein	2	-	-	-	-	-	-	-	-	-	-	-	-	-	-	-	-	-	-	-	-	-	-	-	-	-	-	-	-	-	-	-	-	-	-	-	-	-	-	-	-	+	-	-	-	-	-	-	-	-	+	-	-	-	-	-	-	-
4285	7644	hypothetical protein	2	-	-	-	-	-	-	-	-	-	-	-	-	-	-	-	-	-	-	-	-	-	-	-	-	-	-	-	-	-	-	-	-	-	-	-	-	-	-	-	-	+	-	-	-	-	-	-	-	-	-	-	-	+	-	-	-	-
4286	7646	Arylsulfatase regulator (Fe-S oxidoreductase)	2	-	-	-	-	-	-	-	-	-	-	-	-	-	-	-	-	-	-	-	-	-	-	-	-	-	-	-	-	-	-	-	-	-	-	-	-	-	-	-	-	+	-	-	-	-	-	+	-	-	-	-	-	-	-	-	-	-
4287	7647	oligosaccharide repeat unit polymerase Wzy	2	-	-	-	-	-	-	-	-	-	-	-	-	-	-	-	-	-	-	-	-	-	-	-	-	-	-	-	-	-	-	-	-	-	-	-	-	-	-	-	-	+	-	-	-	-	-	-	-	-	-	-	-	+	-	-	-	-
4288	7648	Dehydrogenase with different specificities (related to short-chain alcohol dehydrogenase)	2	-	-	-	-	-	-	-	-	-	-	-	-	-	-	-	-	-	-	-	-	-	-	-	-	-	-	-	-	-	-	-	-	-	-	-	-	-	-	-	-	+	-	-	-	-	-	-	-	-	-	-	-	+	-	-	-	-
4289	7649	hypothetical protein	2	-	-	-	-	-	-	-	-	-	-	-	-	-	-	-	-	-	-	-	-	-	-	-	-	-	-	-	-	-	-	-	-	-	-	-	-	-	-	-	-	+	-	-	-	-	-	+	-	-	-	-	-	-	-	-	-	-
4290	7650	hypothetical protein	2	-	-	-	-	-	-	-	-	-	-	-	-	-	-	-	-	-	-	-	-	-	-	-	-	-	-	-	-	-	-	-	-	-	-	-	-	-	-	-	-	+	-	+	-	-	-	-	-	-	-	-	-	-	-	-	-	-
4291	7651	hypothetical protein	2	-	-	-	-	-	-	-	-	-	-	-	-	-	-	-	-	-	-	-	-	-	-	-	-	-	-	-	-	-	-	-	-	-	-	-	-	-	-	-	-	+	-	-	-	-	-	+	-	-	-	-	-	-	-	-	-	-
4292	7652	hypothetical protein	2	-	-	-	-	-	-	-	-	-	-	-	-	-	-	-	-	-	-	-	-	-	-	-	-	-	-	-	-	-	-	-	-	-	-	-	-	-	-	-	-	-	-	-	-	-	-	-	-	-	+	-	-	-	-	-	+	-
4293	7653	Chaperone protein DnaJ	2	-	-	-	-	-	-	-	-	-	-	-	-	-	-	-	-	-	-	-	-	-	-	-	-	-	-	-	-	-	-	-	-	-	-	-	-	-	-	-	-	-	-	-	-	-	-	-	-	-	+	-	-	-	-	-	+	-
4294	7654	hypothetical protein	2	-	-	-	-	-	-	-	-	-	-	-	-	-	-	-	-	-	-	-	-	-	-	-	-	-	-	-	-	-	-	-	-	-	-	-	-	-	-	-	-	-	-	-	-	-	-	-	-	-	+	-	-	+	-	-	-	-
4295	7655	hypothetical protein	2	-	-	-	-	-	-	-	-	-	-	-	-	-	-	-	-	-	-	-	-	-	-	-	-	-	-	-	-	-	-	-	-	-	-	-	-	-	-	-	-	-	-	-	-	-	-	-	-	-	+	-	-	-	+	-	-	-
4296	7659	hypothetical protein	2	-	-	-	-	-	-	-	-	-	-	-	-	-	-	-	-	-	-	-	-	-	-	-	-	-	-	-	-	-	-	+	-	-	-	-	-	-	-	-	-	-	-	-	-	-	-	-	-	-	+	-	-	-	-	-	-	-
4297	7663	hypothetical protein	2	-	-	-	-	-	-	-	-	-	-	-	-	-	-	-	-	-	-	-	-	-	-	-	-	-	-	-	-	-	-	-	-	-	-	-	-	-	-	-	-	-	-	-	-	-	-	-	-	-	+	-	+	-	-	-	-	-
4298	7664	Tn5252	2	-	-	-	-	-	-	-	-	-	-	-	-	-	-	-	-	-	-	-	-	-	-	-	-	-	-	-	-	-	-	-	-	-	-	-	-	-	-	-	-	-	-	-	-	-	-	-	-	-	+	-	-	-	-	-	+	-
4299	7665	Propanediol utilization polyhedral body protein PduJ	2	-	-	-	-	-	-	-	-	-	-	-	-	-	-	-	-	-	-	-	-	-	-	-	-	-	-	-	-	-	-	-	-	-	-	-	-	-	-	-	-	-	-	-	-	-	-	-	-	-	+	-	+	-	-	-	-	-
4300	7666	hypothetical protein	2	-	-	-	-	-	-	-	-	-	-	-	-	-	-	-	-	-	-	-	-	-	-	-	-	-	-	-	-	-	-	-	-	-	-	-	-	-	-	-	-	-	-	-	-	-	-	-	-	-	+	-	-	-	-	-	+	-
4301	7668	hypothetical protein	2	-	-	-	-	-	-	-	-	-	-	-	-	-	-	-	-	-	-	-	-	-	-	-	-	-	-	-	-	-	-	-	-	-	-	-	-	-	-	-	-	-	-	-	-	-	-	-	-	-	+	-	-	-	-	-	+	-
4302	7669	hypothetical protein	2	-	-	-	-	-	-	-	-	-	-	-	-	-	-	-	-	-	-	-	-	-	-	-	+	-	-	-	-	-	-	-	-	-	-	-	-	-	-	-	-	-	-	-	-	-	-	-	-	-	+	-	-	-	-	-	-	-
4303	7670	hypothetical protein	2	-	-	-	-	-	-	-	-	-	-	-	-	-	-	-	-	-	-	-	-	-	-	-	-	-	-	-	-	-	-	-	-	-	-	-	-	-	-	-	-	-	-	-	-	-	-	-	-	-	+	-	+	-	-	-	-	-
4304	7671	streptomycin 3"-adenylyltransferase	2	-	-	-	-	-	-	-	-	-	-	-	-	-	-	-	-	-	-	-	-	-	-	-	-	-	-	-	-	-	-	-	-	-	-	-	-	-	-	-	-	-	-	-	-	-	-	-	-	-	+	-	+	-	-	-	-	-
4305	7672	hypothetical protein	2	-	-	-	-	-	-	-	-	-	-	-	-	-	-	-	-	-	-	-	-	-	-	-	-	-	-	-	-	-	-	-	-	-	-	-	-	-	-	-	-	-	-	-	-	-	-	-	-	-	+	-	+	-	-	-	-	-
4306	7674	hypothetical protein	2	-	-	-	-	-	-	-	-	-	-	-	-	-	-	-	-	-	-	-	-	-	-	-	-	-	-	-	-	-	-	-	-	-	-	-	-	-	-	-	-	-	-	-	-	-	-	-	-	-	+	-	-	-	+	-	-	-
4307	7676	hypothetical protein	2	-	-	-	-	-	-	-	-	-	-	-	-	-	-	-	-	-	-	-	-	-	-	-	-	-	-	-	-	-	-	-	-	-	-	-	-	-	-	-	-	-	-	-	-	-	-	-	-	-	+	+	-	-	-	-	-	-
4308	7677	hypothetical protein	2	-	-	-	-	-	-	-	-	-	-	-	-	-	-	-	-	-	-	-	-	-	-	-	-	-	-	-	-	-	-	-	-	-	-	-	-	-	-	-	-	-	-	-	-	-	-	-	-	-	+	+	-	-	-	-	-	-
4309	7678	hypothetical protein	2	-	-	-	-	-	-	-	-	-	-	-	-	-	-	-	-	-	-	-	-	-	-	-	-	-	-	-	-	-	-	-	-	-	-	-	-	-	-	-	-	-	-	-	-	-	-	-	-	-	+	-	-	-	-	-	+	-
4310	7679	hypothetical protein	2	-	-	-	-	-	-	-	-	-	-	-	-	-	-	-	-	-	-	-	-	-	-	-	-	-	-	-	-	-	-	-	-	-	-	-	-	-	-	-	-	-	-	-	-	-	-	-	-	-	+	-	-	+	-	-	-	-
4311	7680	hypothetical protein	2	-	-	-	-	-	-	-	-	-	-	-	-	-	-	-	-	-	-	-	-	-	-	-	-	-	-	-	-	-	-	-	-	-	-	-	-	-	-	-	-	-	-	-	-	-	-	-	-	-	+	-	-	-	-	-	+	-
4312	7682	hypothetical protein	2	-	-	-	-	-	-	-	-	-	-	-	-	-	-	-	-	-	-	-	-	-	-	-	-	-	-	-	-	-	-	-	-	-	-	-	-	-	-	-	-	-	-	-	-	-	-	-	-	-	+	-	+	-	-	-	-	-
4313	7683	hypothetical protein	2	-	-	-	-	-	-	-	-	-	-	-	-	-	-	-	-	-	-	-	-	-	-	-	-	-	-	-	-	-	-	-	-	-	-	-	-	-	-	-	-	-	-	+	-	-	-	-	-	-	+	-	-	-	-	-	-	-
4314	7684	hypothetical protein	2	-	-	-	-	-	-	-	-	-	-	-	-	-	-	-	-	-	-	-	-	-	-	-	-	-	-	-	-	-	-	-	-	-	-	-	-	-	-	-	-	-	-	-	-	-	-	-	-	-	+	-	+	-	-	-	-	-
4315	7685	hypothetical protein	2	-	-	-	-	-	-	-	-	-	-	-	-	-	-	-	-	-	-	-	-	-	-	-	-	-	-	-	-	-	-	-	-	-	-	-	-	-	-	-	-	-	-	-	-	-	-	-	-	-	+	-	+	-	-	-	-	-
4316	7686	hypothetical protein	2	-	-	-	-	-	-	-	-	-	-	-	-	-	-	-	-	-	-	-	-	-	-	-	-	-	-	-	-	-	-	-	-	-	-	-	-	-	-	-	-	-	-	-	-	-	-	-	-	-	+	-	+	-	-	-	-	-
4317	7687	hypothetical protein	2	-	-	-	-	-	-	-	-	-	-	-	-	-	-	-	-	-	-	-	-	-	-	-	-	-	-	-	-	-	-	-	-	-	-	-	-	-	-	-	-	-	-	-	-	-	-	-	-	-	+	-	-	-	-	-	+	-
4318	7691	hypothetical protein	2	-	-	-	-	-	-	-	-	-	-	-	-	-	-	-	-	-	-	-	-	-	-	-	-	-	-	-	-	-	-	-	-	-	-	-	-	-	-	-	-	-	-	-	-	-	-	-	-	-	+	-	+	-	-	-	-	-
4319	7692	hypothetical protein	2	-	-	-	-	-	-	-	-	-	-	-	-	-	-	-	-	-	-	-	-	-	-	-	-	-	-	-	-	-	-	-	-	-	-	-	-	-	-	-	-	-	-	-	-	-	-	-	-	-	+	-	+	-	-	-	-	-
4320	7694	hypothetical protein	2	-	-	-	-	-	-	-	-	-	-	-	-	-	-	-	-	-	-	-	-	-	-	-	-	-	-	-	-	-	-	-	-	-	-	-	-	-	-	-	-	-	-	-	-	-	-	-	-	-	+	-	-	+	-	-	-	-
4321	7695	hypothetical protein	2	-	-	-	-	-	-	-	-	-	-	-	-	-	-	-	-	-	-	-	-	-	-	-	-	-	-	-	-	-	-	-	-	-	-	-	-	-	-	-	-	-	-	-	-	-	-	-	-	-	+	+	-	-	-	-	-	-
4322	7696	hypothetical protein	2	-	-	-	-	-	-	-	-	-	-	-	-	-	-	-	-	-	-	-	-	-	-	-	-	-	-	-	-	-	-	-	-	-	-	-	-	-	-	-	-	-	-	-	-	-	-	-	-	-	+	-	-	-	-	-	+	-
4323	7697	hypothetical protein	2	-	-	-	-	-	-	-	-	-	-	-	-	-	-	-	-	-	-	-	-	-	-	-	-	-	-	-	-	-	-	-	-	-	-	-	-	-	-	-	-	-	-	-	-	-	-	-	-	-	+	-	+	-	-	-	-	-
4324	7699	hypothetical protein	2	-	-	-	-	-	-	-	-	-	-	-	-	-	-	-	-	-	-	-	-	-	-	-	-	-	-	-	-	-	-	-	-	-	-	-	-	-	-	-	-	-	-	-	-	-	-	-	-	-	+	-	-	+	-	-	-	-
4325	7700	Phage protein	2	-	-	-	-	-	-	-	-	-	-	-	-	-	-	-	-	-	-	-	-	-	-	-	-	-	-	-	-	-	-	-	-	-	-	-	-	-	-	-	-	-	-	-	-	-	-	+	-	-	+	-	-	-	-	-	-	-
4326	7701	hypothetical protein	2	-	-	-	-	-	-	-	-	-	-	-	-	-	-	-	-	-	-	-	-	-	-	-	-	-	-	-	-	-	-	-	-	-	-	-	-	-	-	-	-	-	-	-	-	-	-	-	-	-	+	-	+	-	-	-	-	-
4327	7702	hypothetical protein	2	-	-	-	-	-	-	-	-	-	-	-	-	-	-	-	-	-	-	-	-	-	-	-	-	-	-	-	-	-	-	-	-	-	-	-	-	-	-	-	-	-	-	-	-	-	-	-	-	-	+	-	-	-	+	-	-	-
4328	7703	hypothetical protein	2	-	-	-	-	-	-	-	-	-	-	-	-	-	-	-	-	-	-	-	-	-	-	-	-	-	-	-	-	-	-	-	-	-	-	-	-	-	-	-	-	-	-	-	-	-	-	-	-	-	+	-	-	-	-	+	-	-
4329	7704	Beta-glucosidase (EC 3.2.1.21)	2	-	-	-	-	-	-	-	-	-	-	-	-	-	-	-	-	-	-	-	-	-	-	-	-	-	-	-	-	-	-	-	-	-	-	-	-	-	-	-	-	-	-	-	-	-	-	-	-	-	+	-	-	-	-	+	-	-
4330	7705	Tn5252	2	-	-	-	-	-	-	-	-	-	-	-	-	-	-	-	-	-	-	-	-	-	-	-	-	-	-	-	-	-	-	-	-	-	-	-	-	-	-	-	-	-	-	-	-	-	-	-	-	-	+	-	-	-	-	-	+	-
4331	7708	hypothetical protein	2	-	-	-	-	-	-	-	-	-	-	-	-	-	-	-	-	-	-	-	-	-	-	-	-	-	-	-	-	-	-	+	-	-	-	-	-	-	-	-	-	-	-	-	-	-	-	-	-	-	+	-	-	-	-	-	-	-
4332	7709	calcium-binding protein	2	-	-	-	-	-	-	-	-	-	-	-	-	-	-	-	-	-	-	-	-	-	-	-	-	-	-	-	-	-	-	-	-	-	-	-	-	-	-	-	-	-	-	-	-	-	-	-	-	-	+	-	-	-	-	-	+	-
4333	7710	hypothetical protein	2	-	-	-	-	-	-	-	-	-	-	-	-	-	-	-	-	-	-	-	-	-	-	-	-	-	-	-	-	-	-	-	-	-	-	-	-	-	-	-	-	-	-	-	-	-	-	-	-	-	+	-	+	-	-	-	-	-
4334	7711	hypothetical protein	2	-	-	-	-	-	-	-	-	-	-	-	-	-	-	-	-	-	-	-	-	-	-	-	-	-	-	-	-	-	-	-	-	-	-	-	-	-	-	-	-	-	-	-	-	-	-	-	-	-	+	-	-	-	+	-	-	-
4335	7713	IgA1 protease	2	-	-	-	-	-	-	-	-	-	-	-	-	-	-	-	-	-	-	-	-	-	-	-	-	-	-	-	-	-	-	-	-	-	-	-	-	-	-	-	-	-	-	-	-	-	-	-	-	-	+	-	-	-	+	-	-	-
4336	7714	cell wall surface anchor signal protein	2	-	-	-	-	-	-	-	-	-	-	-	-	-	-	-	-	-	-	-	-	-	-	-	-	-	-	-	-	-	-	-	-	-	-	-	-	-	-	-	-	-	-	-	-	-	-	-	-	-	+	-	-	+	-	-	-	-
4337	7715	hypothetical protein	2	-	-	-	-	-	-	-	-	-	-	-	-	-	-	-	-	-	-	-	-	-	-	-	-	-	-	-	-	-	-	-	-	-	-	-	-	-	-	-	-	-	-	-	-	-	-	-	-	-	+	-	-	-	-	-	+	-
4338	7716	hypothetical protein	2	-	-	-	-	-	-	-	-	-	-	-	-	-	-	-	-	-	-	-	-	-	-	-	-	-	-	-	-	-	-	-	-	-	-	-	-	-	-	-	-	-	-	-	-	-	-	-	-	-	+	-	-	+	-	-	-	-
4339	7718	Cell wall surface anchor family protein	2	-	-	-	-	-	-	-	-	-	-	-	-	-	-	-	-	-	-	-	-	-	-	-	-	-	-	-	-	-	-	-	-	-	-	-	-	-	-	-	-	-	-	-	-	-	-	-	-	-	+	-	+	-	-	-	-	-
4340	7719	hypothetical protein	2	-	-	-	-	-	-	-	-	-	-	-	-	-	-	-	-	-	-	-	-	-	-	-	-	-	-	-	-	-	-	-	-	-	-	-	-	-	-	-	-	-	-	-	-	-	-	-	-	-	+	-	+	-	-	-	-	-
4341	7720	hypothetical protein	2	-	-	-	-	-	-	-	-	-	-	-	-	-	-	-	-	-	-	-	-	-	-	-	-	-	-	-	-	-	-	-	-	-	-	-	-	-	-	-	-	-	-	-	-	-	-	-	-	-	+	-	+	-	-	-	-	-
4342	7722	hypothetical protein	2	-	-	-	-	-	-	-	-	-	-	-	-	-	-	-	-	-	-	-	-	-	-	-	-	-	-	-	-	-	-	-	-	-	-	-	-	-	-	-	-	-	-	+	-	-	-	-	-	-	+	-	-	-	-	-	-	-
4343	7723	hypothetical protein	2	-	-	-	-	-	-	-	-	-	-	-	-	-	-	-	-	-	-	-	-	-	-	-	-	-	-	-	-	-	-	-	-	-	-	-	-	-	-	-	-	-	-	-	-	-	-	-	-	-	+	-	-	-	-	+	-	-
4344	7724	DNA topoisomerase III (EC 5.99.1.2)	2	-	-	-	-	-	-	-	-	-	-	-	-	-	-	-	-	-	-	-	-	-	-	-	-	-	-	-	-	-	-	-	-	-	-	-	-	-	-	-	-	-	-	-	-	-	-	-	-	-	+	-	-	-	-	-	+	-
4345	7725	Transmembrane component CbiQ of energizing module of cobalt ECF transporter	2	-	-	-	-	-	-	-	-	-	-	-	-	-	-	-	-	-	-	-	-	-	-	-	-	-	-	-	-	-	-	-	-	-	-	-	-	-	-	-	-	-	-	-	-	-	-	-	-	-	+	-	+	-	-	-	-	-
4346	7726	hypothetical protein	2	-	-	-	-	-	-	-	-	-	-	-	-	-	-	-	-	-	-	-	-	-	-	-	-	-	-	-	-	-	-	-	-	-	-	-	-	-	-	-	-	-	-	-	-	-	-	+	-	-	+	-	-	-	-	-	-	-
4347	7727	hypothetical protein	2	-	-	-	-	-	-	-	-	-	-	-	-	-	-	-	-	-	-	-	-	-	-	-	-	-	-	-	-	-	-	-	-	-	-	-	-	-	-	-	-	-	-	-	-	-	-	-	-	-	+	-	-	-	+	-	-	-
4348	7728	putative plasmid partition protein	2	-	-	-	-	-	-	-	-	-	-	-	-	-	-	-	-	-	-	-	-	-	-	-	-	-	-	-	-	-	-	-	-	-	-	-	-	-	-	-	-	-	-	-	-	-	-	-	-	-	+	-	-	-	-	-	+	-
4349	7729	hypothetical protein	2	-	-	-	-	-	-	-	-	-	-	-	-	-	-	-	-	-	-	-	-	-	-	-	-	-	-	-	-	-	-	-	-	-	-	-	-	-	-	-	-	-	-	-	-	-	-	-	-	-	+	-	-	+	-	-	-	-
4350	7730	hypothetical protein	2	-	-	-	-	-	-	-	-	-	-	-	-	-	-	-	-	-	-	-	-	-	-	-	-	-	-	-	-	-	-	-	-	-	-	-	-	-	-	-	-	-	-	-	-	-	-	-	-	-	+	-	-	-	-	-	+	-
4351	7731	hypothetical protein	2	-	-	-	-	-	-	-	-	-	-	-	-	-	-	-	-	-	-	-	-	-	-	-	-	-	-	-	-	-	-	-	-	-	-	-	-	-	-	-	-	-	-	-	-	-	-	-	-	-	+	-	-	-	-	-	+	-
4352	7734	hypothetical protein	2	-	-	-	-	-	-	-	-	-	-	-	-	-	-	-	-	-	-	-	-	-	-	-	-	-	-	-	-	-	-	-	-	-	-	-	-	-	-	-	-	-	-	-	-	-	-	-	-	-	+	-	+	-	-	-	-	-
4353	7735	Unknown	2	-	-	-	-	-	-	-	-	-	-	-	-	-	-	-	-	-	-	-	-	-	-	-	-	-	-	-	-	-	-	-	-	-	-	-	-	-	-	-	-	-	-	-	-	-	-	-	-	-	+	-	-	+	-	-	-	-
4354	7737	hypothetical protein	2	-	-	-	-	-	-	-	-	-	-	-	-	-	-	-	-	-	-	-	-	-	-	-	-	-	-	-	-	-	-	-	-	-	-	-	-	-	-	-	-	-	-	-	-	-	-	-	-	-	+	-	-	-	-	-	+	-
4355	7738	BlpS protein	2	-	-	-	-	-	-	-	-	-	-	-	-	-	-	-	-	-	-	-	-	-	-	-	-	-	-	-	-	-	-	-	-	-	-	-	-	-	-	-	-	-	-	-	-	-	-	-	-	-	+	-	-	+	-	-	-	-
4356	7739	IgA1 protease (EC 3.4.24.13)	2	-	-	-	-	-	-	-	-	-	-	-	-	-	-	-	-	-	-	-	-	-	-	-	-	-	-	-	-	-	-	-	-	-	-	-	-	-	-	-	-	-	-	-	-	-	-	-	-	-	+	-	-	-	+	-	-	-
4357	7740	Conserved uncharacterized protein	2	-	-	-	-	-	-	-	-	-	-	-	-	-	-	-	-	-	-	-	-	-	-	-	-	-	-	-	-	-	-	-	-	-	-	-	-	-	-	-	-	-	-	-	-	-	-	-	-	-	+	-	+	-	-	-	-	-
4358	7741	hypothetical protein	2	-	-	-	-	-	-	-	-	-	-	-	-	-	-	-	-	-	-	-	-	-	-	-	-	-	-	-	-	-	-	-	-	-	-	-	-	-	-	-	-	-	-	-	-	+	-	-	-	-	+	-	-	-	-	-	-	-
4359	7743	Sialidase (EC 3.2.1.18)	2	-	-	-	-	-	-	-	-	-	-	-	-	-	-	-	-	-	-	-	-	-	-	-	-	-	-	-	-	-	-	-	-	-	-	-	-	-	-	-	-	-	-	-	-	-	-	-	-	-	+	-	+	-	-	-	-	-
4360	7745	hypothetical protein	2	-	-	-	-	-	-	-	-	-	-	-	-	-	-	-	-	-	-	-	-	-	-	-	-	-	-	-	-	-	-	-	-	-	-	-	-	-	-	-	-	-	-	-	-	-	-	-	-	-	+	-	-	-	-	-	+	-
4361	7747	hypothetical protein	2	-	-	-	-	-	-	-	-	-	-	-	-	-	-	-	-	-	-	-	-	-	-	-	-	-	-	-	-	-	-	-	-	-	-	-	-	-	-	-	-	-	-	-	-	-	-	-	-	-	+	-	-	-	-	-	+	-
4362	7748	hypothetical protein	2	-	-	-	-	-	-	-	-	-	-	-	-	-	-	-	-	-	-	-	-	-	-	-	-	-	-	-	-	-	-	-	-	-	-	-	-	-	-	-	-	-	-	+	-	-	-	-	-	-	+	-	-	-	-	-	-	-
4363	7749	Fibroin 1	2	-	-	-	-	-	-	-	-	-	-	-	-	+	-	+	-	-	-	-	-	-	-	-	-	-	-	-	-	-	-	-	-	-	-	-	-	-	-	-	-	-	-	-	-	-	-	-	-	-	-	-	-	-	-	-	-	-
4364	7750	Carbamoyl-phosphate synthase small chain (EC 6.3.5.5)	2	-	-	-	-	-	-	-	-	-	-	-	-	+	-	-	-	-	+	-	-	-	-	-	-	-	-	-	-	-	-	-	-	-	-	-	-	-	-	-	-	-	-	-	-	-	-	-	-	-	-	-	-	-	-	-	-	-
4365	7753	Mevalonate kinase (EC 2.7.1.36)	2	-	-	-	-	-	-	-	-	-	-	-	-	+	-	-	-	-	-	-	-	-	-	-	-	-	-	-	-	-	-	-	-	-	-	-	-	-	-	-	-	-	-	-	-	-	-	-	-	-	-	-	-	-	-	-	+	-
4366	7754	hypothetical protein	2	-	-	-	-	-	-	-	-	-	-	-	-	-	-	+	-	-	-	-	-	-	-	-	-	-	-	-	-	-	-	-	-	-	-	-	-	-	-	-	-	-	-	-	-	+	-	-	-	-	-	-	-	-	-	-	-	-
4367	7758	hypothetical protein	2	-	-	-	-	-	-	-	-	-	-	-	-	-	-	+	-	-	-	-	-	-	-	-	-	-	-	-	-	-	-	-	-	-	-	-	-	-	-	-	-	-	-	-	-	+	-	-	-	-	-	-	-	-	-	-	-	-
4368	7759	Phage integrase	2	-	-	-	-	-	-	-	-	+	-	-	-	-	-	+	-	-	-	-	-	-	-	-	-	-	-	-	-	-	-	-	-	-	-	-	-	-	-	-	-	-	-	-	-	-	-	-	-	-	-	-	-	-	-	-	-	-
4369	7760	hypothetical protein	2	-	-	-	-	-	-	-	-	-	-	-	-	-	-	+	-	-	-	-	-	-	-	-	-	-	-	-	-	-	-	-	-	-	-	-	-	-	-	-	-	-	-	-	+	-	-	-	-	-	-	-	-	-	-	-	-	-
4370	7761	Phage major capsid protein	2	-	-	-	-	-	-	-	-	-	-	-	-	-	-	+	-	-	-	-	-	-	-	-	-	-	-	-	-	-	-	-	-	-	-	-	-	-	-	-	-	-	-	-	-	-	-	+	-	-	-	-	-	-	-	-	-	-
4371	7764	hypothetical protein	2	-	-	-	-	-	-	-	-	-	-	-	-	-	-	+	-	-	-	+	-	-	-	-	-	-	-	-	-	-	-	-	-	-	-	-	-	-	-	-	-	-	-	-	-	-	-	-	-	-	-	-	-	-	-	-	-	-
4372	7766	similar to phage-related terminase small subunit	2	-	-	-	-	-	-	-	-	-	-	-	-	-	-	+	-	-	-	-	-	-	-	-	-	-	-	-	-	-	-	-	-	-	-	-	-	-	-	-	-	-	-	-	-	-	-	+	-	-	-	-	-	-	-	-	-	-
4373	7772	ABC transporter	2	-	-	-	-	-	-	-	-	-	-	-	-	-	-	+	-	-	-	-	-	-	-	-	-	-	-	-	-	-	-	-	-	-	-	-	-	-	-	-	-	-	-	-	-	+	-	-	-	-	-	-	-	-	-	-	-	-
4374	7773	hypothetical protein	2	-	-	-	-	-	-	-	-	-	-	-	-	-	-	-	-	-	-	-	-	-	-	-	-	-	-	-	-	-	-	-	-	-	-	-	-	-	-	-	-	-	-	-	-	+	-	-	-	+	-	-	-	-	-	-	-	-
4375	7774	Putative EsaC protein analog (Listeria type 3)	2	-	-	-	-	-	-	-	-	-	-	-	-	-	-	-	-	-	-	-	-	-	-	-	-	-	-	-	-	-	-	-	-	-	-	-	-	-	-	-	-	-	-	+	-	+	-	-	-	-	-	-	-	-	-	-	-	-
4376	7775	FIG004453: protein YceG like	2	-	-	-	-	-	-	-	-	-	-	-	-	-	-	-	-	-	-	-	-	-	-	-	-	-	-	-	-	-	-	-	-	-	-	-	-	-	-	-	-	-	-	-	-	+	-	-	-	+	-	-	-	-	-	-	-	-
4377	7778	hypothetical protein	2	-	-	-	-	-	-	-	-	-	-	-	-	-	-	-	-	-	-	-	-	-	-	-	-	-	-	-	-	-	-	-	-	-	-	-	-	-	-	-	-	-	-	-	-	+	-	-	-	-	-	-	-	-	+	-	-	-
4378	7781	Mobile element protein	2	-	-	-	-	-	-	-	-	-	-	-	-	-	-	-	-	-	-	-	-	-	-	-	-	-	-	-	-	-	-	-	-	-	-	-	-	-	-	-	-	-	-	-	-	+	-	-	-	+	-	-	-	-	-	-	-	-
4379	7785	hypothetical protein	2	-	-	-	-	-	-	-	-	-	-	-	-	-	-	-	-	-	-	-	-	-	-	-	-	-	-	-	-	-	-	-	-	-	-	-	-	-	-	-	-	-	-	+	-	+	-	-	-	-	-	-	-	-	-	-	-	-
4380	7788	hypothetical protein	2	-	-	-	-	-	-	-	-	-	-	-	-	-	-	-	-	-	-	-	-	-	-	-	-	-	-	-	-	-	-	-	-	-	-	-	-	-	-	-	-	-	-	-	-	+	-	-	-	-	-	-	-	-	-	+	-	-
4381	7789	hypothetical protein	2	-	-	-	-	-	-	-	-	-	-	-	-	-	-	-	-	-	-	-	-	-	-	-	-	-	-	-	-	-	-	-	-	-	-	-	-	-	-	-	-	-	-	-	-	+	-	+	-	-	-	-	-	-	-	-	-	-
4382	7792	transcriptional regulator PlcR, putative	2	-	-	-	-	-	-	-	-	-	-	-	-	-	-	-	-	-	-	-	-	-	-	-	-	-	-	-	-	-	-	-	-	-	-	-	-	-	-	-	-	-	-	-	-	+	-	-	-	-	-	-	-	+	-	-	-	-
4383	7794	hypothetical protein	2	-	-	-	-	-	-	-	-	-	-	-	-	-	-	-	-	-	-	-	-	-	-	-	-	-	-	-	-	-	-	-	-	-	-	-	-	-	-	-	-	-	-	+	-	+	-	-	-	-	-	-	-	-	-	-	-	-
4384	7795	hypothetical protein	2	-	-	-	-	-	-	-	-	-	-	-	-	-	-	-	-	-	-	-	-	-	-	-	-	-	-	-	-	-	-	-	-	-	-	-	-	-	-	-	-	-	-	-	-	+	-	-	-	-	-	-	-	-	-	+	-	-
4385	7796	hypothetical protein	2	-	-	-	-	-	-	-	-	-	-	-	-	-	-	-	-	-	-	-	-	-	-	-	-	-	-	-	-	-	-	-	-	-	-	-	-	-	-	-	-	-	-	+	-	+	-	-	-	-	-	-	-	-	-	-	-	-
4386	7797	hypothetical protein	2	-	-	-	-	-	-	-	-	-	-	-	-	-	-	-	-	-	-	-	-	-	-	-	-	-	-	-	-	-	-	-	-	-	-	-	-	-	-	-	-	-	-	-	-	+	-	-	-	-	-	-	+	-	-	-	-	-
4387	7798	hypothetical protein	2	-	-	-	-	-	-	-	-	-	-	-	-	-	-	-	-	-	-	-	-	-	-	-	-	-	-	-	-	-	-	-	+	-	+	-	-	-	-	-	-	-	-	-	-	-	-	-	-	-	-	-	-	-
4388	7799	hypothetical protein	2	-	-	-	-	-	-	-	-	-	-	-	-	-	-	-	-	-	-	-	-	-	-	-	-	-	-	-	-	-	-	-	+	+	-	-	-	-	-	-	-	-	-	-	-	-	-	-	-	-	-	-	-	-
4389	7800	hypothetical protein	2	-	-	-	-	-	-	-	-	-	-	-	-	-	-	-	-	-	-	-	-	-	-	-	-	-	-	-	-	-	-	-	+	+	-	-	-	-	-	-	-	-	-	-	-	-	-	-	-	-	-	-	-	-
4390	7801	Phage Terminase Small Subunit	2	-	-	-	-	-	-	-	-	-	-	-	-	-	-	-	-	-	-	-	-	-	-	-	-	-	-	-	-	-	-	-	+	+	-	-	-	-	-	-	-	-	-	-	-	-	-	-	-	-	-	-	-	-
4391	7802	FIG042921: similarity to aminoacyl-tRNA editing enzymes YbaK	2	-	-	-	-	-	-	-	-	-	-	-	-	-	-	-	-	-	-	-	-	-	-	-	-	-	-	-	-	-	-	-	+	+	-	-	-	-	-	-	-	-	-	-	-	-	-	-	-	-	-	-	-	-
4392	7803	Beta-1	2	-	-	-	-	-	-	-	-	-	-	-	-	-	-	-	-	-	-	-	-	-	-	-	-	-	-	-	-	-	-	-	+	+	-	-	-	-	-	-	-	-	-	-	-	-	-	-	-	-	-	-	-	-
4393	7804	hypothetical protein	2	-	-	-	-	-	-	-	-	-	-	-	-	-	-	-	-	-	-	-	-	-	-	-	-	-	-	-	-	-	-	-	+	+	-	-	-	-	-	-	-	-	-	-	-	-	-	-	-	-	-	-	-	-
4394	7805	AAA superfamily ATPase	2	-	-	-	-	-	-	-	-	-	-	-	-	-	-	-	-	-	-	-	-	-	-	-	-	-	-	-	-	-	-	-	+	+	-	-	-	-	-	-	-	-	-	-	-	-	-	-	-	-	-	-	-	-
4395	7806	hypothetical protein	2	-	-	-	-	-	-	-	-	-	-	-	-	-	-	-	-	-	-	-	-	-	-	-	-	-	-	-	-	-	-	-	+	+	-	-	-	-	-	-	-	-	-	-	-	-	-	-	-	-	-	-	-	-
4396	7807	hypothetical protein	2	-	-	-	-	-	-	-	-	-	-	-	-	-	-	-	-	-	-	-	-	-	-	-	-	-	-	-	-	-	-	-	+	+	-	-	-	-	-	-	-	-	-	-	-	-	-	-	-	-	-	-	-	-
4397	7808	hypothetical protein	2	-	-	-	-	-	-	-	-	-	-	-	-	-	-	-	-	-	-	-	-	-	-	-	-	-	-	-	-	-	-	-	+	+	-	-	-	-	-	-	-	-	-	-	-	-	-	-	-	-	-	-	-	-
4398	7809	D-alanine--D-alanine ligase A (EC 6.3.2.4) (D-alanylalanine synthetase A) (D-Ala-D-Ala ligase A)	2	-	-	-	-	-	-	-	-	-	-	-	-	-	-	-	-	-	-	-	-	-	-	-	-	-	-	-	-	-	-	-	+	-	+	-	-	-	-	-	-	-	-	-	-	-	-	-	-	-	-	-	-	-
4399	7810	Glutathione S-transferase	2	-	-	-	-	-	-	-	-	-	-	-	-	-	-	-	-	-	-	-	-	-	-	-	-	-	-	-	-	-	-	-	+	-	+	-	-	-	-	-	-	-	-	-	-	-	-	-	-	-	-	-	-	-
4400	7811	Phage transcriptional regulator	2	-	-	-	-	-	-	-	-	-	-	-	-	-	-	-	-	-	-	-	-	-	-	-	-	-	-	-	-	-	-	-	+	+	-	-	-	-	-	-	-	-	-	-	-	-	-	-	-	-	-	-	-	-
4401	7812	hypothetical protein	2	-	-	-	-	-	-	-	-	-	-	-	-	-	-	-	-	-	-	-	-	-	-	-	-	-	-	-	-	-	-	-	+	+	-	-	-	-	-	-	-	-	-	-	-	-	-	-	-	-	-	-	-	-
4402	7813	hypothetical protein	2	-	-	-	-	-	-	-	-	-	-	-	-	-	-	-	-	-	-	-	-	-	-	-	-	-	-	-	-	-	-	-	+	+	-	-	-	-	-	-	-	-	-	-	-	-	-	-	-	-	-	-	-	-
4403	7814	hypothetical protein	2	-	-	-	-	-	-	-	-	-	-	-	-	-	-	-	-	-	-	-	-	-	-	-	-	-	-	-	-	-	-	-	+	-	+	-	-	-	-	-	-	-	-	-	-	-	-	-	-	-	-	-	-	-
4404	7815	NAD(P)HX dehydratase	2	-	-	-	-	-	-	-	-	-	-	-	-	-	-	-	-	-	-	-	-	-	-	-	-	-	-	-	-	-	-	-	+	+	-	-	-	-	-	-	-	-	-	-	-	-	-	-	-	-	-	-	-	-
4405	7816	L-fucose operon activator	2	-	-	-	-	-	-	-	-	-	-	-	-	-	-	-	-	-	-	-	-	-	-	-	-	-	-	-	-	-	-	-	+	-	+	-	-	-	-	-	-	-	-	-	-	-	-	-	-	-	-	-	-	-
4406	7817	hypothetical protein	2	-	-	-	-	-	-	-	-	-	-	-	-	-	-	-	-	-	-	-	-	-	-	-	-	-	-	-	-	-	-	-	+	+	-	-	-	-	-	-	-	-	-	-	-	-	-	-	-	-	-	-	-	-
4407	7818	hypothetical protein	2	-	-	-	-	-	-	-	-	-	-	-	-	-	-	-	-	-	-	-	-	-	-	-	-	-	-	-	-	-	-	-	+	-	-	-	-	-	-	-	-	-	-	-	-	-	-	-	-	-	+	-	-	-
4408	7819	hypothetical protein	2	-	-	-	-	-	-	-	-	-	-	-	-	-	-	-	-	-	-	-	-	-	-	-	-	-	-	-	-	-	-	-	+	+	-	-	-	-	-	-	-	-	-	-	-	-	-	-	-	-	-	-	-	-
4409	7820	hypothetical protein	2	-	-	-	-	-	-	-	-	-	-	-	-	-	-	-	-	-	-	-	-	-	-	-	-	-	-	-	-	-	-	-	+	+	-	-	-	-	-	-	-	-	-	-	-	-	-	-	-	-	-	-	-	-
4410	7821	Phage tail length tape-measure protein	2	-	-	-	-	-	-	-	-	+	-	-	-	-	-	-	-	-	-	-	-	-	-	-	-	-	-	-	-	-	-	-	+	-	-	-	-	-	-	-	-	-	-	-	-	-	-	-	-	-	-	-	-	-
4411	7822	putative serine protease	2	-	-	-	-	-	-	-	-	-	-	-	-	-	-	-	-	-	-	-	-	-	-	-	-	-	-	-	-	-	-	-	+	+	-	-	-	-	-	-	-	-	-	-	-	-	-	-	-	-	-	-	-	-
4412	7823	phage protein	2	-	-	-	-	-	-	-	-	-	-	-	-	-	-	-	-	-	-	-	-	-	-	-	-	-	-	-	-	-	-	-	+	+	-	-	-	-	-	-	-	-	-	-	-	-	-	-	-	-	-	-	-	-
4413	7825	Xaa-His dipeptidase (EC 3.4.13.3)	2	-	-	-	-	-	-	-	-	-	-	-	-	-	-	-	-	-	-	-	-	-	-	-	-	-	-	-	-	-	-	-	+	-	+	-	-	-	-	-	-	-	-	-	-	-	-	-	-	-	-	-	-	-
4414	7826	hypothetical protein	2	-	-	-	-	-	-	-	-	-	-	-	-	-	-	-	-	-	-	-	-	-	-	-	-	-	-	-	-	-	-	-	+	+	-	-	-	-	-	-	-	-	-	-	-	-	-	-	-	-	-	-	-	-
4415	7828	Phage capsid protein	2	-	-	-	-	-	-	-	-	-	-	-	-	-	-	-	-	-	-	-	-	-	-	-	-	-	-	-	-	-	-	-	+	+	-	-	-	-	-	-	-	-	-	-	-	-	-	-	-	-	-	-	-	-
4416	7829	hypothetical protein	2	-	-	-	-	-	-	-	-	-	-	-	-	-	-	-	-	-	-	-	-	-	-	-	-	-	-	-	-	-	-	-	+	+	-	-	-	-	-	-	-	-	-	-	-	-	-	-	-	-	-	-	-	-
4417	7830	hypothetical protein	2	-	-	-	-	-	-	-	-	-	-	-	-	-	-	-	-	-	-	-	-	-	-	-	-	-	-	-	-	-	-	-	+	+	-	-	-	-	-	-	-	-	-	-	-	-	-	-	-	-	-	-	-	-
4418	7831	hypothetical protein	2	-	-	-	-	-	-	-	-	-	-	-	-	-	-	-	-	-	-	-	-	-	-	-	-	-	-	-	-	-	-	-	+	+	-	-	-	-	-	-	-	-	-	-	-	-	-	-	-	-	-	-	-	-
4419	7832	hypothetical protein	2	-	-	-	-	-	-	-	-	-	-	-	-	-	-	-	-	-	-	-	-	-	-	-	-	-	-	-	-	-	-	-	+	-	+	-	-	-	-	-	-	-	-	-	-	-	-	-	-	-	-	-	-	-
4420	7833	hypothetical protein	2	-	-	-	-	-	-	-	-	-	-	-	-	-	-	-	-	-	-	-	-	-	-	-	-	-	-	-	-	-	-	-	+	+	-	-	-	-	-	-	-	-	-	-	-	-	-	-	-	-	-	-	-	-
4421	7834	L-fucose operon activator	2	-	-	-	-	-	-	-	-	-	-	-	-	-	-	-	-	-	-	-	-	-	-	-	-	-	-	-	-	-	+	+	-	-	-	-	-	-	-	-	-	-	-	-	-	-	-	-	-	-	-	-	-	-
4422	7835	hypothetical protein	2	-	-	-	-	-	-	-	-	-	-	-	-	-	-	-	-	-	-	-	-	-	-	-	-	-	-	-	-	-	-	-	+	-	-	-	-	-	-	-	-	-	-	-	-	-	-	-	-	-	+	-	-	-
4423	7836	hypothetical protein	2	-	-	-	-	-	-	-	-	-	-	-	-	-	-	-	-	-	-	-	-	-	-	-	-	-	-	-	-	-	-	-	+	+	-	-	-	-	-	-	-	-	-	-	-	-	-	-	-	-	-	-	-	-
4424	7837	hypothetical protein	2	-	-	-	-	-	-	-	-	-	-	-	-	-	-	-	-	-	-	-	-	-	-	-	-	-	-	-	-	-	-	-	+	+	-	-	-	-	-	-	-	-	-	-	-	-	-	-	-	-	-	-	-	-
4425	7838	hypothetical protein	2	-	-	-	-	-	-	-	-	-	-	-	-	-	-	-	-	-	-	-	-	-	-	-	-	-	-	-	-	-	-	-	+	-	-	-	-	-	-	-	-	-	-	-	-	-	-	-	-	-	-	+	-	-
4426	7839	hypothetical protein	2	-	-	-	-	-	-	-	-	-	-	-	-	-	-	-	-	-	-	-	-	-	-	-	-	-	-	-	-	-	-	-	+	+	-	-	-	-	-	-	-	-	-	-	-	-	-	-	-	-	-	-	-	-
4427	7840	hypothetical protein	2	-	-	-	-	-	-	-	-	-	-	-	-	-	-	-	-	-	-	-	-	-	-	-	-	-	-	-	-	-	-	-	+	+	-	-	-	-	-	-	-	-	-	-	-	-	-	-	-	-	-	-	-	-
4428	7841	hypothetical protein	2	-	-	-	-	-	-	-	-	-	-	-	-	-	-	-	-	-	-	-	-	-	-	-	-	-	-	-	-	-	-	-	+	+	-	-	-	-	-	-	-	-	-	-	-	-	-	-	-	-	-	-	-	-
4429	7842	hypothetical protein	2	-	-	-	-	-	-	-	-	-	-	-	-	-	-	-	-	-	-	-	-	-	-	-	-	-	-	-	-	-	-	-	+	-	-	-	-	-	-	-	-	-	-	-	-	-	-	-	-	-	+	-	-	-
4430	7843	hypothetical protein	2	-	-	-	-	-	-	-	-	-	-	-	-	-	-	-	-	-	-	-	-	-	-	-	-	-	-	-	-	-	-	-	+	+	-	-	-	-	-	-	-	-	-	-	-	-	-	-	-	-	-	-	-	-
4431	7844	SNF2 family protein	2	-	-	-	-	-	-	-	-	-	-	-	-	-	-	-	-	-	-	-	-	-	-	-	-	-	-	-	-	-	-	-	+	+	-	-	-	-	-	-	-	-	-	-	-	-	-	-	-	-	-	-	-	-
4432	7845	hypothetical protein	2	-	-	-	-	-	-	-	-	-	-	-	-	-	-	-	-	-	-	-	-	-	-	-	-	-	-	-	-	-	-	+	+	-	-	-	-	-	-	-	-	-	-	-	-	-	-	-	-	-	-	-	-	-
4433	7846	hypothetical protein	2	-	-	-	-	-	-	-	-	-	-	-	-	-	-	-	-	-	-	-	-	-	-	-	-	-	-	-	-	-	-	-	+	+	-	-	-	-	-	-	-	-	-	-	-	-	-	-	-	-	-	-	-	-
4434	7847	hypothetical protein	2	-	-	-	-	-	-	-	-	-	-	-	-	-	-	-	-	-	-	-	-	-	-	-	-	-	-	-	-	-	-	-	+	+	-	-	-	-	-	-	-	-	-	-	-	-	-	-	-	-	-	-	-	-
4435	7848	hypothetical protein	2	-	-	-	-	-	-	-	-	-	-	-	-	-	-	-	-	-	-	-	-	-	-	-	-	-	-	-	-	-	-	-	-	-	+	-	-	-	-	-	-	-	-	-	-	-	-	-	+	-	-	-	-	-
4436	7849	Thiol peroxidase	2	-	-	-	-	-	-	-	-	-	-	-	-	-	-	-	-	-	-	-	-	-	-	-	-	-	-	-	-	-	-	-	-	-	-	-	-	-	-	-	-	-	-	-	-	-	-	+	-	-	-	+	-	-
4437	7851	Transcriptional regulator	2	-	-	-	-	-	-	-	-	-	-	-	-	-	-	-	-	-	-	-	-	-	-	-	-	-	-	-	-	-	-	-	-	-	+	-	-	-	-	-	-	-	-	-	-	-	-	-	+	-	-	-	-	-
4438	7852	hypothetical protein	2	-	-	-	-	-	-	-	-	-	-	-	-	-	-	-	-	-	-	-	-	-	-	-	-	-	-	-	-	-	-	-	-	+	+	-	-	-	-	-	-	-	-	-	-	-	-	-	-	-	-	-	-	-
4439	7855	hypothetical protein	2	-	-	-	-	-	-	-	-	-	-	-	-	-	-	-	-	-	-	-	-	-	-	-	-	-	-	-	-	-	-	-	-	+	+	-	-	-	-	-	-	-	-	-	-	-	-	-	-	-	-	-	-	-
4440	7857	hypothetical protein	2	-	-	-	-	-	-	-	-	-	-	-	-	-	-	-	-	-	-	-	-	-	-	-	-	-	-	-	-	-	-	-	-	+	+	-	-	-	-	-	-	-	-	-	-	-	-	-	-	-	-	-	-	-
4441	7858	hypothetical protein	2	-	-	-	-	-	-	-	-	-	-	-	-	-	-	-	-	-	-	-	-	-	-	-	-	-	-	-	-	-	-	-	-	-	+	-	-	-	-	-	-	-	-	-	-	-	-	-	-	-	+	-	-	-
4442	7859	glycosyltransferase	2	-	-	-	-	-	-	-	-	-	-	-	-	-	-	-	-	-	-	-	-	-	-	-	-	-	-	-	-	-	-	-	-	-	+	-	-	-	-	-	-	-	-	-	-	-	-	-	-	+	-	-	-	-
4443	7862	hypothetical protein	2	-	-	-	-	-	-	-	-	-	-	-	-	-	-	-	-	-	-	-	-	-	-	-	-	-	-	-	-	-	-	-	-	-	+	-	-	-	-	-	-	-	-	-	-	-	-	+	-	-	-	-	-	-
4444	7866	Exopolysaccharide biosynthesis protein related to N-acetylglucosamine-1-phosphodiester alpha-N-acetylglucosaminidase	2	-	-	-	-	-	-	-	-	-	-	-	-	-	-	-	-	-	-	-	-	-	-	-	-	-	-	-	+	-	-	-	-	-	-	-	-	-	+	-	-	-	-	-	-	-	-	-	-	-	-	-	-	-	-	-	-	-
4445	7867	hypothetical protein	2	-	-	-	-	-	-	-	-	-	-	-	-	-	-	-	-	-	-	-	-	-	-	-	-	-	-	-	-	-	-	-	-	-	+	-	-	-	-	-	-	-	-	-	-	-	-	-	+	-	-	-	-	-
4446	7868	hypothetical protein	2	-	-	-	-	-	-	-	-	-	-	-	-	-	-	-	-	-	-	-	-	-	-	-	-	-	-	-	-	-	-	-	-	-	+	-	-	-	-	-	-	-	-	-	-	-	-	-	-	+	-	-	-	-
4447	7869	Site-specific recombinase	2	-	-	-	-	-	-	-	-	-	-	-	-	-	-	-	-	-	-	-	-	-	-	-	-	-	-	-	-	-	-	-	-	-	+	-	-	-	-	-	-	-	-	-	-	-	-	-	-	-	+	-	-	-
4448	7870	Surface protein from Gram-positive cocci	2	-	-	-	-	-	-	-	-	-	-	-	-	-	-	-	-	-	-	-	-	-	-	-	-	-	-	-	-	-	-	-	-	-	+	-	-	-	-	-	-	-	-	-	-	-	-	-	-	-	+	-	-	-

Table S5. Precence/absence of each HG among 57 *S. suis* strains

				1																13			25					28						104		94			76			53/54		Divergent *S. suis*																
Index[a]	ClusterID	Gene products	Members	98HAH33	05ZYH33	SC84	A7	SS12	P1/7	DAT273	NIAH11434	JS14	GZ1	BM407	S735	13730	MNCM07	NCTC 10446	14636	NCTC 10237	ST1	2651	89-1591	MNCM04	MNCM25	8074	D9	4961	ST3	MNCM43	DAT292	DAT260	NIAH11435	MNCM21	MNCM50	89-2479	DAT234	DAT290	93A	42A	NT77	6407	11538	8830	12814	92-1400	89-590	2726	2524	89-3576-3	4417	D12	10581	89-5259	88-5299A	R61	92-1191	14A	92-4172	22083
4449	7875	Predicted hydrolase (HAD superfamily)	2	-	-	-	-	-	-	-	-	-	-	-	-	-	-	-	-	-	-	-	-	-	-	-	-	-	-	-	+	-	-	-	-	-	-	-	-	+	-	-	-	-	-	-	-	-	-	-	-	-	-	-	-	-	-	-	-	-
4450	7876	hypothetical protein	2	-	-	-	-	-	-	-	-	-	-	-	-	-	-	-	-	-	-	-	-	-	-	-	-	-	-	-	-	-	-	-	-	-	-	-	-	+	-	-	-	-	-	-	-	-	-	-	-	-	-	+	-	-	-	-	-	-
4451	7877	hypothetical protein	2	-	-	-	-	-	-	-	-	-	-	-	-	-	-	-	-	-	-	-	-	-	-	-	-	-	-	-	-	-	-	-	-	-	-	-	-	+	-	-	-	-	-	-	-	-	-	-	-	-	-	-	-	-	+	-	-	-
4452	7879	Substrate-specific component FolT of folate ECF transporter	2	-	-	-	-	-	-	-	-	-	-	-	-	-	-	-	-	-	-	-	-	-	-	-	-	-	-	-	-	-	-	-	-	-	-	-	-	+	-	-	-	-	-	-	-	-	-	-	-	-	-	-	-	-	+	-	-	-
4453	7881	hypothetical protein	2	-	-	-	-	-	-	-	-	-	-	-	-	-	-	-	-	-	-	-	-	-	-	-	-	-	-	-	-	-	-	-	-	-	-	-	-	+	-	-	-	-	-	-	-	-	-	-	-	-	-	-	-	-	+	-	-	-
4454	7882	hypothetical protein	2	-	-	-	-	-	-	-	-	-	-	-	-	-	-	-	-	-	-	-	-	-	-	-	-	-	-	-	-	-	-	-	-	-	-	-	-	+	-	-	-	-	-	-	-	-	-	-	-	-	-	-	-	-	+	-	-	-
4455	7945	hypothetical protein	2	-	-	-	-	-	-	-	-	-	-	-	-	-	-	-	-	-	-	-	-	-	-	-	-	-	-	-	-	-	-	-	-	-	-	-	-	-	-	-	-	-	-	-	-	-	-	-	-	-	-	+	-	-	-	+	-	-
4456	7946	Putative glycosyltransferase	2	-	-	-	-	-	-	-	-	-	-	-	-	-	-	-	-	-	-	-	-	-	-	-	-	-	-	-	-	-	-	-	-	-	-	-	-	-	-	-	-	-	-	+	-	-	-	-	-	-	-	-	-	-	-	+	-	-
4457	7947	hypothetical protein	2	-	-	-	-	-	-	-	-	-	-	-	-	-	-	-	-	-	-	-	-	-	-	-	-	-	-	-	-	-	-	-	-	-	-	-	-	-	-	-	-	-	-	-	-	-	-	+	-	-	-	-	-	-	-	+	-	-
4458	7948	Glycosyl transferase	2	-	-	-	-	-	-	-	-	-	-	-	-	-	-	-	-	-	-	-	-	-	-	-	-	-	-	-	-	-	-	-	-	-	-	-	-	-	-	-	-	-	-	-	-	-	-	-	-	-	-	-	-	-	+	+	-	-
4459	7950	hypothetical protein	2	-	-	-	-	-	-	-	-	-	-	-	-	-	-	-	-	-	-	-	-	-	-	-	-	-	-	-	-	-	-	-	-	-	-	-	-	-	-	-	-	-	-	-	-	-	-	-	-	-	-	+	-	-	-	+	-	-
4460	7951	hypothetical protein	2	-	-	-	-	+	-	-	-	-	-	-	-	-	-	-	-	-	-	-	-	-	-	-	-	-	-	-	-	-	-	-	-	-	-	-	-	-	-	-	-	-	-	-	-	-	-	-	-	-	-	-	-	-	-	+	-	-
4461	7952	hypothetical protein	2	-	-	-	-	-	-	-	-	-	-	-	-	-	-	-	-	-	-	-	-	-	-	-	-	-	-	-	-	-	-	-	-	-	-	-	-	-	-	-	-	-	-	-	-	-	-	-	-	-	-	-	+	-	-	+	-	-
4462	7955	Glycosyltransferase (EC 2.4.1.-)	2	-	-	-	-	-	-	-	-	-	-	-	-	-	-	-	-	-	-	-	-	-	-	-	-	-	-	-	-	-	-	-	-	-	-	-	-	-	-	-	-	-	-	-	-	-	-	-	-	-	-	-	-	-	+	+	-	-
4463	7956	hypothetical protein	2	-	-	-	-	-	-	-	-	-	-	-	-	-	-	-	-	-	-	-	-	-	-	-	-	-	-	-	-	-	-	-	-	-	-	-	-	-	-	-	-	-	-	-	-	-	-	-	-	-	-	-	+	-	-	+	-	-
4464	7957	hypothetical protein	2	-	-	-	-	-	-	-	-	-	-	-	-	-	-	-	-	-	-	-	-	-	-	-	-	-	-	-	-	-	-	-	-	-	-	-	-	-	-	-	-	-	-	-	-	-	-	-	-	-	-	-	-	-	+	+	-	-
4465	7958	hypothetical protein	2	-	-	-	-	-	-	-	-	-	-	-	-	-	-	-	-	-	-	-	-	-	-	-	-	-	-	-	-	-	-	-	-	-	-	-	-	-	-	-	-	-	-	-	-	-	-	-	-	-	-	-	-	-	+	+	-	-
4466	7959	hypothetical protein	2	-	-	-	-	-	-	-	-	-	-	-	-	-	-	-	-	-	-	-	-	-	-	-	-	-	-	-	-	-	-	-	-	-	-	-	-	-	-	-	-	-	-	-	-	-	-	-	-	-	-	-	-	-	+	+	-	-
4467	7960	hypothetical protein	2	-	-	-	-	-	-	-	-	-	-	-	-	-	-	-	-	-	-	-	-	-	-	-	-	-	-	-	-	-	-	-	-	-	-	-	-	-	-	-	-	-	-	-	-	-	-	-	-	-	-	-	-	+	-	+	-	-
4468	7961	hypothetical protein	2	-	-	-	-	-	-	-	-	-	-	-	-	-	-	-	-	-	-	-	-	-	-	-	-	-	-	-	-	-	-	-	-	-	-	-	-	-	-	-	-	-	-	-	-	-	-	-	-	-	-	-	-	+	-	+	-	-
4469	7962	Prophage Clp protease-like protein	2	-	-	-	-	-	-	-	-	-	-	-	-	-	-	-	-	-	-	+	-	-	-	-	-	-	-	-	-	-	-	-	-	-	-	-	-	-	-	-	-	-	-	-	-	-	-	-	-	-	-	-	-	-	-	+	-	-
4470	7964	hypothetical protein	2	-	-	-	-	-	-	-	-	-	-	-	-	-	-	-	-	-	-	-	-	-	-	-	-	-	-	-	-	-	-	-	-	-	-	-	-	-	-	-	-	-	-	-	-	-	-	-	-	-	-	-	-	-	+	+	-	-
4471	7965	hypothetical protein	2	-	-	-	-	-	-	-	-	-	-	-	-	-	-	-	-	-	-	-	-	-	-	-	-	-	-	-	-	-	-	-	-	-	-	-	-	-	-	-	-	-	-	-	-	-	-	-	-	-	-	+	-	-	-	+	-	-
4472	7966	hypothetical protein	2	-	-	-	-	-	-	-	-	-	-	-	-	-	-	-	-	-	-	-	-	-	-	-	-	-	-	-	-	-	-	-	-	-	-	-	-	-	-	-	-	-	-	-	-	-	-	-	-	-	-	-	-	-	+	+	-	-
4473	7967	hypothetical protein	2	-	-	-	-	-	-	-	-	-	-	-	-	-	-	-	-	-	-	-	-	-	-	-	-	-	-	-	-	-	-	-	-	-	-	-	-	-	-	-	-	-	-	-	-	-	-	-	-	-	-	-	-	-	+	+	-	-
4474	7968	hypothetical protein	2	-	-	-	-	-	-	-	-	-	-	-	-	-	-	-	-	-	-	-	-	-	-	-	-	-	-	-	-	-	-	-	-	-	-	-	-	-	-	-	-	-	-	+	-	-	-	-	-	-	-	-	-	-	-	+	-	-
4475	7969	hypothetical protein	2	-	-	-	-	-	-	-	-	-	-	-	-	-	-	-	-	-	-	-	-	-	-	-	-	-	-	-	-	-	-	-	-	-	-	-	-	-	-	-	-	-	-	-	-	-	-	-	-	-	-	-	-	-	+	+	-	-
4476	7970	FtsK/SpoIIIE family protein	2	-	-	-	-	-	-	-	-	-	-	-	-	-	-	-	-	-	-	-	-	-	-	-	-	-	-	-	-	-	-	-	-	-	-	-	-	-	-	-	-	-	-	-	-	-	-	-	-	-	-	+	-	-	-	+	-	-
4477	7971	hypothetical protein	2	-	-	-	-	-	-	-	-	-	-	-	-	-	-	-	-	-	-	-	-	-	-	-	-	-	-	-	-	-	-	-	-	-	-	-	-	-	-	-	-	-	-	-	-	-	-	-	-	-	-	-	-	-	+	+	-	-
4478	7973	hypothetical protein	2	-	-	-	-	-	-	-	-	-	-	-	-	-	-	-	-	-	-	+	-	-	-	-	-	-	-	-	-	-	-	-	-	-	-	-	-	-	-	-	-	-	-	-	-	-	-	-	-	-	-	-	-	-	-	+	-	-
4479	7974	hypothetical protein	2	-	-	-	-	-	-	-	-	-	-	-	-	-	-	-	-	-	-	+	-	-	-	-	-	-	-	-	-	-	-	-	-	-	-	-	-	-	-	-	-	-	-	-	-	-	-	-	-	-	-	-	-	-	-	+	-	-
4480	7975	GTPase and tRNA-U34 5-formylation enzyme TrmE	2	-	-	-	-	-	-	-	-	-	-	-	-	-	-	-	-	-	-	-	-	-	-	-	-	-	-	-	-	-	-	-	-	-	-	-	-	-	-	-	-	-	-	-	-	-	-	-	-	-	-	-	+	-	-	+	-	-
4481	7976	hypothetical protein	2	-	-	-	-	-	-	-	-	-	-	-	-	-	-	-	-	-	-	-	-	-	-	-	-	-	-	-	-	-	-	-	-	-	-	-	-	-	-	-	-	-	-	-	-	-	-	-	-	-	-	-	-	+	-	+	-	-
4482	7977	hypothetical protein	2	-	-	-	-	-	-	-	-	-	-	-	-	-	-	-	-	-	-	-	-	-	-	-	-	-	-	-	-	-	-	-	-	-	-	-	-	-	-	-	-	-	-	-	-	-	-	-	-	-	-	-	-	-	+	+	-	-
4483	7978	hypothetical protein	2	-	-	-	-	-	-	-	-	-	-	-	-	-	-	-	-	-	-	-	-	-	-	-	-	-	-	-	-	-	-	-	-	-	-	-	-	-	-	-	-	-	-	-	-	-	-	-	-	-	-	-	-	-	+	+	-	-
4484	7979	hypothetical protein	2	-	-	-	-	-	-	-	-	-	-	-	-	-	-	-	-	-	-	-	-	-	-	-	-	-	-	-	-	-	-	-	-	-	-	-	-	-	-	-	-	-	-	-	-	-	-	-	-	-	-	-	+	-	-	+	-	-
4485	7980	hypothetical protein	2	-	-	-	-	-	-	-	-	-	-	-	-	-	-	-	-	-	-	-	-	-	-	-	-	-	-	-	-	-	-	-	-	-	-	-	-	-	-	-	-	-	-	-	-	-	-	-	-	-	-	-	-	-	+	+	-	-
4486	8030	hypothetical protein	2	-	-	-	-	-	-	-	-	-	-	-	-	-	-	-	-	-	-	-	-	-	-	-	-	-	-	-	-	-	-	-	-	+	-	+	-	-	-	-	-	-	-	-	-	-	-	-	-	-	-	-	-	-	-	-	-	-
4487	8031	hypothetical protein	2	-	-	-	-	-	-	-	-	-	-	-	-	-	-	-	-	-	-	-	-	-	-	-	-	-	-	-	-	-	-	-	-	+	-	-	-	-	-	-	-	-	-	-	+	-	-	-	-	-	-	-	-	-	-	-	-	-
4488	8032	hypothetical protein	2	-	-	-	-	-	-	-	-	-	-	-	-	-	-	-	-	-	-	-	-	-	-	-	-	-	-	-	-	-	-	-	-	+	-	+	-	-	-	-	-	-	-	-	-	-	-	-	-	-	-	-	-	-	-	-	-	-
4489	8035	hypothetical protein	2	-	-	-	-	-	-	-	-	-	-	-	-	-	-	-	-	-	-	-	-	-	-	-	-	-	-	-	-	-	-	-	-	+	-	-	-	-	-	-	-	-	-	-	-	-	-	-	-	-	-	+	-	-	-	-	-	-
4490	8036	Serine endopeptidase SepC (EC 3.4.21.-)	2	-	-	-	-	-	-	-	-	-	-	-	-	-	-	-	-	-	-	-	-	-	-	-	-	-	-	-	-	-	-	-	-	+	-	+	-	-	-	-	-	-	-	-	-	-	-	-	-	-	-	-	-	-	-	-	-	-
4491	8038	hypothetical protein	2	-	-	-	-	-	-	-	-	-	-	-	-	-	-	-	-	-	-	-	-	+	-	-	-	-	-	-	-	-	-	-	-	+	-	-	-	-	-	-	-	-	-	-	-	-	-	-	-	-	-	-	-	-	-	-	-	-
4492	8040	hypothetical protein	2	-	-	-	-	-	-	-	-	-	-	-	-	-	-	-	-	-	-	-	-	-	-	-	-	-	-	-	-	-	-	-	-	+	+	-	-	-	-	-	-	-	-	-	-	-	-	-	-	-	-	-	-	-	-	-	-	-
4493	8043	hypothetical protein	2	-	-	-	-	-	-	-	-	-	-	-	-	-	-	-	-	-	-	-	-	-	-	-	-	-	-	-	-	-	-	-	-	+	-	+	-	-	-	-	-	-	-	-	-	-	-	-	-	-	-	-	-	-	-	-	-	-
4494	8048	hypothetical protein	2	-	-	-	-	-	-	-	-	-	-	-	-	-	-	-	-	-	-	-	-	-	-	-	-	-	-	-	-	-	-	-	-	-	-	-	-	-	-	-	-	-	-	-	-	-	-	-	-	-	-	-	+	+	-	-	-	-
4495	8050	hypothetical protein	2	-	-	-	+	-	-	-	-	-	-	-	-	-	-	-	-	-	-	-	-	-	-	-	-	-	-	-	-	-	-	-	-	-	-	-	-	-	-	-	-	-	-	-	-	-	-	-	-	-	-	-	+	-	-	-	-	-
4496	8052	ABC transporter	2	-	-	-	-	-	-	-	-	-	-	-	-	-	-	-	-	-	-	-	-	-	-	-	-	-	-	-	-	-	-	-	-	-	-	-	-	-	-	-	-	-	-	-	-	-	-	-	-	-	-	+	+	-	-	-	-	-
4497	8054	hypothetical protein	2	-	-	-	-	-	-	-	-	-	-	-	-	-	-	-	-	-	-	-	-	-	-	-	-	-	-	-	-	-	-	-	-	-	-	-	-	-	-	-	-	-	-	-	-	-	-	-	-	-	-	-	+	-	+	-	-	-
4498	8055	hypothetical protein	2	-	-	-	-	-	-	-	-	-	-	-	-	-	-	-	-	-	-	-	-	-	-	-	-	-	-	-	-	-	-	-	-	-	-	-	-	-	-	-	-	-	-	-	-	-	-	-	-	-	-	-	+	+	-	-	-	-
4499	8056	Peptidase S8 and S53	2	-	-	-	-	-	-	-	-	-	-	-	-	-	-	-	-	-	-	-	-	-	-	-	-	-	-	-	-	-	-	-	-	-	-	-	-	-	-	-	-	-	-	-	-	-	-	-	-	-	-	+	+	-	-	-	-	-
4500	8057	hypothetical protein	2	-	-	-	-	-	-	-	-	-	-	-	-	-	-	-	-	-	-	+	-	-	-	-	-	-	-	-	-	-	-	-	-	-	-	-	-	-	-	-	-	-	-	-	-	-	-	-	-	-	-	-	+	-	-	-	-	-
4501	8058	hypothetical protein	2	-	-	-	-	-	-	-	-	-	-	-	-	-	-	-	-	-	-	-	-	-	-	-	-	-	-	-	-	-	-	-	-	-	-	-	-	-	-	-	-	-	-	-	-	-	-	-	-	-	-	-	+	+	-	-	-	-
4502	8059	Transposase	2	-	-	-	-	-	-	-	-	-	-	-	-	-	-	-	-	-	-	-	-	-	-	-	-	-	-	-	-	-	-	-	-	-	-	-	-	-	-	-	-	-	-	-	-	-	-	-	-	-	-	+	+	-	-	-	-	-
4503	8060	hypothetical protein	2	-	-	-	-	-	-	-	-	-	-	-	-	-	-	-	-	-	-	-	-	-	-	-	-	-	-	-	-	-	-	-	-	-	-	-	-	-	-	-	-	-	-	-	-	-	-	-	-	-	-	-	+	+	-	-	-	-
4504	8061	hypothetical protein	2	-	-	-	-	-	-	-	-	-	-	-	-	-	-	-	-	-	-	-	-	-	-	-	-	-	-	-	-	-	-	-	-	-	-	-	-	-	-	-	-	-	-	-	-	-	-	-	-	-	-	-	+	+	-	-	-	-
4505	8062	hypothetical protein	2	-	-	-	-	-	-	-	-	-	-	-	-	-	-	-	-	-	-	-	-	-	-	-	-	-	-	-	-	-	-	-	-	-	-	-	-	-	-	-	-	-	-	-	-	-	-	-	-	-	-	-	+	+	-	-	-	-
4506	8063	hypothetical protein	2	-	-	-	-	-	-	-	-	-	-	-	-	-	-	-	-	-	-	-	-	-	-	-	-	-	-	-	-	-	-	-	-	-	-	-	-	-	-	-	-	-	-	-	-	-	-	-	-	-	-	-	+	+	-	-	-	-
4507	8064	hypothetical protein	2	-	-	-	-	-	-	-	-	-	-	-	-	-	-	-	-	-	-	+	-	-	-	-	-	-	-	-	-	-	-	-	-	-	-	-	-	-	-	-	-	-	-	-	-	-	-	-	-	-	-	-	+	-	-	-	-	-
4508	8066	hypothetical protein	2	-	-	-	-	-	-	-	-	-	-	-	-	-	-	-	-	-	-	-	-	-	-	-	-	-	-	-	-	-	-	-	-	-	-	-	-	-	-	-	-	-	-	-	-	-	-	-	-	-	-	-	+	+	-	-	-	-
4509	8067	hypothetical protein	2	-	-	-	-	-	-	-	-	-	-	-	-	-	-	-	-	-	-	-	-	-	-	-	-	-	-	-	-	-	-	-	-	-	-	-	-	-	-	-	-	-	-	-	-	-	-	-	-	-	-	+	+	-	-	-	-	-
4510	8070	hypothetical protein	2	-	-	-	-	-	-	-	-	-	-	-	-	-	-	-	-	-	-	-	-	-	-	-	-	-	-	-	-	-	-	-	-	-	-	-	-	-	-	-	-	-	-	-	-	-	-	+	-	+	-	-	-	-	-	-	-	-
4511	8071	hypothetical protein	2	-	-	-	-	-	-	-	-	-	-	-	-	-	-	-	-	-	-	-	-	-	-	-	-	-	-	-	-	-	-	-	-	-	-	-	-	-	-	-	-	-	-	-	-	-	-	+	-	+	-	-	-	-	-	-	-	-
4512	8072	hypothetical protein	2	-	-	-	-	-	-	-	-	-	-	-	-	-	-	-	-	-	-	-	-	-	-	-	-	-	-	-	-	-	-	-	-	-	-	-	-	-	-	-	-	-	-	-	-	-	-	+	-	-	-	-	-	-	-	-	+	-
4513	8073	hypothetical protein	2	-	-	-	-	-	-	-	-	-	-	-	-	-	-	-	-	-	-	-	-	-	-	-	-	-	-	-	-	-	-	-	-	-	-	-	-	-	-	-	-	-	-	-	-	-	-	+	-	+	-	-	-	-	-	-	-	-
4514	8074	hypothetical protein	2	-	-	-	-	-	-	-	-	-	-	-	-	-	-	-	-	-	-	-	-	-	-	-	-	-	-	-	-	-	-	-	-	-	-	-	-	-	-	-	-	-	-	-	-	-	-	+	-	+	-	-	-	-	-	-	-	-
4515	8076	hypothetical protein	2	-	-	-	-	-	-	-	-	-	-	-	-	-	-	-	-	-	-	-	-	-	-	-	-	-	-	-	-	-	-	-	-	-	-	-	-	-	-	-	-	-	-	-	-	-	-	+	-	+	-	-	-	-	-	-	-	-
4516	8077	hypothetical protein	2	-	-	-	-	-	-	-	-	-	-	-	-	-	-	-	-	-	-	-	-	-	-	-	-	-	-	-	-	-	-	-	-	-	-	-	-	-	-	-	+	-	-	+	-	-	-	-	-	-	-	-	-	-
4517	8101	hypothetical protein	2	-	-	-	-	-	-	-	-	-	-	-	-	-	-	-	-	-	-	-	-	-	-	-	-	-	-	-	-	-	-	-	-	-	-	-	-	-	-	-	-	-	-	-	-	-	-	-	-	-	-	+	-	+	-	-	-	-
4518	8105	hypothetical protein	2	-	-	-	-	-	-	-	-	-	-	-	-	-	-	-	-	-	-	-	-	-	-	-	-	-	-	-	-	-	-	-	-	-	-	-	-	-	-	-	-	-	-	-	-	-	-	-	-	-	-	+	-	+	-	-	-	-
4519	8106	rhamnose-containing polysacharide translocation permease	2	-	-	-	-	-	-	-	-	-	-	-	-	-	-	-	-	-	-	-	-	-	-	-	-	-	-	-	-	-	-	-	-	-	-	-	-	-	-	-	-	-	-	-	-	-	-	-	-	-	-	+	-	+	-	-	-	-
4520	8107	hypothetical protein	2	-	-	-	-	-	-	-	-	-	-	-	-	-	-	-	-	-	-	-	-	-	-	-	-	-	-	-	-	-	-	-	-	-	-	-	-	-	-	-	-	-	-	-	-	-	-	-	-	-	-	+	-	+	-	-	-	-
4521	8109	hypothetical protein	2	-	-	-	-	-	-	-	-	-	-	-	-	-	-	-	-	-	-	-	-	-	-	-	-	-	-	-	-	-	-	-	-	-	-	-	-	-	-	-	-	-	-	-	-	-	-	-	-	-	-	+	-	+	-	-	-	-
4522	8110	hypothetical protein	2	-	-	-	-	-	-	-	-	-	-	-	-	-	-	-	-	-	-	-	-	-	-	-	-	-	-	-	-	-	-	-	-	-	-	-	-	-	-	-	-	-	-	-	-	-	-	-	-	+	-	+	-	-	-	-	-	-
4523	8111	hypothetical protein	2	-	-	-	-	-	-	-	-	-	-	-	-	-	-	-	-	-	-	-	-	-	-	-	-	-	-	-	-	-	-	-	-	-	-	-	-	-	-	-	-	-	-	-	-	-	-	-	-	-	-	+	-	-	+	-	-	-
4524	8112	hypothetical protein	2	-	-	-	-	-	-	-	-	-	-	-	-	-	-	-	-	-	-	-	-	-	-	-	-	-	-	-	-	-	-	-	-	-	-	-	-	-	-	-	-	-	-	-	-	-	-	-	-	-	-	+	-	+	-	-	-	-
4525	8113	hypothetical protein	2	-	-	-	-	-	-	-	-	-	-	-	-	-	-	-	-	-	-	-	-	-	-	-	-	-	-	-	-	-	-	-	-	-	-	-	-	-	-	-	-	-	-	-	-	-	-	-	-	-	-	+	-	-	+	-	-	-
4526	8115	Surface antigen	2	-	-	-	-	-	-	-	-	-	-	-	-	-	-	-	-	-	-	-	-	-	-	-	-	-	-	-	-	-	-	-	-	-	-	-	-	-	-	-	-	-	-	-	-	-	-	-	-	-	-	+	-	+	-	-	-	-
4527	8116	hypothetical protein	2	-	-	-	-	-	-	-	-	-	-	-	-	-	-	-	-	-	-	-	-	-	-	-	-	-	-	-	-	-	-	-	-	-	-	-	-	-	-	-	-	-	-	-	-	-	-	-	-	-	-	+	-	+	-	-	-	-
4528	8117	hypothetical protein	2	-	-	-	-	-	-	-	-	-	-	-	-	-	-	-	-	-	-	-	-	-	-	-	-	-	-	-	-	-	-	-	-	-	-	-	-	-	-	-	-	-	-	-	-	-	-	-	-	-	-	+	-	-	+	-	-	-
4529	8118	unnamed protein product	2	-	-	-	-	-	-	-	-	-	-	-	-	-	-	-	-	-	-	-	-	-	-	-	-	-	-	-	-	-	-	-	-	-	-	-	-	-	-	-	-	-	-	-	-	-	-	-	-	-	-	+	-	+	-	-	-	-
4530	8119	hypothetical protein	2	-	-	-	-	-	-	-	-	-	-	-	-	-	-	-	-	-	-	-	-	-	-	-	-	-	-	-	-	-	-	-	-	-	-	-	-	-	-	-	-	-	-	-	-	-	-	-	-	-	-	+	-	+	-	-	-	-
4531	8120	hypothetical protein	2	-	-	-	-	-	-	-	-	-	-	-	-	-	-	-	-	-	-	-	-	-	-	-	-	-	-	-	-	-	-	-	-	-	-	-	-	-	-	-	-	-	-	-	-	-	-	-	-	-	-	+	-	+	-	-	-	-
4532	8129	hypothetical protein	2	-	-	-	-	-	-	-	-	-	-	-	-	-	-	-	-	-	-	-	-	-	-	-	-	-	-	-	-	-	-	-	-	-	-	-	-	-	-	-	-	-	-	-	-	-	-	-	-	-	-	+	-	-	+	-	-	-
4533	8131	phage structural protein	2	-	-	-	-	-	-	-	-	-	-	-	-	-	-	-	-	-	-	-	-	-	-	-	-	-	-	-	-	-	-	-	-	-	-	-	-	-	-	-	-	-	-	-	-	-	-	-	-	-	-	+	-	+	-	-	-	-
4534	8133	Unknown, predicted membrane protein (TMS3)	2	-	-	-	-	-	-	-	-	-	-	-	-	-	-	-	-	-	-	-	-	-	-	-	-	-	-	-	-	-	-	-	-	-	-	-	-	-	-	-	-	-	-	-	-	-	-	-	-	-	-	+	-	+	-	-	-	-
4535	8136	Predicted lactoylglutathione lyase	2	-	-	-	-	-	-	-	-	-	-	-	-	-	-	-	-	-	-	-	-	-	-	-	-	-	-	-	-	-	-	-	-	-	-	-	-	-	-	-	-	-	-	-	-	-	-	-	-	-	-	+	-	-	+	-	-	-
4536	8137	hypothetical protein	2	-	-	-	-	-	-	-	+	-	-	-	-	-	-	-	-	-	-	-	-	-	-	-	-	-	-	-	-	-	-	-	-	-	-	-	-	-	-	-	-	-	-	-	-	-	-	-	-	-	-	+	-	-	-	-	-	-
4537	8139	hypothetical protein	2	-	-	-	-	-	-	-	-	-	-	-	-	-	-	-	-	-	-	-	-	-	-	-	-	-	-	-	-	-	-	-	-	-	-	-	-	-	-	-	-	-	-	-	-	-	-	-	-	-	-	+	-	+	-	-	-	-
4538	8140	hypothetical protein	2	-	-	-	-	-	-	-	-	-	-	-	-	-	-	-	-	-	-	-	-	-	-	-	-	-	-	-	-	-	-	-	-	-	-	-	-	-	-	-	-	-	-	-	-	-	-	-	-	-	-	+	-	+	-	-	-	-
4539	8141	N-acetylmuramoyl-L-alanine amidase (EC 3.5.1.28)	2	-	-	-	-	-	-	-	-	-	-	-	-	-	-	-	-	-	-	-	-	-	-	-	-	-	-	-	-	-	-	-	-	-	-	-	-	-	-	-	-	-	-	-	-	-	-	-	-	-	-	+	-	+	-	-	-	-
4540	8142	hypothetical protein	2	-	-	-	-	-	-	-	+	-	-	-	-	-	-	-	-	-	-	-	-	-	-	-	-	-	-	-	-	-	-	-	-	-	-	-	-	-	-	-	-	-	-	-	-	-	-	-	-	-	-	+	-	-	-	-	-	-
4541	8143	Transcriptional regulator	2	-	-	-	-	-	-	-	-	-	-	-	-	-	-	-	-	-	-	-	-	-	-	-	-	-	-	-	-	-	-	-	-	-	-	-	-	-	-	-	-	-	-	-	-	-	-	-	-	-	-	+	-	-	+	-	-	-
4542	8144	hypothetical protein	2	-	-	-	-	-	-	-	-	-	-	-	-	-	-	-	-	-	-	-	-	-	-	-	-	-	-	-	-	-	-	-	-	-	-	-	-	-	-	-	-	-	-	-	-	-	-	-	-	-	-	+	-	+	-	-	-	-
4543	8145	hypothetical protein	2	-	-	-	-	-	-	-	-	-	-	-	-	-	-	-	-	-	-	-	-	-	-	-	-	-	-	-	-	-	-	-	-	-	-	-	-	-	-	-	-	-	-	-	-	-	-	-	-	-	-	+	-	+	-	-	-	-
4544	8146	Predicted hydrolase (HAD superfamily)	2	-	-	-	-	-	-	-	-	-	-	-	-	-	-	-	-	-	-	-	-	-	-	-	-	-	-	-	-	-	-	-	-	-	-	-	-	-	-	-	-	-	-	-	-	-	-	-	-	-	-	+	-	+	-	-	-	-
4545	8147	hypothetical protein	2	-	-	-	-	-	-	-	-	-	-	-	-	-	-	-	-	-	-	-	-	-	-	-	-	-	-	-	-	-	-	-	-	-	-	-	-	-	-	-	-	-	-	-	-	-	-	-	-	-	-	+	-	+	-	-	-	-
4546	8149	hypothetical protein	2	-	-	-	-	-	-	-	-	-	-	-	-	-	-	-	-	-	-	-	-	-	-	-	-	-	-	-	-	-	-	-	-	-	-	-	-	-	-	-	-	-	-	-	-	-	-	-	-	-	-	+	-	+	-	-	-	-
4547	8150	membrane protein, related to Actinobacillus protein (1944168)	2	-	-	-	-	-	-	-	-	-	-	-	-	-	-	-	-	-	-	-	-	-	-	-	-	-	-	-	-	-	-	-	-	-	-	-	-	-	-	-	-	-	-	-	-	-	-	-	-	-	-	+	-	+	-	-	-	-
4548	8151	hypothetical protein	2	-	-	-	-	-	-	-	-	-	-	-	-	-	-	-	-	-	-	-	-	-	-	-	-	-	-	-	-	-	-	-	-	-	-	-	-	-	-	-	-	-	-	-	-	-	-	-	-	+	-	+	-	-	-	-	-	-
4549	8157	hypothetical protein	2	-	-	-	-	-	-	-	-	-	-	-	-	-	-	-	-	-	-	-	-	-	-	-	-	-	-	-	-	-	-	-	-	-	-	-	-	-	-	-	-	-	-	-	-	-	-	-	-	-	-	+	-	+	-	-	-	-
4550	8158	hypothetical protein	2	-	-	-	-	-	-	-	-	-	-	-	-	-	-	-	-	-	-	-	-	-	-	-	-	-	-	-	-	-	-	-	-	-	-	-	-	-	-	-	-	-	-	-	-	-	-	-	-	-	-	+	-	+	-	-	-	-
4551	8159	Thiol-disulfide isomerase and thioredoxin	2	-	-	-	-	-	-	-	-	-	-	-	-	-	-	-	-	-	-	-	-	-	-	-	-	-	-	-	-	-	-	-	-	-	-	-	-	-	-	-	-	-	-	-	-	-	-	-	-	-	-	+	-	+	-	-	-	-
4552	8160	Type II restriction-modification system restriction subunit(EC:3.1.24.1)	2	-	-	-	-	-	-	-	-	-	-	-	-	-	-	-	-	-	-	-	-	-	-	-	-	-	-	-	-	-	-	-	-	-	-	-	-	-	-	-	-	-	-	-	-	-	-	-	-	-	-	+	-	-	+	-	-	-
4553	8161	hypothetical protein	2	-	-	-	-	-	-	-	-	-	-	-	-	-	-	-	-	-	-	-	-	-	-	-	-	-	-	-	-	-	-	-	-	-	-	-	-	-	-	-	-	-	-	-	-	-	-	-	-	-	-	+	-	+	-	-	-	-
4554	8162	hypothetical protein	2	-	-	-	-	-	-	-	-	-	-	-	-	-	-	-	-	-	-	-	-	-	-	-	-	-	-	-	-	-	-	-	-	-	-	-	-	-	-	-	-	-	-	-	-	-	-	-	-	-	-	+	-	+	-	-	-	-
4555	8165	hypothetical protein	2	-	-	-	-	-	-	-	-	-	-	-	-	-	-	-	-	-	-	-	-	-	-	-	-	-	-	-	-	-	-	-	-	-	-	-	-	-	-	-	-	-	-	-	-	-	-	-	-	+	-	+	-	-	-	-	-	-
4556	8166	transposase	2	-	-	-	-	-	-	-	+	-	-	-	-	-	-	-	-	-	-	-	-	-	-	-	-	-	-	-	-	-	-	-	-	-	-	-	-	-	-	-	-	-	-	-	-	-	-	-	-	-	-	+	-	-	-	-	-	-
4557	8167	hypothetical protein	2	-	-	-	-	-	-	-	-	-	-	-	-	-	-	-	-	-	-	-	-	-	-	-	-	-	-	-	-	-	-	-	-	-	-	-	-	-	-	-	-	-	-	-	-	-	-	-	-	-	-	+	-	+	-	-	-	-
4558	8170	hypothetical protein	2	-	-	-	-	-	-	-	-	-	-	-	-	-	-	-	-	-	-	-	-	-	-	-	-	-	-	-	-	-	-	-	-	-	-	-	-	-	-	-	-	-	-	-	-	-	-	-	-	-	-	+	-	-	+	-	-	-
4559	8171	Type II restriction-modification system restriction subunit	2	-	-	-	-	-	-	-	-	-	-	-	-	-	-	-	-	-	-	-	-	-	-	-	-	-	-	-	-	-	-	-	-	-	-	-	-	-	-	-	-	-	-	-	-	-	-	-	-	-	-	+	-	-	+	-	-	-
4560	8172	hypothetical protein	2	-	-	-	-	-	-	-	+	-	-	-	-	-	-	-	-	-	-	-	-	-	-	-	-	-	-	-	-	-	-	-	-	-	-	-	-	-	-	-	-	-	-	-	-	-	-	-	-	-	-	+	-	-	-	-	-	-
4561	8173	hypothetical protein	2	-	-	-	-	-	-	-	-	-	-	-	-	-	-	-	-	-	-	-	-	-	-	-	-	-	-	-	-	-	-	-	-	-	-	-	-	-	-	-	-	-	-	-	-	-	-	-	-	-	-	+	-	-	+	-	-	-
4562	8174	hypothetical protein	2	-	-	-	-	-	-	-	-	-	-	-	-	-	-	-	-	-	-	-	-	-	-	-	-	-	-	-	-	-	-	-	-	-	-	-	-	-	-	-	+	-	-	-	-	-	-	-	-	-	-	-	+	-
4563	8175	hypothetical protein	2	-	-	-	-	-	-	-	-	-	-	-	-	-	-	-	-	-	-	-	-	-	-	-	-	-	-	-	-	-	-	-	-	-	-	-	-	-	-	-	+	-	-	-	-	-	-	-	-	-	-	-	+	-
4564	8177	hypothetical protein	2	-	-	-	-	-	-	-	-	-	-	-	-	-	-	-	-	-	-	-	-	-	-	-	-	-	-	-	-	-	-	-	-	-	-	-	-	-	-	-	+	-	-	-	-	-	-	-	-	-	-	-	+	-
4565	8178	hypothetical protein	2	-	-	-	-	-	-	-	-	-	-	-	-	-	-	-	-	-	-	-	-	-	-	-	-	-	-	-	-	-	-	-	-	-	-	-	-	-	-	-	+	-	-	-	-	-	-	-	-	-	-	-	+	-
4566	8179	hypothetical protein	2	-	-	-	-	-	-	-	-	-	-	-	-	-	-	-	-	-	-	-	-	-	-	-	-	-	-	-	-	-	-	-	-	-	-	-	-	-	-	+	+	-	-	-	-	-	-	-	-	-	-	-	-	-
4567	8180	N-acetylmuramoyl-L-alanine amidase	2	-	-	-	-	-	-	-	-	-	-	-	-	-	-	-	-	-	-	-	-	-	-	-	-	-	-	-	-	-	-	-	-	-	-	-	-	-	-	-	+	-	-	-	-	-	-	-	-	-	-	-	+	-
4568	8181	hypothetical protein	2	-	-	-	-	-	-	-	-	-	-	-	-	-	-	-	-	-	-	-	-	-	-	-	-	-	-	-	-	-	-	-	-	-	-	-	-	-	-	-	+	-	-	-	-	+	-	-	-	-	-	-	-	-
4569	8182	hypothetical protein	2	-	-	-	-	-	-	-	-	-	-	-	-	-	-	-	-	-	-	-	-	-	-	-	-	-	-	-	-	-	-	-	-	-	-	-	-	-	-	-	+	-	-	-	-	-	-	-	-	-	-	-	+	-
4570	8183	hypothetical protein	2	-	-	-	-	-	-	-	-	-	-	-	-	-	-	-	-	-	-	-	-	-	-	-	-	-	-	-	-	-	-	-	-	-	-	-	-	-	-	+	+	-	-	-	-	-	-	-	-	-	-	-	-	-
4571	8184	conserved domain protein	2	-	-	-	-	-	-	-	-	-	-	-	-	-	-	-	-	-	-	-	-	-	-	-	-	-	-	-	-	-	-	-	-	-	-	-	-	-	-	-	+	-	-	-	-	-	-	-	-	-	-	-	+	-
4572	8185	hypothetical protein	2	-	-	-	-	-	-	-	-	-	-	-	-	-	-	-	-	-	-	-	-	-	-	-	-	-	-	-	-	-	-	-	-	-	-	-	-	-	-	-	+	-	-	-	-	-	-	-	-	-	-	-	+	-
4573	8186	hypothetical protein	2	-	-	-	-	-	-	-	-	-	-	-	-	-	-	-	-	-	-	-	-	-	-	-	-	-	-	-	-	-	-	-	-	-	-	-	-	-	-	-	+	-	-	-	-	-	-	-	-	-	-	-	+	-
4574	8187	hypothetical protein	2	-	-	-	-	-	-	-	-	-	-	-	-	-	-	-	-	-	-	-	-	-	-	-	-	-	-	-	-	-	-	-	-	-	-	-	-	-	-	+	+	-	-	-	-	-	-	-	-	-	-	-	-	-
4575	8188	hypothetical protein	2	-	-	-	-	-	-	-	-	-	-	-	-	-	-	-	-	-	-	-	-	-	-	-	-	-	-	-	-	-	-	-	-	-	-	-	-	-	-	-	+	-	-	-	-	-	-	-	-	-	-	-	+	-
4576	8189	surface protein	2	-	-	-	-	-	-	-	-	-	-	-	-	-	-	-	-	-	-	-	-	-	-	-	-	-	-	-	-	-	-	-	-	-	-	-	-	-	-	-	+	-	-	-	-	-	-	-	-	-	-	-	+	-
4577	8191	hypothetical protein	2	-	-	-	-	-	-	-	-	-	-	-	-	-	-	-	-	-	-	-	-	-	-	-	-	-	-	-	-	-	-	-	-	-	-	-	-	-	-	-	+	-	-	-	-	-	-	-	-	-	-	-	+	-
4578	8192	hypothetical protein	2	-	-	-	-	-	-	-	-	-	-	-	-	-	-	-	-	-	-	-	-	-	-	-	-	-	-	-	-	-	-	-	-	-	-	-	-	-	-	-	+	-	-	-	-	-	-	-	-	-	-	-	+	-
4579	8193	hypothetical protein	2	-	-	-	-	-	-	-	-	-	-	-	-	-	-	-	-	-	-	-	-	-	-	-	-	-	-	-	-	-	-	-	-	-	-	-	-	-	-	-	+	-	-	-	-	-	-	-	-	-	-	-	+	-
4580	8195	hypothetical protein	2	-	-	-	-	-	-	-	-	-	-	-	-	-	-	-	-	-	-	-	-	-	-	-	-	-	-	-	-	-	-	-	-	-	-	-	-	-	-	-	+	-	-	-	-	-	-	-	-	-	-	-	+	-
4581	8196	hypothetical protein	2	-	-	-	-	-	-	-	-	-	-	-	-	-	-	-	-	-	-	-	-	-	-	-	-	-	-	-	-	-	-	-	-	-	-	-	-	-	-	-	+	-	-	-	-	-	-	-	-	-	-	-	+	-
4582	8198	hypothetical protein	2	-	-	-	-	-	-	-	-	-	-	-	-	-	-	-	-	-	-	-	-	-	-	-	-	-	-	-	-	-	-	-	-	-	-	-	-	-	-	-	+	-	-	-	-	-	-	-	-	-	-	-	+	-
4583	8199	hypothetical protein	2	-	-	-	-	-	-	-	-	-	-	-	-	-	-	-	-	-	-	-	-	-	-	-	-	-	-	-	-	-	-	-	-	-	-	-	-	-	-	-	+	-	-	-	-	-	-	-	-	-	-	-	+	-
4584	8201	hypothetical protein	2	-	-	-	-	-	-	-	-	-	-	-	-	-	-	-	-	-	-	-	-	-	-	-	-	-	-	-	-	-	-	+	-	-	-	-	-	-	-	-	-	-	-	-	-	-	-	-	-	-	+	-	-	-
4585	8202	hypothetical protein	2	-	-	-	-	-	-	-	-	-	-	-	-	-	-	-	-	-	-	-	-	-	-	-	-	-	-	-	-	-	-	-	-	-	-	-	-	-	-	-	-	-	-	-	-	-	-	-	-	+	+	-	-	-
4586	8203	hypothetical protein	2	-	-	-	-	-	-	-	-	-	-	-	-	-	-	-	-	-	-	-	-	-	-	-	-	-	-	-	-	-	-	-	-	-	-	-	-	-	-	-	-	-	-	-	-	-	-	-	-	+	+	-	-	-
4587	8210	hypothetical protein	2	-	-	-	-	-	-	-	-	-	-	-	-	-	-	-	-	-	-	-	-	-	-	-	-	-	-	-	-	-	-	-	-	-	-	-	-	-	-	-	-	-	-	-	-	-	-	-	-	+	+	-	-	-
4588	8211	hypothetical protein	2	-	-	-	-	-	-	-	-	-	-	-	-	-	-	-	-	-	-	-	-	-	-	-	-	-	-	-	-	-	-	+	-	-	-	-	-	-	-	-	-	-	-	-	-	-	-	-	-	-	+	-	-	-
4589	8215	Type II restriction enzyme MjaIII (EC 3.1.21.4)	2	-	-	-	-	-	-	-	-	-	-	-	-	-	-	-	-	-	-	-	-	-	-	-	-	-	-	-	-	-	-	-	-	-	-	-	-	-	-	-	-	-	-	-	-	-	-	-	-	+	+	-	-	-
4590	8217	hypothetical protein	2	-	-	-	-	-	-	-	-	-	-	-	-	-	-	-	-	-	-	-	-	-	-	-	-	-	-	-	-	-	-	-	-	-	-	-	-	-	-	-	-	-	-	-	-	-	-	-	-	+	+	-	-	-
4591	8219	Possible protease sohB (EC 3.4.21.-)	2	-	-	-	-	-	-	-	-	-	-	-	-	-	-	-	-	-	-	-	-	-	-	-	-	-	-	-	-	-	-	-	-	-	-	-	-	-	-	-	-	-	-	-	-	-	-	-	-	+	+	-	-	-
4592	8220	terminase small subunit	2	-	-	-	-	-	-	-	-	-	-	-	-	-	-	-	-	-	-	+	-	-	-	-	-	-	-	-	-	-	-	-	-	-	-	-	-	-	-	-	-	-	-	-	-	-	-	-	-	-	+	-	-	-
4593	8234	hypothetical protein	2	-	-	-	-	-	-	-	-	-	-	-	-	-	-	-	-	-	-	+	-	-	-	-	-	-	-	-	-	-	-	-	-	-	-	-	-	-	-	+	-	-	-	-	-	-	-	-	-	-	-	-	-	-
4594	8242	hypothetical protein	2	-	-	-	-	-	-	-	-	-	-	-	-	-	-	-	-	-	-	+	-	-	-	-	-	-	-	-	-	-	-	-	-	-	-	-	-	-	-	+	-	-	-	-	-	-	-	-	-	-	-	-	-	-
4595	8243	hypothetical protein	2	-	-	-	-	-	-	-	-	-	-	-	-	-	-	-	-	-	-	-	-	-	-	-	-	-	-	-	-	-	-	-	-	-	-	-	-	-	-	+	-	-	-	-	-	-	-	-	-	-	-	-	+	-
4596	8245	ABC transporter ATP binding protein	2	-	-	-	-	-	-	-	-	-	-	-	-	-	-	-	-	-	-	-	-	-	-	-	-	-	-	-	-	-	-	-	-	-	-	-	-	-	-	+	-	-	-	-	-	-	-	-	-	-	-	-	+	-
4597	8250	hypothetical protein	2	-	-	-	-	-	-	-	-	-	-	-	-	-	-	-	-	-	-	+	-	-	-	-	-	-	-	-	-	-	-	-	-	-	-	-	-	-	-	-	-	-	-	-	-	-	-	-	-	-	-	-	+	-
4598	8253	Streptothricin acetyltransferase	2	-	-	-	-	-	-	-	+	-	-	-	-	-	-	-	-	-	-	-	-	-	-	-	-	-	-	-	-	-	-	-	-	-	-	-	-	-	-	-	-	-	-	-	-	-	-	-	-	-	-	-	+	-
4599	8254	hypothetical protein	2	-	-	-	-	-	-	-	-	-	-	-	-	-	-	-	-	-	-	-	-	-	-	-	-	-	-	-	-	-	-	-	-	-	-	-	-	-	-	-	-	-	-	-	-	+	-	-	-	-	-	-	+	-
4600	8504	hypothetical protein	2	-	-	-	-	-	-	-	-	-	-	-	-	-	-	-	-	-	-	+	-	-	-	-	-	-	-	-	-	-	-	-	-	-	-	-	-	-	-	-	-	-	-	-	-	-	-	-	-	+	-	-	-	-
4601	8508	O-acetylhomoserine sulfhydrylase (EC 2.5.1.49) / O-succinylhomoserine sulfhydrylase (EC 2.5.1.48)	2	-	-	-	-	-	-	-	-	-	-	-	-	-	-	-	-	-	-	-	-	-	-	-	-	-	-	-	-	-	+	+	-	-	-	-	-	-	-	-	-	-	-	-	-	-	-	-	-	-	-	-	-	-
4602	8509	ORF36	2	-	-	-	-	-	-	-	-	-	-	-	-	-	-	-	-	-	-	-	-	-	-	-	-	-	-	-	-	-	+	+	-	-	-	-	-	-	-	-	-	-	-	-	-	-	-	-	-	-	-	-	-	-
4603	8510	Mobile element protein	2	-	-	-	-	-	-	-	-	-	-	-	-	-	-	-	-	-	-	-	-	-	-	-	-	-	-	-	-	-	+	+	-	-	-	-	-	-	-	-	-	-	-	-	-	-	-	-	-	-	-	-	-	-
4604	8511	hypothetical protein	2	-	-	-	-	-	-	-	-	-	-	-	-	-	-	-	-	-	-	-	-	-	-	-	-	-	-	-	-	-	+	+	-	-	-	-	-	-	-	-	-	-	-	-	-	-	-	-	-	-	-	-	-	-
4605	8512	hypothetical protein	2	-	-	-	-	-	-	-	-	-	-	-	-	-	-	-	-	-	-	-	-	-	-	-	-	-	-	-	-	-	+	+	-	-	-	-	-	-	-	-	-	-	-	-	-	-	-	-	-	-	-	-	-	-
4606	8513	hypothetical protein	2	-	-	-	-	-	-	-	-	-	-	-	-	-	-	-	-	-	-	-	-	-	-	-	-	-	-	-	-	-	+	+	-	-	-	-	-	-	-	-	-	-	-	-	-	-	-	-	-	-	-	-	-	-
4607	8514	Duplicated ATPase component BL0693 of energizing module of predicted ECF transporter	2	-	-	-	-	-	-	-	-	-	-	-	-	-	-	-	-	-	-	-	-	-	-	-	-	-	-	-	-	+	+	-	-	-	-	-	-	-	-	-	-	-	-	-	-	-	-	-	-	-	-	-	-	-
4608	8515	Putative ABC transporter (ATP-binding protein)	2	-	-	-	-	-	-	-	-	-	-	-	-	-	-	-	-	-	-	-	-	-	-	-	-	-	-	-	+	+	-	-	-	-	-	-	-	-	-	-	-	-	-	-	-	-	-	-	-	-	-	-	-	-
4609	8516	PhnB protein; putative DNA binding 3-demethylubiquinone-9 3-methyltransferase domain protein	2	-	-	-	-	-	-	-	-	-	-	-	-	-	-	-	-	-	-	-	-	-	-	-	-	-	-	-	+	+	-	-	-	-	-	-	-	-	-	-	-	-	-	-	-	-	-	-	-	-	-	-	-	-
4610	8522	ATPas	2	-	-	-	-	-	-	-	-	-	-	-	-	-	-	-	-	-	-	-	-	-	-	-	-	-	-	+	+	-	-	-	-	-	-	-	-	-	-	-	-	-	-	-	-	-	-	-	-	-	-	-	-	-
4611	8523	hypothetical protein	2	-	-	-	-	-	-	-	-	-	-	-	-	-	-	-	-	-	-	-	-	-	-	-	-	-	-	+	+	-	-	-	-	-	-	-	-	-	-	-	-	-	-	-	-	-	-	-	-	-	-	-	-	-
4612	8524	hypothetical protein	2	-	-	-	-	-	-	-	-	-	-	-	-	-	-	-	-	-	-	-	-	+	+	-	-	-	-	-	-	-	-	-	-	-	-	-	-	-	-	-	-	-	-	-	-	-	-	-	-	-	-	-	-	-
4613	8525	Type II restriction enzyme MjaIII (EC 3.1.21.4)	2	-	-	-	-	-	-	-	-	-	-	-	-	-	-	-	-	-	-	-	-	+	+	-	-	-	-	-	-	-	-	-	-	-	-	-	-	-	-	-	-	-	-	-	-	-	-	-	-	-	-	-	-	-
4614	8526	hypothetical protein	2	-	-	-	-	-	-	-	-	-	-	-	-	-	-	-	-	-	-	-	-	+	+	-	-	-	-	-	-	-	-	-	-	-	-	-	-	-	-	-	-	-	-	-	-	-	-	-	-	-	-	-	-	-
4615	8527	hypothetical protein	2	-	-	-	-	-	-	-	-	-	-	-	-	-	-	-	-	-	-	-	-	+	+	-	-	-	-	-	-	-	-	-	-	-	-	-	-	-	-	-	-	-	-	-	-	-	-	-	-	-	-	-	-	-
4616	8528	Prephenate dehydrogenase (EC 1.3.1.12)	2	-	-	-	-	-	-	-	-	-	-	-	-	-	-	-	-	-	-	-	-	+	+	-	-	-	-	-	-	-	-	-	-	-	-	-	-	-	-	-	-	-	-	-	-	-	-	-	-	-	-	-	-	-
4617	8529	Cu+ P-type ATPase	2	-	-	-	-	-	-	-	-	-	-	-	-	-	+	-	-	-	-	-	-	-	-	-	-	-	-	-	-	-	-	-	-	-	-	-	-	-	-	-	-	-	-	-	-	-	-	-	-	-	-	-	+	-
4618	8530	Exopolysaccharide biosynthesis transcriptional activator EpsA	2	-	-	-	-	-	-	-	-	+	-	-	-	-	+	-	-	-	-	-	-	-	-	-	-	-	-	-	-	-	-	-	-	-	-	-	-	-	-	-	-	-	-	-	-	-	-	-	-	-	-	-	-	-
4619	8531	hypothetical protein	2	-	-	-	-	-	-	-	-	-	-	-	-	-	+	-	-	-	-	-	-	-	-	-	-	-	-	-	-	-	+	-	-	-	-	-	-	-	-	-	-	-	-	-	-	-	-	-	-	-	-	-	-	-
4620	8532	hypothetical protein	2	-	-	-	-	-	-	-	-	-	+	-	-	-	+	-	-	-	-	-	-	-	-	-	-	-	-	-	-	-	-	-	-	-	-	-	-	-	-	-	-	-	-	-	-	-	-	-	-	-	-	-	-	-
4621	8534	hypothetical protein	2	-	-	-	-	-	-	-	-	-	-	-	-	-	-	-	-	-	-	-	-	-	-	-	-	-	-	+	+	-	-	-	-	-	-	-	-	-	-	-	-	-	-	-	-	-	-	-	-	-	-	-	-	-
4622	8535	hypothetical protein	2	-	-	-	-	-	-	-	-	-	-	-	-	-	-	-	-	-	-	-	-	-	-	-	-	-	-	+	+	-	-	-	-	-	-	-	-	-	-	-	-	-	-	-	-	-	-	-	-	-	-	-	-	-
4623	8536	tRNA-dependent lipid II-Ala--L-alanine ligase	2	-	-	-	-	-	-	-	-	-	-	-	-	-	-	-	-	-	-	-	-	-	-	-	-	-	-	+	+	-	-	-	-	-	-	-	-	-	-	-	-	-	-	-	-	-	-	-	-	-	-	-	-	-
4624	8537	hypothetical protein	2	-	-	-	-	-	-	-	-	-	-	-	-	-	-	-	-	-	-	-	-	-	-	-	-	-	-	+	+	-	-	-	-	-	-	-	-	-	-	-	-	-	-	-	-	-	-	-	-	-	-	-	-	-
4625	8538	hypothetical protein	2	-	-	-	-	-	-	-	-	-	-	-	-	-	-	-	-	-	-	-	-	-	-	-	-	-	-	+	+	-	-	-	-	-	-	-	-	-	-	-	-	-	-	-	-	-	-	-	-	-	-	-	-	-
4626	8539	hypothetical protein	2	-	-	-	-	-	-	-	-	-	-	-	-	-	-	-	-	-	-	-	-	-	-	-	-	-	-	+	+	-	-	-	-	-	-	-	-	-	-	-	-	-	-	-	-	-	-	-	-	-	-	-	-	-

Table S5. Precence/absence of each HG among 57 *S. suis* strains

Index[a]	ClusterID	Gene products	Members	98HAH33	05ZYH33	SC84	A7	SS12	P1/7	DAT273	NIAH11434	JS14	GZ1	BM407	S735	13730	MNCM07	NCTC 10446	14636	NCTC 10237	ST1	2651	89-1591	MNCM04	MNCM25	8074	D9	4961	ST3	MNCM43	DAT292	DAT260	NIAH11435	MNCM21	MNCM50	89-2479	DAT234	DAT290	93A	42A	NT77	6407	11538	8830	12814	92-1400	89-590	2726	2524	89-3576-3	4417	D12	10581	89-5259	88-5299A	R61	92-1191	14A	92-4172	22083
				1																13			25					28						104		94			76			53/54		Divergent *S. suis*																
4627	8540	hypothetical protein	2	-	-	-	-	-	-	-	-	-	-	-	-	-	-	-	-	-	-	-	-	-	-	-	-	-	-	-	-	-	-	+	+	-	-	-	-	-	-	-	-	-	-	-	-	-	-	-	-	-	-	-	-	-	-	-	-	-
4628	8541	hypothetical protein	2	-	-	-	-	-	-	-	-	-	-	-	-	-	-	-	-	-	-	-	-	-	-	-	-	-	-	-	-	-	-	+	+	-	-	-	-	-	-	-	-	-	-	-	-	-	-	-	-	-	-	-	-	-	-	-	-	-
4629	8542	hypothetical protein	2	-	-	-	-	-	-	-	-	-	-	-	-	-	-	-	-	-	-	-	-	-	-	-	-	-	-	-	-	-	-	+	+	-	-	-	-	-	-	-	-	-	-	-	-	-	-	-	-	-	-	-	-	-	-	-	-	-
4630	8543	hypothetical protein	2	-	-	-	-	-	-	-	-	-	-	-	-	-	-	-	-	-	-	-	-	-	-	-	-	-	-	-	-	-	-	+	+	-	-	-	-	-	-	-	-	-	-	-	-	-	-	-	-	-	-	-	-	-	-	-	-	-
4631	8544	hypothetical protein	2	-	-	-	-	-	-	-	-	-	-	-	-	-	-	-	-	-	-	-	-	-	-	-	-	-	-	-	-	-	-	+	+	-	-	-	-	-	-	-	-	-	-	-	-	-	-	-	-	-	-	-	-	-	-	-	-	-
4632	8545	hypothetical protein	2	-	-	-	-	-	-	-	-	-	-	-	-	-	-	-	-	-	-	-	-	-	-	-	-	-	-	-	-	-	-	+	+	-	-	-	-	-	-	-	-	-	-	-	-	-	-	-	-	-	-	-	-	-	-	-	-	-
4633	8546	hypothetical protein	2	-	-	-	-	-	-	-	-	-	-	-	-	-	-	-	-	-	-	-	-	-	-	-	-	-	-	-	-	-	-	+	+	-	-	-	-	-	-	-	-	-	-	-	-	-	-	-	-	-	-	-	-	-	-	-	-	-
4634	8547	hypothetical protein	2	-	-	-	-	-	-	-	-	-	-	-	-	-	-	-	-	-	-	-	-	-	-	-	-	-	-	-	-	-	-	+	+	-	-	-	-	-	-	-	-	-	-	-	-	-	-	-	-	-	-	-	-	-	-	-	-	-
4635	8548	ABC-type multidrug/protein/lipid transport system	2	-	-	-	-	-	-	-	-	-	-	-	-	-	-	-	-	-	-	-	-	-	-	-	-	-	-	-	-	-	-	+	+	-	-	-	-	-	-	-	-	-	-	-	-	-	-	-	-	-	-	-	-	-	-	-	-	-
4636	8549	hypothetical protein	2	-	-	-	-	-	-	-	-	-	-	-	-	-	-	-	-	-	-	-	-	-	-	-	-	-	-	-	-	-	-	+	+	-	-	-	-	-	-	-	-	-	-	-	-	-	-	-	-	-	-	-	-	-	-	-	-	-
4637	8550	hypothetical protein	2	-	-	-	-	-	-	-	-	-	-	-	-	-	-	-	-	-	-	-	-	-	-	-	-	-	-	-	-	-	-	+	+	-	-	-	-	-	-	-	-	-	-	-	-	-	-	-	-	-	-	-	-	-	-	-	-	-
4638	10543	hypothetical protein	2	-	-	-	-	-	-	-	-	-	-	-	-	-	-	-	-	-	+	-	+	-	-	-	-	-	-	-	-	-	-	-	-	-	-	-	-	-	-	-	-	-	-	-	-	-	-	-	-	-	-	-	-	-
4639	10545	hypothetical protein	2	-	-	-	-	-	-	-	-	+	-	-	-	-	-	-	-	-	-	-	-	-	-	-	-	-	-	-	-	-	-	-	-	-	-	-	-	-	-	-	-	-	-	-	-	+	-	-	-	-	-	-	-	-
4640	10551	hypothetical protein	2	-	-	-	-	-	-	-	-	-	-	-	-	-	-	-	-	-	-	-	-	-	-	-	+	-	-	-	-	-	-	-	-	-	-	-	-	-	-	-	-	-	-	-	-	+	-	-	-	-	-	-	-	-
4641	10553	Group B streptococcal surface immunogenic protein	2	-	-	-	+	+	-	-	-	-	-	-	-	-	-	-	-	-	-	-	-	-	-	-	-	-	-	-	-	-	-	-	-	-	-	-	-	-	-	-	-	-	-	-	-	-	-	-	-	-	-	-	-	-
4642	10556	Thymidylate kinase (EC 2.7.4.9)	2	+	+	-	-	-	-	-	-	-	-	-	-	-	-	-	-	-	-	-	-	-	-	-	-	-	-	-	-	-	-	-	-	-	-	-	-	-	-	-	-	-	-	-	-	-	-	-	-	-	-	-	-	-
4643	10557	hypothetical protein	2	+	+	-	-	-	-	-	-	-	-	-	-	-	-	-	-	-	-	-	-	-	-	-	-	-	-	-	-	-	-	-	-	-	-	-	-	-	-	-	-	-	-	-	-	-	-	-	-	-	-	-	-	-
4644	10558	hypothetical protein	2	+	+	-	-	-	-	-	-	-	-	-	-	-	-	-	-	-	-	-	-	-	-	-	-	-	-	-	-	-	-	-	-	-	-	-	-	-	-	-	-	-	-	-	-	-	-	-	-	-	-	-	-	-
4645	10559	hypothetical protein	2	+	+	-	-	-	-	-	-	-	-	-	-	-	-	-	-	-	-	-	-	-	-	-	-	-	-	-	-	-	-	-	-	-	-	-	-	-	-	-	-	-	-	-	-	-	-	-	-	-	-	-	-	-
4646	10560	Phosphoribosylpyrophosphate synthetase	2	+	+	-	-	-	-	-	-	-	-	-	-	-	-	-	-	-	-	-	-	-	-	-	-	-	-	-	-	-	-	-	-	-	-	-	-	-	-	-	-	-	-	-	-	-	-	-	-	-	-	-	-	-
4647	10561	hypothetical protein	2	+	+	-	-	-	-	-	-	-	-	-	-	-	-	-	-	-	-	-	-	-	-	-	-	-	-	-	-	-	-	-	-	-	-	-	-	-	-	-	-	-	-	-	-	-	-	-	-	-	-	-	-	-
4648	10562	hypothetical protein	2	+	+	-	-	-	-	-	-	-	-	-	-	-	-	-	-	-	-	-	-	-	-	-	-	-	-	-	-	-	-	-	-	-	-	-	-	-	-	-	-	-	-	-	-	-	-	-	-	-	-	-	-	-
4649	10563	LSU m3Psi1915 methyltransferase RlmH	2	+	+	-	-	-	-	-	-	-	-	-	-	-	-	-	-	-	-	-	-	-	-	-	-	-	-	-	-	-	-	-	-	-	-	-	-	-	-	-	-	-	-	-	-	-	-	-	-	-	-	-	-	-
4650	10564	hypothetical protein	2	+	+	-	-	-	-	-	-	-	-	-	-	-	-	-	-	-	-	-	-	-	-	-	-	-	-	-	-	-	-	-	-	-	-	-	-	-	-	-	-	-	-	-	-	-	-	-	-	-	-	-	-	-
4651	10565	hypothetical protein	2	+	+	-	-	-	-	-	-	-	-	-	-	-	-	-	-	-	-	-	-	-	-	-	-	-	-	-	-	-	-	-	-	-	-	-	-	-	-	-	-	-	-	-	-	-	-	-	-	-	-	-	-	-
4652	1936	Response regulator CsrR	1	-	-	-	-	-	-	-	-	-	-	-	-	-	-	-	-	-	-	-	-	-	-	-	-	-	-	-	-	-	-	-	-	-	-	-	-	-	-	-	-	-	-	-	-	-	-	+	-	-	-	-	-	-
4653	1991	Immunogenic secreted protein	1	-	-	-	-	-	-	-	-	-	-	-	-	-	-	-	-	-	-	-	-	-	-	-	-	-	-	-	-	-	-	-	-	-	-	-	-	-	-	-	-	-	-	-	-	-	-	-	-	-	-	-	+	-
4654	2082	Phosphate transport system permease protein PstC (TC 3.A.1.7.1)	1	-	-	-	-	-	-	-	-	-	-	-	-	-	-	-	+	-	-	-	-	-	-	-	-	-	-	-	-	-	-	-	-	-	-	-	-	-	-	-	-	-	-	-	-	-	-	-	-	-	-	-	-	-
4655	2089	Mobile element protein	1	-	-	-	-	-	-	-	-	-	-	-	-	-	-	-	-	-	-	-	-	-	-	-	-	-	-	-	-	-	-	-	-	-	-	-	-	-	-	-	-	+	-	-	-	-	-	-	-	-	-	-	-	-
4656	2118	Phosphate transport system permease protein PstA (TC 3.A.1.7.1)	1	-	-	-	-	-	-	-	-	-	-	-	-	-	-	-	+	-	-	-	-	-	-	-	-	-	-	-	-	-	-	-	-	-	-	-	-	-	-	-	-	-	-	-	-	-	-	-	-	-	-	-	-	-
4657	2155	PTS system, fructose- and mannose-inducible putative EII component	1	-	-	-	-	-	-	-	-	-	-	-	-	-	-	-	-	-	-	-	-	-	-	-	-	-	-	-	-	-	-	-	-	-	-	-	-	-	-	-	-	+	-	-	-	-	-	-	-	-	-	-	-	-
4658	2156	periplasmic fructose-binding protein component of signal transduction system LevT	1	-	-	-	-	-	-	-	-	-	-	-	-	-	-	-	-	-	-	-	-	-	-	-	-	-	-	-	-	-	-	-	-	-	-	-	-	-	-	-	-	+	-	-	-	-	-	-	-	-	-	-	-	-
4659	2197	PTS system, fructose- and mannose-inducible IIA component (EC 2.7.1.69)	1	-	-	-	-	-	-	-	-	-	-	-	-	-	-	-	-	-	-	-	-	-	-	-	-	-	-	-	-	-	-	-	-	-	-	-	-	-	-	-	-	+	-	-	-	-	-	-	-	-	-	-	-	-
4660	2198	PTS system, fructose- and mannose-inducible IIC component (EC 2.7.1.69)	1	-	-	-	-	-	-	-	-	-	-	-	-	-	-	-	-	-	-	-	-	-	-	-	-	-	-	-	-	-	-	-	-	-	-	-	-	-	-	-	-	+	-	-	-	-	-	-	-	-	-	-	-	-
4661	2199	periplasmic fructose-binding protein component of signal transduction system LevQ	1	-	-	-	-	-	-	-	-	-	-	-	-	-	-	-	-	-	-	-	-	-	-	-	-	-	-	-	-	-	-	-	-	-	-	-	-	-	-	-	-	+	-	-	-	-	-	-	-	-	-	-	-	-
4662	2200	PTS system, fructose- and mannose-inducible IIB component (EC 2.7.1.69)	1	-	-	-	-	-	-	-	-	-	-	-	-	-	-	-	-	-	-	-	-	-	-	-	-	-	-	-	-	-	-	-	-	-	-	-	-	-	-	-	-	+	-	-	-	-	-	-	-	-	-	-	-	-
4663	2201	fructose response regulator of fruA and EII fructose/mannose	1	-	-	-	-	-	-	-	-	-	-	-	-	-	-	-	-	-	-	-	-	-	-	-	-	-	-	-	-	-	-	-	-	-	-	-	-	-	-	-	-	+	-	-	-	-	-	-	-	-	-	-	-	-
4664	2202	PTS system, fructose- and mannose-inducible IID component (EC 2.7.1.69)	1	-	-	-	-	-	-	-	-	-	-	-	-	-	-	-	-	-	-	-	-	-	-	-	-	-	-	-	-	-	-	-	-	-	-	-	-	-	-	-	-	+	-	-	-	-	-	-	-	-	-	-	-	-
4665	2203	fructose sensor histidine kinase	1	-	-	-	-	-	-	-	-	-	-	-	-	-	-	-	-	-	-	-	-	-	-	-	-	-	-	-	-	-	-	-	-	-	-	-	-	-	-	-	-	+	-	-	-	-	-	-	-	-	-	-	-	-
4666	2319	Mobile element protein	1	-	-	-	-	-	-	-	-	-	-	+	-	-	-	-	-	-	-	-	-	-	-	-	-	-	-	-	-	-	-	-	-	-	-	-	-	-	-	-	-	-	-	-	-	-	-	-	-	-	-	-	-	-
4667	2361	Transmembrane histidine kinase CsrS	1	-	-	-	-	-	-	-	-	-	-	-	-	-	-	-	-	-	-	-	-	-	-	-	-	-	-	-	-	-	-	-	-	-	-	-	-	-	-	-	-	-	-	-	-	-	-	+	-	-	-	-	-	-
4668	2362	Peptide methionine sulfoxide reductase MsrB (EC 1.8.4.12)	1	-	-	-	-	-	-	-	-	-	-	-	-	-	-	-	-	-	-	-	-	-	-	-	-	-	-	-	-	-	-	-	-	-	-	-	-	-	-	-	-	-	-	-	-	-	-	-	-	-	-	-	+	-
4669	2395	Polar amino acid ABC uptake transporter membrane-spanning protein	1	-	-	-	-	-	-	-	-	-	-	-	-	-	-	-	-	-	-	-	-	-	-	-	-	-	-	-	-	-	-	-	-	-	-	-	-	-	-	-	-	-	-	-	-	-	-	-	-	-	-	-	-	+
4670	2396	Lysine-arginine-ornithine-binding periplasmic protein precursor (TC 3.A.1.3.1)	1	-	-	-	-	-	-	-	-	-	-	-	-	-	-	-	-	-	-	-	-	-	-	-	-	-	-	-	-	-	-	-	-	-	-	-	-	-	-	-	-	-	-	-	-	-	-	-	-	-	-	-	-	+
4671	2398	Amino acid ABC transporter, ATP-binding protein	1	-	-	-	-	-	-	-	-	-	-	-	-	-	-	-	-	-	-	-	-	-	-	-	-	-	-	-	-	-	-	-	-	-	-	-	-	-	-	-	-	-	-	-	-	-	-	-	-	-	-	-	-	+
4672	2416	Uridine phosphorylase (EC 2.4.2.3)	1	-	-	-	-	-	-	-	-	-	-	-	-	-	-	-	-	-	-	-	-	-	-	-	-	-	-	-	-	-	-	-	-	-	-	-	-	-	-	+	-	-	-	-	-	-	-	-	-	-	-	-	-	-
4673	2417	hypothetical protein	1	-	-	-	-	-	-	-	-	-	-	-	-	-	-	-	-	-	-	-	-	-	-	-	-	-	-	-	-	-	-	-	-	-	-	-	-	-	-	-	-	-	-	-	-	-	-	+	-	-	-	-	-	-
4674	2455	UDP-N-acetylglucosamine 2-epimerase (EC 5.1.3.14)	1	-	-	-	-	-	-	-	-	-	-	-	-	-	-	-	+	-	-	-	-	-	-	-	-	-	-	-	-	-	-	-	-	-	-	-	-	-	-	-	-	-	-	-	-	-	-	-	-	-	-	-	-	-
4675	2461	ABC-type amino acid transport system, permease component	1	-	-	-	-	-	-	-	-	-	-	-	-	-	-	-	-	-	-	-	-	-	-	-	-	-	-	-	-	-	-	-	-	-	-	-	-	-	-	-	-	-	-	-	-	-	-	-	-	-	-	-	-	+
4676	2476	Arsenate reductase (EC 1.20.4.1)	1	-	-	-	-	-	-	-	-	-	-	-	-	-	-	-	-	-	-	-	-	-	-	-	-	-	-	-	-	-	-	-	-	-	-	-	-	+	-	-	-	-	-	-	-	-	-	-	-	-	-	-	-	-
4677	2493	Aquaporin Z	1	-	-	-	-	-	-	-	-	-	-	-	-	-	-	-	-	-	-	-	-	-	-	-	-	-	-	-	-	-	-	-	-	-	-	-	-	-	-	-	-	-	-	-	-	-	-	-	-	-	-	-	+	-
4678	2512	Transcriptional regulator, TetR family	1	-	-	-	-	-	-	-	-	-	-	-	-	-	-	-	-	-	-	-	-	-	-	-	-	-	-	-	-	-	-	-	-	-	-	-	-	-	-	-	-	-	-	-	-	+	-	-	-	-	-	-	-	-
4679	2592	hypothetical protein	1	-	-	-	-	-	-	-	-	-	-	-	-	-	-	-	-	-	-	+	-	-	-	-	-	-	-	-	-	-	-	-	-	-	-	-	-	-	-	-	-	-	-	-	-	-	-	-	-	-	-	-	-	-	-	-
4680	2676	Integral membrane protein	1	-	-	-	-	-	-	-	-	-	-	-	-	-	-	-	-	-	-	-	-	-	-	-	-	-	-	-	-	-	-	-	-	-	-	-	-	-	-	-	-	-	-	-	-	-	-	+	-	-	-	-	-	-
4681	2791	Hydrolase (HAD superfamily)	1	-	-	-	-	-	-	-	-	-	-	-	-	-	-	-	-	-	-	-	-	-	-	-	-	-	-	-	-	-	-	-	-	-	-	-	-	-	-	-	-	+	-	-	-	-	-	-	-	-	-	-	-	-
4682	2794	Mobile element protein	1	-	-	-	-	-	-	-	-	-	-	-	-	-	-	-	-	-	-	-	-	-	-	-	-	-	-	-	-	-	-	-	-	-	-	-	-	-	-	-	-	+	-	-	-	-	-	-	-	-	-	-	-	-
4683	2800	Mobile element protein	1	-	-	-	-	-	-	-	-	-	-	-	-	-	-	-	-	-	-	-	-	-	-	-	-	-	-	-	-	-	-	-	-	-	-	-	-	-	-	-	-	-	-	-	-	-	-	+	-	-	-	-	-	-
4684	2816	Transcriptional regulator, MarR family	1	-	-	-	-	-	-	-	-	-	-	-	-	-	-	-	-	-	-	+	-	-	-	-	-	-	-	-	-	-	-	-	-	-	-	-	-	-	-	-	-	-	-	-	-	-	-	-	-	-	-	-	-	-	-	-
4685	2923	Mobile element protein	1	-	-	-	-	-	-	-	-	-	-	-	-	-	-	-	-	-	-	-	-	-	-	-	-	-	-	-	-	-	-	-	-	-	-	-	-	-	-	+	-	-	-	-	-	-	-	-	-	-	-	-	-	-
4686	2925	DUF1706 domain-containing protein	1	-	-	-	-	-	-	-	-	-	-	-	-	-	-	-	-	-	-	-	-	-	-	-	-	-	-	-	-	-	-	-	-	-	-	-	-	-	-	+	-	-	-	-	-	-	-	-	-	-	-	-	-	-
4687	3008	Phosphate transport system regulatory protein PhoU	1	-	-	-	-	-	-	-	-	-	-	-	-	-	-	-	+	-	-	-	-	-	-	-	-	-	-	-	-	-	-	-	-	-	-	-	-	-	-	-	-	-	-	-	-	-	-	-	-	-	-	-	-	-
4688	3011	Phosphate regulon transcriptional regulatory protein PhoB (SphR)	1	-	-	-	-	-	-	-	-	-	-	-	-	-	-	-	+	-	-	-	-	-	-	-	-	-	-	-	-	-	-	-	-	-	-	-	-	-	-	-	-	-	-	-	-	-	-	-	-	-	-	-	-	-
4689	3019	Phosphate ABC transporter, periplasmic phosphate-binding protein PstS (TC 3.A.1.7.1)	1	-	-	-	-	-	-	-	-	-	-	-	-	-	-	-	+	-	-	-	-	-	-	-	-	-	-	-	-	-	-	-	-	-	-	-	-	-	-	-	-	-	-	-	-	-	-	-	-	-	-	-	-	-
4690	3158	Transcriptional regulator, XRE family	1	-	-	-	-	-	-	-	-	-	-	-	-	-	-	-	-	-	-	-	-	-	-	-	-	-	-	-	-	-	-	-	-	-	-	-	-	-	-	-	-	+	-	-	-	-	-	-	-	-	-	-	-	-
4691	3159	Sucrose-6-phosphate hydrolase (EC 3.2.1.26)	1	-	-	-	-	-	-	-	-	-	-	-	-	-	-	-	-	-	-	-	-	-	-	-	-	-	-	-	-	-	-	-	-	-	-	-	-	-	-	-	-	+	-	-	-	-	-	-	-	-	-	-	-	-
4692	3173	NLP/P60 family protein	1	-	-	-	-	-	-	-	-	-	-	-	-	-	-	-	-	-	-	-	-	-	-	-	-	-	-	-	-	-	-	-	-	-	-	-	-	-	-	+	-	-	-	-	-	-	-	-	-	-	-	-	-	-
4693	3174	DNA topoisomerase III (EC 5.99.1.2)	1	-	-	-	-	-	-	-	-	-	-	-	-	-	-	-	-	-	-	-	-	-	-	-	-	-	-	-	-	-	-	-	-	-	-	-	-	-	-	+	-	-	-	-	-	-	-	-	-	-	-	-	-	-
4694	3298	oxidoreductase, short-chain dehydrogenase/reductase family	1	-	-	-	-	-	-	-	-	-	-	-	-	-	-	-	-	-	-	-	-	-	-	-	-	-	-	-	-	-	-	-	-	-	-	-	-	-	+	-	-	-	-	-	-	-	-	-	-	-	-	-	-	-
4695	3299	hypothetical protein	1	-	-	-	-	-	-	-	-	-	-	-	-	-	-	-	-	-	-	-	-	-	-	-	-	-	-	-	-	-	-	-	-	-	-	-	-	+	-	-	-	-	-	-	-	-	-	-	-	-	-	-	-	-
4696	3312	Competence-stimulating peptide ABC transporter permease protein ComB	1	-	-	-	-	-	-	-	-	-	-	-	-	-	-	-	-	-	-	-	-	-	-	-	-	-	-	-	-	-	-	-	-	-	-	-	-	-	-	-	-	-	-	-	-	-	+	-	-	-	-	-	-	-
4697	3317	Mobile element protein	1	-	-	-	-	-	-	-	-	-	-	-	-	-	-	-	-	-	-	-	-	-	-	-	-	-	-	-	-	-	-	-	-	-	-	-	-	-	-	-	-	+	-	-	-	-	-	-	-	-	-	-	-	-
4698	3323	putative transcriptional regulator	1	-	-	-	-	-	-	-	-	-	-	-	-	-	-	-	-	-	-	-	-	-	-	-	-	-	-	-	-	-	-	-	-	-	-	-	-	-	-	-	-	-	-	-	-	-	-	-	-	-	-	-	+	-
4699	3324	Mobile element protein	1	-	-	-	-	-	-	-	-	-	-	-	-	-	-	-	-	-	-	-	-	-	-	-	-	-	-	-	-	-	-	-	-	-	-	-	-	-	-	+	-	-	-	-	-	-	-	-	-	-	-	-	-	-
4700	3332	Mature parasite-infected erythrocyte surface antigen (MESA) or PfEMP2	1	-	-	-	-	-	-	-	-	-	-	-	-	-	-	-	-	-	-	-	-	-	-	-	-	-	-	-	-	-	-	-	-	-	-	-	-	-	-	+	-	-	-	-	-	-	-	-	-	-	-	-	-	-
4701	3447	Guanosine-3',5'-bis(Diphosphate) 3'-pyrophosphohydrolase (EC 3.1.7.2)	1	-	-	-	-	-	-	-	-	-	-	-	-	-	-	-	-	-	-	-	-	-	-	-	-	-	-	-	-	-	-	-	-	-	-	-	-	-	-	-	-	-	+	-	-	-	-	-	-	-	-	-	-	-
4702	3467	Lipoteichoic acid synthase LtaS Type IIc	1	-	-	-	-	-	-	-	-	-	-	-	-	-	-	-	-	-	-	-	-	-	-	-	-	-	-	-	-	-	-	-	-	-	-	-	-	-	+	-	-	-	-	-	-	-	-	-	-	-	-	-	-	-
4703	3473	Transporter	1	-	-	-	-	-	-	-	-	-	-	-	-	-	-	-	-	-	-	-	-	-	-	-	-	-	-	-	-	-	-	-	-	-	-	-	-	+	-	-	-	-	-	-	-	-	-	-	-	-	-	-	-	-
4704	3493	transcriptional regulator, Cro/CI family	1	-	-	-	-	-	-	-	-	-	-	-	-	-	-	-	-	-	-	-	-	-	-	-	-	-	-	-	-	-	-	-	-	-	-	-	-	-	-	-	-	-	-	-	-	-	-	-	-	-	-	-	+	-
4705	3494	Endoribonuclease L-PSP	1	-	-	-	-	-	-	-	-	-	-	-	-	-	-	-	-	-	-	-	-	-	-	-	-	-	-	-	-	-	-	-	-	-	-	-	-	-	-	-	-	-	-	-	-	-	-	-	-	+	-	-	-	-
4706	3496	hypothetical protein	1	-	-	-	-	-	-	-	-	-	-	-	-	-	-	-	-	-	-	-	-	-	-	-	-	-	-	-	-	-	-	-	-	-	-	-	-	-	-	-	-	-	-	-	-	-	-	-	-	-	-	+	-	-
4707	3498	hypothetical protein	1	-	-	-	-	-	-	-	-	-	-	-	-	-	-	-	-	-	-	-	-	-	-	-	-	-	-	-	-	-	-	-	-	-	-	-	-	-	-	-	-	-	-	-	-	-	-	-	-	-	-	-	+	-
4708	3502	contains predicted nucleoside-diphosphate-sugar epimerase domain	1	-	-	-	-	-	-	-	-	-	-	-	-	-	-	-	-	-	-	-	-	-	-	-	-	-	-	-	-	-	-	-	-	-	-	-	-	-	-	-	-	-	-	-	-	-	-	+	-	-	-	-	-	-
4709	3506	DNA-damage-inducible protein J, putative	1	-	-	-	-	-	-	-	-	-	-	-	-	-	-	-	-	-	-	-	-	-	-	-	-	-	-	-	-	-	-	-	-	-	-	-	-	-	-	-	-	-	-	-	-	-	-	-	-	-	-	-	+	-
4710	3528	2-amino-3-carboxymuconate-6-semialdehyde decarboxylase (EC 4.1.1.45)	1	-	-	-	-	-	-	-	-	-	-	-	-	-	-	-	-	-	-	+	-	-	-	-	-	-	-	-	-	-	-	-	-	-	-	-	-	-	-	-	-	-	-	-	-	-	-	-	-	-	-	-	-	-	-	-
4711	3538	6-phospho-beta-glucosidase (EC 3.2.1.86)	1	-	-	-	-	-	-	-	-	-	-	-	-	-	-	-	-	-	-	-	-	-	-	-	-	-	-	-	-	-	-	+	-	-	-	-	-	-	-	-	-	-	-	-	-	-	-	-	-	-	-	-	-	-	-	-
4712	3679	transcription regulator, probable	1	-	-	-	-	-	-	-	-	-	-	-	-	-	-	-	-	-	-	-	-	-	-	-	-	-	-	-	-	-	-	-	-	-	-	-	-	-	-	-	-	-	-	-	+	-	-	-	-	-	-	-	-	-
4713	3683	hypothetical protein	1	-	-	-	-	-	-	-	-	-	-	-	-	-	-	-	-	-	-	-	-	-	-	-	-	-	-	-	-	-	-	-	-	-	-	-	-	-	+	-	-	-	-	-	-	-	-	-	-	-	-	-	-	-
4714	3690	redox-active disulfide protein 2	1	-	-	-	-	-	-	-	-	-	-	-	-	-	-	-	-	-	-	-	-	-	-	-	-	-	-	-	-	-	-	-	-	-	-	-	-	+	-	-	-	-	-	-	-	-	-	-	-	-	-	-	-	-
4715	3691	Arsenical resistance operon repressor	1	-	-	-	-	-	-	-	-	-	-	-	-	-	-	-	-	-	-	-	-	-	-	-	-	-	-	-	-	-	-	-	-	-	-	-	-	+	-	-	-	-	-	-	-	-	-	-	-	-	-	-	-	-
4716	3713	Integrase, catalytic region	1	-	-	-	-	-	-	-	-	-	-	-	-	-	-	-	-	-	-	-	-	-	-	-	-	-	-	-	-	-	-	-	-	+	-	-	-	-	-	-	-	-	-	-	-	-	-	-	-	-	-	-	-	-	-	-
4717	3721	Transcriptional regulator	1	-	-	-	-	-	-	-	-	-	-	-	-	-	-	-	-	-	-	-	-	-	-	-	-	-	-	-	-	-	-	-	-	-	-	-	-	-	-	-	-	-	-	-	-	-	-	+	-	-	-	-	-	-
4718	3742	hypothetical protein	1	-	-	-	-	-	-	-	-	-	-	-	-	-	-	-	-	-	-	-	-	-	-	-	-	-	-	-	-	-	-	-	-	-	-	-	-	-	-	+	-	-	-	-	-	-	-	-	-	-	-	-	-	-
4719	3743	Undecaprenyl-phosphate galactosephosphotransferase (EC 2.7.8.6)	1	-	-	-	-	-	-	-	-	-	-	-	-	-	-	-	-	-	-	-	-	-	-	-	-	-	-	-	-	-	-	-	-	-	-	-	-	-	-	-	-	-	-	-	-	-	-	-	-	-	-	-	+	-
4720	3744	FtsK/SpoIIIE family protein	1	-	-	-	-	-	-	-	-	-	-	-	-	-	-	-	-	-	-	-	-	-	-	-	-	-	-	-	-	-	-	-	-	-	-	-	-	-	-	-	-	-	-	-	-	-	-	-	-	-	-	-	+	-
4721	3836	Hydrolase, haloacid dehalogenase-like family	1	-	-	-	-	-	-	-	-	-	-	-	-	-	-	-	-	-	-	-	-	-	-	-	-	-	-	-	-	-	-	-	-	-	-	-	-	-	-	-	-	-	-	-	-	-	-	-	-	+	-	-	-	-
4722	3936	Transposase	1	-	-	-	-	-	-	-	-	-	-	-	-	-	-	-	-	-	-	-	-	-	-	-	-	-	-	-	-	-	-	-	-	-	-	-	-	-	-	-	-	-	-	-	+	-	-	-	-	-	-	-	-	-
4723	3961	hypothetical protein	1	-	-	-	-	-	-	-	-	-	-	-	-	-	-	-	-	-	-	-	-	-	-	-	-	-	-	-	-	-	-	-	-	-	-	-	-	+	-	-	-	-	-	-	-	-	-	-	-	-	-	-	-	-
4724	3970	TSPc, tail specific protease	1	-	-	-	-	-	-	-	-	-	-	-	-	-	-	-	-	-	-	-	-	-	-	-	-	-	-	-	-	-	-	-	-	-	-	-	-	-	-	-	-	-	-	-	-	-	+	-	-	-	-	-	-	-
4725	3985	Degenerate transposase	1	-	-	-	-	-	-	-	-	-	-	-	-	-	-	-	-	-	-	-	-	-	-	-	-	-	-	-	-	-	-	-	-	+	-	-	-	-	-	-	-	-	-	-	-	-	-	-	-	-	-	-	-	-	-	-
4726	3991	Acetyl xylan esterase 1; Cephalosporin-C deacetylase (EC 3.1.1.41)	1	-	-	-	-	-	-	-	-	-	-	-	-	-	-	-	-	-	-	-	-	-	-	-	-	-	-	-	-	-	-	-	-	-	-	-	-	-	-	-	-	-	-	-	-	-	-	+	-	-	-	-	-	-
4727	3992	GNAT family acetyltransferase	1	-	-	-	-	-	-	-	-	-	-	-	-	-	-	-	-	-	-	-	-	-	-	-	-	-	-	-	-	-	-	-	-	-	-	-	-	-	-	-	-	-	-	-	-	-	-	+	-	-	-	-	-	-
4728	3997	DNA topoisomerase III (EC 5.99.1.2)	1	-	-	-	-	-	-	-	-	-	-	-	-	-	-	-	-	-	-	-	-	-	-	-	-	-	-	-	-	-	-	-	-	-	-	-	-	-	-	-	-	-	-	-	-	-	-	-	-	-	-	+	-	-
4729	4003	phage protein	1	-	-	-	-	-	-	-	-	-	-	-	-	-	-	-	-	-	-	-	-	-	-	-	-	-	-	-	-	-	-	-	-	-	-	-	-	-	-	-	-	-	-	-	-	-	-	-	+	-	-	-	-	-
4730	4012	hypothetical protein	1	-	-	-	-	-	-	-	-	-	-	-	-	-	-	-	-	-	-	-	-	-	-	-	-	-	-	-	-	-	-	-	-	-	-	-	-	-	-	-	-	-	-	-	-	-	-	-	-	-	+	-	-	-
4731	4043	ATPase, AAA family	1	-	-	-	-	-	-	-	-	-	-	-	-	-	-	-	-	-	-	+	-	-	-	-	-	-	-	-	-	-	-	-	-	-	-	-	-	-	-	-	-	-	-	-	-	-	-	-	-	-	-	-	-	-	-	-
4732	4160	Transposase	1	-	-	-	-	-	-	-	-	-	-	-	-	-	-	-	-	-	-	-	-	-	-	-	-	-	-	-	-	-	-	-	-	-	-	-	-	-	-	-	-	-	-	-	-	-	-	-	-	+	-	-	-	-
4733	4174	Alpha-galactosidase (EC 3.2.1.22)	1	-	-	-	-	-	-	-	-	-	-	-	-	-	-	-	-	-	-	-	-	-	-	-	-	-	-	-	-	-	-	-	-	-	-	-	-	-	-	-	-	-	-	-	-	-	-	+	-	-	-	-	-	-
4734	4235	hypothetical protein	1	-	-	-	-	-	-	-	-	-	-	-	-	-	-	-	-	-	-	-	-	-	-	-	-	-	-	-	-	-	-	-	-	-	-	-	-	-	-	-	-	-	-	-	-	-	-	-	-	-	-	-	-	+
4735	4260	hypothetical protein	1	-	-	-	-	-	-	-	-	-	-	-	-	-	-	-	-	-	-	-	-	-	-	-	-	-	-	-	-	-	-	-	-	-	-	-	-	+	-	-	-	-	-	-	-	-	-	-	-	-	-	-	-	-
4736	4263	hypothetical protein	1	-	-	-	-	-	-	-	-	-	-	-	-	-	-	-	-	-	-	-	-	-	-	-	-	-	-	-	-	-	-	-	-	-	-	-	-	-	-	-	-	-	-	-	-	-	+	-	-	-	-	-	-	-
4737	4267	Tyrosine recombinase XerC	1	-	-	-	-	-	-	-	-	-	-	-	-	-	-	-	-	-	-	-	-	-	-	-	-	-	-	-	-	-	-	-	-	-	-	-	-	-	-	-	-	-	-	-	-	-	+	-	-	-	-	-	-	-
4738	4286	Mobile element protein	1	-	-	-	-	-	-	-	-	-	-	-	-	-	-	-	-	-	-	-	-	-	-	-	-	-	-	-	-	-	-	-	-	-	-	-	-	-	-	-	-	+	-	-	-	-	-	-	-	-	-	-	-	-
4739	4307	putative HsdR	1	-	-	-	-	-	-	-	-	-	-	-	-	-	-	-	-	-	-	-	-	-	-	-	-	-	-	-	-	-	-	-	-	-	+	-	-	-	-	-	-	-	-	-	-	-	-	-	-	-	-	-	-	-	-	-
4740	4309	peptide ABC transporter ATP binding protein, putative, truncated	1	-	-	-	-	-	-	-	-	-	-	-	-	-	-	-	-	-	-	-	-	-	-	-	-	-	-	-	-	-	-	-	-	-	-	+	-	-	-	-	-	-	-	-	-	-	-	-	-	-	-	-	-	-	-	-
4741	4312	sugar ABC transporter, permease protein	1	-	-	-	-	-	-	-	-	-	-	-	-	-	-	-	-	-	-	-	-	-	-	-	-	-	-	-	-	-	-	-	-	-	-	-	-	-	-	-	-	-	-	-	-	-	-	+	-	-	-	-	-	-
4742	4315	hypothetical protein	1	-	-	-	-	-	-	-	-	-	-	-	-	-	-	-	-	-	-	-	-	-	-	-	-	-	-	-	-	-	-	-	-	-	-	-	-	-	-	-	-	-	-	-	-	-	-	+	-	-	-	-	-	-
4743	4320	Mobile element protein	1	-	-	-	-	-	-	-	-	-	-	-	-	-	-	-	-	-	-	-	-	-	-	-	-	-	-	-	-	-	-	-	-	-	-	-	-	-	-	+	-	-	-	-	-	-	-	-	-	-	-	-	-	-
4744	4322	sugar transport system permease protein	1	-	-	-	-	-	-	-	-	-	-	-	-	-	-	-	-	-	-	-	-	-	-	-	-	-	-	-	-	-	-	-	-	-	-	-	-	-	-	-	-	-	-	-	-	-	-	+	-	-	-	-	-	-
4745	4334	Glycosyltransferase	1	-	-	-	-	-	-	-	-	-	-	-	-	-	-	-	-	-	-	-	-	-	-	-	-	-	-	-	-	-	-	-	-	-	-	-	-	-	-	-	-	-	-	-	-	-	-	-	+	-	-	-	-	-
4746	4340	unknown	1	-	-	-	-	-	-	-	-	-	-	-	-	-	-	-	-	-	-	-	-	-	-	-	-	-	-	-	-	-	-	-	-	-	-	-	-	-	-	-	-	-	-	+	-	-	-	-	-	-	-	-	-	-
4747	4349	Putative ROK-family transcriptional regulator	1	-	-	-	-	-	-	-	-	-	-	-	-	-	-	-	-	-	-	-	-	-	-	-	-	-	-	-	-	-	-	-	-	-	-	-	-	-	-	-	-	-	-	-	-	-	-	-	-	-	+	-	-	-
4748	4350	putative low temperature requirement A protein	1	-	-	-	-	-	-	-	-	-	-	-	-	-	-	-	-	-	-	-	-	-	-	-	-	-	-	-	-	-	-	-	-	-	-	-	-	-	-	+	-	-	-	-	-	-	-	-	-	-	-	-	-	-
4749	4351	TrsK-like protein	1	-	-	-	-	-	-	-	-	-	-	-	-	-	-	-	-	-	-	-	-	-	-	-	-	-	-	-	-	-	-	-	-	-	-	-	-	-	-	+	-	-	-	-	-	-	-	-	-	-	-	-	-	-
4750	4352	hypothetical protein	1	-	-	-	-	-	-	-	-	-	-	-	-	-	-	-	-	-	-	-	-	-	-	-	-	-	-	-	-	-	-	-	-	-	-	-	-	-	-	-	-	-	-	-	-	-	-	-	-	-	-	-	+	-
4751	4517	hypothetical protein	1	-	-	-	-	-	-	-	-	-	-	-	-	-	-	-	-	-	-	-	-	-	-	-	-	-	-	-	-	-	-	-	-	-	-	-	-	-	-	-	-	-	-	-	-	-	-	-	-	+	-	-	-	-
4752	4599	Phage minor capsid protein	1	-	-	-	-	-	-	-	-	-	-	-	-	-	-	+	-	-	-	-	-	-	-	-	-	-	-	-	-	-	-	-	-	-	-	-	-	-	-	-	-	-	-	-	-	-	-	-	-	-	-	-	-	-	-	-
4753	4673	N-acetylmannosaminyltransferase (EC 2.4.1.187)	1	-	-	-	-	-	-	-	-	-	-	-	-	-	-	-	+	-	-	-	-	-	-	-	-	-	-	-	-	-	-	-	-	-	-	-	-	-	-	-	-	-	-	-	-	-	-	-	-	-	-	-	-	-
4754	4677	hypothetical protein	1	-	-	-	-	-	-	-	-	-	-	-	-	-	-	-	-	-	-	-	-	-	-	-	-	-	-	-	-	-	-	-	-	-	-	-	-	-	-	-	-	-	-	-	-	-	-	-	-	-	-	-	-	+
4755	4678	suppressor of fused homolog	1	-	-	-	-	-	-	-	-	-	-	-	-	-	-	-	-	-	-	-	-	-	-	-	-	-	-	-	-	-	-	-	-	-	-	-	-	-	-	-	-	-	-	-	-	-	-	-	-	-	-	-	-	+
4756	4689	AAA ATPase, central region	1	-	-	-	-	-	-	-	-	-	-	-	-	-	-	-	-	-	-	-	-	-	-	-	-	-	-	-	-	-	-	-	-	-	-	-	-	-	-	-	-	-	-	-	-	-	-	-	-	-	-	-	-	+
4757	4697	MobC, Bacterial mobilisation protein (MobC)	1	-	-	-	-	-	-	-	-	-	-	-	-	-	-	-	-	-	-	-	-	-	-	-	-	-	-	-	-	-	-	-	-	-	-	-	-	-	-	-	-	-	-	-	-	-	-	-	-	-	-	+	-	-
4758	4709	Lipoteichoic acid synthase LtaS Type IIc	1	-	-	-	-	-	-	-	-	-	-	-	-	-	-	-	-	-	-	-	-	-	-	-	-	-	-	-	-	-	-	-	-	-	-	-	-	-	-	-	-	-	-	-	-	-	-	-	+	-	-	-	-	-
4759	4739	membrane protein, related to Actinobacillus protein (1944168)	1	-	-	-	-	-	-	-	-	-	-	-	-	-	-	-	-	-	-	-	-	-	-	-	-	-	-	-	-	-	-	-	-	-	-	-	-	-	-	-	-	-	-	-	-	-	+	-	-	-	-	-	-	-
4760	4745	Conserved domain uncharacterized protein	1	-	-	-	-	-	-	-	-	-	-	-	-	-	-	-	-	-	-	-	-	-	-	-	-	-	-	-	-	-	-	-	-	-	-	-	-	-	-	-	-	-	-	-	-	-	+	-	-	-	-	-	-	-
4761	4763	ABC transporter ATP-binding protein	1	-	-	-	-	-	-	-	-	-	-	-	-	-	-	+	-	-	-	-	-	-	-	-	-	-	-	-	-	-	-	-	-	-	-	-	-	-	-	-	-	-	-	-	-	-	-	-	-	-	-	-	-	-	-	-
4762	4764	Phage minor capsid protein	1	-	-	-	-	-	-	-	-	-	-	-	-	-	-	+	-	-	-	-	-	-	-	-	-	-	-	-	-	-	-	-	-	-	-	-	-	-	-	-	-	-	-	-	-	-	-	-	-	-	-	-	-	-	-	-
4763	4790	hypothetical protein	1	-	-	-	-	-	-	-	-	-	-	-	-	-	-	-	-	-	-	-	-	-	-	-	-	-	-	-	-	-	-	-	-	-	-	-	+	-	-	-	-	-	-	-	-	-	-	-	-	-	-	-	-	-	-	-
4764	4798	lipase/acylhydrolase family protein	1	-	-	-	-	-	-	-	-	-	-	-	-	-	-	-	-	-	-	-	-	-	-	-	-	-	-	-	-	-	-	-	-	-	-	-	-	-	-	-	-	-	-	-	-	-	-	+	-	-	-	-	-	-
4765	4807	hypothetical protein	1	-	-	-	-	-	-	-	-	-	-	-	-	-	-	-	-	-	-	-	-	-	-	-	-	-	-	-	-	-	-	-	-	-	-	-	-	-	-	-	-	-	-	-	-	-	-	-	-	-	-	+	-	-
4766	4810	Thioredoxin domain-containing protein	1	-	-	-	-	-	-	-	-	-	-	-	-	-	-	-	-	-	-	-	-	-	-	-	-	-	-	-	-	-	-	-	-	-	-	-	-	-	-	-	-	-	-	-	-	-	-	+	-	-	-	-	-	-
4767	4813	hypothetical protein	1	-	-	-	-	-	-	-	-	-	-	-	-	-	-	-	-	-	-	-	-	-	-	-	-	-	-	-	-	-	-	-	-	-	-	-	-	-	-	-	-	-	-	-	-	-	-	-	-	+	-	-	-	-
4768	4818	Macrolide-efflux protein	1	-	-	-	-	-	-	-	-	-	-	-	-	-	-	-	-	-	-	-	-	-	-	-	-	-	-	-	-	-	-	-	-	-	-	-	-	-	-	-	-	-	-	-	-	-	-	-	+	-	-	-	-	-
4769	4824	putative cI-like repressor, phage associated	1	-	-	-	-	-	-	-	-	-	-	-	-	-	-	-	-	-	-	-	-	-	-	-	-	-	-	-	-	-	-	-	-	-	-	-	-	-	-	-	-	-	+	-	-	-	-	-	-	-	-	-	-	-	-	-
4770	4826	Methylated-DNA--protein-cysteine methyltransferase (EC 2.1.1.63)	1	-	-	-	-	-	-	-	-	-	-	-	-	-	-	-	-	-	-	-	-	-	-	-	-	-	-	-	-	-	-	-	-	-	-	-	-	-	-	-	-	-	-	-	-	-	-	-	-	-	+	-	-	-
4771	4827	PTS system, cellobiose-specific IIC component (EC 2.7.1.69)	1	-	-	-	-	-	-	-	-	-	-	-	-	-	-	-	-	-	-	-	-	-	-	-	-	-	-	-	-	-	-	-	-	-	-	-	-	-	-	-	-	-	-	-	-	-	-	-	-	-	+	-	-	-
4772	4828	hypothetical protein	1	-	-	-	-	-	-	-	-	-	-	-	-	-	-	-	-	-	-	-	-	-	-	-	-	-	-	-	-	-	-	-	-	-	-	-	-	-	-	-	-	-	-	-	-	-	-	-	-	-	-	-	+	-
4773	4874	contains permease domain	1	-	-	-	-	-	-	-	-	-	-	-	-	-	-	-	-	-	-	+	-	-	-	-	-	-	-	-	-	-	-	-	-	-	-	-	-	-	-	-	-	-	-	-	-	-	-	-	-	-	-	-	-	-	-	-
4774	4875	hypothetical protein	1	-	-	-	-	-	-	-	-	-	-	-	-	-	-	-	-	-	-	+	-	-	-	-	-	-	-	-	-	-	-	-	-	-	-	-	-	-	-	-	-	-	-	-	-	-	-	-	-	-	-	-	-	-	-	-
4775	4876	Putative oxidoreductase	1	-	-	-	-	-	-	-	-	-	-	-	-	-	-	-	-	-	-	+	-	-	-	-	-	-	-	-	-	-	-	-	-	-	-	-	-	-	-	-	-	-	-	-	-	-	-	-	-	-	-	-	-	-	-	-
4776	4877	MdaB protein	1	-	-	-	-	-	-	-	-	-	-	-	-	-	-	-	-	-	-	+	-	-	-	-	-	-	-	-	-	-	-	-	-	-	-	-	-	-	-	-	-	-	-	-	-	-	-	-	-	-	-	-	-	-	-	-
4777	5152	Phage protein	1	-	-	-	-	-	-	-	-	-	-	-	-	-	-	+	-	-	-	-	-	-	-	-	-	-	-	-	-	-	-	-	-	-	-	-	-	-	-	-	-	-	-	-	-	-	-	-	-	-	-	-	-	-	-	-
4778	5184	agglutinin receptor	1	-	-	-	-	-	-	-	-	-	-	-	-	-	-	-	-	-	-	-	-	-	-	-	-	-	-	-	-	-	-	-	-	-	-	-	-	-	-	-	-	-	-	-	-	-	-	+	-	-	-	-	-	-	-	-
4779	5196	Mobile element protein	1	-	-	-	-	-	-	-	-	-	-	-	-	-	+	-	-	-	-	-	-	-	-	-	-	-	-	-	-	-	-	-	-	-	-	-	-	-	-	-	-	-	-	-	-	-	-	-	-	-	-	-	-	-	-	-

Table S5. Precence/absence of each HG among 57 *S. suis* strains

Index[a]	ClusterID	Gene products	Members	98HAH33	05ZYH33	SC84	A7	SS12	P1/7	DAT273	NIAH11434	JS14	GZ1	BM407	S735	13730	MNCM07	NCTC 10446	14636	NCTC 10237	ST1	2651	89-1591	MNCM04	MNCM25	8074	D9	4961	ST3	MNCM43	DAT292	DAT260	NIAH11435	MNCM21	MNCM50	89-2479	DAT234	DAT290	93A	42A	NT77	6407	11538	8830	12814	92-1400	89-590	2726	2524	89-3576-3	4417	D12	10581	89-5259	88-5299A	R61	92-1191	14A	92-4172	22083
				1																13			25					28						104		94			76			53/54		Divergent *S. suis*																
4780	5226	Lipopolysaccharide cholinephosphotransferase LicD2 (EC 2.7.8.-)	1	-	-	-	-	-	-	-	-	-	-	-	-	-	-	-	+	-	-	-	-	-	-	-	-	-	-	-	-	-	-	-	-	-	-	-	-	-	-	-	-	-	-	-	-	-	-	-	-	-	-	-	-	-	-	-	-	-
4781	5228	Phosphate regulon sensor protein PhoR (SphS) (EC 2.7.13.3)	1	-	-	-	-	-	-	-	-	-	-	-	-	-	-	-	+	-	-	-	-	-	-	-	-	-	-	-	-	-	-	-	-	-	-	-	-	-	-	-	-	-	-	-	-	-	-	-	-	-	-	-	-	-	-	-	-	-
4782	5230	hypothetical protein	1	-	-	-	-	-	-	-	-	-	-	-	-	-	-	-	+	-	-	-	-	-	-	-	-	-	-	-	-	-	-	-	-	-	-	-	-	-	-	-	-	-	-	-	-	-	-	-	-	-	-	-	-	-	-	-	-	-
4783	5245	Oxidoreductase, putative	1	-	-	-	-	-	-	-	-	-	-	-	-	-	-	-	-	-	-	-	-	-	-	-	-	-	-	-	-	-	-	-	-	-	-	-	-	-	-	-	-	-	-	-	-	-	-	-	-	-	-	-	-	-	-	-	-	+
4784	5258	Two-component response regulator YvcP	1	-	-	-	-	-	-	-	-	-	-	-	-	-	-	-	-	-	-	-	-	-	-	-	-	-	-	-	-	-	-	-	-	-	-	-	-	-	-	-	-	-	-	-	-	-	-	-	+	-	-	-	-	-	-	-	-	-
4785	5259	RNA polymerase ECF-type sigma factor	1	-	-	-	-	-	-	-	-	-	-	-	-	-	-	-	-	-	-	-	-	-	-	-	-	-	-	-	-	-	-	-	-	-	-	-	-	-	-	-	-	-	-	-	-	-	-	-	+	-	-	-	-	-	-	-	-	-
4786	5268	Retron-type RNA-directed DNA polymerase (EC 2.7.7.49)	1	-	-	-	-	-	-	-	-	-	-	-	-	-	-	-	-	-	-	-	-	-	-	-	-	-	-	-	-	-	-	-	-	-	-	-	-	-	-	-	-	-	-	-	-	-	-	-	+	-	-	-	-	-	-	-	-	-
4787	5269	Gfa-like protein	1	-	-	-	-	-	-	-	-	-	-	-	-	-	-	-	-	-	-	-	-	-	-	-	-	-	-	-	-	-	-	-	-	-	-	-	-	-	-	-	-	-	+	-	-	-	-	-	-	-	-	-	-	-	-	-	-	-
4788	5302	prophage ps3 protein 01, putative	1	-	-	-	-	-	-	-	-	-	-	-	-	-	-	-	-	-	-	-	-	-	-	-	-	-	-	-	-	-	-	-	-	-	-	-	-	-	-	-	-	+	-	-	-	-	-	-	-	-	-	-	-	-	-	-	-	-
4789	5309	putative phage protein	1	-	-	-	-	-	-	-	-	-	-	-	-	-	-	-	-	-	-	-	-	-	-	-	-	-	-	-	-	-	-	-	-	-	-	-	-	-	-	-	-	+	-	-	-	-	-	-	-	-	-	-	-	-	-	-	-	-
4790	5330	putative repressor protein	1	-	-	-	-	-	-	-	-	-	-	-	-	-	-	-	-	-	-	-	-	-	-	-	-	-	-	-	-	-	-	-	-	-	-	-	-	-	-	-	-	-	-	-	-	-	-	-	-	-	-	-	-	+	-	-	-	-
4791	5339	putative histidine kinase, ScnK homolog	1	-	-	-	-	-	-	-	-	-	-	-	-	-	-	-	-	-	-	-	-	-	-	-	-	-	-	-	-	-	-	-	-	-	-	-	-	-	-	-	-	-	-	-	-	-	-	-	-	-	+	-	-	-	-	-	-	-
4792	5362	hypothetical protein	1	-	-	-	-	-	-	-	-	-	-	-	-	-	-	-	-	-	-	-	-	-	-	-	-	-	-	-	-	-	-	-	-	-	-	-	-	-	-	-	-	-	-	-	-	-	-	-	-	-	+	-	-	-	-	-	-	-
4793	5371	Beta-1,3-glucosyltransferase	1	-	-	-	-	-	-	-	-	-	-	-	-	-	-	+	-	-	-	-	-	-	-	-	-	-	-	-	-	-	-	-	-	-	-	-	-	-	-	-	-	-	-	-	-	-	-	-	-	-	-	-	-	-	-	-	-	-
4794	5399	probable integrase	1	-	-	-	-	-	-	-	-	-	-	-	-	-	-	-	-	-	-	-	-	-	-	-	-	-	-	-	-	-	-	-	-	-	-	-	+	-	-	-	-	-	-	-	-	-	-	-	-	-	-	-	-	-	-	-	-	-
4795	5419	Tn5252, relaxase	1	-	-	-	-	-	-	-	-	-	-	-	-	-	-	-	-	-	-	-	-	-	-	-	-	-	-	-	-	-	-	-	-	-	-	-	-	-	-	-	-	-	-	-	-	-	-	-	-	+	-	-	-	-	-	-	-	-
4796	5423	DNA-cytosine methyltransferase (EC 2.1.1.37)	1	-	-	-	-	-	-	-	-	-	-	-	-	-	-	-	-	-	-	-	-	-	-	-	-	-	-	-	-	-	-	-	-	-	-	-	-	+	-	-	-	-	-	-	-	-	-	-	-	-	-	-	-	-	-	-	-	-
4797	5424	hypothetical protein	1	-	-	-	-	-	-	-	-	-	-	-	-	-	-	-	-	-	-	-	-	-	-	-	+	-	-	-	-	-	-	-	-	-	-	-	-	-	-	-	-	-	-	-	-	-	-	-	-	-	-	-	-	-	-	-	-	-
4798	5433	Transcriptional regulator	1	-	-	-	-	-	-	-	-	-	-	-	-	-	-	-	-	-	-	-	-	-	-	-	-	-	-	-	-	-	-	-	-	-	-	-	-	-	-	-	-	-	-	+	-	-	-	-	-	-	-	-	-	-	-	-	-	-
4799	5438	hypothetical protein	1	-	-	-	-	-	-	-	-	-	-	-	-	-	-	-	-	-	-	-	-	-	-	-	-	-	-	-	-	-	-	-	-	-	-	-	-	-	-	-	-	-	-	-	-	-	-	-	-	-	-	-	+	-	-	-	-	-
4800	5451	hypothetical protein	1	-	-	-	-	-	-	-	-	-	-	-	-	-	-	-	-	-	-	-	-	-	-	-	-	-	-	-	-	-	-	-	-	-	-	-	-	-	-	-	-	-	-	-	-	-	-	-	-	-	-	-	-	-	-	+	-	-
4801	5460	probable membrane protein yetF	1	-	-	-	-	-	-	-	-	-	-	-	-	-	-	-	-	-	-	-	-	-	-	-	-	-	-	-	-	-	-	-	-	-	-	-	-	-	-	-	-	-	-	-	-	-	-	-	-	-	-	+	-	-	-	-	-	-
4802	5463	hypothetical protein	1	-	-	-	-	-	-	-	-	-	-	-	-	-	-	-	-	-	-	-	-	-	-	-	-	-	-	-	-	-	-	-	-	-	-	-	-	-	-	-	-	-	-	-	-	-	-	-	-	-	-	+	-	-	-	-	-	-
4803	5464	replication protein	1	-	-	-	-	-	-	-	-	-	-	-	-	-	-	-	-	-	-	-	-	-	-	-	-	-	-	-	-	-	-	-	-	-	-	-	-	-	-	-	-	-	-	+	-	-	-	-	-	-	-	-	-	-	-	-	-	-
4804	5470	Huntington interacting protein HYPE	1	-	-	-	-	-	-	-	-	-	-	-	-	-	-	-	-	-	-	-	-	-	-	-	-	-	-	-	-	-	-	-	-	-	-	-	-	-	-	-	-	-	-	-	-	-	-	-	-	-	-	-	+	-	-	-	-	-
4805	5473	hypothetical protein	1	-	-	-	-	-	-	-	-	-	-	-	-	-	-	-	-	-	-	-	-	-	-	-	-	-	-	-	-	-	-	-	-	-	-	-	-	-	-	-	-	-	-	-	-	-	-	+	-	-	-	-	-	-	-	-	-	-
4806	5482	hypothetical protein	1	-	-	-	-	-	-	-	-	-	-	-	-	-	-	-	-	-	-	-	-	-	-	-	-	-	-	-	-	-	-	-	-	-	-	-	-	-	-	-	-	-	-	+	-	-	-	-	-	-	-	-	-	-	-	-	-	-
4807	5486	hypothetical protein	1	-	-	-	-	-	-	-	-	-	-	-	-	-	-	-	-	-	-	-	-	-	-	-	-	-	-	-	-	-	-	-	-	-	-	-	-	-	-	-	-	-	-	-	-	-	-	-	-	-	-	-	-	+	-	-	-	-
4808	5487	hypothetical protein	1	-	-	-	-	-	-	-	-	-	-	-	-	-	-	-	-	-	-	-	-	-	-	-	-	-	-	-	-	-	-	-	-	-	-	-	-	-	-	-	-	-	-	-	-	-	-	-	-	-	-	-	-	-	-	-	+	-
4809	5488	Transposase	1	-	-	-	-	-	-	-	-	-	-	-	-	-	-	-	-	-	-	-	-	-	-	-	-	-	-	-	-	-	-	-	-	-	-	-	-	-	-	-	-	-	-	-	-	-	-	-	-	-	-	+	-	-	-	-	-	-
4810	5489	Glycosyl transferase, family 8	1	-	-	-	-	-	-	-	-	-	-	-	-	-	-	-	-	-	-	-	-	-	-	-	-	-	-	-	-	-	-	-	-	-	-	-	-	-	-	-	-	-	-	-	-	-	-	-	-	-	-	+	-	-	-	-	-	-
4811	5490	hypothetical protein	1	-	-	-	-	-	-	-	-	-	-	-	-	-	-	-	-	-	-	-	-	-	-	-	-	-	-	-	-	-	-	-	-	-	-	-	-	-	-	-	-	-	-	-	-	-	-	-	-	-	-	+	-	-	-	-	-	-
4812	5502	hypothetical protein	1	-	-	-	-	-	-	-	-	-	-	-	-	-	-	-	-	-	-	-	-	-	-	-	-	-	-	-	-	-	-	-	-	-	-	-	-	-	-	-	-	-	-	-	-	-	-	-	-	-	-	-	-	-	+	-	-	-
4813	5504	hypothetical protein	1	-	-	-	-	-	-	-	-	-	-	-	-	-	-	-	-	-	-	-	-	-	-	-	-	-	-	-	-	-	-	-	-	-	-	-	-	-	-	-	-	-	-	+	-	-	-	-	-	-	-	-	-	-	-	-	-	-
4814	5506	hypothetical protein	1	-	-	-	-	-	-	-	-	-	-	-	-	-	-	-	-	-	-	-	-	-	-	-	-	-	-	-	-	-	-	-	-	-	-	-	-	-	-	-	-	-	-	+	-	-	-	-	-	-	-	-	-	-	-	-	-	-
4815	5507	hypothetical protein	1	-	-	-	-	-	-	-	-	-	-	-	-	-	-	-	-	-	-	-	-	-	-	-	-	-	-	-	-	-	-	-	-	-	-	-	-	-	-	-	-	-	-	-	-	-	-	-	-	-	-	-	-	-	-	-	+	-
4816	5508	major facilitator superfamily protein	1	-	-	-	-	-	-	-	-	-	-	-	-	-	-	-	-	-	-	-	-	-	-	-	-	-	-	-	-	-	-	-	-	-	-	-	-	-	-	-	-	-	-	-	-	-	-	-	-	-	-	-	-	-	-	-	+	-
4817	5720	Mobile element protein	1	-	-	-	-	-	-	-	-	-	-	-	-	-	-	-	-	-	-	-	+	-	-	-	-	-	-	-	-	-	-	-	-	-	-	-	-	-	-	-	-	-	-	-	-	-	-	-	-	-	-	-	-	-	-	-	-	-
4818	5734	endonuclease of the HNH family	1	-	-	-	-	-	-	-	-	+	-	-	-	-	-	-	-	-	-	-	-	-	-	-	-	-	-	-	-	-	-	-	-	-	-	-	-	-	-	-	-	-	-	-	-	-	-	-	-	-	-	-	-	-	-	-	-	-
4819	5749	Putrescine transport ATP-binding protein PotA (TC 3.A.1.11.1)	1	-	-	-	-	-	-	-	-	-	-	-	-	-	-	-	-	-	-	-	-	-	-	-	+	-	-	-	-	-	-	-	-	-	-	-	-	-	-	-	-	-	-	-	-	-	-	-	-	-	-	-	-	-	-	-	-	-
4820	5898	DNA-methyltransferase	1	-	-	-	-	-	-	-	-	-	-	-	-	-	-	-	-	-	-	-	-	-	-	-	-	-	-	-	-	-	-	-	-	-	-	-	-	-	-	-	-	-	-	-	-	-	-	-	-	+	-	-	-	-	-	-	-	-
4821	5974	ABC transporter ATP-binding protein YvcR	1	-	-	-	-	-	-	-	-	-	-	-	-	-	-	-	-	-	-	-	-	-	-	-	-	-	-	-	-	-	-	-	-	-	-	-	-	-	-	-	-	-	-	-	-	-	-	-	+	-	-	-	-	-	-	-	-	-
4822	5999	hypothetical protein	1	-	-	-	-	-	-	-	-	-	-	-	-	-	-	-	-	-	-	-	-	-	-	-	-	+	-	-	-	-	-	-	-	-	-	-	-	-	-	-	-	-	-	-	-	-	-	-	-	-	-	-	-	-	-	-	-	-
4823	6044	ABC transporter permease protein	1	-	-	-	-	-	-	-	-	-	-	-	-	-	-	-	-	-	-	-	-	-	-	-	-	-	-	-	-	-	-	-	-	-	-	-	-	-	-	-	+	-	-	-	-	-	-	-	-	-	-	-	-	-	-	-	-	-
4824	6046	hypothetical protein	1	-	-	-	-	-	-	-	-	-	-	-	-	-	-	-	-	-	-	-	-	-	-	-	-	-	-	-	-	-	-	-	-	-	-	-	-	-	-	-	+	-	-	-	-	-	-	-	-	-	-	-	-	-	-	-	-	-
4825	6062	DNA-binding protein	1	-	-	-	-	-	-	-	-	-	-	-	-	-	-	-	+	-	-	-	-	-	-	-	-	-	-	-	-	-	-	-	-	-	-	-	-	-	-	-	-	-	-	-	-	-	-	-	-	-	-	-	-	-	-	-	-	-
4826	6072	hypothetical protein	1	-	-	-	-	-	-	-	-	-	-	-	-	-	-	-	+	-	-	-	-	-	-	-	-	-	-	-	-	-	-	-	-	-	-	-	-	-	-	-	-	-	-	-	-	-	-	-	-	-	-	-	-	-	-	-	-	-
4827	6075	Chaperone protein DnaK	1	-	-	-	-	-	-	-	-	-	-	-	-	-	-	-	-	-	-	-	-	-	-	-	-	-	-	-	-	-	-	-	-	-	-	-	-	-	-	-	-	-	-	-	-	-	-	-	-	-	-	-	-	-	-	-	-	+
4828	6078	hypothetical protein	1	-	-	-	-	-	-	-	-	-	-	-	-	-	-	-	-	-	-	-	-	-	-	-	-	-	-	-	-	-	-	-	-	-	-	-	-	-	-	-	-	-	-	-	-	-	-	-	-	-	-	-	-	-	-	-	-	+
4829	6093	hypothetical protein	1	-	-	-	-	-	-	-	-	-	-	-	-	-	-	-	-	-	-	-	-	-	-	-	-	-	-	-	-	-	-	-	-	-	-	-	-	-	-	-	-	-	-	-	-	-	-	-	+	-	-	-	-	-	-	-	-	-
4830	6094	DNA segregation ATPase FtsK/SpoIIIE related protein	1	-	-	-	-	-	-	-	-	-	-	-	-	-	-	-	-	-	-	-	-	-	-	-	-	-	-	-	-	-	-	-	-	-	-	-	-	-	-	-	-	-	-	-	-	-	-	-	+	-	-	-	-	-	-	-	-	-
4831	6097	Putative EsaC protein analog (Listeria type 3)	1	-	-	-	-	-	-	-	-	-	-	-	-	-	-	-	-	-	-	-	-	-	-	-	-	-	-	-	-	-	-	-	-	-	-	-	-	-	-	-	-	-	+	-	-	-	-	-	-	-	-	-	-	-	-	-	-	-
4832	6110	hypothetical protein	1	-	-	-	-	-	-	-	-	-	-	-	-	-	-	-	-	-	-	-	-	-	-	-	-	-	-	-	-	-	-	-	-	-	-	-	-	-	-	-	-	-	-	-	-	-	-	-	+	-	-	-	-	-	-	-	-	-
4833	6116	Predicted membrane protein	1	-	-	-	-	-	-	-	-	-	-	-	-	-	-	-	-	-	-	-	-	-	-	-	-	-	-	-	-	-	-	-	-	-	-	-	-	-	-	-	-	-	+	-	-	-	-	-	-	-	-	-	-	-	-	-	-	-
4834	6124	YwqJ	1	-	-	-	-	-	-	-	-	-	-	-	-	-	-	-	-	-	-	-	-	-	-	-	-	-	-	-	-	-	-	-	-	-	-	-	-	-	-	-	-	-	+	-	-	-	-	-	-	-	-	-	-	-	-	-	-	-
4835	6127	hypothetical protein	1	-	-	-	-	-	-	-	-	-	-	-	-	-	-	-	-	-	-	-	-	-	-	-	-	-	-	-	-	-	-	-	-	-	-	-	-	-	-	-	-	-	+	-	-	-	-	-	-	-	-	-	-	-	-	-	-	-
4836	6131	hypothetical protein	1	-	-	-	-	-	-	-	-	-	-	-	-	-	-	-	-	-	-	-	-	-	-	-	-	-	-	-	-	-	-	-	-	-	-	-	-	-	-	-	-	-	+	-	-	-	-	-	-	-	-	-	-	-	-	-	-	-
4837	6135	Transcriptional regulator, MerR family	1	-	-	-	-	-	-	-	-	-	-	-	-	-	-	-	-	-	-	-	-	-	-	-	-	-	-	-	-	-	-	-	-	-	-	-	-	-	-	-	-	-	+	-	-	-	-	-	-	-	-	-	-	-	-	-	-	-
4838	6152	Superfamily I DNA/RNA helicase protein	1	-	-	-	-	-	-	-	-	-	-	-	-	-	-	-	-	-	-	-	-	-	-	-	-	-	-	-	-	-	-	-	-	-	-	-	-	-	-	-	-	+	-	-	-	-	-	-	-	-	-	-	-	-	-	-	-	-
4839	6154	hypothetical protein	1	-	-	-	-	-	-	-	-	-	-	-	-	-	-	-	-	-	-	-	-	-	-	-	-	-	-	-	-	-	-	-	-	-	-	-	-	-	-	-	-	+	-	-	-	-	-	-	-	-	-	-	-	-	-	-	-	-
4840	6158	Integrase	1	-	-	-	-	-	-	-	-	-	-	-	-	-	-	-	-	-	-	-	-	-	-	-	-	-	-	-	-	-	-	-	-	-	-	-	-	-	-	-	-	+	-	-	-	-	-	-	-	-	-	-	-	-	-	-	-	-
4841	6163	Ribulose-phosphate 3-epimerase (EC 5.1.3.1)	1	-	-	-	-	-	-	-	-	-	-	-	-	-	-	-	-	-	-	-	-	-	-	-	-	-	-	-	-	-	-	-	-	-	-	-	-	-	-	-	-	-	-	-	-	-	-	-	-	-	+	-	-	-	-	-	-	-
4842	6165	Phage portal protein	1	-	-	-	-	-	-	-	-	-	-	-	-	-	-	-	-	-	-	-	-	-	-	-	-	-	-	-	-	-	-	-	-	-	-	-	-	-	-	-	-	-	-	-	-	-	-	-	-	-	+	-	-	-	-	-	-	-
4843	6170	Type I restriction-modification system, DNA-methyltransferase subunit M (EC 2.1.1.72)	1	-	-	-	-	-	-	-	-	-	-	-	-	-	-	-	-	-	-	-	-	-	-	-	-	-	-	-	-	-	-	-	-	-	-	-	-	-	-	-	-	-	-	-	-	-	-	-	-	-	+	-	-	-	-	-	-	-
4844	6175	putative ABC transporter membrane-spanning subunit	1	-	-	-	-	-	-	-	-	-	-	-	-	-	-	-	-	-	-	-	-	-	-	-	-	-	-	-	-	-	-	-	-	-	-	-	-	-	-	-	-	-	-	-	-	-	-	-	-	-	+	-	-	-	-	-	-	-
4845	6177	Unknown pentose isomerase ECA1953	1	-	-	-	-	-	-	-	-	-	-	-	-	-	-	-	-	-	-	-	-	-	-	-	-	-	-	-	-	-	-	-	-	-	-	-	-	-	-	-	-	-	-	-	-	-	-	-	-	-	+	-	-	-	-	-	-	-
4846	6180	hypothetical protein	1	-	-	-	-	-	-	-	-	-	-	-	-	-	-	-	-	-	-	-	-	-	-	-	-	-	-	-	-	-	-	-	-	-	-	-	-	-	-	-	-	-	-	-	-	-	-	-	-	-	-	-	-	-	+	-	-	-
4847	6183	Ribose 5-phosphate isomerase B (EC 5.3.1.6)	1	-	-	-	-	-	-	-	-	-	-	-	-	-	-	-	-	-	-	-	-	-	-	-	-	-	-	-	-	-	-	-	-	-	-	-	-	-	-	-	-	-	-	-	-	-	-	-	-	-	+	-	-	-	-	-	-	-
4848	6185	PTS system, beta-glucoside-specific IIB component (EC 2.7.1.69) / PTS system, beta-glucoside-specific IIC component (EC 2.7.1.69) / PTS system, beta-glucoside-specific IIA component (EC 2.7.1.69)	1	-	-	-	-	-	-	-	-	-	-	-	-	-	-	-	-	-	-	-	-	-	-	-	-	-	-	-	-	-	-	-	-	-	-	-	-	-	-	-	-	-	-	-	-	-	-	-	-	-	+	-	-	-	-	-	-	-
4849	6187	Type I restriction-modification system, specificity subunit S (EC 3.1.21.3)	1	-	-	-	-	-	-	-	-	-	-	-	-	-	-	-	-	-	-	-	-	-	-	-	-	-	-	-	-	-	-	-	-	-	-	-	-	-	-	-	-	-	-	-	-	-	-	-	-	-	+	-	-	-	-	-	-	-
4850	6190	transcriptional regulator	1	-	-	-	-	-	-	-	-	-	-	-	-	-	-	-	-	-	-	-	-	-	-	-	-	-	-	-	-	-	-	-	-	-	-	-	-	-	-	-	-	-	-	-	-	-	-	-	-	-	+	-	-	-	-	-	-	-
4851	6195	hypothetical protein	1	-	-	-	-	-	-	-	-	-	-	-	-	-	-	-	-	-	-	-	-	-	-	-	-	-	-	-	-	-	-	-	-	-	-	-	-	-	-	-	-	-	-	-	-	-	-	-	-	-	+	-	-	-	-	-	-	-
4852	6197	putative ABC transporter ATP-binding protein	1	-	-	-	-	-	-	-	-	-	-	-	-	-	-	-	-	-	-	-	-	-	-	-	-	-	-	-	-	-	-	-	-	-	-	-	-	-	-	-	-	-	-	-	-	-	-	-	-	-	+	-	-	-	-	-	-	-
4853	6206	Transcriptional regulator, GntR family	1	-	-	-	-	-	-	-	-	-	-	-	-	-	-	-	-	-	-	-	-	-	-	-	-	-	-	-	-	-	-	-	-	-	-	-	-	-	-	-	-	-	-	-	-	+	-	-	-	-	-	-	-	-	-	-	-	-
4854	6211	DNA-cytosine methyltransferase	1	-	-	-	-	-	-	-	-	-	-	-	-	-	-	-	-	-	-	-	-	-	-	-	-	-	-	-	-	-	-	-	-	-	-	-	-	-	-	-	-	-	-	-	-	+	-	-	-	-	-	-	-	-	-	-	-	-
4855	6218	hypothetical protein	1	-	-	-	-	-	-	-	-	-	-	-	-	-	-	-	-	-	-	-	-	-	-	-	-	-	-	-	-	-	-	-	-	-	-	-	-	-	-	-	-	-	-	-	-	+	-	-	-	-	-	-	-	-	-	-	-	-
4856	6227	hypothetical protein	1	-	-	-	-	-	-	-	-	-	-	-	-	-	-	-	-	-	-	-	-	-	-	-	-	-	-	-	-	-	-	-	-	-	-	-	-	-	-	-	-	-	-	-	-	+	-	-	-	-	-	-	-	-	-	-	-	-
4857	6231	ABC transporter membrane-spanning permease - glutamine transport	1	-	-	-	-	-	-	-	-	-	-	-	-	-	-	-	-	-	-	-	-	-	-	-	-	-	-	-	-	-	-	-	-	-	-	-	+	-	-	-	-	-	-	-	-	-	-	-	-	-	-	-	-	-	-	-	-	-
4858	6268	Unknown, predicted membrane protein (TMS3)	1	-	-	-	-	-	-	-	-	-	-	-	-	-	-	-	-	-	-	-	-	-	-	-	-	-	-	-	-	-	-	-	-	-	-	-	-	-	+	-	-	-	-	-	-	-	-	-	-	-	-	-	-	-	-	-	-	-
4859	6274	ABC transporter, ATP-binding/permease protein	1	-	-	-	-	-	-	-	-	-	-	-	-	-	-	-	-	-	-	-	-	-	-	-	-	-	-	-	-	-	-	-	-	-	-	-	-	-	+	-	-	-	-	-	-	-	-	-	-	-	-	-	-	-	-	-	-	-
4860	6275	Type I restriction-modification system, specificity subunit S (EC 3.1.21.3)	1	-	-	-	-	-	-	-	-	-	-	-	-	-	-	-	-	-	-	-	-	-	-	-	-	-	-	-	-	-	-	-	-	-	-	-	-	-	-	-	-	-	-	-	+	-	-	-	-	-	-	-	-	-	-	-	-	-
4861	6276	putative HsdR	1	-	-	-	-	-	-	-	-	-	-	-	-	-	-	-	-	-	-	-	-	-	-	-	-	-	-	-	-	-	-	-	-	-	-	-	-	+	-	-	-	-	-	-	-	-	-	-	-	-	-	-	-	-	-	-	-	-
4862	6290	Distantly related to cell wall-associated hydrolase	1	-	-	-	-	-	-	-	-	-	-	-	-	-	-	-	-	-	-	-	-	-	-	-	-	-	-	-	-	-	-	-	-	-	-	-	-	-	-	-	-	-	-	-	-	-	-	-	-	-	-	+	-	-	-	-	-	-
4863	6312	hypothetical protein	1	-	-	-	-	-	-	-	-	-	-	-	-	-	-	-	-	-	-	-	-	-	-	-	-	-	-	-	-	-	-	-	-	-	-	-	-	-	-	-	-	-	-	+	-	-	-	-	-	-	-	-	-	-	-	-	-	-
4864	6317	hypothetical protein	1	-	-	-	-	-	-	-	-	-	-	-	-	-	-	-	-	-	-	-	-	-	-	-	-	-	-	-	-	-	-	-	-	-	-	-	-	-	-	-	-	-	-	-	-	-	-	-	-	-	-	+	-	-	-	-	-	-
4865	6326	putative lipase	1	-	-	-	-	-	-	-	-	-	-	-	-	-	-	-	-	-	-	-	-	-	-	-	-	-	-	-	-	-	-	-	-	-	-	-	-	-	-	-	-	-	-	-	-	-	-	-	-	-	-	+	-	-	-	-	-	-
4866	6330	hypothetical protein	1	-	-	-	-	-	-	-	-	-	-	-	-	-	-	-	-	-	-	-	-	-	-	-	-	-	-	-	-	-	-	-	-	-	-	-	-	-	-	-	-	-	-	+	-	-	-	-	-	-	-	-	-	-	-	-	-	-
4867	6331	Mannan endo-1,4-beta-mannosidase	1	-	-	-	-	-	-	-	-	-	-	-	-	-	-	-	-	-	-	-	-	-	-	-	-	-	-	-	-	-	-	-	-	-	-	-	-	-	-	-	-	-	-	-	-	-	-	-	-	-	-	+	-	-	-	-	-	-
4868	6355	Phage terminase large subunit	1	-	-	-	-	-	-	-	-	-	-	-	-	-	-	-	-	-	-	-	-	-	-	-	-	-	-	-	-	-	-	-	-	-	-	-	-	-	-	-	-	-	-	-	-	-	-	-	-	-	-	+	-	-	-	-	-	-
4869	6356	hypothetical protein	1	-	-	-	-	-	-	-	-	-	-	-	-	-	-	-	-	-	-	-	-	-	-	-	-	-	-	-	-	-	-	-	-	-	-	-	-	-	-	-	-	-	-	-	-	-	-	-	-	-	-	-	+	-	-	-	-	-
4870	6357	hypothetical protein	1	-	-	-	-	-	-	-	-	-	-	-	-	-	-	-	-	-	-	-	-	-	-	-	-	-	-	-	-	-	-	-	-	-	-	-	-	-	-	-	-	-	-	-	-	-	-	-	-	-	-	-	+	-	-	-	-	-
4871	6367	hypothetical protein	1	-	-	-	-	-	-	-	-	-	-	-	-	-	-	-	-	-	-	-	-	-	-	-	-	-	-	-	-	-	-	-	-	-	-	-	-	-	-	-	-	-	-	+	-	-	-	-	-	-	-	-	-	-	-	-	-	-
4872	6372	Alpha-L-Rha alpha-1,2-L-rhamnosyltransferase/alpha-L-Rha alpha-1,3-L- rhamnosyltransferase (EC 2.4.1.-)	1	-	-	-	-	-	-	-	-	-	-	-	-	-	-	-	-	-	-	-	-	-	-	-	-	-	-	-	-	-	-	-	-	-	-	-	-	-	-	-	-	-	-	-	-	-	-	-	-	-	-	-	+	-	-	-	-	-
4873	6389	hypothetical protein	1	-	-	-	-	-	-	-	-	-	-	-	-	-	-	-	-	-	-	-	-	-	-	-	-	-	-	-	-	-	-	-	-	+	-	-	-	-	-	-	-	-	-	-	-	-	-	-	-	-	-	-	-	-	-	-	-	-
4874	6404	Modification methylase	1	-	-	-	-	-	-	-	-	-	-	-	-	-	-	-	-	-	-	-	-	-	-	-	-	-	-	-	-	-	-	-	-	-	-	-	-	-	-	-	-	-	-	-	-	-	-	+	-	-	-	-	-	-	-	-	-	-
4875	6407	hypothetical protein	1	-	-	-	-	-	-	-	-	-	-	-	-	-	-	-	-	-	-	-	-	-	-	-	-	-	-	-	-	-	-	-	-	-	-	-	-	-	-	-	-	-	-	-	-	-	-	+	-	-	-	-	-	-	-	-	-	-
4876	6442	Phage protein	1	-	-	-	-	-	-	-	-	-	-	-	-	-	-	-	-	-	-	-	-	-	-	-	-	-	-	-	-	-	-	-	-	-	-	-	-	-	-	-	-	-	-	-	-	-	-	-	-	-	-	+	-	-	-	-	-	-
4877	6443	Type I restriction-modification system, specificity subunit S (EC 3.1.21.3)	1	-	-	-	-	-	-	-	-	-	-	-	-	-	-	-	-	-	-	-	-	-	-	-	-	-	-	-	-	-	-	-	-	-	-	-	-	-	-	-	-	-	-	-	-	-	-	-	-	-	-	+	-	-	-	-	-	-
4878	6444	COG4478, integral membrane protein	1	-	-	-	-	-	-	-	-	-	-	-	-	-	-	-	-	-	-	-	-	-	-	-	-	-	-	-	-	-	-	-	-	-	-	-	-	-	-	-	-	-	-	-	-	-	-	-	-	-	-	+	-	-	-	-	-	-
4879	6445	putative protein Ymh	1	-	-	-	-	-	-	-	-	-	-	-	-	-	-	-	-	-	-	-	-	-	-	-	-	-	-	-	-	-	-	-	-	-	-	-	-	-	-	-	-	-	-	-	+	-	-	-	-	-	-	-	-	-	-	-	-	-
4880	6446	Mannosyl-glycoprotein endo-beta-N-acetylglucosamidase	1	-	-	-	-	-	-	-	-	-	-	-	-	-	-	-	-	-	-	-	-	-	-	-	-	-	-	-	-	-	-	-	-	-	-	-	-	-	-	-	-	-	-	-	+	-	-	-	-	-	-	-	-	-	-	-	-	-
4881	6450	Transcriptional regulator, RpiR family	1	-	-	-	-	-	-	-	-	-	-	-	-	-	-	-	-	-	-	-	-	-	-	-	-	-	-	-	-	-	-	-	-	-	-	-	-	-	-	-	-	-	-	-	-	-	-	-	-	-	-	-	-	-	+	-	-	-
4882	6451	Transcriptional regulator, MarR family	1	-	-	-	-	-	-	-	-	-	-	-	-	-	-	-	-	-	-	-	-	-	-	-	-	-	-	-	-	-	-	-	-	-	-	-	-	-	-	-	-	-	-	-	-	-	-	-	-	-	-	-	-	-	+	-	-	-
4883	6453	Alcohol dehydrogenase (EC 1.1.1.1); Acetaldehyde dehydrogenase (EC 1.2.1.10)	1	-	-	-	-	-	-	-	-	-	-	-	-	-	-	-	-	-	-	-	-	-	-	-	-	-	-	-	-	-	-	-	-	-	-	-	-	-	-	-	-	-	-	-	-	-	-	-	-	-	-	-	-	-	-	-	+	-
4884	6454	hypothetical protein	1	-	-	-	-	-	-	-	-	-	-	-	-	-	-	-	-	-	-	-	-	-	-	-	-	-	-	-	-	-	-	-	-	-	-	-	-	-	-	-	-	-	-	-	-	-	-	-	-	-	-	-	-	-	-	-	+	-
4885	6455	hypothetical protein	1	-	-	-	-	-	-	-	-	-	-	-	-	-	-	-	-	-	-	-	-	-	-	-	-	-	-	-	-	-	-	-	-	-	-	-	-	-	-	-	-	-	-	-	-	-	-	-	-	-	-	-	-	-	-	-	+	-
4886	6458	Phage integrase (Site-specific recombinase)	1	-	-	-	-	-	-	-	-	-	-	-	-	-	-	-	-	-	-	-	-	-	-	-	-	-	-	-	-	-	-	-	-	-	-	-	-	-	-	-	-	-	-	-	-	-	-	-	-	-	-	-	-	-	-	-	+	-
4887	6487	Dienelactone hydrolase and related enzymes	1	-	-	-	-	-	-	-	-	-	-	-	-	-	-	-	-	-	-	+	-	-	-	-	-	-	-	-	-	-	-	-	-	-	-	-	-	-	-	-	-	-	-	-	-	-	-	-	-	-	-	-	-	-	-	-	-	-
4888	6503	DNA-cytosine methyltransferase (EC 2.1.1.37)	1	-	-	-	-	-	-	-	-	-	-	-	-	-	-	-	-	-	-	-	-	-	-	-	-	-	-	-	-	-	-	-	-	-	-	-	-	-	-	-	-	-	-	-	-	-	-	-	-	-	-	-	-	+	-	-	-	-
4889	6505	Integrase	1	-	-	-	-	-	-	-	-	-	+	-	-	-	-	-	-	-	-	-	-	-	-	-	-	-	-	-	-	-	-	-	-	-	-	-	-	-	-	-	-	-	-	-	-	-	-	-	-	-	-	-	-	-	-	-	-	-
4890	6508	type II restriction enzyme eco47ii	1	-	-	-	-	-	-	-	-	-	-	-	-	-	-	-	-	-	-	-	-	-	-	-	-	-	-	-	-	-	-	-	-	-	-	-	-	-	-	-	-	-	-	-	-	-	-	-	-	-	-	-	-	+	-	-	-	-
4891	6519	ABC transporter permease protein	1	-	-	-	-	-	-	-	-	-	-	-	-	-	-	-	-	-	-	-	-	-	-	-	-	-	-	-	-	-	-	-	-	-	-	-	-	-	-	-	-	-	-	-	-	-	-	-	-	+	-	-	-	-	-	-	-	-
4892	6523	Prophage Clp protease-like protein	1	-	-	-	-	-	-	-	-	-	-	-	-	-	-	-	-	-	-	-	-	-	-	-	-	-	-	-	-	-	-	-	-	-	-	+	-	-	-	-	-	-	-	-	-	-	-	-	-	-	-	-	-	-	-	-	-	-
4893	6530	transcriptional regulator, MerR family	1	-	-	-	-	-	-	-	-	-	-	-	-	-	-	-	-	-	-	+	-	-	-	-	-	-	-	-	-	-	-	-	-	-	-	-	-	-	-	-	-	-	-	-	-	-	-	-	-	-	-	-	-	-	-	-	-	-
4894	6532	hypothetical protein	1	-	-	-	-	-	-	-	-	-	-	-	-	-	-	-	-	-	-	+	-	-	-	-	-	-	-	-	-	-	-	-	-	-	-	-	-	-	-	-	-	-	-	-	-	-	-	-	-	-	-	-	-	-	-	-	-	-
4895	6534	ABC transporter, ATP-binding/permease protein	1	-	-	-	-	-	-	-	-	-	-	-	-	-	-	-	-	-	-	-	-	-	-	-	-	-	-	-	-	-	-	-	-	-	+	-	-	-	-	-	-	-	-	-	-	-	-	-	-	-	-	-	-	-	-	-	-	-
4896	6541	Phage protein	1	-	-	-	-	-	-	-	-	-	-	-	-	-	-	-	-	-	-	-	-	-	-	-	-	-	-	-	-	-	-	-	-	-	-	+	-	-	-	-	-	-	-	-	-	-	-	-	-	-	-	-	-	-	-	-	-	-
4897	6542	Phage terminase, large subunit	1	-	-	-	-	-	-	-	-	-	-	-	-	-	-	-	-	-	-	-	-	-	-	-	-	-	-	-	-	-	-	-	-	-	-	+	-	-	-	-	-	-	-	-	-	-	-	-	-	-	-	-	-	-	-	-	-	-
4898	6543	DNA packaging protein, phage associated	1	-	-	-	-	-	-	-	-	-	-	-	-	-	-	-	-	-	-	-	-	-	-	-	-	-	-	-	-	-	-	-	-	-	-	+	-	-	-	-	-	-	-	-	-	-	-	-	-	-	-	-	-	-	-	-	-	-
4899	6544	Phage protein	1	-	-	-	-	-	-	-	-	-	-	-	-	-	-	-	-	-	-	-	-	-	-	-	-	-	-	-	-	-	-	-	-	-	-	+	-	-	-	-	-	-	-	-	-	-	-	-	-	-	-	-	-	-	-	-	-	-
4900	6545	Phage portal protein	1	-	-	-	-	-	-	-	-	-	-	-	-	-	-	-	-	-	-	-	-	-	-	-	-	-	-	-	-	-	-	-	-	-	-	+	-	-	-	-	-	-	-	-	-	-	-	-	-	-	-	-	-	-	-	-	-	-
4901	6546	Phage capsid protein	1	-	-	-	-	-	-	-	-	-	-	-	-	-	-	-	-	-	-	-	-	-	-	-	-	-	-	-	-	-	-	-	-	-	-	+	-	-	-	-	-	-	-	-	-	-	-	-	-	-	-	-	-	-	-	-	-	-
4902	6548	Phage protein	1	-	-	-	-	-	-	-	-	-	-	-	-	-	-	-	-	-	-	-	-	-	-	-	-	-	-	-	-	-	-	-	-	-	-	+	-	-	-	-	-	-	-	-	-	-	-	-	-	-	-	-	-	-	-	-	-	-
4903	6549	Phage major tail protein	1	-	-	-	-	-	-	-	-	-	-	-	-	-	-	-	-	-	-	-	-	-	-	-	-	-	-	-	-	-	-	-	-	-	-	+	-	-	-	-	-	-	-	-	-	-	-	-	-	-	-	-	-	-	-	-	-	-
4904	6550	Prophage Clp protease-like protein	1	-	-	-	-	-	-	-	-	-	-	-	-	-	-	-	-	-	-	-	-	-	-	-	-	-	-	-	-	-	-	-	-	-	-	+	-	-	-	-	-	-	-	-	-	-	-	-	-	-	-	-	-	-	-	-	-	-
4905	6717	Threonine synthase (EC 4.2.3.1)	1	-	-	-	-	-	-	-	-	-	-	-	-	-	-	-	-	-	-	-	-	-	-	-	-	-	+	-	-	-	-	-	-	-	-	-	-	-	-	-	-	-	-	-	-	-	-	-	-	-	-	-	-	-	-	-	-	-
4906	6876	ORF065	1	-	-	-	-	-	-	-	-	-	-	-	-	-	-	-	-	-	-	-	-	-	-	-	-	-	-	-	-	-	-	-	-	-	-	-	-	-	-	-	-	-	-	-	-	-	-	-	-	-	-	-	-	+	-	-	-	-
4907	6907	hypothetical protein	1	-	-	-	-	-	-	-	-	-	-	-	-	-	-	-	-	-	-	-	-	-	-	-	-	-	-	-	-	-	-	-	-	-	-	-	-	-	-	-	-	-	-	-	-	-	-	-	-	-	-	-	-	+	-	-	-	-
4908	6962	hypothetical protein	1	-	-	-	-	-	-	-	-	-	-	-	-	-	-	-	-	-	-	-	-	-	-	-	-	-	-	-	-	-	-	-	-	-	-	-	-	-	-	-	-	-	-	-	-	-	-	-	-	-	-	-	-	+	-	-	-	-
4909	7139	DNA, complete genome	1	-	-	-	-	-	-	-	-	-	-	-	-	-	-	-	-	-	-	-	-	-	-	-	-	-	-	-	-	-	-	-	-	-	-	-	-	-	-	-	-	-	-	-	-	-	-	-	-	-	-	-	-	+	-	-	-	-
4910	7222	ABC transporter, substrate-binding protein	1	-	-	-	-	-	-	-	-	-	-	-	-	-	-	-	-	-	-	-	-	-	-	-	+	-	-	-	-	-	-	-	-	-	-	-	-	-	-	-	-	-	-	-	-	-	-	-	-	-	-	-	-	-	-	-	-	-
4911	7223	Ferric iron ABC transporter, permease protein	1	-	-	-	-	-	-	-	-	-	-	-	-	-	-	-	-	-	-	-	-	-	-	-	+	-	-	-	-	-	-	-	-	-	-	-	-	-	-	-	-	-	-	-	-	-	-	-	-	-	-	-	-	-	-	-	-	-
4912	7354	Signal peptidase I (EC 3.4.21.89)	1	-	-	-	-	-	-	-	-	-	-	-	-	-	-	-	-	-	-	-	-	-	-	-	-	-	-	-	-	-	-	-	-	-	-	-	-	-	-	-	+	-	-	-	-	-	-	-	-	-	-	-	-	-	-	-	-	-
4913	7381	hypothetical protein	1	-	-	-	-	-	-	-	-	-	-	-	-	-	-	-	-	-	-	-	-	-	-	-	-	+	-	-	-	-	-	-	-	-	-	-	-	-	-	-	-	-	-	-	-	-	-	-	-	-	-	-	-	-	-	-	-	-
4914	7384	hypothetical protein	1	-	-	-	-	-	-	-	-	-	-	-	-	-	-	-	-	-	-	-	-	-	-	-	-	-	-	-	-	-	-	-	-	-	-	-	-	-	-	+	-	-	-	-	-	-	-	-	-	-	-	-	-	-	-	-	-	-
4915	7394	hypothetical protein	1	-	-	-	-	-	-	-	-	-	-	-	-	-	-	-	-	-	-	-	-	-	-	-	-	-	-	-	-	-	-	-	-	-	-	-	-	-	-	+	-	-	-	-	-	-	-	-	-	-	-	-	-	-	-	-	-	-
4916	7415	hypothetical protein	1	-	-	-	-	-	-	-	-	-	-	-	-	-	-	-	-	-	-	-	-	-	-	-	-	-	-	-	-	-	-	-	-	-	-	-	-	-	-	+	-	-	-	-	-	-	-	-	-	-	-	-	-	-	-	-	-	-
4917	7427	Phage protein	1	-	-	-	-	-	-	-	-	-	-	-	-	-	-	-	-	-	-	-	-	-	-	-	-	-	-	-	-	-	-	-	-	-	-	-	-	-	-	+	-	-	-	-	-	-	-	-	-	-	-	-	-	-	-	-	-	-
4918	7432	hypothetical protein	1	-	-	-	-	-	-	-	-	-	-	-	-	-	-	-	-	-	-	-	-	-	-	-	-	-	-	-	-	-	-	-	-	-	-	-	-	-	-	+	-	-	-	-	-	-	-	-	-	-	-	-	-	-	-	-	-	-
4919	7435	hypothetical protein	1	-	-	-	-	-	-	-	-	-	-	-	-	-	-	-	-	-	-	-	-	-	-	-	-	-	-	-	-	-	-	-	-	-	-	-	-	-	-	-	+	-	-	-	-	-	-	-	-	-	-	-	-	-	-	-	-	-
4920	7443	ATP-dependent DNA helicase recG (EC 3.6.1.-)	1	-	-	-	-	-	-	-	-	-	-	-	-	-	-	-	-	-	-	-	-	-	-	-	-	-	-	-	-	-	-	-	-	-	-	-	-	-	-	-	-	-	-	-	-	-	+	-	-	-	-	-	-	-	-	-	-	-
4921	7461	hypothetical protein	1	-	-	-	-	-	-	-	-	-	-	-	-	-	-	-	-	-	-	-	-	-	-	+	-	-	-	-	-	-	-	-	-	-	-	-	-	-	-	-	-	-	-	-	-	-	-	-	-	-	-	-	-	-	-	-	-	-
4922	7464	hypothetical protein	1	-	-	-	-	-	-	-	-	-	-	-	-	-	-	-	-	-	-	-	-	-	-	+	-	-	-	-	-	-	-	-	-	-	-	-	-	-	-	-	-	-	-	-	-	-	-	-	-	-	-	-	-	-	-	-	-	-
4923	7470	ABC transporter ATP-binding protein	1	-	-	-	-	-	-	-	-	-	-	-	-	-	-	-	-	-	-	-	-	-	-	+	-	-	-	-	-	-	-	-	-	-	-	-	-	-	-	-	-	-	-	-	-	-	-	-	-	-	-	-	-	-	-	-	-	-
4924	7471	hypothetical protein	1	-	-	-	-	-	-	-	-	-	-	-	-	-	-	-	-	-	-	-	-	-	-	+	-	-	-	-	-	-	-	-	-	-	-	-	-	-	-	-	-	-	-	-	-	-	-	-	-	-	-	-	-	-	-	-	-	-
4925	7481	hypothetical protein	1	-	-	-	-	-	-	-	-	-	-	-	-	-	-	-	+	-	-	-	-	-	-	-	-	-	-	-	-	-	-	-	-	-	-	-	-	-	-	-	-	-	-	-	-	-	-	-	-	-	-	-	-	-	-	-	-	-
4926	7490	ABC transporter, ATP-binding protein	1	-	-	-	-	-	-	-	-	-	-	-	-	-	-	-	+	-	-	-	-	-	-	-	-	-	-	-	-	-	-	-	-	-	-	-	-	-	-	-	-	-	-	-	-	-	-	-	-	-	-	-	-	-	-	-	-	-
4927	7492	Phage DNA binding protein	1	-	-	-	-	-	-	-	-	-	-	-	-	-	-	-	+	-	-	-	-	-	-	-	-	-	-	-	-	-	-	-	-	-	-	-	-	-	-	-	-	-	-	-	-	-	-	-	-	-	-	-	-	-	-	-	-	-
4928	7499	ABC transporter, ATP-binding protein	1	-	-	-	-	-	-	-	-	-	-	-	-	-	-	-	-	-	-	-	-	-	-	-	-	-	-	-	-	-	-	-	-	-	-	-	-	-	-	-	-	-	-	-	-	-	-	-	-	-	-	-	-	-	-	-	-	+
4929	7500	AcrR protein	1	-	-	-	-	-	-	-	-	-	-	-	-	-	-	-	-	-	-	-	-	-	-	-	-	-	-	-	-	-	-	-	-	-	-	-	-	-	-	-	-	-	-	-	-	-	-	-	-	-	-	-	-	-	-	-	-	+
4930	7501	hypothetical protein	1	-	-	-	-	-	-	-	-	-	-	-	-	-	-	-	-	-	-	-	-	-	-	-	-	-	-	-	-	-	-	-	-	-	-	-	-	-	-	-	-	-	-	-	-	-	-	-	-	-	-	-	-	-	-	-	-	+
4931	7502	hypothetical protein	1	-	-	-	-	-	-	-	-	-	-	-	-	-	-	-	-	-	-	-	-	-	-	-	-	-	-	-	-	-	-	-	-	-	-	-	-	-	-	-	-	-	-	-	-	-	-	-	-	-	-	-	-	-	-	-	-	+
4932	7508	hypothetical protein	1	-	-	-	-	-	-	-	-	-	-	-	-	-	-	-	-	-	-	-	-	-	-	-	-	-	-	-	-	-	-	-	-	-	-	-	-	-	-	-	-	-	-	-	-	-	-	-	-	-	-	-	-	-	-	-	-	+
4933	7514	hypothetical protein	1	-	-	-	-	-	-	-	-	-	-	-	-	-	-	-	-	-	-	-	-	-	-	-	-	-	-	-	-	-	-	-	-	-	-	-	-	-	-	-	-	-	-	-	-	-	-	-	-	-	-	-	-	-	-	-	-	+
4934	7515	hypothetical protein	1	-	-	-	-	-	-	-	-	-	-	-	-	-	-	-	-	-	-	-	-	-	-	-	-	-	-	-	-	-	-	-	-	-	-	-	-	-	-	-	-	-	-	-	-	-	-	-	-	-	-	-	-	-	-	-	-	+
4935	7517	Lipase (EC 3.1.1.3)	1	-	-	-	-	-	-	-	-	-	-	-	-	-	-	-	-	-	-	-	-	-	-	-	-	-	-	-	-	-	-	-	-	-	-	-	-	-	-	-	-	-	-	-	-	-	-	-	-	-	-	-	-	-	-	-	-	+
4936	7535	Transcriptional regulator, HxlR family	1	-	-	-	-	-	-	-	-	-	+	-	-	-	-	-	-	-	-	-	-	-	-	-	-	-	-	-	-	-	-	-	-	-	-	-	-	-	-	-	-	-	-	-	-	-	-	-	-	-	-	-	-	-	-	-	-	-
4937	7538	hypothetical protein	1	-	-	-	-	-	-	-	-	-	-	-	-	-	-	-	-	-	-	-	-	-	-	-	-	-	-	-	-	-	-	-	-	-	-	-	-	-	-	-	-	-	-	-	-	-	-	-	+	-	-	-	-	-	-	-	-	-
4938	7541	hypothetical protein	1	-	-	-	-	-	-	-	-	-	-	-	-	-	-	-	-	-	-	-	-	-	-	-	-	-	-	-	-	-	-	-	-	-	-	-	-	-	-	-	-	-	-	-	-	-	-	-	+	-	-	-	-	-	-	-	-	-
4939	7543	hypothetical protein	1	-	-	-	-	-	-	-	-	-	-	-	-	-	-	-	-	-	-	-	-	-	-	-	-	-	-	-	-	-	-	-	-	-	-	-	-	-	-	-	-	-	-	-	-	-	-	-	+	-	-	-	-	-	-	-	-	-
4940	7552	sepS10A	1	-	-	-	-	-	-	-	-	-	-	-	-	-	-	-	-	-	-	-	-	-	-	-	-	-	-	-	-	-	-	-	-	-	-	-	-	-	-	-	-	-	-	-	-	-	-	-	+	-	-	-	-	-	-	-	-	-
4941	7556	hypothetical protein	1	-	-	-	-	-	-	-	-	-	-	-	-	-	-	-	-	-	-	-	-	-	-	-	-	-	-	-	-	-	-	-	-	-	-	-	-	-	-	-	-	-	-	-	-	-	-	-	+	-	-	-	-	-	-	-	-	-
4942	7558	hypothetical protein	1	-	-	-	-	-	-	-	-	-	-	-	-	-	-	-	-	-	-	-	-	-	-	-	-	-	-	-	-	-	-	-	-	-	-	-	-	-	-	-	-	-	-	-	-	-	-	-	+	-	-	-	-	-	-	-	-	-
4943	7561	hypothetical protein	1	-	-	-	-	-	-	-	-	-	-	-	-	-	-	-	-	-	-	-	-	-	-	-	-	-	-	-	-	-	-	-	-	-	-	-	-	-	-	-	-	-	-	-	-	-	-	-	+	-	-	-	-	-	-	-	-	-
4944	7562	hypothetical protein	1	-	-	-	-	-	-	-	-	-	-	-	-	-	-	-	-	-	-	-	-	-	-	-	-	-	-	-	-	-	-	-	-	-	-	-	-	-	-	-	-	-	-	-	-	-	-	-	+	-	-	-	-	-	-	-	-	-
4945	7564	hypothetical protein	1	-	-	-	-	-	-	-	-	-	-	-	-	-	-	-	-	-	-	-	-	-	-	-	-	-	-	-	-	-	-	-	-	-	-	-	-	-	-	-	-	-	-	+	-	-	-	-	-	-	-	-	-	-	-	-	-	-
4946	7565	hypothetical protein	1	-	-	-	-	-	-	-	-	-	-	-	-	-	-	-	-	-	-	-	-	-	-	-	-	-	-	-	-	-	-	-	-	-	-	-	-	-	-	-	-	-	-	-	-	-	-	-	+	-	-	-	-	-	-	-	-	-
4947	7571	hypothetical protein	1	-	-	-	-	-	-	-	-	-	-	-	-	-	-	-	-	-	-	-	-	-	-	-	-	-	-	-	-	-	-	-	-	-	-	-	-	-	-	-	-	-	-	-	-	-	-	-	+	-	-	-	-	-	-	-	-	-
4948	7577	Glycerol-3-phosphate cytidylyltransferase (EC 2.7.7.39)	1	-	-	-	-	-	-	-	-	-	-	-	-	-	-	-	-	-	-	-	-	-	-	-	-	-	-	-	-	-	-	-	-	-	-	-	-	-	-	-	-	-	-	-	-	-	-	-	+	-	-	-	-	-	-	-	-	-
4949	7584	hypothetical protein	1	-	-	-	-	-	-	-	-	-	-	-	-	-	-	-	-	-	-	-	-	-	-	-	-	-	-	-	-	-	-	-	-	-	-	-	-	-	-	-	-	-	+	-	-	-	-	-	-	-	-	-	-	-	-	-	-	-
4950	7586	hypothetical protein	1	-	-	-	-	-	-	-	-	-	-	-	-	-	-	-	-	-	-	-	-	-	-	-	-	-	-	-	-	-	-	-	-	-	-	-	-	-	-	-	-	-	+	-	-	-	-	-	-	-	-	-	-	-	-	-	-	-
4951	7588	hypothetical protein	1	-	-	-	-	-	-	-	-	-	-	-	-	-	-	-	-	-	-	-	-	-	-	-	-	-	-	-	-	-	-	-	-	-	-	-	-	-	-	-	-	-	+	-	-	-	-	-	-	-	-	-	-	-	-	-	-	-

Table S5. Precence/absence of each HG among 57 *S. suis* strains

				1																13			25					28						104		94			76			53/54		Divergent *S. suis*																
Index[a]	ClutserID	Gene products	Members	98HAH33	05ZYH33	SC84	A7	SS12	P1/7	DAT273	NIAH11434	JS14	GZ1	BM407	S735	13730	MNCM07	NCTC 10446	14636	NCTC 10237	ST1	2651	89-1591	MNCM04	MNCM25	8074	D9	4961	ST3	MNCM43	DAT292	DAT260	NIAH11435	MNCM21	MNCM50	89-2479	DAT234	DAT290	93A	42A	NT77	6407	11538	8830	12814	92-1400	89-590	2726	2524	89-3576-3	4417	D12	10581	89-5259	88-5299A	R61	92-1191	14A	92-4172	22083
4952	7589	hypothetical protein	1	-	-	-	-	-	-	-	-	-	-	-	-	-	-	-	-	-	-	-	-	-	-	-	-	-	-	-	-	-	-	-	-	-	-	-	-	-	+	-	-	-	-	-	-	-	-	-	-	-	-	-	-	-
4953	7593	hypothetical protein	1	-	-	-	-	-	-	-	-	-	-	-	-	-	-	-	-	-	-	-	-	-	-	-	-	-	-	-	-	-	-	-	-	-	-	-	-	-	+	-	-	-	-	-	-	-	-	-	-	-	-	-	-	-
4954	7594	Membrane protein involved in the export of O-antigen, teichoic acid lipoteichoic acids	1	-	-	-	-	-	-	-	-	-	-	-	-	-	-	-	-	-	-	-	-	-	-	-	-	-	-	-	-	-	-	-	-	-	-	-	-	-	+	-	-	-	-	-	-	-	-	-	-	-	-	-	-	-
4955	7599	hypothetical protein	1	-	-	-	-	-	-	-	-	-	-	-	-	-	-	-	-	-	-	-	-	-	-	-	-	-	-	-	-	-	-	-	-	-	-	-	-	-	+	-	-	-	-	-	-	-	-	-	-	-	-	-	-	-
4956	7600	lipoprotein	1	-	-	-	-	-	-	-	-	-	-	-	-	-	-	-	-	-	-	-	-	-	-	-	-	-	-	-	-	-	-	-	-	-	-	-	-	-	+	-	-	-	-	-	-	-	-	-	-	-	-	-	-	-
4957	7602	hypothetical protein	1	-	-	-	-	-	-	-	-	-	-	-	-	-	-	-	-	-	-	-	-	-	-	-	-	-	-	-	-	-	-	-	-	-	-	-	-	-	+	-	-	-	-	-	-	-	-	-	-	-	-	-	-	-
4958	7603	hypothetical protein	1	-	-	-	-	-	-	-	-	-	-	-	-	-	-	-	-	-	-	-	-	-	-	-	-	-	-	-	-	-	-	-	-	-	-	-	-	-	+	-	-	-	-	-	-	-	-	-	-	-	-	-	-	-
4959	7605	hypothetical protein	1	-	-	-	-	-	-	-	-	-	-	-	-	-	-	-	-	-	-	-	-	-	-	-	-	-	-	-	-	-	-	-	-	-	-	-	-	-	+	-	-	-	-	-	-	-	-	-	-	-	-	-	-	-
4960	7610	PROBABLE HEMAGGLUTININ-RELATED PROTEIN	1	-	-	-	-	-	-	-	-	-	-	-	-	-	-	-	-	-	-	-	-	-	-	-	-	-	-	-	-	-	-	-	-	-	-	-	-	-	+	-	-	-	-	-	-	-	-	-	-	-	-	-	-	-
4961	7614	hypothetical protein	1	-	-	-	-	-	-	-	-	-	-	-	-	-	-	-	-	-	-	-	-	-	-	-	-	-	-	-	-	-	-	-	-	-	-	-	-	+	-	-	-	-	-	-	-	-	-	-	-	-	-	-	-	-
4962	7616	hypothetical protein	1	-	-	-	-	-	-	-	-	-	-	-	-	-	-	-	-	-	-	-	-	-	-	-	-	-	-	-	-	-	-	-	-	-	-	-	-	+	-	-	-	-	-	-	-	-	-	-	-	-	-	-	-	-
4963	7618	hypothetical protein	1	-	-	-	-	-	-	-	-	-	-	-	-	-	-	-	-	-	-	-	-	-	-	-	-	-	-	-	-	-	-	-	-	-	-	-	-	+	-	-	-	-	-	-	-	-	-	-	-	-	-	-	-	-
4964	7621	hypothetical protein	1	-	-	-	-	-	-	-	-	-	-	-	-	-	-	-	-	-	-	-	-	-	-	-	-	-	-	-	-	-	-	-	-	-	-	-	-	+	-	-	-	-	-	-	-	-	-	-	-	-	-	-	-	-
4965	7622	Cell wall surface anchor family protein	1	-	-	-	-	-	-	-	-	-	-	-	-	-	-	-	-	-	-	-	-	-	-	-	-	-	-	-	-	-	-	-	-	-	-	-	-	+	-	-	-	-	-	-	-	-	-	-	-	-	-	-	-	-
4966	7626	hypothetical protein	1	-	-	-	-	-	-	-	-	-	-	-	-	-	-	-	-	-	-	-	-	-	-	-	-	-	-	-	-	-	-	-	-	-	-	-	-	+	-	-	-	-	-	-	-	-	-	-	-	-	-	-	-	-
4967	7628	hypothetical protein	1	-	-	-	-	-	-	-	-	-	-	-	-	-	-	-	-	-	-	-	-	-	-	-	-	-	-	-	-	-	-	-	-	-	-	-	-	+	-	-	-	-	-	-	-	-	-	-	-	-	-	-	-	-
4968	7630	hypothetical protein	1	-	-	-	-	-	-	-	-	-	-	-	-	-	-	-	-	-	-	-	-	-	-	-	-	-	-	-	-	-	-	-	-	-	-	-	-	+	-	-	-	-	-	-	-	-	-	-	-	-	-	-	-	-
4969	7637	hypothetical protein	1	-	-	-	-	-	-	-	-	-	-	-	-	-	-	-	-	-	-	-	-	-	-	-	-	-	-	-	-	-	-	-	-	-	-	-	-	+	-	-	-	-	-	-	-	-	-	-	-	-	-	-	-	-
4970	7640	hypothetical protein	1	-	-	-	-	-	-	-	-	-	-	-	-	-	-	-	-	-	-	-	-	-	-	-	-	-	-	-	-	-	-	-	-	-	-	-	-	+	-	-	-	-	-	-	-	-	-	-	-	-	-	-	-	-
4971	7645	hypothetical protein	1	-	-	-	-	-	-	-	-	-	-	-	-	-	-	-	-	-	-	-	-	-	-	-	-	-	-	-	-	-	-	-	-	-	-	-	-	+	-	-	-	-	-	-	-	-	-	-	-	-	-	-	-	-
4972	7656	Phospholipid-binding protein	1	-	-	-	-	-	-	-	-	-	-	-	-	-	-	-	-	-	-	-	-	-	-	-	-	-	-	-	-	-	-	-	-	-	-	-	-	-	-	-	-	-	-	-	-	-	+	-	-	-	-	-	-	-
4973	7658	putative cell surface protein	1	-	-	-	-	-	-	-	-	-	-	-	-	-	-	-	-	-	-	-	-	-	-	-	-	-	-	-	-	-	-	-	-	-	-	-	-	-	-	-	-	-	-	-	-	-	+	-	-	-	-	-	-	-
4974	7660	Transcriptional regulator	1	-	-	-	-	-	-	-	-	-	-	-	-	-	-	-	-	-	-	-	-	-	-	-	-	-	-	-	-	-	-	-	-	-	-	-	-	-	-	-	-	-	-	-	-	-	+	-	-	-	-	-	-	-
4975	7661	putative ATPase	1	-	-	-	-	-	-	-	-	-	-	-	-	-	-	-	-	-	-	-	-	-	-	-	-	-	-	-	-	-	-	-	-	+	-	-	-	-	-	-	-	-	-	-	-	-	-	-	-	-	-	-	-	-
4976	7662	putative adenylate kinase DNA topology modulator(EC:2.7.4.3)	1	-	-	-	-	-	-	-	-	-	-	-	-	-	-	-	-	-	-	-	-	-	-	-	-	-	-	-	-	-	-	-	-	-	-	-	-	-	-	-	-	-	-	-	-	-	+	-	-	-	-	-	-	-
4977	7667	hypothetical protein	1	-	-	-	-	-	-	-	-	-	-	-	-	-	-	-	-	-	-	-	-	-	-	-	-	-	-	-	-	-	-	-	-	-	-	-	-	-	-	-	-	-	-	-	-	-	+	-	-	-	-	-	-	-
4978	7675	Type I restriction-modification system, specificity subunit S (EC 3.1.21.3)	1	-	-	-	-	-	-	-	-	-	-	-	-	-	-	-	-	-	-	-	-	-	-	-	-	-	-	-	-	-	-	-	-	-	-	-	-	-	-	-	-	-	-	-	-	-	+	-	-	-	-	-	-	-
4979	7681	hypothetical protein	1	-	-	-	-	-	-	-	-	-	-	-	-	-	-	-	-	-	-	-	-	-	-	-	-	-	-	-	-	-	-	-	-	-	-	-	-	-	-	-	-	-	-	-	-	-	+	-	-	-	-	-	-	-
4980	7688	Na+ ABC transporter (ATP-binding protein)	1	-	-	-	-	-	-	-	-	-	-	-	-	-	-	-	-	-	-	-	-	-	-	-	-	-	-	-	-	-	-	-	-	-	-	-	-	-	-	-	-	-	-	-	-	-	+	-	-	-	-	-	-	-
4981	7689	Beta-glucoside bgl operon antiterminator, BglG family	1	-	-	-	-	-	-	-	-	-	-	-	-	-	-	-	-	-	-	-	-	-	-	-	-	-	-	-	-	-	-	-	-	-	-	-	-	-	-	-	-	-	-	-	-	-	+	-	-	-	-	-	-	-
4982	7690	hypothetical protein	1	-	-	-	-	-	-	-	-	-	-	-	-	-	-	-	-	-	-	-	-	-	-	-	-	-	-	-	-	-	-	-	-	-	-	-	-	-	-	-	-	-	-	-	-	-	+	-	-	-	-	-	-	-
4983	7693	ClpB protein	1	-	-	-	-	-	-	-	-	-	-	-	-	-	-	-	-	-	-	-	-	-	-	-	-	-	-	-	-	-	-	-	-	-	-	-	-	-	-	-	-	-	-	-	-	-	+	-	-	-	-	-	-	-
4984	7698	ATP-dependent DNA helicase UvrD/PcrA	1	-	-	-	-	-	-	-	-	-	-	-	-	-	-	-	-	-	-	-	-	-	-	-	-	-	-	-	-	-	-	-	-	-	-	-	-	-	-	-	-	-	-	-	-	-	+	-	-	-	-	-	-	-
4985	7706	hypothetical protein	1	-	-	-	-	-	-	-	-	-	-	-	-	-	-	-	-	-	-	-	-	-	-	-	-	-	-	-	-	-	-	-	-	-	-	-	-	-	-	-	-	-	-	-	-	-	+	-	-	-	-	-	-	-
4986	7712	hypothetical protein	1	-	-	-	-	-	-	-	-	-	-	-	-	-	-	-	-	-	-	-	-	-	-	-	-	-	-	-	-	-	-	-	-	-	-	-	-	-	-	-	-	-	-	-	-	-	+	-	-	-	-	-	-	-
4987	7717	hypothetical protein	1	-	-	-	-	-	-	-	-	-	-	-	-	-	-	-	-	-	-	-	-	-	-	-	-	-	-	-	-	-	-	-	-	-	-	-	-	-	-	-	-	-	-	-	-	-	+	-	-	-	-	-	-	-
4988	7721	hypothetical protein	1	-	-	-	-	-	-	-	-	-	-	-	-	-	-	-	-	-	-	-	-	-	-	-	-	-	-	-	-	-	-	-	-	-	-	-	-	-	-	-	-	-	-	-	-	-	+	-	-	-	-	-	-	-
4989	7733	Copynumber control protein CopG	1	-	-	-	-	-	-	-	-	-	-	-	-	-	-	-	-	-	-	-	-	-	-	-	-	-	-	-	-	-	-	-	-	-	-	-	-	-	-	-	-	-	-	-	-	-	+	-	-	-	-	-	-	-
4990	7736	PTS system, beta-glucoside-specific IIB component (EC 2.7.1.69) / PTS system, beta-glucoside-specific IIC component (EC 2.7.1.69) / PTS system, beta-glucoside-specific IIA component (EC 2.7.1.69)	1	-	-	-	-	-	-	-	-	-	-	-	-	-	-	-	-	-	-	-	-	-	-	-	-	-	-	-	-	-	-	-	-	-	-	-	-	-	-	-	-	-	-	-	-	-	+	-	-	-	-	-	-	-
4991	7742	Site-specific recombinase	1	-	-	-	-	-	-	-	-	-	-	-	-	-	-	-	-	-	-	-	-	-	-	-	-	-	-	-	-	-	-	-	-	-	-	-	-	-	-	-	-	-	-	-	-	-	+	-	-	-	-	-	-	-
4992	7744	hypothetical protein	1	-	-	-	-	-	-	-	-	-	-	-	-	-	-	-	-	-	-	-	-	-	-	-	-	-	-	-	-	-	-	-	-	-	-	-	-	-	-	-	-	-	-	-	-	-	+	-	-	-	-	-	-	-
4993	7746	LysR family regulatory protein	1	-	-	-	-	-	-	-	-	-	-	-	-	-	-	-	-	-	-	-	-	-	-	-	-	-	-	-	-	-	-	-	-	-	-	-	-	-	-	-	-	-	-	-	-	-	+	-	-	-	-	-	-	-
4994	7751	Valyl-tRNA synthetase (EC 6.1.1.9)	1	-	-	-	-	-	-	-	-	-	-	-	-	+	-	-	-	-	-	-	-	-	-	-	-	-	-	-	-	-	-	-	-	-	-	-	-	-	-	-	-	-	-	-	-	-	-	-	-	-	-	-	-	-
4995	7752	ABC transporter permease protein	1	-	-	-	-	-	-	-	-	-	-	-	-	+	-	-	-	-	-	-	-	-	-	-	-	-	-	-	-	-	-	-	-	-	-	-	-	-	-	-	-	-	-	-	-	-	-	-	-	-	-	-	-	-
4996	7755	hypothetical protein	1	-	-	-	-	-	-	-	-	-	-	-	-	-	-	+	-	-	-	-	-	-	-	-	-	-	-	-	-	-	-	-	-	-	-	-	-	-	-	-	-	-	-	-	-	-	-	-	-	-	-	-	-	-
4997	7756	collagen adhesin precursor	1	-	-	-	-	-	-	-	-	-	-	-	-	-	-	+	-	-	-	-	-	-	-	-	-	-	-	-	-	-	-	-	-	-	-	-	-	-	-	-	-	-	-	-	-	-	-	-	-	-	-	-	-	-
4998	7757	hypothetical protein	1	-	-	-	-	-	-	-	-	-	-	-	-	-	-	+	-	-	-	-	-	-	-	-	-	-	-	-	-	-	-	-	-	-	-	-	-	-	-	-	-	-	-	-	-	-	-	-	-	-	-	-	-	-
4999	7762	hypothetical protein	1	-	-	-	-	-	-	-	-	-	-	-	-	-	-	+	-	-	-	-	-	-	-	-	-	-	-	-	-	-	-	-	-	-	-	-	-	-	-	-	-	-	-	-	-	-	-	-	-	-	-	-	-	-
5000	7763	Lipopolysaccharide core biosynthesis protein RfaS	1	-	-	-	-	-	-	-	-	-	-	-	-	-	-	+	-	-	-	-	-	-	-	-	-	-	-	-	-	-	-	-	-	-	-	-	-	-	-	-	-	-	-	-	-	-	-	-	-	-	-	-	-	-
5001	7765	unknown phage protein	1	-	-	-	-	-	-	-	-	-	-	-	-	-	-	+	-	-	-	-	-	-	-	-	-	-	-	-	-	-	-	-	-	-	-	-	-	-	-	-	-	-	-	-	-	-	-	-	-	-	-	-	-	-
5002	7767	hypothetical protein	1	-	-	-	-	-	-	-	-	-	-	-	-	-	-	+	-	-	-	-	-	-	-	-	-	-	-	-	-	-	-	-	-	-	-	-	-	-	-	-	-	-	-	-	-	-	-	-	-	-	-	-	-	-
5003	7768	hypothetical protein	1	-	-	-	-	-	-	-	-	-	-	-	-	-	-	+	-	-	-	-	-	-	-	-	-	-	-	-	-	-	-	-	-	-	-	-	-	-	-	-	-	-	-	-	-	-	-	-	-	-	-	-	-	-
5004	7769	Phage capsid and scaffold	1	-	-	-	-	-	-	-	-	-	-	-	-	-	-	+	-	-	-	-	-	-	-	-	-	-	-	-	-	-	-	-	-	-	-	-	-	-	-	-	-	-	-	-	-	-	-	-	-	-	-	-	-	-
5005	7770	putative minor capsid protein - phage associated	1	-	-	-	-	-	-	-	-	-	-	-	-	-	-	+	-	-	-	-	-	-	-	-	-	-	-	-	-	-	-	-	-	-	-	-	-	-	-	-	-	-	-	-	-	-	-	-	-	-	-	-	-	-
5006	7779	hypothetical protein	1	-	-	-	-	-	-	-	-	-	-	-	-	-	-	-	-	-	-	-	-	-	-	-	-	-	-	-	-	-	-	-	-	-	-	-	-	-	-	-	-	+	-	-	-	-	-	-	-	-	-	-	-	-
5007	7780	Polysaccharide biosynthesis protein CpsM(V)	1	-	-	-	-	-	-	-	-	-	-	-	-	-	-	-	-	-	-	-	-	-	-	-	-	-	-	-	-	-	-	-	-	-	-	-	-	-	-	-	-	+	-	-	-	-	-	-	-	-	-	-	-	-
5008	7782	hypothetical protein	1	-	-	-	-	-	-	-	-	-	-	-	-	-	-	-	-	-	-	-	-	-	-	-	-	-	-	-	-	-	-	-	-	-	-	-	-	-	-	-	-	+	-	-	-	-	-	-	-	-	-	-	-	-
5009	7783	hypothetical protein	1	-	-	-	-	-	-	-	-	-	-	-	-	-	-	-	-	-	-	-	-	-	-	-	-	-	-	-	-	-	-	-	-	-	-	-	-	-	-	-	-	+	-	-	-	-	-	-	-	-	-	-	-	-
5010	7784	ABC transporter, ATP-binding protein	1	-	-	-	-	-	-	-	-	-	-	-	-	-	-	-	-	-	-	-	-	-	-	-	-	-	-	-	-	-	-	-	-	-	-	-	-	-	-	-	-	+	-	-	-	-	-	-	-	-	-	-	-	-
5011	7786	hypothetical protein	1	-	-	-	-	-	-	-	-	-	-	-	-	-	-	-	-	-	-	-	-	-	-	-	-	-	-	-	-	-	-	-	-	-	-	-	-	-	-	-	-	+	-	-	-	-	-	-	-	-	-	-	-	-
5012	7787	hypothetical protein	1	-	-	-	-	-	-	-	-	-	-	-	-	-	-	-	-	-	-	-	-	-	-	-	-	-	-	-	-	-	-	-	-	-	-	-	-	-	-	-	-	+	-	-	-	-	-	-	-	-	-	-	-	-
5013	7790	hypothetical protein	1	-	-	-	-	-	-	-	-	-	-	-	-	-	-	-	-	-	-	-	-	-	-	-	-	-	-	-	-	-	-	-	-	-	-	-	-	-	-	-	-	+	-	-	-	-	-	-	-	-	-	-	-	-
5014	7793	Transcriptional regulator, XRE family	1	-	-	-	-	-	-	-	-	-	-	-	-	-	-	-	-	-	-	-	-	-	-	-	-	-	-	-	-	-	-	-	-	-	-	-	-	-	-	-	-	+	-	-	-	-	-	-	-	-	-	-	-	-
5015	7824	Type I restriction-modification system, specificity subunit S (EC 3.1.21.3)	1	-	-	-	-	-	-	-	-	-	-	-	-	-	-	-	-	-	-	-	-	-	-	-	-	-	-	-	-	-	-	-	+	-	-	-	-	-	-	-	-	-	-	-	-	-	-	-	-	-	-	-	-	-
5016	7850	Signal recognition particle receptor protein FtsY (=alpha subunit) (TC 3.A.5.1.1)	1	-	-	-	-	-	-	-	-	-	-	-	-	-	-	-	-	-	-	-	-	-	-	-	-	-	-	-	-	-	-	-	-	-	+	-	-	-	-	-	-	-	-	-	-	-	-	-	-	-	-	-	-	-
5017	7853	DNA polymerase III delta subunit (EC 2.7.7.7)	1	-	-	-	-	-	-	-	-	-	-	-	-	-	-	-	-	-	-	-	-	-	-	-	-	-	-	-	-	-	-	-	-	-	+	-	-	-	-	-	-	-	-	-	-	-	-	-	-	-	-	-	-	-
5018	7854	hypothetical protein	1	-	-	-	-	-	-	-	-	-	-	-	-	-	-	-	-	-	-	-	-	-	-	-	-	-	-	-	-	-	-	-	-	-	+	-	-	-	-	-	-	-	-	-	-	-	-	-	-	-	-	-	-	-
5019	7856	Thioredoxin domain-containing protein	1	-	-	-	-	-	-	-	-	-	-	-	-	-	-	-	-	-	-	-	-	-	-	-	-	-	-	-	-	-	-	-	-	-	+	-	-	-	-	-	-	-	-	-	-	-	-	-	-	-	-	-	-	-
5020	7860	hypothetical protein	1	-	-	-	-	-	-	-	-	-	-	-	-	-	-	-	-	-	-	-	-	-	-	-	-	-	-	-	-	-	-	-	-	-	+	-	-	-	-	-	-	-	-	-	-	-	-	-	-	-	-	-	-	-
5021	7861	TrsE-like protein	1	-	-	-	-	-	-	-	-	-	-	-	-	-	-	-	-	-	-	-	-	-	-	-	-	-	-	-	-	-	-	-	-	-	+	-	-	-	-	-	-	-	-	-	-	-	-	-	-	-	-	-	-	-
5022	7863	Lanthionine biosynthesis protein LanM	1	-	-	-	-	-	-	-	-	-	-	-	-	-	-	-	-	-	-	-	-	-	-	-	-	-	-	-	-	-	-	-	-	-	+	-	-	-	-	-	-	-	-	-	-	-	-	-	-	-	-	-	-	-
5023	7864	hypothetical protein	1	-	-	-	-	-	-	-	-	-	-	-	-	-	-	-	-	-	-	-	-	-	-	-	-	-	-	-	-	-	-	-	-	-	+	-	-	-	-	-	-	-	-	-	-	-	-	-	-	-	-	-	-	-
5024	7865	hypothetical protein	1	-	-	-	-	-	-	-	-	-	-	-	-	-	-	-	-	-	-	-	-	-	-	-	-	-	-	-	-	-	-	-	-	-	+	-	-	-	-	-	-	-	-	-	-	-	-	-	-	-	-	-	-	-
5025	7871	Type I restriction-modification system, specificity subunit S (EC 3.1.21.3)	1	-	-	-	-	-	-	-	-	-	-	-	-	-	-	-	-	-	-	-	-	-	-	-	-	-	-	-	-	-	-	-	-	-	+	-	-	-	-	-	-	-	-	-	-	-	-	-	-	-	-	-	-	-
5026	7872	possible two-component sensor histidine kinase	1	-	-	-	-	-	-	-	-	-	-	-	-	-	-	-	-	-	-	-	-	-	-	-	-	-	-	-	-	-	-	-	-	-	+	-	-	-	-	-	-	-	-	-	-	-	-	-	-	-	-	-	-	-
5027	7873	hypothetical protein	1	-	-	-	-	-	-	-	-	-	-	-	-	-	-	-	-	-	-	-	-	-	-	-	-	-	-	-	-	-	-	-	-	+	-	-	-	-	-	-	-	-	-	-	-	-	-	-	-	-	-	-	-	-
5028	7874	transcriptional regulator PlcR	1	-	-	-	-	-	-	-	-	-	-	-	-	-	-	-	-	-	-	-	-	-	-	-	-	-	-	-	-	-	-	-	-	+	-	-	-	-	-	-	-	-	-	-	-	-	-	-	-	-	-	-	-	-
5029	7878	hypothetical protein	1	-	-	-	-	-	-	-	-	-	-	-	-	-	-	-	-	-	-	-	-	-	-	-	-	-	-	-	-	-	-	-	-	+	-	-	-	-	-	-	-	-	-	-	-	-	-	-	-	-	-	-	-	-
5030	7880	Tetracycline resistance protein TetM	1	-	-	-	-	-	-	-	-	-	-	-	-	-	-	-	-	-	-	-	-	-	-	-	-	-	-	-	-	-	-	-	-	+	-	-	-	-	-	-	-	-	-	-	-	-	-	-	-	-	-	-	-	-
5031	7894	hypothetical protein	1	-	-	-	-	-	-	-	-	-	-	-	-	-	-	-	-	-	-	-	-	-	-	-	-	-	-	-	-	-	-	-	-	-	-	-	-	-	-	-	-	-	-	-	-	-	-	-	-	-	-	-	+	-
5032	7899	Phosphomannomutase	1	-	-	-	-	-	-	-	-	-	-	-	-	-	-	-	-	-	-	-	-	-	-	-	-	-	-	-	-	+	-	-	-	-	-	-	-	-	-	-	-	-	-	-	-	-	-	-	-	-	-	-	-	-
5033	7900	Protein repA	1	-	-	-	-	-	-	-	-	-	-	-	-	-	+	-	-	-	-	-	-	-	-	-	-	-	-	-	-	-	-	-	-	-	-	-	-	-	-	-	-	-	-	-	-	-	-	-	-	-	-	-	-	-
5034	7911	hypothetical protein	1	-	-	-	-	-	-	-	-	-	-	-	-	-	-	-	-	-	-	-	-	-	-	-	-	-	-	-	-	-	-	-	-	-	-	-	-	-	-	-	-	-	-	-	-	-	-	-	-	-	+	-	-	-
5035	7919	conjugal transfer protein	1	-	-	-	-	-	-	-	-	-	-	-	-	-	-	-	-	-	-	-	-	-	-	-	-	-	-	-	-	-	-	-	-	-	-	-	-	-	-	-	-	-	-	-	-	-	-	-	-	-	-	-	+	-
5036	7923	hypothetical protein	1	-	-	-	-	-	-	-	-	-	-	-	-	-	-	-	-	-	-	-	-	-	-	-	-	-	-	-	-	-	-	-	-	-	-	-	-	-	-	-	-	-	-	-	-	-	-	-	-	+	-	-	-	-
5037	7930	Recombination-mobilization protein	1	-	-	-	-	-	-	-	-	-	-	-	-	-	+	-	-	-	-	-	-	-	-	-	-	-	-	-	-	-	-	-	-	-	-	-	-	-	-	-	-	-	-	-	-	-	-	-	-	-	-	-	-	-
5038	7932	N-carbamoylputrescine amidase (3.5.1.53)	1	-	-	-	-	-	-	-	-	-	-	-	-	-	-	-	-	-	-	-	-	-	-	-	-	-	-	-	-	-	-	-	-	-	-	-	-	-	-	-	-	-	-	-	-	-	-	-	-	-	-	-	+	-
5039	7943	Topoisomerase IV subunit A (EC 5.99.1.-)	1	-	-	-	-	-	-	-	-	-	-	-	-	-	-	-	-	-	-	-	-	-	-	-	-	-	-	-	-	-	-	-	-	-	-	-	-	-	-	-	-	-	-	-	-	-	-	-	-	-	-	-	+	-
5040	7949	Mobile element protein	1	-	-	-	-	-	-	-	-	-	-	-	-	-	-	-	-	-	-	-	-	-	-	-	-	-	-	-	-	-	-	-	-	-	-	-	-	-	-	-	-	-	-	-	-	-	-	-	-	-	-	+	-	-
5041	7953	hypothetical protein	1	-	-	-	-	-	-	-	-	-	-	-	-	-	-	-	-	-	-	-	-	-	-	-	-	-	-	-	-	-	-	-	-	-	-	-	-	-	-	-	-	-	-	-	-	-	-	-	-	-	-	+	-	-
5042	7954	hypothetical protein	1	-	-	-	-	-	-	-	-	-	-	-	-	-	-	-	-	-	-	-	-	-	-	-	-	-	-	-	-	-	-	-	-	-	-	-	-	-	-	-	-	-	-	-	-	-	-	-	-	-	-	+	-	-
5043	7972	hypothetical protein	1	-	-	-	-	-	-	-	-	-	-	-	-	-	-	-	-	-	-	-	-	-	-	-	-	-	-	-	-	-	-	-	-	-	-	-	-	-	-	-	-	-	-	-	-	-	-	-	-	-	-	+	-	-
5044	7981	hypothetical protein	1	-	-	-	-	-	-	-	-	-	-	-	-	-	-	-	-	-	-	-	-	-	-	-	-	-	-	-	-	-	-	-	-	-	-	-	-	-	-	-	-	-	-	-	-	-	-	-	-	-	-	+	-	-
5045	7982	hypothetical protein	1	-	-	-	-	-	-	-	-	-	-	-	-	-	-	-	-	-	-	-	-	-	-	-	-	-	-	-	-	-	-	-	-	-	-	-	-	-	-	-	-	-	-	-	-	-	-	-	-	+	-	-	-	-
5046	7989	Signal peptidase I (EC 3.4.21.89)	1	-	-	-	-	-	-	-	-	-	-	-	-	-	-	-	-	-	+	-	-	-	-	-	-	-	-	-	-	-	-	-	-	-	-	-	-	-	-	-	-	-	-	-	-	-	-	-	-	-	-	-	-	-
5047	7995	hypothetical protein	1	-	-	-	-	-	-	-	-	-	-	-	-	-	-	-	-	-	-	-	-	-	-	-	-	-	-	-	-	-	-	-	-	-	-	-	-	-	-	-	-	-	-	-	-	-	-	-	+	-	-	-	-	-
5048	8000	hypothetical protein	1	-	-	-	-	-	-	-	-	-	-	-	-	-	-	-	-	-	-	-	-	-	-	-	-	-	-	-	-	-	-	-	-	-	-	-	-	-	-	-	-	-	-	-	-	-	-	-	-	-	-	-	+	-
5049	8005	hypothetical protein	1	-	-	-	-	-	-	-	-	-	-	-	-	-	-	-	-	-	-	-	-	-	-	-	-	-	-	-	-	-	-	+	-	-	-	-	-	-	-	-	-	-	-	-	-	-	-	-	-	-	-	-	-	-
5050	8007	tRNA dihydrouridine synthase B (EC 1.-.-.-)	1	-	-	-	-	-	-	-	-	-	-	-	-	-	-	-	-	-	-	-	-	-	-	-	-	-	-	-	-	-	-	-	-	-	-	-	-	-	-	-	-	-	-	-	-	-	-	-	-	-	-	-	+	-
5051	8008	hypothetical protein	1	-	-	-	-	-	-	-	-	-	-	-	-	-	-	-	-	-	-	-	-	-	-	-	-	-	-	-	-	-	-	-	-	-	-	-	-	-	-	-	-	-	-	-	-	-	-	-	+	-	-	-	-	-
5052	8011	GCN5-related N-acetyltransferase	1	-	-	-	-	-	-	-	-	-	-	-	-	-	-	-	-	-	-	-	-	-	-	-	-	-	-	-	-	-	-	-	-	-	-	-	-	-	-	-	-	-	-	-	-	-	-	+	-	-	-	-	-	-
5053	8017	GCN5-related N-acetyltransferase	1	-	-	-	-	-	-	-	-	-	-	-	-	-	-	-	-	-	-	-	-	-	-	-	-	-	-	-	-	-	-	-	-	-	-	-	-	-	-	-	-	-	-	-	-	-	-	-	+	-	-	-	-	-
5054	8019	Tyrosine-protein kinase (capsular polysaccharide biosynthesis)	1	-	-	-	-	-	-	-	-	-	-	-	-	-	-	-	-	-	-	-	-	-	-	-	-	-	-	-	-	-	-	-	-	-	-	-	-	-	-	-	-	-	-	-	-	-	-	-	-	+	-	-	-	-
5055	8020	hypothetical protein	1	-	-	-	-	-	-	-	-	-	-	-	-	-	-	-	-	-	-	-	-	-	-	-	-	-	-	-	-	-	-	-	-	-	-	-	-	-	-	-	-	-	-	-	-	-	-	-	+	-	-	-	-	-
5056	8029	hypothetical protein	1	-	-	-	-	-	-	-	-	-	-	-	-	-	-	-	-	-	-	-	-	-	-	-	-	-	-	-	-	+	-	-	-	-	-	-	-	-	-	-	-	-	-	-	-	-	-	-	-	-	-	-	-	-
5057	8033	ABC transporter, ATP-binding protein	1	-	-	-	-	-	-	-	-	-	-	-	-	-	-	-	-	-	-	-	-	-	-	-	-	-	-	-	-	+	-	-	-	-	-	-	-	-	-	-	-	-	-	-	-	-	-	-	-	-	-	-	-	-
5058	8037	hypothetical protein	1	-	-	-	-	-	-	-	-	-	-	-	-	-	-	-	-	-	-	-	-	-	-	-	-	-	-	-	-	+	-	-	-	-	-	-	-	-	-	-	-	-	-	-	-	-	-	-	-	-	-	-	-	-
5059	8039	Phage single-strand DNA binding protein	1	-	-	-	-	-	-	-	-	-	-	-	-	-	-	-	-	-	-	-	-	-	-	-	-	-	-	-	-	+	-	-	-	-	-	-	-	-	-	-	-	-	-	-	-	-	-	-	-	-	-	-	-	-
5060	8041	Glutathione S-transferase, Streptococcal type (EC 2.5.1.18)	1	-	-	-	-	-	-	-	-	-	-	-	-	-	-	-	-	-	-	-	-	-	-	-	-	-	-	-	-	+	-	-	-	-	-	-	-	-	-	-	-	-	-	-	-	-	-	-	-	-	-	-	-	-
5061	8044	Single-stranded DNA-binding protein	1	-	-	-	-	-	-	-	-	-	-	-	-	-	-	-	-	-	-	-	-	-	-	-	-	-	-	-	-	+	-	-	-	-	-	-	-	-	-	-	-	-	-	-	-	-	-	-	-	-	-	-	-	-
5062	8045	UPF0236 protein in vanSb 3'region	1	-	-	-	-	-	-	-	-	-	-	-	-	-	-	-	-	-	-	-	-	-	-	-	-	-	-	-	-	-	-	-	-	-	-	-	-	-	-	-	-	-	-	-	-	-	-	-	+	-	-	-	-	-
5063	8046	hypothetical protein	1	-	-	-	-	-	-	-	-	-	-	-	-	-	-	-	-	-	-	-	-	-	-	-	-	-	-	-	-	-	-	-	-	-	-	-	-	-	-	-	-	-	-	-	-	-	-	-	+	-	-	-	-	-
5064	8047	hypothetical protein	1	-	-	-	-	-	-	-	-	-	-	-	-	-	-	-	-	-	-	-	-	-	-	-	-	-	-	-	-	-	-	-	-	-	-	-	-	-	-	-	-	-	-	-	-	-	-	-	+	-	-	-	-	-
5065	8049	acetyltransferase, GNAT family family	1	-	-	-	-	-	-	-	-	-	-	-	-	-	-	-	-	-	-	-	-	-	-	-	-	-	-	-	-	-	-	-	-	-	-	-	-	-	-	-	-	-	-	-	-	-	-	-	+	-	-	-	-	-
5066	8051	Helicase loader DnaI	1	-	-	-	-	-	-	-	-	-	-	-	-	-	-	-	-	-	-	-	-	-	-	-	-	-	-	-	-	-	-	-	-	-	-	-	-	-	-	-	-	-	-	+	-	-	-	-	-	-	-	-	-	-
5067	8053	hypothetical protein	1	-	-	-	-	-	-	-	-	-	-	-	-	-	-	-	-	-	-	-	-	-	-	-	-	-	-	-	-	-	-	-	-	-	-	-	-	-	-	-	-	-	-	-	-	-	-	-	+	-	-	-	-	-
5068	8065	DNA adenine methylase(EC:2.1.1.72)	1	-	-	-	-	-	-	-	-	-	-	-	-	-	-	-	-	-	-	-	-	-	-	-	-	-	-	-	-	-	-	-	-	-	-	-	-	-	-	-	-	-	-	-	-	-	-	-	+	-	-	-	-	-
5069	8068	hypothetical protein	1	-	-	-	-	-	-	-	-	-	-	-	-	-	-	-	-	-	-	-	-	-	-	-	-	-	-	-	-	-	-	-	-	-	-	-	-	-	-	-	-	-	-	+	-	-	-	-	-	-	-	-	-	-
5070	8069	hypothetical protein	1	-	-	-	-	-	-	-	-	-	-	-	-	-	-	-	-	-	-	-	-	-	-	-	-	-	-	-	-	-	-	-	-	-	-	-	-	-	-	-	-	-	-	+	-	-	-	-	-	-	-	-	-	-
5071	8075	hypothetical protein	1	-	-	-	-	-	-	-	-	-	-	-	-	-	-	-	-	-	-	-	-	-	-	-	-	-	-	-	-	-	-	-	-	-	-	-	-	-	-	-	-	-	-	+	-	-	-	-	-	-	-	-	-	-
5072	8087	hypothetical protein	1	-	-	-	-	-	-	-	-	-	-	-	-	-	-	-	-	-	-	-	-	-	-	-	-	-	-	-	-	-	-	-	-	-	-	-	-	-	-	-	-	-	-	-	-	-	-	-	-	-	+	-	-	-
5073	8089	hypothetical protein	1	-	-	-	-	-	-	-	-	+	-	-	-	-	-	-	-	-	-	-	-	-	-	-	-	-	-	-	-	-	-	-	-	-	-	-	-	-	-	-	-	-	-	-	-	-	-	-	-	-	-	-	-	-
5074	8091	hypothetical protein	1	-	-	-	-	-	-	-	-	-	-	-	-	-	-	-	-	-	-	-	-	-	-	-	-	-	-	-	-	-	-	-	-	-	-	-	-	-	-	+	-	-	-	-	-	-	-	-	-	-	-	-	-	-
5075	8094	hypothetical protein	1	-	-	-	-	-	-	-	-	+	-	-	-	-	-	-	-	-	-	-	-	-	-	-	-	-	-	-	-	-	-	-	-	-	-	-	-	-	-	-	-	-	-	-	-	-	-	-	-	-	-	-	-	-
5076	8096	hypothetical protein	1	-	-	-	-	-	-	-	-	-	-	-	-	-	-	-	-	-	-	-	-	-	-	-	-	-	-	-	-	-	-	-	-	-	-	-	-	-	-	+	-	-	-	-	-	-	-	-	-	-	-	-	-	-
5077	8098	hypothetical protein	1	-	-	-	-	-	-	-	-	-	-	-	-	-	-	-	-	-	-	-	-	-	-	-	-	-	+	-	-	-	-	-	-	-	-	-	-	-	-	-	-	-	-	-	-	-	-	-	-	-	-	-	-	-
5078	8099	Phage terminase	1	-	-	-	-	-	-	-	-	-	-	-	-	-	-	-	-	-	-	-	-	-	-	-	-	-	-	-	-	-	-	-	-	-	-	-	-	-	-	-	-	-	-	-	-	-	-	+	-	-	-	-	-	-
5079	8100	NisR	1	-	-	-	-	-	-	-	-	-	-	-	-	-	-	-	-	-	-	-	-	-	-	-	-	-	-	-	-	-	-	-	-	-	-	-	-	-	-	-	-	-	-	-	-	-	-	+	-	-	-	-	-	-
5080	8102	Rhamnulokinase (EC 2.7.1.5)	1	-	-	-	-	-	-	-	-	-	-	-	-	-	-	-	-	-	-	-	-	-	-	-	-	-	-	-	-	-	-	-	-	-	-	-	-	-	-	-	-	-	-	-	-	-	-	+	-	-	-	-	-	-
5081	8103	hypothetical protein	1	-	-	-	-	-	-	-	-	-	-	-	-	-	-	-	-	-	-	-	-	-	-	-	-	-	-	-	-	-	-	-	-	-	-	-	-	-	-	-	-	-	-	-	-	-	-	+	-	-	-	-	-	-
5082	8104	Anticodon nuclease	1	-	-	-	-	-	-	-	-	-	-	-	-	-	-	-	-	-	-	-	-	-	-	-	-	-	-	-	-	-	-	-	-	-	-	-	-	-	-	-	-	-	-	-	-	-	-	+	-	-	-	-	-	-
5083	8108	hypothetical protein	1	-	-	-	-	-	-	-	-	-	-	-	-	-	-	-	-	-	-	-	-	-	-	-	-	-	-	-	-	-	-	-	-	-	-	-	-	-	-	-	-	-	-	-	-	-	-	+	-	-	-	-	-	-
5084	8114	conserved domain protein	1	-	-	-	-	-	-	-	-	-	-	-	-	-	-	-	-	-	-	-	-	-	-	-	-	-	-	-	-	-	-	-	-	-	-	-	-	-	-	-	-	-	-	-	-	-	-	+	-	-	-	-	-	-
5085	8121	Rhamnulose-1-phosphate aldolase (EC 4.1.2.19)	1	-	-	-	-	-	-	-	-	-	-	-	-	-	-	-	-	-	-	-	-	-	-	-	-	-	-	-	-	-	-	-	-	-	-	-	-	-	-	-	-	-	-	-	-	-	-	+	-	-	-	-	-	-
5086	8123	hypothetical protein	1	-	-	-	-	-	-	-	-	-	-	-	-	-	-	-	-	-	-	-	-	-	-	-	-	-	-	-	-	-	-	-	-	-	-	-	-	-	-	-	-	-	-	-	-	-	-	+	-	-	-	-	-	-
5087	8124	Phage protein	1	-	-	-	-	-	-	-	-	-	-	-	-	-	-	-	-	-	-	-	-	-	-	-	-	-	-	-	-	-	-	-	-	-	-	-	-	-	-	-	-	-	-	-	-	-	-	+	-	-	-	-	-	-
5088	8125	Transcriptional regulator of rhamnose utilization, AraC family	1	-	-	-	-	-	-	-	-	-	-	-	-	-	-	-	-	-	-	-	-	-	-	-	-	-	-	-	-	-	-	-	-	-	-	-	-	-	-	-	-	-	-	-	-	-	-	+	-	-	-	-	-	-
5089	8126	Pleiotropic regulator of exopolysaccharide synthesis, competence and biofilm formation Ftr, XRE family	1	-	-	-	-	-	-	-	-	-	-	-	-	-	-	-	-	-	-	-	-	-	-	-	-	-	-	-	-	-	-	-	-	-	-	-	-	-	-	-	-	-	-	-	-	-	-	+	-	-	-	-	-	-
5090	8127	Phage tail length tape-measure protein	1	-	-	-	-	-	-	-	-	-	-	-	-	-	-	-	-	-	-	-	-	-	-	-	-	-	-	-	-	-	-	-	-	-	-	-	-	-	-	-	-	-	-	-	-	-	-	+	-	-	-	-	-	-
5091	8128	hypothetical protein	1	-	-	-	-	-	-	-	-	-	-	-	-	-	-	-	-	-	-	-	-	-	-	-	-	-	-	-	-	-	-	-	-	-	-	-	-	-	-	-	-	-	-	-	-	-	-	+	-	-	-	-	-	-
5092	8130	hypothetical protein	1	-	-	-	-	-	-	-	-	-	-	-	-	-	-	-	-	-	-	-	-	-	-	-	-	-	-	-	-	-	-	-	-	-	-	-	-	-	-	-	-	-	-	-	-	-	-	+	-	-	-	-	-	-
5093	8132	hypothetical protein	1	-	-	-	-	-	-	-	-	-	-	-	-	-	-	-	-	-	-	-	-	-	-	-	-	-	-	-	-	-	-	-	-	-	-	-	-	-	-	-	-	-	-	-	-	-	-	+	-	-	-	-	-	-
5094	8134	hypothetical protein	1	-	-	-	-	-	-	-	-	-	-	-	-	-	-	-	-	-	-	-	-	-	-	-	-	-	-	-	-	-	-	-	-	-	-	-	-	-	-	-	-	-	-	-	-	-	-	+	-	-	-	-	-	-
5095	8135	prophage LambdaSa2, protease, putative	1	-	-	-	-	-	-	-	-	-	-	-	-	-	-	-	-	-	-	-	-	-	-	-	-	-	-	-	-	-	-	-	-	-	-	-	-	-	-	-	-	-	-	-	-	-	-	+	-	-	-	-	-	-
5096	8138	hypothetical protein	1	-	-	-	-	-	-	-	-	-	-	-	-	-	-	-	-	-	-	-	-	-	-	-	-	-	-	-	-	-	-	-	-	-	-	-	-	-	-	-	-	-	-	-	-	-	-	+	-	-	-	-	-	-
5097	8148	hypothetical protein	1	-	-	-	-	-	-	-	-	-	-	-	-	-	-	-	-	-	-	-	-	-	-	-	-	-	-	-	-	-	-	-	-	-	-	-	-	-	-	-	-	-	-	-	-	-	-	+	-	-	-	-	-	-
5098	8152	hypothetical protein	1	-	-	-	-	-	-	-	-	-	-	-	-	-	-	-	-	-	-	-	-	-	-	-	-	-	-	-	-	-	-	-	-	-	-	-	-	-	-	-	-	-	-	-	-	-	-	+	-	-	-	-	-	-
5099	8153	hypothetical protein	1	-	-	-	-	-	-	-	-	-	-	-	-	-	-	-	-	-	-	-	-	-	-	-	-	-	-	-	-	-	-	-	-	-	-	-	-	-	-	-	-	-	-	-	-	-	-	+	-	-	-	-	-	-
5100	8154	hypothetical protein	1	-	-	-	-	-	-	-	-	-	-	-	-	-	-	-	-	-	-	-	-	-	-	-	-	-	-	-	-	-	-	-	-	-	-	-	-	-	-	-	-	-	-	-	-	-	-	+	-	-	-	-	-	-
5101	8155	transposase	1	-	-	-	-	-	-	-	-	-	-	-	-	-	-	-	-	-	-	-	-	-	-	-	-	-	-	-	-	-	-	-	-	-	-	-	-	-	-	-	-	-	-	-	-	-	-	+	-	-	-	-	-	-
5102	8163	hypothetical protein	1	-	-	-	-	-	-	-	-	-	-	-	-	-	-	-	-	-	-	-	-	-	-	-	-	-	-	-	-	-	-	-	-	-	-	-	-	-	-	-	-	-	-	-	-	-	-	+	-	-	-	-	-	-
5103	8164	Orf26	1	-	-	-	-	-	-	-	-	-	-	-	-	-	-	-	-	-	-	-	-	-	-	-	-	-	-	-	-	-	-	-	-	-	-	-	-	-	-	-	-	-	-	-	-	-	-	+	-	-	-	-	-	-
5104	8168	hypothetical protein	1	-	-	-	-	-	-	-	-	-	-	-	-	-	-	-	-	-	-	-	-	-	-	-	-	-	-	-	-	-	-	-	-	-	-	-	-	-	-	-	-	-	-	-	-	-	-	+	-	-	-	-	-	-
5105	8169	Phage terminase large subunit	1	-	-	-	-	-	-	-	-	-	-	-	-	-	-	-	-	-	-	-	-	-	-	-	-	-	-	-	-	-	-	-	-	-	-	-	-	-	-	-	-	-	-	-	-	-	-	+	-	-	-	-	-	-
5106	8176	FIG021930: Phage-associated protein	1	-	-	-	-	-	-	-	-	-	-	-	-	-	-	-	-	-	-	-	-	-	-	-	-	-	-	-	-	-	-	-	-	-	-	-	-	-	-	-	+	-	-	-	-	-	-	-	-	-	-	-	-	-
5107	8190	Phage protein	1	-	-	-	-	-	-	-	-	-	-	-	-	-	-	-	-	-	-	-	-	-	-	-	-	-	-	-	-	-	-	-	-	-	-	-	-	-	-	-	+	-	-	-	-	-	-	-	-	-	-	-	-	-
5108	8194	hypothetical protein	1	-	-	-	-	-	-	-	-	-	-	-	-	-	-	-	-	-	-	-	-	-	-	-	-	-	-	-	-	-	-	-	-	-	-	-	-	-	-	-	+	-	-	-	-	-	-	-	-	-	-	-	-	-
5109	8197	hypothetical protein	1	-	-	-	-	-	-	-	-	-	-	-	-	-	-	-	-	-	-	-	-	-	-	-	-	-	-	-	-	-	-	-	-	-	-	-	-	-	-	-	+	-	-	-	-	-	-	-	-	-	-	-	-	-
5110	8200	hypothetical protein	1	-	-	-	-	-	-	-	-	-	-	-	-	-	-	-	-	-	-	-	-	-	-	-	-	-	-	-	-	-	-	-	-	-	-	-	-	-	-	-	+	-	-	-	-	-	-	-	-	-	-	-	-	-
5111	8204	DNA-cytosine methyltransferase (EC 2.1.1.37)	1	-	-	-	-	-	-	-	-	-	-	-	-	-	-	-	-	-	-	-	-	-	-	-	-	-	-	-	-	-	-	-	-	-	-	-	-	-	-	-	-	-	-	-	-	-	-	-	-	-	+	-	-	-
5112	8205	hypothetical protein	1	-	-	-	-	-	-	-	-	-	-	-	-	-	-	-	-	-	-	-	-	-	-	-	-	-	-	-	-	-	-	-	-	-	-	-	-	-	-	-	-	-	-	-	-	-	-	-	-	-	+	-	-	-
5113	8206	hypothetical protein	1	-	-	-	-	-	-	-	-	-	-	-	-	-	-	-	-	-	-	-	-	-	-	-	-	-	-	-	-	-	-	-	-	-	-	-	-	-	-	-	-	-	-	-	-	-	-	-	-	-	+	-	-	-
5114	8207	Phage tail length tape-measure protein	1	-	-	-	-	-	-	-	-	-	-	-	-	-	-	-	-	-	-	+	-	-	-	-	-	-	-	-	-	-	-	-	-	-	-	-	-	-	-	-	-	-	-	-	-	-	-	-	-	-	-	-	-	-	-	-
5115	8208	SSU ribosomal protein S1p	1	-	-	-	-	-	-	-	-	-	-	-	-	-	-	-	-	-	-	-	+	-	-	-	-	-	-	-	-	-	-	-	-	-	-	-	-	-	-	-	-	-	-	-	-	-	-	-	-	-	-	-	-	-	-	-
5116	8209	Helicase conserved C-terminal domain protein	1	-	-	-	-	-	-	-	-	-	-	-	-	-	-	-	-	-	-	-	-	-	-	-	-	-	-	-	-	-	-	-	-	-	-	-	-	-	-	-	-	-	-	-	-	-	-	-	-	-	+	-	-	-
5117	8212	hypothetical protein	1	-	-	-	-	-	-	-	-	-	-	-	-	-	-	-	-	-	-	-	-	-	-	-	-	-	-	-	-	-	-	-	-	-	-	-	-	-	-	-	-	-	-	-	-	-	-	-	-	-	+	-	-	-
5118	8213	hypothetical protein	1	-	-	-	-	-	-	-	-	-	-	-	-	-	-	-	-	-	-	-	-	-	-	-	-	-	-	-	-	-	-	-	-	-	-	-	-	-	-	-	-	-	-	-	-	-	-	-	-	-	+	-	-	-
5119	8214	hypothetical protein	1	-	-	-	-	-	-	-	-	-	-	-	-	-	-	-	-	-	-	-	-	-	-	-	-	-	-	-	-	-	-	-	-	-	-	-	-	-	-	-	-	-	-	-	-	-	-	-	-	-	+	-	-	-
5120	8216	hypothetical protein	1	-	-	-	-	-	-	-	-	-	-	-	-	-	-	-	-	-	-	-	-	-	-	-	-	-	-	-	-	-	-	-	-	-	-	-	-	-	-	-	-	-	-	-	-	-	-	-	-	-	+	-	-	-
5121	8218	hypothetical protein	1	-	-	-	-	-	-	-	-	-	-	-	-	-	-	-	-	-	-	-	-	-	-	-	-	-	-	-	-	-	-	-	-	-	-	-	-	-	-	-	-	-	-	-	-	-	-	-	-	-	+	-	-	-
5122	8221	hypothetical protein	1	-	-	-	-	-	-	-	-	-	-	-	-	-	-	-	-	-	-	-	-	-	-	-	-	-	-	-	-	-	-	-	-	-	-	-	-	-	-	-	-	-	-	-	-	-	-	-	-	-	+	-	-	-
5123	8222	Site-specific recombinase	1	-	-	-	-	-	-	-	-	-	-	-	-	-	-	-	-	-	-	-	-	-	-	-	-	-	-	-	-	-	-	-	-	-	-	-	-	-	-	+	-	-	-	-	-	-	-	-	-	-	-	-	-	-
5124	8223	hypothetical protein	1	-	-	-	-	-	-	-	-	-	-	-	-	-	-	-	-	-	-	-	-	-	-	-	-	-	-	-	-	-	-	-	-	-	-	-	-	-	-	+	-	-	-	-	-	-	-	-	-	-	-	-	-	-

Table S5. Precence/absence of each HG among 57 *S. suis* strains

				1																13			25					28						104		94			76			53/54		Divergent *S. suis*																
Index[a]	ClusterID	Gene products	Members	98HAH33	05ZYH33	SC84	A7	SS12	P1/7	DAT273	NIAH11434	JS14	GZ1	BM407	S735	13730	MNCM07	NCTC 10446	14636	NCTC 10237	ST1	2651	89-1591	MNCM04	MNCM25	8074	D9	4961	ST3	MNCM43	DAT292	DAT260	NIAH11435	MNCM21	MNCM50	89-2479	DAT234	DAT290	93A	42A	NT77	6407	11538	8830	12814	92-1400	89-590	2726	2524	89-3576-3	4417	D12	10581	89-5259	88-5299A	R61	92-1191	14A	92-4172	22083
5125	8224	hypothetical protein	1	-	-	-	-	-	-	-	-	-	-	-	-	-	-	-	-	-	-	-	-	-	-	-	-	-	-	-	-	-	-	-	-	-	-	-	-	-	-	+	-	-	-	-	-	-	-	-	-	-	-	-	-	-
5126	8225	hypothetical protein	1	-	-	-	-	-	-	-	-	-	-	-	-	-	-	-	-	-	-	-	-	-	-	-	-	-	-	-	-	-	-	-	-	-	-	-	-	-	-	+	-	-	-	-	-	-	-	-	-	-	-	-	-	-
5127	8226	hypothetical protein	1	-	-	-	-	-	-	-	-	-	-	-	-	-	-	-	-	-	-	-	-	-	-	-	-	-	-	-	-	-	-	-	-	-	-	-	-	-	-	+	-	-	-	-	-	-	-	-	-	-	-	-	-	-
5128	8227	Chromosome (plasmid) partitioning protein ParA / Sporulation initiation inhibitor protein Soj	1	-	-	-	-	-	-	-	-	-	-	-	-	-	-	-	-	-	-	-	-	-	-	-	-	-	-	-	-	-	-	-	-	-	-	-	-	-	-	+	-	-	-	-	-	-	-	-	-	-	-	-	-	-
5129	8228	hypothetical protein	1	-	-	-	-	-	-	-	-	-	-	-	-	-	-	-	-	-	-	-	-	-	-	-	-	-	-	-	-	-	-	-	-	-	-	-	-	-	-	+	-	-	-	-	-	-	-	-	-	-	-	-	-	-
5130	8229	regulatory protein	1	-	-	-	-	-	-	-	-	-	-	-	-	-	-	-	-	-	-	-	-	-	-	-	-	-	-	-	-	-	-	-	-	-	-	-	-	-	-	+	-	-	-	-	-	-	-	-	-	-	-	-	-	-
5131	8230	hypothetical protein	1	-	-	-	-	-	-	-	-	-	-	-	-	-	-	-	-	-	-	-	-	-	-	-	-	-	-	-	-	-	-	-	-	-	-	-	-	-	-	+	-	-	-	-	-	-	-	-	-	-	-	-	-	-
5132	8231	Hypothetical membrane spanning protein	1	-	-	-	-	-	-	-	-	-	-	-	-	-	-	-	-	-	-	-	-	-	-	-	-	-	-	-	-	-	-	-	-	-	-	-	-	-	-	+	-	-	-	-	-	-	-	-	-	-	-	-	-	-
5133	8232	hypothetical protein	1	-	-	-	-	-	-	-	-	-	-	-	-	-	-	-	-	-	-	-	-	-	-	-	-	-	-	-	-	-	-	-	-	-	-	-	-	-	-	+	-	-	-	-	-	-	-	-	-	-	-	-	-	-
5134	8233	hypothetical protein	1	-	-	-	-	-	-	-	-	-	-	-	-	-	-	-	-	-	-	-	-	-	-	-	-	-	-	-	-	-	-	-	-	-	-	-	-	-	-	+	-	-	-	-	-	-	-	-	-	-	-	-	-	-
5135	8235	hypothetical protein	1	-	-	-	-	-	-	-	-	-	-	-	-	-	-	-	-	-	-	-	-	-	-	-	-	-	-	-	-	-	-	-	-	-	-	-	-	-	-	+	-	-	-	-	-	-	-	-	-	-	-	-	-	-
5136	8236	2-haloalkanoic acid dehalogenase (EC 3.8.1.2)	1	-	-	-	-	-	-	-	-	-	-	-	-	-	-	-	-	-	-	-	-	-	-	-	-	-	-	-	-	-	-	-	-	-	-	-	-	-	-	+	-	-	-	-	-	-	-	-	-	-	-	-	-	-
5137	8237	hypothetical protein	1	-	-	-	-	-	-	-	-	-	-	-	-	-	-	-	-	-	-	-	-	-	-	-	-	-	-	-	-	-	-	-	-	-	-	-	-	-	-	+	-	-	-	-	-	-	-	-	-	-	-	-	-	-
5138	8238	Prophage antirepressor	1	-	-	-	-	-	-	-	-	-	-	-	-	-	-	-	-	-	-	-	-	-	-	-	-	-	-	-	-	-	-	-	-	-	-	-	-	-	-	+	-	-	-	-	-	-	-	-	-	-	-	-	-	-
5139	8239	Modification methylase Cfr9I (EC 2.1.1.113)	1	-	-	-	-	-	-	-	-	-	-	-	-	-	-	-	-	-	-	-	-	-	-	-	-	-	-	-	-	-	-	-	-	-	-	-	-	-	-	+	-	-	-	-	-	-	-	-	-	-	-	-	-	-
5140	8240	hypothetical protein	1	-	-	-	-	-	-	-	-	-	-	-	-	-	-	-	-	-	-	-	-	-	-	-	-	-	-	-	-	-	-	-	-	-	-	-	-	-	-	+	-	-	-	-	-	-	-	-	-	-	-	-	-	-
5141	8241	putative esterase	1	-	-	-	-	-	-	-	-	-	-	-	-	-	-	-	-	-	-	-	-	-	-	-	-	-	-	-	-	-	-	-	-	-	-	-	-	-	-	+	-	-	-	-	-	-	-	-	-	-	-	-	-	-
5142	8244	Chromosome (plasmid) partitioning protein ParB / Stage 0 sporulation protein J	1	-	-	-	-	-	-	-	-	-	-	-	-	-	-	-	-	-	-	-	-	-	-	-	-	-	-	-	-	-	-	-	-	-	-	-	-	-	-	+	-	-	-	-	-	-	-	-	-	-	-	-	-	-
5143	8246	COG0286: Type I restriction-modification system methyltransferase subunit	1	-	-	-	-	-	-	-	-	-	-	-	-	-	-	-	-	-	-	-	-	-	-	-	-	-	-	-	-	-	-	-	-	-	-	-	-	-	-	-	-	-	-	-	-	-	-	-	-	-	-	-	+	-
5144	8247	hypothetical protein	1	-	-	-	-	-	-	-	-	-	-	-	-	-	-	-	-	-	-	-	-	-	-	-	-	-	-	-	-	-	-	-	-	-	-	-	-	-	-	-	-	-	-	-	-	-	-	-	-	-	-	-	+	-
5145	8248	hypothetical protein	1	-	-	-	-	-	-	-	-	-	-	-	-	-	-	-	-	-	-	-	-	-	-	-	-	-	-	-	-	-	-	-	-	-	-	-	-	-	-	-	-	-	-	-	-	-	-	-	-	-	-	-	+	-
5146	8249	Peptide chain release factor 3	1	-	-	-	-	-	-	-	-	-	-	-	-	-	-	-	-	-	-	-	-	-	-	-	-	-	-	-	-	-	-	-	-	-	-	-	-	-	-	-	-	-	-	-	-	-	-	-	-	-	-	-	+	-
5147	8251	Recombinase	1	-	-	-	-	-	-	-	-	-	-	-	-	-	-	-	-	-	-	-	-	-	-	-	-	-	-	-	-	-	-	-	-	-	-	-	-	-	-	-	-	-	-	-	-	-	-	-	-	-	-	-	+	-
5148	8252	hypothetical protein	1	-	-	-	-	-	-	-	-	-	-	-	-	-	-	-	-	-	-	-	-	-	-	-	-	-	-	-	-	-	-	-	-	-	-	-	-	-	-	-	-	-	-	-	-	-	-	-	-	-	-	-	+	-
5149	8255	hypothetical protein	1	-	-	-	-	-	-	-	-	-	-	-	-	-	-	-	-	-	-	-	-	-	-	-	-	-	-	-	-	-	-	-	-	-	-	-	-	-	-	-	-	-	-	-	-	-	-	-	-	-	-	-	+	-
5150	8256	choline binding protein C	1	-	-	-	-	-	-	-	-	-	-	-	-	-	-	-	-	-	-	-	-	-	-	-	-	-	-	-	-	-	-	-	-	-	-	-	-	-	-	-	-	-	-	-	-	-	-	-	-	-	-	-	+	-
5151	8257	Cell division protein FtsW	1	-	-	-	-	-	-	-	-	-	-	-	-	-	-	-	-	-	-	-	-	-	-	-	-	-	-	-	-	-	-	-	-	-	-	-	-	-	-	-	-	-	-	-	-	-	-	-	-	-	-	-	+	-
5152	8258	hypothetical protein	1	-	-	-	-	-	-	-	-	-	-	-	-	-	-	-	-	-	-	-	-	-	-	-	-	-	-	-	-	-	-	-	-	-	-	-	-	-	-	-	-	-	-	-	-	-	-	-	-	-	-	-	+	-
5153	8340	transcriptional regulator	1	-	-	-	-	-	-	-	-	-	-	-	-	-	-	-	-	-	-	+	-	-	-	-	-	-	-	-	-	-	-	-	-	-	-	-	-	-	-	-	-	-	-	-	-	-	-	-	-	-	-	-	-	-	-	-
5154	8352	hypothetical protein	1	-	-	-	-	-	-	-	-	-	-	-	-	-	-	-	-	-	-	+	-	-	-	-	-	-	-	-	-	-	-	-	-	-	-	-	-	-	-	-	-	-	-	-	-	-	-	-	-	-	-	-	-	-	-	-
5155	8416	hypothetical protein	1	-	-	-	-	-	-	-	-	-	+	-	-	-	-	-	-	-	-	-	-	-	-	-	-	-	-	-	-	-	-	-	-	-	-	-	-	-	-	-	-	-	-	-	-	-	-	-	-	-	-	-	-	-
5156	8418	hypothetical protein	1	-	-	-	-	-	-	-	-	-	+	-	-	-	-	-	-	-	-	-	-	-	-	-	-	-	-	-	-	-	-	-	-	-	-	-	-	-	-	-	-	-	-	-	-	-	-	-	-	-	-	-	-	-
5157	8421	Chromosome (plasmid) partitioning protein ParA / Sporulation initiation inhibitor protein Soj	1	-	-	-	-	-	-	-	-	-	+	-	-	-	-	-	-	-	-	-	-	-	-	-	-	-	-	-	-	-	-	-	-	-	-	-	-	-	-	-	-	-	-	-	-	-	-	-	-	-	-	-	-	-
5158	8423	hypothetical protein	1	-	-	-	-	-	-	-	-	-	+	-	-	-	-	-	-	-	-	-	-	-	-	-	-	-	-	-	-	-	-	-	-	-	-	-	-	-	-	-	-	-	-	-	-	-	-	-	-	-	-	-	-	-
5159	8424	hypothetical protein	1	-	-	-	-	-	-	-	-	-	+	-	-	-	-	-	-	-	-	-	-	-	-	-	-	-	-	-	-	-	-	-	-	-	-	-	-	-	-	-	-	-	-	-	-	-	-	-	-	-	-	-	-	-
5160	8426	predicted transcriptional regulator	1	-	-	-	-	-	-	-	-	-	+	-	-	-	-	-	-	-	-	-	-	-	-	-	-	-	-	-	-	-	-	-	-	-	-	-	-	-	-	-	-	-	-	-	-	-	-	-	-	-	-	-	-	-
5161	8427	hypothetical protein	1	-	-	-	-	-	-	-	-	-	+	-	-	-	-	-	-	-	-	-	-	-	-	-	-	-	-	-	-	-	-	-	-	-	-	-	-	-	-	-	-	-	-	-	-	-	-	-	-	-	-	-	-	-
5162	8428	hypothetical protein	1	-	-	-	-	-	-	-	-	-	+	-	-	-	-	-	-	-	-	-	-	-	-	-	-	-	-	-	-	-	-	-	-	-	-	-	-	-	-	-	-	-	-	-	-	-	-	-	-	-	-	-	-	-
5163	8429	LtrC-like protein	1	-	-	-	-	-	-	-	-	-	+	-	-	-	-	-	-	-	-	-	-	-	-	-	-	-	-	-	-	-	-	-	-	-	-	-	-	-	-	-	-	-	-	-	-	-	-	-	-	-	-	-	-	-
5164	8430	MocB-like protein	1	-	-	-	-	-	-	-	-	-	+	-	-	-	-	-	-	-	-	-	-	-	-	-	-	-	-	-	-	-	-	-	-	-	-	-	-	-	-	-	-	-	-	-	-	-	-	-	-	-	-	-	-	-
5165	8432	hypothetical protein	1	-	-	-	-	-	-	-	-	-	+	-	-	-	-	-	-	-	-	-	-	-	-	-	-	-	-	-	-	-	-	-	-	-	-	-	-	-	-	-	-	-	-	-	-	-	-	-	-	-	-	-	-	-
5166	8435	NPQTN specific sortase B	1	-	-	-	-	-	-	-	-	-	+	-	-	-	-	-	-	-	-	-	-	-	-	-	-	-	-	-	-	-	-	-	-	-	-	-	-	-	-	-	-	-	-	-	-	-	-	-	-	-	-	-	-	-
5167	8436	hypothetical protein	1	-	-	-	-	-	-	-	-	-	+	-	-	-	-	-	-	-	-	-	-	-	-	-	-	-	-	-	-	-	-	-	-	-	-	-	-	-	-	-	-	-	-	-	-	-	-	-	-	-	-	-	-	-
5168	8437	hypothetical protein	1	-	-	-	-	-	-	-	-	-	+	-	-	-	-	-	-	-	-	-	-	-	-	-	-	-	-	-	-	-	-	-	-	-	-	-	-	-	-	-	-	-	-	-	-	-	-	-	-	-	-	-	-	-
5169	8438	hypothetical protein	1	-	-	-	-	-	-	-	-	-	+	-	-	-	-	-	-	-	-	-	-	-	-	-	-	-	-	-	-	-	-	-	-	-	-	-	-	-	-	-	-	-	-	-	-	-	-	-	-	-	-	-	-	-
5170	8440	DNA, complete sequence	1	-	-	-	-	-	-	-	-	-	+	-	-	-	-	-	-	-	-	-	-	-	-	-	-	-	-	-	-	-	-	-	-	-	-	-	-	-	-	-	-	-	-	-	-	-	-	-	-	-	-	-	-	-
5171	8441	Relaxase	1	-	-	-	-	-	-	-	-	-	+	-	-	-	-	-	-	-	-	-	-	-	-	-	-	-	-	-	-	-	-	-	-	-	-	-	-	-	-	-	-	-	-	-	-	-	-	-	-	-	-	-	-	-
5172	8442	hypothetical protein	1	-	-	-	-	-	-	-	-	-	+	-	-	-	-	-	-	-	-	-	-	-	-	-	-	-	-	-	-	-	-	-	-	-	-	-	-	-	-	-	-	-	-	-	-	-	-	-	-	-	-	-	-	-
5173	8444	Invasion associated protein p60	1	-	-	-	-	-	-	-	-	-	+	-	-	-	-	-	-	-	-	-	-	-	-	-	-	-	-	-	-	-	-	-	-	-	-	-	-	-	-	-	-	-	-	-	-	-	-	-	-	-	-	-	-	-
5174	8445	hypothetical protein	1	-	-	-	-	-	-	-	-	-	+	-	-	-	-	-	-	-	-	-	-	-	-	-	-	-	-	-	-	-	-	-	-	-	-	-	-	-	-	-	-	-	-	-	-	-	-	-	-	-	-	-	-	-
5175	8446	hypothetical protein	1	-	-	-	-	-	-	-	-	-	+	-	-	-	-	-	-	-	-	-	-	-	-	-	-	-	-	-	-	-	-	-	-	-	-	-	-	-	-	-	-	-	-	-	-	-	-	-	-	-	-	-	-	-
5176	8447	Programmed cell death toxin YdcE	1	-	-	-	-	-	-	-	-	-	+	-	-	-	-	-	-	-	-	-	-	-	-	-	-	-	-	-	-	-	-	-	-	-	-	-	-	-	-	-	-	-	-	-	-	-	-	-	-	-	-	-	-	-
5177	8449	TrsK-like protein	1	-	-	-	-	-	-	-	-	-	+	-	-	-	-	-	-	-	-	-	-	-	-	-	-	-	-	-	-	-	-	-	-	-	-	-	-	-	-	-	-	-	-	-	-	-	-	-	-	-	-	-	-	-
5178	8450	hypothetical protein	1	-	-	-	-	-	-	-	-	-	+	-	-	-	-	-	-	-	-	-	-	-	-	-	-	-	-	-	-	-	-	-	-	-	-	-	-	-	-	-	-	-	-	-	-	-	-	-	-	-	-	-	-	-
5179	8451	TnpY	1	-	-	-	-	-	-	-	-	-	+	-	-	-	-	-	-	-	-	-	-	-	-	-	-	-	-	-	-	-	-	-	-	-	-	-	-	-	-	-	-	-	-	-	-	-	-	-	-	-	-	-	-	-
5180	8453	hypothetical protein	1	-	-	-	-	-	-	-	-	-	+	-	-	-	-	-	-	-	-	-	-	-	-	-	-	-	-	-	-	-	-	-	-	-	-	-	-	-	-	-	-	-	-	-	-	-	-	-	-	-	-	-	-	-
5181	8457	hypothetical protein	1	-	-	-	-	-	-	-	-	-	+	-	-	-	-	-	-	-	-	-	-	-	-	-	-	-	-	-	-	-	-	-	-	-	-	-	-	-	-	-	-	-	-	-	-	-	-	-	-	-	-	-	-	-
5182	8458	hypothetical protein	1	-	-	-	-	-	-	-	-	-	+	-	-	-	-	-	-	-	-	-	-	-	-	-	-	-	-	-	-	-	-	-	-	-	-	-	-	-	-	-	-	-	-	-	-	-	-	-	-	-	-	-	-	-
5183	8459	hypothetical protein	1	-	-	-	-	-	-	-	-	-	+	-	-	-	-	-	-	-	-	-	-	-	-	-	-	-	-	-	-	-	-	-	-	-	-	-	-	-	-	-	-	-	-	-	-	-	-	-	-	-	-	-	-	-
5184	8465	DNA polymerase IV (EC 2.7.7.7)	1	-	-	-	-	-	-	-	-	-	+	-	-	-	-	-	-	-	-	-	-	-	-	-	-	-	-	-	-	-	-	-	-	-	-	-	-	-	-	-	-	-	-	-	-	-	-	-	-	-	-	-	-	-
5185	8469	hypothetical protein	1	-	-	-	-	-	-	-	-	-	-	-	-	-	-	-	-	-	-	-	-	-	-	-	-	-	-	-	-	-	-	+	-	-	-	-	-	-	-	-	-	-	-	-	-	-	-	-	-	-	-	-	-	-
5186	8470	hypothetical protein	1	-	-	-	-	-	-	-	-	-	-	-	-	-	-	-	-	-	-	-	-	-	-	-	-	-	-	-	-	-	-	+	-	-	-	-	-	-	-	-	-	-	-	-	-	-	-	-	-	-	-	-	-	-
5187	8473	hypothetical protein	1	-	-	-	-	-	-	-	-	-	-	-	-	-	-	-	-	-	-	-	-	-	-	-	-	-	-	-	-	-	-	-	-	-	-	-	-	-	-	-	-	-	-	-	-	+	-	-	-	-	-	-	-	-
5188	8476	Phage tail length tape-measure protein	1	-	-	-	-	-	-	-	-	-	-	-	-	-	-	-	-	-	-	-	-	-	-	-	-	-	-	-	-	-	-	+	-	-	-	-	-	-	-	-	-	-	-	-	-	-	-	-	-	-	-	-	-	-
5189	8478	hypothetical protein	1	-	-	-	-	-	-	-	-	-	-	-	-	-	-	-	-	-	-	-	-	-	-	-	-	-	-	-	-	-	-	+	-	-	-	-	-	-	-	-	-	-	-	-	-	-	-	-	-	-	-	-	-	-
5190	8481	hypothetical protein	1	-	-	-	-	-	-	-	-	-	-	-	-	-	-	-	-	-	-	-	-	-	-	-	-	-	-	-	-	-	-	+	-	-	-	-	-	-	-	-	-	-	-	-	-	-	-	-	-	-	-	-	-	-
5191	8483	hypothetical protein	1	-	-	-	-	-	-	-	-	-	-	-	-	-	-	-	-	-	-	-	-	-	-	-	-	-	-	-	-	-	-	+	-	-	-	-	-	-	-	-	-	-	-	-	-	-	-	-	-	-	-	-	-	-
5192	8489	ftsK-like DNA segregation ATPase	1	-	-	-	-	-	-	-	-	-	-	-	-	-	-	-	-	-	-	-	-	-	-	-	-	-	-	-	-	-	-	-	-	-	-	-	-	-	-	-	-	-	-	-	-	+	-	-	-	-	-	-	-	-
5193	8491	hypothetical protein	1	-	-	-	-	-	-	-	-	-	-	-	-	-	-	-	-	-	-	-	-	-	-	-	-	-	-	-	-	-	-	+	-	-	-	-	-	-	-	-	-	-	-	-	-	-	-	-	-	-	-	-	-	-
5194	8495	hypothetical protein	1	-	-	-	-	-	-	-	-	-	-	-	-	-	-	-	-	-	-	-	-	-	-	-	-	-	-	-	-	-	-	+	-	-	-	-	-	-	-	-	-	-	-	-	-	-	-	-	-	-	-	-	-	-
5195	8497	Portal protein	1	-	-	-	-	-	-	-	-	-	-	-	-	-	-	-	-	-	-	-	-	-	-	-	-	-	-	-	-	-	-	+	-	-	-	-	-	-	-	-	-	-	-	-	-	-	-	-	-	-	-	-	-	-
5196	8499	Phage major capsid protein	1	-	-	-	-	-	-	-	-	-	-	-	-	-	-	-	-	-	-	-	-	-	-	-	-	-	-	-	-	-	-	+	-	-	-	-	-	-	-	-	-	-	-	-	-	-	-	-	-	-	-	-	-	-
5197	8502	Phage terminase large subunit	1	-	-	-	-	-	-	-	-	-	-	-	-	-	-	-	-	-	-	-	-	-	-	-	-	-	-	-	-	-	-	+	-	-	-	-	-	-	-	-	-	-	-	-	-	-	-	-	-	-	-	-	-	-
5198	8505	hypothetical protein	1	-	-	-	-	-	-	-	-	-	-	-	-	-	-	-	-	-	-	+	-	-	-	-	-	-	-	-	-	-	-	-	-	-	-	-	-	-	-	-	-	-	-	-	-	-	-	-	-	-	-	-	-	-	-	-
5199	8506	DNA polymerase III epsilon subunit (EC 2.7.7.7)	1	-	-	-	-	-	-	-	-	-	-	-	-	-	-	-	-	-	-	+	-	-	-	-	-	-	-	-	-	-	-	-	-	-	-	-	-	-	-	-	-	-	-	-	-	-	-	-	-	-	-	-	-	-	-	-
5200	8507	Adenosine deaminase (EC 3.5.4.4)	1	-	-	-	-	-	-	-	-	-	-	-	-	-	-	-	-	-	-	+	-	-	-	-	-	-	-	-	-	-	-	-	-	-	-	-	-	-	-	-	-	-	-	-	-	-	-	-	-	-	-	-	-	-	-	-
5201	8517	ABC transporter permease protein	1	-	-	-	-	-	-	-	-	-	-	-	-	-	-	-	-	-	-	-	-	-	-	-	-	-	-	-	-	-	-	+	-	-	-	-	-	-	-	-	-	-	-	-	-	-	-	-	-	-	-	-	-	-
5202	8518	Phage Terminase Small Subunit	1	-	-	-	-	-	-	-	-	-	-	-	-	-	-	-	-	-	-	-	-	-	-	-	-	-	-	-	-	-	-	+	-	-	-	-	-	-	-	-	-	-	-	-	-	-	-	-	-	-	-	-	-	-
5203	8519	hypothetical protein	1	-	-	-	-	-	-	-	-	-	-	-	-	-	-	-	-	-	-	-	-	-	-	-	-	-	-	-	-	-	-	+	-	-	-	-	-	-	-	-	-	-	-	-	-	-	-	-	-	-	-	-	-	-
5204	8521	Phage endonuclease	1	-	-	-	-	-	-	-	-	-	-	-	-	-	-	-	-	-	-	-	-	-	-	-	-	-	-	-	-	-	-	+	-	-	-	-	-	-	-	-	-	-	-	-	-	-	-	-	-	-	-	-	-	-
5205	8533	conserved domain protein	1	-	-	-	-	-	-	-	-	-	-	-	-	-	+	-	-	-	-	-	-	-	-	-	-	-	-	-	-	-	-	-	-	-	-	-	-	-	-	-	-	-	-	-	-	-	-	-	-	-	-	-	-	-	-	-
5206	8551	hypothetical protein	1	-	-	-	-	-	-	-	-	-	-	-	-	-	-	-	-	-	-	-	-	-	-	-	-	-	-	-	+	-	-	-	-	-	-	-	-	-	-	-	-	-	-	-	-	-	-	-	-	-	-	-	-	-
5207	9071	hypothetical protein	1	-	-	-	-	-	-	-	-	-	-	-	-	-	-	-	-	-	-	-	-	-	-	-	-	-	-	-	-	-	-	-	-	-	-	-	-	-	-	-	-	-	-	-	-	+	-	-	-	-	-	-	-	-
5208	9079	hypothetical protein	1	-	-	-	-	-	-	-	-	-	-	-	-	-	-	-	-	-	-	-	-	-	-	-	-	-	-	-	-	-	-	-	-	-	-	-	-	-	-	-	-	-	-	-	-	+	-	-	-	-	-	-	-	-
5209	9166	ABC transporter, ATP-binding protein	1	-	-	-	-	-	-	-	-	-	-	-	-	-	-	-	-	-	-	-	-	-	-	-	-	-	-	-	-	-	-	-	-	-	-	-	-	-	-	-	-	-	-	-	-	-	-	-	+	-	-	-	-
5210	9230	hypothetical protein	1	-	-	-	-	-	-	-	-	-	-	-	-	-	-	-	-	-	-	-	-	-	-	-	-	-	-	-	-	-	-	-	-	-	-	-	-	-	-	-	-	-	-	-	-	+	-	-	-	-	-	-	-	-
5211	9252	hypothetical protein	1	-	-	-	-	-	-	-	-	-	-	-	-	-	-	-	-	-	-	-	-	-	-	-	-	-	-	-	-	-	-	-	-	-	-	-	-	-	-	-	-	-	-	-	-	-	-	-	+	-	-	-	-
5212	9390	hypothetical protein	1	-	-	-	-	-	-	-	-	-	-	-	-	-	-	-	-	-	-	-	-	-	+	-	-	-	-	-	-	-	-	-	-	-	-	-	-	-	-	-	-	-	-	-	-	-	-	-	-	-	-	-	-	-	-	-
5213	9687	Histidine kinase of the competence regulon ComD	1	-	-	-	-	-	-	-	-	-	-	-	-	-	-	-	-	-	-	-	-	-	-	-	-	-	-	-	-	-	-	-	-	-	-	-	-	-	-	-	-	-	-	-	-	+	-	-	-	-	-	-	-	-
5214	10034	hypothetical protein	1	-	-	-	-	-	-	-	-	-	-	-	-	-	-	-	-	-	-	-	-	-	-	-	-	-	-	-	-	-	-	-	-	-	-	-	-	-	-	-	-	-	-	-	-	+	-	-	-	-	-	-	-	-
5215	10126	6'-aminoglycoside N-acetyltransferase (EC 2.3.1.-) (AAC(6')) / 2''-aminoglycoside phosphotransferase (EC 2.7.1.-)	1	-	-	-	-	-	-	-	-	-	-	-	-	-	-	-	-	-	-	-	-	-	-	-	-	-	-	-	-	-	-	-	-	-	-	-	-	-	-	-	-	-	-	-	-	-	-	-	+	-	-	-	-
5216	10127	Plasmid stabilization system antitoxin protein	1	-	-	-	-	-	-	-	-	+	-	-	-	-	-	-	-	-	-	-	-	-	-	-	-	-	-	-	-	-	-	-	-	-	-	-	-	-	-	-	-	-	-	-	-	-	-	-	-	-	-	-	-	-	-	-
5217	10136	Acetyltransferase, GNAT family	1	-	-	-	-	-	-	-	-	-	-	-	-	-	-	-	-	-	-	-	-	-	-	-	-	-	-	-	-	-	-	-	-	-	-	-	-	-	-	-	-	-	-	-	-	-	-	-	+	-	-	-	-
5218	10170	Streptothricin acetyltransferase, Streptomyces lavendulae type	1	-	-	-	-	-	-	-	-	+	-	-	-	-	-	-	-	-	-	-	-	-	-	-	-	-	-	-	-	-	-	-	-	-	-	-	-	-	-	-	-	-	-	-	-	-	-	-	-	-	-	-	-	-	-	-
5219	10315	Plasmid recombination enzyme	1	-	-	-	-	-	-	-	-	-	-	+	-	-	-	-	-	-	-	-	-	-	-	-	-	-	-	-	-	-	-	-	-	-	-	-	-	-	-	-	-	-	-	-	-	-	-	-	-	-	-	-	-	-	-	-
5220	10349	FIG006036: phage encoded DNA polymerase I (EC 2.7.7.7)	1	-	-	-	-	-	-	-	-	-	-	-	-	-	-	-	-	-	-	-	-	-	-	-	-	-	-	-	-	-	-	-	-	-	-	-	-	-	-	-	-	-	-	-	-	+	-	-	-	-	-	-	-	-
5221	10480	COG0488: ATPase components of ABC transporters with duplicated ATPase domains	1	-	-	-	-	-	-	-	-	+	-	-	-	-	-	-	-	-	-	-	-	-	-	-	-	-	-	-	-	-	-	-	-	-	-	-	-	-	-	-	-	-	-	-	-	-	-	-	-	-	-	-	-	-	-	-
5222	10481	DNA-3-methyladenine glycosylase (EC 3.2.2.20)	1	-	-	-	-	-	-	-	-	-	-	-	-	-	-	-	-	-	-	-	-	-	-	-	+	-	-	-	-	-	-	-	-	-	-	-	-	-	-	-	-	-	-	-	-	-	-	-	-	-	-	-	-	-	-	-
5223	10483	hypothetical protein	1	-	-	-	-	-	-	-	-	-	-	-	-	-	-	-	-	-	-	-	+	-	-	-	-	-	-	-	-	-	-	-	-	-	-	-	-	-	-	-	-	-	-	-	-	-	-	-	-	-	-	-	-	-	-	-
5224	10485	hypothetical protein	1	-	-	-	-	-	-	-	-	-	-	-	-	-	-	-	-	-	-	-	-	-	-	-	-	-	-	-	-	-	-	-	-	-	-	-	-	-	-	-	-	-	-	-	-	+	-	-	-	-	-	-	-	-
5225	10542	hypothetical protein	1	-	-	-	-	-	-	-	-	-	-	-	-	-	-	-	-	-	-	-	+	-	-	-	-	-	-	-	-	-	-	-	-	-	-	-	-	-	-	-	-	-	-	-	-	-	-	-	-	-	-	-	-	-	-	-
5226	10544	FIG042921: similarity to aminoacyl-tRNA editing enzymes YbaK, ProX	1	-	-	-	-	-	-	-	-	-	-	-	-	-	-	-	-	-	-	-	+	-	-	-	-	-	-	-	-	-	-	-	-	-	-	-	-	-	-	-	-	-	-	-	-	-	-	-	-	-	-	-	-	-	-	-
5227	10546	hypothetical protein	1	-	-	-	-	-	-	-	-	-	-	-	-	-	-	-	-	-	-	-	-	-	-	-	-	-	+	-	-	-	-	-	-	-	-	-	-	-	-	-	-	-	-	-	-	-	-	-	-	-	-	-	-	-	-	-
5228	10547	hypothetical protein	1	-	-	-	-	-	-	-	-	-	-	-	-	-	-	-	-	-	-	-	-	-	-	-	-	-	-	-	-	-	-	-	-	-	-	-	-	-	-	-	-	-	-	-	-	-	-	-	-	+	-	-	-	-
5229	10548	hypothetical protein	1	-	-	-	-	-	-	-	-	-	-	-	-	-	-	-	-	-	-	-	-	-	-	-	-	-	-	-	-	-	-	-	-	-	-	-	-	-	-	-	-	-	-	-	-	-	-	-	-	+	-	-	-	-
5230	10549	Protein-L-isoD(D-D) O-methyltransferase	1	-	-	-	-	-	-	-	-	-	-	-	-	-	-	-	-	-	-	-	-	-	-	-	-	-	-	-	-	-	-	-	-	-	-	-	-	-	-	-	-	-	-	-	-	+	-	-	-	-	-	-	-	-
5231	10550	Mobile element protein	1	-	-	-	-	-	-	-	-	-	-	-	-	-	-	-	-	-	-	-	-	-	-	-	-	-	-	-	-	-	-	-	-	-	-	-	-	-	-	-	-	-	-	-	-	+	-	-	-	-	-	-	-	-
5232	10552	hypothetical protein	1	-	-	-	-	-	-	-	-	-	-	-	-	-	-	-	-	-	-	-	-	-	-	-	+	-	-	-	-	-	-	-	-	-	-	-	-	-	-	-	-	-	-	-	-	-	-	-	-	-	-	-	-	-	-	-
5233	10554	hypothetical protein	1	-	-	-	-	-	-	-	-	-	-	-	-	-	-	-	-	-	+	-	-	-	-	-	-	-	-	-	-	-	-	-	-	-	-	-	-	-	-	-	-	-	-	-	-	-	-	-	-	-	-	-	-	-	-	-
5234	10555	putative ATPase	1	-	-	-	-	-	-	-	-	-	-	-	-	-	-	-	-	-	+	-	-	-	-	-	-	-	-	-	-	-	-	-	-	-	-	-	-	-	-	-	-	-	-	-	-	-	-	-	-	-	-	-	-	-	-	-
5235	10600	hypothetical protein	1	-	-	-	-	-	-	-	-	-	-	-	-	-	-	-	-	+	-	-	-	-	-	-	-	-	-	-	-	-	-	-	-	-	-	-	-	-	-	-	-	-	-	-	-	-	-	-	-	-	-	-	-	-	-	-
5236	10601	hypothetical protein	1	-	-	-	-	-	-	-	-	-	-	-	-	-	-	-	-	+	-	-	-	-	-	-	-	-	-	-	-	-	-	-	-	-	-	-	-	-	-	-	-	-	-	-	-	-	-	-	-	-	-	-	-	-	-	-
5237	10602	hypothetical protein	1	-	-	-	-	-	-	-	-	-	-	-	-	-	-	-	-	+	-	-	-	-	-	-	-	-	-	-	-	-	-	-	-	-	-	-	-	-	-	-	-	-	-	-	-	-	-	-	-	-	-	-	-	-	-	-
5238	10603	hypothetical protein	1	-	-	-	-	-	-	-	-	-	-	-	-	-	-	-	-	+	-	-	-	-	-	-	-	-	-	-	-	-	-	-	-	-	-	-	-	-	-	-	-	-	-	-	-	-	-	-	-	-	-	-	-	-	-	-
5239	10604	hypothetical protein	1	-	-	-	-	-	-	-	-	-	-	-	-	-	-	-	-	+	-	-	-	-	-	-	-	-	-	-	-	-	-	-	-	-	-	-	-	-	-	-	-	-	-	-	-	-	-	-	-	-	-	-	-	-	-	-
5240	10605	prophage LambdaSa2	1	-	-	-	-	-	-	-	-	-	-	-	-	-	-	-	-	+	-	-	-	-	-	-	-	-	-	-	-	-	-	-	-	-	-	-	-	-	-	-	-	-	-	-	-	-	-	-	-	-	-	-	-	-	-	-
5241	10606	hypothetical protein	1	-	-	-	-	-	-	-	-	-	-	-	-	-	-	-	-	+	-	-	-	-	-	-	-	-	-	-	-	-	-	-	-	-	-	-	-	-	-	-	-	-	-	-	-	-	-	-	-	-	-	-	-	-	-	-
5242	10607	hypothetical protein	1	-	-	-	-	-	-	-	-	-	-	-	-	-	-	-	-	+	-	-	-	-	-	-	-	-	-	-	-	-	-	-	-	-	-	-	-	-	-	-	-	-	-	-	-	-	-	-	-	-	-	-	-	-	-	-
5243	10608	hypothetical protein	1	-	-	-	-	-	-	-	-	-	-	-	-	-	-	-	-	+	-	-	-	-	-	-	-	-	-	-	-	-	-	-	-	-	-	-	-	-	-	-	-	-	-	-	-	-	-	-	-	-	-	-	-	-	-	-
5244	10609	hypothetical protein	1	-	-	-	-	-	-	-	-	-	-	-	-	-	-	-	-	+	-	-	-	-	-	-	-	-	-	-	-	-	-	-	-	-	-	-	-	-	-	-	-	-	-	-	-	-	-	-	-	-	-	-	-	-	-	-
5245	10610	hypothetical protein	1	-	-	-	-	-	-	-	-	-	-	-	-	-	-	-	-	+	-	-	-	-	-	-	-	-	-	-	-	-	-	-	-	-	-	-	-	-	-	-	-	-	-	-	-	-	-	-	-	-	-	-	-	-	-	-
5246	10611	hypothetical protein	1	-	-	-	-	-	-	-	-	-	-	-	-	-	-	-	-	+	-	-	-	-	-	-	-	-	-	-	-	-	-	-	-	-	-	-	-	-	-	-	-	-	-	-	-	-	-	-	-	-	-	-	-	-	-	-
5247	10612	hypothetical protein	1	-	-	-	-	-	-	-	-	-	-	-	-	-	-	-	-	-	-	-	-	-	-	-	-	+	-	-	-	-	-	-	-	-	-	-	-	-	-	-	-	-	-	-	-	-	-	-	-	-	-	-	-	-	-	-
5248	10613	hypothetical protein	1	-	-	-	-	-	-	-	-	-	-	-	-	-	-	-	-	-	-	-	-	-	-	-	-	+	-	-	-	-	-	-	-	-	-	-	-	-	-	-	-	-	-	-	-	-	-	-	-	-	-	-	-	-	-	-
5249	10614	Adenine-specific methyltransferase (EC 2.1.1.72)	1	-	-	-	-	-	-	-	-	-	-	-	-	-	-	-	-	-	-	-	-	-	-	-	-	+	-	-	-	-	-	-	-	-	-	-	-	-	-	-	-	-	-	-	-	-	-	-	-	-	-	-	-	-	-	-
5250	10615	hypothetical protein	1	-	-	-	-	-	-	-	-	-	-	-	-	-	-	-	-	-	-	-	-	-	-	-	-	+	-	-	-	-	-	-	-	-	-	-	-	-	-	-	-	-	-	-	-	-	-	-	-	-	-	-	-	-	-	-
5251	10616	hypothetical protein	1	-	-	-	-	-	-	-	-	-	-	-	-	-	-	-	-	-	-	-	-	-	-	-	-	+	-	-	-	-	-	-	-	-	-	-	-	-	-	-	-	-	-	-	-	-	-	-	-	-	-	-	-	-	-	-
5252	10617	hypothetical protein	1	-	-	-	-	-	-	-	-	-	-	-	-	-	-	-	-	-	-	-	-	-	-	-	-	+	-	-	-	-	-	-	-	-	-	-	-	-	-	-	-	-	-	-	-	-	-	-	-	-	-	-	-	-	-	-
5253	10618	hypothetical protein	1	-	-	-	-	-	-	-	-	-	-	-	-	-	-	-	-	-	-	-	-	-	-	-	-	+	-	-	-	-	-	-	-	-	-	-	-	-	-	-	-	-	-	-	-	-	-	-	-	-	-	-	-	-	-	-
5254	10619	L-fuculokinase (EC 2.7.1.51)	1	-	-	-	-	-	-	-	-	-	-	-	-	-	-	-	-	-	-	-	-	-	-	-	-	-	-	-	-	-	-	-	-	-	-	-	-	+	-	-	-	-	-	-	-	-	-	-	-	-	-	-	-	-	-	-
5255	10620	hypothetical protein	1	-	-	-	-	-	-	-	-	-	-	-	-	-	-	-	-	-	-	-	-	-	-	-	-	-	-	-	-	-	-	-	-	-	-	-	-	+	-	-	-	-	-	-	-	-	-	-	-	-	-	-	-	-	-	-
5256	10621	hypothetical protein	1	-	-	-	-	-	-	-	-	-	-	-	-	-	-	-	-	-	-	-	-	-	-	-	-	-	-	-	-	-	-	-	-	-	-	-	-	+	-	-	-	-	-	-	-	-	-	-	-	-	-	-	-	-	-	-
5257	10622	hypothetical protein	1	-	-	-	-	-	-	-	-	-	-	-	-	-	-	-	-	-	-	-	-	-	-	-	-	-	-	-	-	-	-	-	-	-	-	-	-	+	-	-	-	-	-	-	-	-	-	-	-	-	-	-	-	-	-	-
5258	10623	hypothetical protein	1	-	-	-	-	-	-	-	-	-	-	-	-	-	-	-	-	-	-	-	-	-	-	-	-	-	-	-	-	-	-	-	-	-	-	-	-	+	-	-	-	-	-	-	-	-	-	-	-	-	-	-	-	-	-	-
5259	10624	hypothetical protein	1	-	-	-	-	-	-	-	-	-	-	-	-	-	-	-	-	-	-	-	-	-	-	-	-	-	-	-	-	-	-	-	-	-	-	-	-	+	-	-	-	-	-	-	-	-	-	-	-	-	-	-	-	-	-	-
5260	10625	hypothetical protein	1	-	-	-	-	-	-	-	-	-	-	-	-	-	-	-	-	-	-	-	-	-	-	-	-	-	-	-	-	-	-	-	-	-	-	-	-	+	-	-	-	-	-	-	-	-	-	-	-	-	-	-	-	-	-	-
5261	10626	Integrase	1	-	-	-	-	-	-	-	-	-	-	-	-	-	-	-	-	-	-	-	-	-	-	-	-	-	-	-	-	-	-	-	-	-	-	-	-	+	-	-	-	-	-	-	-	-	-	-	-	-	-	-	-	-	-	-
5262	10627	hypothetical protein	1	-	-	-	-	-	-	-	-	-	-	-	-	-	-	-	-	-	-	-	-	-	-	-	-	-	-	-	-	-	-	-	-	-	-	-	-	+	-	-	-	-	-	-	-	-	-	-	-	-	-	-	-	-	-	-
5263	10628	hypothetical protein	1	-	-	-	-	-	-	-	-	-	-	-	-	-	-	-	-	-	-	-	-	-	-	-	-	-	-	-	-	-	-	-	-	-	-	-	-	+	-	-	-	-	-	-	-	-	-	-	-	-	-	-	-	-	-	-
5264	10629	hypothetical protein	1	-	-	-	-	-	-	-	-	-	-	-	-	-	-	-	-	-	-	-	-	-	-	-	-	-	-	-	-	-	-	-	-	-	-	-	-	+	-	-	-	-	-	-	-	-	-	-	-	-	-	-	-	-	-	-
5265	10630	hypothetical protein	1	-	-	-	-	-	-	-	-	-	-	-	-	-	-	-	-	-	-	-	-	-	-	-	-	-	-	-	-	-	-	-	-	-	-	-	-	+	-	-	-	-	-	-	-	-	-	-	-	-	-	-	-	-	-	-
5266	10631	Site-specific recombinase	1	-	-	-	-	-	-	-	-	-	-	-	-	-	-	-	-	-	-	-	-	-	-	-	-	-	-	-	-	-	-	-	-	-	-	-	-	+	-	-	-	-	-	-	-	-	-	-	-	-	-	-	-	-	-	-
5267	10632	hypothetical protein	1	-	-	-	-	-	-	-	-	-	-	-	-	-	-	-	-	-	-	-	-	-	-	-	-	-	-	-	-	-	-	-	-	-	-	-	-	+	-	-	-	-	-	-	-	-	-	-	-	-	-	-	-	-	-	-
5268	10633	hypothetical protein	1	-	-	-	-	-	-	-	-	-	-	-	-	-	-	-	-	-	-	-	-	-	-	-	-	-	-	-	-	-	-	-	-	-	-	-	-	+	-	-	-	-	-	-	-	-	-	-	-	-	-	-	-	-	-	-
5269	10634	DNA-entry nuclease (Competence-specific nuclease) (EC 3.1.30.-)	1	-	-	-	-	-	-	-	-	-	-	-	-	-	-	-	-	-	-	-	-	-	-	-	-	-	-	-	-	-	-	-	-	-	-	-	+	-	-	-	-	-	-	-	-	-	-	-	-	-	-	-	-	-
5270	10635	Epf-like protein	1	-	-	-	-	-	-	-	-	-	-	-	-	-	-	-	-	-	-	-	-	-	-	-	-	-	-	-	-	-	-	-	-	-	-	-	+	-	-	-	-	-	-	-	-	-	-	-	-	-	-	-	-	-
5271	10636	Maltose O-acetyltransferase (EC 2.3.1.79)	1	-	-	-	-	-	-	-	-	-	-	-	-	-	-	-	-	-	-	-	-	-	-	-	-	-	-	-	-	-	-	-	-	-	-	-	+	-	-	-	-	-	-	-	-	-	-	-	-	-	-	-	-	-
5272	10637	hypothetical protein	1	-	-	-	-	-	-	-	-	-	-	-	-	-	-	-	-	-	-	-	-	-	-	-	-	-	-	-	-	-	-	-	-	-	-	-	+	-	-	-	-	-	-	-	-	-	-	-	-	-	-	-	-	-
5273	10638	hypothetical protein	1	-	-	-	-	-	-	-	-	-	-	-	-	-	-	-	-	-	-	-	-	-	-	-	-	-	-	-	-	-	-	-	-	-	-	-	+	-	-	-	-	-	-	-	-	-	-	-	-	-	-	-	-	-
5274	10639	hypothetical protein	1	-	-	-	-	-	-	-	-	-	-	-	-	-	-	-	-	-	-	-	-	-	-	-	-	-	-	-	-	-	-	-	-	-	-	-	+	-	-	-	-	-	-	-	-	-	-	-	-	-	-	-	-	-
5275	10640	COG0438: Glycosyltransferase	1	-	-	-	-	-	-	-	-	-	-	-	-	-	-	-	-	-	-	-	-	-	-	-	-	-	-	-	-	-	-	-	-	-	-	-	+	-	-	-	-	-	-	-	-	-	-	-	-	-	-	-	-	-
5276	10641	hypothetical protein	1	-	-	-	-	-	-	-	-	-	-	-	-	-	-	-	-	-	-	-	-	-	-	-	-	-	-	-	-	-	-	-	-	-	-	-	+	-	-	-	-	-	-	-	-	-	-	-	-	-	-	-	-	-
5277	10642	hypothetical protein	1	-	-	-	-	-	-	-	-	-	-	-	-	-	-	-	-	-	-	-	-	-	-	-	-	-	-	-	-	-	-	-	-	-	-	-	+	-	-	-	-	-	-	-	-	-	-	-	-	-	-	-	-	-
5278	10643	hypothetical protein	1	-	-	-	-	-	-	-	-	-	-	-	-	-	-	-	-	-	-	-	-	-	-	-	-	-	-	-	-	-	-	-	-	-	-	-	+	-	-	-	-	-	-	-	-	-	-	-	-	-	-	-	-	-
5279	10644	hypothetical protein	1	-	-	-	-	-	-	-	-	-	-	-	-	-	-	-	-	-	-	-	-	-	-	-	-	-	-	-	-	-	-	-	-	-	-	-	+	-	-	-	-	-	-	-	-	-	-	-	-	-	-	-	-	-
5280	10645	hypothetical protein	1	-	-	-	-	-	-	-	-	-	-	-	-	-	-	-	-	-	-	-	-	-	-	-	-	-	-	-	-	-	-	-	-	-	-	-	+	-	-	-	-	-	-	-	-	-	-	-	-	-	-	-	-	-
5281	10646	Glycosyl transferase	1	-	-	-	-	-	-	-	-	-	-	-	-	-	-	-	-	-	-	-	-	-	-	-	-	-	-	-	-	-	-	-	-	-	-	-	+	-	-	-	-	-	-	-	-	-	-	-	-	-	-	-	-	-
5282	10647	hypothetical protein	1	-	-	-	-	-	-	-	-	-	-	-	-	-	-	-	-	-	-	-	-	-	-	-	-	-	-	-	-	-	-	-	-	-	-	-	+	-	-	-	-	-	-	-	-	-	-	-	-	-	-	-	-	-
5283	10648	hypothetical protein	1	-	-	-	-	-	-	-	-	-	-	-	-	-	-	-	-	-	-	-	-	-	-	-	-	-	-	-	-	-	-	-	-	-	-	-	+	-	-	-	-	-	-	-	-	-	-	-	-	-	-	-	-	-
5284	10649	hypothetical protein	1	-	-	-	-	-	-	-	-	-	-	-	-	-	-	-	-	-	-	-	-	-	-	-	-	-	-	-	-	-	-	-	-	-	-	-	+	-	-	-	-	-	-	-	-	-	-	-	-	-	-	-	-	-
5285	10650	hypothetical protein	1	-	-	-	-	-	-	-	-	-	-	-	-	-	-	-	-	-	-	-	-	-	-	-	-	-	-	-	-	-	-	-	-	-	-	-	+	-	-	-	-	-	-	-	-	-	-	-	-	-	-	-	-	-
5286	10651	Glycosyltransferase(EC:2.4.1.-)	1	-	-	-	-	-	-	-	-	-	-	-	-	-	-	-	-	-	-	-	-	-	-	-	-	-	-	-	-	-	-	-	-	-	-	-	-	-	-	-	-	-	+	-	-	-	-	-	-	-	-	-	-	-
5287	10652	Na+-driven multidrug efflux pump	1	-	-	-	-	-	-	-	-	-	-	-	-	-	-	-	-	-	-	-	-	-	-	-	-	-	-	-	-	-	-	-	-	-	-	-	-	-	-	-	-	-	+	-	-	-	-	-	-	-	-	-	-	-
5288	10653	hypothetical protein	1	-	-	-	-	-	-	-	-	-	-	-	-	-	-	-	-	-	-	-	-	-	-	-	-	-	-	-	-	-	-	-	-	-	-	-	-	-	-	-	-	-	+	-	-	-	-	-	-	-	-	-	-	-
5289	10654	DNA-cytosine methyltransferase (EC 2.1.1.37)	1	-	-	-	-	-	-	-	-	-	-	-	-	-	-	-	-	-	-	-	-	-	-	-	-	-	-	-	-	-	-	-	-	-	-	-	-	-	-	-	-	-	+	-	-	-	-	-	-	-	-	-	-	-
5290	10655	hypothetical protein	1	-	-	-	-	-	-	-	-	-	-	-	-	-	-	-	-	-	-	-	-	-	-	-	-	-	-	-	-	-	-	-	-	-	-	-	-	-	-	-	-	-	+	-	-	-	-	-	-	-	-	-	-	-
5291	10656	hypothetical protein	1	-	-	-	-	-	-	-	-	-	-	-	-	-	-	-	-	-	-	-	-	-	-	-	-	-	-	-	-	-	-	-	-	-	-	-	-	-	-	-	-	-	+	-	-	-	-	-	-	-	-	-	-	-
5292	10657	Toll-Interleukin receptor	1	-	-	-	-	-	-	-	-	-	-	-	-	-	-	-	-	-	-	-	-	-	-	-	-	-	-	-	-	-	-	-	-	-	-	-	-	-	-	-	-	-	+	-	-	-	-	-	-	-	-	-	-	-
5293	10658	The type 2 capsule locus of Streptococcus pneumoniae	1	-	-	-	-	-	-	-	-	-	-	-	-	-	-	-	-	-	-	-	-	-	-	-	-	-	-	-	-	-	-	-	-	-	-	-	-	-	-	-	-	-	+	-	-	-	-	-	-	-	-	-	-	-
5294	10659	Putative ABC transporter (ATP-binding protein)	1	-	-	-	-	-	-	-	-	-	-	-	-	-	-	-	-	-	-	-	-	-	-	-	-	-	-	-	-	-	-	-	-	-	-	-	-	-	-	-	-	-	+	-	-	-	-	-	-	-	-	-	-	-
5295	10660	Alpha-L-Rha alpha-1	1	-	-	-	-	-	-	-	-	-	-	-	-	-	-	-	-	-	-	-	-	-	-	-	-	-	-	-	-	-	-	-	-	-	-	-	-	-	-	-	-	-	+	-	-	-	-	-	-	-	-	-	-	-
5296	10661	hypothetical protein	1	-	-	-	-	-	-	-	-	-	-	-	-	-	-	-	-	-	-	-	-	-	-	-	-	-	-	-	-	-	-	-	-	-	-	-	-	-	-	-	-	-	+	-	-	-	-	-	-	-	-	-	-	-
5297	10662	hypothetical protein	1	-	-	-	-	-	-	-	-	-	-	-	-	-	-	-	-	-	-	-	-	-	-	-	-	-	-	-	-	-	-	-	-	-	-	-	-	-	-	-	-	-	+	-	-	-	-	-	-	-	-	-	-	-
5298	10663	hypothetical protein	1	-	-	-	-	-	-	-	-	-	-	-	-	-	-	-	-	-	-	-	-	-	-	-	-	-	-	-	-	-	-	-	-	-	-	-	-	-	-	-	-	-	+	-	-	-	-	-	-	-	-	-	-	-

Table S5. Precence/absence of each HG among 57 *S. suis* strains

				1																13			25					28						104		94			76			53/54		Divergent *S. suis*																
Index[a]	ClusterID	Gene products	Members	98HAH33	05ZYH33	SC84	A7	SS12	P1/7	DAT273	NIAH11434	JS14	GZ1	BM407	S735	13730	MNCM07	NCTC 10446	14636	NCTC 10237	ST1	2651	89-1591	MNCM04	MNCM25	8074	D9	4961	ST3	MNCM43	DAT292	DAT260	NIAH11435	MNCM21	MNCM50	89-2479	DAT234	DAT290	93A	42A	NT77	6407	11538	8830	12814	92-1400	89-590	2726	2524	89-3576-3	4417	D12	10581	89-5259	88-5299A	R61	92-1191	14A	92-4172	22083
5299	10664	Fibroin 1	1	-	-	-	-	-	-	-	-	-	-	-	-	-	-	-	-	-	-	-	-	-	-	-	-	-	-	-	-	-	-	-	-	-	-	-	-	-	-	-	-	-	+	-	-	-	-	-	-	-	-	-	-	-
5300	10665	hypothetical protein	1	-	-	-	-	-	-	-	-	-	-	-	-	-	-	-	-	-	-	-	-	-	-	-	-	-	-	-	-	-	-	-	-	-	-	-	-	-	-	-	-	-	+	-	-	-	-	-	-	-	-	-	-	-
5301	10666	Transcriptional regulator	1	-	-	-	-	-	-	-	-	-	-	-	-	-	-	-	-	-	-	-	-	-	-	-	-	-	-	-	-	-	-	-	-	-	-	-	-	-	-	-	-	-	+	-	-	-	-	-	-	-	-	-	-	-
5302	10667	CDS_ID OB3334	1	-	-	-	-	-	-	-	-	-	-	-	-	-	-	-	-	-	-	-	-	-	-	-	-	-	-	-	-	-	-	-	-	-	-	-	-	-	-	-	-	-	+	-	-	-	-	-	-	-	-	-	-	-
5303	10668	Parallel beta-helix repeat	1	-	-	-	-	-	-	-	-	-	-	-	-	-	-	-	-	-	-	-	-	-	-	+	-	-	-	-	-	-	-	-	-	-	-	-	-	-	-	-	-	-	-	-	-	-	-	-	-	-	-	-	-	-
5304	10669	Muramoyltetrapeptide carboxypeptidase (EC 3.4.17.13)	1	-	-	-	-	-	-	-	-	-	-	-	-	-	-	-	-	-	-	-	-	-	-	+	-	-	-	-	-	-	-	-	-	-	-	-	-	-	-	-	-	-	-	-	-	-	-	-	-	-	-	-	-	-
5305	10670	hypothetical protein	1	-	-	-	-	-	-	-	-	-	-	-	-	-	-	-	-	-	-	-	-	-	-	+	-	-	-	-	-	-	-	-	-	-	-	-	-	-	-	-	-	-	-	-	-	-	-	-	-	-	-	-	-	-
5306	10671	hypothetical protein	1	-	-	-	-	-	-	-	-	-	-	-	-	-	-	-	-	-	-	-	-	-	-	+	-	-	-	-	-	-	-	-	-	-	-	-	-	-	-	-	-	-	-	-	-	-	-	-	-	-	-	-	-	-
5307	10672	putative replication initiator protein	1	-	-	-	-	-	-	-	-	-	-	-	-	-	-	-	-	-	-	-	-	-	-	+	-	-	-	-	-	-	-	-	-	-	-	-	-	-	-	-	-	-	-	-	-	-	-	-	-	-	-	-	-	-
5308	10673	Parallel beta-helix repeat	1	-	-	-	-	-	-	-	-	-	-	-	-	-	-	-	-	-	-	-	-	-	-	+	-	-	-	-	-	-	-	-	-	-	-	-	-	-	-	-	-	-	-	-	-	-	-	-	-	-	-	-	-	-
5309	10674	immunity protein	1	-	-	-	-	-	-	-	-	-	-	-	-	-	-	-	-	-	-	-	-	-	-	+	-	-	-	-	-	-	-	-	-	-	-	-	-	-	-	-	-	-	-	-	-	-	-	-	-	-	-	-	-	-
5310	10675	hypothetical protein	1	-	-	-	-	-	-	-	-	-	-	-	-	-	-	-	-	-	-	-	-	-	-	+	-	-	-	-	-	-	-	-	-	-	-	-	-	-	-	-	-	-	-	-	-	-	-	-	-	-	-	-	-	-
5311	10676	hypothetical protein	1	-	-	-	-	-	-	-	-	-	-	-	-	-	-	-	-	-	-	-	-	-	-	+	-	-	-	-	-	-	-	-	-	-	-	-	-	-	-	-	-	-	-	-	-	-	-	-	-	-	-	-	-	-
5312	10677	hypothetical protein	1	-	-	-	-	-	-	-	-	-	-	-	-	-	-	-	-	-	-	-	-	-	-	+	-	-	-	-	-	-	-	-	-	-	-	-	-	-	-	-	-	-	-	-	-	-	-	-	-	-	-	-	-	-
5313	10678	Parallel beta-helix repeat	1	-	-	-	-	-	-	-	-	-	-	-	-	-	-	-	-	-	-	-	-	-	-	+	-	-	-	-	-	-	-	-	-	-	-	-	-	-	-	-	-	-	-	-	-	-	-	-	-	-	-	-	-	-
5314	10679	pore-forming peptide	1	-	-	-	-	-	-	-	-	-	-	-	-	-	-	-	-	-	-	-	-	-	-	+	-	-	-	-	-	-	-	-	-	-	-	-	-	-	-	-	-	-	-	-	-	-	-	-	-	-	-	-	-	-
5315	10680	hypothetical protein	1	-	-	-	-	-	-	-	-	-	-	-	-	-	-	-	-	-	-	-	-	-	-	+	-	-	-	-	-	-	-	-	-	-	-	-	-	-	-	-	-	-	-	-	-	-	-	-	-	-	-	-	-	-
5316	10681	hypothetical protein	1	-	-	-	-	-	-	-	-	-	-	-	-	-	-	-	-	-	-	-	-	-	-	+	-	-	-	-	-	-	-	-	-	-	-	-	-	-	-	-	-	-	-	-	-	-	-	-	-	-	-	-	-	-
5317	10682	hypothetical protein	1	-	-	-	-	-	-	-	-	-	-	-	-	-	-	-	-	-	-	-	-	-	-	+	-	-	-	-	-	-	-	-	-	-	-	-	-	-	-	-	-	-	-	-	-	-	-	-	-	-	-	-	-	-
5318	10683	Serine hydroxymethyltransferase (EC 2.1.2.1)	1	-	-	-	-	-	-	-	-	-	-	-	-	-	-	-	-	-	-	-	-	-	-	+	-	-	-	-	-	-	-	-	-	-	-	-	-	-	-	-	-	-	-	-	-	-	-	-	-	-	-	-	-	-
5319	10684	hypothetical protein	1	-	-	-	-	-	-	-	-	-	-	-	-	-	-	-	-	-	-	-	-	-	-	+	-	-	-	-	-	-	-	-	-	-	-	-	-	-	-	-	-	-	-	-	-	-	-	-	-	-	-	-	-	-
5320	10685	hypothetical protein	1	-	-	-	-	-	-	-	-	-	-	-	-	-	-	-	-	-	-	-	-	-	-	+	-	-	-	-	-	-	-	-	-	-	-	-	-	-	-	-	-	-	-	-	-	-	-	-	-	-	-	-	-	-
5321	10686	hypothetical protein	1	-	-	-	-	-	-	-	-	-	-	-	-	-	-	-	+	-	-	-	-	-	-	-	-	-	-	-	-	-	-	-	-	-	-	-	-	-	-	-	-	-	-	-	-	-	-	-	-	-	-	-	-	-
5322	10687	hypothetical protein	1	-	-	-	-	-	-	-	-	-	-	-	-	-	-	-	+	-	-	-	-	-	-	-	-	-	-	-	-	-	-	-	-	-	-	-	-	-	-	-	-	-	-	-	-	-	-	-	-	-	-	-	-	-
5323	10688	Oligosaccharide repeat unit polymerase Wzy	1	-	-	-	-	-	-	-	-	-	-	-	-	-	-	-	+	-	-	-	-	-	-	-	-	-	-	-	-	-	-	-	-	-	-	-	-	-	-	-	-	-	-	-	-	-	-	-	-	-	-	-	-	-
5324	10689	hypothetical protein	1	-	-	-	-	-	-	-	-	-	-	-	-	-	-	-	+	-	-	-	-	-	-	-	-	-	-	-	-	-	-	-	-	-	-	-	-	-	-	-	-	-	-	-	-	-	-	-	-	-	-	-	-	-
5325	10690	hypothetical protein	1	-	-	-	-	-	-	-	-	-	-	-	-	-	-	-	+	-	-	-	-	-	-	-	-	-	-	-	-	-	-	-	-	-	-	-	-	-	-	-	-	-	-	-	-	-	-	-	-	-	-	-	-	-
5326	10691	YSIRK Gram-positive signal peptide	1	-	-	-	-	-	-	-	-	-	-	-	-	-	-	-	+	-	-	-	-	-	-	-	-	-	-	-	-	-	-	-	-	-	-	-	-	-	-	-	-	-	-	-	-	-	-	-	-	-	-	-	-	-
5327	10692	hypothetical protein	1	-	-	-	-	-	-	-	-	-	-	-	-	-	-	-	+	-	-	-	-	-	-	-	-	-	-	-	-	-	-	-	-	-	-	-	-	-	-	-	-	-	-	-	-	-	-	-	-	-	-	-	-	-
5328	10693	hypothetical protein	1	-	-	-	-	-	-	-	-	-	-	-	-	-	-	-	+	-	-	-	-	-	-	-	-	-	-	-	-	-	-	-	-	-	-	-	-	-	-	-	-	-	-	-	-	-	-	-	-	-	-	-	-	-
5329	10694	hypothetical protein	1	-	-	-	-	-	-	-	-	-	-	-	-	-	-	-	+	-	-	-	-	-	-	-	-	-	-	-	-	-	-	-	-	-	-	-	-	-	-	-	-	-	-	-	-	-	-	-	-	-	-	-	-	-
5330	10695	Sulfur carrier protein adenylyltransferase ThiF	1	-	-	-	-	-	-	-	-	-	-	-	-	-	-	-	+	-	-	-	-	-	-	-	-	-	-	-	-	-	-	-	-	-	-	-	-	-	-	-	-	-	-	-	-	-	-	-	-	-	-	-	-	-
5331	10696	hypothetical protein	1	-	-	-	-	-	-	-	-	-	-	-	-	-	-	-	+	-	-	-	-	-	-	-	-	-	-	-	-	-	-	-	-	-	-	-	-	-	-	-	-	-	-	-	-	-	-	-	-	-	-	-	-	-
5332	10697	protein of unknown function DUF262	1	-	-	-	-	-	-	-	-	-	-	-	-	-	-	-	+	-	-	-	-	-	-	-	-	-	-	-	-	-	-	-	-	-	-	-	-	-	-	-	-	-	-	-	-	-	-	-	-	-	-	-	-	-
5333	10698	Export protein for polysaccharides and teichoic acids	1	-	-	-	-	-	-	-	-	-	-	-	-	-	-	-	+	-	-	-	-	-	-	-	-	-	-	-	-	-	-	-	-	-	-	-	-	-	-	-	-	-	-	-	-	-	-	-	-	-	-	-	-	-
5334	10699	hypothetical protein	1	-	-	-	-	-	-	-	-	-	-	-	-	-	-	-	+	-	-	-	-	-	-	-	-	-	-	-	-	-	-	-	-	-	-	-	-	-	-	-	-	-	-	-	-	-	-	-	-	-	-	-	-	-
5335	10700	hypothetical protein	1	-	-	-	-	-	-	-	-	-	-	-	-	-	-	-	+	-	-	-	-	-	-	-	-	-	-	-	-	-	-	-	-	-	-	-	-	-	-	-	-	-	-	-	-	-	-	-	-	-	-	-	-	-
5336	10701	hypothetical protein	1	-	-	-	-	-	-	-	-	-	-	-	-	-	-	-	+	-	-	-	-	-	-	-	-	-	-	-	-	-	-	-	-	-	-	-	-	-	-	-	-	-	-	-	-	-	-	-	-	-	-	-	-	-
5337	10702	ABC-type multidrug transport system	1	-	-	-	-	-	-	-	-	-	-	-	-	-	-	-	+	-	-	-	-	-	-	-	-	-	-	-	-	-	-	-	-	-	-	-	-	-	-	-	-	-	-	-	-	-	-	-	-	-	-	-	-	-
5338	10703	hypothetical protein	1	-	-	-	-	-	-	-	-	-	-	-	-	-	-	-	+	-	-	-	-	-	-	-	-	-	-	-	-	-	-	-	-	-	-	-	-	-	-	-	-	-	-	-	-	-	-	-	-	-	-	-	-	-
5339	10704	hypothetical protein	1	-	-	-	-	-	-	-	-	-	-	-	-	-	-	-	+	-	-	-	-	-	-	-	-	-	-	-	-	-	-	-	-	-	-	-	-	-	-	-	-	-	-	-	-	-	-	-	-	-	-	-	-	-
5340	10705	hypothetical protein	1	-	-	-	-	-	-	-	-	-	-	-	-	-	-	-	+	-	-	-	-	-	-	-	-	-	-	-	-	-	-	-	-	-	-	-	-	-	-	-	-	-	-	-	-	-	-	-	-	-	-	-	-	-
5341	10706	hypothetical protein	1	-	-	-	-	-	-	-	-	-	-	-	-	-	-	-	+	-	-	-	-	-	-	-	-	-	-	-	-	-	-	-	-	-	-	-	-	-	-	-	-	-	-	-	-	-	-	-	-	-	-	-	-	-
5342	10707	hypothetical protein	1	-	-	-	-	-	-	-	-	-	-	-	-	-	-	-	+	-	-	-	-	-	-	-	-	-	-	-	-	-	-	-	-	-	-	-	-	-	-	-	-	-	-	-	-	-	-	-	-	-	-	-	-	-
5343	10708	hypothetical protein	1	-	-	-	-	-	-	-	-	-	-	-	-	-	-	-	+	-	-	-	-	-	-	-	-	-	-	-	-	-	-	-	-	-	-	-	-	-	-	-	-	-	-	-	-	-	-	-	-	-	-	-	-	-
5344	10709	Export protein for polysaccharides and teichoic acids	1	-	-	-	-	-	-	-	-	-	-	-	-	-	-	-	+	-	-	-	-	-	-	-	-	-	-	-	-	-	-	-	-	-	-	-	-	-	-	-	-	-	-	-	-	-	-	-	-	-	-	-	-	-
5345	10710	hypothetical protein	1	-	-	-	-	-	-	-	-	-	-	-	-	-	-	-	+	-	-	-	-	-	-	-	-	-	-	-	-	-	-	-	-	-	-	-	-	-	-	-	-	-	-	-	-	-	-	-	-	-	-	-	-	-
5346	10711	UDP-galactopyranose mutase (EC 5.4.99.9)	1	-	-	-	-	-	-	-	-	-	-	-	-	-	-	-	+	-	-	-	-	-	-	-	-	-	-	-	-	-	-	-	-	-	-	-	-	-	-	-	-	-	-	-	-	-	-	-	-	-	-	-	-	-
5347	10712	ABC-type multidrug transport system	1	-	-	-	-	-	-	-	-	-	-	-	-	-	-	-	+	-	-	-	-	-	-	-	-	-	-	-	-	-	-	-	-	-	-	-	-	-	-	-	-	-	-	-	-	-	-	-	-	-	-	-	-	-
5348	10713	hypothetical protein	1	-	-	-	-	-	-	-	-	-	-	-	-	-	-	-	+	-	-	-	-	-	-	-	-	-	-	-	-	-	-	-	-	-	-	-	-	-	-	-	-	-	-	-	-	-	-	-	-	-	-	-	-	-
5349	10714	hypothetical protein	1	-	-	-	-	-	-	-	-	-	-	-	-	-	-	-	+	-	-	-	-	-	-	-	-	-	-	-	-	-	-	-	-	-	-	-	-	-	-	-	-	-	-	-	-	-	-	-	-	-	-	-	-	-
5350	10715	hypothetical protein	1	-	-	-	-	-	-	-	-	-	-	-	-	-	-	-	+	-	-	-	-	-	-	-	-	-	-	-	-	-	-	-	-	-	-	-	-	-	-	-	-	-	-	-	-	-	-	-	-	-	-	-	-	-
5351	10716	hypothetical protein	1	-	-	-	-	-	-	-	-	-	-	-	-	-	-	-	+	-	-	-	-	-	-	-	-	-	-	-	-	-	-	-	-	-	-	-	-	-	-	-	-	-	-	-	-	-	-	-	-	-	-	-	-	-
5352	10717	ABC-type multidrug transport system	1	-	-	-	-	-	-	-	-	-	-	-	-	-	-	-	+	-	-	-	-	-	-	-	-	-	-	-	-	-	-	-	-	-	-	-	-	-	-	-	-	-	-	-	-	-	-	-	-	-	-	-	-	-
5353	10718	hypothetical protein	1	-	-	-	-	-	-	-	-	-	-	-	-	-	-	-	+	-	-	-	-	-	-	-	-	-	-	-	-	-	-	-	-	-	-	-	-	-	-	-	-	-	-	-	-	-	-	-	-	-	-	-	-	-
5354	10719	hypothetical protein	1	-	-	-	-	-	-	-	-	-	-	-	-	-	-	-	+	-	-	-	-	-	-	-	-	-	-	-	-	-	-	-	-	-	-	-	-	-	-	-	-	-	-	-	-	-	-	-	-	-	-	-	-	-
5355	10720	hypothetical protein	1	-	-	-	-	-	-	-	-	-	-	-	-	-	-	-	-	-	-	-	-	-	-	-	-	-	-	-	-	-	-	-	-	-	-	-	-	-	-	-	-	-	-	-	-	-	-	-	-	-	-	-	-	+
5356	10721	hypothetical protein	1	-	-	-	-	-	-	-	-	-	-	-	-	-	-	-	-	-	-	-	-	-	-	-	-	-	-	-	-	-	-	-	-	-	-	-	-	-	-	-	-	-	-	-	-	-	-	-	-	-	-	-	-	+
5357	10722	hypothetical protein	1	-	-	-	-	-	-	-	-	-	-	-	-	-	-	-	-	-	-	-	-	-	-	-	-	-	-	-	-	-	-	-	-	-	-	-	-	-	-	-	-	-	-	-	-	-	-	-	-	-	-	-	-	+
5358	10723	hypothetical protein	1	-	-	-	-	-	-	-	-	-	-	-	-	-	-	-	-	-	-	-	-	-	-	-	-	-	-	-	-	-	-	-	-	-	-	-	-	-	-	-	-	-	-	-	-	-	-	-	-	-	-	-	-	+
5359	10724	hypothetical protein	1	-	-	-	-	-	-	-	-	-	-	-	-	-	-	-	-	-	-	-	-	-	-	-	-	-	-	-	-	-	-	-	-	-	-	-	-	-	-	-	-	-	-	-	-	-	-	-	-	-	-	-	-	+
5360	10725	hypothetical protein	1	-	-	-	-	-	-	-	-	-	-	-	-	-	-	-	-	-	-	-	-	-	-	-	-	-	-	-	-	-	-	-	-	-	-	-	-	-	-	-	-	-	-	-	-	-	-	-	-	-	-	-	-	+
5361	10726	RNA-directed DNA polymerase (Reverse transcriptase)	1	-	-	-	-	-	-	-	-	-	-	-	-	-	-	-	-	-	-	-	-	-	-	-	-	-	-	-	-	-	-	-	-	-	-	-	-	-	-	-	-	-	-	-	-	-	-	-	-	-	-	-	-	+
5362	10727	hypothetical protein	1	-	-	-	-	-	-	-	-	-	-	-	-	-	-	-	-	-	-	-	-	-	-	-	-	-	-	-	-	-	-	-	-	-	-	-	-	-	-	-	-	-	-	-	-	-	-	-	-	-	-	-	-	+
5363	10728	hypothetical protein	1	-	-	-	-	-	-	-	-	-	-	-	-	-	-	-	-	-	-	-	-	-	-	-	-	-	-	-	-	-	-	-	-	-	-	-	-	-	-	-	-	-	-	-	-	-	-	-	-	-	-	-	-	+
5364	10729	hypothetical protein	1	-	-	-	-	-	-	-	-	-	-	-	-	-	-	-	-	-	-	-	-	-	-	-	-	-	-	-	-	-	-	-	-	-	-	-	-	-	-	-	-	-	-	-	-	-	-	-	-	-	-	-	-	+
5365	10730	putative 5-methylcytosine methyltransferase	1	-	-	-	-	-	-	-	-	-	-	-	-	-	-	-	-	-	-	-	-	-	-	-	-	-	-	-	-	-	-	-	-	-	-	-	-	-	-	-	-	-	-	-	-	-	-	-	-	-	-	-	-	+
5366	10731	Mobile element protein	1	-	-	-	-	-	-	-	-	-	-	-	-	-	-	-	-	-	-	-	-	-	-	-	-	-	-	-	-	-	-	-	-	-	-	-	-	-	-	-	-	-	-	-	-	-	-	-	-	-	-	-	-	+
5367	10732	R5 protein	1	-	-	-	-	-	-	-	-	-	-	-	-	-	-	-	-	-	-	-	-	-	-	-	-	-	-	-	-	-	-	-	-	-	-	-	-	-	-	-	-	-	-	-	-	-	-	-	-	-	-	-	-	+
5368	10733	hypothetical protein	1	-	-	-	-	-	-	-	-	-	-	-	-	-	-	-	-	-	-	-	-	-	-	-	-	-	-	-	-	-	-	-	-	-	-	-	-	-	-	-	-	-	-	-	-	-	-	-	-	-	-	-	-	+
5369	10734	hypothetical protein	1	-	-	-	-	-	-	-	-	-	-	-	-	-	-	-	-	-	-	-	-	-	-	-	-	-	-	-	-	-	-	-	-	-	-	-	-	-	-	-	-	-	-	-	-	-	-	-	-	-	-	-	-	+
5370	10735	hypothetical protein	1	-	-	-	-	-	-	-	-	-	-	-	-	-	-	-	-	-	-	-	-	-	-	-	-	-	-	-	-	-	-	-	-	-	-	-	-	-	-	-	-	-	-	-	-	-	-	-	-	-	-	-	-	+
5371	10736	hypothetical protein	1	-	-	-	-	-	-	-	-	-	-	-	-	-	-	-	-	-	-	-	-	-	-	-	-	-	-	-	-	-	-	-	-	-	-	-	-	-	-	-	-	-	-	-	-	-	-	-	-	-	-	-	-	+
5372	10737	hypothetical protein	1	-	-	-	-	-	-	-	-	-	-	-	-	-	-	-	-	-	-	-	-	-	-	-	-	-	-	-	-	-	-	-	-	-	-	-	-	-	-	-	-	-	-	-	-	-	-	-	-	-	-	-	-	+
5373	10738	hypothetical protein	1	-	-	-	-	-	-	-	-	-	-	-	-	-	-	-	-	-	-	-	-	-	-	-	-	-	-	-	-	-	-	-	-	-	-	-	-	-	-	-	-	-	-	-	-	-	-	-	-	-	-	-	-	+
5374	10739	site-specific DNA methylase	1	-	-	-	-	-	-	-	-	-	-	-	-	-	-	-	-	-	-	-	-	-	-	-	-	-	-	-	-	-	-	-	-	-	-	-	-	-	-	-	-	-	-	-	-	-	-	-	-	-	-	-	-	+
5375	10740	hypothetical protein	1	-	-	-	-	-	-	-	-	-	-	-	-	-	-	-	-	-	-	-	-	-	-	-	-	-	-	-	-	-	-	-	-	-	-	-	-	-	-	-	-	-	-	-	-	-	-	-	-	-	-	-	-	+
5376	10741	hypothetical protein	1	-	-	-	-	-	-	-	-	-	-	-	-	-	-	-	-	-	-	-	-	-	-	-	-	-	-	-	-	-	-	-	-	-	-	-	-	-	-	-	-	-	-	-	-	-	-	-	-	-	-	-	-	+
5377	10742	hypothetical protein	1	-	-	-	-	-	-	-	-	-	-	-	-	-	-	-	-	-	-	-	-	-	-	-	-	-	-	-	-	-	-	-	-	-	-	-	-	-	-	-	-	-	-	-	-	-	-	-	-	-	-	-	-	+
5378	10743	hypothetical protein	1	-	-	-	-	-	-	-	-	-	-	-	-	-	-	-	-	-	-	-	-	-	-	-	-	-	-	-	-	-	-	-	-	-	-	-	-	-	-	-	-	-	-	-	-	-	-	-	-	-	-	-	-	+
5379	10744	NAD-dependent epimerase/dehydratase	1	-	-	-	-	-	-	-	-	-	-	-	-	-	-	-	-	-	-	-	-	-	-	-	-	-	-	-	-	-	-	-	-	-	-	-	-	-	-	-	-	-	-	-	-	-	-	-	-	-	-	-	-	+
5380	10745	putative lipoprotein	1	-	-	-	-	-	-	-	-	-	-	-	-	-	-	-	-	-	-	-	-	-	-	-	-	-	-	-	-	-	-	-	-	-	-	-	-	-	-	-	-	-	-	-	-	-	-	-	-	-	-	-	-	+
5381	10746	Collagen alpha-1(IV) chain precursor (Arresten)	1	-	-	-	-	-	-	-	-	-	-	-	-	-	-	-	-	-	-	-	-	-	-	-	-	-	-	-	-	-	-	-	-	-	-	-	-	-	-	-	-	-	-	-	-	-	-	-	-	-	-	-	-	+
5382	10747	hypothetical protein	1	-	-	-	-	-	-	-	-	-	-	-	-	-	-	-	-	-	-	-	-	-	-	-	-	-	-	-	-	-	-	-	-	-	-	-	-	-	-	-	-	-	-	-	-	-	-	-	-	-	-	-	-	+
5383	10748	hypothetical protein	1	-	-	-	-	-	-	-	-	-	-	-	-	-	-	-	-	-	-	-	-	-	-	-	-	-	-	-	-	-	-	-	-	-	-	-	-	-	-	-	-	-	-	-	-	-	-	-	-	-	-	-	-	+
5384	10749	hypothetical protein	1	-	-	-	-	-	-	-	-	-	-	-	-	-	-	-	-	-	-	-	-	-	-	-	-	-	-	-	-	-	-	-	-	-	-	-	-	-	-	-	-	-	-	-	-	-	-	-	-	-	-	-	-	+
5385	10750	hypothetical protein	1	-	-	-	-	-	-	-	-	-	-	-	-	-	-	-	-	-	-	-	-	-	-	-	-	-	-	-	-	-	-	-	-	-	-	-	-	-	-	-	-	-	-	-	-	-	-	-	-	-	-	-	-	+
5386	10751	hypothetical protein	1	-	-	-	-	-	-	-	-	-	-	-	-	-	-	-	-	-	-	-	-	-	-	-	-	-	-	-	-	-	-	-	-	-	-	-	-	-	-	-	-	-	-	-	-	-	-	-	-	-	-	-	-	+
5387	10752	hypothetical protein	1	-	-	-	-	-	-	-	-	-	-	-	-	-	-	-	-	-	-	-	-	-	-	-	-	-	-	-	-	-	-	-	-	-	-	-	-	-	-	-	-	-	-	-	-	-	-	-	-	-	-	-	-	+
5388	10753	hypothetical protein	1	-	-	-	-	-	-	-	-	-	-	-	-	-	-	-	-	-	-	-	-	-	-	-	-	-	-	-	-	-	-	-	-	-	-	-	-	-	-	-	-	-	-	-	-	-	-	-	-	-	-	-	-	+
5389	10754	hypothetical protein	1	-	-	-	-	-	-	-	-	-	-	-	-	-	-	-	-	-	-	-	-	-	-	-	-	-	-	-	-	-	-	-	-	-	-	-	-	-	-	-	-	-	-	-	-	-	-	-	-	-	-	-	-	+
5390	10755	Type II restriction enzyme HphI (EC 3.1.21.4) (Endonuclease HphI) (R.HphI)	1	-	-	-	-	-	-	-	-	-	-	-	-	-	-	-	-	-	-	-	-	-	-	-	-	-	-	-	-	-	-	-	-	-	-	-	-	-	-	-	-	-	-	-	-	-	-	-	-	-	-	-	-	+
5391	10756	Transcriptional regulator	1	-	-	-	-	-	-	-	-	-	-	-	-	-	-	-	-	-	-	-	-	-	-	-	-	-	-	-	-	-	-	-	-	-	-	-	-	-	-	-	-	-	-	-	-	-	-	-	-	-	-	-	-	+
5392	10757	Flavin reductase (EC 1.5.1.30)	1	-	-	-	-	-	-	-	-	-	-	-	-	-	-	-	-	-	-	-	-	-	-	-	-	-	-	-	-	-	-	-	-	-	-	-	-	-	-	-	-	-	-	-	-	-	-	-	-	-	-	-	-	+
5393	10758	hypothetical protein	1	-	-	-	-	-	-	-	-	-	-	-	-	-	-	-	-	-	-	-	-	-	-	-	-	-	-	-	-	-	-	-	-	-	-	-	-	-	-	-	-	-	-	-	-	-	-	-	-	-	-	-	-	+
5394	10759	hypothetical protein	1	-	-	-	-	-	-	-	-	-	-	-	-	-	-	-	-	-	-	-	-	-	-	-	-	-	-	-	-	-	-	-	-	-	-	-	-	-	-	-	-	-	-	-	-	-	-	-	-	-	-	-	-	+
5395	10760	hypothetical protein	1	-	-	-	-	-	-	-	-	-	-	-	-	-	-	-	-	-	-	-	-	-	-	-	-	-	-	-	-	-	-	-	-	-	-	-	-	-	-	-	-	-	-	-	-	-	-	-	-	-	-	-	-	+
5396	10761	hypothetical protein	1	-	-	-	-	-	-	-	-	-	-	-	-	-	-	-	-	-	-	-	-	-	-	-	-	-	-	-	-	-	-	-	-	-	-	-	-	-	-	-	-	-	-	-	-	-	-	-	-	-	-	-	-	+
5397	10762	hypothetical protein	1	-	-	-	-	-	-	-	-	-	-	-	-	-	-	-	-	-	-	-	-	-	-	-	-	-	-	-	-	-	-	-	-	-	-	-	-	-	-	-	-	-	-	-	-	-	-	-	-	-	-	-	-	+
5398	10763	hypothetical protein	1	-	-	-	-	-	-	-	-	-	-	-	-	-	-	-	-	-	-	-	-	-	-	-	-	-	-	-	-	-	-	-	-	-	-	-	-	-	-	-	-	-	-	-	-	-	-	-	-	-	-	-	-	+
5399	10764	transcriptional regulator	1	-	-	-	-	-	-	-	-	-	-	-	-	-	-	-	-	-	-	-	-	-	-	-	-	-	-	-	-	-	-	-	-	-	-	-	-	-	-	-	-	-	-	-	-	-	-	-	-	-	-	-	-	+
5400	10765	hypothetical protein	1	-	-	-	-	-	-	-	-	-	-	-	-	-	-	-	-	-	-	-	-	-	-	-	-	-	-	-	-	-	-	-	-	-	-	-	-	-	-	-	-	-	-	-	-	-	-	-	-	-	-	-	-	+
5401	10766	hypothetical protein	1	-	-	-	-	-	-	-	-	-	-	-	-	-	-	-	-	-	-	-	-	-	-	-	-	-	-	-	-	-	-	-	-	-	-	-	-	-	-	-	-	-	-	-	-	-	-	-	-	-	-	-	-	+
5402	10767	hypothetical protein	1	-	-	-	-	-	-	-	-	-	-	-	-	-	-	-	-	-	-	-	-	-	-	-	-	-	-	-	-	-	-	-	-	-	-	-	-	-	-	-	-	-	-	-	-	-	-	-	-	-	-	-	-	+
5403	10768	GCN5-related N-acetyltransferase	1	-	-	-	-	-	-	-	-	-	-	-	-	-	-	-	-	-	-	-	-	-	-	-	-	-	-	-	-	-	-	-	-	-	-	-	-	-	-	-	-	-	-	-	-	-	-	-	-	-	-	-	-	+
5404	10769	hypothetical protein	1	-	-	-	-	-	-	-	-	-	-	-	-	-	-	-	-	-	-	-	-	-	-	-	-	-	-	-	-	-	-	-	-	-	-	-	-	-	-	-	-	-	-	-	-	-	-	-	-	-	-	-	-	+
5405	10770	hypothetical protein	1	-	-	-	-	-	-	-	-	-	-	-	-	-	-	-	-	-	-	-	-	-	-	-	-	-	-	-	-	-	-	-	-	-	-	-	-	-	-	-	-	-	-	-	-	-	-	-	-	-	-	-	-	+
5406	10771	hypothetical protein	1	-	-	-	-	-	-	-	-	-	-	-	-	-	-	-	-	-	-	-	-	-	-	-	-	-	-	-	-	-	-	-	-	-	-	-	-	-	-	-	-	-	-	-	-	-	-	-	-	-	-	-	-	+
5407	10772	hypothetical protein	1	-	-	-	-	-	-	-	-	-	-	-	-	-	-	-	-	-	-	-	-	-	-	-	-	-	-	-	-	-	-	-	-	-	-	-	-	-	-	-	-	-	-	-	-	-	-	-	-	-	-	-	-	+
5408	10773	hypothetical protein	1	-	-	-	-	-	-	-	-	-	-	-	-	-	-	-	-	-	-	-	-	-	-	-	-	-	-	-	-	-	-	-	-	-	-	-	-	-	-	-	-	-	-	-	-	-	-	-	-	-	-	-	-	+
5409	10774	hypothetical protein	1	-	-	-	-	-	-	-	-	-	-	-	-	-	-	-	-	-	-	-	-	-	-	-	-	-	-	-	-	-	-	-	-	-	-	-	-	-	-	-	-	-	-	-	-	-	-	-	-	-	-	-	-	+
5410	10775	hypothetical protein	1	-	-	-	-	-	-	-	-	-	-	-	-	-	-	-	-	-	-	-	-	-	-	-	-	-	-	-	-	-	-	-	-	-	-	-	-	-	-	-	-	-	-	-	-	-	-	-	-	-	-	-	-	+
5411	10776	hypothetical protein	1	-	-	-	-	-	-	-	-	-	-	-	-	-	-	-	-	-	-	-	-	-	-	-	-	-	-	-	-	-	-	-	-	-	-	-	-	-	-	-	-	-	-	-	-	-	-	-	-	-	-	-	-	+
5412	10777	NAD-dependent protein deacetylase of SIR2 family	1	-	-	-	-	-	-	-	-	-	-	-	-	-	-	-	-	-	-	-	-	-	-	-	-	-	-	-	-	-	-	-	-	-	-	-	-	-	-	-	-	-	-	-	-	-	-	-	-	-	-	-	-	+
5413	10778	hypothetical protein	1	-	-	-	-	-	-	-	-	-	-	-	-	-	-	-	-	-	-	-	-	-	-	-	-	-	-	-	-	-	-	-	-	-	-	-	-	-	-	-	-	-	-	-	-	-	-	-	-	-	-	-	-	+
5414	10779	hypothetical protein	1	-	-	-	-	-	-	-	-	-	-	-	-	-	-	-	-	-	-	-	-	-	-	-	-	-	-	-	-	-	-	-	-	-	-	-	-	-	-	-	-	-	-	-	-	-	-	-	-	-	-	-	-	+
5415	10780	hypothetical protein	1	-	-	-	-	-	-	-	-	-	-	-	-	-	-	-	-	-	-	-	-	-	-	-	-	-	-	-	-	-	-	-	-	-	-	-	-	-	-	-	-	-	-	-	-	-	-	-	-	-	-	-	-	+
5416	10781	hypothetical protein	1	-	-	-	-	-	-	-	-	-	-	-	-	-	-	-	-	-	-	-	-	-	-	-	-	-	-	-	-	-	-	-	-	-	-	-	-	-	-	-	-	-	-	-	-	-	-	-	-	-	-	-	-	+
5417	10782	hypothetical protein	1	-	-	-	-	-	-	-	-	-	-	-	-	-	-	-	-	-	-	-	-	-	-	-	-	-	-	-	-	-	-	-	-	-	-	-	-	-	-	-	-	-	-	-	-	-	-	-	-	-	-	-	-	+
5418	10783	hypothetical protein	1	-	-	-	-	-	-	-	-	-	-	-	-	-	-	-	-	-	-	-	-	-	-	-	-	-	-	-	-	-	-	-	-	-	-	-	-	-	-	-	-	-	-	-	-	-	-	-	-	-	-	-	-	+
5419	10784	Alpha-amylase (EC 3.2.1.1)	1	-	-	-	-	-	-	-	-	-	-	-	-	-	-	-	-	-	-	-	-	-	-	-	-	-	-	-	-	-	-	-	-	-	-	-	-	-	-	-	-	-	-	-	-	-	-	-	-	-	-	-	-	+
5420	10785	hypothetical protein	1	-	-	-	-	-	-	-	-	-	-	-	-	-	-	-	-	-	-	-	-	-	-	-	-	-	-	-	-	-	-	-	-	-	-	-	-	-	-	-	-	-	-	-	-	-	-	-	-	-	-	-	-	+
5421	10786	hypothetical protein	1	-	-	-	-	-	-	-	-	-	-	-	-	-	-	-	-	-	-	-	-	-	-	-	-	-	-	-	-	-	-	-	-	-	-	-	-	-	-	-	-	-	-	-	+	-	-	-	-	-	-	-	-	-
5422	10787	two-component sensor histidine kinase	1	-	-	-	-	-	-	-	-	-	-	-	-	-	-	-	-	-	-	-	-	-	-	-	-	-	-	-	-	-	-	-	-	-	-	-	-	-	-	-	-	-	-	-	+	-	-	-	-	-	-	-	-	-
5423	10788	hypothetical protein	1	-	-	-	-	-	-	-	-	-	-	-	-	-	-	-	-	-	-	-	-	-	-	-	-	-	-	-	-	-	-	-	-	-	-	-	-	-	-	-	-	-	-	-	+	-	-	-	-	-	-	-	-	-
5424	10789	hypothetical protein	1	-	-	-	-	-	-	-	-	-	-	-	-	-	-	-	-	-	-	-	-	-	-	-	-	-	-	-	-	-	-	-	-	-	-	-	-	-	-	-	-	-	-	-	+	-	-	-	-	-	-	-	-	-
5425	10790	hypothetical protein	1	-	-	-	-	-	-	-	-	-	-	-	-	-	-	-	-	-	-	-	-	-	-	-	-	-	-	-	-	-	-	-	-	-	-	-	-	-	-	-	-	-	-	-	+	-	-	-	-	-	-	-	-	-
5426	10791	hypothetical protein	1	-	-	-	-	-	-	-	-	-	-	-	-	-	-	-	-	-	-	-	-	-	-	-	-	-	-	-	-	-	-	-	-	-	-	-	-	-	-	-	-	-	-	-	+	-	-	-	-	-	-	-	-	-
5427	10792	hypothetical protein	1	-	-	-	-	-	-	-	-	-	-	-	-	-	-	-	-	-	-	-	-	-	-	-	-	-	-	-	-	-	-	-	-	-	-	-	-	-	-	-	-	-	-	-	+	-	-	-	-	-	-	-	-	-
5428	10793	hypothetical protein	1	-	-	-	-	-	-	-	-	-	-	-	-	-	-	-	-	-	-	-	-	-	-	-	-	-	-	-	-	-	-	-	-	-	-	-	-	-	-	-	-	-	-	-	+	-	-	-	-	-	-	-	-	-
5429	10794	hypothetical protein	1	-	-	-	-	-	-	-	-	-	-	-	-	-	-	-	-	-	-	-	-	-	-	-	-	-	-	-	-	-	-	-	-	-	-	-	-	-	-	-	-	-	-	-	+	-	-	-	-	-	-	-	-	-
5430	10795	Type I restriction-modification system	1	-	-	-	-	-	-	-	-	-	-	-	-	-	-	-	-	-	-	-	-	-	-	-	-	-	-	-	-	-	-	-	-	-	-	-	-	-	-	-	-	-	-	-	+	-	-	-	-	-	-	-	-	-
5431	10796	hypothetical protein	1	-	-	-	-	-	-	-	-	-	-	-	-	-	-	-	-	-	-	-	-	-	-	-	-	-	-	-	-	-	-	-	-	-	-	-	-	-	-	-	-	-	-	-	+	-	-	-	-	-	-	-	-	-
5432	10797	hypothetical protein	1	-	-	-	-	-	-	-	-	-	-	-	-	-	-	-	-	-	-	-	-	-	-	-	-	-	-	-	-	-	-	-	-	-	-	-	-	-	-	-	-	-	-	-	+	-	-	-	-	-	-	-	-	-
5433	10798	Hydroxymethylglutaryl-CoA reductase (EC 1.1.1.34)	1	-	-	-	-	-	-	-	-	-	-	-	-	-	-	-	-	-	-	-	-	-	-	-	-	-	-	-	-	-	-	-	-	-	-	-	-	-	-	-	-	-	-	-	+	-	-	-	-	-	-	-	-	-
5434	10799	hypothetical protein	1	-	-	-	-	-	-	-	-	-	-	-	-	-	-	-	-	-	-	-	-	-	-	-	-	-	-	-	-	-	-	-	-	-	-	-	-	-	-	-	-	-	-	-	+	-	-	-	-	-	-	-	-	-
5435	10800	polysaccharide biosynthesis protein CpsL	1	-	-	-	-	-	-	-	-	-	-	-	-	-	-	-	-	-	-	-	-	-	-	-	-	-	-	-	-	-	-	-	-	-	-	-	-	-	-	-	-	-	-	-	+	-	-	-	-	-	-	-	-	-
5436	10801	putative transposase	1	-	-	-	-	-	-	-	-	-	-	-	-	-	-	-	-	-	-	-	-	-	-	-	-	-	-	-	-	-	-	-	-	-	-	-	-	-	-	-	-	-	-	-	+	-	-	-	-	-	-	-	-	-
5437	10802	hypothetical protein	1	-	-	-	-	-	-	-	-	-	-	-	-	-	-	-	-	-	-	-	-	-	-	-	-	-	-	-	-	-	-	-	-	-	-	-	-	-	-	-	-	-	-	-	+	-	-	-	-	-	-	-	-	-
5438	10803	hypothetical protein	1	-	-	-	-	-	-	-	-	-	-	-	-	-	-	-	-	-	-	-	-	-	-	-	-	-	-	-	-	-	-	-	-	-	-	-	-	-	-	-	-	-	-	-	+	-	-	-	-	-	-	-	-	-
5439	10804	hypothetical protein	1	-	-	-	-	-	-	-	-	-	-	-	-	-	-	-	-	-	-	-	-	-	-	-	-	-	-	-	-	-	-	-	-	-	-	-	-	-	-	-	-	-	-	-	+	-	-	-	-	-	-	-	-	-
5440	10805	NisK	1	-	-	-	-	-	-	-	-	-	-	-	-	-	-	-	-	-	-	-	-	-	-	-	-	-	-	-	-	-	-	-	-	-	-	-	-	-	-	-	-	-	-	-	+	-	-	-	-	-	-	-	-	-
5441	10806	hypothetical protein	1	-	-	-	-	-	-	-	-	-	-	-	-	-	-	-	-	-	-	-	-	-	-	-	-	-	-	-	-	-	-	-	-	-	-	-	-	-	-	-	-	-	-	-	+	-	-	-	-	-	-	-	-	-
5442	10807	Integral membrane protein	1	-	-	-	-	-	-	-	-	-	-	-	-	-	-	-	-	-	-	-	-	-	-	-	-	-	-	-	-	-	-	-	-	-	-	-	-	-	-	-	-	-	-	-	+	-	-	-	-	-	-	-	-	-
5443	10808	possible EA31 gene protein	1	-	-	-	-	-	-	-	-	-	-	-	-	-	-	-	-	-	-	-	-	-	-	-	-	-	-	-	-	-	-	-	-	-	-	-	-	-	-	-	-	-	-	-	+	-	-	-	-	-	-	-	-	-
5444	10809	hypothetical protein	1	-	-	-	-	-	-	-	-	-	-	-	-	-	-	-	-	-	-	-	-	-	-	-	-	-	-	-	-	-	-	-	-	-	-	-	-	-	-	-	-	-	-	-	+	-	-	-	-	-	-	-	-	-
5445	10810	hypothetical protein	1	-	-	-	-	-	-	-	-	-	-	-	-	-	-	-	-	-	-	-	-	-	-	-	-	-	-	-	-	-	-	-	-	-	-	-	-	-	-	-	-	-	-	-	+	-	-	-	-	-	-	-	-	-
5446	10811	Peptidase M16C associated	1	-	-	-	-	-	-	-	-	-	-	-	-	-	-	-	-	-	-	-	-	-	-	-	-	-	-	-	-	-	-	-	-	-	-	-	-	-	-	-	-	-	-	-	+	-	-	-	-	-	-	-	-	-
5447	10812	hypothetical protein	1	-	-	-	-	-	-	-	-	-	-	-	-	-	-	-	-	-	-	-	-	-	-	-	-	-	-	-	-	-	-	-	-	-	-	-	-	-	-	-	-	-	-	-	+	-	-	-	-	-	-	-	-	-
5448	10813	hypothetical protein	1	-	-	-	-	-	-	-	-	-	-	-	-	-	-	-	-	-	-	-	-	-	-	-	-	-	-	-	-	-	-	-	-	-	-	-	-	-	-	-	-	-	-	-	+	-	-	-	-	-	-	-	-	-
5449	10814	hypothetical protein	1	-	-	-	-	-	-	-	-	-	-	-	-	-	-	-	-	-	-	-	-	-	-	-	-	-	-	-	-	-	-	-	-	-	-	-	-	-	-	-	-	-	-	-	+	-	-	-	-	-	-	-	-	-
5450	10815	Retron-type reverse transcriptase	1	-	-	-	-	-	-	-	-	-	-	-	-	-	-	-	-	-	-	-	-	-	-	-	-	-	-	-	-	-	-	-	-	-	-	-	-	-	-	-	-	-	-	-	+	-	-	-	-	-	-	-	-	-
5451	10816	NisK	1	-	-	-	-	-	-	-	-	-	-	-	-	-	-	-	-	-	-	-	-	-	-	-	-	-	-	-	-	-	-	-	-	-	-	-	-	-	-	-	-	-	-	-	+	-	-	-	-	-	-	-	-	-
5452	10817	type I site-specific deoxyribonuclease	1	-	-	-	-	-	-	-	-	-	-	-	-	-	-	-	-	-	-	-	-	-	-	-	-	-	-	-	-	-	-	-	-	-	-	-	-	-	-	-	-	-	-	-	+	-	-	-	-	-	-	-	-	-
5453	10818	Chloramphenicol acetyltransferase (EC 2.3.1.28)	1	-	-	-	-	-	-	-	-	-	-	-	-	-	-	-	-	-	-	-	-	-	-	-	-	-	-	-	-	-	-	-	-	-	-	-	-	-	-	-	-	-	-	-	+	-	-	-	-	-	-	-	-	-
5454	10819	Type I restriction-modification system	1	-	-	-	-	-	-	-	-	-	-	-	-	-	-	-	-	-	-	-	-	-	-	-	-	-	-	-	-	-	-	-	-	-	-	-	-	-	-	-	-	-	-	-	+	-	-	-	-	-	-	-	-	-
5455	10820	Phosphopantothenoylcysteine decarboxylase (EC 4.1.1.36) / Phosphopantothenoylcysteine synthetase (EC 6.3.2.5)	1	-	-	-	-	-	-	-	-	-	-	-	-	-	-	-	-	-	-	-	-	-	-	-	-	-	-	-	-	-	-	-	-	-	-	-	-	-	-	-	-	-	-	-	+	-	-	-	-	-	-	-	-	-
5456	10821	pyridoxal-dependent decarboxylase	1	-	-	-	-	-	-	-	-	-	-	-	-	-	-	-	-	-	-	-	-	-	-	-	-	-	-	-	-	-	-	-	-	-	-	-	-	-	-	-	-	-	-	-	+	-	-	-	-	-	-	-	-	-
5457	10822	hypothetical protein	1	-	-	-	-	-	-	-	-	-	-	-	-	-	-	-	-	-	-	-	-	-	-	-	-	-	-	-	-	-	-	-	-	-	-	-	-	-	-	-	-	-	-	-	+	-	-	-	-	-	-	-	-	-
5458	10823	Transcriptional regulator	1	-	-	-	-	-	-	-	-	-	-	-	-	-	-	-	-	-	-	-	-	-	-	-	-	-	-	-	-	-	-	-	-	-	-	-	-	-	-	-	-	-	-	-	+	-	-	-	-	-	-	-	-	-
5459	10824	hypothetical protein	1	-	-	-	-	-	-	-	-	-	-	-	-	-	-	-	-	-	-	-	-	-	-	-	-	-	-	-	-	-	-	-	-	-	-	-	-	-	-	-	-	-	-	-	+	-	-	-	-	-	-	-	-	-
5460	10825	PTS system	1	-	-	-	-	-	-	-	-	-	-	-	-	-	-	-	-	-	-	-	-	-	-	-	-	-	-	-	-	-	-	-	-	-	-	-	-	-	-	-	-	-	-	-	+	-	-	-	-	-	-	-	-	-
5461	10826	hypothetical protein	1	-	-	-	-	-	-	-	-	-	-	-	-	-	-	-	-	-	-	-	-	-	-	-	-	-	-	-	-	-	-	-	-	-	-	-	-	-	-	-	-	-	-	-	+	-	-	-	-	-	-	-	-	-
5462	10827	hypothetical protein	1	-	-	-	-	-	-	-	-	-	-	-	-	-	-	-	-	-	-	-	-	-	-	-	-	-	-	-	-	-	-	-	-	-	-	-	-	-	-	-	-	-	-	-	+	-	-	-	-	-	-	-	-	-
5463	10828	hypothetical protein	1	-	-	-	-	-	-	-	-	-	-	-	-	-	-	-	-	-	-	-	-	-	-	-	-	-	-	-	-	-	-	-	-	-	-	-	-	-	-	-	-	-	-	-	+	-	-	-	-	-	-	-	-	-
5464	10829	hypothetical protein	1	-	-	-	-	-	-	-	-	-	-	-	-	-	-	-	-	-	-	-	-	-	-	-	-	-	-	-	-	-	-	-	-	-	-	-	-	-	-	-	-	-	-	-	+	-	-	-	-	-	-	-	-	-
5465	10830	Integral membrane protein	1	-	-	-	-	-	-	-	-	-	-	-	-	-	-	-	-	-	-	-	-	-	-	-	-	-	-	-	-	-	-	-	-	-	-	-	-	-	-	-	-	-	-	-	+	-	-	-	-	-	-	-	-	-
5466	10831	hypothetical protein	1	-	-	-	-	-	-	-	-	-	-	-	-	-	-	-	-	-	-	-	-	-	-	-	-	-	-	-	-	-	-	-	-	-	-	-	-	-	-	-	-	-	-	-	+	-	-	-	-	-	-	-	-	-
5467	10832	hypothetical protein	1	-	-	-	-	-	-	-	-	-	-	-	-	-	-	-	-	-	-	-	-	-	-	-	-	-	-	-	-	-	-	-	-	-	-	-	-	-	-	-	-	-	-	-	+	-	-	-	-	-	-	-	-	-
5468	10833	hypothetical protein	1	-	-	-	-	-	-	-	-	-	-	-	-	-	-	-	-	-	-	-	-	-	-	-	-	-	-	-	-	-	-	-	-	-	-	-	-	-	-	-	-	-	-	-	+	-	-	-	-	-	-	-	-	-
5469	10834	hypothetical protein	1	-	-	-	-	-	-	-	-	-	-	-	-	-	-	-	-	-	-	-	-	-	-	-	-	-	-	-	-	-	-	-	-	-	-	-	-	-	-	-	-	-	-	-	+	-	-	-	-	-	-	-	-	-
5470	10835	hypothetical protein	1	-	-	-	-	-	-	-	-	-	-	-	-	-	-	-	-	-	-	-	-	-	-	-	-	-	-	-	-	-	-	-	-	-	-	-	-	-	-	-	-	-	-	-	+	-	-	-	-	-	-	-	-	-
5471	10836	macrolide efflux protein	1	-	-	-	-	-	-	-	-	-	-	-	-	-	-	-	-	-	-	-	-	-	-	-	-	-	-	-	-	-	-	-	-	-	-	-	-	-	-	-	-	-	-	-	+	-	-	-	-	-	-	-	-	-
5472	10837	hypothetical protein	1	-	-	-	-	-	-	-	-	-	-	-	-	-	-	-	-	-	-	-	-	-	-	-	-	-	-	-	-	-	-	-	-	-	-	-	-	-	-	-	-	-	-	-	+	-	-	-	-	-	-	-	-	-
5473	10838	hypothetical protein	1	-	-	-	-	-	-	-	-	-	-	-	-	-	-	-	-	-	-	-	-	-	-	-	-	-	-	-	-	-	-	-	-	-	-	-	-	-	-	-	-	-	-	-	+	-	-	-	-	-	-	-	-	-
5474	10839	hypothetical protein	1	-	-	-	-	-	-	-	-	-	-	-	-	-	-	-	-	-	-	-	-	-	-	-	-	-	-	-	-	-	-	-	-	-	-	-	-	-	-	-	-	-	-	-	+	-	-	-	-	-	-	-	-	-
5475	10840	hypothetical protein	1	-	-	-	-	-	-	-	-	-	-	-	-	-	-	-	-	-	-	-	-	-	-	-	-	-	-	-	-	-	-	-	-	-	-	-	-	-	-	-	-	-	-	-	+	-	-	-	-	-	-	-	-	-
5476	10841	hypothetical protein	1	-	-	-	-	-	-	-	-	-	-	-	-	-	-	-	-	-	-	-	-	-	-	-	-	-	-	-	-	-	-	-	-	-	-	-	-	-	-	-	-	-	-	-	+	-	-	-	-	-	-	-	-	-
5477	10842	hypothetical protein	1	-	-	-	-	-	-	-	-	-	-	-	-	-	-	-	-	-	-	-	-	-	-	-	-	-	-	-	-	-	-	-	-	-	-	-	-	-	+	-	-	-	-	-	-	-	-	-	-	-	-	-	-	-
5478	10843	hypothetical protein	1	-	-	-	-	-	-	-	-	-	-	-	-	-	-	-	-	-	-	-	-	-	-	-	-	-	-	-	-	-	-	-	-	-	-	-	-	-	+	-	-	-	-	-	-	-	-	-	-	-	-	-	-	-
5479	10844	hypothetical protein	1	-	-	-	-	-	-	-	-	-	-	-	-	-	-	-	-	-	-	-	-	-	-	-	-	-	-	-	-	-	-	-	-	-	-	-	-	-	+	-	-	-	-	-	-	-	-	-	-	-	-	-	-	-
5480	10845	hypothetical protein	1	-	-	-	-	-	-	-	-	-	-	-	-	-	-	-	-	-	-	-	-	-	-	-	-	-	-	-	-	-	-	-	-	-	-	-	-	-	+	-	-	-	-	-	-	-	-	-	-	-	-	-	-	-
5481	10846	hypothetical protein	1	-	-	-	-	-	-	-	-	-	-	-	-	-	-	-	-	-	-	-	-	-	-	-	-	-	-	-	-	-	-	-	-	-	-	-	-	-	+	-	-	-	-	-	-	-	-	-	-	-	-	-	-	-

Table S5. Precence/absence of each HG among 57 *S. suis* strains

				1																13			25					28						104		94			76			53/54		Divergent *S. suis*																
Index[a]	ClutserID	Gene products	Members	98HAH33	05ZYH33	SC84	A7	SS12	P1/7	DAT273	NIAH11434	JS14	GZ1	BM407	S735	13730	MNCM07	NCTC 10446	14636	NCTC 10237	ST1	2651	89-1591	MNCM04	MNCM25	8074	D9	4961	ST3	MNCM43	DAT292	DAT260	NIAH11435	MNCM21	MNCM50	89-2479	DAT234	DAT290	93A	42A	NT77	6407	11538	8830	12814	92-1400	89-590	2726	2524	89-3576-3	4417	D12	10581	89-5259	88-5299A	R61	92-1191	14A	92-4172	22083
5482	10847	hypothetical protein	1	-	-	-	-	-	-	-	-	-	-	-	-	-	-	-	-	-	-	-	-	-	-	-	-	-	-	-	-	-	-	-	-	-	-	-	-	-	-	-	-	-	-	-	-	-	-	-	-	-	-	-	-	-	+	-	-	-	-	-	-	-	-	-	-	-	-	-	-	-
5483	10848	hypothetical protein	1	-	-	-	-	-	-	-	-	-	-	-	-	-	-	-	-	-	-	-	-	-	-	-	-	-	-	-	-	-	-	-	-	-	-	-	-	-	+	-	-	-	-	-	-	-	-	-	-	-	-	-	-	-
5484	10849	hypothetical protein	1	-	-	-	-	-	-	-	-	-	-	-	-	-	-	-	-	-	-	-	-	-	-	-	-	-	-	-	-	-	-	-	-	-	-	-	-	-	+	-	-	-	-	-	-	-	-	-	-	-	-	-	-	-
5485	10850	hypothetical protein	1	-	-	-	-	-	-	-	-	-	-	-	-	-	-	-	-	-	-	-	-	-	-	-	-	-	-	-	-	-	-	-	-	-	-	-	-	-	+	-	-	-	-	-	-	-	-	-	-	-	-	-	-	-
5486	10851	ATP-dependent DNA helicase UvrD/PcrA	1	-	-	-	-	-	-	-	-	-	-	-	-	-	-	-	-	-	-	-	-	-	-	-	-	-	-	-	-	-	-	-	-	-	-	-	-	-	+	-	-	-	-	-	-	-	-	-	-	-	-	-	-	-
5487	10852	hypothetical protein	1	-	-	-	-	-	-	-	-	-	-	-	-	-	-	-	-	-	-	-	-	-	-	-	-	-	-	-	-	-	-	-	-	-	-	-	-	-	+	-	-	-	-	-	-	-	-	-	-	-	-	-	-	-
5488	10853	putative transmembrane protein	1	-	-	-	-	-	-	-	-	-	-	-	-	-	-	-	-	-	-	-	-	-	-	-	-	-	-	-	-	-	-	-	-	-	-	-	-	-	+	-	-	-	-	-	-	-	-	-	-	-	-	-	-	-
5489	10854	hypothetical protein	1	-	-	-	-	-	-	-	-	-	-	-	-	-	-	-	-	-	-	-	-	-	-	-	-	-	-	-	-	-	-	-	-	-	-	-	-	-	+	-	-	-	-	-	-	-	-	-	-	-	-	-	-	-
5490	10855	hypothetical protein	1	-	-	-	-	-	-	-	-	-	-	-	-	-	-	-	-	-	-	-	-	-	-	-	-	-	-	-	-	-	-	-	-	-	-	-	-	-	+	-	-	-	-	-	-	-	-	-	-	-	-	-	-	-
5491	10856	hypothetical protein	1	-	-	-	-	-	-	-	-	-	-	-	-	-	-	-	-	-	-	-	-	-	-	-	-	-	-	-	-	-	-	-	-	-	-	-	-	-	+	-	-	-	-	-	-	-	-	-	-	-	-	-	-	-
5492	10857	Phage integrase	1	-	-	-	-	-	-	-	-	-	-	-	-	-	-	-	-	-	-	-	-	-	-	-	-	-	-	-	-	-	-	-	-	-	-	-	-	-	+	-	-	-	-	-	-	-	-	-	-	-	-	-	-	-
5493	10858	hypothetical protein	1	-	-	-	-	-	-	-	-	-	-	-	-	-	-	-	-	-	-	-	-	-	-	-	-	-	-	-	-	-	-	-	-	-	-	-	-	-	+	-	-	-	-	-	-	-	-	-	-	-	-	-	-	-
5494	10859	hypothetical protein	1	-	-	-	-	-	-	-	-	-	-	-	-	-	-	-	-	-	-	-	-	-	-	-	-	-	-	-	-	-	-	-	-	-	-	-	-	-	+	-	-	-	-	-	-	-	-	-	-	-	-	-	-	-
5495	10860	hypothetical protein	1	-	-	-	-	-	-	-	-	-	-	-	-	-	-	-	-	-	-	-	-	-	-	-	-	-	-	-	-	-	-	-	-	-	-	-	-	-	+	-	-	-	-	-	-	-	-	-	-	-	-	-	-	-
5496	10861	hypothetical protein	1	-	-	-	-	-	-	-	-	-	-	-	-	-	-	-	-	-	-	-	-	-	-	-	-	-	-	-	-	-	-	-	-	-	-	-	-	-	+	-	-	-	-	-	-	-	-	-	-	-	-	-	-	-
5497	10862	hypothetical protein	1	-	-	-	-	-	-	-	-	-	-	-	-	-	-	-	-	-	-	-	-	-	-	-	-	-	-	-	-	-	-	-	-	-	-	-	-	-	+	-	-	-	-	-	-	-	-	-	-	-	-	-	-	-
5498	10863	hypothetical protein	1	-	-	-	-	-	-	-	-	-	-	-	-	-	-	-	-	-	-	-	-	-	-	-	-	-	-	-	-	-	-	-	-	-	-	-	-	-	+	-	-	-	-	-	-	-	-	-	-	-	-	-	-	-
5499	10864	Tn5252	1	-	-	-	-	-	-	-	-	-	-	-	-	-	-	-	-	-	-	-	-	-	-	-	-	-	-	-	-	-	-	-	-	-	-	-	-	-	+	-	-	-	-	-	-	-	-	-	-	-	-	-	-	-
5500	10865	Eps11J	1	-	-	-	-	-	-	-	-	-	-	-	-	-	-	-	-	-	-	-	-	-	-	-	-	-	-	-	-	-	-	-	-	-	-	-	-	-	+	-	-	-	-	-	-	-	-	-	-	-	-	-	-	-
5501	10866	hypothetical protein	1	-	-	-	-	-	-	-	-	-	-	-	-	-	-	-	-	-	-	-	-	-	-	-	-	-	-	-	-	-	-	-	-	-	-	-	-	-	+	-	-	-	-	-	-	-	-	-	-	-	-	-	-	-
5502	10867	hypothetical protein	1	-	-	-	-	-	-	-	-	-	-	-	-	-	-	-	-	-	-	-	-	-	-	-	-	-	-	-	-	-	-	-	-	-	-	-	-	-	+	-	-	-	-	-	-	-	-	-	-	-	-	-	-	-
5503	10868	hypothetical protein	1	-	-	-	-	-	-	-	-	-	-	-	-	-	-	-	-	-	-	-	-	-	-	-	-	-	-	-	-	-	-	-	-	-	-	-	-	-	+	-	-	-	-	-	-	-	-	-	-	-	-	-	-	-
5504	10869	4-carboxymuconolactone decarboxylase (EC 4.1.1.44)	1	-	-	-	-	-	-	-	-	-	-	-	-	-	-	-	-	-	-	-	-	-	-	-	-	-	-	-	-	-	-	-	-	-	-	-	-	-	+	-	-	-	-	-	-	-	-	-	-	-	-	-	-	-
5505	10870	hypothetical protein	1	-	-	-	-	-	-	-	-	-	-	-	-	-	-	-	-	-	-	-	-	-	-	-	-	-	-	-	-	-	-	-	-	-	-	-	-	-	+	-	-	-	-	-	-	-	-	-	-	-	-	-	-	-
5506	10871	hypothetical protein	1	-	-	-	-	-	-	-	-	-	-	-	-	-	-	-	-	-	-	-	-	-	-	-	-	-	-	-	-	-	-	-	-	-	-	-	-	-	+	-	-	-	-	-	-	-	-	-	-	-	-	-	-	-
5507	10872	hypothetical protein	1	-	-	-	-	-	-	-	-	-	-	-	-	-	-	-	-	-	-	-	-	-	-	-	-	-	-	-	-	-	-	-	-	-	-	-	-	-	+	-	-	-	-	-	-	-	-	-	-	-	-	-	-	-
5508	10873	FIG131328: Predicted ATP-dependent endonuclease of the OLD family	1	-	-	-	-	-	-	-	-	-	-	-	-	-	-	-	-	-	-	-	-	-	-	-	-	-	-	-	-	-	-	-	-	-	-	-	-	-	+	-	-	-	-	-	-	-	-	-	-	-	-	-	-	-
5509	10874	hypothetical protein	1	-	-	-	-	-	-	-	-	-	-	-	-	-	-	-	-	-	-	-	-	-	-	-	-	-	-	-	-	-	-	-	-	-	-	-	-	-	+	-	-	-	-	-	-	-	-	-	-	-	-	-	-	-
5510	10875	3-ketoacyl-CoA thiolase (EC 2.3.1.16) @ Acetyl-CoA acetyltransferase (EC 2.3.1.9)	1	-	-	-	-	-	-	-	-	-	-	-	-	-	-	-	-	-	-	-	-	-	-	-	-	-	-	-	-	-	-	-	-	-	-	-	-	-	+	-	-	-	-	-	-	-	-	-	-	-	-	-	-	-
5511	10876	Glycosyltransferase	1	-	-	-	-	-	-	-	-	-	-	-	-	-	-	-	-	-	-	-	-	-	-	-	-	-	-	-	-	-	-	-	-	-	-	-	-	-	+	-	-	-	-	-	-	-	-	-	-	-	-	-	-	-
5512	10877	hypothetical protein	1	-	-	-	-	-	-	-	-	-	-	-	-	-	-	-	-	-	-	-	-	-	-	-	-	-	-	-	-	-	-	-	-	-	-	-	-	-	+	-	-	-	-	-	-	-	-	-	-	-	-	-	-	-
5513	10878	hypothetical protein	1	-	-	-	-	-	-	-	-	-	-	-	-	-	-	-	-	-	-	-	-	-	-	-	-	-	-	-	-	-	-	-	-	-	-	-	-	-	+	-	-	-	-	-	-	-	-	-	-	-	-	-	-	-
5514	10879	hypothetical protein	1	-	-	-	-	-	-	-	-	-	-	-	-	-	-	-	-	-	-	-	-	-	-	-	-	-	-	-	-	-	-	-	-	-	-	-	-	-	+	-	-	-	-	-	-	-	-	-	-	-	-	-	-	-
5515	10880	hypothetical protein	1	-	-	-	-	-	-	-	-	-	-	-	-	-	-	-	-	-	-	-	-	-	-	-	-	-	-	-	-	-	-	-	-	-	-	-	-	-	+	-	-	-	-	-	-	-	-	-	-	-	-	-	-	-
5516	10881	hypothetical protein	1	-	-	-	-	-	-	-	-	-	-	-	-	-	-	-	-	-	-	-	-	-	-	-	-	-	-	-	-	-	-	-	-	-	-	-	-	-	+	-	-	-	-	-	-	-	-	-	-	-	-	-	-	-
5517	10882	hypothetical protein	1	-	-	-	-	-	-	-	-	-	-	-	-	-	-	-	-	-	-	-	-	-	-	-	-	-	-	-	-	-	-	-	-	-	-	-	-	-	+	-	-	-	-	-	-	-	-	-	-	-	-	-	-	-
5518	10883	hypothetical protein	1	-	-	-	-	-	-	-	-	-	-	-	-	-	-	-	-	-	-	-	-	-	-	-	-	-	-	-	-	-	-	-	-	-	-	-	-	-	+	-	-	-	-	-	-	-	-	-	-	-	-	-	-	-
5519	10884	hypothetical protein	1	-	-	-	-	-	-	-	-	-	-	-	-	-	-	-	-	-	-	-	-	-	-	-	-	-	-	-	-	-	-	-	-	-	-	-	-	-	+	-	-	-	-	-	-	-	-	-	-	-	-	-	-	-
5520	10885	hypothetical protein	1	-	-	-	-	-	-	-	-	-	-	-	-	-	-	-	-	-	-	-	-	-	-	-	-	-	-	-	-	-	-	-	-	-	-	-	-	-	+	-	-	-	-	-	-	-	-	-	-	-	-	-	-	-
5521	10886	hypothetical protein	1	-	-	-	-	-	-	-	-	-	-	-	-	-	-	-	-	-	-	-	-	-	-	-	-	-	-	-	-	-	-	-	-	-	-	-	-	-	+	-	-	-	-	-	-	-	-	-	-	-	-	-	-	-
5522	10887	hypothetical protein	1	-	-	-	-	-	-	-	-	-	-	-	-	-	-	-	-	-	-	-	-	-	-	-	-	-	-	-	-	-	-	-	-	-	-	-	-	-	+	-	-	-	-	-	-	-	-	-	-	-	-	-	-	-
5523	10888	hypothetical protein	1	-	-	-	-	-	-	-	-	-	-	-	-	-	-	-	-	-	-	-	-	-	-	-	-	-	-	-	-	-	-	-	-	-	-	-	-	-	+	-	-	-	-	-	-	-	-	-	-	-	-	-	-	-
5524	10889	Predicted membrane protein	1	-	-	-	-	-	-	-	-	-	-	-	-	-	-	-	-	-	-	-	-	-	-	-	-	-	-	-	-	-	-	-	-	-	-	-	-	-	+	-	-	-	-	-	-	-	-	-	-	-	-	-	-	-
5525	10890	hypothetical protein	1	-	-	-	-	-	-	-	-	-	-	-	-	-	-	-	-	-	-	-	-	-	-	-	-	-	-	-	-	-	-	-	-	-	-	-	-	-	+	-	-	-	-	-	-	-	-	-	-	-	-	-	-	-
5526	10891	5'-nucleotidase (EC 3.1.3.5)	1	-	-	-	-	-	-	-	-	-	-	-	-	-	-	-	-	-	-	-	-	-	-	-	-	-	-	-	-	-	-	-	-	-	-	-	-	-	+	-	-	-	-	-	-	-	-	-	-	-	-	-	-	-
5527	10892	hypothetical protein	1	-	-	-	-	-	-	-	-	-	-	-	-	-	-	-	-	-	-	-	-	-	-	-	-	-	-	-	-	-	-	-	-	-	-	-	-	-	+	-	-	-	-	-	-	-	-	-	-	-	-	-	-	-
5528	10893	hypothetical protein	1	-	-	-	-	-	-	-	-	-	-	-	-	-	-	-	-	-	-	-	-	-	-	-	-	-	-	-	-	-	-	-	-	-	-	-	-	-	+	-	-	-	-	-	-	-	-	-	-	-	-	-	-	-
5529	10894	hypothetical protein	1	-	-	-	-	-	-	-	-	-	-	-	-	-	-	-	-	-	-	-	-	-	-	-	-	-	-	-	-	-	-	-	-	-	-	-	-	-	+	-	-	-	-	-	-	-	-	-	-	-	-	-	-	-
5530	10895	hypothetical protein	1	-	-	-	-	-	-	-	-	-	-	-	-	-	-	-	-	-	-	-	-	-	-	-	-	-	-	-	-	-	-	-	-	-	-	-	-	+	-	-	-	-	-	-	-	-	-	-	-	-	-	-	-	-
5531	10896	hypothetical protein	1	-	-	-	-	-	-	-	-	-	-	-	-	-	-	-	-	-	-	-	-	-	-	-	-	-	-	-	-	-	-	-	-	-	-	-	-	+	-	-	-	-	-	-	-	-	-	-	-	-	-	-	-	-
5532	10897	hypothetical protein	1	-	-	-	-	-	-	-	-	-	-	-	-	-	-	-	-	-	-	-	-	-	-	-	-	-	-	-	-	-	-	-	-	-	-	-	-	+	-	-	-	-	-	-	-	-	-	-	-	-	-	-	-	-
5533	10898	hypothetical protein	1	-	-	-	-	-	-	-	-	-	-	-	-	-	-	-	-	-	-	-	-	-	-	-	-	-	-	-	-	-	-	-	-	-	-	-	-	+	-	-	-	-	-	-	-	-	-	-	-	-	-	-	-	-
5534	10899	hypothetical protein	1	-	-	-	-	-	-	-	-	-	-	-	-	-	-	-	-	-	-	-	-	-	-	-	-	-	-	-	-	-	-	-	-	-	-	-	-	+	-	-	-	-	-	-	-	-	-	-	-	-	-	-	-	-
5535	10900	replication initiator protein	1	-	-	-	-	-	-	-	-	-	-	-	-	-	-	-	-	-	-	-	-	-	-	-	-	-	-	-	-	-	-	-	-	-	-	-	-	+	-	-	-	-	-	-	-	-	-	-	-	-	-	-	-	-
5536	10901	hypothetical protein	1	-	-	-	-	-	-	-	-	-	-	-	-	-	-	-	-	-	-	-	-	-	-	-	-	-	-	-	-	-	-	-	-	-	-	-	-	+	-	-	-	-	-	-	-	-	-	-	-	-	-	-	-	-
5537	10902	Threonyl-tRNA synthetase (EC 6.1.1.3)	1	-	-	-	-	-	-	-	-	-	-	-	-	-	-	-	-	-	-	-	-	-	-	-	-	-	-	-	-	-	-	-	-	-	-	-	-	+	-	-	-	-	-	-	-	-	-	-	-	-	-	-	-	-
5538	10903	hypothetical protein	1	-	-	-	-	-	-	-	-	-	-	-	-	-	-	-	-	-	-	-	-	-	-	-	-	-	-	-	-	-	-	-	-	-	-	-	-	+	-	-	-	-	-	-	-	-	-	-	-	-	-	-	-	-
5539	10904	Mobile element protein	1	-	-	-	-	-	-	-	-	-	-	-	-	-	-	-	-	-	-	-	-	-	-	-	-	-	-	-	-	-	-	-	-	-	-	-	-	+	-	-	-	-	-	-	-	-	-	-	-	-	-	-	-	-
5540	10905	hypothetical protein	1	-	-	-	-	-	-	-	-	-	-	-	-	-	-	-	-	-	-	-	-	-	-	-	-	-	-	-	-	-	-	-	-	-	-	-	-	+	-	-	-	-	-	-	-	-	-	-	-	-	-	-	-	-
5541	10906	hypothetical protein	1	-	-	-	-	-	-	-	-	-	-	-	-	-	-	-	-	-	-	-	-	-	-	-	-	-	-	-	-	-	-	-	-	-	-	-	-	+	-	-	-	-	-	-	-	-	-	-	-	-	-	-	-	-
5542	10907	hypothetical protein	1	-	-	-	-	-	-	-	-	-	-	-	-	-	-	-	-	-	-	-	-	-	-	-	-	-	-	-	-	-	-	-	-	-	-	-	-	+	-	-	-	-	-	-	-	-	-	-	-	-	-	-	-	-
5543	10908	hypothetical protein	1	-	-	-	-	-	-	-	-	-	-	-	-	-	-	-	-	-	-	-	-	-	-	-	-	-	-	-	-	-	-	-	-	-	-	-	-	+	-	-	-	-	-	-	-	-	-	-	-	-	-	-	-	-
5544	10909	Rhs family protein	1	-	-	-	-	-	-	-	-	-	-	-	-	-	-	-	-	-	-	-	-	-	-	-	-	-	-	-	-	-	-	-	-	-	-	-	-	+	-	-	-	-	-	-	-	-	-	-	-	-	-	-	-	-
5545	10910	hypothetical protein	1	-	-	-	-	-	-	-	-	-	-	-	-	-	-	-	-	-	-	-	-	-	-	-	-	-	-	-	-	-	-	-	-	-	-	-	-	+	-	-	-	-	-	-	-	-	-	-	-	-	-	-	-	-
5546	10911	hypothetical protein	1	-	-	-	-	-	-	-	-	-	-	-	-	-	-	-	-	-	-	-	-	-	-	-	-	-	-	-	-	-	-	-	-	-	-	-	-	+	-	-	-	-	-	-	-	-	-	-	-	-	-	-	-	-
5547	10912	hypothetical protein	1	-	-	-	-	-	-	-	-	-	-	-	-	-	-	-	-	-	-	-	-	-	-	-	-	-	-	-	-	-	-	-	-	-	-	-	-	+	-	-	-	-	-	-	-	-	-	-	-	-	-	-	-	-
5548	10913	hypothetical protein	1	-	-	-	-	-	-	-	-	-	-	-	-	-	-	-	-	-	-	-	-	-	-	-	-	-	-	-	-	-	-	-	-	-	-	-	-	+	-	-	-	-	-	-	-	-	-	-	-	-	-	-	-	-
5549	10914	hypothetical protein	1	-	-	-	-	-	-	-	-	-	-	-	-	-	-	-	-	-	-	-	-	-	-	-	-	-	-	-	-	-	-	-	-	-	-	-	-	+	-	-	-	-	-	-	-	-	-	-	-	-	-	-	-	-
5550	10915	hypothetical protein	1	-	-	-	-	-	-	-	-	-	-	-	-	-	-	-	-	-	-	-	-	-	-	-	-	-	-	-	-	-	-	-	-	-	-	-	-	+	-	-	-	-	-	-	-	-	-	-	-	-	-	-	-	-
5551	10916	Prophage ps3 protein 07	1	-	-	-	-	-	-	-	-	-	-	-	-	-	-	-	-	-	-	-	-	-	-	-	-	-	-	-	-	-	-	-	-	-	-	-	-	+	-	-	-	-	-	-	-	-	-	-	-	-	-	-	-	-
5552	10917	hypothetical protein	1	-	-	-	-	-	-	-	-	-	-	-	-	-	-	-	-	-	-	-	-	-	-	-	-	-	-	-	-	-	-	-	-	-	-	-	-	+	-	-	-	-	-	-	-	-	-	-	-	-	-	-	-	-
5553	10918	hypothetical protein	1	-	-	-	-	-	-	-	-	-	-	-	-	-	-	-	-	-	-	-	-	-	-	-	-	-	-	-	-	-	-	-	-	-	-	-	-	+	-	-	-	-	-	-	-	-	-	-	-	-	-	-	-	-
5554	10919	FtsK/SpoIIIE family	1	-	-	-	-	-	-	-	-	-	-	-	-	-	-	-	-	-	-	-	-	-	-	-	-	-	-	-	-	-	-	-	-	-	-	-	-	+	-	-	-	-	-	-	-	-	-	-	-	-	-	-	-	-
5555	10920	cytosolic protein	1	-	-	-	-	-	-	-	-	-	-	-	-	-	-	-	-	-	-	-	-	-	-	-	-	-	-	-	-	-	-	-	-	-	-	-	-	+	-	-	-	-	-	-	-	-	-	-	-	-	-	-	-	-
5556	10921	hypothetical protein	1	-	-	-	-	-	-	-	-	-	-	-	-	-	-	-	-	-	-	-	-	-	-	-	-	-	-	-	-	-	-	-	-	-	-	-	-	+	-	-	-	-	-	-	-	-	-	-	-	-	-	-	-	-
5557	10922	hypothetical protein	1	-	-	-	-	-	-	-	-	-	-	-	-	-	-	-	-	-	-	-	-	-	-	-	-	-	-	-	-	-	-	-	-	-	-	-	-	+	-	-	-	-	-	-	-	-	-	-	-	-	-	-	-	-
5558	10923	Replication protein	1	-	-	-	-	-	-	-	-	-	-	-	-	-	-	-	-	-	-	-	-	-	-	-	-	-	-	-	-	-	-	-	-	-	-	-	-	+	-	-	-	-	-	-	-	-	-	-	-	-	-	-	-	-
5559	10924	hypothetical protein	1	-	-	-	-	-	-	-	-	-	-	-	-	-	-	-	-	-	-	-	-	-	-	-	-	-	-	-	-	-	-	-	-	-	-	-	-	+	-	-	-	-	-	-	-	-	-	-	-	-	-	-	-	-
5560	10925	hypothetical protein	1	-	-	-	-	-	-	-	-	-	-	-	-	-	-	-	-	-	-	-	-	-	-	-	-	-	-	-	-	-	-	-	-	-	-	-	-	+	-	-	-	-	-	-	-	-	-	-	-	-	-	-	-	-
5561	10926	hypothetical protein	1	-	-	-	-	-	-	-	-	-	-	-	-	-	-	-	-	-	-	-	-	-	-	-	-	-	-	-	-	-	-	-	-	-	-	-	-	+	-	-	-	-	-	-	-	-	-	-	-	-	-	-	-	-
5562	10927	hypothetical protein	1	-	-	-	-	-	-	-	-	-	-	-	-	-	-	-	-	-	-	-	-	-	-	-	-	-	-	-	-	-	-	-	-	-	-	-	-	+	-	-	-	-	-	-	-	-	-	-	-	-	-	-	-	-
5563	10928	hypothetical protein	1	-	-	-	-	-	-	-	-	-	-	-	-	-	-	-	-	-	-	-	-	-	-	-	-	-	-	-	-	-	-	-	-	-	-	-	-	+	-	-	-	-	-	-	-	-	-	-	-	-	-	-	-	-
5564	10929	hypothetical protein	1	-	-	-	-	-	-	-	-	-	-	-	-	-	-	-	-	-	-	-	-	-	-	-	-	-	-	-	-	-	-	-	-	-	-	-	-	+	-	-	-	-	-	-	-	-	-	-	-	-	-	-	-	-
5565	10930	hypothetical protein	1	-	-	-	-	-	-	-	-	-	-	-	-	-	-	-	-	-	-	-	-	-	-	-	-	-	-	-	-	-	-	-	-	-	-	-	-	+	-	-	-	-	-	-	-	-	-	-	-	-	-	-	-	-
5566	10931	collagen adhesin precursor	1	-	-	-	-	-	-	-	-	-	-	-	-	-	-	-	-	-	-	-	-	-	-	-	-	-	-	-	-	-	-	-	-	-	-	-	-	+	-	-	-	-	-	-	-	-	-	-	-	-	-	-	-	-
5567	10932	hypothetical protein	1	-	-	-	-	-	-	-	-	-	-	-	-	-	-	-	-	-	-	-	-	-	-	-	-	-	-	-	-	-	-	-	-	-	-	-	-	+	-	-	-	-	-	-	-	-	-	-	-	-	-	-	-	-
5568	10933	hypothetical protein	1	-	-	-	-	-	-	-	-	-	-	-	-	-	-	-	-	-	-	-	-	-	-	-	-	-	-	-	-	-	-	-	-	-	-	-	-	+	-	-	-	-	-	-	-	-	-	-	-	-	-	-	-	-
5569	10934	cAMP-binding proteins - catabolite gene activator and regulatory subunit of cAMP-dependent protein kinases	1	-	-	-	-	-	-	-	-	-	-	-	-	-	-	-	-	-	-	-	-	-	-	-	-	-	-	-	-	-	-	-	-	-	-	-	-	+	-	-	-	-	-	-	-	-	-	-	-	-	-	-	-	-
5570	10935	hypothetical protein	1	-	-	-	-	-	-	-	-	-	-	-	-	-	-	-	-	-	-	-	-	-	-	-	-	-	-	-	-	-	-	-	-	-	-	-	-	+	-	-	-	-	-	-	-	-	-	-	-	-	-	-	-	-
5571	10936	hypothetical protein	1	-	-	-	-	-	-	-	-	-	-	-	-	-	-	-	-	-	-	-	-	-	-	-	-	-	-	-	-	-	-	-	-	-	-	-	-	+	-	-	-	-	-	-	-	-	-	-	-	-	-	-	-	-
5572	10937	Rhs family protein	1	-	-	-	-	-	-	-	-	-	-	-	-	-	-	-	-	-	-	-	-	-	-	-	-	-	-	-	-	-	-	-	-	-	-	-	-	+	-	-	-	-	-	-	-	-	-	-	-	-	-	-	-	-
5573	10938	hypothetical protein	1	-	-	-	-	-	-	-	-	-	-	-	-	-	-	-	-	-	-	-	-	-	-	-	-	-	-	-	-	-	-	-	-	-	-	-	-	+	-	-	-	-	-	-	-	-	-	-	-	-	-	-	-	-
5574	10939	hypothetical protein	1	-	-	-	-	-	-	-	-	-	-	-	-	-	-	-	-	-	-	-	-	-	-	-	-	-	-	-	-	-	-	-	-	-	-	-	-	+	-	-	-	-	-	-	-	-	-	-	-	-	-	-	-	-
5575	10940	hypothetical protein	1	-	-	-	-	-	-	-	-	-	-	-	-	-	-	-	-	-	-	-	-	-	-	-	-	-	-	-	-	-	-	-	-	-	-	-	-	+	-	-	-	-	-	-	-	-	-	-	-	-	-	-	-	-
5576	10941	hypothetical protein	1	-	-	-	-	-	-	-	-	-	-	-	-	-	-	-	-	-	-	-	-	-	-	-	-	-	-	-	-	-	-	-	-	-	-	-	-	+	-	-	-	-	-	-	-	-	-	-	-	-	-	-	-	-
5577	10942	hypothetical protein	1	-	-	-	-	-	-	-	-	-	-	-	-	-	-	-	-	-	-	-	-	-	-	-	-	-	-	-	-	-	-	-	-	-	-	-	-	+	-	-	-	-	-	-	-	-	-	-	-	-	-	-	-	-
5578	10943	hypothetical protein	1	-	-	-	-	-	-	-	-	-	-	-	-	-	-	-	-	-	-	-	-	-	-	-	-	-	-	-	-	-	-	-	-	-	-	-	-	+	-	-	-	-	-	-	-	-	-	-	-	-	-	-	-	-
5579	10944	hypothetical protein	1	-	-	-	-	-	-	-	-	-	-	-	-	-	-	-	-	-	-	-	-	-	-	-	-	-	-	-	-	-	-	-	-	-	-	-	-	+	-	-	-	-	-	-	-	-	-	-	-	-	-	-	-	-
5580	10945	hypothetical protein	1	-	-	-	-	-	-	-	-	-	-	-	-	-	-	-	-	-	-	-	-	-	-	-	-	-	-	-	-	-	-	-	-	-	-	-	-	+	-	-	-	-	-	-	-	-	-	-	-	-	-	-	-	-
5581	10946	hypothetical protein	1	-	-	-	-	-	-	-	-	-	-	-	-	-	-	-	-	-	-	-	-	-	-	-	-	-	-	-	-	-	-	-	-	-	-	-	-	+	-	-	-	-	-	-	-	-	-	-	-	-	-	-	-	-
5582	10947	hypothetical protein	1	-	-	-	-	-	-	-	-	-	-	-	-	-	-	-	-	-	-	-	-	-	-	-	-	-	-	-	-	-	-	-	-	-	-	-	-	+	-	-	-	-	-	-	-	-	-	-	-	-	-	-	-	-
5583	10948	hypothetical protein	1	-	-	-	-	-	-	-	-	-	-	-	-	-	-	-	-	-	-	-	-	-	-	-	-	-	-	-	-	-	-	-	-	-	-	-	-	+	-	-	-	-	-	-	-	-	-	-	-	-	-	-	-	-
5584	10949	hypothetical protein	1	-	-	-	-	-	-	-	-	-	-	-	-	-	-	-	-	-	-	-	-	-	-	-	-	-	-	-	-	-	-	-	-	-	-	-	-	+	-	-	-	-	-	-	-	-	-	-	-	-	-	-	-	-
5585	10950	hypothetical protein	1	-	-	-	-	-	-	-	-	-	-	-	-	-	-	-	-	-	-	-	-	-	-	-	-	-	-	-	-	-	-	-	-	-	-	-	-	+	-	-	-	-	-	-	-	-	-	-	-	-	-	-	-	-
5586	10951	Type I restriction-modification system	1	-	-	-	-	-	-	-	-	-	-	-	-	-	-	-	-	-	-	-	-	-	-	-	-	-	-	-	-	-	-	-	-	-	-	-	-	+	-	-	-	-	-	-	-	-	-	-	-	-	-	-	-	-
5587	10952	ATPase component of ABC transporters with duplicated ATPase domains	1	-	-	-	-	-	-	-	-	-	-	-	-	-	-	-	-	-	-	-	-	-	-	-	-	-	-	-	-	-	-	-	-	-	-	-	-	+	-	-	-	-	-	-	-	-	-	-	-	-	-	-	-	-
5588	10953	hypothetical protein	1	-	-	-	-	-	-	-	-	-	-	-	-	-	-	-	-	-	-	-	-	-	-	-	-	-	-	-	-	-	-	-	-	-	-	-	-	+	-	-	-	-	-	-	-	-	-	-	-	-	-	-	-	-
5589	10954	hypothetical protein	1	-	-	-	-	-	-	-	-	-	-	-	-	-	-	-	-	-	-	-	-	-	-	-	-	-	-	-	-	-	-	-	-	-	-	-	-	+	-	-	-	-	-	-	-	-	-	-	-	-	-	-	-	-
5590	10955	Putative NADPH-quinone reductase (modulator of drug activity B)	1	-	-	-	-	-	-	-	-	-	-	-	-	-	-	-	-	-	-	-	-	-	-	-	-	-	-	-	-	-	-	-	-	-	-	-	-	+	-	-	-	-	-	-	-	-	-	-	-	-	-	-	-	-
5591	10956	hypothetical protein	1	-	-	-	-	-	-	-	-	-	-	-	-	-	-	-	-	-	-	-	-	-	-	-	-	-	-	-	-	-	-	-	-	-	-	-	-	+	-	-	-	-	-	-	-	-	-	-	-	-	-	-	-	-
5592	10957	hypothetical protein	1	-	-	-	-	-	-	-	-	-	-	-	-	-	-	-	-	-	-	-	-	-	-	-	-	-	-	-	-	-	-	-	-	-	-	-	-	+	-	-	-	-	-	-	-	-	-	-	-	-	-	-	-	-
5593	10958	hypothetical protein	1	-	-	-	-	-	-	-	-	-	-	-	-	-	-	-	-	-	-	-	-	-	-	-	-	-	-	-	-	-	-	-	-	-	-	-	-	-	-	-	-	-	-	-	-	-	+	-	-	-	-	-	-	-
5594	10959	N-Acetylneuraminate cytidylyltransferase (EC 2.7.7.43)	1	-	-	-	-	-	-	-	-	-	-	-	-	-	-	-	-	-	-	-	-	-	-	-	-	-	-	-	-	-	-	-	-	-	-	-	-	-	-	-	-	-	-	-	-	-	+	-	-	-	-	-	-	-
5595	10960	hypothetical protein	1	-	-	-	-	-	-	-	-	-	-	-	-	-	-	-	-	-	-	-	-	-	-	-	-	-	-	-	-	-	-	-	-	-	-	-	-	-	-	-	-	-	-	-	-	-	+	-	-	-	-	-	-	-
5596	10961	ABC transporter ATP-binding protein - Na+ export	1	-	-	-	-	-	-	-	-	-	-	-	-	-	-	-	-	-	-	-	-	-	-	-	-	-	-	-	-	-	-	-	-	-	-	-	-	-	-	-	-	-	-	-	-	-	+	-	-	-	-	-	-	-
5597	10962	hypothetical protein	1	-	-	-	-	-	-	-	-	-	-	-	-	-	-	-	-	-	-	-	-	-	-	-	-	-	-	-	-	-	-	-	-	-	-	-	-	-	-	-	-	-	-	-	-	-	+	-	-	-	-	-	-	-
5598	10963	hypothetical protein	1	-	-	-	-	-	-	-	-	-	-	-	-	-	-	-	-	-	-	-	-	-	-	-	-	-	-	-	-	-	-	-	-	-	-	-	-	-	-	-	-	-	-	-	-	-	+	-	-	-	-	-	-	-
5599	10964	hypothetical protein	1	-	-	-	-	-	-	-	-	-	-	-	-	-	-	-	-	-	-	-	-	-	-	-	-	-	-	-	-	-	-	-	-	-	-	-	-	-	-	-	-	-	-	-	-	-	+	-	-	-	-	-	-	-
5600	10965	hypothetical protein	1	-	-	-	-	-	-	-	-	-	-	-	-	-	-	-	-	-	-	-	-	-	-	-	-	-	-	-	-	-	-	-	-	-	-	-	-	-	-	-	-	-	-	-	-	-	+	-	-	-	-	-	-	-
5601	10966	ABC transporter	1	-	-	-	-	-	-	-	-	-	-	-	-	-	-	-	-	-	-	-	-	-	-	-	-	-	-	-	-	-	-	-	-	-	-	-	-	-	-	-	-	-	-	-	-	-	+	-	-	-	-	-	-	-
5602	10967	hypothetical protein	1	-	-	-	-	-	-	-	-	-	-	-	-	-	-	-	-	-	-	-	-	-	-	-	-	-	-	-	-	-	-	-	-	-	-	-	-	-	-	-	-	-	-	-	-	-	+	-	-	-	-	-	-	-
5603	10968	Doc protein	1	-	-	-	-	-	-	-	-	-	-	-	-	-	-	-	-	-	-	-	-	-	-	-	-	-	-	-	-	-	-	-	-	-	-	-	-	-	-	-	-	-	-	-	-	-	+	-	-	-	-	-	-	-
5604	10969	hypothetical protein	1	-	-	-	-	-	-	-	-	-	-	-	-	-	-	-	-	-	-	-	-	-	-	-	-	-	-	-	-	-	-	-	-	-	-	-	-	-	-	-	-	-	-	-	-	-	+	-	-	-	-	-	-	-
5605	10970	hypothetical protein	1	-	-	-	-	-	-	-	-	-	-	-	-	-	-	-	-	-	-	-	-	-	-	-	-	-	-	-	-	-	-	-	-	-	-	-	-	-	-	-	-	-	-	-	-	-	+	-	-	-	-	-	-	-
5606	10971	NisK	1	-	-	-	-	-	-	-	-	-	-	-	-	-	-	-	-	-	-	-	-	-	-	-	-	-	-	-	-	-	-	-	-	-	-	-	-	-	-	-	-	-	-	-	-	-	+	-	-	-	-	-	-	-
5607	10972	hypothetical protein	1	-	-	-	-	-	-	-	-	-	-	-	-	-	-	-	-	-	-	-	-	-	-	-	-	-	-	-	-	-	-	-	-	-	-	-	-	-	-	-	-	-	-	-	-	-	+	-	-	-	-	-	-	-
5608	10973	PTS system	1	-	-	-	-	-	-	-	-	-	-	-	-	-	-	-	-	-	-	-	-	-	-	-	-	-	-	-	-	-	-	-	-	-	-	-	-	-	-	-	-	-	-	-	-	-	+	-	-	-	-	-	-	-
5609	10974	hypothetical protein	1	-	-	-	-	-	-	-	-	-	-	-	-	-	-	-	-	-	-	-	-	-	-	-	-	-	-	-	-	-	-	-	-	-	-	-	-	-	-	-	-	-	-	-	-	-	+	-	-	-	-	-	-	-
5610	10975	Site-specific recombinase	1	-	-	-	-	-	-	-	-	-	-	-	-	-	-	-	-	-	-	-	-	-	-	-	-	-	-	-	-	-	-	-	-	-	-	-	-	-	-	-	-	-	-	-	-	-	+	-	-	-	-	-	-	-
5611	10976	hypothetical protein	1	-	-	-	-	-	-	-	-	-	-	-	-	-	-	-	-	-	-	-	-	-	-	-	-	-	-	-	-	-	-	-	-	-	-	-	-	-	-	-	-	-	-	-	-	-	+	-	-	-	-	-	-	-
5612	10977	hypothetical protein	1	-	-	-	-	-	-	-	-	-	-	-	-	-	-	-	-	-	-	-	-	-	-	-	-	-	-	-	-	-	-	-	-	-	-	-	-	-	-	-	-	-	-	-	-	-	+	-	-	-	-	-	-	-
5613	10978	hypothetical protein	1	-	-	-	-	-	-	-	-	-	-	-	-	-	-	-	-	-	-	-	-	-	-	-	-	-	-	-	-	-	-	-	-	-	-	-	-	-	-	-	-	-	-	-	-	-	+	-	-	-	-	-	-	-
5614	10979	hypothetical protein	1	-	-	-	-	-	-	-	-	-	-	-	-	-	-	-	-	-	-	-	-	-	-	-	-	-	-	-	-	-	-	-	-	-	-	-	-	-	-	-	-	-	-	-	-	-	+	-	-	-	-	-	-	-
5615	10980	flippase Wzx	1	-	-	-	-	-	-	-	-	-	-	-	-	-	-	-	-	-	-	-	-	-	-	-	-	-	-	-	-	-	-	-	-	-	-	-	-	-	-	-	-	-	-	-	-	-	+	-	-	-	-	-	-	-
5616	10981	Exonuclease motif predicted by PFAM	1	-	-	-	-	-	-	-	-	-	-	-	-	-	-	-	-	-	-	-	-	-	-	-	-	-	-	-	-	-	-	-	-	-	-	-	-	-	-	-	-	-	-	-	-	-	+	-	-	-	-	-	-	-
5617	10982	hypothetical protein	1	-	-	-	-	-	-	-	-	-	-	-	-	-	-	-	-	-	-	-	-	-	-	-	-	-	-	-	-	-	-	-	-	-	-	-	-	-	-	-	-	-	-	-	-	-	+	-	-	-	-	-	-	-
5618	10983	hypothetical protein	1	-	-	-	-	-	-	-	-	-	-	-	-	-	-	-	-	-	-	-	-	-	-	-	-	-	-	-	-	-	-	-	-	-	-	-	-	-	-	-	-	-	-	-	-	-	+	-	-	-	-	-	-	-
5619	10984	hypothetical protein	1	-	-	-	-	-	-	-	-	-	-	-	-	-	-	-	-	-	-	-	-	-	-	-	-	-	-	-	-	-	-	-	-	-	-	-	-	-	-	-	-	-	-	-	-	-	+	-	-	-	-	-	-	-
5620	10985	hypothetical protein	1	-	-	-	-	-	-	-	-	-	-	-	-	-	-	-	-	-	-	-	-	-	-	-	-	-	-	-	-	-	-	-	-	-	-	-	-	-	-	-	-	-	-	-	-	-	+	-	-	-	-	-	-	-
5621	10986	hypothetical protein	1	-	-	-	-	-	-	-	-	-	-	-	-	-	-	-	-	-	-	-	-	-	-	-	-	-	-	-	-	-	-	-	-	-	-	-	-	-	-	-	-	-	-	-	-	-	+	-	-	-	-	-	-	-
5622	10987	Type I restriction-modification system	1	-	-	-	-	-	-	-	-	-	-	-	-	-	-	-	-	-	-	-	-	-	-	-	-	-	-	-	-	-	-	-	-	-	-	-	-	-	-	-	-	-	-	-	-	-	+	-	-	-	-	-	-	-
5623	10988	hypothetical protein	1	-	-	-	-	-	-	-	-	-	-	-	-	-	-	-	-	-	-	-	-	-	-	-	-	-	-	-	-	-	-	-	-	-	-	-	-	-	-	-	-	-	-	-	-	-	+	-	-	-	-	-	-	-
5624	10989	hypothetical protein	1	-	-	-	-	-	-	-	-	-	-	-	-	-	-	-	-	-	-	-	-	-	-	-	-	-	-	-	-	-	-	-	-	-	-	-	-	-	-	-	-	-	-	-	-	-	+	-	-	-	-	-	-	-
5625	10990	hypothetical protein	1	-	-	-	-	-	-	-	-	-	-	-	-	-	-	-	-	-	-	-	-	-	-	-	-	-	-	-	-	-	-	-	-	-	-	-	-	-	-	-	-	-	-	-	-	-	+	-	-	-	-	-	-	-
5626	10991	hypothetical protein	1	-	-	-	-	-	-	-	-	-	-	-	-	-	-	-	-	-	-	-	-	-	-	-	-	-	-	-	-	-	-	-	-	-	-	-	-	-	-	-	-	-	-	-	-	-	+	-	-	-	-	-	-	-
5627	10992	hypothetical protein	1	-	-	-	-	-	-	-	-	-	-	-	-	-	-	-	-	-	-	-	-	-	-	-	-	-	-	-	-	-	-	-	-	-	-	-	-	-	-	-	-	-	-	-	-	-	+	-	-	-	-	-	-	-
5628	10993	muramidase-released protein	1	-	-	-	-	-	-	-	-	-	-	-	-	-	-	-	-	-	-	-	-	-	-	-	-	-	-	-	-	-	-	-	-	-	-	-	-	-	-	-	-	-	-	-	-	-	+	-	-	-	-	-	-	-
5629	10994	hypothetical protein	1	-	-	-	-	-	-	-	-	-	-	-	-	-	-	-	-	-	-	-	-	-	-	-	-	-	-	-	-	-	-	-	-	-	-	-	-	-	-	-	-	-	-	-	-	-	+	-	-	-	-	-	-	-
5630	10995	hypothetical protein	1	-	-	-	-	-	-	-	-	-	-	-	-	-	-	-	-	-	-	-	-	-	-	-	-	-	-	-	-	-	-	-	-	-	-	-	-	-	-	-	-	-	-	-	-	-	+	-	-	-	-	-	-	-
5631	10996	hypothetical protein	1	-	-	-	-	-	-	-	-	-	-	-	-	-	-	-	-	-	-	-	-	-	-	-	-	-	-	-	-	-	-	-	-	-	-	-	-	-	-	-	-	-	-	-	-	-	+	-	-	-	-	-	-	-
5632	10997	Transcriptional antiterminator bglG	1	-	-	-	-	-	-	-	-	-	-	-	-	-	-	-	-	-	-	-	-	-	-	-	-	-	-	-	-	-	-	-	-	-	-	-	-	-	-	-	-	-	-	-	-	-	+	-	-	-	-	-	-	-
5633	10998	hypothetical protein	1	-	-	-	-	-	-	-	-	-	-	-	-	-	-	-	-	-	-	-	-	-	-	-	-	-	-	-	-	-	-	-	-	-	-	-	-	-	-	-	-	-	-	-	-	-	+	-	-	-	-	-	-	-
5634	10999	response regulator receiver domain protein (CheY-like)	1	-	-	-	-	-	-	-	-	-	-	-	-	-	-	-	-	-	-	-	-	-	-	-	-	-	-	-	-	-	-	-	-	-	-	-	-	-	-	-	-	-	-	-	-	-	+	-	-	-	-	-	-	-
5635	11000	hypothetical protein	1	-	-	-	-	-	-	-	-	-	-	-	-	-	-	-	-	-	-	-	-	-	-	-	-	-	-	-	-	-	-	-	-	-	-	-	-	-	-	-	-	-	-	-	-	-	+	-	-	-	-	-	-	-
5636	11001	hypothetical protein	1	-	-	-	-	-	-	-	-	-	-	-	-	-	-	-	-	-	-	-	-	-	-	-	-	-	-	-	-	-	-	-	-	-	-	-	-	-	-	-	-	-	-	-	-	-	+	-	-	-	-	-	-	-
5637	11002	hypothetical protein	1	-	-	-	-	-	-	-	-	-	-	-	-	-	-	-	-	-	-	-	-	-	-	-	-	-	-	-	-	-	-	-	-	-	-	-	-	-	-	-	-	-	-	-	-	-	+	-	-	-	-	-	-	-
5638	11003	hypothetical protein	1	-	-	-	-	-	-	-	-	-	-	-	-	-	-	-	-	-	-	-	-	-	-	-	-	-	-	-	-	-	-	-	-	-	-	-	-	-	-	-	-	-	-	-	-	-	+	-	-	-	-	-	-	-
5639	11004	hypothetical protein	1	-	-	-	-	-	-	-	-	-	-	-	-	-	-	-	-	-	-	-	-	-	-	-	-	-	-	-	-	-	-	-	-	-	-	-	-	-	-	-	-	-	-	-	-	-	+	-	-	-	-	-	-	-
5640	11005	hypothetical protein	1	-	-	-	-	-	-	-	-	-	-	-	-	-	-	-	-	-	-	-	-	-	-	-	-	-	-	-	-	-	-	-	-	-	-	-	-	-	-	-	-	-	-	-	-	-	+	-	-	-	-	-	-	-
5641	11006	hypothetical protein	1	-	-	-	-	-	-	-	-	-	-	-	-	-	-	-	-	-	-	-	-	-	-	-	-	-	-	-	-	-	-	-	-	-	-	-	-	-	-	-	-	-	-	-	-	-	+	-	-	-	-	-	-	-
5642	11007	hypothetical protein	1	-	-	-	-	-	-	-	-	-	-	-	-	-	-	-	-	-	-	-	-	-	-	-	-	-	-	-	-	-	-	-	-	-	-	-	-	-	-	-	-	-	-	-	-	-	+	-	-	-	-	-	-	-
5643	11008	hypothetical protein	1	-	-	-	-	-	-	-	-	-	-	-	-	-	-	-	-	-	-	-	-	-	-	-	-	-	-	-	-	-	-	-	-	-	-	-	-	-	-	-	-	-	-	-	-	-	+	-	-	-	-	-	-	-
5644	11009	hypothetical protein	1	-	-	-	-	-	-	-	-	-	-	-	-	-	-	-	-	-	-	-	-	-	-	-	-	-	-	-	-	-	-	-	-	-	-	-	-	-	-	-	-	-	-	-	-	-	+	-	-	-	-	-	-	-
5645	11010	hypothetical protein	1	-	-	-	-	-	-	-	-	-	-	-	-	-	-	-	-	-	-	-	-	-	-	-	-	-	-	-	-	-	-	-	-	-	-	-	-	-	-	-	-	-	-	-	-	-	+	-	-	-	-	-	-	-
5646	11011	hypothetical protein	1	-	-	-	-	-	-	-	-	-	-	-	-	-	-	-	-	-	-	-	-	-	-	-	-	-	-	-	-	-	-	-	-	-	-	-	-	-	-	-	-	-	-	-	-	-	+	-	-	-	-	-	-	-
5647	11012	Glutamyl-tRNA synthetase (EC 6.1.1.17) @ Glutamyl-tRNA(Gln) synthetase (EC 6.1.1.24)	1	-	-	-	-	-	-	-	-	-	-	-	-	-	-	-	-	-	-	-	-	-	-	-	-	-	-	-	-	-	-	-	-	-	-	-	-	-	-	-	-	-	-	-	-	-	+	-	-	-	-	-	-	-
5648	11013	hypothetical protein	1	-	-	-	-	-	-	-	-	-	-	-	-	-	-	-	-	-	-	-	-	-	-	-	-	-	-	-	-	-	-	-	-	-	-	-	-	-	-	-	-	-	-	-	-	-	+	-	-	-	-	-	-	-
5649	11014	hypothetical protein	1	-	-	-	-	-	-	-	-	-	-	-	-	-	-	-	-	-	-	-	-	-	-	-	-	-	-	-	-	-	-	-	-	-	-	-	-	-	-	-	-	-	-	-	-	-	+	-	-	-	-	-	-	-
5650	11015	hypothetical protein	1	-	-	-	-	-	-	-	-	-	-	-	-	-	-	-	-	-	-	-	-	-	-	-	-	-	-	-	-	-	-	-	-	-	-	-	-	-	-	-	-	-	-	-	-	-	+	-	-	-	-	-	-	-
5651	11016	hypothetical protein	1	-	-	-	-	-	-	-	-	-	-	-	-	-	-	-	-	-	-	-	-	-	-	-	-	-	-	-	-	-	-	-	-	-	-	-	-	-	-	-	-	-	-	-	-	-	+	-	-	-	-	-	-	-
5652	11017	acetyltransferase (isoleucine patch superfamily)	1	-	-	-	-	-	-	-	-	-	-	-	-	-	-	-	-	-	-	-	-	-	-	-	-	-	-	-	-	-	-	-	-	-	-	-	-	-	-	-	-	-	-	-	-	-	+	-	-	-	-	-	-	-
5653	11018	response regulator receiver domain protein (CheY-like)	1	-	-	-	-	-	-	-	-	-	-	-	-	-	-	-	-	-	-	-	-	-	-	-	-	-	-	-	-	-	-	-	-	-	-	-	-	-	-	-	-	-	-	-	-	-	+	-	-	-	-	-	-	-
5654	11019	toxin secretion ABC transporter	1	-	-	-	-	-	-	-	-	-	-	-	-	-	-	-	-	-	-	-	-	-	-	-	-	-	-	-	-	-	-	-	-	-	-	-	-	-	-	-	-	-	-	-	-	-	+	-	-	-	-	-	-	-
5655	11020	hypothetical protein	1	-	-	-	-	-	-	-	-	-	-	-	-	-	-	-	-	-	-	-	-	-	-	-	-	-	-	-	-	-	-	-	-	-	-	-	-	-	-	-	-	-	-	-	-	-	+	-	-	-	-	-	-	-
5656	11021	hypothetical protein	1	-	-	-	-	-	-	-	-	-	-	-	-	-	-	-	-	-	-	-	-	-	-	-	-	-	-	-	-	-	-	-	-	-	-	-	-	-	-	-	-	-	-	-	-	-	+	-	-	-	-	-	-	-
5657	11022	Cell-wall biogenesis glycosyltransferase	1	-	-	-	-	-	-	-	-	-	-	-	-	-	-	-	-	-	-	-	-	-	-	-	-	-	-	-	-	-	-	-	-	-	-	-	-	-	-	-	-	-	-	-	-	-	+	-	-	-	-	-	-	-
5658	11023	Type I restriction-modification system	1	-	-	-	-	-	-	-	-	-	-	-	-	-	-	-	-	-	-	-	-	-	-	-	-	-	-	-	-	-	-	-	-	-	-	-	-	-	-	-	-	-	-	-	-	-	+	-	-	-	-	-	-	-
5659	11024	hypothetical protein	1	-	-	-	-	-	-	-	-	-	-	-	-	-	-	-	-	-	-	-	-	-	-	-	-	-	-	-	-	-	-	-	-	-	-	-	-	-	-	-	-	-	-	-	-	-	+	-	-	-	-	-	-	-
5660	11025	hypothetical protein	1	-	-	-	-	-	-	-	-	-	-	-	-	-	-	-	-	-	-	-	-	-	-	-	-	-	-	-	-	-	-	-	-	-	-	-	-	-	-	-	-	-	-	-	-	-	+	-	-	-	-	-	-	-

Table S5. Precence/absence of each HG among 57 *S. suis* strains

				1																13			25					28						104		94			76			53/54		Divergent *S. suis*																
Index[a]	ClusterID	Gene products	Members	98HAH33	05ZYH33	SC84	A7	SS12	P1/7	DAT273	NIAH11434	JS14	GZ1	BM407	S735	13730	MNCM07	NCTC 10446	14636	NCTC 10237	ST1	2651	89-1591	MNCM04	MNCM25	8074	D9	4961	ST3	MNCM43	DAT292	DAT260	NIAH11435	MNCM21	MNCM50	89-2479	DAT234	DAT290	93A	42A	NT77	6407	11538	8830	12814	92-1400	89-590	2726	2524	89-3576-3	4417	D12	10581	89-5259	88-5299A	R61	92-1191	14A	92-4172	22083
5661	11026	hypothetical protein	1	-	-	-	-	-	-	-	-	-	-	-	-	-	-	-	-	-	-	-	-	-	-	-	-	-	-	-	-	-	-	-	-	-	-	-	-	-	-	-	-	-	-	-	-	-	+	-	-	-	-	-	-	-
5662	11027	hypothetical protein	1	-	-	-	-	-	-	-	-	-	-	-	-	-	-	-	-	-	-	-	-	-	-	-	-	-	-	-	-	-	-	-	-	-	-	-	-	-	-	-	-	-	-	-	-	-	+	-	-	-	-	-	-	-
5663	11028	PTS system	1	-	-	-	-	-	-	-	-	-	-	-	-	-	-	-	-	-	-	-	-	-	-	-	-	-	-	-	-	-	-	-	-	-	-	-	-	-	-	-	-	-	-	-	-	-	+	-	-	-	-	-	-	-
5664	11029	hypothetical protein	1	-	-	-	-	-	-	-	-	-	-	-	-	-	-	-	-	-	-	-	-	-	-	-	-	-	-	-	-	-	-	-	-	-	-	-	-	-	-	-	-	-	-	-	-	-	+	-	-	-	-	-	-	-
5665	11030	hypothetical protein	1	-	-	-	-	-	-	-	-	-	-	-	-	-	-	-	-	-	-	-	-	-	-	-	-	-	-	-	-	-	-	-	-	-	-	-	-	-	-	-	-	-	-	-	-	-	+	-	-	-	-	-	-	-
5666	11031	hypothetical protein	1	-	-	-	-	-	-	-	-	-	-	-	-	-	-	-	-	-	-	-	-	-	-	-	-	-	-	-	-	-	-	-	-	-	-	-	-	-	-	-	-	-	-	-	-	-	+	-	-	-	-	-	-	-
5667	11032	Site-specific recombinase	1	-	-	-	-	-	-	-	-	-	-	-	-	-	-	-	-	-	-	-	-	-	-	-	-	-	-	-	-	-	-	-	-	-	-	-	-	-	-	-	-	-	-	-	-	-	+	-	-	-	-	-	-	-
5668	11033	hypothetical protein	1	-	-	-	-	-	-	-	-	-	-	-	-	-	-	-	-	-	-	-	-	-	-	-	-	-	-	-	-	-	-	-	-	-	-	-	-	-	-	-	-	-	-	-	-	-	+	-	-	-	-	-	-	-
5669	11034	Site-specific recombinase	1	-	-	-	-	-	-	-	-	-	-	-	-	-	-	-	-	-	-	-	-	-	-	-	-	-	-	-	-	-	-	-	-	-	-	-	-	-	-	-	-	-	-	-	-	-	+	-	-	-	-	-	-	-
5670	11035	Predicted hydrolase (HAD superfamily)	1	-	-	-	-	-	-	-	-	-	-	-	-	-	-	-	-	-	-	-	-	-	-	-	-	-	-	-	-	-	-	-	-	-	-	-	-	-	-	-	-	-	-	-	-	-	+	-	-	-	-	-	-	-
5671	11036	COG0657: Esterase/lipase	1	-	-	-	-	-	-	-	-	-	-	-	-	-	-	-	-	-	-	-	-	-	-	-	-	-	-	-	-	-	-	-	-	-	-	-	-	-	-	-	-	-	-	-	-	-	+	-	-	-	-	-	-	-
5672	11037	Helix-turn-helix	1	-	-	-	-	-	-	-	-	-	-	-	-	-	-	-	-	-	-	-	-	-	-	-	-	-	-	-	-	-	-	-	-	-	-	-	-	-	-	-	-	-	-	-	-	-	+	-	-	-	-	-	-	-
5673	11038	hypothetical protein	1	-	-	-	-	-	-	-	-	-	-	-	-	-	-	-	-	-	-	-	-	-	-	-	-	-	-	-	-	-	-	-	-	-	-	-	-	-	-	-	-	-	-	-	-	-	+	-	-	-	-	-	-	-
5674	11039	Tn5252	1	-	-	-	-	-	-	-	-	-	-	-	-	-	-	-	-	-	-	-	-	-	-	-	-	-	-	-	-	-	-	-	-	-	-	-	-	-	-	-	-	-	-	-	-	-	+	-	-	-	-	-	-	-
5675	11040	hypothetical protein	1	-	-	-	-	-	-	-	-	-	-	-	-	-	-	-	-	-	-	-	-	-	-	-	-	-	-	-	-	-	-	-	-	-	-	-	-	-	-	-	-	-	-	-	-	-	+	-	-	-	-	-	-	-
5676	11041	putative serine/threonine protein kinase	1	-	-	-	-	-	-	-	-	-	-	-	-	-	-	-	-	-	-	-	-	-	-	-	-	-	-	-	-	-	-	-	-	-	-	-	-	-	-	-	-	-	-	-	-	-	+	-	-	-	-	-	-	-
5677	11042	hypothetical protein	1	-	-	-	-	-	-	-	-	-	-	-	-	-	-	-	-	-	-	-	-	-	-	-	-	-	-	-	-	-	-	-	-	-	-	-	-	-	-	-	-	-	-	-	-	-	+	-	-	-	-	-	-	-
5678	11043	hypothetical protein	1	-	-	-	-	-	-	-	-	-	-	-	-	-	-	-	-	-	-	-	-	-	-	-	-	-	-	-	-	-	-	-	-	-	-	-	-	-	-	-	-	-	-	-	-	-	+	-	-	-	-	-	-	-
5679	11044	hypothetical protein	1	-	-	-	-	-	-	-	-	-	-	-	-	-	-	-	-	-	-	-	-	-	-	-	-	-	-	-	-	-	-	-	-	-	-	-	-	-	-	-	-	-	-	-	-	-	+	-	-	-	-	-	-	-
5680	11045	hypothetical protein	1	-	-	-	-	-	-	-	-	-	-	-	-	-	-	-	-	-	-	-	-	-	-	-	-	-	-	-	-	-	-	-	-	-	-	-	-	-	-	-	-	-	-	-	-	-	+	-	-	-	-	-	-	-
5681	11046	hypothetical protein	1	-	-	-	-	-	-	-	-	-	-	-	-	-	-	-	-	-	-	-	-	-	-	-	-	-	-	-	-	-	-	-	-	-	-	-	-	-	-	-	-	-	-	-	-	-	+	-	-	-	-	-	-	-
5682	11047	hypothetical protein	1	-	-	-	-	-	-	-	-	-	-	-	-	-	-	-	-	-	-	-	-	-	-	-	-	-	-	-	-	-	-	-	-	-	-	-	-	-	-	-	-	-	-	-	-	-	+	-	-	-	-	-	-	-
5683	11048	UDP-N-acetylglucosamine 2-epimerase (EC 5.1.3.14)	1	-	-	-	-	-	-	-	-	-	-	-	-	-	-	-	-	-	-	-	-	-	-	-	-	-	-	-	-	-	-	-	-	-	-	-	-	-	-	-	-	-	-	-	-	-	+	-	-	-	-	-	-	-
5684	11049	ABC transporter ATP-binding protein	1	-	-	-	-	-	-	-	-	-	-	-	-	-	-	-	-	-	-	-	-	-	-	-	-	-	-	-	-	-	-	-	-	-	-	-	-	-	-	-	-	-	-	-	-	-	+	-	-	-	-	-	-	-
5685	11050	hypothetical protein	1	-	-	-	-	-	-	-	-	-	-	-	-	-	-	-	-	-	-	-	-	-	-	-	-	-	-	-	-	-	-	-	-	-	-	-	-	-	-	-	-	-	-	-	-	-	+	-	-	-	-	-	-	-
5686	11051	sepS13	1	-	-	-	-	-	-	-	-	-	-	-	-	-	-	-	-	-	-	-	-	-	-	-	-	-	-	-	-	-	-	-	-	-	-	-	-	-	-	-	-	-	-	-	-	-	+	-	-	-	-	-	-	-
5687	11052	hypothetical protein	1	-	-	-	-	-	-	-	-	-	-	-	-	-	-	-	-	-	-	-	-	-	-	-	-	-	-	-	-	-	-	-	-	-	-	-	-	-	-	-	-	-	-	-	-	-	+	-	-	-	-	-	-	-
5688	11053	hypothetical protein	1	-	-	-	-	-	-	-	-	-	-	-	-	-	-	-	-	-	-	-	-	-	-	-	-	-	-	-	-	-	-	-	-	-	-	-	-	-	-	-	-	-	-	-	-	-	+	-	-	-	-	-	-	-
5689	11054	hypothetical protein	1	-	-	-	-	-	-	-	-	-	-	-	-	-	-	-	-	-	-	-	-	-	-	-	-	-	-	-	-	-	-	-	-	-	-	-	-	-	-	-	-	-	-	-	-	-	+	-	-	-	-	-	-	-
5690	11055	Type I restriction-modification system	1	-	-	-	-	-	-	-	-	-	-	-	-	-	-	-	-	-	-	-	-	-	-	-	-	-	-	-	-	-	-	-	-	-	-	-	-	-	-	-	-	-	-	-	-	-	+	-	-	-	-	-	-	-
5691	11056	hypothetical protein	1	-	-	-	-	-	-	-	-	-	-	-	-	-	-	-	-	-	-	-	-	-	-	-	-	-	-	-	-	-	-	-	-	-	-	-	-	-	-	-	-	-	-	-	-	-	+	-	-	-	-	-	-	-
5692	11057	hypothetical protein	1	-	-	-	-	-	-	-	-	-	-	-	-	-	-	-	-	-	-	-	-	-	-	-	-	-	-	-	-	-	-	-	-	-	-	-	-	-	-	-	-	-	-	-	-	-	+	-	-	-	-	-	-	-
5693	11058	hypothetical protein	1	-	-	-	-	-	-	-	-	-	-	-	-	-	-	-	-	-	-	-	-	-	-	-	-	-	-	-	-	-	-	-	-	-	-	-	-	-	-	-	-	-	-	-	-	-	+	-	-	-	-	-	-	-
5694	11059	Phage protein	1	-	-	-	-	-	-	-	-	-	-	-	-	-	-	-	-	-	-	-	-	-	-	-	-	-	-	-	-	-	-	-	-	-	-	-	-	-	-	-	-	-	-	-	-	-	+	-	-	-	-	-	-	-
5695	11060	hypothetical protein	1	-	-	-	-	-	-	-	-	-	-	-	-	-	-	-	-	-	-	-	-	-	-	-	-	-	-	-	-	-	-	-	-	-	-	-	-	-	-	-	-	-	-	-	-	-	+	-	-	-	-	-	-	-
5696	11061	N-acetylneuraminate synthase(EC:4.1.3.19)	1	-	-	-	-	-	-	-	-	-	-	-	-	-	-	-	-	-	-	-	-	-	-	-	-	-	-	-	-	-	-	-	-	-	-	-	-	-	-	-	-	-	-	-	-	-	+	-	-	-	-	-	-	-
5697	11062	YpfJ protein	1	-	-	-	-	-	-	-	-	-	-	-	-	-	-	-	-	-	-	-	-	-	-	-	-	-	-	-	-	-	-	-	-	-	-	-	-	-	-	-	-	-	-	-	-	-	+	-	-	-	-	-	-	-
5698	11063	Site-specific recombinase	1	-	-	-	-	-	-	-	-	-	-	-	-	-	-	-	-	-	-	-	-	-	-	-	-	-	-	-	-	-	-	-	-	-	-	-	-	-	-	-	-	-	-	-	-	-	+	-	-	-	-	-	-	-
5699	11064	hypothetical protein	1	-	-	-	-	-	-	-	-	-	-	-	-	-	-	-	-	-	-	-	-	-	-	-	-	-	-	-	-	-	-	-	-	-	-	-	-	-	-	-	-	-	-	-	-	-	+	-	-	-	-	-	-	-
5700	11065	Lanthionine biosynthesis protein LanM	1	-	-	-	-	-	-	-	-	-	-	-	-	-	-	-	-	-	-	-	-	-	-	-	-	-	-	-	-	-	-	-	-	-	-	-	-	-	-	-	-	-	-	-	-	-	+	-	-	-	-	-	-	-
5701	11066	hypothetical protein	1	-	-	-	-	-	-	-	-	-	-	-	-	-	-	-	-	-	-	-	-	-	-	-	-	-	-	-	-	-	-	-	-	-	-	-	-	-	-	-	-	-	-	-	-	-	+	-	-	-	-	-	-	-
5702	11067	Phenylalanyl-tRNA synthetase beta chain (EC 6.1.1.20)	1	-	-	-	-	-	-	-	-	-	-	-	-	+	-	-	-	-	-	-	-	-	-	-	-	-	-	-	-	-	-	-	-	-	-	-	-	-	-	-	-	-	-	-	-	-	-	-	-	-	-	-	-	-
5703	11068	Phosphate transport ATP-binding protein PstB (TC 3.A.1.7.1)	1	-	-	-	-	-	-	-	-	-	-	-	-	+	-	-	-	-	-	-	-	-	-	-	-	-	-	-	-	-	-	-	-	-	-	-	-	-	-	-	-	-	-	-	-	-	-	-	-	-	-	-	-	-
5704	11069	hypothetical protein	1	-	-	-	-	-	-	-	-	-	-	-	-	+	-	-	-	-	-	-	-	-	-	-	-	-	-	-	-	-	-	-	-	-	-	-	-	-	-	-	-	-	-	-	-	-	-	-	-	-	-	-	-	-
5705	11070	Uncharacterized protein involved in cytokinesis	1	-	-	-	-	-	-	-	-	-	-	-	-	+	-	-	-	-	-	-	-	-	-	-	-	-	-	-	-	-	-	-	-	-	-	-	-	-	-	-	-	-	-	-	-	-	-	-	-	-	-	-	-	-
5706	11071	S-adenosylmethionine synthetase (EC 2.5.1.6)	1	-	-	-	-	-	-	-	-	-	-	-	-	+	-	-	-	-	-	-	-	-	-	-	-	-	-	-	-	-	-	-	-	-	-	-	-	-	-	-	-	-	-	-	-	-	-	-	-	-	-	-	-	-
5707	11072	Tyrosine-protein kinase transmembrane modulator EpsC	1	-	-	-	-	-	-	-	-	-	-	-	-	+	-	-	-	-	-	-	-	-	-	-	-	-	-	-	-	-	-	-	-	-	-	-	-	-	-	-	-	-	-	-	-	-	-	-	-	-	-	-	-	-
5708	11073	Putative ABC transporter (ATP-binding protein)	1	-	-	-	-	-	-	-	-	-	-	-	-	+	-	-	-	-	-	-	-	-	-	-	-	-	-	-	-	-	-	-	-	-	-	-	-	-	-	-	-	-	-	-	-	-	-	-	-	-	-	-	-	-
5709	11074	flippase Wzx	1	-	-	-	-	-	-	-	-	-	-	-	-	-	-	+	-	-	-	-	-	-	-	-	-	-	-	-	-	-	-	-	-	-	-	-	-	-	-	-	-	-	-	-	-	-	-	-	-	-	-	-	-	-
5710	11075	hypothetical protein	1	-	-	-	-	-	-	-	-	-	-	-	-	-	-	+	-	-	-	-	-	-	-	-	-	-	-	-	-	-	-	-	-	-	-	-	-	-	-	-	-	-	-	-	-	-	-	-	-	-	-	-	-	-
5711	11076	hypothetical protein	1	-	-	-	-	-	-	-	-	-	-	-	-	-	-	+	-	-	-	-	-	-	-	-	-	-	-	-	-	-	-	-	-	-	-	-	-	-	-	-	-	-	-	-	-	-	-	-	-	-	-	-	-	-
5712	11077	hypothetical protein	1	-	-	-	-	-	-	-	-	-	-	-	-	-	-	+	-	-	-	-	-	-	-	-	-	-	-	-	-	-	-	-	-	-	-	-	-	-	-	-	-	-	-	-	-	-	-	-	-	-	-	-	-	-
5713	11078	hypothetical protein	1	-	-	-	-	-	-	-	-	-	-	-	-	-	-	+	-	-	-	-	-	-	-	-	-	-	-	-	-	-	-	-	-	-	-	-	-	-	-	-	-	-	-	-	-	-	-	-	-	-	-	-	-	-
5714	11079	hypothetical protein	1	-	-	-	-	-	-	-	-	-	-	-	-	-	-	+	-	-	-	-	-	-	-	-	-	-	-	-	-	-	-	-	-	-	-	-	-	-	-	-	-	-	-	-	-	-	-	-	-	-	-	-	-	-
5715	11080	ABC transporter ATP-binding protein	1	-	-	-	-	-	-	-	-	-	-	-	-	-	-	+	-	-	-	-	-	-	-	-	-	-	-	-	-	-	-	-	-	-	-	-	-	-	-	-	-	-	-	-	-	-	-	-	-	-	-	-	-	-
5716	11081	hypothetical protein	1	-	-	-	-	-	-	-	-	-	-	-	-	-	-	+	-	-	-	-	-	-	-	-	-	-	-	-	-	-	-	-	-	-	-	-	-	-	-	-	-	-	-	-	-	-	-	-	-	-	-	-	-	-
5717	11082	hypothetical protein	1	-	-	-	-	-	-	-	-	-	-	-	-	-	-	+	-	-	-	-	-	-	-	-	-	-	-	-	-	-	-	-	-	-	-	-	-	-	-	-	-	-	-	-	-	-	-	-	-	-	-	-	-	-
5718	11083	hypothetical protein	1	-	-	-	-	-	-	-	-	-	-	-	-	-	-	+	-	-	-	-	-	-	-	-	-	-	-	-	-	-	-	-	-	-	-	-	-	-	-	-	-	-	-	-	-	-	-	-	-	-	-	-	-	-
5719	11084	glycosyl transferase	1	-	-	-	-	-	-	-	-	-	-	-	-	-	-	+	-	-	-	-	-	-	-	-	-	-	-	-	-	-	-	-	-	-	-	-	-	-	-	-	-	-	-	-	-	-	-	-	-	-	-	-	-	-
5720	11085	hypothetical protein	1	-	-	-	-	-	-	-	-	-	-	-	-	-	-	+	-	-	-	-	-	-	-	-	-	-	-	-	-	-	-	-	-	-	-	-	-	-	-	-	-	-	-	-	-	-	-	-	-	-	-	-	-	-
5721	11086	oligosaccharide repeat unit polymerase Wzy	1	-	-	-	-	-	-	-	-	-	-	-	-	-	-	+	-	-	-	-	-	-	-	-	-	-	-	-	-	-	-	-	-	-	-	-	-	-	-	-	-	-	-	-	-	-	-	-	-	-	-	-	-	-
5722	11087	major head protein	1	-	-	-	-	-	-	-	-	-	-	-	-	-	-	+	-	-	-	-	-	-	-	-	-	-	-	-	-	-	-	-	-	-	-	-	-	-	-	-	-	-	-	-	-	-	-	-	-	-	-	-	-	-
5723	11088	Helix-turn-helix motif	1	-	-	-	-	-	-	-	-	-	-	-	-	-	-	+	-	-	-	-	-	-	-	-	-	-	-	-	-	-	-	-	-	-	-	-	-	-	-	-	-	-	-	-	-	-	-	-	-	-	-	-	-	-
5724	11089	hypothetical protein	1	-	-	-	-	-	-	-	-	-	-	-	-	-	-	+	-	-	-	-	-	-	-	-	-	-	-	-	-	-	-	-	-	-	-	-	-	-	-	-	-	-	-	-	-	-	-	-	-	-	-	-	-	-
5725	11090	hypothetical protein	1	-	-	-	-	-	-	-	-	-	-	-	-	-	-	+	-	-	-	-	-	-	-	-	-	-	-	-	-	-	-	-	-	-	-	-	-	-	-	-	-	-	-	-	-	-	-	-	-	-	-	-	-	-
5726	11091	Thiamin-phosphate pyrophosphorylase (EC 2.5.1.3)	1	-	-	-	-	-	-	-	-	-	-	-	-	-	-	+	-	-	-	-	-	-	-	-	-	-	-	-	-	-	-	-	-	-	-	-	-	-	-	-	-	-	-	-	-	-	-	-	-	-	-	-	-	-
5727	11092	Phage tail assembly	1	-	-	-	-	-	-	-	-	-	-	-	-	-	-	-	-	-	-	-	-	-	-	-	-	-	-	-	-	-	-	-	-	-	-	-	-	-	-	-	-	+	-	-	-	-	-	-	-	-	-	-	-	-
5728	11093	hypothetical protein	1	-	-	-	-	-	-	-	-	-	-	-	-	-	-	-	-	-	-	-	-	-	-	-	-	-	-	-	-	-	-	-	-	-	-	-	-	-	-	-	-	+	-	-	-	-	-	-	-	-	-	-	-	-
5729	11094	hypothetical protein	1	-	-	-	-	-	-	-	-	-	-	-	-	-	-	-	-	-	-	-	-	-	-	-	-	-	-	-	-	-	-	-	-	-	-	-	-	-	-	-	-	+	-	-	-	-	-	-	-	-	-	-	-	-
5730	11095	hypothetical protein	1	-	-	-	-	-	-	-	-	-	-	-	-	-	-	-	-	-	-	-	-	-	-	-	-	-	-	-	-	-	-	-	-	-	-	-	-	-	-	-	-	+	-	-	-	-	-	-	-	-	-	-	-	-
5731	11096	hypothetical protein	1	-	-	-	-	-	-	-	-	-	-	-	-	-	-	-	-	-	-	-	-	-	-	-	-	-	-	-	-	-	-	-	-	-	-	-	-	-	-	-	-	+	-	-	-	-	-	-	-	-	-	-	-	-
5732	11097	hypothetical protein	1	-	-	-	-	-	-	-	-	-	-	-	-	-	-	-	-	-	-	-	-	-	-	-	-	-	-	-	-	-	-	-	-	-	-	-	-	-	-	-	-	+	-	-	-	-	-	-	-	-	-	-	-	-
5733	11098	Cyclic nucleotide-regulated ABC bacteriocin/lantibiotic exporters	1	-	-	-	-	-	-	-	-	-	-	-	-	-	-	-	-	-	-	-	-	-	-	-	-	-	-	-	-	-	-	-	-	-	-	-	-	-	-	-	-	+	-	-	-	-	-	-	-	-	-	-	-	-
5734	11099	hypothetical protein	1	-	-	-	-	-	-	-	-	-	-	-	-	-	-	-	-	-	-	-	-	-	-	-	-	-	-	-	-	-	-	-	-	-	-	-	-	-	-	-	-	+	-	-	-	-	-	-	-	-	-	-	-	-
5735	11100	hypothetical protein	1	-	-	-	-	-	-	-	-	-	-	-	-	-	-	-	-	-	-	-	-	-	-	-	-	-	-	-	-	-	-	-	-	-	-	-	-	-	-	-	-	+	-	-	-	-	-	-	-	-	-	-	-	-
5736	11101	hypothetical protein	1	-	-	-	-	-	-	-	-	-	-	-	-	-	-	-	-	-	-	-	-	-	-	-	-	-	-	-	-	-	-	-	-	-	-	-	-	-	-	-	-	+	-	-	-	-	-	-	-	-	-	-	-	-
5737	11102	hypothetical protein	1	-	-	-	-	-	-	-	-	-	-	-	-	-	-	-	-	-	-	-	-	-	-	-	-	-	-	-	-	-	-	-	-	-	-	-	-	-	-	-	-	+	-	-	-	-	-	-	-	-	-	-	-	-
5738	11103	hypothetical protein	1	-	-	-	-	-	-	-	-	-	-	-	-	-	-	-	-	-	-	-	-	-	-	-	-	-	-	-	-	-	-	-	-	-	-	-	-	-	-	-	-	+	-	-	-	-	-	-	-	-	-	-	-	-
5739	11104	hypothetical protein	1	-	-	-	-	-	-	-	-	-	-	-	-	-	-	-	-	-	-	-	-	-	-	-	-	-	-	-	-	-	-	-	-	-	-	-	-	-	-	-	-	+	-	-	-	-	-	-	-	-	-	-	-	-
5740	11105	hypothetical protein	1	-	-	-	-	-	-	-	-	-	-	-	-	-	-	-	-	-	-	-	-	-	-	-	-	-	-	-	-	-	-	-	-	-	-	-	-	-	-	-	-	+	-	-	-	-	-	-	-	-	-	-	-	-
5741	11106	hypothetical protein	1	-	-	-	-	-	-	-	-	-	-	-	-	-	-	-	-	-	-	-	-	-	-	-	-	-	-	-	-	-	-	-	-	-	-	-	-	-	-	-	-	+	-	-	-	-	-	-	-	-	-	-	-	-
5742	11107	hypothetical protein	1	-	-	-	-	-	-	-	-	-	-	-	-	-	-	-	-	-	-	-	-	-	-	-	-	-	-	-	-	-	-	-	-	-	-	-	-	-	-	-	-	+	-	-	-	-	-	-	-	-	-	-	-	-
5743	11108	hypothetical protein	1	-	-	-	-	-	-	-	-	-	-	-	-	-	-	-	-	-	-	-	-	-	-	-	-	-	-	-	-	-	-	-	-	-	-	-	-	-	-	-	-	+	-	-	-	-	-	-	-	-	-	-	-	-
5744	11109	hypothetical protein	1	-	-	-	-	-	-	-	-	-	-	-	-	-	-	-	-	-	-	-	-	-	-	-	-	-	-	-	-	-	-	-	-	-	-	-	-	-	-	-	-	+	-	-	-	-	-	-	-	-	-	-	-	-
5745	11110	hypothetical protein	1	-	-	-	-	-	-	-	-	-	-	-	-	-	-	-	-	-	-	-	-	-	-	-	-	-	-	-	-	-	-	-	-	-	-	-	-	-	-	-	-	+	-	-	-	-	-	-	-	-	-	-	-	-
5746	11111	hypothetical protein	1	-	-	-	-	-	-	-	-	-	-	-	-	-	-	-	-	-	-	-	-	-	-	-	-	-	-	-	-	-	-	-	-	-	-	-	-	-	-	-	-	+	-	-	-	-	-	-	-	-	-	-	-	-
5747	11112	Export protein for polysaccharides and teichoic acids	1	-	-	-	-	-	-	-	-	-	-	-	-	-	-	-	-	-	-	-	-	-	-	-	-	-	-	-	-	-	-	-	-	-	-	-	-	-	-	-	-	+	-	-	-	-	-	-	-	-	-	-	-	-
5748	11113	hypothetical protein	1	-	-	-	-	-	-	-	-	-	-	-	-	-	-	-	-	-	-	-	-	-	-	-	-	-	-	-	-	-	-	-	-	-	-	-	-	-	-	-	-	+	-	-	-	-	-	-	-	-	-	-	-	-
5749	11114	Uncharacterized membrane protein	1	-	-	-	-	-	-	-	-	-	-	-	-	-	-	-	-	-	-	-	-	-	-	-	-	-	-	-	-	-	-	-	-	-	-	-	-	-	-	-	-	+	-	-	-	-	-	-	-	-	-	-	-	-
5750	11115	polysaccharide biosynthesis protein cpsH(V)	1	-	-	-	-	-	-	-	-	-	-	-	-	-	-	-	-	-	-	-	-	-	-	-	-	-	-	-	-	-	-	-	-	-	-	-	-	-	-	-	-	+	-	-	-	-	-	-	-	-	-	-	-	-
5751	11116	hypothetical protein	1	-	-	-	-	-	-	-	-	-	-	-	-	-	-	-	-	-	-	-	-	-	-	-	-	-	-	-	-	-	-	-	-	-	-	-	-	-	-	-	-	+	-	-	-	-	-	-	-	-	-	-	-	-
5752	11117	hypothetical protein	1	-	-	-	-	-	-	-	-	-	-	-	-	-	-	-	-	-	-	-	-	-	-	-	-	-	-	-	-	-	-	-	-	-	-	-	-	-	-	-	-	+	-	-	-	-	-	-	-	-	-	-	-	-
5753	11118	Zeta toxin	1	-	-	-	-	-	-	-	-	-	-	-	-	-	-	-	-	-	-	-	-	-	-	-	-	-	-	-	-	-	-	-	-	-	-	-	-	-	-	-	-	+	-	-	-	-	-	-	-	-	-	-	-	-
5754	11119	hypothetical protein	1	-	-	-	-	-	-	-	-	-	-	-	-	-	-	-	-	-	-	-	-	-	-	-	-	-	-	-	-	-	-	-	-	-	-	-	-	-	-	-	-	+	-	-	-	-	-	-	-	-	-	-	-	-
5755	11120	hypothetical protein	1	-	-	-	-	-	-	-	-	-	-	-	-	-	-	-	-	-	-	-	-	-	-	-	-	-	-	-	-	-	-	-	-	-	-	-	-	-	-	-	-	+	-	-	-	-	-	-	-	-	-	-	-	-
5756	11121	hypothetical protein	1	-	-	-	-	-	-	-	-	-	-	-	-	-	-	-	-	-	-	-	-	-	-	-	-	-	-	-	-	-	-	-	-	-	-	-	-	-	-	-	-	+	-	-	-	-	-	-	-	-	-	-	-	-
5757	11122	hypothetical protein	1	-	-	-	-	-	-	-	-	-	-	-	-	-	-	-	-	-	-	-	-	-	-	-	-	-	-	-	-	-	-	-	-	-	-	-	-	-	-	-	-	+	-	-	-	-	-	-	-	-	-	-	-	-
5758	11123	Polysaccharide biosynthesis protein CpsF	1	-	-	-	-	-	-	-	-	-	-	-	-	-	-	-	-	-	-	-	-	-	-	-	-	-	-	-	-	-	-	-	-	-	-	-	-	-	-	-	-	+	-	-	-	-	-	-	-	-	-	-	-	-
5759	11124	hypothetical protein	1	-	-	-	-	-	-	-	-	-	-	-	-	-	-	-	-	-	-	-	-	-	-	-	-	-	-	-	-	-	-	-	-	-	-	-	-	-	-	-	-	+	-	-	-	-	-	-	-	-	-	-	-	-
5760	11125	Predicted ATP-binding protein involved in virulence	1	-	-	-	-	-	-	-	-	-	-	-	-	-	-	-	-	-	-	-	-	-	-	-	-	-	-	-	-	-	-	-	-	-	-	-	-	-	-	-	-	+	-	-	-	-	-	-	-	-	-	-	-	-
5761	11126	hypothetical protein	1	-	-	-	-	-	-	-	-	-	-	-	-	-	-	-	-	-	-	-	-	-	-	-	-	-	-	-	-	-	-	-	-	-	-	-	-	-	-	-	-	+	-	-	-	-	-	-	-	-	-	-	-	-
5762	11127	hypothetical protein	1	-	-	-	-	-	-	-	-	-	-	-	-	-	-	-	-	-	-	-	-	-	-	-	-	-	-	-	-	-	-	-	-	-	-	-	-	-	-	-	-	+	-	-	-	-	-	-	-	-	-	-	-	-
5763	11128	hypothetical protein	1	-	-	-	-	-	-	-	-	-	-	-	-	-	-	-	-	-	-	-	-	-	-	-	-	-	-	-	-	-	-	-	-	-	-	-	-	-	-	-	-	+	-	-	-	-	-	-	-	-	-	-	-	-
5764	11129	hypothetical protein	1	-	-	-	-	-	-	-	-	-	-	-	-	-	-	-	-	-	-	-	-	-	-	-	-	-	-	-	-	-	-	-	-	-	-	-	-	-	-	-	-	+	-	-	-	-	-	-	-	-	-	-	-	-
5765	11130	Translation initiation factor 2 (IF-2; GTPase)	1	-	-	-	-	-	-	-	-	-	-	-	-	-	-	-	-	-	-	-	-	-	-	-	-	-	-	-	-	-	-	-	-	-	-	-	-	-	-	-	-	+	-	-	-	-	-	-	-	-	-	-	-	-
5766	11131	hypothetical protein	1	-	-	-	-	-	-	-	-	-	-	-	-	-	-	-	-	-	-	-	-	-	-	-	-	-	-	-	-	-	-	-	-	-	-	-	-	-	-	-	-	+	-	-	-	-	-	-	-	-	-	-	-	-
5767	11132	hypothetical protein	1	-	-	-	-	-	-	-	-	-	-	-	-	-	-	-	-	-	-	-	-	-	-	-	-	-	-	-	-	-	-	-	-	-	-	-	-	-	-	-	-	+	-	-	-	-	-	-	-	-	-	-	-	-
5768	11133	hypothetical protein	1	-	-	-	-	-	-	-	-	-	-	-	-	-	-	-	-	-	-	-	-	-	-	-	-	-	-	-	-	-	-	-	-	-	-	-	-	-	-	-	-	+	-	-	-	-	-	-	-	-	-	-	-	-
5769	11134	hypothetical protein	1	-	-	-	-	-	-	-	-	-	-	-	-	-	-	-	-	-	-	-	-	-	-	-	-	-	-	-	-	-	-	-	-	-	-	-	-	-	-	-	-	+	-	-	-	-	-	-	-	-	-	-	-	-
5770	11135	hypothetical protein	1	-	-	-	-	-	-	-	-	-	-	-	-	-	-	-	-	-	-	-	-	-	-	-	-	-	-	-	-	-	-	-	-	-	-	-	-	-	-	-	-	+	-	-	-	-	-	-	-	-	-	-	-	-
5771	11136	Transcriptional regulator	1	-	-	-	-	-	-	-	-	-	-	-	-	-	-	-	-	-	-	-	-	-	-	-	-	-	-	-	-	-	-	-	-	-	-	-	-	-	-	-	-	+	-	-	-	-	-	-	-	-	-	-	-	-
5772	11137	hypothetical protein	1	-	-	-	-	-	-	-	-	-	-	-	-	-	-	-	-	-	-	-	-	-	-	-	-	-	-	-	-	-	-	-	-	-	-	-	-	-	-	-	-	+	-	-	-	-	-	-	-	-	-	-	-	-
5773	11138	hypothetical protein	1	-	-	-	-	-	-	-	-	-	-	-	-	-	-	-	-	-	-	-	-	-	-	-	-	-	-	-	-	-	-	-	-	-	-	-	-	-	-	-	-	+	-	-	-	-	-	-	-	-	-	-	-	-
5774	11139	hypothetical protein	1	-	-	-	-	-	-	-	-	-	-	-	-	-	-	-	-	-	-	-	-	-	-	-	-	-	-	-	-	-	-	-	-	-	-	-	-	-	-	-	-	+	-	-	-	-	-	-	-	-	-	-	-	-
5775	11140	hypothetical protein	1	-	-	-	-	-	-	-	-	-	-	-	-	-	-	-	-	-	-	-	-	-	-	-	-	-	-	-	-	-	-	-	-	-	-	-	-	-	-	-	-	+	-	-	-	-	-	-	-	-	-	-	-	-
5776	11141	hypothetical protein	1	-	-	-	-	-	-	-	-	-	-	-	-	-	-	-	-	-	-	-	-	-	-	-	-	-	-	-	-	-	-	-	-	-	-	-	-	-	-	-	-	+	-	-	-	-	-	-	-	-	-	-	-	-
5777	11142	hypothetical protein	1	-	-	-	-	-	-	-	-	-	-	-	-	-	-	-	-	-	-	-	-	-	-	-	-	-	-	-	-	-	-	-	-	-	-	-	-	-	-	-	-	+	-	-	-	-	-	-	-	-	-	-	-	-
5778	11143	hypothetical protein	1	-	-	-	-	-	-	-	-	-	-	-	-	-	-	-	-	-	-	-	-	-	-	-	-	-	-	-	-	-	-	-	-	-	-	-	-	-	-	-	-	+	-	-	-	-	-	-	-	-	-	-	-	-
5779	11144	putative glycosyl transferase	1	-	-	-	-	-	-	-	-	-	-	-	-	-	-	-	-	-	-	-	-	-	-	-	-	-	-	-	-	-	-	-	-	-	-	-	-	-	-	-	-	+	-	-	-	-	-	-	-	-	-	-	-	-
5780	11145	MutT/nudix family protein	1	-	-	-	-	-	-	-	-	-	-	-	-	-	-	-	-	-	-	-	-	-	-	-	-	-	-	-	-	-	-	-	-	-	-	-	-	-	-	-	-	+	-	-	-	-	-	-	-	-	-	-	-	-
5781	11146	hypothetical protein	1	-	-	-	-	-	-	-	-	-	-	-	-	-	-	-	-	-	-	-	-	-	-	-	-	-	-	-	-	-	-	-	-	-	-	-	-	-	-	-	-	+	-	-	-	-	-	-	-	-	-	-	-	-
5782	11147	hypothetical protein	1	-	-	-	-	-	-	-	-	-	-	-	-	-	-	-	-	-	-	-	-	-	-	-	-	-	-	-	-	-	-	-	-	-	-	-	-	-	-	-	-	+	-	-	-	-	-	-	-	-	-	-	-	-
5783	11148	hypothetical protein	1	-	-	-	-	-	-	-	-	-	-	-	-	-	-	-	-	-	-	-	-	-	-	-	-	-	-	-	-	-	-	-	-	-	-	-	-	-	-	-	-	+	-	-	-	-	-	-	-	-	-	-	-	-
5784	11149	Transposase	1	-	-	-	-	-	-	-	-	-	-	-	-	-	-	-	-	-	-	-	-	-	-	-	-	-	-	-	-	-	-	-	-	-	-	-	-	-	-	-	-	+	-	-	-	-	-	-	-	-	-	-	-	-
5785	11150	hypothetical protein	1	-	-	-	-	-	-	-	-	-	-	-	-	-	-	-	-	-	-	-	-	-	-	-	-	-	-	-	-	-	-	-	-	-	-	-	-	-	-	-	-	+	-	-	-	-	-	-	-	-	-	-	-	-
5786	11151	lantibiotic modifying enzyme	1	-	-	-	-	-	-	-	-	-	-	-	-	-	-	-	-	-	-	-	-	-	-	-	-	-	-	-	-	-	-	-	-	-	-	-	-	-	-	-	-	+	-	-	-	-	-	-	-	-	-	-	-	-
5787	11152	putative glycosyl transferase	1	-	-	-	-	-	-	-	-	-	-	-	-	-	-	-	-	-	-	-	-	-	-	-	-	-	-	-	-	-	-	-	-	-	-	-	-	-	-	-	-	+	-	-	-	-	-	-	-	-	-	-	-	-
5788	11153	polysaccharide biosynthesis protein	1	-	-	-	-	-	-	-	-	-	-	-	-	-	-	-	-	-	-	-	-	-	-	-	-	-	-	-	-	-	-	-	+	-	-	-	-	-	-	-	-	-	-	-	-	-	-	-	-	-	-	-	-	-
5789	11154	D-alanyl transfer protein DltB	1	-	-	-	-	-	-	-	-	-	-	-	-	-	-	-	-	-	-	-	-	-	-	-	-	-	-	-	-	-	-	-	+	-	-	-	-	-	-	-	-	-	-	-	-	-	-	-	-	-	-	-	-	-
5790	11155	hypothetical protein	1	-	-	-	-	-	-	-	-	-	-	-	-	-	-	-	-	-	-	-	-	-	-	-	-	-	-	-	-	-	-	-	+	-	-	-	-	-	-	-	-	-	-	-	-	-	-	-	-	-	-	-	-	-
5791	11156	hypothetical protein	1	-	-	-	-	-	-	-	-	-	-	-	-	-	-	-	-	-	-	-	-	-	-	-	-	-	-	-	-	-	-	-	+	-	-	-	-	-	-	-	-	-	-	-	-	-	-	-	-	-	-	-	-	-
5792	11157	hypothetical protein	1	-	-	-	-	-	-	-	-	-	-	-	-	-	-	-	-	-	-	-	-	-	-	-	-	-	-	-	-	-	-	-	+	-	-	-	-	-	-	-	-	-	-	-	-	-	-	-	-	-	-	-	-	-
5793	11158	Glycosyltransferase (EC 2.4.1.-)	1	-	-	-	-	-	-	-	-	-	-	-	-	-	-	-	-	-	-	-	-	-	-	-	-	-	-	-	-	-	-	-	+	-	-	-	-	-	-	-	-	-	-	-	-	-	-	-	-	-	-	-	-	-
5794	11159	hypothetical protein	1	-	-	-	-	-	-	-	-	-	-	-	-	-	-	-	-	-	-	-	-	-	-	-	-	-	-	-	-	-	-	-	+	-	-	-	-	-	-	-	-	-	-	-	-	-	-	-	-	-	-	-	-	-
5795	11160	hypothetical protein	1	-	-	-	-	-	-	-	-	-	-	-	-	-	-	-	-	-	-	-	-	-	-	-	-	-	-	-	-	-	-	-	+	-	-	-	-	-	-	-	-	-	-	-	-	-	-	-	-	-	-	-	-	-
5796	11161	hypothetical protein	1	-	-	-	-	-	-	-	-	-	-	-	-	-	-	-	-	-	-	-	-	-	-	-	-	-	-	-	-	-	-	-	+	-	-	-	-	-	-	-	-	-	-	-	-	-	-	-	-	-	-	-	-	-
5797	11162	hypothetical protein	1	-	-	-	-	-	-	-	-	-	-	-	-	-	-	-	-	-	-	-	-	-	-	-	-	-	-	-	-	-	-	-	+	-	-	-	-	-	-	-	-	-	-	-	-	-	-	-	-	-	-	-	-	-
5798	11163	hypothetical protein	1	-	-	-	-	-	-	-	-	-	-	-	-	-	-	-	-	-	-	-	-	-	-	-	-	-	-	-	-	-	-	-	+	-	-	-	-	-	-	-	-	-	-	-	-	-	-	-	-	-	-	-	-	-
5799	11164	glycosyl transferase	1	-	-	-	-	-	-	-	-	-	-	-	-	-	-	-	-	-	-	-	-	-	-	-	-	-	-	-	-	-	-	-	+	-	-	-	-	-	-	-	-	-	-	-	-	-	-	-	-	-	-	-	-	-
5800	11165	hypothetical protein	1	-	-	-	-	-	-	-	-	-	-	-	-	-	-	-	-	-	-	-	-	-	-	-	-	-	-	-	-	-	-	-	+	-	-	-	-	-	-	-	-	-	-	-	-	-	-	-	-	-	-	-	-	-
5801	11166	hypothetical protein	1	-	-	-	-	-	-	-	-	-	-	-	-	-	-	-	-	-	-	-	-	-	-	-	-	-	-	-	-	-	-	-	+	-	-	-	-	-	-	-	-	-	-	-	-	-	-	-	-	-	-	-	-	-
5802	11167	hypothetical protein	1	-	-	-	-	-	-	-	-	-	-	-	-	-	-	-	-	-	-	-	-	-	-	-	-	-	-	-	-	-	-	-	+	-	-	-	-	-	-	-	-	-	-	-	-	-	-	-	-	-	-	-	-	-
5803	11168	Positive transcriptional regulator	1	-	-	-	-	-	-	-	-	-	-	-	-	-	-	-	-	-	-	-	-	-	-	-	-	-	-	-	-	-	-	-	+	-	-	-	-	-	-	-	-	-	-	-	-	-	-	-	-	-	-	-	-	-
5804	11169	Maltose O-acetyltransferase (EC 2.3.1.79)	1	-	-	-	-	-	-	-	-	-	-	-	-	-	-	-	-	-	-	-	-	-	-	-	-	-	-	-	-	-	-	-	+	-	-	-	-	-	-	-	-	-	-	-	-	-	-	-	-	-	-	-	-	-
5805	11170	Type II restriction endonuclease	1	-	-	-	-	-	-	-	-	-	-	-	-	-	-	-	-	-	-	-	-	-	-	-	-	-	-	-	-	-	-	-	+	-	-	-	-	-	-	-	-	-	-	-	-	-	-	-	-	-	-	-	-	-
5806	11171	Glucuronide permease	1	-	-	-	-	-	-	-	-	-	-	-	-	-	-	-	-	-	-	-	-	-	-	-	-	-	-	-	-	-	-	-	+	-	-	-	-	-	-	-	-	-	-	-	-	-	-	-	-	-	-	-	-	-
5807	11172	hypothetical protein	1	-	-	-	-	-	-	-	-	-	-	-	-	-	-	-	-	-	-	-	-	-	-	-	-	-	-	-	-	-	-	-	-	-	+	-	-	-	-	-	-	-	-	-	-	-	-	-	-	-	-	-	-	-
5808	11173	hypothetical protein	1	-	-	-	-	-	-	-	-	-	-	-	-	-	-	-	-	-	-	-	-	-	-	-	-	-	-	-	-	-	-	-	-	-	+	-	-	-	-	-	-	-	-	-	-	-	-	-	-	-	-	-	-	-
5809	11174	hypothetical protein	1	-	-	-	-	-	-	-	-	-	-	-	-	-	-	-	-	-	-	-	-	-	-	-	-	-	-	-	-	-	-	-	-	-	+	-	-	-	-	-	-	-	-	-	-	-	-	-	-	-	-	-	-	-
5810	11175	hypothetical protein	1	-	-	-	-	-	-	-	-	-	-	-	-	-	-	-	-	-	-	-	-	-	-	-	-	-	-	-	-	-	-	-	-	-	+	-	-	-	-	-	-	-	-	-	-	-	-	-	-	-	-	-	-	-
5811	11176	Epf-like protein	1	-	-	-	-	-	-	-	-	-	-	-	-	-	-	-	-	-	-	-	-	-	-	-	-	-	-	-	-	-	-	-	-	-	+	-	-	-	-	-	-	-	-	-	-	-	-	-	-	-	-	-	-	-
5812	11177	L-fucose isomerase (EC 5.3.1.25)	1	-	-	-	-	-	-	-	-	-	-	-	-	-	-	-	-	-	-	-	-	-	-	-	-	-	-	-	-	-	-	-	-	-	+	-	-	-	-	-	-	-	-	-	-	-	-	-	-	-	-	-	-	-
5813	11178	hypothetical protein	1	-	-	-	-	-	-	-	-	-	-	-	-	-	-	-	-	-	-	-	-	-	-	-	-	-	-	-	-	-	-	-	-	-	+	-	-	-	-	-	-	-	-	-	-	-	-	-	-	-	-	-	-	-
5814	11179	hypothetical protein	1	-	-	-	-	-	-	-	-	-	-	-	-	-	-	-	-	-	-	-	-	-	-	-	-	-	-	-	-	-	-	-	-	-	+	-	-	-	-	-	-	-	-	-	-	-	-	-	-	-	-	-	-	-
5815	11180	L-fuculokinase (EC 2.7.1.51)	1	-	-	-	-	-	-	-	-	-	-	-	-	-	-	-	-	-	-	-	-	-	-	-	-	-	-	-	-	-	-	-	-	-	+	-	-	-	-	-	-	-	-	-	-	-	-	-	-	-	-	-	-	-
5816	11181	Oligoendopeptidase F	1	-	-	-	-	-	-	-	-	-	-	-	-	-	-	-	-	-	-	-	-	-	-	-	-	-	-	-	-	-	-	-	-	-	+	-	-	-	-	-	-	-	-	-	-	-	-	-	-	-	-	-	-	-
5817	11182	hypothetical protein	1	-	-	-	-	-	-	-	-	-	-	-	-	-	-	-	-	-	-	-	-	-	-	-	-	-	-	-	-	-	-	-	-	-	+	-	-	-	-	-	-	-	-	-	-	-	-	-	-	-	-	-	-	-
5818	11183	TrsE-like protein	1	-	-	-	-	-	-	-	-	-	-	-	-	-	-	-	-	-	-	-	-	-	-	-	-	-	-	-	-	-	-	-	-	-	+	-	-	-	-	-	-	-	-	-	-	-	-	-	-	-	-	-	-	-
5819	11184	Cysteine desulfurase (EC 2.8.1.7)	1	-	-	-	-	-	-	-	-	-	-	-	-	-	-	-	-	-	-	-	-	-	-	-	-	-	-	-	-	-	-	-	-	-	+	-	-	-	-	-	-	-	-	-	-	-	-	-	-	-	-	-	-	-
5820	11185	hypothetical protein	1	-	-	-	-	-	-	-	-	-	-	-	-	-	-	-	-	-	-	-	-	-	-	-	-	-	-	-	-	-	-	-	-	-	+	-	-	-	-	-	-	-	-	-	-	-	-	-	-	-	-	-	-	-
5821	11186	Transcriptional regulator	1	-	-	-	-	-	-	-	-	-	-	-	-	-	-	-	-	-	-	-	-	-	-	-	-	-	-	-	-	-	-	-	-	-	+	-	-	-	-	-	-	-	-	-	-	-	-	-	-	-	-	-	-	-
5822	11187	hypothetical protein	1	-	-	-	-	-	-	-	-	-	-	-	-	-	-	-	-	-	-	-	-	-	-	-	-	-	-	-	-	-	-	-	-	-	+	-	-	-	-	-	-	-	-	-	-	-	-	-	-	-	-	-	-	-
5823	11188	ABC-type multidrug transport system	1	-	-	-	-	-	-	-	-	-	-	-	-	-	-	-	-	-	-	-	-	-	-	-	-	-	-	-	-	-	-	-	-	-	+	-	-	-	-	-	-	-	-	-	-	-	-	-	-	-	-	-	-	-
5824	11189	hypothetical protein	1	-	-	-	-	-	-	-	-	-	-	-	-	-	-	-	-	-	-	-	-	-	-	-	-	-	-	-	-	-	-	-	-	-	+	-	-	-	-	-	-	-	-	-	-	-	-	-	-	-	-	-	-	-
5825	11190	polysaccharide biosynthesis protein	1	-	-	-	-	-	-	-	-	-	-	-	-	-	-	-	-	-	-	-	-	-	-	-	-	-	-	-	-	-	-	-	-	-	+	-	-	-	-	-	-	-	-	-	-	-	-	-	-	-	-	-	-	-
5826	11191	kilA protein	1	-	-	-	-	-	-	-	-	-	-	-	-	-	-	-	-	-	-	-	-	-	-	-	-	-	-	-	-	-	-	-	-	-	+	-	-	-	-	-	-	-	-	-	-	-	-	-	-	-	-	-	-	-
5827	11192	Glycosyl transferase	1	-	-	-	-	-	-	-	-	-	-	-	-	-	-	-	-	-	-	-	-	-	-	-	-	-	-	-	-	-	-	-	-	-	+	-	-	-	-	-	-	-	-	-	-	-	-	-	-	-	-	-	-	-
5828	11193	Exopolysaccharide biosynthesis protein	1	-	-	-	-	-	-	-	-	-	-	-	-	-	-	-	-	-	-	-	-	-	-	-	-	-	-	-	-	-	-	-	-	-	+	-	-	-	-	-	-	-	-	-	-	-	-	-	-	-	-	-	-	-
5829	11194	hypothetical protein	1	-	-	-	-	-	-	-	-	-	-	-	-	-	-	-	-	-	-	-	-	-	-	-	-	-	-	-	-	-	-	-	-	-	+	-	-	-	-	-	-	-	-	-	-	-	-	-	-	-	-	-	-	-
5830	11195	Putative RTX family exoprotein A gene	1	-	-	-	-	-	-	-	-	-	-	-	-	-	-	-	-	-	-	-	-	-	-	-	-	-	-	-	-	-	-	-	-	-	+	-	-	-	-	-	-	-	-	-	-	-	-	-	-	-	-	-	-	-
5831	11196	hypothetical protein	1	-	-	-	-	-	-	-	-	-	-	-	-	-	-	-	-	-	-	-	-	-	-	-	-	-	-	-	-	-	-	-	-	-	+	-	-	-	-	-	-	-	-	-	-	-	-	-	-	-	-	-	-	-
5832	11197	hypothetical protein	1	-	-	-	-	-	-	-	-	-	-	-	-	-	-	-	-	-	-	-	-	-	-	-	-	-	-	-	-	-	-	-	-	-	+	-	-	-	-	-	-	-	-	-	-	-	-	-	-	-	-	-	-	-
5833	11198	hypothetical protein	1	-	-	-	-	-	-	-	-	-	-	-	-	-	-	-	-	-	-	-	-	-	-	-	-	-	-	-	-	-	-	-	-	-	+	-	-	-	-	-	-	-	-	-	-	-	-	-	-	-	-	-	-	-
5834	11199	Lipid carrier : UDP-N-acetylgalactosaminyltransferase (EC 2.4.1.-) / Alpha-1	1	-	-	-	-	-	-	-	-	-	-	-	-	-	-	-	-	-	-	-	-	-	-	-	-	-	-	-	-	-	-	-	-	-	+	-	-	-	-	-	-	-	-	-	-	-	-	-	-	-	-	-	-	-
5835	11200	hypothetical protein	1	-	-	-	-	-	-	-	-	-	-	-	-	-	-	-	-	-	-	-	-	-	-	-	-	-	-	-	-	-	-	-	-	-	+	-	-	-	-	-	-	-	-	-	-	-	-	-	-	-	-	-	-	-
5836	11201	hypothetical protein	1	-	-	-	-	-	-	-	-	-	-	-	-	-	-	-	-	-	-	-	-	-	-	-	-	-	-	-	-	-	-	-	-	-	+	-	-	-	-	-	-	-	-	-	-	-	-	-	-	-	-	-	-	-
5837	11202	hypothetical protein	1	-	-	-	-	-	-	-	-	-	-	-	-	-	-	-	-	-	-	-	-	-	-	-	-	-	-	-	-	-	-	-	-	-	+	-	-	-	-	-	-	-	-	-	-	-	-	-	-	-	-	-	-	-
5838	11203	hypothetical protein	1	-	-	-	-	-	-	-	-	-	-	-	-	-	-	-	-	-	-	-	-	-	-	-	-	-	-	-	-	-	-	-	-	-	+	-	-	-	-	-	-	-	-	-	-	-	-	-	-	-	-	-	-	-
5839	11204	Putative type II restriction enzyme	1	-	-	-	-	-	-	-	-	-	-	-	-	-	-	-	-	-	-	-	-	-	-	-	-	-	-	-	-	-	-	-	-	-	+	-	-	-	-	-	-	-	-	-	-	-	-	-	-	-	-	-	-	-
5840	11205	hypothetical protein	1	-	-	-	-	-	-	-	-	-	-	-	-	-	-	-	-	-	-	-	-	-	-	-	-	-	-	-	-	-	-	-	-	+	-	-	-	-	-	-	-	-	-	-	-	-	-	-	-	-	-	-	-	-
5841	11206	Helicase encoded by the Y' element of subtelomeric regions	1	-	-	-	-	-	-	-	-	-	-	-	-	-	-	-	-	-	-	-	-	-	-	-	-	-	-	-	-	-	-	-	-	+	-	-	-	-	-	-	-	-	-	-	-	-	-	-	-	-	-	-	-	-
5842	11207	Mobile element protein	1	-	-	-	-	-	-	-	-	-	-	-	-	-	-	-	-	-	-	-	-	-	-	-	-	-	-	-	-	-	-	-	-	+	-	-	-	-	-	-	-	-	-	-	-	-	-	-	-	-	-	-	-	-

Table S5. Precence/absence of each HG among 57 *S. suis* strains

				1																13			25					28						104		94			76			53/54		Divergent *S. suis*																
Index[a]	ClusterID	Gene products	Members	98HAH33	05ZYH33	SC84	A7	SS12	P1/7	DAT273	NIAH11434	JS14	GZ1	BM407	S735	13730	MNCM07	NCTC 10446	14636	NCTC 10237	ST1	2651	89-1591	MNCM04	MNCM25	8074	D9	4961	ST3	MNCM43	DAT292	DAT260	NIAH11435	MNCM21	MNCM50	89-2479	DAT234	DAT290	93A	42A	NT77	6407	11538	8830	12814	92-1400	89-590	2726	2524	89-3576-3	4417	D12	10581	89-5259	88-5299A	R61	92-1191	14A	92-4172	22083
5843	11208	hypothetical protein	1	-	-	-	-	-	-	-	-	-	-	-	-	-	-	-	-	-	-	-	-	-	-	-	-	-	-	-	-	-	-	-	-	-	-	-	-	+	-	-	-	-	-	-	-	-	-	-	-	-	-	-	-	-	-	-	-	-
5844	11209	PTS system	1	-	-	-	-	-	-	-	-	-	-	-	-	-	-	-	-	-	-	-	-	-	-	-	-	-	-	-	-	-	-	-	-	-	-	-	-	+	-	-	-	-	-	-	-	-	-	-	-	-	-	-	-	-	-	-	-	-
5845	11210	N-6 DNA methylase	1	-	-	-	-	-	-	-	-	-	-	-	-	-	-	-	-	-	-	-	-	-	-	-	-	-	-	-	-	-	-	-	-	-	-	-	-	+	-	-	-	-	-	-	-	-	-	-	-	-	-	-	-	-	-	-	-	-
5846	11211	Serine acetyltransferase (EC 2.3.1.30)	1	-	-	-	-	-	-	-	-	-	-	-	-	-	-	-	-	-	-	-	-	-	-	-	-	-	-	-	-	-	-	-	-	-	-	-	-	+	-	-	-	-	-	-	-	-	-	-	-	-	-	-	-	-	-	-	-	-
5847	11212	Hydrolase (HAD superfamily)	1	-	-	-	-	-	-	-	-	-	-	-	-	-	-	-	-	-	-	-	-	-	-	-	-	-	-	-	-	-	-	-	-	-	-	-	-	+	-	-	-	-	-	-	-	-	-	-	-	-	-	-	-	-	-	-	-	-
5848	11213	hypothetical protein	1	-	-	-	-	-	-	-	-	-	-	-	-	-	-	-	-	-	-	-	-	-	-	-	-	-	-	-	-	-	-	-	-	-	-	-	-	+	-	-	-	-	-	-	-	-	-	-	-	-	-	-	-	-	-	-	-	-
5849	11214	hypothetical protein	1	-	-	-	-	-	-	-	-	-	-	-	-	-	-	-	-	-	-	-	-	-	-	-	-	-	-	-	-	-	-	-	-	-	-	-	-	+	-	-	-	-	-	-	-	-	-	-	-	-	-	-	-	-	-	-	-	-
5850	11215	PTS system	1	-	-	-	-	-	-	-	-	-	-	-	-	-	-	-	-	-	-	-	-	-	-	-	-	-	-	-	-	-	-	-	-	-	-	-	-	+	-	-	-	-	-	-	-	-	-	-	-	-	-	-	-	-	-	-	-	-
5851	11216	hypothetical protein	1	-	-	-	-	-	-	-	-	-	-	-	-	-	-	-	-	-	-	-	-	-	-	-	-	-	-	-	-	-	-	-	-	-	-	-	-	+	-	-	-	-	-	-	-	-	-	-	-	-	-	-	-	-	-	-	-	-
5852	11217	dTDP-glucose 4	1	-	-	-	-	-	-	-	-	-	-	-	-	-	-	-	-	-	-	-	-	-	-	-	-	-	-	-	-	-	-	-	-	-	-	-	-	+	-	-	-	-	-	-	-	-	-	-	-	-	-	-	-	-	-	-	-	-
5853	11218	Acetylornithine aminotransferase (EC 2.6.1.11)	1	-	-	-	-	-	-	-	-	-	-	-	-	-	-	-	-	-	-	-	-	-	-	-	-	-	-	-	-	-	-	-	-	-	-	-	-	+	-	-	-	-	-	-	-	-	-	-	-	-	-	-	-	-	-	-	-	-
5854	11219	Lipopolysaccharide biosynthesis protein WzxC	1	-	-	-	-	-	-	-	-	-	-	-	-	-	-	-	-	-	-	-	-	-	-	-	-	-	-	-	-	-	-	-	-	-	-	-	-	+	-	-	-	-	-	-	-	-	-	-	-	-	-	-	-	-	-	-	-	-
5855	11220	NAD(P)HX dehydratase	1	-	-	-	-	-	-	-	-	-	-	-	-	-	-	-	-	-	-	-	-	-	-	-	-	-	-	-	-	-	-	-	-	-	-	-	-	+	-	-	-	-	-	-	-	-	-	-	-	-	-	-	-	-	-	-	-	-
5856	11221	hypothetical protein	1	-	-	-	-	-	-	-	-	-	-	-	-	-	-	-	-	-	-	-	-	-	-	-	-	-	-	-	-	-	-	-	-	-	-	-	-	+	-	-	-	-	-	-	-	-	-	-	-	-	-	-	-	-	-	-	-	-
5857	11222	Predicted membrane protein	1	-	-	-	-	-	-	-	-	-	-	-	-	-	-	-	-	-	-	-	-	-	-	-	-	-	-	-	-	-	-	-	-	-	-	-	-	+	-	-	-	-	-	-	-	-	-	-	-	-	-	-	-	-	-	-	-	-
5858	11223	SNF2 family protein	1	-	-	-	-	-	-	-	-	-	-	-	-	-	-	-	-	-	-	-	-	-	-	-	-	-	-	-	-	-	-	-	-	-	-	-	-	+	-	-	-	-	-	-	-	-	-	-	-	-	-	-	-	-	-	-	-	-
5859	11224	putative glycosyltransferase	1	-	-	-	-	-	-	-	-	-	-	-	-	-	-	-	-	-	-	-	-	-	-	-	-	-	-	-	-	-	-	-	-	-	-	-	-	+	-	-	-	-	-	-	-	-	-	-	-	-	-	-	-	-	-	-	-	-
5860	11225	putative HsdR	1	-	-	-	-	-	-	-	-	-	-	-	-	-	-	-	-	-	-	-	-	-	-	-	-	-	-	-	-	-	-	-	-	-	-	-	-	+	-	-	-	-	-	-	-	-	-	-	-	-	-	-	-	-	-	-	-	-
5861	11226	teichoic acid biosynthesis protein	1	-	-	-	-	-	-	-	-	-	-	-	-	-	-	-	-	-	-	-	-	-	-	-	-	-	-	-	-	-	-	-	-	-	-	-	-	+	-	-	-	-	-	-	-	-	-	-	-	-	-	-	-	-	-	-	-	-
5862	11227	Glycosyl transferase	1	-	-	-	-	-	-	-	-	-	-	-	-	-	-	-	-	-	-	-	-	-	-	-	-	-	-	-	-	-	-	-	-	-	-	-	-	+	-	-	-	-	-	-	-	-	-	-	-	-	-	-	-	-	-	-	-	-
5863	11228	hypothetical protein	1	-	-	-	-	-	-	-	-	-	-	-	-	-	-	-	-	-	-	-	-	-	-	-	-	-	-	-	-	-	-	-	-	-	-	-	-	+	-	-	-	-	-	-	-	-	-	-	-	-	-	-	-	-	-	-	-	-
5864	11229	ClpB protein	1	-	-	-	-	-	-	-	-	-	-	-	-	-	-	-	-	-	-	-	-	-	-	-	-	-	-	-	-	-	-	-	-	-	-	-	-	+	-	-	-	-	-	-	-	-	-	-	-	-	-	-	-	-	-	-	-	-
5865	11230	hypothetical protein	1	-	-	-	-	-	-	-	-	-	-	-	-	-	-	-	-	-	-	-	-	-	-	-	-	-	-	-	-	-	-	-	-	-	-	-	-	+	-	-	-	-	-	-	-	-	-	-	-	-	-	-	-	-	-	-	-	-
5866	11367	dipeptidyl aminopeptidase/acylaminoacyl-peptidase related protein	1	-	-	-	-	-	-	-	-	-	-	-	-	-	-	-	-	-	-	-	-	-	-	-	-	-	-	-	-	-	-	-	-	-	-	-	-	-	-	-	-	-	-	-	-	-	-	-	-	-	-	-	-	-	-	+	-	-
5867	11368	hypothetical protein	1	-	-	-	-	-	-	-	-	-	-	-	-	-	-	-	-	-	-	-	-	-	-	-	-	-	-	-	-	-	-	-	-	-	-	-	-	-	-	-	-	-	-	-	-	-	-	-	-	-	-	-	-	-	-	+	-	-
5868	11369	Type II restriction enzyme HpaII (EC 3.1.21.4) (Endonuclease HpaII) (R.HpaII)	1	-	-	-	-	-	-	-	-	-	-	-	-	-	-	-	-	-	-	-	-	-	-	-	-	-	-	-	-	-	-	-	-	-	-	-	-	-	-	-	-	-	-	-	-	-	-	-	-	-	-	-	-	-	-	+	-	-
5869	11370	hypothetical protein	1	-	-	-	-	-	-	-	-	-	-	-	-	-	-	-	-	-	-	-	-	-	-	-	-	-	-	-	-	-	-	-	-	-	-	-	-	-	-	-	-	-	-	-	-	-	-	-	-	-	-	-	-	-	-	+	-	-
5870	11371	Alpha-L-Rha alpha-1	1	-	-	-	-	-	-	-	-	-	-	-	-	-	-	-	-	-	-	-	-	-	-	-	-	-	-	-	-	-	-	-	-	-	-	-	-	-	-	-	-	-	-	-	-	-	-	-	-	-	-	-	-	-	-	+	-	-
5871	11372	hypothetical protein	1	-	-	-	-	-	-	-	-	-	-	-	-	-	-	-	-	-	-	-	-	-	-	-	-	-	-	-	-	-	-	-	-	-	-	-	-	-	-	-	-	-	-	-	-	-	-	-	-	-	-	-	-	-	-	+	-	-
5872	11373	Acetyltransferase	1	-	-	-	-	-	-	-	-	-	-	-	-	-	-	-	-	-	-	-	-	-	-	-	-	-	-	-	-	-	-	-	-	-	-	-	-	-	-	-	-	-	-	-	-	-	-	-	-	-	-	-	-	-	-	+	-	-
5873	11374	hypothetical protein	1	-	-	-	-	-	-	-	-	-	-	-	-	-	-	-	-	-	-	-	-	-	-	-	-	-	-	-	-	-	-	-	-	-	-	-	-	-	-	-	-	-	-	-	-	-	-	-	-	-	-	-	-	-	-	+	-	-
5874	11375	hypothetical protein	1	-	-	-	-	-	-	-	-	-	-	-	-	-	-	-	-	-	-	-	-	-	-	-	-	-	-	-	-	-	-	-	-	-	-	-	-	-	-	-	-	-	-	-	-	-	-	-	-	-	-	-	-	-	-	+	-	-
5875	11376	hypothetical protein	1	-	-	-	-	-	-	-	-	-	-	-	-	-	-	-	-	-	-	-	-	-	-	-	-	-	-	-	-	-	-	-	-	-	-	-	-	-	-	-	-	-	-	-	-	-	-	-	-	-	-	-	-	-	-	+	-	-
5876	11377	IS66 family element	1	-	-	-	-	-	-	-	-	-	-	-	-	-	-	-	-	-	-	-	-	-	-	-	-	-	-	-	-	-	-	-	-	-	-	-	-	-	-	-	-	-	-	-	-	-	-	-	-	-	-	-	-	-	-	+	-	-
5877	11378	hypothetical protein	1	-	-	-	-	-	-	-	-	-	-	-	-	-	-	-	-	-	-	-	-	-	-	-	-	-	-	-	-	-	-	-	-	-	-	-	-	-	-	-	-	-	-	-	-	-	-	-	-	-	-	-	-	-	-	+	-	-
5878	11379	hypothetical protein	1	-	-	-	-	-	-	-	-	-	-	-	-	-	-	-	-	-	-	-	-	-	-	-	-	-	-	-	-	-	-	-	-	-	-	-	-	-	-	-	-	-	-	-	-	-	-	-	-	-	-	-	-	-	-	+	-	-
5879	11380	hypothetical protein	1	-	-	-	-	-	-	-	-	-	-	-	-	-	-	-	-	-	-	-	-	-	-	-	-	-	-	-	-	-	-	-	-	-	-	-	-	-	-	-	-	-	-	-	-	-	-	-	-	-	-	-	-	-	-	+	-	-
5880	11381	hypothetical protein	1	-	-	-	-	-	-	-	-	-	-	-	-	-	-	-	-	-	-	-	-	-	-	-	-	-	-	-	-	-	-	-	-	-	-	-	-	-	-	-	-	-	-	-	-	-	-	-	-	-	-	-	-	-	-	+	-	-
5881	11382	rha protein	1	-	-	-	-	-	-	-	-	-	-	-	-	-	-	-	-	-	-	-	-	-	-	-	-	-	-	-	-	-	-	-	-	-	-	-	-	-	-	-	-	-	-	-	-	-	-	-	-	-	-	-	-	-	-	+	-	-
5882	11383	hypothetical protein	1	-	-	-	-	-	-	-	-	-	-	-	-	-	-	-	-	-	-	-	-	-	-	-	-	-	-	-	-	-	-	-	-	-	-	-	-	-	-	-	-	-	-	-	-	-	-	-	-	-	-	-	-	-	-	+	-	-
5883	11384	capsule repeat unit polymerase	1	-	-	-	-	-	-	-	-	-	-	-	-	-	-	-	-	-	-	-	-	-	-	-	-	-	-	-	-	-	-	-	-	-	-	-	-	-	-	-	-	-	-	-	-	-	-	-	-	-	-	-	-	-	-	+	-	-
5884	11385	hypothetical protein	1	-	-	-	-	-	-	-	-	-	-	-	-	-	-	-	-	-	-	-	-	-	-	-	-	-	-	-	-	-	-	-	-	-	-	-	-	-	-	-	-	-	-	-	-	-	-	-	-	-	-	-	-	-	-	+	-	-
5885	11386	hypothetical protein	1	-	-	-	-	-	-	-	-	-	-	-	-	-	-	-	-	-	-	-	-	-	-	-	-	-	-	-	-	-	-	-	-	-	-	-	-	-	-	-	-	-	-	-	-	-	-	-	-	-	-	-	-	-	-	+	-	-
5886	11387	hypothetical protein	1	-	-	-	-	-	-	-	-	-	-	-	-	-	-	-	-	-	-	-	-	-	-	-	-	-	-	-	-	-	-	-	-	-	-	-	-	-	-	-	-	-	-	-	-	-	-	-	-	-	-	-	-	-	-	+	-	-
5887	11388	hypothetical protein	1	-	-	-	-	-	-	-	-	-	-	-	-	-	-	-	-	-	-	-	-	-	-	-	-	-	-	-	-	-	-	-	-	-	-	-	-	-	-	-	-	-	-	-	-	-	-	-	-	-	-	-	-	-	-	+	-	-
5888	11389	hypothetical protein	1	-	-	-	-	-	-	-	-	-	-	-	-	-	-	-	-	-	-	-	-	-	-	-	-	-	-	-	-	-	-	-	-	-	-	-	-	-	-	-	-	-	-	-	-	-	-	-	-	-	-	-	-	-	-	+	-	-
5889	11390	hypothetical protein	1	-	-	-	-	-	-	-	-	-	-	-	-	-	-	-	-	-	-	-	-	-	-	-	-	-	-	-	-	-	-	-	-	-	-	-	-	-	-	-	-	-	-	-	-	-	-	-	-	-	-	-	-	-	-	+	-	-
5890	11391	hypothetical protein	1	-	-	-	-	-	-	-	-	-	-	-	-	-	-	-	-	-	-	-	-	-	-	-	-	-	-	-	-	-	-	-	-	-	-	-	-	-	-	-	-	-	-	-	-	-	-	-	-	-	-	-	-	-	-	+	-	-
5891	11392	hypothetical protein	1	-	-	-	-	-	-	-	-	-	-	-	-	-	-	-	-	-	-	-	-	-	-	-	-	-	-	-	-	-	-	-	-	-	-	-	-	-	-	-	-	-	-	-	-	-	-	-	-	-	-	-	-	-	-	+	-	-
5892	11393	hypothetical protein	1	-	-	-	-	-	-	-	-	-	-	-	-	-	-	-	-	-	-	-	-	-	-	-	-	-	-	-	-	-	-	-	-	-	-	-	-	-	-	-	-	-	-	-	-	-	-	-	-	-	-	-	-	-	-	+	-	-
5893	11394	hypothetical protein	1	-	-	-	-	-	-	-	-	-	-	-	-	-	-	-	-	-	-	-	-	-	-	-	-	-	-	-	-	-	-	-	-	-	-	-	-	-	-	-	-	-	-	-	-	-	-	-	-	-	-	-	-	-	-	+	-	-
5894	11395	hypothetical protein	1	-	-	-	-	-	-	-	-	-	-	-	-	-	-	-	-	-	-	-	-	-	-	-	-	-	-	-	-	-	-	-	-	-	-	-	-	-	-	-	-	-	-	-	-	-	-	-	-	-	-	-	-	-	-	+	-	-
5895	11396	DNA-cytosine methyltransferase (EC 2.1.1.37)	1	-	-	-	-	-	-	-	-	-	-	-	-	-	-	-	-	-	-	-	-	-	-	-	-	-	-	-	-	-	-	-	-	-	-	-	-	-	-	-	-	-	-	-	-	-	-	-	-	-	-	-	-	-	-	+	-	-
5896	11397	hypothetical protein	1	-	-	-	-	-	-	-	-	-	-	-	-	-	-	-	-	-	-	-	-	-	-	-	-	-	-	-	-	-	-	-	-	-	-	-	-	-	-	-	-	-	-	-	-	-	-	-	-	-	-	-	-	-	-	+	-	-
5897	11398	hypothetical protein	1	-	-	-	-	-	-	-	-	-	-	-	-	-	-	-	-	-	-	-	-	-	-	-	-	-	-	-	-	-	-	-	-	-	-	-	-	-	-	-	-	-	-	-	-	-	-	-	-	-	-	-	-	-	-	+	-	-
5898	11399	hypothetical protein	1	-	-	-	-	-	-	-	-	-	-	-	-	-	-	-	-	-	-	-	-	-	-	-	-	-	-	-	-	-	-	-	-	-	-	-	-	-	-	-	-	-	-	-	-	-	-	-	-	-	-	-	-	-	-	+	-	-
5899	11400	hypothetical protein	1	-	-	-	-	-	-	-	-	-	-	-	-	-	-	-	-	-	-	-	-	-	-	-	-	-	-	-	-	-	-	-	-	-	-	-	-	-	-	-	-	-	-	-	-	-	-	-	-	-	-	-	-	-	-	+	-	-
5900	11401	hypothetical protein	1	-	-	-	-	-	-	-	-	-	-	-	-	-	-	-	-	-	-	-	-	-	-	-	-	-	-	-	-	-	-	-	-	-	-	-	-	-	-	-	-	-	-	-	-	-	-	-	-	-	-	-	-	-	-	+	-	-
5901	11402	hypothetical protein	1	-	-	-	-	-	-	-	-	-	-	-	-	-	-	-	-	-	-	-	-	-	-	-	-	-	-	-	-	-	-	-	-	-	-	-	-	-	-	-	-	-	-	-	-	-	-	-	-	-	-	-	-	-	-	+	-	-
5902	11403	hypothetical protein	1	-	-	-	-	-	-	-	-	-	-	-	-	-	-	-	-	-	-	-	-	-	-	-	-	-	-	-	-	-	-	-	-	-	-	-	-	-	-	-	-	-	-	-	-	-	-	-	-	-	-	-	-	-	-	+	-	-
5903	11404	EXONUCLEASE SBCC(EC:3.1.11.-)	1	-	-	-	-	-	-	-	-	-	-	-	-	-	-	-	-	-	-	-	-	-	-	-	-	-	-	-	-	-	-	-	-	-	-	-	-	-	-	-	-	-	-	-	-	-	-	-	-	-	-	-	-	-	-	+	-	-
5904	11405	some similarities to phage related proteins	1	-	-	-	-	-	-	-	-	-	-	-	-	-	-	-	-	-	-	-	-	-	-	-	-	-	-	-	-	-	-	-	-	-	-	-	-	-	-	-	-	-	-	-	-	-	-	-	-	-	-	-	-	-	-	+	-	-
5905	11406	hypothetical protein	1	-	-	-	-	-	-	-	-	-	-	-	-	-	-	-	-	-	-	-	-	-	-	-	-	-	-	-	-	-	-	-	-	-	-	-	-	-	-	-	-	-	-	-	-	-	-	-	-	-	-	-	-	-	-	+	-	-
5906	11407	hypothetical protein	1	-	-	-	-	-	-	-	-	-	-	-	-	-	-	-	-	-	-	-	-	-	-	-	-	-	-	-	-	-	-	-	-	-	-	-	-	-	-	-	-	-	-	-	-	-	-	-	-	-	-	-	-	-	-	+	-	-
5907	11408	hypothetical protein	1	-	-	-	-	-	-	-	-	-	-	-	-	-	-	-	-	-	-	-	-	-	-	-	-	-	-	-	-	-	-	-	-	-	-	-	-	-	-	-	-	-	-	-	-	-	-	-	-	-	-	-	-	-	-	+	-	-
5908	11409	hypothetical protein	1	-	-	-	-	-	-	-	-	-	-	-	-	-	-	-	-	-	-	-	-	-	-	-	-	-	-	-	-	-	-	-	-	-	-	-	-	-	-	-	-	-	-	-	-	-	-	-	-	-	-	-	-	-	-	+	-	-
5909	11410	hypothetical protein	1	-	-	-	-	-	-	-	-	-	-	-	-	-	-	-	-	-	-	-	-	-	-	-	-	-	-	-	-	-	-	-	-	-	-	-	-	-	-	-	-	-	-	-	-	-	-	-	-	-	-	-	-	-	-	+	-	-
5910	11411	Mobile element protein	1	-	-	-	-	-	-	-	-	-	-	-	-	-	-	-	-	-	-	-	-	-	-	-	-	-	-	-	-	-	-	-	-	-	-	-	-	-	-	-	-	-	-	-	-	-	-	-	-	-	-	-	-	-	-	+	-	-
5911	11412	hypothetical protein	1	-	-	-	-	-	-	-	-	-	-	-	-	-	-	-	-	-	-	-	-	-	-	-	-	-	-	-	-	-	-	-	-	-	-	-	-	-	-	-	-	-	-	-	-	-	-	-	-	-	-	-	-	-	-	+	-	-
5912	11413	hypothetical protein	1	-	-	-	-	-	-	-	-	-	-	-	-	-	-	-	-	-	-	-	-	-	-	-	-	-	-	-	-	-	-	-	-	-	-	-	-	-	-	-	-	-	-	-	-	-	-	-	-	-	-	-	-	-	-	+	-	-
5913	11414	hypothetical protein	1	-	-	-	-	-	-	-	-	-	-	-	-	-	-	-	-	-	-	-	-	-	-	-	-	-	-	-	-	-	-	-	-	-	-	-	-	-	-	-	-	-	-	-	-	-	-	-	-	-	-	-	-	-	-	+	-	-
5914	11415	hypothetical protein	1	-	-	-	-	-	-	-	-	-	-	-	-	-	-	-	-	-	-	-	-	-	-	-	-	-	-	-	-	-	-	-	-	-	-	-	-	-	-	-	-	-	-	-	-	-	-	-	-	-	-	-	-	-	-	+	-	-
5915	11416	hypothetical protein	1	-	-	-	-	-	-	-	-	-	-	-	-	-	-	-	-	-	-	-	-	-	-	-	-	-	-	-	-	-	-	-	-	-	-	-	-	-	-	-	-	-	-	-	-	-	-	-	-	-	-	-	-	-	-	+	-	-
5916	11417	Cu+ P-type ATPase	1	-	-	-	-	-	-	-	-	-	-	-	-	-	-	-	-	-	-	-	-	-	-	-	-	-	-	-	-	-	-	-	-	-	-	-	-	-	-	-	-	-	-	-	-	-	-	-	-	-	-	-	-	-	-	+	-	-
5917	11418	hypothetical protein	1	-	-	-	-	-	-	-	-	-	-	-	-	-	-	-	-	-	-	-	-	-	-	-	-	-	-	-	-	-	-	-	-	-	-	-	-	-	-	-	-	-	-	-	-	-	-	-	-	-	-	-	-	-	-	+	-	-
5918	11419	hypothetical protein	1	-	-	-	-	-	-	-	-	-	-	-	-	-	-	-	-	-	-	-	-	-	-	-	-	-	-	-	-	-	-	-	-	-	-	-	-	-	-	-	-	-	-	-	-	-	-	-	-	-	-	-	-	-	-	+	-	-
5919	11420	hypothetical protein	1	-	-	-	-	-	-	-	-	-	-	-	-	-	-	-	-	-	-	-	-	-	-	-	-	-	-	-	-	-	-	-	-	-	-	-	-	-	-	-	-	-	-	-	-	-	-	-	-	-	-	-	-	-	-	+	-	-
5920	11421	hypothetical protein	1	-	-	-	-	-	-	-	-	-	-	-	-	-	-	-	-	-	-	-	-	-	-	-	-	-	-	-	-	-	-	-	-	-	-	-	-	-	-	-	-	-	-	-	-	-	-	-	-	-	-	-	-	-	-	+	-	-
5921	11422	hypothetical protein	1	-	-	-	-	-	-	-	-	-	-	-	-	-	-	-	-	-	-	-	-	-	-	-	-	-	-	-	-	-	-	-	-	-	-	-	-	-	-	-	-	-	-	-	-	-	-	-	-	-	-	-	-	-	-	+	-	-
5922	11423	hypothetical protein	1	-	-	-	-	-	-	-	-	-	-	-	-	-	-	-	-	-	-	-	-	-	-	-	-	-	-	-	-	-	-	-	-	-	-	-	-	-	-	-	-	-	-	-	-	-	-	-	-	-	-	-	-	-	-	+	-	-
5923	11424	hypothetical protein	1	-	-	-	-	-	-	-	-	-	-	-	-	-	-	-	-	-	-	-	-	-	-	-	-	-	-	-	-	-	-	-	-	-	-	-	-	-	-	-	-	-	-	-	-	-	-	-	-	-	-	-	-	-	-	+	-	-
5924	11425	hypothetical protein	1	-	-	-	-	-	-	-	-	-	-	-	-	-	-	-	-	-	-	-	-	-	-	-	-	-	-	-	-	-	-	-	-	-	-	-	-	-	-	-	-	-	-	-	-	-	-	-	-	-	-	-	-	-	-	+	-	-
5925	11426	hypothetical protein	1	-	-	-	-	-	-	-	-	-	-	-	-	-	-	-	-	-	-	-	-	-	-	-	-	-	-	-	-	-	-	-	-	-	-	-	-	-	-	-	-	-	-	-	-	-	-	-	-	-	-	-	-	-	-	+	-	-
5926	11427	hypothetical protein	1	-	-	-	-	-	-	-	-	-	-	-	-	-	-	-	-	-	-	-	-	-	-	-	-	-	-	-	-	-	-	-	-	-	-	-	-	-	-	-	-	-	-	-	-	-	-	-	-	-	-	-	-	-	-	+	-	-
5927	11428	hypothetical protein	1	-	-	-	-	-	-	-	-	-	-	-	-	-	-	-	-	-	-	-	-	-	-	-	-	-	-	-	-	-	-	-	-	-	-	-	-	-	-	-	-	-	-	-	-	-	-	-	-	-	-	-	-	-	-	+	-	-
5928	11429	hypothetical protein	1	-	-	-	-	-	-	-	-	-	-	-	-	-	-	-	-	-	-	-	-	-	-	-	-	-	-	-	-	-	-	-	-	-	-	-	-	-	-	-	-	-	-	-	-	-	-	-	-	-	-	-	-	-	-	+	-	-
5929	11430	Hypothetical protein	1	-	-	-	-	-	-	-	-	-	-	-	-	-	-	-	-	-	-	-	-	-	-	-	-	-	-	-	-	-	-	-	-	-	-	-	-	-	-	-	-	-	-	-	-	-	-	-	-	-	-	-	-	-	-	+	-	-
5930	11431	hypothetical protein	1	-	-	-	-	-	-	-	-	-	-	-	-	-	-	-	-	-	-	-	-	-	-	-	-	-	-	-	-	-	-	-	-	-	-	-	-	-	-	-	-	-	-	-	-	-	-	-	-	-	-	-	-	-	-	+	-	-
5931	11432	hypothetical protein	1	-	-	-	-	-	-	-	-	-	-	-	-	-	-	-	-	-	-	-	-	-	-	-	-	-	-	-	-	-	-	-	-	-	-	-	-	-	-	-	-	-	-	-	-	-	-	-	-	-	-	-	-	-	-	+	-	-
5932	11433	hypothetical protein	1	-	-	-	-	-	-	-	-	-	-	-	-	-	-	-	-	-	-	-	-	-	-	-	-	-	-	-	-	-	-	-	-	-	-	-	-	-	-	-	-	-	-	-	-	-	-	-	-	-	-	-	-	-	-	+	-	-
5933	11434	Glycosyl transferase	1	-	-	-	-	-	-	-	-	-	-	-	-	-	-	-	-	-	-	-	-	-	-	-	-	-	-	-	-	-	-	-	-	-	-	-	-	-	-	-	-	-	-	-	-	-	-	-	-	-	-	-	-	-	-	+	-	-
5934	11435	ABC transporter	1	-	-	-	-	-	-	-	-	-	-	-	-	-	-	-	-	-	-	-	-	-	-	-	-	-	-	-	-	-	-	-	-	-	-	-	-	-	-	-	-	-	-	-	-	-	-	-	-	-	-	-	-	-	-	+	-	-
5935	11436	hypothetical protein	1	-	-	-	-	-	-	-	-	-	-	-	-	-	-	-	-	-	-	-	-	-	-	-	-	-	-	-	-	-	-	-	-	-	-	-	-	-	-	-	-	-	-	-	-	-	-	-	-	-	-	-	-	-	-	+	-	-
5936	11437	hypothetical protein	1	-	-	-	-	-	-	-	-	-	-	-	-	-	-	-	-	-	-	-	-	-	-	-	-	-	-	-	-	-	-	-	-	-	-	-	-	-	-	-	-	-	-	-	-	-	-	-	-	-	-	-	-	-	-	+	-	-
5937	11438	hypothetical protein	1	-	-	-	-	-	-	-	-	-	-	-	-	-	-	-	-	-	-	-	-	-	-	-	-	-	-	-	-	-	-	-	-	-	-	-	-	-	-	-	-	-	-	-	-	-	-	-	-	-	-	-	-	-	-	+	-	-
5938	11439	hypothetical protein	1	-	-	-	-	-	-	-	-	-	-	-	-	-	-	-	-	-	-	-	-	-	-	-	-	-	-	-	-	-	-	-	-	-	-	-	-	-	-	-	-	-	-	-	-	-	-	-	-	-	-	-	-	-	-	+	-	-
5939	11440	hypothetical protein	1	-	-	-	-	-	-	-	-	-	-	-	-	-	-	-	-	-	-	-	-	-	-	-	-	-	-	-	-	-	-	-	-	-	-	-	-	-	-	-	-	-	-	-	-	-	-	-	-	-	-	-	-	-	-	+	-	-
5940	11441	hypothetical protein	1	-	-	-	-	-	-	-	-	-	-	-	-	-	-	-	-	-	-	-	-	-	-	-	-	-	-	-	-	-	-	-	-	-	-	-	-	-	-	-	-	-	-	-	-	-	-	-	-	-	-	-	-	-	-	+	-	-
5941	11442	hypothetical protein	1	-	-	-	-	-	-	-	-	-	-	-	-	-	-	-	-	-	-	-	-	-	-	-	-	-	-	-	-	-	-	-	-	-	-	-	-	-	-	-	-	-	-	-	-	-	-	-	-	-	-	-	-	-	-	+	-	-
5942	11443	Sucrose-6-phosphate hydrolase (EC 3.2.1.26)	1	-	-	-	-	-	-	-	-	-	-	-	-	-	-	-	-	-	-	-	-	-	-	-	-	-	-	-	-	-	-	-	-	-	-	-	-	-	-	-	-	-	-	-	-	-	-	-	-	-	-	-	-	-	-	+	-	-
5943	11444	hypothetical protein	1	-	-	-	-	-	-	-	-	-	-	-	-	-	-	-	-	-	-	-	-	-	-	-	-	-	-	-	-	-	-	-	-	-	-	-	-	-	-	-	-	-	-	-	-	-	-	-	-	-	-	-	-	-	-	+	-	-
5944	11445	Beta-1	1	-	-	-	-	-	-	-	-	-	-	-	-	-	-	-	-	-	-	-	-	-	-	-	-	-	-	-	-	-	-	-	-	-	-	-	-	-	-	-	-	-	-	-	-	-	-	-	-	-	-	-	-	-	-	+	-	-
5945	11446	hypothetical protein	1	-	-	-	-	-	-	-	-	-	-	-	-	-	-	-	-	-	-	-	-	-	-	-	-	-	-	-	-	-	-	-	-	-	-	-	-	-	-	-	-	-	-	-	-	-	-	-	-	-	-	-	-	-	-	+	-	-
5946	11447	hypothetical protein	1	-	-	-	-	-	-	-	-	-	-	-	-	-	-	-	-	-	-	-	-	-	-	-	-	-	-	-	-	-	-	-	-	-	-	-	-	-	-	-	-	-	-	-	-	-	-	-	-	-	-	-	-	-	-	+	-	-
5947	11448	hypothetical protein	1	-	-	-	-	-	-	-	-	-	-	-	-	-	-	-	-	-	-	-	-	-	-	-	-	-	-	-	-	-	-	-	-	-	-	-	-	-	-	-	-	-	-	-	-	-	-	-	-	-	-	-	-	-	-	+	-	-
5948	11449	hypothetical protein	1	-	-	-	-	-	-	-	-	-	-	-	-	-	-	-	-	-	-	-	-	-	-	-	-	-	-	-	-	-	-	-	-	-	-	-	-	-	-	-	-	-	-	-	-	-	-	-	-	-	-	-	-	-	-	+	-	-
5949	11450	putative minor structural protein	1	-	-	-	-	-	-	-	-	-	-	-	-	-	-	-	-	-	-	-	-	-	-	-	-	-	-	-	-	-	-	-	-	-	-	-	-	-	-	-	-	-	-	-	-	-	-	-	-	-	-	-	-	-	-	+	-	-
5950	11451	hypothetical protein	1	-	-	-	-	-	-	-	-	-	-	-	-	-	-	-	-	-	-	-	-	-	-	-	-	-	-	-	-	-	-	-	-	-	-	-	-	-	-	-	-	-	-	-	-	-	-	-	-	-	-	-	-	-	-	+	-	-
5951	11452	hypothetical protein	1	-	-	-	-	-	-	-	-	-	-	-	-	-	-	-	-	-	-	-	-	-	-	-	-	-	-	-	-	-	-	-	-	-	-	-	-	-	-	-	-	-	-	-	-	-	-	-	-	-	-	-	-	-	-	+	-	-
5952	11453	hypothetical protein	1	-	-	-	-	-	-	-	-	-	-	-	-	-	-	-	-	-	-	-	-	-	-	-	-	-	-	-	-	-	-	-	-	-	-	-	-	-	-	-	-	-	-	-	-	-	-	-	-	-	-	-	-	-	-	+	-	-
5953	11454	hypothetical protein	1	-	-	-	-	-	-	-	-	-	-	-	-	-	-	-	-	-	-	-	-	-	-	-	-	-	-	-	-	-	-	-	-	-	-	-	-	-	-	-	-	-	-	-	-	-	-	-	-	-	-	-	-	-	-	+	-	-
5954	11455	LPS glycosyltransferase	1	-	-	-	-	-	-	-	-	-	-	-	-	-	-	-	-	-	-	-	-	-	-	-	-	-	-	-	-	-	-	-	-	-	-	-	-	-	-	-	-	-	-	-	-	-	-	-	-	-	-	-	-	-	-	+	-	-
5955	11456	protein of unknown function DUF1535	1	-	-	-	-	-	-	-	-	-	-	-	-	-	-	-	-	-	-	-	-	-	-	-	-	-	-	-	-	-	-	-	-	-	-	-	-	-	-	-	-	-	-	-	-	-	-	-	-	-	-	-	-	-	-	+	-	-
5956	11457	hypothetical protein	1	-	-	-	-	-	-	-	-	-	-	-	-	-	-	-	-	-	-	-	-	-	-	-	-	-	-	-	-	-	-	-	-	-	-	-	-	-	-	-	-	-	-	-	-	-	-	-	-	-	-	-	-	-	-	+	-	-
5957	11458	hypothetical protein	1	-	-	-	-	-	-	-	-	-	-	-	-	-	-	-	-	-	-	-	-	-	-	-	-	-	-	-	-	-	-	-	-	-	-	-	-	-	-	-	-	-	-	-	-	-	-	-	-	-	-	-	-	-	-	+	-	-
5958	11459	hypothetical protein	1	-	-	-	-	-	-	-	-	-	-	-	-	-	-	-	-	-	-	-	-	-	-	-	-	-	-	-	-	-	-	-	-	-	-	-	-	-	-	-	-	-	-	-	-	-	-	-	-	-	-	-	-	-	-	+	-	-
5959	11460	hypothetical protein	1	-	-	-	-	-	-	-	-	-	-	-	-	-	-	-	-	-	-	-	-	-	-	-	-	-	-	-	-	-	-	-	-	-	-	-	-	-	-	-	-	-	-	-	-	-	-	-	-	-	-	-	-	-	-	+	-	-
5960	11461	hypothetical protein	1	-	-	-	-	-	-	-	-	-	-	-	-	-	-	-	-	-	-	-	-	-	-	-	-	-	-	-	-	-	-	-	-	-	-	-	-	-	-	-	-	-	-	-	-	-	-	-	-	-	-	-	-	-	-	+	-	-
5961	11462	hypothetical protein	1	-	-	-	-	-	-	-	-	-	-	-	-	-	-	-	-	-	-	-	-	-	-	-	-	-	-	-	-	-	-	-	-	-	-	-	-	-	-	-	-	-	-	-	-	-	-	-	-	-	-	-	-	-	-	+	-	-
5962	11463	hypothetical protein	1	-	-	-	-	-	-	-	-	-	-	-	-	-	-	-	-	-	-	-	-	-	-	-	-	-	-	-	-	-	-	-	-	-	-	-	-	-	-	-	-	-	-	-	-	-	-	-	-	-	-	-	-	-	-	+	-	-
5963	11464	Type I restriction-modification system	1	-	-	-	-	-	-	-	-	-	-	-	-	-	-	-	-	-	-	-	-	-	-	-	-	-	-	-	-	-	-	-	-	-	-	-	-	-	-	-	-	-	-	-	-	-	-	-	-	-	-	-	-	-	-	+	-	-
5964	11465	Phosphomannomutase	1	-	-	-	-	-	-	-	-	-	-	-	-	-	-	-	-	-	-	-	-	-	-	-	-	-	-	-	-	-	-	-	-	-	-	-	-	-	-	-	-	-	-	-	-	-	-	-	-	-	-	-	-	-	-	+	-	-
5965	11466	hypothetical protein	1	-	-	-	-	-	-	-	-	-	-	-	-	-	-	-	-	-	-	-	-	-	-	-	-	-	-	-	-	-	-	-	-	-	-	-	-	-	-	-	-	-	-	-	-	-	-	-	-	-	-	-	-	-	-	+	-	-
5966	11467	hypothetical protein	1	-	-	-	-	-	-	-	-	-	-	-	-	-	-	-	-	-	-	-	-	-	-	-	-	-	-	-	-	-	-	-	-	-	-	-	-	-	-	-	-	-	-	-	-	-	-	-	-	-	-	-	-	-	-	+	-	-
5967	11468	Membrane protein involved in the export of O-antigen	1	-	-	-	-	-	-	-	-	-	-	-	-	-	-	-	-	-	-	-	-	-	-	-	-	-	-	-	-	-	-	-	-	-	-	-	-	-	-	-	-	-	-	-	-	-	-	-	-	-	-	-	-	-	-	+	-	-
5968	11469	ORF55	1	-	-	-	-	-	-	-	-	-	-	-	-	-	-	-	-	-	-	-	-	-	-	-	-	-	-	-	-	-	-	-	-	-	-	-	-	-	-	-	-	-	-	-	-	-	-	-	-	-	-	-	-	-	-	+	-	-
5969	11470	hypothetical protein	1	-	-	-	-	-	-	-	-	-	-	-	-	-	-	-	-	-	-	-	-	-	-	-	-	-	-	-	-	-	-	-	-	-	-	-	-	-	-	-	-	-	-	-	-	-	-	-	-	-	-	-	-	-	-	+	-	-
5970	11471	ABC-type multidrug transport system (daunorubicin resistance)	1	-	-	-	-	-	-	-	-	-	-	-	-	-	-	-	-	-	-	-	-	-	-	-	-	-	-	-	-	-	-	-	-	-	-	-	-	-	-	-	-	-	-	-	-	-	-	-	-	-	-	-	-	-	-	+	-	-
5971	11472	integral membrane protein PlnU	1	-	-	-	-	-	-	-	-	-	-	-	-	-	-	-	-	-	-	-	-	-	-	-	-	-	-	-	-	-	-	-	-	-	-	-	-	-	-	-	-	-	-	-	-	-	-	-	-	-	-	-	-	-	-	+	-	-
5972	11655	Protein of unknown function DUF975	1	-	-	-	-	-	-	-	-	-	-	-	-	-	-	-	-	-	-	-	-	-	-	-	-	-	-	-	-	-	-	-	-	+	-	-	-	-	-	-	-	-	-	-	-	-	-	-	-	-	-	-	-	-	-	-	-	-
5973	11656	hypothetical protein	1	-	-	-	-	-	-	-	-	-	-	-	-	-	-	-	-	-	-	-	-	-	-	-	-	-	-	-	-	-	-	-	-	+	-	-	-	-	-	-	-	-	-	-	-	-	-	-	-	-	-	-	-	-	-	-	-	-
5974	11657	hypothetical protein	1	-	-	-	-	-	-	-	-	-	-	-	-	-	-	-	-	-	-	-	-	-	-	-	-	-	-	-	-	-	-	-	-	+	-	-	-	-	-	-	-	-	-	-	-	-	-	-	-	-	-	-	-	-	-	-	-	-
5975	11658	glycosyltransferase	1	-	-	-	-	-	-	-	-	-	-	-	-	-	-	-	-	-	-	-	-	-	-	-	-	-	-	-	-	-	-	-	-	+	-	-	-	-	-	-	-	-	-	-	-	-	-	-	-	-	-	-	-	-	-	-	-	-
5976	11659	hypothetical protein	1	-	-	-	-	-	-	-	-	-	-	-	-	-	-	-	-	-	-	-	-	-	-	-	-	-	-	-	-	-	-	-	-	+	-	-	-	-	-	-	-	-	-	-	-	-	-	-	-	-	-	-	-	-	-	-	-	-
5977	11660	Beta-glucosidase (EC 3.2.1.21)	1	-	-	-	-	-	-	-	-	-	-	-	-	-	-	-	-	-	-	-	-	-	-	-	-	-	-	-	-	-	-	-	-	+	-	-	-	-	-	-	-	-	-	-	-	-	-	-	-	-	-	-	-	-	-	-	-	-
5978	11661	hypothetical protein	1	-	-	-	-	-	-	-	-	-	-	-	-	-	-	-	-	-	-	-	-	-	-	-	-	-	-	-	-	-	-	-	-	+	-	-	-	-	-	-	-	-	-	-	-	-	-	-	-	-	-	-	-	-	-	-	-	-
5979	11662	Tagatose-6-phosphate kinase (EC 2.7.1.144) / 1-phosphofructokinase (EC 2.7.1.56)	1	-	-	-	-	-	-	-	-	-	-	-	-	-	-	-	-	-	-	-	-	-	-	-	-	-	-	-	-	-	-	-	-	+	-	-	-	-	-	-	-	-	-	-	-	-	-	-	-	-	-	-	-	-	-	-	-	-
5980	11663	Putative N-acetylgalactosaminyl-diphosphoundecaprenol glucuronosyltransferase	1	-	-	-	-	-	-	-	-	-	-	-	-	-	-	-	-	-	-	-	-	-	-	-	-	-	-	-	-	-	-	-	-	+	-	-	-	-	-	-	-	-	-	-	-	-	-	-	-	-	-	-	-	-	-	-	-	-
5981	11664	Tn5252	1	-	-	-	-	-	-	-	-	-	-	-	-	-	-	-	-	-	-	-	-	-	-	-	-	-	-	-	-	-	-	-	-	+	-	-	-	-	-	-	-	-	-	-	-	-	-	-	-	-	-	-	-	-	-	-	-	-
5982	11665	Membrane protein involved in the export of O-antigen	1	-	-	-	-	-	-	-	-	-	-	-	-	-	-	-	-	-	-	-	-	-	-	-	-	-	-	-	-	-	-	-	-	+	-	-	-	-	-	-	-	-	-	-	-	-	-	-	-	-	-	-	-	-	-	-	-	-
5983	11666	hypothetical protein	1	-	-	-	-	-	-	-	-	-	-	-	-	-	-	-	-	-	-	-	-	-	-	-	-	-	-	-	-	-	-	-	-	+	-	-	-	-	-	-	-	-	-	-	-	-	-	-	-	-	-	-	-	-	-	-	-	-
5984	11667	hypothetical protein	1	-	-	-	-	-	-	-	-	-	-	-	-	-	-	-	-	-	-	-	-	-	-	-	-	-	-	-	-	-	-	-	-	+	-	-	-	-	-	-	-	-	-	-	-	-	-	-	-	-	-	-	-	-	-	-	-	-
5985	11668	Cysteine synthase (EC 2.5.1.47)	1	-	-	-	-	-	-	-	-	-	-	-	-	-	-	-	-	-	-	-	-	-	-	-	-	-	-	-	-	-	-	-	-	+	-	-	-	-	-	-	-	-	-	-	-	-	-	-	-	-	-	-	-	-	-	-	-	-
5986	11669	hypothetical protein	1	-	-	-	-	-	-	-	-	-	-	-	-	-	-	-	-	-	-	-	-	-	-	-	-	-	-	-	-	-	-	-	-	+	-	-	-	-	-	-	-	-	-	-	-	-	-	-	-	-	-	-	-	-	-	-	-	-
5987	11670	Tagatose-6-phosphate kinase (EC 2.7.1.144) / 1-phosphofructokinase (EC 2.7.1.56)	1	-	-	-	-	-	-	-	-	-	-	-	-	-	-	-	-	-	-	-	-	-	-	-	-	-	-	-	-	-	-	-	-	+	-	-	-	-	-	-	-	-	-	-	-	-	-	-	-	-	-	-	-	-	-	-	-	-
5988	11671	hypothetical protein	1	-	-	-	-	-	-	-	-	-	-	-	-	-	-	-	-	-	-	-	-	-	-	-	-	-	-	-	-	-	-	-	-	+	-	-	-	-	-	-	-	-	-	-	-	-	-	-	-	-	-	-	-	-	-	-	-	-
5989	11672	hypothetical protein	1	-	-	-	-	-	-	-	-	-	-	-	-	-	-	-	-	-	-	-	-	-	-	-	-	-	-	-	-	-	-	-	-	+	-	-	-	-	-	-	-	-	-	-	-	-	-	-	-	-	-	-	-	-	-	-	-	-
5990	11673	hypothetical protein	1	-	-	-	-	-	-	-	-	-	-	-	-	-	-	-	-	-	-	-	-	-	-	-	-	-	-	-	-	-	-	-	-	+	-	-	-	-	-	-	-	-	-	-	-	-	-	-	-	-	-	-	-	-	-	-	-	-
5991	11674	hypothetical protein	1	-	-	-	-	-	-	-	-	-	-	-	-	-	-	-	-	-	-	-	-	-	-	-	-	-	-	-	-	-	-	-	-	+	-	-	-	-	-	-	-	-	-	-	-	-	-	-	-	-	-	-	-	-	-	-	-	-
5992	11675	hypothetical protein	1	-	-	-	-	-	-	-	-	-	-	-	-	-	-	-	-	-	-	-	-	-	-	-	-	-	-	-	-	-	-	-	-	+	-	-	-	-	-	-	-	-	-	-	-	-	-	-	-	-	-	-	-	-	-	-	-	-
5993	11676	AraC-type DNA-binding domain-containing protein	1	-	-	-	-	-	-	-	-	-	-	-	-	-	-	-	-	-	-	-	-	-	-	-	-	-	-	-	-	-	-	-	-	+	-	-	-	-	-	-	-	-	-	-	-	-	-	-	-	-	-	-	-	-	-	-	-	-
5994	11677	hypothetical protein	1	-	-	-	-	-	-	-	-	-	-	-	-	-	-	-	-	-	-	-	-	-	-	-	-	-	-	-	-	-	-	-	-	+	-	-	-	-	-	-	-	-	-	-	-	-	-	-	-	-	-	-	-	-	-	-	-	-
5995	11678	hypothetical protein	1	-	-	-	-	-	-	-	-	-	-	-	-	-	-	-	-	-	-	-	-	-	-	-	-	-	-	-	-	-	-	-	-	+	-	-	-	-	-	-	-	-	-	-	-	-	-	-	-	-	-	-	-	-	-	-	-	-
5996	11679	hypothetical protein	1	-	-	-	-	-	-	-	-	-	-	-	-	-	-	-	-	-	-	-	-	-	-	-	-	-	-	-	-	-	-	-	-	+	-	-	-	-	-	-	-	-	-	-	-	-	-	-	-	-	-	-	-	-	-	-	-	-
5997	11680	hypothetical protein	1	-	-	-	-	-	-	-	-	-	-	-	-	-	-	-	-	-	-	-	-	-	-	-	-	-	-	-	-	-	-	-	-	+	-	-	-	-	-	-	-	-	-	-	-	-	-	-	-	-	-	-	-	-	-	-	-	-
5998	11681	Glycosyl transferase	1	-	-	-	-	-	-	-	-	-	-	-	-	-	-	-	-	-	-	-	-	-	-	-	-	-	-	-	-	-	-	-	-	+	-	-	-	-	-	-	-	-	-	-	-	-	-	-	-	-	-	-	-	-	-	-	-	-
5999	11682	hypothetical protein	1	-	-	-	-	-	-	-	-	-	-	-	-	-	-	-	-	-	-	-	-	-	-	-	-	-	-	-	-	-	-	-	-	-	-	-	-	-	-	-	-	-	-	-	-	-	-	-	-	-	+	-	-	-	-	-
6000	11683	membrane-bound protease	1	-	-	-	-	-	-	-	-	-	-	-	-	-	-	-	-	-	-	-	-	-	-	-	-	-	-	-	-	-	-	-	-	-	-	-	-	-	-	-	-	-	-	-	-	-	-	-	+	-	-	-	-	-
6001	11684	hypothetical protein	1	-	-	-	-	-	-	-	-	-	-	-	-	-	-	-	-	-	-	-	-	-	-	-	-	-	-	-	-	-	-	-	-	-	-	-	-	-	-	-	-	-	-	-	-	-	-	-	+	-	-	-	-	-
6002	11685	hypothetical protein	1	-	-	-	-	-	-	-	-	-	-	-	-	-	-	-	-	-	-	-	-	-	-	-	-	-	-	-	-	-	-	-	-	-	-	-	-	-	-	-	-	-	-	-	-	-	-	-	+	-	-	-	-	-
6003	11686	hypothetical protein	1	-	-	-	-	-	-	-	-	-	-	-	-	-	-	-	-	-	-	-	-	-	-	-	-	-	-	-	-	-	-	-	-	-	-	-	-	-	-	-	-	-	-	-	-	-	-	-	+	-	-	-	-	-
6004	11687	hypothetical protein	1	-	-	-	-	-	-	-	-	-	-	-	-	-	-	-	-	-	-	-	-	-	-	-	-	-	-	-	-	-	-	-	-	-	-	-	-	-	-	-	-	-	-	-	-	-	-	-	+	-	-	-	-	-
6005	11688	ABC transporter	1	-	-	-	-	-	-	-	-	-	-	-	-	-	-	-	-	-	-	-	-	-	-	-	-	-	-	-	-	-	-	-	-	-	-	-	-	-	-	-	-	-	-	-	-	-	-	-	+	-	-	-	-	-
6006	11689	hypothetical protein	1	-	-	-	-	-	-	-	-	-	-	-	-	-	-	-	-	-	-	-	-	-	-	-	-	-	-	-	-	-	-	-	-	-	-	-	-	-	-	-	-	-	-	-	-	-	-	-	+	-	-	-	-	-
6007	11690	hypothetical protein	1	-	-	-	-	-	-	-	-	-	-	-	-	-	-	-	-	-	-	-	-	-	-	-	-	-	-	-	-	-	-	-	-	-	-	-	-	-	-	-	-	-	-	-	-	-	-	-	+	-	-	-	-	-
6008	11691	hypothetical protein	1	-	-	-	-	-	-	-	-	-	-	-	-	-	-	-	-	-	-	-	-	-	-	-	-	-	-	-	-	-	-	-	-	-	-	-	-	-	-	-	-	-	-	-	-	-	-	-	+	-	-	-	-	-
6009	11692	hypothetical protein	1	-	-	-	-	-	-	-	-	-	-	-	-	-	-	-	-	-	-	-	-	-	-	-	-	-	-	-	-	-	-	-	-	-	-	-	-	-	-	-	-	-	-	-	-	-	-	-	+	-	-	-	-	-
6010	11693	hypothetical protein	1	-	-	-	-	-	-	-	-	-	-	-	-	-	-	-	-	-	-	-	-	-	-	-	-	-	-	-	-	-	-	-	-	-	-	-	-	-	-	-	-	-	-	-	-	-	-	-	+	-	-	-	-	-
6011	11694	hypothetical protein	1	-	-	-	-	-	-	-	-	-	-	-	-	-	-	-	-	-	-	-	-	-	-	-	-	-	-	-	-	-	-	-	-	-	-	-	-	-	-	-	-	-	-	-	-	-	-	-	+	-	-	-	-	-
6012	11695	hypothetical protein	1	-	-	-	-	-	-	-	-	-	-	-	-	-	-	-	-	-	-	-	-	-	-	-	-	-	-	-	-	-	-	-	-	-	-	-	-	-	-	-	-	-	-	-	-	-	-	-	+	-	-	-	-	-
6013	11696	hypothetical protein	1	-	-	-	-	-	-	-	-	-	-	-	-	-	-	-	-	-	-	-	-	-	-	-	-	-	-	-	-	-	-	-	-	-	-	-	-	-	-	-	-	-	-	-	-	-	-	-	+	-	-	-	-	-
6014	11697	Membrane protein involved in the export of O-antigen	1	-	-	-	-	-	-	-	-	-	-	-	-	-	-	-	-	-	-	-	-	-	-	-	-	-	-	-	-	-	-	-	-	-	-	-	-	-	-	-	-	-	-	-	-	-	-	-	+	-	-	-	-	-
6015	11698	hypothetical protein	1	-	-	-	-	-	-	-	-	-	-	-	-	-	-	-	-	-	-	-	-	-	-	-	-	-	-	-	-	-	-	-	-	-	-	-	-	-	-	-	-	-	-	-	-	-	-	-	+	-	-	-	-	-
6016	11699	NTP pyrophosphohydrolase including oxidative damage repair enzyme	1	-	-	-	-	-	-	-	-	-	-	-	-	-	-	-	-	-	-	-	-	-	-	-	-	-	-	-	-	-	-	-	-	-	-	-	-	-	-	-	-	-	-	-	-	-	-	-	+	-	-	-	-	-
6017	11700	hypothetical protein	1	-	-	-	-	-	-	-	-	-	-	-	-	-	-	-	-	-	-	-	-	-	-	-	-	-	-	-	-	-	-	-	-	-	-	-	-	-	-	-	-	-	-	-	-	-	-	-	+	-	-	-	-	-
6018	11701	hypothetical protein	1	-	-	-	-	-	-	-	-	-	-	-	-	-	-	-	-	-	-	-	-	-	-	-	-	-	-	-	-	-	-	-	-	-	-	-	-	-	-	-	-	-	-	-	-	-	-	-	+	-	-	-	-	-
6019	11702	hypothetical protein	1	-	-	-	-	-	-	-	-	-	-	-	-	-	-	-	-	-	-	-	-	-	-	-	-	-	-	-	-	-	-	-	-	-	-	-	-	-	-	-	-	-	-	-	-	-	-	-	+	-	-	-	-	-
6020	11703	hypothetical protein	1	-	-	-	-	-	-	-	-	-	-	-	-	-	-	-	-	-	-	-	-	-	-	-	-	-	-	-	-	-	-	-	-	-	-	-	-	-	-	-	-	-	-	-	-	-	-	-	+	-	-	-	-	-

Table S5. Precence/absence of each HG among 57 *S. suis* strains

				1																13			25					28						104		94			76			53/54		Divergent *S. suis*																
Index[a]	ClutserID	Gene products	Members	98HAH33	05ZYH33	SC84	A7	SS12	P1/7	DAT273	NIAH11434	JS14	GZ1	BM407	S735	13730	MNCM07	NCTC 10446	14636	NCTC 10237	ST1	2651	89-1591	MNCM04	MNCM25	8074	D9	4961	ST3	MNCM43	DAT292	DAT260	NIAH11435	MNCM21	MNCM50	89-2479	DAT234	DAT290	93A	42A	NT77	6407	11538	8830	12814	92-1400	89-590	2726	2524	89-3576-3	4417	D12	10581	89-5259	88-5299A	R61	92-1191	14A	92-4172	22083
6021	11704	hypothetical protein	1	-	-	-	-	-	-	-	-	-	-	-	-	-	-	-	-	-	-	-	-	-	-	-	-	-	-	-	-	-	-	-	-	-	-	-	-	-	-	-	-	-	-	-	-	-	-	-	-	-	-	-	+	-	-	-	-	-
6022	11705	agglutinin receptor	1	-	-	-	-	-	-	-	-	-	-	-	-	-	-	-	-	-	-	-	-	-	-	-	-	-	-	-	-	-	-	-	-	-	-	-	-	-	-	-	-	-	-	-	-	-	-	-	-	-	-	-	+	-	-	-	-	-
6023	11706	DNA primase	1	-	-	-	-	-	-	-	-	-	-	-	-	-	-	-	-	-	-	-	-	-	-	-	-	-	-	-	-	-	-	-	-	-	-	-	-	-	-	-	-	-	-	-	-	-	-	-	-	-	-	-	+	-	-	-	-	-
6024	11707	hypothetical protein	1	-	-	-	-	-	-	-	-	-	-	-	-	-	-	-	-	-	-	-	-	-	-	-	-	-	-	-	-	-	-	-	-	-	-	-	-	-	-	-	-	-	-	-	-	-	-	-	-	-	-	-	+	-	-	-	-	-
6025	11708	hypothetical protein	1	-	-	-	-	-	-	-	-	-	-	-	-	-	-	-	-	-	-	-	-	-	-	-	-	-	-	-	-	-	-	-	-	-	-	-	-	-	-	-	-	-	-	-	-	-	-	-	-	-	-	-	+	-	-	-	-	-
6026	11709	hypothetical protein	1	-	-	-	-	-	-	-	-	-	-	-	-	-	-	-	-	-	-	-	-	-	-	-	-	-	-	-	-	-	-	-	-	-	-	-	-	-	-	-	-	-	-	-	-	-	-	-	-	-	-	-	+	-	-	-	-	-
6027	11710	hypothetical protein	1	-	-	-	-	-	-	-	-	-	-	-	-	-	-	-	-	-	-	-	-	-	-	-	-	-	-	-	-	-	-	-	-	-	-	-	-	-	-	-	-	-	-	-	-	-	-	-	-	-	-	-	+	-	-	-	-	-
6028	11711	hypothetical protein	1	-	-	-	-	-	-	-	-	-	-	-	-	-	-	-	-	-	-	-	-	-	-	-	-	-	-	-	-	-	-	-	-	-	-	-	-	-	-	-	-	-	-	-	-	-	-	-	-	-	-	-	+	-	-	-	-	-
6029	11712	hypothetical protein	1	-	-	-	-	-	-	-	-	-	-	-	-	-	-	-	-	-	-	-	-	-	-	-	-	-	-	-	-	-	-	-	-	-	-	-	-	-	-	-	-	-	-	-	-	-	-	-	-	-	-	-	+	-	-	-	-	-
6030	11713	Helicase loader DnaI	1	-	-	-	-	-	-	-	-	-	-	-	-	-	-	-	-	-	-	-	-	-	-	-	-	-	-	-	-	-	-	-	-	-	-	-	-	-	-	-	-	-	-	-	-	-	-	-	-	-	-	-	+	-	-	-	-	-
6031	11714	hypothetical protein	1	-	-	-	-	-	-	-	-	-	-	-	-	-	-	-	-	-	-	-	-	-	-	-	-	-	-	-	-	-	-	-	-	-	-	-	-	-	-	-	-	-	-	-	-	-	-	-	-	-	-	-	+	-	-	-	-	-
6032	11715	Poly(glycerol-phosphate) alpha-glucosyltransferase GftA (EC 2.4.1.52)	1	-	-	-	-	-	-	-	-	-	-	-	-	-	-	-	-	-	-	-	-	-	-	-	-	-	-	-	-	-	-	-	-	-	-	-	-	-	-	-	-	-	-	-	-	-	-	-	-	-	-	-	+	-	-	-	-	-
6033	11716	hypothetical protein	1	-	-	-	-	-	-	-	-	-	-	-	-	-	-	-	-	-	-	-	-	-	-	-	-	-	-	-	-	-	-	-	-	-	-	-	-	-	-	-	-	-	-	-	-	-	-	-	-	-	-	-	+	-	-	-	-	-
6034	11717	putative O-acetyl transferase	1	-	-	-	-	-	-	-	-	-	-	-	-	-	-	-	-	-	-	-	-	-	-	-	-	-	-	-	-	-	-	-	-	-	-	-	-	-	-	-	-	-	-	-	-	-	-	-	-	-	-	-	+	-	-	-	-	-
6035	11718	Choline binding protein D	1	-	-	-	-	-	-	-	-	-	-	-	-	-	-	-	-	-	-	-	-	-	-	-	-	-	-	-	-	-	-	-	-	-	-	-	-	-	-	-	-	-	-	-	-	-	-	-	-	-	-	-	+	-	-	-	-	-
6036	11719	hypothetical protein	1	-	-	-	-	-	-	-	-	-	-	-	-	-	-	-	-	-	-	-	-	-	-	-	-	-	-	-	-	-	-	-	-	-	-	-	-	-	-	-	-	-	-	-	-	-	-	-	-	-	-	-	+	-	-	-	-	-
6037	11720	hypothetical protein	1	-	-	-	-	-	-	-	-	-	-	-	-	-	-	-	-	-	-	-	-	-	-	-	-	-	-	-	-	-	-	-	-	-	-	-	-	-	-	-	-	-	-	-	-	-	-	-	-	-	-	-	+	-	-	-	-	-
6038	11721	hypothetical protein	1	-	-	-	-	-	-	-	-	-	-	-	-	-	-	-	-	-	-	-	-	-	-	-	-	-	-	-	-	-	-	-	-	-	-	-	-	-	-	-	-	-	-	-	-	-	-	-	-	-	-	-	+	-	-	-	-	-
6039	11722	hypothetical protein	1	-	-	-	-	-	-	-	-	-	-	-	-	-	-	-	-	-	-	-	-	-	-	-	-	-	-	-	-	-	-	-	-	-	-	-	-	-	-	-	-	-	-	-	-	-	-	-	-	-	-	-	+	-	-	-	-	-
6040	11723	hypothetical protein	1	-	-	-	-	-	-	-	-	-	-	-	-	-	-	-	-	-	-	-	-	-	-	-	-	-	-	-	-	-	-	-	-	-	-	-	-	-	-	-	-	-	-	-	-	-	-	-	-	-	-	-	+	-	-	-	-	-
6041	11724	Mobile element protein	1	-	-	-	-	-	-	-	-	-	-	-	-	-	-	-	-	-	-	-	-	-	-	-	-	-	-	-	-	-	-	-	-	-	-	-	-	-	-	-	-	-	-	-	-	-	-	-	-	-	-	-	+	-	-	-	-	-
6042	11725	hypothetical protein	1	-	-	-	-	-	-	-	-	-	-	-	-	-	-	-	-	-	-	-	-	-	-	-	-	-	-	-	-	-	-	-	-	-	-	-	-	-	-	-	-	-	-	-	-	-	-	-	-	-	-	-	+	-	-	-	-	-
6043	11726	ABC-type transport systems	1	-	-	-	-	-	-	-	-	-	-	-	-	-	-	-	-	-	-	-	-	-	-	-	-	-	-	-	-	-	-	-	-	-	-	-	-	-	-	-	-	-	-	-	-	-	-	-	-	-	-	-	+	-	-	-	-	-
6044	11727	Mu-like prophage protein gp29	1	-	-	-	-	-	-	-	-	-	-	-	-	-	-	-	-	-	-	-	-	-	-	-	-	-	-	-	-	-	-	-	-	-	-	-	-	-	-	-	-	-	-	-	-	-	-	-	-	-	-	-	+	-	-	-	-	-
6045	11728	hypothetical protein	1	-	-	-	-	-	-	-	-	-	-	-	-	-	-	-	-	-	-	-	-	-	-	-	-	-	-	-	-	-	-	-	-	-	-	-	-	-	-	-	-	-	-	-	-	-	-	-	-	-	-	-	+	-	-	-	-	-
6046	11729	hypothetical protein	1	-	-	-	-	-	-	-	-	-	-	-	-	-	-	-	-	-	-	-	-	-	-	-	-	-	-	-	-	-	-	-	-	-	-	-	-	-	-	-	-	-	-	-	-	-	-	-	-	-	-	-	+	-	-	-	-	-
6047	11730	hypothetical protein	1	-	-	-	-	-	-	-	-	-	-	-	-	-	-	-	-	-	-	-	-	-	-	-	-	-	-	-	-	-	-	-	-	-	-	-	-	-	-	-	-	-	-	-	-	-	-	-	-	-	-	-	+	-	-	-	-	-
6048	11731	Muramidase-released protein precursor (136 kDa surface protein)	1	-	-	-	-	-	-	-	-	-	-	-	-	-	-	-	-	-	-	-	-	-	-	-	-	-	-	-	-	-	-	-	-	-	-	-	-	-	-	-	-	-	-	-	-	-	-	-	-	-	-	-	+	-	-	-	-	-
6049	11732	hypothetical protein	1	-	-	-	-	-	-	-	-	-	-	-	-	-	-	-	-	-	-	-	-	-	-	-	-	-	-	-	-	-	-	-	-	-	-	-	-	-	-	-	-	-	-	-	-	-	-	-	-	-	-	-	+	-	-	-	-	-
6050	11733	hypothetical protein	1	-	-	-	-	-	-	-	-	-	-	-	-	-	-	-	-	-	-	-	-	-	-	-	-	-	-	-	-	-	-	-	-	-	-	-	-	-	-	-	-	-	-	-	-	-	-	-	-	-	-	-	+	-	-	-	-	-
6051	11734	acetyltransferase	1	-	-	-	-	-	-	-	-	-	-	-	-	-	-	-	-	-	-	-	-	-	-	-	-	-	-	-	-	-	-	-	-	-	-	-	-	-	-	-	-	-	-	-	-	-	-	-	-	-	-	-	+	-	-	-	-	-
6052	11735	Alpha-glycerophosphate oxidase (EC 1.1.3.21)	1	-	-	-	-	-	-	-	-	-	-	-	-	-	-	-	-	-	-	-	-	-	-	-	-	-	-	-	-	-	-	-	-	-	-	-	-	-	-	-	-	-	-	-	-	-	-	-	-	-	-	-	+	-	-	-	-	-
6053	11736	hypothetical protein	1	-	-	-	-	-	-	-	-	-	-	-	-	-	-	-	-	-	-	-	-	-	-	-	-	-	-	-	-	-	-	-	-	-	-	-	-	-	-	-	-	-	-	-	-	-	-	-	-	-	-	-	+	-	-	-	-	-
6054	11737	maltose O-acetyltransferase(EC:2.3.1.79)	1	-	-	-	-	-	-	-	-	-	-	-	-	-	-	-	-	-	-	-	-	-	-	-	-	-	-	-	-	-	-	-	-	-	-	-	-	-	-	-	-	-	-	-	-	-	-	-	-	-	-	-	+	-	-	-	-	-
6055	11738	hypothetical protein	1	-	-	-	-	-	-	-	-	-	-	-	-	-	-	-	-	-	-	-	-	-	-	-	-	-	-	-	-	-	-	-	-	-	-	-	-	-	-	-	-	-	-	-	-	-	-	-	-	-	-	-	+	-	-	-	-	-
6056	11739	hypothetical protein	1	-	-	-	-	-	-	-	-	-	-	-	-	-	-	-	-	-	-	-	-	-	-	-	-	-	-	-	-	-	-	-	-	-	-	-	-	-	-	-	-	-	-	-	-	-	-	-	-	-	-	-	+	-	-	-	-	-
6057	11740	hypothetical protein	1	-	-	-	-	-	-	-	-	-	-	-	-	-	-	-	-	-	-	-	-	-	-	-	-	-	-	-	-	-	-	-	-	-	-	-	-	-	-	-	-	-	-	-	-	-	-	-	-	-	-	-	+	-	-	-	-	-
6058	11741	hypothetical protein	1	-	-	-	-	-	-	-	-	-	-	-	-	-	-	-	-	-	-	-	-	-	-	-	-	-	-	-	-	-	-	-	-	-	-	-	-	-	-	-	-	-	-	-	-	-	-	-	-	-	-	-	+	-	-	-	-	-
6059	11742	hypothetical protein	1	-	-	-	-	-	-	-	-	-	-	-	-	-	-	-	-	-	-	-	-	-	-	-	-	-	-	-	-	-	-	-	-	-	-	-	-	-	-	-	-	-	-	-	-	-	-	-	-	-	-	-	+	-	-	-	-	-
6060	11743	Lin2574 protein	1	-	-	-	-	-	-	-	-	-	-	-	-	-	-	-	-	-	-	-	-	-	-	-	-	-	-	-	-	-	-	-	-	-	-	-	-	-	-	-	-	-	-	-	-	-	-	-	-	-	-	-	+	-	-	-	-	-
6061	11744	hypothetical protein	1	-	-	-	-	-	-	-	-	-	-	-	-	-	-	-	-	-	-	-	-	-	-	-	-	-	-	-	-	-	-	-	-	-	-	-	-	-	-	-	-	-	-	-	-	-	-	-	-	-	-	-	+	-	-	-	-	-
6062	11745	hypothetical protein	1	-	-	-	-	-	-	-	-	-	-	-	-	-	-	-	-	-	-	-	-	-	-	-	-	-	-	-	-	-	-	-	-	-	-	-	-	-	-	-	-	-	-	-	-	-	-	-	-	-	-	-	+	-	-	-	-	-
6063	11746	hypothetical protein	1	-	-	-	-	-	-	-	-	-	-	-	-	-	-	-	-	-	-	-	-	-	-	-	-	-	-	-	-	-	-	-	-	-	-	-	-	-	-	-	-	-	-	-	-	-	-	-	-	-	-	-	+	-	-	-	-	-
6064	11747	hypothetical protein	1	-	-	-	-	-	-	-	-	-	-	-	-	-	-	-	-	-	-	-	-	-	-	-	-	-	-	-	-	-	-	-	-	-	-	-	-	-	-	-	-	-	-	-	-	-	-	-	-	-	-	-	+	-	-	-	-	-
6065	11748	DNA helicase II(EC:3.6.1.-)	1	-	-	-	-	-	-	-	-	-	-	-	-	-	-	-	-	-	-	-	-	-	-	-	-	-	-	-	-	-	-	-	-	-	-	-	-	-	-	-	-	-	-	-	-	-	-	-	-	-	-	-	+	-	-	-	-	-
6066	11749	N-Acetyl-D-glucosamine ABC transport system	1	-	-	-	-	-	-	-	-	-	-	-	-	-	-	-	-	-	-	-	-	-	-	-	-	-	-	-	-	-	-	-	-	-	-	-	-	-	-	-	-	-	-	-	-	-	-	-	-	-	-	-	+	-	-	-	-	-
6067	11750	hypothetical protein	1	-	-	-	-	-	-	-	-	-	-	-	-	-	-	-	-	-	-	-	-	-	-	-	-	-	-	-	-	-	-	-	-	-	-	-	-	-	-	-	-	-	-	-	-	-	-	-	-	-	-	-	+	-	-	-	-	-
6068	11751	hypothetical protein	1	-	-	-	-	-	-	-	-	-	-	-	-	-	-	-	-	-	-	-	-	-	-	-	-	-	-	-	-	-	-	-	-	-	-	-	-	-	-	-	-	-	-	-	-	-	-	-	-	-	-	-	+	-	-	-	-	-
6069	11752	hypothetical protein	1	-	-	-	-	-	-	-	-	-	-	-	-	-	-	-	-	-	-	-	-	-	-	-	-	-	-	-	-	-	-	-	-	-	-	-	-	-	-	-	-	-	-	-	-	-	-	-	-	-	-	-	+	-	-	-	-	-
6070	11753	hypothetical protein	1	-	-	-	-	-	-	-	-	-	-	-	-	-	-	-	-	-	-	-	-	-	-	-	-	-	-	-	-	-	-	-	-	-	-	-	-	-	-	-	-	-	-	-	-	-	-	-	-	-	-	-	+	-	-	-	-	-
6071	11754	Alpha-L-Rha alpha-1	1	-	-	-	-	-	-	-	-	-	-	-	-	-	-	-	-	-	-	-	-	-	-	-	-	-	-	-	-	-	-	-	-	-	-	-	-	-	-	-	-	-	-	-	-	-	-	-	-	-	-	-	+	-	-	-	-	-
6072	11755	MutT/NudX family protein (putative)	1	-	-	-	-	-	-	-	-	-	-	-	-	-	-	-	-	-	-	-	-	-	-	-	-	-	-	-	-	-	-	-	-	-	-	-	-	-	-	-	-	-	-	-	-	-	-	-	-	-	-	-	+	-	-	-	-	-
6073	11756	N-Acetylneuraminate cytidylyltransferase (EC 2.7.7.43)	1	-	-	-	-	-	-	-	-	-	-	-	-	-	-	-	-	-	-	-	-	-	-	-	-	-	-	-	-	-	-	-	-	-	-	-	-	-	-	-	-	-	-	-	-	-	-	-	-	-	-	-	+	-	-	-	-	-
6074	11757	radical SAM domain protein	1	-	-	-	-	-	-	-	-	-	-	-	-	-	-	-	-	-	-	-	-	-	-	-	-	-	-	-	-	-	-	-	-	-	-	-	-	-	-	-	-	-	-	-	-	-	-	-	-	-	-	-	+	-	-	-	-	-
6075	11758	lipoprotein	1	-	-	-	-	-	-	-	-	-	-	-	-	-	-	-	-	-	-	-	-	-	-	-	-	-	-	-	-	-	-	-	-	-	-	-	-	-	-	-	-	-	-	-	-	-	-	-	-	-	-	-	+	-	-	-	-	-
6076	11759	hypothetical protein	1	-	-	-	-	-	-	-	-	-	-	-	-	-	-	-	-	-	-	-	-	-	-	-	-	-	-	-	-	-	-	-	-	-	-	-	-	-	-	-	-	-	-	-	-	-	-	-	-	-	-	-	+	-	-	-	-	-
6077	11760	hypothetical protein	1	-	-	-	-	-	-	-	-	-	-	-	-	-	-	-	-	-	-	-	-	-	-	-	-	-	-	-	-	-	-	-	-	-	-	-	-	-	-	-	-	-	-	-	-	-	-	-	-	-	-	-	+	-	-	-	-	-
6078	11761	hypothetical protein	1	-	-	-	-	-	-	-	-	-	-	-	-	-	-	-	-	-	-	-	-	-	-	-	-	-	-	-	-	-	-	-	-	-	-	-	-	-	-	-	-	-	-	-	-	-	-	-	-	-	-	-	+	-	-	-	-	-
6079	11762	hypothetical protein	1	-	-	-	-	-	-	-	-	-	-	-	-	-	-	-	-	-	-	-	-	-	-	-	-	-	-	-	-	-	-	-	-	-	-	-	-	-	-	-	-	-	-	-	-	-	-	-	-	-	-	-	+	-	-	-	-	-
6080	11763	hypothetical protein	1	-	-	-	-	-	-	-	-	-	-	-	-	-	-	-	-	-	-	-	-	-	-	-	-	-	-	-	-	-	-	-	-	-	-	-	-	-	-	-	-	-	-	-	-	-	-	-	-	-	-	-	+	-	-	-	-	-
6081	11764	hypothetical protein	1	-	-	-	-	-	-	-	-	-	-	-	-	-	-	-	-	-	-	-	-	-	-	-	-	-	-	-	-	-	-	-	-	-	-	-	-	-	-	-	-	-	-	-	-	-	-	-	-	-	-	-	+	-	-	-	-	-
6082	11765	hypothetical protein	1	-	-	-	-	-	-	-	-	-	-	-	-	-	-	-	-	-	-	-	-	-	-	-	-	-	-	-	-	-	-	-	-	-	-	-	-	-	-	-	-	-	-	-	-	-	-	-	-	-	-	-	+	-	-	-	-	-
6083	11766	hypothetical protein	1	-	-	-	-	-	-	-	-	-	-	-	-	-	-	-	-	-	-	-	-	-	-	-	-	-	-	-	-	-	-	-	-	-	-	-	-	-	-	-	-	-	-	-	-	-	-	-	-	-	-	-	+	-	-	-	-	-
6084	11767	CMP-N-acetylneuraminate-beta-galactosamide-alpha-2	1	-	-	-	-	-	-	-	-	-	-	-	-	-	-	-	-	-	-	-	-	-	-	-	-	-	-	-	-	-	-	-	-	-	-	-	-	-	-	-	-	-	-	-	-	-	-	-	-	-	-	-	+	-	-	-	-	-
6085	11768	hypothetical protein	1	-	-	-	-	-	-	-	-	-	-	-	-	-	-	-	-	-	-	-	-	-	-	-	-	-	-	-	-	-	-	-	-	-	-	-	-	-	-	-	-	-	-	-	-	-	-	-	-	-	-	-	+	-	-	-	-	-
6086	11769	hypothetical protein	1	-	-	-	-	-	-	-	-	-	-	-	-	-	-	-	-	-	-	-	-	-	-	-	-	-	-	-	-	-	-	-	-	-	-	-	-	-	-	-	-	-	-	-	-	-	-	-	-	-	-	-	+	-	-	-	-	-
6087	11770	hypothetical protein	1	-	-	-	-	-	-	-	-	-	-	-	-	-	-	-	-	-	-	-	-	-	-	-	-	-	-	-	-	-	-	-	-	-	-	-	-	-	-	-	-	-	-	-	-	-	-	-	-	-	-	-	+	-	-	-	-	-
6088	11771	hypothetical protein	1	-	-	-	-	-	-	-	-	-	-	-	-	-	-	-	-	-	-	-	-	-	-	-	-	-	-	-	-	-	-	-	-	-	-	-	-	-	-	-	-	-	-	-	-	-	-	-	-	-	-	-	+	-	-	-	-	-
6089	11772	UPF0236 protein in vanSb 3'region	1	-	-	-	-	-	-	-	-	-	-	-	-	-	-	-	-	-	-	-	-	-	-	-	-	-	-	-	-	-	-	-	-	-	-	-	-	-	-	-	-	-	-	-	-	-	-	-	-	-	-	-	+	-	-	-	-	-
6090	11773	Type I restriction-modification system	1	-	-	-	-	-	-	-	-	-	-	-	-	-	-	-	-	-	-	-	-	-	-	-	-	-	-	-	-	-	-	-	-	-	-	-	-	-	-	-	-	-	-	-	-	-	-	-	-	-	-	-	+	-	-	-	-	-
6091	11774	hypothetical protein	1	-	-	-	-	-	-	-	-	-	-	-	-	-	-	-	-	-	-	-	-	-	-	-	-	-	-	-	-	-	-	-	-	-	-	-	-	-	-	-	-	-	-	-	-	-	-	-	-	-	-	-	+	-	-	-	-	-
6092	11775	agglutinin receptor	1	-	-	-	-	-	-	-	-	-	-	-	-	-	-	-	-	-	-	-	-	-	-	-	-	-	-	-	-	-	-	-	-	-	-	-	-	-	-	-	-	-	-	-	-	-	-	-	-	-	-	-	+	-	-	-	-	-
6093	11776	hypothetical protein	1	-	-	-	-	-	-	-	-	-	-	-	-	-	-	-	-	-	-	-	-	-	-	-	-	-	-	-	-	-	-	-	-	-	-	-	-	-	-	-	-	-	-	-	-	-	-	-	-	-	-	-	+	-	-	-	-	-
6094	11777	hypothetical protein	1	-	-	-	-	-	-	-	-	-	-	-	-	-	-	-	-	-	-	-	-	-	-	-	-	-	-	-	-	-	-	-	-	-	-	-	-	-	-	-	-	-	-	-	-	-	-	-	-	-	-	-	+	-	-	-	-	-
6095	11778	hypothetical protein	1	-	-	-	-	-	-	-	-	-	-	-	-	-	-	-	-	-	-	-	-	-	-	-	-	-	-	-	-	-	-	-	-	-	-	-	-	-	-	-	-	-	-	-	-	-	-	-	-	-	-	-	+	-	-	-	-	-
6096	11779	hypothetical protein	1	-	-	-	-	-	-	-	-	-	-	-	-	-	-	-	-	-	-	-	-	-	-	-	-	-	-	-	-	-	-	-	-	-	-	-	-	-	-	-	-	-	-	-	-	-	-	-	-	-	-	-	+	-	-	-	-	-
6097	11780	hypothetical protein	1	-	-	-	-	-	-	-	-	-	-	-	-	-	-	-	-	-	-	-	-	-	-	-	-	-	-	-	-	-	-	-	-	-	-	-	-	-	-	-	-	-	-	+	-	-	-	-	-	-	-	-	-	-
6098	11781	glycosyl transferase	1	-	-	-	-	-	-	-	-	-	-	-	-	-	-	-	-	-	-	-	-	-	-	-	-	-	-	-	-	-	-	-	-	-	-	-	-	-	-	-	-	-	-	+	-	-	-	-	-	-	-	-	-	-
6099	11782	putative scaffolding protein	1	-	-	-	-	-	-	-	-	-	-	-	-	-	-	-	-	-	-	-	-	-	-	-	-	-	-	-	-	-	-	-	-	-	-	-	-	-	-	-	-	-	-	+	-	-	-	-	-	-	-	-	-	-
6100	11783	hypothetical protein	1	-	-	-	-	-	-	-	-	-	-	-	-	-	-	-	-	-	-	-	-	-	-	-	-	-	-	-	-	-	-	-	-	-	-	-	-	-	-	-	-	-	-	+	-	-	-	-	-	-	-	-	-	-
6101	11784	hypothetical protein	1	-	-	-	-	-	-	-	-	-	-	-	-	-	-	-	-	-	-	-	-	-	-	-	-	-	-	-	-	-	-	-	-	-	-	-	-	-	-	-	-	-	-	+	-	-	-	-	-	-	-	-	-	-
6102	11785	EpsJ	1	-	-	-	-	-	-	-	-	-	-	-	-	-	-	-	-	-	-	-	-	-	-	-	-	-	-	-	-	-	-	-	-	-	-	-	-	-	-	-	-	-	-	+	-	-	-	-	-	-	-	-	-	-
6103	11786	hypothetical protein	1	-	-	-	-	-	-	-	-	-	-	-	-	-	-	-	-	-	-	-	-	-	-	-	-	-	-	-	-	-	-	-	-	-	-	-	-	-	-	-	-	-	-	+	-	-	-	-	-	-	-	-	-	-
6104	11787	hypothetical protein	1	-	-	-	-	-	-	-	-	-	-	-	-	-	-	-	-	-	-	-	-	-	-	-	-	-	-	-	-	-	-	-	-	-	-	-	-	-	-	-	-	-	-	+	-	-	-	-	-	-	-	-	-	-
6105	11788	hypothetical protein	1	-	-	-	-	-	-	-	-	-	-	-	-	-	-	-	-	-	-	-	-	-	-	-	-	-	-	-	-	-	-	-	-	-	-	-	-	-	-	-	-	-	-	+	-	-	-	-	-	-	-	-	-	-
6106	11789	hypothetical protein	1	-	-	-	-	-	-	-	-	-	-	-	-	-	-	-	-	-	-	-	-	-	-	-	-	-	-	-	-	-	-	-	-	-	-	-	-	-	-	-	-	-	-	+	-	-	-	-	-	-	-	-	-	-
6107	11790	hypothetical protein	1	-	-	-	-	-	-	-	-	-	-	-	-	-	-	-	-	-	-	-	-	-	-	-	-	-	-	-	-	-	-	-	-	-	-	-	-	-	-	-	-	-	-	+	-	-	-	-	-	-	-	-	-	-
6108	11791	Predicted lactoylglutathione lyase	1	-	-	-	-	-	-	-	-	-	-	-	-	-	-	-	-	-	-	-	-	-	-	-	-	-	-	-	-	-	-	-	-	-	-	-	-	-	-	-	-	-	-	+	-	-	-	-	-	-	-	-	-	-
6109	11792	hypothetical protein	1	-	-	-	-	-	-	-	-	-	-	-	-	-	-	-	-	-	-	-	-	-	-	-	-	-	-	-	-	-	-	-	-	-	-	-	-	-	-	-	-	-	-	+	-	-	-	-	-	-	-	-	-	-
6110	11793	hypothetical protein	1	-	-	-	-	-	-	-	-	-	-	-	-	-	-	-	-	-	-	-	-	-	-	-	-	-	-	-	-	-	-	-	-	-	-	-	-	-	-	-	-	-	-	+	-	-	-	-	-	-	-	-	-	-
6111	11794	hypothetical protein	1	-	-	-	-	-	-	-	-	-	-	-	-	-	-	-	-	-	-	-	-	-	-	-	-	-	-	-	-	-	-	-	-	-	-	-	-	-	-	-	-	-	-	+	-	-	-	-	-	-	-	-	-	-
6112	11795	hypothetical protein	1	-	-	-	-	-	-	-	-	-	-	-	-	-	-	-	-	-	-	-	-	-	-	-	-	-	-	-	-	-	-	-	-	-	-	-	-	-	-	-	-	-	-	+	-	-	-	-	-	-	-	-	-	-
6113	11796	minor capsid protein	1	-	-	-	-	-	-	-	-	-	-	-	-	-	-	-	-	-	-	-	-	-	-	-	-	-	-	-	-	-	-	-	-	-	-	-	-	-	-	-	-	-	-	+	-	-	-	-	-	-	-	-	-	-
6114	11797	hypothetical protein	1	-	-	-	-	-	-	-	-	-	-	-	-	-	-	-	-	-	-	-	-	-	-	-	-	-	-	-	-	-	-	-	-	-	-	-	-	-	-	-	-	-	-	+	-	-	-	-	-	-	-	-	-	-
6115	11798	minor capsid protein	1	-	-	-	-	-	-	-	-	-	-	-	-	-	-	-	-	-	-	-	-	-	-	-	-	-	-	-	-	-	-	-	-	-	-	-	-	-	-	-	-	-	-	+	-	-	-	-	-	-	-	-	-	-
6116	11799	oligosaccharide repeat unit polymerase Wzy	1	-	-	-	-	-	-	-	-	-	-	-	-	-	-	-	-	-	-	-	-	-	-	-	-	-	-	-	-	-	-	-	-	-	-	-	-	-	-	-	-	-	-	+	-	-	-	-	-	-	-	-	-	-
6117	11800	hypothetical protein	1	-	-	-	-	-	-	-	-	-	-	-	-	-	-	-	-	-	-	-	-	-	-	-	-	-	-	-	-	-	-	-	-	-	-	-	-	-	-	-	-	-	-	+	-	-	-	-	-	-	-	-	-	-
6118	11801	Serine acetyltransferase (EC 2.3.1.30)	1	-	-	-	-	-	-	-	-	-	-	-	-	-	-	-	-	-	-	-	-	-	-	-	-	-	-	-	-	-	-	-	-	-	-	-	-	-	-	-	-	-	-	+	-	-	-	-	-	-	-	-	-	-
6119	11802	hypothetical protein	1	-	-	-	-	-	-	-	-	-	-	-	-	-	-	-	-	-	-	-	-	-	-	-	-	-	-	-	-	-	-	-	-	-	-	-	-	-	-	-	-	-	-	+	-	-	-	-	-	-	-	-	-	-
6120	11803	hypothetical protein	1	-	-	-	-	-	-	-	-	-	-	-	-	-	-	-	-	-	-	-	-	-	-	-	-	-	-	-	-	-	-	-	-	-	-	-	-	-	-	-	-	-	-	+	-	-	-	-	-	-	-	-	-	-
6121	11804	hypothetical protein	1	-	-	-	-	-	-	-	-	-	-	-	-	-	-	-	-	-	-	-	-	-	-	-	-	-	-	-	-	-	-	-	-	-	-	-	-	-	-	-	-	-	-	+	-	-	-	-	-	-	-	-	-	-
6122	11805	Putative JHP540-like protein	1	-	-	-	-	-	-	-	-	-	-	-	-	-	-	-	-	-	-	-	-	-	-	-	-	-	-	-	-	-	-	-	-	-	-	-	-	-	-	-	-	-	-	+	-	-	-	-	-	-	-	-	-	-
6123	11806	hypothetical protein	1	-	-	-	-	-	-	-	-	-	-	-	-	-	-	-	-	-	-	-	-	-	-	-	-	-	-	-	-	-	-	-	-	-	-	-	-	-	-	-	-	-	-	+	-	-	-	-	-	-	-	-	-	-
6124	11807	hypothetical protein	1	-	-	-	-	-	-	-	-	-	-	-	-	-	-	-	-	-	-	-	-	-	-	-	-	-	-	-	-	-	-	-	-	-	-	-	-	-	-	-	-	-	-	+	-	-	-	-	-	-	-	-	-	-
6125	11808	hypothetical protein	1	-	-	-	-	-	-	-	-	-	-	-	-	-	-	-	-	-	-	-	-	-	-	-	-	-	-	-	-	-	-	-	-	-	-	-	-	-	-	-	-	-	-	+	-	-	-	-	-	-	-	-	-	-
6126	11809	hypothetical protein	1	-	-	-	-	-	-	-	-	-	-	-	-	-	-	-	-	-	-	-	-	-	-	-	-	-	-	-	-	-	-	-	-	-	-	-	-	-	-	-	-	-	-	+	-	-	-	-	-	-	-	-	-	-
6127	11810	hypothetical protein	1	-	-	-	-	-	-	-	-	-	-	-	-	-	-	-	-	-	-	-	-	-	-	-	-	-	-	-	-	-	-	-	-	-	-	-	-	-	-	-	-	-	-	+	-	-	-	-	-	-	-	-	-	-
6128	11811	hypothetical protein	1	-	-	-	-	-	-	-	-	-	-	-	-	-	-	-	-	-	-	-	-	-	-	-	-	-	-	-	-	-	-	-	-	-	-	-	-	-	-	-	-	-	-	+	-	-	-	-	-	-	-	-	-	-
6129	11812	hypothetical protein	1	-	-	-	-	-	-	-	-	-	-	-	-	-	-	-	-	-	-	-	-	-	-	-	-	-	-	-	-	-	-	-	-	-	-	-	-	-	-	-	-	-	-	+	-	-	-	-	-	-	-	-	-	-
6130	11813	Type I restriction-modification system	1	-	-	-	-	-	-	-	-	-	-	-	-	-	-	-	-	-	-	-	-	-	-	-	-	-	-	-	-	-	-	-	-	-	-	-	-	-	-	-	-	-	-	+	-	-	-	-	-	-	-	-	-	-
6131	11814	Beta-1	1	-	-	-	-	-	-	-	-	-	-	-	-	-	-	-	-	-	-	-	-	-	-	-	-	-	-	-	-	-	-	-	-	-	-	-	-	-	-	-	-	-	-	+	-	-	-	-	-	-	-	-	-	-
6132	11815	hypothetical protein	1	-	-	-	-	-	-	-	-	-	-	-	-	-	-	-	-	-	-	-	-	-	-	-	-	-	-	-	-	-	-	-	-	-	-	-	-	-	-	-	-	-	-	+	-	-	-	-	-	-	-	-	-	-
6133	11816	hypothetical protein	1	-	-	-	-	-	-	-	-	-	-	-	-	-	-	-	-	-	-	-	-	-	-	-	-	-	-	-	-	-	-	-	-	-	-	-	-	-	-	-	-	-	-	+	-	-	-	-	-	-	-	-	-	-
6134	11817	hypothetical protein	1	-	-	-	-	-	-	-	-	-	-	-	-	-	-	-	-	-	-	-	-	-	-	-	-	-	-	-	-	-	-	-	-	-	-	-	-	-	-	-	-	-	-	+	-	-	-	-	-	-	-	-	-	-
6135	11818	hypothetical protein	1	-	-	-	-	-	-	-	-	-	-	-	-	-	-	-	-	-	-	-	-	-	-	-	-	-	-	-	-	-	-	-	-	-	-	-	-	-	-	-	-	-	-	+	-	-	-	-	-	-	-	-	-	-
6136	11819	Membrane protein involved in the export of O-antigen	1	-	-	-	-	-	-	-	-	-	-	-	-	-	-	-	-	-	-	-	-	-	-	-	-	-	-	-	-	-	-	-	-	-	-	-	-	-	-	-	-	-	-	+	-	-	-	-	-	-	-	-	-	-
6137	11901	hypothetical protein	1	-	-	-	-	-	-	-	-	-	-	-	-	-	-	-	-	-	-	-	-	-	-	-	-	-	-	-	-	-	-	-	-	-	-	-	-	-	-	-	-	-	-	-	-	-	-	-	+	-	-	-	-	-	-
6138	11902	glycosyl transferase	1	-	-	-	-	-	-	-	-	-	-	-	-	-	-	-	-	-	-	-	-	-	-	-	-	-	-	-	-	-	-	-	-	-	-	-	-	-	-	-	-	-	-	-	-	-	-	-	+	-	-	-	-	-	-
6139	11903	hypothetical protein	1	-	-	-	-	-	-	-	-	-	-	-	-	-	-	-	-	-	-	-	-	-	-	-	-	-	-	-	-	-	-	-	-	-	-	-	-	-	-	-	-	-	-	-	-	-	-	-	+	-	-	-	-	-	-
6140	11904	hypothetical protein	1	-	-	-	-	-	-	-	-	-	-	-	-	-	-	-	-	-	-	-	-	-	-	-	-	-	-	-	-	-	-	-	-	-	-	-	-	-	-	-	-	-	-	-	-	-	-	-	+	-	-	-	-	-	-
6141	11905	hypothetical protein	1	-	-	-	-	-	-	-	-	-	-	-	-	-	-	-	-	-	-	-	-	-	-	-	-	-	-	-	-	-	-	-	-	-	-	-	-	-	-	-	-	-	-	-	-	-	-	-	+	-	-	-	-	-	-
6142	11906	hypothetical protein	1	-	-	-	-	-	-	-	-	-	-	-	-	-	-	-	-	-	-	-	-	-	-	-	-	-	-	-	-	-	-	-	-	-	-	-	-	-	-	-	-	-	-	-	-	-	-	-	+	-	-	-	-	-	-
6143	11907	hypothetical protein	1	-	-	-	-	-	-	-	-	-	-	-	-	-	-	-	-	-	-	-	-	-	-	-	-	-	-	-	-	-	-	-	-	-	-	-	-	-	-	-	-	-	-	-	-	-	-	-	+	-	-	-	-	-	-
6144	11908	Lipoteichoic acid synthase LtaS Type IVa	1	-	-	-	-	-	-	-	-	-	-	-	-	-	-	-	-	-	-	-	-	-	-	-	-	-	-	-	-	-	-	-	-	-	-	-	-	-	-	-	-	-	-	-	-	-	-	-	+	-	-	-	-	-	-
6145	11909	Hypothetical lipoprotein precursor	1	-	-	-	-	-	-	-	-	-	-	-	-	-	-	-	-	-	-	-	-	-	-	-	-	-	-	-	-	-	-	-	-	-	-	-	-	-	-	-	-	-	-	-	-	-	-	-	+	-	-	-	-	-	-
6146	11910	hypothetical protein	1	-	-	-	-	-	-	-	-	-	-	-	-	-	-	-	-	-	-	-	-	-	-	-	-	-	-	-	-	-	-	-	-	-	-	-	-	-	-	-	-	-	-	-	-	-	-	-	+	-	-	-	-	-	-
6147	11911	hypothetical protein	1	-	-	-	-	-	-	-	-	-	-	-	-	-	-	-	-	-	-	-	-	-	-	-	-	-	-	-	-	-	-	-	-	-	-	-	-	-	-	-	-	-	-	-	-	-	-	-	+	-	-	-	-	-	-
6148	11912	hypothetical protein	1	-	-	-	-	-	-	-	-	-	-	-	-	-	-	-	-	-	-	-	-	-	-	-	-	-	-	-	-	-	-	-	-	-	-	-	-	-	-	-	-	-	-	-	-	-	-	-	+	-	-	-	-	-	-
6149	11913	hypothetical protein	1	-	-	-	-	-	-	-	-	-	-	-	-	-	-	-	-	-	-	-	-	-	-	-	-	-	-	-	-	-	-	-	-	-	-	-	-	-	-	-	-	-	-	-	-	-	-	-	+	-	-	-	-	-	-
6150	11914	PTS system	1	-	-	-	-	-	-	-	-	-	-	-	-	-	-	-	-	-	-	-	-	-	-	-	-	-	-	-	-	-	-	-	-	-	-	-	-	-	-	-	-	-	-	-	-	-	-	-	+	-	-	-	-	-	-
6151	11915	hypothetical protein	1	-	-	-	-	-	-	-	-	-	-	-	-	-	-	-	-	-	-	-	-	-	-	-	-	-	-	-	-	-	-	-	-	-	-	-	-	-	-	-	-	-	-	-	-	-	-	-	+	-	-	-	-	-	-
6152	11916	hypothetical protein	1	-	-	-	-	-	-	-	-	-	-	-	-	-	-	-	-	-	-	-	-	-	-	-	-	-	-	-	-	-	-	-	-	-	-	-	-	-	-	-	-	-	-	-	-	-	-	-	+	-	-	-	-	-	-
6153	11917	hypothetical protein	1	-	-	-	-	-	-	-	-	-	-	-	-	-	-	-	-	-	-	-	-	-	-	-	-	-	-	-	-	-	-	-	-	-	-	-	-	-	-	-	-	-	-	-	-	-	-	-	+	-	-	-	-	-	-
6154	11918	hypothetical protein	1	-	-	-	-	-	-	-	-	-	-	-	-	-	-	-	-	-	-	-	-	-	-	-	-	-	-	-	-	-	-	-	-	-	-	-	-	-	-	-	-	-	-	-	-	-	-	-	+	-	-	-	-	-	-
6155	11919	hypothetical protein	1	-	-	-	-	-	-	-	-	-	-	-	-	-	-	-	-	-	-	-	-	-	-	-	-	-	-	-	-	-	-	-	-	-	-	-	-	-	-	-	-	-	-	-	-	-	-	-	+	-	-	-	-	-	-
6156	11920	hypothetical protein	1	-	-	-	-	-	-	-	-	-	-	-	-	-	-	-	-	-	-	-	-	-	-	-	-	-	-	-	-	-	-	-	-	-	-	-	-	-	-	-	-	-	-	-	-	-	-	-	+	-	-	-	-	-	-
6157	11921	hypothetical protein	1	-	-	-	-	-	-	-	-	-	-	-	-	-	-	-	-	-	-	-	-	-	-	-	-	-	-	-	-	-	-	-	-	-	-	-	-	-	-	-	-	-	-	-	-	-	-	-	+	-	-	-	-	-	-
6158	11922	ABC transporter ATPase component	1	-	-	-	-	-	-	-	-	-	-	-	-	-	-	-	-	-	-	-	-	-	-	-	-	-	-	-	-	-	-	-	-	-	-	-	-	-	-	-	-	-	-	-	-	-	-	-	+	-	-	-	-	-	-
6159	11923	hypothetical protein	1	-	-	-	-	-	-	-	-	-	-	-	-	-	-	-	-	-	-	-	-	-	-	-	-	-	-	-	-	-	-	-	-	-	-	-	-	-	-	-	-	-	-	-	-	-	-	-	+	-	-	-	-	-	-
6160	11924	hypothetical protein	1	-	-	-	-	-	-	-	-	-	-	-	-	-	-	-	-	-	-	-	-	-	-	-	-	-	-	-	-	-	-	-	-	-	-	-	-	-	-	-	-	-	-	-	-	-	-	-	+	-	-	-	-	-	-
6161	11925	Transposase	1	-	-	-	-	-	-	-	-	-	-	-	-	-	-	-	-	-	-	-	-	-	-	-	-	-	-	-	-	-	-	-	-	-	-	-	-	-	-	-	-	-	-	-	-	-	-	-	+	-	-	-	-	-	-
6162	11926	Homocysteine S-methyltransferase (EC 2.1.1.10)	1	-	-	-	-	-	-	-	-	-	-	-	-	-	-	-	-	-	-	-	-	-	-	-	-	-	-	-	-	-	-	-	-	-	-	-	-	-	-	-	-	-	-	-	-	-	-	-	+	-	-	-	-	-	-
6163	11927	Phage tail length tape-measure protein	1	-	-	-	-	-	-	-	-	-	-	-	-	-	-	-	-	-	-	-	-	-	-	-	-	-	-	-	-	-	-	-	-	-	-	-	-	-	-	-	-	-	-	-	-	-	-	-	+	-	-	-	-	-	-
6164	11928	ABC transporter	1	-	-	-	-	-	-	-	-	-	-	-	-	-	-	-	-	-	-	-	-	-	-	-	-	-	-	-	-	-	-	-	-	-	-	-	-	-	-	-	-	-	-	-	-	-	-	-	+	-	-	-	-	-	-
6165	11929	hypothetical protein	1	-	-	-	-	-	-	-	-	-	-	-	-	-	-	-	-	-	-	-	-	-	-	-	-	-	-	-	-	-	-	-	-	-	-	-	-	-	-	-	-	-	-	-	-	-	-	-	+	-	-	-	-	-	-
6166	11930	hypothetical protein	1	-	-	-	-	-	-	-	-	-	-	-	-	-	-	-	-	-	-	-	-	-	-	-	-	-	-	-	-	-	-	-	-	-	-	-	-	-	-	-	-	-	-	-	-	-	-	-	+	-	-	-	-	-	-
6167	11931	BlpS protein	1	-	-	-	-	-	-	-	-	-	-	-	-	-	-	-	-	-	-	-	-	-	-	-	-	-	-	-	-	-	-	-	-	-	-	-	-	-	-	-	-	-	-	-	-	-	-	-	+	-	-	-	-	-	-
6168	11932	hypothetical protein	1	-	-	-	-	-	-	-	-	-	-	-	-	-	-	-	-	-	-	-	-	-	-	-	-	-	-	-	-	-	-	-	-	-	-	-	-	-	-	-	-	-	-	-	-	-	-	-	+	-	-	-	-	-	-
6169	11933	hypothetical protein	1	-	-	-	-	-	-	-	-	-	-	-	-	-	-	-	-	-	-	-	-	-	-	-	-	-	-	-	-	-	-	-	-	-	-	-	-	-	-	-	-	-	-	-	-	-	-	-	+	-	-	-	-	-	-
6170	11934	hypothetical protein	1	-	-	-	-	-	-	-	-	-	-	-	-	-	-	-	-	-	-	-	-	-	-	-	-	-	-	-	-	-	-	-	-	-	-	-	-	-	-	-	-	-	-	-	-	-	-	-	+	-	-	-	-	-	-
6171	11935	hypothetical protein	1	-	-	-	-	-	-	-	-	-	-	-	-	-	-	-	-	-	-	-	-	-	-	-	-	-	-	-	-	-	-	-	-	-	-	-	-	-	-	-	-	-	-	-	-	-	-	-	+	-	-	-	-	-	-
6172	11936	Cu+ P-type ATPase	1	-	-	-	-	-	-	-	-	-	-	-	-	-	-	-	-	-	-	-	-	-	-	-	-	-	-	-	-	-	-	-	-	-	-	-	-	-	-	-	-	-	-	-	-	-	-	-	+	-	-	-	-	-	-
6173	11937	hypothetical protein	1	-	-	-	-	-	-	-	-	-	-	-	-	-	-	-	-	-	-	-	-	-	-	-	-	-	-	-	-	-	-	-	-	-	-	-	-	-	-	-	-	-	-	-	-	-	-	-	+	-	-	-	-	-	-
6174	11938	hypothetical protein	1	-	-	-	-	-	-	-	-	-	-	-	-	-	-	-	-	-	-	-	-	-	-	-	-	-	-	-	-	-	-	-	-	-	-	-	-	-	-	-	-	-	-	-	-	-	-	-	+	-	-	-	-	-	-
6175	11939	hypothetical protein	1	-	-	-	-	-	-	-	-	-	-	-	-	-	-	-	-	-	-	-	-	-	-	-	-	-	-	-	-	-	-	-	-	-	-	-	-	-	-	-	-	-	-	-	-	-	-	-	+	-	-	-	-	-	-
6176	11940	hypothetical protein	1	-	-	-	-	-	-	-	-	-	-	-	-	-	-	-	-	-	-	-	-	-	-	-	-	-	-	-	-	-	-	-	-	-	-	-	-	-	-	-	-	-	-	-	-	-	-	-	+	-	-	-	-	-	-
6177	11941	Cu+ P-type ATPase	1	-	-	-	-	-	-	-	-	-	-	-	-	-	-	-	-	-	-	-	-	-	-	-	-	-	-	-	-	-	-	-	-	-	-	-	-	-	-	-	-	-	-	-	-	-	-	-	+	-	-	-	-	-	-
6178	11942	Phage integrase	1	-	-	-	-	-	-	-	-	-	-	-	-	-	-	-	-	-	-	-	-	-	-	-	-	-	-	-	-	-	-	-	-	-	-	-	-	-	-	-	-	-	-	-	-	-	-	-	+	-	-	-	-	-	-
6179	11943	hypothetical protein	1	-	-	-	-	-	-	-	-	-	-	-	-	-	-	-	-	-	-	-	-	-	-	-	-	-	-	-	-	-	-	-	-	-	-	-	-	-	-	-	-	-	-	-	-	-	-	-	+	-	-	-	-	-	-
6180	11944	hypothetical protein	1	-	-	-	-	-	-	-	-	-	-	-	-	-	-	-	-	-	-	-	-	-	-	-	-	-	-	-	-	-	-	-	-	-	-	-	-	-	-	-	-	-	-	-	-	-	-	-	+	-	-	-	-	-	-
6181	11945	hypothetical protein	1	-	-	-	-	-	-	-	-	-	-	-	-	-	-	-	-	-	-	-	-	-	-	-	-	-	-	-	-	-	-	-	-	-	-	-	-	-	-	-	-	-	-	-	-	-	-	-	+	-	-	-	-	-	-
6182	11946	hypothetical protein	1	-	-	-	-	-	-	-	-	-	-	-	-	-	-	-	-	-	-	-	-	-	-	-	-	-	-	-	-	-	-	-	-	-	-	-	-	-	-	-	-	-	-	-	-	-	-	-	+	-	-	-	-	-	-
6183	11947	hypothetical protein	1	-	-	-	-	-	-	-	-	-	-	-	-	-	-	-	-	-	-	-	-	-	-	-	-	-	-	-	-	-	-	-	-	-	-	-	-	-	-	-	-	-	-	-	-	-	-	-	+	-	-	-	-	-	-
6184	11948	N-acetylmuramoyl-L-alanine amidase(EC:3.5.1.28)	1	-	-	-	-	-	-	-	-	-	-	-	-	-	-	-	-	-	-	-	-	-	-	-	-	-	-	-	-	-	-	-	-	-	-	-	-	-	-	-	-	-	-	-	-	-	-	-	+	-	-	-	-	-	-
6185	11949	hypothetical protein	1	-	-	-	-	-	-	-	-	-	-	-	-	-	-	-	-	-	-	-	-	-	-	-	-	-	-	-	-	-	-	-	-	-	-	-	-	-	-	-	-	-	-	-	-	-	-	-	+	-	-	-	-	-	-
6186	11950	hypothetical protein	1	-	-	-	-	-	-	-	-	-	-	-	-	-	-	-	-	-	-	-	-	-	-	-	-	-	-	-	-	-	-	-	-	-	-	-	-	-	-	-	-	-	-	-	-	-	-	-	+	-	-	-	-	-	-
6187	11951	hypothetical protein	1	-	-	-	-	-	-	-	-	-	-	-	-	-	-	-	-	-	-	-	-	-	-	-	-	-	-	-	-	-	-	-	-	-	-	-	-	-	-	-	-	-	-	-	-	-	-	-	+	-	-	-	-	-	-
6188	11952	hypothetical protein	1	-	-	-	-	-	-	-	-	-	-	-	-	-	-	-	-	-	-	-	-	-	-	-	-	-	-	-	-	-	-	-	-	-	-	-	-	-	-	-	-	-	-	-	-	-	-	-	+	-	-	-	-	-	-
6189	11953	hypothetical protein	1	-	-	-	-	-	-	-	-	-	-	-	-	-	-	-	-	-	-	-	-	-	-	-	-	-	-	-	-	-	-	-	-	-	-	-	-	-	-	-	-	-	-	-	-	-	-	-	+	-	-	-	-	-	-
6190	11954	hypothetical protein	1	-	-	-	-	-	-	-	-	-	-	-	-	-	-	-	-	-	-	-	-	-	-	-	-	-	-	-	-	-	-	-	-	-	-	-	-	-	-	-	-	-	-	-	-	-	-	-	+	-	-	-	-	-	-
6191	11955	hypothetical protein	1	-	-	-	-	-	-	-	-	-	-	-	-	-	-	-	-	-	-	-	-	-	-	-	-	-	-	-	-	-	-	-	-	-	-	-	-	-	-	-	-	-	-	-	-	-	-	-	+	-	-	-	-	-	-
6192	11956	hypothetical protein	1	-	-	-	-	-	-	-	-	-	-	-	-	-	-	-	-	-	-	-	-	-	-	-	-	-	-	-	-	-	-	-	-	-	-	-	-	-	-	-	-	-	-	-	-	-	-	-	+	-	-	-	-	-	-
6193	11957	hypothetical protein	1	-	-	-	-	-	-	-	-	-	-	-	-	-	-	-	-	-	-	-	-	-	-	-	-	-	-	-	-	-	-	-	-	-	-	-	-	-	-	-	-	-	-	-	-	-	-	-	+	-	-	-	-	-	-
6194	11958	hypothetical protein	1	-	-	-	-	-	-	-	-	-	-	-	-	-	-	-	-	-	-	-	-	-	-	-	-	-	-	-	-	-	-	-	-	-	-	-	-	-	-	-	-	-	-	-	-	-	-	-	+	-	-	-	-	-	-
6195	11959	hypothetical protein	1	-	-	-	-	-	-	-	-	-	-	-	-	-	-	-	-	-	-	-	-	-	-	-	-	-	-	-	-	-	-	-	-	-	-	-	-	-	-	-	-	-	-	-	-	-	-	-	+	-	-	-	-	-	-
6196	11960	hypothetical protein	1	-	-	-	-	-	-	-	-	-	-	-	-	-	-	-	-	-	-	-	-	-	-	-	-	-	-	-	-	-	-	-	-	-	-	-	-	-	-	-	-	-	-	-	-	-	-	-	+	-	-	-	-	-	-
6197	11961	hypothetical protein	1	-	-	-	-	-	-	-	-	-	-	-	-	-	-	-	-	-	-	-	-	-	-	-	-	-	-	-	-	-	-	-	-	-	-	-	-	-	-	-	-	-	-	-	-	-	-	-	+	-	-	-	-	-	-
6198	11962	hypothetical protein	1	-	-	-	-	-	-	-	-	-	-	-	-	-	-	-	-	-	-	-	-	-	-	-	-	-	-	-	-	-	-	-	-	-	-	-	-	-	-	-	-	-	-	-	-	-	-	-	+	-	-	-	-	-	-
6199	11963	hypothetical protein	1	-	-	-	-	-	-	-	-	-	-	-	-	-	-	-	-	-	-	-	-	-	-	-	-	-	-	-	-	-	-	-	-	-	-	-	-	-	-	-	-	-	-	-	-	-	-	-	+	-	-	-	-	-	-
6200	11964	Two-component response regulator yesN	1	-	-	-	-	-	-	-	-	-	-	-	-	-	-	-	-	-	-	-	-	-	-	-	-	-	-	-	-	-	-	-	-	-	-	-	-	-	-	-	-	-	-	-	-	-	-	-	+	-	-	-	-	-	-
6201	11965	hypothetical protein	1	-	-	-	-	-	-	-	-	-	-	-	-	-	-	-	-	-	-	-	-	-	-	-	-	-	-	-	-	-	-	-	-	-	-	-	-	-	-	-	-	-	-	-	-	-	-	-	+	-	-	-	-	-	-
6202	11966	hypothetical protein	1	-	-	-	-	-	-	-	-	-	-	-	-	-	-	-	-	-	-	-	-	-	-	-	-	-	-	-	-	-	-	-	-	-	-	-	-	-	-	-	-	-	-	-	-	-	-	-	+	-	-	-	-	-	-
6203	11967	hypothetical protein	1	-	-	-	-	-	-	-	-	-	-	-	-	-	-	-	-	-	-	-	-	-	-	-	-	-	-	-	-	-	-	-	-	-	-	-	-	-	-	-	-	-	-	-	-	-	-	-	+	-	-	-	-	-	-

Table S5. Precence/absence of each HG among 57 *S. suis* strains

				1																13			25					28						104		94			76			53/54		Divergent *S. suis*																
Index[a]	ClusterID	Gene products	Members	98HAH33	05ZYH33	SC84	A7	SS12	P1/7	DAT273	NIAH11434	JS14	GZ1	BM407	S735	13730	MNCM07	NCTC 10446	14636	NCTC 10237	ST1	2651	89-1591	MNCM04	MNCM25	8074	D9	4961	ST3	MNCM43	DAT292	DAT260	NIAH11435	MNCM21	MNCM50	89-2479	DAT234	DAT290	93A	42A	NT77	6407	11538	8830	12814	92-1400	89-590	2726	2524	89-3576-3	4417	D12	10581	89-5259	88-5299A	R61	92-1191	14A	92-4172	22083
6204	11968	hypothetical protein	1	-	-	-	-	-	-	-	-	-	-	-	-	-	-	-	-	-	-	-	-	-	-	-	-	-	-	-	-	-	-	-	-	-	-	-	-	-	-	-	-	-	-	-	-	-	-	-	-	-	-	+	-	-	-	-	-	-
6205	11969	hypothetical protein	1	-	-	-	-	-	-	-	-	-	-	-	-	-	-	-	-	-	-	-	-	-	-	-	-	-	-	-	-	-	-	-	-	-	-	-	-	-	-	-	-	-	-	-	-	-	-	-	-	-	-	+	-	-	-	-	-	-
6206	11970	acetyltransferase	1	-	-	-	-	-	-	-	-	-	-	-	-	-	-	-	-	-	-	-	-	-	-	-	-	-	-	-	-	-	-	-	-	-	-	-	-	-	-	-	-	-	-	-	-	-	-	-	-	-	-	+	-	-	-	-	-	-
6207	11971	hypothetical protein	1	-	-	-	-	-	-	-	-	-	-	-	-	-	-	-	-	-	-	-	-	-	-	-	-	-	-	-	-	-	-	-	-	-	-	-	-	-	-	-	-	-	-	-	-	-	-	-	-	-	-	+	-	-	-	-	-	-
6208	11972	hypothetical protein	1	-	-	-	-	-	-	-	-	-	-	-	-	-	-	-	-	-	-	-	-	-	-	-	-	-	-	-	-	-	-	-	-	-	-	-	-	-	-	-	-	-	-	-	-	-	-	-	-	-	-	+	-	-	-	-	-	-
6209	11973	hypothetical protein	1	-	-	-	-	-	-	-	-	-	-	-	-	-	-	-	-	-	-	-	-	-	-	-	-	-	-	-	-	-	-	-	-	-	-	-	-	-	-	-	-	-	-	-	-	-	-	-	-	-	-	+	-	-	-	-	-	-
6210	11974	hypothetical protein	1	-	-	-	-	-	-	-	-	-	-	-	-	-	-	-	-	-	-	-	-	-	-	-	-	-	-	-	-	-	-	-	-	-	-	-	-	-	-	-	-	-	-	-	-	-	-	-	-	-	-	+	-	-	-	-	-	-
6211	11975	hypothetical protein	1	-	-	-	-	-	-	-	-	-	-	-	-	-	-	-	-	-	-	-	-	-	-	-	-	-	-	-	-	-	-	-	-	-	-	-	-	-	-	-	-	-	-	-	-	-	-	-	-	-	-	+	-	-	-	-	-	-
6212	11976	hypothetical protein	1	-	-	-	-	-	-	-	-	-	-	-	-	-	-	-	-	-	-	-	-	-	-	-	-	-	-	-	-	-	-	-	-	-	-	-	-	-	-	-	-	-	-	-	-	-	-	-	-	-	-	+	-	-	-	-	-	-
6213	11977	hypothetical protein	1	-	-	-	-	-	-	-	-	-	-	-	-	-	-	-	-	-	-	-	-	-	-	-	-	-	-	-	-	-	-	-	-	-	-	-	-	-	-	-	-	-	-	-	-	-	-	-	-	-	-	+	-	-	-	-	-	-
6214	11978	hypothetical protein	1	-	-	-	-	-	-	-	-	-	-	-	-	-	-	-	-	-	-	-	-	-	-	-	-	-	-	-	-	-	-	-	-	-	-	-	-	-	-	-	-	-	-	-	-	-	-	-	-	-	-	+	-	-	-	-	-	-
6215	11979	hypothetical protein	1	-	-	-	-	-	-	-	-	-	-	-	-	-	-	-	-	-	-	-	-	-	-	-	-	-	-	-	-	-	-	-	-	-	-	-	-	-	-	-	-	-	-	-	-	-	-	-	-	-	-	+	-	-	-	-	-	-
6216	11980	hypothetical protein	1	-	-	-	-	-	-	-	-	-	-	-	-	-	-	-	-	-	-	-	-	-	-	-	-	-	-	-	-	-	-	-	-	-	-	-	-	-	-	-	-	-	-	-	-	-	-	-	-	-	-	+	-	-	-	-	-	-
6217	11981	Capsular polysaccharide synthesis	1	-	-	-	-	-	-	-	-	-	-	-	-	-	-	-	-	-	-	-	-	-	-	-	-	-	-	-	-	-	-	-	-	-	-	-	-	-	-	-	-	-	-	-	-	-	-	-	-	-	-	+	-	-	-	-	-	-
6218	11982	hypothetical protein	1	-	-	-	-	-	-	-	-	-	-	-	-	-	-	-	-	-	-	-	-	-	-	-	-	-	-	-	-	-	-	-	-	-	-	-	-	-	-	-	-	-	-	-	-	-	-	-	-	-	-	+	-	-	-	-	-	-
6219	11983	Beta-glucosidase (EC 3.2.1.21)	1	-	-	-	-	-	-	-	-	-	-	-	-	-	-	-	-	-	-	-	-	-	-	-	-	-	-	-	-	-	-	-	-	-	-	-	-	-	-	-	-	-	-	-	-	-	-	-	-	-	-	+	-	-	-	-	-	-
6220	11984	hypothetical protein	1	-	-	-	-	-	-	-	-	-	-	-	-	-	-	-	-	-	-	-	-	-	-	-	-	-	-	-	-	-	-	-	-	-	-	-	-	-	-	-	-	-	-	-	-	-	-	-	-	-	-	+	-	-	-	-	-	-
6221	11985	Xyloside transporter XynT	1	-	-	-	-	-	-	-	-	-	-	-	-	-	-	-	-	-	-	-	-	-	-	-	-	-	-	-	-	-	-	-	-	-	-	-	-	-	-	-	-	-	-	-	-	-	-	-	-	-	-	+	-	-	-	-	-	-
6222	11986	hypothetical protein	1	-	-	-	-	-	-	-	-	-	-	-	-	-	-	-	-	-	-	-	-	-	-	-	-	-	-	-	-	-	-	-	-	-	-	-	-	-	-	-	-	-	-	-	-	-	-	-	-	-	-	+	-	-	-	-	-	-
6223	11987	hypothetical protein	1	-	-	-	-	-	-	-	-	-	-	-	-	-	-	-	-	-	-	-	-	-	-	-	-	-	-	-	-	-	-	-	-	-	-	-	-	-	-	-	-	-	-	-	-	-	-	-	-	-	-	+	-	-	-	-	-	-
6224	11988	hypothetical protein	1	-	-	-	-	-	-	-	-	-	-	-	-	-	-	-	-	-	-	-	-	-	-	-	-	-	-	-	-	-	-	-	-	-	-	-	-	-	-	-	-	-	-	-	-	-	-	-	-	-	-	+	-	-	-	-	-	-
6225	11989	hypothetical protein	1	-	-	-	-	-	-	-	-	-	-	-	-	-	-	-	-	-	-	-	-	-	-	-	-	-	-	-	-	-	-	-	-	-	-	-	-	-	-	-	-	-	-	-	-	-	-	-	-	-	-	+	-	-	-	-	-	-
6226	11990	hypothetical protein	1	-	-	-	-	-	-	-	-	-	-	-	-	-	-	-	-	-	-	-	-	-	-	-	-	-	-	-	-	-	-	-	-	-	-	-	-	-	-	-	-	-	-	-	-	-	-	-	-	-	-	+	-	-	-	-	-	-
6227	11991	hypothetical protein	1	-	-	-	-	-	-	-	-	-	-	-	-	-	-	-	-	-	-	-	-	-	-	-	-	-	-	-	-	-	-	-	-	-	-	-	-	-	-	-	-	-	-	-	-	-	-	-	-	-	-	+	-	-	-	-	-	-
6228	11992	hypothetical protein	1	-	-	-	-	-	-	-	-	-	-	-	-	-	-	-	-	-	-	-	-	-	-	-	-	-	-	-	-	-	-	-	-	-	-	-	-	-	-	-	-	-	-	-	-	-	-	-	-	-	-	+	-	-	-	-	-	-
6229	11993	hypothetical protein	1	-	-	-	-	-	-	-	-	-	-	-	-	-	-	-	-	-	-	-	-	-	-	-	-	-	-	-	-	-	-	-	-	-	-	-	-	-	-	-	-	-	-	-	-	-	-	-	-	-	-	+	-	-	-	-	-	-
6230	11994	hypothetical protein	1	-	-	-	-	-	-	-	-	-	-	-	-	-	-	-	-	-	-	-	-	-	-	-	-	-	-	-	-	-	-	-	-	-	-	-	-	-	-	-	-	-	-	-	-	-	-	-	-	-	-	+	-	-	-	-	-	-
6231	11995	hypothetical protein	1	-	-	-	-	-	-	-	-	-	-	-	-	-	-	-	-	-	-	-	-	-	-	-	-	-	-	-	-	-	-	-	-	-	-	-	-	-	-	-	-	-	-	-	-	-	-	-	-	-	-	+	-	-	-	-	-	-
6232	11996	hypothetical protein	1	-	-	-	-	-	-	-	-	-	-	-	-	-	-	-	-	-	-	-	-	-	-	-	-	-	-	-	-	-	-	-	-	-	-	-	-	-	-	-	-	-	-	-	-	-	-	-	-	-	-	+	-	-	-	-	-	-
6233	11997	hypothetical protein	1	-	-	-	-	-	-	-	-	-	-	-	-	-	-	-	-	-	-	-	-	-	-	-	-	-	-	-	-	-	-	-	-	-	-	-	-	-	-	-	-	-	-	-	-	-	-	-	-	-	-	+	-	-	-	-	-	-
6234	11998	hypothetical protein	1	-	-	-	-	-	-	-	-	-	-	-	-	-	-	-	-	-	-	-	-	-	-	-	-	-	-	-	-	-	-	-	-	-	-	-	-	-	-	-	-	-	-	-	-	-	-	-	-	-	-	+	-	-	-	-	-	-
6235	11999	hypothetical protein	1	-	-	-	-	-	-	-	-	-	-	-	-	-	-	-	-	-	-	-	-	-	-	-	-	-	-	-	-	-	-	-	-	-	-	-	-	-	-	-	-	-	-	-	-	-	-	-	-	-	-	+	-	-	-	-	-	-
6236	12000	hypothetical protein	1	-	-	-	-	-	-	-	-	-	-	-	-	-	-	-	-	-	-	-	-	-	-	-	-	-	-	-	-	-	-	-	-	-	-	-	-	-	-	-	-	-	-	-	-	-	-	-	-	-	-	+	-	-	-	-	-	-
6237	12001	hypothetical protein	1	-	-	-	-	-	-	-	-	-	-	-	-	-	-	-	-	-	-	-	-	-	-	-	-	-	-	-	-	-	-	-	-	-	-	-	-	-	-	-	-	-	-	-	-	-	-	-	-	-	-	+	-	-	-	-	-	-
6238	12002	hypothetical protein	1	-	-	-	-	-	-	-	-	-	-	-	-	-	-	-	-	-	-	-	-	-	-	-	-	-	-	-	-	-	-	-	-	-	-	-	-	-	-	-	-	-	-	-	-	-	-	-	-	-	-	+	-	-	-	-	-	-
6239	12003	capsular polysaccharide biosynthesis proteinCps4H	1	-	-	-	-	-	-	-	-	-	-	-	-	-	-	-	-	-	-	-	-	-	-	-	-	-	-	-	-	-	-	-	-	-	-	-	-	-	-	-	-	-	-	-	-	-	-	-	-	-	-	+	-	-	-	-	-	-
6240	12004	hypothetical protein	1	-	-	-	-	-	-	-	-	-	-	-	-	-	-	-	-	-	-	-	-	-	-	-	-	-	-	-	-	-	-	-	-	-	-	-	-	-	-	-	-	-	-	-	-	-	-	-	-	-	-	+	-	-	-	-	-	-
6241	12005	hypothetical protein	1	-	-	-	-	-	-	-	-	-	-	-	-	-	-	-	-	-	-	-	-	-	-	-	-	-	-	-	-	-	-	-	-	-	-	-	-	-	-	-	-	-	-	-	-	-	-	-	-	-	-	+	-	-	-	-	-	-
6242	12006	hypothetical protein	1	-	-	-	-	-	-	-	-	-	-	-	-	-	-	-	-	-	-	-	-	-	-	-	-	-	-	-	-	-	-	-	-	-	-	-	-	-	-	-	-	-	-	-	-	-	-	-	-	-	-	+	-	-	-	-	-	-
6243	12007	hypothetical protein	1	-	-	-	-	-	-	-	-	-	-	-	-	-	-	-	-	-	-	-	-	-	-	-	-	-	-	-	-	-	-	-	-	-	-	-	-	-	-	-	-	-	-	-	-	-	-	-	-	-	-	+	-	-	-	-	-	-
6244	12008	hypothetical protein	1	-	-	-	-	-	-	-	-	-	-	-	-	-	-	-	-	-	-	-	-	-	-	-	-	-	-	-	-	-	-	-	-	-	-	-	-	-	-	-	-	-	-	-	-	-	-	-	-	-	-	+	-	-	-	-	-	-
6245	12009	hypothetical protein	1	-	-	-	-	-	-	-	-	-	-	-	-	-	-	-	-	-	-	-	-	-	-	-	-	-	-	-	-	-	-	-	-	-	-	-	-	-	-	-	-	-	-	-	-	-	-	-	-	-	-	+	-	-	-	-	-	-
6246	12010	hypothetical protein	1	-	-	-	-	-	-	-	-	-	-	-	-	-	-	-	-	-	-	-	-	-	-	-	-	-	-	-	-	-	-	-	-	-	-	-	-	-	-	-	-	-	-	-	-	-	-	-	-	-	-	+	-	-	-	-	-	-
6247	12011	DNA-cytosine methyltransferase (EC 2.1.1.37)	1	-	-	-	-	-	-	-	-	-	-	-	-	-	-	-	-	-	-	-	-	-	-	-	-	-	-	-	-	-	-	-	-	-	-	-	-	-	-	-	-	-	-	-	-	-	-	-	-	-	-	+	-	-	-	-	-	-
6248	12012	Glucuronide permease	1	-	-	-	-	-	-	-	-	-	-	-	-	-	-	-	-	-	-	-	-	-	-	-	-	-	-	-	-	-	-	-	-	-	-	-	-	-	-	-	-	-	-	-	-	-	-	-	-	-	-	+	-	-	-	-	-	-
6249	12013	hypothetical protein	1	-	-	-	-	-	-	-	-	-	-	-	-	-	-	-	-	-	-	-	-	-	-	-	-	-	-	-	-	-	-	-	-	-	-	-	-	-	-	-	-	-	-	-	-	-	-	-	-	-	-	+	-	-	-	-	-	-
6250	12014	putative low temperature requirement A protein	1	-	-	-	-	-	-	-	-	-	-	-	-	-	-	-	-	-	-	-	-	-	-	-	-	-	-	-	-	-	-	-	-	-	-	-	-	-	-	-	-	-	-	-	-	-	-	-	-	-	-	+	-	-	-	-	-	-
6251	12015	hypothetical protein	1	-	-	-	-	-	-	-	-	-	-	-	-	-	-	-	-	-	-	-	-	-	-	-	-	-	-	-	-	-	-	-	-	-	-	-	-	-	-	-	-	-	-	-	-	-	-	-	-	-	-	+	-	-	-	-	-	-
6252	12016	hypothetical protein	1	-	-	-	-	-	-	-	-	-	-	-	-	-	-	-	-	-	-	-	-	-	-	-	-	-	-	-	-	-	-	-	-	-	-	-	-	-	-	-	-	-	-	-	-	-	-	-	-	-	-	+	-	-	-	-	-	-
6253	12017	hypothetical protein	1	-	-	-	-	-	-	-	-	-	-	-	-	-	-	-	-	-	-	-	-	-	-	-	-	-	-	-	-	-	-	-	-	-	-	-	-	-	-	-	-	-	-	-	-	-	-	-	-	-	-	+	-	-	-	-	-	-
6254	12018	Site-specific recombinase	1	-	-	-	-	-	-	-	-	-	-	-	-	-	-	-	-	-	-	-	-	-	-	-	-	-	-	-	-	-	-	-	-	-	-	-	-	-	-	-	-	-	-	-	-	-	-	-	-	-	-	+	-	-	-	-	-	-
6255	12019	LPS biosynthesis protein	1	-	-	-	-	-	-	-	-	-	-	-	-	-	-	-	-	-	-	-	-	-	-	-	-	-	-	-	-	-	-	-	-	-	-	-	-	-	-	-	-	-	-	-	-	-	-	-	-	-	-	+	-	-	-	-	-	-
6256	12020	hypothetical protein	1	-	-	-	-	-	-	-	-	-	-	-	-	-	-	-	-	-	-	-	-	-	-	-	-	-	-	-	-	-	-	-	-	-	-	-	-	-	-	-	-	-	-	-	-	-	-	-	-	-	-	+	-	-	-	-	-	-
6257	12021	hypothetical protein	1	-	-	-	-	-	-	-	-	-	-	-	-	-	-	-	-	-	-	-	-	-	-	-	-	-	-	-	-	-	-	-	-	-	-	-	-	-	-	-	-	-	-	-	-	-	-	-	-	-	-	+	-	-	-	-	-	-
6258	12022	Beta-1	1	-	-	-	-	-	-	-	-	-	-	-	-	-	-	-	-	-	-	-	-	-	-	-	-	-	-	-	-	-	-	-	-	-	-	-	-	-	-	-	-	-	-	-	-	-	-	-	-	-	-	+	-	-	-	-	-	-
6259	12023	ClpB protein	1	-	-	-	-	-	-	-	-	-	-	-	-	-	-	-	-	-	-	-	-	-	-	-	-	-	-	-	-	-	-	-	-	-	-	-	-	-	-	-	-	-	-	-	-	-	-	-	-	-	-	+	-	-	-	-	-	-
6260	12024	hypothetical protein	1	-	-	-	-	-	-	-	-	-	-	-	-	-	-	-	-	-	-	-	-	-	-	-	-	-	-	-	-	-	-	-	-	-	-	-	-	-	-	-	-	-	-	-	-	-	-	-	-	-	-	+	-	-	-	-	-	-
6261	12025	hypothetical protein	1	-	-	-	-	-	-	-	-	-	-	-	-	-	-	-	-	-	-	-	-	-	-	-	-	-	-	-	-	-	-	-	-	-	-	-	-	-	-	-	-	-	-	-	-	-	-	-	-	-	-	+	-	-	-	-	-	-
6262	12026	hypothetical protein	1	-	-	-	-	-	-	-	-	-	-	-	-	-	-	-	-	-	-	-	-	-	-	-	-	-	-	-	-	-	-	-	-	-	-	-	-	-	-	-	-	-	-	-	-	-	-	-	-	-	-	+	-	-	-	-	-	-
6263	12027	hypothetical protein	1	-	-	-	-	-	-	-	-	-	-	-	-	-	-	-	-	-	-	-	-	-	-	-	-	-	-	-	-	-	-	-	-	-	-	-	-	-	-	-	-	-	-	-	-	-	-	-	-	-	-	+	-	-	-	-	-	-
6264	12028	Beta-1	1	-	-	-	-	-	-	-	-	-	-	-	-	-	-	-	-	-	-	-	-	-	-	-	-	-	-	-	-	-	-	-	-	-	-	-	-	-	-	-	-	-	-	-	-	-	-	-	-	-	-	+	-	-	-	-	-	-
6265	12029	possible transcriptional regulator	1	-	-	-	-	-	-	-	-	-	-	-	-	-	-	-	-	-	-	-	-	-	-	-	-	-	-	-	-	-	-	-	-	-	-	-	-	-	-	-	-	-	-	-	-	-	-	-	-	-	-	+	-	-	-	-	-	-
6266	12030	hypothetical protein	1	-	-	-	-	-	-	-	-	-	-	-	-	-	-	-	-	-	-	-	-	-	-	-	-	-	-	-	-	-	-	-	-	-	-	-	-	-	-	-	-	-	-	-	-	-	-	-	-	-	-	+	-	-	-	-	-	-
6267	12031	hypothetical protein	1	-	-	-	-	-	-	-	-	-	-	-	-	-	-	-	-	-	-	-	-	-	-	-	-	-	-	-	-	-	-	-	-	-	-	-	-	-	-	-	-	-	-	-	-	-	-	-	-	-	-	+	-	-	-	-	-	-
6268	12032	hypothetical protein	1	-	-	-	-	-	-	-	-	-	-	-	-	-	-	-	-	-	-	-	-	-	-	-	-	-	-	-	-	-	-	-	-	-	-	-	-	-	-	-	-	-	-	-	-	-	-	-	-	-	-	+	-	-	-	-	-	-
6269	12033	hypothetical protein	1	-	-	-	-	-	-	-	-	-	-	-	-	-	-	-	-	-	-	-	-	-	-	-	-	-	-	-	-	-	-	-	-	-	-	-	-	-	-	-	-	-	-	-	-	-	-	-	-	-	-	+	-	-	-	-	-	-
6270	12034	hypothetical protein	1	-	-	-	-	-	-	-	-	-	-	-	-	-	-	-	-	-	-	-	-	-	-	-	-	-	-	-	-	-	-	-	-	-	-	-	-	-	-	-	-	-	-	-	-	-	-	-	-	-	-	+	-	-	-	-	-	-
6271	12035	Beta-galactosidase (EC 3.2.1.23)	1	-	-	-	-	-	-	-	-	-	-	-	-	-	-	-	-	-	-	-	-	-	-	-	-	-	-	-	-	-	-	-	-	-	-	-	-	-	-	-	-	-	-	-	-	-	-	-	-	-	-	+	-	-	-	-	-	-
6272	12036	hypothetical protein	1	-	-	-	-	-	-	-	-	-	-	-	-	-	-	-	-	-	-	-	-	-	-	-	-	-	-	-	-	-	-	-	-	-	-	-	-	-	-	-	-	-	-	-	-	-	-	-	-	-	-	+	-	-	-	-	-	-
6273	12037	hypothetical protein	1	-	-	-	-	-	-	-	-	-	-	-	-	-	-	-	-	-	-	-	-	-	-	-	-	-	-	-	-	-	-	-	-	-	-	-	-	-	-	-	-	-	-	-	-	-	-	-	-	-	-	+	-	-	-	-	-	-
6274	12038	Alfa-L-rhamnosidase (EC 3.2.1.40)	1	-	-	-	-	-	-	-	-	-	-	-	-	-	-	-	-	-	-	-	-	-	-	-	-	-	-	-	-	-	-	-	-	-	-	-	-	-	-	-	-	-	-	-	-	-	-	-	-	-	-	+	-	-	-	-	-	-
6275	12039	hypothetical protein	1	-	-	-	-	-	-	-	-	-	-	-	-	-	-	-	-	-	-	-	-	-	-	-	-	-	-	-	-	-	-	-	-	-	-	-	-	-	-	-	-	-	-	-	-	-	-	-	-	-	-	+	-	-	-	-	-	-
6276	12040	Helix-turn-helix	1	-	-	-	-	-	-	-	-	-	-	-	-	-	-	-	-	-	-	-	-	-	-	-	-	-	-	-	-	-	-	-	-	-	-	-	-	-	-	-	-	-	-	-	-	-	-	-	-	-	-	+	-	-	-	-	-	-
6277	12041	hypothetical protein	1	-	-	-	-	-	-	-	-	-	-	-	-	-	-	-	-	-	-	-	-	-	-	-	-	-	-	-	-	-	-	-	-	-	-	-	-	-	-	-	-	-	-	-	-	-	-	-	-	-	-	+	-	-	-	-	-	-
6278	12042	hypothetical protein	1	-	-	-	-	-	-	-	-	-	-	-	-	-	-	-	-	-	-	-	-	-	-	-	-	-	-	-	-	-	-	-	-	-	-	-	-	-	-	-	-	-	-	-	-	-	-	-	-	-	-	+	-	-	-	-	-	-
6279	12043	hypothetical protein	1	-	-	-	-	-	-	-	-	-	-	-	-	-	-	-	-	-	-	-	-	-	-	-	-	-	-	-	-	-	-	-	-	-	-	-	-	-	-	-	-	-	-	-	-	-	-	-	-	-	-	+	-	-	-	-	-	-
6280	12044	Type I restriction-modification system	1	-	-	-	-	-	-	-	-	-	-	-	-	-	-	-	-	-	-	-	-	-	-	-	-	-	-	-	-	-	-	-	-	-	-	-	-	-	-	-	-	-	-	-	-	-	-	-	-	-	-	+	-	-	-	-	-	-
6281	12045	hypothetical protein	1	-	-	-	-	-	-	-	-	-	-	-	-	-	-	-	-	-	-	-	-	-	-	-	-	-	-	-	-	-	-	-	-	-	-	-	-	-	-	-	-	-	-	-	-	-	-	-	-	-	-	+	-	-	-	-	-	-
6282	12046	Transposase	1	-	-	-	-	-	-	-	-	-	-	-	-	-	-	-	-	-	-	-	-	-	-	-	-	-	-	-	-	-	-	-	-	-	-	-	-	-	-	-	-	-	-	-	-	-	-	-	-	-	-	+	-	-	-	-	-	-
6283	12047	hypothetical protein	1	-	-	-	-	-	-	-	-	-	-	-	-	-	-	-	-	-	-	-	-	-	-	-	-	-	-	-	-	-	-	-	-	-	-	-	-	-	-	-	-	-	-	-	-	-	-	-	-	-	-	+	-	-	-	-	-	-
6284	12048	membrane protein	1	-	-	-	-	-	-	-	-	-	-	-	-	-	-	-	-	-	-	-	-	-	-	-	-	-	-	-	-	-	-	-	-	-	-	-	-	-	-	-	-	-	-	-	-	-	-	-	-	-	-	+	-	-	-	-	-	-
6285	12049	hypothetical protein	1	-	-	-	-	-	-	-	-	-	-	-	-	-	-	-	-	-	-	-	-	-	-	-	-	-	-	-	-	-	-	-	-	-	-	-	-	-	-	-	-	-	-	-	-	-	-	-	-	-	-	+	-	-	-	-	-	-
6286	12050	conserved domain protein	1	-	-	-	-	-	-	-	-	-	-	-	-	-	-	-	-	-	-	-	-	-	-	-	-	-	-	-	-	-	-	-	-	-	-	-	-	-	-	-	-	-	-	-	-	-	-	-	-	-	-	+	-	-	-	-	-	-
6287	12051	ADP-ribose pyrophosphatase	1	-	-	-	-	-	-	-	-	-	-	-	-	-	-	-	-	-	-	-	-	-	-	-	-	-	-	-	-	-	-	-	-	-	-	-	-	-	-	-	-	-	-	-	-	-	-	-	-	-	-	+	-	-	-	-	-	-
6288	12052	Glycosyl transferase	1	-	-	-	-	-	-	-	-	-	-	-	-	-	-	-	-	-	-	-	-	-	-	-	-	-	-	-	-	-	-	-	-	-	-	-	-	-	-	-	-	-	-	-	-	-	-	-	-	-	-	+	-	-	-	-	-	-
6289	12053	hypothetical protein	1	-	-	-	-	-	-	-	-	-	-	-	-	-	-	-	-	-	-	-	-	-	-	-	-	-	-	-	-	-	-	-	-	-	-	-	-	-	-	-	-	-	-	-	-	-	-	-	-	-	-	+	-	-	-	-	-	-
6290	12054	sensor histidine kinase	1	-	-	-	-	-	-	-	-	-	-	-	-	-	-	-	-	-	-	-	-	-	-	-	-	-	-	-	-	-	-	-	-	-	-	-	-	-	-	-	-	-	-	-	-	-	-	-	-	-	-	+	-	-	-	-	-	-
6291	12055	hypothetical protein	1	-	-	-	-	-	-	-	-	-	-	-	-	-	-	-	-	-	-	-	-	-	-	-	-	-	-	-	-	-	-	-	-	-	-	-	-	-	-	-	-	-	-	-	-	-	-	-	-	-	-	+	-	-	-	-	-	-
6292	12056	hypothetical protein	1	-	-	-	-	-	-	-	-	-	-	-	-	-	-	-	-	-	-	-	-	-	-	-	-	-	-	-	-	-	-	-	-	-	-	-	-	-	-	-	-	-	-	-	-	-	-	-	-	-	-	+	-	-	-	-	-	-
6293	12057	Alfa-L-rhamnosidase (EC 3.2.1.40)	1	-	-	-	-	-	-	-	-	-	-	-	-	-	-	-	-	-	-	-	-	-	-	-	-	-	-	-	-	-	-	-	-	-	-	-	-	-	-	-	-	-	-	-	-	-	-	-	-	-	-	+	-	-	-	-	-	-
6294	12058	hypothetical protein	1	-	-	-	-	-	-	-	-	-	-	-	-	-	-	-	-	-	-	-	-	-	-	-	-	-	-	-	-	-	-	-	-	-	-	-	-	-	-	-	-	-	-	-	-	-	-	-	-	-	-	+	-	-	-	-	-	-
6295	12059	Histidine kinase of the competence regulon ComD	1	-	-	-	-	-	-	-	-	-	-	-	-	-	-	-	-	-	-	-	-	-	-	-	-	-	-	-	-	-	-	-	-	-	-	-	-	-	-	-	-	-	-	-	-	-	-	-	-	-	-	+	-	-	-	-	-	-
6296	12060	hypothetical protein	1	-	-	-	-	-	-	-	-	-	-	-	-	-	-	-	-	-	-	-	-	-	-	-	-	-	-	-	-	-	-	-	-	-	-	-	-	-	-	-	-	-	-	-	-	-	-	-	-	-	-	+	-	-	-	-	-	-
6297	12061	hypothetical protein	1	-	-	-	-	-	-	-	-	-	-	-	-	-	-	-	-	-	-	-	-	-	-	-	-	-	-	-	-	-	-	-	-	-	-	-	-	-	-	-	-	-	-	-	-	-	-	-	-	-	-	+	-	-	-	-	-	-
6298	12062	hypothetical protein	1	-	-	-	-	-	-	-	-	-	-	-	-	-	-	-	-	-	-	-	-	-	-	-	-	-	-	-	-	-	-	-	-	-	-	-	-	-	-	-	-	-	-	-	-	-	-	-	-	-	-	+	-	-	-	-	-	-
6299	12063	hypothetical protein	1	-	-	-	-	-	-	-	-	-	-	-	-	-	-	-	-	-	-	-	-	-	-	-	-	-	-	-	-	-	-	-	-	-	-	-	-	-	-	-	-	-	-	-	-	-	-	-	-	-	-	+	-	-	-	-	-	-
6300	12064	hypothetical protein	1	-	-	-	-	-	-	-	-	-	-	-	-	-	-	-	-	-	-	-	-	-	-	-	-	-	-	-	-	-	-	-	-	-	-	-	-	-	-	-	-	-	-	-	-	-	-	-	-	-	-	+	-	-	-	-	-	-
6301	12065	hypothetical protein	1	-	-	-	-	-	-	-	-	-	-	-	-	-	-	-	-	-	-	-	-	-	-	-	-	-	-	-	-	-	-	-	-	-	-	-	-	-	-	-	-	-	-	-	-	-	-	-	-	-	-	+	-	-	-	-	-	-
6302	12066	hypothetical protein	1	-	-	-	-	-	-	-	-	-	-	-	-	-	-	-	-	-	-	-	-	-	-	-	-	-	-	-	-	-	-	-	-	-	-	-	-	-	-	-	-	-	-	-	-	-	-	-	-	-	-	+	-	-	-	-	-	-
6303	12067	hypothetical protein	1	-	-	-	-	-	-	-	-	-	-	-	-	-	-	-	-	-	-	-	-	-	-	-	-	-	-	-	-	-	-	-	-	-	-	-	-	-	-	-	-	-	-	-	-	-	-	-	-	-	-	+	-	-	-	-	-	-
6304	12068	Transcriptional regulator	1	-	-	-	-	-	-	-	-	-	-	-	-	-	-	-	-	-	-	-	-	-	-	-	-	-	-	-	-	-	-	-	-	-	-	-	-	-	-	-	-	-	-	-	-	-	-	-	-	-	-	+	-	-	-	-	-	-
6305	12069	hypothetical protein	1	-	-	-	-	-	-	-	-	-	-	-	-	-	-	-	-	-	-	-	-	-	-	-	-	-	-	-	-	-	-	-	-	-	-	-	-	-	-	-	-	-	-	-	-	-	-	-	-	-	-	+	-	-	-	-	-	-
6306	12070	hypothetical protein	1	-	-	-	-	-	-	-	-	-	-	-	-	-	-	-	-	-	-	-	-	-	-	-	-	-	-	-	-	-	-	-	-	-	-	-	-	-	-	-	-	-	-	-	-	-	-	-	-	-	-	+	-	-	-	-	-	-
6307	12071	hypothetical protein	1	-	-	-	-	-	-	-	-	-	-	-	-	-	-	-	-	-	-	-	-	-	-	-	-	-	-	-	-	-	-	-	-	-	-	-	-	-	-	-	-	-	-	-	-	-	-	-	-	-	-	+	-	-	-	-	-	-
6308	12072	hypothetical protein	1	-	-	-	-	-	-	-	-	-	-	-	-	-	-	-	-	-	-	-	-	-	-	-	-	-	-	-	-	-	-	-	-	-	-	-	-	-	-	-	-	-	-	-	-	-	-	-	-	-	-	+	-	-	-	-	-	-
6309	12073	hypothetical protein	1	-	-	-	-	-	-	-	-	-	-	-	-	-	-	-	-	-	-	-	-	-	-	-	-	-	-	-	-	-	-	-	-	-	-	-	-	-	-	-	-	-	-	-	-	-	-	-	-	-	-	+	-	-	-	-	-	-
6310	12074	hypothetical protein	1	-	-	-	-	-	-	-	-	-	-	-	-	-	-	-	-	-	-	-	-	-	-	-	-	-	-	-	-	-	-	-	-	-	-	-	-	-	-	-	+	-	-	-	-	-	-	-	-	-	-	-	-	-
6311	12075	hypothetical protein	1	-	-	-	-	-	-	-	-	-	-	-	-	-	-	-	-	-	-	-	-	-	-	-	-	-	-	-	-	-	-	-	-	-	-	-	-	-	-	-	+	-	-	-	-	-	-	-	-	-	-	-	-	-
6312	12076	hypothetical protein	1	-	-	-	-	-	-	-	-	-	-	-	-	-	-	-	-	-	-	-	-	-	-	-	-	-	-	-	-	-	-	-	-	-	-	-	-	-	-	-	+	-	-	-	-	-	-	-	-	-	-	-	-	-
6313	12077	hypothetical protein	1	-	-	-	-	-	-	-	-	-	-	-	-	-	-	-	-	-	-	-	-	-	-	-	-	-	-	-	-	-	-	-	-	-	-	-	-	-	-	-	+	-	-	-	-	-	-	-	-	-	-	-	-	-
6314	12078	hypothetical protein	1	-	-	-	-	-	-	-	-	-	-	-	-	-	-	-	-	-	-	-	-	-	-	-	-	-	-	-	-	-	-	-	-	-	-	-	-	-	-	-	+	-	-	-	-	-	-	-	-	-	-	-	-	-
6315	12079	putative inner membrane protein	1	-	-	-	-	-	-	-	-	-	-	-	-	-	-	-	-	-	-	-	-	-	-	-	-	-	-	-	-	-	-	-	-	-	-	-	-	-	-	-	+	-	-	-	-	-	-	-	-	-	-	-	-	-
6316	12080	Membrane protein involved in the export of O-antigen and teichoic acid	1	-	-	-	-	-	-	-	-	-	-	-	-	-	-	-	-	-	-	-	-	-	-	-	-	-	-	-	-	-	-	-	-	-	-	-	-	-	-	-	+	-	-	-	-	-	-	-	-	-	-	-	-	-
6317	12081	hypothetical protein	1	-	-	-	-	-	-	-	-	-	-	-	-	-	-	-	-	-	-	-	-	-	-	-	-	-	-	-	-	-	-	-	-	-	-	-	-	-	-	-	+	-	-	-	-	-	-	-	-	-	-	-	-	-
6318	12082	hypothetical protein	1	-	-	-	-	-	-	-	-	-	-	-	-	-	-	-	-	-	-	-	-	-	-	-	-	-	-	-	-	-	-	-	-	-	-	-	-	-	-	-	+	-	-	-	-	-	-	-	-	-	-	-	-	-
6319	12083	Lj928 prophage protein	1	-	-	-	-	-	-	-	-	-	-	-	-	-	-	-	-	-	-	-	-	-	-	-	-	-	-	-	-	-	-	-	-	-	-	-	-	-	-	-	+	-	-	-	-	-	-	-	-	-	-	-	-	-
6320	12084	Maltose O-acetyltransferase (EC 2.3.1.79)	1	-	-	-	-	-	-	-	-	-	-	-	-	-	-	-	-	-	-	-	-	-	-	-	-	-	-	-	-	-	-	-	-	-	-	-	-	-	-	-	+	-	-	-	-	-	-	-	-	-	-	-	-	-
6321	12085	hypothetical protein	1	-	-	-	-	-	-	-	-	-	-	-	-	-	-	-	-	-	-	-	-	-	-	-	-	-	-	-	-	-	-	-	-	-	-	-	-	-	-	-	+	-	-	-	-	-	-	-	-	-	-	-	-	-
6322	12086	hypothetical protein	1	-	-	-	-	-	-	-	-	-	-	-	-	-	-	-	-	-	-	-	-	-	-	-	-	-	-	-	-	-	-	-	-	-	-	-	-	-	-	-	+	-	-	-	-	-	-	-	-	-	-	-	-	-
6323	12087	hypothetical protein	1	-	-	-	-	-	-	-	-	-	-	-	-	-	-	-	-	-	-	-	-	-	-	-	-	-	-	-	-	-	-	-	-	-	-	-	-	-	-	-	+	-	-	-	-	-	-	-	-	-	-	-	-	-
6324	12088	hypothetical protein	1	-	-	-	-	-	-	-	-	-	-	-	-	-	-	-	-	-	-	-	-	-	-	-	-	-	-	-	-	-	-	-	-	-	-	-	-	-	-	-	+	-	-	-	-	-	-	-	-	-	-	-	-	-
6325	12089	hypothetical protein	1	-	-	-	-	-	-	-	-	-	-	-	-	-	-	-	-	-	-	-	-	-	-	-	-	-	-	-	-	-	-	-	-	-	-	-	-	-	-	-	+	-	-	-	-	-	-	-	-	-	-	-	-	-
6326	12090	Phage terminase	1	-	-	-	-	-	-	-	-	-	-	-	-	-	-	-	-	-	-	-	-	-	-	-	-	-	-	-	-	-	-	-	-	-	-	-	-	-	-	-	+	-	-	-	-	-	-	-	-	-	-	-	-	-
6327	12091	Phage minor tail protein	1	-	-	-	-	-	-	-	-	-	-	-	-	-	-	-	-	-	-	-	-	-	-	-	-	-	-	-	-	-	-	-	-	-	-	-	-	-	-	-	+	-	-	-	-	-	-	-	-	-	-	-	-	-
6328	12092	hypothetical protein	1	-	-	-	-	-	-	-	-	-	-	-	-	-	-	-	-	-	-	-	-	-	-	-	-	-	-	-	-	-	-	-	-	-	-	-	-	-	-	-	+	-	-	-	-	-	-	-	-	-	-	-	-	-
6329	12093	hypothetical protein	1	-	-	-	-	-	-	-	-	-	-	-	-	-	-	-	-	-	-	-	-	-	-	-	-	-	-	-	-	-	-	-	-	-	-	-	-	-	-	-	+	-	-	-	-	-	-	-	-	-	-	-	-	-
6330	12094	hypothetical protein	1	-	-	-	-	-	-	-	-	-	-	-	-	-	-	-	-	-	-	-	-	-	-	-	-	-	-	-	-	-	-	-	-	-	-	-	-	-	-	-	+	-	-	-	-	-	-	-	-	-	-	-	-	-
6331	12095	hypothetical protein	1	-	-	-	-	-	-	-	-	-	-	-	-	-	-	-	-	-	-	-	-	-	-	-	-	-	-	-	-	-	-	-	-	-	-	-	-	-	-	-	+	-	-	-	-	-	-	-	-	-	-	-	-	-
6332	12096	hypothetical protein	1	-	-	-	-	-	-	-	-	-	-	-	-	-	-	-	-	-	-	-	-	-	-	-	-	-	-	-	-	-	-	-	-	-	-	-	-	-	-	-	+	-	-	-	-	-	-	-	-	-	-	-	-	-
6333	12097	hypothetical protein	1	-	-	-	-	-	-	-	-	-	-	-	-	-	-	-	-	-	-	-	-	-	-	-	-	-	-	-	-	-	-	-	-	-	-	-	-	-	-	-	+	-	-	-	-	-	-	-	-	-	-	-	-	-
6334	12098	hypothetical protein	1	-	-	-	-	-	-	-	-	-	-	-	-	-	-	-	-	-	-	-	-	-	-	-	-	-	-	-	-	-	-	-	-	-	-	-	-	-	-	-	+	-	-	-	-	-	-	-	-	-	-	-	-	-
6335	12099	hypothetical protein	1	-	-	-	-	-	-	-	-	-	-	-	-	-	-	-	-	-	-	-	-	-	-	-	-	-	-	-	-	-	-	-	-	-	-	-	-	-	-	-	+	-	-	-	-	-	-	-	-	-	-	-	-	-
6336	12100	hypothetical protein	1	-	-	-	-	-	-	-	-	-	-	-	-	-	-	-	-	-	-	-	-	-	-	-	-	-	-	-	-	-	-	-	-	-	-	-	-	-	-	-	+	-	-	-	-	-	-	-	-	-	-	-	-	-
6337	12101	Glycosyltransferase(EC:2.4.1.-)	1	-	-	-	-	-	-	-	-	-	-	-	-	-	-	-	-	-	-	-	-	-	-	-	-	-	-	-	-	-	-	-	-	-	-	-	-	-	-	-	+	-	-	-	-	-	-	-	-	-	-	-	-	-
6338	12102	hypothetical protein	1	-	-	-	-	-	-	-	-	-	-	-	-	-	-	-	-	-	-	-	-	-	-	-	-	-	-	-	-	-	-	-	-	-	-	-	-	-	-	-	+	-	-	-	-	-	-	-	-	-	-	-	-	-
6339	12103	hypothetical protein	1	-	-	-	-	-	-	-	-	-	-	-	-	-	-	-	-	-	-	-	-	-	-	-	-	-	-	-	-	-	-	-	-	-	-	-	-	-	-	-	+	-	-	-	-	-	-	-	-	-	-	-	-	-
6340	12104	hypothetical protein	1	-	-	-	-	-	-	-	-	-	-	-	-	-	-	-	-	-	-	-	-	-	-	-	-	-	-	-	-	-	-	-	-	-	-	-	-	-	-	-	+	-	-	-	-	-	-	-	-	-	-	-	-	-
6341	12105	putative glucose transferase	1	-	-	-	-	-	-	-	-	-	-	-	-	-	-	-	-	-	-	-	-	-	-	-	-	-	-	-	-	-	-	-	-	-	-	-	-	-	-	-	+	-	-	-	-	-	-	-	-	-	-	-	-	-
6342	12106	hypothetical protein	1	-	-	-	-	-	-	-	-	-	-	-	-	-	-	-	-	-	-	-	-	-	-	-	-	-	-	-	-	-	-	-	-	-	-	-	-	-	-	-	+	-	-	-	-	-	-	-	-	-	-	-	-	-
6343	12107	Helix-turn-helix domain protein	1	-	-	-	-	-	-	-	-	-	-	-	-	-	-	-	-	-	-	-	-	-	-	-	-	-	-	-	-	-	-	-	-	-	-	-	-	-	-	-	+	-	-	-	-	-	-	-	-	-	-	-	-	-
6344	12108	putative lincosamide nucleotidyltransferase	1	-	-	-	-	-	-	-	-	-	-	-	-	-	-	-	-	-	-	-	-	-	-	-	-	-	-	-	-	-	-	-	-	-	-	-	-	-	-	-	+	-	-	-	-	-	-	-	-	-	-	-	-	-
6345	12109	hypothetical protein	1	-	-	-	-	-	-	-	-	-	-	-	-	-	-	-	-	-	-	-	-	-	-	-	-	-	-	-	-	-	-	-	-	-	-	-	-	-	-	-	+	-	-	-	-	-	-	-	-	-	-	-	-	-
6346	12110	hypothetical protein	1	-	-	-	-	-	-	-	-	-	-	-	-	-	-	-	-	-	-	-	-	-	-	-	-	-	-	-	-	-	-	-	-	-	-	-	-	-	-	-	+	-	-	-	-	-	-	-	-	-	-	-	-	-
6347	12111	hypothetical protein	1	-	-	-	-	-	-	-	-	-	-	-	-	-	-	-	-	-	-	-	-	-	-	-	-	-	-	-	-	-	-	-	-	-	-	-	-	-	-	-	+	-	-	-	-	-	-	-	-	-	-	-	-	-
6348	12112	SNF2 family protein	1	-	-	-	-	-	-	-	-	-	-	-	-	-	-	-	-	-	-	-	-	-	-	-	-	-	-	-	-	-	-	-	-	-	-	-	-	-	-	-	+	-	-	-	-	-	-	-	-	-	-	-	-	-
6349	12113	hypothetical protein	1	-	-	-	-	-	-	-	-	-	-	-	-	-	-	-	-	-	-	-	-	-	-	-	-	-	-	-	-	-	-	-	-	-	-	-	-	-	-	-	+	-	-	-	-	-	-	-	-	-	-	-	-	-
6350	12114	endonuclease III domain protein	1	-	-	-	-	-	-	-	-	-	-	-	-	-	-	-	-	-	-	-	-	-	-	-	-	-	-	-	-	-	-	-	-	-	-	-	-	-	-	-	+	-	-	-	-	-	-	-	-	-	-	-	-	-
6351	12115	hypothetical protein	1	-	-	-	-	-	-	-	-	-	-	-	-	-	-	-	-	-	-	-	-	-	-	-	-	-	-	-	-	-	-	-	-	-	-	-	-	-	-	-	+	-	-	-	-	-	-	-	-	-	-	-	-	-
6352	12116	hypothetical protein	1	-	-	-	-	-	-	-	-	-	-	-	-	-	-	-	-	-	-	-	-	-	-	-	-	-	-	-	-	-	-	-	-	-	-	-	-	-	-	-	+	-	-	-	-	-	-	-	-	-	-	-	-	-
6353	12117	hypothetical protein	1	-	-	-	-	-	-	-	-	-	-	-	-	-	-	-	-	-	-	-	-	-	-	-	-	-	-	-	-	-	-	-	-	-	-	-	-	-	-	-	+	-	-	-	-	-	-	-	-	-	-	-	-	-
6354	12118	Mobile element protein	1	-	-	-	-	-	-	-	-	-	-	-	-	-	-	-	-	-	-	-	-	-	-	-	-	-	-	-	-	-	-	-	-	-	-	-	-	-	-	-	+	-	-	-	-	-	-	-	-	-	-	-	-	-
6355	12119	hypothetical protein	1	-	-	-	-	-	-	-	-	-	-	-	-	-	-	-	-	-	-	-	-	-	-	-	-	-	-	-	-	-	-	-	-	-	-	-	-	-	-	-	+	-	-	-	-	-	-	-	-	-	-	-	-	-
6356	12120	hypothetical protein	1	-	-	-	-	-	-	-	-	-	-	-	-	-	-	-	-	-	-	-	-	-	-	-	-	-	-	-	-	-	-	-	-	-	-	-	-	-	-	-	+	-	-	-	-	-	-	-	-	-	-	-	-	-
6357	12121	hypothetical protein	1	-	-	-	-	-	-	-	-	-	-	-	-	-	-	-	-	-	-	-	-	-	-	-	-	-	-	-	-	-	-	-	-	-	-	-	-	-	-	-	+	-	-	-	-	-	-	-	-	-	-	-	-	-
6358	12122	hypothetical protein	1	-	-	-	-	-	-	-	-	-	-	-	-	-	-	-	-	-	-	-	-	-	-	-	-	-	-	-	-	-	-	-	-	-	-	-	-	-	-	-	+	-	-	-	-	-	-	-	-	-	-	-	-	-
6359	12123	hypothetical protein	1	-	-	-	-	-	-	-	-	-	-	-	-	-	-	-	-	-	-	-	-	-	-	-	-	-	-	-	-	-	-	-	-	-	-	-	-	-	-	-	+	-	-	-	-	-	-	-	-	-	-	-	-	-
6360	12124	hypothetical protein	1	-	-	-	-	-	-	-	-	-	-	-	-	-	-	-	-	-	-	-	-	-	-	-	-	-	-	-	-	-	-	-	-	-	-	-	-	-	-	-	+	-	-	-	-	-	-	-	-	-	-	-	-	-
6361	12125	DinG family ATP-dependent helicase YoaA	1	-	-	-	-	-	-	-	-	-	-	-	-	-	-	-	-	-	-	-	-	-	-	-	-	-	-	-	-	-	-	-	-	-	-	-	-	-	-	-	+	-	-	-	-	-	-	-	-	-	-	-	-	-
6362	12126	hypothetical protein	1	-	-	-	-	-	-	-	-	-	-	-	-	-	-	-	-	-	-	-	-	-	-	-	-	-	-	-	-	-	-	-	-	-	-	-	-	-	-	-	+	-	-	-	-	-	-	-	-	-	-	-	-	-
6363	12127	hypothetical protein	1	-	-	-	-	-	-	-	-	-	-	-	-	-	-	-	-	-	-	-	-	-	-	-	-	-	-	-	-	-	-	-	-	-	-	-	-	-	-	-	+	-	-	-	-	-	-	-	-	-	-	-	-	-
6364	12128	Glycosyltransferase (EC 2.4.1.-)	1	-	-	-	-	-	-	-	-	-	-	-	-	-	-	-	-	-	-	-	-	-	-	-	-	-	-	-	-	-	-	-	-	-	-	-	-	-	-	-	+	-	-	-	-	-	-	-	-	-	-	-	-	-
6365	12129	hypothetical protein	1	-	-	-	-	-	-	-	-	-	-	-	-	-	-	-	-	-	-	-	-	-	-	-	-	-	-	-	-	-	-	-	-	-	-	-	-	-	-	-	+	-	-	-	-	-	-	-	-	-	-	-	-	-
6366	12130	ABC transporter	1	-	-	-	-	-	-	-	-	-	-	-	-	-	-	-	-	-	-	-	-	-	-	-	-	-	-	-	-	-	-	-	-	-	-	-	-	-	-	-	+	-	-	-	-	-	-	-	-	-	-	-	-	-
6367	12131	hypothetical protein	1	-	-	-	-	-	-	-	-	-	-	-	-	-	-	-	-	-	-	-	-	-	-	-	-	-	-	-	-	-	-	-	-	-	-	-	-	-	-	-	+	-	-	-	-	-	-	-	-	-	-	-	-	-
6368	12132	hypothetical protein	1	-	-	-	-	-	-	-	-	-	-	-	-	-	-	-	-	-	-	-	-	-	-	-	-	-	-	-	-	-	-	-	-	-	-	-	-	-	-	-	+	-	-	-	-	-	-	-	-	-	-	-	-	-
6369	12133	hypothetical protein	1	-	-	-	-	-	-	-	-	-	-	-	-	-	-	-	-	-	-	-	-	-	-	-	-	-	-	-	-	-	-	-	-	-	-	-	-	-	-	-	+	-	-	-	-	-	-	-	-	-	-	-	-	-
6370	12134	hypothetical protein	1	-	-	-	-	-	-	-	-	-	-	-	-	-	-	-	-	-	-	-	-	-	-	-	-	-	-	-	-	-	-	-	-	-	-	-	-	-	-	-	+	-	-	-	-	-	-	-	-	-	-	-	-	-
6371	12135	hypothetical protein	1	-	-	-	-	-	-	-	-	-	-	-	-	-	-	-	-	-	-	-	-	-	-	-	-	-	-	-	-	-	-	-	-	-	-	-	-	-	-	-	+	-	-	-	-	-	-	-	-	-	-	-	-	-
6372	12136	hypothetical protein	1	-	-	-	-	-	-	-	-	-	-	-	-	-	-	-	-	-	-	-	-	-	-	-	-	-	-	-	-	-	-	-	-	-	-	-	-	-	-	-	+	-	-	-	-	-	-	-	-	-	-	-	-	-
6373	12137	hypothetical protein	1	-	-	-	-	-	-	-	-	-	-	-	-	-	-	-	-	-	-	-	-	-	-	-	-	-	-	-	-	-	-	-	-	-	-	-	-	-	-	-	+	-	-	-	-	-	-	-	-	-	-	-	-	-
6374	12138	hypothetical protein	1	-	-	-	-	-	-	-	-	-	-	-	-	-	-	-	-	-	-	-	-	-	-	-	-	-	-	-	-	-	-	-	-	-	-	-	-	-	-	-	-	-	-	-	-	-	-	-	-	-	-	+	-	-	-
6375	12139	hypothetical protein	1	-	-	-	-	-	-	-	-	-	-	-	-	-	-	-	-	-	-	-	-	-	-	-	-	-	-	-	-	-	-	-	-	-	-	-	-	-	-	-	-	-	-	-	-	-	-	-	-	-	-	+	-	-	-
6376	12140	hypothetical protein	1	-	-	-	-	-	-	-	-	-	-	-	-	-	-	-	-	-	-	-	-	-	-	-	-	-	-	-	-	-	-	-	-	-	-	-	-	-	-	-	-	-	-	-	-	-	-	-	-	-	-	+	-	-	-
6377	12141	hypothetical protein	1	-	-	-	-	-	-	-	-	-	-	-	-	-	-	-	-	-	-	-	-	-	-	-	-	-	-	-	-	-	-	-	-	-	-	-	-	-	-	-	-	-	-	-	-	-	-	-	-	-	-	+	-	-	-
6378	12142	hypothetical protein	1	-	-	-	-	-	-	-	-	-	-	-	-	-	-	-	-	-	-	-	-	-	-	-	-	-	-	-	-	-	-	-	-	-	-	-	-	-	-	-	-	-	-	-	-	-	-	-	-	-	-	+	-	-	-
6379	12143	hypothetical protein	1	-	-	-	-	-	-	-	-	-	-	-	-	-	-	-	-	-	-	-	-	-	-	-	-	-	-	-	-	-	-	-	-	-	-	-	-	-	-	-	-	-	-	-	-	-	-	-	-	-	-	+	-	-	-
6380	12144	hypothetical protein	1	-	-	-	-	-	-	-	-	-	-	-	-	-	-	-	-	-	-	-	-	-	-	-	-	-	-	-	-	-	-	-	-	-	-	-	-	-	-	-	-	-	-	-	-	-	-	-	-	-	-	+	-	-	-
6381	12145	Diaminopimelate decarboxylase (EC 4.1.1.20)	1	-	-	-	-	-	-	-	-	-	-	-	-	-	-	-	-	-	-	-	-	-	-	-	-	-	-	-	-	-	-	-	-	-	-	-	-	-	-	-	-	-	-	-	-	-	-	-	-	-	-	+	-	-	-
6382	12146	hypothetical protein	1	-	-	-	-	-	-	-	-	-	-	-	-	-	-	-	-	-	-	-	-	-	-	-	-	-	-	-	-	-	-	-	-	-	-	-	-	-	-	-	-	-	-	-	-	-	-	-	-	-	-	+	-	-	-
6383	12147	hypothetical protein	1	-	-	-	-	-	-	-	-	-	-	-	-	-	-	-	-	-	-	-	-	-	-	-	-	-	-	-	-	-	-	-	-	-	-	-	-	-	-	-	-	-	-	-	-	-	-	-	-	-	-	+	-	-	-
6384	12148	adenine/cytosine DNA methyltransferase	1	-	-	-	-	-	-	-	-	-	-	-	-	-	-	-	-	-	-	-	-	-	-	-	-	-	-	-	-	-	-	-	-	-	-	-	-	-	-	-	-	-	-	-	-	-	-	-	-	-	-	+	-	-	-
6385	12149	hypothetical protein	1	-	-	-	-	-	-	-	-	-	-	-	-	-	-	-	-	-	-	-	-	-	-	-	-	-	-	-	-	-	-	-	-	-	-	-	-	-	-	-	-	-	-	-	-	-	-	-	-	-	-	+	-	-	-
6386	12150	hypothetical protein	1	-	-	-	-	-	-	-	-	-	-	-	-	-	-	-	-	-	-	-	-	-	-	-	-	-	-	-	-	-	-	-	-	-	-	-	-	-	-	-	-	-	-	-	-	-	-	-	-	-	-	+	-	-	-
6387	12151	hypothetical protein	1	-	-	-	-	-	-	-	-	-	-	-	-	-	-	-	-	-	-	-	-	-	-	-	-	-	-	-	-	-	-	-	-	-	-	-	-	-	-	-	-	-	-	-	-	-	-	-	-	-	-	+	-	-	-

Table S5. Precence/absence of each HG among 57 *S. suis* strains

				1																13			25					28						104		94			76			53/54		Divergent *S. suis*																
Index[a]	ClutserID	Gene products	Members	98HAH33	05ZYH33	SC84	A7	SS12	P1/7	DAT273	NIAH11434	JS14	GZ1	BM407	S735	13730	MNCM07	NCTC 10446	14636	NCTC 10237	ST1	2651	89-1591	MNCM04	MNCM25	8074	D9	4961	ST3	MNCM43	DAT292	DAT260	NIAH11435	MNCM21	MNCM50	89-2479	DAT234	DAT290	93A	42A	NT77	6407	11538	8830	12814	92-1400	89-590	2726	2524	89-3576-3	4417	D12	10581	89-5259	88-5299A	R61	92-1191	14A	92-4172	22083
6388	12152	ABC transporter permease protein	1	-	-	-	-	-	-	-	-	-	-	-	-	-	-	-	-	-	-	-	-	-	-	-	-	-	-	-	-	-	-	-	-	-	-	-	-	-	-	-	-	-	-	-	-	-	-	-	-	-	+	-	-	-
6389	12153	lipase	1	-	-	-	-	-	-	-	-	-	-	-	-	-	-	-	-	-	-	-	-	-	-	-	-	-	-	-	-	-	-	-	-	-	-	-	-	-	-	-	-	-	-	-	-	-	-	-	-	-	+	-	-	-
6390	12154	hypothetical protein	1	-	-	-	-	-	-	-	-	-	-	-	-	-	-	-	-	-	-	-	-	-	-	-	-	-	-	-	-	-	-	-	-	-	-	-	-	-	-	-	-	-	-	-	-	-	-	-	-	-	+	-	-	-
6391	12155	hypothetical protein	1	-	-	-	-	-	-	-	-	-	-	-	-	-	-	-	-	-	-	-	-	-	-	-	-	-	-	-	-	-	-	-	-	-	-	-	-	-	-	-	-	-	-	-	-	-	-	-	-	-	+	-	-	-
6392	12156	hypothetical protein	1	-	-	-	-	-	-	-	-	-	-	-	-	-	-	-	-	-	-	-	-	-	-	-	-	-	-	-	-	-	-	-	-	-	-	-	-	-	-	-	-	-	-	-	-	-	-	-	-	-	+	-	-	-
6393	12157	cI-like repressor	1	-	-	-	-	-	-	-	-	-	-	-	-	-	-	-	-	-	-	-	-	-	-	-	-	-	-	-	-	-	-	-	-	-	-	-	-	-	-	-	-	-	-	-	-	-	-	-	-	-	+	-	-	-
6394	12158	nitroreductase family protein	1	-	-	-	-	-	-	-	-	-	-	-	-	-	-	-	-	-	-	-	-	-	-	-	-	-	-	-	-	-	-	-	-	-	-	-	-	-	-	-	-	-	-	-	-	-	-	-	-	-	+	-	-	-
6395	12159	hypothetical protein	1	-	-	-	-	-	-	-	-	-	-	-	-	-	-	-	-	-	-	-	-	-	-	-	-	-	-	-	-	-	-	-	-	-	-	-	-	-	-	-	-	-	-	-	-	-	-	-	-	-	+	-	-	-
6396	12160	hypothetical protein	1	-	-	-	-	-	-	-	-	-	-	-	-	-	-	-	-	-	-	-	-	-	-	-	-	-	-	-	-	-	-	-	-	-	-	-	-	-	-	-	-	-	-	-	-	-	-	-	-	-	+	-	-	-
6397	12161	Zinc metalloprotease zmpC precursor	1	-	-	-	-	-	-	-	-	-	-	-	-	-	-	-	-	-	-	-	-	-	-	-	-	-	-	-	-	-	-	-	-	-	-	-	-	-	-	-	-	-	-	-	-	-	-	-	-	-	+	-	-	-
6398	12162	hypothetical protein	1	-	-	-	-	-	-	-	-	-	-	-	-	-	-	-	-	-	-	-	-	-	-	-	-	-	-	-	-	-	-	-	-	-	-	-	-	-	-	-	-	-	-	-	-	-	-	-	-	-	+	-	-	-
6399	12163	hypothetical protein	1	-	-	-	-	-	-	-	-	-	-	-	-	-	-	-	-	-	-	-	-	-	-	-	-	-	-	-	-	-	-	-	-	-	-	-	-	-	-	-	-	-	-	-	-	-	-	-	-	-	+	-	-	-
6400	12164	hypothetical protein	1	-	-	-	-	-	-	-	-	-	-	-	-	-	-	-	-	-	-	-	-	-	-	-	-	-	-	-	-	-	-	-	-	-	-	-	-	-	-	-	-	-	-	-	-	-	-	-	-	-	+	-	-	-
6401	12165	hypothetical protein	1	-	-	-	-	-	-	-	-	-	-	-	-	-	-	-	-	-	-	-	-	-	-	-	-	-	-	-	-	-	-	-	-	-	-	-	-	-	-	-	-	-	-	-	-	-	-	-	-	-	+	-	-	-
6402	12166	hypothetical protein	1	-	-	-	-	-	-	-	-	-	-	-	-	-	-	-	-	-	-	-	-	-	-	-	-	-	-	-	-	-	-	-	-	-	-	-	-	-	-	-	-	-	-	-	-	-	-	-	-	-	+	-	-	-
6403	12167	hypothetical protein	1	-	-	-	-	-	-	-	-	-	-	-	-	-	-	-	-	-	-	-	-	-	-	-	-	-	-	-	-	-	-	-	-	-	-	-	-	-	-	-	-	-	-	-	-	-	-	-	-	-	+	-	-	-
6404	12168	Putative RTX family exoprotein A gene	1	-	-	-	-	-	-	-	-	-	-	-	-	-	-	-	-	-	-	-	-	-	-	-	-	-	-	-	-	-	-	-	-	-	-	-	-	-	-	-	-	-	-	-	-	-	-	-	-	-	+	-	-	-
6405	12169	hypothetical protein	1	-	-	-	-	-	-	-	-	-	-	-	-	-	-	-	-	-	-	-	-	-	-	-	-	-	-	-	-	-	-	-	-	-	-	-	-	-	-	-	-	-	-	-	-	-	-	-	-	-	+	-	-	-
6406	12170	hypothetical protein	1	-	-	-	-	-	-	-	-	-	-	-	-	-	-	-	-	-	-	-	-	-	-	-	-	-	-	-	-	-	-	-	-	-	-	-	-	-	-	-	-	-	-	-	-	-	-	-	-	-	+	-	-	-
6407	12171	hypothetical protein	1	-	-	-	-	-	-	-	-	-	-	-	-	-	-	-	-	-	-	-	-	-	-	-	-	-	-	-	-	-	-	-	-	-	-	-	-	-	-	-	-	-	-	-	-	-	-	-	-	-	+	-	-	-
6408	12172	IS66 family element	1	-	-	-	-	-	-	-	-	-	-	-	-	-	-	-	-	-	-	-	-	-	-	-	-	-	-	-	-	-	-	-	-	-	-	-	-	-	-	-	-	-	-	-	-	-	-	-	-	-	+	-	-	-
6409	12173	hypothetical protein	1	-	-	-	-	-	-	-	-	-	-	-	-	-	-	-	-	-	-	-	-	-	-	-	-	-	-	-	-	-	-	-	-	-	-	-	-	-	-	-	-	-	-	-	-	-	-	-	-	-	+	-	-	-
6410	12174	hypothetical protein	1	-	-	-	-	-	-	-	-	-	-	-	-	-	-	-	-	-	-	-	-	-	-	-	-	-	-	-	-	-	-	-	-	-	-	-	-	-	-	-	-	-	-	-	-	-	-	-	-	-	+	-	-	-
6411	12175	hypothetical protein	1	-	-	-	-	-	-	-	-	-	-	-	-	-	-	-	-	-	-	-	-	-	-	-	-	-	-	-	-	-	-	-	-	-	-	-	-	-	-	-	-	-	-	-	-	-	-	-	-	-	+	-	-	-
6412	12176	ADA regulatory protein	1	-	-	-	-	-	-	-	-	-	-	-	-	-	-	-	-	-	-	-	-	-	-	-	-	-	-	-	-	-	-	-	-	-	-	-	-	-	-	-	-	-	-	-	-	-	-	-	-	-	+	-	-	-
6413	12177	hypothetical protein	1	-	-	-	-	-	-	-	-	-	-	-	-	-	-	-	-	-	-	-	-	-	-	-	-	-	-	-	-	-	-	-	-	-	-	-	-	-	-	-	-	-	-	-	-	-	-	-	-	-	+	-	-	-
6414	12178	putative chromosome segregation ATPase	1	-	-	-	-	-	-	-	-	-	-	-	-	-	-	-	-	-	-	-	-	-	-	-	-	-	-	-	-	-	-	-	-	-	-	-	-	-	-	-	-	-	-	-	-	-	-	-	-	-	+	-	-	-
6415	12179	hypothetical protein	1	-	-	-	-	-	-	-	-	-	-	-	-	-	-	-	-	-	-	-	-	-	-	-	-	-	-	-	-	-	-	-	-	-	-	-	-	-	-	-	-	-	-	-	-	-	-	-	-	-	+	-	-	-
6416	12180	hypothetical protein	1	-	-	-	-	-	-	-	-	-	-	-	-	-	-	-	-	-	-	-	-	-	-	-	-	-	-	-	-	-	-	-	-	-	-	-	-	-	-	-	-	-	-	-	-	-	-	-	-	-	+	-	-	-
6417	12181	hypothetical protein	1	-	-	-	-	-	-	-	-	-	-	-	-	-	-	-	-	-	-	-	-	-	-	-	-	-	-	-	-	-	-	-	-	-	-	-	-	-	-	-	-	-	-	-	-	-	-	-	-	-	+	-	-	-
6418	12182	hypothetical protein	1	-	-	-	-	-	-	-	-	-	-	-	-	-	-	-	-	-	-	-	-	-	-	-	-	-	-	-	-	-	-	-	-	-	-	-	-	-	-	-	-	-	-	-	-	-	-	-	-	-	+	-	-	-
6419	12183	hypothetical protein	1	-	-	-	-	-	-	-	-	-	-	-	-	-	-	-	-	-	-	-	-	-	-	-	-	-	-	-	-	-	-	-	-	-	-	-	-	-	-	-	-	-	-	-	-	-	-	-	-	-	+	-	-	-
6420	12184	hypothetical protein	1	-	-	-	-	-	-	-	-	-	-	-	-	-	-	-	-	-	-	-	-	-	-	-	-	-	-	-	-	-	-	-	-	-	-	-	-	-	-	-	-	-	-	-	-	-	-	-	-	-	+	-	-	-
6421	12185	hypothetical protein	1	-	-	-	-	-	-	-	-	-	-	-	-	-	-	-	-	-	-	-	-	-	-	-	-	-	-	-	-	-	-	-	-	-	-	-	-	-	-	-	-	-	-	-	-	-	-	-	-	-	+	-	-	-
6422	12186	Glycosyltransferase (EC 2.4.1.-)	1	-	-	-	-	-	-	-	-	-	-	-	-	-	-	-	-	-	-	-	-	-	-	-	-	-	-	-	-	-	-	-	-	-	-	-	-	-	-	-	-	-	-	-	-	-	-	-	-	-	+	-	-	-
6423	12187	hypothetical protein	1	-	-	-	-	-	-	-	-	-	-	-	-	-	-	-	-	-	-	-	-	-	-	-	-	-	-	-	-	-	-	-	-	-	-	-	-	-	-	-	-	-	-	-	-	-	-	-	-	-	+	-	-	-
6424	12188	hypothetical protein	1	-	-	-	-	-	-	-	-	-	-	-	-	-	-	-	-	-	-	-	-	-	-	-	-	-	-	-	-	-	-	-	-	-	-	-	-	-	-	-	-	-	-	-	-	-	-	-	-	-	+	-	-	-
6425	12189	hypothetical protein	1	-	-	-	-	-	-	-	-	-	-	-	-	-	-	-	-	-	-	-	-	-	-	-	-	-	-	-	-	-	-	-	-	-	-	-	-	-	-	-	-	-	-	-	-	-	-	-	-	-	+	-	-	-
6426	12190	COG4260: Putative virion core protein (lumpy skin disease virus)	1	-	-	-	-	-	-	-	-	-	-	-	-	-	-	-	-	-	-	-	-	-	-	-	-	-	-	-	-	-	-	-	-	-	-	-	-	-	-	-	-	-	-	-	-	-	-	-	-	-	+	-	-	-
6427	12191	hypothetical protein	1	-	-	-	-	-	-	-	-	-	-	-	-	-	-	-	-	-	-	-	-	-	-	-	-	-	-	-	-	-	-	-	-	-	-	-	-	-	-	-	-	-	-	-	-	-	-	-	-	-	+	-	-	-
6428	12192	membrane protein	1	-	-	-	-	-	-	-	-	-	-	-	-	-	-	-	-	-	-	-	-	-	-	-	-	-	-	-	-	-	-	-	-	-	-	-	-	-	-	-	-	-	-	-	-	-	-	-	-	-	+	-	-	-
6429	12193	hypothetical protein	1	-	-	-	-	-	-	-	-	-	-	-	-	-	-	-	-	-	-	-	-	-	-	-	-	-	-	-	-	-	-	-	-	-	-	-	-	-	-	-	-	-	-	-	-	-	-	-	-	-	+	-	-	-
6430	12194	hypothetical protein	1	-	-	-	-	-	-	-	-	-	-	-	-	-	-	-	-	-	-	-	-	-	-	-	-	-	-	-	-	-	-	-	-	-	-	-	-	-	-	-	-	-	-	-	-	-	-	-	-	-	+	-	-	-
6431	12195	hypothetical protein	1	-	-	-	-	-	-	-	-	-	-	-	-	-	-	-	-	-	-	-	-	-	-	-	-	-	-	-	-	-	-	-	-	-	-	-	-	-	-	-	-	-	-	-	-	-	-	-	-	-	+	-	-	-
6432	12196	FIG131328: Predicted ATP-dependent endonuclease of the OLD family	1	-	-	-	-	-	-	-	-	-	-	-	-	-	-	-	-	-	-	-	-	-	-	-	-	-	-	-	-	-	-	-	-	-	-	-	-	-	-	-	-	-	-	-	-	-	-	-	-	-	+	-	-	-
6433	12197	hypothetical protein	1	-	-	-	-	-	-	-	-	-	-	-	-	-	-	-	-	-	-	-	-	-	-	-	-	-	-	-	-	-	-	-	-	-	-	-	-	-	-	-	-	-	-	-	-	-	-	-	-	-	+	-	-	-
6434	12198	Transcriptional regulator	1	-	-	-	-	-	-	-	-	-	-	-	-	-	-	-	-	-	-	-	-	-	-	-	-	-	-	-	-	-	-	-	-	-	-	-	-	-	-	-	-	-	-	-	-	-	-	-	-	-	+	-	-	-
6435	12199	hypothetical protein	1	-	-	-	-	-	-	-	-	-	-	-	-	-	-	-	-	-	-	-	-	-	-	-	-	-	-	-	-	-	-	-	-	-	-	-	-	-	-	-	-	-	-	-	-	-	-	-	-	-	+	-	-	-
6436	12200	hypothetical protein	1	-	-	-	-	-	-	-	-	-	-	-	-	-	-	-	-	-	-	-	-	-	-	-	-	-	-	-	-	-	-	-	-	-	-	-	-	-	-	-	-	-	-	-	-	-	-	-	-	-	+	-	-	-
6437	12201	hypothetical protein	1	-	-	-	-	-	-	-	-	-	-	-	-	-	-	-	-	-	-	-	-	-	-	-	-	-	-	-	-	-	-	-	-	-	-	-	-	-	-	-	-	-	-	-	-	-	-	-	-	-	+	-	-	-
6438	12202	hypothetical protein	1	-	-	-	-	-	-	-	-	-	-	-	-	-	-	-	-	-	-	-	-	-	-	-	-	-	-	-	-	-	-	-	-	-	-	-	-	-	-	-	-	-	-	-	-	-	-	-	-	-	+	-	-	-
6439	12203	hypothetical protein	1	-	-	-	-	-	-	-	-	-	-	-	-	-	-	-	-	-	-	-	-	-	-	-	-	-	-	-	-	-	-	-	-	-	-	-	-	-	-	-	-	-	-	-	-	-	-	-	-	-	+	-	-	-
6440	12204	hypothetical protein	1	-	-	-	-	-	-	-	-	-	-	-	-	-	-	-	-	-	-	-	-	-	-	-	-	-	-	-	-	-	-	-	-	-	-	-	-	-	-	-	-	-	-	-	-	-	-	-	-	-	+	-	-	-
6441	12205	hypothetical protein	1	-	-	-	-	-	-	-	-	-	-	-	-	-	-	-	-	-	-	-	-	-	-	-	-	-	-	-	-	-	-	-	-	-	-	-	-	-	-	-	-	-	-	-	-	-	-	-	-	-	+	-	-	-
6442	12206	hypothetical protein	1	-	-	-	-	-	-	-	-	-	-	-	-	-	-	-	-	-	-	-	-	-	-	-	-	-	-	-	-	-	-	-	-	-	-	-	-	-	-	-	-	-	-	-	-	-	-	-	-	-	+	-	-	-
6443	12207	hypothetical protein	1	-	-	-	-	-	-	-	-	-	-	-	-	-	-	-	-	-	-	-	-	-	-	-	-	-	-	-	-	-	-	-	-	-	-	-	-	-	-	-	-	-	-	-	-	-	-	-	-	-	+	-	-	-
6444	12208	hypothetical protein	1	-	-	-	-	-	-	-	-	-	-	-	-	-	-	-	-	-	-	-	-	-	-	-	-	-	-	-	-	-	-	-	-	-	-	-	-	-	-	-	-	-	-	-	-	-	-	-	-	-	+	-	-	-
6445	12209	immunity protein	1	-	-	-	-	-	-	-	-	-	-	-	-	-	-	-	-	-	-	-	-	-	-	-	-	-	-	-	-	-	-	-	-	-	-	-	-	-	-	-	-	-	-	-	-	-	-	-	-	-	+	-	-	-
6446	12210	hypothetical protein	1	-	-	-	-	-	-	-	-	-	-	-	-	-	-	-	-	-	-	-	-	-	-	-	-	-	-	-	-	-	-	-	-	-	-	-	-	-	-	-	-	-	-	-	-	-	-	-	-	-	+	-	-	-
6447	12211	hypothetical protein	1	-	-	-	-	-	-	-	-	-	-	-	-	-	-	-	-	-	-	-	-	-	-	-	-	-	-	-	-	-	-	-	-	-	-	-	-	-	-	-	-	-	-	-	-	-	-	-	-	-	+	-	-	-
6448	12212	hypothetical protein	1	-	-	-	-	-	-	-	-	-	-	-	-	-	-	-	-	-	-	-	-	-	-	-	-	-	-	-	-	-	-	-	-	-	-	-	-	-	-	-	-	-	-	-	-	-	-	-	-	-	+	-	-	-
6449	12213	hypothetical protein	1	-	-	-	-	-	-	-	-	-	-	-	-	-	-	-	-	-	-	-	-	-	-	-	-	-	-	-	-	-	-	-	-	-	-	-	-	-	-	-	-	-	-	-	-	-	-	-	-	-	+	-	-	-
6450	12214	Hydrolase (HAD superfamily)	1	-	-	-	-	-	-	-	-	-	-	-	-	-	-	-	-	-	-	-	-	-	-	-	-	-	-	-	-	-	-	-	-	-	-	-	-	-	-	-	-	-	-	-	-	-	-	-	-	-	+	-	-	-
6451	12215	Capsular polysaccharide synthesis enzyme Cap5L	1	-	-	-	-	-	-	-	-	-	-	-	-	-	-	-	-	-	-	-	-	-	-	-	-	-	-	-	-	-	-	-	-	-	-	-	-	-	-	-	-	-	-	-	-	-	-	-	-	-	+	-	-	-
6452	12216	hypothetical protein	1	-	-	-	-	-	-	-	-	-	-	-	-	-	-	-	-	-	-	-	-	-	-	-	-	-	-	-	-	-	-	-	-	-	-	-	-	-	-	-	-	-	-	-	-	-	-	-	-	-	+	-	-	-
6453	12217	hypothetical protein	1	-	-	-	-	-	-	-	-	-	-	-	-	-	-	-	-	-	-	-	-	-	-	-	-	-	-	-	-	-	-	-	-	-	-	-	-	-	-	-	-	-	-	-	-	-	-	-	-	-	+	-	-	-
6454	12218	hypothetical protein	1	-	-	-	-	-	-	-	-	-	-	-	-	-	-	-	-	-	-	-	-	-	-	-	-	-	-	-	-	-	-	-	-	-	-	-	-	-	-	-	-	-	-	-	-	-	-	-	-	-	+	-	-	-
6455	12219	Modification methylase HgaIA (EC 2.1.1.37) (Cytosine-specific methyltransferase HgaIA) (M.HgaIA) (M.HgaI-1)	1	-	-	-	-	-	-	-	-	-	-	-	-	-	-	-	-	-	-	-	-	-	-	-	-	-	-	-	-	-	-	-	-	-	-	-	-	-	-	-	-	-	-	-	-	-	-	-	-	-	+	-	-	-
6456	12220	hypothetical protein	1	-	-	-	-	-	-	-	-	-	-	-	-	-	-	-	-	-	-	-	-	-	-	-	-	-	-	-	-	-	-	-	-	-	-	-	-	-	-	-	-	-	-	-	-	-	-	-	-	-	+	-	-	-
6457	12221	putative prophage encoded two-component system response regulator	1	-	-	-	-	-	-	-	-	-	-	-	-	-	-	-	-	-	-	-	-	-	-	-	-	-	-	-	-	-	-	-	-	-	-	-	-	-	-	-	-	-	-	-	-	-	-	-	-	-	+	-	-	-
6458	12222	hypothetical protein	1	-	-	-	-	-	-	-	-	-	-	-	-	-	-	-	-	-	-	-	-	-	-	-	-	-	-	-	-	-	-	-	-	-	-	-	-	-	-	-	-	-	-	-	-	-	-	-	-	-	+	-	-	-
6459	12223	DNA-cytosine methyltransferase (EC 2.1.1.37)	1	-	-	-	-	-	-	-	-	-	-	-	-	-	-	-	-	-	-	-	-	-	-	-	-	-	-	-	-	-	-	-	-	-	-	-	-	-	-	-	-	-	-	-	-	-	-	-	-	-	+	-	-	-
6460	12224	DNA-3-methyladenine glycosylase II (EC 3.2.2.21)	1	-	-	-	-	-	-	-	-	-	-	-	-	-	-	-	-	-	-	-	-	-	-	-	-	-	-	-	-	-	-	-	-	-	-	-	-	-	-	-	-	-	-	-	-	-	-	-	-	-	+	-	-	-
6461	12225	hypothetical protein	1	-	-	-	-	-	-	-	-	-	-	-	-	-	-	-	-	-	-	-	-	-	-	-	-	-	-	-	-	-	-	-	-	-	-	-	-	-	-	-	-	-	-	-	-	-	-	-	-	-	+	-	-	-
6462	12226	hypothetical protein	1	-	-	-	-	-	-	-	-	-	-	-	-	-	-	-	-	-	-	-	-	-	-	-	-	-	-	-	-	-	-	-	-	-	-	-	-	-	-	-	-	-	-	-	-	-	-	-	-	-	+	-	-	-
6463	12227	hypothetical protein	1	-	-	-	-	-	-	-	-	-	-	-	-	-	-	-	-	-	-	-	-	-	-	-	-	-	-	-	-	-	-	-	-	-	-	-	-	-	-	-	-	-	-	-	-	-	-	-	-	-	+	-	-	-
6464	12228	hypothetical protein	1	-	-	-	-	-	-	-	-	-	-	-	-	-	-	-	-	-	-	-	-	-	-	-	-	-	-	-	-	-	-	-	-	-	-	-	-	-	-	-	-	-	-	-	-	-	-	-	-	-	+	-	-	-
6465	12229	Phage tail assembly	1	-	-	-	-	-	-	-	-	-	-	-	-	-	-	-	-	-	-	-	-	-	-	-	-	-	-	-	-	-	-	-	-	-	-	-	-	-	-	-	-	-	-	-	-	-	-	-	-	-	+	-	-	-
6466	12230	NADH dehydrogenase (EC 1.6.99.3)	1	-	-	-	-	-	-	-	-	-	-	-	-	-	-	-	-	-	-	-	-	-	-	-	-	-	-	-	-	-	-	-	-	-	-	-	-	-	-	-	-	-	-	-	-	-	-	-	-	-	+	-	-	-
6467	12231	hypothetical protein	1	-	-	-	-	-	-	-	-	-	-	-	-	-	-	-	-	-	-	-	-	-	-	-	-	-	-	-	-	-	-	-	-	-	-	-	-	-	-	-	-	-	-	-	-	-	-	-	-	-	+	-	-	-
6468	12232	hypothetical protein	1	-	-	-	-	-	-	-	-	-	-	-	-	-	-	-	-	-	-	-	-	-	-	-	-	-	-	-	-	-	-	-	-	-	-	-	-	-	-	-	-	-	-	-	-	-	-	-	-	-	+	-	-	-
6469	12233	hypothetical protein	1	-	-	-	-	-	-	-	-	-	-	-	-	-	-	-	-	-	-	-	-	-	-	-	-	-	-	-	-	-	-	-	-	-	-	-	-	-	-	-	-	-	-	-	-	-	-	-	-	-	+	-	-	-
6470	12234	hypothetical protein	1	-	-	-	-	-	-	-	-	-	-	-	-	-	-	-	-	-	-	-	-	-	-	-	-	-	-	-	-	-	-	-	-	-	-	-	-	-	-	-	-	-	-	-	-	-	-	-	-	-	+	-	-	-
6471	12235	hypothetical protein	1	-	-	-	-	-	-	-	-	-	-	-	-	-	-	-	-	-	-	-	-	-	-	-	-	-	-	-	-	-	-	-	-	-	-	-	-	-	-	-	-	-	-	-	-	-	-	-	-	-	+	-	-	-
6472	12236	phage protein	1	-	-	-	-	-	-	-	-	-	-	-	-	-	-	-	-	-	-	-	-	-	-	-	-	-	-	-	-	-	-	-	-	-	-	-	-	-	-	+	-	-	-	-	-	-	-	-	-	-	-	-	-	-
6473	12237	hypothetical protein	1	-	-	-	-	-	-	-	-	-	-	-	-	-	-	-	-	-	-	-	-	-	-	-	-	-	-	-	-	-	-	-	-	-	-	-	-	-	-	+	-	-	-	-	-	-	-	-	-	-	-	-	-	-
6474	12238	hypothetical protein	1	-	-	-	-	-	-	-	-	-	-	-	-	-	-	-	-	-	-	-	-	-	-	-	-	-	-	-	-	-	-	-	-	-	-	-	-	-	-	+	-	-	-	-	-	-	-	-	-	-	-	-	-	-
6475	12239	Glycerol uptake facilitator protein	1	-	-	-	-	-	-	-	-	-	-	-	-	-	-	-	-	-	-	-	-	-	-	-	-	-	-	-	-	-	-	-	-	-	-	-	-	-	-	+	-	-	-	-	-	-	-	-	-	-	-	-	-	-
6476	12240	Na(+)/H(+) antiporter	1	-	-	-	-	-	-	-	-	-	-	-	-	-	-	-	-	-	-	-	-	-	-	-	-	-	-	-	-	-	-	-	-	-	-	-	-	-	-	+	-	-	-	-	-	-	-	-	-	-	-	-	-	-
6477	12241	hypothetical protein	1	-	-	-	-	-	-	-	-	-	-	-	-	-	-	-	-	-	-	-	-	-	-	-	-	-	-	-	-	-	-	-	-	-	-	-	-	-	-	+	-	-	-	-	-	-	-	-	-	-	-	-	-	-
6478	12242	hypothetical protein	1	-	-	-	-	-	-	-	-	-	-	-	-	-	-	-	-	-	-	-	-	-	-	-	-	-	-	-	-	-	-	-	-	-	-	-	-	-	-	+	-	-	-	-	-	-	-	-	-	-	-	-	-	-
6479	12243	glycosyltransferase	1	-	-	-	-	-	-	-	-	-	-	-	-	-	-	-	-	-	-	-	-	-	-	-	-	-	-	-	-	-	-	-	-	-	-	-	-	-	-	+	-	-	-	-	-	-	-	-	-	-	-	-	-	-
6480	12244	replication protein	1	-	-	-	-	-	-	-	-	-	-	-	-	-	-	-	-	-	-	-	-	-	-	-	-	-	-	-	-	-	-	-	-	-	-	-	-	-	-	+	-	-	-	-	-	-	-	-	-	-	-	-	-	-
6481	12245	hypothetical protein	1	-	-	-	-	-	-	-	-	-	-	-	-	-	-	-	-	-	-	-	-	-	-	-	-	-	-	-	-	-	-	-	-	-	-	-	-	-	-	+	-	-	-	-	-	-	-	-	-	-	-	-	-	-
6482	12246	Superfamily II DNA and RNA helicase	1	-	-	-	-	-	-	-	-	-	-	-	-	-	-	-	-	-	-	-	-	-	-	-	-	-	-	-	-	-	-	-	-	-	-	-	-	-	-	+	-	-	-	-	-	-	-	-	-	-	-	-	-	-
6483	12247	hypothetical protein	1	-	-	-	-	-	-	-	-	-	-	-	-	-	-	-	-	-	-	-	-	-	-	-	-	-	-	-	-	-	-	-	-	-	-	-	-	-	-	+	-	-	-	-	-	-	-	-	-	-	-	-	-	-
6484	12248	hypothetical protein	1	-	-	-	-	-	-	-	-	-	-	-	-	-	-	-	-	-	-	-	-	-	-	-	-	-	-	-	-	-	-	-	-	-	-	-	-	-	-	+	-	-	-	-	-	-	-	-	-	-	-	-	-	-
6485	12249	Site-specific recombinase	1	-	-	-	-	-	-	-	-	-	-	-	-	-	-	-	-	-	-	-	-	-	-	-	-	-	-	-	-	-	-	-	-	-	-	-	-	-	-	+	-	-	-	-	-	-	-	-	-	-	-	-	-	-
6486	12250	hypothetical protein	1	-	-	-	-	-	-	-	-	-	-	-	-	-	-	-	-	-	-	-	-	-	-	-	-	-	-	-	-	-	-	-	-	-	-	-	-	-	-	+	-	-	-	-	-	-	-	-	-	-	-	-	-	-
6487	12251	hypothetical protein	1	-	-	-	-	-	-	-	-	-	-	-	-	-	-	-	-	-	-	-	-	-	-	-	-	-	-	-	-	-	-	-	-	-	-	-	-	-	-	+	-	-	-	-	-	-	-	-	-	-	-	-	-	-
6488	12252	hypothetical protein	1	-	-	-	-	-	-	-	-	-	-	-	-	-	-	-	-	-	-	-	-	-	-	-	-	-	-	-	-	-	-	-	-	-	-	-	-	-	-	+	-	-	-	-	-	-	-	-	-	-	-	-	-	-
6489	12253	putative low temperature requirement A protein	1	-	-	-	-	-	-	-	-	-	-	-	-	-	-	-	-	-	-	-	-	-	-	-	-	-	-	-	-	-	-	-	-	-	-	-	-	-	-	+	-	-	-	-	-	-	-	-	-	-	-	-	-	-
6490	12254	hypothetical protein	1	-	-	-	-	-	-	-	-	-	-	-	-	-	-	-	-	-	-	-	-	-	-	-	-	-	-	-	-	-	-	-	-	-	-	-	-	-	-	+	-	-	-	-	-	-	-	-	-	-	-	-	-	-
6491	12255	FtsK/SpoIIIE family protein	1	-	-	-	-	-	-	-	-	-	-	-	-	-	-	-	-	-	-	-	-	-	-	-	-	-	-	-	-	-	-	-	-	-	-	-	-	-	-	+	-	-	-	-	-	-	-	-	-	-	-	-	-	-
6492	12256	hypothetical protein	1	-	-	-	-	-	-	-	-	-	-	-	-	-	-	-	-	-	-	-	-	-	-	-	-	-	-	-	-	-	-	-	-	-	-	-	-	-	-	+	-	-	-	-	-	-	-	-	-	-	-	-	-	-
6493	12257	DNA-cytosine methyltransferase (EC 2.1.1.37)	1	-	-	-	-	-	-	-	-	-	-	-	-	-	-	-	-	-	-	-	-	-	-	-	-	-	-	-	-	-	-	-	-	-	-	-	-	-	-	+	-	-	-	-	-	-	-	-	-	-	-	-	-	-
6494	12258	hypothetical protein	1	-	-	-	-	-	-	-	-	-	-	-	-	-	-	-	-	-	-	-	-	-	-	-	-	-	-	-	-	-	-	-	-	-	-	-	-	-	-	+	-	-	-	-	-	-	-	-	-	-	-	-	-	-
6495	12259	hypothetical protein	1	-	-	-	-	-	-	-	-	-	-	-	-	-	-	-	-	-	-	-	-	-	-	-	-	-	-	-	-	-	-	-	-	-	-	-	-	-	-	+	-	-	-	-	-	-	-	-	-	-	-	-	-	-
6496	12260	Transcriptional regulator	1	-	-	-	-	-	-	-	-	-	-	-	-	-	-	-	-	-	-	-	-	-	-	-	-	-	-	-	-	-	-	-	-	-	-	-	-	-	-	+	-	-	-	-	-	-	-	-	-	-	-	-	-	-
6497	12261	hypothetical protein	1	-	-	-	-	-	-	-	-	-	-	-	-	-	-	-	-	-	-	-	-	-	-	-	-	-	-	-	-	-	-	-	-	-	-	-	-	-	-	+	-	-	-	-	-	-	-	-	-	-	-	-	-	-
6498	12262	internalin	1	-	-	-	-	-	-	-	-	-	-	-	-	-	-	-	-	-	-	-	-	-	-	-	-	-	-	-	-	-	-	-	-	-	-	-	-	-	-	+	-	-	-	-	-	-	-	-	-	-	-	-	-	-
6499	12263	hypothetical protein	1	-	-	-	-	-	-	-	-	-	-	-	-	-	-	-	-	-	-	-	-	-	-	-	-	-	-	-	-	-	-	-	-	-	-	-	-	-	-	+	-	-	-	-	-	-	-	-	-	-	-	-	-	-
6500	12264	hypothetical protein	1	-	-	-	-	-	-	-	-	-	-	-	-	-	-	-	-	-	-	-	-	-	-	-	-	-	-	-	-	-	-	-	-	-	-	-	-	-	-	+	-	-	-	-	-	-	-	-	-	-	-	-	-	-
6501	12265	hypothetical protein	1	-	-	-	-	-	-	-	-	-	-	-	-	-	-	-	-	-	-	-	-	-	-	-	-	-	-	-	-	-	-	-	-	-	-	-	-	-	-	+	-	-	-	-	-	-	-	-	-	-	-	-	-	-
6502	12266	DNA primase (EC 2.7.7.-)	1	-	-	-	-	-	-	-	-	-	-	-	-	-	-	-	-	-	-	-	-	-	-	-	-	-	-	-	-	-	-	-	-	-	-	-	-	-	-	+	-	-	-	-	-	-	-	-	-	-	-	-	-	-
6503	12267	hypothetical protein	1	-	-	-	-	-	-	-	-	-	-	-	-	-	-	-	-	-	-	-	-	-	-	-	-	-	-	-	-	-	-	-	-	-	-	-	-	-	-	+	-	-	-	-	-	-	-	-	-	-	-	-	-	-
6504	12268	Site-specific recombinase	1	-	-	-	-	-	-	-	-	-	-	-	-	-	-	-	-	-	-	-	-	-	-	-	-	-	-	-	-	-	-	-	-	-	-	-	-	-	-	+	-	-	-	-	-	-	-	-	-	-	-	-	-	-
6505	12269	hypothetical protein	1	-	-	-	-	-	-	-	-	-	-	-	-	-	-	-	-	-	-	-	-	-	-	-	-	-	-	-	-	-	-	-	-	-	-	-	-	-	-	+	-	-	-	-	-	-	-	-	-	-	-	-	-	-
6506	12270	hypothetical protein	1	-	-	-	-	-	-	-	-	-	-	-	-	-	-	-	-	-	-	-	-	-	-	-	-	-	-	-	-	-	-	-	-	-	-	-	-	-	-	+	-	-	-	-	-	-	-	-	-	-	-	-	-	-
6507	12271	hypothetical protein	1	-	-	-	-	-	-	-	-	-	-	-	-	-	-	-	-	-	-	-	-	-	-	-	-	-	-	-	-	-	-	-	-	-	-	-	-	-	-	+	-	-	-	-	-	-	-	-	-	-	-	-	-	-
6508	12272	hypothetical protein	1	-	-	-	-	-	-	-	-	-	-	-	-	-	-	-	-	-	-	-	-	-	-	-	-	-	-	-	-	-	-	-	-	-	-	-	-	-	-	+	-	-	-	-	-	-	-	-	-	-	-	-	-	-
6509	12273	Site-specific recombinase	1	-	-	-	-	-	-	-	-	-	-	-	-	-	-	-	-	-	-	-	-	-	-	-	-	-	-	-	-	-	-	-	-	-	-	-	-	-	-	+	-	-	-	-	-	-	-	-	-	-	-	-	-	-
6510	12274	hypothetical protein	1	-	-	-	-	-	-	-	-	-	-	-	-	-	-	-	-	-	-	-	-	-	-	-	-	-	-	-	-	-	-	-	-	-	-	-	-	-	-	+	-	-	-	-	-	-	-	-	-	-	-	-	-	-
6511	12275	hypothetical protein	1	-	-	-	-	-	-	-	-	-	-	-	-	-	-	-	-	-	-	-	-	-	-	-	-	-	-	-	-	-	-	-	-	-	-	-	-	-	-	+	-	-	-	-	-	-	-	-	-	-	-	-	-	-
6512	12276	DNA-cytosine methyltransferase	1	-	-	-	-	-	-	-	-	-	-	-	-	-	-	-	-	-	-	-	-	-	-	-	-	-	-	-	-	-	-	-	-	-	-	-	-	-	-	+	-	-	-	-	-	-	-	-	-	-	-	-	-	-
6513	12277	hypothetical protein	1	-	-	-	-	-	-	-	-	-	-	-	-	-	-	-	-	-	-	-	-	-	-	-	-	-	-	-	-	-	-	-	-	-	-	-	-	-	-	+	-	-	-	-	-	-	-	-	-	-	-	-	-	-
6514	12278	hypothetical protein	1	-	-	-	-	-	-	-	-	-	-	-	-	-	-	-	-	-	-	-	-	-	-	-	-	-	-	-	-	-	-	-	-	-	-	-	-	-	-	+	-	-	-	-	-	-	-	-	-	-	-	-	-	-
6515	12279	hypothetical protein	1	-	-	-	-	-	-	-	-	-	-	-	-	-	-	-	-	-	-	-	-	-	-	-	-	-	-	-	-	-	-	-	-	-	-	-	-	-	-	+	-	-	-	-	-	-	-	-	-	-	-	-	-	-
6516	12280	mannosyltransferase B	1	-	-	-	-	-	-	-	-	-	-	-	-	-	-	-	-	-	-	-	-	-	-	-	-	-	-	-	-	-	-	-	-	-	-	-	-	-	-	+	-	-	-	-	-	-	-	-	-	-	-	-	-	-
6517	12281	hypothetical protein	1	-	-	-	-	-	-	-	-	-	-	-	-	-	-	-	-	-	-	-	-	-	-	-	-	-	-	-	-	-	-	-	-	-	-	-	-	-	-	+	-	-	-	-	-	-	-	-	-	-	-	-	-	-
6518	12282	hypothetical protein	1	-	-	-	-	-	-	-	-	-	-	-	-	-	-	-	-	-	-	-	-	-	-	-	-	-	-	-	-	-	-	-	-	-	-	-	-	-	-	+	-	-	-	-	-	-	-	-	-	-	-	-	-	-
6519	12283	Nicotine adenine dinucleotide glycohydrolase (NADGH) (EC 3.2.2.5)	1	-	-	-	-	-	-	-	-	-	-	-	-	-	-	-	-	-	-	-	-	-	-	-	-	-	-	-	-	-	-	-	-	-	-	-	-	-	-	+	-	-	-	-	-	-	-	-	-	-	-	-	-	-
6520	12284	hypothetical protein	1	-	-	-	-	-	-	-	-	-	-	-	-	-	-	-	-	-	-	-	-	-	-	-	-	-	-	-	-	-	-	-	-	-	-	-	-	-	-	+	-	-	-	-	-	-	-	-	-	-	-	-	-	-
6521	12285	hypothetical protein	1	-	-	-	-	-	-	-	-	-	-	-	-	-	-	-	-	-	-	-	-	-	-	-	-	-	-	-	-	-	-	-	-	-	-	-	-	-	-	+	-	-	-	-	-	-	-	-	-	-	-	-	-	-
6522	12286	hypothetical protein	1	-	-	-	-	-	-	-	-	-	-	-	-	-	-	-	-	-	-	-	-	-	-	-	-	-	-	-	-	-	-	-	-	-	-	-	-	-	-	+	-	-	-	-	-	-	-	-	-	-	-	-	-	-
6523	12287	hypothetical protein	1	-	-	-	-	-	-	-	-	-	-	-	-	-	-	-	-	-	-	-	-	-	-	-	-	-	-	-	-	-	-	-	-	-	-	-	-	-	-	+	-	-	-	-	-	-	-	-	-	-	-	-	-	-
6524	12288	hypothetical protein	1	-	-	-	-	-	-	-	-	-	-	-	-	-	-	-	-	-	-	-	-	-	-	-	-	-	-	-	-	-	-	-	-	-	-	-	-	-	-	+	-	-	-	-	-	-	-	-	-	-	-	-	-	-
6525	12289	hypothetical protein	1	-	-	-	-	-	-	-	-	-	-	-	-	-	-	-	-	-	-	-	-	-	-	-	-	-	-	-	-	-	-	-	-	-	-	-	-	-	-	+	-	-	-	-	-	-	-	-	-	-	-	-	-	-
6526	12290	hypothetical protein	1	-	-	-	-	-	-	-	-	-	-	-	-	-	-	-	-	-	-	-	-	-	-	-	-	-	-	-	-	-	-	-	-	-	-	-	-	-	-	+	-	-	-	-	-	-	-	-	-	-	-	-	-	-
6527	12291	hypothetical protein	1	-	-	-	-	-	-	-	-	-	-	-	-	-	-	-	-	-	-	-	-	-	-	-	-	-	-	-	-	-	-	-	-	-	-	-	-	-	-	+	-	-	-	-	-	-	-	-	-	-	-	-	-	-
6528	12292	hypothetical protein	1	-	-	-	-	-	-	-	-	-	-	-	-	-	-	-	-	-	-	-	-	-	-	-	-	-	-	-	-	-	-	-	-	-	-	-	-	-	-	+	-	-	-	-	-	-	-	-	-	-	-	-	-	-
6529	12293	2-haloalkanoic acid dehalogenase (EC 3.8.1.2)	1	-	-	-	-	-	-	-	-	-	-	-	-	-	-	-	-	-	-	-	-	-	-	-	-	-	-	-	-	-	-	-	-	-	-	-	-	-	-	+	-	-	-	-	-	-	-	-	-	-	-	-	-	-
6530	12294	hypothetical protein	1	-	-	-	-	-	-	-	-	-	-	-	-	-	-	-	-	-	-	-	-	-	-	-	-	-	-	-	-	-	-	-	-	-	-	-	-	-	-	+	-	-	-	-	-	-	-	-	-	-	-	-	-	-
6531	12295	hypothetical protein	1	-	-	-	-	-	-	-	-	-	-	-	-	-	-	-	-	-	-	-	-	-	-	-	-	-	-	-	-	-	-	-	-	-	-	-	-	-	-	+	-	-	-	-	-	-	-	-	-	-	-	-	-	-
6532	12296	hypothetical protein	1	-	-	-	-	-	-	-	-	-	-	-	-	-	-	-	-	-	-	-	-	-	-	-	-	-	-	-	-	-	-	-	-	-	-	-	-	-	-	+	-	-	-	-	-	-	-	-	-	-	-	-	-	-
6533	12297	hypothetical protein	1	-	-	-	-	-	-	-	-	-	-	-	-	-	-	-	-	-	-	-	-	-	-	-	-	-	-	-	-	-	-	-	-	-	-	-	-	-	-	+	-	-	-	-	-	-	-	-	-	-	-	-	-	-
6534	12298	FtsK/SpoIIIE family protein	1	-	-	-	-	-	-	-	-	-	-	-	-	-	-	-	-	-	-	-	-	-	-	-	-	-	-	-	-	-	-	-	-	-	-	-	-	-	-	+	-	-	-	-	-	-	-	-	-	-	-	-	-	-
6535	12299	hypothetical protein	1	-	-	-	-	-	-	-	-	-	-	-	-	-	-	-	-	-	-	-	-	-	-	-	-	-	-	-	-	-	-	-	-	-	-	-	-	-	-	+	-	-	-	-	-	-	-	-	-	-	-	-	-	-
6536	12300	hypothetical protein	1	-	-	-	-	-	-	-	-	-	-	-	-	-	-	-	-	-	-	-	-	-	-	-	-	-	-	-	-	-	-	-	-	-	-	-	-	-	-	+	-	-	-	-	-	-	-	-	-	-	-	-	-	-
6537	12301	hypothetical protein	1	-	-	-	-	-	-	-	-	-	-	-	-	-	-	-	-	-	-	-	-	-	-	-	-	-	-	-	-	-	-	-	-	-	-	-	-	-	-	+	-	-	-	-	-	-	-	-	-	-	-	-	-	-
6538	12302	hypothetical protein	1	-	-	-	-	-	-	-	-	-	-	-	-	-	-	-	-	-	-	-	-	-	-	-	-	-	-	-	-	-	-	-	-	-	-	-	-	-	-	+	-	-	-	-	-	-	-	-	-	-	-	-	-	-
6539	12303	Acetyltransferase (EC 2.3.1.-)	1	-	-	-	-	-	-	-	-	-	-	-	-	-	-	-	-	-	-	-	-	-	-	-	-	-	-	-	-	-	-	-	-	-	-	-	-	-	-	+	-	-	-	-	-	-	-	-	-	-	-	-	-	-
6540	12304	Helicase loader DnaI	1	-	-	-	-	-	-	-	-	-	-	-	-	-	-	-	-	-	-	-	-	-	-	-	-	-	-	-	-	-	-	-	-	-	-	-	-	-	-	+	-	-	-	-	-	-	-	-	-	-	-	-	-	-
6541	12305	hypothetical protein	1	-	-	-	-	-	-	-	-	-	-	-	-	-	-	-	-	-	-	-	-	-	-	-	-	-	-	-	-	-	-	-	-	-	-	-	-	-	-	+	-	-	-	-	-	-	-	-	-	-	-	-	-	-
6542	12306	hypothetical protein	1	-	-	-	-	-	-	-	-	-	-	-	-	-	-	-	-	-	-	-	-	-	-	-	-	-	-	-	-	-	-	-	-	-	-	-	-	-	-	+	-	-	-	-	-	-	-	-	-	-	-	-	-	-
6543	12307	OmpA domain protein	1	-	-	-	-	-	-	-	-	-	-	-	-	-	-	-	-	-	-	-	-	-	-	-	-	-	-	-	-	-	-	-	-	-	-	-	-	-	-	+	-	-	-	-	-	-	-	-	-	-	-	-	-	-
6544	12308	hypothetical protein	1	-	-	-	-	-	-	-	-	-	-	-	-	-	-	-	-	-	-	-	-	-	-	-	-	-	-	-	-	-	-	-	-	-	-	-	-	-	-	+	-	-	-	-	-	-	-	-	-	-	-	-	-	-
6545	12309	hypothetical protein	1	-	-	-	-	-	-	-	-	-	-	-	-	-	-	-	-	-	-	-	-	-	-	-	-	-	-	-	-	-	-	-	-	-	-	-	-	-	-	+	-	-	-	-	-	-	-	-	-	-	-	-	-	-
6546	12310	FtsK/SpoIIIE family protein	1	-	-	-	-	-	-	-	-	-	-	-	-	-	-	-	-	-	-	-	-	-	-	-	-	-	-	-	-	-	-	-	-	-	-	-	-	-	-	+	-	-	-	-	-	-	-	-	-	-	-	-	-	-
6547	12311	2-oxoglutarate dehydrogenase E1 component (EC 1.2.4.2)	1	-	-	-	-	-	-	-	-	-	-	-	-	-	-	-	-	-	-	-	-	-	-	-	-	-	-	-	-	-	-	-	-	-	-	-	-	-	-	+	-	-	-	-	-	-	-	-	-	-	-	-	-	-
6548	12312	hypothetical protein	1	-	-	-	-	-	-	-	-	-	-	-	-	-	-	-	-	-	-	-	-	-	-	-	-	-	-	-	-	-	-	-	-	-	-	-	-	-	-	+	-	-	-	-	-	-	-	-	-	-	-	-	-	-
6549	12313	hypothetical protein	1	-	-	-	-	-	-	-	-	-	-	-	-	-	-	-	-	-	-	-	-	-	-	-	-	-	-	-	-	-	-	-	-	-	-	-	-	-	-	+	-	-	-	-	-	-	-	-	-	-	-	-	-	-
6550	12314	Type I restriction-modification system	1	-	-	-	-	-	-	-	-	-	-	-	-	-	-	-	-	-	-	-	-	-	-	-	-	-	-	-	-	-	-	-	-	-	-	-	-	-	-	+	-	-	-	-	-	-	-	-	-	-	-	-	-	-
6551	12315	hypothetical protein	1	-	-	-	-	-	-	-	-	-	-	-	-	-	-	-	-	-	-	-	-	-	-	-	-	-	-	-	-	-	-	-	-	-	-	-	-	-	-	+	-	-	-	-	-	-	-	-	-	-	-	-	-	-
6552	12316	hypothetical protein	1	-	-	-	-	-	-	-	-	-	-	-	-	-	-	-	-	-	-	-	-	-	-	-	-	-	-	-	-	-	-	-	-	-	-	-	-	-	-	+	-	-	-	-	-	-	-	-	-	-	-	-	-	-
6553	12317	ATP-dependent DNA helicase	1	-	-	-	-	-	-	-	-	-	-	-	-	-	-	-	-	-	-	-	-	-	-	-	-	-	-	-	-	-	-	-	-	-	-	-	-	-	-	+	-	-	-	-	-	-	-	-	-	-	-	-	-	-
6554	12318	FunZ family protein	1	-	-	-	-	-	-	-	-	-	-	-	-	-	-	-	-	-	-	-	-	-	-	-	-	-	-	-	-	-	-	-	-	-	-	-	-	-	-	+	-	-	-	-	-	-	-	-	-	-	-	-	-	-
6555	12319	hypothetical protein	1	-	-	-	-	-	-	-	-	-	-	-	-	-	-	-	-	-	-	-	-	-	-	-	-	-	-	-	-	-	-	-	-	-	-	-	-	-	-	+	-	-	-	-	-	-	-	-	-	-	-	-	-	-
6556	12320	hypothetical protein	1	-	-	-	-	-	-	-	-	-	-	-	-	-	-	-	-	-	-	-	-	-	-	-	-	-	-	-	-	-	-	-	-	-	-	-	-	-	-	+	-	-	-	-	-	-	-	-	-	-	-	-	-	-
6557	12321	hypothetical protein	1	-	-	-	-	-	-	-	-	-	-	-	-	-	-	-	-	-	-	-	-	-	-	-	-	-	-	-	-	-	-	-	-	-	-	-	-	-	-	+	-	-	-	-	-	-	-	-	-	-	-	-	-	-
6558	12322	hypothetical protein	1	-	-	-	-	-	-	-	-	-	-	-	-	-	-	-	-	-	-	-	-	-	-	-	-	-	-	-	-	-	-	-	-	-	-	-	-	-	-	+	-	-	-	-	-	-	-	-	-	-	-	-	-	-
6559	12323	Hyaluronan mediated motility receptor	1	-	-	-	-	-	-	-	-	-	-	-	-	-	-	-	-	-	-	-	-	-	-	-	-	-	-	-	-	-	-	-	-	-	-	-	-	-	-	+	-	-	-	-	-	-	-	-	-	-	-	-	-	-
6560	12324	hypothetical protein	1	-	-	-	-	-	-	-	-	-	-	-	-	-	-	-	-	-	-	-	-	-	-	-	-	-	-	-	-	-	-	-	-	-	-	-	-	-	-	+	-	-	-	-	-	-	-	-	-	-	-	-	-	-
6561	12325	hypothetical protein	1	-	-	-	-	-	-	-	-	-	-	-	-	-	-	-	-	-	-	-	-	-	-	-	-	-	-	-	-	-	-	-	-	-	-	-	-	-	-	+	-	-	-	-	-	-	-	-	-	-	-	-	-	-
6562	12326	hypothetical protein	1	-	-	-	-	-	-	-	-	-	-	-	-	-	-	-	-	-	-	-	-	-	-	-	-	-	-	-	-	-	-	-	-	-	-	-	-	-	-	+	-	-	-	-	-	-	-	-	-	-	-	-	-	-
6563	12327	hypothetical protein	1	-	-	-	-	-	-	-	-	-	-	-	-	-	-	-	-	-	-	-	-	-	-	-	-	-	-	-	-	-	-	-	-	-	-	-	-	-	-	+	-	-	-	-	-	-	-	-	-	-	-	-	-	-
6564	12328	hypothetical protein	1	-	-	-	-	-	-	-	-	-	-	-	-	-	-	-	-	-	-	-	-	-	-	-	-	-	-	-	-	-	-	-	-	-	-	-	-	-	-	+	-	-	-	-	-	-	-	-	-	-	-	-	-	-
6565	12329	hypothetical protein	1	-	-	-	-	-	-	-	-	-	-	-	-	-	-	-	-	-	-	-	-	-	-	-	-	-	-	-	-	-	-	-	-	-	-	-	-	-	-	+	-	-	-	-	-	-	-	-	-	-	-	-	-	-
6566	12330	hypothetical protein	1	-	-	-	-	-	-	-	-	-	-	-	-	-	-	-	-	-	-	-	-	-	-	-	-	-	-	-	-	-	-	-	-	-	-	-	-	-	-	+	-	-	-	-	-	-	-	-	-	-	-	-	-	-
6567	12331	hypothetical protein	1	-	-	-	-	-	-	-	-	-	-	-	-	-	-	-	-	-	-	-	-	-	-	-	-	-	-	-	-	-	-	-	-	-	-	-	-	-	-	+	-	-	-	-	-	-	-	-	-	-	-	-	-	-

Table S5. Precence/absence of each HG among 57 *S. suis* strains

				1																13			25					28						104		94			76			53/54		Divergent *S. suis*																
Index[a]	ClusterID	Gene products	Members	98HAH33	05ZYH33	SC84	A7	SS12	P1/7	DAT273	NIAH11434	JS14	GZ1	BM407	S735	13730	MNCM07	NCTC 10446	14636	NCTC 10237	ST1	2651	89-1591	MNCM04	MNCM25	8074	D9	4961	ST3	MNCM43	DAT292	DAT260	NIAH11435	MNCM21	MNCM50	89-2479	DAT234	DAT290	93A	42A	NT77	6407	11538	8830	12814	92-1400	89-590	2726	2524	89-3576-3	4417	D12	10581	89-5259	88-5299A	R61	92-1191	14A	92-4172	22083
6568	12332	hypothetical protein	1	-	-	-	-	-	-	-	-	-	-	-	-	-	-	-	-	-	-	-	-	-	-	-	-	-	-	-	-	-	-	-	-	-	-	-	-	-	-	-	-	-	-	+	-	-	-	-	-	-	-	-	-	-	-	-	-	-
6569	12333	hypothetical protein	1	-	-	-	-	-	-	-	-	-	-	-	-	-	-	-	-	-	-	-	-	-	-	-	-	-	-	-	-	-	-	-	-	-	-	-	-	-	-	-	-	-	-	+	-	-	-	-	-	-	-	-	-	-	-	-	-	-
6570	12334	hypothetical protein	1	-	-	-	-	-	-	-	-	-	-	-	-	-	-	-	-	-	-	-	-	-	-	-	-	-	-	-	-	-	-	-	-	-	-	-	-	-	-	-	-	-	-	+	-	-	-	-	-	-	-	-	-	-	-	-	-	-
6571	12335	hypothetical protein	1	-	-	-	-	-	-	-	-	-	-	-	-	-	-	-	-	-	-	-	-	-	-	-	-	-	-	-	-	-	-	-	-	-	-	-	-	-	-	-	-	-	-	+	-	-	-	-	-	-	-	-	-	-	-	-	-	-
6572	12336	Retron-type RNA-directed DNA polymerase (EC 2.7.7.49)	1	-	-	-	-	-	-	-	-	-	-	-	-	-	-	-	-	-	-	-	-	-	-	-	-	-	-	-	-	-	-	-	-	-	-	-	-	-	-	-	-	-	-	+	-	-	-	-	-	-	-	-	-	-	-	-	-	-
6573	12337	hypothetical protein	1	-	-	-	-	-	-	-	-	-	-	-	-	-	-	-	-	-	-	-	-	-	-	-	-	-	-	-	-	-	-	-	-	-	-	-	-	-	-	-	-	-	-	+	-	-	-	-	-	-	-	-	-	-	-	-	-	-
6574	12338	hypothetical protein	1	-	-	-	-	-	-	-	-	-	-	-	-	-	-	-	-	-	-	-	-	-	-	-	-	-	-	-	-	-	-	-	-	-	-	-	-	-	-	-	-	-	-	+	-	-	-	-	-	-	-	-	-	-	-	-	-	-
6575	12339	hypothetical protein	1	-	-	-	-	-	-	-	-	-	-	-	-	-	-	-	-	-	-	-	-	-	-	-	-	-	-	-	-	-	-	-	-	-	-	-	-	-	-	-	-	-	-	+	-	-	-	-	-	-	-	-	-	-	-	-	-	-
6576	12340	hypothetical protein	1	-	-	-	-	-	-	-	-	-	-	-	-	-	-	-	-	-	-	-	-	-	-	-	-	-	-	-	-	-	-	-	-	-	-	-	-	-	-	-	-	-	-	+	-	-	-	-	-	-	-	-	-	-	-	-	-	-
6577	12341	hypothetical protein	1	-	-	-	-	-	-	-	-	-	-	-	-	-	-	-	-	-	-	-	-	-	-	-	-	-	-	-	-	-	-	-	-	-	-	-	-	-	-	-	-	-	-	+	-	-	-	-	-	-	-	-	-	-	-	-	-	-
6578	12342	hypothetical protein	1	-	-	-	-	-	-	-	-	-	-	-	-	-	-	-	-	-	-	-	-	-	-	-	-	-	-	-	-	-	-	-	-	-	-	-	-	-	-	-	-	-	-	+	-	-	-	-	-	-	-	-	-	-	-	-	-	-
6579	12343	hypothetical protein	1	-	-	-	-	-	-	-	-	-	-	-	-	-	-	-	-	-	-	-	-	-	-	-	-	-	-	-	-	-	-	-	-	-	-	-	-	-	-	-	-	-	-	+	-	-	-	-	-	-	-	-	-	-	-	-	-	-
6580	12344	hypothetical protein	1	-	-	-	-	-	-	-	-	-	-	-	-	-	-	-	-	-	-	-	-	-	-	-	-	-	-	-	-	-	-	-	-	-	-	-	-	-	-	-	-	-	-	+	-	-	-	-	-	-	-	-	-	-	-	-	-	-
6581	12345	FtsK/SpoIIIE family protein	1	-	-	-	-	-	-	-	-	-	-	-	-	-	-	-	-	-	-	-	-	-	-	-	-	-	-	-	-	-	-	-	-	-	-	-	-	-	-	-	-	-	-	+	-	-	-	-	-	-	-	-	-	-	-	-	-	-
6582	12346	hypothetical protein	1	-	-	-	-	-	-	-	-	-	-	-	-	-	-	-	-	-	-	-	-	-	-	-	-	-	-	-	-	-	-	-	-	-	-	-	-	-	-	-	-	-	-	+	-	-	-	-	-	-	-	-	-	-	-	-	-	-
6583	12347	hypothetical protein	1	-	-	-	-	-	-	-	-	-	-	-	-	-	-	-	-	-	-	-	-	-	-	-	-	-	-	-	-	-	-	-	-	-	-	-	-	-	-	-	-	-	-	+	-	-	-	-	-	-	-	-	-	-	-	-	-	-
6584	12348	hypothetical protein	1	-	-	-	-	-	-	-	-	-	-	-	-	-	-	-	-	-	-	-	-	-	-	-	-	-	-	-	-	-	-	-	-	-	-	-	-	-	-	-	-	-	-	+	-	-	-	-	-	-	-	-	-	-	-	-	-	-
6585	12349	hypothetical protein	1	-	-	-	-	-	-	-	-	-	-	-	-	-	-	-	-	-	-	-	-	-	-	-	-	-	-	-	-	-	-	-	-	-	-	-	-	-	-	-	-	-	-	+	-	-	-	-	-	-	-	-	-	-	-	-	-	-
6586	12350	hypothetical protein	1	-	-	-	-	-	-	-	-	-	-	-	-	-	-	-	-	-	-	-	-	-	-	-	-	-	-	-	-	-	-	-	-	-	-	-	-	-	-	-	-	-	-	-	-	-	-	-	-	-	-	-	-	-	-	-	+	-
6587	12351	Aminoglycoside 3'-phosphotransferase (EC 2.7.1.95)	1	-	-	-	-	-	-	-	-	-	-	-	-	-	-	-	-	-	-	-	-	-	-	-	-	-	-	-	-	-	-	-	-	-	-	-	-	-	-	-	-	-	-	-	-	-	-	-	-	-	-	-	-	-	-	-	+	-
6588	12352	Mobile element protein	1	-	-	-	-	-	-	-	-	-	-	-	-	-	-	-	-	-	-	-	-	-	-	-	-	-	-	-	-	-	-	-	-	-	-	-	-	-	-	-	-	-	-	-	-	-	-	-	-	-	-	-	-	-	-	-	+	-
6589	12353	hypothetical protein	1	-	-	-	-	-	-	-	-	-	-	-	-	-	-	-	-	-	-	-	-	-	-	-	-	-	-	-	-	-	-	-	-	-	-	-	-	-	-	-	-	-	-	-	-	-	-	-	-	-	-	-	-	-	-	-	+	-
6590	12354	glycosyl transferase group 1	1	-	-	-	-	-	-	-	-	-	-	-	-	-	-	-	-	-	-	-	-	-	-	-	-	-	-	-	-	-	-	-	-	-	-	-	-	-	-	-	-	-	-	-	-	-	-	-	-	-	-	-	-	-	-	-	+	-
6591	12355	hypothetical protein	1	-	-	-	-	-	-	-	-	-	-	-	-	-	-	-	-	-	-	-	-	-	-	-	-	-	-	-	-	-	-	-	-	-	-	-	-	-	-	-	-	-	-	-	-	-	-	-	-	-	-	-	-	-	-	-	+	-
6592	12356	Phage tape measure	1	-	-	-	-	-	-	-	-	-	-	-	-	-	-	-	-	-	-	-	-	-	-	-	-	-	-	-	-	-	-	-	-	-	-	-	-	-	-	-	-	-	-	-	-	-	-	-	-	-	-	-	-	-	-	-	+	-
6593	12357	putative transcriptional regulator	1	-	-	-	-	-	-	-	-	-	-	-	-	-	-	-	-	-	-	-	-	-	-	-	-	-	-	-	-	-	-	-	-	-	-	-	-	-	-	-	-	-	-	-	-	-	-	-	-	-	-	-	-	-	-	-	+	-
6594	12358	hypothetical protein	1	-	-	-	-	-	-	-	-	-	-	-	-	-	-	-	-	-	-	-	-	-	-	-	-	-	-	-	-	-	-	-	-	-	-	-	-	-	-	-	-	-	-	-	-	-	-	-	-	-	-	-	-	-	-	-	+	-
6595	12359	Uracil permease	1	-	-	-	-	-	-	-	-	-	-	-	-	-	-	-	-	-	-	-	-	-	-	-	-	-	-	-	-	-	-	-	-	-	-	-	-	-	-	-	-	-	-	-	-	-	-	-	-	-	-	-	-	-	-	-	+	-
6596	12360	VrlE	1	-	-	-	-	-	-	-	-	-	-	-	-	-	-	-	-	-	-	-	-	-	-	-	-	-	-	-	-	-	-	-	-	-	-	-	-	-	-	-	-	-	-	-	-	-	-	-	-	-	-	-	-	-	-	-	+	-
6597	12361	hypothetical protein	1	-	-	-	-	-	-	-	-	-	-	-	-	-	-	-	-	-	-	-	-	-	-	-	-	-	-	-	-	-	-	-	-	-	-	-	-	-	-	-	-	-	-	-	-	-	-	-	-	-	-	-	-	-	-	-	+	-
6598	12362	uncharacterized phage protein (possible DNA packaging)	1	-	-	-	-	-	-	-	-	-	-	-	-	-	-	-	-	-	-	-	-	-	-	-	-	-	-	-	-	-	-	-	-	-	-	-	-	-	-	-	-	-	-	-	-	-	-	-	-	-	-	-	-	-	-	-	+	-
6599	12363	hypothetical protein	1	-	-	-	-	-	-	-	-	-	-	-	-	-	-	-	-	-	-	-	-	-	-	-	-	-	-	-	-	-	-	-	-	-	-	-	-	-	-	-	-	-	-	-	-	-	-	-	-	-	-	-	-	-	-	-	+	-
6600	12364	Aspartate--ammonia ligase (EC 6.3.1.1)	1	-	-	-	-	-	-	-	-	-	-	-	-	-	-	-	-	-	-	-	-	-	-	-	-	-	-	-	-	-	-	-	-	-	-	-	-	-	-	-	-	-	-	-	-	-	-	-	-	-	-	-	-	-	-	-	+	-
6601	12365	hypothetical protein	1	-	-	-	-	-	-	-	-	-	-	-	-	-	-	-	-	-	-	-	-	-	-	-	-	-	-	-	-	-	-	-	-	-	-	-	-	-	-	-	-	-	-	-	-	-	-	-	-	-	-	-	-	-	-	-	+	-
6602	12366	hypothetical protein	1	-	-	-	-	-	-	-	-	-	-	-	-	-	-	-	-	-	-	-	-	-	-	-	-	-	-	-	-	-	-	-	-	-	-	-	-	-	-	-	-	-	-	-	-	-	-	-	-	-	-	-	-	-	-	-	+	-
6603	12367	DNA primase	1	-	-	-	-	-	-	-	-	-	-	-	-	-	-	-	-	-	-	-	-	-	-	-	-	-	-	-	-	-	-	-	-	-	-	-	-	-	-	-	-	-	-	-	-	-	-	-	-	-	-	-	-	-	-	-	+	-
6604	12368	N-Acetyl-D-glucosamine ABC transport system	1	-	-	-	-	-	-	-	-	-	-	-	-	-	-	-	-	-	-	-	-	-	-	-	-	-	-	-	-	-	-	-	-	-	-	-	-	-	-	-	-	-	-	-	-	-	-	-	-	-	-	-	-	-	-	-	+	-
6605	12369	Methionine ABC transporter ATP-binding protein	1	-	-	-	-	-	-	-	-	-	-	-	-	-	-	-	-	-	-	-	-	-	-	-	-	-	-	-	-	-	-	-	-	-	-	-	-	-	-	-	-	-	-	-	-	-	-	-	-	-	-	-	-	-	-	-	+	-
6606	12370	hypothetical protein	1	-	-	-	-	-	-	-	-	-	-	-	-	-	-	-	-	-	-	-	-	-	-	-	-	-	-	-	-	-	-	-	-	-	-	-	-	-	-	-	-	-	-	-	-	-	-	-	-	-	-	-	-	-	-	-	+	-
6607	12371	L-serine dehydratase	1	-	-	-	-	-	-	-	-	-	-	-	-	-	-	-	-	-	-	-	-	-	-	-	-	-	-	-	-	-	-	-	-	-	-	-	-	-	-	-	-	-	-	-	-	-	-	-	-	-	-	-	-	-	-	-	+	-
6608	12372	transcription regulator	1	-	-	-	-	-	-	-	-	-	-	-	-	-	-	-	-	-	-	-	-	-	-	-	-	-	-	-	-	-	-	-	-	-	-	-	-	-	-	-	-	-	-	-	-	-	-	-	-	-	-	-	-	-	-	-	+	-
6609	12373	D-beta-hydroxybutyrate permease	1	-	-	-	-	-	-	-	-	-	-	-	-	-	-	-	-	-	-	-	-	-	-	-	-	-	-	-	-	-	-	-	-	-	-	-	-	-	-	-	-	-	-	-	-	-	-	-	-	-	-	-	-	-	-	-	+	-
6610	12374	Cation-transporting ATPase	1	-	-	-	-	-	-	-	-	-	-	-	-	-	-	-	-	-	-	-	-	-	-	-	-	-	-	-	-	-	-	-	-	-	-	-	-	-	-	-	-	-	-	-	-	-	-	-	-	-	-	-	-	-	-	-	+	-
6611	12375	Lipopolysaccharide cholinephosphotransferase LicD1 (EC 2.7.8.-)	1	-	-	-	-	-	-	-	-	-	-	-	-	-	-	-	-	-	-	-	-	-	-	-	-	-	-	-	-	-	-	-	-	-	-	-	-	-	-	-	-	-	-	-	-	-	-	-	-	-	-	-	-	-	-	-	+	-
6612	12376	hypothetical protein	1	-	-	-	-	-	-	-	-	-	-	-	-	-	-	-	-	-	-	-	-	-	-	-	-	-	-	-	-	-	-	-	-	-	-	-	-	-	-	-	-	-	-	-	-	-	-	-	-	-	-	-	-	-	-	-	+	-
6613	12377	hypothetical protein	1	-	-	-	-	-	-	-	-	-	-	-	-	-	-	-	-	-	-	-	-	-	-	-	-	-	-	-	-	-	-	-	-	-	-	-	-	-	-	-	-	-	-	-	-	-	-	-	-	-	-	-	-	-	-	-	+	-
6614	12378	hypothetical protein	1	-	-	-	-	-	-	-	-	-	-	-	-	-	-	-	-	-	-	-	-	-	-	-	-	-	-	-	-	-	-	-	-	-	-	-	-	-	-	-	-	-	-	-	-	-	-	-	-	-	-	-	-	-	-	-	+	-
6615	12379	hypothetical protein	1	-	-	-	-	-	-	-	-	-	-	-	-	-	-	-	-	-	-	-	-	-	-	-	-	-	-	-	-	-	-	-	-	-	-	-	-	-	-	-	-	-	-	-	-	-	-	-	-	-	-	-	-	-	-	-	+	-
6616	12380	Transcriptional regulator	1	-	-	-	-	-	-	-	-	-	-	-	-	-	-	-	-	-	-	-	-	-	-	-	-	-	-	-	-	-	-	-	-	-	-	-	-	-	-	-	-	-	-	-	-	-	-	-	-	-	-	-	-	-	-	-	+	-
6617	12381	SSU ribosomal protein S5p (S2e)	1	-	-	-	-	-	-	-	-	-	-	-	-	-	-	-	-	-	-	-	-	-	-	-	-	-	-	-	-	-	-	-	-	-	-	-	-	-	-	-	-	-	-	-	-	-	-	-	-	-	-	-	-	-	-	-	+	-
6618	12382	Heat shock protein 60 family chaperone GroEL	1	-	-	-	-	-	-	-	-	-	-	-	-	-	-	-	-	-	-	-	-	-	-	-	-	-	-	-	-	-	-	-	-	-	-	-	-	-	-	-	-	-	-	-	-	-	-	-	-	-	-	-	-	-	-	-	+	-
6619	12383	hypothetical protein	1	-	-	-	-	-	-	-	-	-	-	-	-	-	-	-	-	-	-	-	-	-	-	-	-	-	-	-	-	-	-	-	-	-	-	-	-	-	-	-	-	-	-	-	-	-	-	-	-	-	-	-	-	-	-	-	+	-
6620	12384	hypothetical protein	1	-	-	-	-	-	-	-	-	-	-	-	-	-	-	-	-	-	-	-	-	-	-	-	-	-	-	-	-	-	-	-	-	-	-	-	-	-	-	-	-	-	-	-	-	-	-	-	-	-	-	-	-	-	-	-	+	-
6621	12385	hypothetical protein	1	-	-	-	-	-	-	-	-	-	-	-	-	-	-	-	-	-	-	-	-	-	-	-	-	-	-	-	-	-	-	-	-	-	-	-	-	-	-	-	-	-	-	-	-	-	-	-	-	-	-	-	-	-	-	-	+	-
6622	12386	polysaccharide polymerase	1	-	-	-	-	-	-	-	-	-	-	-	-	-	-	-	-	-	-	-	-	-	-	-	-	-	-	-	-	-	-	-	-	-	-	-	-	-	-	-	-	-	-	-	-	-	-	-	-	-	-	-	-	-	-	-	+	-
6623	12387	Helicase PriA essential for oriC/DnaA-independent DNA replication	1	-	-	-	-	-	-	-	-	-	-	-	-	-	-	-	-	-	-	-	-	-	-	-	-	-	-	-	-	-	-	-	-	-	-	-	-	-	-	-	-	-	-	-	-	-	-	-	-	-	-	-	-	-	-	-	+	-
6624	12388	hypothetical protein	1	-	-	-	-	-	-	-	-	-	-	-	-	-	-	-	-	-	-	-	-	-	-	-	-	-	-	-	-	-	-	-	-	-	-	-	-	-	-	-	-	-	-	-	-	-	-	-	-	-	-	-	-	-	-	-	+	-
6625	12389	hypothetical protein	1	-	-	-	-	-	-	-	-	-	-	-	-	-	-	-	-	-	-	-	-	-	-	-	-	-	-	-	-	-	-	-	-	-	-	-	-	-	-	-	-	-	-	-	-	-	-	-	-	-	-	-	-	-	-	-	+	-
6626	12390	hypothetical protein	1	-	-	-	-	-	-	-	-	-	-	-	-	-	-	-	-	-	-	-	-	-	-	-	-	-	-	-	-	-	-	-	-	-	-	-	-	-	-	-	-	-	-	-	-	-	-	-	-	-	-	-	-	-	-	-	+	-
6627	12391	hypothetical protein	1	-	-	-	-	-	-	-	-	-	-	-	-	-	-	-	-	-	-	-	-	-	-	-	-	-	-	-	-	-	-	-	-	-	-	-	-	-	-	-	-	-	-	-	-	-	-	-	-	-	-	-	-	-	-	-	+	-
6628	12392	hypothetical protein	1	-	-	-	-	-	-	-	-	-	-	-	-	-	-	-	-	-	-	-	-	-	-	-	-	-	-	-	-	-	-	-	-	-	-	-	-	-	-	-	-	-	-	-	-	-	-	-	-	-	-	-	-	-	-	-	+	-
6629	12393	hypothetical protein	1	-	-	-	-	-	-	-	-	-	-	-	-	-	-	-	-	-	-	-	-	-	-	-	-	-	-	-	-	-	-	-	-	-	-	-	-	-	-	-	-	-	-	-	-	-	-	-	-	-	-	-	-	-	-	-	+	-
6630	12394	hypothetical protein	1	-	-	-	-	-	-	-	-	-	-	-	-	-	-	-	-	-	-	-	-	-	-	-	-	-	-	-	-	-	-	-	-	-	-	-	-	-	-	-	-	-	-	-	-	-	-	-	-	-	-	-	-	-	-	-	+	-
6631	12395	phage head-tail adaptor	1	-	-	-	-	-	-	-	-	-	-	-	-	-	-	-	-	-	-	-	-	-	-	-	-	-	-	-	-	-	-	-	-	-	-	-	-	-	-	-	-	-	-	-	-	-	-	-	-	-	-	-	-	-	-	-	+	-
6632	12396	Protein export cytoplasm protein SecA ATPase RNA helicase (TC 3.A.5.1.1)	1	-	-	-	-	-	-	-	-	-	-	-	-	-	-	-	-	-	-	-	-	-	-	-	-	-	-	-	-	-	-	-	-	-	-	-	-	-	-	-	-	-	-	-	-	-	-	-	-	-	-	-	-	-	-	-	+	-
6633	12397	hypothetical protein	1	-	-	-	-	-	-	-	-	-	-	-	-	-	-	-	-	-	-	-	-	-	-	-	-	-	-	-	-	-	-	-	-	-	-	-	-	-	-	-	-	-	-	-	-	-	-	-	-	-	-	-	-	-	-	-	+	-
6634	12398	hypothetical protein	1	-	-	-	-	-	-	-	-	-	-	-	-	-	-	-	-	-	-	-	-	-	-	-	-	-	-	-	-	-	-	-	-	-	-	-	-	-	-	-	-	-	-	-	-	-	-	-	-	-	-	-	-	-	-	-	+	-
6635	12399	hypothetical protein	1	-	-	-	-	-	-	-	-	-	-	-	-	-	-	-	-	-	-	-	-	-	-	-	-	-	-	-	-	-	-	-	-	-	-	-	-	-	-	-	-	-	-	-	-	-	-	-	-	-	-	-	-	-	-	-	+	-
6636	12400	Replication initiator protein	1	-	-	-	-	-	-	-	-	-	-	-	-	-	-	-	-	-	-	-	-	-	-	-	-	-	-	-	-	-	-	-	-	-	-	-	-	-	-	-	-	-	-	-	-	-	-	-	-	-	-	-	-	-	-	-	+	-
6637	12401	hypothetical protein	1	-	-	-	-	-	-	-	-	-	-	-	-	-	-	-	-	-	-	-	-	-	-	-	-	-	-	-	-	-	-	-	-	-	-	-	-	-	-	-	-	-	-	-	-	-	-	-	-	-	-	-	-	-	-	-	+	-
6638	12402	hypothetical protein	1	-	-	-	-	-	-	-	-	-	-	-	-	-	-	-	-	-	-	-	-	-	-	-	-	-	-	-	-	-	-	-	-	-	-	-	-	-	-	-	-	-	-	-	-	-	-	-	-	-	-	-	-	-	-	-	+	-
6639	12403	rRNA small subunit methyltransferase H	1	-	-	-	-	-	-	-	-	-	-	-	-	-	-	-	-	-	-	-	-	-	-	-	-	-	-	-	-	-	-	-	-	-	-	-	-	-	-	-	-	-	-	-	-	-	-	-	-	-	-	-	-	-	-	-	+	-
6640	12404	hypothetical protein	1	-	-	-	-	-	-	-	-	-	-	-	-	-	-	-	-	-	-	-	-	-	-	-	-	-	-	-	-	-	-	-	-	-	-	-	-	-	-	-	-	-	-	-	-	-	-	-	-	-	-	-	-	-	-	-	+	-
6641	12405	similar to membrane protein	1	-	-	-	-	-	-	-	-	-	-	-	-	-	-	-	-	-	-	-	-	-	-	-	-	-	-	-	-	-	-	-	-	-	-	-	-	-	-	-	-	-	-	-	-	-	-	-	-	-	-	-	-	-	-	-	+	-
6642	12406	hypothetical protein	1	-	-	-	-	-	-	-	-	-	-	-	-	-	-	-	-	-	-	-	-	-	-	-	-	-	-	-	-	-	-	-	-	-	-	-	-	-	-	-	-	-	-	-	-	-	-	-	-	-	-	-	-	-	-	-	+	-
6643	12407	hypothetical protein	1	-	-	-	-	-	-	-	-	-	-	-	-	-	-	-	-	-	-	-	-	-	-	-	-	-	-	-	-	-	-	-	-	-	-	-	-	-	-	-	-	-	-	-	-	-	-	-	-	-	-	-	-	-	-	-	+	-
6644	12408	Putative ABC transporter ATP-binding protein	1	-	-	-	-	-	-	-	-	-	-	-	-	-	-	-	-	-	-	-	-	-	-	-	-	-	-	-	-	-	-	-	-	-	-	-	-	-	-	-	-	-	-	-	-	-	-	-	-	-	-	-	-	-	-	-	+	-
6645	12409	hypothetical protein	1	-	-	-	-	-	-	-	-	-	-	-	-	-	-	-	-	-	-	-	-	-	-	-	-	-	-	-	-	-	-	-	-	-	-	-	-	-	-	-	-	-	-	-	-	-	-	-	-	-	-	-	-	-	-	-	+	-
6646	12410	hypothetical protein	1	-	-	-	-	-	-	-	-	-	-	-	-	-	-	-	-	-	-	-	-	-	-	-	-	-	-	-	-	-	-	-	-	-	-	-	-	-	-	-	-	-	-	-	-	-	-	-	-	-	-	-	-	-	-	-	+	-
6647	12411	rRNA small subunit methyltransferase I	1	-	-	-	-	-	-	-	-	-	-	-	-	-	-	-	-	-	-	-	-	-	-	-	-	-	-	-	-	-	-	-	-	-	-	-	-	-	-	-	-	-	-	-	-	-	-	-	-	-	-	-	-	-	-	-	+	-
6648	12412	hypothetical protein	1	-	-	-	-	-	-	-	-	-	-	-	-	-	-	-	-	-	-	-	-	-	-	-	-	-	-	-	-	-	-	-	-	-	-	-	-	-	-	-	-	-	-	-	-	-	-	-	-	-	-	-	-	-	-	-	+	-
6649	12413	Transcriptional regulator	1	-	-	-	-	-	-	-	-	-	-	-	-	-	-	-	-	-	-	-	-	-	-	-	-	-	-	-	-	-	-	-	-	-	-	-	-	-	-	-	-	-	-	-	-	-	-	-	-	-	-	-	-	-	-	-	+	-
6650	12414	hypothetical protein	1	-	-	-	-	-	-	-	-	-	-	-	-	-	-	-	-	-	-	-	-	-	-	-	-	-	-	-	-	-	-	-	-	-	-	-	-	-	-	-	-	-	-	-	-	-	-	-	-	-	-	-	-	-	-	-	+	-
6651	12415	hypothetical protein	1	-	-	-	-	-	-	-	-	-	-	-	-	-	-	-	-	-	-	-	-	-	-	-	-	-	-	-	-	-	-	-	-	-	-	-	-	-	-	-	-	-	-	-	-	-	-	-	-	-	-	-	-	-	-	-	+	-
6652	12416	cell wall surface anchor family protein	1	-	-	-	-	-	-	-	-	-	-	-	-	-	-	-	-	-	-	-	-	-	-	-	-	-	-	-	-	-	-	-	-	-	-	-	-	-	-	-	-	-	-	-	-	-	-	-	-	-	-	-	-	-	-	-	+	-
6653	12417	hypothetical protein	1	-	-	-	-	-	-	-	-	-	-	-	-	-	-	-	-	-	-	-	-	-	-	-	-	-	-	-	-	-	-	-	-	-	-	-	-	-	-	-	-	-	-	-	-	-	-	-	-	-	-	-	-	-	-	-	+	-
6654	12418	hypothetical protein	1	-	-	-	-	-	-	-	-	-	-	-	-	-	-	-	-	-	-	-	-	-	-	-	-	-	-	-	-	-	-	-	-	-	-	-	-	-	-	-	-	-	-	-	-	-	-	-	-	-	-	-	-	-	-	-	+	-
6655	12419	Acetyltransferase	1	-	-	-	-	-	-	-	-	-	-	-	-	-	-	-	-	-	-	-	-	-	-	-	-	-	-	-	-	-	-	-	-	-	-	-	-	-	-	-	-	-	-	-	-	-	-	-	-	-	-	-	-	-	-	-	+	-
6656	12420	cell wall-anchored protein	1	-	-	-	-	-	-	-	-	-	-	-	-	-	-	-	-	-	-	-	-	-	-	-	-	-	-	-	-	-	-	-	-	-	-	-	-	-	-	-	-	-	-	-	-	-	-	-	-	-	-	-	-	-	-	-	+	-
6657	12421	tRNA (5-methylaminomethyl-2-thiouridylate)-methyltransferase (EC 2.1.1.61)	1	-	-	-	-	-	-	-	-	-	-	-	-	-	-	-	-	-	-	-	-	-	-	-	-	-	-	-	-	-	-	-	-	-	-	-	-	-	-	-	-	-	-	-	-	-	-	-	-	-	-	-	-	-	-	-	+	-
6658	12422	hypothetical protein	1	-	-	-	-	-	-	-	-	-	-	-	-	-	-	-	-	-	-	-	-	-	-	-	-	-	-	-	-	-	-	-	-	-	-	-	-	-	-	-	-	-	-	-	-	-	-	-	-	-	-	-	-	-	-	-	+	-
6659	12423	rRNA biogenesis protein rrp5	1	-	-	-	-	-	-	-	-	-	-	-	-	-	-	-	-	-	-	-	-	-	-	-	-	-	-	-	-	-	-	-	-	-	-	-	-	-	-	-	-	-	-	-	-	-	-	-	-	-	-	-	-	-	-	-	+	-
6660	12424	hypothetical protein	1	-	-	-	-	-	-	-	-	-	-	-	-	-	-	-	-	-	-	-	-	-	-	-	-	-	-	-	-	-	-	-	-	-	-	-	-	-	-	-	-	-	-	-	-	-	-	-	-	-	-	-	-	-	-	-	+	-
6661	12425	hypothetical protein	1	-	-	-	-	-	-	-	-	-	-	-	-	-	-	-	-	-	-	-	-	-	-	-	-	-	-	-	-	-	-	-	-	-	-	-	-	-	-	-	-	-	-	-	-	-	-	-	-	-	-	-	-	-	-	-	+	-
6662	12426	hypothetical protein	1	-	-	-	-	-	-	-	-	-	-	-	-	-	-	-	-	-	-	-	-	-	-	-	-	-	-	-	-	-	-	-	-	-	-	-	-	-	-	-	-	-	-	-	-	-	-	-	-	-	-	-	-	-	-	-	+	-
6663	12427	hypothetical protein	1	-	-	-	-	-	-	-	-	-	-	-	-	-	-	-	-	-	-	-	-	-	-	-	-	-	-	-	-	-	-	-	-	-	-	-	-	-	-	-	-	-	-	-	-	-	-	-	-	-	-	-	-	-	-	-	+	-
6664	12428	Cation-transporting ATPase	1	-	-	-	-	-	-	-	-	-	-	-	-	-	-	-	-	-	-	-	-	-	-	-	-	-	-	-	-	-	-	-	-	-	-	-	-	-	-	-	-	-	-	-	-	-	-	-	-	-	-	-	-	-	-	-	+	-
6665	12429	Phage protein	1	-	-	-	-	-	-	-	-	-	-	-	-	-	-	-	-	-	-	-	-	-	-	-	-	-	-	-	-	-	-	-	-	-	-	-	-	-	-	-	-	-	-	-	-	-	-	-	-	-	-	-	-	-	-	-	+	-
6666	12430	hypothetical protein	1	-	-	-	-	-	-	-	-	-	-	-	-	-	-	-	-	-	-	-	-	-	-	-	-	-	-	-	-	-	-	-	-	-	-	-	-	-	-	-	-	-	-	-	-	-	-	-	-	-	-	-	-	-	-	-	+	-
6667	12431	hypothetical protein	1	-	-	-	-	-	-	-	-	-	-	-	-	-	-	-	-	-	-	-	-	-	-	-	-	-	-	-	-	-	-	-	-	-	-	-	-	-	-	-	-	-	-	-	-	-	-	-	-	-	-	-	-	-	-	-	+	-
6668	12432	hypothetical protein	1	-	-	-	-	-	-	-	-	-	-	-	-	-	-	-	-	-	-	-	-	-	-	-	-	-	-	-	-	-	-	-	-	-	-	-	-	-	-	-	-	-	-	-	-	-	-	-	-	-	-	-	-	-	-	-	+	-
6669	12433	hypothetical protein	1	-	-	-	-	-	-	-	-	-	-	-	-	-	-	-	-	-	-	-	-	-	-	-	-	-	-	-	-	-	-	-	-	-	-	-	-	-	-	-	-	-	-	-	-	-	-	-	-	-	-	-	-	-	-	-	+	-
6670	12434	hypothetical protein	1	-	-	-	-	-	-	-	-	-	-	-	-	-	-	-	-	-	-	-	-	-	-	-	-	-	-	-	-	-	-	-	-	-	-	-	-	-	-	-	-	-	-	-	-	-	-	-	-	-	-	-	-	-	-	-	+	-
6671	12435	hypothetical protein	1	-	-	-	-	-	-	-	-	-	-	-	-	-	-	-	-	-	-	-	-	-	-	-	-	-	-	-	-	-	-	-	-	-	-	-	-	-	-	-	-	-	-	-	-	-	-	-	-	-	-	-	-	-	-	-	+	-
6672	12436	hypothetical protein	1	-	-	-	-	-	-	-	-	-	-	-	-	-	-	-	-	-	-	-	-	-	-	-	-	-	-	-	-	-	-	-	-	-	-	-	-	-	-	-	-	-	-	-	-	-	-	-	-	-	-	-	-	-	-	-	+	-
6673	12437	hypothetical protein	1	-	-	-	-	-	-	-	-	-	-	-	-	-	-	-	-	-	-	-	-	-	-	-	-	-	-	-	-	-	-	-	-	-	-	-	-	-	-	-	-	-	-	-	-	-	-	-	-	-	-	-	-	-	-	-	+	-
6674	12438	hypothetical protein	1	-	-	-	-	-	-	-	-	-	-	-	-	-	-	-	-	-	-	-	-	-	-	-	-	-	-	-	-	-	-	-	-	-	-	-	-	-	-	-	-	-	-	-	-	-	-	-	-	-	-	-	-	-	-	-	+	-
6675	12439	hypothetical protein	1	-	-	-	-	-	-	-	-	-	-	-	-	-	-	-	-	-	-	-	-	-	-	-	-	-	-	-	-	-	-	-	-	-	-	-	-	-	-	-	-	-	-	-	-	-	-	-	-	-	-	-	-	-	-	-	+	-
6676	12440	hypothetical protein	1	-	-	-	-	-	-	-	-	-	-	-	-	-	-	-	-	-	-	-	-	-	-	-	-	-	-	-	-	-	-	-	-	-	-	-	-	-	-	-	-	-	-	-	-	-	-	-	-	-	-	-	-	-	-	-	+	-
6677	12441	hypothetical protein	1	-	-	-	-	-	-	-	-	-	-	-	-	-	-	-	-	-	-	-	-	-	-	-	-	-	-	-	-	-	-	-	-	-	-	-	-	-	-	-	-	-	-	-	-	-	-	-	-	-	-	-	-	-	-	-	+	-
6678	12442	Hydroxyethylthiazole kinase (EC 2.7.1.50)	1	-	-	-	-	-	-	-	-	-	-	-	-	-	-	-	-	-	-	-	-	-	-	-	-	-	-	-	-	-	-	-	-	-	-	-	-	-	-	-	-	-	-	-	-	-	-	-	-	-	-	-	-	-	-	-	+	-
6679	12443	Phage-related protein	1	-	-	-	-	-	-	-	-	-	-	-	-	-	-	-	-	-	-	-	-	-	-	-	-	-	-	-	-	-	-	-	-	-	-	-	-	-	-	-	-	-	-	-	-	-	-	-	-	-	-	-	-	-	-	-	+	-
6680	12444	DNA polymerase III alpha subunit (EC 2.7.7.7)	1	-	-	-	-	-	-	-	-	-	-	-	-	-	-	-	-	-	-	-	-	-	-	-	-	-	-	-	-	-	-	-	-	-	-	-	-	-	-	-	-	-	-	-	-	-	-	-	-	-	-	-	-	-	-	-	+	-
6681	12445	TPR-repeat-containing protein	1	-	-	-	-	-	-	-	-	-	-	-	-	-	-	-	-	-	-	-	-	-	-	-	-	-	-	-	-	-	-	-	-	-	-	-	-	-	-	-	-	-	-	-	-	-	-	-	-	-	-	-	-	-	-	-	+	-
6682	12446	hypothetical protein	1	-	-	-	-	-	-	-	-	-	-	-	-	-	-	-	-	-	-	-	-	-	-	-	-	-	-	-	-	-	-	-	-	-	-	-	-	-	-	-	-	-	-	-	-	-	-	-	-	-	-	-	-	-	-	-	+	-
6683	12447	CS_D9/CD37/CD63 antigen	1	-	-	-	-	-	-	-	-	-	-	-	-	-	-	-	-	-	-	-	-	-	-	-	-	-	-	-	-	-	-	-	-	-	-	-	-	-	-	-	-	-	-	-	-	-	-	-	-	-	-	-	-	-	-	-	+	-
6684	12448	hypothetical protein	1	-	-	-	-	-	-	-	-	-	-	-	-	-	-	-	-	-	-	-	-	-	-	-	-	-	-	-	-	-	-	-	-	-	-	-	-	-	-	-	-	-	-	-	-	-	-	-	-	-	-	-	-	-	-	-	+	-
6685	12449	hypothetical protein	1	-	-	-	-	-	-	-	-	-	-	-	-	-	-	-	-	-	-	-	-	-	-	-	-	-	-	-	-	-	-	-	-	-	-	-	-	-	-	-	-	-	-	-	-	-	-	-	-	-	-	-	-	-	-	-	+	-
6686	12450	O-antigen flippase Wzx	1	-	-	-	-	-	-	-	-	-	-	-	-	-	-	-	-	-	-	-	-	-	-	-	-	-	-	-	-	-	-	-	-	-	-	-	-	-	-	-	-	-	-	-	-	-	-	-	-	-	-	-	-	-	-	-	+	-
6687	12451	Serine--glyoxylate aminotransferase (EC 2.6.1.45)	1	-	-	-	-	-	-	-	-	-	-	-	-	-	-	-	-	-	-	-	-	-	-	-	-	-	-	-	-	-	-	-	-	-	-	-	-	-	-	-	-	-	-	-	-	-	-	-	-	-	-	-	-	-	-	-	+	-
6688	12452	hypothetical protein	1	-	-	-	-	-	-	-	-	-	-	-	-	-	-	-	-	-	-	-	-	-	-	-	-	-	-	-	-	-	-	-	-	-	-	-	-	-	-	-	-	-	-	-	-	-	-	-	-	-	-	-	-	-	-	-	+	-
6689	12453	hypothetical protein	1	-	-	-	-	-	-	-	-	-	-	-	-	-	-	-	-	-	-	-	-	-	-	-	-	-	-	-	-	-	-	-	-	-	-	-	-	-	-	-	-	-	-	-	-	-	-	-	-	-	-	-	-	-	-	-	+	-
6690	12454	Leucyl-tRNA synthetase (EC 6.1.1.4)	1	-	-	-	-	-	-	-	-	-	-	-	-	-	-	-	-	-	-	-	-	-	-	-	-	-	-	-	-	-	-	-	-	-	-	-	-	-	-	-	-	-	-	-	-	-	-	-	-	-	-	-	-	-	-	-	+	-
6691	12455	Phage major tail protein phi13	1	-	-	-	-	-	-	-	-	-	-	-	-	-	-	-	-	-	-	-	-	-	-	-	-	-	-	-	-	-	-	-	-	-	-	-	-	-	-	-	-	-	-	-	-	-	-	-	-	-	-	-	-	-	-	-	+	-
6692	12456	Dimethyladenosine transferase (EC 2.1.1.-)	1	-	-	-	-	-	-	-	-	-	-	-	-	-	-	-	-	-	-	-	-	-	-	-	-	-	-	-	-	-	-	-	-	-	-	-	-	-	-	-	-	-	-	-	-	-	-	-	-	-	-	-	-	-	-	-	+	-
6693	12457	Cro-like protein	1	-	-	-	-	-	-	-	-	-	-	-	-	-	-	-	-	-	-	-	-	-	-	-	-	-	-	-	-	-	-	-	-	-	-	-	-	-	-	-	-	-	-	-	-	-	-	-	-	-	-	-	-	-	-	-	+	-
6694	12458	hypothetical protein	1	-	-	-	-	-	-	-	-	-	-	-	-	-	-	-	-	-	-	-	-	-	-	-	-	-	-	-	-	-	-	-	-	-	-	-	-	-	-	-	-	-	-	-	-	-	-	-	-	-	-	-	-	-	-	-	+	-
6695	12459	hypothetical protein	1	-	-	-	-	-	-	-	-	-	-	-	-	-	-	-	-	-	-	-	-	-	-	-	-	-	-	-	-	-	-	-	-	-	-	-	-	-	-	-	-	-	-	-	-	-	-	-	-	-	-	-	-	-	-	-	+	-
6696	12460	hypothetical protein	1	-	-	-	-	-	-	-	-	-	-	-	-	-	-	-	-	-	-	-	-	-	-	-	-	-	-	-	-	-	-	-	-	-	-	-	-	-	-	-	-	-	-	-	-	-	-	-	-	-	-	-	-	-	-	-	+	-
6697	12461	hypothetical protein	1	-	-	-	-	-	-	-	-	-	-	-	-	-	-	-	-	-	-	-	-	-	-	-	-	-	-	-	-	-	-	-	-	-	-	-	-	-	-	-	-	-	-	-	-	-	-	-	-	-	-	-	-	-	-	-	+	-
6698	12462	hypothetical protein	1	-	-	-	-	-	-	-	-	-	-	-	-	-	-	-	-	-	-	-	-	-	-	-	-	-	-	-	-	-	-	-	-	-	-	-	-	-	-	-	-	-	-	-	-	-	-	-	-	-	-	-	-	-	-	-	+	-
6699	12463	hypothetical protein	1	-	-	-	-	-	-	-	-	-	-	-	-	-	-	-	-	-	-	-	-	-	-	-	-	-	-	-	-	-	-	-	-	-	-	-	-	-	-	-	-	-	-	-	-	-	-	-	-	-	-	-	-	-	-	-	+	-
6700	12464	hypothetical protein	1	-	-	-	-	-	-	-	-	-	-	-	-	-	-	-	-	-	-	-	-	-	-	-	-	-	-	-	-	-	-	-	-	-	-	-	-	-	-	-	-	-	-	-	-	-	-	-	-	-	-	-	-	-	-	-	+	-
6701	12465	hypothetical protein	1	-	-	-	-	-	-	-	-	-	-	-	-	-	-	-	-	-	-	-	-	-	-	-	-	-	-	-	-	-	-	-	-	-	-	-	-	-	-	-	-	-	-	-	-	-	-	-	-	-	-	-	-	-	-	-	+	-
6702	12466	Predicted cell-wall-anchored protein SasA (LPXTG motif)	1	-	-	-	-	-	-	-	-	-	-	-	-	-	-	-	-	-	-	-	-	-	-	-	-	-	-	-	-	-	-	-	-	-	-	-	-	-	-	-	-	-	-	-	-	-	-	-	-	-	-	-	-	-	-	-	+	-
6703	12467	hypothetical protein	1	-	-	-	-	-	-	-	-	-	-	-	-	-	-	-	-	-	-	-	-	-	-	-	-	-	-	-	-	-	-	-	-	-	-	-	-	-	-	-	-	-	-	-	-	-	-	-	-	-	-	-	-	-	-	-	+	-
6704	12468	Cholinephosphate cytidylyltransferase (EC 2.7.7.15)	1	-	-	-	-	-	-	-	-	-	-	-	-	-	-	-	-	-	-	-	-	-	-	-	-	-	-	-	-	-	-	-	-	-	-	-	-	-	-	-	-	-	-	-	-	-	-	-	-	-	-	-	-	-	-	-	+	-
6705	12469	membrane protein	1	-	-	-	-	-	-	-	-	-	-	-	-	-	-	-	-	-	-	-	-	-	-	-	-	-	-	-	-	-	-	-	-	-	-	-	-	-	-	-	-	-	-	-	-	-	-	-	-	-	-	-	-	-	-	-	+	-
6706	12470	Adenylosuccinate lyase (EC 4.3.2.2)	1	-	-	-	-	-	-	-	-	-	-	-	-	-	-	-	-	-	-	-	-	-	-	-	-	-	-	-	-	-	-	-	-	-	-	-	-	-	-	-	-	-	-	-	-	-	-	-	-	-	-	-	-	-	-	-	+	-
6707	12471	virulence-related protein	1	-	-	-	-	-	-	-	-	-	-	-	-	-	-	-	-	-	-	-	-	-	-	-	-	-	-	-	-	-	-	-	-	-	-	-	-	-	-	-	-	-	-	-	-	-	-	-	-	-	-	-	-	-	-	-	+	-
6708	12472	hypothetical protein	1	-	-	-	-	-	-	-	-	-	-	-	-	-	-	-	-	-	-	-	-	-	-	-	-	-	-	-	-	-	-	-	-	-	-	-	-	-	-	-	-	-	-	-	-	-	-	-	-	-	-	-	-	-	-	-	+	-
6709	12473	hypothetical protein	1	-	-	-	-	-	-	-	-	-	-	-	-	-	-	-	-	-	-	-	-	-	-	-	-	-	-	-	-	-	-	-	-	-	-	-	-	-	-	-	-	-	-	-	-	-	-	-	-	-	-	-	-	-	-	-	+	-
6710	12474	hypothetical protein	1	-	-	-	-	-	-	-	-	-	-	-	-	-	-	-	-	-	-	-	-	-	-	-	-	-	-	-	-	-	-	-	-	-	-	-	-	-	-	-	-	-	-	-	-	-	-	-	-	-	-	-	-	-	-	-	+	-
6711	12475	hypothetical protein	1	-	-	-	-	-	-	-	-	-	-	-	-	-	-	-	-	-	-	-	-	-	-	-	-	-	-	-	-	-	-	-	-	-	-	-	-	-	-	-	-	-	-	-	-	-	-	-	-	-	-	-	-	-	-	-	+	-
6712	12476	Cytosine (N4) specific methyltransferase(EC:2.1.1.113)	1	-	-	-	-	-	-	-	-	-	-	-	-	-	-	-	-	-	-	-	-	-	-	-	-	-	-	-	-	-	-	-	-	-	-	-	-	-	-	-	-	-	-	-	-	-	-	-	-	-	-	-	-	-	-	-	+	-
6713	12477	hypothetical protein	1	-	-	-	-	-	-	-	-	-	-	-	-	-	-	-	-	-	-	-	-	-	-	-	-	-	-	-	-	-	-	-	-	-	-	-	-	-	-	-	-	-	-	-	-	-	-	-	-	-	-	-	-	-	-	-	+	-
6714	12478	N-acetylmuramoyl-L-alanine amidase domain protein	1	-	-	-	-	-	-	-	-	-	-	-	-	-	-	-	-	-	-	-	-	-	-	-	-	-	-	-	-	-	-	-	-	-	-	-	-	-	-	-	-	-	-	-	-	-	-	-	-	-	-	-	-	-	-	-	+	-
6715	12479	hypothetical protein	1	-	-	-	-	-	-	-	-	-	-	-	-	-	-	-	-	-	-	-	-	-	-	-	-	-	-	-	-	-	-	-	-	-	-	-	-	-	-	-	-	-	-	-	-	-	-	-	-	-	-	-	-	-	-	-	+	-
6716	12480	Isopentenyl-diphosphate delta-isomerase	1	-	-	-	-	-	-	-	-	-	-	-	-	-	-	-	-	-	-	-	-	-	-	-	-	-	-	-	-	-	-	-	-	-	-	-	-	-	-	-	-	-	-	-	-	-	-	-	-	-	-	-	-	-	-	-	+	-
6717	12481	Glycosyl transferase	1	-	-	-	-	-	-	-	-	-	-	-	-	-	-	-	-	-	-	-	-	-	-	-	-	-	-	-	-	-	-	-	-	-	-	-	-	-	-	-	-	-	-	-	-	-	-	-	-	-	-	-	-	-	-	-	+	-
6718	12482	hypothetical protein	1	-	-	-	-	-	-	-	-	-	-	-	-	-	-	-	-	-	-	-	-	-	-	-	-	-	-	-	-	-	-	-	-	-	-	-	-	-	-	-	-	-	-	-	-	-	-	-	-	-	-	-	-	-	-	-	+	-
6719	12483	hypothetical protein	1	-	-	-	-	-	-	-	-	-	-	-	-	-	-	-	-	-	-	-	-	-	-	-	-	-	-	-	-	-	-	-	-	-	-	-	-	-	-	-	-	-	-	-	-	-	-	-	-	-	-	-	-	-	-	-	+	-
6720	12484	hypothetical protein	1	-	-	-	-	-	-	-	-	-	-	-	-	-	-	-	-	-	-	-	-	-	-	-	-	-	-	-	-	-	-	-	-	-	-	-	-	-	-	-	-	-	-	-	-	-	-	-	-	-	-	-	-	-	-	-	+	-
6721	12485	hypothetical protein	1	-	-	-	-	-	-	-	-	-	-	-	-	-	-	-	-	-	-	-	-	-	-	-	-	-	-	-	-	-	-	-	-	-	-	-	-	-	-	-	-	-	-	-	-	-	-	-	-	-	-	-	-	-	-	-	+	-
6722	12486	hypothetical protein	1	-	-	-	-	-	-	-	-	-	-	-	-	-	-	-	-	-	-	-	-	-	-	-	-	-	-	-	-	-	-	-	-	-	-	-	-	-	-	-	-	-	-	-	-	-	-	-	-	-	-	-	-	-	-	-	+	-
6723	12487	hypothetical protein	1	-	-	-	-	-	-	-	-	-	-	-	-	-	-	-	-	-	-	-	-	-	-	-	-	-	-	-	-	-	-	-	-	-	-	-	-	-	-	-	-	-	-	-	-	-	-	-	-	-	-	-	-	-	-	-	+	-
6724	12488	Phage antirepressor protein	1	-	-	-	-	-	-	-	-	-	-	-	-	-	-	-	-	-	-	-	-	-	-	-	-	-	-	-	-	-	-	-	-	-	-	-	-	-	-	-	-	-	-	-	-	-	-	-	-	-	-	-	-	-	-	-	+	-
6725	12489	hypothetical protein	1	-	-	-	-	-	-	-	-	-	-	-	-	-	-	-	-	-	-	-	-	-	-	-	-	-	-	-	-	-	-	-	-	-	-	-	-	-	-	-	-	-	-	-	-	-	-	-	-	-	-	-	-	-	-	-	+	-
6726	12490	hypothetical protein	1	-	-	-	-	-	-	-	-	-	-	-	-	-	-	-	-	-	-	-	-	-	-	-	-	-	-	-	-	-	-	-	-	-	-	-	-	-	-	-	-	-	-	-	-	-	-	-	-	-	-	-	-	-	-	-	+	-
6727	12491	virulence-related protein	1	-	-	-	-	-	-	-	-	-	-	-	-	-	-	-	-	-	-	-	-	-	-	-	-	-	-	-	-	-	-	-	-	-	-	-	-	-	-	-	-	-	-	-	-	-	-	-	-	-	-	-	-	-	-	-	+	-
6728	12492	hypothetical protein	1	-	-	-	-	-	-	-	-	-	-	-	-	-	-	-	-	-	-	-	-	-	-	-	-	-	-	-	-	-	-	-	-	-	-	-	-	-	-	-	-	-	-	-	-	-	-	-	-	-	-	-	-	-	-	-	+	-
6729	12493	hypothetical protein	1	-	-	-	-	-	-	-	-	-	-	-	-	-	-	-	-	-	-	-	-	-	-	-	-	-	-	-	-	-	-	-	-	-	-	-	-	-	-	-	-	-	-	-	-	-	-	-	-	-	-	-	-	-	-	-	+	-
6730	12494	Hydroxymethylpyrimidine phosphate kinase ThiD (EC 2.7.4.7) / Thiamin-phosphate pyrophosphorylase (EC 2.5.1.3) / Hydroxymethylpyrimidine kinase (EC 2.7.1.49)	1	-	-	-	-	-	-	-	-	-	-	-	-	-	-	-	-	-	-	-	-	-	-	-	-	-	-	-	-	-	-	-	-	-	-	-	-	-	-	-	-	-	-	-	-	-	-	-	-	-	-	-	-	-	-	-	-	-	+	-
6731	12495	hypothetical protein	1	-	-	-	-	-	-	-	-	-	-	-	-	-	-	-	-	-	-	-	-	-	-	-	-	-	-	-	-	-	-	-	-	-	-	-	-	-	-	-	-	-	-	-	-	-	-	-	-	-	-	-	-	-	-	-	+	-
6732	12496	hypothetical protein	1	-	-	-	-	-	-	-	-	-	-	-	-	-	-	-	-	-	-	-	-	-	-	-	-	-	-	-	-	-	-	-	-	-	-	-	-	-	-	-	-	-	-	-	-	-	-	-	-	-	-	-	-	-	-	-	+	-
6733	12497	hypothetical protein	1	-	-	-	-	-	-	-	-	-	-	-	-	-	-	-	-	-	-	-	-	-	-	-	-	-	-	-	-	-	-	-	-	-	-	-	-	-	-	-	-	-	-	-	-	-	-	-	-	-	-	-	-	-	-	-	+	-
6734	12498	lantibiotic efflux protein	1	-	-	-	-	-	-	-	-	-	-	-	-	-	-	-	-	-	-	-	-	-	-	-	-	-	-	-	-	-	-	-	-	-	-	-	-	-	-	-	-	-	-	-	-	-	-	-	-	-	-	-	-	-	-	-	+	-
6735	12499	hypothetical protein	1	-	-	-	-	-	-	-	-	-	-	-	-	-	-	-	-	-	-	-	-	-	-	-	-	-	-	-	-	-	-	-	-	-	-	-	-	-	-	-	-	-	-	-	-	-	-	-	-	-	-	-	-	-	-	-	+	-
6736	12500	transcriptional regulator	1	-	-	-	-	-	-	-	-	-	-	-	-	-	-	-	-	-	-	-	-	-	-	-	-	-	-	-	-	-	-	-	-	-	-	-	-	-	-	-	-	-	-	-	-	-	-	-	-	-	-	-	-	-	-	-	+	-
6737	12501	hypothetical protein	1	-	-	-	-	-	-	-	-	-	-	-	-	-	-	-	-	-	-	-	-	-	-	-	-	-	-	-	-	-	-	-	-	-	-	-	-	-	-	-	-	-	-	-	-	-	-	-	-	-	-	-	-	-	-	-	+	-
6738	12502	hypothetical protein	1	-	-	-	-	-	-	-	-	-	-	-	-	-	-	-	-	-	-	-	-	-	-	-	-	-	-	-	-	-	-	-	-	-	-	-	-	-	-	-	-	-	-	-	-	-	-	-	-	-	-	-	-	-	-	-	+	-
6739	12503	hypothetical protein	1	-	-	-	-	-	-	-	-	-	-	-	-	-	-	-	-	-	-	-	-	-	-	-	-	-	-	-	-	-	-	-	-	-	-	-	-	-	-	-	-	-	-	-	-	-	-	-	-	-	-	-	-	-	-	-	+	-
6740	12504	hypothetical protein	1	-	-	-	-	-	-	-	-	-	-	-	-	-	-	-	-	-	-	-	-	-	-	-	-	-	-	-	-	-	-	-	-	-	-	-	-	-	-	-	-	-	-	-	-	-	-	-	-	-	-	-	-	-	-	-	+	-
6741	12505	Muramidase-released protein precursor (136 kDa surface protein)	1	-	-	-	-	-	-	-	-	-	-	-	-	-	-	-	-	-	-	-	-	-	-	-	-	-	-	-	-	-	-	-	-	-	-	-	-	-	-	-	-	-	-	-	-	-	-	-	-	-	-	-	-	-	-	-	+	-
6742	12506	hypothetical protein	1	-	-	-	-	-	-	-	-	-	-	-	-	-	-	-	-	-	-	-	-	-	-	-	-	-	-	-	-	-	-	-	-	-	-	-	-	-	-	-	-	-	-	-	-	-	-	-	-	-	-	-	-	-	-	-	+	-
6743	12507	hypothetical protein	1	-	-	-	-	-	-	-	-	-	-	-	-	-	-	-	-	-	-	-	-	-	-	-	-	-	-	-	-	-	-	-	-	-	-	-	-	-	-	-	-	-	-	-	-	-	-	-	-	-	-	-	-	-	-	-	+	-

Table S5. Precence/absence of each HG among 57 *S. suis* strains

				1																13			25					28						104		94			76			53/54		Divergent *S. suis*																
Index[a]	ClusterID	Gene products	Members	98HAH33	05ZYH33	SC84	A7	SS12	P1/7	DAT273	NIAH11434	JS14	GZ1	BM407	S735	13730	MNCM07	NCTC 10446	14636	NCTC 10237	ST1	2651	89-1591	MNCM04	MNCM25	8074	D9	4961	ST3	MNCM43	DAT292	DAT260	NIAH11435	MNCM21	MNCM50	89-2479	DAT234	DAT290	93A	42A	NT77	6407	11538	8830	12814	92-1400	89-590	2726	2524	89-3576-3	4417	D12	10581	89-5259	88-5299A	R61	92-1191	14A	92-4172	22083
6744	12508	IMP cyclohydrolase (EC 3.5.4.10) / Phosphoribosylaminoimidazolecarboxamide formyltransferase (EC 2.1.2.3)	1	-	-	-	-	-	-	-	-	-	-	-	-	-	-	-	-	-	-	-	-	-	-	-	-	-	-	-	-	-	-	-	-	-	-	-	-	-	-	-	-	-	-	-	-	-	-	-	-	-	-	-	-	-	-	-	+	-
6745	12509	Phosphoribosylformylglycinamidine synthase	1	-	-	-	-	-	-	-	-	-	-	-	-	-	-	-	-	-	-	-	-	-	-	-	-	-	-	-	-	-	-	-	-	-	-	-	-	-	-	-	-	-	-	-	-	-	-	-	-	-	-	-	-	-	-	-	+	-
6746	12510	DNA-directed RNA polymerase beta subunit (EC 2.7.7.6)	1	-	-	-	-	-	-	-	-	-	-	-	-	-	-	-	-	-	-	-	-	-	-	-	-	-	-	-	-	-	-	-	-	-	-	-	-	-	-	-	-	-	-	-	-	-	-	-	-	-	-	-	-	-	-	-	+	-
6747	12511	tRNA S(4)U 4-thiouridine synthase (former ThiI)	1	-	-	-	-	-	-	-	-	-	-	-	-	-	-	-	-	-	-	-	-	-	-	-	-	-	-	-	-	-	-	-	-	-	-	-	-	-	-	-	-	-	-	-	-	-	-	-	-	-	-	-	-	-	-	-	+	-
6748	12512	hypothetical protein	1	-	-	-	-	-	-	-	-	-	-	-	-	-	-	-	-	-	-	-	-	-	-	-	-	-	-	-	-	-	-	-	-	-	-	-	-	-	-	-	-	-	-	-	-	-	-	-	-	-	-	-	-	-	-	-	+	-
6749	12513	Methionine ABC transporter ATP-binding protein	1	-	-	-	-	-	-	-	-	-	-	-	-	-	-	-	-	-	-	-	-	-	-	-	-	-	-	-	-	-	-	-	-	-	-	-	-	-	-	-	-	-	-	-	-	-	-	-	-	-	-	-	-	-	-	-	+	-
6750	12514	Translation elongation factor LepA	1	-	-	-	-	-	-	-	-	-	-	-	-	-	-	-	-	-	-	-	-	-	-	-	-	-	-	-	-	-	-	-	-	-	-	-	-	-	-	-	-	-	-	-	-	-	-	-	-	-	-	-	-	-	-	-	+	-
6751	12515	site-specific recombinase	1	-	-	-	-	-	-	-	-	-	-	-	-	-	-	-	-	-	-	-	-	-	-	-	-	-	-	-	-	-	-	-	-	-	-	-	-	-	-	-	-	-	-	-	-	-	-	-	-	-	-	-	-	-	-	-	+	-
6752	12516	hypothetical protein	1	-	-	-	-	-	-	-	-	-	-	-	-	-	-	-	-	-	-	-	-	-	-	-	-	-	-	-	-	-	-	-	-	-	-	-	-	-	-	-	-	-	-	-	-	-	-	-	-	-	-	-	-	-	-	-	+	-
6753	13133	Integrase/recombinase	1	-	-	-	-	-	-	-	-	-	-	-	-	-	-	-	-	-	-	+	-	-	-	-	-	-	-	-	-	-	-	-	-	-	-	-	-	-	-	-	-	-	-	-	-	-	-	-	-	-	-	-	-	-	-	-	-	-
6754	13134	hypothetical protein	1	-	-	-	-	-	-	-	-	-	-	-	-	-	-	-	-	-	-	+	-	-	-	-	-	-	-	-	-	-	-	-	-	-	-	-	-	-	-	-	-	-	-	-	-	-	-	-	-	-	-	-	-	-	-	-	-	-
6755	13135	Tryptophan synthase beta chain (EC 4.2.1.20)	1	-	-	-	-	-	-	-	-	-	-	-	-	-	-	-	-	-	-	+	-	-	-	-	-	-	-	-	-	-	-	-	-	-	-	-	-	-	-	-	-	-	-	-	-	-	-	-	-	-	-	-	-	-	-	-	-	-
6756	13136	PTS system	1	-	-	-	-	-	-	-	-	-	-	-	-	-	-	-	-	-	-	+	-	-	-	-	-	-	-	-	-	-	-	-	-	-	-	-	-	-	-	-	-	-	-	-	-	-	-	-	-	-	-	-	-	-	-	-	-	-
6757	13137	hypothetical protein	1	-	-	-	-	-	-	-	-	-	-	-	-	-	-	-	-	-	-	+	-	-	-	-	-	-	-	-	-	-	-	-	-	-	-	-	-	-	-	-	-	-	-	-	-	-	-	-	-	-	-	-	-	-	-	-	-	-
6758	13138	Type III restriction-modification system methylation subunit (EC 2.1.1.72)	1	-	-	-	-	-	-	-	-	-	-	-	-	-	-	-	-	-	-	+	-	-	-	-	-	-	-	-	-	-	-	-	-	-	-	-	-	-	-	-	-	-	-	-	-	-	-	-	-	-	-	-	-	-	-	-	-	-
6759	13139	hypothetical protein	1	-	-	-	-	-	-	-	-	-	-	-	-	-	-	-	-	-	-	+	-	-	-	-	-	-	-	-	-	-	-	-	-	-	-	-	-	-	-	-	-	-	-	-	-	-	-	-	-	-	-	-	-	-	-	-	-	-
6760	13140	hypothetical protein	1	-	-	-	-	-	-	-	-	-	-	-	-	-	-	-	-	-	-	+	-	-	-	-	-	-	-	-	-	-	-	-	-	-	-	-	-	-	-	-	-	-	-	-	-	-	-	-	-	-	-	-	-	-	-	-	-	-
6761	13141	phage protein	1	-	-	-	-	-	-	-	-	-	-	-	-	-	-	-	-	-	-	+	-	-	-	-	-	-	-	-	-	-	-	-	-	-	-	-	-	-	-	-	-	-	-	-	-	-	-	-	-	-	-	-	-	-	-	-	-	-
6762	13142	hypothetical protein	1	-	-	-	-	-	-	-	-	-	-	-	-	-	-	-	-	-	-	+	-	-	-	-	-	-	-	-	-	-	-	-	-	-	-	-	-	-	-	-	-	-	-	-	-	-	-	-	-	-	-	-	-	-	-	-	-	-
6763	13143	hypothetical protein	1	-	-	-	-	-	-	-	-	-	-	-	-	-	-	-	-	-	-	+	-	-	-	-	-	-	-	-	-	-	-	-	-	-	-	-	-	-	-	-	-	-	-	-	-	-	-	-	-	-	-	-	-	-	-	-	-	-
6764	13144	PTS system	1	-	-	-	-	-	-	-	-	-	-	-	-	-	-	-	-	-	-	+	-	-	-	-	-	-	-	-	-	-	-	-	-	-	-	-	-	-	-	-	-	-	-	-	-	-	-	-	-	-	-	-	-	-	-	-	-	-
6765	13145	Mobile element protein	1	-	-	-	-	-	-	-	-	-	-	-	-	-	-	-	-	-	-	+	-	-	-	-	-	-	-	-	-	-	-	-	-	-	-	-	-	-	-	-	-	-	-	-	-	-	-	-	-	-	-	-	-	-	-	-	-	-
6766	13146	Superfamily II DNA/RNA helicases	1	-	-	-	-	-	-	-	-	-	-	-	-	-	-	-	-	-	-	+	-	-	-	-	-	-	-	-	-	-	-	-	-	-	-	-	-	-	-	-	-	-	-	-	-	-	-	-	-	-	-	-	-	-	-	-	-	-
6767	13147	extracellular factor protein	1	-	-	-	-	-	-	-	-	-	-	-	-	-	-	-	-	-	-	+	-	-	-	-	-	-	-	-	-	-	-	-	-	-	-	-	-	-	-	-	-	-	-	-	-	-	-	-	-	-	-	-	-	-	-	-	-	-
6768	13148	Superfamily II DNA/RNA helicases	1	-	-	-	-	-	-	-	-	-	-	-	-	-	-	-	-	-	-	+	-	-	-	-	-	-	-	-	-	-	-	-	-	-	-	-	-	-	-	-	-	-	-	-	-	-	-	-	-	-	-	-	-	-	-	-	-	-
6769	13149	hypothetical protein	1	-	-	-	-	-	-	-	-	-	-	-	-	-	-	-	-	-	-	+	-	-	-	-	-	-	-	-	-	-	-	-	-	-	-	-	-	-	-	-	-	-	-	-	-	-	-	-	-	-	-	-	-	-	-	-	-	-
6770	13150	Cystathionine gamma-synthase (EC 2.5.1.48)	1	-	-	-	-	-	-	-	-	-	-	-	-	-	-	-	-	-	-	+	-	-	-	-	-	-	-	-	-	-	-	-	-	-	-	-	-	-	-	-	-	-	-	-	-	-	-	-	-	-	-	-	-	-	-	-	-	-
6771	13151	Tryptophan synthase beta chain (EC 4.2.1.20)	1	-	-	-	-	-	-	-	-	-	-	-	-	-	-	-	-	-	-	+	-	-	-	-	-	-	-	-	-	-	-	-	-	-	-	-	-	-	-	-	-	-	-	-	-	-	-	-	-	-	-	-	-	-	-	-	-	-
6772	13152	hypothetical protein	1	-	-	-	-	-	-	-	-	-	-	-	-	-	-	-	-	-	-	+	-	-	-	-	-	-	-	-	-	-	-	-	-	-	-	-	-	-	-	-	-	-	-	-	-	-	-	-	-	-	-	-	-	-	-	-	-	-
6773	13153	Tryptophan synthase beta chain (EC 4.2.1.20)	1	-	-	-	-	-	-	-	-	-	-	-	-	-	-	-	-	-	-	+	-	-	-	-	-	-	-	-	-	-	-	-	-	-	-	-	-	-	-	-	-	-	-	-	-	-	-	-	-	-	-	-	-	-	-	-	-	-
6774	13154	hypothetical protein	1	-	-	-	-	-	-	-	-	-	-	-	-	-	-	-	-	-	-	+	-	-	-	-	-	-	-	-	-	-	-	-	-	-	-	-	-	-	-	-	-	-	-	-	-	-	-	-	-	-	-	-	-	-	-	-	-	-
6775	13155	hypothetical protein	1	-	-	-	-	-	-	-	-	-	-	-	-	-	-	-	-	-	-	+	-	-	-	-	-	-	-	-	-	-	-	-	-	-	-	-	-	-	-	-	-	-	-	-	-	-	-	-	-	-	-	-	-	-	-	-	-	-
6776	13156	hypothetical protein	1	-	-	-	-	-	-	-	-	-	-	-	-	-	-	-	-	-	-	+	-	-	-	-	-	-	-	-	-	-	-	-	-	-	-	-	-	-	-	-	-	-	-	-	-	-	-	-	-	-	-	-	-	-	-	-	-	-
6777	13157	hypothetical protein	1	-	-	-	-	-	-	-	-	-	-	-	-	-	-	-	-	-	-	+	-	-	-	-	-	-	-	-	-	-	-	-	-	-	-	-	-	-	-	-	-	-	-	-	-	-	-	-	-	-	-	-	-	-	-	-	-	-
6778	13158	hypothetical protein	1	-	-	-	-	-	-	-	-	-	-	-	-	-	-	-	-	-	-	+	-	-	-	-	-	-	-	-	-	-	-	-	-	-	-	-	-	-	-	-	-	-	-	-	-	-	-	-	-	-	-	-	-	-	-	-	-	-
6779	13159	hypothetical protein	1	-	-	-	-	-	-	-	-	-	-	-	-	-	-	-	-	-	-	+	-	-	-	-	-	-	-	-	-	-	-	-	-	-	-	-	-	-	-	-	-	-	-	-	-	-	-	-	-	-	-	-	-	-	-	-	-	-
6780	13160	DNA-cytosine methyltransferase	1	-	-	-	-	-	-	-	-	-	-	-	-	-	-	-	-	-	-	+	-	-	-	-	-	-	-	-	-	-	-	-	-	-	-	-	-	-	-	-	-	-	-	-	-	-	-	-	-	-	-	-	-	-	-	-	-	-
6781	13161	helicase	1	-	-	-	-	-	-	-	-	-	-	-	-	-	-	-	-	-	-	+	-	-	-	-	-	-	-	-	-	-	-	-	-	-	-	-	-	-	-	-	-	-	-	-	-	-	-	-	-	-	-	-	-	-	-	-	-	-
6782	13162	hypothetical protein	1	-	-	-	-	-	-	-	-	-	-	-	-	-	-	-	-	-	-	+	-	-	-	-	-	-	-	-	-	-	-	-	-	-	-	-	-	-	-	-	-	-	-	-	-	-	-	-	-	-	-	-	-	-	-	-	-	-
6783	13163	hypothetical protein	1	-	-	-	-	-	-	-	-	-	-	-	-	-	-	-	-	-	-	+	-	-	-	-	-	-	-	-	-	-	-	-	-	-	-	-	-	-	-	-	-	-	-	-	-	-	-	-	-	-	-	-	-	-	-	-	-	-
6784	13164	hypothetical protein	1	-	-	-	-	-	-	-	-	-	-	-	-	-	-	-	-	-	-	+	-	-	-	-	-	-	-	-	-	-	-	-	-	-	-	-	-	-	-	-	-	-	-	-	-	-	-	-	-	-	-	-	-	-	-	-	-	-
6785	13165	hypothetical protein	1	-	-	-	-	-	-	-	-	-	-	-	-	-	-	-	-	-	-	+	-	-	-	-	-	-	-	-	-	-	-	-	-	-	-	-	-	-	-	-	-	-	-	-	-	-	-	-	-	-	-	-	-	-	-	-	-	-
6786	13166	hypothetical protein	1	-	-	-	-	-	-	-	-	-	-	-	-	-	-	-	-	-	-	+	-	-	-	-	-	-	-	-	-	-	-	-	-	-	-	-	-	-	-	-	-	-	-	-	-	-	-	-	-	-	-	-	-	-	-	-	-	-
6787	13167	2-dehydropantoate 2-reductase (EC 1.1.1.169)	1	-	-	-	-	-	-	-	-	-	-	-	-	-	-	-	-	-	-	+	-	-	-	-	-	-	-	-	-	-	-	-	-	-	-	-	-	-	-	-	-	-	-	-	-	-	-	-	-	-	-	-	-	-	-	-	-	-
6788	13168	hypothetical protein	1	-	-	-	-	-	-	-	-	-	-	-	-	-	-	-	-	-	-	+	-	-	-	-	-	-	-	-	-	-	-	-	-	-	-	-	-	-	-	-	-	-	-	-	-	-	-	-	-	-	-	-	-	-	-	-	-	-
6789	13169	GIY-YIG catalytic domain containing protein; possible intron encoded endonuclease	1	-	-	-	-	-	-	-	-	-	-	-	-	-	-	-	-	-	-	+	-	-	-	-	-	-	-	-	-	-	-	-	-	-	-	-	-	-	-	-	-	-	-	-	-	-	-	-	-	-	-	-	-	-	-	-	-	-
6790	13170	5'-nucleotidase (EC 3.1.3.5)	1	-	-	-	-	-	-	-	-	-	-	-	-	-	-	-	-	-	-	-	-	-	-	-	-	-	-	-	-	-	-	-	-	-	+	-	-	-	-	-	-	-	-	-	-	-	-	-	-	-	-	-	-	-	-	-	-	-
6791	13171	hypothetical protein	1	-	-	-	-	-	-	-	-	-	-	-	-	-	-	-	-	-	-	-	-	-	-	-	-	-	-	-	-	-	-	-	-	-	+	-	-	-	-	-	-	-	-	-	-	-	-	-	-	-	-	-	-	-	-	-	-	-
6792	13172	hypothetical protein	1	-	-	-	-	-	-	-	-	-	-	-	-	-	-	-	-	-	-	-	-	-	-	-	-	-	-	-	-	-	-	-	-	-	+	-	-	-	-	-	-	-	-	-	-	-	-	-	-	-	-	-	-	-	-	-	-	-
6793	13173	hypothetical protein	1	-	-	-	-	-	-	-	-	-	-	-	-	-	-	-	-	-	-	-	-	-	-	-	-	-	-	-	-	-	-	-	-	-	+	-	-	-	-	-	-	-	-	-	-	-	-	-	-	-	-	-	-	-	-	-	-	-
6794	13174	Phage tail assembly	1	-	-	-	-	-	-	-	-	-	-	-	-	-	-	-	-	-	-	-	-	-	-	-	-	-	-	-	-	-	-	-	-	-	+	-	-	-	-	-	-	-	-	-	-	-	-	-	-	-	-	-	-	-	-	-	-	-
6795	13175	Phosphoglucomutase	1	-	-	-	-	-	-	-	-	-	-	-	-	-	-	-	-	-	-	-	-	-	-	-	-	-	-	-	-	-	-	-	-	-	+	-	-	-	-	-	-	-	-	-	-	-	-	-	-	-	-	-	-	-	-	-	-	-
6796	13176	hypothetical protein	1	-	-	-	-	-	-	-	-	-	-	-	-	-	-	-	-	-	-	-	-	-	-	-	-	-	-	-	-	-	-	-	-	-	+	-	-	-	-	-	-	-	-	-	-	-	-	-	-	-	-	-	-	-	-	-	-	-
6797	13177	inosine-5'-monophosphate dehydrogenase	1	-	-	-	-	-	-	-	-	-	-	-	-	-	-	-	-	-	-	-	-	-	-	-	-	-	-	-	-	-	-	-	-	-	+	-	-	-	-	-	-	-	-	-	-	-	-	-	-	-	-	-	-	-	-	-	-	-
6798	13178	orfG	1	-	-	-	-	-	-	-	-	-	-	-	-	-	-	-	-	-	-	-	-	-	-	-	-	-	-	-	-	+	-	-	-	-	-	-	-	-	-	-	-	-	-	-	-	-	-	-	-	-	-	-	-	-	-	-	-	-
6799	13179	Ascorbate-specific PTS system	1	-	-	-	-	-	-	-	-	-	-	-	-	-	-	-	-	-	-	-	-	-	-	-	-	-	-	-	-	+	-	-	-	-	-	-	-	-	-	-	-	-	-	-	-	-	-	-	-	-	-	-	-	-	-	-	-	-
6800	13180	rRNA small subunit 7-methylguanosine (m7G) methyltransferase GidB	1	-	-	-	-	-	-	-	-	-	-	-	-	-	-	-	-	-	-	-	-	-	-	-	-	-	-	-	-	+	-	-	-	-	-	-	-	-	-	-	-	-	-	-	-	-	-	-	-	-	-	-	-	-	-	-	-	-
6801	13181	unknown	1	-	-	-	-	-	-	-	-	-	-	-	-	-	-	-	-	-	-	-	-	-	-	-	-	-	-	-	-	+	-	-	-	-	-	-	-	-	-	-	-	-	-	-	-	-	-	-	-	-	-	-	-	-	-	-	-	-
6802	13182	hypothetical protein	1	-	-	-	-	-	-	-	-	-	-	-	-	-	-	-	-	-	-	-	-	-	-	-	-	-	-	-	-	+	-	-	-	-	-	-	-	-	-	-	-	-	-	-	-	-	-	-	-	-	-	-	-	-	-	-	-	-
6803	13183	Transcriptional regulator	1	-	-	-	-	-	-	-	-	-	-	-	-	-	-	-	-	-	-	-	-	-	-	-	-	-	-	-	-	+	-	-	-	-	-	-	-	-	-	-	-	-	-	-	-	-	-	-	-	-	-	-	-	-	-	-	-	-
6804	13184	DNA topoisomerase III (EC 5.99.1.2)	1	-	-	-	-	-	-	-	-	-	-	-	-	-	-	-	-	-	-	-	-	-	-	-	-	-	-	-	-	+	-	-	-	-	-	-	-	-	-	-	-	-	-	-	-	-	-	-	-	-	-	-	-	-	-	-	-	-
6805	13185	hypothetical protein	1	-	-	-	-	-	-	-	-	-	-	-	-	-	-	-	-	-	-	-	-	-	-	-	-	-	-	-	-	+	-	-	-	-	-	-	-	-	-	-	-	-	-	-	-	-	-	-	-	-	-	-	-	-	-	-	-	-
6806	13186	Guanine-hypoxanthine permease	1	-	-	-	-	-	-	-	-	-	-	-	-	-	-	-	-	-	-	-	-	-	-	-	-	-	-	-	-	+	-	-	-	-	-	-	-	-	-	-	-	-	-	-	-	-	-	-	-	-	-	-	-	-	-	-	-	-
6807	13187	hypothetical protein	1	-	-	-	-	-	-	-	-	-	-	-	-	-	-	-	-	-	-	-	-	-	-	-	-	-	-	-	-	+	-	-	-	-	-	-	-	-	-	-	-	-	-	-	-	-	-	-	-	-	-	-	-	-	-	-	-	-
6808	13188	hypothetical protein	1	-	-	-	-	-	-	+	-	-	-	-	-	-	-	-	-	-	-	-	-	-	-	-	-	-	-	-	-	-	-	-	-	-	-	-	-	-	-	-	-	-	-	-	-	-	-	-	-	-	-	-	-	-	-	-	-	-
6809	13189	nisin biosynthesis sensor protein NisK	1	-	-	-	-	-	-	+	-	-	-	-	-	-	-	-	-	-	-	-	-	-	-	-	-	-	-	-	-	-	-	-	-	-	-	-	-	-	-	-	-	-	-	-	-	-	-	-	-	-	-	-	-	-	-	-	-	-
6810	13190	hypothetical protein	1	-	-	-	-	-	-	+	-	-	-	-	-	-	-	-	-	-	-	-	-	-	-	-	-	-	-	-	-	-	-	-	-	-	-	-	-	-	-	-	-	-	-	-	-	-	-	-	-	-	-	-	-	-	-	-	-	-
6811	13191	CopS protein	1	-	-	-	-	-	-	+	-	-	-	-	-	-	-	-	-	-	-	-	-	-	-	-	-	-	-	-	-	-	-	-	-	-	-	-	-	-	-	-	-	-	-	-	-	-	-	-	-	-	-	-	-	-	-	-	-	-
6812	13192	Phage tail length tape-measure protein	1	-	-	-	-	-	-	-	-	-	-	-	-	-	-	-	-	-	-	-	-	-	-	-	-	-	-	-	-	-	-	-	-	-	-	+	-	-	-	-	-	-	-	-	-	-	-	-	-	-	-	-	-	-	-	-	-	-
6813	13193	hypothetical protein	1	-	-	-	-	-	-	-	-	-	-	-	-	-	-	-	-	-	-	-	-	-	-	-	-	-	-	-	-	-	-	-	-	-	-	+	-	-	-	-	-	-	-	-	-	-	-	-	-	-	-	-	-	-	-	-	-	-
6814	13194	hypothetical protein	1	-	-	-	-	-	-	-	-	-	-	-	-	-	-	-	-	-	-	-	-	-	-	-	-	-	-	-	-	-	-	-	-	-	-	+	-	-	-	-	-	-	-	-	-	-	-	-	-	-	-	-	-	-	-	-	-	-
6815	13195	hypothetical protein	1	-	-	-	-	-	-	-	-	-	-	-	-	-	-	-	-	-	-	-	-	-	-	-	-	-	-	-	-	-	-	-	-	-	-	+	-	-	-	-	-	-	-	-	-	-	-	-	-	-	-	-	-	-	-	-	-	-
6816	13196	hypothetical protein	1	-	-	-	-	-	-	-	-	-	-	-	-	-	-	-	-	-	-	-	-	-	-	-	-	-	-	-	-	-	-	-	-	-	-	+	-	-	-	-	-	-	-	-	-	-	-	-	-	-	-	-	-	-	-	-	-	-
6817	13197	putative DNA-binding phage protein	1	-	-	-	-	-	-	-	-	-	-	-	-	-	-	-	-	-	-	-	-	-	-	-	-	-	-	-	-	-	-	-	-	-	-	+	-	-	-	-	-	-	-	-	-	-	-	-	-	-	-	-	-	-	-	-	-	-
6818	13198	hypothetical protein	1	-	-	-	-	-	-	-	-	-	-	-	-	-	-	-	-	-	-	-	-	-	-	-	-	-	-	-	-	-	-	-	-	-	-	+	-	-	-	-	-	-	-	-	-	-	-	-	-	-	-	-	-	-	-	-	-	-
6819	13199	hypothetical protein	1	-	-	-	-	-	-	-	-	-	-	-	-	-	-	-	-	-	-	-	-	-	-	-	-	-	-	-	-	-	-	-	-	-	-	+	-	-	-	-	-	-	-	-	-	-	-	-	-	-	-	-	-	-	-	-	-	-
6820	13200	hypothetical protein	1	-	-	-	-	-	-	-	-	-	-	-	-	-	-	-	-	-	-	-	-	-	-	-	-	-	-	-	-	-	-	-	-	-	-	+	-	-	-	-	-	-	-	-	-	-	-	-	-	-	-	-	-	-	-	-	-	-
6821	13201	hypothetical protein	1	-	-	-	-	-	-	-	-	-	-	-	-	-	-	-	-	-	-	-	-	-	-	-	-	-	-	-	-	-	-	-	-	-	-	+	-	-	-	-	-	-	-	-	-	-	-	-	-	-	-	-	-	-	-	-	-	-
6822	13202	hypothetical protein	1	-	-	-	-	-	-	-	-	-	-	-	-	-	-	-	-	-	-	-	-	-	-	-	-	-	-	-	-	-	-	-	-	-	-	+	-	-	-	-	-	-	-	-	-	-	-	-	-	-	-	-	-	-	-	-	-	-
6823	13203	Beta-lactamase class C and other penicillin binding proteins	1	-	-	-	-	-	-	-	-	-	-	-	-	-	-	-	-	-	-	-	-	-	-	-	-	-	-	-	-	-	-	-	-	-	-	+	-	-	-	-	-	-	-	-	-	-	-	-	-	-	-	-	-	-	-	-	-	-
6824	13204	hypothetical protein	1	-	-	-	-	-	-	-	-	-	-	-	-	-	-	-	-	-	-	-	-	-	-	-	-	-	-	-	-	-	-	-	-	-	-	+	-	-	-	-	-	-	-	-	-	-	-	-	-	-	-	-	-	-	-	-	-	-
6825	13205	Phage major tail protein	1	-	-	-	-	-	-	-	-	-	-	-	-	-	-	-	-	-	-	-	-	-	-	-	-	-	-	-	-	-	-	-	-	-	-	+	-	-	-	-	-	-	-	-	-	-	-	-	-	-	-	-	-	-	-	-	-	-
6826	13206	Phage tail assembly	1	-	-	-	-	-	-	-	-	-	-	-	-	-	-	-	-	-	-	-	-	-	-	-	-	-	-	-	-	-	-	-	-	-	-	+	-	-	-	-	-	-	-	-	-	-	-	-	-	-	-	-	-	-	-	-	-	-
6827	13207	hypothetical protein	1	-	-	-	-	-	-	-	-	-	-	-	-	-	-	-	-	-	-	-	-	-	-	-	-	-	-	-	-	-	-	-	-	-	-	+	-	-	-	-	-	-	-	-	-	-	-	-	-	-	-	-	-	-	-	-	-	-
6828	13208	COG2910: Putative NADH-flavin reductase	1	-	-	-	-	-	-	-	-	-	-	-	-	-	-	-	-	-	-	-	-	-	-	-	-	-	-	-	-	-	-	-	-	-	-	+	-	-	-	-	-	-	-	-	-	-	-	-	-	-	-	-	-	-	-	-	-	-
6829	13209	Epsilon antitoxin	1	-	-	-	-	-	-	-	-	-	-	-	-	-	-	-	-	-	-	-	-	-	-	-	-	-	-	-	-	-	-	-	-	-	-	+	-	-	-	-	-	-	-	-	-	-	-	-	-	-	-	-	-	-	-	-	-	-
6830	13210	hypothetical protein	1	-	-	-	-	-	-	-	-	-	-	-	-	-	-	-	-	-	-	-	-	-	-	-	-	-	-	-	-	-	-	-	-	-	-	+	-	-	-	-	-	-	-	-	-	-	-	-	-	-	-	-	-	-	-	-	-	-
6831	13211	hypothetical protein	1	-	-	-	-	-	-	-	-	-	-	-	-	-	-	-	-	-	-	-	-	-	-	-	-	-	-	-	-	-	-	-	-	-	-	+	-	-	-	-	-	-	-	-	-	-	-	-	-	-	-	-	-	-	-	-	-	-
6832	13212	hypothetical protein	1	-	-	-	-	-	-	-	-	-	-	-	-	-	-	-	-	-	-	-	-	-	-	-	-	-	-	-	-	-	-	-	-	-	-	+	-	-	-	-	-	-	-	-	-	-	-	-	-	-	-	-	-	-	-	-	-	-
6833	13213	hypothetical protein	1	-	-	-	-	-	-	-	-	-	-	-	-	-	-	-	-	-	-	-	-	-	-	-	-	-	-	-	-	-	-	-	-	-	-	+	-	-	-	-	-	-	-	-	-	-	-	-	-	-	-	-	-	-	-	-	-	-
6834	13214	hypothetical protein	1	-	-	-	-	-	-	-	-	-	-	-	-	-	-	-	-	-	-	-	-	-	-	-	-	-	-	-	-	-	-	-	-	-	-	+	-	-	-	-	-	-	-	-	-	-	-	-	-	-	-	-	-	-	-	-	-	-
6835	13215	hypothetical protein	1	-	-	-	-	-	-	-	-	-	-	-	-	-	-	-	-	-	-	-	-	-	-	-	-	-	-	-	-	-	-	-	-	-	-	+	-	-	-	-	-	-	-	-	-	-	-	-	-	-	-	-	-	-	-	-	-	-
6836	13216	Beta-glucosidase (EC 3.2.1.21)	1	-	-	-	-	-	-	-	-	-	-	-	-	-	-	-	-	-	-	-	-	-	-	-	-	-	-	-	-	-	-	-	-	-	-	+	-	-	-	-	-	-	-	-	-	-	-	-	-	-	-	-	-	-	-	-	-	-
6837	13217	hypothetical protein	1	-	-	-	-	-	-	-	-	-	-	-	-	-	-	-	-	-	-	-	-	-	-	-	-	-	-	-	-	-	-	-	-	-	-	+	-	-	-	-	-	-	-	-	-	-	-	-	-	-	-	-	-	-	-	-	-	-
6838	13218	hypothetical protein	1	-	-	-	-	-	-	-	-	-	-	-	-	-	-	-	-	-	-	-	-	-	-	-	-	-	-	-	-	-	-	-	-	-	-	+	-	-	-	-	-	-	-	-	-	-	-	-	-	-	-	-	-	-	-	-	-	-
6839	13219	hypothetical protein	1	-	-	-	-	-	-	-	-	-	-	-	-	-	-	-	-	-	-	-	-	-	-	-	-	-	-	-	+	-	-	-	-	-	-	-	-	-	-	-	-	-	-	-	-	-	-	-	-	-	-	-	-	-	-	-	-	-
6840	13220	hypothetical protein	1	-	-	-	-	-	-	-	-	-	-	-	-	-	-	-	-	-	-	-	-	-	-	-	-	-	-	-	+	-	-	-	-	-	-	-	-	-	-	-	-	-	-	-	-	-	-	-	-	-	-	-	-	-	-	-	-	-
6841	13221	hypothetical protein	1	-	-	-	-	-	-	-	-	-	-	-	-	-	-	-	-	-	-	-	-	-	-	-	-	-	-	-	+	-	-	-	-	-	-	-	-	-	-	-	-	-	-	-	-	-	-	-	-	-	-	-	-	-	-	-	-	-
6842	13222	hypothetical protein	1	-	-	-	-	-	-	-	-	-	-	-	-	-	-	-	-	-	-	-	-	-	-	-	-	-	-	-	+	-	-	-	-	-	-	-	-	-	-	-	-	-	-	-	-	-	-	-	-	-	-	-	-	-	-	-	-	-
6843	13223	Predicted transcriptional regulator	1	-	-	-	-	-	-	-	-	-	-	-	-	-	-	-	-	-	-	-	-	-	-	-	-	-	-	-	+	-	-	-	-	-	-	-	-	-	-	-	-	-	-	-	-	-	-	-	-	-	-	-	-	-	-	-	-	-
6844	13224	hypothetical protein	1	-	-	-	-	-	-	-	-	-	-	-	-	-	-	-	-	-	-	-	-	-	-	-	-	-	-	-	+	-	-	-	-	-	-	-	-	-	-	-	-	-	-	-	-	-	-	-	-	-	-	-	-	-	-	-	-	-
6845	13225	conserved domain protein	1	-	-	-	-	-	-	-	-	-	-	-	-	-	-	-	-	-	-	-	-	-	-	-	-	-	-	-	+	-	-	-	-	-	-	-	-	-	-	-	-	-	-	-	-	-	-	-	-	-	-	-	-	-	-	-	-	-
6846	13226	PTS system	1	-	-	-	-	-	-	-	-	-	-	-	-	-	-	-	-	-	-	-	-	-	-	-	-	-	-	-	+	-	-	-	-	-	-	-	-	-	-	-	-	-	-	-	-	-	-	-	-	-	-	-	-	-	-	-	-	-
6847	13227	6-phospho-beta-glucosidase (EC 3.2.1.86)	1	-	-	-	-	-	-	-	-	-	-	-	-	-	-	-	-	-	-	-	-	-	-	-	-	-	-	-	+	-	-	-	-	-	-	-	-	-	-	-	-	-	-	-	-	-	-	-	-	-	-	-	-	-	-	-	-	-
6848	13228	Predicted aminoglycoside phosphotransferase	1	-	-	-	-	-	-	-	-	-	-	-	-	-	-	-	-	-	-	-	-	-	-	-	-	-	-	-	+	-	-	-	-	-	-	-	-	-	-	-	-	-	-	-	-	-	-	-	-	-	-	-	-	-	-	-	-	-
6849	13229	hypothetical protein	1	-	-	-	-	-	-	-	-	-	-	-	-	-	-	-	-	-	-	-	-	-	-	-	-	-	-	-	+	-	-	-	-	-	-	-	-	-	-	-	-	-	-	-	-	-	-	-	-	-	-	-	-	-	-	-	-	-
6850	13230	hypothetical protein	1	-	-	-	-	-	-	-	-	-	-	-	-	-	-	-	-	-	-	-	-	+	-	-	-	-	-	-	-	-	-	-	-	-	-	-	-	-	-	-	-	-	-	-	-	-	-	-	-	-	-	-	-	-	-	-	-	-
6851	13231	hypothetical protein	1	-	-	-	-	-	-	-	-	-	-	-	-	-	-	-	-	-	-	-	-	+	-	-	-	-	-	-	-	-	-	-	-	-	-	-	-	-	-	-	-	-	-	-	-	-	-	-	-	-	-	-	-	-	-	-	-	-
6852	13232	hypothetical protein	1	-	-	-	-	-	-	-	-	-	-	-	-	-	-	-	-	-	-	-	-	+	-	-	-	-	-	-	-	-	-	-	-	-	-	-	-	-	-	-	-	-	-	-	-	-	-	-	-	-	-	-	-	-	-	-	-	-
6853	13233	PTS system	1	-	-	-	-	-	-	-	-	-	-	-	-	-	+	-	-	-	-	-	-	-	-	-	-	-	-	-	-	-	-	-	-	-	-	-	-	-	-	-	-	-	-	-	-	-	-	-	-	-	-	-	-	-	-	-	-	-
6854	13234	hypothetical protein	1	-	-	-	-	-	-	-	-	-	-	-	-	-	-	-	-	-	-	-	-	-	-	-	-	-	-	-	-	-	-	+	-	-	-	-	-	-	-	-	-	-	-	-	-	-	-	-	-	-	-	-	-	-	-	-	-	-
6855	13235	hypothetical protein	1	-	-	-	-	-	-	-	-	-	-	-	-	-	-	-	-	-	-	-	-	-	-	-	-	-	-	-	-	-	-	+	-	-	-	-	-	-	-	-	-	-	-	-	-	-	-	-	-	-	-	-	-	-	-	-	-	-
6856	13236	hypothetical protein	1	-	-	-	-	-	-	-	-	-	-	-	-	-	-	-	-	-	-	-	-	-	+	-	-	-	-	-	-	-	-	-	-	-	-	-	-	-	-	-	-	-	-	-	-	-	-	-	-	-	-	-	-	-	-	-	-	-
6857	13237	hypothetical protein	1	-	-	-	-	-	-	-	-	-	-	-	-	-	-	-	-	-	-	-	-	-	+	-	-	-	-	-	-	-	-	-	-	-	-	-	-	-	-	-	-	-	-	-	-	-	-	-	-	-	-	-	-	-	-	-	-	-
6858	13238	hypothetical protein	1	-	-	-	-	-	-	-	-	-	-	-	-	-	-	-	-	-	-	-	-	-	+	-	-	-	-	-	-	-	-	-	-	-	-	-	-	-	-	-	-	-	-	-	-	-	-	-	-	-	-	-	-	-	-	-	-	-
6859	13239	Pleiotropic regulator of exopolysaccharide synthesis	1	-	-	-	-	-	-	-	-	-	-	-	-	-	-	-	-	-	-	-	-	-	-	-	-	-	-	+	-	-	-	-	-	-	-	-	-	-	-	-	-	-	-	-	-	-	-	-	-	-	-	-	-	-	-	-	-	-
6860	13240	Collagen adhesion protein	1	-	-	-	-	-	-	-	-	-	-	-	-	-	-	-	-	-	-	-	-	-	-	-	-	-	-	+	-	-	-	-	-	-	-	-	-	-	-	-	-	-	-	-	-	-	-	-	-	-	-	-	-	-	-	-	-	-
6861	13241	hypothetical protein	1	-	-	-	-	-	-	-	-	-	-	-	-	-	-	-	-	-	-	-	-	-	-	-	-	-	-	+	-	-	-	-	-	-	-	-	-	-	-	-	-	-	-	-	-	-	-	-	-	-	-	-	-	-	-	-	-	-
6862	13242	hypothetical protein	1	-	-	-	-	-	-	-	-	-	-	-	-	-	-	-	-	-	-	-	-	-	-	-	-	-	-	-	-	-	-	-	+	-	-	-	-	-	-	-	-	-	-	-	-	-	-	-	-	-	-	-	-	-	-	-	-	-
6863	13243	unknown	1	-	-	-	-	-	-	-	+	-	-	-	-	-	-	-	-	-	-	-	-	-	-	-	-	-	-	-	-	-	-	-	-	-	-	-	-	-	-	-	-	-	-	-	-	-	-	-	-	-	-	-	-	-	-	-	-	-
6864	13244	hypothetical protein	1	-	-	-	-	-	-	-	+	-	-	-	-	-	-	-	-	-	-	-	-	-	-	-	-	-	-	-	-	-	-	-	-	-	-	-	-	-	-	-	-	-	-	-	-	-	-	-	-	-	-	-	-	-	-	-	-	-
6865	13245	DNA repair protein RadC	1	-	-	-	-	-	-	-	-	-	-	-	-	-	-	-	-	-	-	-	-	-	-	-	-	-	-	-	-	-	+	-	-	-	-	-	-	-	-	-	-	-	-	-	-	-	-	-	-	-	-	-	-	-	-	-	-	-
6866	13246	hypothetical protein	1	-	-	-	-	-	-	-	-	-	-	-	-	-	-	-	-	-	-	-	-	-	-	-	-	-	-	-	-	-	+	-	-	-	-	-	-	-	-	-	-	-	-	-	-	-	-	-	-	-	-	-	-	-	-	-	-	-
6867	20963	hypothetical protein	1	-	-	-	-	-	-	-	-	-	-	-	-	-	-	-	-	-	-	-	+	-	-	-	-	-	-	-	-	-	-	-	-	-	-	-	-	-	-	-	-	-	-	-	-	-	-	-	-	-	-	-	-	-	-	-	-	-
6868	20964	hypothetical protein	1	-	-	-	-	-	-	-	-	-	-	-	-	-	-	-	-	-	-	-	+	-	-	-	-	-	-	-	-	-	-	-	-	-	-	-	-	-	-	-	-	-	-	-	-	-	-	-	-	-	-	-	-	-	-	-	-	-
6869	20965	CDP-diacylglycerol--glycerol-3-phosphate 3-phosphatidyltransferase (EC 2.7.8.5)	1	-	-	-	-	-	-	-	-	-	-	-	-	-	-	-	-	-	-	-	+	-	-	-	-	-	-	-	-	-	-	-	-	-	-	-	-	-	-	-	-	-	-	-	-	-	-	-	-	-	-	-	-	-	-	-	-	-
6870	20966	Response regulator of the LytR/AlgR family	1	-	-	-	-	-	-	-	-	-	-	-	-	-	-	-	-	-	-	-	+	-	-	-	-	-	-	-	-	-	-	-	-	-	-	-	-	-	-	-	-	-	-	-	-	-	-	-	-	-	-	-	-	-	-	-	-	-
6871	20967	ABC transporter, ATP-binding/permease protein	1	-	-	-	-	-	-	-	-	-	-	-	-	-	-	-	-	-	-	-	+	-	-	-	-	-	-	-	-	-	-	-	-	-	-	-	-	-	-	-	-	-	-	-	-	-	-	-	-	-	-	-	-	-	-	-	-	-
6872	20968	hypothetical protein	1	-	-	-	-	-	-	-	-	-	-	-	-	-	-	-	-	-	-	-	+	-	-	-	-	-	-	-	-	-	-	-	-	-	-	-	-	-	-	-	-	-	-	-	-	-	-	-	-	-	-	-	-	-	-	-	-	-
6873	20969	hypothetical protein	1	-	-	-	-	-	-	-	-	-	-	-	-	-	-	-	-	-	-	-	+	-	-	-	-	-	-	-	-	-	-	-	-	-	-	-	-	-	-	-	-	-	-	-	-	-	-	-	-	-	-	-	-	-	-	-	-	-
6874	20970	hypothetical protein	1	-	-	-	-	-	-	-	-	-	-	-	-	-	-	-	-	-	-	-	+	-	-	-	-	-	-	-	-	-	-	-	-	-	-	-	-	-	-	-	-	-	-	-	-	-	-	-	-	-	-	-	-	-	-	-	-	-
6875	20971	hypothetical protein	1	-	-	-	-	-	-	-	-	-	-	-	-	-	-	-	-	-	-	-	+	-	-	-	-	-	-	-	-	-	-	-	-	-	-	-	-	-	-	-	-	-	-	-	-	-	-	-	-	-	-	-	-	-	-	-	-	-
6876	20972	hypothetical protein	1	-	-	-	-	-	-	-	-	-	-	-	-	-	-	-	-	-	-	-	+	-	-	-	-	-	-	-	-	-	-	-	-	-	-	-	-	-	-	-	-	-	-	-	-	-	-	-	-	-	-	-	-	-	-	-	-	-
6877	20973	hypothetical protein	1	-	-	-	-	-	-	-	-	-	-	-	-	-	-	-	-	-	-	-	+	-	-	-	-	-	-	-	-	-	-	-	-	-	-	-	-	-	-	-	-	-	-	-	-	-	-	-	-	-	-	-	-	-	-	-	-	-
6878	20974	LSU ribosomal protein L23p (L23Ae)	1	-	-	-	-	-	-	-	-	-	-	-	-	-	-	-	-	-	-	-	+	-	-	-	-	-	-	-	-	-	-	-	-	-	-	-	-	-	-	-	-	-	-	-	-	-	-	-	-	-	-	-	-	-	-	-	-	-
6879	20975	2',3'-cyclic-nucleotide 2'-phosphodiesterase (EC 3.1.4.16)	1	-	-	-	-	-	-	-	-	-	-	-	-	-	-	-	-	-	-	-	+	-	-	-	-	-	-	-	-	-	-	-	-	-	-	-	-	-	-	-	-	-	-	-	-	-	-	-	-	-	-	-	-	-	-	-	-	-
6880	20976	hypothetical protein	1	-	-	-	-	-	-	-	-	-	-	-	-	-	-	-	-	-	-	-	+	-	-	-	-	-	-	-	-	-	-	-	-	-	-	-	-	-	-	-	-	-	-	-	-	-	-	-	-	-	-	-	-	-	-	-	-	-
6881	20977	Integrase	1	-	-	-	-	-	-	-	-	-	-	-	-	-	-	-	-	-	-	-	+	-	-	-	-	-	-	-	-	-	-	-	-	-	-	-	-	-	-	-	-	-	-	-	-	-	-	-	-	-	-	-	-	-	-	-	-	-
6882	20978	hypothetical protein	1	-	-	-	-	-	-	-	-	-	-	-	-	-	-	-	-	-	-	-	+	-	-	-	-	-	-	-	-	-	-	-	-	-	-	-	-	-	-	-	-	-	-	-	-	-	-	-	-	-	-	-	-	-	-	-	-	-
6883	20979	hypothetical protein	1	-	-	-	-	-	-	-	-	-	-	-	-	-	-	-	-	-	-	-	+	-	-	-	-	-	-	-	-	-	-	-	-	-	-	-	-	-	-	-	-	-	-	-	-	-	-	-	-	-	-	-	-	-	-	-	-	-
6884	20980	hypothetical protein	1	-	-	-	-	-	-	-	-	-	-	-	-	-	-	-	-	-	-	-	+	-	-	-	-	-	-	-	-	-	-	-	-	-	-	-	-	-	-	-	-	-	-	-	-	-	-	-	-	-	-	-	-	-	-	-	-	-
6885	20981	Transcription antitermination protein NusG	1	-	-	-	-	-	-	-	-	-	-	-	-	-	-	-	-	-	-	-	+	-	-	-	-	-	-	-	-	-	-	-	-	-	-	-	-	-	-	-	-	-	-	-	-	-	-	-	-	-	-	-	-	-	-	-	-	-
6886	20982	Mobile element protein	1	-	-	-	-	-	-	-	-	-	-	-	-	-	-	-	-	-	-	-	+	-	-	-	-	-	-	-	-	-	-	-	-	-	-	-	-	-	-	-	-	-	-	-	-	-	-	-	-	-	-	-	-	-	-	-	-	-
6887	20983	hypothetical protein	1	-	-	-	-	-	-	-	-	-	-	-	-	-	-	-	-	-	-	-	+	-	-	-	-	-	-	-	-	-	-	-	-	-	-	-	-	-	-	-	-	-	-	-	-	-	-	-	-	-	-	-	-	-	-	-	-	-
6888	20984	hypothetical protein	1	-	-	-	-	-	-	-	-	-	-	-	-	-	-	-	-	-	-	-	+	-	-	-	-	-	-	-	-	-	-	-	-	-	-	-	-	-	-	-	-	-	-	-	-	-	-	-	-	-	-	-	-	-	-	-	-	-
6889	20985	hypothetical protein	1	-	-	-	-	-	-	-	-	-	-	-	-	-	-	-	-	-	-	-	+	-	-	-	-	-	-	-	-	-	-	-	-	-	-	-	-	-	-	-	-	-	-	-	-	-	-	-	-	-	-	-	-	-	-	-	-	-
6890	20986	4-alpha-glucanotransferase (amylomaltase) (EC 2.4.1.25)	1	-	-	-	-	-	-	-	-	-	-	-	-	-	-	-	-	-	-	-	+	-	-	-	-	-	-	-	-	-	-	-	-	-	-	-	-	-	-	-	-	-	-	-	-	-	-	-	-	-	-	-	-	-	-	-	-	-
6891	20987	hypothetical protein	1	-	-	-	-	-	-	-	-	-	-	-	-	-	-	-	-	-	-	-	+	-	-	-	-	-	-	-	-	-	-	-	-	-	-	-	-	-	-	-	-	-	-	-	-	-	-	-	-	-	-	-	-	-	-	-	-	-
6892	20988	hypothetical protein	1	-	-	-	-	-	-	-	-	-	-	-	-	-	-	-	-	-	-	-	+	-	-	-	-	-	-	-	-	-	-	-	-	-	-	-	-	-	-	-	-	-	-	-	-	-	-	-	-	-	-	-	-	-	-	-	-	-
6893	20989	hypothetical protein	1	-	-	-	-	-	-	-	-	-	-	+	-	-	-	-	-	-	-	-	-	-	-	-	-	-	-	-	-	-	-	-	-	-	-	-	-	-	-	-	-	-	-	-	-	-	-	-	-	-	-	-	-	-	-	-	-	-
6894	20990	Chloramphenicol acetyltransferase (EC 2.3.1.28)	1	-	-	-	-	-	-	-	-	-	-	+	-	-	-	-	-	-	-	-	-	-	-	-	-	-	-	-	-	-	-	-	-	-	-	-	-	-	-	-	-	-	-	-	-	-	-	-	-	-	-	-	-	-	-	-	-	-
6895	20991	hypothetical protein	1	-	-	-	-	-	-	-	-	-	-	+	-	-	-	-	-	-	-	-	-	-	-	-	-	-	-	-	-	-	-	-	-	-	-	-	-	-	-	-	-	-	-	-	-	-	-	-	-	-	-	-	-	-	-	-	-	-
6896	20992	hypothetical protein	1	-	-	-	-	-	-	-	-	-	-	+	-	-	-	-	-	-	-	-	-	-	-	-	-	-	-	-	-	-	-	-	-	-	-	-	-	-	-	-	-	-	-	-	-	-	-	-	-	-	-	-	-	-	-	-	-	-
6897	20993	membrane protein, putative	1	-	-	-	-	-	-	-	-	-	-	+	-	-	-	-	-	-	-	-	-	-	-	-	-	-	-	-	-	-	-	-	-	-	-	-	-	-	-	-	-	-	-	-	-	-	-	-	-	-	-	-	-	-	-	-	-	-
6898	20994	Zn-finger DNA-binding domain	1	-	-	-	-	-	-	-	-	-	-	+	-	-	-	-	-	-	-	-	-	-	-	-	-	-	-	-	-	-	-	-	-	-	-	-	-	-	-	-	-	-	-	-	-	-	-	-	-	-	-	-	-	-	-	-	-	-
6899	20995	hypothetical protein	1	-	-	-	-	-	-	-	-	-	-	+	-	-	-	-	-	-	-	-	-	-	-	-	-	-	-	-	-	-	-	-	-	-	-	-	-	-	-	-	-	-	-	-	-	-	-	-	-	-	-	-	-	-	-	-	-	-
6900	20996	Dihydrofolate reductase (EC 1.5.1.3)	1	-	-	-	-	-	-	-	-	-	-	+	-	-	-	-	-	-	-	-	-	-	-	-	-	-	-	-	-	-	-	-	-	-	-	-	-	-	-	-	-	-	-	-	-	-	-	-	-	-	-	-	-	-	-	-	-	-
6901	20997	hypothetical protein	1	-	-	-	-	-	-	-	-	-	-	+	-	-	-	-	-	-	-	-	-	-	-	-	-	-	-	-	-	-	-	-	-	-	-	-	-	-	-	-	-	-	-	-	-	-	-	-	-	-	-	-	-	-	-	-	-	-
6902	20998	D-alanine--D-alanine ligase (EC 6.3.2.4)	1	-	-	-	-	-	-	-	-	-	-	+	-	-	-	-	-	-	-	-	-	-	-	-	-	-	-	-	-	-	-	-	-	-	-	-	-	-	-	-	-	-	-	-	-	-	-	-	-	-	-	-	-	-	-	-	-	-
6903	20999	hypothetical protein	1	-	-	+	-	-	-	-	-	-	-	-	-	-	-	-	-	-	-	-	-	-	-	-	-	-	-	-	-	-	-	-	-	-	-	-	-	-	-	-	-	-	-	-	-	-	-	-	-	-	-	-	-	-	-	-	-	-
6904	21000	FIG137478: Hypothetical protein	1	-	-	-	-	-	-	-	-	-	-	-	+	-	-	-	-	-	-	-	-	-	-	-	-	-	-	-	-	-	-	-	-	-	-	-	-	-	-	-	-	-	-	-	-	-	-	-	-	-	-	-	-	-	-	-	-	-
6905	21001	Phosphotransferase system IIC component, glucose/maltose/N-acetylglucosamine-specific	1	-	-	-	-	-	-	-	-	-	-	-	+	-	-	-	-	-	-	-	-	-	-	-	-	-	-	-	-	-	-	-	-	-	-	-	-	-	-	-	-	-	-	-	-	-	-	-	-	-	-	-	-	-	-	-	-	-
6906	21002	hypothetical protein	1	-	-	-	-	-	-	-	-	-	-	-	+	-	-	-	-	-	-	-	-	-	-	-	-	-	-	-	-	-	-	-	-	-	-	-	-	-	-	-	-	-	-	-	-	-	-	-	-	-	-	-	-	-	-	-	-	-
6907	21003	ATP-dependent Clp protease, ATP-binding subunit ClpE	1	-	-	-	-	-	-	-	-	-	-	-	+	-	-	-	-	-	-	-	-	-	-	-	-	-	-	-	-	-	-	-	-	-	-	-	-	-	-	-	-	-	-	-	-	-	-	-	-	-	-	-	-	-	-	-	-	-
6908	21004	hypothetical protein	1	-	-	-	-	-	-	-	-	-	+	-	-	-	-	-	-	-	-	-	-	-	-	-	-	-	-	-	-	-	-	-	-	-	-	-	-	-	-	-	-	-	-	-	-	-	-	-	-	-	-	-	-	-	-	-	-	-
6909	21005	Acetylornithine deacetylase/Succinyl-diaminopimelate desuccinylase and related deacylases	1	-	-	-	-	-	-	-	-	-	+	-	-	-	-	-	-	-	-	-	-	-	-	-	-	-	-	-	-	-	-	-	-	-	-	-	-	-	-	-	-	-	-	-	-	-	-	-	-	-	-	-	-	-	-	-	-	-
6910	21006	hypothetical protein	1	-	-	-	-	-	-	-	-	-	+	-	-	-	-	-	-	-	-	-	-	-	-	-	-	-	-	-	-	-	-	-	-	-	-	-	-	-	-	-	-	-	-	-	-	-	-	-	-	-	-	-	-	-	-	-	-	-
6911	21007	hypothetical protein	1	-	-	-	-	-	-	-	-	-	+	-	-	-	-	-	-	-	-	-	-	-	-	-	-	-	-	-	-	-	-	-	-	-	-	-	-	-	-	-	-	-	-	-	-	-	-	-	-	-	-	-	-	-	-	-	-	-
6912	21008	Acetylornithine deacetylase/Succinyl-diaminopimelate desuccinylase and related deacylases	1	-	-	-	-	-	-	-	-	-	+	-	-	-	-	-	-	-	-	-	-	-	-	-	-	-	-	-	-	-	-	-	-	-	-	-	-	-	-	-	-	-	-	-	-	-	-	-	-	-	-	-	-	-	-	-	-	-
6913	21009	hypothetical protein	1	-	-	-	-	-	-	-	-	-	+	-	-	-	-	-	-	-	-	-	-	-	-	-	-	-	-	-	-	-	-	-	-	-	-	-	-	-	-	-	-	-	-	-	-	-	-	-	-	-	-	-	-	-	-	-	-	-
6914	21010	hypothetical protein	1	-	-	-	-	-	-	-	-	-	+	-	-	-	-	-	-	-	-	-	-	-	-	-	-	-	-	-	-	-	-	-	-	-	-	-	-	-	-	-	-	-	-	-	-	-	-	-	-	-	-	-	-	-	-	-	-	-
6915	21011	hypothetical protein	1	-	-	-	-	-	-	-	-	-	+	-	-	-	-	-	-	-	-	-	-	-	-	-	-	-	-	-	-	-	-	-	-	-	-	-	-	-	-	-	-	-	-	-	-	-	-	-	-	-	-	-	-	-	-	-	-	-
6916	21012	Permease of the major facilitator superfamily	1	-	-	-	-	-	-	-	-	-	+	-	-	-	-	-	-	-	-	-	-	-	-	-	-	-	-	-	-	-	-	-	-	-	-	-	-	-	-	-	-	-	-	-	-	-	-	-	-	-	-	-	-	-	-	-	-	-
6917	21013	Type I restriction-modification system, DNA-methyltransferase subunit M (EC 2.1.1.72)	1	-	-	-	-	-	-	-	-	-	+	-	-	-	-	-	-	-	-	-	-	-	-	-	-	-	-	-	-	-	-	-	-	-	-	-	-	-	-	-	-	-	-	-	-	-	-	-	-	-	-	-	-	-	-	-	-	-

Table S5. Precence/absence of each HG among 57 *S. suis* strains

				1																13			25					28						104		94			76			53/54		Divergent *S. suis*																
Index[a]	ClusterID	Gene products	Members	98HAH33	05ZYH33	SC84	A7	SS12	P1/7	DAT273	NIAH11434	JS14	GZ1	BM407	S735	13730	MNCM07	NCTC 10446	14636	NCTC 10237	ST1	2651	89-1591	MNCM04	MNCM25	8074	D9	4961	ST3	MNCM43	DAT292	DAT260	NIAH11435	MNCM21	MNCM50	89-2479	DAT234	DAT290	93A	42A	NT77	6407	11538	8830	12814	92-1400	89-590	2726	2524	89-3576-3	4417	D12	10581	89-5259	88-5299A	R61	92-1191	14A	92-4172	22083
6918	21014	hypothetical protein	1	-	-	-	-	-	-	-	-	-	+	-	-	-	-	-	-	-	-	-	-	-	-	-	-	-	-	-	-	-	-	-	-	-	-	-	-	-	-	-	-	-	-	-	-	-	-	-	-	-	-	-	-	-
6919	21015	hypothetical protein	1	-	-	-	-	-	-	-	-	-	+	-	-	-	-	-	-	-	-	-	-	-	-	-	-	-	-	-	-	-	-	-	-	-	-	-	-	-	-	-	-	-	-	-	-	-	-	-	-	-	-	-	-	-
6920	21016	hypothetical protein	1	-	-	-	-	-	-	-	-	-	+	-	-	-	-	-	-	-	-	-	-	-	-	-	-	-	-	-	-	-	-	-	-	-	-	-	-	-	-	-	-	-	-	-	-	-	-	-	-	-	-	-	-	-
6921	21017	2-isopropylmalate synthase (EC 2.3.3.13)	1	-	-	-	-	-	-	-	-	-	+	-	-	-	-	-	-	-	-	-	-	-	-	-	-	-	-	-	-	-	-	-	-	-	-	-	-	-	-	-	-	-	-	-	-	-	-	-	-	-	-	-	-	-
6922	21018	hypothetical protein	1	-	-	-	-	-	-	-	-	+	-	-	-	-	-	-	-	-	-	-	-	-	-	-	-	-	-	-	-	-	-	-	-	-	-	-	-	-	-	-	-	-	-	-	-	-	-	-	-	-	-	-	-	-
6923	21019	Plasmid stabilization system toxin protein	1	-	-	-	-	-	-	-	-	+	-	-	-	-	-	-	-	-	-	-	-	-	-	-	-	-	-	-	-	-	-	-	-	-	-	-	-	-	-	-	-	-	-	-	-	-	-	-	-	-	-	-	-	-
6924	21020	hypothetical protein	1	-	-	-	-	-	-	-	-	+	-	-	-	-	-	-	-	-	-	-	-	-	-	-	-	-	-	-	-	-	-	-	-	-	-	-	-	-	-	-	-	-	-	-	-	-	-	-	-	-	-	-	-	-
6925	21021	hypothetical protein	1	-	-	-	-	-	-	-	-	+	-	-	-	-	-	-	-	-	-	-	-	-	-	-	-	-	-	-	-	-	-	-	-	-	-	-	-	-	-	-	-	-	-	-	-	-	-	-	-	-	-	-	-	-
6926	21022	UDP-glucose 4-epimerase (EC 5.1.3.2)	1	-	-	-	-	-	-	-	-	+	-	-	-	-	-	-	-	-	-	-	-	-	-	-	-	-	-	-	-	-	-	-	-	-	-	-	-	-	-	-	-	-	-	-	-	-	-	-	-	-	-	-	-	-
6927	21023	Tetracycline resistance protein	1	-	-	-	-	-	-	-	-	+	-	-	-	-	-	-	-	-	-	-	-	-	-	-	-	-	-	-	-	-	-	-	-	-	-	-	-	-	-	-	-	-	-	-	-	-	-	-	-	-	-	-	-	-
6928	21024	Epidermin leader peptide processing serine protease epiP precursor (EC 3.4.21.-)	1	-	-	-	-	-	-	-	-	+	-	-	-	-	-	-	-	-	-	-	-	-	-	-	-	-	-	-	-	-	-	-	-	-	-	-	-	-	-	-	-	-	-	-	-	-	-	-	-	-	-	-	-	-
6929	21025	hypothetical protein	1	-	-	-	-	-	-	-	-	+	-	-	-	-	-	-	-	-	-	-	-	-	-	-	-	-	-	-	-	-	-	-	-	-	-	-	-	-	-	-	-	-	-	-	-	-	-	-	-	-	-	-	-	-
6930	21026	Two-component response regulator	1	-	-	-	-	-	-	-	-	+	-	-	-	-	-	-	-	-	-	-	-	-	-	-	-	-	-	-	-	-	-	-	-	-	-	-	-	-	-	-	-	-	-	-	-	-	-	-	-	-	-	-	-	-
6931	21027	PTS system, cellobiose-specific IIB component (EC 2.7.1.69)	1	-	-	-	-	-	-	-	-	+	-	-	-	-	-	-	-	-	-	-	-	-	-	-	-	-	-	-	-	-	-	-	-	-	-	-	-	-	-	-	-	-	-	-	-	-	-	-	-	-	-	-	-	-
6932	21028	hypothetical protein	1	-	-	-	-	-	-	-	-	-	-	-	-	-	-	-	-	-	-	-	-	-	-	-	-	-	+	-	-	-	-	-	-	-	-	-	-	-	-	-	-	-	-	-	-	-	-	-	-	-	-	-	-	-
6933	21029	hypothetical protein	1	-	-	-	-	-	-	-	-	-	-	-	-	-	-	-	-	-	-	-	-	-	-	-	-	-	+	-	-	-	-	-	-	-	-	-	-	-	-	-	-	-	-	-	-	-	-	-	-	-	-	-	-	-
6934	21030	Na+-driven multidrug efflux pump	1	-	-	-	-	-	-	-	-	-	-	-	-	-	-	-	-	-	-	-	-	-	-	-	-	-	+	-	-	-	-	-	-	-	-	-	-	-	-	-	-	-	-	-	-	-	-	-	-	-	-	-	-	-
6935	21031	hypothetical protein	1	-	-	-	-	-	-	-	-	-	-	-	-	-	-	-	-	-	-	-	-	-	-	-	-	-	+	-	-	-	-	-	-	-	-	-	-	-	-	-	-	-	-	-	-	-	-	-	-	-	-	-	-	-
6936	21032	membrane protein, putative	1	-	-	-	-	-	-	-	-	-	-	-	-	-	-	-	-	-	-	-	-	-	-	-	-	-	-	-	-	-	-	-	-	-	-	-	-	-	-	-	-	-	-	-	-	-	-	-	-	+	-	-	-	-
6937	21033	hypothetical protein	1	-	-	-	-	-	-	-	-	-	-	-	-	-	-	-	-	-	-	-	-	-	-	-	-	-	-	-	-	-	-	-	-	-	-	-	-	-	-	-	-	-	-	-	-	-	-	-	-	+	-	-	-	-
6938	21034	Prolyl-tRNA synthetase (EC 6.1.1.15)	1	-	-	-	-	-	-	-	-	-	-	-	-	-	-	-	-	-	-	-	-	-	-	-	-	-	-	-	-	-	-	-	-	-	-	-	-	-	-	-	-	-	-	-	-	-	-	-	-	+	-	-	-	-
6939	21035	Export protein for polysaccharides and teichoic acids	1	-	-	-	-	-	-	-	-	-	-	-	-	-	-	-	-	-	-	-	-	-	-	-	-	-	-	-	-	-	-	-	-	-	-	-	-	-	-	-	-	-	-	-	-	-	-	-	-	+	-	-	-	-
6940	21036	hypothetical protein	1	-	-	-	-	-	-	-	-	-	-	-	-	-	-	-	-	-	-	-	-	-	-	-	-	-	-	-	-	-	-	-	-	-	-	-	-	-	-	-	-	-	-	-	-	-	-	-	-	+	-	-	-	-
6941	21037	hypothetical protein	1	-	-	-	-	-	-	-	-	-	-	-	-	-	-	-	-	-	-	-	-	-	-	-	-	-	-	-	-	-	-	-	-	-	-	-	-	-	-	-	-	-	-	-	-	-	-	-	-	+	-	-	-	-
6942	21038	Glutathione peroxidase (EC 1.11.1.9)	1	-	-	-	-	-	-	-	-	-	-	-	-	-	-	-	-	-	-	-	-	-	-	-	-	-	-	-	-	-	-	-	-	-	-	-	-	-	-	-	-	-	-	-	-	-	-	-	-	+	-	-	-	-
6943	21039	hypothetical protein	1	-	-	-	-	-	-	-	-	-	-	-	-	-	-	-	-	-	-	-	-	-	-	-	-	-	-	-	-	-	-	-	-	-	-	-	-	-	-	-	-	-	-	-	-	-	-	-	-	+	-	-	-	-
6944	21040	Glycosyl transferase, group 2 family protein	1	-	-	-	-	-	-	-	-	-	-	-	-	-	-	-	-	-	-	-	-	-	-	-	-	-	-	-	-	-	-	-	-	-	-	-	-	-	-	-	-	-	-	-	-	-	-	-	-	+	-	-	-	-
6945	21041	hypothetical protein	1	-	-	-	-	-	-	-	-	-	-	-	-	-	-	-	-	-	-	-	-	-	-	-	-	-	-	-	-	-	-	-	-	-	-	-	-	-	-	-	-	-	-	-	-	-	-	-	-	+	-	-	-	-
6946	21042	probable arylsulfatase regulator	1	-	-	-	-	-	-	-	-	-	-	-	-	-	-	-	-	-	-	-	-	-	-	-	-	-	-	-	-	-	-	-	-	-	-	-	-	-	-	-	-	-	-	-	-	-	-	-	-	+	-	-	-	-
6947	21043	Aspartate aminotransferase (EC 2.6.1.1)	1	-	-	-	-	-	-	-	-	-	-	-	-	-	-	-	-	-	-	-	-	-	-	-	-	-	-	-	-	-	-	-	-	-	-	-	-	-	-	-	-	-	-	-	-	-	-	-	-	+	-	-	-	-
6948	21044	Sucrose-6-phosphate hydrolase (EC 3.2.1.26)	1	-	-	-	-	-	-	-	-	-	-	-	-	-	-	-	-	-	-	-	-	-	-	-	-	-	-	-	-	-	-	-	-	-	-	-	-	-	-	-	-	-	-	-	-	-	-	-	-	+	-	-	-	-
6949	21045	ABC transporter, ATP-binding protein	1	-	-	-	-	-	-	-	-	-	-	-	-	-	-	-	-	-	-	-	-	-	-	-	-	-	-	-	-	-	-	-	-	-	-	-	-	-	-	-	-	-	-	-	-	-	-	-	-	+	-	-	-	-
6950	21046	hypothetical protein	1	-	-	-	-	-	-	-	-	-	-	-	-	-	-	-	-	-	-	-	-	-	-	-	-	-	-	-	-	-	-	-	-	-	-	-	-	-	-	-	-	-	-	-	-	-	-	-	-	+	-	-	-	-
6951	21047	COG3955: Exopolysaccharide biosynthesis protein	1	-	-	-	-	-	-	-	-	-	-	-	-	-	-	-	-	-	-	-	-	-	-	-	-	-	-	-	-	-	-	-	-	-	-	-	-	-	-	-	-	-	-	-	-	-	-	-	-	+	-	-	-	-
6952	21048	hypothetical protein	1	-	-	-	-	-	-	-	-	-	-	-	-	-	-	-	-	-	-	-	-	-	-	-	-	-	-	-	-	-	-	-	-	-	-	-	-	-	-	-	-	-	-	-	-	-	-	-	-	+	-	-	-	-
6953	21049	hypothetical protein	1	-	-	-	-	-	-	-	-	-	-	-	-	-	-	-	-	-	-	-	-	-	-	-	-	-	-	-	-	-	-	-	-	-	-	-	-	-	-	-	-	-	-	-	-	-	-	-	-	+	-	-	-	-
6954	21050	hypothetical protein	1	-	-	-	-	-	-	-	-	-	-	-	-	-	-	-	-	-	-	-	-	-	-	-	-	-	-	-	-	-	-	-	-	-	-	-	-	-	-	-	-	-	-	-	-	-	-	-	-	+	-	-	-	-
6955	21051	Sulfatase modifying factor 1 precursor (C-alpha-formyglycine- generating enzyme 1)	1	-	-	-	-	-	-	-	-	-	-	-	-	-	-	-	-	-	-	-	-	-	-	-	-	-	-	-	-	-	-	-	-	-	-	-	-	-	-	-	-	-	-	-	-	-	-	-	-	+	-	-	-	-
6956	21052	hypothetical protein	1	-	-	-	-	-	-	-	-	-	-	-	-	-	-	-	-	-	-	-	-	-	-	-	-	-	-	-	-	-	-	-	-	-	-	-	-	-	-	-	-	-	-	-	-	-	-	-	-	+	-	-	-	-
6957	21053	hypothetical protein	1	-	-	-	-	-	-	-	-	-	-	-	-	-	-	-	-	-	-	-	-	-	-	-	-	-	-	-	-	-	-	-	-	-	-	-	-	-	-	-	-	-	-	-	-	-	-	-	-	+	-	-	-	-
6958	21054	hypothetical protein	1	-	-	-	-	-	-	-	-	-	-	-	-	-	-	-	-	-	-	-	-	-	-	-	-	-	-	-	-	-	-	-	-	-	-	-	-	-	-	-	-	-	-	-	-	-	-	-	-	+	-	-	-	-
6959	21055	hypothetical protein	1	-	-	-	-	-	-	-	-	-	-	-	-	-	-	-	-	-	-	-	-	-	-	-	-	-	-	-	-	-	-	-	-	-	-	-	-	-	-	-	-	-	-	-	-	-	-	-	-	+	-	-	-	-
6960	21056	hypothetical protein	1	-	-	-	-	-	-	-	-	-	-	-	-	-	-	-	-	-	-	-	-	-	-	-	-	-	-	-	-	-	-	-	-	-	-	-	-	-	-	-	-	-	-	-	-	-	-	-	-	+	-	-	-	-
6961	21057	hypothetical protein	1	-	-	-	-	-	-	-	-	-	-	-	-	-	-	-	-	-	-	-	-	-	-	-	-	-	-	-	-	-	-	-	-	-	-	-	-	-	-	-	-	-	-	-	-	-	-	-	-	+	-	-	-	-
6962	21058	hypothetical protein	1	-	-	-	-	-	-	-	-	-	-	-	-	-	-	-	-	-	-	-	-	-	-	-	-	-	-	-	-	-	-	-	-	-	-	-	-	-	-	-	-	-	-	-	-	-	-	-	-	+	-	-	-	-
6963	21059	polysaccharide transport protein, putative	1	-	-	-	-	-	-	-	-	-	-	-	-	-	-	-	-	-	-	-	-	-	-	-	-	-	-	-	-	-	-	-	-	-	-	-	-	-	-	-	-	-	-	-	-	-	-	-	-	+	-	-	-	-
6964	21060	hypothetical protein	1	-	-	-	-	-	-	-	-	-	-	-	-	-	-	-	-	-	-	-	-	-	-	-	-	-	-	-	-	-	-	-	-	-	-	-	-	-	-	-	-	-	-	-	-	-	-	-	-	+	-	-	-	-
6965	21061	Galactose operon repressor, GalR-LacI family of transcriptional regulators	1	-	-	-	-	-	-	-	-	-	-	-	-	-	-	-	-	-	-	-	-	-	-	-	-	-	-	-	-	-	-	-	-	-	-	-	-	-	-	-	-	-	-	-	-	-	-	-	-	+	-	-	-	-
6966	21062	hypothetical protein	1	-	-	-	-	-	-	-	-	-	-	-	-	-	-	-	-	-	-	-	-	-	-	-	-	-	-	-	-	-	-	-	-	-	-	-	-	-	-	-	-	-	-	-	-	-	-	-	-	+	-	-	-	-
6967	21063	hypothetical protein	1	-	-	-	-	-	-	-	-	-	-	-	-	-	-	-	-	-	-	-	-	-	-	-	-	-	-	-	-	-	-	-	-	-	-	-	-	-	-	-	-	-	-	-	-	-	-	-	-	+	-	-	-	-
6968	21064	Lj965 prophage protein	1	-	-	-	-	-	-	-	-	-	-	-	-	-	-	-	-	-	-	-	-	-	-	-	-	-	-	-	-	-	-	-	-	-	-	-	-	-	-	-	-	-	-	-	-	-	-	-	-	+	-	-	-	-
6969	21065	hypothetical protein	1	-	-	-	-	-	-	-	-	-	-	-	-	-	-	-	-	-	-	-	-	-	-	-	-	-	-	-	-	-	-	-	-	-	-	-	-	-	-	-	-	-	-	-	-	-	-	-	-	+	-	-	-	-
6970	21066	Poly(glycerol-phosphate) alpha-glucosyltransferase GftA (EC 2.4.1.52)	1	-	-	-	-	-	-	-	-	-	-	-	-	-	-	-	-	-	-	-	-	-	-	-	-	-	-	-	-	-	-	-	-	-	-	-	-	-	-	-	-	-	-	-	-	-	-	-	-	+	-	-	-	-
6971	21067	Mobile element protein	1	-	-	-	-	-	-	-	-	-	-	-	-	-	-	-	-	-	-	-	-	-	-	-	-	-	-	-	-	-	-	-	-	-	-	-	-	-	-	-	-	-	-	-	-	-	-	-	-	+	-	-	-	-
6972	21068	hypothetical protein	1	-	-	-	-	-	-	-	-	-	-	-	-	-	-	-	-	-	-	-	-	-	-	-	-	-	-	-	-	-	-	-	-	-	-	-	-	-	-	-	-	-	-	-	-	-	-	-	-	+	-	-	-	-
6973	21069	hypothetical protein	1	-	-	-	-	-	-	-	-	-	-	-	-	-	-	-	-	-	-	-	-	-	-	-	-	-	-	-	-	-	-	-	-	-	-	-	-	-	-	-	-	-	-	-	-	-	-	-	-	+	-	-	-	-
6974	21070	hypothetical protein	1	-	-	-	-	-	-	-	-	-	-	-	-	-	-	-	-	-	-	-	-	-	-	-	-	-	-	-	-	-	-	-	-	-	-	-	-	-	-	-	-	-	-	-	-	-	-	-	-	+	-	-	-	-
6975	21071	hypothetical protein	1	-	-	-	-	-	-	-	-	-	-	-	-	-	-	-	-	-	-	-	-	-	-	-	-	-	-	-	-	-	-	-	-	-	-	-	-	-	-	-	-	-	-	-	-	-	-	-	-	+	-	-	-	-
6976	21072	hypothetical protein	1	-	-	-	-	-	-	-	-	-	-	-	-	-	-	-	-	-	-	-	-	-	-	-	-	-	-	-	-	-	-	-	-	-	-	-	-	-	-	-	-	-	-	-	-	-	-	-	-	+	-	-	-	-
6977	21073	hypothetical protein	1	-	-	-	-	-	-	-	-	-	-	-	-	-	-	-	-	-	-	-	-	-	-	-	-	-	-	-	-	-	-	-	-	-	-	-	-	-	-	-	-	-	-	-	-	-	-	-	-	+	-	-	-	-
6978	21074	hypothetical protein	1	-	-	-	-	-	-	-	-	-	-	-	-	-	-	-	-	-	-	-	-	-	-	-	-	-	-	-	-	-	-	-	-	-	-	-	-	-	-	-	-	-	-	-	-	-	-	-	-	+	-	-	-	-
6979	21075	Lj965 prophage protein	1	-	-	-	-	-	-	-	-	-	-	-	-	-	-	-	-	-	-	-	-	-	-	-	-	-	-	-	-	-	-	-	-	-	-	-	-	-	-	-	-	-	-	-	-	-	-	-	-	+	-	-	-	-
6980	21076	hypothetical protein	1	-	-	-	-	-	-	-	-	-	-	-	-	-	-	-	-	-	-	-	-	-	-	-	-	-	-	-	-	-	-	-	-	-	-	-	-	-	-	-	-	-	-	-	-	-	-	-	-	+	-	-	-	-
6981	21077	hypothetical protein	1	-	-	-	-	-	-	-	-	-	-	-	-	-	-	-	-	-	-	-	-	-	-	-	-	-	-	-	-	-	-	-	-	-	-	-	-	-	-	-	-	-	-	-	-	-	-	-	-	+	-	-	-	-
6982	21078	hypothetical protein	1	-	-	-	-	-	-	-	-	-	-	-	-	-	-	-	-	-	-	-	-	-	-	-	-	-	-	-	-	-	-	-	-	-	-	-	-	-	-	-	-	-	-	-	-	-	-	-	-	+	-	-	-	-
6983	21079	Cell wall surface anchor family protein	1	-	-	-	-	-	-	-	-	-	-	-	-	-	-	-	-	-	-	-	-	-	-	-	-	-	-	-	-	-	-	-	-	-	-	-	-	-	-	-	-	-	-	-	-	-	-	-	-	+	-	-	-	-
6984	21080	putative glycosyltransferase	1	-	-	-	-	-	-	-	-	-	-	-	-	-	-	-	-	-	-	-	-	-	-	-	-	-	-	-	-	-	-	-	-	-	-	-	-	-	-	-	-	-	-	-	-	-	-	-	-	+	-	-	-	-
6985	21081	hypothetical protein	1	-	-	-	-	-	-	-	-	-	-	-	-	-	-	-	-	-	-	-	-	-	-	-	-	-	-	-	-	-	-	-	-	-	-	-	-	-	-	-	-	-	-	-	-	-	-	-	-	+	-	-	-	-
6986	21082	hypothetical protein	1	-	-	-	-	-	-	-	-	-	-	-	-	-	-	-	-	-	-	-	-	-	-	-	-	-	-	-	-	-	-	-	-	-	-	-	-	-	-	-	-	-	-	-	-	-	-	-	-	+	-	-	-	-
6987	21083	hypothetical protein	1	-	-	-	-	-	-	-	-	-	-	-	-	-	-	-	-	-	-	-	-	-	-	-	-	-	-	-	-	-	-	-	-	-	-	-	-	-	-	-	-	-	-	-	-	-	-	-	-	+	-	-	-	-
6988	21084	Beta-1,3-glucosyltransferase	1	-	-	-	-	-	-	-	-	-	-	-	-	-	-	-	-	-	-	-	-	-	-	-	-	-	-	-	-	-	-	-	-	-	-	-	-	-	-	-	-	-	-	-	-	-	-	-	-	+	-	-	-	-
6989	21085	hypothetical protein	1	-	-	-	-	-	-	-	-	-	-	-	-	-	-	-	-	-	-	-	-	-	-	-	-	-	-	-	-	-	-	-	-	-	-	-	-	-	-	-	-	-	-	-	-	-	-	-	-	+	-	-	-	-
6990	21086	putative transcriptional regulator	1	-	-	-	-	-	-	-	-	-	-	-	-	-	-	-	-	-	-	-	-	-	-	-	-	-	-	-	-	-	-	-	-	-	-	-	-	-	-	-	-	-	-	-	-	-	-	-	-	+	-	-	-	-
6991	21087	hypothetical protein	1	-	-	-	-	-	-	-	-	-	-	-	-	-	-	-	-	-	-	-	-	-	-	-	-	-	-	-	-	-	-	-	-	-	-	-	-	-	-	-	-	-	-	-	-	-	-	-	-	+	-	-	-	-
6992	21088	hypothetical protein	1	-	-	-	-	-	-	-	-	-	-	-	-	-	-	-	-	-	-	-	-	-	-	-	-	-	-	-	-	-	-	-	-	-	-	-	-	-	-	-	-	-	-	-	-	-	-	-	-	+	-	-	-	-
6993	21089	hypothetical protein	1	-	-	-	-	-	-	-	-	-	-	-	-	-	-	-	-	-	-	-	-	-	-	-	-	-	-	-	-	-	-	-	-	-	-	-	-	-	-	-	-	-	-	-	-	-	-	-	-	+	-	-	-	-
6994	21090	Glutathione peroxidase (EC 1.11.1.9)	1	-	-	-	-	-	-	-	-	-	-	-	-	-	-	-	-	-	-	-	-	-	-	-	-	-	-	-	-	-	-	-	-	-	-	-	-	-	-	-	-	-	-	-	-	-	-	-	-	+	-	-	-	-
6995	21091	hypothetical protein	1	-	-	-	-	-	-	-	-	-	-	-	-	-	-	-	-	-	-	-	-	-	-	-	-	-	-	-	-	-	-	-	-	-	-	-	-	-	-	-	-	-	-	-	-	-	-	-	-	+	-	-	-	-
6996	21092	hypothetical protein	1	-	-	-	-	-	-	-	-	-	-	-	-	-	-	-	-	-	-	-	-	-	-	-	-	-	-	-	-	-	-	-	-	-	-	-	-	-	-	-	-	-	-	-	-	-	-	-	-	+	-	-	-	-
6997	21093	hypothetical protein	1	-	-	-	-	-	-	-	-	-	-	-	-	-	-	-	-	-	-	-	-	-	-	-	-	-	-	-	-	-	-	-	-	-	-	-	-	-	-	-	-	-	-	-	-	-	-	-	-	+	-	-	-	-
6998	21094	hypothetical protein	1	-	-	-	-	-	-	-	-	-	-	-	-	-	-	-	-	-	-	-	-	-	-	-	-	-	-	-	-	-	-	-	-	-	-	-	-	-	-	-	-	-	-	-	-	-	-	-	-	+	-	-	-	-
6999	21095	extracellular factor protein	1	-	-	-	-	-	-	-	-	-	-	-	-	-	-	-	-	-	-	-	-	-	-	-	-	-	-	-	-	-	-	-	-	-	-	-	-	-	-	-	-	-	-	-	-	-	-	-	-	+	-	-	-	-
7000	21096	hypothetical protein	1	-	-	-	-	-	-	-	-	-	-	-	-	-	-	-	-	-	-	-	-	-	-	-	-	-	-	-	-	-	-	-	-	-	-	-	-	-	-	-	-	-	-	-	-	-	-	-	-	+	-	-	-	-
7001	21097	hypothetical protein	1	-	-	-	-	-	-	-	-	-	-	-	-	-	-	-	-	-	-	-	-	-	-	-	-	-	-	-	-	-	-	-	-	-	-	-	-	-	-	-	-	-	-	-	-	-	-	-	-	+	-	-	-	-
7002	21098	Alpha-L-Rha alpha-1,3-L-rhamnosyltransferase (EC 2.4.1.-)	1	-	-	-	-	-	-	-	-	-	-	-	-	-	-	-	-	-	-	-	-	-	-	-	-	-	-	-	-	-	-	-	-	-	-	-	-	-	-	-	-	-	-	-	-	-	-	-	-	+	-	-	-	-
7003	21099	hypothetical protein	1	-	-	-	-	-	-	-	-	-	-	-	-	-	-	-	-	-	-	-	-	-	-	-	-	-	-	-	-	-	-	-	-	-	-	-	-	-	-	-	-	-	-	-	-	-	-	-	-	+	-	-	-	-
7004	21100	Mobile element protein	1	-	-	-	-	-	-	-	-	-	-	-	-	-	-	-	-	-	-	-	-	-	-	-	-	-	-	-	-	-	-	-	-	-	-	-	-	-	-	-	-	-	-	-	-	-	-	-	-	+	-	-	-	-
7005	21101	dCMP deaminase (EC 3.5.4.12); Late competence protein ComEB	1	-	-	-	-	-	-	-	-	-	-	-	-	-	-	-	-	-	-	-	-	-	-	-	-	-	-	-	-	-	-	-	-	-	-	-	-	-	-	-	-	-	-	-	-	-	-	-	-	+	-	-	-	-
7006	21102	hypothetical protein	1	-	-	-	-	-	-	-	-	-	-	-	-	-	-	-	-	-	-	-	-	-	-	-	-	-	-	-	-	-	-	-	-	-	-	-	-	-	-	-	-	-	-	-	-	-	-	-	-	+	-	-	-	-
7007	21103	hypothetical protein	1	-	-	-	-	-	-	-	-	-	-	-	-	-	-	-	-	-	-	-	-	-	-	-	-	-	-	-	-	-	-	-	-	-	-	-	-	-	-	-	-	-	-	-	-	-	-	-	-	+	-	-	-	-
7008	21104	hypothetical protein	1	-	-	-	-	-	-	-	-	-	-	-	-	-	-	-	-	-	-	-	-	-	-	-	-	-	-	-	-	-	-	-	-	-	-	-	-	-	-	-	-	-	-	-	-	-	-	-	-	+	-	-	-	-
7009	21105	hypothetical protein	1	-	-	-	-	-	-	-	-	-	-	-	-	-	-	-	-	-	-	-	-	-	-	-	-	-	-	-	-	-	-	-	-	-	-	-	-	-	-	-	-	-	-	-	-	-	-	-	-	+	-	-	-	-
7010	21106	methylase	1	-	-	-	-	-	-	-	-	-	-	-	-	-	-	-	-	-	-	-	-	-	-	-	-	-	-	-	-	-	-	-	-	-	-	-	-	-	-	-	-	-	-	-	-	-	-	-	-	+	-	-	-	-
7011	21107	hypothetical protein	1	-	-	-	-	-	-	-	-	-	-	-	-	-	-	-	-	-	-	-	-	-	-	-	-	-	-	-	-	-	-	-	-	-	-	-	-	-	-	-	-	-	-	-	-	-	-	-	-	+	-	-	-	-
7012	21108	hypothetical protein	1	-	-	-	-	-	-	-	-	-	-	-	-	-	-	-	-	-	-	-	-	-	-	-	-	-	-	-	-	-	-	-	-	-	-	-	-	-	-	-	-	-	-	-	-	-	-	-	-	+	-	-	-	-
7013	21109	hypothetical protein	1	-	-	-	-	-	-	-	-	-	-	-	-	-	-	-	-	-	-	-	-	-	-	-	-	-	-	-	-	-	-	-	-	-	-	-	-	-	-	-	-	-	-	-	-	-	-	-	-	+	-	-	-	-
7014	21110	Mobile element protein	1	-	-	-	-	-	-	-	-	-	-	-	-	-	-	-	-	-	-	-	-	-	-	-	-	-	-	-	-	-	-	-	-	-	-	-	-	-	-	-	-	-	-	-	-	-	-	-	-	+	-	-	-	-
7015	21111	hypothetical protein	1	-	-	-	-	-	-	-	-	-	-	-	-	-	-	-	-	-	-	-	-	-	-	-	-	-	-	-	-	-	-	-	-	-	-	-	-	-	-	-	-	-	-	-	-	-	-	-	-	+	-	-	-	-
7016	21112	Ribonuclease HI-related protein	1	-	-	-	-	-	-	-	-	-	-	-	-	-	-	-	-	-	-	-	-	-	-	-	-	-	-	-	-	-	-	-	-	-	-	-	-	-	-	-	-	-	-	-	-	-	-	-	-	+	-	-	-	-
7017	21113	Mobile element protein	1	-	-	-	-	-	-	-	-	-	-	-	-	-	-	-	-	-	-	-	-	-	-	-	-	-	-	-	-	-	-	-	-	-	-	-	-	-	-	-	-	-	-	-	-	-	-	-	-	+	-	-	-	-
7018	21114	hypothetical protein	1	-	-	-	-	-	-	-	-	-	-	-	-	-	-	-	-	-	-	-	-	-	-	-	-	-	-	-	-	-	-	-	-	-	-	-	-	-	-	-	-	-	-	-	-	-	-	-	-	+	-	-	-	-
7019	21115	hypothetical protein	1	-	-	-	-	-	-	-	-	-	-	-	-	-	-	-	-	-	-	-	-	-	-	-	-	-	-	-	-	-	-	-	-	-	-	-	-	-	-	-	-	-	-	-	-	-	-	-	-	+	-	-	-	-
7020	21116	hypothetical protein	1	-	-	-	-	-	-	-	-	-	-	-	-	-	-	-	-	-	-	-	-	-	-	-	-	-	-	-	-	-	-	-	-	-	-	-	-	-	-	-	-	-	-	-	-	-	-	-	-	+	-	-	-	-
7021	21117	Lipopolysaccharide biosynthesis	1	-	-	-	-	-	-	-	-	-	-	-	-	-	-	-	-	-	-	-	-	-	-	-	-	-	-	-	-	-	-	-	-	-	-	-	-	-	-	-	-	-	-	-	-	-	-	-	-	+	-	-	-	-
7022	21118	cell wall surface anchor family protein	1	-	-	-	-	-	-	-	-	-	-	-	-	-	-	-	-	-	-	-	-	-	-	-	-	-	-	-	-	-	-	-	-	-	-	-	-	-	-	-	-	-	-	-	-	-	-	-	-	+	-	-	-	-
7023	21119	hypothetical protein	1	-	-	-	-	-	-	-	-	-	-	-	-	-	-	-	-	-	-	-	-	-	-	-	-	-	-	-	-	-	-	-	-	-	-	-	-	-	-	-	-	-	-	-	-	-	-	-	-	+	-	-	-	-
7024	21120	hypothetical protein	1	-	-	-	-	-	-	-	-	-	-	-	-	-	-	-	-	-	-	-	-	-	-	-	-	-	-	-	-	-	-	-	-	-	-	-	-	-	-	-	-	-	-	-	-	-	-	-	-	+	-	-	-	-
7025	21121	hypothetical protein	1	-	-	-	-	-	-	-	-	-	-	-	-	-	-	-	-	-	-	-	-	-	-	-	-	-	-	-	-	-	-	-	-	-	-	-	-	-	-	-	-	-	-	-	-	-	-	-	-	+	-	-	-	-
7026	21122	hypothetical protein	1	-	-	-	-	-	-	-	-	-	-	-	-	-	-	-	-	-	-	-	-	-	-	-	-	-	-	-	-	-	-	-	-	-	-	-	-	-	-	-	-	-	-	-	-	-	-	-	-	+	-	-	-	-
7027	21123	hypothetical protein	1	-	-	-	-	-	-	-	-	-	-	-	-	-	-	-	-	-	-	-	-	-	-	-	-	-	-	-	-	-	-	-	-	-	-	-	-	-	-	-	-	-	-	-	-	-	-	-	-	+	-	-	-	-
7028	21124	hypothetical protein	1	-	-	-	-	-	-	-	-	-	-	-	-	-	-	-	-	-	-	-	-	-	-	-	-	-	-	-	-	-	-	-	-	-	-	-	-	-	-	-	-	-	-	-	-	-	-	-	-	+	-	-	-	-
7029	21125	FtsK/SpoIIIE family	1	-	-	-	-	-	-	-	-	-	-	-	-	-	-	-	-	-	-	-	-	-	-	-	-	-	-	-	-	-	-	-	-	-	-	-	-	-	-	-	-	-	-	-	-	-	-	-	-	+	-	-	-	-
7030	21126	hypothetical protein	1	-	-	-	-	-	-	-	-	-	-	-	-	-	-	-	-	-	-	-	-	-	-	-	-	-	-	-	-	-	-	-	-	-	-	-	-	-	-	-	-	-	-	-	-	-	-	-	-	+	-	-	-	-
7031	21127	hypothetical protein	1	-	-	-	-	-	-	-	-	-	-	-	-	-	-	-	-	-	-	-	-	-	-	-	-	-	-	-	-	-	-	-	-	-	-	-	-	-	-	-	-	-	-	-	-	-	-	-	-	+	-	-	-	-
7032	21128	periplasmic copper-binding	1	-	-	-	-	-	-	-	-	-	-	-	-	-	-	-	-	-	-	-	-	-	-	-	-	-	-	-	-	-	-	-	-	-	-	-	-	-	-	-	-	-	-	-	-	-	-	-	-	+	-	-	-	-
7033	21129	hypothetical protein	1	-	-	-	-	-	-	-	-	-	-	-	-	-	-	-	-	-	-	-	-	-	-	-	-	-	-	-	-	-	-	-	-	-	-	-	-	-	-	-	-	-	-	-	-	-	-	-	-	+	-	-	-	-
7034	21130	FIG131328: Predicted ATP-dependent endonuclease of the OLD family	1	-	-	-	-	-	-	-	-	-	-	-	-	-	-	-	-	-	-	-	-	-	-	-	-	-	-	-	-	-	-	-	-	-	-	-	-	-	-	-	-	-	-	-	-	-	-	-	-	+	-	-	-	-
7035	21131	hypothetical protein	1	-	-	-	-	-	-	-	-	-	-	-	-	-	-	-	-	-	-	-	-	-	-	-	-	-	-	-	-	-	-	-	-	-	-	-	-	-	-	-	-	-	-	-	-	-	-	-	-	+	-	-	-	-
7036	21132	hypothetical protein	1	-	-	-	-	-	-	-	-	-	-	-	-	-	-	-	-	-	-	-	-	-	-	-	-	-	-	-	-	-	-	-	-	-	-	-	-	-	-	-	-	-	-	-	-	-	-	-	-	+	-	-	-	-
7037	21133	glycosyl transferase, family 2	1	-	-	-	-	-	-	-	-	-	-	-	-	-	-	-	-	-	-	-	-	-	-	-	-	-	-	-	-	-	-	-	-	-	-	-	-	-	-	-	-	-	-	-	-	-	-	-	-	+	-	-	-	-
7038	21134	hypothetical protein	1	-	-	-	-	-	-	-	-	-	-	-	-	-	-	-	-	-	-	-	-	-	-	-	-	-	-	-	-	-	-	-	-	-	-	-	-	-	-	-	-	-	-	-	-	-	-	-	-	+	-	-	-	-
7039	21135	predicted 4-deoxy-L-threo-5-hexosulose-uronate ketol-isomerase (EC 5.3.1.17)	1	-	-	-	-	-	-	-	-	-	-	-	-	-	-	-	-	-	-	-	-	-	-	-	-	-	-	-	-	-	-	-	-	-	-	-	-	-	-	-	-	-	-	-	-	-	-	-	-	+	-	-	-	-
7040	21136	hypothetical protein	1	-	-	-	-	-	-	-	-	-	-	-	-	-	-	-	-	-	-	-	-	-	-	-	-	-	-	-	-	-	-	-	-	-	-	-	-	-	-	-	-	-	-	-	-	-	-	-	-	+	-	-	-	-
7041	21137	Alpha-glycerophosphate oxidase (EC 1.1.3.21)	1	-	-	-	-	-	-	-	-	-	-	-	-	-	-	-	-	-	-	-	-	-	-	-	-	-	-	-	-	-	-	-	-	-	-	-	-	-	-	-	-	-	-	-	-	-	-	-	-	+	-	-	-	-
7042	21138	hypothetical protein	1	-	-	-	-	-	-	-	-	-	-	-	-	-	-	-	-	-	-	-	-	-	-	-	-	-	-	-	-	-	-	-	-	-	-	-	-	-	-	-	-	-	-	-	-	-	-	-	-	+	-	-	-	-
7043	21139	hypothetical protein	1	-	-	-	-	-	-	-	-	-	-	-	-	-	-	-	-	-	-	-	-	-	-	-	-	-	-	-	-	-	-	-	-	-	-	-	-	-	-	-	-	-	-	-	-	-	-	-	-	+	-	-	-	-
7044	21140	hypothetical protein	1	-	-	-	-	-	-	-	-	-	-	-	-	-	-	-	-	-	-	-	-	-	-	-	-	-	-	-	-	-	-	-	-	-	-	-	-	-	-	-	-	-	-	-	-	-	-	-	-	+	-	-	-	-
7045	21141	Beta-1,3-glucosyltransferase	1	-	-	-	-	-	-	-	-	-	-	-	-	-	-	-	-	-	-	-	-	-	-	-	-	-	-	-	-	-	-	-	-	-	-	-	-	-	-	-	-	-	-	-	-	-	-	-	-	+	-	-	-	-
7046	21142	hypothetical protein	1	-	-	-	-	-	-	-	-	-	-	-	-	-	-	-	-	-	-	-	-	-	-	-	-	-	-	-	-	-	-	-	-	-	-	-	-	-	-	-	-	-	-	-	-	-	-	-	-	+	-	-	-	-
7047	21143	hypothetical protein	1	-	-	-	-	-	-	-	-	-	-	-	-	-	-	-	-	-	-	-	-	-	-	-	-	-	-	-	-	-	-	-	-	-	-	-	-	-	-	-	-	-	-	-	-	-	-	-	-	+	-	-	-	-
7048	21144	hypothetical protein	1	-	-	-	-	-	-	-	-	-	-	-	-	-	-	-	-	-	-	-	-	-	-	-	-	-	-	-	-	-	-	-	-	-	-	-	-	-	-	-	-	-	-	-	-	-	-	-	-	+	-	-	-	-
7049	21145	transporter accessory protein, putative	1	-	-	-	-	-	-	-	-	-	-	-	-	-	-	-	-	-	-	-	-	-	-	-	-	-	-	-	-	-	-	-	-	-	-	-	-	-	-	-	-	-	-	-	-	-	-	-	-	+	-	-	-	-
7050	21146	PTS system, mannose-specific IIC component (EC 2.7.1.69)	1	-	-	-	-	-	-	-	-	-	-	-	-	-	-	-	-	-	-	-	-	-	-	-	-	-	-	-	-	-	-	-	-	-	-	-	-	-	-	-	-	-	-	-	-	-	-	-	-	+	-	-	-	-
7051	21147	predicted protein	1	-	-	-	-	-	-	-	-	-	-	-	-	-	-	-	-	-	-	-	-	-	-	-	-	-	-	-	-	-	-	-	-	-	-	-	-	-	-	-	-	-	-	-	-	-	-	-	-	+	-	-	-	-
7052	21148	hypothetical protein	1	-	-	-	-	-	-	-	-	-	-	-	-	-	-	-	-	-	-	-	-	-	-	-	-	-	-	-	-	-	-	-	-	-	-	-	-	-	-	-	-	-	-	-	-	-	-	-	-	+	-	-	-	-
7053	21149	hypothetical protein	1	-	-	-	-	-	-	-	-	-	-	-	-	-	-	-	-	-	-	-	-	-	-	-	-	-	-	-	-	-	-	-	-	-	-	-	-	-	-	-	-	-	-	-	-	-	-	-	-	+	-	-	-	-
7054	21150	hypothetical protein	1	-	-	-	-	-	-	-	-	-	-	-	-	-	-	-	-	-	-	-	-	-	-	-	-	-	-	-	-	-	-	-	-	-	-	-	-	-	-	-	-	-	-	-	-	-	-	-	-	+	-	-	-	-
7055	21151	hypothetical protein	1	-	-	-	-	-	-	-	-	-	-	-	-	-	-	-	-	-	-	-	-	-	-	-	-	-	-	-	-	-	-	-	-	-	-	-	-	-	-	-	-	-	-	-	-	-	-	-	-	+	-	-	-	-
7056	21152	YheO-like PAS domain	1	-	-	-	-	-	-	-	-	-	-	-	-	-	-	-	-	-	-	-	-	-	-	-	-	-	-	-	-	-	-	-	-	-	-	-	-	-	-	-	-	-	-	-	-	-	-	-	-	+	-	-	-	-
7057	21153	Glycosyltransferases involved in cell wall biogenesis	1	-	-	-	-	-	-	-	-	-	-	-	-	-	-	-	-	-	-	-	-	-	-	-	-	-	-	-	-	-	-	-	-	-	-	-	-	-	-	-	-	-	-	-	-	-	-	-	-	+	-	-	-	-
7058	21154	hypothetical protein	1	-	-	-	-	-	-	-	-	-	-	-	-	-	-	-	-	-	-	-	-	-	-	-	-	-	-	-	-	-	-	-	-	-	-	-	-	-	-	-	-	-	-	-	-	-	-	-	-	+	-	-	-	-
7059	21155	hypothetical protein	1	-	-	-	-	-	-	-	-	-	-	-	-	-	-	-	-	-	-	-	-	-	-	-	-	-	-	-	-	-	-	-	-	-	-	-	-	-	-	-	-	-	-	-	-	-	-	-	-	+	-	-	-	-
7060	21156	hypothetical protein	1	-	-	-	-	-	-	-	-	-	-	-	-	-	-	-	-	-	-	-	-	-	-	-	-	-	-	-	-	-	-	-	-	-	-	-	-	-	-	-	-	-	-	-	-	-	-	-	-	+	-	-	-	-
7061	21157	hypothetical protein	1	-	-	-	-	-	-	-	-	-	-	-	-	-	-	-	-	-	-	-	-	-	-	-	-	-	-	-	-	-	-	-	-	-	-	-	-	-	-	-	-	-	-	-	-	-	-	-	-	+	-	-	-	-
7062	21158	hypothetical protein	1	-	-	-	-	-	-	-	-	-	-	-	-	-	-	-	-	-	-	-	-	-	-	-	-	-	-	-	-	-	-	-	-	-	-	-	-	-	-	-	-	-	-	-	-	-	-	-	-	+	-	-	-	-
7063	21159	hypothetical protein	1	-	-	-	-	-	-	-	-	-	-	-	-	-	-	-	-	-	-	-	-	-	-	-	-	-	-	-	-	-	-	-	-	-	-	-	-	-	-	-	-	-	-	-	-	-	-	-	-	+	-	-	-	-
7064	21160	hypothetical protein	1	-	-	-	-	-	-	-	-	-	-	-	-	-	-	-	-	-	-	-	-	-	-	-	-	-	-	-	-	-	-	-	-	-	-	-	-	-	-	-	-	-	-	-	-	-	-	-	-	+	-	-	-	-
7065	21161	hypothetical protein	1	-	-	-	-	-	-	-	-	-	-	-	-	-	-	-	-	-	-	-	-	-	-	-	-	-	-	-	-	-	-	-	-	-	-	-	-	-	-	-	-	-	-	-	-	-	-	-	-	+	-	-	-	-
7066	21162	putative fucosyl transferase	1	-	-	-	-	-	-	-	-	-	-	-	-	-	-	-	-	-	-	-	-	-	-	-	-	-	-	-	-	-	-	-	-	-	-	-	-	-	-	-	-	-	-	-	-	-	-	-	-	+	-	-	-	-
7067	21163	PTS system, mannose-specific IID component (EC 2.7.1.69)	1	-	-	-	-	-	-	-	-	-	-	-	-	-	-	-	-	-	-	-	-	-	-	-	-	-	-	-	-	-	-	-	-	-	-	-	-	-	-	-	-	-	-	-	-	-	-	-	-	+	-	-	-	-
7068	21164	Type I restriction-modification system, specificity subunit S (EC 3.1.21.3)	1	-	-	-	-	-	-	-	-	-	-	-	-	-	-	-	-	-	-	-	-	-	-	-	-	-	-	-	-	-	-	-	-	-	-	-	-	-	-	-	-	-	-	-	-	-	-	-	-	+	-	-	-	-
7069	21165	Glycosyl transferase, family 8	1	-	-	-	-	-	-	-	-	-	-	-	-	-	-	-	-	-	-	-	-	-	-	-	-	-	-	-	-	-	-	-	-	-	-	-	-	-	-	-	-	-	-	-	-	-	-	-	-	+	-	-	-	-
7070	21166	glycosyl transferase	1	-	-	-	-	-	-	-	-	-	-	-	-	-	-	-	-	-	-	-	-	-	-	-	-	-	-	-	-	-	-	-	-	-	-	-	-	-	-	-	-	-	-	-	-	-	-	-	-	+	-	-	-	-
7071	21167	hypothetical protein	1	-	-	-	-	-	-	-	-	-	-	-	-	-	-	-	-	-	-	-	-	-	-	-	-	-	-	-	-	-	-	-	-	-	-	-	-	-	-	-	-	-	-	-	-	-	-	-	-	+	-	-	-	-
7072	21168	hypothetical protein	1	-	-	-	-	-	-	-	-	-	-	-	-	-	-	-	-	-	-	-	-	-	-	-	-	-	-	-	-	-	-	-	-	-	-	-	-	-	-	-	-	-	-	-	-	-	-	-	-	+	-	-	-	-
7073	21169	membrane protein, putative	1	-	-	-	-	-	-	-	-	-	-	-	-	-	-	-	-	-	-	-	-	-	-	-	-	-	-	-	-	-	-	-	-	-	-	-	-	-	-	-	-	-	-	-	-	-	-	-	-	+	-	-	-	-
7074	21170	Choline-sulfatase (EC 3.1.6.6)	1	-	-	-	-	-	-	-	-	-	-	-	-	-	-	-	-	-	-	-	-	-	-	-	-	-	-	-	-	-	-	-	-	-	-	-	-	-	-	-	-	-	-	-	-	-	-	-	-	+	-	-	-	-
7075	21171	hypothetical protein	1	-	-	-	-	-	-	-	-	-	-	-	-	-	-	-	-	-	-	-	-	-	-	-	-	-	-	-	-	-	-	-	-	-	-	-	-	-	-	-	-	-	-	-	-	-	-	-	-	+	-	-	-	-
7076	21172	hypothetical protein	1	-	-	-	-	-	-	-	-	-	-	-	-	-	-	-	-	-	-	-	-	-	-	-	-	-	-	-	-	-	-	-	-	-	-	-	-	-	-	-	-	-	-	-	-	-	-	-	-	+	-	-	-	-
7077	21173	hypothetical protein	1	-	-	-	-	-	-	-	-	-	-	-	-	-	-	-	-	-	-	-	-	-	-	-	-	-	-	-	-	-	-	-	-	-	-	-	-	-	-	-	-	-	-	-	-	-	-	-	-	+	-	-	-	-
7078	21174	hypothetical protein	1	-	-	-	-	-	-	-	-	-	-	-	-	-	-	-	-	-	-	-	-	-	-	-	-	-	-	-	-	-	-	-	-	-	-	-	-	-	-	-	-	-	-	-	-	-	-	-	-	+	-	-	-	-
7079	21175	hypothetical protein	1	-	-	-	-	-	-	-	-	-	-	-	-	-	-	-	-	-	-	-	-	-	-	-	-	-	-	-	-	-	-	-	-	-	-	-	-	-	-	-	-	-	-	-	-	-	-	-	-	+	-	-	-	-
7080	21176	hypothetical protein	1	-	-	-	-	-	-	-	-	-	-	-	-	-	-	-	-	-	-	-	-	-	-	-	-	-	-	-	-	-	-	-	-	-	-	-	-	-	-	-	-	-	-	-	-	-	-	-	-	+	-	-	-	-
7081	21177	hypothetical protein	1	-	-	-	-	-	-	-	-	-	-	-	-	-	-	-	-	-	-	-	-	-	-	-	-	-	-	-	-	-	-	-	-	-	-	-	-	-	-	-	-	-	-	-	-	-	-	-	-	+	-	-	-	-
7082	21178	hypothetical protein	1	-	-	-	-	-	-	-	-	-	-	-	-	-	-	-	-	-	-	-	-	-	-	-	-	-	-	-	-	-	-	-	-	-	-	-	-	-	-	-	-	-	-	-	-	-	-	-	-	+	-	-	-	-
7083	21179	hypothetical protein	1	-	-	-	-	-	-	-	-	-	-	-	-	-	-	-	-	-	-	-	-	-	-	-	-	-	-	-	-	-	-	-	-	-	-	-	-	-	-	-	-	-	-	-	-	+	-	-	-	-	-	-	-	-
7084	21180	Type I restriction-modification system, DNA-methyltransferase subunit M (EC 2.1.1.72)	1	-	-	-	-	-	-	-	-	-	-	-	-	-	-	-	-	-	-	-	-	-	-	-	-	-	-	-	-	-	-	-	-	-	-	-	-	-	-	-	-	-	-	-	-	+	-	-	-	-	-	-	-	-
7085	21181	BlpS protein	1	-	-	-	-	-	-	-	-	-	-	-	-	-	-	-	-	-	-	-	-	-	-	-	-	-	-	-	-	-	-	-	-	-	-	-	-	-	-	-	-	-	-	-	-	+	-	-	-	-	-	-	-	-
7086	21182	hypothetical protein	1	-	-	-	-	-	-	-	-	-	-	-	-	-	-	-	-	-	-	-	-	-	-	-	-	-	-	-	-	-	-	-	-	-	-	-	-	-	-	-	-	-	-	-	-	+	-	-	-	-	-	-	-	-
7087	21183	putative	1	-	-	-	-	-	-	-	-	-	-	-	-	-	-	-	-	-	-	-	-	-	-	-	-	-	-	-	-	-	-	-	-	-	-	-	-	-	-	-	-	-	-	-	-	+	-	-	-	-	-	-	-	-
7088	21184	hypothetical protein	1	-	-	-	-	-	-	-	-	-	-	-	-	-	-	-	-	-	-	-	-	-	-	-	-	-	-	-	-	-	-	-	-	-	-	-	-	-	-	-	-	-	-	-	-	+	-	-	-	-	-	-	-	-
7089	21185	Mobile element protein	1	-	-	-	-	-	-	-	-	-	-	-	-	-	-	-	-	-	-	-	-	-	-	-	-	-	-	-	-	-	-	-	-	-	-	-	-	-	-	-	-	-	-	-	-	+	-	-	-	-	-	-	-	-

Table S5. Precence/absence of each HG among 57 *S. suis* strains

				1																13			25					28						104		94			76			53/54		Divergent *S. suis*																
Index[a]	ClutserID	Gene products	Members	98HAH33	05ZYH33	SC84	A7	SS12	P1/7	DAT273	NIAH11434	JS14	GZ1	BM407	S735	13730	MNCM07	NCTC 10446	14636	NCTC 10237	ST1	2651	89-1591	MNCM04	MNCM25	8074	D9	4961	ST3	MNCM43	DAT292	DAT260	NIAH11435	MNCM21	MNCM50	89-2479	DAT234	DAT290	93A	42A	NT77	6407	11538	8830	12814	92-1400	89-590	2726	2524	89-3576-3	4417	D12	10581	89-5259	88-5299A	R61	92-1191	14A	92-4172	22083
7090	21186	hypothetical protein	1	-	-	-	-	-	-	-	-	-	-	-	-	-	-	-	-	-	-	-	-	-	-	-	-	-	-	-	-	-	-	-	-	-	-	-	-	-	-	-	-	-	-	-	-	+	-	-	-	-	-	-	-	-
7091	21187	McrBC restriction endonuclease system, McrB subunit, putative	1	-	-	-	-	-	-	-	-	-	-	-	-	-	-	-	-	-	-	-	-	-	-	-	-	-	-	-	-	-	-	-	-	-	-	-	-	-	-	-	-	-	-	-	-	+	-	-	-	-	-	-	-	-
7092	21188	Ribosomal RNA large subunit methyltransferase N (EC 2.1.1.-)	1	-	-	-	-	-	-	-	-	-	-	-	-	-	-	-	-	-	-	-	-	-	-	-	-	-	-	-	-	-	-	-	-	-	-	-	-	-	-	-	-	-	-	-	-	+	-	-	-	-	-	-	-	-
7093	21189	hypothetical protein	1	-	-	-	-	-	-	-	-	-	-	-	-	-	-	-	-	-	-	-	-	-	-	-	-	-	-	-	-	-	-	-	-	-	-	-	-	-	-	-	-	-	-	-	-	+	-	-	-	-	-	-	-	-
7094	21190	hypothetical protein	1	-	-	-	-	-	-	-	-	-	-	-	-	-	-	-	-	-	-	-	-	-	-	-	-	-	-	-	-	-	-	-	-	-	-	-	-	-	-	-	-	-	-	-	-	+	-	-	-	-	-	-	-	-
7095	21191	Tn5252, relaxase	1	-	-	-	-	-	-	-	-	-	-	-	-	-	-	-	-	-	-	-	-	-	-	-	-	-	-	-	-	-	-	-	-	-	-	-	-	-	-	-	-	-	-	-	-	+	-	-	-	-	-	-	-	-
7096	21192	Phosphoribosylformylglycinamidine cyclo-ligase (EC 6.3.3.1)	1	-	-	-	-	-	-	-	-	-	-	-	-	-	-	-	-	-	-	-	-	-	-	-	-	-	-	-	-	-	-	-	-	-	-	-	-	-	-	-	-	-	-	-	-	+	-	-	-	-	-	-	-	-
7097	21193	Site-specific recombinase	1	-	-	-	-	-	-	-	-	-	-	-	-	-	-	-	-	-	-	-	-	-	-	-	-	-	-	-	-	-	-	-	-	-	-	-	-	-	-	-	-	-	-	-	-	+	-	-	-	-	-	-	-	-
7098	21194	hypothetical protein	1	-	-	-	-	-	-	-	-	-	-	-	-	-	-	-	-	-	-	-	-	-	-	-	-	-	-	-	-	-	-	-	-	-	-	-	-	-	-	-	-	-	-	-	-	+	-	-	-	-	-	-	-	-
7099	21195	hypothetical protein	1	-	-	-	-	-	-	-	-	-	-	-	-	-	-	-	-	-	-	-	-	-	-	-	-	-	-	-	-	-	-	-	-	-	-	-	-	-	-	-	-	-	-	-	-	+	-	-	-	-	-	-	-	-
7100	21196	hypothetical protein	1	-	-	-	-	-	-	-	-	-	-	-	-	-	-	-	-	-	-	-	-	-	-	-	-	-	-	-	-	-	-	-	-	-	-	-	-	-	-	-	-	-	-	-	-	+	-	-	-	-	-	-	-	-
7101	21197	hypothetical protein	1	-	-	-	-	-	-	-	-	-	-	-	-	-	-	-	-	-	-	-	-	-	-	-	-	-	-	-	-	-	-	-	-	-	-	-	-	-	-	-	-	-	-	-	-	+	-	-	-	-	-	-	-	-
7102	21198	hypothetical protein	1	-	-	-	-	-	-	-	-	-	-	-	-	-	-	-	-	-	-	-	-	-	-	-	-	-	-	-	-	-	-	-	-	-	-	-	-	-	-	-	-	-	-	-	-	+	-	-	-	-	-	-	-	-
7103	21199	hypothetical protein	1	-	-	-	-	-	-	-	-	-	-	-	-	-	-	-	-	-	-	-	-	-	-	-	-	-	-	-	-	-	-	-	-	-	-	-	-	-	-	-	-	-	-	-	-	+	-	-	-	-	-	-	-	-
7104	21200	hypothetical protein	1	-	-	-	-	-	-	-	-	-	-	-	-	-	-	-	-	-	-	-	-	-	-	-	-	-	-	-	-	-	-	-	-	-	-	-	-	-	-	-	-	-	-	-	-	+	-	-	-	-	-	-	-	-
7105	21201	Transcriptional regulator, MarR family	1	-	-	-	-	-	-	-	-	-	-	-	-	-	-	-	-	-	-	-	-	-	-	-	-	-	-	-	-	-	-	-	-	-	-	-	-	-	-	-	-	-	-	-	-	+	-	-	-	-	-	-	-	-
7106	21202	unknown protein	1	-	-	-	-	-	-	-	-	-	-	-	-	-	-	-	-	-	-	-	-	-	-	-	-	-	-	-	-	-	-	-	-	-	-	-	-	-	-	-	-	-	-	-	-	+	-	-	-	-	-	-	-	-
7107	21203	hypothetical protein	1	-	-	-	-	-	-	-	-	-	-	-	-	-	-	-	-	-	-	-	-	-	-	-	-	-	-	-	-	-	-	-	-	-	-	-	-	-	-	-	-	-	-	-	-	+	-	-	-	-	-	-	-	-
7108	21204	hypothetical protein	1	-	-	-	-	-	-	-	-	-	-	-	-	-	-	-	-	-	-	-	-	-	-	-	-	-	-	-	-	-	-	-	-	-	-	-	-	-	-	-	-	-	-	-	-	+	-	-	-	-	-	-	-	-
7109	21205	Site-specific recombinase	1	-	-	-	-	-	-	-	-	-	-	-	-	-	-	-	-	-	-	-	-	-	-	-	-	-	-	-	-	-	-	-	-	-	-	-	-	-	-	-	-	-	-	-	-	+	-	-	-	-	-	-	-	-
7110	21206	hypothetical protein	1	-	-	-	-	-	-	-	-	-	-	-	-	-	-	-	-	-	-	-	-	-	-	-	-	-	-	-	-	-	-	-	-	-	-	-	-	-	-	-	-	-	-	-	-	+	-	-	-	-	-	-	-	-
7111	21207	hypothetical protein	1	-	-	-	-	-	-	-	-	-	-	-	-	-	-	-	-	-	-	-	-	-	-	-	-	-	-	-	-	-	-	-	-	-	-	-	-	-	-	-	-	-	-	-	-	+	-	-	-	-	-	-	-	-
7112	21208	N-acetylmuramoyl-L-alanine amidase (EC 3.5.1.28)	1	-	-	-	-	-	-	-	-	-	-	-	-	-	-	-	-	-	-	-	-	-	-	-	-	-	-	-	-	-	-	-	-	-	-	-	-	-	-	-	-	-	-	-	-	+	-	-	-	-	-	-	-	-
7113	21209	hypothetical protein	1	-	-	-	-	-	-	-	-	-	-	-	-	-	-	-	-	-	-	-	-	-	-	-	-	-	-	-	-	-	-	-	-	-	-	-	-	-	-	-	-	-	-	-	-	+	-	-	-	-	-	-	-	-
7114	21210	hypothetical protein	1	-	-	-	-	-	-	-	-	-	-	-	-	-	-	-	-	-	-	-	-	-	-	-	-	-	-	-	-	-	-	-	-	-	-	-	-	-	-	-	-	-	-	-	-	+	-	-	-	-	-	-	-	-
7115	21211	hypothetical protein	1	-	-	-	-	-	-	-	-	-	-	-	-	-	-	-	-	-	-	-	-	-	-	-	-	-	-	-	-	-	-	-	-	-	-	-	-	-	-	-	-	-	-	-	-	+	-	-	-	-	-	-	-	-
7116	21212	hypothetical protein	1	-	-	-	-	-	-	-	-	-	-	-	-	-	-	-	-	-	-	-	-	-	-	-	-	-	-	-	-	-	-	-	-	-	-	-	-	-	-	-	-	-	-	-	-	+	-	-	-	-	-	-	-	-
7117	21213	Predicted hydrolase (HAD superfamily)	1	-	-	-	-	-	-	-	-	-	-	-	-	-	-	-	-	-	-	-	-	-	-	-	-	-	-	-	-	-	-	-	-	-	-	-	-	-	-	-	-	-	-	-	-	+	-	-	-	-	-	-	-	-
7118	21214	DNA-cytosine methyltransferase (EC 2.1.1.37)	1	-	-	-	-	-	-	-	-	-	-	-	-	-	-	-	-	-	-	-	-	-	-	-	-	-	-	-	-	-	-	-	-	-	-	-	-	-	-	-	-	-	-	-	-	+	-	-	-	-	-	-	-	-
7119	21215	hypothetical protein	1	-	-	-	-	-	-	-	-	-	-	-	-	-	-	-	-	-	-	-	-	-	-	-	-	-	-	-	-	-	-	-	-	-	-	-	-	-	-	-	-	-	-	-	-	+	-	-	-	-	-	-	-	-
7120	21216	hypothetical protein	1	-	-	-	-	-	-	-	-	-	-	-	-	-	-	-	-	-	-	-	-	-	-	-	-	-	-	-	-	-	-	-	-	-	-	-	-	-	-	-	-	-	-	-	-	+	-	-	-	-	-	-	-	-
7121	21217	hypothetical protein	1	-	-	-	-	-	-	-	-	-	-	-	-	-	-	-	-	-	-	-	-	-	-	-	-	-	-	-	-	-	-	-	-	-	-	-	-	-	-	-	-	-	-	-	-	+	-	-	-	-	-	-	-	-
7122	21218	Translation initiation factor 2 (IF-2; GTPase)	1	-	-	-	-	-	-	-	-	-	-	-	-	-	-	-	-	-	-	-	-	-	-	-	-	-	-	-	-	-	-	-	-	-	-	-	-	-	-	-	-	-	-	-	-	+	-	-	-	-	-	-	-	-
7123	21219	hypothetical protein	1	-	-	-	-	-	-	-	-	-	-	-	-	-	-	-	-	-	-	-	-	-	-	-	-	-	-	-	-	-	-	-	-	-	-	-	-	-	-	-	-	-	-	-	-	+	-	-	-	-	-	-	-	-
7124	21220	Type I restriction-modification system, specificity subunit S (EC 3.1.21.3)	1	-	-	-	-	-	-	-	-	-	-	-	-	-	-	-	-	-	-	-	-	-	-	-	-	-	-	-	-	-	-	-	-	-	-	-	-	-	-	-	-	-	-	-	-	+	-	-	-	-	-	-	-	-
7125	21221	hypothetical protein	1	-	-	-	-	-	-	-	-	-	-	-	-	-	-	-	-	-	-	-	-	-	-	-	-	-	-	-	-	-	-	-	-	-	-	-	-	-	-	-	-	-	-	-	-	+	-	-	-	-	-	-	-	-
7126	21222	hypothetical protein	1	-	-	-	-	-	-	-	-	-	-	-	-	-	-	-	-	-	-	-	-	-	-	-	-	-	-	-	-	-	-	-	-	-	-	-	-	-	-	-	-	-	-	-	-	+	-	-	-	-	-	-	-	-
7127	21223	Adenine-specific methyltransferase (EC 2.1.1.72)	1	-	-	-	-	-	-	-	-	-	-	-	-	-	-	-	-	-	-	-	-	-	-	-	-	-	-	-	-	-	-	-	-	-	-	-	-	-	-	-	-	-	-	-	-	+	-	-	-	-	-	-	-	-
7128	21224	Transcriptional regulator, MarR family	1	-	-	-	-	-	-	-	-	-	-	-	-	-	-	-	-	-	-	-	-	-	-	-	-	-	-	-	-	-	-	-	-	-	-	-	-	-	-	-	-	-	-	-	-	+	-	-	-	-	-	-	-	-
7129	21225	hypothetical protein	1	-	-	-	-	-	-	-	-	-	-	-	-	-	-	-	-	-	-	-	-	-	-	-	-	-	-	-	-	-	-	-	-	-	-	-	-	-	-	-	-	-	-	-	-	+	-	-	-	-	-	-	-	-
7130	21226	hypothetical protein	1	-	-	-	-	-	-	-	-	-	-	-	-	-	-	-	-	-	-	-	-	-	-	-	+	-	-	-	-	-	-	-	-	-	-	-	-	-	-	-	-	-	-	-	-	-	-	-	-	-	-	-	-	-
7131	21227	Mg(2+) transport ATPase protein C	1	-	-	-	-	-	-	-	-	-	-	-	-	-	-	-	-	-	-	-	-	-	-	-	+	-	-	-	-	-	-	-	-	-	-	-	-	-	-	-	-	-	-	-	-	-	-	-	-	-	-	-	-	-
7132	21228	hypothetical protein	1	-	-	-	-	-	-	-	-	-	-	-	-	-	-	-	-	-	-	-	-	-	-	-	+	-	-	-	-	-	-	-	-	-	-	-	-	-	-	-	-	-	-	-	-	-	-	-	-	-	-	-	-	-
7133	21229	Glycerophosphoryl diester phosphodiesterase (EC 3.1.4.46)	1	-	-	-	-	-	-	-	-	-	-	-	-	-	-	-	-	-	-	-	-	-	-	-	+	-	-	-	-	-	-	-	-	-	-	-	-	-	-	-	-	-	-	-	-	-	-	-	-	-	-	-	-	-
7134	21230	hypothetical protein	1	-	-	-	-	-	-	-	-	-	-	-	-	-	-	-	-	-	-	-	-	-	-	-	+	-	-	-	-	-	-	-	-	-	-	-	-	-	-	-	-	-	-	-	-	-	-	-	-	-	-	-	-	-
7135	21231	hypothetical protein	1	-	-	-	-	-	-	-	-	-	-	-	-	-	-	-	-	-	-	-	-	-	-	-	+	-	-	-	-	-	-	-	-	-	-	-	-	-	-	-	-	-	-	-	-	-	-	-	-	-	-	-	-	-
7136	21232	hypothetical protein	1	-	-	-	-	-	-	-	-	-	-	-	-	-	-	-	-	-	-	-	-	-	-	-	+	-	-	-	-	-	-	-	-	-	-	-	-	-	-	-	-	-	-	-	-	-	-	-	-	-	-	-	-	-
7137	21233	PTS system, mannose-specific IID component (EC 2.7.1.69)	1	-	-	-	-	-	-	-	-	-	-	-	-	-	-	-	-	-	-	-	-	-	-	-	+	-	-	-	-	-	-	-	-	-	-	-	-	-	-	-	-	-	-	-	-	-	-	-	-	-	-	-	-	-
7138	21234	hypothetical protein	1	-	-	-	-	-	-	-	-	-	-	-	-	-	-	-	-	-	-	-	-	-	-	-	+	-	-	-	-	-	-	-	-	-	-	-	-	-	-	-	-	-	-	-	-	-	-	-	-	-	-	-	-	-
7139	21235	Group B streptococcal surface immunogenic protein	1	-	-	-	-	-	-	-	-	-	-	-	-	-	-	-	-	-	-	-	-	-	-	-	+	-	-	-	-	-	-	-	-	-	-	-	-	-	-	-	-	-	-	-	-	-	-	-	-	-	-	-	-	-
7140	21236	hypothetical protein	1	-	-	-	-	-	-	-	-	-	-	-	-	-	-	-	-	-	-	-	-	-	-	-	+	-	-	-	-	-	-	-	-	-	-	-	-	-	-	-	-	-	-	-	-	-	-	-	-	-	-	-	-	-
7141	21237	PTS system, mannose-specific IIC component (EC 2.7.1.69)	1	-	-	-	-	-	-	-	-	-	-	-	-	-	-	-	-	-	-	-	-	-	-	-	+	-	-	-	-	-	-	-	-	-	-	-	-	-	-	-	-	-	-	-	-	-	-	-	-	-	-	-	-	-
7142	21238	PTS system, mannose-specific IIB component (EC 2.7.1.69)	1	-	-	-	-	-	-	-	-	-	-	-	-	-	-	-	-	-	-	-	-	-	-	-	+	-	-	-	-	-	-	-	-	-	-	-	-	-	-	-	-	-	-	-	-	-	-	-	-	-	-	-	-	-
7143	21239	hypothetical protein	1	-	-	-	-	-	-	-	-	-	-	-	-	-	-	-	-	-	-	-	-	-	-	-	+	-	-	-	-	-	-	-	-	-	-	-	-	-	-	-	-	-	-	-	-	-	-	-	-	-	-	-	-	-
7144	21240	ABC-type sugar transport system, periplasmic component	1	-	-	-	-	-	-	-	-	-	-	-	-	-	-	-	-	-	-	-	-	-	-	-	+	-	-	-	-	-	-	-	-	-	-	-	-	-	-	-	-	-	-	-	-	-	-	-	-	-	-	-	-	-
7145	21241	pts system fructose IIA component	1	-	-	-	-	-	-	-	-	-	-	-	-	-	-	-	-	-	-	-	-	-	-	-	+	-	-	-	-	-	-	-	-	-	-	-	-	-	-	-	-	-	-	-	-	-	-	-	-	-	-	-	-	-
7146	21242	HAD superfamily hydrolase	1	-	-	-	-	-	-	-	-	-	-	-	-	-	-	-	-	-	-	-	-	-	-	-	+	-	-	-	-	-	-	-	-	-	-	-	-	-	-	-	-	-	-	-	-	-	-	-	-	-	-	-	-	-
7147	21243	transcriptional antiterminator, BglG	1	-	-	-	-	-	-	-	-	-	-	-	-	-	-	-	-	-	-	-	-	-	-	-	+	-	-	-	-	-	-	-	-	-	-	-	-	-	-	-	-	-	-	-	-	-	-	-	-	-	-	-	-	-
7148	21244	hypothetical protein	1	-	-	-	-	-	-	-	-	-	-	-	-	-	-	-	-	-	-	-	-	-	-	-	+	-	-	-	-	-	-	-	-	-	-	-	-	-	-	-	-	-	-	-	-	-	-	-	-	-	-	-	-	-
7149	21245	Ribose 5-phosphate isomerase A (EC 5.3.1.6)	1	-	-	-	-	-	-	-	-	-	-	-	-	-	-	-	-	-	-	-	-	-	-	-	+	-	-	-	-	-	-	-	-	-	-	-	-	-	-	-	-	-	-	-	-	-	-	-	-	-	-	-	-	-
7150	21246	Integrase/recombinase, putative, truncated	1	-	-	-	-	-	-	-	-	-	-	-	-	-	-	-	-	-	-	-	-	-	-	-	+	-	-	-	-	-	-	-	-	-	-	-	-	-	-	-	-	-	-	-	-	-	-	-	-	-	-	-	-	-
7151	21247	Mg(2+) transport ATPase protein C	1	-	-	-	-	-	-	-	-	-	-	-	-	-	-	-	-	-	-	-	-	-	-	-	+	-	-	-	-	-	-	-	-	-	-	-	-	-	-	-	-	-	-	-	-	-	-	-	-	-	-	-	-	-
7152	21248	Muramidase-released protein precursor (136 kDa surface protein)	1	-	-	-	-	-	-	-	-	-	-	-	-	-	-	-	-	-	-	-	-	-	-	-	+	-	-	-	-	-	-	-	-	-	-	-	-	-	-	-	-	-	-	-	-	-	-	-	-	-	-	-	-	-
7153	21249	Possible protein-tyrosine-phosphatase (EC 3.1.3.48)	1	-	-	-	-	-	-	-	-	-	-	-	-	-	-	-	-	-	-	-	-	-	-	-	+	-	-	-	-	-	-	-	-	-	-	-	-	-	-	-	-	-	-	-	-	-	-	-	-	-	-	-	-	-
7154	21250	hypothetical protein	1	-	-	-	-	+	-	-	-	-	-	-	-	-	-	-	-	-	-	-	-	-	-	-	-	-	-	-	-	-	-	-	-	-	-	-	-	-	-	-	-	-	-	-	-	-	-	-	-	-	-	-	-	-
7155	21251	Putative deoxyribose-specific ABC transporter, permease protein	1	-	-	-	-	+	-	-	-	-	-	-	-	-	-	-	-	-	-	-	-	-	-	-	-	-	-	-	-	-	-	-	-	-	-	-	-	-	-	-	-	-	-	-	-	-	-	-	-	-	-	-	-	-
7156	21252	hypothetical protein	1	-	-	-	-	+	-	-	-	-	-	-	-	-	-	-	-	-	-	-	-	-	-	-	-	-	-	-	-	-	-	-	-	-	-	-	-	-	-	-	-	-	-	-	-	-	-	-	-	-	-	-	-	-
7157	21253	Peptidyl-prolyl cis-trans isomerase (EC 5.2.1.8)	1	-	-	-	-	-	-	-	-	-	-	-	-	-	-	-	-	-	+	-	-	-	-	-	-	-	-	-	-	-	-	-	-	-	-	-	-	-	-	-	-	-	-	-	-	-	-	-	-	-	-	-	-	-
7158	21254	ABC transporter, permease protein	1	-	-	-	-	-	-	-	-	-	-	-	-	-	-	-	-	-	+	-	-	-	-	-	-	-	-	-	-	-	-	-	-	-	-	-	-	-	-	-	-	-	-	-	-	-	-	-	-	-	-	-	-	-
7159	21255	hypothetical protein	1	-	-	-	-	-	-	-	-	-	-	-	-	-	-	-	-	-	+	-	-	-	-	-	-	-	-	-	-	-	-	-	-	-	-	-	-	-	-	-	-	-	-	-	-	-	-	-	-	-	-	-	-	-
7160	21256	hypothetical protein	1	-	-	-	-	-	-	-	-	-	-	-	-	-	-	-	-	-	+	-	-	-	-	-	-	-	-	-	-	-	-	-	-	-	-	-	-	-	-	-	-	-	-	-	-	-	-	-	-	-	-	-	-	-
7161	21257	hypothetical protein	1	-	-	-	-	-	-	-	-	-	-	-	-	-	-	-	-	-	+	-	-	-	-	-	-	-	-	-	-	-	-	-	-	-	-	-	-	-	-	-	-	-	-	-	-	-	-	-	-	-	-	-	-	-
7162	21258	N-Acetyl-D-glucosamine ABC transport system, permease protein 1	1	-	-	-	-	-	-	-	-	-	-	-	-	-	-	-	-	-	+	-	-	-	-	-	-	-	-	-	-	-	-	-	-	-	-	-	-	-	-	-	-	-	-	-	-	-	-	-	-	-	-	-	-	-
7163	21259	Hypothetical protein	1	-	-	-	-	-	-	-	-	-	-	-	-	-	-	-	-	-	+	-	-	-	-	-	-	-	-	-	-	-	-	-	-	-	-	-	-	-	-	-	-	-	-	-	-	-	-	-	-	-	-	-	-	-
7164	21260	Transposase	1	-	-	-	-	-	-	-	-	-	-	-	-	-	-	-	-	-	+	-	-	-	-	-	-	-	-	-	-	-	-	-	-	-	-	-	-	-	-	-	-	-	-	-	-	-	-	-	-	-	-	-	-	-
7165	21261	hypothetical protein	1	-	-	-	-	-	-	-	-	-	-	-	-	-	-	-	-	-	+	-	-	-	-	-	-	-	-	-	-	-	-	-	-	-	-	-	-	-	-	-	-	-	-	-	-	-	-	-	-	-	-	-	-	-
7166	21262	Isocitrate dehydrogenase [NADP] (EC 1.1.1.42)	1	-	-	-	-	-	-	-	-	-	-	-	-	-	-	-	-	-	+	-	-	-	-	-	-	-	-	-	-	-	-	-	-	-	-	-	-	-	-	-	-	-	-	-	-	-	-	-	-	-	-	-	-	-
7167	21263	hypothetical protein	1	-	-	-	-	-	-	-	-	-	-	-	-	-	-	-	-	-	+	-	-	-	-	-	-	-	-	-	-	-	-	-	-	-	-	-	-	-	-	-	-	-	-	-	-	-	-	-	-	-	-	-	-	-
7168	21264	hypothetical protein	1	-	-	-	-	-	-	-	-	-	-	-	-	-	-	-	+	-	-	-	-	-	-	-	-	-	-	-	-	-	-	-	-	-	-	-	-	-	-	-	-	-	-	-	-	-	-	-	-	-	-	-	-	-
7169	21265	hypothetical protein	1	-	-	-	-	-	-	-	-	-	-	-	-	-	-	-	-	-	+	-	-	-	-	-	-	-	-	-	-	-	-	-	-	-	-	-	-	-	-	-	-	-	-	-	-	-	-	-	-	-	-	-	-	-
7170	21266	hypothetical protein	1	-	-	-	-	-	-	-	-	-	-	-	-	-	-	-	-	-	+	-	-	-	-	-	-	-	-	-	-	-	-	-	-	-	-	-	-	-	-	-	-	-	-	-	-	-	-	-	-	-	-	-	-	-
7171	21267	Inosine-5'-monophosphate dehydrogenase (EC 1.1.1.205)	1	-	-	-	+	-	-	-	-	-	-	-	-	-	-	-	-	-	-	-	-	-	-	-	-	-	-	-	-	-	-	-	-	-	-	-	-	-	-	-	-	-	-	-	-	-	-	-	-	-	-	-	-	-
7172	22567	hypothetical protein	1	-	-	-	-	-	-	-	-	-	-	-	-	-	-	-	-	+	-	-	-	-	-	-	-	-	-	-	-	-	-	-	-	-	-	-	-	-	-	-	-	-	-	-	-	-	-	-	-	-	-	-	-	-
7173	22568	Group B streptococcal surface immunogenic protein	1	-	-	-	-	-	-	-	-	-	-	-	-	-	-	-	-	+	-	-	-	-	-	-	-	-	-	-	-	-	-	-	-	-	-	-	-	-	-	-	-	-	-	-	-	-	-	-	-	-	-	-	-	-
7174	22569	hypothetical protein	1	-	-	-	-	-	-	-	-	-	-	-	-	-	-	-	-	+	-	-	-	-	-	-	-	-	-	-	-	-	-	-	-	-	-	-	-	-	-	-	-	-	-	-	-	-	-	-	-	-	-	-	-	-
7175	22570	hypothetical protein	1	-	-	-	-	-	-	-	-	-	-	-	-	-	-	-	-	+	-	-	-	-	-	-	-	-	-	-	-	-	-	-	-	-	-	-	-	-	-	-	-	-	-	-	-	-	-	-	-	-	-	-	-	-
7176	22571	hypothetical protein	1	-	-	-	-	-	-	-	-	-	-	-	-	-	-	-	-	+	-	-	-	-	-	-	-	-	-	-	-	-	-	-	-	-	-	-	-	-	-	-	-	-	-	-	-	-	-	-	-	-	-	-	-	-
7177	22572	hypothetical protein	1	-	-	-	-	-	-	-	-	-	-	-	-	-	-	-	-	+	-	-	-	-	-	-	-	-	-	-	-	-	-	-	-	-	-	-	-	-	-	-	-	-	-	-	-	-	-	-	-	-	-	-	-	-
7178	22573	hypothetical protein	1	-	-	-	-	-	-	-	-	-	-	-	-	-	-	-	-	-	-	-	-	-	-	-	-	+	-	-	-	-	-	-	-	-	-	-	-	-	-	-	-	-	-	-	-	-	-	-	-	-	-	-	-	-
7179	22574	Periaxin	1	-	-	-	-	-	-	-	-	-	-	-	-	-	-	-	-	-	-	-	-	-	-	-	-	+	-	-	-	-	-	-	-	-	-	-	-	-	-	-	-	-	-	-	-	-	-	-	-	-	-	-	-	-
7180	22575	hypothetical protein	1	-	-	-	-	-	-	-	-	-	-	-	-	-	-	-	-	-	-	-	-	-	-	-	-	+	-	-	-	-	-	-	-	-	-	-	-	-	-	-	-	-	-	-	-	-	-	-	-	-	-	-	-	-
7181	22576	hypothetical protein	1	-	-	-	-	-	-	-	-	-	-	-	-	-	-	-	-	-	-	-	-	-	-	-	-	+	-	-	-	-	-	-	-	-	-	-	-	-	-	-	-	-	-	-	-	-	-	-	-	-	-	-	-	-
7182	22577	Group B streptococcal surface immunogenic protein	1	-	-	-	-	-	-	-	-	-	-	-	-	-	-	-	-	-	-	-	-	-	-	-	-	+	-	-	-	-	-	-	-	-	-	-	-	-	-	-	-	-	-	-	-	-	-	-	-	-	-	-	-	-
7183	22578	hypothetical protein	1	-	-	-	-	-	-	-	-	-	-	-	-	-	-	-	-	-	-	-	-	-	-	-	-	+	-	-	-	-	-	-	-	-	-	-	-	-	-	-	-	-	-	-	-	-	-	-	-	-	-	-	-	-
7184	22579	Integrase	1	-	-	-	-	-	-	-	-	-	-	-	-	-	-	-	-	-	-	-	-	-	-	-	-	+	-	-	-	-	-	-	-	-	-	-	-	-	-	-	-	-	-	-	-	-	-	-	-	-	-	-	-	-
7185	22580	hypothetical protein	1	-	-	-	-	-	-	-	-	-	-	-	-	-	-	-	-	-	-	-	-	-	-	-	-	+	-	-	-	-	-	-	-	-	-	-	-	-	-	-	-	-	-	-	-	-	-	-	-	-	-	-	-	-
7186	22581	hypothetical protein	1	-	-	-	-	-	-	-	-	-	-	-	-	-	-	-	-	-	-	-	-	-	-	-	-	-	-	-	-	-	-	-	-	-	-	-	-	-	-	+	-	-	-	-	-	-	-	-	-	-	-	-	-	-	-	-
7187	22582	hypothetical protein	1	-	-	-	-	-	-	-	-	-	-	-	-	-	-	-	-	-	-	-	-	-	-	-	-	-	-	-	-	-	-	-	-	-	-	-	-	-	-	+	-	-	-	-	-	-	-	-	-	-	-	-	-	-	-	-
7188	22583	hypothetical protein	1	-	-	-	-	-	-	-	-	-	-	-	-	-	-	-	-	-	-	-	-	-	-	-	-	-	-	-	-	-	-	-	-	-	-	-	-	-	-	+	-	-	-	-	-	-	-	-	-	-	-	-	-	-	-	-
7189	22584	Periaxin	1	-	-	-	-	-	-	-	-	-	-	-	-	-	-	-	-	-	-	-	-	-	-	-	-	-	-	-	-	-	-	-	-	-	-	-	-	-	-	+	-	-	-	-	-	-	-	-	-	-	-	-	-	-	-	-
7190	22585	hypothetical protein	1	-	-	-	-	-	-	-	-	-	-	-	-	-	-	-	-	-	-	-	-	-	-	-	-	-	-	-	-	-	-	-	-	-	-	-	-	-	-	+	-	-	-	-	-	-	-	-	-	-	-	-	-	-	-	-
7191	22586	hypothetical protein	1	-	-	-	-	-	-	-	-	-	-	-	-	-	-	-	-	-	-	-	-	-	-	-	-	-	-	-	-	-	-	-	-	-	-	-	-	-	-	+	-	-	-	-	-	-	-	-	-	-	-	-	-	-	-	-
7192	22587	hypothetical protein	1	-	-	-	-	-	-	-	-	-	-	-	-	-	-	-	-	-	-	-	-	-	-	-	-	-	-	-	-	-	-	-	-	-	-	-	-	-	-	+	-	-	-	-	-	-	-	-	-	-	-	-	-	-	-	-
7193	22588	hypothetical protein	1	-	-	-	-	-	-	-	-	-	-	-	-	-	-	-	-	-	-	-	-	-	-	-	-	-	-	-	-	-	-	-	-	-	-	-	-	-	-	+	-	-	-	-	-	-	-	-	-	-	-	-	-	-	-	-
7194	22589	hypothetical protein	1	-	-	-	-	-	-	-	-	-	-	-	-	-	-	-	-	-	-	-	-	-	-	-	-	-	-	-	-	-	-	-	-	-	-	-	-	-	-	+	-	-	-	-	-	-	-	-	-	-	-	-	-	-	-	-
7195	22590	Group B streptococcal surface immunogenic protein	1	-	-	-	-	-	-	-	-	-	-	-	-	-	-	-	-	-	-	-	-	-	-	-	-	-	-	-	-	-	-	-	-	-	-	-	-	-	-	+	-	-	-	-	-	-	-	-	-	-	-	-	-	-	-	-
7196	22591	Periaxin	1	-	-	-	-	-	-	-	-	-	-	-	-	-	-	-	-	-	-	-	-	-	-	-	-	-	-	-	-	-	-	-	-	-	-	-	-	-	-	-	+	-	-	-	-	-	-	-	-	-	-	-	-	-	-	-
7197	22592	hypothetical protein	1	-	-	-	-	-	-	-	-	-	-	-	-	-	-	-	-	-	-	-	-	-	-	-	-	-	-	-	-	-	-	-	-	-	-	-	-	-	-	-	+	-	-	-	-	-	-	-	-	-	-	-	-	-	-	-
7198	22593	hypothetical protein	1	-	-	-	-	-	-	-	-	-	-	-	-	-	-	-	-	-	-	-	-	-	-	-	-	-	-	-	-	-	-	-	-	-	-	-	-	-	-	-	+	-	-	-	-	-	-	-	-	-	-	-	-	-	-	-
7199	22594	hypothetical protein	1	-	-	-	-	-	-	-	-	-	-	-	-	-	-	-	-	-	-	-	-	-	-	-	-	-	-	-	-	-	-	-	-	-	-	-	-	-	-	-	+	-	-	-	-	-	-	-	-	-	-	-	-	-	-	-
7200	22595	hypothetical protein	1	-	-	-	-	-	-	-	-	-	-	-	-	-	-	-	-	-	-	-	-	-	-	-	-	-	-	-	-	-	-	-	-	-	-	-	-	-	-	-	+	-	-	-	-	-	-	-	-	-	-	-	-	-	-	-
7201	22596	Group B streptococcal surface immunogenic protein	1	-	-	-	-	-	-	-	-	-	-	-	-	-	-	-	-	-	-	-	-	-	-	-	-	-	-	-	-	-	-	-	-	-	-	-	-	-	-	-	+	-	-	-	-	-	-	-	-	-	-	-	-	-	-	-
7202	22597	Group B streptococcal surface immunogenic protein	1	-	-	-	-	-	-	-	-	-	-	-	-	-	-	-	-	-	-	-	-	-	-	-	-	-	-	-	-	-	-	-	-	-	-	-	-	-	-	-	-	-	-	-	-	-	+	-	-	-	-	-	-	-	-	-	-	-
7203	22598	hypothetical protein	1	-	-	-	-	-	-	-	-	-	-	-	-	-	-	-	-	-	-	-	-	-	-	-	-	-	-	-	-	-	-	-	-	-	-	-	-	-	-	-	-	-	-	-	-	-	+	-	-	-	-	-	-	-	-	-	-	-
7204	22599	Integrase	1	-	-	-	-	-	-	-	-	-	-	-	-	-	-	-	-	-	-	-	-	-	-	-	-	-	-	-	-	-	-	-	-	-	-	-	-	-	-	-	-	-	-	-	-	-	+	-	-	-	-	-	-	-	-	-	-	-
7205	22600	hypothetical protein	1	-	-	-	-	-	-	-	-	-	-	-	-	-	-	-	-	-	-	-	-	-	-	-	-	-	-	-	-	-	-	-	-	-	-	-	-	-	-	-	-	-	-	-	-	-	+	-	-	-	-	-	-	-	-	-	-	-
7206	22601	hypothetical protein	1	-	-	-	-	-	-	-	-	-	-	-	-	-	-	-	-	-	-	-	-	-	-	-	-	-	-	-	-	-	-	-	-	-	-	-	-	-	-	-	-	-	-	-	-	-	+	-	-	-	-	-	-	-	-	-	-	-
7207	22602	Cellulase(EC:3.2.1.4)	1	-	-	-	-	-	-	-	-	-	-	-	-	-	-	-	-	-	-	-	-	-	-	-	-	-	-	-	-	-	-	-	-	-	-	-	-	-	-	-	-	-	-	-	-	-	+	-	-	-	-	-	-	-	-	-	-	-
7208	22603	Group B streptococcal surface immunogenic protein	1	-	-	-	-	-	-	-	-	-	-	-	-	-	-	-	-	-	-	-	-	-	-	+	-	-	-	-	-	-	-	-	-	-	-	-	-	-	-	-	-	-	-	-	-	-	-	-	-	-	-	-	-	-	-	-
7209	22604	hypothetical protein	1	-	-	-	-	-	-	-	-	-	-	-	-	-	-	-	-	-	-	-	-	-	-	+	-	-	-	-	-	-	-	-	-	-	-	-	-	-	-	-	-	-	-	-	-	-	-	-	-	-	-	-	-	-	-	-
7210	22605	hypothetical protein	1	-	-	-	-	-	-	-	-	-	-	-	-	-	-	-	-	-	-	-	-	-	-	+	-	-	-	-	-	-	-	-	-	-	-	-	-	-	-	-	-	-	-	-	-	-	-	-	-	-	-	-	-	-	-	-
7211	22606	Mobile element protein	1	-	-	-	-	-	-	-	-	-	-	-	-	-	-	-	-	-	-	-	-	-	-	+	-	-	-	-	-	-	-	-	-	-	-	-	-	-	-	-	-	-	-	-	-	-	-	-	-	-	-	-	-	-	-	-
7212	22607	hypothetical protein	1	-	-	-	-	-	-	-	-	-	-	-	-	-	-	-	-	-	-	-	-	-	-	+	-	-	-	-	-	-	-	-	-	-	-	-	-	-	-	-	-	-	-	-	-	-	-	-	-	-	-	-	-	-	-	-
7213	22608	hypothetical protein	1	-	-	-	-	-	-	-	-	-	-	-	-	-	-	-	-	-	-	-	-	-	-	+	-	-	-	-	-	-	-	-	-	-	-	-	-	-	-	-	-	-	-	-	-	-	-	-	-	-	-	-	-	-	-	-
7214	22609	hypothetical protein	1	-	-	-	-	-	-	-	-	-	-	-	-	-	-	-	-	-	-	-	-	-	-	+	-	-	-	-	-	-	-	-	-	-	-	-	-	-	-	-	-	-	-	-	-	-	-	-	-	-	-	-	-	-	-	-
7215	22610	Integrase	1	-	-	-	-	-	-	-	-	-	-	-	-	-	-	-	-	-	-	-	-	-	-	+	-	-	-	-	-	-	-	-	-	-	-	-	-	-	-	-	-	-	-	-	-	-	-	-	-	-	-	-	-	-	-	-
7216	22611	hypothetical protein	1	-	-	-	-	-	-	-	-	-	-	-	-	-	-	-	-	-	-	-	-	-	-	+	-	-	-	-	-	-	-	-	-	-	-	-	-	-	-	-	-	-	-	-	-	-	-	-	-	-	-	-	-	-	-	-
7217	22612	hypothetical protein	1	-	-	-	-	-	-	-	-	-	-	-	-	-	-	-	-	-	-	-	-	-	-	+	-	-	-	-	-	-	-	-	-	-	-	-	-	-	-	-	-	-	-	-	-	-	-	-	-	-	-	-	-	-	-	-
7218	22613	hypothetical protein	1	-	-	-	-	-	-	-	-	-	-	-	-	-	-	-	+	-	-	-	-	-	-	-	-	-	-	-	-	-	-	-	-	-	-	-	-	-	-	-	-	-	-	-	-	-	-	-	-	-	-	-	-	-	-	-
7219	22614	hypothetical protein	1	-	-	-	-	-	-	-	-	-	-	-	-	-	+	-	-	-	-	-	-	-	-	-	-	-	-	-	-	-	-	-	-	-	-	-	-	-	-	-	-	-	-	-	-	-	-	-	-	-	-	-	-	-	-	-
7220	22615	hypothetical protein	1	-	-	-	-	-	-	-	-	-	-	-	-	-	-	-	+	-	-	-	-	-	-	-	-	-	-	-	-	-	-	-	-	-	-	-	-	-	-	-	-	-	-	-	-	-	-	-	-	-	-	-	-	-	-	-
7221	22616	hypothetical protein	1	-	-	-	-	-	-	-	-	-	-	-	-	-	-	-	+	-	-	-	-	-	-	-	-	-	-	-	-	-	-	-	-	-	-	-	-	-	-	-	-	-	-	-	-	-	-	-	-	-	-	-	-	-	-	-
7222	22617	hypothetical protein	1	-	-	-	-	-	-	-	-	-	-	-	-	-	-	-	+	-	-	-	-	-	-	-	-	-	-	-	-	-	-	-	-	-	-	-	-	-	-	-	-	-	-	-	-	-	-	-	-	-	-	-	-	-	-	-
7223	22618	hypothetical protein	1	-	-	-	-	-	-	-	-	-	-	-	-	-	-	-	+	-	-	-	-	-	-	-	-	-	-	-	-	-	-	-	-	-	-	-	-	-	-	-	-	-	-	-	-	-	-	-	-	-	-	-	-	-	-	-
7224	22619	hypothetical protein	1	-	-	-	-	-	-	-	-	-	-	-	-	-	-	-	+	-	-	-	-	-	-	-	-	-	-	-	-	-	-	-	-	-	-	-	-	-	-	-	-	-	-	-	-	-	-	-	-	-	-	-	-	-	-	-
7225	22620	Integrase	1	-	-	-	-	-	-	-	-	-	-	-	-	-	-	-	+	-	-	-	-	-	-	-	-	-	-	-	-	-	-	-	-	-	-	-	-	-	-	-	-	-	-	-	-	-	-	-	-	-	-	-	-	-	-	-
7226	22621	Group B streptococcal surface immunogenic protein	1	-	-	-	-	-	-	-	-	-	-	-	-	-	-	-	+	-	-	-	-	-	-	-	-	-	-	-	-	-	-	-	-	-	-	-	-	-	-	-	-	-	-	-	-	-	-	-	-	-	-	-	-	-	-	-
7227	22622	Potassium voltage-gated channel subfamily KQT; possible potassium channel	1	-	-	-	-	-	-	-	-	-	-	-	-	-	-	-	-	-	-	-	-	-	-	-	-	-	-	-	-	-	-	-	-	-	-	-	-	-	-	-	-	-	-	-	-	-	-	-	-	-	-	-	-	+
7228	22623	hypothetical protein	1	-	-	-	-	-	-	-	-	-	-	-	-	-	-	-	-	-	-	-	-	-	-	-	-	-	-	-	-	-	-	-	-	-	-	-	-	-	-	-	-	-	-	-	-	-	-	-	-	-	-	-	-	+
7229	22624	hypothetical protein	1	-	-	-	-	-	-	-	-	-	-	-	-	-	-	-	-	-	-	-	-	-	-	-	-	-	-	-	-	-	-	-	-	-	-	-	-	-	-	-	-	-	-	-	-	-	-	-	-	-	-	-	-	+
7230	22625	cell surface SD repeat protein precursor	1	-	-	-	-	-	-	-	-	-	-	-	-	-	-	-	-	-	-	-	-	-	-	-	-	-	-	-	-	-	-	-	-	-	-	-	-	-	-	-	-	-	-	-	-	-	-	-	-	-	-	-	-	+
7231	22626	Group B streptococcal surface immunogenic protein	1	-	-	-	-	-	-	-	-	-	-	-	-	-	-	-	-	-	-	-	-	-	-	-	-	-	-	-	-	-	-	-	-	-	-	-	-	-	-	-	-	-	-	-	-	-	-	-	-	-	-	-	-	+
7232	22627	hypothetical protein	1	-	-	-	-	-	-	-	-	-	-	-	-	-	-	-	-	-	-	-	-	-	-	-	-	-	-	-	-	-	-	-	-	-	-	-	-	-	-	-	-	-	-	-	-	-	-	-	-	-	-	-	-	+
7233	22628	Predicted cell-wall-anchored protein SasA (LPXTG motif)	1	-	-	-	-	-	-	-	-	-	-	-	-	-	-	-	-	-	-	-	-	-	-	-	-	-	-	-	-	-	-	-	-	-	-	-	-	-	-	-	-	-	-	-	-	-	-	-	-	-	-	-	-	+
7234	22629	Predicted cell-wall-anchored protein SasA (LPXTG motif)	1	-	-	-	-	-	-	-	-	-	-	-	-	-	-	-	-	-	-	-	-	-	-	-	-	-	-	-	-	-	-	-	-	-	-	-	-	-	-	-	-	-	-	-	-	-	-	-	-	-	-	-	-	+
7235	22630	Predicted cell-wall-anchored protein SasA (LPXTG motif)	1	-	-	-	-	-	-	-	-	-	-	-	-	-	-	-	-	-	-	-	-	-	-	-	-	-	-	-	-	-	-	-	-	-	-	-	-	-	-	-	-	-	-	-	-	-	-	-	-	-	-	-	-	+
7236	22631	Predicted cell-wall-anchored protein SasA (LPXTG motif)	1	-	-	-	-	-	-	-	-	-	-	-	-	-	-	-	-	-	-	-	-	-	-	-	-	-	-	-	-	-	-	-	-	-	-	-	-	-	-	-	-	-	-	-	-	-	-	-	-	-	-	-	-	+
7237	22632	Predicted cell-wall-anchored protein SasA (LPXTG motif)	1	-	-	-	-	-	-	-	-	-	-	-	-	-	-	-	-	-	-	-	-	-	-	-	-	-	-	-	-	-	-	-	-	-	-	-	-	-	-	-	-	-	-	-	-	-	-	-	-	-	-	-	-	+
7238	22633	clumping factor	1	-	-	-	-	-	-	-	-	-	-	-	-	-	-	-	-	-	-	-	-	-	-	-	-	-	-	-	-	-	-	-	-	-	-	-	-	-	-	-	-	-	-	-	-	-	-	-	-	-	-	-	-	+
7239	22634	hypothetical protein	1	-	-	-	-	-	-	-	-	-	-	-	-	-	-	-	-	-	-	-	-	-	-	-	-	-	-	-	-	-	-	-	-	-	-	-	-	-	-	-	-	-	-	-	-	-	-	-	-	-	-	-	-	+
7240	22635	hypothetical protein	1	-	-	-	-	-	-	-	-	-	-	-	-	-	-	-	-	-	-	-	-	-	-	-	-	-	-	-	-	-	-	-	-	-	-	-	-	-	-	-	-	-	-	-	-	-	-	-	-	-	-	-	-	+
7241	22636	hypothetical protein	1	-	-	-	-	-	-	-	-	-	-	-	-	-	-	-	-	-	-	-	-	-	-	-	-	-	-	-	-	-	-	-	-	-	-	-	-	-	-	-	-	-	-	-	-	-	-	-	-	-	-	-	-	+
7242	22637	hypothetical protein	1	-	-	-	-	-	-	-	-	-	-	-	-	-	-	-	-	-	-	-	-	-	-	-	-	-	-	-	-	-	-	-	-	-	-	-	-	-	-	-	-	-	-	-	+	-	-	-	-	-	-	-	-	-
7243	22638	hypothetical protein	1	-	-	-	-	-	-	-	-	-	-	-	-	-	-	-	-	-	-	-	-	-	-	-	-	-	-	-	-	-	-	-	-	-	-	-	-	-	-	-	-	-	-	-	+	-	-	-	-	-	-	-	-	-
7244	22639	hypothetical protein	1	-	-	-	-	-	-	-	-	-	-	-	-	-	-	-	-	-	-	-	-	-	-	-	-	-	-	-	-	-	-	-	-	-	-	-	-	-	-	-	-	-	-	-	+	-	-	-	-	-	-	-	-	-
7245	22640	hypothetical protein	1	-	-	-	-	-	-	-	-	-	-	-	-	-	-	-	-	-	-	-	-	-	-	-	-	-	-	-	-	-	-	-	-	-	-	-	-	-	-	-	-	-	-	-	+	-	-	-	-	-	-	-	-	-
7246	22641	hypothetical protein	1	-	-	-	-	-	-	-	-	-	-	-	-	-	-	-	-	-	-	-	-	-	-	-	-	-	-	-	-	-	-	-	-	-	-	-	-	-	-	-	-	-	-	-	+	-	-	-	-	-	-	-	-	-
7247	22642	Group B streptococcal surface immunogenic protein	1	-	-	-	-	-	-	-	-	-	-	-	-	-	-	-	-	-	-	-	-	-	-	-	-	-	-	-	-	-	-	-	-	-	-	-	-	-	-	-	-	-	-	-	+	-	-	-	-	-	-	-	-	-
7248	22643	hypothetical protein	1	-	-	-	-	-	-	-	-	-	-	-	-	-	-	-	-	-	-	-	-	-	-	-	-	-	-	-	-	-	-	-	-	-	-	-	-	-	-	-	-	-	-	-	+	-	-	-	-	-	-	-	-	-
7249	22644	UDP-galactopyranose mutase (EC 5.4.99.9)	1	-	-	-	-	-	-	-	-	-	-	-	-	-	-	-	-	-	-	-	-	-	-	-	-	-	-	-	-	-	-	-	-	-	-	-	-	-	-	-	-	-	-	-	+	-	-	-	-	-	-	-	-	-
7250	22645	hypothetical protein	1	-	-	-	-	-	-	-	-	-	-	-	-	-	-	-	-	-	-	-	-	-	-	-	-	-	-	-	-	-	-	-	-	-	-	-	-	-	-	-	-	-	-	-	+	-	-	-	-	-	-	-	-	-
7251	22646	hypothetical protein	1	-	-	-	-	-	-	-	-	-	-	-	-	-	-	-	-	-	-	-	-	-	-	-	-	-	-	-	-	-	-	-	-	-	-	-	-	-	+	-	-	-	-	-	-	-	-	-	-	-	-	-	-	-
7252	22647	hypothetical protein	1	-	-	-	-	-	-	-	-	-	-	-	-	-	-	-	-	-	-	-	-	-	-	-	-	-	-	-	-	-	-	-	-	-	-	-	-	-	+	-	-	-	-	-	-	-	-	-	-	-	-	-	-	-
7253	22648	hypothetical protein	1	-	-	-	-	-	-	-	-	-	-	-	-	-	-	-	-	-	-	-	-	-	-	-	-	-	-	-	-	-	-	-	-	-	-	-	-	-	+	-	-	-	-	-	-	-	-	-	-	-	-	-	-	-
7254	22649	Group B streptococcal surface immunogenic protein	1	-	-	-	-	-	-	-	-	-	-	-	-	-	-	-	-	-	-	-	-	-	-	-	-	-	-	-	-	-	-	-	-	-	-	-	-	-	+	-	-	-	-	-	-	-	-	-	-	-	-	-	-	-
7255	22650	hypothetical protein	1	-	-	-	-	-	-	-	-	-	-	-	-	-	-	-	-	-	-	-	-	-	-	-	-	-	-	-	-	-	-	-	-	-	-	-	-	-	+	-	-	-	-	-	-	-	-	-	-	-	-	-	-	-
7256	22651	hypothetical protein	1	-	-	-	-	-	-	-	-	-	-	-	-	-	-	-	-	-	-	-	-	-	-	-	-	-	-	-	-	-	-	-	-	-	-	-	-	-	+	-	-	-	-	-	-	-	-	-	-	-	-	-	-	-
7257	22652	hypothetical protein	1	-	-	-	-	-	-	-	-	-	-	-	-	-	-	-	-	-	-	-	-	-	-	-	-	-	-	-	-	-	-	-	-	-	-	-	-	-	+	-	-	-	-	-	-	-	-	-	-	-	-	-	-	-
7258	22653	sepS25B	1	-	-	-	-	-	-	-	-	-	-	-	-	-	-	-	-	-	-	-	-	-	-	-	-	-	-	-	-	-	-	-	-	-	-	-	-	+	-	-	-	-	-	-	-	-	-	-	-	-	-	-	-	-
7259	22654	hypothetical protein	1	-	-	-	-	-	-	-	-	-	-	-	-	-	-	-	-	-	-	-	-	-	-	-	-	-	-	-	-	-	-	-	-	-	-	-	-	+	-	-	-	-	-	-	-	-	-	-	-	-	-	-	-	-
7260	22655	hypothetical protein	1	-	-	-	-	-	-	-	-	-	-	-	-	-	-	-	-	-	-	-	-	-	-	-	-	-	-	-	-	-	-	-	-	-	-	-	-	+	-	-	-	-	-	-	-	-	-	-	-	-	-	-	-	-
7261	22656	hypothetical protein	1	-	-	-	-	-	-	-	-	-	-	-	-	-	-	-	-	-	-	-	-	-	-	-	-	-	-	-	-	-	-	-	-	-	-	-	-	+	-	-	-	-	-	-	-	-	-	-	-	-	-	-	-	-
7262	22657	Group B streptococcal surface immunogenic protein	1	-	-	-	-	-	-	-	-	-	-	-	-	-	-	-	-	-	-	-	-	-	-	-	-	-	-	-	-	-	-	-	-	-	-	-	-	+	-	-	-	-	-	-	-	-	-	-	-	-	-	-	-	-
7263	22658	hypothetical protein	1	-	-	-	-	-	-	-	-	-	-	-	-	-	-	-	-	-	-	-	-	-	-	-	-	-	-	-	-	-	-	-	-	-	-	-	-	+	-	-	-	-	-	-	-	-	-	-	-	-	-	-	-	-

Table S5. Precence/absence of each HG among 57 *S. suis* strains

				1																13			25					28						104		94			76			53/54		Divergent *S. suis*																
Index[a]	ClutserID	Gene products	Members	98HAH33	05ZYH33	SC84	A7	SS12	P1/7	DAT273	NIAH11434	JS14	GZ1	BM407	S735	13730	MNCM07	NCTC 10446	14636	NCTC 10237	ST1	2651	89-1591	MNCM04	MNCM25	8074	D9	4961	ST3	MNCM43	DAT292	DAT260	NIAH11435	MNCM21	MNCM50	89-2479	DAT234	DAT290	93A	42A	NT77	6407	11538	8830	12814	92-1400	89-590	2726	2524	89-3576-3	4417	D12	10581	89-5259	88-5299A	R61	92-1191	14A	92-4172	22083
7264	22659	hypothetical protein	1	-	-	-	-	-	-	-	-	-	-	-	-	-	-	-	-	-	-	-	-	-	-	-	-	-	-	-	-	-	-	-	-	-	-	-	-	-	-	-	-	+	-	-	-	-	-	-	-	-	-	-	-	-	-	-	-	-
7265	22660	hypothetical protein	1	-	-	-	-	-	-	-	-	-	-	-	-	-	-	-	-	-	-	-	-	-	-	-	-	-	-	-	-	-	-	-	-	-	-	-	-	-	-	-	-	+	-	-	-	-	-	-	-	-	-	-	-	-	-	-	-	-
7266	22661	hypothetical protein	1	-	-	-	-	-	-	-	-	-	-	-	-	-	-	-	-	-	-	-	-	-	-	-	-	-	-	-	-	-	-	-	-	-	-	-	-	-	-	-	-	+	-	-	-	-	-	-	-	-	-	-	-	-	-	-	-	-
7267	22662	hypothetical protein	1	-	-	-	-	-	-	-	-	-	-	-	-	-	-	-	-	-	-	-	-	-	-	-	-	-	-	-	-	-	-	-	-	-	-	-	-	-	-	-	-	+	-	-	-	-	-	-	-	-	-	-	-	-	-	-	-	-
7268	22663	hypothetical protein	1	-	-	-	-	-	-	-	-	-	-	-	-	-	-	-	-	-	-	-	-	-	-	-	-	-	-	-	-	-	-	-	-	-	-	-	-	-	-	-	-	+	-	-	-	-	-	-	-	-	-	-	-	-	-	-	-	-
7269	22664	hypothetical protein	1	-	-	-	-	-	-	-	-	-	-	-	-	-	-	-	-	-	-	-	-	-	-	-	-	-	-	-	-	-	-	-	-	-	-	-	-	-	-	-	-	-	-	-	-	-	-	-	-	-	+	-	-	-	-	-	-	-
7270	22665	hypothetical protein	1	-	-	-	-	-	-	-	-	-	-	-	-	-	-	-	-	-	-	-	-	-	-	-	-	-	-	-	-	-	-	-	-	-	-	-	-	-	-	-	-	-	-	-	-	-	-	-	-	-	+	-	-	-	-	-	-	-
7271	22666	hypothetical protein	1	-	-	-	-	-	-	-	-	-	-	-	-	-	-	-	-	-	-	-	-	-	-	-	-	-	-	-	-	-	-	-	-	-	-	-	-	-	-	-	-	-	-	-	-	-	-	-	-	-	+	-	-	-	-	-	-	-
7272	22667	hypothetical protein	1	-	-	-	-	-	-	-	-	-	-	-	-	-	-	-	-	-	-	-	-	-	-	-	-	-	-	-	-	-	-	-	-	-	-	-	-	-	-	-	-	-	-	-	-	-	-	-	-	-	+	-	-	-	-	-	-	-
7273	22668	hypothetical protein	1	-	-	-	-	-	-	-	-	-	-	-	-	-	-	-	-	-	-	-	-	-	-	-	-	-	-	-	-	-	-	-	-	-	-	-	-	-	-	-	-	-	-	-	-	-	-	-	-	-	+	-	-	-	-	-	-	-
7274	22669	hypothetical protein	1	-	-	-	-	-	-	-	-	-	-	-	-	-	-	-	-	-	-	-	-	-	-	-	-	-	-	-	-	-	-	-	-	-	-	-	-	-	-	-	-	-	-	-	-	-	-	-	-	-	+	-	-	-	-	-	-	-
7275	22670	Group B streptococcal surface immunogenic protein	1	-	-	-	-	-	-	-	-	-	-	-	-	-	-	-	-	-	-	-	-	-	-	-	-	-	-	-	-	-	-	-	-	-	-	-	-	-	-	-	-	-	-	-	-	-	-	-	-	-	+	-	-	-	-	-	-	-
7276	22671	hypothetical protein	1	-	-	-	-	-	-	-	-	-	-	-	-	-	-	-	-	-	-	-	-	-	-	-	-	-	-	-	-	-	-	-	-	-	-	-	-	-	-	-	-	-	-	-	-	-	-	-	-	-	+	-	-	-	-	-	-	-
7277	22672	hypothetical protein	1	-	-	-	-	-	-	-	-	-	-	-	-	-	-	-	-	-	-	-	-	-	-	-	-	-	-	-	-	-	-	-	-	-	-	-	-	-	-	-	-	-	-	-	-	-	-	-	-	-	+	-	-	-	-	-	-	-
7278	22673	hypothetical protein	1	-	-	-	-	-	-	-	-	-	-	-	-	+	-	-	-	-	-	-	-	-	-	-	-	-	-	-	-	-	-	-	-	-	-	-	-	-	-	-	-	-	-	-	-	-	-	-	-	-	-	-	-	-	-	-	-	-
7279	22674	Hypothetical DUF1027 domain protein	1	-	-	-	-	-	-	-	-	-	-	-	-	+	-	-	-	-	-	-	-	-	-	-	-	-	-	-	-	-	-	-	-	-	-	-	-	-	-	-	-	-	-	-	-	-	-	-	-	-	-	-	-	-	-	-	-	-
7280	22675	hypothetical protein	1	-	-	-	-	-	-	-	-	-	-	-	-	+	-	-	-	-	-	-	-	-	-	-	-	-	-	-	-	-	-	-	-	-	-	-	-	-	-	-	-	-	-	-	-	-	-	-	-	-	-	-	-	-	-	-	-	-
7281	22676	Integrase	1	-	-	-	-	-	-	-	-	-	-	-	-	+	-	-	-	-	-	-	-	-	-	-	-	-	-	-	-	-	-	-	-	-	-	-	-	-	-	-	-	-	-	-	-	-	-	-	-	-	-	-	-	-	-	-	-	-
7282	22677	hypothetical protein	1	-	-	-	-	-	-	-	-	-	-	-	-	+	-	-	-	-	-	-	-	-	-	-	-	-	-	-	-	-	-	-	-	-	-	-	-	-	-	-	-	-	-	-	-	-	-	-	-	-	-	-	-	-	-	-	-	-
7283	22678	hypothetical protein	1	-	-	-	-	-	-	-	-	-	-	-	-	+	-	-	-	-	-	-	-	-	-	-	-	-	-	-	-	-	-	-	-	-	-	-	-	-	-	-	-	-	-	-	-	-	-	-	-	-	-	-	-	-	-	-	-	-
7284	22679	Integrase	1	-	-	-	-	-	-	-	-	-	-	-	-	-	-	+	-	-	-	-	-	-	-	-	-	-	-	-	-	-	-	-	-	-	-	-	-	-	-	-	-	-	-	-	-	-	-	-	-	-	-	-	-	-	-	-	-	-
7285	22680	hypothetical protein	1	-	-	-	-	-	-	-	-	-	-	-	-	-	-	+	-	-	-	-	-	-	-	-	-	-	-	-	-	-	-	-	-	-	-	-	-	-	-	-	-	-	-	-	-	-	-	-	-	-	-	-	-	-	-	-	-	-
7286	22681	hypothetical protein	1	-	-	-	-	-	-	-	-	-	-	-	-	-	-	+	-	-	-	-	-	-	-	-	-	-	-	-	-	-	-	-	-	-	-	-	-	-	-	-	-	-	-	-	-	-	-	-	-	-	-	-	-	-	-	-	-	-
7287	22682	hypothetical protein	1	-	-	-	-	-	-	-	-	-	-	-	-	-	-	+	-	-	-	-	-	-	-	-	-	-	-	-	-	-	-	-	-	-	-	-	-	-	-	-	-	-	-	-	-	-	-	-	-	-	-	-	-	-	-	-	-	-
7288	22683	hypothetical protein	1	-	-	-	-	-	-	-	-	-	-	-	-	-	-	+	-	-	-	-	-	-	-	-	-	-	-	-	-	-	-	-	-	-	-	-	-	-	-	-	-	-	-	-	-	-	-	-	-	-	-	-	-	-	-	-	-	-
7289	22684	hypothetical protein	1	-	-	-	-	-	-	-	-	-	-	-	-	-	-	-	-	-	-	-	-	-	-	-	-	-	-	-	-	-	-	-	-	-	-	-	-	-	-	-	-	+	-	-	-	-	-	-	-	-	-	-	-	-
7290	22685	hypothetical protein	1	-	-	-	-	-	-	-	-	-	-	-	-	-	-	-	-	-	-	-	-	-	-	-	-	-	-	-	-	-	-	-	-	-	-	-	-	-	-	-	-	+	-	-	-	-	-	-	-	-	-	-	-	-
7291	22686	hypothetical protein	1	-	-	-	-	-	-	-	-	-	-	-	-	-	-	-	-	-	-	-	-	-	-	-	-	-	-	-	-	-	-	-	-	-	-	-	-	-	-	-	-	+	-	-	-	-	-	-	-	-	-	-	-	-
7292	22687	hypothetical protein	1	-	-	-	-	-	-	-	-	-	-	-	-	-	-	-	-	-	-	-	-	-	-	-	-	-	-	-	-	-	-	-	-	-	-	-	-	-	-	-	-	+	-	-	-	-	-	-	-	-	-	-	-	-
7293	22688	hypothetical protein	1	-	-	-	-	-	-	-	-	-	-	-	-	-	-	-	-	-	-	-	-	-	-	-	-	-	-	-	-	-	-	-	-	-	-	-	-	-	-	-	-	+	-	-	-	-	-	-	-	-	-	-	-	-
7294	22689	hypothetical protein	1	-	-	-	-	-	-	-	-	-	-	-	-	-	-	-	-	-	-	-	-	-	-	-	-	-	-	-	-	-	-	-	-	-	-	-	-	-	-	-	-	+	-	-	-	-	-	-	-	-	-	-	-	-
7295	22690	hypothetical protein	1	-	-	-	-	-	-	-	-	-	-	-	-	-	-	-	-	-	-	-	-	-	-	-	-	-	-	-	-	-	-	-	-	-	-	-	-	-	-	-	-	+	-	-	-	-	-	-	-	-	-	-	-	-
7296	22691	hypothetical protein	1	-	-	-	-	-	-	-	-	-	-	-	-	-	-	-	-	-	-	-	-	-	-	-	-	-	-	-	-	-	-	-	-	-	-	-	-	-	-	-	-	+	-	-	-	-	-	-	-	-	-	-	-	-
7297	22692	hypothetical protein	1	-	-	-	-	-	-	-	-	-	-	-	-	-	-	-	-	-	-	-	-	-	-	-	-	-	-	-	-	-	-	-	-	-	-	-	-	-	-	-	-	+	-	-	-	-	-	-	-	-	-	-	-	-
7298	22693	Group B streptococcal surface immunogenic protein	1	-	-	-	-	-	-	-	-	-	-	-	-	-	-	-	-	-	-	-	-	-	-	-	-	-	-	-	-	-	-	-	-	-	-	-	-	-	-	-	-	+	-	-	-	-	-	-	-	-	-	-	-	-
7299	22694	hypothetical protein	1	-	-	-	-	-	-	-	-	-	-	-	-	-	-	-	-	-	-	-	-	-	-	-	-	-	-	-	-	-	-	-	-	-	-	-	-	-	-	-	-	+	-	-	-	-	-	-	-	-	-	-	-	-
7300	22695	putative tail fiber protein	1	-	-	-	-	-	-	-	-	-	-	-	-	-	-	-	-	-	-	-	-	-	-	-	-	-	-	-	-	-	-	-	+	-	-	-	-	-	-	-	-	-	-	-	-	-	-	-	-	-	-	-	-	-
7301	22696	hypothetical protein	1	-	-	-	-	-	-	-	-	-	-	-	-	-	-	-	-	-	-	-	-	-	-	-	-	-	-	-	-	-	-	-	+	-	-	-	-	-	-	-	-	-	-	-	-	-	-	-	-	-	-	-	-	-
7302	22697	Mobile element protein	1	-	-	-	-	-	-	-	-	-	-	-	-	-	-	-	-	-	-	-	-	-	-	-	-	-	-	-	-	-	-	-	+	-	-	-	-	-	-	-	-	-	-	-	-	-	-	-	-	-	-	-	-	-
7303	22698	hypothetical protein	1	-	-	-	-	-	-	-	-	-	-	-	-	-	-	-	-	-	-	-	-	-	-	-	-	-	-	-	-	-	-	-	+	-	-	-	-	-	-	-	-	-	-	-	-	-	-	-	-	-	-	-	-	-
7304	22699	hypothetical protein	1	-	-	-	-	-	-	-	-	-	-	-	-	-	-	-	-	-	-	-	-	-	-	-	-	-	-	-	-	-	-	-	+	-	-	-	-	-	-	-	-	-	-	-	-	-	-	-	-	-	-	-	-	-
7305	22700	Group B streptococcal surface immunogenic protein	1	-	-	-	-	-	-	-	-	-	-	-	-	-	-	-	-	-	-	-	-	-	-	-	-	-	-	-	-	-	-	-	+	-	-	-	-	-	-	-	-	-	-	-	-	-	-	-	-	-	-	-	-	-
7306	22701	hypothetical protein	1	-	-	-	-	-	-	-	-	-	-	-	-	-	-	-	-	-	-	-	-	-	-	-	-	-	-	-	-	-	-	-	+	-	-	-	-	-	-	-	-	-	-	-	-	-	-	-	-	-	-	-	-	-
7307	22702	hypothetical protein	1	-	-	-	-	-	-	-	-	-	-	-	-	-	-	-	-	-	-	-	-	-	-	-	-	-	-	-	-	-	-	-	+	-	-	-	-	-	-	-	-	-	-	-	-	-	-	-	-	-	-	-	-	-
[truncated: 871,647 more chars]
